# The Fecal Metagenomics of Malayan Pangolins Identifies an Extensive Adaptation to Myrmecophagy

**DOI:** 10.3389/fmicb.2018.02793

**Published:** 2018-11-23

**Authors:** Jing-E Ma, Hai-Ying Jiang, Lin-Miao Li, Xiu-Juan Zhang, Guan-Yu Li, Hui-Ming Li, Xue-Jun Jin, Jin-Ping Chen

**Affiliations:** Guangdong Key Laboratory of Animal Conservation and Resource Utilization, Guangdong Public Laboratory of Wild Animal Conservation and Utilization, Guangdong Institute of Applied Biological Resources, Guangzhou, China

**Keywords:** myrmecophagy, chitin-degrading enzymes, rescue, intestinal microflora, cellulose digestion

## Abstract

The characteristics of flora in the intestine of an animal, including the number and abundance of different microbial species and their functions, are closely related to the diets of the animal and affect the physical condition of the host. The Malayan pangolin (*Manis javanica*) is an endangered species that specializes in myrmecophagy. Analyzing the microbiome in the intestine of the pangolin is imperative to protect this species. By sequencing the metagenomes of the feces of four pangolins, we constructed a non-redundant catalog of 211,868 genes representing 1,811 metagenomic species. Taxonomic annotation revealed that Bacteroidetes (49.9%), Proteobacteria (32.2%), and Firmicutes (12.6%) are the three main phyla. The annotation of gene functions identified 5,044 genes from 88 different glycoside hydrolase (GH) families in the Carbohydrate-Active enZYmes database and 114 gene modules related to chitin-degrading enzymes, corresponding to the catalytic domains of GH18 family enzymes, containing chitinase genes of classes III and V in the dataset. Fourteen gene modules corresponded to the catalytic domains of GH19 family enzymes, containing chitinase genes of classes I, II, and IV. These genes were found in 37 species belonging to four phyla: Bacteroidetes, Cyanobacteria, Firmicutes, and Proteobacteria. Moreover, when the metabolic pathways of these genes were summarized, 41,711 genes were associated with 147 unique KEGG metabolic pathways, and these genes were assigned to two Gene Ontology terms: metabolic process and catalytic activity. We also found several species that likely play roles in the digestion of cellulose and may be able to degrade chitin, including *Enterobacter cloacae, Lactococcus lactis, Chitinimonas koreensis*, and *Chitinophaga pinensis*. In addition, we identified some intestinal microflora and genes related to diseases in pangolins. Twenty-seven species were identified by STAMP analysis as differentially abundant in healthy and diseased animals: 20 species, including *Cellulosilyticum lentocellum* and *Lactobacillus reuteri*, were more abundant in healthy pangolins, while seven species, including *Odoribacter splanchnicus, Marinilabilia salmonicolor, Xanthomonas citri, Xanthomonas vasicola, Oxalobacter formigenes, Prolixibacter bellariivorans*, and *Clostridium bolteae*, were more abundant in diseased pangolins. These results will support the efforts to conserve pangolins.

## Introduction

The Malayan pangolin (*Manis javanica*) is a toothless mammal that specializes in myrmecophagy, eating mostly ants and termites, and is classified as critically endangered in accordance with the International Union for Conservation of Nature (IUCN) Red List of Threatened Species R (Challender et al., 2014). This species is also included in Appendix I (Available online at: https://cites.org/eng/app/appendices.php#) of the Convention on International Trade in Endangered Species of Wild Fauna and Flora (CITES I). Concerted efforts have been made to conserve and rescue this species in captivity in China because of its threatened status and continuing decline in the wild (Ma et al., 2017). It is a significant challenge to maintain this species in captivity. One of the reasons is that the molecular mechanism of its digestion is unknown, and its diet causes great digestive challenges to *M. javanica*. The nutritional values of ants and termites are not obviously different from other terrestrial invertebrates, but much more fat is present in their larval and alate forms than in their other forms (Redford and Dorea, 1984). There is also high protein content in termites and ants, as well as, chitin in the exoskeletons of these insects. The digestive tracts of *M. javanica* do not show notable anatomical adaptations that might present an increased potential for the digestion of particular nutrients, such as chitin. Instead, pangolins have a muscular stomach and a large oxyntic gland mass, which is thought to assist with the digestion of termites and ants (Nisa et al., 2010). Nevertheless, macroscopic inspection of fecal samples in diet characterization studies (Taylor et al., 2002; Miranda et al., 2009) and some functional assays of fecal intestinal microbes (Delsuc et al., 2014; Macdonald et al., 2014) have suggested that the intestinal microbiomes of mammalian myrmecophages could facilitate chitin degradation and assimilation, implying that intestinal bacteria might help *M. javanica* adapt to their ant and termite diet. Another reason for the poor adaptability of pangolins to the captive environment relates to disease control. Pangolins are highly susceptible to different kinds of illnesses, including pneumonia, gastrointestinal diseases, skin diseases, and parasites (Chin et al., 2006, 2012; Wicker et al., 2008; Bao et al., 2013). More knowledge of the pathogen spectrum might help improve the health of captive pangolins. Unfortunately, directly analyzing the molecular mechanisms of the immune system and diseases in pangolins is difficult because of the limited number of available samples. Recent studies have found that microbes play important roles in the health of the host, having profound influences on vertebrate physiology, metabolism, and immune functions (Cani et al., 2008; Clemente et al., 2012; Martín et al., 2014; Angelakis et al., 2016; Boursier et al., 2016; He et al., 2016; Xiao et al., 2016). For example, gut microbes play a role in the host's immunity, behavior, reproductive isolation, and metabolism (Cryan and Dinan, 2012; Brucker and Bordenstein, 2013; Ramakrishna, 2013; Thaiss et al., 2016). Conversely, many factors affecting the health of the host, including certain diseases, can alter microbial function (David et al., 2014; Goodrich et al., 2014). The gut microbes rely on the animal host and help the host complete a variety of physiological and biochemical functions. The gut is vital to digestion and nutrient absorption, and it also plays an important role in animal metabolism (Duffy et al., 2015). In addition, gut microbes compose the largest immune system organ, which not only can adjust its composition and its metabolites to the animal, but can also mediate the interactions between the host and its diet (Cani and Knauf, 2016). A previous study found that a pathobiont in the gut, *Enterococcus gallinarum*, could translocate to the liver and other tissues and trigger autoimmune responses in individuals with a genetic background predisposed to autoimmunity (Manfredo Vieira et al., 2018). The communication that happens on the intestinal mucosal surface helps with the establishment and development of the immune system, making the surface an important immune barrier in the host.

Here, two questions are raised for *M. javanica*. How do the nutrient metabolism functions of the gut microbiome relate to myrmecophagy? Is the gut microbiota associated with diseases in *M. javanica*? To answer these questions and to facilitate the protection of this endangered species, we analyzed the microbiome in fecal samples of *M. javanica* using shotgun metagenomic sequencing. We compared the gut microbiota of healthy individuals with that of individuals suffering from diseases, in order to gain an understanding of the microbial ecosystem in the gut, the interactions among specific dietary factors and diseases, and the gut microbiota in this interesting species.

## Materials and methods

### Sample collection

Fecal samples from *M. javanica* were collected, immediately frozen in liquid nitrogen, and then stored at −80°C until DNA extraction. The four individuals were two healthy female *M. javanica*, named A-1 and A-2 (Group A), and two individuals that had died of diseases. Post-mortem analysis of the two dead pangolins, a male named B-1 and a female named B-2 (Group B), from the Dongguan Institute of Qingfengyuan Animal Medicine (Dongguan, Guangdong, China) revealed inflammation in the lungs. Detailed information about these samples are presented in Table S1. DNA extractions were performed according to the protocol recommended by the manufacturer of the Stool DNA Kit (Magen, Guangdong, China) by using 200 mg feces per sample. The resulting concentrations of fecal DNA were measured by using a NanoDrop spectrophotometer.

### DNA sequencing

Following the instructions of the manufacturer (BGI Genomics, BGI-Shenzhen, Shenzhen 518083, China), we constructed paired-end DNA libraries with different insert sizes of clones and carried out sequencing on the Illumina HiSeq platform.

### DNA assembly and construction of the gene catalog

To obtain clean data, raw reads were filtered to eliminate adaptor contamination and low-quality reads, including (a) reads containing 10% or more ambiguous bases (N bases), (b) reads containing adapter sequences (default: 15 bases overlapped between read and adapter), and (c) reads containing 50% or more low-quality (Q < 20) bases. The remaining reads were considered high-quality reads. For host-related environmental samples, an additional preprocessing step was provided to ensure the reliability of subsequent bioinformatics analysis. This preprocessing step removes sequences that map to the host genome (INSDC: JSZB00000000.1) with more than 90% similarity. To construct a comprehensive gene catalog of gut microbes of *M. javanica*, we assembled the reads *de novo* from each sample into longer contigs with SOAPdenovo2 (Luo et al., 2012) and Rabbit (You et al., 2013). The reads were assembled with a series of different k-mer sizes in parallel per sample. Then, the reads were mapped back to each assembly result with SOAP 2 (Li et al., 2009), and the optimal k-mer size and assembly result were chosen depending on both mapping rate and contig N50. N50 is important to evaluate the integrity of gene sequencing. To identify N50, we arranged the contigs or scaffolds from the largest to the smallest and calculated the total length of all the assembly sequences. Then, the size of the contig or scaffold at which the cumulative length became 50% of the total length defined the value of N50. During the assembly process, the contigs longer than 500 bp were analyzed further. Based on the assembly results, the open reading frames were predicted using the software MetaGeneMark (Zhu et al., 2010) (version 2.10, default parameters, http://topaz.gatech.edu/GeneMark). Genes from different samples were combined and clustered using CD-Hit (Li and Godzik, 2006) (threshold of sequence identity 95% and threshold of alignment coverage 90%). For each sample, the high-quality reads were aligned against the gene catalog by Bowtie2 (Langmead et al., 2009) using the very-sensitive parameter. The Bowtie2 parameters for paired-end reads are listed below: -p8 –very-sensitive-local -k 100 –score-min L, 0, 1, 2.

All the predicted genes were blasted against public databases (blast, *e* < 0.00001), including the Antibiotic Resistance Genes Database (ARDB, Version 1.1, http://ardb.cbcb.umd.edu/); the Carbohydrate-Active enZYmes Database (CAZy, Version 20120313, http://www.cazy.org/); Gene Ontology (GO, http://www.geneontology.org/), describing biological processes, cellular components, and molecular functions; the Kyoto Encyclopedia of Genes and Genomes (KEGG, Version 81, http://www.genome.jp/kegg/), based on molecular-level information, to understand the high-level functions and utilities of biological systems, such as the cell, organism, and ecosystem; the non-redundant protein database (nr, Version 20160219, ftp://ftp.ncbi.nih.gov/blast/db/); and a manually annotated, non-redundant protein sequence database (Swiss-Prot, Version release-2015_04, http://www.uniprot.org/).

### Taxonomic assignment of genes and construction of taxonomy and profiles of relative abundance

The output files of nr BLAST were analyzed using MEGAN (Version 5, http://ab.inf.uni-tuebingen.de/software/megan4/) (Huson et al., 2007). The software reads the results of a BLAST comparison as input and attempts to place each read on a node in the NCBI taxonomy using the lowest common ancestor (LCA) algorithm. The NCBI taxonomy is displayed as a tree, and the size of each node is scaled to show how many reads are assigned to the corresponding taxon. Then, the relative abundance of each taxonomic level was summed from the same taxonomy. The relative abundances of all taxonomic groups were used to generate taxonomy–relative abundance profiles of all samples.

### Computation of relative gene abundance

Reads mapping to multiple genes were reassigned to a “most likely” gene using PathoScope v1.0 (Francis et al., 2013), which uses a Bayesian framework to examine each read's sequence and mapping quality within the context of a global reassignment.

### Differential analysis of the genus abundance between the two groups

To identify genera showing different abundances in the group A and group B gut metagenomic datasets, the differences between the two groups were analyzed using STAMP (http://kiwi.cs.dal.ca/Software/STAMP), and the software package (Parks et al., 2014) was used to test for significant differences. A two-sided Welch's *t*-test was used in the two-group analysis (Parks et al., 2014).

## Results

### Sequencing and *de novo* assembly of metagenome

A total of 28.66 Gb of high-quality data were sequenced, with an average of 7.18 Gb for each sample (SRA accession: SRP 152412) after removing low-quality reads, N reads, and adaptor sequences. Their proportions in the raw data are shown in Figure S1. The statistics of the raw data, clean data, and preprocessed data are shown separately in Table 1.

**Table 1:** Summary of sequencing data for each sample.

**Sample**	**Raw reads**	**Clean reads**	**Clean/Raw (%)**	**Preprocessed reads**	**Preprocessed/Raw (%)**
A-1	62,101,040	47,667,966	76.76	NA	NA
A-2	62,042,452	47,239,496	76.14	NA	NA
B-1	63,606,778	48,32,110	76.14	NA	NA
B-2	65,136,336	47,759,588	73.32	NA	NA

The best assembly with different k-mer sizes was chosen based on contig N50 and mapping rate. A summary of the assembly results is shown in Table 2. The distribution of the lengths of the contigs is shown in Figure S2.

**Table 2:** Statistics of the assembly results.

**Sample**	**Kmer (bp)**	**Contig number**	**Assembly length (bp)**	**N50 (bp)**	**N90 (bp)**	**Max (bp)**	**Min (bp)**	**Average size (bp)**	**Mapping rate (%)**
A-1	51	31,605	97,804,197	9,296	974	470,796	500	3,094	71.55
A-2	71	28,268	64,981,646	5,352	770	561,084	500	2,298	60.05
B-1	51	43,354	106,048,061	5,488	824	721,113	500	2,446	55.79
B-2	71	18,081	53,676,611	9,409	929	524,603	500	2,968	74.7

### A gene catalog of the *manis javanica* gut microbiome

**Figure 1:**
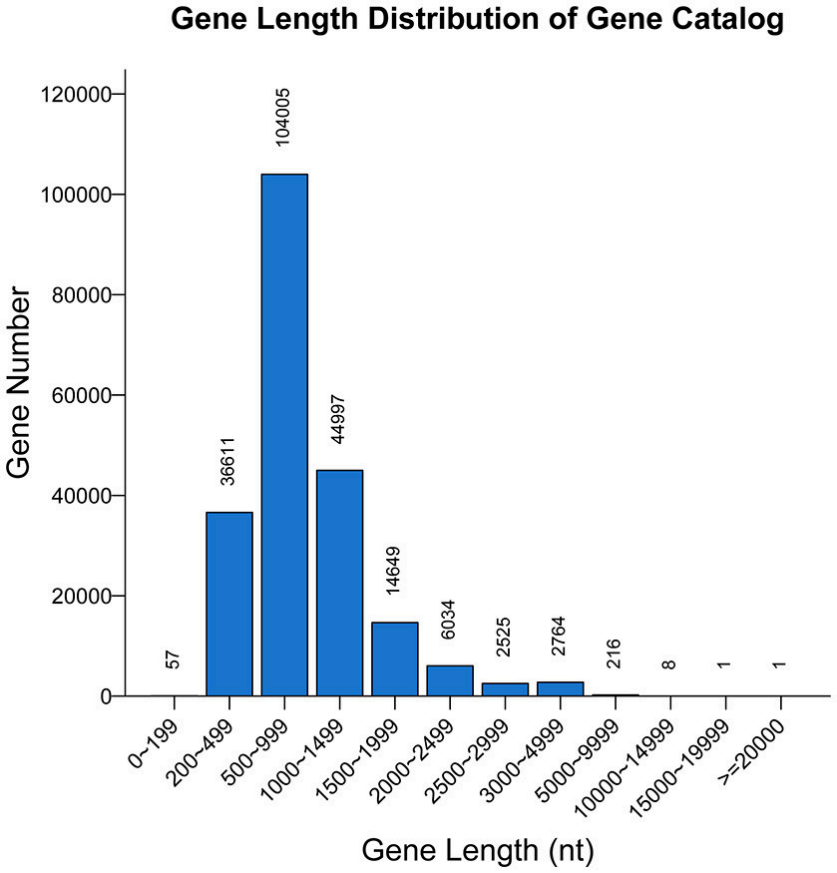
Length distribution of genes. The X-axis indicates the length intervals, and the Y-axis represents the number of genes.

A non-redundant catalog of 211,868 genes was constructed. The distribution of the lengths of the genes is shown in Figure 1. The numbers of genes in each sample were as follows: A-1, 128,854; A-2, 107,488; B-1, 130,868; and B-2, 79,124. To obtain information on gene function, the genes were blasted separately against the ARDB, CAZy, GO, KEGG, Swiss-Prot, and nr databases. Overall, a total of 202,939 genes matched the six databases. A total of 200,380 genes were annotated in the nr database, followed by 74,273, 153,664, 109,337, 8,535, and 247 genes annotated in the Swiss-Prot, GO, KEGG, CAZy, and ARDB databases, respectively (File S1).

### Analysis of KEGG pathways

KEGG metabolic pathways provide a description of the metabolism of *M. javanica* gut cells (Figure S3). A total of 41,711 genes were associated with 147 unique KEGG metabolic pathways, suggesting that the gut microbiome of *M. javanica* has been involved in the metabolism of carbohydrates (14,134 genes), amino acids (9,936 genes), vitamins (7,244 genes), nucleotides (5,576 genes), lipids (3,496 genes), glycans (2,975 genes), and xenobiotics (1,643 genes). Some genes were associated with global and overview maps (34,796 genes), energy metabolism (6,797 genes), and the metabolism of other amino acids (2,833 genes), whereas some of the genes were relevant to secondary metabolite biosynthesis (1,832 genes) and terpenoid and polyketide metabolism (1,565 genes) (File S2).

### Carbohydrate metabolism

**Figure 2:**
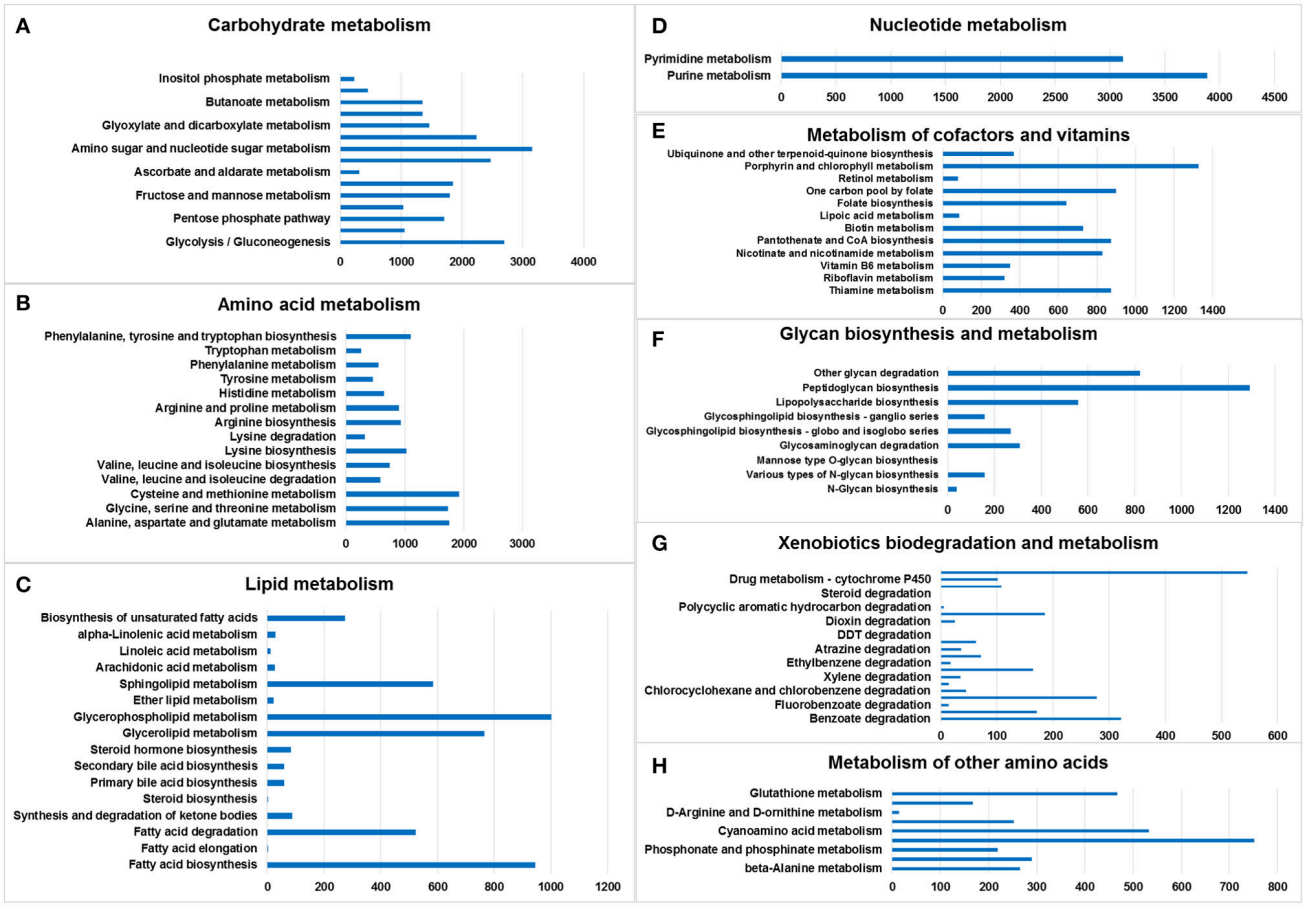
Metabolic pathway analysis of genes. The X-axis shows the numbers of annotated genes in one class, and the Y-axis shows the KEGG functional classes. **(A)** Carbohydrate metabolism, **(B)** amino acid metabolism, **(C)** lipid metabolism, **(D)** nucleotide metabolism, **(E)** metabolism of cofactors and vitamins, **(F)** glycan biosynthesis and metabolism, **(G)** xenobiotics biodegradation and metabolism, and **(H)** metabolism of other amino acids.

Amino sugar and nucleotide sugar metabolism (3,155 genes), glycolysis/gluconeogenesis (2,690 genes), starch and sucrose metabolism (2,471 genes), and pyruvate metabolism (2,235 genes) accounted for the largest number of genes in the lists, whereas inositol phosphate metabolism (228 genes) was associated with the smallest number of genes (Figure 2A).

The chitin-degrading enzyme in the insect cuticle and the peritrophic membrane of ants was found in the pathway of amino sugar and nucleotide sugar metabolism (KEGG: 00520), suggesting that the gut microbiome of *M. javanica* may utilize this pathway to facilitate the digestion of ants (Figure S4).

### Amino acid metabolism

Cysteine and methionine metabolism (1,926 genes); alanine, aspartate, and glutamate metabolism (1,757 genes); glycine, serine and threonine metabolism (1,735 genes); and phenylalanine, tyrosine, and tryptophan biosynthesis (1,096 genes) accounted for the largest number of genes in the amino acid metabolism list. Tryptophan metabolism was associated with the smallest number of genes (Figure 2B).

### Lipid metabolism

Glycerophospholipid metabolism (1,003 genes), fatty acid biosynthesis (945 genes), glycerolipid metabolism (766 genes), and sphingolipid metabolism (584 genes) included the largest number of genes in the lipid metabolism list; in contrast, fatty acid elongation (1 gene) and steroid biosynthesis (1 gene) involved the smallest number of genes (Figure 2C).

### Nucleotide metabolism

Only two pathways were listed: purine metabolism (3,885 genes) and pyrimidine metabolism (3,118 genes) (Figure 2D).

### Metabolism of cofactors and vitamins

Porphyrin and chlorophyll metabolism (1,326 genes), one carbon pool by folate (900 genes), thiamine metabolism (875 genes), and pantothenate and CoA biosynthesis (875 genes) accounted for the largest number of genes in the cofactor and vitamin metabolism list. The smallest number was related to retinol metabolism (78 genes) (Figure 2E).

### Glycan biosynthesis and metabolism

Peptidoglycan biosynthesis (1,291 genes), other glycan degradation (824 genes), lipopolysaccharide biosynthesis (557 genes), and glycosaminoglycan degradation (308 genes) accounted for the largest number of genes in the glycan biosynthesis and metabolism list. Mannose type O-glycan biosynthesis (1 gene) involved the smallest number of genes. No genes were found in the following categories: mucin type O-glycan biosynthesis, other types of O-glycan biosynthesis, glycosaminoglycan biosynthesis—chondroitin sulfate/dermatan sulfate,—heparan sulfate/heparin, —keratan sulfate, glycosylphosphatidylinositol (GPI)—anchor biosynthesis, and glycosphingolipid biosynthesis—lacto and neolacto series (Figure 2F).

### Xenobiotics biodegradation and metabolism

Drug metabolism—other enzymes (546 genes), benzoate degradation (321 genes), chloroalkane and chloroalkene degradation (278 genes), and naphthalene degradation (185 genes) accounted for the largest number of genes in the xenobiotic biodegradation and metabolism list. Steroid degradation (1 gene) involved the smallest number of genes (Figure 2G).

### Metabolism of other amino acids

There were six lists, namely selenocompound metabolism (752 genes), cyanoamino acid metabolism (533 genes), glutathione metabolism (467 genes), taurine and hypotaurine metabolism (290 genes), beta-alanine metabolism (265 genes), and D-glutamine and D-glutamate metabolism (252 genes) (Figure 2H).

### Gene ontology (GO)

There were 153,664 genes classified into 54 small classes in three ontology categories, namely biological process, molecular function, and cellular component, when the *M. javanica* gut microbiome genes were annotated with the GO database.

Under biological process, the terms with the largest numbers of genes were metabolic process (88,850), cellular process (79,805), and single-organism process (64,590). The fourth most abundant process was biological regulation (17,948), which was followed by regulation of biological process (17,277), response to stimulus (12,030), cellular component organization or biogenesis (5,688), and signaling (5,108). The terms associated with metabolic process and biological regulation may indicate the involvement of the gut microbiome of *M. javanica* in varied digestive activities.

In the ontology category of molecular function, the sequences were mainly assigned to catalytic activity (95,050) and binding (67,865), followed by transporter activity (15,375), nucleic acid binding transcription factor activity (6,135), receptor activity (4,936), and molecular transducer activity (3,105), which might be associated with food digestion and absorption.

**Figure 3:**
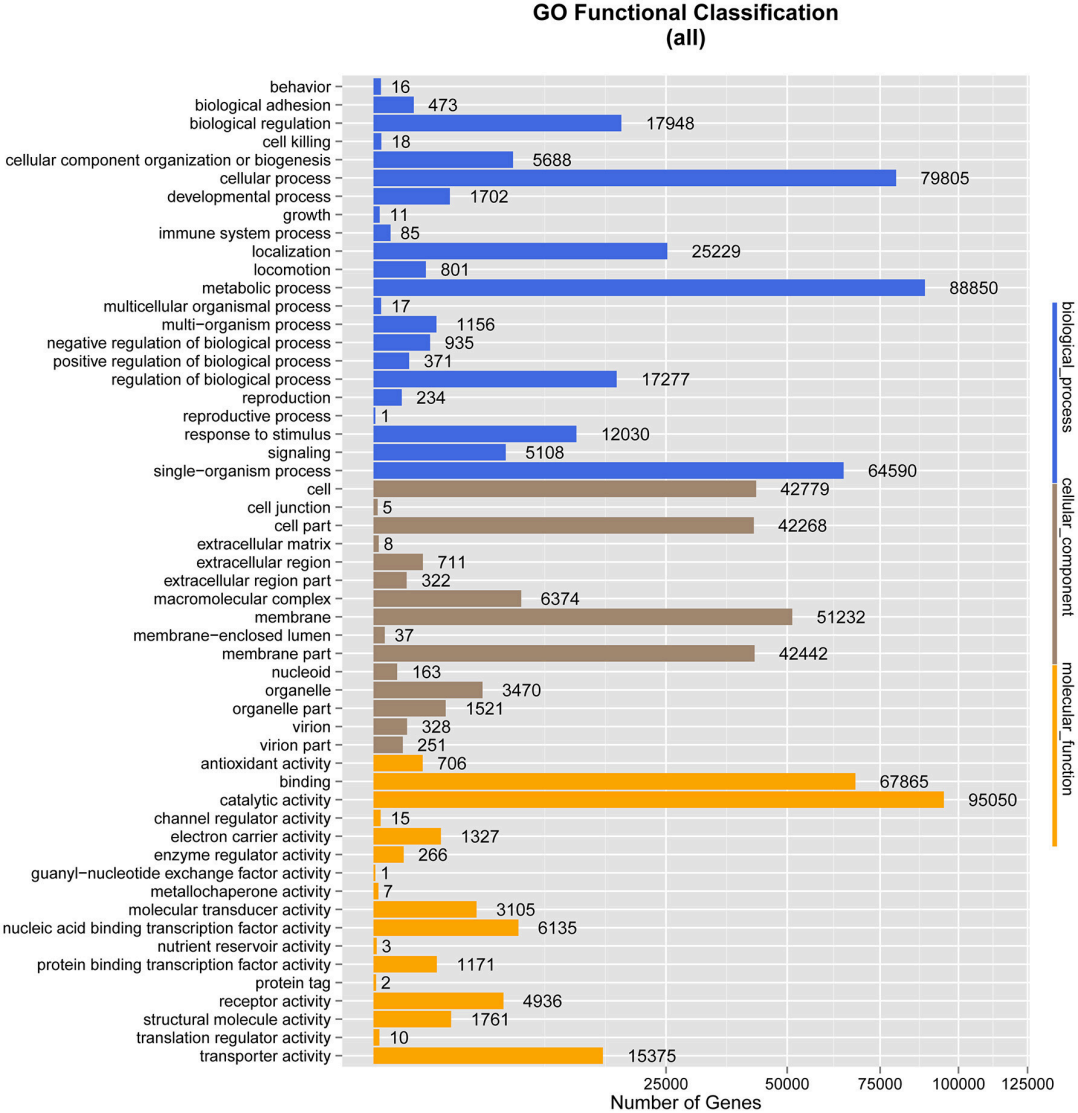
Gene Ontology (GO) classification analysis of genes. The X-axis shows the GO function classes. The right side of the Y-axis shows the number of genes having that GO function, and the left side shows the percentage.

As anticipated, membrane (51,232), cell (42,779), and cell part (42,268) were the predominant terms under the cellular component category, followed by membrane part (42,442), macromolecular complex (6,374), and organelle (3,470) (Figure 3).

### Carbohydrate-active enZYmes (CAZy) database

Four main kinds of enzymes are related to carbohydrate degradation: glycoside hydrolases (GHs), glycosyl transferases (GTs), polysaccharide lyases (PLs), and carbohydrate esterases (CEs). In addition, carbohydrate-binding modules (CBMs), representing adhesion to carbohydrates, were also included. These analyses found 5,044 genes from 88 different GH families, 2,309 genes from 31 different GT families, 165 genes from 14 PL families, 619 genes from 13 CE families, and 989 genes from 33 CBM families in the CAZy database (Table 3).

**Table 3:** Enzymes related to carbohydrate degradations.

**Carbohydrate-Active Enzymes**	**Gene family**
Glycoside Hydrolases (GHs)	GH0,GH1,GH10,GH101,GH102,GH103,GH104,GH105,GH106,GH108,GH109,GH110,GH112,GH113,GH115,GH116,GH117,GH12,GH120,GH123,GH125,GH126,GH127,GH128,GH129,GH13,GH130,GH15,GH16,GH17,GH18,GH19,GH2,GH20,GH23,GH24,GH25,GH26,GH27,GH28,GH29,GH3,GH30,GH31,GH32,GH33,GH35,GH36,GH37,GH38,GH39,GH4,GH42,GH43,GH48,GH5,GH50,GH51,GH53,GH55,GH57,GH59,GH63,GH65,GH66,GH67,GH73,GH74,GH76,GH77,GH78,GH79,GH8,GH81,GH84,GH85,GH88,GH89,GH9,GH90,GH91,GH92,GH93,GH94,GH95,GH97,GH98,GH99
Glycosyl Transferases (GTs)	GT9,GT11,GT35,GT4,GT2,GT56,GT8,GT28,GT0,GT51,GT32,GT83,GT5,GT14,GT20,GT26,GT30,GT19,GT1,GT44,GT25,GT3,GT6,GT23,GT39,GT66,GT73,GT10,GT63,GT84,GT41
Polysaccharide Lyases (PLs)	PL0,PL1,PL10,PL11,PL12,PL13,PL15,PL17,PL21,PL5,PL6,PL7,PL8,PL9
Carbohydrate Esterases (CEs)	CE0,CE1,CE10,CE11,CE12,CE14,CE15,CE2,CE4,CE6,CE7,CE8,CE9
Carbohydrate-Binding Modules (CBMs)	CBM0,CBM12,CBM13,CBM18,CBM2,CBM20,CBM22,CBM23,CBM25,CBM26,CBM27,CBM3,CBM32,CBM33,CBM34,CBM35,CBM37,CBM4,CBM40,CBM41,CBM46,CBM47,CBM48,CBM5,CBM50,CBM51,CBM56,CBM57,CBM58,CBM59,CBM6,CBM61,CBM9

*There are mainly four kinds of enzymes related to carbohydrate degradations: Glycoside Hydrolases (GHs), Glycosyl Transferases (GTs), Polysaccharide Lyases (PLs), Carbohydrate Esterases (CEs). In addition, Carbohydrate-Binding Modules (CBMs) representing adhesion to carbohydrates are also included*.

More than 100 gene modules involved in cellulose hydrolysis were found in our dataset, including the catalytic domains of GH5 cellulases, GH51 endoglucanase/arabinofuranosidases, GH94 cellobiose or cellodextrin phosphorylases, and a smaller number of GH48, GH74, GH8, and GH9 endoglucanases. By comparison, gene modules relevant to the catalytic domains of important components of several well-studied microbial cellulase systems (e.g., GH44, GH45, and GH6 endoglucanases/cellobiohydrolases) were absent.

A total of 114 gene modules were found in our dataset corresponding to the catalytic domains of the GH18 family enzymes, including chitinase genes of classes III and V. Fourteen gene modules corresponded to the catalytic domains of GH19 family enzymes, including chitinase genes of classes I, II, and IV.

### Taxonomic statistics

**Figure 4:**
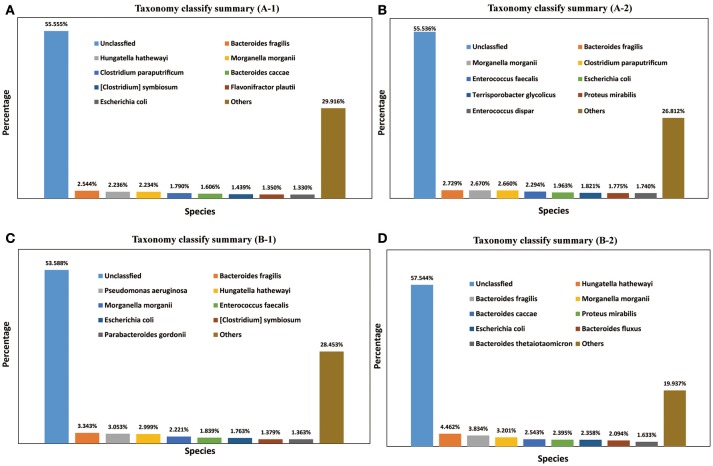
Taxonomy classification summary. The top 8 annotations in four *Manis javanica* at species level are shown in different colors. **(A)** Taxonomy classify summary (A-1), **(B)** taxonomy classify summary (A-2), **(C)** taxonomy classify summary (B-1), and **(D)** taxonomy classify summary (B-2).

A total of 1,811 microbe species were found. The number of microbial species in each individual was 886, 1,157, 1,237, and 1,355 (File S3). Many strains belonged to the following genera: *Bacteroides* (59 species), *Clostridium* (57 species), *Bacillus* (48 species), *Streptococcus* (48 species), *Lactobacillus* (42 species), *Paenibacillus* (41 species), *Prevotella* (41 species), *Bifidobacterium* (33 species), *Enterococcus* (33 species), *Pseudomonas* (26 species), *Lachnospiraceae* (20 species), and *Vibrio* (20 species). The dominant species in all four *M. javanica* samples were *Bacteroides caccae, B. fluxus, B. fragilis, B. thetaiotaomicron, Clostridium paraputrificum, C. symbiosum, Enterococcus dispar, E. faecalis, Escherichia coli, Flavonifractor plautii, Hungatella hathewayi, Morganella morganii, Parabacteroides gordonii, Proteus mirabilis, Pseudomonas aeruginosa*, and *Terrisporobacter glycolicus*. The specific dominant species in the two healthy individuals were *C. paraputrificum* and *T. glycolicus*, while *P. aeruginosa* and *P. gordonii* were the dominant species in the two diseased individuals (Figure 4). We also identified a number of species in the genera *Cellulosimicrobium, Arthrobacter, Chitinimonas, Chitinophaga, Enterobacter, Klebsiella, Staphylococcus*, and *Lactococcus* (Table 4) that probably play a role in cellulose digestion and may degrade chitin. Several of these species, including *B. fragilis, E. coli, B. fluxus, B. caccae, B. thetaiotaomicron*, and *B. ovatus*, were prevalent in all four samples.

**Table 4:** Microbe species related to cellulose digestion or chitin degradation.

**Genus**	**Species**
*Cellulosimicrobium*	*Cellulosimicrobium cellulans*
*Chitinimonas*	*Chitinimonas koreensis*
*Chitinophaga*	*Chitinophaga pinensis*
*Enterobacter*	*Enterobacter cloacae*
*Klebsiella*	*Klebsiella oxytoca*
	*Klebsiella pneumoniae*
*Staphylococcus*	*Staphylococcus aureus*
	*Staphylococcus epidermidis*
	*Staphylococcus haemolyticus*
	*Staphylococcus hyicus*
	*Staphylococcus phage Twort*
	*Staphylococcus sciuri*
*Lactococcus*	*Lactococcus lactis*

A total of 37 species belonging to four phyla (Bacteroidetes, Cyanobacteria, Firmicutes, and Proteobacteria) contained 58 gene modules of GH18 and GH19; 13 *Bacteroides* species are shown in Table 5, and the sequences of the genes are shown in File S4.

**Table 5:** Microbe species related to genes of chitin digestion.

**Gene_id**	**Sepcies**
[denovogenes]_68467	*[Clostridium] cellulolyticum*
[denovogenes]_151116, [denovogenes]_74830	*[Ruminococcus] gnavus*
[denovogenes]_42081	*Anaerostip*es sp. 3_2_56FAA
[denovogenes]_61326	*Bacteroides caccae*
[denovogenes]_79414	*Bacteroides faecis*
[denovogenes]_17967	*Bacteroides fluxus*
[denovogenes]_61119, [denovogenes]_64063, [denovogenes]_136569	*Bacteroides fragilis*
[denovogenes]_55755	*Bacteroides helcogenes*
[denovogenes]_66063	*Bacteroides massiliensis*
[denovogenes]_61999, [denovogenes]_76501	*Bacteroides ovatus*
[denovogenes]_121873, [denovogenes]_55512	*Bacteroides* sp. *D2*
[denovogenes]_20169, [denovogenes]_56831	*Bacteroides* sp. *D22*
[denovogenes]_21169, [denovogenes]_24780, [denovogenes]_55984, [denovogenes]_61438, [denovogenes]_65266, [denovogenes]_82405	*Bacteroides thetaiotaomicron*
[denovogenes]_100953	*Bacteroides uniformis*
[denovogenes]_60040	*Bacteroides vulgatus*
[denovogenes]_55052	*Bacteroides xylanisolvens*
[denovogenes]_134103	*Butyricicoccus pullicaecorum*
[denovogenes]_106655	*Cellulosilyticum lentocellum*
[denovogenes]_7860	*Citrobacter rodentium*
[denovogenes]_43771	*Citrobacter werkmanii*
[denovogenes]_101794, [denovogenes]_116160	*Clostridiales bacterium 1_7_47FAA*
[denovogenes]_90393	*Clostridioides difficile*
[denovogenes]_84094, [denovogenes]_98045	*Clostridium botulinum*
[denovogenes]_93205	*Clostridium celatum*
[denovogenes]_28627, [denovogenes]_5543, [denovogenes]_67187	*Clostridium paraputrificum*
[denovogenes]_64813, [denovogenes]_78835	*Enterococcus faecalis*
[denovogenes]_34460	*Epulopiscium* sp. ‘*N.t. morphotype B'*
[denovogenes]_4353	*Escherichia coli*
[denovogenes]_19914, [denovogenes]_20262	*Flavonifractor plautii*
[denovogenes]_115156, [denovogenes]_143438	*Heliobacillus mobilis*
[denovogenes]_121842	*Hungatella hathewayi*
[denovogenes]_77129	*Lachnoclostridium phytofermentans*
[denovogenes]_41394	*Lachnospiraceae bacterium 7_1_58FAA*
[denovogenes]_119305	*Nodosilinea nodulosa*
[denovogenes]_28742, [denovogenes]_139898, [denovogenes]_139972, [denovogenes]_142955	*Pseudomonas aeruginosa*
[denovogenes]_139146	*Ruminococcaceae bacterium D16*
[denovogenes]_67050	*Salmonella enterica*

### Differential analysis of gene abundance

**Figure 5:**
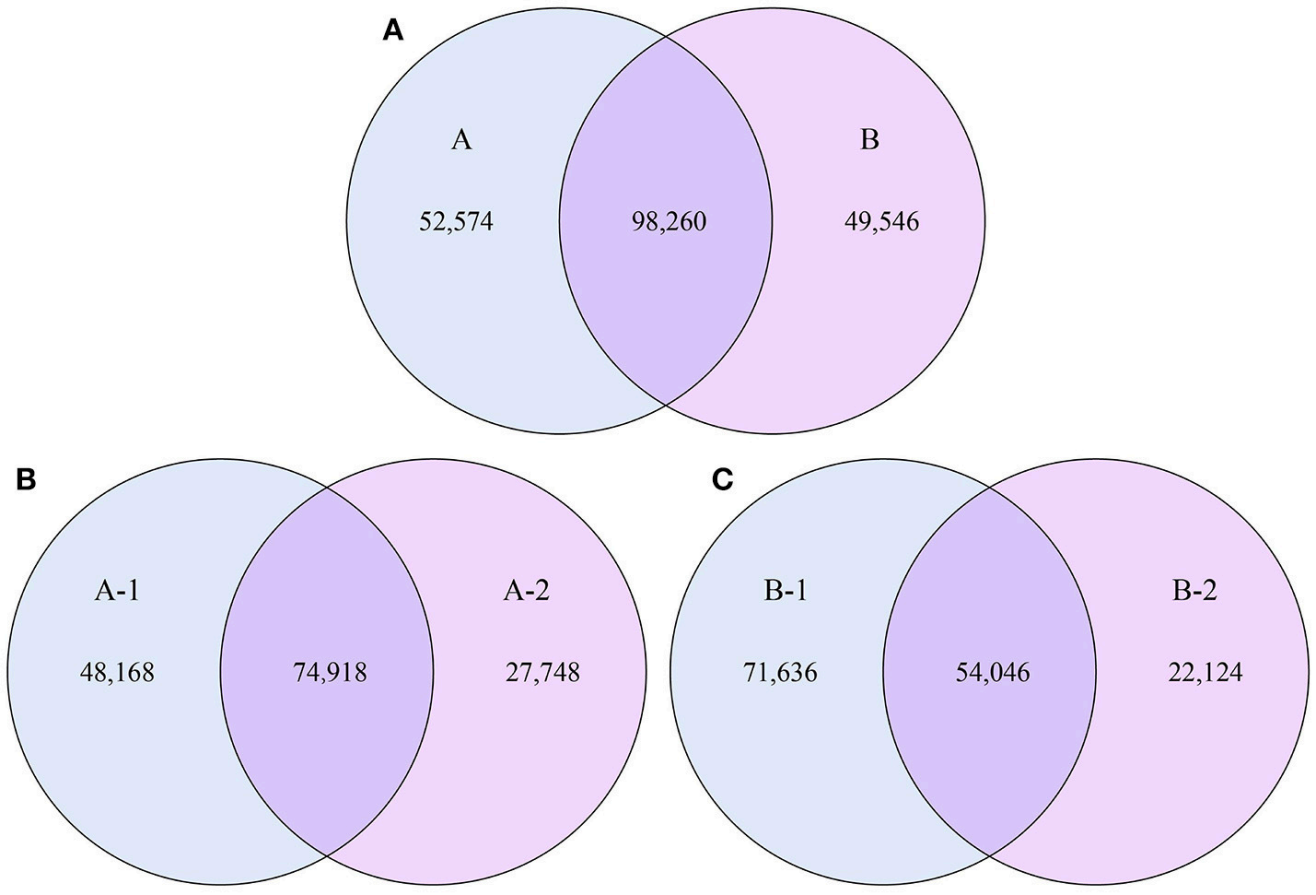
Venn diagrams. The numbers of genes that were shared or not shared by the four individuals, depending on overlaps, are shown. For this presentation, two individuals had to be combined, thus affecting the number of genes shared by both individuals. **(A)** The number of genes shared by groups A and B. **(B)** The number of genes shared by the two individuals of group A. **(C)** The number of genes shared by the two individuals of group B.

To examine the similarities between groups A and B, the relative abundances of genes and their corresponding functions in the different samples were calculated. A total of 98,260 genes were abundant in the two groups. The results of the differential analysis of gene abundance showed that 52,574 genes were more abundant in group A than in group B, while 49,546 were more abundant in group B than in group A. A total of 74,918 genes were abundant in both samples of group A, while 54,046 genes were abundant in both samples of group B (Figure 5).

**Figure 6:**
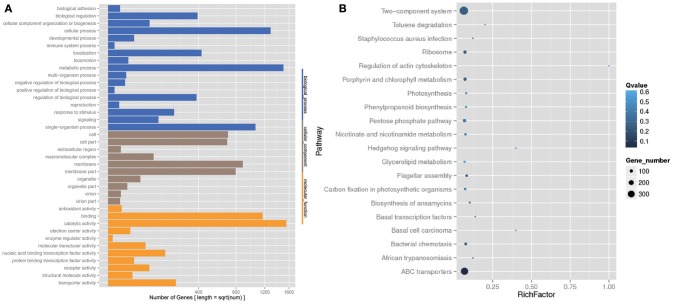
KEGG pathway and Gene Ontology (GO) classification analysis of genes with different abundance between the group A and B samples. **(A)** GO functional classification. **(B)** Top 20 statistics of pathway enrichment for A group vs. B group.

**Figure 7:**
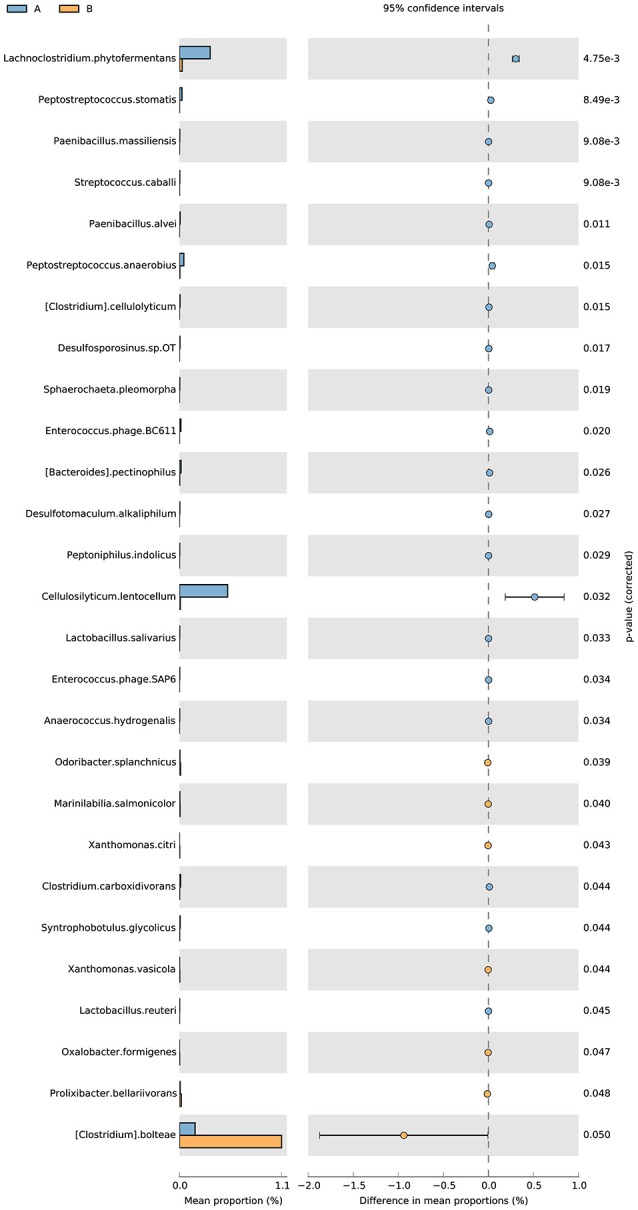
Metagenomic profile comparisons of differentially abundant species between the group A and B samples using STAMP analysis. A positive difference between proportions denotes a greater abundance in group A (orange), whereas a negative difference between proportions shows a greater abundance in group B (orange) for the given gene. Corrected *P*-values (*q*-values) were calculated based on G Test (w + Yates) + Fisher's statistical test using Story's FDR approach. Features with *q* < 0.05 were considered significant and were thus retained.

The analysis of the function of these genes with differential abundance was carried out based on the GO and KEGG databases. The top terms in the GO database were catalytic activity and metabolic process (Figure 6A). For the analysis of KEGG pathways, two-component system and ABC transporters were the most enriched pathways (Figure 6B). A STAMP analysis was also used to establish the relative proportions of the various species between groups A and B. The STAMP analysis revealed 27 species that were differentially abundant between the two groups. Twenty species had greater abundance in healthy pangolin individuals; these species were *Anaerococcus hydrogenalis, Bacteroides pectinophilus, Cellulosilyticum lentocellum, Clostridium carboxidivorans, C. cellulolyticum, Desulfotomaculum alkaliphilum, Desulfosporosinus sp. OT, Enterococcus phage SAP6, E. phage BC611, Lachnoclostridium phytofermentans, Lactobacillus salivarius, L. reuteri, Paenibacillus alvei, P. massiliensis, Peptostreptococcus anaerobius, P. stomatis, Peptoniphilus indolicus, Sphaerochaeta pleomorpha, Streptococcus caballi*, and *Syntrophobotulus glycolicus*, among which *C. lentocellum* and *L. reuteri* were the most abundant. In contrast, seven species, namely *Odoribacter splanchnicus, Marinilabilia salmonicolor, X. citri, Xanthomonas vasicola, Oxalobacter formigenes, Prolixibacter bellariivorans*, and *C. bolteae*, had greater abundance in diseased pangolin individuals (Figure 7).

## Discussion

In this work, we present the first Illumina GA short-read-based sequencing of total fecal DNA from a cohort of four pangolins—two healthy and two diseased individuals—to obtain a catalog of non-redundant intestinal microbial genes. The sample size was limited to two individuals for each study group due to the difficulty of obtaining samples from this critically endangered mammal, and particularly from diseased individuals. The differences between the two groups were analyzed using STAMP software. A two-sided Welch's *t*-test was used for the two-group analysis. Some of our results are preliminary, and more samples are needed for further studies. The resulting catalog contains 211,868 microbial genes and constitutes a primary source to determine the role of the intestinal bacterial community in myrmecophagy. Our results represent a proof of principle, as they can be used to characterize complex microbiomes of different physical origins.

We predicted 1,811 metagenomic species in the intestinal microbiome from fecal samples. In *M. javanica*, the three main phyla were Bacteroidetes, Proteobacteria, and Firmicutes. This result was similar to that of previous studies on myrmecophages, for which the predominant phyla were Firmicutes, Bacteroidetes, and Proteobacteria (Delsuc et al., 2014), whereas Firmicutes are mostly predominant in Carnivora (Delsuc et al., 2014). In anteaters, the gut microbiomes seem to have a high abundance of Proteobacteria (Delsuc et al., 2014), consistent with those in *M. javanica*. This result was also generally consistent with the results of Sanders' studies, which showed by 16S RNA analysis that Bacteroidetes and Firmicutes dominated the intestinal microflora of baleen whales feeding on crustacean shells (Sanders et al., 2015). Crustacean shells are chitin-rich food, similar to the diet of *M. javanica*.

The results of the analyses of the chitinase gene of the gut microbiota of the pangolins indicated that some of the gut microbiota, such as species of *Bacteroides* (Borrelli et al., 2017), *Clostridiales* (Sebaihia et al., 2007), and *Enterococcus faecalis* (Leisner et al., 2009; Vaaje-Kolstad et al., 2012) could potentially function in chitin degradation.

Several species were found in the intestinal microflora of the pangolins that may play a role in cellulose digestion (Schumann et al., 2001; Bakalidou et al., 2002). The first of these species is *Cellulosimicrobium cellulans*, which was originally isolated from Antarctic snow (Antony et al., 2009). In a second group were the *Arthrobacter* species, which have been found in various environmental and animal-derived samples, such as Antarctic marine sediment (Pindi et al., 2010), penguin guano (Wang et al., 2009), soil (Zhang et al., 2010), sewage (Kim et al., 2008), seawater (Chen et al., 2009), and the nasal passages of the common seal (Collins et al., 2002). One of the *Arthrobacter* species was *Arthrobacter psychrochitiniphilus*, which was originally isolated from penguin guano (Wang et al., 2009). This organism can degrade both cellulose and chitin. However, the bacterium *A. nitrophenolicus*, which was also found in our samples, has been reported to degrade only 2-chloro-4-nitrophenol (Arora and Jain, 2013). In the third group were the *Enterobacter* species, including *E. asburiae, E. cancerogenus, E. cloacae, E. sp. 638, E. sp. DC1*, and *E. sp. R4-368*.

Some *Enterobacter* species, including *E. aerogenes* (Brzezinska, 2006), *E. agglomerans* (Chernin et al., 1995), *E. spp. strain NRG4* (Neufeld, 2005), as well as, *E. cloacae* (Lorito et al., 1993), have previously been shown to have chitin-degrading activity, which was also found in our samples. Moreover, numerous *Klebsiella* species appeared in our results, including *K. oxytoca*, which has been identified as having cellulolytic activity (Prem Anand and Sripathi, 2004; Anand et al., 2010). Finally, some *Staphylococcus species* (Wadström, 1971) and *Lactococcus lactis* (Vaajekolstad et al., 2009) found in our samples have also been reported to have the ability to degrade chitin.

For the 179 CAZy families, some GH families, including the GH18 family associated with the degradation of chitin (Xu et al., 2004), were abundant in the metagenomes of *M. javanica*. Genes related to the carbohydrate-binding module of the CBM37 family were also found, and the encoded proteins are described as enzymes associated with chitin binding. However, this module has only been described from the bacterial genus *Ruminococcus* (Ezer et al., 2008). Together with chitinase GH18, these CAZymes might help to digest chitin in *M. javanica*.

Our results also showed that *M. javanica* gut microbes, similar to herbivore microbiomes, are able to metabolize carbohydrates and amino acids (Sanders et al., 2015), reflecting the ability of the microbial communities of the gut to ferment polysaccharides. The chitinous exoskeletons of termites and ants comprise the major source of carbohydrate in the pangolin's diet, making up as much as 10% of the total calories present in these insects (Clarke, 1980). *M. javanica* microbiomes were also found to have abundant genes in the metabolic pathways of amino sugar and nucleotide sugar (KEGG: 00520), and these genes might be directly involved in chitin digestion. A large number of genes were assigned to the metabolism of D-glutamine and D-glutamate. Glutamate metabolism is one of the signature pathways that distinguish microbiomes exposed to preponderantly animal-based diets from those exposed to plant-based diets in humans (David et al., 2014) and mammals generally (Muegge et al., 2011). All these genes suggest a predominantly catabolic direction of protein metabolism in the animal-based diet of *M. javanica*.

Many studies have shown that the gut microbiota is related to a variety of diseases. In our study, the specific dominant species in the two healthy individuals were *Clostridium paraputrificum* and *Terrisporobacter glycolicus*, while *Pseudomonas aeruginosa* and *Parabacteroides gordonii* were the dominant species in the two diseased individuals. *Lactobacillus salivarius* and *L. reuteri* were more abundant in the healthy individuals, and these lactic acid bacteria are frequently used as probiotic agents. *L. reuteri*, one of the few endogenous *Lactobacillus* species, is found in the gastrointestinal tracts of vertebrates, such as humans, rats, pigs and chickens (Hou et al., 2015), and the use of *L. salivarius* as a probiotic in humans and other animals has addressed safety concerns with its use on live hosts (Chaves et al., 2017). *Cellulosilyticum lentocellum*, a cellulolytic bacterium, was also found to be abundant in samples from the healthy pangolins and might contribute to their health. However, two potential pathogens, *Clostridium bolteae* and *O. formigenes*, were found in this study. *O. formigenes* has been reported to be involved in kidney diseases in humans and other animals (Baloglu and Turkmen, 2018), and *C. bolteae* was found to increase during acute liver rejection (Ren et al., 2014). Although these species may not directly cause the observed diseases, they may have some influence on the death of the host.

Although sequencing of metagenomes can compensate for the deficiencies of pure culture technology, the analysis of the metagenome itself can provide information about the composition and function of the intestinal microbiome. The microbial genes expressed and their particular functions remain unknown. With the development of sequencing technology, much more detailed expression and functional information of protein-coding genes can be obtained through the metatranscriptome or metaproteome.

Here, we report the first *M. javanica* microbiota characterization using the method of metagenomic sequencing. Our findings offer an in-depth look at the microbiome of *M. javanica*, an animal with an endangered status and an unusually narrow dietary niche. This dietary restriction explains the putative harboring of cellulose and chitin-digesting microbes in the gut of *M. javanica*, together with a suite of chitinase genes, including some in the GH18 family. We also detected microbial species associated with chitin degradation and diseases. Finally, this is the first study of the gut microbiome in pangolins. Due to the deficient understanding of the functions of the microbial genes and species associated with *M. javanica*, further research is needed to gain deeper knowledge of their relationships with health and disease.

## Ethics statement

Full name of the ethics committee that approved the study: the Guangdong Institute of Applied Biological Resources.

Consent procedure used for human participants or for animal owners.

1. Does laboratory animal must be used in the project? Could other methods such as computer simulation, cell culture or using the low-grade animal instead of the high-grade animal?
  Yes. The laboratory animal must be used in this project. The other methods can't complete the experiment.
2. Are the qualification of applicant, species or strain, grade and specifications of animals suitable? Could the quantity of animals be reduced by improving the study design or using high quality animals?
  The qualification of applicant, species or strain, grade, and specifications of animals are suitable. The quantity of animals could not be reduced by improving the study design or using high quality animals.
3. Could the study design and animal treatment be refined by ameliorating experimental method, adjusting observational index or executing animal method?

We did no damage to the simples in our study.

## Author contributions

J-EM designed the assays and wrote drafted paper. L-ML, H-YJ, X-JZ, X-JJ prepared the materials. H-ML and G-YL analyzed data. J-PC designed the assays and revised drafted paper.

### Conflict of interest statement

The authors declare that the research was conducted in the absence of any commercial or financial relationships that could be construed as a potential conflict of interest.

## References

[B1] Anand A. A. Vennison S. J. Sankar S. G. Prabhu D. I. Vasan P. T. Raghuraman T.. (2010). Isolation and characterization of bacteria from the gut of *Bombyx mori* that degrade cellulose, xylan, pectin and starch and their impact on digestion. J. Insect Sci. 10:107. 10.1673/031.010.10701 20874394 PMC3016902

[B2] Angelakis E. Yasir M. Bachar D. Azhar E. I. Lagier J. C. Bibi F.. (2016). Gut microbiome and dietary patterns in different Saudi populations and monkeys. Sci. Rep. 6:32191. 10.1038/srep32191 27578328 PMC5006041

[B3] Antony R. Krishnan K. P. Thomas S. Abraham W. P. Thamban M. (2009). Phenotypic and molecular identification of *Cellulosimicrobium cellulans* isolated from Antarctic snow. Antonie Van Leeuwenhoek 96, 627–634. 10.1007/s10482-009-9377-9 19760124

[B4] Arora P. K. Jain R. K. (2013). *Arthrobacter nitrophenolicus* sp. nov . a new 2-chloro-4-nitrophenol degrading bacterium isolated from contaminated soil. Biotech 3, 29–32. 10.1007/s13205-012-0066-4 28324343 PMC3563742

[B5] Bakalidou A. Kampfer P. Berchtold M. Kuhnigk T. Wenzel M. Konig H. (2002). Cellulosimicrobium variabile sp. nov., a cellulolytic bacterium from the hindgut of the termite Mastotermes darwiniensis. Int. J. Syst. Evol. Microbiol. 52, 1185–1192. 10.1099/00207713-52-4-1185 12148626

[B6] Baloglu I. Turkmen K. (2018). The importance of *Oxalobacter formigenes* and oxalic acid in the pathogenesis of chronic kidney disease. Int. Urol. Nephrol. 50, 1189–1189 10.1007/s11255-018-1848-3 29556902

[B7] Bao F. Wu S. Su C. Yang L. Zhang F. Ma G. (2013). Air temperature changes in a burrow of Chinese pangolin, *Manis pentadactyla*, in Winter. Folia Zool. Praha 62, 42–47. 10.25225/fozo.v62.i1.a6.2013

[B8] Borrelli L. Coretti L. Dipineto L. Bovera F. Menna F. Chiariotti L.. (2017). Insect-based diet, a promising nutritional source, modulates gut microbiota composition and SCFAs production in laying hens. Sci. Rep. 7:16269. 10.1038/s41598-017-16560-6 29176587 PMC5701250

[B9] Boursier J. Mueller O. Barret M. Machado M. Fizanne L. Araujo-Perez F.. (2016). The severity of nonalcoholic fatty liver disease is associated with gut dysbiosis and shift in the metabolic function of the gut microbiota. Hepatology 63, 764–775. 10.1002/hep.28356 26600078 PMC4975935

[B10] Brucker R. M. Bordenstein S. R. (2013). The hologenomic basis of speciation: gut bacteria cause hybrid lethality in the genus Nasonia. Science 341, 667–669. 10.1126/science.1240659 23868918

[B11] Brzezinska M. S. (2006). Chitinolytic bacteria in two lakes of different trophic status. Poljecol 54, 295–301.

[B12] Cani P. D. Bibiloni R. Knauf C. Waget A. Neyrinck A. M. Delzenne N. M.. (2008). Changes in gut microbiota control metabolic endotoxemia-induced inflammation in high-fat diet-induced obesity and diabetes in mice. Diabetes 57, 1470–1481. 10.2337/db07-1403 18305141

[B13] Cani P. D. Knauf C. (2016). How gut microbes talk to organs: the role of endocrine and nervous routes. Mol. Metabol. 5, 743–752. 10.1016/j.molmet.2016.05.011 27617197 PMC5004142

[B14] Challender D. Nguyen Van T. Shepherd C. Krishnasamy K. Wang A. Lee B. (2014). Manis javanica. the IUCN Red List of Threatened Species 2014. Version 2017-1. Available online at: http://www.iucnredlist.org/details/12763/0 (Accessed August 15, 2017).

[B15] Chaves B. D. Brashears M. M. Nightingale K. K. (2017). Applications and safety considerations of *Lactobacillus salivarius* as a probiotic in animal and human health. J. Appl. Microbiol. 123, 18–28. 10.1111/jam.13438 28256040

[B16] Chen Y. G. Tang S. K. Zhang Y. Q. Li Z. Y. Yi L. B. Wang Y. X.. (2009). *Arthrobacter halodurans* sp. nov., a new halotolerant bacterium isolated from sea water. Antonie Van Leeuw. 96, 63–70. 10.1007/s10482-009-9336-5 19337850

[B17] Chernin L. Ismailov Z. Haran S. Chet I. (1995). Chitinolytic cagglomerans antagonistic to fungal plant pathogens. Appl. Environ. Microbiol. 61, 1720–1726. 16535017 10.1128/aem.61.5.1720-1726.1995 PMC1388435

[B18] Chin S. C. Lien C. Y. Chan Y. T. Chen C. L. Yang Y. C. Yeh L. S. (2012). Monitoring the gestation period of rescued formosan pangolin (*Manis pentadactyla pentadactyla*) with progesterone radioimmunoassay. Zoo Biol. 31, 479–489. 10.1002/zoo.20413 21866570

[B19] Chin S. C. Liu C. H. Guo J. C. Chen S. Y. Yeh L. S. (2006). “*A 10-year review of autopsy of rescued Formosan pangolin (Manis pentadactyla pentadactyla) in* Taipei zoo,” in Proceedings of AZWMP 2006 (Bangkok: Chulalongkorn University Faculty of Veterinary), 6–29.

[B20] Clarke A. (1980). The biochemical composition of krill, *Euphausia superba* Dana, from South Georgia. J. Exp. Mar. Biol. Ecol. 43, 221–236. 10.1016/0022-0981(80)90049-0

[B21] Clemente J. C. Ursell L. K. Parfrey L. W. Knight R. (2012). The impact of the gut microbiota on human health: an integrative view. Cell 148, 1258–1270. 10.1016/j.cell.2012.01.035 22424233 PMC5050011

[B22] Collins M. D. Hoyles L. Foster G. Falsen E. Weiss N. (2002). *Arthrobacter nasiphocae* sp. nov., from the common seal (Phoca vitulina). Int. J. Syst. Evol. Microbiol 52, 569–571. 10.1099/00207713-52-2-569 11931170

[B23] Cryan J. F. Dinan T. G. (2012). Mind-altering microorganisms: the impact of the gut microbiota on brain and behaviour. Nat. Rev. Neurosci. 13, 701–712. 10.1038/nrn3346 22968153

[B24] David L. A. Maurice C. F. Carmody R. N. Gootenberg D. B. Button J. E. Wolfe B. E.. (2014). Diet rapidly and reproducibly alters the human gut microbiome. Nature 505, 559–563. 10.1038/nature12820 24336217 PMC3957428

[B25] Delsuc F. Metcalf J. L. Wegener Parfrey L. Song S. J. González A. Knight R. (2014). Convergence of gut microbiomes in myrmecophagous mammals. Mol. Ecol. 23, 1301–1317. 10.1111/mec.12501 24118574

[B26] Duffy L. C. Raiten D. J. Hubbard V. S. Starkereed P. (2015). Progress and challenges in developing metabolic footprints from diet in human gut microbial cometabolism. J. Nutr. 145:1123S−1130S. 10.3945/jn.114.194936 25833886 PMC4410496

[B27] Ezer A. Matalon E. Jindou S. Borovok I. Atamna N. Yu Z.. (2008). Cell surface enzyme attachment is mediated by family 37 carbohydrate-binding modules, unique to Ruminococcus albus. J. Bacteriol. 190, 8220–8222. 10.1128/JB.00609-08 18931104 PMC2593223

[B28] Francis O. E. Bendall M. Manimaran S. Hong C. Clement N. L. Castro-Nallar E.. (2013). Pathoscope: species identification and strain attribution with unassembled sequencing data. Genome Res. 23, 1721–1729. 10.1101/gr.150151.112 23843222 PMC3787268

[B29] Goodrich J. K. Waters J. L. Poole A. C. Sutter J. L. Koren O. Blekhman R.. (2014). Human genetics shape the gut microbiome. Cell 159, 789–799. 10.1016/j.cell.2014.09.053 25417156 PMC4255478

[B30] He X. Slupsky C. M. Dekker J. W. Haggarty N. W. Lönnerdal B. (2016). Integrated role of *Bifidobacterium animalis* subsp. lactis supplementation in gut microbiota, immunity, and metabolism of infant Rhesus Monkeys. mSystems 1:e00128-16. 10.1128/mSystems.00128-16 27921083 PMC5128019

[B31] Hou C. Zeng X. Yang F. Liu H. Qiao S. (2015). Study and use of the probiotic *Lactobacillus reuteri* in pigs: a review. J. Anim. Sci. Biotechnol. 6:14. 10.1186/s40104-015-0014-3 25954504 PMC4423586

[B32] Huson D. H. Auch A. F. Qi J. Schuster S. C. (2007). MEGAN analysis of metagenomic data. Genome Res. 17, 377–386. 10.1101/gr5969107 17255551 PMC1800929

[B33] Kim K. K. Lee K. C. Oh H. M. Kim M. J. Eom M. K. Lee J. S. (2008). *Arthrobacter defluvii* sp. nov., 4-chlorophenol-degrading bacteria isolated from sewage. Int. J. syst. Evol. Microbiol. 58, 1916–1921. 10.1099/ijs.0.65550-0 18676480

[B34] Langmead B. Trapnell C. Pop M. Salzberg S. L. (2009). Ultrafast and memory-efficient alignment of short DNA sequences to the human genome. Genome Biol. 10:R25. 10.1186/gb-2009-10-3-r25 19261174 PMC2690996

[B35] Leisner J. J. Larsen M. H. Ingmer H. Petersen B. O. Duus J. O. Palcic M. M. (2009). Cloning and comparison of phylogenetically related chitinases from *Listeria monocytogenes* EGD and Enterococcus faecalis V583. J. Appl. Microbiol. 107, 2080–2087. 10.1111/j.1365-2672.2009.04420.x 19583793

[B36] Li R. Yu C. Li Y. Lam T. W. Yiu S. M. Kristiansen K.. (2009). SOAP2: an improved ultrafast tool for short read alignment. Bioinformatics 25, 1966–1967. 10.1093/bioinformatics/btp336 19497933

[B37] Li W. Godzik A. (2006). Cd-hit: a fast program for clustering and comparing large sets of protein or nucleotide sequences. Bioinformatics 22:1658–1659. 10.1093/bioinformatics/btl158 16731699

[B38] Lorito M. Di P. A. Hayes C. K. Woo S. L. Harman G. E. (1993). Antifungal, synergistic interaction between chitinolytic enzymes from *Trichoderma harzianum* and *Enterobacter cloacae*. Phytopathology 83, 721–728. 10.1094/Phyto-83-721

[B39] Luo R. Liu B. Xie Y. Li Z. Huang W. Yuan J.. (2012). SOAPdenovo2: an empirically improved memory-efficient short-readde novoassembler. Gigascience 1:18. 10.1186/2047-217X-1-18 23587118 PMC3626529

[B40] Ma J. E. Li L. M. Jiang H. Y. Zhang X. J. Li J. Li G. Y.. (2017). Transcriptomic analysis identifies genes and pathways related to myrmecophagy in the Malayan pangolin (*Manis javanica*). PeerJ 5:e4140. 10.7717/peerj.4140 29302388 PMC5742527

[B41] Macdonald C. Barden S. Foley S. (2014). Isolation and characterization of chitin-degrading micro-organisms from the faeces of Goeldi's monkey, *Callimico goeldii*. J. Appl. Microbiol. 116, 52–59. 10.1111/jam.12338 24033399

[B42] Manfredo Vieira S. M. Hiltensperger M. Kumar V. Zegarra-Ruiz D. Dehner C. Khan N.. (2018). Translocation of a gut pathobiont drives autoimmunity in mice and humans. Science 359, 1156–1161. 10.1126/science.aar7201 29590047 PMC5959731

[B43] Martín R. Miquel S. Langella P. Bermúdez-Humarán L. G. (2014). The role of metagenomics in understanding the human microbiome in health and disease. Virulence 5, 413–423. 10.4161/viru.27864 24429972 PMC3979869

[B44] Miranda F. Veloso R. Superina M. Zara F. J. (2009). Food habits of wild silky anteaters (*Cyclopes didactylus*) of São Luis do Maranhão, Brazil. Edentata 8, 1–5. 10.1896/020.010.0109

[B45] Muegge B. D. Kuczynski J. Knights D. Clemente J. C. González A. Fontana L.. (2011). Diet drives convergence in gut microbiome functions across mammalian phylogeny and within humans. Science 332, 970–974. 10.1126/science.1198719 21596990 PMC3303602

[B46] Neufeld D. (2005). Chitinase from *Enterobacter* sp. NRG4: its purification, characterization and reaction pattern. Electro. J. Biotechnol. 8, 134–145. 10.2225/vol8-issue2-fulltext-6

[B47] Nisa C. Agungpriyono S. Kitamura N. Sasaki M. Yamada J. Sigit K. (2010). Morphological features of the stomach of Malayan pangolin, *Manis javanica*. Anat. Histol. Embryol. 39, 432–439. 10.1111/j.1439-0264.2010.01015.x 20645954

[B48] Parks D. H. Tyson G. W. Hugenholtz P. Beiko R. G. (2014). STAMP: statistical analysis of taxonomic and functional profiles. Bioinformatics 30, 3123–3124. 10.1093/bioinformatics/btu494 25061070 PMC4609014

[B49] Pindi P. K. Manorama R. Begum Z. Shivaji S. (2010). Arthrobacter antarcticus sp. nov., isolated from an Antarctic marine sediment. Int. J. Syst. Evol. Microbiol. 60, 2263–2266. 10.1099/ijs.0.012989-0 19783612

[B50] Prem Anand A. A. Sripathi K. (2004). Digestion of cellulose and xylan by symbiotic bacteria in the intestine of the Indian flying fox (*Pteropus giganteus*). Comp. Biochem. Physiol. Part A 139, 65–69. 10.1016/j.cbpb.2004.07.006 15471682

[B51] Ramakrishna B. S. (2013). Role of the gut microbiota in human nutrition and metabolism. J. Gastroenterol. Hepatol. 28, 9–17. 10.1111/jgh.12294 24251697

[B52] Redford K. H. Dorea J. G. (1984). The nutritional value of invertebrates with emphasis on ants and termites as food for mammals. Proc. Zool. Soc. Lond. U.S.A. 203, 385–395. 10.1111/j.1469-7998.1984.tb02339.x

[B53] Ren Z. Jiang J. Lu H. Chen X. He Y. Zhang H.. (2014). Intestinal microbial variation may predict early acute rejection after liver transplantation in rats. Transplantation 98, 844–852. 10.1097/TP.0000000000000334 25321166 PMC4206351

[B54] Sanders J. G. Beichman A. C. Roman J. Scott J. J. Emerson D. McCarthy J. J.. (2015). Baleen whales host a unique gut microbiome with similarities to both carnivores and herbivores. Nat. Commun. 6:8285. 10.1038/ncomms9285 26393325 PMC4595633

[B55] Schumann P. Weiss N. Stackebrandt E. (2001). Reclassification of *Cellulomonas cellulans* (Stackebrandt and Keddie 1986) as *Cellulosimicrobium cellulans* gen. nov., comb. nov. Int. J. Syst. Evol. Microbiol. 51, 1007–1010. 10.1099/00207713-51-3-1007 11411667

[B56] Sebaihia M. Peck M. W. Minton N. P. Thomson N. R. Holden M. T. Mitchell W. J.. (2007). Genome sequence of a proteolytic (Group I) *Clostridium botulinum* strain Hall A and comparative analysis of the clostridial genomes. Genome Res. 17, 1082–1092. 10.1101/gr.6282807 17519437 PMC1899119

[B57] Taylor W. A. Lindsey P. A. Skinner J. D. (2002). The feeding ecology of the aardvark *Orycteropus afer*. J. Arid Environ. 50, 135–152. 10.1006/jare.2001.0854

[B58] Thaiss C. A. Zmora N. Levy M. Elinav E. (2016). The microbiome and innate immunity. Nature 535, 65–74. 10.1038/nature18847 27383981

[B59] Vaaje-Kolstad G. Bøhle L. A. Gåseidnes S. Dalhus B. Bjørås M. Mathiesen G.. (2012). Characterization of the chitinolytic machinery of *Enterococcus faecalis* V583 and high-resolution structure of its oxidative CBM33 enzyme. J. Mol. Biol. 416, 239–254. 10.1016/j.jmb.2011.12.033 22210154

[B60] Vaajekolstad G. Bunaes A. C. Mathiesen G. Eijsink V. G. (2009). The chitinolytic system of *Lactococcus lactis* ssp. lactis comprises a nonprocessive chitinase and a chitin-binding protein that promotes the degradation of alpha- and beta-chitin. FEBS J. 276, 2402–2415. 10.1111/j.1742-4658.2009.06972.x 19348025

[B61] Wadström T. (1971). Chitinase activity and substrate specificity of endo- -N-acetyl-glucosaminidase of *Staphylococcus aureus*, strain M18. Acta Chem. Scand. 25, 1807–1812. 10.3891/acta.chem.scand.25-1807 5099106

[B62] Wang F. Gai Y. Chen M. Xiao X. (2009). Arthrobacter psychrochitiniphilus sp. nov., a psychrotrophic bacterium isolated from Antarctica. Int. J. Syst. Evol. Microbiol. 59, 2759–2762. 10.1099/ijs.0.008912-0 19625417

[B63] Wicker L. Thai N. V. Phuong T. Q. (2008). A Long Way from Home: The Health Status of Asian Pangolins Confiscated from the Illegal Wildlife Trade in Vietnam. Paper presented at: The Workshop on Trade and Conservation of Pangolins Native to South and Southeast Asia.

[B64] Xiao L. Estelle J. Kiilerich P. Ramayo-Caldas Y. Xia Z. Feng Q. (2016). A reference gene catalogue of the pig gut microbiome. Nat. Microbiol. 1:16161 10.1038/nmicrobiol.2016.161 27643971

[B65] Xu Q. Morrison M. Nelson K. E. Bayer E. A. Atamna N. Lamed R. (2004). A novel family of carbohydrate-binding modules identified with Ruminococcus albus proteins. FEBS Lett. 566, 11–16. 10.1016/j.febslet.2004.04.005 15147860

[B66] You M. Yue Z. He W. Yang X. Yang G. Xie M.. (2013). A heterozygous moth genome provides insights into herbivory and detoxification. Nat. Genet. 45, 220–225. 10.1038/ng.2524 23313953

[B67] Zhang D. C. Schumann P. Liu H. C. Xin Y. H. Zhou Y. G. Schinner F.. (2010). *Arthrobacter alpinus* sp. nov., a psychrophilic bacterium isolated from alpine soil. Int. J. Syst. Evol. Microbiol. 60, 2149–2153. 10.1099/ijs.0.017178-0 19880631

[B68] Zhu W. Lomsadze A. Borodovsky M. (2010). Ab initio gene identification in metagenomic sequences. Nucleic Acids Res. 38:e132. 10.1093/nar/gkq275 20403810 PMC2896542

