# Supplementary material for: The Fecal Metagenomics of Malayan Pangolins Identifies an Extensive Adaptation to Myrmecophagy
Source: Front Microbiol. 2018 Nov 23;9:2793. doi: 10.3389/fmicb.2018.02793 (PMC6265309; doi:10.3389/fmicb.2018.02793)

**Figure S1 Composition of raw reads for each sample.** Reads containing ambiguous base (N bases), adapter sequences, and low-quality bases were removed from the raw data.


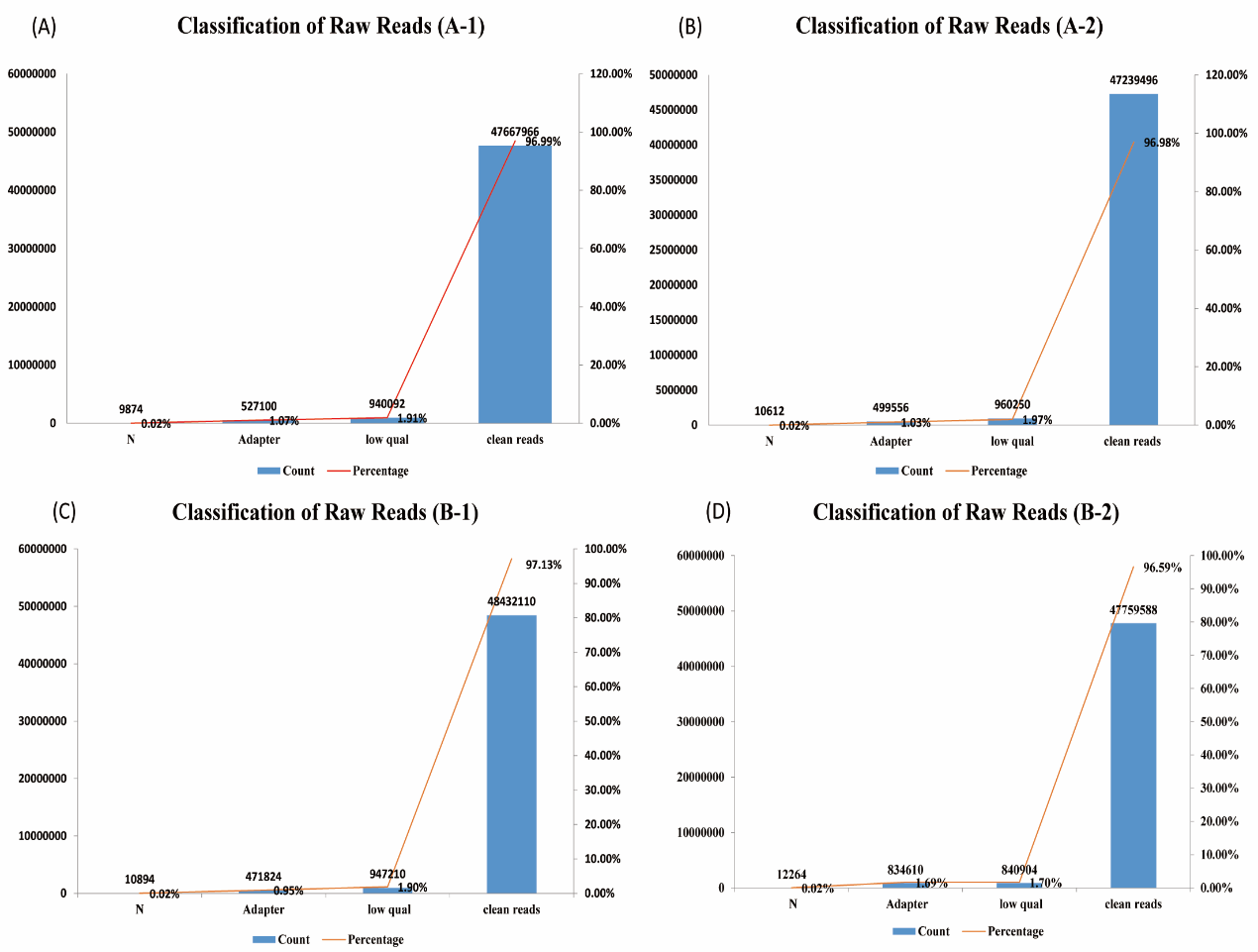


**Figure S2 Length distribution of assembled contigs.** The X-axis indicates the length intervals, and the Y-axis represents the number of contigs.


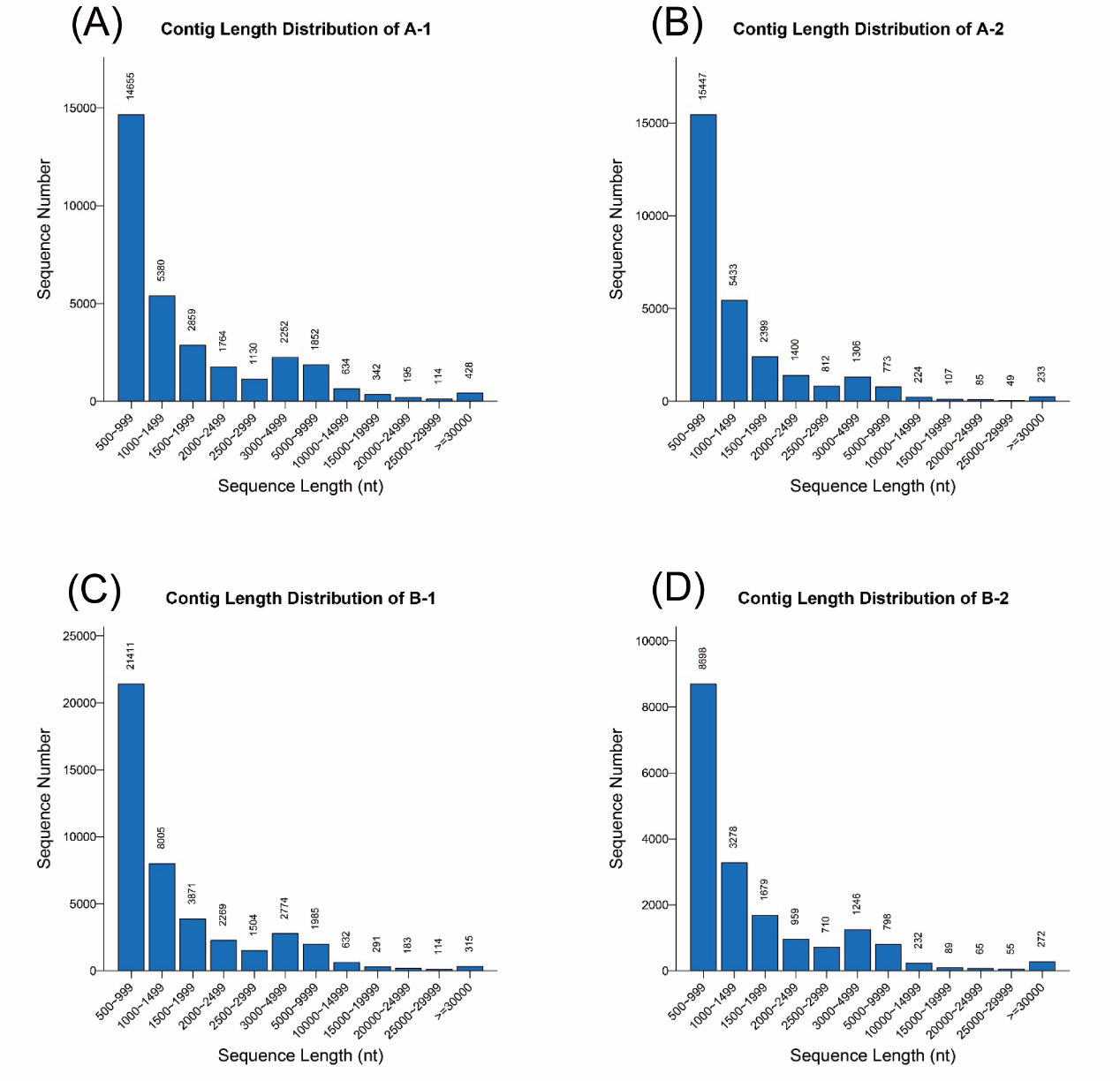


**Figure S3 Summary of Kyoto Encyclopedia of Genes and Genomes (KEGG) annotations.** The X-axis represents the number of genes annotated in each pathway, and the Y-axis lists annotated pathways in the particular subclass.


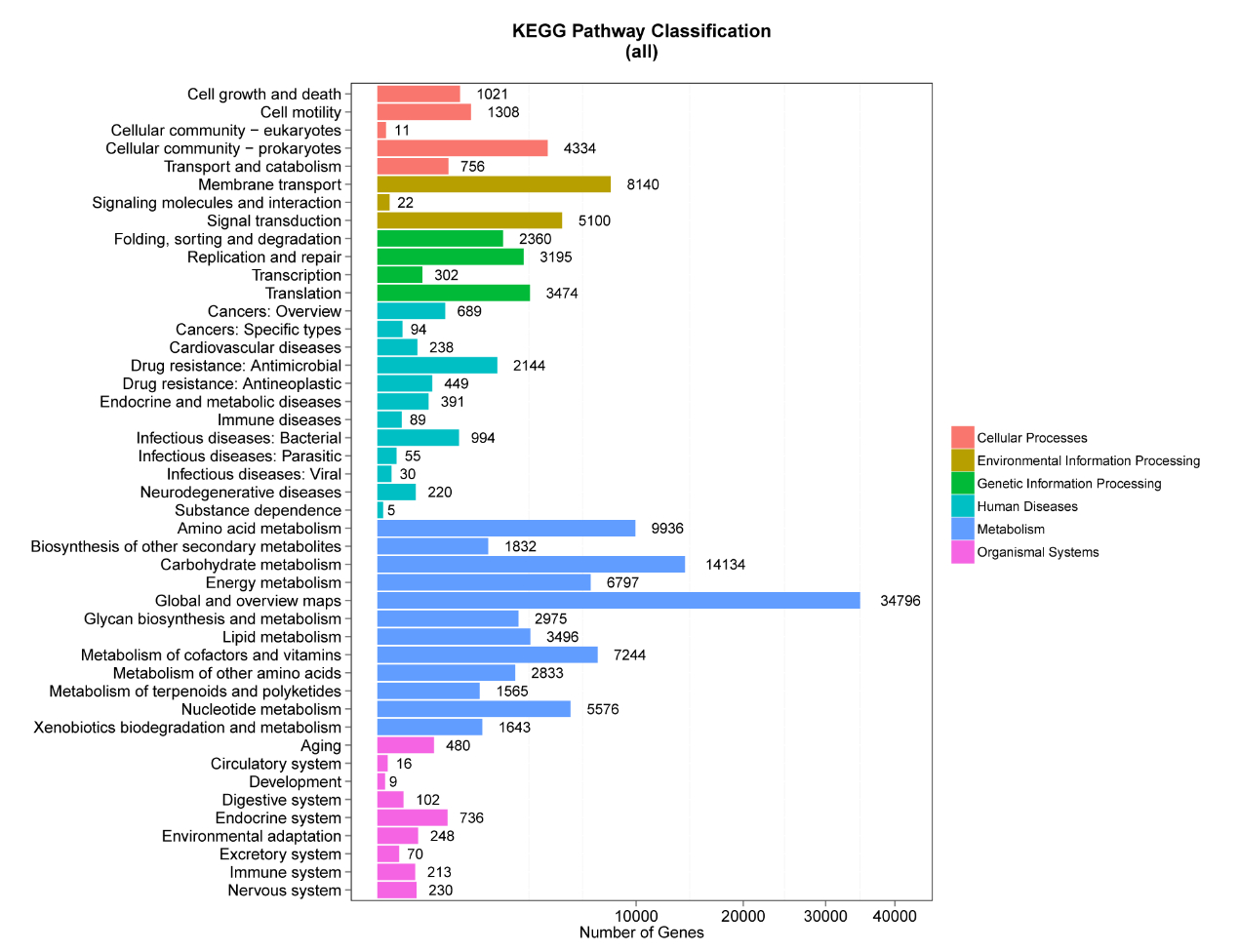


**Figure S4 Amino sugar and nucleotide sugar metabolism (KEGG map 00520).**


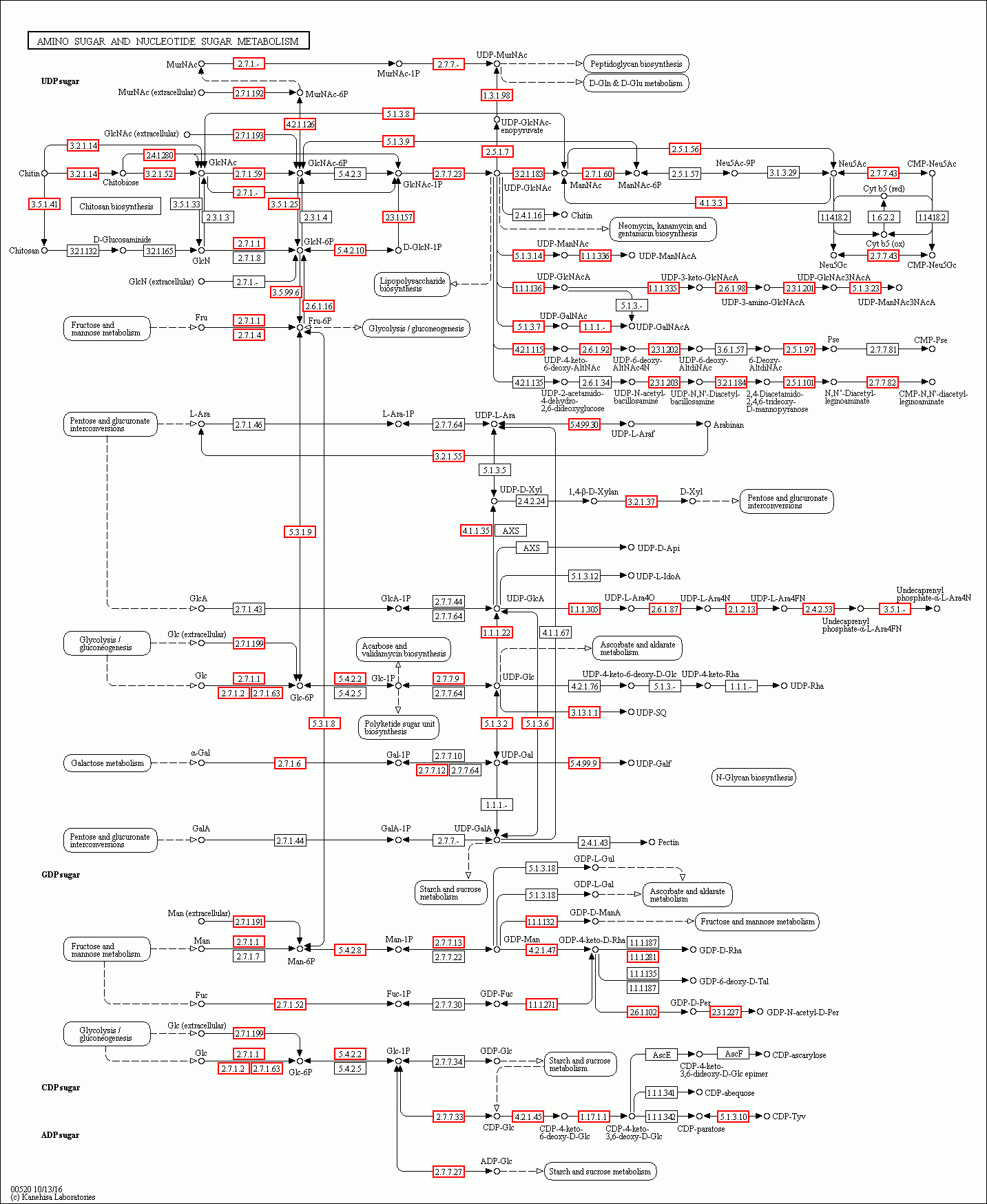

Supplement: Supplementary file 1 [file Data_Sheet_1.zip › 410136-supplementary materials/Figure S .docx]
