# Supplementary material for: The Fecal Metagenomics of Malayan Pangolins Identifies an Extensive Adaptation to Myrmecophagy
Source: Front Microbiol. 2018 Nov 23;9:2793. doi: 10.3389/fmicb.2018.02793 (PMC6265309; doi:10.3389/fmicb.2018.02793)
Supplement: Supplementary file 1 [file Data_Sheet_1.zip › 410136-supplementary materials/S Files/File S4.docx]

>[denovogenes]_68467

CCAGACAAAGGGTTTAAGCTTACTACAGAGGTTTTGCGCCGTAAGCCGGATTCAGCGTTAGCTTGGTATGAAGACTTTAGAGATCCCGTCATCCTGCCAGAAAGCTACTGGCAGACGTTATCGGGGAGTTGGAGTGTATGGCAAAATCCTGAAGACAATGGCAATCGTCCGTATTCTCAGCTTGAAGGAAGTGGTCAGCTTGCTTGGAAATACGATAGCTTTCGTGATGTGCATATCCGTGCTAGAATTGCCTTTCCTATGAATGGTAGCGGAAGAGCAGGGATATTTTGTGGGAATGTGTTTTGCTGTATCAACATTGACACCCAACAAGTGGAGCTATACCAAGGCTCTACATTACTTGGTAGTTATCCGGCAAGTTACTATAAGACTTCAGATGCTGACATTCGCACCAATCCGAATATGTATCTCATTGAAATGAGGAAACGTGGAAATAAGGTGCGTATCTACTCTGGGAATTCCAACACACTTCGTTTTACTGCAAACATCTCAAGTACGGGTGGCTATTGTGGTATTCAGTCCGATGGGCAAATTAAATGTGAACTTCTCCGCTTAGGCGATGCATGGACTTATGAACCTTACGAAGCCTTTGATGTCACCATGCCAGACGGATCACTTATGCAGTATGGCAGAATTGCTAGGAATGGTGTAACTTGGGATAGTAAGTTTCATGTATTTACTCTTACAGCTGATGTAGAGGAAGCCTCCACCAGAAGTGAGGATATTTCCATGGATTATGACTTTTATCATAGTAATCTCTTGCAGATACCATGCAATGCAGATTATACAGCGAAGGTTATACCAAAGGACATAAACGTCTGGATATCTCGGCTGTTTCTCGGTGATGCAGATGGCTTTTCAATTTTGTACTATCAAGATGTGGATTCGCTGGTCTACTGGTCAAACGAAGCGGCTTATCGTTGGGACCTACGGGGTATTGCTATCTGGTCATTGGGACAGGAAGATATGCGTCTTTGGGAAGCTTTGCCAAAACAAATATAG

[denovogenes]_151116

CCGGGAGATACCGTATCTTCGATCTCCCGTCAAAGTGGGGTTCCTGCCTGGAAGATTATTTATGATAATCAGCTGGGAGAGGAGGGAAAACTGACAGTGGGTCAGGCGCTGCTCTTGTTAAAACCACAGGAGAATGCGGAATTCCGGGAGGATCTGTATGTGACAGGGTATGCATATTCTTTTATTGAACCCTATGTGCTGGAAATGGCATTTCCGGCACTCAATGAACTGCTTGTGTTTTCTTATGGCTTTACGTTTGAAGGAGAGTTGGTTCCGCCCATACAGGATGAAACATGGATGATACAGCTGGCCTGGGAGAATGGGATAGAACCGATGCTGGTTCTGACCCCTTTTACACAGGGGGTCTTTAACAATCAGCTGATTCAGACTCTGGTGGAGGAAGAATCCGTACGTGAAAATGTGATTACCAATCTGCTGAAGGTGGTGGAAGAAAAAGGATATGTGGGTGTGGATGTAGATTTTGAATATGTCCGTGCGCAGAACAGGGAAGGATATGCCGGGTTTGTCGGAGAACTGCGGGCTGCCATGAACAAAAAGGGATATCGCGTGTCGGTGGCTCTGGCG

>[denovogenes]_74830

GTCAGCGAACAAAAAAGTGAAGAGTATGTCATCTGGCAGACAGTCGATAATAAGGCATATGTTGCTTTGGACTTTGTGAAAAAGTACACGAATATGGAATGCAAAGAACATCAAGATCCGAATCGTGTGATGATCGTGAATGAGTTCGGAAAGACGACTGTGGCAGAGATGAAGCGGGATACACAAGTGCGGTTTCAGGGTGGAGTAAAGAGTATAATCCTGACAGAGGTAAAAAAATCAGAAAAAGTGACTGTGATCGAGGATGAGGATGGCTGGAAGAAAGTTCGCACCAGCGATGGATTTATCGGATATGTACAGACGAATTCGCTGAAACATATCAAAGAGGAGACGATTTCCAGCAGCTTTGAAGAACCACAGTACACAGGTATATCGAAAGATTACAAGATTAATATGGCCTGGCACAATGTAGAAAATACTACTGCCAACGGGTATATTCAGGATATGCTTGCATCCACGAAAGGTCTGACGACGATCGCACCGACCTGGTTTCATATTGCAGATACGCAGGGGAATCTGAATTCAATCGCGGACGCAGATTATGTGAATTATGCGCATCAGTCCAATTTGGAAGTGTGGGCTGTTCTCAGGGATTTTCATGGGGGAATCAATTCGGCGGATGAGACTTATGAGGTGTTAAGTCATACTTCCAGGAGAACGAACCTGATCGATCAGGTTATAGCAGCAGCTCTGCAGGCTGGGATTGATGGAATCAATCTGGATTTTGAACTGATTTCTGCAGAGTGCGGAGAAGATTATGTACAGTTTGTGAGAGAACTTTCCATCAAGTGCCATCAGAATGGACTGGCATTTTCCGTAGACAATTATGTTCCGATGCCTTACAACACATTTTATGATCTGGAAGAACAGTCAGTATTTGCTGATTATGTAGTGATCATGGGGTATGATGAGCATGTGGAGGGTTCTTATGAAGCAGGTTCTGTGGCGTCTTACGGA

>[denovogenes]_42081

ATGCCGATACATGTTGTAAAAAGTGGAGAGACAATCTATTCGATCGCTCAGCTATATGATGTCTCAGCTGATCGGATTGTGTATGACAATGAGCTGGCAGCGCAGCAAAATCTTGTTCCAGGGCAAGCTCTTTTGATTCTGATGCCGAGTCGGGTTCATATTGTGAGAGAAGGACAGACGGTAGAGCAGATCGCGGAAGAATATAGTATCACGGTCAAAAGTCTATATCAAAACAATCCGTTTCTGTTGAATCAGACCTATTTGTTAGAGGGACAGAGTTTAGTCATTTCTTATGAGGGAGAACCGCTTATGCAAGGTAGGATCAGCGGGTATGCATATCCGTTTATCGAGCCGTATATTTTGCGTGAGGTACTTCTTTATATAGATGAAATACTTATTTTTTCTTATGGATTTACGTCAGAAGGAGAATTGATTCCACCTCAGATTGACGAGACGTGGGTGATTCAGGAGGCTTGGAATCAGCAGGTGGAACCGATTCTGGTGCTGACGCCGTTTGCGGAGACGGGAACCTTTAACAGTGGGCTGATTCAAATATTATCGGAAAATGAGACGGTTCAGGACAATTTGATTGAAAATCTTTTGGAGACAGTACGAGAAAAGGGGTATGTAGGTGTAGATGTGGACTTTGAATATATTCGGCCGGAAGATCGGGTGGGGTATGCAGATTTTGTGAATCGTTTGCGGGAGACGATGAATGAAAATGGATATCGGGTGTCGGTGGCGCTTGCTCCGAAGACCTCTTCTTATCAAAAAGGGTTGTTGTATGAGGCAATGGATTATCATCTGTTGGGGCAAAGTGCAAATACGGTGTTTCTGATGACGTATGAATGGGGGTATACATACGGACCACCGCTTCCGGTAGCGCCGCTGCCAAATGTCCGACAGGTGTTGGAGTATGCCCTCACGGAAATTCCGAAAGAAAAAATTGTGCTTGGGATTCCTAATTACGGGTACAGTTGGCCGCTCCCGTATGAGCGAGGTGTGACAAAAGCAAGATTGATTGGAAATGTTGAGGCGAATGTCATTGCGGCAGAGCGAGGTGTGGAGATTCAGTACGATGAGCGTTATCAAAGTCCGTTTTTCTATTATGAAATTGGTGGCAGGAGATATGAGGTCTGGTTCGAGGATGTGAGAAGTATTTATGCAAAATTGCAGCTGGCTGCAGAAAAGGATATCCGTGGCGTCGGGTATTGGAACTTGATGCGTCCGTTTCGGGCAAACTGGCTGCTGGTGAATCAGATGCTTCATTGA

>[denovogenes]_61326

ATGGCAAGTTTCATGTTTTCATGTACTGACATTGAAACTATCGACTTGGAAAAAGAAGCCGTGAAAGATCTGTATGAGAATCGTGATAAAGATAAATGGGCTGAGGAAGATGCCCAGAAACAACAGAATTATGAGGACTCTGTACGCATAGCCGAGGAGAACAAACGCCTCTATGAACTCTACCTTGCAGATTTAAGGGAGTACAAAGAAACCAAGCATCCGGTGATGTTCGGCTGGTTCAACGCATGGAGTGCTGAAACTCCGGGCGAATACTCCAATCTGACGTTGATTCCCGACAGTATGGATATCGTTTCTATTTGGGGAAATTGTTTCAACATTAATGAAAAACGTCTGAAACAGATGAGAGAGGTGCAGAGTAAAGGTACCAAGGTCATTGTGGGTTGGATCGTAGAAAACGTGGGTAACGGACTTAGTAATATTCCTGAAGGCGGATGGTCTGATGACCCGACAACAGGTATCAAGCAATACGCACAAGCCATTTTGGACTCCATCGCCAAGTATGGATACGACGGCTTCGATATTGACTATGAACCTTCCTATGCCTCTCCTTTCAAACCAGGCAATCACTGTGGCGACTGGACAAACGACTGGACAGACTACAGGCCTATCATCTCTTGCAGCTCGTATGACAATAAAGAATATGAAAATCTTTTCTTTCAAACATTACGCAATGGTTTGGACAAATTAGAAGCCAAAGACGGCAAGAAGAAGATTCTGAACATCAATGGCTCAATCCACTATCTATCCCCGGAAATGGCACCTCTCTTCTCCTACTTTGTGGCACAATCCTATAATGGTAGTTATAGTGGATGGACATCCAGAATAACCAACCGTTTGGGGAATGATGTGAAAGACCAGATTATCTATACAGAGACATTCGAAAGCGGACAAGCCAACCGGATAAGTTTTGAAAATTATGCAAACTTTGTTGTTAACAACCTCAACCGGGAAGCTGGTGGTATCGGTGCTTATCACATTAATGCAGATTCATTTGACAAAAACGAATATCGTTATGTACGAAATGCAATTTCCATCATGAATCCTCCTATCAAGTAA

>[denovogenes]_79414

ATGAAAACAAAAATAACAGGTTTACTGACTTTCCTTATCGGAACATTCTTTTTCTATAGTTGTGATACCGATGTGGAAGCGTTAGAAATTCAGAAGCTGAAAACATATGACGAGCAGTATTTTGAGAATCTGCGTGCTTTCAAGAAAAGCGATCATGAGATAAGTTATGCTTATTACGAAGCGTGGTCACCGATTGAAGGTGTGACAGGCTATAAAGATCCCGCGTCGTGGGGTGAACGTATGGTAGGACTACCTGACAGTATAGATATTGTAAATCTTTGGATGGGAATACCGACAGCAGAGACACATCCGATAGCATATGCGGATATGCAATACTGCCAGCGGAAATTGGGTACACGTTTTGTCATGCATGCCGATGCTTCTCATTACAGACACCAGTTTACCGTTGATGGGGTTGATTATGATCTTAGCCAGAATAAGGACGATGAAGCGATGGCTGCATATGCAAAATGGATTGTGAATCAAGTGTTGGAACCGGGACTTGATGGAGTAGACGTTGACTGGGAAGGTTGGAGTGGTTCCGATTTGGTCAGATTGATAAAGGAACTTGGCAAGTATTTTGGTCCTGAAGGCGAACAGCCGGATAAGTTGCTTATCGTTGACTACTTTAGTGGAACACCACCGACAGACATCATACCTTATTGTGATTATATCGTTCAACAGGCTTATAGTGATCAAGTGGGGTTCTTGACTCAACCCAGTAACTTTCCACCCGAGAAGATGATTTACTGCGAATCGTTCGGAGTATTCTATGCCGATGGGGGACAACTTATGAACTATGCACGTTGGGAACCGTCGAAAGGCCGTAAAGGCGGTTGTGGAGTATTCTACTTAGGAAGAAATTATTATTCTTCATCGGGTATACCTTATAATGAATTCCGGGAGGCTATTCAGATAATGAATCCTGCTGTAAATGAGTAA

>[denovogenes]_17967

ATGAAAAAGCTGAAAATGATGATGGCGGCTGCATTGGCTGTATTGGCTTCATGCGGCGGCACCAAGTCGGGGGAAAGCGTGCAGGATCCCCTGGATTCAAAAGTGATTGTCGCTTATGTCACTTCCTGGAGTGATGTATTGCCGGATCCCCGGTACATGACGCATATCAATTATGCCTTCGGTCATGTTAACGAAAGTTTCAACGGTGTCGGAATTGATAATGAAGAGCGTTTGAAACAGATTGTAGATTTGAAGAAACAGAAACCGGAGTTGAAAGTATTGCTTTCTATCGGCGGTTGGGGCAGCGGGCGTTTCAGTGAAATGGCTGCCAGTGATGAGTACCGTATGGCATTTGCCAAGGACTGCGACCGTGTTGTGAAAGAATATAGTTTGGATGGTATCGACATTGATTGGGAATATCCTACCAGTTCCATGGCGAATATTTCTTCTTCGGCAGATGATACGAAGAACTTTACACTGCTGATGCGTGATATCAGAACGGCCATCGGTGACAAGAAGGAACTGACGCTGGCAACGGTTGCGTCTGCCAAGTATATAGATTTCAAGGCTATTCTTCCTTTTGTGGATTTTGTGAATATCATGGCCTATGATATGGCGTCTGCACCCAAGCATCATTCGGCGCTGTATCCTTCGGAACATAGTGGTGACATCACTTCGGATGCAGCCGTTGCTGCACATCTGAAAGCCGGCGTGCCTCCTTCCAAGTTAGTGATGGGTATGCCTTTTTATGGCCGTGGAGGCAATGGTTATCCCGATTTCCAGGATTTCAATAAGGTAGGAAACACGAATAAGGATTTTACGGAAAAATGGGACAGCGTAGCGCAGGTTCCTTATCTGGTCAACGGGGATGATACATTGGTATTCGGTTTTGAGAATCCCCGCTCCCTGGCCATCAAATGCCAGTATATTCTGGACAAGGATTTGCTGGGCGGCATGTATTGGGATTATAGCGGTGATAACGAGCAAGGTGACTTGCGGCGTACGGTAGCCGAGAATCTGTTGGGCAAGAAGCATAAGACCAAGGTGCTGGTGTTGACGGAGAGGGGCGGCCAACATGGAGGATTTACCGATGCCGGTATGCGATGGTTGGCGGACGAAAGTGCACGACAGAATTTCAGCATAACAGAAATCAATAATGCGGCTCCTGTCACAGAGGCCTACCTGTCACAATTCAGTTTGGTTATCCAATTGGATTTCCCTCCTTATACCTGGCCTGAGGAAGCGGAGAAAGCTTTTATCAATTATATAGAACAAGGTAAAGGCGGCTGGATCGGTTTCCATCATGCTACTTTATTGGGTGAGTTTGACGGTTATCCCATGTGGCAGTGGTTTTCGGACTTTATGGGCGGAGTACGTTTCAAGAGCTATATCGCTCCTCTTGCCGATGGCACGTTGGTGGTAGAAGACCGGGAACACCCGGTCATGAAAGGGGTGCCTGCCTCTTTCGTGGTTCCGGATGATGAATGGTACACCTATGACAAAAGTCCCCGTCCGAATGTGCATGTACTTGCCAATGTGGATGAATCAAGCTATACACCTGCTTCGGATATCAAGATGGGCGATCATCCGGTGGTCTGGGTGAATGAAAAGGTAAAAGCCCGCAATGTTTATTTCCAGATTGGGCATAGCGGCAAACTGTACGAAACGGAAGGATTCACTACTATGTTCAGAAATGCCATAAGTTGGACATTGAACCGATAA

>[denovogenes]_61119

ATGTTTAAGCTAAAAAGAGAAAAGAAAATGAAAAAGAGATATTTAAATATAGTGGTTTTTGCGTTGTTCGTCAGTATGCTTCTGGGTTGCAGTGACTGGACCAAACCCGAAGCAGAGGATTTTTTTGAAATGCCGGGAAATGACTACTACGAGAATCTGAGAGCTTATAAACGTTCGGAGCATTCGGTTGCGTTTGGCTGGTTTGGAGGCTGGACAGGTGTGGGAGCTTCGATGGTGGGGAGTCTGATGGGACTTCCCGATAGTGTGGACTTCGTTTCCATCTGGGGAAACTGGAAGAATCTGGATGAAGCCCGGATGCTGGATAAGAAAAAAGTGAAAGAACTGAAAGGTACGCGTGCATTGATGTGCTTTATCGTAGCTAATGTAGGCGATCAGTTGACCCCCGAAGAGTATAAAGAGAATTATAAAGAATATTGGGGGTGGAAAGATGGCGATCAGGAAGCTATCGACGGCGCGATCAGAAAATATGCAAATGCCATTTGTGACTCCATCGATAAGTATGGATATGATGGTTTCGATATCGATTATGAACCTAATTATGGGTCTCCCGGAAATCTGGCCAGCTATCCCGAAAATATGCTCACTTTCGTAAAGGCGTTAGGAGAGAGAATAGGACCCAAATCGGGCACGGGGCGACTGCTTGTGATCGATGGTGAGCCACAAAGCATTCATCCGGAAACCGGACCTTATTTTGATTACTTCATCGTGCAGGCATACTCCAACCTTGCGGGTAACTCCGATACTAATCTTGACAGGCGGCTGGCCGGTACAATTGCCAATTTTAAAGAGGTACTCACTCCCGAAAAAGTGGCTAATATGTACATCGTCACAGAAAATTTCGAATCCTATGCTCCGACAGGAGGAGGCGATTATGTAGATCGTTACGGAAATAAAATGCGGGCACTTGCCGGAATGGCACGCTGGACTCCGGTCATTGATGGTAAACAAGTTCGAAAGGGCGGAGTAGGGACGTATCATATGGAATATGATTATCCGGGCGACGTTGAATACAAATACTTGCGTGAGGCAATCAGAATTATGAATCCCGCTGTAAAATGA

>[denovogenes]_64063

ATGAAAAAGAGATATTTAAATATAGTAGTTTTTGCATTGTTCGTCAGCATGCTTTGGGGTTGCAGTGACTGGACCAAACCTGAAGCGGAAGACTTTTTTGAAATGCCGGGAAATGATTATTACGAGAATCTGAGAGCATATAAACGTTCGGAGCATTCGGTTGCATTCGGTTGGTTTGGAGGCTGGACGGGTGTCGGGGCTTCGATGGTGGGCAGTCTGATGGGGCTTCCCGATAGTGTGGACTTCGTTTCGATTTGGGGTAACTGGAAAAATCTTGATGAGGCCCGGATGTTGGACAAAAAGAAGGTGAAAGAACAGAAGGGTACACGCGCCTTAATGTGTTTTATCGTGGCTAATGTAGGCGATCAGTTGACTCCGGAAGAGCATAAAGAGAATTATAAGGAGTACTGGGGCTGGAAGGATGGTGATCAGGAAGCTATCGACGGTGCCATCAAAAAATATGCGAATGCTATTTGTGACTCCATTGATAAGTATGGATACGATGGTTTCGACATCGATTATGAACCTAATTATGGTTCTCCCGGAAACTTGGCAAGTTATCCCGAGAATATGCTCACTTTCGTGAAAGCACTGGGAGAAAGAATCGGACCTAAATCGGGTACGGGACGGCTACTTGTGATTGATGGCGAGCCTCAAAGTATTCATCCGGAAACCGGGCCTTACTTTGACTATTTCATTGTGCAGGCATACTCTAACCTTGCGGGTAATTCCGACGCTAATCTTGACAGACGTCTGGCTGGTACGATTGCCAACTTTAAAGGAATACTTCCTCCGGAGAAAGTGGCTAATATGTACATCGTTACGGAGAATTTCGAATCTTATGCTCCGACAGGAGGAGGTGATTATGTAGATCGTTACGGAAACAAAATGAGAGCACTTGCCGGCATGGCACGCTGGACTCCGACTATTGACGGAAAACAGGTACGGAAGGGTGGAGTAGGCACCTATCACATGGAATATGACTATCCGGGCGACATCGAATATAAGTACCTGCGTGAAGCAATCAGAATAATGAATCCTGCTGTAAAATGA

>[denovogenes]_136569

ATGAACGAGATTTTAAAACAGATTTTCCCAAATAGCACGGAGGAAAACCGGGCTAAATTTGCACAACCATTGCAATTGGCAATGATGCGGTACAATATTAACAACCCAAAACGAATACGGGCGTTTTTGGCGCAAATAGGACACGAAAGCGGGCAATTATCCGCCGTCGTGGAAAACCTTAATTACAGCGCAAAAGGATTGCGGGCAACGTTTGGGAAATATTTTAAAACGGATGCCGAGGCGGAGAAATACGCACGTAAACCGGAGGAAATCGCAAATATAGTGTACGCAAACCGATTGGGGAACGGCGACACGAAAAGCGGCGACGGTTGGAAATATCGGGGGCGGGGCTTAATTCAGATTACCGGAAAAGACAATTACACGGAGGCAAGCAATAAAATGTACGCATTGCCGTTGGGCGTTGACTTTGTGGACGAACCCGAATTGTTGGCAACCCCGGAATACGCCGCACAATCGGCGGCGTGGTGGTGGGACAATGCCGGGTTAAACTCTATTGCCGACCAATTAGGCGGGGCGGACGATACCGAGGTTTTCAAACGCATAACCAAACGAGTAAACGGGGGTTATGGCGGATTGGACGACCGATTGGCGATTTATAACCGTGCAAAATCTGTTATAGTATGA

>[denovogenes]_55755

ATGAAAAAACTGATTTTTATTTTTTTTACATACATGTGTTTGGCTACTTCATTTTTTACCTCATGTTCTGATTGGACGGATGCTGAGCGTGAAATGTTTCCTGAACAAGAAGAAATTAACCGCATAATTCCTTTTTTGGAAGCTCAATCGGAAGATGATCTGATGCCTTCCGAACGTGCTTATTACCGCAAATTACGGGAAGAATATCGGCCGTCCCCTCATGTAAAAGGATTTGGCTGGTTTGGTAACTGGACCGGTAAGGGTACTAATGAGCAGAATTATTTGAAATGCCTGCCCGATAGTGTCGATTTTGTGTCTTTGTGGGGAGCACGCGGCAACTTTTCTTCTGAGCAAAAAGCCGATTTGAGGTTTTTCCAAGAAGTAAAAGGGGGGAAGGCCCTGATGTGCTGGATTGTTCAGGATATAGGTTCAGCCATAACTCCTGAAGGAAGGGATCCAAATCAATATTGGGTACAGGAGTTAGGGCAGGGTGACCCTTTGAAAGGGGCCGAAGCTTATGCTAATGCCATTGCTGATACTATCATTAAGTATAATTTGGATGGTTTTGATATTGATTACGAACCGAATTACGGACATAGCGGTACCTTGGCAAATAGAAGCGAAATTAATGAAAAATCGGGTAACGTGAATATGTATCGTTTCATTAAGACACTTTATGATCGCTTCCAAAACCATGAGCGAGAGACTGGAAAAGCCATGATGCTGGTTATGGATGGTGAACCTTATTTGTGTAGTACAGAAACCTCCAAAATGATTGACCACTATATTTATCAGGCGTATTGGGAGAGCTCAACGCGCGCAGTTATTTCTAAAATCAACAGAACCAATCTCACTGATTGGGAACGTAAAACGATTATTACGGTTGAATTTGAGCAAGGCTGGCGTACAGGGGGGATTACGGGTTACTTTAGCGCGGCCCGCCCAGAGTTGAATAGTGAGAAGGGACGTCAGATTTTAGACTATGCTACCTTGGATCTGCCTAGCGGCTTGCGTATCGGTGGCATTGGCACCTACCACATGGAGTATGATTATAAAAATGATCCTCCCTATAAATGGTTGCGTAGAGCGTTGTATTATGGCAATCAGAAATATCCCGGAAAATTTCAATAA

>[denovogenes]_66063

ATGAAAATATTCAGGCTGTTATCTCTGCTGCTGGTTGTATTATTGGCTTCTTGTCTGAATAAGAAGAAGTCGGCAGATACCGGTATGCCGGGAAATGAATCTCGGTATGTGGTGTTGGCTTATGTTACTTCATGGGGAATAAGTACTCCCGAAACATCGTGCCTGACTCATATCAATTATGCTTTTGGGCATGTGGCGGATGATAACAAGGGAATACGGATAGATAACGAGCCTCGCCTGCAGATGATAGTGGGTTTGAAAAAGGAAAAACCTTCTTTAAAGGTCTTATTGTCTGTGGGAGGCTGGGGTAGTAGCCGTTTCAGTGAAATGGCGGCCAATGACAGTACTCGTACTGCTTTTGCTGCCGATTGCAGAAGGGTGATTGATAAATTTGGATTGGATGGTATTGATATTGACTGGGAGTATCCGGGTAGTGGAACGGCCGGCATATCTTTTTCTTCTGCCGATAAGGAGAATTTTACTCTTTTGATGCGTGATGTCCGGCAGGCTGTCGGGAAGAACAAACTTCTGACGATAGCGACTGCTGCCACCGGGCGTTATTATGATTTTAGAGCTATCGAACCGTATGTGGATTATGTAAATATTATGGCCTATGATATGGAAGAGGCGCCTTTTCATCATGCTGCCTTGCATTGTTCGGACATGACGGAGGAATGGTCATGTGAGAAGGCAGTGGCCGGGCATATTGCCGGTGGTTTTCCGGTACAGCGTCTGGTATTGGGGATTCCTTTCTATGGACATGGAACCAACGAGGCGCCTGAGTCTTTGGATTATCGCCATATCATATCTATCGACAGTCTGTATAGTCGCTGGGATAGTGTGGCACAAGTTCCTTATTTGGTTAATTCCAAAGGAACAGTCGTCGTGAATTATGAAAATGCCCAATCCATCGAGCTTAAATGTCGCTTTCTGCACCGGCAAGGTATGCTGGGAGCTATGTACTGGGATTATGATAGCGACGATGAAATGGGCACTTTACGACACGCTGTTTATCGCGGGGTTATGCAACTTCCTTGA

>[denovogenes]_61999

ATGAAAAGAATAGTAAAAAACAGTTTGTTTACAGCAATATTGATGGTGTGGGGGATGATGTATTCCTCATGCGAAGACTGGACAGACCCAGAAAGCATTAACATCCATTACTCTACATTTGAAGAGCAAAACCCACAACTGTATGCTGACTATATCAGAGATCTGAAACGCTACAAGGATGGAGAACATAAGTTAGTCTTTGTCTCTTTTGACAATCCGGAAACTAACCCAATCAATCAAACCGAACGTCTCACCGCTATACCGGACAGTGTGGATTTTATCTGTTTGAATAATCCGGAAAAGCTCTCGTCTGAAACACAGGCCGAAATGGTAAAAATACGCGAGAAGAATATCCGCACGCTTTCCTGCATTAATTATGAGAGTTTAGAACAAGAATGGAACAATAAGGCAAAGGATAATTCCGAATTGACGGAAGAAGATGCACAAAGATATCTGAATGAACGTACGGATGCTATGCTGGCTCTATGTGACAGTTATGGATATGATGGTATCCTAATTGACTATACAGGACTTTCAATGGTGGGTATGCAAGAAGATGTGTTACAACAATACAAGGCACGTCAGCAAAACTTCTTCAGCAGAGTGTTGGATTGGCGGATTAAGCATACCGACAAGACATTGGTTTTTTACGGTTATGTGCAGTATTTGGCTCCCGAAAACATGGATATGCTGGATAAATACAATCATCTGATACTGAAAACCGCTTCTTCTAAAAACATGGAGGATCTGACATTAAATGTTTTTATGGCTATTCAAGCAGGCATAGACGTAGCAGGAACGAATGCGGATCTTGTTCCAAAAGATCGTTTTATTGCCTGTACACAGTTTCCTCAACAGGAAGATAAGGATATGATAATTGGTTATTGGGATACACGGGATGCAAATGGAAATAAAGTATTGGCTGCACAAGGTACAGCTCAATGGATTGTACAGACATCACCAGATTATACCTGCACTGGTATTTTTATCATCAATATCCAAAAGGATTATTACAATAATACTTATGGAACAGTACGTGAAACTATTCATATAATGAATCCTAATAAATAG

>[denovogenes]_76501

ATGTTAATGCTAAGGAAAATTGTATTCTTCCTTTTTTTATTTGTAGGATTATCGTTATTTGCACAACAAGATTATTTAGTGAGTTCTTATGTGCGTGGAAATTTTTACAATCGGGGACGTGTCGCAACTGAAAGTCTGCGGGCAAGTGACGACCTTATTTTCTTGAATGTTCATCCTAATAAAGATGGAACACTTTCATTTGAAAATCCGCGTGCATTTCAAGGAAAAGGGGTGACAACTTGGGAAGGATTGATTAAATCGGTTCGTGCAAAAGTAAAAGGTACAAAAGCGAAAATACGTCTGGGAGCTTCCAGTGGCGAATGGAAAGCGATGGTGGCTGATGAGGCTGCTCGTACGGCATTTGCAAAGAACATAAAGACTGTACTGGAAAAGAATAAGCTGGATGGCATTGATCTTGATTTTGAGTGGGCGGAGAATGAGAAAGAATACAAAGATTATAGCCTGGCTATCGTGAAGATGAGGGAGGTGCTGGGAGATCAATATCTATTTAGTGTATCTCTGCATCCTGTTTGTTATAAGATTAGTAAAGAAGCCATTGAAGCCGTTGATTTTATTTCACTCCAATGTTACGGTCCTTCACCGGTTCGTTTTCCGGTAGAGAAATATGATTCGGATATTCAGATGGTACTGAAGTATGGTATTCCCAAAGAGAAACTGGTGGCGGGGGTTCCTTTCTATGGTGTGACAAAGGATAATTCGAAGAAGACAGAGGCTTATTTCAGTTTTGTACAAGAGGGACTGATTACGGAGCCTGCACAAAATGAAGTAATATATAAGGGAGAGAAATATGTGTTTGACGGACAGGACAATATCCGCACTAAGACCCGTTATGCGATGGAGCAAGGACTGAAAGGCATGATGAGTTGGGATTTAGCCATGGATTTGCCACTGGATGATTCTAAATCATTGCTCAAAGCGATGGTTGAAGAATTAAGGAAATGA

>[denovogenes]_121873

AAAGAATTTGTGCAAAAAGTGAAAGGTACGAAGTTGTTGCAAGTGAGTCTACTTTCGTATATCGGTAAAGGAGCAACTCCGGGATCGGTATATGCTGATGCGGAAAAGCAGGCAGAGGCAGAAGGTTGGACTGATAAACAATTAGAAGAAGCTAAAAAGCAAGCTCGCTGGAAATACTGGGGATTTGAAGGTCAATTCGAAAGTGAAAATCACTATCAATGTCTGGCTAAGTTTGCCAAGGCCCTCTGTGACTCTCTATACGCAAACGAATGGGACGGTTACGATGTTGACTGGGAAATCGGAAGCGGTGTATTCGATATGGACGGAACATTGAGTGCTAATAAACATTTGATTTATTTGGTCAAGGAGATGAATAATTACATTGGTCCGAAGAGCGACCCAGAAGGCAAAGGACATAAGATGATTTGTATTGATGGTAGTATCGGTGGTCTTACCAGAGAATTGGATGAGTATGTCGATTATTGGATTATACAGAGCTATGGTTCTTCCAGACCTGGTCTTGAAGGTTATGGAGTTGATCCTAAGAAAATAATTTGCACAGAAAATTTCGAAGCCTACGCTCCTACCGGTGGCGCACTGTTGAGCCAGGCTGCTACTATGCCATCAAAAGGCTATAAAGGCGGAGTGGGGGCTTATCGCTTCGAAAAAGATTATGACAATACACCTGATTACAAGTTTATGCGTCAGGCT

>[denovogenes]_55512

ATGAAACATATAAAGAAAATGTTAGGATTTGCATTTGCTGCGACATTCTTTGTTGGATGCACGGATGTGGAATCTATTAATGACATAGATTTGAATCAAACAACTAAAACTGATGAATATTATGCTGCTTTGCGTGAATGGAAAGAGACACCGGGACTTCCGCAAGTGTTTGTATGGTTCGACAACTGGACAGGGACTTCTCCTACAGGTGAAAATAGCTTAAGAGGTTTGCCTGATTCTATCACCATTGCGTCCAACTGGGGTGGTCATCCTAAATTTGATTTAACTCCGGAACGAAAAGCTGATATGGAATATATGCAAAAAGTGAAAGGAACAAAGGTTGTCGTTACTCTCTTTTCTCAAAACATTGGTGATGATCTTCCTGATAAAGAAATTTATCACGAAGCCGGAACCAGTAGTGATCCGGAAAAAGTGAGACCTGCAATCAAAGCCTATGCCGAGGATATTTACAAGGCTTGCCTTGAATGTGGATATGATGGTTATGACTGGGATTATGAACCTGGTGGTGGAATGGGAGTAGGTCCGCTCTGGTCAAATAAAGTTCAGAGAACTATATTTGTAGAGGAACTTTCCTACTGGTTTGGACATGGCGCAATGGATCCCAACCGTGATCGTGGAGATCGCGCTATGCCGGAAAAACGCCTTCTCTTCTTGATTGACGGTGAGGTCGGTATTCGAACAGGTATGGATAAGGAATGGCTGTCATATTATGTAGATTATTTTGTATTGCAGGCTTATGGCGGAATGGCAGACTACAGAATGAAAGGGGTTCTTGACGATATGAGCGATTGGATTCAGCAGGGAATTATTACTCCGGATGAAATCGTTCGTAGAACCATAGCCACAGAAAATTTTGAATCGTATGCAAATACAGGGGGAGGATTTCTCGGCATGTCAAATTATGTATATAAAACAACGTATACCACCGGTGGAAAATCGTATGAAATAGATCAGTTGATTGGTGGATGCGGGCTTTATCGTGTAGGCTTTGATTATGCACAGGGTAAGGGTGATTATGATGGTTCACCGGAATACTATTTTCTGCGTCAAGGCATTACGAACATATACAAGAATTTCTATAGCCGTATGAATGCTACTGCGAATGAAGAATAA

>[denovogenes]_20169

ATGAGAAACAAATTCTTTTTATGTCTTTTCGTGTTAGTATCGCTAACAGCTTGTAAAGACACTAAATGGGTAGAAGTTCCGGGTGGAATGCCCCCTGAAGGAGCAATCACCGGACAGCCGGGAGTGGATGATCCGGCTGAGAAAACATTAATCAATTGTCAATACATTGGTGGTGACCCTTTCGAATTAAACCGTATTCCGGTAGAAAATCTGATTGTCAGCAATGATCTTATTTATTTGGCTGCGCGTCCTTATGCTAATGGAGATATAGCTTTTGACTTGCCTATAAATGATGCAACTTTTACAGGCGGGGTTTCGCATGAAGCTACTTATGAGGGACGTAGCGGTGTGATAAAATTTGACGGAACCGGAAAAATGAATGGAAAAGATGGATTATTGCATTCTACAGAAGAGACATTTAATAAAAGTTTCACTTTTGCTACCTATATATTTATAGATGAGTGGGTGCCCGGAGCATATATCTTTAAAAAAACAAGTGGAGATAAAGTGTTGGCTGCCTTACAATTAGGTGCTAACGAGCAGACTGTGAGCTGGGCGTTGGGAAATGCATCAGTGACAGCAGGTACCTATGTGGATGCCAAGGGTTTGGCTCCGGGAGCTTGGCATTATCTGGCGGTAGATTATGATGGCAATAAAAAGCAATTCAGGATTTTTGTAGATGCGTGGTCTTCTACGCTTGACAAAGAAATCGTATTGCCTACTGCCCGTGCCGATTTTTATATAGGTGAAAACTTCAAAGGCCGTTTGGATGAAACTTCTTTTTGGAGTATGGCAGCAGGTACTGGTGGGAAGAATGGTATAAAATTCGATAATTGGAATATGGTAGCAAAAGTGTTGGCTTATTGGCAATATGATGATTCTACCAATCCGGGGAAGGACTCTCACACTTGGTTGGACCGTTGGAAAAAAATACGCGAAACCTTGGCAGCTCAGGCAGGTAATGACAGCCGAAAATTACGATTAGGATTCGGCAGCGGGCAATGGAGGCAAATGATGAGTGCTGATAATACGCGTACCGCATTTGCTAATAATTTGAAGAGTGTTTTGAAGGAATATGAATTTGACGGAGTAGACCTTGATATTGAATGGCCTACTACGGAGACAGAATATGCTAACTACAGTGCGACGATTGTGAAAATCAGACAGATACTTGGCAAAGACGTATGTTTCTCCGTATCCCTTCATCCGGTTGCTTATAAAATTTCGAAAAATGCGATTGAAGCTTTGGATTTTATGTCTTACCAATGCTACGGACCGGCAGTGATGCGATATTCTTACGAGCAATTTGTAAAAGATGCAGAAATGGCTGTTGAGTACGGGATTCCGGCAGATAAACTGGTTCTCGGTGTGCCTTTTATCGGGACTACCGGTGTAAATGGAGAACAAGTCTCTTACTCTGATTTTATAAATGGTGGACTTTCGGATGTTGCTTTGGATGAATTTACGTATAATGGCAAAACGTATACTCTTAATGGTCAGAATACGATTCGTAAAAAAGCGAAGTATGCTTGTGAGGAAGGCTATAGAGGAATAATGAGTTGGGGGTCCGATGTGTCGGTAAATAATGAAAAATCATTGTTGAAAGCGATACAAGAGGAGTTTGTTGCTTATGAGATTAATAATCCCAAGTAA

>[denovogenes]_56831

ATGAAACGATTATATAAATATTTCTTTGGAATTTTAGGAATATCATTTGCTTTGACGGCCTGTGATGATTGGTTGGATACGGAAATTAAAGATCCTGCGAATCTGACTATATCTAATAAGGATGAGGCTTATTATGCCCGTCTGCGTGAATATAAAAAGAGTGACCATCCGGTTGCGTTCGGATGGTATGGTAACTGGACGGGAACCGGTGCTTCTTATGAGAATTCCTTAAAAGGATTGCCGGATAGTGTAGATTTTGTGTCATTGTGGGGAAACTGGAAGAATCCGTCACCTGCTATGATGGAAGATTTACGTTATGTGCAAGAAAAAAAGGGAACGAAGGTATTGTTCTGTTTCTTGGTGCTTGATATTGGCGACCAGATTACACCGCCGATGCCACAGGAGGAAATAAACAATGGTACATCTATAGAAGACTGGAGACATAAGTTCTGGGGGTGGGACTATTCACTGGAGAATCGGTTGGTTGCAGTGGAGAAATATGCGAACGCCTTGTGTGATACGATCGAAAAGTATAATTATGATGGGTTCGATTTTGATGCTGAACCGAATGTACAGCATCCGTTTCCTACCGATAAGGAACTTTGGCAAAATAACGGTCAAGTAATCGCGAAGTTTGTGGAAACGATGTCGAAGAGAGTCGGTCCGAAGTCTGGTACGGGAAAAATGCTGGTTGTGGATGGTGAACCGGATGCACTGCCTGCTGAGCTGTTTCACCATTTTGATTATCTGATTCTGCAAACGTATACAACTTATTATAAGCAAGACAACAGTCGGCTGGATGCTCGCTTCGACAAACAGTATGCCCACTTCAAAGATGTGGCGACAGCTGGAGAGATTGCGAAAAAGATTATTATCTGTGAGAATTTTGAGGATCATGCCAAGACTGGTGGGGCGGCTTTTATACTTCCTGACGGCACGGAGATAAATTCACTTAGTGGTTTTGCTTATTGGAATCCGTCGGTTGGTGGCATACAATATCGCAAAGGCGGTGTAGGAACTTACCATATGGAATATGAGTATAAAGTGAATGCACAAGGGAGCGAAACTTATCCGGCGTTGCGTAAAGCCATACAAATTCAGAATCCAAGTATTAAGTAA

>[denovogenes]_21169

ATGTTGACAGCTTGCAAAGACACAAAATGGGTAGATGTTCCTACCGGTACTCCTCCCGAAGGTTCAGTTACGGGAAAACCGGGCGAAACAGAAGAACCCGATGATCCTGATCCTACAGATAAAACATTTATCAACTGTTCTTATATCCGTGGTGATTTTTTTGAAACGAATCGAATTTCGGGAGCAAGTATGGGTGCTTGCAACGATTTGATTTATCTGACGGCACGTCCTTATGCTGACGGTGATTTGACTTTTGACTTACCCGTGAATGATGCAACACTTACAGGGGCAGCAACGTATGCTGCTTCTTATAATGGACGCAACGGAGTACTCAAATTGGATGGTACCGGCAAGATGAACGCCGGCGACGGTCTGTTGCATTCACCGGACGGAGCATTCAAGAAATTCACATTCGGTACTTATATATATGTCAGCGAGTGGGTGGACGGAGCTTGCTTGTTTAAAAAGGTAGACAGCGGTTCTGTTGTCATCGCTTTCCAGTTGGGAGCAACGGAAGGTAATTTGAAACTGACTGTCGGAAGTGCTACGGCCACAGTGACGAATAGCGCATTGAAATCAGGAGCATGGCATTATGTAGCGGTGACTTATGATGGTGGCGCTGCTAAGTTATATATAGATACGAACAATACTGCCACTGACTTTACAGGCTCTCTGCCTGCTTCGGTTCCTAATACCCGTGCTGACTTTGTGATGGGAGAGAACCTGAAAGGTTATCTGGATGAAACATTTGTGAACAGTCTGCTGATGGGTACATTAGGAAGAAATCCCATCTCTTTTGATAATTGGAATAACACGAAAACACTTGCTTACTGGAAATATGATGACGCTGCAAAACCTGGTAAGGACTCGCATACATGGGCTATTCGTCTGGAGCAGATTCGTACTGCCTTGAATGGACAGGCAGGTGACCGGAAGATACGTTTGGGTATTGCTGGCGGCGAATGGTTGAAAATGGTCGGCAACGCAACTGCCCGTACTAACTTTGCCAATAACGTAAAGAAGGTGATCGAGCAATATAATCTGGATGGTGCGGACCTCGACTTTGAATGGGCTTATTCAGGTACTGATTTATCCAATTACAGTAAAGCCATCCTACAATTGCGTGAAGTATTGGGGAAAGATGTATTTTTGACCGTATCCCTACATCCTGTTTCTTACAAAATAAGTGCGGAAGCTATTGCAGCAGTCGATTTTATCTCGTTGCAATGCTATGGACCTTCAGTAGAACTTTTCTCTATGGAACGTTTCAAATCGGATGGTAAAGCTGCAGTCGATTATGGAATACCTCAGGATAAACTGGTCATGGGAGTACCTTTCTACGGGACTACGGGTACTGCCGGCGAGCAGGCTGCTTATTTTGATCTTGTCGGTAAGGGTAACCTGACTAATACTTCTGCAGACACATGGAGTTATGAAGGAAAGAACTATACGCTCAACAGCCAGAATACGATTCGCCAGAAGACACAATATGTGTGCGAGAATGGTTTTGGAGGAATCATGAGCTGGGACTTGGCTACAGACGTAGACGTGACGCATGAGATGTCTTTGTTGAAAGCAGTGAAAGAAGAGCTTGATTACTATGCCAATCCTACTGTAGAATAA

>[denovogenes]_24780

ATGAAACAAATATTGAAACATATACTGATTCTGGCATTTGCGGCAGGTTTTATTGGGACTGCCTGCGAAAATAACGATCTTACTATCGATGCCGGTACATTCCCCGAAACCGGAGGTATAGGTCTTTCTATGGGTATATTACAAAGCGATAATTATGCGATGGAAAACCCGCAGATAAACATGGACCATGCCAGTTTGAGCGATCAATTTCACATCAGCTTAACAGAACCGGCTTCACAGACAGGAAATTACACCGTAAAAGTAGACGAATCCAAAGTACTGGACTTCAATTCGAAGCACGGAACGAGTTACCCTCTGTATCCTACAGAATACGTAGATTTAGGTAACAGTGGAAAAATGACAATAGAGAAAGGAGAACAGCAGTCAAACTCTGTCTCTATTGCCTTCAAATATGATGAAGCCATAGAAGATTCTGTTATTTATGTACTCCCTCTGACAGTGGAAGAGAACAATAGTTCCCCGGCCATGTCTAGTGAACGCAAAACATTATATTATATAATTAATGTATGGGGAATGGCTCCTGCCGAATATAACGCTATAAAGAAAAACTTCATTCAGATAGCAGGGGTAGATCCCGAATTCACCAATCCTCTACTGCTCAACAAACTCTATTTTGAGTCTATGTCTCTTTCATCACCAGAAGTAGATTACTACAATCCATTCGATATAATCAACCTACAGTTCGCTACCGTCAAGGCAGACGATAATCTGTTGCCATCCTTATATCTAAAAGATGATCTGGCATACGTACTAAAGAAACGTGAAAAGTACATCGTTCCATTGCAACAGTTAGACCACAAAGTTTGCCTTGCCATTAAGGGAGCGGGAGAAGGAATTGGTTTTTCCAATCTGGGGGAAAAAGAAATGATGATATTTGTAGAAAGAATAAAACAAGTGATTGATATATATCATTTAGATGGAGTCAATCTATATGATGCTAATTTTTCCTATGAAGAAAGTAATGAAAACATTAATTACTCAAACAATCTATGCAAATTCGTAGCATCTTTACGAGATAAGCTAGGAAACAAGATCATAACCTATACACAAACTTCTGAATCTCCGGAAGGGATCACTAATGACGCTAATTTGAAATTAGGAGAACTTTTAGATTATGCTTGGTGTGATCAATTGAACACAATTATTGATCCTTGGAGTACTCCTGAAAAATGGACACGTCCTATTGCAGGATTAAACAAAGAAAAATGGGGAGCTTTAAATACTGATATACATATGTCAAGTGAGCAAGCAAATATTTTAGATCAAGTGATAGAAATGTTTACTCAACCCTCACTAATGATAACCGCTGGAATAAATCATGTATTTGTAGTAAATAGAGTAGACTATGTTAGTGCAGGAACGGAATCTTATGCACCGACTTATATGGCTTATGGTGCTATCTGTAACTTATGTGATATGGAAAAAGAATATTTTGTAACAGGTATTAACTCTCCAAATAATCAATACCTCAACATTCATGATCTTCTCATGCCTAAAGATTATTAA

>[denovogenes]_55984

ATGAAAAAGAATTATTTACTATATTTTTTATTGTTAGCGTTGAGCACCCCAATATGGGTGAGCTGTAATGACTGGACAGAATCAGAAGCAAAAGATTACTTCGAAGGTCCTTCAGAAGAATACTATGCAGCCCTGCGCGCATATAAAAAGTCTGATCATCCCAAAGCTTTCGGATGGTTCGGCAACTGGACAGGCGAAGGCGCTTCATTAGTAAACAGCATGGCAGGAATCCCGGATAGCGTGGATGTCGTTTCCATCTGGGGGAATTGGTCGAATATTACAGAAGCGCAAAAGAAGGATCTCAAGTTCTGTCAAGAAGTAAAAGGAACTCGCTTCACCATGTGCTTTATCATCACTTCGGTAGGTACACAGATCACTCCACAACATATATATGACAATTGGGAGTCAATGGGATTCGCAAGCCAACAGGAAGCTGTTAATGATTTCTGGGGCTGGCCTTCGGACGAGTCTGATAAAGAAGCCGTCGAAGCATCTATACGCAAATATGCAAATGCTATCGCTGATACGATCAACAAATACGGCTATGACGGATTCGATATCGACTACGAACCAAACTATGGTAATAAAGGTAACATTGTGGACGACGATGACAGAATGTTCATATTCGTAGACGAACTTGGCAAACACTTCGGTCCTAAATCGGGAACCGGCAAGTTATTGATCATTGACGGCGAACCGCAAAGTATAAAGAACAGACCCGATGTAGGCCCTTACTTTGATTACTTTATCATTCAGGCCTATAAACCCGGTAATGACAACAATCTGGACAAACGCCTGATAGACGGTGGTGTAGCAGGTCCGGGACTCGTTCAGACTTACGGTAGCGTAATGTCCGAAGAGCAAATCACAAAGATGACCATCATGACCGAAAACTTTGAGGCTGTAGATGCTGCTATGGATGGCGGATATCCTTATACCGACCGTTATGGCAACAGCATGAAATCTTTGGAAGGAATGGCACGCTGGCAACCGAAAAATGGTTTCCGTAAAGGCGGAGTAGGAAGCTATCATATGGAAGCAGAGTATCCGACAAATCCGGAATACAAGAATCTACGCAAAGCTATCCAGATTATGAATCCTTCTACCAAACCGTTAATTAAATATTAA

[denovogenes]_61438

ATGAAAAATAACCTGAAATATAGATTGTCTGCTCTGCTGTTTCTGGCATCAGCTCTGACAATAACTTCATGTAGTGATTGGACAGATGTGGAAAGTCTCCAATTGAATACTCCTACATTTGAAGAGCAGAATCCTCAGTTGTATGCTGACTATCTGAAGGATTTGAACAGATATAAGTCTGAAGAACATAAGGTCACTTTTGTTTCTTTCGAGAACCCTAAGGGATCTCCCGGCAAGCAGGCTGAACGTCTGACCGTCGTACCTGACAGCGTTGATTTCATTTGTCTGAATAATCCGGAAGTAAGTCCCGAAGTACAGGCAGAAATGGTCAAAATACGCGAGAAAGGAACTCGTACGGTTTACAGTATTGATTATTCCAGCATTGAAAATGCCTGGAAGGAGAAAGTAAAAGCCGAACCGGAGTTGACAGAAGAGGATGCTCTTCAGTATATCGGTGAACGCACCAATGAGATGTTGGCACTCTGTGACAAATATAACTTTGATGGTGTTATTGCTGATTATACAGGGCGTTCGCTGGTGAGCCTTCCGGAGGCAGCTCTGAAAGAATATAATGACCGTCAACAGAAGTTTTTTGGCGAGGTGATGAACTGGCAAGGCAATCATGATGACAAGACTCTGGTATTCTACGGAAATGTACAATATTTAGTGCCGGAGAATATGGATATGTTAAGCAAGTTTGACTACATCATGTTGAAAACTGCTTCTTCTACCAATGCTGATGATCTTGCTTTAAAAGCATATTTGGCTATTCAGGCGGGTATAGATGCAGTAGGCGGTACAGAAGGAGGAGTGAATCCTGTTCCTGCTGATCGTTTTATCGTTTGTGTCGAATTGCCGCAGGCTGACGATAAGGATAAAGTGAAGGGTTACTGGAGTACAGTGGATGAAAAAGGCAATAAACTAGTAGCAGCTCCGGGAGCAGCCCAGTGGATGGTTGAAGCATCTCCTAATTACACCCGGAAAGGAATCTTTATTATGAATGTCCATAATGATTATTATAACAATACTTATGGATATGTGCGTGAAGTTATTCGTATTATGAATCCTAATAAATAA

>[denovogenes]_65266

ATGAAACATGTATTATACGCATTATTAACGGCTGTAAGCATTTTATTCACTAGTTGCGGACCCTCTTCTAATACCAACAACCCACAGGTGCCCAATGTGCCTCAACCGCAGCCTCAGCCCCAACCGGAAGTCACCCAGAAAGTAGTTATCGGCTACCTTGCGCTTGACGACTGGGAGTTTGAAAGTTTATTTCCTACGATAGAATGGAAATATCTGACGCATATCAATGCCAGCTTTGCAAGAGTAAAAGCAGACGGCACTCTAAACATTAATCCTGTCCGGAAGAGAATTGAAAGTGTACGTGAAACGGCTCACAAGCATAATGTCAAGATTCTGATATCTTTGGCTAAGAACAGTCCGGGAGAATTCACTACCGCCATCAATGATCCGAAAGCCAGAAAAGAACTGATACAGCAGATTATTGCATTCACTAAAGAATATAAACTGGACGGCTTCGACATCGATTATGAGGAATATGACAACTGGGATAAGAATTTCCCCTCATTATTGGTTTTTGCCAGAGGTTTATACCTGGCAAAAGAGAAAAATATGCTGATGACCTGTGCCGTCAACAGCCGCTGGCTTAATTACGGAACCGAATGGGAGCAATACTTCGACTATATCAACCTGATGAGTTATGACCGGGGAGCGTTTACCGATAAACCGGTCCAGCATGCTTCATACGATGACTTTGTGAAAGACCTCAAATACTGGAATGAACAATGTCGGGCATCCAAAAGTAAAATAGTGGGAGGACTTCCTTTTTATGGATATTCCTGGGAGGAAAGTCTGCAAGGCGCAGTGGATGACGTTCGTGGAATCCGCTATAGCGGCATACTCAAACATCTTGGAAATGAAGCAGCGGACAAGGATAATATAGGCAAGACTTATTATAACGGCCGCCCTACTATAGCCAACAAATGTAAGTTCATCAAAGAAAACGACTATGCCGGCGTGATGATCTGGCAATTATTCCAAGATGCCCACAATGATAACTATGACTTAAAGCTCATAAACGTTGTAGGCAGAGAAATGATGGAATAG

>[denovogenes]_82405

ATGAAAAAATTATTATCTGTTTTTGCAACATTGATGATGGTTTTGTTTGCTACAGGTTGTAGTAACAATGACGACATGGTAGTAGCATCTCAAGATGCCCAAACTACTGTTACCCGTGCTGTGTCCGATAAGACTCCAAAACTGACCACCTATATCGAAACAAACGATATTAATCCGTTAAATATGGGTGAATACTACTTCTGTGGCACCAATCCCAAAGACTATGTAGTAGATCATGTAATACTGTTTGCATCAAACATTCGCGGTACGGCTTCAACAGTTGAGTTGTATCACAATCCAAACCAAAGTCATATTTTGGCAAATGTAAATACACTAGTACGTCCATTGCAAGATAAAGGGATCAAAGTATTGCTCGGTTTGCTAGGCGACCATACAGGTGTCGGCTTCGCTAATCTGAACGACGCTCAGATAGAGTCATTTGCGCAGCAGGTAGCCGATTGCGTCAATAATTACGGACTCGACGGAGTGGACTTTGATGATGAATATGCTGAGTATGGAAGAATATCAGGTACCCCTTCTCCTAGCAGTGCTCTCTATGGCAAACTAATTCAACGTTTACGTGAGATAATGCCCAACAAACTGATCACTGCTTTCTATTATGGTTATGCCGGCGGTTTCAATCAATATGCTGATTACATGTGGCCTAATTTTGGTTCAGGCTGGAGTGCTCCTAGCGGATTTGCACACAGCAAATGGGCTAGTATGTCAATACAATATACCCAAGGCATCCCTAGCGATAGAGAAATTCGAGCTGCCGCACAAAACTACGGTGATTATGGCGCCATCATGATGTTCAATGTACGCGATACAGATGCTTCAGGAAAGATGAATCTCTTTGCTCCATACGTATGGGGTGGCAAGACAGTATGTTGGACCGGTGTTTCTTATCCAAAGAATTACTAA

>[denovogenes]_100953

CTGGCCGATAAGAATGATACGTTGGTATTTGGTTTTGAGAATCCCCGTTCACTTGCCATCAAGTGTCAGTATATTCTGGATAAAGATTTGCTGGGAGGCATGTATTGGGATTATAGCGGAGACAATGAACAGGGTGATTTGCGCCGTACGGTAGCAGAAAACCTATTGGGCAAGCCGCACAAGGCGAAGGTACTGGTGTTGACGGAACGTGGCGGACAGCACGGAGGATTTACGGATGCCGGTTTGAGGTGGTTGGCGGCCGAAGGTGTAAAGGGGAATTTCAGTATTACCGAGATAAACAATGCCAGGAACATAACGGAGGCTTACTTGTCGCAGTTCAGCCTGGTTATTCAATTGGATTTCCCGCCTTATACTTGGCCGAAGGAAGCGGAAGATGCGTTCGTCAAGTATATAGAAGAAGGCCGTGGCGGCTGGATAGGTTTCCATCATGCTACTTTGCTGGGCGAGTTTGACGGTTATCCCATGTGGCAATGGTTCTCTGATTTCATGGGTGGTGTACGTTTCAAGAACTACATAGCTCCGCTGGCCAACGGTACGCTGATTGTGGAAGATAAACAGCATCCGGTAATGAAAGATGTTCCGGCCTCTTTCGTGGTGCCCGATGACGAGTGGTATACTTATGACAAGAGCCCGCGCCCCAATGTACATGTACTGGCCAATGTAGACGAATCGAGCTACACGCCTGCATCGGACATCAAGATGGGCGATCATCCGGTGGTTTGGGTCAATGAAAGCAAAAAGGCACGCAATGTTTATTTCCAGATAGGGCATAGCAGCAAATTGTACGAAACGGAA

>[denovogenes]_60040

ATGAAAGTAAGATACATTTATATAGCACTTGTAGCATTGCTTGGAGGTATATTCTGGGGATGTAGCGATTGGACGGAAGTCGAATCAAAAGACTTTTTCAATCCACCCACAGATAGTTATTATGAAGCTTTGCGTGAGTATAAAAAAAGTGATCATGAAATCTCTTTCGGCTGGTTTGGTAACTGGACAGGAGTAGGTGCTTCACTGGTAAATAGTATGAGAGGGATTCCGGATAGTGTCGATCTGGTTTCTATTTGGGGAAATTGGAAGAATATCACTCCTGATCAGGATGCAGACTTGAGATTTTGCCAAAAGGTAAAAGGTACGAAGTTCCTACTTTGCTTTATCGTACATAACTTGGGCGACCAATTAACTCCTGAAGGACAAACTATTCAAGAATATTGGGGATGGGAAGGAGAATTGATACCGGACAGACCTTACCAAAGATGGGAAATGATTGATACAGAGGTAACTCCTGATCAAGAAACCATTATCCGTAAATATGCAAAACAAATCATAGATACGATCGCTAAATATAACTATGACGGTTTTGATATTGACTATGAGCCTAACTATCAAGGAAAATGGGGCAGCTTAGCTAACTACCCTAAAAGAATGTCTGTTTTCATTGACGAATTAAGTAAATATCTTGGTCCGAAATCAGGAGGTGAAAAAATATTAGTAATAGATGGTGAACCTCAATCAATGCCTTCTGAAAGAGGAGAATGCATGAATTACTTTATAGTACAGGCTTATGAATGCTCGGGAGATGCAAATTTAGATTCCAGGCTAAAATCAACAATTGATAATTTTGATGGCTATTTATCACCTCAAGAAGTTGCAAAGAAATATATTGTAACAGAAAACTTTGAATCTTTTGCACAAGATGGTGGTGTTGCTTTTACTGATCGCTATGGAAATAAAATGCAGTCCCTTGAAGGTATGGCTCGGTGGACACCAATAATAGATGATCAAAAGGTTAGTAAAGGGGGAGTCGGTACATATCACATGGAATATGATTATCCATTTACTCCGGAGTATAAATGGCTTCGTGCAGCTATACAAATCATGAATCCTTCTGTTAAATAA

>[denovogenes]_55052

ATGAATATAAAGCATATAAAAAATGCATTCGGATTTGTATTTGCCGCTATATTCTTCGTTGGATGTACGGATGTGGAATCTATTAATGATGTTGATTTGAATCAAACAATTAAAACTGATGAATATTATGCTGCTTTGCGCAAGTGGAAAGAAACACCGGGACTTCCACAAGTGTTCGTTTGGTTCGACAATTGGACAGGAACTTCTCCTACTGGTGAAAATAGCTTGAGAGGTCTGCCTGATTCTGTAACCATTGCTTCCAACTGGGGTGGGCATCCGAAATTTGAATTAACTTCGGAGCGAAAGGCTGATATGGAATATGTACAAAAAGTGAAAGGCACAAAGGTTGTTGTTACCTTATTTTCTCAAAATGTAGGCGACGACCTTCCGGATAAAGAAATTTATCACGAAGCTGGAACCAGTAGTGATCCGGAAAAGGTGAGGCCTGCAATTAAAGCCTATGCAGAGGATATTTATAAAGCTTGTCTTGAATGTGGATATGATGGTTATGACTGGGATTATGAGCCTGGTGGTGGAATGGGAGTTGGTCCGCTTTGGTCAAATAAAGTACAGAGAACTATTTTTGTCGAGGAACTTTCCTACTGGTTTGGACATGGTGCTATGGATCCTAACCGCGATCGTGGAGATCGCCCTATGCCAGAGAAACGCCTTCTTTTCTTGATTGACGGTGAGGTTGGAATCCGAACAGGTATGGATAAAGAATGGCTTTCATACTATGTAGACTATTTTGTATTGCAAGCTTATGGTGGAATGGCAGATTATAGAATGACAGGCGTGTTGCGTGATATGGATGATTGGATACAACAGGGGATTATTACTCCGGATGAAATTGTCCGTAGAACCATAGCTACCGAGAATTTTGAATCGTATGCTAATACTGGAGGAGGATTCCTAGGTATGTCAAACTACGTATATAAAAATACTTATACTGTTAATGGAAAACCTTATGAAATAGACCAGTTAATTGGTGGATGTGGTCTTTATCGTGTAGGCTTTGACTATGCACAAGGTAAGGGCGATTATGATGGTTCACCGGAATATTATTTCTTGCGTCAGGGAATTACAAACATATATAGGAATTTCTATGATCGCATGAATGCTGCCAACGAAGAATAA

>[denovogenes]_134103

ATGAACAAAAAAATTCTCTCACTTCTGACAGTAGCCACTCTGTCATTGAGCGCAGCCAGTGCAACCAGTTTGGTTGTAGATGGAGTTCCAATTCAGACTGATGTTCCGCCAGTTGTAATAGACGGCAGAACTCTGGTTCCTGTTCGTGCTCTATTTGAATCGCTCGGAGCGACTGTCGGCTGGAATGAAGCAACACAGACTGCAACAGCTACCAAAGGAGAAACTATTGTTTCTGTACAAATCGGCAGCACTACTGCCCATGTAAGCGGTGTTGCAAAAACTTTGGATGTCCCTGCACAGACTATAGAAGGGCGCACAATGGTTCCCGCTCGATTTGTTGCGGAATCACTGGGTGCAACTGTAGCTTGGGATAATGCAACAGAAACCGTGAAAATTTCCACAGCGAATCAATCCGCTTCTTCCCCGTCTATCCCTGACAAGGCAACTCCAGCTGAAAAACCCACCACGCCTACAAAACCTTCCACTCCCGCTCCATCCAAACCTGACAGCAGTGCATCTACTACAGTTTATATAACCAAGACGGGAAAACGGTATCACTATGATGGAAACTGTAATGGCGGTGCTTACACTCCCAGTACGCTTACAGAAGCAAAGGGTCGTGGACTTACCCCTTGTAATAAGTGCGTTCAGTAA

>[denovogenes]_106655

ATGATGCTTAGTATAGCTACAGGAGTATATGCACAAGAAGTGACCAATATTTCTACATTAGAATTTACTAAACTTTTAAAACCACTTGATACAAGTAATAGATTAAATATAAATGAAAGTTCTAAGGCACTGCTTACAAGAGAGAAGGCAGCAGCGCTGATTATTAAGCTTATGGGTTATGAAGGTATTGCAAAGGACTATAAAAATAGTACCCTTTATAAGGATGTAACGGCTTATAAAGGAGAGATACATATTGTAAGTCAACTCGGTATTATGAGTGGCACAGGTACAGGATTATTTACGCCTACAAAACCAGTGACTTATGAGCAAGCTAAAATCATTACCCAAAGAATCCAAAATAAGCTAAATAAGCCAGCAACATGGAATCATGCTTTTTATGCTATTTCTTCTAGTAGCCAGATGGAGCTTATACCTACTTATGATGCAGTGAGTTTTGGTTGGGCGCAAGTAGGTTATGATGCAGCAAAACAAAGCTTTGGCATTCAGACTACCCAGGGAGATTTTAAAGTACCCACAGGTTTTTACAAACCACTCGATTTAGCAAAATCTAATGGTGTAGAAACTTATCTTATGGTTTTCTTTGAAGACAAAAATGGAATGGCTAAGCAGCTTCTTAATAATGAGACGCAGGTAGATAAACTCATTAAACAGATTGTAGACTTAAGTAATGGCATTACAAAAGATGGTGTAACCAGGGCCTTTGATGGTGTAACCATTGACTTTGAAAACTTTATAAGTAGTGATTTAGCGATGCCATATACT

>[denovogenes]_7860

ATGAAACTAAATAAATTAACAGCGGCAATACTTGCTGCTGGGATGTCATTGTCTTTTTCATCATTTGCTGCAACGTTGTCGATGAATGACGACACTAATACAATCAGCGGCCTTGATAATAGTCAGATGTTGACGAAGGATAATGGTAATACCTGGGTCGCTTATACAGACAGTAGCCAAAATAATTTCCCGGGTACGGTGACGGTAACCGTAGCGGAAAAGGATAATAACGCAATTAATTCTGTAGACTATGATCCCACTGCCACTTATGGTACGACAGGAACAATTGTTCGATATAATGGCTATTACTGGAAAAGTCAGTGGTGGGTTGATCCCGGTATGGTTCCAGGAAGTGACTCTGTATGGCTGAAGGTCGGTCCTATTCAAATACAAAATCTTGCCACGTTCAGTTTTACGCCTTATACCGGCCAGAAAGCGGCAGATCTGCAAAAACAAGGTAAAGATAAGGCTGCCGCGCAACGTAAAGTTATCGGCTATTTCCCTGAGTGGGGCGTTTATGAAGCACATAACTATTTCACGCCGGATAAAATTGACTTCAGTCAGTTAACCCACCTTAACTATGGCTTTGCGGTTATTAAGGATGGTGAAGTTATTGTCCATGATACCTATAAAGGCCCGGATCTGCTCCGTCAGCTGGACAAACTGACTGAGCAAAACAACGTCACAAATATGGTCTCCGTTGGCGGATGGAATAACTCTGAAGAAGGTGTATTCGAAGAAGCCACCAAAACGGATGACGGTATCAATAAACTGGCAGACAGCATGATTGCTTTCATGCAGGAGTGGGGGTTTGATGGTATTGATGTAGACTGGGAATACCCGGATAGCGATACTGAAAAGACCAATTTTACTAAACTTATCCAGACGCTCAGAAGTAAACTGGATACCCTTGGTTTACAGAGCGATAAGTACTTCCAACTTTCCGCAGCAGTAACAACCAACCATAATAATATTGAGTACATCAACCCAGAGGTCACCGCACCATTGCTGGATAGCGTCAATGTCATGGCATATGATATTCATGGCGCTTTTGACCCTATCACAGGCCACAATGCTCCGCTCTATGCCAACAGCCAGGATGCCGATCAGTTGCTAAATGTCGCTTCCACGATGCAGGAATACAGTTCGAAATGGCATGTGCCCAAAAGTAAGCTGATGATGGGGATCCCGTTCTACGGCCGTGGCTGGGGTAGCGTGGCGCCGACGGAAATCGTTAAAGGTCTGCCTGGTCTGTTCGCTTCAGGTTCCGCTACCGTACACGGCGCGTGGGATGATGAAGATCAGTATACTGGTACTAACCCCTACTATCTGTTAAAACAGTATTCTTCGTCTGCTGACTACACTCGCTACTGGGATCCGGAATCACAGGTTCCTTACTTGTATAATGCGAAAACGCAAGAGTTCTTGACGTATGACGATCCGGAGTCGATTCAGAAAAAAGTTGCCTACATTAACCAGCAAGGATACGGCGGAGCGATAATCTGGGATATTAGCGGTGATACTCCAGAACATGAATTGGGTGAGATTGTCGATGATATAATGGAAGTGCCTTTATCCGAATGCCGCGCGAATCTGTCAGATTTTGGCATATATCTGCGAGACGGTACGCCGATGACGGAATTCACCATCACCAAAGCGGCACATGAGGCCAGGAATATGAACTATTCTGTTTATCAGAATGGAAAATATTACGGTAAGAGCCAGCACGGCACCATTTATGATTGGGGTAAAAAAGAAATTAATGAAGAGGAAGGTACTGTTACTGCGAGCTGGGGAATGCCGCTGAAAGTTGGCGATCTAATTGAACTGAGCTGCTACAGCAATGGTGTACTCTACCCACTGGAGCAAGTAACGGTGACAGAAGAAACTCTGGCAGGTAAAAAAGATATGCGTCCGGAGATTGCCAGTGAACTGAAAGAGTTCCAGGTTGTTATTCAGGACGACAAGCCTGCGTTAAGTATGACTCTGACTACTGCCGCTCATGAGGCAAGAAATAAAAACTACTCTATTCAGTTAAATGGAAAATATATCGGCAAAAGCCAGTTTGGTTCAATCAGTGGTGGTCAGAAACGCGTTAATAAGGCGAATGATACCGTCACGGGGACCTGGGTCAAAGAATTGAAAGCGGGAGATACTGTTCGCTTGTATCGTTTCAGTAATAGCGTTGAAACGACTATTGCAGAAGTGGTACTTACAGACGATATTCTGAAGGATGGTGGTGCTCTGGTCCATTAA

>[denovogenes]_43771

ATGAAACTATTGCCTTTGTTGGCAGCATTCCCCCTGCTCTGCGCCAGCGTTGTTTCCGCCAATTCACTGATGTCAGTTGGCTACTTTAATGGCGGTGGCGATGTCACTGCCGGTCCAGGCGGCGATATCAATAAACTGGATGTGCGCCAGATAACCCACCTCAACTATTCGTTTGGTCTTGTCTACAACAACGAGAAAGACGAAACCAATGACGCCCTAAAAGACGCCAGCAAGCTGCATCAGATTTGGCTTTCAGAGAAAGTGCAGGCTGATTTGCAGAAAATCCCGGAGCTGCGTAAGCAGAATCCGAATCTGAAAGTTCTGCTTTCTGTCGGGGGTTGGGGGGCGCGTGGATTCTCAGGCGCGGCAGCAACGAAAGAAACACGAGCGATATTCATTCAGTCTGCGCAGGAAATCATTGAGAAATACGGTCTGGATGGTATCGACCTCGACTGGGAATATCCGGTCAATGGCGCATGGGGACTGGTCGAAAGCCAACCAACCGATCGCGCTAACTTTACCGCGCTGTTAAAAGAGCTGCGGGCAGCATTAGGGCATAAAAAGCTACTGACCATTGCCGTGGGTGCGAATGCGGAAAGTCCGAAAAGTTGGGTGGATGTAAAAGCCATTGCGCCATCACTCGACTACATCAATTTGATGACCTATGACATGGCCTACGGCACTCAATATTTCAACTCCAACCTGTATGATTCGACGAAATGGCCGACCGTCGCCGCCGCAGATAAGTACAGTGCGGATTTTGTGGTCAATAACTATCTCGCCGCCGGGTTAAAACCGAGCCAGATGAATCTGGGTATTGGTTTTTATGGACGCGTACCAAAACGCGCCGTTGAACCGGGAATTGACTGGAGCAAACCTGACGCGCAGAAGAACCCTGCCACCCAGCCCTATTTCGAAGCAGCACAAATTGCGTTGTTCAAATCATTGGGCGTCGATCTGAGCAAAGATACCTATGTGAAGTACAACGACATCGTGGCGAAGCTGATCAACGATCCGCAGAAGCGCTTTAGCCAGCATTGGGATGATGAGGCGAAAGTACCGTGGCTGTCGATACAGTCAGCAGACGGGAAAGCGCTCTTTGCCCTCTCCTATGAAAACCCGCGCTCGGTGGCGATTAAAGCCGACTACATCAAGAGTAAGGGACTGGGAGGCGCCATGTTCTGGGAATACGGTGCGGATGACAACAATCAACTGGCTAAACAGTTAGCCGATTCATTGGGCATCAAACACTGA

>[denovogenes]_101794

TATAATTCCCTTGCCAGCAAGGAGTACGTGGACAAAGCCCATGAGATGGGGCTTCAGGTATGGGCCATGGTAGAAAACGTAAGCACGCAGGAAAGCGTGAAGAACCTTAATACCAAGACACTTATGTCCTCCACCAGCACGCGCAGGAAACTGATTGAAAACCTTATGAAGGAAGCCGATACCTATGGATTTGACGGATTCAATCTGGATTTTGAGAGCCTGAAGGCAGAGGCCGGCCCCCACTATGTACAGTTCATCCGGGAGCTGTCCGTGTCCTGCAGACAGAAGGGGCTGGTGCTGTCCGTTGATAATTACGTCCCTTCCCCGTATTCTGCCTTTTATAACAGACGGGAGCAGGGGATTGTGGCGGATTATGTAATCATCATGGGATATGATGAACATTACGCGGGCGGGGACGCTGGTTCGGTTGCATCCATATCCTATGTAAAAGACGGGATAGAGAACACCCTGAAGGAGGTTCCCAAGGAAAAGGTCATTAACGCCGTGCCTTTCTACACAAGGGTATGGACGGTCAATGAAGGAAAGACCACATCCAAGGCTTACGGCATTGCGGACGCCAGGCAATGGGTTGCGGACAATCATGTGGATCTGACCTGGGACCAGGAGCTTGGACAGTTTTATGGAAGCACGGTTAACGGCAATGGGGAGCAATATATATGGATGGAAGATGAGAAATCCATGGCTCTCAAGATTGGCCTGGTGAAGGATTTTGACCTGGCAGGCGTGGCGTGCTGGAAGCTTGGATTTGAATCATCTGACATATGGGACATTGTGTCAACAGTGAAATAG

>[denovogenes]_116160

ATCATATGGGAGCTGGGCAGGACCATGCAGGATAAGGGGTTTGGCGGCCTGGACATTGATTTTGAATACGTGCTGGCAGATGACAGGGAGGGCTTTGCCGCATTTGTGGACCTGACCACCAGGGTCATGAACCTGTTCGGTTATCCGGTAACCGTGGCCCTGGCCCCCAAGACTTCGGCTGAACAGAGGGGGCTTTTGTATGAGGGGATTGATTACGCCCTTTTGGGCGCGGCCGCCAACCGTGCCATGCTGATGACTTATGAGTGGGGATACAGCCAGGGCCCGCCCATGGCCGTGGCCCCCATCAACATGGTGAGGCGGGTGGTGGACTATGCGGTCACCGCCATTCCAAGGGAGAAACTGAGCCTGGGCATACCTAATTACGGATATGACTGGGCCCTTCCTTATGAGCGGGGCGTGACCAGGGCCAAAACCATTAACAACCACCAGGCAGTGCAGCTTGCCATTGATTTCGGGGTGGATATACGGTTTGATGAGACTGCCATGTCCCCTTACTTCCGGTATTGGCAGTACGGGGTCCAGCATGAGGTGTGGTTTGAGGATGTGAGGAGCATGAAGGCCAAGTTTGACCTTATCAAGGAATATGGACTGTCAGGGGCCGGGTACTGGCAGCTGATGAGCCTTTTCAGGGCCAACTGGCTGATGCTGAACGAGATGTTTTACATTGAGAGGGAGTGGCCGGTGATGGAACGGCTGGACGGGGCAGATGGGGCATAA

>[denovogenes]_90393

AAGGAAAAATTAACAGGTGCAGACAGAAATGAAACATCAGTGAAAATAAGTCAAAAAGGATGGAATAAAGCTGATAATATAGTGTTAATAAATGACTCTAGCATATCGGATGCTTTATCAGCAACACCATTTGCTAAGTCTAAAGATGCACCAATATTACTTACTAAAAATAATAACTTAAATAAATTAACAGAAAAAGAAATAAATAGATTAGAAGCAAAAAATGTGTACATAGTGGGTGGATTAAAGTCTGTTGATGAAAAAGTAGTGTCTGATTTAAAGAAAAAAGGATTAAATGTAATTAGAATATCTGGAAATGATAGATATGAAACATCAATAAAACTTGCAAAAGAATTAGACAAAAATTCTAACCTGTCAAAAGTAGTAGTGGTAAATGGAGAAAAGGGACTAGCTGATGCAGTAAGTATGGGAGCTATATCAGCAAAAGAGGAAATGCCAATTCTTCTTACTAATCAAAATGATGATATGAAAGATATAAAAGACTTAATAGCTAATAAAAACATATCAAAATCATATGTAATAGGTGGAGAATCTTTATTTAATAATAAAGAAGTGAATAACACATTACCATCTGTAACTAAAATAGCAGGTTCTGATAGAACAGAAACTAACTCAAAAGTAATAAGTCATTTCTATAGCAAAGATACACTTAATGACTTATATGTAGCTAAGAATGGAATGAATAAACAAGACGATTTAGTAGATGCCTTATCAGTAGGAGTGCTTGCAGGTAAAACTGAATCTCCAGTAGTATTAGTTGGAAATGGATTAGATGATAGCCAAAAAGAGTTAATCAAGAATAAAAAGTTTAAAAATATAACTCAAATTGGTGGAAATGGAAATGAAAAAGCATTT

>[denovogenes]_84094

AACGTAGCTTTTGGAGAAACATATAGTGATAGAGCTGTTGTTGAATTTACACCTTGCTATGATGAAGAACAGTTTATTTCAGATGTTCAATACATTAAGAGCAAAGGTAAAAAAGTTATTTTATCAATAGGTGGACAAAATGGTGTGGTATTATTACCAGATGCAACAGCTAAAGAAAAATTTGTTAAGTCAATATGTGATTTAGTAGATAAATATGGCTTTGATGGTTTGGATATAGACTTAGAATCCGGTATATCATTACAAGCATCAGATACAGATTTCAAGAATCCTAAAACACCACAAATAGTTAATCTTATTTCAGGTGTAAGAGAAATATCTGATAAATATGGGTCGAACTTTATTATAAGTATGGCTCCAGAAACAGCTTATGTTCAAGGTGGAATTACTGCTTATGGAAACATTTGGGGAGCTTATCTTCCAATAATATATGGATTAAGAGACAAATTAACTTATATTCATGTACAACATTATAATGCTGGTGGTAATCAAGGGTTAGATGGTGTCACATATACACAAGGAACAGCAGATTATGAAGTAGCAATGGCAGAAATGCTTTTATATGGATTCCCTATAGCAGGTAATAGTAATAATATGTTCCCAGCTTTAAGAGAAGACCAAGTTATGATTGGCTTGCCAGCAACTCAGGCTGCTGCACCAAGTGGTGGATATATAAATCCAACAGAAATGAAGAAAGCATTAGATTATTTAATTAAAGGTATATCATATGGTGGAAAGTATAAGTTAGCTAATGGCAAAGGATATCCAGCATTCAGAGGTTTAATGACATGGTCTATTAATTGGGATGCTAAAAATAATTTTGAGTTTTCTAATAATTATAGAGATTATTTTGATAGACTAACTCCAGTTCAAAATACATTAAAGGCTGCAACT

>[denovogenes]_98045

GATAATTTATCAATAGATGAGTCTGTTGATTATTTAATAGAGCAAGGTGCAGAGCCTGAAAAGATAGTAATAGGTGCAGCATTTTATACAAGAGGATGGGATAAAGTAGCTAAAGGTGATGATGCAAAGTTACCAGGGTTACATCAACCAGCACAAAAAACAGGAAAAGATGCAGACCAAACTCCATCATATGGAGCACCAAATGAAGCACCATTAACAAATGGAGACAGTGGACGTGCTGCAGGATGTTGGACATATAGAGGTTTAGATAAGCTAAAAGCTCAATACCCAGGATTAAAAGAATATTGGGATGATACAGCTAAAGCCCCATACCTTTATGATGAGTCAACAGGTAAATTCTTTACATACGATAATGTAAAATCAGTAGAAGAAAAGGCTAAATATGTTAATGAAAATAACTTAGGAGGAATGATTGGGTGGATGTCATCTCAAGATAAGCCAACTAATGGATCAACTAAGAGAGATGAATTAACAAAAACTATGAAAAAAGGACTATTCGGAGATTCTGAGTTGTCAGATCACGAAATAGTTTACTCAGATTTGGATATAGCTGCTACAGTAAATACTTATGAACAAGAGTGGGGAGCAGATAGAAAAGGGTACGAAATAACTATAAAGAACAACGAAAGAGCAGAAGAAAGTGATGCAGTACTTAAAGAAGTTGAAAAAGGTGCTGAAACTATAAAAGCTCCTAAATTATACATAAAGACAGATAAGCCTTTAAACAGAGGAGACCATATGTCAGGAACTGTAACATATGAAAATGGGTACACAGTTGTTGATCTAAAATCTGTATGGGAAGGCAAAAAT

>[denovogenes]_93205

ACTAGTTATCCAGAAAGCAATTCAATATGGGGAAGTAGCTATGCAGGATGGCAATATTCAGATACTGGATATACACCAGGTGTAAATGGAAATACAGATTTAGATACCTTTAATAGTGAGATATTACTAGATAGTAAAGTAACTATATCTGGAAGTAGAAATCAAGAGTCAGAAAATGGGGCTATAAAATATTATGTAGTTCAGGCAGGGAATACATTGAGTGGAATAGCAGCAAAGTTTGGAACTACAGTTCAGGCATTATCTAAATTAAATAATATAACAAATCCTAATTTAATCTATGTAGGACAAGTTTTAAAGATATATGGAGATAACAAAGTTCAAAGAGGAAATAATAATTTCAGTACTACCTATGTTGTACAATCAGGAGATACTTTAAGTGGAATAGCAACAAGATTTGGTACAACTGTACAAGAGTTAACACAATTAAATGATATATCAAATCCTAATTTGATATATGTTGGAGAAGTATTAAAACTTCCAGTTTCTAATTCAGTTAAGAGTGGAGCATCGTCTAAGCAATATCAAACAACTTATGTAGTACAATCAGGAGATACATTAAGTGGAATAGCAGCAAGATTTGGAACTACAGTACAATATTTAGCTAGAATAAATGGAATAGTAAATCCGAACTTAATATATGTAGGACAAATATTAAAAATTAGAGCATCTGGGGTATCAGCACAAAGAGGAAATTCTACAACAACTTATGTAGTCAAATCAGGTGATACATTAAGTGGAATAGCAACAAGGTTTGGAACTACAGTTTCTAATTTGGTTGCATTAAATGATATTTCAAATCCTAATTTAATATATGCAGGACAAGTTTTAAAAGTTTAG

>[denovogenes]_28627

ATGATGATGAAAACTAGAAGGTTGAAAAAGTTTTTATCGACACTTTTAGTAGTTGCGCTAACGTCAGCAGGTATTACAGTAGGTACTAGTGCAACGGAAGCAAAGGATAACGGACAGCTTAACGTTACAAATGAAAAAAATTCAGATGAACTTAAGAGAAAAATAGTAGGATACTTTCCAGAATGGGCTTACTCAAGTGAAGCTCAAGGATACTTTAATGCAACAGATCTACAGTGGGACTCTCTAACTCATATCCAATATTCCTTTGCAATGGTTGATCCATCCACTAATAAAATTACATTAGGAGATAAGCATGCTGCTATTGAAGAGGATTTTGCTGGATATGATCTAAGCTATAAGGGAAAGAAAGTAGAACTAGACTCTTCTTTACCTTATAAAGGTCATTTTAATGTTCTGCAGACTATGAAGAAAAGCTATCCAGATGTAGATTTGTTAATTTCCGTTGGGGGATGGGCAGGATCAAGAGGTTTTTATACTATGCTAGATACTGACAGTGGAATCAATACCTTTGCAGATTCATGTGTGGACTTTATAAGACAATATGGATTTGATGGTGTAGATATTGATTTTGAATATCCTTCAGCCACAAGCCAATCAGGAAATCCAGATGATTTTGATCTTGCAGAACCAAGAAGATCAAATCTTAACGCAAGATATAATGTTTTAATAAAAACTTTAAGAGAAAAGATAGATGAAGCAGCTAAAGCAGATGGAAAAGACTATTTACTAACAGCTGCAGTTACTGCATCTCCTTGGGTGTTAGGTGGAGTAAGTGATAATACTTACGCTAAGTACCTAGATTTCTTAAGTGTAATGTCTTATGACTATCATGGTGGTTGGAATGAATATGTTGAACATTTAGCTGGTATATATCCAAATGCAGAGGATAGAGAAACAACAACTCAAATAATGCCAACTTTATGTATGGATTGGGCATACAGATATTACAGAGGTGTTTTACCTTCTGAGAAAATCTTAATGGGTATTCCTTACTACACAAGAGGATGGGAAAATGTTCAAGGTGGTACTAATGGACTTCATGGAACAAGCAAAACTCCAGCTTCAGGAAAATACAATATATGGGGAGATGATCTTGATGGTGATGGTAATTTAGAACCAGCAGGAGCAAATCCTTTATGGCACGTATTAAACCTTATTGAGCAAGATCCTAACTTAAAAGTATATTGGGATGATACTTCTAAGGTACCATATGTTTGGCAAAACGATGAAAAGGTATTCCTATCTTTTGAAAACGAAAAATCTATAGATGCAAGATTAGAGTATATTCAAAATAAAAACCTTGGTGGAGCATTAATTTGGGTAATGAATGGAGATTATGGATTAAATCCAAATTATGTAGAAGGTTCAAAAGATGTTAATGAAGGAAAGTATACATTTGGTGATACCTTAACAAAGAGATTAAGTGAGGGA

>[denovogenes]_5543

ATGGCAATAAGAAAATTAAAAAAGAACTTAAAAACTTTTGTTGCTTTTAGTGCTATTACAGCTTTATTACTAACTAATGGTACTTCTATTAGTGCACTAACTCAATCTCCTAATAGAAGTGAGGTTTCTTCACAAGCTACTACAGGATTACGAAATGTAATGTATTATGGAGATTGGTCTATTTGGGGAGGACAAGGTAATTTCTATCCAAAGGATATACCAGCAGATAAGTTAACTCATTTAAATTTTGCTTTTATGGACTTTAATTCTTCAGGTGAATTGATATATTGCGATAAGGATGCAGCTACAGGACATCCTTTGGGAAATGCAGGTGTAACCTATGGAGATGTCAATGGTGGTATATTAAATGCATTTCAAGTATTAAGAGCAGAGAATCCTAACTTAAAGATAGGTATATCTTTAGGTGGATGGTCTAAGTCTGGAGATTTTTCAACAATAGCTGCAAATGCTTCAACAAGAGCTAAGTTTGTAGAAAACGTTATGAAATTTATTAAATACACAAATATGGATTTTGTTGATATTGACTGGGAATACCCAGGTGATTATAGGGAACCAGATAAGACTGATAATACAAATGATGAAGGAACACCAAATGCTACTTCAGCAGATAAAGAAAATTATATTTTACTATTGCAGGATTTAAAGAATGCTTTAAATAAACAAGGTAAGGAATTGGGTAAGACTTATGAGCTATCAGTAGCATTACCAGCAGGAGTACCAAAAATAGAAAAGGGTATAGATGTTGATAAACTATTTAATATAGTAGATTTCGCTAATATAATGACATATGACATGGCAGGAGCTTGGAGTACAACAAGTGGACATCAAACAGCATTATATACTAATCCTAATGCTCCAGAGGAATATAAAGGACTTTCTGTAGATGAAAGTGTTAAATATTATATTTCCCAAGGTGCAGAGAGAGAAAAGATAATTGTAGGTGCAGCATATTACACTCGTGGATGGGAACAAGTGTCTGATAAAGGTGTTGATCCAAAGAATCCAGGATTATTTGGAGAGGCTGCTGTAGTTAATAAAAATGCAGATCTTTCACCAACACCAGGTGCTCTTAATGAAGCTCCAATGAAGAATGGTGAAGGCGGAAGAGCAGGAGGTGTATGGGGCTACAATGCTTTGGATAAGCTAAAAGCTAAATATGCTGGCTTAAAAGAATATTGGGATGATAGTGCTAAGGCTCCATATTTATATAATTCATCCACTGGAGCATTCTTTACTTACGATAATGTAAGATCAATTGAAGAAAAAGCTAAATATGTTAAAGAAAATAATCTTGGTGGTATGATTGCTTGGATGGCATCACAGGATGCAACTACAACATCAACTAAGAGGGATGAGTTAACAAATGCTACAAAGGAAGCTTTATTTGGATCAGGTGAATTGCCTAAATATGAAATAAAATATACAAACCCTAACTTAAGTTGTACAGTAACTCCAGTAACTCAATCTTGGGGTACTGGTGGCGTATTAAATATGTCAATAAAGAATAATGAAAAATTGAGTGAATCAGGTGAAGTATTATCAACTGTTGAAACTTCAGCTAAAACAGTTAAAAATATGAAGTTATATATTAAGACTGATGGGGTAGCAATTACAGGTTCGCAATATCCAGCAGGACCAGTTTCAAAAGTAGGTGAGTACTATGTTGTAGACTTTGGAGCTGTGTATGATGGAAAACTAATGCAACCTGGAACAACCTTTACTTTTGATTTAAATCTTGATAAAGCTATTGAAGATACTAGTAAAATTATAAGTGTTGAAGTATCACAAAGAATGTATCAAACATCTCCTGAAATTATGAGAGAAACAATTTGGGGTGATACAAACTCAGCACCAATTATATCTGGAGCTGAAAATATTACTATAAACTTAGGAGATAAGTTTAATGCTTTAAGCAGTATAAAGGCTACTGATAAGGAAGATGGGGATTTAACTAGTAAAGTAGTAGTTACAGGAACTGTAGATACTTCAAAAGCAGGAGAATATACTTTGGTTTATTCTGTTAAAGATAATAACGGAAAAGAAACAACAGTTACAAGAGTTATAACTGTGTTTGATAAAAATGCACCAGTAGTAAATACAAAACCAGAAATTACAGGTGTTAAAGATCAAACTATAAAGCTTGGATCAACTTTTAATCCATTAGATGGAGTAAAAGCAACAGACAAGGAAGATGGAGATTTAACTAGTAAGATTTTAGTAACAGGAAAAGTAGATGCTTCGAAAGAAGGAAAATATAATATAACTTATTCTGTTGTAGATAAAGATGGTGGAAAAGCAGAAGTTTCATGTTGTATAACAGTTGAAGGTCAATTGTATGAAACTTTTGATTCTAAGACAATCTATACTGGGGGAGATATGGTAACTTATAACGGTGAAGTTTATAGATGTAAGTGGTGGACTCAAGGTGAAATACCTGGAGTTTCAGAATGGGGAGCATGGGAAAAAATTAGTTAA

>[denovogenes]_67187

ACAGGTAACTTAGGTACTGGAGGTCAATACTCTTGGTATGATATTAAAAATATTGAAAAAACAGCTGGATGGGTTAAATATAGAGATCCAATAGCTAAAGTACCTTATTTATATAACTCTTCAACCAAGCAATTCTTGACCTATGAGGATGAAACTTCTCTACAAGATAGAATAAATTATATAAATGATAATGGCTACGGCGGTCTAATTGTTTGGGATTGCAGTGGTGATGACGTTAAAAGTGGATGGCCAATGCACACAATAATGTTTAATGGATTAATAAAAGATGGTTCAGAAACTCCTCCTACACAAAATACACTTAAAGCTGCTAGTTTAACAGCTGGACCAGTTTCTAATGGAAAGTATACTTTAACAGCTGTTGTACCAGCACATAATACAGCAACAAGTTATCAAATATTAGAAGGAGCTACTGTTATATCAAGTGGTGCATTGAATTCAGGAAGTGAAGCACCAGTAACTATAACCTATGATATAACAGGTAAAGGAAGTGGAACTTATAGTTATACTGTAGTTTTAAGTGATGGTAGTACTAAAGTTACATCCACAGAAGTAAAAGTTACAGTATTAGCTCCAGTAGAAAATACGTTACAAGCAGCATCTTTAACTGCAGGACCAGTTAGCAATGGATCATTTACCCTAAATGCTCAAATACCAGCAAATAACACAGCAACAAGTTATCAGATATTAGAAGGAAATGCTGTAATATCTACTGGTAGTTTAACTGCAGGTAGTTCATTAAGAAATATAAGCTACAATGTAACAGGAAAAGGGGAAGGAAATTATAATTATTCTGTAGTTTTAAGTGATGGTATTAAAACAGTAACATCTAATATAGTTTCAGTTTCTGTAGAAAAACCATCAACTTATCCGGCATGGGATGCATGGGTAAGCTATAAAACAGGTGATATAGTAAGTTATAATGGAAAAAATTATACATGTAGACAAGGACATACATCATTAGCAGGATGGGAACCATCTAATGTACCAGCATTATGGAGTTTAATGTAG

>[denovogenes]_64813

ATGAAGCTAAAAAAAATAATTCCTGCTTTTCTCCTTCTTTCAACCGTTGCAGTTGGCTTGTGGTTAACGCCTACTCAAGCTTCTGCAGATGCTGCGGATACGATGGTAGATATCTCTGGCAAAAAAGTGTTGGTTGGATATTGGCATAACTGGGCCTCAAAAGGACGCGATGGTTACAAACAAGGAACATCAGCATCACTAAACCTTTCAGAAGTAAATCAAGCCTACAATGTCGTACCGGTTTCCTTCATGAAAAGCGATGGCACGACACGGATTCCTACGTTCAAGCCTTATAACCAAACGGACACTGCCTTCCGACAAGAAGTCGCACAATTAAATAGTCAAGGTCGCGCAGTTTTATTGGCACTTGGTGGAGCAGATGCACATATTCAATTAGTCAAAGGCGATGAACAAGCCTTTGCGAATGAAATCATTCGTCAAGTGGAAACATACGGCTTTGATGGTTTAGACATCGACTTAGAGCAATTGGCGATTACTGCTGGCGACAACCAAACCGTCATCCCTGCTACGTTGAAAATAGTCAAAGACCATTATCGAGCACAAGGAAAAAATTTCATCATTACGATGGCACCAGAATTCCCTTATTTAAAACCTGGTGCCGCTTATGAAACATACATTACTTCCCTAAATGGTTATTATGATTACATTGCCCCACAATTATATAACCAAGGCGGCGACGGTGTCTGGGTTGATGAAATTATGACTTGGGTTGCTCAAAGCAACGATGCTCTAAAATACGAGTTCCTCTATTATATGTCTGATTCATTAATCCATGGCACGCGCGGCTATCTACAGATACCAAACGATAAATTAGTCCTTGGTTTACCAGCAAATCGGGATGCTGCTGGTAGCGGCTATGTCGTTGAGGCTACCCCTGTAGCCAAAACTTTTGATCAGTTAGCAAAAGACGGTAATCCTATCCGTGGCTTAATGACTTGGTCTGCAAATTGGGACGTTGGTCAAGATGTCAACGGCAAGTCCTACAACAATGAATTTGCGACACGTTATAGTAACCTTGTAAAATAA

>[denovogenes]_78835

ATGGGGCATCAATTGAGCACTCATTTTTTTAACAAGAAAACAGGGATTATTTCAACTTTTTTAAGTTTGATACTTTTAGTCGGTGGTTTACTGTTTGCACTACCTGCATTTGAAGTACAAGCAGCATCAACTGTTACACCTAAAACAGTCATGTATGTAGAAGTAAACAACCACGATTTTAACAATGTGGGAAAATATACTTTAGCTGGAACCAATCAACCTGCTTTTGATATGGGGATTATTTTTGCAGCCAATATTAATTATGACACGGTCAATAAAAAACCTTACTTATATCTAAATGAACGGGTCCAACAAACTTTAAACGAAGCTGAAACACAAATTCGTCCAGTTCAAGCACGCGGCACTAAAGTCCTATTGTCTATTTTAGGCAACCATGAAGGTGCTGGCTTTGCTAATTTCCCAACTTACGAAAGTGCGGATGCCTTTGCAGCTCAATTAGAACAAGTCGTTAATACGTATCATTTAGATGGTATTGATTTTGATGATGAGTATGCTGAATACGGAAAAAATGGGACGCCTCAACCAAATAATTCTTCGTTCATTTGGTTACTTCAAGCCTTAAGAAATCGCTTAGGCAATGATAAATTAATTACTTTTTATAATATTGGACCAGCGGCTGCGAACTCTTCAGCTAATCCTCAAATGAGTTCTTTAATTGATTATGCTTGGAACCCTTATTATAGTACCTGGAATCCGCCACAAATTGCGGGGATGCCTGCTTCTCGTTTAGGTGCTTCTGCCGTTGAAGTTGGTGTCAATCAAAATCTAGCTGCGCAATATGCTAAACGTACAAAAGCGGAGCAATATGGTATTTATCTAATGTATAACCTCCCAGGAAAAGATTCATCTGCGTATATCTCGGCGGCGACACAAGAACTTTATGGTCGAAAAACGAACTATTCACCAACTGTTCCAACACCTTAA

>[denovogenes]_34460

ATGCCACGTATTCCTCAAATAGGTTATAAATTTCCTCAACTTCGTGGAATGAGTAGTAATAATAAGTGGACAGTTGTTTGTAATGCCCCTACATCCTATCTTAAACATACTAGTTTATTAAGGGGTCCGGATGAAATCCTTCATCAAAATGATATTCAAATCGTACAGCTCCCTAGTCGAACCACAGATACTACTCTTTATATTATAGATCCTAGTGAAATTCTCAGAGCTATCATTCATGATGATATCTCTTCTCCATTAACTGCTTCCCACGTTGTGACTATTGTAGATTCTCTTAAAAAAGGTTATAGGCCTGAAATTCAAAGTGTTTCCAACCAACCTACTAACCCAGCCTGTCCTAATATCCAAAAAGTAGTAGGTGAATATGTTCTTGGCGACCCTCAAAATGTAGACCCATTTCTTTTAGACTTTGTTATTTATGCTTTTGCACTTATTCAGCCTGATGGATCGCTAGATGTATATTCTAGAAGATATCTTCAGGAACTTGCTAACCTTAGATTTTCAGACCCTAATCTTAAAGTTATATTAGGTATTGGTGGCTGGGGTAATGATGGTTTCTCTGACGCCGCTCTTACACCACAGTCTCGCTACAATTTTGCTAGAGAAGTTAGAAGCTGGGTAAATGAATATGACTTAGATGGGGTAGATATTGACTGGGAGTATCCTGGTAGTAGTGCCGCTGGTATTACTTCAAGACCTCAAGATAGGGAAAACTTTACACTCTTACTCCAAGCATTACGTGATGTATTAGGTCCTGATGCATGGATTAGTGTAGCCGGTACTGGGGATGCATCTTATATTCGTAATGTAGATATTGCAGGTATTGCCCCTATTATTAACTATTTCAATATTATGGCTTATGACTTTACAGCTGGTGTAACAGGCGCTCAAGGTGCAAGACATCATTCTAATCTTTTTCCATCAGACCTTGCCTTTAACAATATAAGTGCTGATTTATATGTTCAAAATCTTATTAATGCTGGTATGCCTCCAGAACAGCTTCTACTAGGTCTTCCTCTTTACGGCAGAAATGGTGCCACAATTACAAGGACCTTCGATGAAATCCGTAGGTCTTACCTTAACACAAATGGTTATACTGTAAGATGGGATAATGTTGCTAAAGCTCCTTATATCGTTGACCAATTCGGAAACTTCTTCCTTGCCTTTGATAACGGCCTTTCTATTTACTTCAAAGGCCAATATGTTGCTGATAATTGTTTAGGTGGCCTTTTTACATGGCAAACCAATATGGACCAGGCAAATATATTAGCTAATGATATGTATCTTGCTATTAATGACCCAAGAACATTAGAAGAAATCCTTGCTCAAGAATATTTAGGTTAA

>[denovogenes]_4353

ATGATTGGTATGGGGCTGGTGTGTTCCGCTCTGCCAGCATTGGCAATGGAAGCATGGAATAACCAACAAGGTGGTAATAAATATCAGGTTATTTTCGATGGCAAAATTTATGAAAATGCCTGGTGGGTTTCTTCTACAAATTGCCCGGGAAAAGCGAAAGCAAATGATGCAACTAACCCGTGGCGTTTAAAGCGTACCGCAACAGCTGCTGAAATTAGTCAGTTTGGCAATACACTTTCCTGCGAAAAGAGCGGCAGCTCATCTTCTTCAAATTCAAATACGCCTGCATCCAATACGCCGGCTAATGGCGGTTCGGCTACACCAGCACAGGGCACTGTTCCGTCTAATTCTTCTGTAGTTGCCTGGAATAAACAGCAGGGCGGTCAGACCTGGTATGTCGTCTTTAATGGTGCGGTATATAAAAATGCCTGGTGGGTAGCCTCTTCTAACTGTCCGGGTGATGCGAAAAGCAATGATGCCAGCAACCCATGGCGTTATGTTCGTGCCGCTACGGCAACGGAAATCTCAGAAACCAGTAATCCACAGTCCTGTACTTCAGCACCACAGCCTTCACCGGATGTGAAACCGGCACCGGACGTTAAACCGGCTCCTGATGTTCAGCCAGCCCCAGCTGATAAGTCAAACGACAACTATGCTGTAGTAGCCTGGAAAGGTCAGGAAGGTTCTTCTACATGGTACGTTATCTATAACGGCGGCATTTATAAGAACGCCTGGTGGGTAGGCGCGGCAAATTGCCCAGGCGATGCGAAAGAAAACGATGCCAGTAACCCATGGCGTTATGTTCGCGCGGCAACGGCAACAGAAATCAGCCAGTATGGTAACCCTGGCTCCTGTTCCGTTAAGCCGGATAATAATGGCGGTGCTGTGACTCCGGTTGATCCAACTCCGGAAACACCGGTGACCCCAACCCCGGATAACAACGAGCCATCAACACCAGCGGATAGCGGTAACGATTACTCATTGCAAACGTGGAGCGGCCAGGAAGGTAGCGAAATTTACCATGTTATTTTCAATGGTAATGTTTACAAGAACGCCTGGTGGGTTGGGTCTAAAGATTGCCCACGGGGTACCAGCGCTGAAAACTCCAATAACCCATGGCGTCTCGTGCGTACAGCTACCGCTGCGGAATTGAGTCAGTACGGTAACCCGACTACCTGTGAAATTGATAACGGCGGCGTCATTGTTGCGGATGGTTTCCAGGCCAGCAAAGCGTACAGCGCGGACAGCATCGTAGATTATAACGATGCACATTATAAAACTTCTGTCGATCAAGACGCATGGGGCTTTGTCCCGGGCGGCGATAACCCGTGGAAGAAATACGAACCGGCGAAAGCATGGTCCGCATCCACTGTGTACGTGAAAGGTGATCGCGTTGTTGTTGATGGGCAGGCTTATGAAGCGCTGTTCTGGACGCAAAGTGACAACCCTGCTCTGGTTGCGAACCAAAACGCCACCGGTAGCAATAGCCGCCCGTGGAAGCCGTTAGGTAAGGCTCAGAGCTATAGCAACGAAGAGCTGAATAATGCGCCGCAGTTTAATCCAGAAACGCTTTATGCCAGCGATACGCTGATTCGCTTTAACGGTGTGAACTACATTTCTCAGAGTAAAGTGCAGAAAGTTTCTCCTTCTGACAGCAACCCGTGGCGTGTTTTTGTTGACTGGACCGGAACCAAAGAACGTGTAGGTACGCCGAAGAAAGCATGGCCGAAACACGTTTATGCACCGTATGTTGACTTTACGCTGAATACGATCCCGGATTTGGCTGCGCTGGCTAAGAATCATAACGTCAACCACTTCACGCTGGCGTTTGTGGTGAGTAAAGATGCGAACACCTGTCTGCCGACATGGGGTACCGCTTATGGTATGCAGAATTACGCTCAGTACAGCAAAATCAAAGCTCTGCGTGAGGCTGGCGGCGATGTGATGCTGTCTATCGGTGGTGCTAACAACGCTCCGCTGGCTGCTTCCTGTAAGAACGTAGACGATCTGATGCAGCATTATTATGACATCGTTGATAACCTGAACCTCAAAGTCCTGGACTTCGATATCGAAGGCACCTGGGTTGCGGATCAGGCATCTATTGAACGTCGTAACCTTGCTGTGAAGAAAGTGCAGGATAAATGGAAGTCAGAAGGCAAAGATATTGCTATCTGGTACACCTTGCCAATTCTGCCGACTGGCCTGACGCCGGAAGGGATGAATGTCCTGAGCGATGCCAAAGCGAAAGGTGTTGAGCTGGCGGGTGTGAACGTGATGACAATGGACTACGGTAACGCGATTTGTCAGTCTGCAAATACCGAAGGCCAGAACATTCACGGTAAGTGTGCAACGTCTGCGATTGCCAACCTGCATTCACAATTGAAAGGCCTCCATCCCAATAAGAGCGATGCAGAAATTGACGCTATGATGGGTACCACGCCGATGGTTGGCGTGAACGACGTTCAGGGCGAGGTGTTCTATCTCTCTGATGCTCGTCTGGTCATGCAGGATGCGCAGAAGCGTAATCTCGGTATGGTTGGTATCTGGTCAATCGCGCGCGACCTGCCGGGCGGCACTAACCTGTCTCCGGAATTCCACGGCCTGACTAAAGAACAGGCACCGAAGTACGCATTTAGCGAAATCTTCGCGCCGTTTACTAAGCAATAA

>[denovogenes]_19914

ATGAAACACCGTTTCTGTGCGGCAGCCCTGGCCGCCGTTTTGCTTTTGAGCGCCGCACCCCTCTCCCCCGCAGCCAGCGCCGCATTTTCCGACGCGGAGGGCACCTGGGCCGCAGAGGTCATCGAAAAGGCGGAGGGCTATGGCCTGATGAACGGCTACCCCGACGGCACCTTCGGCGTGGGGAAGGAGCTCACACGGGGCGAGTTTGTGGCCGTGCTCTGCCGCATGTTCGGCTGGGATGCCGCCTCCCCGGGCGCGCCCTCGTTTTCCGACTGCCCCGCCTCTCACTGGGCCTACTCCTATGTGGAGACCGCCCTGGCCCACGGCGTGATGGATGCCGGCGGCGCCTTCCGTCCGGAGGACTATATCAGCCGGGAGGAGATGGCGGTCATGCTGGTGCGGGCCCTGGGCTACGGCACGCTGGCCCAGTCCCTGTCCGGGCTGGAGCTGCCCTTTGACGACATCACGGACAACCGGGGGTACATCGCCATCGCCTACGACATCGGCATGATCACCGGCGTGGCCGGGGCGGGCGGCCAGCTCAAGTTCCTGCCCCGCGACTCCGCCACCCGGGAGGAGGCCGCCGCCATGCTGGTGCGGGTGTACGAGCGGTACACCTCCAAGCTGGACTGGCTCCACGGCTTTTACGCCTTCTCCTCCTATTCCCAGATTGATCTCACCGCCTCCATGGACGCGGTGAGCGTAGGCTGGGCCCGGATGGAGTACGACCCCGCCGCCGGGCCCGTCCTCAACTCCTCCCGCACCAACGGCAACGACTGGGTGCGCCCCGACGACCCCACCCCCGCCACCGATTACTGGGATGCCCGCAGCCTCCCCTACAACTTAAATGTATATGCCTCAGCGGGCGATTCCATCCCCCTCCCCGACGGCACCACCACCTCCACGGTGGCGGCGGTGACCGGAACGCCCGAGGTCCGGGCCCAGGCCGTGGCCGCCCTGGCGGCCGCCAGCGCGGACTACGCGGGCCTGACCATCGACTTTGAGGGCCTGAAGGGGGATACCATAAAACTGAATTATGTAACCTTTCTAAAAGAGCTGGATGCCGTCCTGCCCCAGGGGAAAACCCTCTATGTCTGCGTCCAGCCCGACACCTGGTACACCGGCTTTGACTACCGGGGCATTGGCGAGGCCGCCGACAAGGTGATCCTCATGGCCCACGACTACCAGTGGACCTCCGTCCCCGACTCCTATGTAGGCACTACCAACACCGACTCCCCCGTCACCCCCTTCGCCAGCGTGTACGAGGCTCTGCGTGATCTTACCGACCCGGCCACCGGCGTGGCCGACCGGAGCAAGCTGGCCCTGCAGATCTCCTTTGGCTCGGCGGGCTTCCACGTGGACGGAGAGGACCGCCTGCTGGAGACCACTATCTACCACCCCGCTCCCTCCACCCTGGCCCTGCGCCTGGCCCAGCCCGACACTCAGGTGGTCTACTCGGAGGAATTCCGCAATCCCTGCGCCCTCTACACCACCGAGGACGGCAGCCGTTACAAGGTCTGGTACGAGAACGAGCAAAGCGTGCTGGACAAGGTGGCGCTGGCCCGGATGTTCGGCATCACAGGAGTCTCCCTGTGGCGCATCGGCAACATTCCGGCGGACGGTACCTACGACGTGTGGAGCGCCCTGCTGACCCAGCGCTGA

>[denovogenes]_20262

ATGCGACGCAGGCTTCTCACACTGGCGCTCTCCGCCGGATTGATGCTCTCTCTGTCCGCCCCTGCCCTGGCTTACAGCGATACCGCCGGCCACTGGGCGGAGTCGGCCATCCAGCGGGCCGCCGATTACGGCCTGATGGTGGGCTATGAGGACGGCCGCTTCGGCGTGGCCGACAACCTCAACCGCGCTTCCTTTGTGACCATTCTCTGCCAGATGTTCTCCTGGGAGGCCGTCCGGCCCGGCCAGGCCAGCTTCATCGACTGCCCCTCCTCCCACTGGGCCTACGGCTATGTGGAGGCGGCCCGGGCCCACGGCGTCGTAGACGCGGGCGGGAGCTTCCGCCCCGACGACTACATCAGCCGGGAGGAGATGGCCGTCATGCTGGTCAAGGCCCTGGGCTACGACACCCTGGCAAAGAGCGCCGCGGCCGACCTGCCCTTCTCCGACGTGACGGAGAATCAGGGCTACATCTCCCTGGCCTACCGCATCGGCATGGTCTCCGGCATCGAGGAGGACGGCAGGCTCCTCTTCAAGCCCGCCAGCTCGGCCACACGGGCCGAGGCCGCCACCATGCTGGTACAGGTCTATGAGCGCTATACCTCCTCCACCGAGTGGCTCCACGGGTTCTATGCCCTCTCCTCGTACAGCCAGATCGACCTGACCCGGCAGATGGACGCCGTCTCGGTGGGCTGGGCCCGGATGGAGTACGACCCCCAGCGGGGGGCGGTCCTCAACACCACCGGTGCGGGGGACAACGACTGGAAGATCCCCGCCCAGAGCGAGGCGGCCACCGGCTATTTTGAGCGCAACGGCATCCCCTATAACCTGTGCGTCTTTGCCAGCGCTTCGGACTCCGTGACCCTTTCGGACGGCGCCGCCGCCTCCACCGTGGCGGCCGTGACCGCCTCCCCCGCCAGCCGGGCCCAGGCCGTGGCCGCGCTGGCCGCCGCCGCGTCCGACTACGCGGGCATCACCATCGACTTTGAGGGGCTCAAGGACAAGAACGGCCTGAAGGAGCACTTTGCCGCCTTCATGGCCGAGCTGCGCGCCGCCCTTCCGGCGGACAAGCGCCTGTATGTGTGCGTCCAGCCGGGGCCCTACAACGACGGCTTCGATTTCAAATCCCTGGGGGAGAGCTGCGACAAGGTCATCCTGATGGCCCACGACTACCGTCCCCCGGTCTCCGATCTGAAGGTGGGCTCCATCCCCTCGGAGAGCTTCGCCCTCACCCCCTTCTCCAAGATCTATGCCGCCCTGGTGGAGATCACCGATCCCCTCACCGGCGTGCAGGACAAGAGCAAGCTGGCCCTGGCCTTCTCCATGGATACCACCGGTTTCCGGATCGATGAGGCCGGACGTGTGCTGGAGAGCAAGTATTACCGCCCCGCCATAGAGACCCTCTCCCAGCGCCTGGCCCAGAGCGGCACTGCCGTTACTTACAGCCAGAGCGCCCGCAACCCTTACGCCGTCTACACCGACGAGAGCGGGGCGCGCTATCAGGTCTGGTTTGAGAACGCCCAGAGCATCGCCGACAAAATCGATCTGGCCCAGATGTTCGGCATTACCGGCTTCTCCTTCTGGCGCCTGGGGAATATCCCCACCTACGACAGCTACAACCTGTGGACCGCCGTCCAGGCCGAGCGTCCATAG

>[denovogenes]_115156

GTTGTTGAAAGAATAAAAAAACAAGAAGGTAAGACAAAGTATGAAATAGGTAGAGAAGAATTTTTAAAGAGAGCCTGGGAATGGAAAGATGAATTTGGAGGAAAAATATCTAATCAGCTTAAACAACTAGGTGATTCTTGTGATTGGGATAAAGAAAGATTTACTATGGATGAAGGGTGTAATGAAGCAGTTATAGAATTTTTTGTAAGCCTATATGAAAAAGGTCACATATACAGAGGAAATAGAATAATAAACTGGTGCCCAGATTGCAAAACTACTTTATCAGATGCAGAAGTTGAACATGAAGAACACGACGGAAACTTTTATCATATAAAGTACCCACTAAAAGATAGTGAAGACTTTTTAGAAATAGCTACAACTAGACCTGAGACAATGATAGGAGATACTGGTATAGCAGTAAATCCAGAAGATGATAGATATAAACATTTAATAGGAAAAACAGCAATACTTCCATTAGTAGGAAGAGAACTTCCAATAGTTGCAGATAGTTATGTTGATTTAGAGTTTGGAACTGGTGCAGTTAAGATGACTCCAGCACATGACCCTAATGATTTTGAAGTAGGATTAAGACATAATCTTGAGCAGTTAAATACTATGAATGAAGATGGAACTATGAATGAAGTTTGTGGAAAATATGAAGGTATGGATAGATTTGAGTGTAGAAAAGCTATCGTAGCAGACTTAAAAGAACAAGGTTACCTAATTAAGATAAAAGAGCATAAT

>[denovogenes]_143438

TTTACAATAATGCTTCCACCGCCAAATATAACAGGTCAATTACATATGGGACATGCACTTGACCATACGTTACAAGATATACTTGTAAGATGGAAGAGAATGGATGGATATGAAACATTATGGCAACCAGGAACTGACCATGCTTCTATAGCAACAGAAGTTAAAGTTGTAGAAAGAATAAAAGAACAAGAAGGAAAAACAAAGTACGAATTAGGAAGAGAAGAATTCTTAAAGAGAGCTATGGACTGGAGAAATGAATTTGGTAGAAAAATAGTAGACCAAATGAAGCAGTTAGGAGATTCTTGTGACTGGGATAGAGAAAGATTTACAATGGATGAAGGATGTAACGAAGCTGTTACTGAGTTCTTTGTGAAACTTTATGAAAAAGGTCAAATATACAGAGGAAACAGAATAATAAACTGGTGTCCAGATTGTAAAACAACTTTATCAGATGCAGAAGTTGAACATGAAGAGCATGATGGAAAATTCTATCACATAAAATACCCAATAGCAGGTAGTGATGAATTCTTAGAAATAGCTACAACTAGACCAGAAACAATGTTAGGAGATACTGGTATAGCTGTAAACCCAGAAGATGAAAGATACAAGCACTTA

>[denovogenes]_121842

CCGGATGGTTATATCGGGTACGTGGAGCATAAGCGTCTGGGCGAGAGCCGGAGCGAGGCGCCGGTGAGCACATTTGTGGCTCCGGTCTATAAGAATATTTCTCTCGATGAGCCGGTATGCATGGCATGGCACCAGGTTACGAAACCGGAGGGGAACGCCTCCTTTGATAATCTGATCGCCAATACAAAGGGACTAAATGTTATTTCTCCAACCTGGTATGAGCTGACCGACAATGAAGGCGGATTCAATTCCTATGCGGATGCTGCGTATGTACAGAAAGCCCATGACATGGGGCTCCAGGTGTGGGCCCTGATCAATAATTTCAGCGGCAACGTGCAGACGGAGGTTCTGCTTTCGAAGACCTCCACGCGGCAGAAGCTGATTGAGAAGCTGATGGCCGAGGTGGAGCAGTATGGTTTGGACGGACTGAATCTGGATTTCGAGGGAATTAAGAAGGAAGCCGGAGTTCACTATATCCAGTTTATCAGAGAACTCTCTGTATCCTGCAGGAAAGAGGGGATCGTGCTGTCCGTGGATAATTACGTTCCCTATGCCGGCAATGAGTTCTATAACCGGAAGGAACAGGGAATTGTGGCCGATTACGTCATCGTTATGGGGTACGACGAGCATTACGCCGGCGGCGAGCCGGGATCGGTAGCTTCGGTCGGCTATGTGAATGATGGCATTGCCAACACGTTAAAACAGGTTCCG

>[denovogenes]_77129

TATACGAGTATATCTAAGGATTATAAGATTAATTTGACATTCCATCAAGTTACGAACCAAACTGCTAATAATCATGTACTAGAGTTACTTGGTAATACAAAAGGGGTAACGACTATATCACCTACTTGGTTTAAGGTATCAAGTGTTGAAGGGGATGTAGAATCACTGGCAAGTGAAACGTATGTGCAACGTGCTCATAACTACGGTGTTGAAGTTTGGGCATTGGTGGATGACTTCACCAATGGTATCAATATGGAAGAATTGTTATCGTATACAAGTAGAAGAGATAAATTATCCAATGAATTAATCTCATTAGCAATCAAATATAATTTAGATGGTCTAAATATGGATTTTGAAAAGATCCCTCAAGCTGCAGGTAATCATTATATTCAGTTCTTACGTGAAATGTCTGTAAAATGCCGTAATAATGGTATAATTTTATCTGTAGATAATTATGTTCCAAGTGCGTATACTTCTTATTATGATAGAGCAGAACAGGGTGTACTTGTGGATTATGTAGTAACTATGGCATATGATGAACATATCAATGGTTCAGACGGAAGTGGTTCAGTAGCATCCATAGATTTTGTGAAAGATGCAGTTACGAATGTACTAAAAGAAGTTCCGGCTGAGAAAAACATAATTGCAATCCCGTTCTATACTAGATTATGGGAAGAGTCAGCAGAAGGTGTAACAGCAAAGGCTTATTCTATGGAACAAGCACAAGCGATTCTTGACCAGAATAATGCAGAAGCTACTTGGGATAACGAAACAATGCAGTATTATGCAGAGTATGAAAGTGGTGGAAACACATATAAGATCTGGTTAGAAGAGGAGAAATCCTTAGAAGAAAAGCTTAAGGTTATTATGGATAACAACGTTGCAGGTATTGCAAGTTGGAAGCTTGGACTAGAAAAAAGCAGTGTCTGGAGCGTTATTTTAAAATATGTTAATTAA

>[denovogenes]_41394

ATGCTCATCCACGTAGTCAAACCCGGCGATACTGTCTACTCCATTGCCCTGGAGTACGGCATTCCCATGTCCCAGCTCATCTTGGACAATGGCCTGGAAACCTCCTCCCGTCTGGCTGTAGGGCAGGCGCTGGTGGTTCAATTTCCAACCCAGACCCACACGGTCCAGCCCGGAGAAACCCTGGCCTCCATCGCCGCGGCCTATCAGCTCCCACTGCGCCAGCTCTACCGCAACAATCCCATACTCGGCGGTATCCCGGAGATCTATCCTGGCCAGACCCTGGTGCTCGCCTACGACAGCACGCCGGAGGCCACCCTCTCCGTCAACGGCTACGCCTATCCCTTTATCGACCCGGCGCTGCTCCAATCCACCGTCCCTTATCTCACCTATCTGACCCCCTTTACCTATGGCTTTACCCCTGACGGCACGCTGGTGGAGTTGGATGACGAGGCCCTGCTCGCCGCAGCACGCCAAGGTGGAGCCGCTCCTCTGATGCACCTGTCCACCCTGACCGAAGAGGGCGGCTTCTCCAATGAGCTGGCCCACTTGGCCCTCACTCAGCCTGCGGTACAGGATACGCTGGTTGACAATCTGGAGGCCATGCTGGTGCAAAAGGGCTACCGGGGGCTGGATGTGGATTTTGAATATGTCTACGCCGAGGATGCGGGGGCCTATGCCACATTTCTGGGCCGCCTCACAGAGCGGCTCAATCCCCTGGGCTATCCGGTTATCGCCGCCTTGGCCCCCAAAATTGCCGCCGATCAGCCCGGAACACTTTATGAGGGCCACGACTTCGCCGCCATTGGTGCGGCGGTCAATCAGGTACTCCTTATGACCTATGAGTGGGGCTATACCTATGGTCCGCCGATGGCGGTGGCTCCCCTGCCCAATGTGCGCCAAGTGGTGGAGTATGCCCTGACAGAAATTCCATCTTCCAAAATCTGGCTTGGCGTCCCACTCTACGGCTACGACTGGCCGCTCCCCTTTGTGCAGGGACGTACCAAGGCTCAGTCCATTTCCCCCCAGGAGGCGGTGGCCAGGGCGGTCCGCTACGGTGTGGATATCCAATACAGCGAGCAGGCCCAGGCCCCCTGGTACCGCTATACTGATGGCTACGGCATAACCCATGAGGTCTGGTTTGAGGATGCCCGTAGCATACAGGCCAAGCTGGCCTTGATCCCCGAATATGGTCTATACGGGGTTGGCTATTGGAACCTGATGCGGCCCTTCCCCCAGAACTGGCGGGTGCTCAACGCCCTGTACCGCATTCGTACCGTGTAG

>[denovogenes]_119305

ATGTATGATCAGATGCTTAAGCATCGCAACAATGCTGCCTGCGAAGGAAAGGGAGATTTCTATAGTTATGATGCATTCATCACTGCTGCAAGCTTTTTTCCTAACTTCGGCACCACTGGTGATATCACTGCCCGTAAAAGGGAAATTGCTGCTTTCTTTGGCCAAACCTCCCATGAAACTACTGGAGGGTGGGCGGACGCACCAGATGGACCATTTGCTTGGGGTTACTGTTTCATCAGAGAAATAGGTAGGCCGAGTGGTTACTGCGTACCAAATAATCAATGGCCTTGTGCTCCTGGACAGAGTTATTACGGAAGGGGACCCATTCAAATCTCATACAACTACAACTATGGACCATGTGGAGCAGCTATCGGAGAGGACCTTTTAAACAATCCTGATTTAGTAGCCACAGACTCACTCATCTCATTCAAATCGGCTCTTTGGTTCTGGATGACCCCTCAACCACCAAAACCTTCTTGCCATGATGTAATTCTCGGAAGATGGAAACCATCAAACTCTGATCTGGCAGCCAATCGCCTCCCAGGATACGGCGTCTGCACAAATATCATCAATGGTGGCCTGGAATGTGGTCGTGGTAATGACAGCAGGGTCCAGAATCGAATTGGGTTTTACAGAAGATATTGCCAAATTCTTGGAGTTAGTCCTGGTGAAAATCTTGATTGTGGAAACCAAAGGCCATTTGGATATGGACTCTTAGTCGAT

>[denovogenes]_28742

ATGATCAGGATCGACTTTTCCCAGTTGCACCAGGCCCGCGAAGATGCCGCGGCGGCCATGCCGAGCATCGCCGGCAAGAAGATTCTCATGGGCTTCTGGCACAACTGGCCGGCCGGCGCCGCCGACGGCTACCAGCAGGGCTCGTTCGCCAACATCGCGCTGGAAGACGTGCCGAGCGAGTACAACGTGGTCGCCGTGGCCTTCATGAAAGGGCGCGGCATCCCGACCTTCCAGCCATACAACCTGTCCGACGCGGAGTTTCGCCGCCAGGTCGGCGTGCTCAACGCCCAGGGCCGCGCGGTGCTGATTTCGCTGGGGGGCGCCGACGCGCACATCGAGTTGCACGCCGGGCAGGAGCAGGCGCTGGCCGCCGAGATCGTCCGTCTGGTGGAAACCTACGGTTTCGACGGCCTGGACATCGACCTCGAGCAGAGCGCCATCGACCTGGCCGACAACCAGCGGGTGCTGCCGGCGGCCCTCAAGCTGGTGCGCGAGCACTACGCCGGGCAGGGCAAGCACTTCATCGTCAGCATGGCCCCGGAGTTTCCCTATCTGCACAAGAACGGCAAGTACGTGCCTTATCTGCAGGCCCTGGAAGGCGTCTACGACTTCATCGCGCCGCAGTACTACAACCAGGGCGGCGACGGCCTGTGGGTCCAGGAGGCGAACGGCGGCAAGGGCGCCTGGATCGCGCAGAACAACGACGCGATGAAAGAAGACTTCCTCTACTACCTCACCGAGAGCCTGGCCACCGGCAGCCGCGACTTCGTGCGGATCCCGGCGCAGCGCCTGGCCATCGGCCTGCCGAGCAACGTCGATGCGGCGGCTACCGGCTACGTGATCGATCCCGCCGCGGTGAGCAACGCGTTTCGCCGCCTGGAAGCCGCCGGTCACGCGATCAAGGGCCTGATGACCTGGTCGGTGAACTGGGACGATGGCCTGAACAAGCGCGGCGAGCGCTACAACTGGGAGTTCCGCAAGCGCTACGCCAGCCTCATCCATGACGGCGAGGGCGGCGACCAGCGCCCGGCGGCGCCGCAGGGCCTGCGTCTGCTGGAGCGCGGCGAGACCAGCCTGGTGCTGGCCTGGAACGCCTCCAGCGGGCAGCGTCCGATCGATTACTACAGCCTCTATCGCGACGGCGCCATGGTTGGCCAGAGCGCCGCGCTGGGTTCCACCGACAGCGGCCTGACGGCGGACACCCGCTACAGCTATTTCGTCACCGCCACCGATACCCAGGGCAACCAGTCGCTGCCCAGCGAAGGCCTGGAGGTCAGCACCAGCGGCGGGGCGGTGGATCCGCAATTCCCGCAATGGCGGGAGAACCAGGCCTACCGGGTCGACGATGGGGTGACCTACGAGGGGCTGCGCTATCTCTGCCTGCAGGCGCACACCTCCAACAGCGGCTGGACGCCGCCGGTAGCCTTCACCCTCTGGCGGCCGCTGCGCTGA

>[denovogenes]_139898

ATGAAACTGACCGAGCAGCAATTGCTGCGCATTTTTCCCAACGCCCGCCTCGTCGCGGGCGTTTTCGTTGCGGCGTTGCAACGGGCCATGGACGAGAGGGAGATCGACACACCGGCGCGGCGTGCCGCGTTTCTCGCCCAGGTCGGCCACGAAAGCAGCCAGTTGACCCGGCTGGTGGAGAACCTCAACTACAGTGCCCAAGGCTTGGCGGCGACCTGGCCGGGCCGCTATCTCGGCCCCGACGGGCAGCCCAACGCGTTGGCCTTGCGGCTGGCGCGCAATCCGCAGGCGATTGCCGACAACACCTACGCCACGCGCAACGGCAATGGCGACGAAGCGTCCGGCGATGGCTGGCGCTTTCGCGGGCGTGGCTTGCTACAAATCACCGGGCGTGCCAACTACCGGTTGGTCGGCGAGGCCCTCGGCGAGCCGCTGGAAGCCGAGCCCTGGCGCCTGGAGCAGCCCGTGCCGGCGGCCCGCAGCGCCGCCTGGTGGTGGGCCGGTCACGGGCTCAACGAGCTGGCCGACCGCGGCGAGTTCGCTGCCATCACCCGCCGCATCAACGGCGGCCTGAATGGCCAGGCGGAGCGCCTGGCGTTGTGGCAGCGGGCCAGGGCGGTGCTGTCATGA

>[denovogenes]_139972

ATGCCTATCACTGAGCAGCAACTGCTGCAAATCCTCCCGAACGCCGGCCCTCGCGCCGGCTTTTTTGTGCCCGCACTGAATGCTGCCATGGGGCGCTTCGGCATTACCTCGCCGGTGCGGGTCGCCGCGTTCCTCGCCCAAGTCGGGCACGAGAGCGCCCACCTGACCCAGTTGGTTGAGAACCTCAACTATAGCGCCCGCGGCCTGGCGGCGACCTGGCCGAGCCGGTACCGCGGCGCCGACGGCCAGCCCAACGCCCTGGCGCAGCGCCTGGCGCGGAACCCCCGGGCTATCGCCAACAACGCCTATGCCTCGCGCAACGGGAACGGCGATGAGGCCTCCGGCGACGGCTGGCGCTTCCGCGGGCGCGGCCTGCTGCAGATCACCGGCCGGTCGAACTACCGCGCCGCCGGCGCCGGGCTGGGCCAGCCGCTGGAGCAGGAACCCGAACTGCTCGAGCAGCCGGAGTTCGCAGCGCTGTCTGCCGCCTGGTGGTGGTCGACGCACGGTCTGAACGAACTGGCCGACCGCGGTGAGTTCGCCGCCATCACCCGCCGGATCAACGGTGGGCTCAACGGCCAGGCGGAGCGCCTGCAACTGTGGGAGCGGGCGAAGAGGGTGCTGTCGTGA

>[denovogenes]_142955

ATGAAGATCACTGCCGATCAACTCGACCGCGCTACTGGCTGCGGTGCCGCTACTGCCTCGGCTTGGGTCGAGCACATCAACGGCGCCATGGCCCGGTTCGAGATCAACACGGCTGAGCGGGTGGCGATGTTCCTGGCTCAGGTCGGACACGAAAGCCAGAGCCTCAAGCGTCTGATCGAGAACCTGAACTACTCCGCCGAATTGTTGCTCCGTACTTGGCCGAAGCGGTTCACGCCGGCAGAGGCGAAGCAGTACGCACGCCAGCCTGAGCGCATCGCGAACCGCGTCTACGCAAACCGGATGGGCAACGGGTCGCCGGATACGGGCGATGGGTATCGATACCGGGGACGCGGCCTGATCATGATTACGGGCCACGACAACTACGCGGAAGCTGCACGCGCCCTGGCGCTGCCGCTGGTAGCGCAGCCTGAACTGCTGGAGCAACGGACCTGGGCAGCAATTGCCGCGGGTTGGTTCTGGCAGTCGCGGGGTTTAAACGACCTGGCTGACCAAGGCCGATTCGAGAAGATTACTCTCCGCATAAACGGATCGTTCACCGGGGCTGAGGATCGCAACGCCCGGCTCGAATGGGCGCGTGCTGCGCTCAAGGGGGAATGA

>[denovogenes]_139146

CTCGCCCCCTTCACCTACCGGCCCACCGTCCGCGGGGATCTGATCCCTCTGTCGGACAACGCCATGGTGGCGGCGGCCCAGGTCATGGGGGTCCGTCCCCTGCTACACCTGGCCAACCTCACGGAGGCGGACAGCTTCTCCGGCGAGCTGGCCCATGCCCTTCTGTCCAGCCCAGAGGCCCGGACACGCCTGGCGGATCATTTGATGGAACTCCTGCACCGAAATGGGTACCGCGGGATCGATGTAGATCTGGAGTCCATCTTCCCCGAGGACGCCCAAAACTATGTCCGATTCCTCTTCCTACTGCGGGAGCGTCTGGAGCCCCTGGGGTATCCCCTGCTGGCCGCCCTGGCGCCCAAAACCTCCGCCTGTCAGCAGGGAGAACTGTACCAGGGCCATGATTACCGCGGCCTGGGGGAAGCGGCGGACGGGGTCCTCCTGATGACCTACGAGTGGGGCTACGCCTTCGGCCCTCCTATGGCGGTGGCCCCTCTGGATCAGGTGCGGCGTGTGGCGGAGTATGCCCTTACAGAGGTCCCGGCGGAAAAAATCTGGCTGGGCATCCCCAACTACGGCTACGACTGGCTCCTCTCCGCGCCACAGGGAAGCCAGGCCCGCTCCCTGTCCAATCCG

>[denovogenes]_67050

GGTCGCGAAGCGTTTATCGACAAAATCTGGCAGTGGAAAGCGGAATCTGGCGGCACCATTACCCGTCAGATGCGCCGTCTCGGCAACTCCGTTGACTGGGAGCGCGAGCGCTTCACCATGGACGAAGGTCTTTCCAATGCCGTGAAAGAAGTCTTTGTTCGCCTGTACAAAGAAGACCTGATTTACCGTGGCAAACGCCTGGTGAACTGGGACCCGAAACTGCGCACCGCCATCTCTGACCTGGAAGTGGAAAACCGCGAGTCCAAAGGCTCAATGTGGCACATCCGCTATCCGCTGGCCGACGGCGTGAAGACCGCAGACGGTAAAGATTACCTGGTCGTCGCTACCACCCGTCCGGAAACTATTCTCGGGGATACCGGCGTGGCCGTTAACCCGGAAGATCCGCGCTACAAAGATCTGATCGGTAAATTCGTTGTCCTGCCGCTGGTTGACCGCCGTATTCCGATCGTGGGCGACGAACACGCCGACATGGAAAAAGGCACCGGCTGCGTGAAGATCACCCCGGCGCACGACTTTAATGACTACGAAGTCGGGAAACGTCACGGCCTGCCGATGATCAACATTCTGACCTTTGACGGCGACATCCGTGAAACTGCGGAAGTGTACGACACCAAAGGCGAAGAGTCCGACGTTTACTCCAACGCGATTCCGGCTGAGTTCCAGAAGCTGGAACGCTTTGCCGCGCGTAAAGCGATCGTCGCAGCCGTTGACGCGCTGGGCCTGCTGGAAGAAATTAAACCGCACGACCTGACCGTTCCTTACGGCGACCGTGGCGGCGTGGTTATCGAACCGATGCTGACCGACCAGTGGTACGTCCGTGCCGACGTGCTGGCGAAACCAGCGGTTGAAGCCGTTGAAAACGGCAGCATTCAGTTCGTGCCGAAGCAGTACGAAAACATGTACTTCTCCTGGATGCGTGATATTCAGGACTGGTGTATCTCTCGTCAACTGTGGTGGGGTCACCGTATCCCGGCATGGTACGACAACGACGGCAACGTTTATGTAGGCCGT
