# Supplementary material for: The Fecal Metagenomics of Malayan Pangolins Identifies an Extensive Adaptation to Myrmecophagy
Source: Front Microbiol. 2018 Nov 23;9:2793. doi: 10.3389/fmicb.2018.02793 (PMC6265309; doi:10.3389/fmicb.2018.02793)
Supplement: Supplementary file 1 [file Data_Sheet_1.zip › 410136-supplementary materials/Table S1.docx]

Table S1 The detail information of the samples

|  | A-1 | A-2 | B-1 | B-2 |
| --- | --- | --- | --- | --- |
| Wild  or captive | Captive | Captive | Captive | Captive |
| Age | Adult | Adult | Adult | Adult |
| Weight | 2.0 kg | 2.25kg | 3.05 kg | 2.6 kg |
| Gender | Female | Female | Male | Female |
| Diet  per day | dried ants: 10g, dried termites: 10g, and water: 80g | dried ants: 10g, dried termites: 10g, and water: 80g | dried ants: 10g, dried termites: 10g, and water: 80g | dried ants: 10g, dried termites: 10g, and water: 80g |
| Physiological state | Healthy | Healthy | Dead | Dead |
