# Supplementary material for: The Fecal Metagenomics of Malayan Pangolins Identifies an Extensive Adaptation to Myrmecophagy
Source: Front Microbiol. 2018 Nov 23;9:2793. doi: 10.3389/fmicb.2018.02793 (PMC6265309; doi:10.3389/fmicb.2018.02793)
Supplement: Supplementary file 1 [file Data_Sheet_1.zip › 410136-supplementary materials/S Files/File S2.doc]

Gene_id	Identity	E_value	Kegg_geneID	Ko_id	Ko_name	Ko_defi	Ko_EC	Ko_class

[denovogenes]_10	99.85	0.0	mmk:MU9_60	K13735	yeeJ	adhesin/invasin	--	Human Diseases--Infectious diseases: Bacterial--Bacterial invasion of epithelial cells

[denovogenes]_1000	69.40	0.0	bfs:BF9343_0283	K01191	E3.2.1.24	alpha-mannosidase	3.2.1.24	Metabolism--Glycan biosynthesis and metabolism--Other glycan degradation

[denovogenes]_10000	98.14	0.0	eoh:ECO103_4157	K03654	recQ	ATP-dependent DNA helicase RecQ	3.6.4.12	Genetic Information Processing--Folding, sorting and degradation--RNA degradation

[denovogenes]_100001	100.00	0.0	efi:OG1RF_12555	K00068	srlD	sorbitol-6-phosphate 2-dehydrogenase	1.1.1.140	Metabolism--Carbohydrate metabolism--Fructose and mannose metabolism

[denovogenes]_100004	98.16	0.0	mmk:MU9_273	K00014	aroE	shikimate dehydrogenase	1.1.1.25	Metabolism--Amino acid metabolism--Phenylalanine, tyrosine and tryptophan biosynthesis|Metabolism--Global and overview maps--Metabolic pathways|Metabolism--Global and overview maps--Biosynthesis of secondary metabolites|Metabolism--Global and overview maps--Biosynthesis of antibiotics|Metabolism--Global and overview maps--Biosynthesis of amino acids

[denovogenes]_100005	89.01	0.0	cbi:CLJ_B0343	K01232	glvA	maltose-6'-phosphate glucosidase	3.2.1.122	Metabolism--Carbohydrate metabolism--Starch and sucrose metabolism

[denovogenes]_100006	72.10	2e-120	lacy:A4V08_32475	K06182	rluF	23S rRNA pseudouridine2604 synthase	5.4.99.21	--

[denovogenes]_100007	93.75	0.0	ecy:ECSE_P3-0002	K16951	--	--	--	Metabolism--Energy metabolism--Sulfur metabolism|Metabolism--Global and overview maps--Microbial metabolism in diverse environments

[denovogenes]_100008	89.45	4e-148	bprs:CK3_04800	K07497	K07497	putative transposase	--	--

[denovogenes]_100009	59.71	1e-107	cbh:CLC_1220	K07238	TC.ZIP, zupT, ZRT3, ZIP2	zinc transporter, ZIP family	--	--

[denovogenes]_100010	41.73	9e-56	ctc:CTC_01849	K07720	yesN	two-component system, response regulator YesN	--	Environmental Information Processing--Signal transduction--Two-component system

[denovogenes]_100011	100.00	0.0	efd:EFD32_2103	K02073	metQ	D-methionine transport system substrate-binding protein	--	Environmental Information Processing--Membrane transport--ABC transporters

[denovogenes]_100012	100.00	0.0	ene:ENT_26720	K02026	ABC.MS.P1	multiple sugar transport system permease protein	--	--

[denovogenes]_100014	100.00	0.0	stb:SGPB_0080	K09816	znuB	zinc transport system permease protein	--	Environmental Information Processing--Membrane transport--ABC transporters

[denovogenes]_100015	100.00	0.0	stb:SGPB_0715	K02796	PTS-Man-EIID, manZ	PTS system, mannose-specific IID component	--	Metabolism--Carbohydrate metabolism--Fructose and mannose metabolism|Metabolism--Carbohydrate metabolism--Amino sugar and nucleotide sugar metabolism|Metabolism--Global and overview maps--Metabolic pathways|Environmental Information Processing--Membrane transport--Phosphotransferase system (PTS)

[denovogenes]_100016	97.06	0.0	sgt:SGGB_2157	K03496	parA, soj	chromosome partitioning protein	--	--

[denovogenes]_100019	56.11	4e-103	cpae:CPAST_c16040	K06864	K06864	uncharacterized protein	--	--

[denovogenes]_100020	100.00	0.0	seh:SeHA_A0102	K12204	dotC, traI	defect in organelle trafficking protein DotC	--	--

[denovogenes]_100021	87.13	2e-172	cbv:U729_2175	K02026	ABC.MS.P1	multiple sugar transport system permease protein	--	--

[denovogenes]_100023	99.63	0.0	efi:OG1RF_12137	K00878	thiM	hydroxyethylthiazole kinase	2.7.1.50	Metabolism--Metabolism of cofactors and vitamins--Thiamine metabolism|Metabolism--Global and overview maps--Metabolic pathways

[denovogenes]_100024	85.93	9e-173	cbv:U729_787	K00615	E2.2.1.1, tktA, tktB	transketolase	2.2.1.1	Metabolism--Carbohydrate metabolism--Pentose phosphate pathway|Metabolism--Energy metabolism--Carbon fixation in photosynthetic organisms|Metabolism--Metabolism of terpenoids and polyketides--Biosynthesis of ansamycins|Metabolism--Global and overview maps--Metabolic pathways|Metabolism--Global and overview maps--Biosynthesis of secondary metabolites|Metabolism--Global and overview maps--Microbial metabolism in diverse environments|Metabolism--Global and overview maps--Biosynthesis of antibiotics|Metabolism--Global and overview maps--Carbon metabolism|Metabolism--Global and overview maps--Biosynthesis of amino acids

[denovogenes]_100025	69.85	3e-140	cbut:ATN24_18130	K16785	ecfT	energy-coupling factor transport system permease protein	--	Environmental Information Processing--Membrane transport--ABC transporters

[denovogenes]_100026	89.67	0.0	eas:Entas_3214	K03337	iolB	5-deoxy-glucuronate isomerase	5.3.1.30	Metabolism--Carbohydrate metabolism--Inositol phosphate metabolism|Metabolism--Global and overview maps--Metabolic pathways|Metabolism--Global and overview maps--Microbial metabolism in diverse environments

[denovogenes]_100028	99.63	0.0	pmib:BB2000_2511	K08994	yneE	putative membrane protein	--	--

[denovogenes]_100029	46.27	3e-73	cbk:CLL_A1189	K06383	spoIIGA	stage II sporulation protein GA (sporulation sigma-E factor processing peptidase)	3.4.23.-	--

[denovogenes]_10003	41.94	7e-175	cap:CLDAP_05760	K01811	xylS, yicI	alpha-D-xyloside xylohydrolase	3.2.1.177	--

[denovogenes]_100031	76.69	1e-140	cdg:CDBI1_09195	K02050	ABC.SN.P	NitT/TauT family transport system permease protein	--	--

[denovogenes]_100033	72.06	1e-144	cno:NT01CX_1498	K03783	punA, PNP	purine-nucleoside phosphorylase	2.4.2.1	Metabolism--Nucleotide metabolism--Purine metabolism|Metabolism--Nucleotide metabolism--Pyrimidine metabolism|Metabolism--Metabolism of cofactors and vitamins--Nicotinate and nicotinamide metabolism|Metabolism--Global and overview maps--Metabolic pathways|Metabolism--Global and overview maps--Biosynthesis of secondary metabolites

[denovogenes]_100034	100.00	0.0	efi:OG1RF_11829	K09013	sufC	Fe-S cluster assembly ATP-binding protein	--	--

[denovogenes]_100035	77.32	1e-153	eal:EAKF1_ch3928	K11014	cdtB	cytolethal distending toxin subunit B	--	--

[denovogenes]_100036	70.97	1e-145	drm:Dred_2235	K17073	lysX1	putative lysine transport system substrate-binding protein	--	Environmental Information Processing--Membrane transport--ABC transporters

[denovogenes]_100037	63.45	1e-101	btrh:F543_15750	K08357	ttrA	tetrathionate reductase subunit A	--	Metabolism--Energy metabolism--Sulfur metabolism|Metabolism--Global and overview maps--Microbial metabolism in diverse environments|Environmental Information Processing--Signal transduction--Two-component system

[denovogenes]_100038	77.21	7e-156	cdg:CDBI1_02390	K01258	pepT	tripeptide aminopeptidase	3.4.11.4	--

[denovogenes]_100043	99.27	0.0	cbut:ATN24_05290	K06438	yqfD	similar to stage IV sporulation protein	--	--

[denovogenes]_100045	99.63	0.0	cbut:ATN24_16715	K02030	ABC.PA.S	polar amino acid transport system substrate-binding protein	--	--

[denovogenes]_100046	68.77	1e-127	vpr:Vpar_0292	K18672	dacA	diadenylate cyclase	2.7.7.85	--

[denovogenes]_100047	81.44	3e-159	cpr:CPR_0076	K09773	ppsR	[pyruvate, water dikinase]-phosphate phosphotransferase / [pyruvate, water dikinase] kinase	2.7.4.28 2.7.11.33	--

[denovogenes]_100048	42.48	9e-56	vap:Vapar_6281	K03574	mutT, NUDT15, MTH2	8-oxo-dGTP diphosphatase	3.6.1.55	--

[denovogenes]_100049	83.52	6e-163	fnt:HMPREF0405_00588	K19509	PTS-Gfr-EIID, gfrD	PTS system, fructoselysine/glucoselysine-specific IID component	--	Environmental Information Processing--Membrane transport--Phosphotransferase system (PTS)

[denovogenes]_10005	99.43	0.0	boa:Bovatus_04041	K03655	recG	ATP-dependent DNA helicase RecG	3.6.4.12	Genetic Information Processing--Replication and repair--Homologous recombination

[denovogenes]_100050	89.30	0.0	eas:Entas_3214	K03337	iolB	5-deoxy-glucuronate isomerase	5.3.1.30	Metabolism--Carbohydrate metabolism--Inositol phosphate metabolism|Metabolism--Global and overview maps--Metabolic pathways|Metabolism--Global and overview maps--Microbial metabolism in diverse environments

[denovogenes]_100053	100.00	0.0	cbut:ATN24_05025	K02556	motA	chemotaxis protein MotA	--	Environmental Information Processing--Signal transduction--Two-component system|Cellular Processes--Cell motility--Bacterial chemotaxis|Cellular Processes--Cell motility--Flagellar assembly

[denovogenes]_100055	62.59	2e-117	csb:CLSA_c14030	K03470	rnhB	ribonuclease HII	3.1.26.4	Genetic Information Processing--Replication and repair--DNA replication

[denovogenes]_100056	90.41	1e-179	cif:AL515_11185	K02028	ABC.PA.A	polar amino acid transport system ATP-binding protein	3.6.3.21	--

[denovogenes]_100057	40.62	1e-16	abl:A7H1H_1606	K03496	parA, soj	chromosome partitioning protein	--	--

[denovogenes]_100058	70.89	2e-126	cce:Ccel_0195	K01990	ABC-2.A	ABC-2 type transport system ATP-binding protein	--	--

[denovogenes]_100059	43.46	3e-67	cpr:CPR_0474	K02099	--	--	--	--

[denovogenes]_10006	88.25	0.0	pdi:BDI_2699	K03655	recG	ATP-dependent DNA helicase RecG	3.6.4.12	Genetic Information Processing--Replication and repair--Homologous recombination

[denovogenes]_100060	100.00	0.0	pvl:AOB99_10020	K06897	K06897	7,8-dihydropterin-6-yl-methyl-4-(beta-D-ribofuranosyl)aminobenzene 5'-phosphate synthase	2.5.1.105	--

[denovogenes]_100061	78.38	6e-148	prg:RB151_002650	K02170	bioH	pimeloyl-[acyl-carrier protein] methyl ester esterase	3.1.1.85	Metabolism--Metabolism of cofactors and vitamins--Biotin metabolism|Metabolism--Global and overview maps--Metabolic pathways

[denovogenes]_100064	79.85	8e-156	vpr:Vpar_0540	K02527	kdtA, waaA	3-deoxy-D-manno-octulosonic-acid transferase	2.4.99.12 2.4.99.13 2.4.99.14 2.4.99.15	Metabolism--Glycan biosynthesis and metabolism--Lipopolysaccharide biosynthesis|Metabolism--Global and overview maps--Metabolic pathways

[denovogenes]_100065	44.07	9e-79	dba:Dbac_2248	K00459	ncd2, npd	nitronate monooxygenase	1.13.12.16	Metabolism--Energy metabolism--Nitrogen metabolism

[denovogenes]_100066	52.59	4e-95	vpr:Vpar_0430	K01776	murI	glutamate racemase	5.1.1.3	Metabolism--Metabolism of other amino acids--D-Glutamine and D-glutamate metabolism|Metabolism--Global and overview maps--Metabolic pathways

[denovogenes]_10007	97.13	0.0	bxy:BXY_03600	K09955	K09955	uncharacterized protein	--	--

[denovogenes]_100071	100.00	0.0	pmr:PMI3408	K07259	dacB	D-alanyl-D-alanine carboxypeptidase / D-alanyl-D-alanine-endopeptidase (penicillin-binding protein 4)	3.4.16.4 3.4.21.-	Metabolism--Glycan biosynthesis and metabolism--Peptidoglycan biosynthesis

[denovogenes]_100074	93.41	2e-171	cama:F384_03215	K03453	TC.BASS	bile acid:Na+ symporter, BASS family	--	--

[denovogenes]_100075	51.46	8e-81	agl:PYTT_1124	K01686	uxuA	mannonate dehydratase	4.2.1.8	Metabolism--Carbohydrate metabolism--Pentose and glucuronate interconversions|Metabolism--Global and overview maps--Metabolic pathways

[denovogenes]_100078	100.00	0.0	cbut:ATN24_15455	K04517	tyrA2	prephenate dehydrogenase	1.3.1.12	Metabolism--Amino acid metabolism--Phenylalanine, tyrosine and tryptophan biosynthesis|Metabolism--Biosynthesis of other secondary metabolites--Novobiocin biosynthesis|Metabolism--Global and overview maps--Metabolic pathways|Metabolism--Global and overview maps--Biosynthesis of secondary metabolites|Metabolism--Global and overview maps--Biosynthesis of antibiotics|Metabolism--Global and overview maps--Biosynthesis of amino acids

[denovogenes]_100079	95.59	0.0	psx:DR96_1138	K02108	ATPF0A, atpB	F-type H+-transporting ATPase subunit a	--	Metabolism--Energy metabolism--Oxidative phosphorylation|Metabolism--Energy metabolism--Photosynthesis|Metabolism--Global and overview maps--Metabolic pathways

[denovogenes]_10008	99.85	0.0	ecc:c5174	K16089	TC.FEV.OM2, cirA, cfrA, hmuR	outer membrane receptor for ferrienterochelin and colicins	--	--

[denovogenes]_100080	66.79	5e-119	str:Sterm_1670	K03458	TC.NCS2	nucleobase:cation symporter-2, NCS2 family	--	--

[denovogenes]_100082	90.07	0.0	prg:RB151_005520	K01652	E2.2.1.6L, ilvB, ilvG, ilvI	acetolactate synthase I/II/III large subunit	2.2.1.6	Metabolism--Amino acid metabolism--Valine, leucine and isoleucine biosynthesis|Metabolism--Carbohydrate metabolism--Butanoate metabolism|Metabolism--Carbohydrate metabolism--C5-Branched dibasic acid metabolism|Metabolism--Metabolism of cofactors and vitamins--Pantothenate and CoA biosynthesis|Metabolism--Global and overview maps--Metabolic pathways|Metabolism--Global and overview maps--Biosynthesis of secondary metabolites|Metabolism--Global and overview maps--Biosynthesis of antibiotics|Metabolism--Global and overview maps--2-Oxocarboxylic acid metabolism|Metabolism--Global and overview maps--Biosynthesis of amino acids

[denovogenes]_100083	53.56	7e-105	shv:AAT16_11215	K02034	ABC.PE.P1	peptide/nickel transport system permease protein	--	Cellular Processes--Cellular community - prokaryotes--Quorum sensing

[denovogenes]_100086	96.25	0.0	spen:107002298	K03386	PRDX2_4, ahpC	peroxiredoxin (alkyl hydroperoxide reductase subunit C)	1.11.1.15	Cellular Processes--Cell growth and death--Apoptosis - fly

[denovogenes]_100087	99.63	0.0	ecm:EcSMS35_1804	K10112	msmX, msmK, malK, sugC, ggtA, msiK	multiple sugar transport system ATP-binding protein	--	Environmental Information Processing--Membrane transport--ABC transporters

[denovogenes]_100088	48.00	5e-80	cbt:CLH_0561	K08301	rng, cafA	ribonuclease G	3.1.26.-	--

[denovogenes]_100089	87.69	9e-172	cle:Clole_1453	K01624	FBA, fbaA	fructose-bisphosphate aldolase, class II	4.1.2.13	Metabolism--Carbohydrate metabolism--Glycolysis / Gluconeogenesis|Metabolism--Carbohydrate metabolism--Pentose phosphate pathway|Metabolism--Carbohydrate metabolism--Fructose and mannose metabolism|Metabolism--Energy metabolism--Methane metabolism|Metabolism--Energy metabolism--Carbon fixation in photosynthetic organisms|Metabolism--Global and overview maps--Metabolic pathways|Metabolism--Global and overview maps--Biosynthesis of secondary metabolites|Metabolism--Global and overview maps--Microbial metabolism in diverse environments|Metabolism--Global and overview maps--Biosynthesis of antibiotics|Metabolism--Global and overview maps--Carbon metabolism|Metabolism--Global and overview maps--Biosynthesis of amino acids

[denovogenes]_100090	100.00	0.0	eum:ECUMN_4241	K08163	mdtL	MFS transporter, DHA1 family, multidrug resistance protein	--	--

[denovogenes]_100091	78.02	2e-161	fnc:HMPREF0946_00150	K14534	abfD	4-hydroxybutyryl-CoA dehydratase / vinylacetyl-CoA-Delta-isomerase	4.2.1.120 5.3.3.3	Metabolism--Carbohydrate metabolism--Butanoate metabolism|Metabolism--Energy metabolism--Carbon fixation pathways in prokaryotes|Metabolism--Global and overview maps--Metabolic pathways|Metabolism--Global and overview maps--Microbial metabolism in diverse environments|Metabolism--Global and overview maps--Carbon metabolism

[denovogenes]_100092	45.79	2e-80	seni:CY43_24445	K12063	traC	conjugal transfer ATP-binding protein TraC	--	--

[denovogenes]_100096	73.53	8e-134	cbv:U729_1366	K03076	secY	preprotein translocase subunit SecY	--	Cellular Processes--Cellular community - prokaryotes--Quorum sensing|Genetic Information Processing--Folding, sorting and degradation--Protein export|Environmental Information Processing--Membrane transport--Bacterial secretion system

[denovogenes]_100097	69.37	3e-119	cno:NT01CX_0984	K00865	glxK, garK	glycerate 2-kinase	2.7.1.165	Metabolism--Amino acid metabolism--Glycine, serine and threonine metabolism|Metabolism--Lipid metabolism--Glycerolipid metabolism|Metabolism--Carbohydrate metabolism--Glyoxylate and dicarboxylate metabolism|Metabolism--Global and overview maps--Metabolic pathways|Metabolism--Global and overview maps--Microbial metabolism in diverse environments|Metabolism--Global and overview maps--Biosynthesis of antibiotics

[denovogenes]_100098	71.79	7e-144	cbz:Cbs_0246	K00820	glmS, GFPT	glucosamine--fructose-6-phosphate aminotransferase (isomerizing)	2.6.1.16	Metabolism--Amino acid metabolism--Alanine, aspartate and glutamate metabolism|Metabolism--Carbohydrate metabolism--Amino sugar and nucleotide sugar metabolism|Metabolism--Global and overview maps--Metabolic pathways|Metabolism--Global and overview maps--Biosynthesis of antibiotics|Human Diseases--Endocrine and metabolic diseases--Insulin resistance

[denovogenes]_100100	79.85	9e-159	cdl:CDR20291_1228	K00864	glpK, GK	glycerol kinase	2.7.1.30	Metabolism--Lipid metabolism--Glycerolipid metabolism|Metabolism--Global and overview maps--Metabolic pathways|Organismal Systems--Endocrine system--PPAR signaling pathway|Organismal Systems--Environmental adaptation--Plant-pathogen interaction

[denovogenes]_100101	96.70	0.0	cfd:CFNIH1_11655	K01258	pepT	tripeptide aminopeptidase	3.4.11.4	--

[denovogenes]_100106	93.77	0.0	vpr:Vpar_0877	K03596	lepA	GTP-binding protein LepA	--	Human Diseases--Infectious diseases: Bacterial--Legionellosis

[denovogenes]_100108	52.73	1e-94	cbv:U729_2415	K11104	melB	melibiose permease	--	--

[denovogenes]_100109	49.82	6e-90	lha:LHA_2867	K07316	mod	adenine-specific DNA-methyltransferase	2.1.1.72	--

[denovogenes]_100110	43.43	4e-80	ain:Acin_2092	K05825	LYSN	2-aminoadipate transaminase	2.6.1.-	Metabolism--Amino acid metabolism--Lysine biosynthesis|Metabolism--Global and overview maps--Metabolic pathways|Metabolism--Global and overview maps--Biosynthesis of antibiotics|Metabolism--Global and overview maps--2-Oxocarboxylic acid metabolism|Metabolism--Global and overview maps--Biosynthesis of amino acids

[denovogenes]_100111	46.67	7e-80	cle:Clole_1134	K07636	phoR	two-component system, OmpR family, phosphate regulon sensor histidine kinase PhoR	2.7.13.3	Environmental Information Processing--Signal transduction--Two-component system

[denovogenes]_100112	68.91	3e-130	afn:Acfer_0852	K06442	tlyA	23S rRNA (cytidine1920-2'-O)/16S rRNA (cytidine1409-2'-O)-methyltransferase	2.1.1.226 2.1.1.227	--

[denovogenes]_100113	46.93	9e-79	afn:Acfer_1026	K07053	E3.1.3.97	3',5'-nucleoside bisphosphate phosphatase	3.1.3.97	--

[denovogenes]_100117	51.84	1e-96	rto:RTO_25320	K06864	K06864	uncharacterized protein	--	--

[denovogenes]_100118	65.40	7e-121	mhg:MHY_08200	K00868	pdxK, pdxY	pyridoxine kinase	2.7.1.35	Metabolism--Metabolism of cofactors and vitamins--Vitamin B6 metabolism|Metabolism--Global and overview maps--Metabolic pathways

[denovogenes]_100119	83.70	2e-168	bhl:Bache_2855	K01082	cysQ, MET22, BPNT1	3'(2'), 5'-bisphosphate nucleotidase	3.1.3.7	Metabolism--Energy metabolism--Sulfur metabolism|Metabolism--Global and overview maps--Metabolic pathways|Metabolism--Global and overview maps--Microbial metabolism in diverse environments|Metabolism--Global and overview maps--Biosynthesis of antibiotics

[denovogenes]_100120	76.75	1e-152	ain:Acin_0629	K01627	kdsA	2-dehydro-3-deoxyphosphooctonate aldolase (KDO 8-P synthase)	2.5.1.55	Metabolism--Glycan biosynthesis and metabolism--Lipopolysaccharide biosynthesis|Metabolism--Global and overview maps--Metabolic pathways

[denovogenes]_100121	64.34	4e-118	afn:Acfer_0363	K00757	udp, UPP	uridine phosphorylase	2.4.2.3	Metabolism--Nucleotide metabolism--Pyrimidine metabolism|Metabolism--Xenobiotics biodegradation and metabolism--Drug metabolism - other enzymes|Metabolism--Global and overview maps--Metabolic pathways

[denovogenes]_100125	92.04	5e-130	bcel:BcellWH2_01404	K03550	ruvA	holliday junction DNA helicase RuvA	3.6.4.12	Genetic Information Processing--Replication and repair--Homologous recombination

[denovogenes]_100128	70.68	3e-125	ain:Acin_0429	K16785	ecfT	energy-coupling factor transport system permease protein	--	Environmental Information Processing--Membrane transport--ABC transporters

[denovogenes]_100129	99.63	0.0	bdh:GV66_00535	K01591	pyrF	orotidine-5'-phosphate decarboxylase	4.1.1.23	Metabolism--Nucleotide metabolism--Pyrimidine metabolism|Metabolism--Global and overview maps--Metabolic pathways

[denovogenes]_100132	74.81	7e-144	byl:A4V09_12350	K11071	potB	spermidine/putrescine transport system permease protein	--	Environmental Information Processing--Membrane transport--ABC transporters

[denovogenes]_100133	71.85	9e-135	ssg:Selsp_1204	K02588	nifH	nitrogenase iron protein NifH	1.18.6.1	Metabolism--Xenobiotics biodegradation and metabolism--Chloroalkane and chloroalkene degradation|Metabolism--Energy metabolism--Nitrogen metabolism|Metabolism--Global and overview maps--Metabolic pathways|Metabolism--Global and overview maps--Microbial metabolism in diverse environments

[denovogenes]_100134	47.01	6e-74	sgp:SpiGrapes_0573	K01201	GBA, srfJ	glucosylceramidase	3.2.1.45	Metabolism--Glycan biosynthesis and metabolism--Other glycan degradation|Metabolism--Lipid metabolism--Sphingolipid metabolism|Metabolism--Global and overview maps--Metabolic pathways|Cellular Processes--Transport and catabolism--Lysosome

[denovogenes]_100135	67.07	5e-128	pgi:PG_0425	K01070	frmB, ESD, fghA	S-formylglutathione hydrolase	3.1.2.12	Metabolism--Energy metabolism--Methane metabolism|Metabolism--Global and overview maps--Microbial metabolism in diverse environments|Metabolism--Global and overview maps--Carbon metabolism

[denovogenes]_100138	51.72	9e-98	rbc:BN938_0136	K06223	dam	DNA adenine methylase	2.1.1.72	Genetic Information Processing--Replication and repair--Mismatch repair

[denovogenes]_10014	99.71	0.0	mmk:MU9_439	K01414	prlC	oligopeptidase A	3.4.24.70	--

[denovogenes]_100141	70.82	4e-135	byl:A4V09_19315	K01567	pdaA	peptidoglycan-N-acetylmuramic acid deacetylase	3.5.1.-	--

[denovogenes]_100142	75.66	2e-150	fpla:A4U99_05115	K04070	pflX	putative pyruvate formate lyase activating enzyme	1.97.1.4	--

[denovogenes]_100143	62.65	1e-120	pgi:PG_0425	K01070	frmB, ESD, fghA	S-formylglutathione hydrolase	3.1.2.12	Metabolism--Energy metabolism--Methane metabolism|Metabolism--Global and overview maps--Microbial metabolism in diverse environments|Metabolism--Global and overview maps--Carbon metabolism

[denovogenes]_100144	100.00	0.0	bvu:BVU_3057	K07387	K07387	putative metalloprotease	3.4.24.-	--

[denovogenes]_100147	86.94	3e-173	ruk:A4V00_18300	K01990	ABC-2.A	ABC-2 type transport system ATP-binding protein	--	--

[denovogenes]_100148	69.74	6e-132	bhl:Bache_2367	K00941	thiD	hydroxymethylpyrimidine/phosphomethylpyrimidine kinase	2.7.1.49 2.7.4.7	Metabolism--Metabolism of cofactors and vitamins--Thiamine metabolism|Metabolism--Global and overview maps--Metabolic pathways

[denovogenes]_10015	40.03	3e-144	thl:TEH_14470	K02238	comEC	competence protein ComEC	--	--

[denovogenes]_100150	42.86	3e-26	mro:MROS_0358	K02477	K02477	two-component system, LytT family, response regulator	--	--

[denovogenes]_100153	69.78	1e-134	cad:Curi_c18810	K06153	bacA	undecaprenyl-diphosphatase	3.6.1.27	Metabolism--Glycan biosynthesis and metabolism--Peptidoglycan biosynthesis

[denovogenes]_100154	61.45	2e-121	pgi:PG_0425	K01070	frmB, ESD, fghA	S-formylglutathione hydrolase	3.1.2.12	Metabolism--Energy metabolism--Methane metabolism|Metabolism--Global and overview maps--Microbial metabolism in diverse environments|Metabolism--Global and overview maps--Carbon metabolism

[denovogenes]_100155	57.14	3e-94	byl:A4V09_17760	K02025	ABC.MS.P	multiple sugar transport system permease protein	--	--

[denovogenes]_100156	91.91	0.0	fpla:A4U99_12195	K07053	E3.1.3.97	3',5'-nucleoside bisphosphate phosphatase	3.1.3.97	--

[denovogenes]_100157	99.21	0.0	btho:Btheta7330_00522	K01803	TPI, tpiA	triosephosphate isomerase (TIM)	5.3.1.1	Metabolism--Carbohydrate metabolism--Glycolysis / Gluconeogenesis|Metabolism--Carbohydrate metabolism--Fructose and mannose metabolism|Metabolism--Carbohydrate metabolism--Inositol phosphate metabolism|Metabolism--Energy metabolism--Carbon fixation in photosynthetic organisms|Metabolism--Global and overview maps--Metabolic pathways|Metabolism--Global and overview maps--Biosynthesis of secondary metabolites|Metabolism--Global and overview maps--Microbial metabolism in diverse environments|Metabolism--Global and overview maps--Biosynthesis of antibiotics|Metabolism--Global and overview maps--Carbon metabolism|Metabolism--Global and overview maps--Biosynthesis of amino acids

[denovogenes]_100159	52.59	4e-96	bmyc:DJ92_1181	K03783	punA, PNP	purine-nucleoside phosphorylase	2.4.2.1	Metabolism--Nucleotide metabolism--Purine metabolism|Metabolism--Nucleotide metabolism--Pyrimidine metabolism|Metabolism--Metabolism of cofactors and vitamins--Nicotinate and nicotinamide metabolism|Metabolism--Global and overview maps--Metabolic pathways|Metabolism--Global and overview maps--Biosynthesis of secondary metabolites

[denovogenes]_100164	77.70	6e-158	bfs:BF9343_1487	K07011	K07011	uncharacterized protein	--	--

[denovogenes]_100166	98.16	0.0	lacy:A4V08_27595	K03496	parA, soj	chromosome partitioning protein	--	--

[denovogenes]_100169	63.24	4e-130	lbn:LBUCD034_0350	K10536	aguA	agmatine deiminase	3.5.3.12	Metabolism--Amino acid metabolism--Arginine and proline metabolism|Metabolism--Global and overview maps--Metabolic pathways

[denovogenes]_10017	98.85	0.0	cif:AL515_05865	K02400	flhA	flagellar biosynthesis protein FlhA	--	Cellular Processes--Cell motility--Flagellar assembly

[denovogenes]_100170	61.63	9e-108	bprl:CL2_15630	K03769	ppiC	peptidyl-prolyl cis-trans isomerase C	5.2.1.8	--

[denovogenes]_100174	99.26	0.0	fpla:A4U99_15740	K10709	frlC	protein FrlC	--	--

[denovogenes]_10018	61.78	0.0	cpr:CPR_0819	K07003	K07003	uncharacterized protein	--	--

[denovogenes]_100181	55.51	6e-109	byl:A4V09_08195	K20489	nisI	lantibiotic immunity protein	--	Environmental Information Processing--Signal transduction--Two-component system|Cellular Processes--Cellular community - prokaryotes--Quorum sensing

[denovogenes]_100182	98.90	0.0	bvu:BVU_2653	K13002	wbyL	glycosyltransferase	2.4.1.-	--

[denovogenes]_100185	41.27	4e-45	bfi:CIY_18840	K00969	nadD	nicotinate-nucleotide adenylyltransferase	2.7.7.18	Metabolism--Metabolism of cofactors and vitamins--Nicotinate and nicotinamide metabolism|Metabolism--Global and overview maps--Metabolic pathways

[denovogenes]_100187	93.68	8e-177	cfd:CFNIH1_16970	K02028	ABC.PA.A	polar amino acid transport system ATP-binding protein	3.6.3.21	--

[denovogenes]_100188	61.92	3e-120	cpro:CPRO_13780	K06223	dam	DNA adenine methylase	2.1.1.72	Genetic Information Processing--Replication and repair--Mismatch repair

[denovogenes]_100189	51.78	2e-88	deh:cbdbA122	K03605	hyaD, hybD	hydrogenase maturation protease	3.4.23.-	--

[denovogenes]_10019	73.13	0.0	ctx:Clo1313_2038	K01915	glnA, GLUL	glutamine synthetase	6.3.1.2	Metabolism--Amino acid metabolism--Arginine biosynthesis|Metabolism--Amino acid metabolism--Alanine, aspartate and glutamate metabolism|Metabolism--Carbohydrate metabolism--Glyoxylate and dicarboxylate metabolism|Metabolism--Energy metabolism--Nitrogen metabolism|Metabolism--Global and overview maps--Metabolic pathways|Metabolism--Global and overview maps--Microbial metabolism in diverse environments|Metabolism--Global and overview maps--Biosynthesis of amino acids|Environmental Information Processing--Signal transduction--Two-component system|Organismal Systems--Nervous system--Glutamatergic synapse|Organismal Systems--Nervous system--GABAergic synapse

[denovogenes]_100190	49.40	6e-74	ibu:IB211_00542	K02013	ABC.FEV.A	iron complex transport system ATP-binding protein	3.6.3.34	Environmental Information Processing--Membrane transport--ABC transporters

[denovogenes]_100191	59.63	1e-114	csh:Closa_1225	K02026	ABC.MS.P1	multiple sugar transport system permease protein	--	--

[denovogenes]_100192	97.06	0.0	cfd:CFNIH1_11825	K05818	mhpR	IclR family transcriptional regulator, mhp operon transcriptional activator	--	--

[denovogenes]_100193	97.06	0.0	lacy:A4V08_08260	K07699	spo0A	two-component system, response regulator, stage 0 sporulation protein A	--	Environmental Information Processing--Signal transduction--Two-component system|Cellular Processes--Cellular community - prokaryotes--Quorum sensing

[denovogenes]_100194	82.33	6e-163	spe:Spro_4278	K02771	PTS-Fru1-EIID, levG	PTS system, fructose-specific IID component	--	Metabolism--Carbohydrate metabolism--Fructose and mannose metabolism|Environmental Information Processing--Membrane transport--Phosphotransferase system (PTS)

[denovogenes]_100195	100.00	0.0	fpla:A4U99_02930	K18013	kce	3-keto-5-aminohexanoate cleavage enzyme	2.3.1.247	Metabolism--Amino acid metabolism--Lysine degradation

[denovogenes]_100196	57.20	8e-110	pdi:BDI_3839	K01784	galE, GALE	UDP-glucose 4-epimerase	5.1.3.2	Metabolism--Carbohydrate metabolism--Galactose metabolism|Metabolism--Carbohydrate metabolism--Amino sugar and nucleotide sugar metabolism|Metabolism--Global and overview maps--Metabolic pathways

[denovogenes]_100198	76.01	7e-153	twi:Thewi_0850	K18013	kce	3-keto-5-aminohexanoate cleavage enzyme	2.3.1.247	Metabolism--Amino acid metabolism--Lysine degradation

[denovogenes]_100199	91.54	2e-180	csr:Cspa_c04460	K01951	guaA, GMPS	GMP synthase (glutamine-hydrolysing)	6.3.5.2	Metabolism--Nucleotide metabolism--Purine metabolism|Metabolism--Xenobiotics biodegradation and metabolism--Drug metabolism - other enzymes|Metabolism--Global and overview maps--Metabolic pathways

[denovogenes]_1002	61.99	0.0	afn:Acfer_1370	K03763	DPO3A2, polC	DNA polymerase III subunit alpha, Gram-positive type	2.7.7.7	Metabolism--Nucleotide metabolism--Purine metabolism|Metabolism--Nucleotide metabolism--Pyrimidine metabolism|Metabolism--Global and overview maps--Metabolic pathways|Genetic Information Processing--Replication and repair--DNA replication|Genetic Information Processing--Replication and repair--Mismatch repair|Genetic Information Processing--Replication and repair--Homologous recombination

[denovogenes]_10020	40.19	6e-101	pses:PSCI_4305	K21023	mucR	diguanylate cyclase	2.7.7.65	Cellular Processes--Cellular community - prokaryotes--Biofilm formation - Pseudomonas aeruginosa

[denovogenes]_100200	69.38	3e-131	ova:OBV_10550	K17074	lysX2	putative lysine transport system permease protein	--	Environmental Information Processing--Membrane transport--ABC transporters

[denovogenes]_100202	52.40	3e-99	ccel:CCDG5_0441	K07566	tsaC, rimN, SUA5	L-threonylcarbamoyladenylate synthase	2.7.7.87	--

[denovogenes]_100203	98.90	0.0	cfd:CFNIH1_04410	K03772	fkpA	FKBP-type peptidyl-prolyl cis-trans isomerase FkpA	5.2.1.8	--

[denovogenes]_100205	42.55	2e-74	bon:A361_23270	K19421	epsC	polysaccharide biosynthesis protein EpsC	--	--

[denovogenes]_100206	98.90	0.0	cfd:CFNIH1_02490	K06153	bacA	undecaprenyl-diphosphatase	3.6.1.27	Metabolism--Glycan biosynthesis and metabolism--Peptidoglycan biosynthesis

[denovogenes]_100209	71.10	8e-138	lacy:A4V08_31120	K02556	motA	chemotaxis protein MotA	--	Environmental Information Processing--Signal transduction--Two-component system|Cellular Processes--Cell motility--Bacterial chemotaxis|Cellular Processes--Cell motility--Flagellar assembly

[denovogenes]_100211	45.11	7e-69	caby:Cabys_3959	K03442	mscS	small conductance mechanosensitive channel	--	--

[denovogenes]_100213	82.22	3e-166	fpla:A4U99_13270	K04024	eutJ	ethanolamine utilization protein EutJ	--	--

[denovogenes]_100216	100.00	0.0	bvu:BVU_3940	K02040	pstS	phosphate transport system substrate-binding protein	--	Environmental Information Processing--Membrane transport--ABC transporters|Environmental Information Processing--Signal transduction--Two-component system|Human Diseases--Infectious diseases: Bacterial--Tuberculosis

[denovogenes]_100217	62.08	2e-122	cpe:CPE0296	K00615	E2.2.1.1, tktA, tktB	transketolase	2.2.1.1	Metabolism--Carbohydrate metabolism--Pentose phosphate pathway|Metabolism--Energy metabolism--Carbon fixation in photosynthetic organisms|Metabolism--Metabolism of terpenoids and polyketides--Biosynthesis of ansamycins|Metabolism--Global and overview maps--Metabolic pathways|Metabolism--Global and overview maps--Biosynthesis of secondary metabolites|Metabolism--Global and overview maps--Microbial metabolism in diverse environments|Metabolism--Global and overview maps--Biosynthesis of antibiotics|Metabolism--Global and overview maps--Carbon metabolism|Metabolism--Global and overview maps--Biosynthesis of amino acids

[denovogenes]_100218	100.00	0.0	bfr:BF1907	K01607	pcaC	4-carboxymuconolactone decarboxylase	4.1.1.44	Metabolism--Xenobiotics biodegradation and metabolism--Benzoate degradation|Metabolism--Global and overview maps--Metabolic pathways|Metabolism--Global and overview maps--Microbial metabolism in diverse environments|Metabolism--Global and overview maps--Degradation of aromatic compounds

[denovogenes]_100219	86.40	5e-174	lacy:A4V08_00925	K01714	dapA	4-hydroxy-tetrahydrodipicolinate synthase	4.3.3.7	Metabolism--Biosynthesis of other secondary metabolites--Monobactam biosynthesis|Metabolism--Amino acid metabolism--Lysine biosynthesis|Metabolism--Global and overview maps--Metabolic pathways|Metabolism--Global and overview maps--Biosynthesis of secondary metabolites|Metabolism--Global and overview maps--Microbial metabolism in diverse environments|Metabolism--Global and overview maps--Biosynthesis of antibiotics|Metabolism--Global and overview maps--Biosynthesis of amino acids

[denovogenes]_100220	62.59	3e-112	bfi:CIY_00340	K01870	IARS, ileS	isoleucyl-tRNA synthetase	6.1.1.5	Genetic Information Processing--Translation--Aminoacyl-tRNA biosynthesis

[denovogenes]_100221	48.96	4e-73	csh:Closa_0024	K07718	yesM	two-component system, sensor histidine kinase YesM	2.7.13.3	Environmental Information Processing--Signal transduction--Two-component system

[denovogenes]_100226	60.89	6e-112	csh:Closa_2567	K02755	PTS-Bgl-EIIA, bglF, bglP	PTS system, beta-glucoside-specific IIA component	2.7.1.-	Environmental Information Processing--Membrane transport--Phosphotransferase system (PTS)

[denovogenes]_100226	60.89	6e-112	csh:Closa_2567	K02756	PTS-Bgl-EIIB, bglF, bglP	PTS system, beta-glucoside-specific IIB component	2.7.1.-	Environmental Information Processing--Membrane transport--Phosphotransferase system (PTS)

[denovogenes]_100226	60.89	6e-112	csh:Closa_2567	K02757	PTS-Bgl-EIIC, bglF, bglP	PTS system, beta-glucoside-specific IIC component	--	Environmental Information Processing--Membrane transport--Phosphotransferase system (PTS)

[denovogenes]_100227	65.81	8e-133	paee:R70331_27175	K02057	ABC.SS.P	simple sugar transport system permease protein	--	--

[denovogenes]_100228	68.11	2e-125	fnc:HMPREF0946_01351	K00065	kduD	2-deoxy-D-gluconate 3-dehydrogenase	1.1.1.125	Metabolism--Carbohydrate metabolism--Pentose and glucuronate interconversions

[denovogenes]_100229	65.17	3e-117	cpy:Cphy_3446	K02123	ATPVI, ntpI, atpI	V/A-type H+/Na+-transporting ATPase subunit I	--	Metabolism--Energy metabolism--Oxidative phosphorylation|Metabolism--Global and overview maps--Metabolic pathways

[denovogenes]_100230	43.68	4e-75	mpg:Theba_2186	K02034	ABC.PE.P1	peptide/nickel transport system permease protein	--	Cellular Processes--Cellular community - prokaryotes--Quorum sensing

[denovogenes]_100231	45.26	2e-85	csh:Closa_2556	K02647	--	--	--	--

[denovogenes]_100232	97.06	0.0	cif:AL515_08930	K06911	K06911	uncharacterized protein	--	--

[denovogenes]_100233	74.43	6e-140	lacy:A4V08_06420	K18350	vanSC, vanSE, vanSG	two-component system, OmpR family, sensor histidine kinase VanS	2.7.13.3	Human Diseases--Drug resistance: Antimicrobial--Vancomycin resistance|Environmental Information Processing--Signal transduction--Two-component system

[denovogenes]_100234	40.00	3e-43	far:ABE41_015635	K18218	tet35	tetracycline resistance efflux pump	--	--

[denovogenes]_100236	40.39	6e-53	aeq:AEQU_1936	K07238	TC.ZIP, zupT, ZRT3, ZIP2	zinc transporter, ZIP family	--	--

[denovogenes]_100238	78.39	7e-158	psts:E05_27080	K01952	purL, PFAS	phosphoribosylformylglycinamidine synthase	6.3.5.3	Metabolism--Nucleotide metabolism--Purine metabolism|Metabolism--Global and overview maps--Metabolic pathways|Metabolism--Global and overview maps--Biosynthesis of secondary metabolites|Metabolism--Global and overview maps--Biosynthesis of antibiotics

[denovogenes]_100240	98.53	0.0	spen:107004399	K03231	EEF1A	elongation factor 1-alpha	--	Genetic Information Processing--Translation--RNA transport|Human Diseases--Infectious diseases: Bacterial--Legionellosis

[denovogenes]_100242	99.26	0.0	bvu:BVU_1601	K00946	thiL	thiamine-monophosphate kinase	2.7.4.16	Metabolism--Metabolism of cofactors and vitamins--Thiamine metabolism|Metabolism--Global and overview maps--Metabolic pathways

[denovogenes]_100243	83.58	1e-170	rto:RTO_27020	K00948	PRPS, prsA	ribose-phosphate pyrophosphokinase	2.7.6.1	Metabolism--Carbohydrate metabolism--Pentose phosphate pathway|Metabolism--Nucleotide metabolism--Purine metabolism|Metabolism--Global and overview maps--Metabolic pathways|Metabolism--Global and overview maps--Biosynthesis of secondary metabolites|Metabolism--Global and overview maps--Microbial metabolism in diverse environments|Metabolism--Global and overview maps--Biosynthesis of antibiotics|Metabolism--Global and overview maps--Carbon metabolism|Metabolism--Global and overview maps--Biosynthesis of amino acids

[denovogenes]_100245	100.00	0.0	shq:A0259_18145	K05596	iciA	LysR family transcriptional regulator, chromosome initiation inhibitor	--	--

[denovogenes]_100246	92.70	0.0	bcel:BcellWH2_02477	K09458	fabF	3-oxoacyl-[acyl-carrier-protein] synthase II	2.3.1.179	Metabolism--Lipid metabolism--Fatty acid biosynthesis|Metabolism--Metabolism of cofactors and vitamins--Biotin metabolism|Metabolism--Global and overview maps--Metabolic pathways|Metabolism--Global and overview maps--Fatty acid metabolism

[denovogenes]_100248	67.40	2e-130	lmob:BN419_2981	K03070	secA	preprotein translocase subunit SecA	--	Cellular Processes--Cellular community - prokaryotes--Quorum sensing|Genetic Information Processing--Folding, sorting and degradation--Protein export|Environmental Information Processing--Membrane transport--Bacterial secretion system

[denovogenes]_100252	71.64	8e-125	bprs:CK3_18990	K07717	ycbA, glnK	two-component system, sensor histidine kinase YcbA	2.7.13.3	Environmental Information Processing--Signal transduction--Two-component system

[denovogenes]_100256	97.80	0.0	cfd:CFNIH1_14280	K13892	gsiA	glutathione transport system ATP-binding protein	--	Environmental Information Processing--Membrane transport--ABC transporters

[denovogenes]_100257	63.74	2e-137	ibu:IB211_01815c	K00812	aspB	aspartate aminotransferase	2.6.1.1	Metabolism--Amino acid metabolism--Arginine biosynthesis|Metabolism--Amino acid metabolism--Alanine, aspartate and glutamate metabolism|Metabolism--Amino acid metabolism--Cysteine and methionine metabolism|Metabolism--Amino acid metabolism--Arginine and proline metabolism|Metabolism--Amino acid metabolism--Tyrosine metabolism|Metabolism--Amino acid metabolism--Phenylalanine metabolism|Metabolism--Amino acid metabolism--Phenylalanine, tyrosine and tryptophan biosynthesis|Metabolism--Biosynthesis of other secondary metabolites--Novobiocin biosynthesis|Metabolism--Biosynthesis of other secondary metabolites--Isoquinoline alkaloid biosynthesis|Metabolism--Biosynthesis of other secondary metabolites--Tropane, piperidine and pyridine alkaloid biosynthesis|Metabolism--Global and overview maps--Metabolic pathways|Metabolism--Global and overview maps--Biosynthesis of secondary metabolites|Metabolism--Global and overview maps--Biosynthesis of antibiotics|Metabolism--Global and overview maps--2-Oxocarboxylic acid metabolism|Metabolism--Global and overview maps--Biosynthesis of amino acids

[denovogenes]_100258	70.14	1e-132	cpro:CPRO_11450	K06147	ABCB-BAC	ATP-binding cassette, subfamily B, bacterial	--	--

[denovogenes]_10026	49.48	0.0	byl:A4V09_02620	K12132	prkC, stkP	eukaryotic-like serine/threonine-protein kinase	2.7.11.1	--

[denovogenes]_100261	49.26	4e-84	gho:AL542_17915	K16786	ecfA1	energy-coupling factor transport system ATP-binding protein	3.6.3.-	Environmental Information Processing--Membrane transport--ABC transporters

[denovogenes]_100261	49.26	4e-84	gho:AL542_17915	K16787	ecfA2	energy-coupling factor transport system ATP-binding protein	3.6.3.-	Environmental Information Processing--Membrane transport--ABC transporters

[denovogenes]_100262	95.60	0.0	bhl:Bache_0671	K06942	ychF	ribosome-binding ATPase	--	--

[denovogenes]_100263	68.63	2e-125	cbi:CLJ_B3928	K09458	fabF	3-oxoacyl-[acyl-carrier-protein] synthase II	2.3.1.179	Metabolism--Lipid metabolism--Fatty acid biosynthesis|Metabolism--Metabolism of cofactors and vitamins--Biotin metabolism|Metabolism--Global and overview maps--Metabolic pathways|Metabolism--Global and overview maps--Fatty acid metabolism

[denovogenes]_100264	76.56	2e-157	bfb:VU15_03045	K12373	HEXA_B	hexosaminidase	3.2.1.52	Metabolism--Glycan biosynthesis and metabolism--Other glycan degradation|Metabolism--Glycan biosynthesis and metabolism--Various types of N-glycan biosynthesis|Metabolism--Carbohydrate metabolism--Amino sugar and nucleotide sugar metabolism|Metabolism--Glycan biosynthesis and metabolism--Glycosaminoglycan degradation|Metabolism--Glycan biosynthesis and metabolism--Glycosphingolipid biosynthesis - globo and isoglobo series|Metabolism--Glycan biosynthesis and metabolism--Glycosphingolipid biosynthesis - ganglio series|Metabolism--Global and overview maps--Metabolic pathways|Cellular Processes--Transport and catabolism--Lysosome

[denovogenes]_100265	100.00	0.0	ebf:D782_0449	K03569	mreB	rod shape-determining protein MreB and related proteins	--	--

[denovogenes]_100266	90.74	2e-180	pfre:RM25_2107	K15525	mshB	N-acetyl-1-D-myo-inositol-2-amino-2-deoxy-alpha-D-glucopyranoside deacetylase	3.5.1.103	--

[denovogenes]_100267	58.85	2e-93	mph:MLP_15170	K10119	msmG	raffinose/stachyose/melibiose transport system permease protein	--	Environmental Information Processing--Membrane transport--ABC transporters

[denovogenes]_100268	49.18	4e-61	pac:PPA0317	K07052	K07052	uncharacterized protein	--	--

[denovogenes]_100270	80.63	2e-142	pfre:RM25_0824	K02340	DPO3D1, holA	DNA polymerase III subunit delta	2.7.7.7	Metabolism--Nucleotide metabolism--Purine metabolism|Metabolism--Nucleotide metabolism--Pyrimidine metabolism|Metabolism--Global and overview maps--Metabolic pathways|Genetic Information Processing--Replication and repair--DNA replication|Genetic Information Processing--Replication and repair--Mismatch repair|Genetic Information Processing--Replication and repair--Homologous recombination

[denovogenes]_100272	97.79	2e-162	pfre:RM25_0537	K02982	RP-S3, rpsC	small subunit ribosomal protein S3	--	Genetic Information Processing--Translation--Ribosome

[denovogenes]_100274	80.51	4e-163	pfr:PFREUD_01580	K00014	aroE	shikimate dehydrogenase	1.1.1.25	Metabolism--Amino acid metabolism--Phenylalanine, tyrosine and tryptophan biosynthesis|Metabolism--Global and overview maps--Metabolic pathways|Metabolism--Global and overview maps--Biosynthesis of secondary metabolites|Metabolism--Global and overview maps--Biosynthesis of antibiotics|Metabolism--Global and overview maps--Biosynthesis of amino acids

[denovogenes]_100277	94.12	3e-176	pfre:RM25_0725	K01776	murI	glutamate racemase	5.1.1.3	Metabolism--Metabolism of other amino acids--D-Glutamine and D-glutamate metabolism|Metabolism--Global and overview maps--Metabolic pathways

[denovogenes]_100279	41.10	3e-58	eay:EAM_2167	K16702	amsE	amylovoran biosynthesis glycosyltransferase AmsE	2.4.-.-	--

[denovogenes]_10028	45.78	0.0	fpla:A4U99_16975	K08303	K08303	putative protease	3.4.-.-	Human Diseases--Infectious diseases: Bacterial--Epithelial cell signaling in Helicobacter pylori infection

[denovogenes]_100281	99.63	0.0	cfd:CFNIH1_11825	K05818	mhpR	IclR family transcriptional regulator, mhp operon transcriptional activator	--	--

[denovogenes]_100282	100.00	0.0	paeo:M801_299	K12251	aguB	N-carbamoylputrescine amidase	3.5.1.53	Metabolism--Amino acid metabolism--Arginine and proline metabolism|Metabolism--Global and overview maps--Metabolic pathways

[denovogenes]_100283	100.00	0.0	paeo:M801_288	K02046	cysU	sulfate transport system permease protein	--	Metabolism--Energy metabolism--Sulfur metabolism|Environmental Information Processing--Membrane transport--ABC transporters

[denovogenes]_100284	74.26	1e-149	eha:Ethha_1878	K03496	parA, soj	chromosome partitioning protein	--	--

[denovogenes]_100285	44.69	3e-82	bvu:BVU_2953	K07271	licD	lipopolysaccharide cholinephosphotransferase	2.7.8.-	--

[denovogenes]_100288	51.87	5e-101	fia:NA23_06155	K00615	E2.2.1.1, tktA, tktB	transketolase	2.2.1.1	Metabolism--Carbohydrate metabolism--Pentose phosphate pathway|Metabolism--Energy metabolism--Carbon fixation in photosynthetic organisms|Metabolism--Metabolism of terpenoids and polyketides--Biosynthesis of ansamycins|Metabolism--Global and overview maps--Metabolic pathways|Metabolism--Global and overview maps--Biosynthesis of secondary metabolites|Metabolism--Global and overview maps--Microbial metabolism in diverse environments|Metabolism--Global and overview maps--Biosynthesis of antibiotics|Metabolism--Global and overview maps--Carbon metabolism|Metabolism--Global and overview maps--Biosynthesis of amino acids

[denovogenes]_10029	44.32	1e-89	dai:Desaci_1889	K02688	prpR	transcriptional regulator, propionate catabolism operon regulatory protein	--	--

[denovogenes]_100290	100.00	0.0	pael:T223_05435	K03769	ppiC	peptidyl-prolyl cis-trans isomerase C	5.2.1.8	--

[denovogenes]_100293	89.71	1e-174	bfg:BF638R_1676	K01082	cysQ, MET22, BPNT1	3'(2'), 5'-bisphosphate nucleotidase	3.1.3.7	Metabolism--Energy metabolism--Sulfur metabolism|Metabolism--Global and overview maps--Metabolic pathways|Metabolism--Global and overview maps--Microbial metabolism in diverse environments|Metabolism--Global and overview maps--Biosynthesis of antibiotics

[denovogenes]_100294	44.78	1e-78	aoe:Clos_1349	K02034	ABC.PE.P1	peptide/nickel transport system permease protein	--	Cellular Processes--Cellular community - prokaryotes--Quorum sensing

[denovogenes]_100295	87.50	3e-168	pdi:BDI_2592	K02040	pstS	phosphate transport system substrate-binding protein	--	Environmental Information Processing--Membrane transport--ABC transporters|Environmental Information Processing--Signal transduction--Two-component system|Human Diseases--Infectious diseases: Bacterial--Tuberculosis

[denovogenes]_100296	100.00	0.0	paeo:M801_3931	K15552	tauC	taurine transport system permease protein	--	Metabolism--Energy metabolism--Sulfur metabolism|Environmental Information Processing--Membrane transport--ABC transporters

[denovogenes]_100298	69.14	1e-117	dvl:Dvul_2054	K00941	thiD	hydroxymethylpyrimidine/phosphomethylpyrimidine kinase	2.7.1.49 2.7.4.7	Metabolism--Metabolism of cofactors and vitamins--Thiamine metabolism|Metabolism--Global and overview maps--Metabolic pathways

[denovogenes]_1003	77.06	0.0	pfr:PFREUD_15640	K14162	dnaE2	error-prone DNA polymerase	2.7.7.7	--

[denovogenes]_10030	100.00	0.0	bdh:GV66_16985	K00527	nrdD	ribonucleoside-triphosphate reductase	1.17.4.2	Metabolism--Nucleotide metabolism--Purine metabolism|Metabolism--Nucleotide metabolism--Pyrimidine metabolism|Metabolism--Global and overview maps--Metabolic pathways

[denovogenes]_100302	79.12	3e-149	pdi:BDI_2635	K03787	surE	5'-nucleotidase	3.1.3.5	Metabolism--Nucleotide metabolism--Purine metabolism|Metabolism--Nucleotide metabolism--Pyrimidine metabolism|Metabolism--Metabolism of cofactors and vitamins--Nicotinate and nicotinamide metabolism|Metabolism--Global and overview maps--Metabolic pathways|Metabolism--Global and overview maps--Biosynthesis of secondary metabolites

[denovogenes]_100303	99.63	0.0	paec:M802_5139	K03651	cpdA	3',5'-cyclic-AMP phosphodiesterase	3.1.4.53	Metabolism--Nucleotide metabolism--Purine metabolism|Cellular Processes--Cellular community - prokaryotes--Biofilm formation - Pseudomonas aeruginosa

[denovogenes]_100304	73.13	6e-143	fpa:FPR_11090	K16785	ecfT	energy-coupling factor transport system permease protein	--	Environmental Information Processing--Membrane transport--ABC transporters

[denovogenes]_100305	45.93	2e-71	tlt:OCC_03968	K16786	ecfA1	energy-coupling factor transport system ATP-binding protein	3.6.3.-	Environmental Information Processing--Membrane transport--ABC transporters

[denovogenes]_100305	45.93	2e-71	tlt:OCC_03968	K16787	ecfA2	energy-coupling factor transport system ATP-binding protein	3.6.3.-	Environmental Information Processing--Membrane transport--ABC transporters

[denovogenes]_100308	47.73	4e-83	elo:EC042_0332	K06221	dkgA	2,5-diketo-D-gluconate reductase A	1.1.1.346	--

[denovogenes]_10031	51.58	0.0	tto:Thethe_01165	K01915	glnA, GLUL	glutamine synthetase	6.3.1.2	Metabolism--Amino acid metabolism--Arginine biosynthesis|Metabolism--Amino acid metabolism--Alanine, aspartate and glutamate metabolism|Metabolism--Carbohydrate metabolism--Glyoxylate and dicarboxylate metabolism|Metabolism--Energy metabolism--Nitrogen metabolism|Metabolism--Global and overview maps--Metabolic pathways|Metabolism--Global and overview maps--Microbial metabolism in diverse environments|Metabolism--Global and overview maps--Biosynthesis of amino acids|Environmental Information Processing--Signal transduction--Two-component system|Organismal Systems--Nervous system--Glutamatergic synapse|Organismal Systems--Nervous system--GABAergic synapse

[denovogenes]_100315	98.53	0.0	cfd:CFNIH1_16970	K02028	ABC.PA.A	polar amino acid transport system ATP-binding protein	3.6.3.21	--

[denovogenes]_100317	49.26	2e-94	esi:Exig_2824	K06221	dkgA	2,5-diketo-D-gluconate reductase A	1.1.1.346	--

[denovogenes]_100318	97.34	0.0	bfg:BF638R_0281	K11717	sufS	cysteine desulfurase / selenocysteine lyase	2.8.1.7 4.4.1.16	Metabolism--Metabolism of other amino acids--Selenocompound metabolism|Metabolism--Global and overview maps--Metabolic pathways

[denovogenes]_100319	40.52	2e-19	tgy:X802_01400	K01126	E3.1.4.46, glpQ, ugpQ	glycerophosphoryl diester phosphodiesterase	3.1.4.46	Metabolism--Lipid metabolism--Glycerophospholipid metabolism

[denovogenes]_10032	41.69	1e-53	scu:SCE1572_11985	K15921	xynD	arabinoxylan arabinofuranohydrolase	3.2.1.55	Metabolism--Carbohydrate metabolism--Amino sugar and nucleotide sugar metabolism

[denovogenes]_100320	46.94	4e-60	ccl:Clocl_1087	K03768	PPIB, ppiB	peptidyl-prolyl cis-trans isomerase B (cyclophilin B)	5.2.1.8	--

[denovogenes]_100321	42.97	4e-55	bbe:BBR47_16690	K13767	fadB	enoyl-CoA hydratase	4.2.1.17	Metabolism--Lipid metabolism--Fatty acid degradation|Metabolism--Xenobiotics biodegradation and metabolism--Benzoate degradation|Metabolism--Global and overview maps--Metabolic pathways|Metabolism--Global and overview maps--Microbial metabolism in diverse environments|Metabolism--Global and overview maps--Fatty acid metabolism

[denovogenes]_100322	45.72	5e-74	cbw:RR42_m2422	K16203	dppA1	D-amino peptidase	3.4.11.-	--

[denovogenes]_100325	100.00	0.0	efq:DR75_2446	K02796	PTS-Man-EIID, manZ	PTS system, mannose-specific IID component	--	Metabolism--Carbohydrate metabolism--Fructose and mannose metabolism|Metabolism--Carbohydrate metabolism--Amino sugar and nucleotide sugar metabolism|Metabolism--Global and overview maps--Metabolic pathways|Environmental Information Processing--Membrane transport--Phosphotransferase system (PTS)

[denovogenes]_100326	62.65	8e-124	pgi:PG_0425	K01070	frmB, ESD, fghA	S-formylglutathione hydrolase	3.1.2.12	Metabolism--Energy metabolism--Methane metabolism|Metabolism--Global and overview maps--Microbial metabolism in diverse environments|Metabolism--Global and overview maps--Carbon metabolism

[denovogenes]_100327	66.29	9e-127	fpa:FPR_12780	K01992	ABC-2.P	ABC-2 type transport system permease protein	--	--

[denovogenes]_100328	82.33	6e-163	spe:Spro_4278	K02771	PTS-Fru1-EIID, levG	PTS system, fructose-specific IID component	--	Metabolism--Carbohydrate metabolism--Fructose and mannose metabolism|Environmental Information Processing--Membrane transport--Phosphotransferase system (PTS)

[denovogenes]_10033	94.69	0.0	sly:101245299	K00615	E2.2.1.1, tktA, tktB	transketolase	2.2.1.1	Metabolism--Carbohydrate metabolism--Pentose phosphate pathway|Metabolism--Energy metabolism--Carbon fixation in photosynthetic organisms|Metabolism--Metabolism of terpenoids and polyketides--Biosynthesis of ansamycins|Metabolism--Global and overview maps--Metabolic pathways|Metabolism--Global and overview maps--Biosynthesis of secondary metabolites|Metabolism--Global and overview maps--Microbial metabolism in diverse environments|Metabolism--Global and overview maps--Biosynthesis of antibiotics|Metabolism--Global and overview maps--Carbon metabolism|Metabolism--Global and overview maps--Biosynthesis of amino acids

[denovogenes]_100330	99.26	0.0	cfd:CFNIH1_02490	K06153	bacA	undecaprenyl-diphosphatase	3.6.1.27	Metabolism--Glycan biosynthesis and metabolism--Peptidoglycan biosynthesis

[denovogenes]_100331	100.00	0.0	fpla:A4U99_09625	K06442	tlyA	23S rRNA (cytidine1920-2'-O)/16S rRNA (cytidine1409-2'-O)-methyltransferase	2.1.1.226 2.1.1.227	--

[denovogenes]_100333	100.00	0.0	paem:U769_03110	K01091	gph	phosphoglycolate phosphatase	3.1.3.18	Metabolism--Carbohydrate metabolism--Glyoxylate and dicarboxylate metabolism|Metabolism--Global and overview maps--Metabolic pathways|Metabolism--Global and overview maps--Biosynthesis of secondary metabolites|Metabolism--Global and overview maps--Biosynthesis of antibiotics

[denovogenes]_100336	99.26	0.0	psg:G655_27010	K07052	K07052	uncharacterized protein	--	--

[denovogenes]_100338	67.65	1e-142	baci:B1NLA3E_21025	K00615	E2.2.1.1, tktA, tktB	transketolase	2.2.1.1	Metabolism--Carbohydrate metabolism--Pentose phosphate pathway|Metabolism--Energy metabolism--Carbon fixation in photosynthetic organisms|Metabolism--Metabolism of terpenoids and polyketides--Biosynthesis of ansamycins|Metabolism--Global and overview maps--Metabolic pathways|Metabolism--Global and overview maps--Biosynthesis of secondary metabolites|Metabolism--Global and overview maps--Microbial metabolism in diverse environments|Metabolism--Global and overview maps--Biosynthesis of antibiotics|Metabolism--Global and overview maps--Carbon metabolism|Metabolism--Global and overview maps--Biosynthesis of amino acids

[denovogenes]_10034	56.98	0.0	seep:I137_12925	K20276	bapA	large repetitive protein	--	Cellular Processes--Cellular community - prokaryotes--Quorum sensing

[denovogenes]_100341	48.72	9e-87	str:Sterm_2098	K02795	PTS-Man-EIIC, manY	PTS system, mannose-specific IIC component	--	Metabolism--Carbohydrate metabolism--Fructose and mannose metabolism|Metabolism--Carbohydrate metabolism--Amino sugar and nucleotide sugar metabolism|Metabolism--Global and overview maps--Metabolic pathways|Environmental Information Processing--Membrane transport--Phosphotransferase system (PTS)

[denovogenes]_100342	82.53	5e-163	fpr:FP2_09410	K03205	virD4, lvhD4	type IV secretion system protein VirD4	--	Environmental Information Processing--Membrane transport--Bacterial secretion system

[denovogenes]_100344	44.65	2e-69	bacc:BRDCF_p935	K03768	PPIB, ppiB	peptidyl-prolyl cis-trans isomerase B (cyclophilin B)	5.2.1.8	--

[denovogenes]_100345	98.90	0.0	cfd:CFNIH1_19720	K01297	ldcA	muramoyltetrapeptide carboxypeptidase	3.4.17.13	--

[denovogenes]_100347	60.29	2e-116	pdi:BDI_1896	K05810	yfiH	laccase domain-containing protein	--	--

[denovogenes]_100348	70.74	4e-138	lacy:A4V08_29825	K03079	ulaE, sgaU	L-ribulose-5-phosphate 3-epimerase	5.1.3.22	Metabolism--Carbohydrate metabolism--Ascorbate and aldarate metabolism|Metabolism--Global and overview maps--Metabolic pathways|Metabolism--Global and overview maps--Microbial metabolism in diverse environments

[denovogenes]_100349	53.53	1e-111	ruk:A4V00_07330	K03437	spoU	RNA methyltransferase, TrmH family	--	--

[denovogenes]_10035	89.94	0.0	pfr:PFREUD_06230	K02355	fusA, GFM, EFG	elongation factor G	--	--

[denovogenes]_100350	72.24	1e-151	rto:RTO_13780	K19509	PTS-Gfr-EIID, gfrD	PTS system, fructoselysine/glucoselysine-specific IID component	--	Environmental Information Processing--Membrane transport--Phosphotransferase system (PTS)

[denovogenes]_100352	100.00	0.0	paem:U769_16870	K09773	ppsR	[pyruvate, water dikinase]-phosphate phosphotransferase / [pyruvate, water dikinase] kinase	2.7.4.28 2.7.11.33	--

[denovogenes]_100355	100.00	0.0	paeo:M801_2020	K06153	bacA	undecaprenyl-diphosphatase	3.6.1.27	Metabolism--Glycan biosynthesis and metabolism--Peptidoglycan biosynthesis

[denovogenes]_100357	100.00	0.0	bbrd:BBBR_1543	K00048	fucO	lactaldehyde reductase	1.1.1.77	Metabolism--Carbohydrate metabolism--Glyoxylate and dicarboxylate metabolism|Metabolism--Carbohydrate metabolism--Propanoate metabolism|Metabolism--Global and overview maps--Microbial metabolism in diverse environments

[denovogenes]_100358	59.69	8e-114	csh:Closa_0816	K02190	cbiK	sirohydrochlorin cobaltochelatase	4.99.1.3	Metabolism--Metabolism of cofactors and vitamins--Porphyrin and chlorophyll metabolism|Metabolism--Global and overview maps--Metabolic pathways

[denovogenes]_100359	43.72	9e-65	paeh:H70357_08235	K16868	tehB	tellurite methyltransferase	2.1.1.265	--

[denovogenes]_10036	100.00	0.0	cfd:CFNIH1_10855	K02400	flhA	flagellar biosynthesis protein FlhA	--	Cellular Processes--Cell motility--Flagellar assembly

[denovogenes]_100362	99.63	0.0	cif:AL515_17320	K03595	era	GTPase	--	--

[denovogenes]_100363	72.43	9e-149	byl:A4V09_00025	K01235	aguA	alpha-glucuronidase	3.2.1.139	--

[denovogenes]_100365	45.49	2e-67	tng:GSTEN00027603G001	K05850	ATP2B	Ca2+ transporting ATPase, plasma membrane	3.6.3.8	Environmental Information Processing--Signal transduction--Calcium signaling pathway|Environmental Information Processing--Signal transduction--cGMP-PKG signaling pathway|Environmental Information Processing--Signal transduction--cAMP signaling pathway|Organismal Systems--Circulatory system--Adrenergic signaling in cardiomyocytes|Organismal Systems--Digestive system--Salivary secretion|Organismal Systems--Digestive system--Pancreatic secretion

[denovogenes]_100366	73.53	1e-157	xpo:XPG1_1367	K10906	recE	exodeoxyribonuclease VIII	3.1.11.-	--

[denovogenes]_100367	43.12	4e-69	bsa:Bacsa_1396	K03768	PPIB, ppiB	peptidyl-prolyl cis-trans isomerase B (cyclophilin B)	5.2.1.8	--

[denovogenes]_100369	56.85	2e-115	erb:A4V01_13955	K01990	ABC-2.A	ABC-2 type transport system ATP-binding protein	--	--

[denovogenes]_100371	40.83	8e-23	tig:THII_0937	K20974	K20974	two-component system, sensor histidine kinase	2.7.13.3	Environmental Information Processing--Signal transduction--Two-component system|Cellular Processes--Cellular community - prokaryotes--Biofilm formation - Pseudomonas aeruginosa

[denovogenes]_100373	85.66	7e-172	csh:Closa_0072	K00615	E2.2.1.1, tktA, tktB	transketolase	2.2.1.1	Metabolism--Carbohydrate metabolism--Pentose phosphate pathway|Metabolism--Energy metabolism--Carbon fixation in photosynthetic organisms|Metabolism--Metabolism of terpenoids and polyketides--Biosynthesis of ansamycins|Metabolism--Global and overview maps--Metabolic pathways|Metabolism--Global and overview maps--Biosynthesis of secondary metabolites|Metabolism--Global and overview maps--Microbial metabolism in diverse environments|Metabolism--Global and overview maps--Biosynthesis of antibiotics|Metabolism--Global and overview maps--Carbon metabolism|Metabolism--Global and overview maps--Biosynthesis of amino acids

[denovogenes]_100374	99.26	0.0	pdi:BDI_2022	K01586	lysA	diaminopimelate decarboxylase	4.1.1.20	Metabolism--Amino acid metabolism--Lysine biosynthesis|Metabolism--Global and overview maps--Metabolic pathways|Metabolism--Global and overview maps--Biosynthesis of secondary metabolites|Metabolism--Global and overview maps--Microbial metabolism in diverse environments|Metabolism--Global and overview maps--Biosynthesis of antibiotics|Metabolism--Global and overview maps--Biosynthesis of amino acids

[denovogenes]_100375	40.76	6e-53	sgp:SpiGrapes_1524	K00848	rhaB	rhamnulokinase	2.7.1.5	Metabolism--Carbohydrate metabolism--Pentose and glucuronate interconversions|Metabolism--Carbohydrate metabolism--Fructose and mannose metabolism|Metabolism--Global and overview maps--Microbial metabolism in diverse environments

[denovogenes]_100378	99.63	0.0	paem:U769_04015	K06222	dkgB	2,5-diketo-D-gluconate reductase B	1.1.1.346	--

[denovogenes]_100379	40.83	3e-16	pca:Pcar_0611	K03148	thiF	sulfur carrier protein ThiS adenylyltransferase	2.7.7.73	Metabolism--Metabolism of cofactors and vitamins--Thiamine metabolism|Metabolism--Global and overview maps--Metabolic pathways|Genetic Information Processing--Folding, sorting and degradation--Sulfur relay system

[denovogenes]_10038	57.54	0.0	ral:Rumal_1648	K03798	ftsH, hflB	cell division protease FtsH	3.4.24.-	--

[denovogenes]_100381	50.00	6e-37	wjo:FOL01_0729	K03930	estA	putative tributyrin esterase	3.1.1.-	--

[denovogenes]_100382	74.26	4e-155	euu:ADJ67_01795	K00615	E2.2.1.1, tktA, tktB	transketolase	2.2.1.1	Metabolism--Carbohydrate metabolism--Pentose phosphate pathway|Metabolism--Energy metabolism--Carbon fixation in photosynthetic organisms|Metabolism--Metabolism of terpenoids and polyketides--Biosynthesis of ansamycins|Metabolism--Global and overview maps--Metabolic pathways|Metabolism--Global and overview maps--Biosynthesis of secondary metabolites|Metabolism--Global and overview maps--Microbial metabolism in diverse environments|Metabolism--Global and overview maps--Biosynthesis of antibiotics|Metabolism--Global and overview maps--Carbon metabolism|Metabolism--Global and overview maps--Biosynthesis of amino acids

[denovogenes]_100383	67.17	6e-126	cle:Clole_3217	K03458	TC.NCS2	nucleobase:cation symporter-2, NCS2 family	--	--

[denovogenes]_100384	100.00	0.0	ecol:LY180_16905	K00014	aroE	shikimate dehydrogenase	1.1.1.25	Metabolism--Amino acid metabolism--Phenylalanine, tyrosine and tryptophan biosynthesis|Metabolism--Global and overview maps--Metabolic pathways|Metabolism--Global and overview maps--Biosynthesis of secondary metabolites|Metabolism--Global and overview maps--Biosynthesis of antibiotics|Metabolism--Global and overview maps--Biosynthesis of amino acids

[denovogenes]_100385	75.28	9e-155	lacy:A4V08_24930	K00018	hprA	glycerate dehydrogenase	1.1.1.29	Metabolism--Amino acid metabolism--Glycine, serine and threonine metabolism|Metabolism--Carbohydrate metabolism--Glyoxylate and dicarboxylate metabolism|Metabolism--Energy metabolism--Methane metabolism|Metabolism--Global and overview maps--Metabolic pathways|Metabolism--Global and overview maps--Biosynthesis of secondary metabolites|Metabolism--Global and overview maps--Microbial metabolism in diverse environments|Metabolism--Global and overview maps--Biosynthesis of antibiotics|Metabolism--Global and overview maps--Carbon metabolism

[denovogenes]_100386	97.79	0.0	cama:F384_17205	K02026	ABC.MS.P1	multiple sugar transport system permease protein	--	--

[denovogenes]_100387	85.87	5e-102	lacy:A4V08_08380	K03625	nusB	N utilization substance protein B	--	--

[denovogenes]_100389	40.44	2e-63	ccl:Clocl_1786	K13542	cobA-hemD	uroporphyrinogen III methyltransferase / synthase	2.1.1.107 4.2.1.75	Metabolism--Metabolism of cofactors and vitamins--Porphyrin and chlorophyll metabolism|Metabolism--Global and overview maps--Metabolic pathways|Metabolism--Global and overview maps--Biosynthesis of secondary metabolites

[denovogenes]_100394	98.13	0.0	lacy:A4V08_15500	K07258	dacC, dacA, dacD	D-alanyl-D-alanine carboxypeptidase (penicillin-binding protein 5/6)	3.4.16.4	Metabolism--Glycan biosynthesis and metabolism--Peptidoglycan biosynthesis|Metabolism--Global and overview maps--Metabolic pathways

[denovogenes]_100396	67.51	2e-112	sbu:SpiBuddy_0342	K10241	cebF	cellobiose transport system permease protein	--	Environmental Information Processing--Membrane transport--ABC transporters

[denovogenes]_100397	100.00	0.0	paeo:M801_2347	K13979	yahK	uncharacterized zinc-type alcohol dehydrogenase-like protein	1.-.-.-	--

[denovogenes]_100399	91.18	0.0	pdi:BDI_2435	K03595	era	GTPase	--	--

[denovogenes]_100401	75.82	1e-157	byl:A4V09_00090	K01206	FUCA	alpha-L-fucosidase	3.2.1.51	Metabolism--Glycan biosynthesis and metabolism--Other glycan degradation

[denovogenes]_100405	97.43	0.0	lacy:A4V08_25220	K03484	scrR	LacI family transcriptional regulator, sucrose operon repressor	--	--

[denovogenes]_100407	89.91	3e-148	bprs:CK3_04800	K07497	K07497	putative transposase	--	--

[denovogenes]_100409	99.25	0.0	ecos:EC958_2409	K04046	yegD	hypothetical chaperone protein	--	--

[denovogenes]_10041	42.04	0.0	cdg:CDBI1_06430	K06978	K06978	uncharacterized protein	--	--

[denovogenes]_100413	63.08	8e-102	csh:Closa_0780	K04088	hflK	membrane protease subunit HflK	3.4.-.-	--

[denovogenes]_100416	86.03	4e-178	pdi:BDI_2390	K03771	surA	peptidyl-prolyl cis-trans isomerase SurA	5.2.1.8	--

[denovogenes]_100418	97.79	0.0	ebw:BWG_1830	K00067	rfbD, rmlD	dTDP-4-dehydrorhamnose reductase	1.1.1.133	Metabolism--Biosynthesis of other secondary metabolites--Streptomycin biosynthesis|Metabolism--Metabolism of terpenoids and polyketides--Polyketide sugar unit biosynthesis|Metabolism--Global and overview maps--Biosynthesis of antibiotics

[denovogenes]_100421	50.39	1e-85	shi:Shel_21260	K03734	apbE	FAD:protein FMN transferase	2.7.1.180	--

[denovogenes]_100423	92.65	0.0	pvl:AOB99_08715	K06131	clsA_B	cardiolipin synthase A/B	2.7.8.-	Metabolism--Lipid metabolism--Glycerophospholipid metabolism|Metabolism--Global and overview maps--Metabolic pathways

[denovogenes]_100424	72.66	9e-148	rim:ROI_16840	K02529	lacI, galR	LacI family transcriptional regulator	--	--

[denovogenes]_100426	95.22	0.0	cfd:CFNIH1_19525	K02493	hemK, prmC, HEMK	release factor glutamine methyltransferase	2.1.1.297	--

[denovogenes]_100429	89.50	4e-159	bcel:BcellWH2_02478	K03685	rnc, DROSHA, RNT1	ribonuclease III	3.1.26.3	Genetic Information Processing--Translation--Ribosome biogenesis in eukaryotes|Human Diseases--Cancers: Overview--Proteoglycans in cancer

[denovogenes]_100431	87.08	5e-170	lacy:A4V08_32675	K01572	oadB	oxaloacetate decarboxylase, beta subunit	4.1.1.3	Metabolism--Carbohydrate metabolism--Pyruvate metabolism|Metabolism--Global and overview maps--Metabolic pathways

[denovogenes]_100433	43.12	4e-64	paa:Paes_0086	K00986	E2.7.7.49	RNA-directed DNA polymerase	2.7.7.49	--

[denovogenes]_100434	84.98	3e-172	byl:A4V09_04390	K01755	argH, ASL	argininosuccinate lyase	4.3.2.1	Metabolism--Amino acid metabolism--Arginine biosynthesis|Metabolism--Amino acid metabolism--Alanine, aspartate and glutamate metabolism|Metabolism--Global and overview maps--Metabolic pathways|Metabolism--Global and overview maps--Biosynthesis of secondary metabolites|Metabolism--Global and overview maps--Biosynthesis of antibiotics|Metabolism--Global and overview maps--Biosynthesis of amino acids

[denovogenes]_100436	47.60	1e-77	byl:A4V09_22945	K07718	yesM	two-component system, sensor histidine kinase YesM	2.7.13.3	Environmental Information Processing--Signal transduction--Two-component system

[denovogenes]_100437	99.63	0.0	pdi:BDI_0592	K01696	trpB	tryptophan synthase beta chain	4.2.1.20	Metabolism--Amino acid metabolism--Glycine, serine and threonine metabolism|Metabolism--Amino acid metabolism--Phenylalanine, tyrosine and tryptophan biosynthesis|Metabolism--Global and overview maps--Metabolic pathways|Metabolism--Global and overview maps--Biosynthesis of secondary metabolites|Metabolism--Global and overview maps--Biosynthesis of antibiotics|Metabolism--Global and overview maps--Biosynthesis of amino acids

[denovogenes]_100438	95.24	0.0	lacy:A4V08_18550	K00005	gldA	glycerol dehydrogenase	1.1.1.6	Metabolism--Lipid metabolism--Glycerolipid metabolism|Metabolism--Carbohydrate metabolism--Propanoate metabolism|Metabolism--Global and overview maps--Metabolic pathways

[denovogenes]_10044	97.99	0.0	bfb:VU15_04185	K01278	DPP4, CD26	dipeptidyl-peptidase 4	3.4.14.5	Organismal Systems--Digestive system--Protein digestion and absorption

[denovogenes]_100443	91.58	0.0	pvl:AOB99_12250	K00052	leuB	3-isopropylmalate dehydrogenase	1.1.1.85	Metabolism--Amino acid metabolism--Valine, leucine and isoleucine biosynthesis|Metabolism--Carbohydrate metabolism--C5-Branched dibasic acid metabolism|Metabolism--Global and overview maps--Metabolic pathways|Metabolism--Global and overview maps--Biosynthesis of secondary metabolites|Metabolism--Global and overview maps--2-Oxocarboxylic acid metabolism|Metabolism--Global and overview maps--Biosynthesis of amino acids

[denovogenes]_100445	82.05	1e-151	rto:RTO_25350	K03703	uvrC	excinuclease ABC subunit C	--	Genetic Information Processing--Replication and repair--Nucleotide excision repair

[denovogenes]_100450	76.56	2e-143	rim:ROI_21440	K03763	DPO3A2, polC	DNA polymerase III subunit alpha, Gram-positive type	2.7.7.7	Metabolism--Nucleotide metabolism--Purine metabolism|Metabolism--Nucleotide metabolism--Pyrimidine metabolism|Metabolism--Global and overview maps--Metabolic pathways|Genetic Information Processing--Replication and repair--DNA replication|Genetic Information Processing--Replication and repair--Mismatch repair|Genetic Information Processing--Replication and repair--Homologous recombination

[denovogenes]_100451	94.51	2e-179	lacy:A4V08_34330	K03699	tlyC	putative hemolysin	--	--

[denovogenes]_100453	41.36	1e-25	meso:BSQ44_06170	K11927	rhlE	ATP-dependent RNA helicase RhlE	3.6.4.13	Genetic Information Processing--Folding, sorting and degradation--RNA degradation

[denovogenes]_100454	73.63	3e-143	afd:Alfi_2530	K02316	dnaG	DNA primase	2.7.7.-	Genetic Information Processing--Replication and repair--DNA replication

[denovogenes]_10046	53.67	0.0	pmw:B2K_10390	K01811	xylS, yicI	alpha-D-xyloside xylohydrolase	3.2.1.177	--

[denovogenes]_100460	73.43	5e-152	sele:ADJ74_02585	K10206	E2.6.1.83	LL-diaminopimelate aminotransferase	2.6.1.83	Metabolism--Amino acid metabolism--Lysine biosynthesis|Metabolism--Global and overview maps--Metabolic pathways|Metabolism--Global and overview maps--Biosynthesis of secondary metabolites|Metabolism--Global and overview maps--Biosynthesis of antibiotics|Metabolism--Global and overview maps--Biosynthesis of amino acids

[denovogenes]_100466	61.85	1e-121	pgi:PG_0425	K01070	frmB, ESD, fghA	S-formylglutathione hydrolase	3.1.2.12	Metabolism--Energy metabolism--Methane metabolism|Metabolism--Global and overview maps--Microbial metabolism in diverse environments|Metabolism--Global and overview maps--Carbon metabolism

[denovogenes]_100471	42.86	1e-26	mro:MROS_0358	K02477	K02477	two-component system, LytT family, response regulator	--	--

[denovogenes]_100482	83.82	6e-163	vpr:Vpar_1646	K02073	metQ	D-methionine transport system substrate-binding protein	--	Environmental Information Processing--Membrane transport--ABC transporters

[denovogenes]_100483	56.99	8e-96	ipo:Ilyop_1858	K01929	murF	UDP-N-acetylmuramoyl-tripeptide--D-alanyl-D-alanine ligase	6.3.2.10	Metabolism--Amino acid metabolism--Lysine biosynthesis|Metabolism--Glycan biosynthesis and metabolism--Peptidoglycan biosynthesis|Metabolism--Global and overview maps--Metabolic pathways|Human Diseases--Drug resistance: Antimicrobial--Vancomycin resistance

[denovogenes]_100484	73.09	4e-133	gfe:Gferi_09720	K02106	atoE	short-chain fatty acids transporter	--	Environmental Information Processing--Signal transduction--Two-component system

[denovogenes]_100485	68.00	1e-131	fhw:RN87_06395	K03074	secF	preprotein translocase subunit SecF	--	Genetic Information Processing--Folding, sorting and degradation--Protein export|Environmental Information Processing--Membrane transport--Bacterial secretion system

[denovogenes]_100487	94.85	0.0	pge:LG71_05475	K10110	malG	maltose/maltodextrin transport system permease protein	--	Environmental Information Processing--Membrane transport--ABC transporters

[denovogenes]_100490	100.00	0.0	pvl:AOB99_07465	K07139	K07139	uncharacterized protein	--	--

[denovogenes]_100496	86.03	5e-171	ehr:EHR_01490	K02026	ABC.MS.P1	multiple sugar transport system permease protein	--	--

[denovogenes]_100498	100.00	0.0	sft:NCTC1_03042	K03087	SIG2, rpoS	RNA polymerase nonessential primary-like sigma factor	--	Cellular Processes--Cellular community - prokaryotes--Biofilm formation - Escherichia coli|Cellular Processes--Cellular community - prokaryotes--Biofilm formation - Vibrio cholerae

[denovogenes]_1005	99.18	0.0	bfb:VU15_03025	K01190	lacZ	beta-galactosidase	3.2.1.23	Metabolism--Carbohydrate metabolism--Galactose metabolism|Metabolism--Glycan biosynthesis and metabolism--Other glycan degradation|Metabolism--Lipid metabolism--Sphingolipid metabolism|Metabolism--Global and overview maps--Metabolic pathways

[denovogenes]_10050	99.43	0.0	bbre:B12L_0067	K01278	DPP4, CD26	dipeptidyl-peptidase 4	3.4.14.5	Organismal Systems--Digestive system--Protein digestion and absorption

[denovogenes]_100500	100.00	0.0	ecos:EC958_4540	K14393	actP	cation/acetate symporter	--	--

[denovogenes]_100504	91.54	0.0	fnt:HMPREF0405_01546	K01878	glyQ	glycyl-tRNA synthetase alpha chain	6.1.1.14	Genetic Information Processing--Translation--Aminoacyl-tRNA biosynthesis

[denovogenes]_100505	62.61	1e-46	cbj:H04402_01693	K00548	metH, MTR	5-methyltetrahydrofolate--homocysteine methyltransferase	2.1.1.13	Metabolism--Amino acid metabolism--Cysteine and methionine metabolism|Metabolism--Metabolism of other amino acids--Selenocompound metabolism|Metabolism--Metabolism of cofactors and vitamins--One carbon pool by folate|Metabolism--Global and overview maps--Metabolic pathways|Metabolism--Global and overview maps--Biosynthesis of secondary metabolites|Metabolism--Global and overview maps--Biosynthesis of amino acids

[denovogenes]_100508	53.68	2e-96	lli:uc509_p6028	K01183	E3.2.1.14	chitinase	3.2.1.14	Metabolism--Carbohydrate metabolism--Amino sugar and nucleotide sugar metabolism|Metabolism--Global and overview maps--Metabolic pathways

[denovogenes]_100509	61.42	1e-116	saf:SULAZ_1709	K01869	LARS, leuS	leucyl-tRNA synthetase	6.1.1.4	Genetic Information Processing--Translation--Aminoacyl-tRNA biosynthesis

[denovogenes]_10051	100.00	0.0	paer:PA1R_gp5396	K00372	nasA	assimilatory nitrate reductase catalytic subunit	1.7.99.4	Metabolism--Energy metabolism--Nitrogen metabolism|Metabolism--Global and overview maps--Microbial metabolism in diverse environments

[denovogenes]_100511	100.00	0.0	pmib:BB2000_0221	K03311	TC.LIVCS	branched-chain amino acid:cation transporter, LIVCS family	--	--

[denovogenes]_100513	66.29	3e-63	ecos:EC958_2297	K13735	yeeJ	adhesin/invasin	--	Human Diseases--Infectious diseases: Bacterial--Bacterial invasion of epithelial cells

[denovogenes]_100514	64.47	1e-118	sfw:WN53_06685	K02790	PTS-MalGlc-EIIB, malX	PTS system, maltose/glucose-specific IIB component	2.7.1.199 2.7.1.208	Metabolism--Carbohydrate metabolism--Glycolysis / Gluconeogenesis|Metabolism--Carbohydrate metabolism--Starch and sucrose metabolism|Metabolism--Carbohydrate metabolism--Amino sugar and nucleotide sugar metabolism|Environmental Information Processing--Membrane transport--Phosphotransferase system (PTS)

[denovogenes]_100514	64.47	1e-118	sfw:WN53_06685	K02791	PTS-MalGlc-EIIC, malX	PTS system, maltose/glucose-specific IIC component	--	Metabolism--Carbohydrate metabolism--Glycolysis / Gluconeogenesis|Metabolism--Carbohydrate metabolism--Starch and sucrose metabolism|Metabolism--Carbohydrate metabolism--Amino sugar and nucleotide sugar metabolism|Environmental Information Processing--Membrane transport--Phosphotransferase system (PTS)

[denovogenes]_100518	71.53	2e-142	cpro:CPRO_07070	K03701	uvrA	excinuclease ABC subunit A	--	Genetic Information Processing--Replication and repair--Nucleotide excision repair

[denovogenes]_10052	42.71	0.0	ana:alr7295	K06148	ABCC-BAC	ATP-binding cassette, subfamily C, bacterial	--	--

[denovogenes]_100520	99.63	0.0	mmk:MU9_2618	K02073	metQ	D-methionine transport system substrate-binding protein	--	Environmental Information Processing--Membrane transport--ABC transporters

[denovogenes]_100522	72.49	2e-142	efm:M7W_1573	K00941	thiD	hydroxymethylpyrimidine/phosphomethylpyrimidine kinase	2.7.1.49 2.7.4.7	Metabolism--Metabolism of cofactors and vitamins--Thiamine metabolism|Metabolism--Global and overview maps--Metabolic pathways

[denovogenes]_100524	75.28	2e-142	ega:AL523_15215	K06442	tlyA	23S rRNA (cytidine1920-2'-O)/16S rRNA (cytidine1409-2'-O)-methyltransferase	2.1.1.226 2.1.1.227	--

[denovogenes]_100525	48.71	2e-79	ova:OBV_01290	K07705	lytT, lytR	two-component system, LytT family, response regulator LytT	--	Environmental Information Processing--Signal transduction--Two-component system

[denovogenes]_100527	98.52	0.0	mmk:MU9_855	K00215	dapB	4-hydroxy-tetrahydrodipicolinate reductase	1.17.1.8	Metabolism--Biosynthesis of other secondary metabolites--Monobactam biosynthesis|Metabolism--Amino acid metabolism--Lysine biosynthesis|Metabolism--Global and overview maps--Metabolic pathways|Metabolism--Global and overview maps--Biosynthesis of secondary metabolites|Metabolism--Global and overview maps--Microbial metabolism in diverse environments|Metabolism--Global and overview maps--Biosynthesis of antibiotics|Metabolism--Global and overview maps--Biosynthesis of amino acids

[denovogenes]_100528	50.38	2e-93	cpy:Cphy_1635	K01992	ABC-2.P	ABC-2 type transport system permease protein	--	--

[denovogenes]_100530	81.48	1e-159	csb:CLSA_c01040	K09685	purR	purine operon repressor	--	--

[denovogenes]_100531	80.00	4e-158	cbut:ATN24_01945	K09685	purR	purine operon repressor	--	--

[denovogenes]_100532	55.36	2e-102	csb:CLSA_c12920	K02030	ABC.PA.S	polar amino acid transport system substrate-binding protein	--	--

[denovogenes]_100533	85.44	8e-161	csb:CLSA_c03560	K01715	crt	enoyl-CoA hydratase	4.2.1.17	Metabolism--Carbohydrate metabolism--Butanoate metabolism|Metabolism--Global and overview maps--Carbon metabolism

[denovogenes]_100534	67.80	6e-127	ega:AL523_07315	K02030	ABC.PA.S	polar amino acid transport system substrate-binding protein	--	--

[denovogenes]_100535	65.19	3e-138	bpsc:BBPC_1495	K02026	ABC.MS.P1	multiple sugar transport system permease protein	--	--

[denovogenes]_100536	86.84	7e-160	eft:M395_12360	K09816	znuB	zinc transport system permease protein	--	Environmental Information Processing--Membrane transport--ABC transporters

[denovogenes]_100537	58.80	3e-103	lacy:A4V08_31180	K02405	fliA	RNA polymerase sigma factor for flagellar operon FliA	--	Environmental Information Processing--Signal transduction--Two-component system|Cellular Processes--Cellular community - prokaryotes--Biofilm formation - Pseudomonas aeruginosa|Cellular Processes--Cellular community - prokaryotes--Biofilm formation - Escherichia coli|Cellular Processes--Cell motility--Flagellar assembly|Cellular Processes--Cellular community - prokaryotes--Biofilm formation - Vibrio cholerae

[denovogenes]_100538	73.61	4e-147	pdf:CD630DERM_07270	K15023	acsE	5-methyltetrahydrofolate corrinoid/iron sulfur protein methyltransferase	2.1.1.258	Metabolism--Energy metabolism--Carbon fixation pathways in prokaryotes|Metabolism--Global and overview maps--Microbial metabolism in diverse environments|Metabolism--Global and overview maps--Carbon metabolism

[denovogenes]_100539	46.00	5e-63	cdg:CDBI1_13455	K01448	E3.5.1.28B, amiA, amiB, amiC	N-acetylmuramoyl-L-alanine amidase	3.5.1.28	Human Diseases--Drug resistance: Antimicrobial--Cationic antimicrobial peptide (CAMP) resistance

[denovogenes]_10054	42.02	1e-148	pru:PRU_1453	K03701	uvrA	excinuclease ABC subunit A	--	Genetic Information Processing--Replication and repair--Nucleotide excision repair

[denovogenes]_100540	72.43	9e-134	fpr:FP2_30970	K02746	PTS-Aga-EIIC, agaW	PTS system, N-acetylgalactosamine-specific IIC component	--	Metabolism--Carbohydrate metabolism--Galactose metabolism|Environmental Information Processing--Membrane transport--Phosphotransferase system (PTS)

[denovogenes]_100544	100.00	0.0	mmk:MU9_957	K02073	metQ	D-methionine transport system substrate-binding protein	--	Environmental Information Processing--Membrane transport--ABC transporters

[denovogenes]_100545	66.42	9e-130	lacy:A4V08_13045	K09692	tagG	teichoic acid transport system permease protein	--	Environmental Information Processing--Membrane transport--ABC transporters

[denovogenes]_100546	100.00	0.0	efi:OG1RF_10037	K00931	proB	glutamate 5-kinase	2.7.2.11	Metabolism--Amino acid metabolism--Arginine and proline metabolism|Metabolism--Biosynthesis of other secondary metabolites--Carbapenem biosynthesis|Metabolism--Global and overview maps--Metabolic pathways|Metabolism--Global and overview maps--Biosynthesis of antibiotics|Metabolism--Global and overview maps--Biosynthesis of amino acids

[denovogenes]_100547	63.37	6e-110	cls:CXIVA_11480	K15460	yfiC, trmX	tRNA1Val (adenine37-N6)-methyltransferase	2.1.1.223	--

[denovogenes]_100548	99.63	0.0	efq:DR75_13	K00286	proC	pyrroline-5-carboxylate reductase	1.5.1.2	Metabolism--Amino acid metabolism--Arginine and proline metabolism|Metabolism--Global and overview maps--Metabolic pathways|Metabolism--Global and overview maps--Biosynthesis of secondary metabolites|Metabolism--Global and overview maps--Biosynthesis of antibiotics|Metabolism--Global and overview maps--Biosynthesis of amino acids

[denovogenes]_100550	99.63	0.0	efi:OG1RF_12319	K03478	chbG	chitin disaccharide deacetylase	3.5.1.105	--

[denovogenes]_100551	99.26	0.0	efq:DR75_215	K16869	lipL	octanoyl-[GcvH]:protein N-octanoyltransferase	2.3.1.204	--

[denovogenes]_100552	72.06	1e-133	fpr:FP2_30970	K02746	PTS-Aga-EIIC, agaW	PTS system, N-acetylgalactosamine-specific IIC component	--	Metabolism--Carbohydrate metabolism--Galactose metabolism|Environmental Information Processing--Membrane transport--Phosphotransferase system (PTS)

[denovogenes]_100553	70.30	4e-139	csb:CLSA_c45260	K00286	proC	pyrroline-5-carboxylate reductase	1.5.1.2	Metabolism--Amino acid metabolism--Arginine and proline metabolism|Metabolism--Global and overview maps--Metabolic pathways|Metabolism--Global and overview maps--Biosynthesis of secondary metabolites|Metabolism--Global and overview maps--Biosynthesis of antibiotics|Metabolism--Global and overview maps--Biosynthesis of amino acids

[denovogenes]_100555	50.42	7e-74	cno:NT01CX_0726	K01227	E3.2.1.96	mannosyl-glycoprotein endo-beta-N-acetylglucosaminidase	3.2.1.96	Metabolism--Glycan biosynthesis and metabolism--Other glycan degradation

[denovogenes]_100557	100.00	0.0	bfb:VU15_18975	K03832	tonB	periplasmic protein TonB	--	--

[denovogenes]_100559	76.75	1e-144	cbv:U729_1328	K03218	rlmB	23S rRNA (guanosine2251-2'-O)-methyltransferase	2.1.1.185	--

[denovogenes]_10056	99.86	0.0	boa:Bovatus_01430	K00527	nrdD	ribonucleoside-triphosphate reductase	1.17.4.2	Metabolism--Nucleotide metabolism--Purine metabolism|Metabolism--Nucleotide metabolism--Pyrimidine metabolism|Metabolism--Global and overview maps--Metabolic pathways

[denovogenes]_100561	68.01	9e-127	fpr:FP2_30970	K02746	PTS-Aga-EIIC, agaW	PTS system, N-acetylgalactosamine-specific IIC component	--	Metabolism--Carbohydrate metabolism--Galactose metabolism|Environmental Information Processing--Membrane transport--Phosphotransferase system (PTS)

[denovogenes]_100562	75.57	7e-148	cdg:CDBI1_05260	K03521	fixA, etfB	electron transfer flavoprotein beta subunit	--	--

[denovogenes]_100564	54.55	7e-95	eft:M395_06405	K03565	recX	regulatory protein	--	--

[denovogenes]_100565	99.63	0.0	ene:ENT_17080	K00806	uppS	undecaprenyl diphosphate synthase	2.5.1.31	Metabolism--Metabolism of terpenoids and polyketides--Terpenoid backbone biosynthesis|Metabolism--Global and overview maps--Biosynthesis of secondary metabolites

[denovogenes]_100567	98.82	0.0	prg:RB151_017020	K03981	dsbC	thiol:disulfide interchange protein DsbC	5.3.4.1	--

[denovogenes]_100569	41.97	1e-54	aoe:Clos_0336	K06872	K06872	uncharacterized protein	--	--

[denovogenes]_10057	97.70	0.0	boa:Bovatus_04091	K02238	comEC	competence protein ComEC	--	--

[denovogenes]_100570	99.63	0.0	sgt:SGGB_2060	K03217	yidC, spoIIIJ, OXA1, ccfA	YidC/Oxa1 family membrane protein insertase	--	Cellular Processes--Cellular community - prokaryotes--Quorum sensing|Genetic Information Processing--Folding, sorting and degradation--Protein export|Environmental Information Processing--Membrane transport--Bacterial secretion system

[denovogenes]_100571	69.40	2e-134	cbt:CLH_0138	K09685	purR	purine operon repressor	--	--

[denovogenes]_100573	64.12	2e-124	pdf:CD630DERM_11230	K07098	K07098	uncharacterized protein	--	--

[denovogenes]_100575	99.63	0.0	stb:SGPB_0139	K06896	mapP	maltose 6'-phosphate phosphatase	3.1.3.90	Metabolism--Carbohydrate metabolism--Starch and sucrose metabolism

[denovogenes]_100577	100.00	0.0	pvl:AOB99_09075	K02016	ABC.FEV.S	iron complex transport system substrate-binding protein	--	Environmental Information Processing--Membrane transport--ABC transporters

[denovogenes]_100578	81.72	3e-160	cbt:CLH_0046	K00931	proB	glutamate 5-kinase	2.7.2.11	Metabolism--Amino acid metabolism--Arginine and proline metabolism|Metabolism--Biosynthesis of other secondary metabolites--Carbapenem biosynthesis|Metabolism--Global and overview maps--Metabolic pathways|Metabolism--Global and overview maps--Biosynthesis of antibiotics|Metabolism--Global and overview maps--Biosynthesis of amino acids

[denovogenes]_100579	48.16	3e-93	fpa:FPR_08880	K03488	licT, bglG	beta-glucoside operon transcriptional antiterminator	--	--

[denovogenes]_100580	100.00	0.0	pvl:AOB99_16620	K06223	dam	DNA adenine methylase	2.1.1.72	Genetic Information Processing--Replication and repair--Mismatch repair

[denovogenes]_100582	100.00	0.0	pmib:BB2000_0220	K07636	phoR	two-component system, OmpR family, phosphate regulon sensor histidine kinase PhoR	2.7.13.3	Environmental Information Processing--Signal transduction--Two-component system

[denovogenes]_100588	53.52	2e-93	cld:CLSPO_c27300	K02556	motA	chemotaxis protein MotA	--	Environmental Information Processing--Signal transduction--Two-component system|Cellular Processes--Cell motility--Bacterial chemotaxis|Cellular Processes--Cell motility--Flagellar assembly

[denovogenes]_100589	100.00	0.0	pvl:AOB99_00405	K02440	GLPF	glycerol uptake facilitator protein	--	--

[denovogenes]_10059	89.81	0.0	clt:CM240_1735	K00656	E2.3.1.54, pflD	formate C-acetyltransferase	2.3.1.54	Metabolism--Carbohydrate metabolism--Pyruvate metabolism|Metabolism--Carbohydrate metabolism--Propanoate metabolism|Metabolism--Carbohydrate metabolism--Butanoate metabolism|Metabolism--Global and overview maps--Metabolic pathways

[denovogenes]_100591	100.00	0.0	cbut:ATN24_00885	K02747	PTS-Aga-EIID, agaE	PTS system, N-acetylgalactosamine-specific IID component	--	Metabolism--Carbohydrate metabolism--Galactose metabolism|Environmental Information Processing--Membrane transport--Phosphotransferase system (PTS)

[denovogenes]_100592	62.08	1e-119	cle:Clole_2837	K03324	yjbB	phosphate:Na+ symporter	--	--

[denovogenes]_100594	56.32	1e-98	cle:Clole_2422	K00981	E2.7.7.41, CDS1, CDS2, cdsA	phosphatidate cytidylyltransferase	2.7.7.41	Metabolism--Lipid metabolism--Glycerophospholipid metabolism|Metabolism--Global and overview maps--Metabolic pathways|Metabolism--Global and overview maps--Biosynthesis of secondary metabolites|Environmental Information Processing--Signal transduction--Phosphatidylinositol signaling system

[denovogenes]_100596	98.15	0.0	sea:SeAg_B4889	K01619	deoC, DERA	deoxyribose-phosphate aldolase	4.1.2.4	Metabolism--Carbohydrate metabolism--Pentose phosphate pathway

[denovogenes]_100597	48.33	6e-95	csb:CLSA_c06850	K00014	aroE	shikimate dehydrogenase	1.1.1.25	Metabolism--Amino acid metabolism--Phenylalanine, tyrosine and tryptophan biosynthesis|Metabolism--Global and overview maps--Metabolic pathways|Metabolism--Global and overview maps--Biosynthesis of secondary metabolites|Metabolism--Global and overview maps--Biosynthesis of antibiotics|Metabolism--Global and overview maps--Biosynthesis of amino acids

[denovogenes]_100598	100.00	0.0	eum:ECUMN_1034	K20861	ybjI	FMN hydrolase / 5-amino-6-(5-phospho-D-ribitylamino)uracil phosphatase	3.1.3.102 3.1.3.104	Metabolism--Metabolism of cofactors and vitamins--Riboflavin metabolism|Metabolism--Global and overview maps--Metabolic pathways|Metabolism--Global and overview maps--Biosynthesis of secondary metabolites

[denovogenes]_100599	95.57	0.0	cfd:CFNIH1_14345	K20861	ybjI	FMN hydrolase / 5-amino-6-(5-phospho-D-ribitylamino)uracil phosphatase	3.1.3.102 3.1.3.104	Metabolism--Metabolism of cofactors and vitamins--Riboflavin metabolism|Metabolism--Global and overview maps--Metabolic pathways|Metabolism--Global and overview maps--Biosynthesis of secondary metabolites

[denovogenes]_1006	54.99	0.0	rto:RTO_05780	K16898	addA	ATP-dependent helicase/nuclease subunit A	3.1.-.- 3.6.4.12	--

[denovogenes]_10060	79.26	0.0	bcel:BcellWH2_00345	K16089	TC.FEV.OM2, cirA, cfrA, hmuR	outer membrane receptor for ferrienterochelin and colicins	--	--

[denovogenes]_100601	73.16	2e-136	fpr:FP2_30970	K02746	PTS-Aga-EIIC, agaW	PTS system, N-acetylgalactosamine-specific IIC component	--	Metabolism--Carbohydrate metabolism--Galactose metabolism|Environmental Information Processing--Membrane transport--Phosphotransferase system (PTS)

[denovogenes]_100603	70.61	2e-144	drm:Dred_2235	K17073	lysX1	putative lysine transport system substrate-binding protein	--	Environmental Information Processing--Membrane transport--ABC transporters

[denovogenes]_100604	100.00	0.0	ece:Z4263	K01624	FBA, fbaA	fructose-bisphosphate aldolase, class II	4.1.2.13	Metabolism--Carbohydrate metabolism--Glycolysis / Gluconeogenesis|Metabolism--Carbohydrate metabolism--Pentose phosphate pathway|Metabolism--Carbohydrate metabolism--Fructose and mannose metabolism|Metabolism--Energy metabolism--Methane metabolism|Metabolism--Energy metabolism--Carbon fixation in photosynthetic organisms|Metabolism--Global and overview maps--Metabolic pathways|Metabolism--Global and overview maps--Biosynthesis of secondary metabolites|Metabolism--Global and overview maps--Microbial metabolism in diverse environments|Metabolism--Global and overview maps--Biosynthesis of antibiotics|Metabolism--Global and overview maps--Carbon metabolism|Metabolism--Global and overview maps--Biosynthesis of amino acids

[denovogenes]_100607	98.90	0.0	eum:ECUMN_0683	K02015	ABC.FEV.P	iron complex transport system permease protein	--	Environmental Information Processing--Membrane transport--ABC transporters

[denovogenes]_100609	62.91	7e-116	cdg:CDBI1_04130	K10823	oppF	oligopeptide transport system ATP-binding protein	--	Human Diseases--Drug resistance: Antimicrobial--beta-Lactam resistance|Environmental Information Processing--Membrane transport--ABC transporters|Cellular Processes--Cellular community - prokaryotes--Quorum sensing

[denovogenes]_100611	91.12	0.0	fnt:HMPREF0405_01546	K01878	glyQ	glycyl-tRNA synthetase alpha chain	6.1.1.14	Genetic Information Processing--Translation--Aminoacyl-tRNA biosynthesis

[denovogenes]_100612	40.15	2e-67	pbj:VN24_14895	K02025	ABC.MS.P	multiple sugar transport system permease protein	--	--

[denovogenes]_100613	100.00	0.0	cif:AL515_02785	K02815	PTS-Sor-EIID, sorM	PTS system, sorbose-specific IID component	--	Metabolism--Carbohydrate metabolism--Fructose and mannose metabolism|Environmental Information Processing--Membrane transport--Phosphotransferase system (PTS)

[denovogenes]_100615	63.60	7e-124	vpr:Vpar_0488	K17836	penP	beta-lactamase class A	3.5.2.6	Metabolism--Biosynthesis of other secondary metabolites--Penicillin and cephalosporin biosynthesis|Metabolism--Global and overview maps--Biosynthesis of antibiotics|Human Diseases--Drug resistance: Antimicrobial--beta-Lactam resistance

[denovogenes]_100617	68.28	3e-132	cpas:Clopa_4804	K01962	accA	acetyl-CoA carboxylase carboxyl transferase subunit alpha	6.4.1.2	Metabolism--Lipid metabolism--Fatty acid biosynthesis|Metabolism--Carbohydrate metabolism--Pyruvate metabolism|Metabolism--Carbohydrate metabolism--Propanoate metabolism|Metabolism--Energy metabolism--Carbon fixation pathways in prokaryotes|Metabolism--Global and overview maps--Metabolic pathways|Metabolism--Global and overview maps--Biosynthesis of secondary metabolites|Metabolism--Global and overview maps--Microbial metabolism in diverse environments|Metabolism--Global and overview maps--Biosynthesis of antibiotics|Metabolism--Global and overview maps--Carbon metabolism|Metabolism--Global and overview maps--Fatty acid metabolism

[denovogenes]_100618	89.43	3e-179	vpr:Vpar_1560	K02032	ABC.PE.A1	peptide/nickel transport system ATP-binding protein	--	Cellular Processes--Cellular community - prokaryotes--Quorum sensing

[denovogenes]_10062	68.68	0.0	fpla:A4U99_05070	K03655	recG	ATP-dependent DNA helicase RecG	3.6.4.12	Genetic Information Processing--Replication and repair--Homologous recombination

[denovogenes]_100620	72.22	3e-147	gfe:Gferi_10445	K00603	fctD	glutamate formiminotransferase	2.1.2.5	Metabolism--Amino acid metabolism--Histidine metabolism|Metabolism--Metabolism of cofactors and vitamins--One carbon pool by folate|Metabolism--Global and overview maps--Metabolic pathways

[denovogenes]_100623	48.34	2e-76	gsb:GSUB_11365	K07714	atoC	two-component system, NtrC family, response regulator AtoC	--	Environmental Information Processing--Signal transduction--Two-component system

[denovogenes]_100624	100.00	0.0	shq:A0259_07345	K01903	sucC	succinyl-CoA synthetase beta subunit	6.2.1.5	Metabolism--Carbohydrate metabolism--Citrate cycle (TCA cycle)|Metabolism--Carbohydrate metabolism--Propanoate metabolism|Metabolism--Carbohydrate metabolism--C5-Branched dibasic acid metabolism|Metabolism--Energy metabolism--Carbon fixation pathways in prokaryotes|Metabolism--Global and overview maps--Metabolic pathways|Metabolism--Global and overview maps--Biosynthesis of secondary metabolites|Metabolism--Global and overview maps--Microbial metabolism in diverse environments|Metabolism--Global and overview maps--Biosynthesis of antibiotics|Metabolism--Global and overview maps--Carbon metabolism

[denovogenes]_100625	46.99	1e-84	has:Halsa_0319	K01436	K01436	amidohydrolase	3.5.1.-	--

[denovogenes]_100626	72.32	3e-148	koy:J415_17065	K19272	aph3-I	aminoglycoside 3'-phosphotransferase I	2.7.1.95	--

[denovogenes]_100627	79.34	2e-157	csb:CLSA_c01040	K09685	purR	purine operon repressor	--	--

[denovogenes]_100629	100.00	0.0	pvl:AOB99_11830	K20968	exsA	AraC family transcriptional regulator, exoenzyme S synthesis regulatory protein ExsA	--	Cellular Processes--Cellular community - prokaryotes--Biofilm formation - Pseudomonas aeruginosa

[denovogenes]_100632	61.24	4e-107	ccb:Clocel_3180	K01255	CARP, pepA	leucyl aminopeptidase	3.4.11.1	Metabolism--Metabolism of other amino acids--Glutathione metabolism|Metabolism--Global and overview maps--Metabolic pathways

[denovogenes]_100638	95.57	0.0	cbut:ATN24_03210	K01951	guaA, GMPS	GMP synthase (glutamine-hydrolysing)	6.3.5.2	Metabolism--Nucleotide metabolism--Purine metabolism|Metabolism--Xenobiotics biodegradation and metabolism--Drug metabolism - other enzymes|Metabolism--Global and overview maps--Metabolic pathways

[denovogenes]_100639	54.04	1e-80	mmaz:MmTuc01_0717	K15987	hppA	K(+)-stimulated pyrophosphate-energized sodium pump	3.6.1.1	Metabolism--Energy metabolism--Oxidative phosphorylation

[denovogenes]_10064	83.88	0.0	rto:RTO_19580	K00962	pnp, PNPT1	polyribonucleotide nucleotidyltransferase	2.7.7.8	Metabolism--Nucleotide metabolism--Purine metabolism|Metabolism--Nucleotide metabolism--Pyrimidine metabolism|Genetic Information Processing--Folding, sorting and degradation--RNA degradation

[denovogenes]_100640	62.08	8e-117	tae:TepiRe1_0197	K03046	rpoC	DNA-directed RNA polymerase subunit beta'	2.7.7.6	Metabolism--Nucleotide metabolism--Purine metabolism|Metabolism--Nucleotide metabolism--Pyrimidine metabolism|Metabolism--Global and overview maps--Metabolic pathways|Genetic Information Processing--Transcription--RNA polymerase

[denovogenes]_100641	100.00	0.0	ecm:EcSMS35_0811	K07027	K07027	glycosyltransferase 2 family protein	--	--

[denovogenes]_100643	68.95	4e-129	tae:TepiRe1_0197	K03046	rpoC	DNA-directed RNA polymerase subunit beta'	2.7.7.6	Metabolism--Nucleotide metabolism--Purine metabolism|Metabolism--Nucleotide metabolism--Pyrimidine metabolism|Metabolism--Global and overview maps--Metabolic pathways|Genetic Information Processing--Transcription--RNA polymerase

[denovogenes]_100644	95.96	5e-178	cfd:CFNIH1_02850	K00865	glxK, garK	glycerate 2-kinase	2.7.1.165	Metabolism--Amino acid metabolism--Glycine, serine and threonine metabolism|Metabolism--Lipid metabolism--Glycerolipid metabolism|Metabolism--Carbohydrate metabolism--Glyoxylate and dicarboxylate metabolism|Metabolism--Global and overview maps--Metabolic pathways|Metabolism--Global and overview maps--Microbial metabolism in diverse environments|Metabolism--Global and overview maps--Biosynthesis of antibiotics

[denovogenes]_100645	57.20	2e-118	bcf:bcf_25570	K12573	rnr, vacB	ribonuclease R	3.1.-.-	Genetic Information Processing--Folding, sorting and degradation--RNA degradation

[denovogenes]_100647	98.53	0.0	cfd:CFNIH1_20610	K05875	tar	methyl-accepting chemotaxis protein II, aspartate sensor receptor	--	Environmental Information Processing--Signal transduction--Two-component system|Cellular Processes--Cell motility--Bacterial chemotaxis

[denovogenes]_100648	65.41	1e-127	cpr:CPR_0334	K00075	murB	UDP-N-acetylmuramate dehydrogenase	1.3.1.98	Metabolism--Carbohydrate metabolism--Amino sugar and nucleotide sugar metabolism|Metabolism--Glycan biosynthesis and metabolism--Peptidoglycan biosynthesis|Metabolism--Global and overview maps--Metabolic pathways

[denovogenes]_100649	95.17	0.0	bhl:Bache_0402	K07106	murQ	N-acetylmuramic acid 6-phosphate etherase	4.2.1.126	Metabolism--Carbohydrate metabolism--Amino sugar and nucleotide sugar metabolism|Metabolism--Global and overview maps--Metabolic pathways

[denovogenes]_100652	69.89	2e-130	ssg:Selsp_1170	K13292	lgt, umpA	phosphatidylglycerol:prolipoprotein diacylglycerol transferase	2.-.-.-	--

[denovogenes]_100653	58.65	1e-107	ain:Acin_0341	K07053	E3.1.3.97	3',5'-nucleoside bisphosphate phosphatase	3.1.3.97	--

[denovogenes]_100654	84.79	1e-167	bth:BT_2863	K01991	ABC-2.OM, wza	polysaccharide biosynthesis/export protein	--	Cellular Processes--Cellular community - prokaryotes--Biofilm formation - Escherichia coli

[denovogenes]_100655	40.82	5e-62	crn:CAR_c04960	K20859	phnPP	phosphoribosyl 1,2-cyclic phosphate 1,2-diphosphodiesterase	3.1.4.57	Metabolism--Metabolism of other amino acids--Phosphonate and phosphinate metabolism

[denovogenes]_100656	68.63	4e-135	csh:Closa_1181	K02053	ABC.SP.P	putative spermidine/putrescine transport system permease protein	--	Cellular Processes--Cellular community - prokaryotes--Quorum sensing

[denovogenes]_100658	75.28	2e-148	byl:A4V09_12350	K11071	potB	spermidine/putrescine transport system permease protein	--	Environmental Information Processing--Membrane transport--ABC transporters

[denovogenes]_100659	89.89	0.0	cct:CC1_08860	K00571	E2.1.1.72	site-specific DNA-methyltransferase (adenine-specific)	2.1.1.72	--

[denovogenes]_10066	77.09	0.0	cso:CLS_26700	K03655	recG	ATP-dependent DNA helicase RecG	3.6.4.12	Genetic Information Processing--Replication and repair--Homologous recombination

[denovogenes]_100660	75.57	3e-148	ccel:CCDG5_0302	K01611	speD, AMD1	S-adenosylmethionine decarboxylase	4.1.1.50	Metabolism--Amino acid metabolism--Cysteine and methionine metabolism|Metabolism--Amino acid metabolism--Arginine and proline metabolism|Metabolism--Global and overview maps--Metabolic pathways

[denovogenes]_100661	100.00	0.0	boa:Bovatus_03009	K03734	apbE	FAD:protein FMN transferase	2.7.1.180	--

[denovogenes]_100662	92.06	3e-177	amu:Amuc_1806	K01607	pcaC	4-carboxymuconolactone decarboxylase	4.1.1.44	Metabolism--Xenobiotics biodegradation and metabolism--Benzoate degradation|Metabolism--Global and overview maps--Metabolic pathways|Metabolism--Global and overview maps--Microbial metabolism in diverse environments|Metabolism--Global and overview maps--Degradation of aromatic compounds

[denovogenes]_100663	94.10	1e-171	bhl:Bache_1107	K03832	tonB	periplasmic protein TonB	--	--

[denovogenes]_100664	74.54	8e-159	bprm:CL3_08540	K09692	tagG	teichoic acid transport system permease protein	--	Environmental Information Processing--Membrane transport--ABC transporters

[denovogenes]_100667	65.19	4e-122	fpla:A4U99_17385	K05810	yfiH	laccase domain-containing protein	--	--

[denovogenes]_100668	61.39	6e-106	rto:RTO_24290	K07402	xdhC	xanthine dehydrogenase accessory factor	--	--

[denovogenes]_10067	40.65	5e-78	nth:Nther_0090	K03721	tyrR	transcriptional regulator of aroF, aroG, tyrA and aromatic amino acid transport	--	--

[denovogenes]_100670	62.65	3e-118	pgi:PG_0425	K01070	frmB, ESD, fghA	S-formylglutathione hydrolase	3.1.2.12	Metabolism--Energy metabolism--Methane metabolism|Metabolism--Global and overview maps--Microbial metabolism in diverse environments|Metabolism--Global and overview maps--Carbon metabolism

[denovogenes]_100673	100.00	0.0	bxy:BXY_29390	K07106	murQ	N-acetylmuramic acid 6-phosphate etherase	4.2.1.126	Metabolism--Carbohydrate metabolism--Amino sugar and nucleotide sugar metabolism|Metabolism--Global and overview maps--Metabolic pathways

[denovogenes]_100674	41.67	4e-62	rob:CK5_31320	K01104	E3.1.3.48	protein-tyrosine phosphatase	3.1.3.48	--

[denovogenes]_100675	48.55	7e-73	agl:PYTT_1124	K01686	uxuA	mannonate dehydratase	4.2.1.8	Metabolism--Carbohydrate metabolism--Pentose and glucuronate interconversions|Metabolism--Global and overview maps--Metabolic pathways

[denovogenes]_100677	48.28	2e-82	mas:Mahau_0155	K01101	E3.1.3.41	4-nitrophenyl phosphatase	3.1.3.41	Metabolism--Xenobiotics biodegradation and metabolism--Aminobenzoate degradation|Metabolism--Global and overview maps--Microbial metabolism in diverse environments

[denovogenes]_100679	98.89	0.0	boa:Bovatus_02305	K03327	TC.MATE, SLC47A, norM, mdtK, dinF	multidrug resistance protein, MATE family	--	--

[denovogenes]_100681	100.00	0.0	bfb:VU15_18400	K06861	lptB	lipopolysaccharide export system ATP-binding protein	3.6.3.-	Environmental Information Processing--Membrane transport--ABC transporters

[denovogenes]_10069	93.39	0.0	bdh:GV66_06305	K12573	rnr, vacB	ribonuclease R	3.1.-.-	Genetic Information Processing--Folding, sorting and degradation--RNA degradation

[denovogenes]_100693	64.45	2e-121	cay:CEA_G1513	K07074	K07074	uncharacterized protein	--	--

[denovogenes]_100695	46.79	6e-84	fpla:A4U99_16940	K19427	epsJ	glycosyltransferase EpsJ	2.4.-.-	--

[denovogenes]_100696	100.00	0.0	bxy:BXY_16620	K03832	tonB	periplasmic protein TonB	--	--

[denovogenes]_100698	66.54	2e-128	mhg:MHY_20840	K03337	iolB	5-deoxy-glucuronate isomerase	5.3.1.30	Metabolism--Carbohydrate metabolism--Inositol phosphate metabolism|Metabolism--Global and overview maps--Metabolic pathways|Metabolism--Global and overview maps--Microbial metabolism in diverse environments

[denovogenes]_100699	45.66	3e-48	sfo:Z042_09270	K18910	dpe, lre	D-psicose/D-tagatose/L-ribulose 3-epimerase	5.1.3.30 5.1.3.31	--

[denovogenes]_10070	82.33	0.0	lacy:A4V08_25405	K03169	topB	DNA topoisomerase III	5.99.1.2	--

[denovogenes]_100702	100.00	0.0	elw:ECW_m3304	K15777	DOPA	4,5-DOPA dioxygenase extradiol	1.13.11.-	Metabolism--Biosynthesis of other secondary metabolites--Betalain biosynthesis

[denovogenes]_100703	99.26	0.0	eld:i02_3483	K07238	TC.ZIP, zupT, ZRT3, ZIP2	zinc transporter, ZIP family	--	--

[denovogenes]_100704	56.83	5e-103	ccb:Clocel_2976	K01715	crt	enoyl-CoA hydratase	4.2.1.17	Metabolism--Carbohydrate metabolism--Butanoate metabolism|Metabolism--Global and overview maps--Carbon metabolism

[denovogenes]_100705	100.00	0.0	sbc:SbBS512_E2177	K00575	cheR	chemotaxis protein methyltransferase CheR	2.1.1.80	Environmental Information Processing--Signal transduction--Two-component system|Cellular Processes--Cell motility--Bacterial chemotaxis

[denovogenes]_100706	92.25	2e-178	bxy:BXY_16620	K03832	tonB	periplasmic protein TonB	--	--

[denovogenes]_100707	48.19	8e-74	agl:PYTT_1124	K01686	uxuA	mannonate dehydratase	4.2.1.8	Metabolism--Carbohydrate metabolism--Pentose and glucuronate interconversions|Metabolism--Global and overview maps--Metabolic pathways

[denovogenes]_100709	77.12	8e-156	lacy:A4V08_13045	K09692	tagG	teichoic acid transport system permease protein	--	Environmental Information Processing--Membrane transport--ABC transporters

[denovogenes]_100711	44.83	2e-34	woc:BA177_07085	K08676	tri	tricorn protease	3.4.21.-	--

[denovogenes]_100712	72.34	3e-150	lacy:A4V08_34100	K07095	K07095	uncharacterized protein	--	--

[denovogenes]_100713	62.06	2e-106	tae:TepiRe1_1951	K01995	livG	branched-chain amino acid transport system ATP-binding protein	--	Environmental Information Processing--Membrane transport--ABC transporters|Cellular Processes--Cellular community - prokaryotes--Quorum sensing

[denovogenes]_100716	93.75	0.0	bdo:EL88_13150	K03596	lepA	GTP-binding protein LepA	--	Human Diseases--Infectious diseases: Bacterial--Legionellosis

[denovogenes]_100718	99.63	0.0	bvu:BVU_1615	K03561	exbB	biopolymer transport protein ExbB	--	--

[denovogenes]_100719	58.21	3e-112	ctx:Clo1313_0651	K01778	dapF	diaminopimelate epimerase	5.1.1.7	Metabolism--Amino acid metabolism--Lysine biosynthesis|Metabolism--Global and overview maps--Metabolic pathways|Metabolism--Global and overview maps--Biosynthesis of secondary metabolites|Metabolism--Global and overview maps--Microbial metabolism in diverse environments|Metabolism--Global and overview maps--Biosynthesis of antibiotics|Metabolism--Global and overview maps--Biosynthesis of amino acids

[denovogenes]_10072	58.61	3e-109	rpm:RSPPHO_00982	K03406	mcp	methyl-accepting chemotaxis protein	--	Environmental Information Processing--Signal transduction--Two-component system|Cellular Processes--Cell motility--Bacterial chemotaxis

[denovogenes]_100724	99.63	0.0	ecy:ECSE_2185	K07026	E3.1.3.70	mannosyl-3-phosphoglycerate phosphatase	3.1.3.70	Metabolism--Carbohydrate metabolism--Fructose and mannose metabolism

[denovogenes]_100728	47.25	1e-78	ibu:IB211_00237	K00563	rlmA1	23S rRNA (guanine745-N1)-methyltransferase	2.1.1.187	--

[denovogenes]_100729	90.74	2e-175	lacy:A4V08_02545	K03310	TC.AGCS	alanine or glycine:cation symporter, AGCS family	--	--

[denovogenes]_10073	50.41	4e-109	dca:Desca_2486	K03738	aor	aldehyde:ferredoxin oxidoreductase	1.2.7.5	Metabolism--Carbohydrate metabolism--Pentose phosphate pathway|Metabolism--Global and overview maps--Metabolic pathways|Metabolism--Global and overview maps--Microbial metabolism in diverse environments|Metabolism--Global and overview maps--Carbon metabolism

[denovogenes]_100731	59.84	8e-52	rim:ROI_08810	K07720	yesN	two-component system, response regulator YesN	--	Environmental Information Processing--Signal transduction--Two-component system

[denovogenes]_100732	58.30	3e-111	rho:RHOM_10495	K01992	ABC-2.P	ABC-2 type transport system permease protein	--	--

[denovogenes]_100733	74.91	1e-138	cpy:Cphy_0315	K02406	fliC	flagellin	--	Environmental Information Processing--Signal transduction--Two-component system|Cellular Processes--Cell motility--Flagellar assembly|Organismal Systems--Immune system--NOD-like receptor signaling pathway|Organismal Systems--Environmental adaptation--Plant-pathogen interaction|Human Diseases--Infectious diseases: Bacterial--Salmonella infection|Human Diseases--Infectious diseases: Bacterial--Legionellosis

[denovogenes]_100738	62.75	5e-115	dsy:DSY3394	K02050	ABC.SN.P	NitT/TauT family transport system permease protein	--	--

[denovogenes]_10074	56.00	0.0	clb:Clo1100_0792	K01915	glnA, GLUL	glutamine synthetase	6.3.1.2	Metabolism--Amino acid metabolism--Arginine biosynthesis|Metabolism--Amino acid metabolism--Alanine, aspartate and glutamate metabolism|Metabolism--Carbohydrate metabolism--Glyoxylate and dicarboxylate metabolism|Metabolism--Energy metabolism--Nitrogen metabolism|Metabolism--Global and overview maps--Metabolic pathways|Metabolism--Global and overview maps--Microbial metabolism in diverse environments|Metabolism--Global and overview maps--Biosynthesis of amino acids|Environmental Information Processing--Signal transduction--Two-component system|Organismal Systems--Nervous system--Glutamatergic synapse|Organismal Systems--Nervous system--GABAergic synapse

[denovogenes]_100740	100.00	0.0	eum:ECUMN_1010	K07757	ybiV	sugar-phosphatase	3.1.3.23	--

[denovogenes]_100743	98.16	0.0	cif:AL515_00100	K03305	TC.POT	proton-dependent oligopeptide transporter, POT family	--	--

[denovogenes]_100749	100.00	0.0	shq:A0259_04835	K02073	metQ	D-methionine transport system substrate-binding protein	--	Environmental Information Processing--Membrane transport--ABC transporters

[denovogenes]_10075	99.86	0.0	paeb:NCGM1900_0064	K01414	prlC	oligopeptidase A	3.4.24.70	--

[denovogenes]_100750	92.28	0.0	lacy:A4V08_09195	K02434	gatB, PET112	aspartyl-tRNA(Asn)/glutamyl-tRNA(Gln) amidotransferase subunit B	6.3.5.6 6.3.5.7	Genetic Information Processing--Translation--Aminoacyl-tRNA biosynthesis|Metabolism--Global and overview maps--Metabolic pathways

[denovogenes]_100752	72.63	9e-125	ova:OBV_13530	K02040	pstS	phosphate transport system substrate-binding protein	--	Environmental Information Processing--Membrane transport--ABC transporters|Environmental Information Processing--Signal transduction--Two-component system|Human Diseases--Infectious diseases: Bacterial--Tuberculosis

[denovogenes]_100753	71.32	3e-139	bcel:BcellWH2_01155	K01740	metY	O-acetylhomoserine (thiol)-lyase	2.5.1.49	Metabolism--Amino acid metabolism--Cysteine and methionine metabolism|Metabolism--Global and overview maps--Metabolic pathways

[denovogenes]_100754	97.79	0.0	cfd:CFNIH1_24340	K03761	kgtP	MFS transporter, MHS family, alpha-ketoglutarate permease	--	--

[denovogenes]_100755	100.00	0.0	ecoa:APECO78_12640	K00262	E1.4.1.4, gdhA	glutamate dehydrogenase (NADP+)	1.4.1.4	Metabolism--Amino acid metabolism--Arginine biosynthesis|Metabolism--Amino acid metabolism--Alanine, aspartate and glutamate metabolism|Metabolism--Energy metabolism--Nitrogen metabolism|Metabolism--Global and overview maps--Metabolic pathways

[denovogenes]_100756	100.00	0.0	lacy:A4V08_31035	K00575	cheR	chemotaxis protein methyltransferase CheR	2.1.1.80	Environmental Information Processing--Signal transduction--Two-component system|Cellular Processes--Cell motility--Bacterial chemotaxis

[denovogenes]_100757	93.63	0.0	bcel:BcellWH2_02249	K07106	murQ	N-acetylmuramic acid 6-phosphate etherase	4.2.1.126	Metabolism--Carbohydrate metabolism--Amino sugar and nucleotide sugar metabolism|Metabolism--Global and overview maps--Metabolic pathways

[denovogenes]_100759	99.63	0.0	cfd:CFNIH1_09990	K05801	djlA	DnaJ like chaperone protein	--	--

[denovogenes]_10076	92.66	0.0	bfb:VU15_01205	K01190	lacZ	beta-galactosidase	3.2.1.23	Metabolism--Carbohydrate metabolism--Galactose metabolism|Metabolism--Glycan biosynthesis and metabolism--Other glycan degradation|Metabolism--Lipid metabolism--Sphingolipid metabolism|Metabolism--Global and overview maps--Metabolic pathways

[denovogenes]_100760	98.89	0.0	cif:AL515_10150	K02006	cbiO	cobalt/nickel transport system ATP-binding protein	--	Environmental Information Processing--Membrane transport--ABC transporters

[denovogenes]_100761	96.31	0.0	bhl:Bache_0491	K02470	gyrB	DNA gyrase subunit B	5.99.1.3	--

[denovogenes]_100762	75.37	1e-145	cle:Clole_4168	K02071	metN	D-methionine transport system ATP-binding protein	--	Environmental Information Processing--Membrane transport--ABC transporters

[denovogenes]_100763	100.00	0.0	shq:A0259_06200	K10973	allR	IclR family transcriptional regulator, negative regulator of allantoin and glyoxylate utilization operons	--	--

[denovogenes]_100764	84.56	3e-160	lacy:A4V08_32660	K06147	ABCB-BAC	ATP-binding cassette, subfamily B, bacterial	--	--

[denovogenes]_100765	97.41	0.0	cfd:CFNIH1_14235	K07757	ybiV	sugar-phosphatase	3.1.3.23	--

[denovogenes]_100767	57.25	6e-106	dsy:DSY3417	K07484	K07484	transposase	--	--

[denovogenes]_100768	100.00	0.0	eoi:ECO111_2930	K02200	ccmH	cytochrome c-type biogenesis protein CcmH	--	--

[denovogenes]_100770	99.63	0.0	pdi:BDI_2256	K00931	proB	glutamate 5-kinase	2.7.2.11	Metabolism--Amino acid metabolism--Arginine and proline metabolism|Metabolism--Biosynthesis of other secondary metabolites--Carbapenem biosynthesis|Metabolism--Global and overview maps--Metabolic pathways|Metabolism--Global and overview maps--Biosynthesis of antibiotics|Metabolism--Global and overview maps--Biosynthesis of amino acids

[denovogenes]_100771	87.87	1e-172	tfo:BFO_2808	K08138	xylE	MFS transporter, SP family, xylose:H+ symportor	--	--

[denovogenes]_100772	76.67	6e-156	bprs:CK3_19000	K07719	ycbB, glnL	two-component system, response regulator YcbB	--	Environmental Information Processing--Signal transduction--Two-component system

[denovogenes]_100777	61.03	8e-116	bdh:GV66_19800	K05970	SIAE	sialate O-acetylesterase	3.1.1.53	--

[denovogenes]_100779	77.21	2e-156	cle:Clole_1001	K19294	algI	alginate O-acetyltransferase complex protein AlgI	--	--

[denovogenes]_10078	99.28	0.0	cfd:CFNIH1_02485	K01183	E3.2.1.14	chitinase	3.2.1.14	Metabolism--Carbohydrate metabolism--Amino sugar and nucleotide sugar metabolism|Metabolism--Global and overview maps--Metabolic pathways

[denovogenes]_100780	85.98	2e-170	erb:A4V01_18025	K03431	glmM	phosphoglucosamine mutase	5.4.2.10	Metabolism--Carbohydrate metabolism--Amino sugar and nucleotide sugar metabolism|Metabolism--Global and overview maps--Metabolic pathways|Metabolism--Global and overview maps--Biosynthesis of antibiotics

[denovogenes]_100782	64.41	2e-109	ess:ATZ33_10370	K02755	PTS-Bgl-EIIA, bglF, bglP	PTS system, beta-glucoside-specific IIA component	2.7.1.-	Environmental Information Processing--Membrane transport--Phosphotransferase system (PTS)

[denovogenes]_100782	64.41	2e-109	ess:ATZ33_10370	K02756	PTS-Bgl-EIIB, bglF, bglP	PTS system, beta-glucoside-specific IIB component	2.7.1.-	Environmental Information Processing--Membrane transport--Phosphotransferase system (PTS)

[denovogenes]_100782	64.41	2e-109	ess:ATZ33_10370	K02757	PTS-Bgl-EIIC, bglF, bglP	PTS system, beta-glucoside-specific IIC component	--	Environmental Information Processing--Membrane transport--Phosphotransferase system (PTS)

[denovogenes]_100783	50.94	2e-85	mpz:Marpi_0697	K02056	ABC.SS.A	simple sugar transport system ATP-binding protein	3.6.3.17	--

[denovogenes]_100784	73.87	2e-115	bprs:CK3_22980	K07258	dacC, dacA, dacD	D-alanyl-D-alanine carboxypeptidase (penicillin-binding protein 5/6)	3.4.16.4	Metabolism--Glycan biosynthesis and metabolism--Peptidoglycan biosynthesis|Metabolism--Global and overview maps--Metabolic pathways

[denovogenes]_100785	57.25	2e-103	ova:OBV_27790	K04759	feoB	ferrous iron transport protein B	--	--

[denovogenes]_100786	85.38	5e-161	cpy:Cphy_2192	K17319	lplB	putative aldouronate transport system permease protein	--	Environmental Information Processing--Membrane transport--ABC transporters

[denovogenes]_100788	92.65	0.0	rto:RTO_26370	K01940	argG, ASS1	argininosuccinate synthase	6.3.4.5	Metabolism--Amino acid metabolism--Arginine biosynthesis|Metabolism--Amino acid metabolism--Alanine, aspartate and glutamate metabolism|Metabolism--Global and overview maps--Metabolic pathways|Metabolism--Global and overview maps--Biosynthesis of secondary metabolites|Metabolism--Global and overview maps--Biosynthesis of antibiotics|Metabolism--Global and overview maps--Biosynthesis of amino acids|Human Diseases--Cardiovascular diseases--Fluid shear stress and atherosclerosis

[denovogenes]_100789	100.00	0.0	sfe:SFxv_0973	K00656	E2.3.1.54, pflD	formate C-acetyltransferase	2.3.1.54	Metabolism--Carbohydrate metabolism--Pyruvate metabolism|Metabolism--Carbohydrate metabolism--Propanoate metabolism|Metabolism--Carbohydrate metabolism--Butanoate metabolism|Metabolism--Global and overview maps--Metabolic pathways

[denovogenes]_100791	90.07	0.0	erb:A4V01_15570	K03705	hrcA	heat-inducible transcriptional repressor	--	--

[denovogenes]_100792	77.94	5e-154	fpla:A4U99_09575	K07568	queA	S-adenosylmethionine:tRNA ribosyltransferase-isomerase	2.4.99.17	--

[denovogenes]_100793	99.63	0.0	pdi:BDI_2906	K05592	deaD, cshA	ATP-dependent RNA helicase DeaD	3.6.4.13	Genetic Information Processing--Folding, sorting and degradation--RNA degradation

[denovogenes]_100798	62.77	4e-118	bur:Bcep18194_B1321	K05882	E1.1.1.91	aryl-alcohol dehydrogenase (NADP+)	1.1.1.91	--

[denovogenes]_1008	65.69	0.0	cai:Caci_3728	K01941	E6.3.4.6	urea carboxylase	6.3.4.6	Metabolism--Amino acid metabolism--Arginine biosynthesis|Metabolism--Xenobiotics biodegradation and metabolism--Atrazine degradation|Metabolism--Global and overview maps--Metabolic pathways

[denovogenes]_10080	98.71	0.0	pdi:BDI_1729	K03546	sbcC	exonuclease SbcC	--	--

[denovogenes]_100801	72.63	4e-140	rto:RTO_10340	K01962	accA	acetyl-CoA carboxylase carboxyl transferase subunit alpha	6.4.1.2	Metabolism--Lipid metabolism--Fatty acid biosynthesis|Metabolism--Carbohydrate metabolism--Pyruvate metabolism|Metabolism--Carbohydrate metabolism--Propanoate metabolism|Metabolism--Energy metabolism--Carbon fixation pathways in prokaryotes|Metabolism--Global and overview maps--Metabolic pathways|Metabolism--Global and overview maps--Biosynthesis of secondary metabolites|Metabolism--Global and overview maps--Microbial metabolism in diverse environments|Metabolism--Global and overview maps--Biosynthesis of antibiotics|Metabolism--Global and overview maps--Carbon metabolism|Metabolism--Global and overview maps--Fatty acid metabolism

[denovogenes]_100801	72.63	4e-140	rto:RTO_10340	K01963	accD	acetyl-CoA carboxylase carboxyl transferase subunit beta	6.4.1.2	Metabolism--Lipid metabolism--Fatty acid biosynthesis|Metabolism--Carbohydrate metabolism--Pyruvate metabolism|Metabolism--Carbohydrate metabolism--Propanoate metabolism|Metabolism--Energy metabolism--Carbon fixation pathways in prokaryotes|Metabolism--Global and overview maps--Metabolic pathways|Metabolism--Global and overview maps--Biosynthesis of secondary metabolites|Metabolism--Global and overview maps--Microbial metabolism in diverse environments|Metabolism--Global and overview maps--Biosynthesis of antibiotics|Metabolism--Global and overview maps--Carbon metabolism|Metabolism--Global and overview maps--Fatty acid metabolism

[denovogenes]_100802	78.31	1e-156	ccel:CCDG5_0699	K02470	gyrB	DNA gyrase subunit B	5.99.1.3	--

[denovogenes]_100804	80.19	5e-117	awo:Awo_c04530	K00527	nrdD	ribonucleoside-triphosphate reductase	1.17.4.2	Metabolism--Nucleotide metabolism--Purine metabolism|Metabolism--Nucleotide metabolism--Pyrimidine metabolism|Metabolism--Global and overview maps--Metabolic pathways

[denovogenes]_100806	84.93	1e-168	pfr:PFREUD_23220	K01011	TST, MPST, sseA	thiosulfate/3-mercaptopyruvate sulfurtransferase	2.8.1.1 2.8.1.2	Metabolism--Amino acid metabolism--Cysteine and methionine metabolism|Metabolism--Energy metabolism--Sulfur metabolism|Metabolism--Global and overview maps--Metabolic pathways|Metabolism--Global and overview maps--Microbial metabolism in diverse environments|Genetic Information Processing--Folding, sorting and degradation--Sulfur relay system

[denovogenes]_100807	93.92	0.0	pfre:RM25_0076	K00655	plsC	1-acyl-sn-glycerol-3-phosphate acyltransferase	2.3.1.51	Metabolism--Lipid metabolism--Glycerolipid metabolism|Metabolism--Lipid metabolism--Glycerophospholipid metabolism|Metabolism--Global and overview maps--Metabolic pathways|Metabolism--Global and overview maps--Biosynthesis of secondary metabolites

[denovogenes]_100808	66.93	9e-112	aaci:ASQ49_14900	K02049	ABC.SN.A	NitT/TauT family transport system ATP-binding protein	--	--

[denovogenes]_100809	89.30	2e-154	pfre:RM25_1168	K02034	ABC.PE.P1	peptide/nickel transport system permease protein	--	Cellular Processes--Cellular community - prokaryotes--Quorum sensing

[denovogenes]_10081	46.70	2e-173	sen:SACE_2851	K01895	ACSS, acs	acetyl-CoA synthetase	6.2.1.1	Metabolism--Carbohydrate metabolism--Glycolysis / Gluconeogenesis|Metabolism--Carbohydrate metabolism--Pyruvate metabolism|Metabolism--Carbohydrate metabolism--Propanoate metabolism|Metabolism--Energy metabolism--Methane metabolism|Metabolism--Energy metabolism--Carbon fixation pathways in prokaryotes|Metabolism--Global and overview maps--Metabolic pathways|Metabolism--Global and overview maps--Biosynthesis of secondary metabolites|Metabolism--Global and overview maps--Microbial metabolism in diverse environments|Metabolism--Global and overview maps--Biosynthesis of antibiotics|Metabolism--Global and overview maps--Carbon metabolism

[denovogenes]_100810	89.39	2e-171	pfre:RM25_1081	K00721	DPM1	dolichol-phosphate mannosyltransferase	2.4.1.83	Metabolism--Glycan biosynthesis and metabolism--N-Glycan biosynthesis|Metabolism--Global and overview maps--Metabolic pathways

[denovogenes]_100811	95.33	9e-175	pfr:PFREUD_16550	K03149	thiG	thiazole synthase	2.8.1.10	Metabolism--Metabolism of cofactors and vitamins--Thiamine metabolism|Metabolism--Global and overview maps--Metabolic pathways

[denovogenes]_100818	73.98	3e-136	pfr:PFREUD_07850	K01104	E3.1.3.48	protein-tyrosine phosphatase	3.1.3.48	--

[denovogenes]_100819	98.14	0.0	btho:Btheta7330_01808	K06897	K06897	7,8-dihydropterin-6-yl-methyl-4-(beta-D-ribofuranosyl)aminobenzene 5'-phosphate synthase	2.5.1.105	--

[denovogenes]_100820	99.60	0.0	btho:Btheta7330_00976	K12343	SRD5A1	3-oxo-5-alpha-steroid 4-dehydrogenase 1	1.3.1.22	Metabolism--Lipid metabolism--Steroid hormone biosynthesis

[denovogenes]_100823	74.25	7e-150	dvm:DvMF_0432	K01627	kdsA	2-dehydro-3-deoxyphosphooctonate aldolase (KDO 8-P synthase)	2.5.1.55	Metabolism--Glycan biosynthesis and metabolism--Lipopolysaccharide biosynthesis|Metabolism--Global and overview maps--Metabolic pathways

[denovogenes]_100827	99.63	0.0	cfd:CFNIH1_00680	K11607	sitB	manganese/iron transport system ATP-binding protein	--	Environmental Information Processing--Membrane transport--ABC transporters

[denovogenes]_100828	43.22	3e-64	kom:HR38_10750	K07454	K07454	putative restriction endonuclease	--	--

[denovogenes]_100831	62.22	5e-121	pak:HMPREF0675_4659	K05522	nei	endonuclease VIII	3.2.2.- 4.2.99.18	Genetic Information Processing--Replication and repair--Base excision repair

[denovogenes]_100833	100.00	0.0	paeo:M801_3640	K00981	E2.7.7.41, CDS1, CDS2, cdsA	phosphatidate cytidylyltransferase	2.7.7.41	Metabolism--Lipid metabolism--Glycerophospholipid metabolism|Metabolism--Global and overview maps--Metabolic pathways|Metabolism--Global and overview maps--Biosynthesis of secondary metabolites|Environmental Information Processing--Signal transduction--Phosphatidylinositol signaling system

[denovogenes]_100837	44.83	4e-44	ftx:AW25_572	K15915	pglC	undecaprenyl phosphate N,N'-diacetylbacillosamine 1-phosphate transferase	2.7.8.36	--

[denovogenes]_100839	99.63	0.0	shq:A0259_20360	K02452	gspC	general secretion pathway protein C	--	Environmental Information Processing--Membrane transport--Bacterial secretion system|Cellular Processes--Cellular community - prokaryotes--Biofilm formation - Vibrio cholerae

[denovogenes]_100843	100.00	0.0	paes:SCV20265_5144	K07126	K07126	uncharacterized protein	--	--

[denovogenes]_100845	68.66	7e-140	str:Sterm_3749	K06971	K06971	uncharacterized protein	--	--

[denovogenes]_100846	43.25	1e-63	dfi:AXF13_01035	K02624	pcaR	IclR family transcriptional regulator, pca regulon regulatory protein	--	--

[denovogenes]_100847	100.00	0.0	efn:DENG_01114	K06442	tlyA	23S rRNA (cytidine1920-2'-O)/16S rRNA (cytidine1409-2'-O)-methyltransferase	2.1.1.226 2.1.1.227	--

[denovogenes]_100848	100.00	0.0	paec:M802_5126	K01011	TST, MPST, sseA	thiosulfate/3-mercaptopyruvate sulfurtransferase	2.8.1.1 2.8.1.2	Metabolism--Amino acid metabolism--Cysteine and methionine metabolism|Metabolism--Energy metabolism--Sulfur metabolism|Metabolism--Global and overview maps--Metabolic pathways|Metabolism--Global and overview maps--Microbial metabolism in diverse environments|Genetic Information Processing--Folding, sorting and degradation--Sulfur relay system

[denovogenes]_100849	70.68	5e-139	ibu:IB211_00948	K05833	ABC.X4.A	putative ABC transport system ATP-binding protein	--	--

[denovogenes]_10085	58.61	1e-109	rpm:RSPPHO_00982	K03406	mcp	methyl-accepting chemotaxis protein	--	Environmental Information Processing--Signal transduction--Two-component system|Cellular Processes--Cell motility--Bacterial chemotaxis

[denovogenes]_100850	98.15	0.0	bfb:VU15_18975	K03832	tonB	periplasmic protein TonB	--	--

[denovogenes]_100853	99.63	0.0	paeo:M801_3221	K03609	minD	septum site-determining protein MinD	--	--

[denovogenes]_100855	98.89	0.0	cfd:CFNIH1_14345	K20861	ybjI	FMN hydrolase / 5-amino-6-(5-phospho-D-ribitylamino)uracil phosphatase	3.1.3.102 3.1.3.104	Metabolism--Metabolism of cofactors and vitamins--Riboflavin metabolism|Metabolism--Global and overview maps--Metabolic pathways|Metabolism--Global and overview maps--Biosynthesis of secondary metabolites

[denovogenes]_100857	100.00	0.0	paeo:M801_3587	K18540	ramA	(R)-amidase	3.5.1.100	--

[denovogenes]_100860	73.61	4e-153	lacy:A4V08_31035	K00575	cheR	chemotaxis protein methyltransferase CheR	2.1.1.80	Environmental Information Processing--Signal transduction--Two-component system|Cellular Processes--Cell motility--Bacterial chemotaxis

[denovogenes]_100861	99.26	0.0	fpla:A4U99_01120	K02564	nagB, GNPDA	glucosamine-6-phosphate deaminase	3.5.99.6	Metabolism--Carbohydrate metabolism--Amino sugar and nucleotide sugar metabolism|Metabolism--Global and overview maps--Metabolic pathways

[denovogenes]_100862	69.74	1e-126	cgo:Corgl_0270	K13292	lgt, umpA	phosphatidylglycerol:prolipoprotein diacylglycerol transferase	2.-.-.-	--

[denovogenes]_100864	66.91	2e-137	lir:LAW_00739	K02823	pyrDII	dihydroorotate dehydrogenase electron transfer subunit	--	Metabolism--Nucleotide metabolism--Pyrimidine metabolism|Metabolism--Global and overview maps--Metabolic pathways

[denovogenes]_100866	100.00	0.0	paec:M802_3495	K05781	phnK	putative phosphonate transport system ATP-binding protein	--	--

[denovogenes]_100868	100.00	0.0	fpla:A4U99_10330	K01449	E3.5.1.28C, cwlJ, sleB	N-acetylmuramoyl-L-alanine amidase	3.5.1.28	--

[denovogenes]_100872	98.15	0.0	lacy:A4V08_12270	K05815	ugpE	sn-glycerol 3-phosphate transport system permease protein	--	Environmental Information Processing--Membrane transport--ABC transporters

[denovogenes]_100874	100.00	0.0	paeo:M801_4721	K17103	CHO1, pssA	CDP-diacylglycerol---serine O-phosphatidyltransferase	2.7.8.8	Metabolism--Amino acid metabolism--Glycine, serine and threonine metabolism|Metabolism--Lipid metabolism--Glycerophospholipid metabolism|Metabolism--Global and overview maps--Metabolic pathways|Metabolism--Global and overview maps--Biosynthesis of secondary metabolites

[denovogenes]_100877	85.13	8e-162	pdi:BDI_0097	K02001	proW	glycine betaine/proline transport system permease protein	--	Environmental Information Processing--Membrane transport--ABC transporters

[denovogenes]_100879	71.64	5e-146	csh:Closa_3643	K10710	frlD	fructoselysine 6-kinase	2.7.1.-	--

[denovogenes]_100883	97.05	0.0	pdi:BDI_0715	K00919	ispE	4-diphosphocytidyl-2-C-methyl-D-erythritol kinase	2.7.1.148	Metabolism--Metabolism of terpenoids and polyketides--Terpenoid backbone biosynthesis|Metabolism--Global and overview maps--Metabolic pathways|Metabolism--Global and overview maps--Biosynthesis of secondary metabolites|Metabolism--Global and overview maps--Biosynthesis of antibiotics

[denovogenes]_100884	81.48	8e-158	bur:Bcep18194_B3062	K00130	betB, gbsA	betaine-aldehyde dehydrogenase	1.2.1.8	Metabolism--Amino acid metabolism--Glycine, serine and threonine metabolism|Metabolism--Global and overview maps--Metabolic pathways

[denovogenes]_100885	93.33	9e-180	csh:Closa_1845	K03706	codY	transcriptional pleiotropic repressor	--	--

[denovogenes]_100886	70.74	5e-127	smf:Smon_1300	K02795	PTS-Man-EIIC, manY	PTS system, mannose-specific IIC component	--	Metabolism--Carbohydrate metabolism--Fructose and mannose metabolism|Metabolism--Carbohydrate metabolism--Amino sugar and nucleotide sugar metabolism|Metabolism--Global and overview maps--Metabolic pathways|Environmental Information Processing--Membrane transport--Phosphotransferase system (PTS)

[denovogenes]_100887	77.49	1e-161	csh:Closa_4275	K19510	gfrF	fructoselysine-6-phosphate deglycase	--	--

[denovogenes]_100888	76.81	6e-144	csh:Closa_0834	K06147	ABCB-BAC	ATP-binding cassette, subfamily B, bacterial	--	--

[denovogenes]_100889	100.00	0.0	paeo:M801_817	K07090	K07090	uncharacterized protein	--	--

[denovogenes]_10089	99.86	0.0	bxy:BXY_18580	K03046	rpoC	DNA-directed RNA polymerase subunit beta'	2.7.7.6	Metabolism--Nucleotide metabolism--Purine metabolism|Metabolism--Nucleotide metabolism--Pyrimidine metabolism|Metabolism--Global and overview maps--Metabolic pathways|Genetic Information Processing--Transcription--RNA polymerase

[denovogenes]_100891	100.00	0.0	paem:U769_27020	K02314	dnaB	replicative DNA helicase	3.6.4.12	Genetic Information Processing--Replication and repair--DNA replication|Cellular Processes--Cell growth and death--Cell cycle - Caulobacter

[denovogenes]_100892	81.25	4e-164	pdi:BDI_1755	K13051	iaaA, ASRGL1	beta-aspartyl-peptidase (threonine type)	3.4.19.5	--

[denovogenes]_100893	76.32	2e-120	olo:ADJ70_04955	K20490	nisF, spaF, cprA, epiF	lantibiotic transport system ATP-binding protein	--	Environmental Information Processing--Membrane transport--ABC transporters|Environmental Information Processing--Signal transduction--Two-component system|Cellular Processes--Cellular community - prokaryotes--Quorum sensing

[denovogenes]_100894	99.63	0.0	blz:BLGT_01725	K02112	ATPF1B, atpD	F-type H+-transporting ATPase subunit beta	3.6.3.14	Metabolism--Energy metabolism--Oxidative phosphorylation|Metabolism--Energy metabolism--Photosynthesis|Metabolism--Global and overview maps--Metabolic pathways

[denovogenes]_100895	100.00	0.0	ecv:APECO1_499	K07455	recT	recombination protein RecT	--	--

[denovogenes]_100898	100.00	0.0	paec:M802_817	K09788	prpF	2-methylaconitate isomerase	5.2.1.-	Metabolism--Carbohydrate metabolism--Propanoate metabolism

[denovogenes]_1009	98.63	0.0	efd:EFD32_0057	K01119	cpdB	2',3'-cyclic-nucleotide 2'-phosphodiesterase / 3'-nucleotidase	3.1.4.16 3.1.3.6	Metabolism--Nucleotide metabolism--Purine metabolism|Metabolism--Nucleotide metabolism--Pyrimidine metabolism

[denovogenes]_10090	99.71	0.0	bvu:BVU_0486	K03546	sbcC	exonuclease SbcC	--	--

[denovogenes]_100900	83.76	1e-161	csh:Closa_0061	K02026	ABC.MS.P1	multiple sugar transport system permease protein	--	--

[denovogenes]_100902	97.27	0.0	lacy:A4V08_12875	K00978	rfbF	glucose-1-phosphate cytidylyltransferase	2.7.7.33	Metabolism--Carbohydrate metabolism--Starch and sucrose metabolism|Metabolism--Carbohydrate metabolism--Amino sugar and nucleotide sugar metabolism|Metabolism--Global and overview maps--Metabolic pathways

[denovogenes]_100904	50.94	5e-93	esi:Exig_2824	K06221	dkgA	2,5-diketo-D-gluconate reductase A	1.1.1.346	--

[denovogenes]_100906	86.30	5e-163	bcel:BcellWH2_02085	K02856	rhaT	L-rhamnose-H+ transport protein	--	--

[denovogenes]_100907	99.26	0.0	boa:Bovatus_02632	K00286	proC	pyrroline-5-carboxylate reductase	1.5.1.2	Metabolism--Amino acid metabolism--Arginine and proline metabolism|Metabolism--Global and overview maps--Metabolic pathways|Metabolism--Global and overview maps--Biosynthesis of secondary metabolites|Metabolism--Global and overview maps--Biosynthesis of antibiotics|Metabolism--Global and overview maps--Biosynthesis of amino acids

[denovogenes]_100908	60.29	2e-115	shc:Shell_1120	K02031	ABC.PE.A	peptide/nickel transport system ATP-binding protein	--	Cellular Processes--Cellular community - prokaryotes--Quorum sensing

[denovogenes]_10091	51.52	0.0	tpk:JO40_04375	K15771	ganP	arabinogalactan oligomer / maltooligosaccharide transport system permease protein	--	Environmental Information Processing--Membrane transport--ABC transporters

[denovogenes]_100912	99.63	0.0	paeg:AI22_18435	K05820	hcaT	MFS transporter, PPP family, 3-phenylpropionic acid transporter	--	--

[denovogenes]_100913	100.00	0.0	cif:AL515_02105	K00124	fdoH	formate dehydrogenase iron-sulfur subunit	--	Metabolism--Carbohydrate metabolism--Glyoxylate and dicarboxylate metabolism|Metabolism--Energy metabolism--Methane metabolism|Metabolism--Global and overview maps--Metabolic pathways|Metabolism--Global and overview maps--Microbial metabolism in diverse environments|Metabolism--Global and overview maps--Carbon metabolism

[denovogenes]_100914	100.00	0.0	cfd:CFNIH1_03235	K06041	kdsD, kpsF	arabinose-5-phosphate isomerase	5.3.1.13	Metabolism--Glycan biosynthesis and metabolism--Lipopolysaccharide biosynthesis|Metabolism--Global and overview maps--Metabolic pathways

[denovogenes]_100915	40.32	7e-22	rmg:Rhom172_1718	K02014	TC.FEV.OM	iron complex outermembrane recepter protein	--	--

[denovogenes]_100918	81.92	4e-173	osp:Odosp_1505	K01425	glsA, GLS	glutaminase	3.5.1.2	Metabolism--Amino acid metabolism--Arginine biosynthesis|Metabolism--Amino acid metabolism--Alanine, aspartate and glutamate metabolism|Metabolism--Metabolism of other amino acids--D-Glutamine and D-glutamate metabolism|Metabolism--Global and overview maps--Metabolic pathways|Organismal Systems--Nervous system--Glutamatergic synapse|Organismal Systems--Nervous system--GABAergic synapse|Organismal Systems--Excretory system--Proximal tubule bicarbonate reclamation|Human Diseases--Cancers: Overview--MicroRNAs in cancer|Human Diseases--Cancers: Overview--Central carbon metabolism in cancer

[denovogenes]_10092	100.00	0.0	bfs:BF9343_0320	K01190	lacZ	beta-galactosidase	3.2.1.23	Metabolism--Carbohydrate metabolism--Galactose metabolism|Metabolism--Glycan biosynthesis and metabolism--Other glycan degradation|Metabolism--Lipid metabolism--Sphingolipid metabolism|Metabolism--Global and overview maps--Metabolic pathways

[denovogenes]_100921	52.83	7e-101	bcel:BcellWH2_01825	K01190	lacZ	beta-galactosidase	3.2.1.23	Metabolism--Carbohydrate metabolism--Galactose metabolism|Metabolism--Glycan biosynthesis and metabolism--Other glycan degradation|Metabolism--Lipid metabolism--Sphingolipid metabolism|Metabolism--Global and overview maps--Metabolic pathways

[denovogenes]_100922	97.79	0.0	bcel:BcellWH2_03498	K03832	tonB	periplasmic protein TonB	--	--

[denovogenes]_100925	43.75	7e-78	raq:Rahaq2_3333	K06223	dam	DNA adenine methylase	2.1.1.72	Genetic Information Processing--Replication and repair--Mismatch repair

[denovogenes]_100929	77.41	2e-150	csh:Closa_3058	K02034	ABC.PE.P1	peptide/nickel transport system permease protein	--	Cellular Processes--Cellular community - prokaryotes--Quorum sensing

[denovogenes]_10093	69.22	0.0	cbut:ATN24_00345	K02355	fusA, GFM, EFG	elongation factor G	--	--

[denovogenes]_100931	99.63	0.0	pdf:CD630DERM_28720	K16850	uxaA2	altronate dehydratase large subunit	4.2.1.7	Metabolism--Carbohydrate metabolism--Pentose and glucuronate interconversions|Metabolism--Global and overview maps--Metabolic pathways

[denovogenes]_100933	99.63	0.0	bcel:BcellWH2_00942	K00174	korA, oorA, oforA	2-oxoglutarate/2-oxoacid ferredoxin oxidoreductase subunit alpha	1.2.7.3 1.2.7.11	Metabolism--Carbohydrate metabolism--Glycolysis / Gluconeogenesis|Metabolism--Carbohydrate metabolism--Citrate cycle (TCA cycle)|Metabolism--Carbohydrate metabolism--Pyruvate metabolism|Metabolism--Carbohydrate metabolism--Butanoate metabolism|Metabolism--Energy metabolism--Carbon fixation pathways in prokaryotes|Metabolism--Global and overview maps--Metabolic pathways|Metabolism--Global and overview maps--Microbial metabolism in diverse environments|Metabolism--Global and overview maps--Biosynthesis of antibiotics|Metabolism--Global and overview maps--Carbon metabolism

[denovogenes]_100938	79.41	1e-147	erb:A4V01_07330	K00865	glxK, garK	glycerate 2-kinase	2.7.1.165	Metabolism--Amino acid metabolism--Glycine, serine and threonine metabolism|Metabolism--Lipid metabolism--Glycerolipid metabolism|Metabolism--Carbohydrate metabolism--Glyoxylate and dicarboxylate metabolism|Metabolism--Global and overview maps--Metabolic pathways|Metabolism--Global and overview maps--Microbial metabolism in diverse environments|Metabolism--Global and overview maps--Biosynthesis of antibiotics

[denovogenes]_100939	70.26	3e-132	pvl:AOB99_15955	K15587	nikD	nickel transport system ATP-binding protein	3.6.3.24	Environmental Information Processing--Membrane transport--ABC transporters

[denovogenes]_10094	58.78	0.0	ent:Ent638_3829	K00705	malQ	4-alpha-glucanotransferase	2.4.1.25	Metabolism--Carbohydrate metabolism--Starch and sucrose metabolism|Metabolism--Global and overview maps--Metabolic pathways

[denovogenes]_100940	92.19	0.0	pvl:AOB99_04980	K03820	lnt	apolipoprotein N-acyltransferase	2.3.1.-	--

[denovogenes]_100945	69.58	4e-128	boa:Bovatus_02659	K01534	zntA	Cd2+/Zn2+-exporting ATPase	3.6.3.3 3.6.3.5	--

[denovogenes]_100948	99.26	0.0	kqu:AVR78_07190	K00611	OTC, argF, argI	ornithine carbamoyltransferase	2.1.3.3	Metabolism--Amino acid metabolism--Arginine biosynthesis|Metabolism--Global and overview maps--Metabolic pathways|Metabolism--Global and overview maps--Biosynthesis of secondary metabolites|Metabolism--Global and overview maps--Biosynthesis of antibiotics|Metabolism--Global and overview maps--Biosynthesis of amino acids

[denovogenes]_100950	47.86	4e-83	ldb:Ldb0617	K16203	dppA1	D-amino peptidase	3.4.11.-	--

[denovogenes]_100951	100.00	0.0	pdi:BDI_0667	K03284	corA	magnesium transporter	--	--

[denovogenes]_100953	86.76	6e-178	bcel:BcellWH2_01442	K01183	E3.2.1.14	chitinase	3.2.1.14	Metabolism--Carbohydrate metabolism--Amino sugar and nucleotide sugar metabolism|Metabolism--Global and overview maps--Metabolic pathways

[denovogenes]_100954	90.44	2e-180	afd:Alfi_1495	K04773	sppA	protease IV	3.4.21.-	--

[denovogenes]_100955	56.85	6e-94	ssu:SSU05_1385	K17686	copA, ATP7	Cu+-exporting ATPase	3.6.3.54	Human Diseases--Drug resistance: Antineoplastic--Platinum drug resistance|Environmental Information Processing--Signal transduction--MAPK signaling pathway - plant

[denovogenes]_100956	97.43	0.0	cif:AL515_06590	K01077	E3.1.3.1, phoA, phoB	alkaline phosphatase	3.1.3.1	Metabolism--Xenobiotics biodegradation and metabolism--Aminobenzoate degradation|Metabolism--Metabolism of cofactors and vitamins--Thiamine metabolism|Metabolism--Metabolism of cofactors and vitamins--Folate biosynthesis|Metabolism--Global and overview maps--Metabolic pathways|Metabolism--Global and overview maps--Microbial metabolism in diverse environments|Environmental Information Processing--Signal transduction--Two-component system

[denovogenes]_100963	99.26	0.0	bcel:BcellWH2_04203	K03797	E3.4.21.102, prc, ctpA	carboxyl-terminal processing protease	3.4.21.102	--

[denovogenes]_100964	42.38	3e-44	pmic:NW74_04125	K08303	K08303	putative protease	3.4.-.-	Human Diseases--Infectious diseases: Bacterial--Epithelial cell signaling in Helicobacter pylori infection

[denovogenes]_100967	82.35	2e-164	pmib:BB2000_3152	K00375	K00375	GntR family transcriptional regulator / MocR family aminotransferase	--	--

[denovogenes]_100968	87.13	2e-175	rob:CK5_29120	K01990	ABC-2.A	ABC-2 type transport system ATP-binding protein	--	--

[denovogenes]_10097	65.95	0.0	ccel:CCDG5_0790	K01915	glnA, GLUL	glutamine synthetase	6.3.1.2	Metabolism--Amino acid metabolism--Arginine biosynthesis|Metabolism--Amino acid metabolism--Alanine, aspartate and glutamate metabolism|Metabolism--Carbohydrate metabolism--Glyoxylate and dicarboxylate metabolism|Metabolism--Energy metabolism--Nitrogen metabolism|Metabolism--Global and overview maps--Metabolic pathways|Metabolism--Global and overview maps--Microbial metabolism in diverse environments|Metabolism--Global and overview maps--Biosynthesis of amino acids|Environmental Information Processing--Signal transduction--Two-component system|Organismal Systems--Nervous system--Glutamatergic synapse|Organismal Systems--Nervous system--GABAergic synapse

[denovogenes]_100970	99.63	0.0	cfd:CFNIH1_24400	K01626	E2.5.1.54, aroF, aroG, aroH	3-deoxy-7-phosphoheptulonate synthase	2.5.1.54	Metabolism--Amino acid metabolism--Phenylalanine, tyrosine and tryptophan biosynthesis|Metabolism--Global and overview maps--Metabolic pathways|Metabolism--Global and overview maps--Biosynthesis of secondary metabolites|Metabolism--Global and overview maps--Biosynthesis of antibiotics|Metabolism--Global and overview maps--Biosynthesis of amino acids|Cellular Processes--Cellular community - prokaryotes--Quorum sensing

[denovogenes]_100972	100.00	0.0	cif:AL515_05270	K03590	ftsA	cell division protein FtsA	--	Cellular Processes--Cell growth and death--Cell cycle - Caulobacter

[denovogenes]_100974	77.57	5e-158	sri:SELR_08720	K01740	metY	O-acetylhomoserine (thiol)-lyase	2.5.1.49	Metabolism--Amino acid metabolism--Cysteine and methionine metabolism|Metabolism--Global and overview maps--Metabolic pathways

[denovogenes]_100976	97.79	0.0	lrc:LOCK908_2896	K02029	ABC.PA.P	polar amino acid transport system permease protein	--	--

[denovogenes]_100976	97.79	0.0	lrc:LOCK908_2896	K02030	ABC.PA.S	polar amino acid transport system substrate-binding protein	--	--

[denovogenes]_100977	66.18	4e-135	bfi:CIY_07080	K02035	ABC.PE.S	peptide/nickel transport system substrate-binding protein	--	Cellular Processes--Cellular community - prokaryotes--Quorum sensing

[denovogenes]_100981	47.83	1e-71	agl:PYTT_1124	K01686	uxuA	mannonate dehydratase	4.2.1.8	Metabolism--Carbohydrate metabolism--Pentose and glucuronate interconversions|Metabolism--Global and overview maps--Metabolic pathways

[denovogenes]_100982	94.83	0.0	boa:Bovatus_03745	K07106	murQ	N-acetylmuramic acid 6-phosphate etherase	4.2.1.126	Metabolism--Carbohydrate metabolism--Amino sugar and nucleotide sugar metabolism|Metabolism--Global and overview maps--Metabolic pathways

[denovogenes]_100983	50.92	3e-96	erb:A4V01_12325	K07699	spo0A	two-component system, response regulator, stage 0 sporulation protein A	--	Environmental Information Processing--Signal transduction--Two-component system|Cellular Processes--Cellular community - prokaryotes--Quorum sensing

[denovogenes]_100985	43.23	1e-61	lli:uc509_1342	K00563	rlmA1	23S rRNA (guanine745-N1)-methyltransferase	2.1.1.187	--

[denovogenes]_100988	99.63	0.0	boa:Bovatus_02719	K03832	tonB	periplasmic protein TonB	--	--

[denovogenes]_100989	84.31	7e-134	asb:RATSFB_0276	K02795	PTS-Man-EIIC, manY	PTS system, mannose-specific IIC component	--	Metabolism--Carbohydrate metabolism--Fructose and mannose metabolism|Metabolism--Carbohydrate metabolism--Amino sugar and nucleotide sugar metabolism|Metabolism--Global and overview maps--Metabolic pathways|Environmental Information Processing--Membrane transport--Phosphotransferase system (PTS)

[denovogenes]_10099	89.49	0.0	btho:Btheta7330_02037	K00937	ppk	polyphosphate kinase	2.7.4.1	Metabolism--Energy metabolism--Oxidative phosphorylation|Genetic Information Processing--Folding, sorting and degradation--RNA degradation

[denovogenes]_100991	41.95	4e-74	ag:AAC27537	K18788	wbbD	UDP-Gal:alpha-D-GlcNAc-diphosphoundecaprenol beta-1,3-galactosyltransferase	2.4.1.303	--

[denovogenes]_100994	40.98	6e-61	fbt:D770_24445	K08138	xylE	MFS transporter, SP family, xylose:H+ symportor	--	--

[denovogenes]_10100	75.29	0.0	bxy:BXY_34000	K03169	topB	DNA topoisomerase III	5.99.1.2	--

[denovogenes]_101000	100.00	0.0	efe:EFER_4037	K02108	ATPF0A, atpB	F-type H+-transporting ATPase subunit a	--	Metabolism--Energy metabolism--Oxidative phosphorylation|Metabolism--Energy metabolism--Photosynthesis|Metabolism--Global and overview maps--Metabolic pathways

[denovogenes]_101002	100.00	0.0	pvl:AOB99_12390	K16868	tehB	tellurite methyltransferase	2.1.1.265	--

[denovogenes]_101003	40.00	8e-20	bthi:BTK_27880	K16698	tuaG	teichuronic acid biosynthesis glycosyltransferase TuaG	2.4.-.-	--

[denovogenes]_101005	100.00	0.0	pmr:PMI2739	K05539	dusA	tRNA-dihydrouridine synthase A	1.-.-.-	--

[denovogenes]_101008	92.19	1e-175	vpr:Vpar_0238	K09816	znuB	zinc transport system permease protein	--	Environmental Information Processing--Membrane transport--ABC transporters

[denovogenes]_10101	92.07	0.0	boa:Bovatus_01952	K00937	ppk	polyphosphate kinase	2.7.4.1	Metabolism--Energy metabolism--Oxidative phosphorylation|Genetic Information Processing--Folding, sorting and degradation--RNA degradation

[denovogenes]_101010	40.00	3e-60	anl:GFC29_1062	K01011	TST, MPST, sseA	thiosulfate/3-mercaptopyruvate sulfurtransferase	2.8.1.1 2.8.1.2	Metabolism--Amino acid metabolism--Cysteine and methionine metabolism|Metabolism--Energy metabolism--Sulfur metabolism|Metabolism--Global and overview maps--Metabolic pathways|Metabolism--Global and overview maps--Microbial metabolism in diverse environments|Genetic Information Processing--Folding, sorting and degradation--Sulfur relay system

[denovogenes]_101013	65.88	5e-122	bprl:CL2_30910	K03438	mraW, rsmH	16S rRNA (cytosine1402-N4)-methyltransferase	2.1.1.199	--

[denovogenes]_101015	65.94	5e-129	fnu:FN1133	K01443	nagA, AMDHD2	N-acetylglucosamine-6-phosphate deacetylase	3.5.1.25	Metabolism--Carbohydrate metabolism--Amino sugar and nucleotide sugar metabolism|Metabolism--Global and overview maps--Biosynthesis of antibiotics

[denovogenes]_101017	50.22	1e-72	vpr:Vpar_0661	K02453	gspD	general secretion pathway protein D	--	Environmental Information Processing--Membrane transport--Bacterial secretion system|Cellular Processes--Cellular community - prokaryotes--Biofilm formation - Vibrio cholerae

[denovogenes]_101018	91.51	0.0	bfg:BF638R_0779	K00978	rfbF	glucose-1-phosphate cytidylyltransferase	2.7.7.33	Metabolism--Carbohydrate metabolism--Starch and sucrose metabolism|Metabolism--Carbohydrate metabolism--Amino sugar and nucleotide sugar metabolism|Metabolism--Global and overview maps--Metabolic pathways

[denovogenes]_10102	92.37	0.0	boa:Bovatus_02918	K01284	dcp	peptidyl-dipeptidase Dcp	3.4.15.5	--

[denovogenes]_101021	73.13	3e-136	pgb:H744_1c0298	K01243	mtnN, mtn, pfs	adenosylhomocysteine nucleosidase	3.2.2.9	Metabolism--Amino acid metabolism--Cysteine and methionine metabolism|Metabolism--Global and overview maps--Metabolic pathways|Metabolism--Global and overview maps--Biosynthesis of amino acids

[denovogenes]_101022	98.89	0.0	mmk:MU9_683	K02358	tuf, TUFM	elongation factor Tu	--	--

[denovogenes]_101024	69.85	4e-139	bfr:BF2005	K07011	K07011	uncharacterized protein	--	--

[denovogenes]_101027	43.77	3e-66	cbt:CLH_2931	K07271	licD	lipopolysaccharide cholinephosphotransferase	2.7.8.-	--

[denovogenes]_101029	100.00	0.0	mmk:MU9_2091	K03609	minD	septum site-determining protein MinD	--	--

[denovogenes]_101035	99.59	4e-178	mmk:MU9_1307	K05807	bamD	outer membrane protein assembly factor BamD	--	--

[denovogenes]_101036	71.48	3e-140	ene:ENT_05440	K01776	murI	glutamate racemase	5.1.1.3	Metabolism--Metabolism of other amino acids--D-Glutamine and D-glutamate metabolism|Metabolism--Global and overview maps--Metabolic pathways

[denovogenes]_101039	100.00	0.0	bfb:VU15_11930	K01609	trpC	indole-3-glycerol phosphate synthase	4.1.1.48	Metabolism--Amino acid metabolism--Phenylalanine, tyrosine and tryptophan biosynthesis|Metabolism--Global and overview maps--Metabolic pathways|Metabolism--Global and overview maps--Biosynthesis of secondary metabolites|Metabolism--Global and overview maps--Biosynthesis of antibiotics|Metabolism--Global and overview maps--Biosynthesis of amino acids

[denovogenes]_101040	100.00	0.0	mmk:MU9_888	K02528	ksgA	16S rRNA (adenine1518-N6/adenine1519-N6)-dimethyltransferase	2.1.1.182	--

[denovogenes]_101041	100.00	0.0	bfb:VU15_04210	K02564	nagB, GNPDA	glucosamine-6-phosphate deaminase	3.5.99.6	Metabolism--Carbohydrate metabolism--Amino sugar and nucleotide sugar metabolism|Metabolism--Global and overview maps--Metabolic pathways

[denovogenes]_101042	52.06	1e-98	ecas:ECBG_02341	K06896	mapP	maltose 6'-phosphate phosphatase	3.1.3.90	Metabolism--Carbohydrate metabolism--Starch and sucrose metabolism

[denovogenes]_101043	60.62	8e-114	clt:CM240_2549	K04486	E3.1.3.15B	histidinol-phosphatase (PHP family)	3.1.3.15	Metabolism--Amino acid metabolism--Histidine metabolism|Metabolism--Global and overview maps--Metabolic pathways|Metabolism--Global and overview maps--Biosynthesis of secondary metabolites|Metabolism--Global and overview maps--Biosynthesis of amino acids

[denovogenes]_101044	99.63	0.0	bfg:BF638R_2781	K02040	pstS	phosphate transport system substrate-binding protein	--	Environmental Information Processing--Membrane transport--ABC transporters|Environmental Information Processing--Signal transduction--Two-component system|Human Diseases--Infectious diseases: Bacterial--Tuberculosis

[denovogenes]_101046	56.82	2e-107	ckr:CKR_0278	K00286	proC	pyrroline-5-carboxylate reductase	1.5.1.2	Metabolism--Amino acid metabolism--Arginine and proline metabolism|Metabolism--Global and overview maps--Metabolic pathways|Metabolism--Global and overview maps--Biosynthesis of secondary metabolites|Metabolism--Global and overview maps--Biosynthesis of antibiotics|Metabolism--Global and overview maps--Biosynthesis of amino acids

[denovogenes]_101047	75.56	2e-143	lhi:JP39_02095	K18104	abcA, bmrA	ATP-binding cassette, subfamily B, bacterial AbcA/BmrA	3.6.3.44	Human Diseases--Drug resistance: Antimicrobial--beta-Lactam resistance|Environmental Information Processing--Membrane transport--ABC transporters

[denovogenes]_101049	91.48	0.0	eft:M395_08280	K19509	PTS-Gfr-EIID, gfrD	PTS system, fructoselysine/glucoselysine-specific IID component	--	Environmental Information Processing--Membrane transport--Phosphotransferase system (PTS)

[denovogenes]_101050	72.31	8e-140	lacy:A4V08_31120	K02556	motA	chemotaxis protein MotA	--	Environmental Information Processing--Signal transduction--Two-component system|Cellular Processes--Cell motility--Bacterial chemotaxis|Cellular Processes--Cell motility--Flagellar assembly

[denovogenes]_101051	67.41	2e-142	lacy:A4V08_31035	K00575	cheR	chemotaxis protein methyltransferase CheR	2.1.1.80	Environmental Information Processing--Signal transduction--Two-component system|Cellular Processes--Cell motility--Bacterial chemotaxis

[denovogenes]_101052	100.00	0.0	bfr:BF1122	K05810	yfiH	laccase domain-containing protein	--	--

[denovogenes]_101053	40.16	3e-14	pre:PCA10_17880	K03566	gcvA	LysR family transcriptional regulator, glycine cleavage system transcriptional activator	--	Cellular Processes--Cellular community - prokaryotes--Biofilm formation - Escherichia coli

[denovogenes]_101056	100.00	0.0	stb:SGPB_0304	K21064	ycsE, yitU, ywtE	5-amino-6-(5-phospho-D-ribitylamino)uracil phosphatase	3.1.3.104	Metabolism--Metabolism of cofactors and vitamins--Riboflavin metabolism|Metabolism--Global and overview maps--Metabolic pathways|Metabolism--Global and overview maps--Biosynthesis of secondary metabolites

[denovogenes]_101059	70.48	2e-142	cbk:CLL_A1156	K01962	accA	acetyl-CoA carboxylase carboxyl transferase subunit alpha	6.4.1.2	Metabolism--Lipid metabolism--Fatty acid biosynthesis|Metabolism--Carbohydrate metabolism--Pyruvate metabolism|Metabolism--Carbohydrate metabolism--Propanoate metabolism|Metabolism--Energy metabolism--Carbon fixation pathways in prokaryotes|Metabolism--Global and overview maps--Metabolic pathways|Metabolism--Global and overview maps--Biosynthesis of secondary metabolites|Metabolism--Global and overview maps--Microbial metabolism in diverse environments|Metabolism--Global and overview maps--Biosynthesis of antibiotics|Metabolism--Global and overview maps--Carbon metabolism|Metabolism--Global and overview maps--Fatty acid metabolism

[denovogenes]_101060	72.93	2e-141	rbr:RBR_02340	K00941	thiD	hydroxymethylpyrimidine/phosphomethylpyrimidine kinase	2.7.1.49 2.7.4.7	Metabolism--Metabolism of cofactors and vitamins--Thiamine metabolism|Metabolism--Global and overview maps--Metabolic pathways

[denovogenes]_101062	51.29	1e-92	eft:M395_07450	K07260	vanY	D-alanyl-D-alanine carboxypeptidase	3.4.16.4	Metabolism--Glycan biosynthesis and metabolism--Peptidoglycan biosynthesis|Human Diseases--Drug resistance: Antimicrobial--Vancomycin resistance|Environmental Information Processing--Signal transduction--Two-component system

[denovogenes]_101064	54.62	5e-102	amt:Amet_3994	K03496	parA, soj	chromosome partitioning protein	--	--

[denovogenes]_101065	99.63	0.0	bfb:VU15_18990	K03561	exbB	biopolymer transport protein ExbB	--	--

[denovogenes]_101067	99.26	0.0	ene:ENT_16970	K09692	tagG	teichoic acid transport system permease protein	--	Environmental Information Processing--Membrane transport--ABC transporters

[denovogenes]_101069	100.00	0.0	efq:DR75_86	K09773	ppsR	[pyruvate, water dikinase]-phosphate phosphotransferase / [pyruvate, water dikinase] kinase	2.7.4.28 2.7.11.33	--

[denovogenes]_10107	49.71	0.0	fpla:A4U99_05380	K03581	recD	exodeoxyribonuclease V alpha subunit	3.1.11.5	Genetic Information Processing--Replication and repair--Homologous recombination

[denovogenes]_101071	41.18	4e-17	goq:ACH46_03010	K01066	aes	acetyl esterase	3.1.1.-	--

[denovogenes]_101072	45.45	3e-61	scc:Spico_1756	K07090	K07090	uncharacterized protein	--	--

[denovogenes]_101075	82.59	9e-164	pdf:CD630DERM_12080	K06442	tlyA	23S rRNA (cytidine1920-2'-O)/16S rRNA (cytidine1409-2'-O)-methyltransferase	2.1.1.226 2.1.1.227	--

[denovogenes]_101076	99.26	0.0	stb:SGPB_1451	K10039	ABC.GLN1.S	putative glutamine transport system substrate-binding protein	--	Environmental Information Processing--Membrane transport--ABC transporters

[denovogenes]_101077	100.00	0.0	stb:SGPB_1437	K08986	ycgQ	putative membrane protein	--	--

[denovogenes]_101078	100.00	0.0	pvl:AOB99_02100	K02575	NRT, narK, nrtP, nasA	MFS transporter, NNP family, nitrate/nitrite transporter	--	Metabolism--Energy metabolism--Nitrogen metabolism

[denovogenes]_101081	99.63	0.0	smn:SMA_0846	K03783	punA, PNP	purine-nucleoside phosphorylase	2.4.2.1	Metabolism--Nucleotide metabolism--Purine metabolism|Metabolism--Nucleotide metabolism--Pyrimidine metabolism|Metabolism--Metabolism of cofactors and vitamins--Nicotinate and nicotinamide metabolism|Metabolism--Global and overview maps--Metabolic pathways|Metabolism--Global and overview maps--Biosynthesis of secondary metabolites

[denovogenes]_101082	86.62	6e-180	cld:CLSPO_c03170	K01232	glvA	maltose-6'-phosphate glucosidase	3.2.1.122	Metabolism--Carbohydrate metabolism--Starch and sucrose metabolism

[denovogenes]_101084	100.00	0.0	pvl:AOB99_14205	K02034	ABC.PE.P1	peptide/nickel transport system permease protein	--	Cellular Processes--Cellular community - prokaryotes--Quorum sensing

[denovogenes]_101085	45.28	4e-66	ctc:CTC_00821	K02238	comEC	competence protein ComEC	--	--

[denovogenes]_101086	100.00	0.0	pvl:AOB99_02540	K02065	mlaF, linL, mkl	phospholipid/cholesterol/gamma-HCH transport system ATP-binding protein	--	Environmental Information Processing--Membrane transport--ABC transporters

[denovogenes]_101087	95.56	0.0	cif:AL515_02775	K00014	aroE	shikimate dehydrogenase	1.1.1.25	Metabolism--Amino acid metabolism--Phenylalanine, tyrosine and tryptophan biosynthesis|Metabolism--Global and overview maps--Metabolic pathways|Metabolism--Global and overview maps--Biosynthesis of secondary metabolites|Metabolism--Global and overview maps--Biosynthesis of antibiotics|Metabolism--Global and overview maps--Biosynthesis of amino acids

[denovogenes]_101088	93.33	4e-170	cko:CKO_03622	K05874	tsr	methyl-accepting chemotaxis protein I, serine sensor receptor	--	Environmental Information Processing--Signal transduction--Two-component system|Cellular Processes--Cell motility--Bacterial chemotaxis

[denovogenes]_10109	82.99	0.0	bdh:GV66_15625	K03628	rho	transcription termination factor Rho	--	Genetic Information Processing--Folding, sorting and degradation--RNA degradation

[denovogenes]_101091	100.00	0.0	pvl:AOB99_07760	K03609	minD	septum site-determining protein MinD	--	--

[denovogenes]_101094	40.89	5e-60	ols:Olsu_1293	K00278	nadB	L-aspartate oxidase	1.4.3.16	Metabolism--Amino acid metabolism--Alanine, aspartate and glutamate metabolism|Metabolism--Metabolism of cofactors and vitamins--Nicotinate and nicotinamide metabolism|Metabolism--Global and overview maps--Metabolic pathways

[denovogenes]_101096	67.91	3e-133	cle:Clole_2934	K11071	potB	spermidine/putrescine transport system permease protein	--	Environmental Information Processing--Membrane transport--ABC transporters

[denovogenes]_101098	50.19	2e-87	cpr:CPR_0416	K02529	lacI, galR	LacI family transcriptional regulator	--	--

[denovogenes]_101099	41.40	2e-47	amg:AMEC673_14330	K07497	K07497	putative transposase	--	--

[denovogenes]_1011	75.47	0.0	afn:Acfer_0343	K03043	rpoB	DNA-directed RNA polymerase subunit beta	2.7.7.6	Metabolism--Nucleotide metabolism--Purine metabolism|Metabolism--Nucleotide metabolism--Pyrimidine metabolism|Metabolism--Global and overview maps--Metabolic pathways|Genetic Information Processing--Transcription--RNA polymerase

[denovogenes]_101100	45.45	5e-79	ehr:EHR_05835	K03930	estA	putative tributyrin esterase	3.1.1.-	--

[denovogenes]_101101	100.00	0.0	ecx:EcHS_A1029	K03633	mukF	chromosome partition protein MukF	--	--

[denovogenes]_101102	41.88	1e-15	vvm:VVMO6_01884	K02169	bioC	malonyl-CoA O-methyltransferase	2.1.1.197	Metabolism--Metabolism of cofactors and vitamins--Biotin metabolism|Metabolism--Global and overview maps--Metabolic pathways

[denovogenes]_101103	82.16	8e-146	ipo:Ilyop_2564	K03388	hdrA	heterodisulfide reductase subunit A	1.8.98.1	Metabolism--Energy metabolism--Methane metabolism|Metabolism--Global and overview maps--Metabolic pathways|Metabolism--Global and overview maps--Microbial metabolism in diverse environments|Metabolism--Global and overview maps--Carbon metabolism

[denovogenes]_101106	98.52	0.0	cbut:ATN24_01625	K00931	proB	glutamate 5-kinase	2.7.2.11	Metabolism--Amino acid metabolism--Arginine and proline metabolism|Metabolism--Biosynthesis of other secondary metabolites--Carbapenem biosynthesis|Metabolism--Global and overview maps--Metabolic pathways|Metabolism--Global and overview maps--Biosynthesis of antibiotics|Metabolism--Global and overview maps--Biosynthesis of amino acids

[denovogenes]_101107	61.65	5e-116	erb:A4V01_01630	K00800	aroA	3-phosphoshikimate 1-carboxyvinyltransferase	2.5.1.19	Metabolism--Amino acid metabolism--Phenylalanine, tyrosine and tryptophan biosynthesis|Metabolism--Global and overview maps--Metabolic pathways|Metabolism--Global and overview maps--Biosynthesis of secondary metabolites|Metabolism--Global and overview maps--Biosynthesis of antibiotics|Metabolism--Global and overview maps--Biosynthesis of amino acids

[denovogenes]_101108	41.71	1e-41	ctyk:CTK_C19560	K06396	spoIIIAG	stage III sporulation protein AG	--	--

[denovogenes]_101109	42.96	1e-71	spa:M6_Spy1124	K06400	spoIVCA	site-specific DNA recombinase	--	--

[denovogenes]_101112	99.63	0.0	cbut:ATN24_11900	K01999	livK	branched-chain amino acid transport system substrate-binding protein	--	Environmental Information Processing--Membrane transport--ABC transporters|Cellular Processes--Cellular community - prokaryotes--Quorum sensing

[denovogenes]_101113	92.19	0.0	krd:A3780_05900	K00615	E2.2.1.1, tktA, tktB	transketolase	2.2.1.1	Metabolism--Carbohydrate metabolism--Pentose phosphate pathway|Metabolism--Energy metabolism--Carbon fixation in photosynthetic organisms|Metabolism--Metabolism of terpenoids and polyketides--Biosynthesis of ansamycins|Metabolism--Global and overview maps--Metabolic pathways|Metabolism--Global and overview maps--Biosynthesis of secondary metabolites|Metabolism--Global and overview maps--Microbial metabolism in diverse environments|Metabolism--Global and overview maps--Biosynthesis of antibiotics|Metabolism--Global and overview maps--Carbon metabolism|Metabolism--Global and overview maps--Biosynthesis of amino acids

[denovogenes]_101116	67.90	4e-137	csr:Cspa_c14680	K02227	cbiB, cobD	adenosylcobinamide-phosphate synthase	6.3.1.10	Metabolism--Metabolism of cofactors and vitamins--Porphyrin and chlorophyll metabolism|Metabolism--Global and overview maps--Metabolic pathways

[denovogenes]_101117	85.61	5e-172	fhw:RN87_04330	K01639	E4.1.3.3, nanA, NPL	N-acetylneuraminate lyase	4.1.3.3	Metabolism--Carbohydrate metabolism--Amino sugar and nucleotide sugar metabolism

[denovogenes]_101119	71.59	1e-136	csr:Cspa_c23360	K03488	licT, bglG	beta-glucoside operon transcriptional antiterminator	--	--

[denovogenes]_10112	98.71	0.0	cfd:CFNIH1_17935	K08348	fdnG	formate dehydrogenase-N, alpha subunit	1.1.5.6	Environmental Information Processing--Signal transduction--Two-component system

[denovogenes]_101120	65.06	2e-117	ipo:Ilyop_0329	K10440	rbsC	ribose transport system permease protein	--	Environmental Information Processing--Membrane transport--ABC transporters

[denovogenes]_101122	100.00	0.0	ecx:EcHS_A1829	K06447	astD	succinylglutamic semialdehyde dehydrogenase	1.2.1.71	Metabolism--Amino acid metabolism--Arginine and proline metabolism|Metabolism--Global and overview maps--Metabolic pathways

[denovogenes]_101124	68.73	1e-121	cpf:CPF_1621	K03308	TC.NSS	neurotransmitter:Na+ symporter, NSS family	--	--

[denovogenes]_10113	54.19	0.0	mta:Moth_1999	K00087	ygeS, xdhA	xanthine dehydrogenase molybdenum-binding subunit	1.17.1.4	Metabolism--Nucleotide metabolism--Purine metabolism|Metabolism--Global and overview maps--Metabolic pathways|Metabolism--Global and overview maps--Microbial metabolism in diverse environments

[denovogenes]_101131	71.22	4e-139	cko:CKO_05081	K02847	waaL, rfaL	O-antigen ligase	2.4.1.-	Metabolism--Glycan biosynthesis and metabolism--Lipopolysaccharide biosynthesis|Metabolism--Global and overview maps--Metabolic pathways

[denovogenes]_101133	87.41	2e-175	cle:Clole_1334	K01886	QARS, glnS	glutaminyl-tRNA synthetase	6.1.1.18	Genetic Information Processing--Translation--Aminoacyl-tRNA biosynthesis|Metabolism--Global and overview maps--Metabolic pathways

[denovogenes]_101134	90.41	7e-178	psx:DR96_793	K03665	hflX	GTPase	--	--

[denovogenes]_101135	44.69	3e-67	dpn:BCB69_04425	K03655	recG	ATP-dependent DNA helicase RecG	3.6.4.12	Genetic Information Processing--Replication and repair--Homologous recombination

[denovogenes]_101139	51.84	1e-87	mst:Msp_0528	K01919	gshA	glutamate--cysteine ligase	6.3.2.2	Metabolism--Amino acid metabolism--Cysteine and methionine metabolism|Metabolism--Metabolism of other amino acids--Glutathione metabolism|Metabolism--Global and overview maps--Metabolic pathways

[denovogenes]_101140	51.98	9e-92	ddh:Desde_3843	K01992	ABC-2.P	ABC-2 type transport system permease protein	--	--

[denovogenes]_101141	82.23	7e-145	sri:SELR_22550	K02967	RP-S2, MRPS2, rpsB	small subunit ribosomal protein S2	--	Genetic Information Processing--Translation--Ribosome

[denovogenes]_101142	98.15	0.0	bhl:Bache_1339	K02564	nagB, GNPDA	glucosamine-6-phosphate deaminase	3.5.99.6	Metabolism--Carbohydrate metabolism--Amino sugar and nucleotide sugar metabolism|Metabolism--Global and overview maps--Metabolic pathways

[denovogenes]_101143	40.96	1e-57	aac:Aaci_2531	K13641	iclR	IclR family transcriptional regulator, acetate operon repressor	--	--

[denovogenes]_101144	45.77	1e-80	fnu:FN1255	K02566	nagD	NagD protein	--	--

[denovogenes]_101146	56.89	4e-104	hsd:SD1D_0719	K17734	aprX	serine protease AprX	3.4.21.-	--

[denovogenes]_101147	48.26	2e-71	afn:Acfer_1496	K03437	spoU	RNA methyltransferase, TrmH family	--	--

[denovogenes]_10115	98.13	0.0	btho:Btheta7330_00237	K01284	dcp	peptidyl-dipeptidase Dcp	3.4.15.5	--

[denovogenes]_101150	43.45	8e-70	epr:EPYR_01486	K16702	amsE	amylovoran biosynthesis glycosyltransferase AmsE	2.4.-.-	--

[denovogenes]_101151	96.30	0.0	bhl:Bache_1104	K03561	exbB	biopolymer transport protein ExbB	--	--

[denovogenes]_101152	72.28	2e-136	fpla:A4U99_00115	K03497	parB, spo0J	chromosome partitioning protein, ParB family	--	--

[denovogenes]_101153	60.97	6e-125	csh:Closa_3425	K00575	cheR	chemotaxis protein methyltransferase CheR	2.1.1.80	Environmental Information Processing--Signal transduction--Two-component system|Cellular Processes--Cell motility--Bacterial chemotaxis

[denovogenes]_101154	90.74	0.0	bxy:BXY_26680	K07271	licD	lipopolysaccharide cholinephosphotransferase	2.7.8.-	--

[denovogenes]_101156	93.33	7e-170	boa:Bovatus_01343	K02040	pstS	phosphate transport system substrate-binding protein	--	Environmental Information Processing--Membrane transport--ABC transporters|Environmental Information Processing--Signal transduction--Two-component system|Human Diseases--Infectious diseases: Bacterial--Tuberculosis

[denovogenes]_101157	81.85	2e-167	boa:Bovatus_04642	K05810	yfiH	laccase domain-containing protein	--	--

[denovogenes]_101158	94.44	0.0	bdh:GV66_03975	K01126	E3.1.4.46, glpQ, ugpQ	glycerophosphoryl diester phosphodiesterase	3.1.4.46	Metabolism--Lipid metabolism--Glycerophospholipid metabolism

[denovogenes]_10116	61.64	0.0	dde:Dde_2033	K01879	glyS	glycyl-tRNA synthetase beta chain	6.1.1.14	Genetic Information Processing--Translation--Aminoacyl-tRNA biosynthesis

[denovogenes]_101162	57.26	4e-90	ibu:IB211_01843c	K07098	K07098	uncharacterized protein	--	--

[denovogenes]_101164	72.96	1e-150	csh:Closa_3425	K00575	cheR	chemotaxis protein methyltransferase CheR	2.1.1.80	Environmental Information Processing--Signal transduction--Two-component system|Cellular Processes--Cell motility--Bacterial chemotaxis

[denovogenes]_101165	99.26	0.0	boa:Bovatus_04387	K02564	nagB, GNPDA	glucosamine-6-phosphate deaminase	3.5.99.6	Metabolism--Carbohydrate metabolism--Amino sugar and nucleotide sugar metabolism|Metabolism--Global and overview maps--Metabolic pathways

[denovogenes]_101166	68.40	7e-145	csh:Closa_3425	K00575	cheR	chemotaxis protein methyltransferase CheR	2.1.1.80	Environmental Information Processing--Signal transduction--Two-component system|Cellular Processes--Cell motility--Bacterial chemotaxis

[denovogenes]_101167	56.68	2e-107	rto:RTO_19760	K00858	ppnK, NADK	NAD+ kinase	2.7.1.23	Metabolism--Metabolism of cofactors and vitamins--Nicotinate and nicotinamide metabolism|Metabolism--Global and overview maps--Metabolic pathways

[denovogenes]_101168	65.30	1e-122	csr:Cspa_c08660	K17074	lysX2	putative lysine transport system permease protein	--	Environmental Information Processing--Membrane transport--ABC transporters

[denovogenes]_10117	99.86	0.0	fpla:A4U99_03295	K02400	flhA	flagellar biosynthesis protein FlhA	--	Cellular Processes--Cell motility--Flagellar assembly

[denovogenes]_101170	65.80	4e-135	cbl:CLK_0246	K00433	cpo	non-heme chloroperoxidase	1.11.1.10	--

[denovogenes]_101172	40.00	1e-45	rpm:RSPPHO_01357	K01692	paaF, echA	enoyl-CoA hydratase	4.2.1.17	Metabolism--Lipid metabolism--Fatty acid degradation|Metabolism--Amino acid metabolism--Valine, leucine and isoleucine degradation|Metabolism--Metabolism of terpenoids and polyketides--Geraniol degradation|Metabolism--Amino acid metabolism--Lysine degradation|Metabolism--Amino acid metabolism--Phenylalanine metabolism|Metabolism--Xenobiotics biodegradation and metabolism--Benzoate degradation|Metabolism--Amino acid metabolism--Tryptophan metabolism|Metabolism--Metabolism of other amino acids--beta-Alanine metabolism|Metabolism--Xenobiotics biodegradation and metabolism--Aminobenzoate degradation|Metabolism--Carbohydrate metabolism--Propanoate metabolism|Metabolism--Carbohydrate metabolism--Butanoate metabolism|Metabolism--Metabolism of terpenoids and polyketides--Limonene and pinene degradation|Metabolism--Xenobiotics biodegradation and metabolism--Caprolactam degradation|Metabolism--Global and overview maps--Metabolic pathways|Metabolism--Global and overview maps--Biosynthesis of secondary metabolites|Metabolism--Global and overview maps--Microbial metabolism in diverse environments|Metabolism--Global and overview maps--Biosynthesis of antibiotics|Metabolism--Global and overview maps--Fatty acid metabolism

[denovogenes]_101173	45.59	5e-64	agl:PYTT_1124	K01686	uxuA	mannonate dehydratase	4.2.1.8	Metabolism--Carbohydrate metabolism--Pentose and glucuronate interconversions|Metabolism--Global and overview maps--Metabolic pathways

[denovogenes]_101174	63.18	6e-107	rto:RTO_02520	K06346	jag	spoIIIJ-associated protein	--	--

[denovogenes]_101177	89.86	2e-136	cso:CLS_13040	K07816	E2.7.6.5X	putative GTP pyrophosphokinase	2.7.6.5	Metabolism--Nucleotide metabolism--Purine metabolism

[denovogenes]_101178	42.86	2e-25	doi:FH5T_12030	K02477	K02477	two-component system, LytT family, response regulator	--	--

[denovogenes]_101179	98.52	0.0	bxy:BXY_26680	K07271	licD	lipopolysaccharide cholinephosphotransferase	2.7.8.-	--

[denovogenes]_101180	64.41	6e-107	bprm:CL3_15690	K03763	DPO3A2, polC	DNA polymerase III subunit alpha, Gram-positive type	2.7.7.7	Metabolism--Nucleotide metabolism--Purine metabolism|Metabolism--Nucleotide metabolism--Pyrimidine metabolism|Metabolism--Global and overview maps--Metabolic pathways|Genetic Information Processing--Replication and repair--DNA replication|Genetic Information Processing--Replication and repair--Mismatch repair|Genetic Information Processing--Replication and repair--Homologous recombination

[denovogenes]_101182	98.89	0.0	bxy:BXY_42920	K02040	pstS	phosphate transport system substrate-binding protein	--	Environmental Information Processing--Membrane transport--ABC transporters|Environmental Information Processing--Signal transduction--Two-component system|Human Diseases--Infectious diseases: Bacterial--Tuberculosis

[denovogenes]_101184	49.63	5e-70	agl:PYTT_1124	K01686	uxuA	mannonate dehydratase	4.2.1.8	Metabolism--Carbohydrate metabolism--Pentose and glucuronate interconversions|Metabolism--Global and overview maps--Metabolic pathways

[denovogenes]_101185	44.70	2e-25	nso:NIASO_12840	K02477	K02477	two-component system, LytT family, response regulator	--	--

[denovogenes]_101186	41.03	5e-48	erl:AOC36_04380	K05366	mrcA	penicillin-binding protein 1A	2.4.1.- 3.4.-.-	Metabolism--Glycan biosynthesis and metabolism--Peptidoglycan biosynthesis|Metabolism--Global and overview maps--Metabolic pathways|Human Diseases--Drug resistance: Antimicrobial--beta-Lactam resistance

[denovogenes]_101188	67.87	2e-119	lacy:A4V08_20955	K06206	sfsA	sugar fermentation stimulation protein A	--	--

[denovogenes]_10119	64.73	0.0	fpa:FPR_13660	K07003	K07003	uncharacterized protein	--	--

[denovogenes]_101191	50.74	2e-98	osp:Odosp_3010	K07011	K07011	uncharacterized protein	--	--

[denovogenes]_101192	99.63	0.0	fpla:A4U99_17385	K05810	yfiH	laccase domain-containing protein	--	--

[denovogenes]_101194	84.81	1e-170	aoe:Clos_0382	K01611	speD, AMD1	S-adenosylmethionine decarboxylase	4.1.1.50	Metabolism--Amino acid metabolism--Cysteine and methionine metabolism|Metabolism--Amino acid metabolism--Arginine and proline metabolism|Metabolism--Global and overview maps--Metabolic pathways

[denovogenes]_101195	100.00	0.0	btho:Btheta7330_04096	K02564	nagB, GNPDA	glucosamine-6-phosphate deaminase	3.5.99.6	Metabolism--Carbohydrate metabolism--Amino sugar and nucleotide sugar metabolism|Metabolism--Global and overview maps--Metabolic pathways

[denovogenes]_101197	72.96	7e-133	cpy:Cphy_0896	K11071	potB	spermidine/putrescine transport system permease protein	--	Environmental Information Processing--Membrane transport--ABC transporters

[denovogenes]_10120	81.18	0.0	dvm:DvMF_2415	K03230	yscV, sctV, hrcV	type III secretion protein V	--	Environmental Information Processing--Membrane transport--Bacterial secretion system

[denovogenes]_101201	72.93	3e-141	ibu:IB211_02702	K00931	proB	glutamate 5-kinase	2.7.2.11	Metabolism--Amino acid metabolism--Arginine and proline metabolism|Metabolism--Biosynthesis of other secondary metabolites--Carbapenem biosynthesis|Metabolism--Global and overview maps--Metabolic pathways|Metabolism--Global and overview maps--Biosynthesis of antibiotics|Metabolism--Global and overview maps--Biosynthesis of amino acids

[denovogenes]_101202	98.52	0.0	fpla:A4U99_17560	K01995	livG	branched-chain amino acid transport system ATP-binding protein	--	Environmental Information Processing--Membrane transport--ABC transporters|Cellular Processes--Cellular community - prokaryotes--Quorum sensing

[denovogenes]_101203	75.28	3e-147	str:Sterm_3749	K06971	K06971	uncharacterized protein	--	--

[denovogenes]_101208	62.78	2e-122	bhy:BHWA1_02373	K11071	potB	spermidine/putrescine transport system permease protein	--	Environmental Information Processing--Membrane transport--ABC transporters

[denovogenes]_101209	78.54	5e-150	csh:Closa_3004	K03521	fixA, etfB	electron transfer flavoprotein beta subunit	--	--

[denovogenes]_10121	54.73	0.0	fpa:FPR_31500	K03572	mutL	DNA mismatch repair protein MutL	--	Genetic Information Processing--Replication and repair--Mismatch repair

[denovogenes]_101212	99.63	0.0	bxy:BXY_35250	K05810	yfiH	laccase domain-containing protein	--	--

[denovogenes]_101213	98.15	0.0	fpla:A4U99_00980	K00563	rlmA1	23S rRNA (guanine745-N1)-methyltransferase	2.1.1.187	--

[denovogenes]_101217	62.73	1e-119	hsd:SD1D_2200	K02392	flgG	flagellar basal-body rod protein FlgG	--	Cellular Processes--Cell motility--Flagellar assembly

[denovogenes]_101218	62.70	4e-111	pdt:Prede_2711	K19286	nfrA2	FMN reductase [NAD(P)H]	1.5.1.39	Metabolism--Metabolism of cofactors and vitamins--Riboflavin metabolism|Metabolism--Global and overview maps--Metabolic pathways

[denovogenes]_101220	72.48	9e-129	lacy:A4V08_34485	K09861	K09861	uncharacterized protein	--	--

[denovogenes]_101226	100.00	0.0	btho:Btheta7330_04447	K03832	tonB	periplasmic protein TonB	--	--

[denovogenes]_101227	100.00	0.0	bvu:BVU_1618	K03832	tonB	periplasmic protein TonB	--	--

[denovogenes]_101229	47.51	1e-80	tbe:Trebr_1227	K02013	ABC.FEV.A	iron complex transport system ATP-binding protein	3.6.3.34	Environmental Information Processing--Membrane transport--ABC transporters

[denovogenes]_10123	70.36	0.0	fpr:FP2_01730	K01915	glnA, GLUL	glutamine synthetase	6.3.1.2	Metabolism--Amino acid metabolism--Arginine biosynthesis|Metabolism--Amino acid metabolism--Alanine, aspartate and glutamate metabolism|Metabolism--Carbohydrate metabolism--Glyoxylate and dicarboxylate metabolism|Metabolism--Energy metabolism--Nitrogen metabolism|Metabolism--Global and overview maps--Metabolic pathways|Metabolism--Global and overview maps--Microbial metabolism in diverse environments|Metabolism--Global and overview maps--Biosynthesis of amino acids|Environmental Information Processing--Signal transduction--Two-component system|Organismal Systems--Nervous system--Glutamatergic synapse|Organismal Systems--Nervous system--GABAergic synapse

[denovogenes]_101230	51.87	1e-100	cle:Clole_0461	K02594	nifV	homocitrate synthase NifV	2.3.3.14	Metabolism--Carbohydrate metabolism--Pyruvate metabolism

[denovogenes]_101231	50.74	2e-86	fpla:A4U99_17385	K05810	yfiH	laccase domain-containing protein	--	--

[denovogenes]_101233	100.00	0.0	bfg:BF638R_1556	K07271	licD	lipopolysaccharide cholinephosphotransferase	2.7.8.-	--

[denovogenes]_101234	40.45	2e-61	rus:RBI_II00272	K00286	proC	pyrroline-5-carboxylate reductase	1.5.1.2	Metabolism--Amino acid metabolism--Arginine and proline metabolism|Metabolism--Global and overview maps--Metabolic pathways|Metabolism--Global and overview maps--Biosynthesis of secondary metabolites|Metabolism--Global and overview maps--Biosynthesis of antibiotics|Metabolism--Global and overview maps--Biosynthesis of amino acids

[denovogenes]_101235	54.04	4e-100	byl:A4V09_08195	K20489	nisI	lantibiotic immunity protein	--	Environmental Information Processing--Signal transduction--Two-component system|Cellular Processes--Cellular community - prokaryotes--Quorum sensing

[denovogenes]_101237	99.26	0.0	fpla:A4U99_14245	K16203	dppA1	D-amino peptidase	3.4.11.-	--

[denovogenes]_101239	69.06	4e-134	hsd:SD1D_2337	K01776	murI	glutamate racemase	5.1.1.3	Metabolism--Metabolism of other amino acids--D-Glutamine and D-glutamate metabolism|Metabolism--Global and overview maps--Metabolic pathways

[denovogenes]_10124	97.70	0.0	lacy:A4V08_02345	K03168	topA	DNA topoisomerase I	5.99.1.2	--

[denovogenes]_101240	41.74	1e-24	mph:MLP_04780	K18910	dpe, lre	D-psicose/D-tagatose/L-ribulose 3-epimerase	5.1.3.30 5.1.3.31	--

[denovogenes]_101245	97.41	0.0	bth:BT_4389	K05810	yfiH	laccase domain-containing protein	--	--

[denovogenes]_10125	98.99	0.0	bfr:BF1373	K03169	topB	DNA topoisomerase III	5.99.1.2	--

[denovogenes]_101251	82.46	6e-164	fpla:A4U99_16730	K03783	punA, PNP	purine-nucleoside phosphorylase	2.4.2.1	Metabolism--Nucleotide metabolism--Purine metabolism|Metabolism--Nucleotide metabolism--Pyrimidine metabolism|Metabolism--Metabolism of cofactors and vitamins--Nicotinate and nicotinamide metabolism|Metabolism--Global and overview maps--Metabolic pathways|Metabolism--Global and overview maps--Biosynthesis of secondary metabolites

[denovogenes]_101253	43.03	6e-53	sbh:SBI_02938	K13641	iclR	IclR family transcriptional regulator, acetate operon repressor	--	--

[denovogenes]_101254	98.14	0.0	cfd:CFNIH1_15660	K19236	ycfS	L,D-transpeptidase YcfS	--	Human Diseases--Drug resistance: Antimicrobial--Cationic antimicrobial peptide (CAMP) resistance

[denovogenes]_101256	40.17	5e-46	dca:Desca_0562	K07043	K07043	uncharacterized protein	--	--

[denovogenes]_101259	100.00	0.0	fpla:A4U99_08840	K00981	E2.7.7.41, CDS1, CDS2, cdsA	phosphatidate cytidylyltransferase	2.7.7.41	Metabolism--Lipid metabolism--Glycerophospholipid metabolism|Metabolism--Global and overview maps--Metabolic pathways|Metabolism--Global and overview maps--Biosynthesis of secondary metabolites|Environmental Information Processing--Signal transduction--Phosphatidylinositol signaling system

[denovogenes]_10126	99.14	0.0	cfd:CFNIH1_04740	K00705	malQ	4-alpha-glucanotransferase	2.4.1.25	Metabolism--Carbohydrate metabolism--Starch and sucrose metabolism|Metabolism--Global and overview maps--Metabolic pathways

[denovogenes]_101261	60.26	2e-91	bprs:CK3_13700	K03545	tig	trigger factor	--	--

[denovogenes]_101263	58.87	2e-111	dor:Desor_2116	K02013	ABC.FEV.A	iron complex transport system ATP-binding protein	3.6.3.34	Environmental Information Processing--Membrane transport--ABC transporters

[denovogenes]_101266	94.44	0.0	bhl:Bache_1104	K03561	exbB	biopolymer transport protein ExbB	--	--

[denovogenes]_101267	62.73	1e-105	bprs:CK3_01670	K02051	ABC.SN.S	NitT/TauT family transport system substrate-binding protein	--	--

[denovogenes]_101269	73.43	2e-140	cbh:CLC_2498	K02117	ATPVA, ntpA, atpA	V/A-type H+/Na+-transporting ATPase subunit A	3.6.3.14 3.6.3.15	Metabolism--Energy metabolism--Oxidative phosphorylation|Metabolism--Global and overview maps--Metabolic pathways

[denovogenes]_101270	41.34	7e-51	bad:BAD_0181	K03655	recG	ATP-dependent DNA helicase RecG	3.6.4.12	Genetic Information Processing--Replication and repair--Homologous recombination

[denovogenes]_101271	97.79	0.0	cfd:CFNIH1_03420	K15548	aaeA	p-hydroxybenzoic acid efflux pump subunit AaeA	--	--

[denovogenes]_101272	40.19	7e-30	csb:CLSA_c14430	K06881	nrnA	bifunctional oligoribonuclease and PAP phosphatase NrnA	3.1.3.7 3.1.13.3	Metabolism--Energy metabolism--Sulfur metabolism|Metabolism--Global and overview maps--Metabolic pathways|Metabolism--Global and overview maps--Microbial metabolism in diverse environments

[denovogenes]_101273	99.63	0.0	cfd:CFNIH1_24395	K14187	tyrA	chorismate mutase / prephenate dehydrogenase	5.4.99.5 1.3.1.12	Metabolism--Amino acid metabolism--Phenylalanine, tyrosine and tryptophan biosynthesis|Metabolism--Biosynthesis of other secondary metabolites--Novobiocin biosynthesis|Metabolism--Global and overview maps--Metabolic pathways|Metabolism--Global and overview maps--Biosynthesis of secondary metabolites|Metabolism--Global and overview maps--Biosynthesis of antibiotics|Metabolism--Global and overview maps--Biosynthesis of amino acids

[denovogenes]_101274	100.00	0.0	sbc:SbBS512_E2696	K06173	truA, PUS1	tRNA pseudouridine38-40 synthase	5.4.99.12	--

[denovogenes]_101275	76.01	5e-156	bhl:Bache_2255	K04720	cobD	threonine-phosphate decarboxylase	4.1.1.81	Metabolism--Metabolism of cofactors and vitamins--Porphyrin and chlorophyll metabolism

[denovogenes]_10128	63.24	0.0	btho:Btheta7330_00947	K09955	K09955	uncharacterized protein	--	--

[denovogenes]_101283	100.00	0.0	sbo:SBO_2565	K05711	hcaB	2,3-dihydroxy-2,3-dihydrophenylpropionate dehydrogenase	1.3.1.87	Metabolism--Amino acid metabolism--Phenylalanine metabolism|Metabolism--Global and overview maps--Microbial metabolism in diverse environments|Metabolism--Global and overview maps--Degradation of aromatic compounds

[denovogenes]_101285	95.56	0.0	cfd:CFNIH1_15920	K02034	ABC.PE.P1	peptide/nickel transport system permease protein	--	Cellular Processes--Cellular community - prokaryotes--Quorum sensing

[denovogenes]_101288	59.77	1e-107	riv:Riv7116_5318	K00800	aroA	3-phosphoshikimate 1-carboxyvinyltransferase	2.5.1.19	Metabolism--Amino acid metabolism--Phenylalanine, tyrosine and tryptophan biosynthesis|Metabolism--Global and overview maps--Metabolic pathways|Metabolism--Global and overview maps--Biosynthesis of secondary metabolites|Metabolism--Global and overview maps--Biosynthesis of antibiotics|Metabolism--Global and overview maps--Biosynthesis of amino acids

[denovogenes]_101291	89.67	0.0	bhl:Bache_0658	K03703	uvrC	excinuclease ABC subunit C	--	Genetic Information Processing--Replication and repair--Nucleotide excision repair

[denovogenes]_101292	57.52	1e-87	ele:Elen_1241	K03429	ugtP	processive 1,2-diacylglycerol beta-glucosyltransferase	2.4.1.315	Metabolism--Lipid metabolism--Glycerolipid metabolism|Metabolism--Global and overview maps--Metabolic pathways

[denovogenes]_101293	96.30	0.0	bhl:Bache_2901	K02564	nagB, GNPDA	glucosamine-6-phosphate deaminase	3.5.99.6	Metabolism--Carbohydrate metabolism--Amino sugar and nucleotide sugar metabolism|Metabolism--Global and overview maps--Metabolic pathways

[denovogenes]_101299	46.44	5e-78	aar:Acear_1071	K00872	thrB1	homoserine kinase	2.7.1.39	Metabolism--Amino acid metabolism--Glycine, serine and threonine metabolism|Metabolism--Global and overview maps--Metabolic pathways|Metabolism--Global and overview maps--Biosynthesis of secondary metabolites|Metabolism--Global and overview maps--Microbial metabolism in diverse environments|Metabolism--Global and overview maps--Biosynthesis of amino acids

[denovogenes]_1013	92.47	0.0	cso:CLS_36100	K03046	rpoC	DNA-directed RNA polymerase subunit beta'	2.7.7.6	Metabolism--Nucleotide metabolism--Purine metabolism|Metabolism--Nucleotide metabolism--Pyrimidine metabolism|Metabolism--Global and overview maps--Metabolic pathways|Genetic Information Processing--Transcription--RNA polymerase

[denovogenes]_10130	98.82	0.0	fpla:A4U99_10665	K19701	ywaD	aminopeptidase YwaD	3.4.11.6 3.4.11.10	--

[denovogenes]_101300	95.54	0.0	bdh:GV66_17700	K03110	ftsY	fused signal recognition particle receptor	--	Cellular Processes--Cellular community - prokaryotes--Quorum sensing|Genetic Information Processing--Folding, sorting and degradation--Protein export|Environmental Information Processing--Membrane transport--Bacterial secretion system

[denovogenes]_101301	40.43	3e-49	ksk:KSE_18590	K01133	betC	choline-sulfatase	3.1.6.6	--

[denovogenes]_101302	90.77	1e-174	bvu:BVU_2087	K03310	TC.AGCS	alanine or glycine:cation symporter, AGCS family	--	--

[denovogenes]_101303	57.25	5e-98	ljo:LJ_1808	K02003	ABC.CD.A	putative ABC transport system ATP-binding protein	--	--

[denovogenes]_101305	41.86	2e-59	ccv:CCV52592_2101	K07129	K07129	uncharacterized protein	--	--

[denovogenes]_101308	43.17	2e-60	ssv:SSU98_0695	K01153	hsdR	type I restriction enzyme, R subunit	3.1.21.3	--

[denovogenes]_10131	99.14	0.0	boa:Bovatus_02918	K01284	dcp	peptidyl-dipeptidase Dcp	3.4.15.5	--

[denovogenes]_101311	74.79	3e-123	bprs:CK3_08130	K02283	cpaF, tadA	pilus assembly protein CpaF	--	--

[denovogenes]_101315	84.48	1e-160	pfr:PFREUD_20200	K16785	ecfT	energy-coupling factor transport system permease protein	--	Environmental Information Processing--Membrane transport--ABC transporters

[denovogenes]_101316	93.23	2e-178	pfr:PFREUD_19890	K00020	mmsB, HIBADH	3-hydroxyisobutyrate dehydrogenase	1.1.1.31	Metabolism--Amino acid metabolism--Valine, leucine and isoleucine degradation|Metabolism--Global and overview maps--Metabolic pathways

[denovogenes]_101318	96.30	1e-180	pfre:RM25_1398	K02357	tsf, TSFM	elongation factor Ts	--	--

[denovogenes]_10132	99.57	0.0	boa:Bovatus_01952	K00937	ppk	polyphosphate kinase	2.7.4.1	Metabolism--Energy metabolism--Oxidative phosphorylation|Genetic Information Processing--Folding, sorting and degradation--RNA degradation

[denovogenes]_101321	98.86	0.0	bth:BT_3966	K02477	K02477	two-component system, LytT family, response regulator	--	--

[denovogenes]_101323	82.46	5e-152	pdi:BDI_1123	K00348	nqrC	Na+-transporting NADH:ubiquinone oxidoreductase subunit C	1.6.5.8	--

[denovogenes]_101324	91.85	0.0	pfre:RM25_0728	K00560	thyA, TYMS	thymidylate synthase	2.1.1.45	Metabolism--Nucleotide metabolism--Pyrimidine metabolism|Metabolism--Metabolism of cofactors and vitamins--One carbon pool by folate|Metabolism--Global and overview maps--Metabolic pathways|Human Diseases--Drug resistance: Antineoplastic--Antifolate resistance

[denovogenes]_101326	74.53	5e-141	pdi:BDI_0588	K01609	trpC	indole-3-glycerol phosphate synthase	4.1.1.48	Metabolism--Amino acid metabolism--Phenylalanine, tyrosine and tryptophan biosynthesis|Metabolism--Global and overview maps--Metabolic pathways|Metabolism--Global and overview maps--Biosynthesis of secondary metabolites|Metabolism--Global and overview maps--Biosynthesis of antibiotics|Metabolism--Global and overview maps--Biosynthesis of amino acids

[denovogenes]_101327	94.44	0.0	pdi:BDI_3568	K02564	nagB, GNPDA	glucosamine-6-phosphate deaminase	3.5.99.6	Metabolism--Carbohydrate metabolism--Amino sugar and nucleotide sugar metabolism|Metabolism--Global and overview maps--Metabolic pathways

[denovogenes]_101329	100.00	0.0	ecv:APECO1_O1R219	K06223	dam	DNA adenine methylase	2.1.1.72	Genetic Information Processing--Replication and repair--Mismatch repair

[denovogenes]_10133	94.68	0.0	bfg:BF638R_1377	K03169	topB	DNA topoisomerase III	5.99.1.2	--

[denovogenes]_101330	46.07	3e-64	koe:A225_R1p0855	K12064	traV	conjugal transfer pilus assembly protein TraV	--	--

[denovogenes]_101332	58.15	2e-109	pdi:BDI_1896	K05810	yfiH	laccase domain-containing protein	--	--

[denovogenes]_101333	66.41	9e-127	smf:Smon_1468	K02031	ABC.PE.A	peptide/nickel transport system ATP-binding protein	--	Cellular Processes--Cellular community - prokaryotes--Quorum sensing

[denovogenes]_101335	99.63	0.0	fpla:A4U99_10080	K11071	potB	spermidine/putrescine transport system permease protein	--	Environmental Information Processing--Membrane transport--ABC transporters

[denovogenes]_101336	58.03	5e-107	bdh:GV66_08380	K03832	tonB	periplasmic protein TonB	--	--

[denovogenes]_101340	100.00	0.0	paeo:M801_366	K10563	mutM, fpg	formamidopyrimidine-DNA glycosylase	3.2.2.23 4.2.99.18	Genetic Information Processing--Replication and repair--Base excision repair

[denovogenes]_101343	100.00	0.0	stb:SGPB_0907	K02531	lacT	transcriptional antiterminator	--	--

[denovogenes]_101346	98.89	0.0	fpla:A4U99_05460	K18346	vanW	vancomycin resistance protein VanW	--	Human Diseases--Drug resistance: Antimicrobial--Vancomycin resistance|Environmental Information Processing--Signal transduction--Two-component system

[denovogenes]_101347	52.67	7e-51	dja:HY57_16740	K13653	K13653	AraC family transcriptional regulator	--	--

[denovogenes]_101349	90.74	0.0	lacy:A4V08_13590	K18013	kce	3-keto-5-aminohexanoate cleavage enzyme	2.3.1.247	Metabolism--Amino acid metabolism--Lysine degradation

[denovogenes]_10135	100.00	0.0	pvl:AOB99_15575	K03655	recG	ATP-dependent DNA helicase RecG	3.6.4.12	Genetic Information Processing--Replication and repair--Homologous recombination

[denovogenes]_101350	99.63	0.0	paeo:M801_4600	K07074	K07074	uncharacterized protein	--	--

[denovogenes]_101351	99.63	0.0	cfd:CFNIH1_15920	K02034	ABC.PE.P1	peptide/nickel transport system permease protein	--	Cellular Processes--Cellular community - prokaryotes--Quorum sensing

[denovogenes]_101352	49.63	4e-71	agl:PYTT_1124	K01686	uxuA	mannonate dehydratase	4.2.1.8	Metabolism--Carbohydrate metabolism--Pentose and glucuronate interconversions|Metabolism--Global and overview maps--Metabolic pathways

[denovogenes]_101353	97.78	0.0	fpla:A4U99_08295	K16785	ecfT	energy-coupling factor transport system permease protein	--	Environmental Information Processing--Membrane transport--ABC transporters

[denovogenes]_101356	40.48	5e-14	auh:AWM75_05855	K16698	tuaG	teichuronic acid biosynthesis glycosyltransferase TuaG	2.4.-.-	--

[denovogenes]_101357	55.56	1e-104	cbv:U729_1252	K03783	punA, PNP	purine-nucleoside phosphorylase	2.4.2.1	Metabolism--Nucleotide metabolism--Purine metabolism|Metabolism--Nucleotide metabolism--Pyrimidine metabolism|Metabolism--Metabolism of cofactors and vitamins--Nicotinate and nicotinamide metabolism|Metabolism--Global and overview maps--Metabolic pathways|Metabolism--Global and overview maps--Biosynthesis of secondary metabolites

[denovogenes]_101358	75.93	5e-151	lip:LI0941	K06189	corC	magnesium and cobalt transporter	--	--

[denovogenes]_10136	46.24	0.0	ipo:Ilyop_2387	K03296	TC.HAE1	hydrophobic/amphiphilic exporter-1 (mainly G- bacteria), HAE1 family	--	--

[denovogenes]_101360	99.26	0.0	paeo:M801_1875	K01692	paaF, echA	enoyl-CoA hydratase	4.2.1.17	Metabolism--Lipid metabolism--Fatty acid degradation|Metabolism--Amino acid metabolism--Valine, leucine and isoleucine degradation|Metabolism--Metabolism of terpenoids and polyketides--Geraniol degradation|Metabolism--Amino acid metabolism--Lysine degradation|Metabolism--Amino acid metabolism--Phenylalanine metabolism|Metabolism--Xenobiotics biodegradation and metabolism--Benzoate degradation|Metabolism--Amino acid metabolism--Tryptophan metabolism|Metabolism--Metabolism of other amino acids--beta-Alanine metabolism|Metabolism--Xenobiotics biodegradation and metabolism--Aminobenzoate degradation|Metabolism--Carbohydrate metabolism--Propanoate metabolism|Metabolism--Carbohydrate metabolism--Butanoate metabolism|Metabolism--Metabolism of terpenoids and polyketides--Limonene and pinene degradation|Metabolism--Xenobiotics biodegradation and metabolism--Caprolactam degradation|Metabolism--Global and overview maps--Metabolic pathways|Metabolism--Global and overview maps--Biosynthesis of secondary metabolites|Metabolism--Global and overview maps--Microbial metabolism in diverse environments|Metabolism--Global and overview maps--Biosynthesis of antibiotics|Metabolism--Global and overview maps--Fatty acid metabolism

[denovogenes]_101361	99.26	0.0	pdi:BDI_1123	K00348	nqrC	Na+-transporting NADH:ubiquinone oxidoreductase subunit C	1.6.5.8	--

[denovogenes]_101362	86.99	2e-169	bfr:BF1122	K05810	yfiH	laccase domain-containing protein	--	--

[denovogenes]_101363	63.97	1e-99	cgo:Corgl_0905	K06178	rluB	23S rRNA pseudouridine2605 synthase	5.4.99.22	--

[denovogenes]_101364	40.00	1e-12	lpo:LPO_3305	K01126	E3.1.4.46, glpQ, ugpQ	glycerophosphoryl diester phosphodiesterase	3.1.4.46	Metabolism--Lipid metabolism--Glycerophospholipid metabolism

[denovogenes]_101365	97.04	1e-175	bfb:VU15_12405	K02040	pstS	phosphate transport system substrate-binding protein	--	Environmental Information Processing--Membrane transport--ABC transporters|Environmental Information Processing--Signal transduction--Two-component system|Human Diseases--Infectious diseases: Bacterial--Tuberculosis

[denovogenes]_101367	42.37	5e-62	mes:Meso_3285	K15866	paaG	2-(1,2-epoxy-1,2-dihydrophenyl)acetyl-CoA isomerase	5.3.3.18	Metabolism--Amino acid metabolism--Phenylalanine metabolism|Metabolism--Global and overview maps--Microbial metabolism in diverse environments

[denovogenes]_10137	92.82	0.0	ecas:ECBG_03008	K03046	rpoC	DNA-directed RNA polymerase subunit beta'	2.7.7.6	Metabolism--Nucleotide metabolism--Purine metabolism|Metabolism--Nucleotide metabolism--Pyrimidine metabolism|Metabolism--Global and overview maps--Metabolic pathways|Genetic Information Processing--Transcription--RNA polymerase

[denovogenes]_101370	94.81	0.0	cif:AL515_02775	K00014	aroE	shikimate dehydrogenase	1.1.1.25	Metabolism--Amino acid metabolism--Phenylalanine, tyrosine and tryptophan biosynthesis|Metabolism--Global and overview maps--Metabolic pathways|Metabolism--Global and overview maps--Biosynthesis of secondary metabolites|Metabolism--Global and overview maps--Biosynthesis of antibiotics|Metabolism--Global and overview maps--Biosynthesis of amino acids

[denovogenes]_101372	100.00	0.0	paeo:M801_2482	K01142	E3.1.11.2, xthA	exodeoxyribonuclease III	3.1.11.2	Genetic Information Processing--Replication and repair--Base excision repair

[denovogenes]_101374	99.26	0.0	cama:F384_27265	K02073	metQ	D-methionine transport system substrate-binding protein	--	Environmental Information Processing--Membrane transport--ABC transporters

[denovogenes]_101375	99.26	0.0	cfd:CFNIH1_13870	K03517	nadA	quinolinate synthase	2.5.1.72	Metabolism--Metabolism of cofactors and vitamins--Nicotinate and nicotinamide metabolism|Metabolism--Global and overview maps--Metabolic pathways

[denovogenes]_101377	99.63	0.0	efs:EFS1_2412	K17467	PTS-Dga-EIID, dgaD	PTS system, D-glucosaminate-specific IID component	--	Metabolism--Carbohydrate metabolism--Pentose phosphate pathway|Metabolism--Global and overview maps--Microbial metabolism in diverse environments|Environmental Information Processing--Membrane transport--Phosphotransferase system (PTS)

[denovogenes]_101378	99.26	0.0	paeo:M801_3604	K06966	K06966	uncharacterized protein	--	--

[denovogenes]_101379	67.04	9e-131	cle:Clole_3965	K01990	ABC-2.A	ABC-2 type transport system ATP-binding protein	--	--

[denovogenes]_10138	100.00	0.0	bfs:BF9343_1555	K11381	bkdA	2-oxoisovalerate dehydrogenase E1 component	1.2.4.4	Metabolism--Amino acid metabolism--Valine, leucine and isoleucine degradation|Metabolism--Carbohydrate metabolism--Propanoate metabolism|Metabolism--Global and overview maps--Metabolic pathways|Metabolism--Global and overview maps--Biosynthesis of secondary metabolites|Metabolism--Global and overview maps--Biosynthesis of antibiotics

[denovogenes]_101380	62.50	2e-112	erb:A4V01_19345	K06859	pgi1	glucose-6-phosphate isomerase, archaeal	5.3.1.9	Metabolism--Carbohydrate metabolism--Glycolysis / Gluconeogenesis|Metabolism--Carbohydrate metabolism--Pentose phosphate pathway|Metabolism--Carbohydrate metabolism--Starch and sucrose metabolism|Metabolism--Carbohydrate metabolism--Amino sugar and nucleotide sugar metabolism|Metabolism--Global and overview maps--Metabolic pathways|Metabolism--Global and overview maps--Biosynthesis of secondary metabolites|Metabolism--Global and overview maps--Microbial metabolism in diverse environments|Metabolism--Global and overview maps--Biosynthesis of antibiotics|Metabolism--Global and overview maps--Carbon metabolism

[denovogenes]_101382	58.27	3e-111	osp:Odosp_3039	K07011	K07011	uncharacterized protein	--	--

[denovogenes]_101384	99.63	0.0	pae:PA0820	K07453	K07453	putative restriction endonuclease	--	--

[denovogenes]_101385	51.91	4e-90	cgo:Corgl_1365	K01104	E3.1.3.48	protein-tyrosine phosphatase	3.1.3.48	--

[denovogenes]_101386	99.26	0.0	lacy:A4V08_13045	K09692	tagG	teichoic acid transport system permease protein	--	Environmental Information Processing--Membrane transport--ABC transporters

[denovogenes]_101388	97.78	0.0	cfd:CFNIH1_24140	K05711	hcaB	2,3-dihydroxy-2,3-dihydrophenylpropionate dehydrogenase	1.3.1.87	Metabolism--Amino acid metabolism--Phenylalanine metabolism|Metabolism--Global and overview maps--Microbial metabolism in diverse environments|Metabolism--Global and overview maps--Degradation of aromatic compounds

[denovogenes]_101389	100.00	0.0	efq:DR75_2511	K02796	PTS-Man-EIID, manZ	PTS system, mannose-specific IID component	--	Metabolism--Carbohydrate metabolism--Fructose and mannose metabolism|Metabolism--Carbohydrate metabolism--Amino sugar and nucleotide sugar metabolism|Metabolism--Global and overview maps--Metabolic pathways|Environmental Information Processing--Membrane transport--Phosphotransferase system (PTS)

[denovogenes]_10139	94.52	0.0	ehr:EHR_04625	K02355	fusA, GFM, EFG	elongation factor G	--	--

[denovogenes]_101393	47.33	3e-73	pgi:PG_0437	K01991	ABC-2.OM, wza	polysaccharide biosynthesis/export protein	--	Cellular Processes--Cellular community - prokaryotes--Biofilm formation - Escherichia coli

[denovogenes]_101395	98.52	0.0	lacy:A4V08_24750	K13890	gsiC	glutathione transport system permease protein	--	Environmental Information Processing--Membrane transport--ABC transporters

[denovogenes]_101396	99.63	0.0	cfd:CFNIH1_12240	K00077	panE, apbA	2-dehydropantoate 2-reductase	1.1.1.169	Metabolism--Metabolism of cofactors and vitamins--Pantothenate and CoA biosynthesis|Metabolism--Global and overview maps--Metabolic pathways|Metabolism--Global and overview maps--Biosynthesis of secondary metabolites

[denovogenes]_101398	100.00	0.0	bbrs:BS27_0174	K02564	nagB, GNPDA	glucosamine-6-phosphate deaminase	3.5.99.6	Metabolism--Carbohydrate metabolism--Amino sugar and nucleotide sugar metabolism|Metabolism--Global and overview maps--Metabolic pathways

[denovogenes]_101399	40.00	4e-18	bcen:DM39_2950	K21029	moeB	molybdopterin-synthase adenylyltransferase	2.7.7.80	Genetic Information Processing--Folding, sorting and degradation--Sulfur relay system

[denovogenes]_1014	74.36	0.0	paeb:NCGM1900_0040	K15125	fhaB	filamentous hemagglutinin	--	Human Diseases--Infectious diseases: Bacterial--Pertussis

[denovogenes]_10140	65.02	0.0	cpr:CPR_1767	K00372	nasA	assimilatory nitrate reductase catalytic subunit	1.7.99.4	Metabolism--Energy metabolism--Nitrogen metabolism|Metabolism--Global and overview maps--Microbial metabolism in diverse environments

[denovogenes]_101403	70.79	2e-127	cgo:Corgl_1775	K01945	purD	phosphoribosylamine--glycine ligase	6.3.4.13	Metabolism--Nucleotide metabolism--Purine metabolism|Metabolism--Global and overview maps--Metabolic pathways|Metabolism--Global and overview maps--Biosynthesis of secondary metabolites|Metabolism--Global and overview maps--Biosynthesis of antibiotics

[denovogenes]_101405	50.55	6e-90	sat:SYN_00382	K03813	modD	molybdenum transport protein	2.4.2.-	--

[denovogenes]_101413	64.93	2e-119	ols:Olsu_1240	K15342	cas1	CRISP-associated protein Cas1	--	--

[denovogenes]_101414	80.74	4e-165	pdi:BDI_0715	K00919	ispE	4-diphosphocytidyl-2-C-methyl-D-erythritol kinase	2.7.1.148	Metabolism--Metabolism of terpenoids and polyketides--Terpenoid backbone biosynthesis|Metabolism--Global and overview maps--Metabolic pathways|Metabolism--Global and overview maps--Biosynthesis of secondary metabolites|Metabolism--Global and overview maps--Biosynthesis of antibiotics

[denovogenes]_101415	40.00	1e-26	byl:A4V09_20970	K01426	E3.5.1.4, amiE	amidase	3.5.1.4	Metabolism--Amino acid metabolism--Arginine and proline metabolism|Metabolism--Amino acid metabolism--Phenylalanine metabolism|Metabolism--Amino acid metabolism--Tryptophan metabolism|Metabolism--Xenobiotics biodegradation and metabolism--Aminobenzoate degradation|Metabolism--Xenobiotics biodegradation and metabolism--Styrene degradation|Metabolism--Global and overview maps--Microbial metabolism in diverse environments

[denovogenes]_101416	100.00	0.0	cfd:CFNIH1_24060	K02058	ABC.SS.S	simple sugar transport system substrate-binding protein	--	--

[denovogenes]_101417	89.26	5e-171	pdi:BDI_1192	K05807	bamD	outer membrane protein assembly factor BamD	--	--

[denovogenes]_10142	86.33	0.0	lacy:A4V08_02345	K03168	topA	DNA topoisomerase I	5.99.1.2	--

[denovogenes]_101424	100.00	0.0	cfd:CFNIH1_23140	K06173	truA, PUS1	tRNA pseudouridine38-40 synthase	5.4.99.12	--

[denovogenes]_101428	47.50	6e-28	bdh:GV66_11825	K06079	cutF, nlpE	copper homeostasis protein (lipoprotein)	--	Human Diseases--Drug resistance: Antimicrobial--Cationic antimicrobial peptide (CAMP) resistance

[denovogenes]_101429	98.15	0.0	bcel:BcellWH2_02396	K15633	gpmI	2,3-bisphosphoglycerate-independent phosphoglycerate mutase	5.4.2.12	Metabolism--Carbohydrate metabolism--Glycolysis / Gluconeogenesis|Metabolism--Amino acid metabolism--Glycine, serine and threonine metabolism|Metabolism--Energy metabolism--Methane metabolism|Metabolism--Global and overview maps--Metabolic pathways|Metabolism--Global and overview maps--Biosynthesis of secondary metabolites|Metabolism--Global and overview maps--Microbial metabolism in diverse environments|Metabolism--Global and overview maps--Biosynthesis of antibiotics|Metabolism--Global and overview maps--Carbon metabolism|Metabolism--Global and overview maps--Biosynthesis of amino acids

[denovogenes]_101430	68.08	6e-130	ele:Elen_1120	K02013	ABC.FEV.A	iron complex transport system ATP-binding protein	3.6.3.34	Environmental Information Processing--Membrane transport--ABC transporters

[denovogenes]_101432	92.22	0.0	lacy:A4V08_12180	K01571	oadA	oxaloacetate decarboxylase, alpha subunit	4.1.1.3	Metabolism--Carbohydrate metabolism--Pyruvate metabolism|Metabolism--Global and overview maps--Metabolic pathways

[denovogenes]_101433	49.02	1e-75	cch:Cag_0506	K00721	DPM1	dolichol-phosphate mannosyltransferase	2.4.1.83	Metabolism--Glycan biosynthesis and metabolism--N-Glycan biosynthesis|Metabolism--Global and overview maps--Metabolic pathways

[denovogenes]_101434	100.00	0.0	bfb:VU15_04210	K02564	nagB, GNPDA	glucosamine-6-phosphate deaminase	3.5.99.6	Metabolism--Carbohydrate metabolism--Amino sugar and nucleotide sugar metabolism|Metabolism--Global and overview maps--Metabolic pathways

[denovogenes]_101435	61.85	8e-118	pgi:PG_0425	K01070	frmB, ESD, fghA	S-formylglutathione hydrolase	3.1.2.12	Metabolism--Energy metabolism--Methane metabolism|Metabolism--Global and overview maps--Microbial metabolism in diverse environments|Metabolism--Global and overview maps--Carbon metabolism

[denovogenes]_101437	100.00	0.0	paeu:BN889_01937	K01338	lon	ATP-dependent Lon protease	3.4.21.53	Cellular Processes--Cell growth and death--Cell cycle - Caulobacter

[denovogenes]_101439	65.68	2e-120	csh:Closa_4134	K02030	ABC.PA.S	polar amino acid transport system substrate-binding protein	--	--

[denovogenes]_101440	63.67	1e-122	psn:Pedsa_0103	K05349	bglX	beta-glucosidase	3.2.1.21	Metabolism--Metabolism of other amino acids--Cyanoamino acid metabolism|Metabolism--Carbohydrate metabolism--Starch and sucrose metabolism|Metabolism--Biosynthesis of other secondary metabolites--Phenylpropanoid biosynthesis|Metabolism--Global and overview maps--Metabolic pathways|Metabolism--Global and overview maps--Biosynthesis of secondary metabolites

[denovogenes]_101442	99.63	0.0	paeo:M801_2010	K02529	lacI, galR	LacI family transcriptional regulator	--	--

[denovogenes]_101445	100.00	0.0	pdf:CD630DERM_23410	K14534	abfD	4-hydroxybutyryl-CoA dehydratase / vinylacetyl-CoA-Delta-isomerase	4.2.1.120 5.3.3.3	Metabolism--Carbohydrate metabolism--Butanoate metabolism|Metabolism--Energy metabolism--Carbon fixation pathways in prokaryotes|Metabolism--Global and overview maps--Metabolic pathways|Metabolism--Global and overview maps--Microbial metabolism in diverse environments|Metabolism--Global and overview maps--Carbon metabolism

[denovogenes]_101446	100.00	0.0	sbc:SbBS512_E0570	K01886	QARS, glnS	glutaminyl-tRNA synthetase	6.1.1.18	Genetic Information Processing--Translation--Aminoacyl-tRNA biosynthesis|Metabolism--Global and overview maps--Metabolic pathways

[denovogenes]_101447	88.89	1e-162	afd:Alfi_0169	K06861	lptB	lipopolysaccharide export system ATP-binding protein	3.6.3.-	Environmental Information Processing--Membrane transport--ABC transporters

[denovogenes]_10145	99.71	0.0	efa:EF2406	K01879	glyS	glycyl-tRNA synthetase beta chain	6.1.1.14	Genetic Information Processing--Translation--Aminoacyl-tRNA biosynthesis

[denovogenes]_101451	100.00	0.0	cfd:CFNIH1_09990	K05801	djlA	DnaJ like chaperone protein	--	--

[denovogenes]_101454	88.85	3e-177	pmib:BB2000_2643	K03522	fixB, etfA	electron transfer flavoprotein alpha subunit	--	--

[denovogenes]_101456	41.22	2e-16	pbj:VN24_06975	K01126	E3.1.4.46, glpQ, ugpQ	glycerophosphoryl diester phosphodiesterase	3.1.4.46	Metabolism--Lipid metabolism--Glycerophospholipid metabolism

[denovogenes]_101458	99.26	0.0	cfd:CFNIH1_00400	K02001	proW	glycine betaine/proline transport system permease protein	--	Environmental Information Processing--Membrane transport--ABC transporters

[denovogenes]_101459	59.41	3e-117	rto:RTO_27470	K14170	pheA	chorismate mutase / prephenate dehydratase	5.4.99.5 4.2.1.51	Metabolism--Amino acid metabolism--Phenylalanine, tyrosine and tryptophan biosynthesis|Metabolism--Global and overview maps--Metabolic pathways|Metabolism--Global and overview maps--Biosynthesis of secondary metabolites|Metabolism--Global and overview maps--Biosynthesis of antibiotics|Metabolism--Global and overview maps--Biosynthesis of amino acids

[denovogenes]_10146	98.99	0.0	cbut:ATN24_14620	K12308	bgaB, lacA	beta-galactosidase	3.2.1.23	Metabolism--Carbohydrate metabolism--Galactose metabolism

[denovogenes]_101461	92.62	5e-176	afd:Alfi_0008	K03553	recA	recombination protein RecA	--	Genetic Information Processing--Replication and repair--Homologous recombination

[denovogenes]_101463	64.71	1e-119	bca:BCE_0877	K00001	E1.1.1.1, adh	alcohol dehydrogenase	1.1.1.1	Metabolism--Carbohydrate metabolism--Glycolysis / Gluconeogenesis|Metabolism--Lipid metabolism--Fatty acid degradation|Metabolism--Amino acid metabolism--Tyrosine metabolism|Metabolism--Xenobiotics biodegradation and metabolism--Chloroalkane and chloroalkene degradation|Metabolism--Xenobiotics biodegradation and metabolism--Naphthalene degradation|Metabolism--Metabolism of cofactors and vitamins--Retinol metabolism|Metabolism--Xenobiotics biodegradation and metabolism--Metabolism of xenobiotics by cytochrome P450|Metabolism--Xenobiotics biodegradation and metabolism--Drug metabolism - cytochrome P450|Metabolism--Global and overview maps--Metabolic pathways|Metabolism--Global and overview maps--Biosynthesis of secondary metabolites|Metabolism--Global and overview maps--Microbial metabolism in diverse environments|Metabolism--Global and overview maps--Biosynthesis of antibiotics|Metabolism--Global and overview maps--Degradation of aromatic compounds

[denovogenes]_101466	46.40	5e-59	pob:LPB03_13510	K07497	K07497	putative transposase	--	--

[denovogenes]_101467	100.00	0.0	pdf:CD630DERM_27190	K01567	pdaA	peptidoglycan-N-acetylmuramic acid deacetylase	3.5.1.-	--

[denovogenes]_101468	97.42	0.0	bfg:BF638R_1509	K02005	ABC.CD.TX	HlyD family secretion protein	--	--

[denovogenes]_10147	98.85	0.0	cif:AL515_21810	K00700	GBE1, glgB	1,4-alpha-glucan branching enzyme	2.4.1.18	Metabolism--Carbohydrate metabolism--Starch and sucrose metabolism|Metabolism--Global and overview maps--Metabolic pathways|Metabolism--Global and overview maps--Biosynthesis of secondary metabolites

[denovogenes]_101472	100.00	0.0	ecm:EcSMS35_4475	K00014	aroE	shikimate dehydrogenase	1.1.1.25	Metabolism--Amino acid metabolism--Phenylalanine, tyrosine and tryptophan biosynthesis|Metabolism--Global and overview maps--Metabolic pathways|Metabolism--Global and overview maps--Biosynthesis of secondary metabolites|Metabolism--Global and overview maps--Biosynthesis of antibiotics|Metabolism--Global and overview maps--Biosynthesis of amino acids

[denovogenes]_101474	99.63	0.0	sdz:Asd1617_03227	K00156	poxB	pyruvate dehydrogenase (quinone)	1.2.5.1	Metabolism--Carbohydrate metabolism--Pyruvate metabolism

[denovogenes]_101475	55.43	5e-62	eyy:EGYY_22050	K20492	nisG, spaG, cprC	lantibiotic transport system permease protein	--	Environmental Information Processing--Membrane transport--ABC transporters|Environmental Information Processing--Signal transduction--Two-component system|Cellular Processes--Cellular community - prokaryotes--Quorum sensing

[denovogenes]_101476	56.51	2e-102	cbv:U729_1026	K01223	E3.2.1.86B, bglA	6-phospho-beta-glucosidase	3.2.1.86	Metabolism--Carbohydrate metabolism--Glycolysis / Gluconeogenesis|Metabolism--Carbohydrate metabolism--Starch and sucrose metabolism

[denovogenes]_101477	43.39	1e-45	top:TOPB45_0113	K07814	K07814	putative two-component system response regulator	--	--

[denovogenes]_101478	79.70	4e-162	shi:Shel_01700	K01887	RARS, argS	arginyl-tRNA synthetase	6.1.1.19	Genetic Information Processing--Translation--Aminoacyl-tRNA biosynthesis

[denovogenes]_101479	61.48	6e-118	cgo:Corgl_0967	K03429	ugtP	processive 1,2-diacylglycerol beta-glucosyltransferase	2.4.1.315	Metabolism--Lipid metabolism--Glycerolipid metabolism|Metabolism--Global and overview maps--Metabolic pathways

[denovogenes]_10148	87.03	0.0	bhl:Bache_0925	K01284	dcp	peptidyl-dipeptidase Dcp	3.4.15.5	--

[denovogenes]_101480	61.07	8e-109	lbf:LBF_0719	K01845	hemL	glutamate-1-semialdehyde 2,1-aminomutase	5.4.3.8	Metabolism--Metabolism of cofactors and vitamins--Porphyrin and chlorophyll metabolism|Metabolism--Global and overview maps--Metabolic pathways|Metabolism--Global and overview maps--Biosynthesis of secondary metabolites

[denovogenes]_101485	98.52	0.0	pdi:BDI_1046	K06872	K06872	uncharacterized protein	--	--

[denovogenes]_101486	67.03	5e-136	osp:Odosp_1131	K02343	DPO3G, dnaX	DNA polymerase III subunit gamma/tau	2.7.7.7	Metabolism--Nucleotide metabolism--Purine metabolism|Metabolism--Nucleotide metabolism--Pyrimidine metabolism|Metabolism--Global and overview maps--Metabolic pathways|Genetic Information Processing--Replication and repair--DNA replication|Genetic Information Processing--Replication and repair--Mismatch repair|Genetic Information Processing--Replication and repair--Homologous recombination

[denovogenes]_101489	82.29	6e-164	afd:Alfi_2895	K01939	purA, ADSS	adenylosuccinate synthase	6.3.4.4	Metabolism--Nucleotide metabolism--Purine metabolism|Metabolism--Amino acid metabolism--Alanine, aspartate and glutamate metabolism|Metabolism--Global and overview maps--Metabolic pathways

[denovogenes]_101492	43.49	3e-71	ctc:CTC_02417	K00533	E1.12.7.2L	ferredoxin hydrogenase large subunit	1.12.7.2	--

[denovogenes]_101493	88.93	0.0	bcel:BcellWH2_01809	K01992	ABC-2.P	ABC-2 type transport system permease protein	--	--

[denovogenes]_101494	65.68	1e-132	mhg:MHY_12980	K03737	por, nifJ	pyruvate-ferredoxin/flavodoxin oxidoreductase	1.2.7.1 1.2.7.-	Metabolism--Carbohydrate metabolism--Glycolysis / Gluconeogenesis|Metabolism--Carbohydrate metabolism--Citrate cycle (TCA cycle)|Metabolism--Carbohydrate metabolism--Pyruvate metabolism|Metabolism--Carbohydrate metabolism--Butanoate metabolism|Metabolism--Energy metabolism--Carbon fixation pathways in prokaryotes|Metabolism--Global and overview maps--Metabolic pathways|Metabolism--Global and overview maps--Microbial metabolism in diverse environments|Metabolism--Global and overview maps--Biosynthesis of antibiotics|Metabolism--Global and overview maps--Carbon metabolism

[denovogenes]_101495	97.78	0.0	bfs:BF9343_3148	K07027	K07027	glycosyltransferase 2 family protein	--	--

[denovogenes]_101501	68.27	8e-135	cct:CC1_29470	K06147	ABCB-BAC	ATP-binding cassette, subfamily B, bacterial	--	--

[denovogenes]_101502	96.67	0.0	bcel:BcellWH2_00368	K02015	ABC.FEV.P	iron complex transport system permease protein	--	Environmental Information Processing--Membrane transport--ABC transporters

[denovogenes]_101503	53.62	3e-93	pgm:PGRAT_11175	K03657	uvrD, pcrA	DNA helicase II / ATP-dependent DNA helicase PcrA	3.6.4.12	Genetic Information Processing--Replication and repair--Nucleotide excision repair|Genetic Information Processing--Replication and repair--Mismatch repair

[denovogenes]_101505	42.50	2e-68	lgi:LOTGIDRAFT_154011	K05349	bglX	beta-glucosidase	3.2.1.21	Metabolism--Metabolism of other amino acids--Cyanoamino acid metabolism|Metabolism--Carbohydrate metabolism--Starch and sucrose metabolism|Metabolism--Biosynthesis of other secondary metabolites--Phenylpropanoid biosynthesis|Metabolism--Global and overview maps--Metabolic pathways|Metabolism--Global and overview maps--Biosynthesis of secondary metabolites

[denovogenes]_101506	98.89	0.0	cfd:CFNIH1_10130	K01928	murE	UDP-N-acetylmuramoyl-L-alanyl-D-glutamate--2,6-diaminopimelate ligase	6.3.2.13	Metabolism--Amino acid metabolism--Lysine biosynthesis|Metabolism--Glycan biosynthesis and metabolism--Peptidoglycan biosynthesis

[denovogenes]_101507	40.88	6e-26	thp:BG95_03480	K05349	bglX	beta-glucosidase	3.2.1.21	Metabolism--Metabolism of other amino acids--Cyanoamino acid metabolism|Metabolism--Carbohydrate metabolism--Starch and sucrose metabolism|Metabolism--Biosynthesis of other secondary metabolites--Phenylpropanoid biosynthesis|Metabolism--Global and overview maps--Metabolic pathways|Metabolism--Global and overview maps--Biosynthesis of secondary metabolites

[denovogenes]_101510	99.63	0.0	boa:Bovatus_02266	K09816	znuB	zinc transport system permease protein	--	Environmental Information Processing--Membrane transport--ABC transporters

[denovogenes]_101515	62.83	2e-124	awo:Awo_c31660	K10563	mutM, fpg	formamidopyrimidine-DNA glycosylase	3.2.2.23 4.2.99.18	Genetic Information Processing--Replication and repair--Base excision repair

[denovogenes]_101516	63.94	2e-118	dhd:Dhaf_1915	K02440	GLPF	glycerol uptake facilitator protein	--	--

[denovogenes]_101517	100.00	0.0	boa:Bovatus_03501	K07271	licD	lipopolysaccharide cholinephosphotransferase	2.7.8.-	--

[denovogenes]_101519	84.44	2e-177	bfg:BF638R_0779	K00978	rfbF	glucose-1-phosphate cytidylyltransferase	2.7.7.33	Metabolism--Carbohydrate metabolism--Starch and sucrose metabolism|Metabolism--Carbohydrate metabolism--Amino sugar and nucleotide sugar metabolism|Metabolism--Global and overview maps--Metabolic pathways

[denovogenes]_101520	52.42	2e-97	eas:Entas_1330	K20861	ybjI	FMN hydrolase / 5-amino-6-(5-phospho-D-ribitylamino)uracil phosphatase	3.1.3.102 3.1.3.104	Metabolism--Metabolism of cofactors and vitamins--Riboflavin metabolism|Metabolism--Global and overview maps--Metabolic pathways|Metabolism--Global and overview maps--Biosynthesis of secondary metabolites

[denovogenes]_101522	45.59	5e-64	agl:PYTT_1124	K01686	uxuA	mannonate dehydratase	4.2.1.8	Metabolism--Carbohydrate metabolism--Pentose and glucuronate interconversions|Metabolism--Global and overview maps--Metabolic pathways

[denovogenes]_101523	58.89	4e-114	fsc:FSU_1819	K00868	pdxK, pdxY	pyridoxine kinase	2.7.1.35	Metabolism--Metabolism of cofactors and vitamins--Vitamin B6 metabolism|Metabolism--Global and overview maps--Metabolic pathways

[denovogenes]_101527	56.51	6e-112	ccl:Clocl_3012	K03783	punA, PNP	purine-nucleoside phosphorylase	2.4.2.1	Metabolism--Nucleotide metabolism--Purine metabolism|Metabolism--Nucleotide metabolism--Pyrimidine metabolism|Metabolism--Metabolism of cofactors and vitamins--Nicotinate and nicotinamide metabolism|Metabolism--Global and overview maps--Metabolic pathways|Metabolism--Global and overview maps--Biosynthesis of secondary metabolites

[denovogenes]_101528	57.58	2e-112	puf:UFO1_1796	K00615	E2.2.1.1, tktA, tktB	transketolase	2.2.1.1	Metabolism--Carbohydrate metabolism--Pentose phosphate pathway|Metabolism--Energy metabolism--Carbon fixation in photosynthetic organisms|Metabolism--Metabolism of terpenoids and polyketides--Biosynthesis of ansamycins|Metabolism--Global and overview maps--Metabolic pathways|Metabolism--Global and overview maps--Biosynthesis of secondary metabolites|Metabolism--Global and overview maps--Microbial metabolism in diverse environments|Metabolism--Global and overview maps--Biosynthesis of antibiotics|Metabolism--Global and overview maps--Carbon metabolism|Metabolism--Global and overview maps--Biosynthesis of amino acids

[denovogenes]_10153	91.50	0.0	peo:AS203_05400	K03169	topB	DNA topoisomerase III	5.99.1.2	--

[denovogenes]_101530	60.00	1e-110	cld:CLSPO_c06830	K07053	E3.1.3.97	3',5'-nucleoside bisphosphate phosphatase	3.1.3.97	--

[denovogenes]_101531	99.63	0.0	btho:Btheta7330_04096	K02564	nagB, GNPDA	glucosamine-6-phosphate deaminase	3.5.99.6	Metabolism--Carbohydrate metabolism--Amino sugar and nucleotide sugar metabolism|Metabolism--Global and overview maps--Metabolic pathways

[denovogenes]_101534	100.00	0.0	ecm:EcSMS35_2915	K06872	K06872	uncharacterized protein	--	--

[denovogenes]_101537	58.87	2e-97	sbv:N643_02395	K02795	PTS-Man-EIIC, manY	PTS system, mannose-specific IIC component	--	Metabolism--Carbohydrate metabolism--Fructose and mannose metabolism|Metabolism--Carbohydrate metabolism--Amino sugar and nucleotide sugar metabolism|Metabolism--Global and overview maps--Metabolic pathways|Environmental Information Processing--Membrane transport--Phosphotransferase system (PTS)

[denovogenes]_101539	60.24	1e-94	bvu:BVU_1815	K01051	E3.1.1.11	pectinesterase	3.1.1.11	Metabolism--Carbohydrate metabolism--Pentose and glucuronate interconversions|Metabolism--Global and overview maps--Metabolic pathways

[denovogenes]_10154	99.85	0.0	bxy:BXY_44690	K05349	bglX	beta-glucosidase	3.2.1.21	Metabolism--Metabolism of other amino acids--Cyanoamino acid metabolism|Metabolism--Carbohydrate metabolism--Starch and sucrose metabolism|Metabolism--Biosynthesis of other secondary metabolites--Phenylpropanoid biosynthesis|Metabolism--Global and overview maps--Metabolic pathways|Metabolism--Global and overview maps--Biosynthesis of secondary metabolites

[denovogenes]_101540	75.10	9e-142	cpe:CPE0095	K01715	crt	enoyl-CoA hydratase	4.2.1.17	Metabolism--Carbohydrate metabolism--Butanoate metabolism|Metabolism--Global and overview maps--Carbon metabolism

[denovogenes]_101541	100.00	0.0	shq:A0259_22490	K03629	recF	DNA replication and repair protein RecF	--	Genetic Information Processing--Replication and repair--Homologous recombination

[denovogenes]_101542	53.39	5e-39	hhd:HBHAL_2055	K19224	lytE	peptidoglycan endopeptidase LytE	3.4.-.-	--

[denovogenes]_101544	100.00	0.0	pvl:AOB99_01905	K03576	metR	LysR family transcriptional regulator, regulator for metE and metH	--	--

[denovogenes]_101545	99.63	0.0	abab:BJAB0715_02891	K10673	strA	streptomycin 3"-kinase	2.7.1.87	--

[denovogenes]_101547	59.85	2e-117	ctx:Clo1313_2054	K01940	argG, ASS1	argininosuccinate synthase	6.3.4.5	Metabolism--Amino acid metabolism--Arginine biosynthesis|Metabolism--Amino acid metabolism--Alanine, aspartate and glutamate metabolism|Metabolism--Global and overview maps--Metabolic pathways|Metabolism--Global and overview maps--Biosynthesis of secondary metabolites|Metabolism--Global and overview maps--Biosynthesis of antibiotics|Metabolism--Global and overview maps--Biosynthesis of amino acids|Human Diseases--Cardiovascular diseases--Fluid shear stress and atherosclerosis

[denovogenes]_101548	40.89	9e-40	chd:Calhy_0355	K02315	dnaC	DNA replication protein DnaC	--	--

[denovogenes]_10155	70.29	0.0	rto:RTO_24880	K03798	ftsH, hflB	cell division protease FtsH	3.4.24.-	--

[denovogenes]_101550	100.00	0.0	pvl:AOB99_13095	K03087	SIG2, rpoS	RNA polymerase nonessential primary-like sigma factor	--	Cellular Processes--Cellular community - prokaryotes--Biofilm formation - Escherichia coli|Cellular Processes--Cellular community - prokaryotes--Biofilm formation - Vibrio cholerae

[denovogenes]_101551	55.38	8e-86	euc:EC1_16520	K01537	E3.6.3.8	Ca2+-transporting ATPase	3.6.3.8	--

[denovogenes]_101552	100.00	0.0	pvl:AOB99_16870	K05306	phnX	phosphonoacetaldehyde hydrolase	3.11.1.1	Metabolism--Metabolism of other amino acids--Phosphonate and phosphinate metabolism|Metabolism--Global and overview maps--Metabolic pathways|Metabolism--Global and overview maps--Microbial metabolism in diverse environments

[denovogenes]_101553	73.23	3e-150	bvu:BVU_0520	K01163	K01163	uncharacterized protein	--	--

[denovogenes]_101554	100.00	0.0	ecoa:APECO78_03725	K03771	surA	peptidyl-prolyl cis-trans isomerase SurA	5.2.1.8	--

[denovogenes]_101558	100.00	0.0	pdi:BDI_0605	K02014	TC.FEV.OM	iron complex outermembrane recepter protein	--	--

[denovogenes]_101562	86.62	2e-170	cbv:U729_2667	K06442	tlyA	23S rRNA (cytidine1920-2'-O)/16S rRNA (cytidine1409-2'-O)-methyltransferase	2.1.1.226 2.1.1.227	--

[denovogenes]_101563	64.44	8e-118	csb:CLSA_c14030	K03470	rnhB	ribonuclease HII	3.1.26.4	Genetic Information Processing--Replication and repair--DNA replication

[denovogenes]_101564	99.63	0.0	mmk:MU9_2021	K09817	znuC	zinc transport system ATP-binding protein	3.6.3.-	Environmental Information Processing--Membrane transport--ABC transporters

[denovogenes]_101565	100.00	0.0	mmk:MU9_1366	K02564	nagB, GNPDA	glucosamine-6-phosphate deaminase	3.5.99.6	Metabolism--Carbohydrate metabolism--Amino sugar and nucleotide sugar metabolism|Metabolism--Global and overview maps--Metabolic pathways

[denovogenes]_101567	100.00	0.0	mmk:MU9_3477	K10824	nikE	nickel transport system ATP-binding protein	3.6.3.24	Environmental Information Processing--Membrane transport--ABC transporters

[denovogenes]_101568	99.63	0.0	mmk:MU9_2604	K00286	proC	pyrroline-5-carboxylate reductase	1.5.1.2	Metabolism--Amino acid metabolism--Arginine and proline metabolism|Metabolism--Global and overview maps--Metabolic pathways|Metabolism--Global and overview maps--Biosynthesis of secondary metabolites|Metabolism--Global and overview maps--Biosynthesis of antibiotics|Metabolism--Global and overview maps--Biosynthesis of amino acids

[denovogenes]_101572	40.38	4e-68	eay:EAM_2167	K16702	amsE	amylovoran biosynthesis glycosyltransferase AmsE	2.4.-.-	--

[denovogenes]_101573	100.00	0.0	mmk:MU9_580	K02440	GLPF	glycerol uptake facilitator protein	--	--

[denovogenes]_101574	100.00	0.0	bfb:VU15_00675	K09013	sufC	Fe-S cluster assembly ATP-binding protein	--	--

[denovogenes]_101575	100.00	0.0	bfb:VU15_06445	K00978	rfbF	glucose-1-phosphate cytidylyltransferase	2.7.7.33	Metabolism--Carbohydrate metabolism--Starch and sucrose metabolism|Metabolism--Carbohydrate metabolism--Amino sugar and nucleotide sugar metabolism|Metabolism--Global and overview maps--Metabolic pathways

[denovogenes]_101576	87.36	1e-171	ega:AL523_00945	K11071	potB	spermidine/putrescine transport system permease protein	--	Environmental Information Processing--Membrane transport--ABC transporters

[denovogenes]_101578	100.00	0.0	mmk:MU9_2378	K19230	sapF	cationic peptide transport system ATP-binding protein	--	Human Diseases--Drug resistance: Antimicrobial--Cationic antimicrobial peptide (CAMP) resistance|Environmental Information Processing--Membrane transport--ABC transporters

[denovogenes]_101580	99.63	0.0	bfr:BF2642	K01778	dapF	diaminopimelate epimerase	5.1.1.7	Metabolism--Amino acid metabolism--Lysine biosynthesis|Metabolism--Global and overview maps--Metabolic pathways|Metabolism--Global and overview maps--Biosynthesis of secondary metabolites|Metabolism--Global and overview maps--Microbial metabolism in diverse environments|Metabolism--Global and overview maps--Biosynthesis of antibiotics|Metabolism--Global and overview maps--Biosynthesis of amino acids

[denovogenes]_101581	100.00	0.0	mmk:MU9_877	K03651	cpdA	3',5'-cyclic-AMP phosphodiesterase	3.1.4.53	Metabolism--Nucleotide metabolism--Purine metabolism|Cellular Processes--Cellular community - prokaryotes--Biofilm formation - Pseudomonas aeruginosa

[denovogenes]_101582	100.00	0.0	bfb:VU15_02975	K09816	znuB	zinc transport system permease protein	--	Environmental Information Processing--Membrane transport--ABC transporters

[denovogenes]_101584	84.70	6e-167	cbt:CLH_0046	K00931	proB	glutamate 5-kinase	2.7.2.11	Metabolism--Amino acid metabolism--Arginine and proline metabolism|Metabolism--Biosynthesis of other secondary metabolites--Carbapenem biosynthesis|Metabolism--Global and overview maps--Metabolic pathways|Metabolism--Global and overview maps--Biosynthesis of antibiotics|Metabolism--Global and overview maps--Biosynthesis of amino acids

[denovogenes]_101587	84.01	2e-166	cbt:CLH_0046	K00931	proB	glutamate 5-kinase	2.7.2.11	Metabolism--Amino acid metabolism--Arginine and proline metabolism|Metabolism--Biosynthesis of other secondary metabolites--Carbapenem biosynthesis|Metabolism--Global and overview maps--Metabolic pathways|Metabolism--Global and overview maps--Biosynthesis of antibiotics|Metabolism--Global and overview maps--Biosynthesis of amino acids

[denovogenes]_101588	98.82	1e-118	mmk:MU9_413	K12288	hofM	pilus assembly protein HofM	--	--

[denovogenes]_101589	64.39	1e-126	vte:BHY08_05050	K02042	phnE	phosphonate transport system permease protein	--	Environmental Information Processing--Membrane transport--ABC transporters

[denovogenes]_10159	79.19	0.0	efau:EFAU085_00720	K03169	topB	DNA topoisomerase III	5.99.1.2	--

[denovogenes]_101590	71.75	8e-130	vte:BHY08_05055	K02042	phnE	phosphonate transport system permease protein	--	Environmental Information Processing--Membrane transport--ABC transporters

[denovogenes]_101591	72.12	1e-140	cbut:ATN24_07085	K01962	accA	acetyl-CoA carboxylase carboxyl transferase subunit alpha	6.4.1.2	Metabolism--Lipid metabolism--Fatty acid biosynthesis|Metabolism--Carbohydrate metabolism--Pyruvate metabolism|Metabolism--Carbohydrate metabolism--Propanoate metabolism|Metabolism--Energy metabolism--Carbon fixation pathways in prokaryotes|Metabolism--Global and overview maps--Metabolic pathways|Metabolism--Global and overview maps--Biosynthesis of secondary metabolites|Metabolism--Global and overview maps--Microbial metabolism in diverse environments|Metabolism--Global and overview maps--Biosynthesis of antibiotics|Metabolism--Global and overview maps--Carbon metabolism|Metabolism--Global and overview maps--Fatty acid metabolism

[denovogenes]_101592	79.70	5e-155	cbut:ATN24_16680	K00796	folP	dihydropteroate synthase	2.5.1.15	Metabolism--Metabolism of cofactors and vitamins--Folate biosynthesis|Metabolism--Global and overview maps--Metabolic pathways

[denovogenes]_101593	40.91	2e-71	lrm:LRC_01360	K07271	licD	lipopolysaccharide cholinephosphotransferase	2.7.8.-	--

[denovogenes]_101594	73.96	2e-144	cbv:U729_387	K00941	thiD	hydroxymethylpyrimidine/phosphomethylpyrimidine kinase	2.7.1.49 2.7.4.7	Metabolism--Metabolism of cofactors and vitamins--Thiamine metabolism|Metabolism--Global and overview maps--Metabolic pathways

[denovogenes]_101595	57.99	2e-114	pdf:CD630DERM_18370	K00014	aroE	shikimate dehydrogenase	1.1.1.25	Metabolism--Amino acid metabolism--Phenylalanine, tyrosine and tryptophan biosynthesis|Metabolism--Global and overview maps--Metabolic pathways|Metabolism--Global and overview maps--Biosynthesis of secondary metabolites|Metabolism--Global and overview maps--Biosynthesis of antibiotics|Metabolism--Global and overview maps--Biosynthesis of amino acids

[denovogenes]_101596	86.79	2e-171	efm:M7W_2223	K03481	glvR	RpiR family transcriptional regulator, glv operon transcriptional regulator	--	--

[denovogenes]_101597	79.69	2e-152	cbt:CLH_2384	K02050	ABC.SN.P	NitT/TauT family transport system permease protein	--	--

[denovogenes]_101598	83.64	3e-167	cbv:U729_2667	K06442	tlyA	23S rRNA (cytidine1920-2'-O)/16S rRNA (cytidine1409-2'-O)-methyltransferase	2.1.1.226 2.1.1.227	--

[denovogenes]_101599	77.70	2e-159	ecas:ECBG_01929	K09692	tagG	teichoic acid transport system permease protein	--	Environmental Information Processing--Membrane transport--ABC transporters

[denovogenes]_10160	78.29	0.0	eha:Ethha_1073	K02355	fusA, GFM, EFG	elongation factor G	--	--

[denovogenes]_101600	81.20	2e-158	cbut:ATN24_16680	K00796	folP	dihydropteroate synthase	2.5.1.15	Metabolism--Metabolism of cofactors and vitamins--Folate biosynthesis|Metabolism--Global and overview maps--Metabolic pathways

[denovogenes]_101605	100.00	0.0	ene:ENT_29620	K02795	PTS-Man-EIIC, manY	PTS system, mannose-specific IIC component	--	Metabolism--Carbohydrate metabolism--Fructose and mannose metabolism|Metabolism--Carbohydrate metabolism--Amino sugar and nucleotide sugar metabolism|Metabolism--Global and overview maps--Metabolic pathways|Environmental Information Processing--Membrane transport--Phosphotransferase system (PTS)

[denovogenes]_101606	99.63	0.0	bfb:VU15_00020	K09817	znuC	zinc transport system ATP-binding protein	3.6.3.-	Environmental Information Processing--Membrane transport--ABC transporters

[denovogenes]_101608	79.40	3e-136	ess:ATZ33_02955	K02003	ABC.CD.A	putative ABC transport system ATP-binding protein	--	--

[denovogenes]_101609	99.63	0.0	mmk:MU9_3201	K09973	K09973	uncharacterized protein	--	--

[denovogenes]_10161	100.00	0.0	btho:Btheta7330_00431	K03654	recQ	ATP-dependent DNA helicase RecQ	3.6.4.12	Genetic Information Processing--Folding, sorting and degradation--RNA degradation

[denovogenes]_101610	68.57	1e-101	pdf:CD630DERM_07840	K01448	E3.5.1.28B, amiA, amiB, amiC	N-acetylmuramoyl-L-alanine amidase	3.5.1.28	Human Diseases--Drug resistance: Antimicrobial--Cationic antimicrobial peptide (CAMP) resistance

[denovogenes]_101613	81.23	8e-152	cbv:U729_2008	K02050	ABC.SN.P	NitT/TauT family transport system permease protein	--	--

[denovogenes]_101614	49.42	8e-83	pdf:CD630DERM_22300	K02598	nirC	nitrite transporter NirC	--	--

[denovogenes]_101615	98.51	0.0	efa:EF2479	K06872	K06872	uncharacterized protein	--	--

[denovogenes]_101619	59.23	2e-106	cpr:CPR_0637	K04069	pflA, pflC, pflE	pyruvate formate lyase activating enzyme	1.97.1.4	--

[denovogenes]_101623	68.94	2e-140	cld:CLSPO_c21790	K03148	thiF	sulfur carrier protein ThiS adenylyltransferase	2.7.7.73	Metabolism--Metabolism of cofactors and vitamins--Thiamine metabolism|Metabolism--Global and overview maps--Metabolic pathways|Genetic Information Processing--Folding, sorting and degradation--Sulfur relay system

[denovogenes]_101624	100.00	0.0	ene:ENT_11650	K02036	pstB	phosphate transport system ATP-binding protein	3.6.3.27	Environmental Information Processing--Membrane transport--ABC transporters

[denovogenes]_101625	69.32	7e-136	llm:llmg_0412	K00784	rnz	ribonuclease Z	3.1.26.11	Genetic Information Processing--Translation--RNA transport

[denovogenes]_101627	100.00	0.0	ene:ENT_12160	K02796	PTS-Man-EIID, manZ	PTS system, mannose-specific IID component	--	Metabolism--Carbohydrate metabolism--Fructose and mannose metabolism|Metabolism--Carbohydrate metabolism--Amino sugar and nucleotide sugar metabolism|Metabolism--Global and overview maps--Metabolic pathways|Environmental Information Processing--Membrane transport--Phosphotransferase system (PTS)

[denovogenes]_101628	64.63	4e-109	cco:CCC13826_0177	K03315	nhaC	Na+:H+ antiporter, NhaC family	--	--

[denovogenes]_101629	100.00	0.0	pvl:AOB99_15955	K15587	nikD	nickel transport system ATP-binding protein	3.6.3.24	Environmental Information Processing--Membrane transport--ABC transporters

[denovogenes]_10163	100.00	0.0	ecol:LY180_17520	K00705	malQ	4-alpha-glucanotransferase	2.4.1.25	Metabolism--Carbohydrate metabolism--Starch and sucrose metabolism|Metabolism--Global and overview maps--Metabolic pathways

[denovogenes]_101631	40.18	9e-09	rsa:RSal33209_2638	K03183	ubiE	demethylmenaquinone methyltransferase / 2-methoxy-6-polyprenyl-1,4-benzoquinol methylase	2.1.1.163 2.1.1.201	Metabolism--Metabolism of cofactors and vitamins--Ubiquinone and other terpenoid-quinone biosynthesis|Metabolism--Global and overview maps--Metabolic pathways|Metabolism--Global and overview maps--Biosynthesis of secondary metabolites

[denovogenes]_101632	99.63	0.0	ecos:EC958_3431	K02013	ABC.FEV.A	iron complex transport system ATP-binding protein	3.6.3.34	Environmental Information Processing--Membrane transport--ABC transporters

[denovogenes]_101638	90.33	5e-173	cfd:CFNIH1_15485	K02619	pabC	4-amino-4-deoxychorismate lyase	4.1.3.38	Metabolism--Metabolism of cofactors and vitamins--Folate biosynthesis

[denovogenes]_10164	99.71	0.0	btho:Btheta7330_01029	K00937	ppk	polyphosphate kinase	2.7.4.1	Metabolism--Energy metabolism--Oxidative phosphorylation|Genetic Information Processing--Folding, sorting and degradation--RNA degradation

[denovogenes]_101643	75.46	6e-152	cpy:Cphy_1451	K02042	phnE	phosphonate transport system permease protein	--	Environmental Information Processing--Membrane transport--ABC transporters

[denovogenes]_101644	95.17	0.0	cfd:CFNIH1_05780	K10563	mutM, fpg	formamidopyrimidine-DNA glycosylase	3.2.2.23 4.2.99.18	Genetic Information Processing--Replication and repair--Base excision repair

[denovogenes]_101647	58.09	3e-79	csb:CLSA_c32410	K06872	K06872	uncharacterized protein	--	--

[denovogenes]_10165	96.69	0.0	pfre:RM25_1247	K00239	sdhA, frdA	succinate dehydrogenase / fumarate reductase, flavoprotein subunit	1.3.5.1 1.3.5.4	Metabolism--Carbohydrate metabolism--Citrate cycle (TCA cycle)|Metabolism--Energy metabolism--Oxidative phosphorylation|Metabolism--Carbohydrate metabolism--Butanoate metabolism|Metabolism--Energy metabolism--Carbon fixation pathways in prokaryotes|Metabolism--Global and overview maps--Metabolic pathways|Metabolism--Global and overview maps--Biosynthesis of secondary metabolites|Metabolism--Global and overview maps--Microbial metabolism in diverse environments|Metabolism--Global and overview maps--Biosynthesis of antibiotics|Metabolism--Global and overview maps--Carbon metabolism|Human Diseases--Infectious diseases: Bacterial--Legionellosis

[denovogenes]_101650	71.32	2e-134	vpr:Vpar_0120	K16787	ecfA2	energy-coupling factor transport system ATP-binding protein	3.6.3.-	Environmental Information Processing--Membrane transport--ABC transporters

[denovogenes]_101651	100.00	0.0	pvl:AOB99_08670	K01695	trpA	tryptophan synthase alpha chain	4.2.1.20	Metabolism--Amino acid metabolism--Glycine, serine and threonine metabolism|Metabolism--Amino acid metabolism--Phenylalanine, tyrosine and tryptophan biosynthesis|Metabolism--Global and overview maps--Metabolic pathways|Metabolism--Global and overview maps--Biosynthesis of secondary metabolites|Metabolism--Global and overview maps--Biosynthesis of antibiotics|Metabolism--Global and overview maps--Biosynthesis of amino acids

[denovogenes]_101653	98.51	0.0	cif:AL515_02455	K00557	trmA	tRNA (uracil-5-)-methyltransferase	2.1.1.35	--

[denovogenes]_101654	100.00	0.0	sdz:Asd1617_02758	K02619	pabC	4-amino-4-deoxychorismate lyase	4.1.3.38	Metabolism--Metabolism of cofactors and vitamins--Folate biosynthesis

[denovogenes]_101655	78.89	1e-156	csr:Cspa_c02790	K01687	ilvD	dihydroxy-acid dehydratase	4.2.1.9	Metabolism--Amino acid metabolism--Valine, leucine and isoleucine biosynthesis|Metabolism--Metabolism of cofactors and vitamins--Pantothenate and CoA biosynthesis|Metabolism--Global and overview maps--Metabolic pathways|Metabolism--Global and overview maps--Biosynthesis of secondary metabolites|Metabolism--Global and overview maps--Biosynthesis of antibiotics|Metabolism--Global and overview maps--2-Oxocarboxylic acid metabolism|Metabolism--Global and overview maps--Biosynthesis of amino acids

[denovogenes]_101656	56.55	2e-104	cbut:ATN24_15570	K01992	ABC-2.P	ABC-2 type transport system permease protein	--	--

[denovogenes]_101659	47.77	7e-33	cyt:cce_0698	K18234	vat	virginiamycin A acetyltransferase	2.3.1.-	--

[denovogenes]_10166	97.69	0.0	pfre:RM25_1449	K03702	uvrB	excinuclease ABC subunit B	--	Genetic Information Processing--Replication and repair--Nucleotide excision repair

[denovogenes]_101660	100.00	0.0	eoh:ECO103_0981	K15553	ssuA	sulfonate transport system substrate-binding protein	--	Metabolism--Energy metabolism--Sulfur metabolism|Environmental Information Processing--Membrane transport--ABC transporters

[denovogenes]_101661	70.15	8e-138	etc:ETAC_16325	K02440	GLPF	glycerol uptake facilitator protein	--	--

[denovogenes]_101662	67.04	5e-130	cbz:Cbs_4422	K02529	lacI, galR	LacI family transcriptional regulator	--	--

[denovogenes]_101663	96.65	0.0	sene:IA1_18730	K02036	pstB	phosphate transport system ATP-binding protein	3.6.3.27	Environmental Information Processing--Membrane transport--ABC transporters

[denovogenes]_101664	65.41	3e-128	paej:H70737_04055	K16785	ecfT	energy-coupling factor transport system permease protein	--	Environmental Information Processing--Membrane transport--ABC transporters

[denovogenes]_101666	100.00	0.0	shq:A0259_19725	K02065	mlaF, linL, mkl	phospholipid/cholesterol/gamma-HCH transport system ATP-binding protein	--	Environmental Information Processing--Membrane transport--ABC transporters

[denovogenes]_101667	100.00	0.0	sbc:SbBS512_E4060	K10563	mutM, fpg	formamidopyrimidine-DNA glycosylase	3.2.2.23 4.2.99.18	Genetic Information Processing--Replication and repair--Base excision repair

[denovogenes]_101668	100.00	4e-179	pvl:AOB99_04945	K10003	gltJ, aatQ	glutamate/aspartate transport system permease protein	--	Environmental Information Processing--Membrane transport--ABC transporters|Environmental Information Processing--Signal transduction--Two-component system

[denovogenes]_101669	49.07	3e-71	bpb:bpr_I1669	K02025	ABC.MS.P	multiple sugar transport system permease protein	--	--

[denovogenes]_10167	84.64	0.0	pdi:BDI_2189	K00937	ppk	polyphosphate kinase	2.7.4.1	Metabolism--Energy metabolism--Oxidative phosphorylation|Genetic Information Processing--Folding, sorting and degradation--RNA degradation

[denovogenes]_101672	83.27	2e-166	cbv:U729_2667	K06442	tlyA	23S rRNA (cytidine1920-2'-O)/16S rRNA (cytidine1409-2'-O)-methyltransferase	2.1.1.226 2.1.1.227	--

[denovogenes]_101673	46.67	1e-81	tae:TepiRe1_2396	K00634	ptb	phosphate butyryltransferase	2.3.1.19	Metabolism--Carbohydrate metabolism--Butanoate metabolism|Metabolism--Global and overview maps--Metabolic pathways

[denovogenes]_101674	56.77	2e-111	byl:A4V09_08685	K02025	ABC.MS.P	multiple sugar transport system permease protein	--	--

[denovogenes]_101678	64.50	5e-113	csb:CLSA_c14030	K03470	rnhB	ribonuclease HII	3.1.26.4	Genetic Information Processing--Replication and repair--DNA replication

[denovogenes]_101679	99.63	0.0	cbut:ATN24_13405	K00873	PK, pyk	pyruvate kinase	2.7.1.40	Metabolism--Carbohydrate metabolism--Glycolysis / Gluconeogenesis|Metabolism--Nucleotide metabolism--Purine metabolism|Metabolism--Carbohydrate metabolism--Pyruvate metabolism|Metabolism--Global and overview maps--Metabolic pathways|Metabolism--Global and overview maps--Biosynthesis of secondary metabolites|Metabolism--Global and overview maps--Microbial metabolism in diverse environments|Metabolism--Global and overview maps--Biosynthesis of antibiotics|Metabolism--Global and overview maps--Carbon metabolism|Metabolism--Global and overview maps--Biosynthesis of amino acids|Organismal Systems--Endocrine system--Glucagon signaling pathway|Human Diseases--Endocrine and metabolic diseases--Type II diabetes mellitus|Human Diseases--Cancers: Overview--Viral carcinogenesis|Human Diseases--Cancers: Overview--Central carbon metabolism in cancer

[denovogenes]_10168	55.87	0.0	dvl:Dvul_0179	K01147	rnb	exoribonuclease II	3.1.13.1	--

[denovogenes]_101680	42.98	2e-51	hhd:HBHAL_1355	K08974	K08974	putative membrane protein	--	--

[denovogenes]_101681	99.63	0.0	ecg:E2348C_4281	K00557	trmA	tRNA (uracil-5-)-methyltransferase	2.1.1.35	--

[denovogenes]_101682	67.29	1e-129	cst:CLOST_2139	K02446	glpX	fructose-1,6-bisphosphatase II	3.1.3.11	Metabolism--Carbohydrate metabolism--Glycolysis / Gluconeogenesis|Metabolism--Carbohydrate metabolism--Pentose phosphate pathway|Metabolism--Carbohydrate metabolism--Fructose and mannose metabolism|Metabolism--Energy metabolism--Methane metabolism|Metabolism--Energy metabolism--Carbon fixation in photosynthetic organisms|Metabolism--Global and overview maps--Metabolic pathways|Metabolism--Global and overview maps--Biosynthesis of secondary metabolites|Metabolism--Global and overview maps--Microbial metabolism in diverse environments|Metabolism--Global and overview maps--Biosynthesis of antibiotics|Metabolism--Global and overview maps--Carbon metabolism

[denovogenes]_101684	87.73	3e-172	pod:PODO_20015	K09157	K09157	uncharacterized protein	--	--

[denovogenes]_101685	100.00	0.0	cif:AL515_00930	K02770	PTS-Fru-EIIC, fruA	PTS system, fructose-specific IIC component	--	Metabolism--Carbohydrate metabolism--Fructose and mannose metabolism|Metabolism--Global and overview maps--Metabolic pathways|Metabolism--Global and overview maps--Microbial metabolism in diverse environments|Environmental Information Processing--Membrane transport--Phosphotransferase system (PTS)

[denovogenes]_101686	98.14	0.0	cif:AL515_02455	K00557	trmA	tRNA (uracil-5-)-methyltransferase	2.1.1.35	--

[denovogenes]_101687	81.11	1e-163	cbz:Cbs_4870	K00615	E2.2.1.1, tktA, tktB	transketolase	2.2.1.1	Metabolism--Carbohydrate metabolism--Pentose phosphate pathway|Metabolism--Energy metabolism--Carbon fixation in photosynthetic organisms|Metabolism--Metabolism of terpenoids and polyketides--Biosynthesis of ansamycins|Metabolism--Global and overview maps--Metabolic pathways|Metabolism--Global and overview maps--Biosynthesis of secondary metabolites|Metabolism--Global and overview maps--Microbial metabolism in diverse environments|Metabolism--Global and overview maps--Biosynthesis of antibiotics|Metabolism--Global and overview maps--Carbon metabolism|Metabolism--Global and overview maps--Biosynthesis of amino acids

[denovogenes]_101688	91.45	1e-174	cbv:U729_1706	K03569	mreB	rod shape-determining protein MreB and related proteins	--	--

[denovogenes]_101689	70.00	4e-135	ipo:Ilyop_0967	K03799	htpX	heat shock protein HtpX	3.4.24.-	--

[denovogenes]_10169	54.15	0.0	lir:LAW_01140	K03587	ftsI	cell division protein FtsI (penicillin-binding protein 3)	--	Metabolism--Glycan biosynthesis and metabolism--Peptidoglycan biosynthesis|Human Diseases--Drug resistance: Antimicrobial--beta-Lactam resistance

[denovogenes]_101691	97.04	0.0	cif:AL515_03845	K03079	ulaE, sgaU	L-ribulose-5-phosphate 3-epimerase	5.1.3.22	Metabolism--Carbohydrate metabolism--Ascorbate and aldarate metabolism|Metabolism--Global and overview maps--Metabolic pathways|Metabolism--Global and overview maps--Microbial metabolism in diverse environments

[denovogenes]_101692	42.07	7e-60	doa:AXF15_11390	K11261	fwdE, fmdE	formylmethanofuran dehydrogenase subunit E	1.2.99.5	Metabolism--Energy metabolism--Methane metabolism|Metabolism--Global and overview maps--Metabolic pathways|Metabolism--Global and overview maps--Microbial metabolism in diverse environments|Metabolism--Global and overview maps--Carbon metabolism

[denovogenes]_101698	100.00	0.0	cbut:ATN24_09315	K01258	pepT	tripeptide aminopeptidase	3.4.11.4	--

[denovogenes]_101699	79.48	8e-148	cbut:ATN24_06165	K03310	TC.AGCS	alanine or glycine:cation symporter, AGCS family	--	--

[denovogenes]_101700	53.82	4e-89	csb:CLSA_c31330	K00817	hisC	histidinol-phosphate aminotransferase	2.6.1.9	Metabolism--Amino acid metabolism--Histidine metabolism|Metabolism--Amino acid metabolism--Tyrosine metabolism|Metabolism--Amino acid metabolism--Phenylalanine metabolism|Metabolism--Amino acid metabolism--Phenylalanine, tyrosine and tryptophan biosynthesis|Metabolism--Biosynthesis of other secondary metabolites--Novobiocin biosynthesis|Metabolism--Biosynthesis of other secondary metabolites--Tropane, piperidine and pyridine alkaloid biosynthesis|Metabolism--Global and overview maps--Metabolic pathways|Metabolism--Global and overview maps--Biosynthesis of secondary metabolites|Metabolism--Global and overview maps--Biosynthesis of antibiotics|Metabolism--Global and overview maps--Biosynthesis of amino acids

[denovogenes]_101702	85.33	4e-163	cce:Ccel_2808	K00984	aadA	streptomycin 3"-adenylyltransferase	2.7.7.47	--

[denovogenes]_101703	52.42	1e-92	cem:LH23_10685	K11902	impA	type VI secretion system protein ImpA	--	Cellular Processes--Cellular community - prokaryotes--Biofilm formation - Pseudomonas aeruginosa

[denovogenes]_101705	76.67	6e-157	pdf:CD630DERM_01920	K06131	clsA_B	cardiolipin synthase A/B	2.7.8.-	Metabolism--Lipid metabolism--Glycerophospholipid metabolism|Metabolism--Global and overview maps--Metabolic pathways

[denovogenes]_101706	100.00	0.0	efe:EFER_3686	K03284	corA	magnesium transporter	--	--

[denovogenes]_101707	66.91	2e-133	ccb:Clocel_3829	K06147	ABCB-BAC	ATP-binding cassette, subfamily B, bacterial	--	--

[denovogenes]_101708	73.33	4e-145	cpf:CPF_0770	K09759	aspC	nondiscriminating aspartyl-tRNA synthetase	6.1.1.23	Genetic Information Processing--Translation--Aminoacyl-tRNA biosynthesis

[denovogenes]_101709	80.00	5e-155	tur:AT726_11140	K03665	hflX	GTPase	--	--

[denovogenes]_10171	78.29	0.0	byl:A4V09_00285	K01547	kdpB	K+-transporting ATPase ATPase B chain	3.6.3.12	Environmental Information Processing--Signal transduction--Two-component system

[denovogenes]_101710	100.00	0.0	kpb:FH42_00045	K06907	K06907	uncharacterized protein	--	--

[denovogenes]_101712	77.78	3e-153	yel:LC20_03403	K03658	helD	DNA helicase IV	3.6.4.12	--

[denovogenes]_101714	84.81	2e-169	prg:RB151_010310	K00998	pssA	CDP-diacylglycerol---serine O-phosphatidyltransferase	2.7.8.8	Metabolism--Amino acid metabolism--Glycine, serine and threonine metabolism|Metabolism--Lipid metabolism--Glycerophospholipid metabolism|Metabolism--Global and overview maps--Metabolic pathways|Metabolism--Global and overview maps--Biosynthesis of secondary metabolites

[denovogenes]_101715	99.63	0.0	cfd:CFNIH1_13335	K05515	mrdA	penicillin-binding protein 2	--	Metabolism--Glycan biosynthesis and metabolism--Peptidoglycan biosynthesis|Human Diseases--Drug resistance: Antimicrobial--beta-Lactam resistance

[denovogenes]_101716	52.65	3e-85	ain:Acin_0405	K03470	rnhB	ribonuclease HII	3.1.26.4	Genetic Information Processing--Replication and repair--DNA replication

[denovogenes]_101717	42.65	1e-57	agl:PYTT_1124	K01686	uxuA	mannonate dehydratase	4.2.1.8	Metabolism--Carbohydrate metabolism--Pentose and glucuronate interconversions|Metabolism--Global and overview maps--Metabolic pathways

[denovogenes]_101718	81.78	6e-154	rto:RTO_19750	K06442	tlyA	23S rRNA (cytidine1920-2'-O)/16S rRNA (cytidine1409-2'-O)-methyltransferase	2.1.1.226 2.1.1.227	--

[denovogenes]_101719	75.10	1e-133	cle:Clole_1602	K00286	proC	pyrroline-5-carboxylate reductase	1.5.1.2	Metabolism--Amino acid metabolism--Arginine and proline metabolism|Metabolism--Global and overview maps--Metabolic pathways|Metabolism--Global and overview maps--Biosynthesis of secondary metabolites|Metabolism--Global and overview maps--Biosynthesis of antibiotics|Metabolism--Global and overview maps--Biosynthesis of amino acids

[denovogenes]_10172	99.42	0.0	pae:PA4156	K02014	TC.FEV.OM	iron complex outermembrane recepter protein	--	--

[denovogenes]_101720	77.19	5e-120	rto:RTO_03720	K02050	ABC.SN.P	NitT/TauT family transport system permease protein	--	--

[denovogenes]_101722	73.76	3e-142	rbr:RBR_02340	K00941	thiD	hydroxymethylpyrimidine/phosphomethylpyrimidine kinase	2.7.1.49 2.7.4.7	Metabolism--Metabolism of cofactors and vitamins--Thiamine metabolism|Metabolism--Global and overview maps--Metabolic pathways

[denovogenes]_101723	52.79	2e-101	pdf:CD630DERM_25480	K02026	ABC.MS.P1	multiple sugar transport system permease protein	--	--

[denovogenes]_101725	93.61	6e-175	bhl:Bache_1324	K03118	tatC	sec-independent protein translocase protein TatC	--	Genetic Information Processing--Folding, sorting and degradation--Protein export|Environmental Information Processing--Membrane transport--Bacterial secretion system

[denovogenes]_101726	40.72	3e-37	adf:107341013	K11150	RDH8	retinol dehydrogenase 8	1.1.1.-	Metabolism--Metabolism of cofactors and vitamins--Retinol metabolism|Metabolism--Global and overview maps--Metabolic pathways

[denovogenes]_101727	95.54	0.0	bcel:BcellWH2_01482	K03783	punA, PNP	purine-nucleoside phosphorylase	2.4.2.1	Metabolism--Nucleotide metabolism--Purine metabolism|Metabolism--Nucleotide metabolism--Pyrimidine metabolism|Metabolism--Metabolism of cofactors and vitamins--Nicotinate and nicotinamide metabolism|Metabolism--Global and overview maps--Metabolic pathways|Metabolism--Global and overview maps--Biosynthesis of secondary metabolites

[denovogenes]_10173	44.57	0.0	bfg:BF638R_0648	K01205	NAGLU	alpha-N-acetylglucosaminidase	3.2.1.50	Metabolism--Glycan biosynthesis and metabolism--Glycosaminoglycan degradation|Metabolism--Global and overview maps--Metabolic pathways|Cellular Processes--Transport and catabolism--Lysosome

[denovogenes]_101731	61.17	2e-124	csh:Closa_4134	K02030	ABC.PA.S	polar amino acid transport system substrate-binding protein	--	--

[denovogenes]_101733	61.36	9e-112	rum:CK1_37630	K01609	trpC	indole-3-glycerol phosphate synthase	4.1.1.48	Metabolism--Amino acid metabolism--Phenylalanine, tyrosine and tryptophan biosynthesis|Metabolism--Global and overview maps--Metabolic pathways|Metabolism--Global and overview maps--Biosynthesis of secondary metabolites|Metabolism--Global and overview maps--Biosynthesis of antibiotics|Metabolism--Global and overview maps--Biosynthesis of amino acids

[denovogenes]_101734	100.00	0.0	bxy:BXY_10910	K09816	znuB	zinc transport system permease protein	--	Environmental Information Processing--Membrane transport--ABC transporters

[denovogenes]_10174	62.83	0.0	cpf:CPF_1117	K05342	E2.4.1.64	alpha,alpha-trehalose phosphorylase	2.4.1.64	Metabolism--Carbohydrate metabolism--Starch and sucrose metabolism|Metabolism--Global and overview maps--Metabolic pathways

[denovogenes]_101741	87.36	0.0	btho:Btheta7330_04522	K01629	rhaD	rhamnulose-1-phosphate aldolase	4.1.2.19	Metabolism--Carbohydrate metabolism--Pentose and glucuronate interconversions|Metabolism--Carbohydrate metabolism--Fructose and mannose metabolism|Metabolism--Global and overview maps--Microbial metabolism in diverse environments

[denovogenes]_101744	100.00	0.0	bvu:BVU_0598	K01629	rhaD	rhamnulose-1-phosphate aldolase	4.1.2.19	Metabolism--Carbohydrate metabolism--Pentose and glucuronate interconversions|Metabolism--Carbohydrate metabolism--Fructose and mannose metabolism|Metabolism--Global and overview maps--Microbial metabolism in diverse environments

[denovogenes]_101745	72.69	4e-134	csh:Closa_1973	K07124	K07124	uncharacterized protein	--	--

[denovogenes]_101747	97.40	0.0	bth:BT_2206	K09816	znuB	zinc transport system permease protein	--	Environmental Information Processing--Membrane transport--ABC transporters

[denovogenes]_101748	67.91	2e-133	lio:JL53_00120	K02026	ABC.MS.P1	multiple sugar transport system permease protein	--	--

[denovogenes]_10175	80.55	0.0	cpy:Cphy_1812	K15987	hppA	K(+)-stimulated pyrophosphate-energized sodium pump	3.6.1.1	Metabolism--Energy metabolism--Oxidative phosphorylation

[denovogenes]_101750	60.30	1e-106	abao:BEQ56_03185	K16787	ecfA2	energy-coupling factor transport system ATP-binding protein	3.6.3.-	Environmental Information Processing--Membrane transport--ABC transporters

[denovogenes]_101752	92.94	0.0	bhl:Bache_2368	K07001	K07001	NTE family protein	--	--

[denovogenes]_101754	80.00	8e-154	rto:RTO_19750	K06442	tlyA	23S rRNA (cytidine1920-2'-O)/16S rRNA (cytidine1409-2'-O)-methyltransferase	2.1.1.226 2.1.1.227	--

[denovogenes]_101755	100.00	0.0	boa:Bovatus_03888	K01629	rhaD	rhamnulose-1-phosphate aldolase	4.1.2.19	Metabolism--Carbohydrate metabolism--Pentose and glucuronate interconversions|Metabolism--Carbohydrate metabolism--Fructose and mannose metabolism|Metabolism--Global and overview maps--Microbial metabolism in diverse environments

[denovogenes]_101757	40.93	3e-49	mar:MAE_42710	K12132	prkC, stkP	eukaryotic-like serine/threonine-protein kinase	2.7.11.1	--

[denovogenes]_101758	84.79	1e-149	csh:Closa_4173	K09816	znuB	zinc transport system permease protein	--	Environmental Information Processing--Membrane transport--ABC transporters

[denovogenes]_101759	68.85	1e-118	cls:CXIVA_24220	K06442	tlyA	23S rRNA (cytidine1920-2'-O)/16S rRNA (cytidine1409-2'-O)-methyltransferase	2.1.1.226 2.1.1.227	--

[denovogenes]_10176	98.70	0.0	bcel:BcellWH2_01757	K02316	dnaG	DNA primase	2.7.7.-	Genetic Information Processing--Replication and repair--DNA replication

[denovogenes]_101760	59.11	9e-108	hsd:SD1D_0161	K16511	mecA1_2	adapter protein MecA 1/2	--	--

[denovogenes]_101762	42.44	8e-68	fsc:FSU_1433	K07271	licD	lipopolysaccharide cholinephosphotransferase	2.7.8.-	--

[denovogenes]_101763	86.62	4e-172	cso:CLS_13370	K06442	tlyA	23S rRNA (cytidine1920-2'-O)/16S rRNA (cytidine1409-2'-O)-methyltransferase	2.1.1.226 2.1.1.227	--

[denovogenes]_101764	76.03	1e-149	bprs:CK3_04450	K01778	dapF	diaminopimelate epimerase	5.1.1.7	Metabolism--Amino acid metabolism--Lysine biosynthesis|Metabolism--Global and overview maps--Metabolic pathways|Metabolism--Global and overview maps--Biosynthesis of secondary metabolites|Metabolism--Global and overview maps--Microbial metabolism in diverse environments|Metabolism--Global and overview maps--Biosynthesis of antibiotics|Metabolism--Global and overview maps--Biosynthesis of amino acids

[denovogenes]_101768	64.84	3e-124	bxy:BXY_28940	K03734	apbE	FAD:protein FMN transferase	2.7.1.180	--

[denovogenes]_101769	73.13	8e-145	byl:A4V09_04270	K10979	ku	DNA end-binding protein Ku	--	Genetic Information Processing--Replication and repair--Non-homologous end-joining

[denovogenes]_101770	85.13	4e-171	bdh:GV66_21825	K01448	E3.5.1.28B, amiA, amiB, amiC	N-acetylmuramoyl-L-alanine amidase	3.5.1.28	Human Diseases--Drug resistance: Antimicrobial--Cationic antimicrobial peptide (CAMP) resistance

[denovogenes]_101771	53.03	1e-91	tde:TDE2371	K02574	napH	ferredoxin-type protein NapH	--	--

[denovogenes]_101772	49.81	3e-93	sbr:SY1_18660	K02013	ABC.FEV.A	iron complex transport system ATP-binding protein	3.6.3.34	Environmental Information Processing--Membrane transport--ABC transporters

[denovogenes]_101773	87.40	1e-172	bvu:BVU_3325	K01092	E3.1.3.25, IMPA, suhB	myo-inositol-1(or 4)-monophosphatase	3.1.3.25	Metabolism--Biosynthesis of other secondary metabolites--Streptomycin biosynthesis|Metabolism--Carbohydrate metabolism--Inositol phosphate metabolism|Metabolism--Global and overview maps--Metabolic pathways|Environmental Information Processing--Signal transduction--Phosphatidylinositol signaling system

[denovogenes]_101774	68.66	1e-136	pste:PSTEL_18805	K06971	K06971	uncharacterized protein	--	--

[denovogenes]_101775	99.63	0.0	btho:Btheta7330_04522	K01629	rhaD	rhamnulose-1-phosphate aldolase	4.1.2.19	Metabolism--Carbohydrate metabolism--Pentose and glucuronate interconversions|Metabolism--Carbohydrate metabolism--Fructose and mannose metabolism|Metabolism--Global and overview maps--Microbial metabolism in diverse environments

[denovogenes]_101777	41.84	2e-33	pbi:103051172	K11150	RDH8	retinol dehydrogenase 8	1.1.1.-	Metabolism--Metabolism of cofactors and vitamins--Retinol metabolism|Metabolism--Global and overview maps--Metabolic pathways

[denovogenes]_101778	100.00	0.0	shq:A0259_05565	K00286	proC	pyrroline-5-carboxylate reductase	1.5.1.2	Metabolism--Amino acid metabolism--Arginine and proline metabolism|Metabolism--Global and overview maps--Metabolic pathways|Metabolism--Global and overview maps--Biosynthesis of secondary metabolites|Metabolism--Global and overview maps--Biosynthesis of antibiotics|Metabolism--Global and overview maps--Biosynthesis of amino acids

[denovogenes]_10178	44.87	0.0	tur:AT726_12390	K01200	pulA	pullulanase	3.2.1.41	Metabolism--Carbohydrate metabolism--Starch and sucrose metabolism|Metabolism--Global and overview maps--Metabolic pathways|Metabolism--Global and overview maps--Biosynthesis of secondary metabolites

[denovogenes]_101780	60.46	2e-112	ruk:A4V00_06655	K01567	pdaA	peptidoglycan-N-acetylmuramic acid deacetylase	3.5.1.-	--

[denovogenes]_101781	97.40	0.0	kpa:KPNJ1_01148	K03555	mutS	DNA mismatch repair protein MutS	--	Genetic Information Processing--Replication and repair--Mismatch repair

[denovogenes]_101782	43.15	4e-38	nve:NEMVE_v1g91483	K11150	RDH8	retinol dehydrogenase 8	1.1.1.-	Metabolism--Metabolism of cofactors and vitamins--Retinol metabolism|Metabolism--Global and overview maps--Metabolic pathways

[denovogenes]_101785	99.26	0.0	ecz:ECS88_p0088	K12072	traH	conjugative transfer pilus assembly protein TraH	--	--

[denovogenes]_101786	97.03	0.0	btho:Btheta7330_04522	K01629	rhaD	rhamnulose-1-phosphate aldolase	4.1.2.19	Metabolism--Carbohydrate metabolism--Pentose and glucuronate interconversions|Metabolism--Carbohydrate metabolism--Fructose and mannose metabolism|Metabolism--Global and overview maps--Microbial metabolism in diverse environments

[denovogenes]_101787	99.63	0.0	btho:Btheta7330_01131	K03783	punA, PNP	purine-nucleoside phosphorylase	2.4.2.1	Metabolism--Nucleotide metabolism--Purine metabolism|Metabolism--Nucleotide metabolism--Pyrimidine metabolism|Metabolism--Metabolism of cofactors and vitamins--Nicotinate and nicotinamide metabolism|Metabolism--Global and overview maps--Metabolic pathways|Metabolism--Global and overview maps--Biosynthesis of secondary metabolites

[denovogenes]_101789	67.55	4e-135	blz:BLGT_07955	K16787	ecfA2	energy-coupling factor transport system ATP-binding protein	3.6.3.-	Environmental Information Processing--Membrane transport--ABC transporters

[denovogenes]_101793	99.63	0.0	ecol:LY180_09485	K00563	rlmA1	23S rRNA (guanine745-N1)-methyltransferase	2.1.1.187	--

[denovogenes]_101795	43.75	9e-30	bprm:CL3_14780	K07720	yesN	two-component system, response regulator YesN	--	Environmental Information Processing--Signal transduction--Two-component system

[denovogenes]_101796	72.01	8e-142	byl:A4V09_04270	K10979	ku	DNA end-binding protein Ku	--	Genetic Information Processing--Replication and repair--Non-homologous end-joining

[denovogenes]_101798	72.86	4e-145	clb:Clo1100_0657	K00266	gltD	glutamate synthase (NADPH/NADH) small chain	1.4.1.13 1.4.1.14	Metabolism--Amino acid metabolism--Alanine, aspartate and glutamate metabolism|Metabolism--Energy metabolism--Nitrogen metabolism|Metabolism--Global and overview maps--Metabolic pathways|Metabolism--Global and overview maps--Biosynthesis of secondary metabolites|Metabolism--Global and overview maps--Microbial metabolism in diverse environments|Metabolism--Global and overview maps--Biosynthesis of antibiotics|Metabolism--Global and overview maps--Biosynthesis of amino acids

[denovogenes]_101799	98.07	0.0	lacy:A4V08_31185	K02392	flgG	flagellar basal-body rod protein FlgG	--	Cellular Processes--Cell motility--Flagellar assembly

[denovogenes]_1018	78.06	0.0	pdi:BDI_0934	K06894	K06894	uncharacterized protein	--	--

[denovogenes]_101800	56.37	2e-101	byl:A4V09_04290	K11070	potC	spermidine/putrescine transport system permease protein	--	Environmental Information Processing--Membrane transport--ABC transporters

[denovogenes]_101801	55.29	3e-93	caby:Cabys_1887	K01966	PCCB, pccB	propionyl-CoA carboxylase beta chain	6.4.1.3	Metabolism--Amino acid metabolism--Valine, leucine and isoleucine degradation|Metabolism--Carbohydrate metabolism--Glyoxylate and dicarboxylate metabolism|Metabolism--Carbohydrate metabolism--Propanoate metabolism|Metabolism--Global and overview maps--Metabolic pathways|Metabolism--Global and overview maps--Microbial metabolism in diverse environments|Metabolism--Global and overview maps--Biosynthesis of antibiotics|Metabolism--Global and overview maps--Carbon metabolism

[denovogenes]_101802	69.89	2e-140	lacy:A4V08_29105	K04486	E3.1.3.15B	histidinol-phosphatase (PHP family)	3.1.3.15	Metabolism--Amino acid metabolism--Histidine metabolism|Metabolism--Global and overview maps--Metabolic pathways|Metabolism--Global and overview maps--Biosynthesis of secondary metabolites|Metabolism--Global and overview maps--Biosynthesis of amino acids

[denovogenes]_101803	43.01	7e-57	agl:PYTT_1124	K01686	uxuA	mannonate dehydratase	4.2.1.8	Metabolism--Carbohydrate metabolism--Pentose and glucuronate interconversions|Metabolism--Global and overview maps--Metabolic pathways

[denovogenes]_101804	99.63	0.0	bth:BT_2206	K09816	znuB	zinc transport system permease protein	--	Environmental Information Processing--Membrane transport--ABC transporters

[denovogenes]_101805	91.45	0.0	lacy:A4V08_28705	K02747	PTS-Aga-EIID, agaE	PTS system, N-acetylgalactosamine-specific IID component	--	Metabolism--Carbohydrate metabolism--Galactose metabolism|Environmental Information Processing--Membrane transport--Phosphotransferase system (PTS)

[denovogenes]_101806	98.51	0.0	lacy:A4V08_12480	K02054	ABC.SP.P1	putative spermidine/putrescine transport system permease protein	--	Cellular Processes--Cellular community - prokaryotes--Quorum sensing

[denovogenes]_101807	97.40	0.0	bvu:BVU_0598	K01629	rhaD	rhamnulose-1-phosphate aldolase	4.1.2.19	Metabolism--Carbohydrate metabolism--Pentose and glucuronate interconversions|Metabolism--Carbohydrate metabolism--Fructose and mannose metabolism|Metabolism--Global and overview maps--Microbial metabolism in diverse environments

[denovogenes]_101809	95.17	0.0	bfg:BF638R_3308	K03783	punA, PNP	purine-nucleoside phosphorylase	2.4.2.1	Metabolism--Nucleotide metabolism--Purine metabolism|Metabolism--Nucleotide metabolism--Pyrimidine metabolism|Metabolism--Metabolism of cofactors and vitamins--Nicotinate and nicotinamide metabolism|Metabolism--Global and overview maps--Metabolic pathways|Metabolism--Global and overview maps--Biosynthesis of secondary metabolites

[denovogenes]_101812	97.40	0.0	cfd:CFNIH1_11845	K02554	mhpD	2-keto-4-pentenoate hydratase	4.2.1.80	Metabolism--Amino acid metabolism--Phenylalanine metabolism|Metabolism--Xenobiotics biodegradation and metabolism--Benzoate degradation|Metabolism--Xenobiotics biodegradation and metabolism--Dioxin degradation|Metabolism--Xenobiotics biodegradation and metabolism--Xylene degradation|Metabolism--Global and overview maps--Metabolic pathways|Metabolism--Global and overview maps--Microbial metabolism in diverse environments|Metabolism--Global and overview maps--Degradation of aromatic compounds

[denovogenes]_101814	74.07	4e-147	bhu:bhn_I2630	K02042	phnE	phosphonate transport system permease protein	--	Environmental Information Processing--Membrane transport--ABC transporters

[denovogenes]_101816	44.36	2e-71	aar:Acear_0798	K01436	K01436	amidohydrolase	3.5.1.-	--

[denovogenes]_101818	68.48	2e-126	gpa:GPA_12960	K03496	parA, soj	chromosome partitioning protein	--	--

[denovogenes]_101819	78.15	3e-142	rto:RTO_26170	K00981	E2.7.7.41, CDS1, CDS2, cdsA	phosphatidate cytidylyltransferase	2.7.7.41	Metabolism--Lipid metabolism--Glycerophospholipid metabolism|Metabolism--Global and overview maps--Metabolic pathways|Metabolism--Global and overview maps--Biosynthesis of secondary metabolites|Environmental Information Processing--Signal transduction--Phosphatidylinositol signaling system

[denovogenes]_10182	99.57	0.0	mmk:MU9_620	K03655	recG	ATP-dependent DNA helicase RecG	3.6.4.12	Genetic Information Processing--Replication and repair--Homologous recombination

[denovogenes]_101820	70.74	3e-132	fpla:A4U99_08295	K16785	ecfT	energy-coupling factor transport system permease protein	--	Environmental Information Processing--Membrane transport--ABC transporters

[denovogenes]_101821	97.40	0.0	cfd:CFNIH1_12725	K02083	allC	allantoate deiminase	3.5.3.9	Metabolism--Nucleotide metabolism--Purine metabolism|Metabolism--Global and overview maps--Microbial metabolism in diverse environments

[denovogenes]_101824	85.56	3e-177	rto:RTO_09100	K01628	fucA	L-fuculose-phosphate aldolase	4.1.2.17	Metabolism--Carbohydrate metabolism--Fructose and mannose metabolism|Metabolism--Global and overview maps--Microbial metabolism in diverse environments

[denovogenes]_101825	98.89	0.0	cfd:CFNIH1_24245	K03100	lepB, TPP	signal peptidase I	3.4.21.89	Cellular Processes--Cellular community - prokaryotes--Quorum sensing|Genetic Information Processing--Folding, sorting and degradation--Protein export

[denovogenes]_101827	65.27	6e-122	ert:EUR_18270	K13292	lgt, umpA	phosphatidylglycerol:prolipoprotein diacylglycerol transferase	2.-.-.-	--

[denovogenes]_101829	100.00	0.0	btho:Btheta7330_02671	K03832	tonB	periplasmic protein TonB	--	--

[denovogenes]_10183	56.78	0.0	ene:ENT_08550	K01534	zntA	Cd2+/Zn2+-exporting ATPase	3.6.3.3 3.6.3.5	--

[denovogenes]_101830	92.57	0.0	btho:Btheta7330_02671	K03832	tonB	periplasmic protein TonB	--	--

[denovogenes]_101831	56.88	2e-108	mwo:MWSIV6_0325	K00973	E2.7.7.24, rfbA, rffH	glucose-1-phosphate thymidylyltransferase	2.7.7.24	Metabolism--Biosynthesis of other secondary metabolites--Streptomycin biosynthesis|Metabolism--Metabolism of terpenoids and polyketides--Polyketide sugar unit biosynthesis|Metabolism--Biosynthesis of other secondary metabolites--Acarbose and validamycin biosynthesis|Metabolism--Global and overview maps--Biosynthesis of antibiotics

[denovogenes]_101832	87.73	0.0	bcel:BcellWH2_02084	K01629	rhaD	rhamnulose-1-phosphate aldolase	4.1.2.19	Metabolism--Carbohydrate metabolism--Pentose and glucuronate interconversions|Metabolism--Carbohydrate metabolism--Fructose and mannose metabolism|Metabolism--Global and overview maps--Microbial metabolism in diverse environments

[denovogenes]_101833	64.75	7e-121	rob:CK5_05950	K03215	rumA	23S rRNA (uracil1939-C5)-methyltransferase	2.1.1.190	--

[denovogenes]_101836	51.35	2e-90	dae:Dtox_4158	K03569	mreB	rod shape-determining protein MreB and related proteins	--	--

[denovogenes]_101837	90.12	2e-165	rto:RTO_23010	K02118	ATPVB, ntpB, atpB	V/A-type H+/Na+-transporting ATPase subunit B	--	Metabolism--Energy metabolism--Oxidative phosphorylation|Metabolism--Global and overview maps--Metabolic pathways

[denovogenes]_101838	88.85	6e-179	bhl:Bache_1092	K01468	hutI, AMDHD1	imidazolonepropionase	3.5.2.7	Metabolism--Amino acid metabolism--Histidine metabolism|Metabolism--Global and overview maps--Metabolic pathways

[denovogenes]_101839	65.19	5e-116	cle:Clole_3775	K07652	vicK	two-component system, OmpR family, sensor histidine kinase VicK	2.7.13.3	Environmental Information Processing--Signal transduction--Two-component system

[denovogenes]_10184	93.80	0.0	bfs:BF9343_1192	K03169	topB	DNA topoisomerase III	5.99.1.2	--

[denovogenes]_101840	100.00	0.0	shq:A0259_14100	K01711	gmd, GMDS	GDPmannose 4,6-dehydratase	4.2.1.47	Metabolism--Carbohydrate metabolism--Fructose and mannose metabolism|Metabolism--Carbohydrate metabolism--Amino sugar and nucleotide sugar metabolism|Metabolism--Global and overview maps--Metabolic pathways

[denovogenes]_101843	94.05	4e-175	cif:AL515_14185	K05877	tap	methyl-accepting chemotaxis protein IV, peptide sensor receptor	--	Environmental Information Processing--Signal transduction--Two-component system|Cellular Processes--Cell motility--Bacterial chemotaxis

[denovogenes]_101844	98.52	0.0	cfd:CFNIH1_17245	K09001	anmK	anhydro-N-acetylmuramic acid kinase	2.7.1.170	--

[denovogenes]_101846	100.00	0.0	sft:NCTC1_02840	K05817	hcaR	LysR family transcriptional regulator, hca operon transcriptional activator	--	--

[denovogenes]_101847	91.87	8e-162	cfd:CFNIH1_05890	K01972	E6.5.1.2, ligA, ligB	DNA ligase (NAD+)	6.5.1.2	Genetic Information Processing--Replication and repair--DNA replication|Genetic Information Processing--Replication and repair--Base excision repair|Genetic Information Processing--Replication and repair--Nucleotide excision repair|Genetic Information Processing--Replication and repair--Mismatch repair

[denovogenes]_101848	61.36	7e-52	cpro:CPRO_15160	K03546	sbcC	exonuclease SbcC	--	--

[denovogenes]_10185	63.10	0.0	ess:ATZ33_00295	K03169	topB	DNA topoisomerase III	5.99.1.2	--

[denovogenes]_101855	44.63	6e-66	clb:Clo1100_2597	K02594	nifV	homocitrate synthase NifV	2.3.3.14	Metabolism--Carbohydrate metabolism--Pyruvate metabolism

[denovogenes]_10186	79.71	0.0	pdf:CD630DERM_13180	K00962	pnp, PNPT1	polyribonucleotide nucleotidyltransferase	2.7.7.8	Metabolism--Nucleotide metabolism--Purine metabolism|Metabolism--Nucleotide metabolism--Pyrimidine metabolism|Genetic Information Processing--Folding, sorting and degradation--RNA degradation

[denovogenes]_101860	96.28	0.0	pfre:RM25_1773	K18928	lldE	L-lactate dehydrogenase complex protein LldE	--	--

[denovogenes]_101862	81.78	1e-153	pfre:RM25_1046	K01591	pyrF	orotidine-5'-phosphate decarboxylase	4.1.1.23	Metabolism--Nucleotide metabolism--Pyrimidine metabolism|Metabolism--Global and overview maps--Metabolic pathways

[denovogenes]_101864	91.82	0.0	pfre:RM25_1615	K12503	E2.5.1.68	short-chain Z-isoprenyl diphosphate synthase	2.5.1.68	Metabolism--Metabolism of terpenoids and polyketides--Terpenoid backbone biosynthesis|Metabolism--Global and overview maps--Biosynthesis of secondary metabolites

[denovogenes]_101867	58.96	4e-106	puf:UFO1_3641	K02172	blaR1	bla regulator protein blaR1	--	Human Diseases--Drug resistance: Antimicrobial--beta-Lactam resistance

[denovogenes]_10187	99.57	0.0	efq:DR75_1194	K02238	comEC	competence protein ComEC	--	--

[denovogenes]_101870	44.55	4e-25	cdi:DIP0190	K07741	antB	anti-repressor protein	--	--

[denovogenes]_101871	88.48	1e-171	pdi:BDI_1192	K05807	bamD	outer membrane protein assembly factor BamD	--	--

[denovogenes]_101874	82.20	8e-167	pdi:BDI_0792	K01082	cysQ, MET22, BPNT1	3'(2'), 5'-bisphosphate nucleotidase	3.1.3.7	Metabolism--Energy metabolism--Sulfur metabolism|Metabolism--Global and overview maps--Metabolic pathways|Metabolism--Global and overview maps--Microbial metabolism in diverse environments|Metabolism--Global and overview maps--Biosynthesis of antibiotics

[denovogenes]_101876	45.35	2e-74	doi:FH5T_06225	K03768	PPIB, ppiB	peptidyl-prolyl cis-trans isomerase B (cyclophilin B)	5.2.1.8	--

[denovogenes]_101877	99.63	0.0	btho:Btheta7330_03869	K06173	truA, PUS1	tRNA pseudouridine38-40 synthase	5.4.99.12	--

[denovogenes]_101878	99.63	0.0	paec:M802_5685	K09817	znuC	zinc transport system ATP-binding protein	3.6.3.-	Environmental Information Processing--Membrane transport--ABC transporters

[denovogenes]_101879	50.37	7e-80	mmy:MSC_0215	K01155	E3.1.21.4	type II restriction enzyme	3.1.21.4	--

[denovogenes]_10188	74.71	0.0	clt:CM240_1762	K07003	K07003	uncharacterized protein	--	--

[denovogenes]_101880	99.63	0.0	cfd:CFNIH1_11845	K02554	mhpD	2-keto-4-pentenoate hydratase	4.2.1.80	Metabolism--Amino acid metabolism--Phenylalanine metabolism|Metabolism--Xenobiotics biodegradation and metabolism--Benzoate degradation|Metabolism--Xenobiotics biodegradation and metabolism--Dioxin degradation|Metabolism--Xenobiotics biodegradation and metabolism--Xylene degradation|Metabolism--Global and overview maps--Metabolic pathways|Metabolism--Global and overview maps--Microbial metabolism in diverse environments|Metabolism--Global and overview maps--Degradation of aromatic compounds

[denovogenes]_101881	42.41	3e-40	tad:TRIADDRAFT_63470	K11150	RDH8	retinol dehydrogenase 8	1.1.1.-	Metabolism--Metabolism of cofactors and vitamins--Retinol metabolism|Metabolism--Global and overview maps--Metabolic pathways

[denovogenes]_101882	54.28	3e-96	ibu:IB211_00427	K16203	dppA1	D-amino peptidase	3.4.11.-	--

[denovogenes]_101883	43.82	6e-86	clt:CM240_3240	K02006	cbiO	cobalt/nickel transport system ATP-binding protein	--	Environmental Information Processing--Membrane transport--ABC transporters

[denovogenes]_101884	100.00	0.0	paeo:M801_210	K11070	potC	spermidine/putrescine transport system permease protein	--	Environmental Information Processing--Membrane transport--ABC transporters

[denovogenes]_101886	98.51	0.0	bfb:VU15_02975	K09816	znuB	zinc transport system permease protein	--	Environmental Information Processing--Membrane transport--ABC transporters

[denovogenes]_101889	77.78	1e-160	bhu:bhn_I2630	K02042	phnE	phosphonate transport system permease protein	--	Environmental Information Processing--Membrane transport--ABC transporters

[denovogenes]_10189	79.71	0.0	cbv:U729_2787	K02519	infB, MTIF2	translation initiation factor IF-2	--	--

[denovogenes]_101892	77.04	2e-151	pdi:BDI_0715	K00919	ispE	4-diphosphocytidyl-2-C-methyl-D-erythritol kinase	2.7.1.148	Metabolism--Metabolism of terpenoids and polyketides--Terpenoid backbone biosynthesis|Metabolism--Global and overview maps--Metabolic pathways|Metabolism--Global and overview maps--Biosynthesis of secondary metabolites|Metabolism--Global and overview maps--Biosynthesis of antibiotics

[denovogenes]_101895	60.15	1e-91	dfi:AXF13_11340	K07308	dmsC	anaerobic dimethyl sulfoxide reductase subunit C (DMSO reductase anchor subunit)	--	Metabolism--Energy metabolism--Sulfur metabolism

[denovogenes]_101896	77.24	6e-133	dfi:AXF13_13365	K02073	metQ	D-methionine transport system substrate-binding protein	--	Environmental Information Processing--Membrane transport--ABC transporters

[denovogenes]_101897	60.70	5e-111	lir:LAW_01102	K06173	truA, PUS1	tRNA pseudouridine38-40 synthase	5.4.99.12	--

[denovogenes]_101899	100.00	0.0	stb:SGPB_0347	K02796	PTS-Man-EIID, manZ	PTS system, mannose-specific IID component	--	Metabolism--Carbohydrate metabolism--Fructose and mannose metabolism|Metabolism--Carbohydrate metabolism--Amino sugar and nucleotide sugar metabolism|Metabolism--Global and overview maps--Metabolic pathways|Environmental Information Processing--Membrane transport--Phosphotransferase system (PTS)

[denovogenes]_10190	100.00	0.0	ene:ENT_02680	K02355	fusA, GFM, EFG	elongation factor G	--	--

[denovogenes]_101900	63.94	1e-122	bcel:BcellWH2_04146	K06897	K06897	7,8-dihydropterin-6-yl-methyl-4-(beta-D-ribofuranosyl)aminobenzene 5'-phosphate synthase	2.5.1.105	--

[denovogenes]_101901	99.63	0.0	cfd:CFNIH1_05780	K10563	mutM, fpg	formamidopyrimidine-DNA glycosylase	3.2.2.23 4.2.99.18	Genetic Information Processing--Replication and repair--Base excision repair

[denovogenes]_101903	99.63	0.0	bfb:VU15_07810	K07271	licD	lipopolysaccharide cholinephosphotransferase	2.7.8.-	--

[denovogenes]_101904	72.18	1e-142	fpa:FPR_17970	K03686	dnaJ	molecular chaperone DnaJ	--	--

[denovogenes]_101907	76.38	3e-148	pdi:BDI_0384	K00677	lpxA	UDP-N-acetylglucosamine acyltransferase	2.3.1.129	Metabolism--Glycan biosynthesis and metabolism--Lipopolysaccharide biosynthesis|Metabolism--Global and overview maps--Metabolic pathways|Human Diseases--Drug resistance: Antimicrobial--Cationic antimicrobial peptide (CAMP) resistance

[denovogenes]_101909	100.00	0.0	stb:SGPB_1611	K01776	murI	glutamate racemase	5.1.1.3	Metabolism--Metabolism of other amino acids--D-Glutamine and D-glutamate metabolism|Metabolism--Global and overview maps--Metabolic pathways

[denovogenes]_101912	95.54	0.0	bfr:BF3334	K02002	proX	glycine betaine/proline transport system substrate-binding protein	--	Environmental Information Processing--Membrane transport--ABC transporters

[denovogenes]_101913	100.00	0.0	cfd:CFNIH1_11995	K00286	proC	pyrroline-5-carboxylate reductase	1.5.1.2	Metabolism--Amino acid metabolism--Arginine and proline metabolism|Metabolism--Global and overview maps--Metabolic pathways|Metabolism--Global and overview maps--Biosynthesis of secondary metabolites|Metabolism--Global and overview maps--Biosynthesis of antibiotics|Metabolism--Global and overview maps--Biosynthesis of amino acids

[denovogenes]_101916	42.15	1e-18	tsh:Tsac_2616	K02557	motB	chemotaxis protein MotB	--	Cellular Processes--Cell motility--Bacterial chemotaxis|Cellular Processes--Cell motility--Flagellar assembly

[denovogenes]_101918	58.89	5e-101	euc:EC1_02260	K01006	ppdK	pyruvate, orthophosphate dikinase	2.7.9.1	Metabolism--Carbohydrate metabolism--Pyruvate metabolism|Metabolism--Energy metabolism--Carbon fixation in photosynthetic organisms|Metabolism--Energy metabolism--Carbon fixation pathways in prokaryotes|Metabolism--Global and overview maps--Metabolic pathways|Metabolism--Global and overview maps--Microbial metabolism in diverse environments|Metabolism--Global and overview maps--Carbon metabolism

[denovogenes]_101919	74.16	7e-93	gfe:Gferi_03765	K02279	cpaB, rcpC	pilus assembly protein CpaB	--	--

[denovogenes]_101920	99.26	0.0	cfd:CFNIH1_04575	K02598	nirC	nitrite transporter NirC	--	--

[denovogenes]_101921	68.77	7e-137	cpy:Cphy_3860	K10119	msmG	raffinose/stachyose/melibiose transport system permease protein	--	Environmental Information Processing--Membrane transport--ABC transporters

[denovogenes]_101923	42.38	4e-58	byl:A4V09_12320	K07720	yesN	two-component system, response regulator YesN	--	Environmental Information Processing--Signal transduction--Two-component system

[denovogenes]_101924	67.04	2e-140	bhl:Bache_0670	K00784	rnz	ribonuclease Z	3.1.26.11	Genetic Information Processing--Translation--RNA transport

[denovogenes]_101925	75.20	6e-137	bprm:CL3_16910	K00655	plsC	1-acyl-sn-glycerol-3-phosphate acyltransferase	2.3.1.51	Metabolism--Lipid metabolism--Glycerolipid metabolism|Metabolism--Lipid metabolism--Glycerophospholipid metabolism|Metabolism--Global and overview maps--Metabolic pathways|Metabolism--Global and overview maps--Biosynthesis of secondary metabolites

[denovogenes]_101926	94.80	0.0	lacy:A4V08_29105	K04486	E3.1.3.15B	histidinol-phosphatase (PHP family)	3.1.3.15	Metabolism--Amino acid metabolism--Histidine metabolism|Metabolism--Global and overview maps--Metabolic pathways|Metabolism--Global and overview maps--Biosynthesis of secondary metabolites|Metabolism--Global and overview maps--Biosynthesis of amino acids

[denovogenes]_101928	68.94	2e-131	fpr:FP2_08080	K11071	potB	spermidine/putrescine transport system permease protein	--	Environmental Information Processing--Membrane transport--ABC transporters

[denovogenes]_101929	93.70	0.0	bfb:VU15_01075	K01785	galM, GALM	aldose 1-epimerase	5.1.3.3	Metabolism--Carbohydrate metabolism--Glycolysis / Gluconeogenesis|Metabolism--Carbohydrate metabolism--Galactose metabolism|Metabolism--Global and overview maps--Metabolic pathways|Metabolism--Global and overview maps--Biosynthesis of secondary metabolites|Metabolism--Global and overview maps--Microbial metabolism in diverse environments|Metabolism--Global and overview maps--Biosynthesis of antibiotics

[denovogenes]_101930	51.13	1e-91	ccel:CCDG5_0227	K02499	yabN	tetrapyrrole methylase family protein / MazG family protein	--	--

[denovogenes]_101931	100.00	0.0	paeo:M801_2473	K00981	E2.7.7.41, CDS1, CDS2, cdsA	phosphatidate cytidylyltransferase	2.7.7.41	Metabolism--Lipid metabolism--Glycerophospholipid metabolism|Metabolism--Global and overview maps--Metabolic pathways|Metabolism--Global and overview maps--Biosynthesis of secondary metabolites|Environmental Information Processing--Signal transduction--Phosphatidylinositol signaling system

[denovogenes]_101932	75.65	1e-134	sns:VC03_02200	K02795	PTS-Man-EIIC, manY	PTS system, mannose-specific IIC component	--	Metabolism--Carbohydrate metabolism--Fructose and mannose metabolism|Metabolism--Carbohydrate metabolism--Amino sugar and nucleotide sugar metabolism|Metabolism--Global and overview maps--Metabolic pathways|Environmental Information Processing--Membrane transport--Phosphotransferase system (PTS)

[denovogenes]_101933	99.26	0.0	cfd:CFNIH1_15485	K02619	pabC	4-amino-4-deoxychorismate lyase	4.1.3.38	Metabolism--Metabolism of cofactors and vitamins--Folate biosynthesis

[denovogenes]_101935	56.88	4e-122	pow:IJ21_39060	K17319	lplB	putative aldouronate transport system permease protein	--	Environmental Information Processing--Membrane transport--ABC transporters

[denovogenes]_101936	95.93	3e-164	csh:Closa_1846	K02967	RP-S2, MRPS2, rpsB	small subunit ribosomal protein S2	--	Genetic Information Processing--Translation--Ribosome

[denovogenes]_101937	100.00	0.0	pdi:BDI_1192	K05807	bamD	outer membrane protein assembly factor BamD	--	--

[denovogenes]_101939	100.00	0.0	cfd:CFNIH1_12200	K00795	ispA	farnesyl diphosphate synthase	2.5.1.1 2.5.1.10	Metabolism--Metabolism of terpenoids and polyketides--Terpenoid backbone biosynthesis|Metabolism--Global and overview maps--Metabolic pathways|Metabolism--Global and overview maps--Biosynthesis of secondary metabolites|Metabolism--Global and overview maps--Biosynthesis of antibiotics

[denovogenes]_101940	68.52	7e-138	bhu:bhn_I2630	K02042	phnE	phosphonate transport system permease protein	--	Environmental Information Processing--Membrane transport--ABC transporters

[denovogenes]_101945	100.00	0.0	cfd:CFNIH1_07015	K02380	fdhE	FdhE protein	--	--

[denovogenes]_101946	92.22	0.0	bcel:BcellWH2_00935	K01209	abfA	alpha-N-arabinofuranosidase	3.2.1.55	Metabolism--Carbohydrate metabolism--Amino sugar and nucleotide sugar metabolism

[denovogenes]_101947	43.49	1e-74	esi:Exig_2824	K06221	dkgA	2,5-diketo-D-gluconate reductase A	1.1.1.346	--

[denovogenes]_101949	87.16	5e-167	rto:RTO_09200	K00065	kduD	2-deoxy-D-gluconate 3-dehydrogenase	1.1.1.125	Metabolism--Carbohydrate metabolism--Pentose and glucuronate interconversions

[denovogenes]_101951	99.63	0.0	pag:PLES_32381	K05549	benA-xylX	benzoate/toluate 1,2-dioxygenase subunit alpha	1.14.12.10 1.14.12.-	Metabolism--Xenobiotics biodegradation and metabolism--Benzoate degradation|Metabolism--Xenobiotics biodegradation and metabolism--Fluorobenzoate degradation|Metabolism--Xenobiotics biodegradation and metabolism--Xylene degradation|Metabolism--Global and overview maps--Metabolic pathways|Metabolism--Global and overview maps--Microbial metabolism in diverse environments|Metabolism--Global and overview maps--Degradation of aromatic compounds

[denovogenes]_101952	50.94	6e-91	eyy:EGYY_17530	K04486	E3.1.3.15B	histidinol-phosphatase (PHP family)	3.1.3.15	Metabolism--Amino acid metabolism--Histidine metabolism|Metabolism--Global and overview maps--Metabolic pathways|Metabolism--Global and overview maps--Biosynthesis of secondary metabolites|Metabolism--Global and overview maps--Biosynthesis of amino acids

[denovogenes]_101954	98.14	0.0	cfd:CFNIH1_19925	K00563	rlmA1	23S rRNA (guanine745-N1)-methyltransferase	2.1.1.187	--

[denovogenes]_101955	86.62	4e-163	csh:Closa_0265	K02042	phnE	phosphonate transport system permease protein	--	Environmental Information Processing--Membrane transport--ABC transporters

[denovogenes]_101958	98.88	0.0	pdi:BDI_0792	K01082	cysQ, MET22, BPNT1	3'(2'), 5'-bisphosphate nucleotidase	3.1.3.7	Metabolism--Energy metabolism--Sulfur metabolism|Metabolism--Global and overview maps--Metabolic pathways|Metabolism--Global and overview maps--Microbial metabolism in diverse environments|Metabolism--Global and overview maps--Biosynthesis of antibiotics

[denovogenes]_101959	98.51	0.0	bcel:BcellWH2_01482	K03783	punA, PNP	purine-nucleoside phosphorylase	2.4.2.1	Metabolism--Nucleotide metabolism--Purine metabolism|Metabolism--Nucleotide metabolism--Pyrimidine metabolism|Metabolism--Metabolism of cofactors and vitamins--Nicotinate and nicotinamide metabolism|Metabolism--Global and overview maps--Metabolic pathways|Metabolism--Global and overview maps--Biosynthesis of secondary metabolites

[denovogenes]_10196	80.38	0.0	rto:RTO_15660	K03168	topA	DNA topoisomerase I	5.99.1.2	--

[denovogenes]_101960	42.15	5e-40	pmf:P9303_05381	K00975	glgC	glucose-1-phosphate adenylyltransferase	2.7.7.27	Metabolism--Carbohydrate metabolism--Starch and sucrose metabolism|Metabolism--Carbohydrate metabolism--Amino sugar and nucleotide sugar metabolism|Metabolism--Global and overview maps--Metabolic pathways|Metabolism--Global and overview maps--Biosynthesis of secondary metabolites|Cellular Processes--Cellular community - prokaryotes--Biofilm formation - Escherichia coli

[denovogenes]_101966	70.26	2e-138	ear:CCG32700	K02440	GLPF	glycerol uptake facilitator protein	--	--

[denovogenes]_101967	45.95	5e-74	doi:FH5T_06225	K03768	PPIB, ppiB	peptidyl-prolyl cis-trans isomerase B (cyclophilin B)	5.2.1.8	--

[denovogenes]_101968	61.54	1e-108	lip:LI0018	K03309	TC.DAACS	dicarboxylate/amino acid:cation (Na+ or H+) symporter, DAACS family	--	--

[denovogenes]_10197	71.98	0.0	fpla:A4U99_16975	K08303	K08303	putative protease	3.4.-.-	Human Diseases--Infectious diseases: Bacterial--Epithelial cell signaling in Helicobacter pylori infection

[denovogenes]_101971	62.45	3e-118	dvm:DvMF_2926	K12574	rnj	ribonuclease J	3.1.-.-	Genetic Information Processing--Folding, sorting and degradation--RNA degradation

[denovogenes]_101972	82.16	3e-171	bvu:BVU_0598	K01629	rhaD	rhamnulose-1-phosphate aldolase	4.1.2.19	Metabolism--Carbohydrate metabolism--Pentose and glucuronate interconversions|Metabolism--Carbohydrate metabolism--Fructose and mannose metabolism|Metabolism--Global and overview maps--Microbial metabolism in diverse environments

[denovogenes]_101974	51.34	1e-96	sbr:SY1_18660	K02013	ABC.FEV.A	iron complex transport system ATP-binding protein	3.6.3.34	Environmental Information Processing--Membrane transport--ABC transporters

[denovogenes]_101975	88.85	0.0	bfb:VU15_11875	K01778	dapF	diaminopimelate epimerase	5.1.1.7	Metabolism--Amino acid metabolism--Lysine biosynthesis|Metabolism--Global and overview maps--Metabolic pathways|Metabolism--Global and overview maps--Biosynthesis of secondary metabolites|Metabolism--Global and overview maps--Microbial metabolism in diverse environments|Metabolism--Global and overview maps--Biosynthesis of antibiotics|Metabolism--Global and overview maps--Biosynthesis of amino acids

[denovogenes]_101977	60.55	1e-97	ele:Elen_1122	K09138	K09138	uncharacterized protein	--	--

[denovogenes]_101978	48.70	4e-58	afd:Alfi_1204	K01126	E3.1.4.46, glpQ, ugpQ	glycerophosphoryl diester phosphodiesterase	3.1.4.46	Metabolism--Lipid metabolism--Glycerophospholipid metabolism

[denovogenes]_101981	96.63	0.0	bcel:BcellWH2_02249	K07106	murQ	N-acetylmuramic acid 6-phosphate etherase	4.2.1.126	Metabolism--Carbohydrate metabolism--Amino sugar and nucleotide sugar metabolism|Metabolism--Global and overview maps--Metabolic pathways

[denovogenes]_101982	67.32	3e-124	bhl:Bache_1104	K03561	exbB	biopolymer transport protein ExbB	--	--

[denovogenes]_101984	82.59	2e-156	csh:Closa_1274	K01778	dapF	diaminopimelate epimerase	5.1.1.7	Metabolism--Amino acid metabolism--Lysine biosynthesis|Metabolism--Global and overview maps--Metabolic pathways|Metabolism--Global and overview maps--Biosynthesis of secondary metabolites|Metabolism--Global and overview maps--Microbial metabolism in diverse environments|Metabolism--Global and overview maps--Biosynthesis of antibiotics|Metabolism--Global and overview maps--Biosynthesis of amino acids

[denovogenes]_101986	85.56	7e-170	bprs:CK3_32140	K02945	RP-S1, rpsA	small subunit ribosomal protein S1	--	Genetic Information Processing--Translation--Ribosome

[denovogenes]_101987	52.40	4e-92	tbe:Trebr_2583	K02025	ABC.MS.P	multiple sugar transport system permease protein	--	--

[denovogenes]_10199	60.84	0.0	csh:Closa_3423	K03407	cheA	two-component system, chemotaxis family, sensor kinase CheA	2.7.13.3	Environmental Information Processing--Signal transduction--Two-component system|Cellular Processes--Cell motility--Bacterial chemotaxis

[denovogenes]_101990	97.03	0.0	bfb:VU15_00790	K03284	corA	magnesium transporter	--	--

[denovogenes]_101992	42.86	1e-72	lae:LBAT_1105	K02004	ABC.CD.P	putative ABC transport system permease protein	--	--

[denovogenes]_101994	42.80	4e-62	rum:CK1_17490	K01104	E3.1.3.48	protein-tyrosine phosphatase	3.1.3.48	--

[denovogenes]_101995	53.21	8e-94	lsa:LCA_1859	K00853	araB	L-ribulokinase	2.7.1.16	Metabolism--Carbohydrate metabolism--Pentose and glucuronate interconversions|Metabolism--Global and overview maps--Metabolic pathways

[denovogenes]_101997	97.01	0.0	cif:AL515_00610	K09798	K09798	uncharacterized protein	--	--

[denovogenes]_101998	99.26	0.0	boa:Bovatus_02451	K03783	punA, PNP	purine-nucleoside phosphorylase	2.4.2.1	Metabolism--Nucleotide metabolism--Purine metabolism|Metabolism--Nucleotide metabolism--Pyrimidine metabolism|Metabolism--Metabolism of cofactors and vitamins--Nicotinate and nicotinamide metabolism|Metabolism--Global and overview maps--Metabolic pathways|Metabolism--Global and overview maps--Biosynthesis of secondary metabolites

[denovogenes]_1020	99.92	0.0	pvl:AOB99_13505	K03582	recB	exodeoxyribonuclease V beta subunit	3.1.11.5	Genetic Information Processing--Replication and repair--Homologous recombination

[denovogenes]_102000	58.24	6e-120	plv:ERIC2_c30530	K17319	lplB	putative aldouronate transport system permease protein	--	Environmental Information Processing--Membrane transport--ABC transporters

[denovogenes]_102001	94.44	1e-169	bcel:BcellWH2_00514	K03313	nhaA	Na+:H+ antiporter, NhaA family	--	--

[denovogenes]_102005	81.93	2e-143	fpla:A4U99_09710	K02015	ABC.FEV.P	iron complex transport system permease protein	--	Environmental Information Processing--Membrane transport--ABC transporters

[denovogenes]_102006	50.93	3e-91	pro:HMPREF0669_00917	K03773	fklB	FKBP-type peptidyl-prolyl cis-trans isomerase FklB	5.2.1.8	--

[denovogenes]_102009	63.37	2e-112	csh:Closa_0133	K12510	tadB	tight adherence protein B	--	--

[denovogenes]_102011	57.03	8e-96	peo:AS203_07780	K12373	HEXA_B	hexosaminidase	3.2.1.52	Metabolism--Glycan biosynthesis and metabolism--Other glycan degradation|Metabolism--Glycan biosynthesis and metabolism--Various types of N-glycan biosynthesis|Metabolism--Carbohydrate metabolism--Amino sugar and nucleotide sugar metabolism|Metabolism--Glycan biosynthesis and metabolism--Glycosaminoglycan degradation|Metabolism--Glycan biosynthesis and metabolism--Glycosphingolipid biosynthesis - globo and isoglobo series|Metabolism--Glycan biosynthesis and metabolism--Glycosphingolipid biosynthesis - ganglio series|Metabolism--Global and overview maps--Metabolic pathways|Cellular Processes--Transport and catabolism--Lysosome

[denovogenes]_102012	57.62	2e-115	erb:A4V01_19030	K00662	aacC	aminoglycoside 3-N-acetyltransferase	2.3.1.81	--

[denovogenes]_102013	72.87	3e-138	fpr:FP2_04100	K03292	TC.GPH	glycoside/pentoside/hexuronide:cation symporter, GPH family	--	--

[denovogenes]_102014	94.40	0.0	cko:CKO_05126	K02073	metQ	D-methionine transport system substrate-binding protein	--	Environmental Information Processing--Membrane transport--ABC transporters

[denovogenes]_102017	64.81	3e-122	psi:S70_09040	K03655	recG	ATP-dependent DNA helicase RecG	3.6.4.12	Genetic Information Processing--Replication and repair--Homologous recombination

[denovogenes]_102018	100.00	0.0	bbrn:B2258_0215	K00036	G6PD, zwf	glucose-6-phosphate 1-dehydrogenase	1.1.1.49 1.1.1.363	Metabolism--Carbohydrate metabolism--Pentose phosphate pathway|Metabolism--Metabolism of other amino acids--Glutathione metabolism|Metabolism--Global and overview maps--Metabolic pathways|Metabolism--Global and overview maps--Biosynthesis of secondary metabolites|Metabolism--Global and overview maps--Microbial metabolism in diverse environments|Metabolism--Global and overview maps--Biosynthesis of antibiotics|Metabolism--Global and overview maps--Carbon metabolism|Human Diseases--Cancers: Overview--Central carbon metabolism in cancer

[denovogenes]_102019	94.92	3e-180	bhl:Bache_1522	K02428	rdgB	XTP/dITP diphosphohydrolase	3.6.1.66	Metabolism--Nucleotide metabolism--Purine metabolism

[denovogenes]_10202	41.71	2e-85	noc:Noc_1826	K00705	malQ	4-alpha-glucanotransferase	2.4.1.25	Metabolism--Carbohydrate metabolism--Starch and sucrose metabolism|Metabolism--Global and overview maps--Metabolic pathways

[denovogenes]_102021	99.63	0.0	elr:ECO55CA74_22565	K00848	rhaB	rhamnulokinase	2.7.1.5	Metabolism--Carbohydrate metabolism--Pentose and glucuronate interconversions|Metabolism--Carbohydrate metabolism--Fructose and mannose metabolism|Metabolism--Global and overview maps--Microbial metabolism in diverse environments

[denovogenes]_102024	100.00	0.0	cfd:CFNIH1_08280	K07792	dcuB	anaerobic C4-dicarboxylate transporter DcuB	--	Environmental Information Processing--Signal transduction--Two-component system

[denovogenes]_102026	93.70	0.0	pdi:BDI_1452	K03553	recA	recombination protein RecA	--	Genetic Information Processing--Replication and repair--Homologous recombination

[denovogenes]_102027	100.00	0.0	pdf:CD630DERM_16650	K01738	cysK	cysteine synthase A	2.5.1.47	Metabolism--Amino acid metabolism--Cysteine and methionine metabolism|Metabolism--Energy metabolism--Sulfur metabolism|Metabolism--Global and overview maps--Metabolic pathways|Metabolism--Global and overview maps--Biosynthesis of secondary metabolites|Metabolism--Global and overview maps--Microbial metabolism in diverse environments|Metabolism--Global and overview maps--Biosynthesis of antibiotics|Metabolism--Global and overview maps--Carbon metabolism|Metabolism--Global and overview maps--Biosynthesis of amino acids

[denovogenes]_10203	45.03	0.0	fpl:Ferp_2326	K01473	hyuA	N-methylhydantoinase A	3.5.2.14	Metabolism--Amino acid metabolism--Arginine and proline metabolism|Metabolism--Global and overview maps--Metabolic pathways

[denovogenes]_102030	85.56	2e-168	kpu:KP1_2270	K03781	katE, CAT, catB, srpA	catalase	1.11.1.6	Metabolism--Amino acid metabolism--Tryptophan metabolism|Metabolism--Carbohydrate metabolism--Glyoxylate and dicarboxylate metabolism|Metabolism--Global and overview maps--Biosynthesis of secondary metabolites|Metabolism--Global and overview maps--Biosynthesis of antibiotics|Metabolism--Global and overview maps--Carbon metabolism|Environmental Information Processing--Signal transduction--MAPK signaling pathway - yeast|Environmental Information Processing--Signal transduction--MAPK signaling pathway - plant|Environmental Information Processing--Signal transduction--FoxO signaling pathway|Cellular Processes--Transport and catabolism--Peroxisome|Organismal Systems--Aging--Longevity regulating pathway|Organismal Systems--Aging--Longevity regulating pathway - worm|Organismal Systems--Aging--Longevity regulating pathway - multiple species|Human Diseases--Neurodegenerative diseases--Amyotrophic lateral sclerosis (ALS)

[denovogenes]_102031	63.94	5e-130	euu:ADJ67_03900	K01775	alr	alanine racemase	5.1.1.1	Metabolism--Metabolism of other amino acids--D-Alanine metabolism|Metabolism--Global and overview maps--Metabolic pathways|Human Diseases--Drug resistance: Antimicrobial--Vancomycin resistance

[denovogenes]_102033	100.00	0.0	pdf:CD630DERM_01070	K00812	aspB	aspartate aminotransferase	2.6.1.1	Metabolism--Amino acid metabolism--Arginine biosynthesis|Metabolism--Amino acid metabolism--Alanine, aspartate and glutamate metabolism|Metabolism--Amino acid metabolism--Cysteine and methionine metabolism|Metabolism--Amino acid metabolism--Arginine and proline metabolism|Metabolism--Amino acid metabolism--Tyrosine metabolism|Metabolism--Amino acid metabolism--Phenylalanine metabolism|Metabolism--Amino acid metabolism--Phenylalanine, tyrosine and tryptophan biosynthesis|Metabolism--Biosynthesis of other secondary metabolites--Novobiocin biosynthesis|Metabolism--Biosynthesis of other secondary metabolites--Isoquinoline alkaloid biosynthesis|Metabolism--Biosynthesis of other secondary metabolites--Tropane, piperidine and pyridine alkaloid biosynthesis|Metabolism--Global and overview maps--Metabolic pathways|Metabolism--Global and overview maps--Biosynthesis of secondary metabolites|Metabolism--Global and overview maps--Biosynthesis of antibiotics|Metabolism--Global and overview maps--2-Oxocarboxylic acid metabolism|Metabolism--Global and overview maps--Biosynthesis of amino acids

[denovogenes]_102038	44.65	3e-68	raq:Rahaq2_2216	K06610	iolF	MFS transporter, SP family, inositol transporter	--	--

[denovogenes]_102040	54.24	2e-97	aoe:Clos_0274	K03315	nhaC	Na+:H+ antiporter, NhaC family	--	--

[denovogenes]_102045	99.26	0.0	cif:AL515_18135	K08305	mltB	membrane-bound lytic murein transglycosylase B	--	--

[denovogenes]_10205	42.52	0.0	byl:A4V09_08355	K07814	K07814	putative two-component system response regulator	--	--

[denovogenes]_102051	88.52	6e-178	afd:Alfi_0434	K02117	ATPVA, ntpA, atpA	V/A-type H+/Na+-transporting ATPase subunit A	3.6.3.14 3.6.3.15	Metabolism--Energy metabolism--Oxidative phosphorylation|Metabolism--Global and overview maps--Metabolic pathways

[denovogenes]_102054	55.67	4e-96	pit:PIN17_A1510	K03169	topB	DNA topoisomerase III	5.99.1.2	--

[denovogenes]_102056	61.15	1e-115	tfo:BFO_1070	K00648	fabH	3-oxoacyl-[acyl-carrier-protein] synthase III	2.3.1.180	Metabolism--Lipid metabolism--Fatty acid biosynthesis|Metabolism--Global and overview maps--Metabolic pathways|Metabolism--Global and overview maps--Fatty acid metabolism

[denovogenes]_102058	40.23	3e-55	euc:EC1_17290	K02004	ABC.CD.P	putative ABC transport system permease protein	--	--

[denovogenes]_10206	81.61	0.0	cso:CLS_24180	K03168	topA	DNA topoisomerase I	5.99.1.2	--

[denovogenes]_102060	40.51	2e-33	prt:AUC31_01390	K00059	fabG	3-oxoacyl-[acyl-carrier protein] reductase	1.1.1.100	Metabolism--Lipid metabolism--Fatty acid biosynthesis|Metabolism--Metabolism of cofactors and vitamins--Biotin metabolism|Metabolism--Lipid metabolism--Biosynthesis of unsaturated fatty acids|Metabolism--Global and overview maps--Metabolic pathways|Metabolism--Global and overview maps--Fatty acid metabolism

[denovogenes]_102062	95.70	0.0	bxy:BXY_28940	K03734	apbE	FAD:protein FMN transferase	2.7.1.180	--

[denovogenes]_102063	98.88	0.0	boa:Bovatus_03888	K01629	rhaD	rhamnulose-1-phosphate aldolase	4.1.2.19	Metabolism--Carbohydrate metabolism--Pentose and glucuronate interconversions|Metabolism--Carbohydrate metabolism--Fructose and mannose metabolism|Metabolism--Global and overview maps--Microbial metabolism in diverse environments

[denovogenes]_102065	98.14	0.0	boa:Bovatus_01200	K01778	dapF	diaminopimelate epimerase	5.1.1.7	Metabolism--Amino acid metabolism--Lysine biosynthesis|Metabolism--Global and overview maps--Metabolic pathways|Metabolism--Global and overview maps--Biosynthesis of secondary metabolites|Metabolism--Global and overview maps--Microbial metabolism in diverse environments|Metabolism--Global and overview maps--Biosynthesis of antibiotics|Metabolism--Global and overview maps--Biosynthesis of amino acids

[denovogenes]_102073	98.88	0.0	efe:EFER_3913	K12981	waaZ, rfaZ	KDO transferase III	2.4.99.-	Metabolism--Glycan biosynthesis and metabolism--Lipopolysaccharide biosynthesis

[denovogenes]_102074	97.38	0.0	boa:Bovatus_00229	K01991	ABC-2.OM, wza	polysaccharide biosynthesis/export protein	--	Cellular Processes--Cellular community - prokaryotes--Biofilm formation - Escherichia coli

[denovogenes]_102078	100.00	0.0	pvl:AOB99_08840	K19230	sapF	cationic peptide transport system ATP-binding protein	--	Human Diseases--Drug resistance: Antimicrobial--Cationic antimicrobial peptide (CAMP) resistance|Environmental Information Processing--Membrane transport--ABC transporters

[denovogenes]_10208	46.79	5e-81	tae:TepiRe1_2022	K03721	tyrR	transcriptional regulator of aroF, aroG, tyrA and aromatic amino acid transport	--	--

[denovogenes]_102080	67.29	2e-126	ipo:Ilyop_1714	K06442	tlyA	23S rRNA (cytidine1920-2'-O)/16S rRNA (cytidine1409-2'-O)-methyltransferase	2.1.1.226 2.1.1.227	--

[denovogenes]_102082	86.25	1e-170	ega:AL523_00945	K11071	potB	spermidine/putrescine transport system permease protein	--	Environmental Information Processing--Membrane transport--ABC transporters

[denovogenes]_102084	100.00	0.0	pvl:AOB99_05925	K06212	focA	formate transporter	--	--

[denovogenes]_102085	80.38	9e-156	vpr:Vpar_1358	K03429	ugtP	processive 1,2-diacylglycerol beta-glucosyltransferase	2.4.1.315	Metabolism--Lipid metabolism--Glycerolipid metabolism|Metabolism--Global and overview maps--Metabolic pathways

[denovogenes]_102087	100.00	0.0	bvu:BVU_1602	K03783	punA, PNP	purine-nucleoside phosphorylase	2.4.2.1	Metabolism--Nucleotide metabolism--Purine metabolism|Metabolism--Nucleotide metabolism--Pyrimidine metabolism|Metabolism--Metabolism of cofactors and vitamins--Nicotinate and nicotinamide metabolism|Metabolism--Global and overview maps--Metabolic pathways|Metabolism--Global and overview maps--Biosynthesis of secondary metabolites

[denovogenes]_102089	100.00	0.0	ecm:EcSMS35_2675	K07751	pepB	PepB aminopeptidase	3.4.11.23	Metabolism--Metabolism of other amino acids--Glutathione metabolism|Metabolism--Global and overview maps--Metabolic pathways

[denovogenes]_102090	74.72	5e-152	bdo:EL88_07255	K01012	bioB	biotin synthase	2.8.1.6	Metabolism--Metabolism of cofactors and vitamins--Biotin metabolism|Metabolism--Global and overview maps--Metabolic pathways

[denovogenes]_102091	54.83	5e-96	tur:AT726_05330	K02548	menA	1,4-dihydroxy-2-naphthoate octaprenyltransferase	2.5.1.74 2.5.1.-	Metabolism--Metabolism of cofactors and vitamins--Ubiquinone and other terpenoid-quinone biosynthesis|Metabolism--Global and overview maps--Metabolic pathways|Metabolism--Global and overview maps--Biosynthesis of secondary metabolites

[denovogenes]_102092	57.26	1e-89	gfo:GFO_0359	K01138	K01138	uncharacterized sulfatase	3.1.6.-	--

[denovogenes]_102095	52.91	7e-80	cama:F384_26955	K02338	DPO3B, dnaN	DNA polymerase III subunit beta	2.7.7.7	Metabolism--Nucleotide metabolism--Purine metabolism|Metabolism--Nucleotide metabolism--Pyrimidine metabolism|Metabolism--Global and overview maps--Metabolic pathways|Genetic Information Processing--Replication and repair--DNA replication|Genetic Information Processing--Replication and repair--Mismatch repair|Genetic Information Processing--Replication and repair--Homologous recombination

[denovogenes]_102096	46.27	2e-88	csh:Closa_2388	K10118	msmF	raffinose/stachyose/melibiose transport system permease protein	--	Environmental Information Processing--Membrane transport--ABC transporters

[denovogenes]_102098	79.40	4e-150	vpr:Vpar_1847	K03497	parB, spo0J	chromosome partitioning protein, ParB family	--	--

[denovogenes]_1021	100.00	0.0	paeg:AI22_28515	K07679	evgS, bvgS	two-component system, NarL family, sensor histidine kinase EvgS	2.7.13.3	Environmental Information Processing--Signal transduction--Two-component system|Human Diseases--Infectious diseases: Bacterial--Pertussis

[denovogenes]_102100	99.18	1e-178	ecol:LY180_12235	K01577	oxc	oxalyl-CoA decarboxylase	4.1.1.8	Metabolism--Carbohydrate metabolism--Glyoxylate and dicarboxylate metabolism|Metabolism--Global and overview maps--Metabolic pathways

[denovogenes]_102106	100.00	0.0	shq:A0259_19080	K03086	SIG1, rpoD	RNA polymerase primary sigma factor	--	--

[denovogenes]_102107	100.00	0.0	mmk:MU9_1779	K02389	flgD	flagellar basal-body rod modification protein FlgD	--	Cellular Processes--Cell motility--Flagellar assembly

[denovogenes]_102108	99.63	0.0	mmk:MU9_1916	K06897	K06897	7,8-dihydropterin-6-yl-methyl-4-(beta-D-ribofuranosyl)aminobenzene 5'-phosphate synthase	2.5.1.105	--

[denovogenes]_10211	82.80	0.0	pdi:BDI_3779	K01277	DPP3	dipeptidyl-peptidase III	3.4.14.4	--

[denovogenes]_102110	100.00	0.0	mmk:MU9_1744	K08358	ttrB	tetrathionate reductase subunit B	--	Metabolism--Energy metabolism--Sulfur metabolism|Metabolism--Global and overview maps--Microbial metabolism in diverse environments|Environmental Information Processing--Signal transduction--Two-component system

[denovogenes]_102115	98.77	4e-177	mmk:MU9_1154	K06858	ABC.VB12.S1, btuF	vitamin B12 transport system substrate-binding protein	--	Environmental Information Processing--Membrane transport--ABC transporters

[denovogenes]_102116	79.85	5e-150	ega:AL523_12845	K10039	ABC.GLN1.S	putative glutamine transport system substrate-binding protein	--	Environmental Information Processing--Membrane transport--ABC transporters

[denovogenes]_102117	99.24	0.0	mmk:MU9_586	K03591	ftsN	cell division protein FtsN	--	--

[denovogenes]_102118	74.24	1e-143	ess:ATZ33_13250	K00931	proB	glutamate 5-kinase	2.7.2.11	Metabolism--Amino acid metabolism--Arginine and proline metabolism|Metabolism--Biosynthesis of other secondary metabolites--Carbapenem biosynthesis|Metabolism--Global and overview maps--Metabolic pathways|Metabolism--Global and overview maps--Biosynthesis of antibiotics|Metabolism--Global and overview maps--Biosynthesis of amino acids

[denovogenes]_102120	88.81	1e-159	ega:AL523_06105	K02746	PTS-Aga-EIIC, agaW	PTS system, N-acetylgalactosamine-specific IIC component	--	Metabolism--Carbohydrate metabolism--Galactose metabolism|Environmental Information Processing--Membrane transport--Phosphotransferase system (PTS)

[denovogenes]_102121	47.55	2e-82	eft:M395_03700	K16869	lipL	octanoyl-[GcvH]:protein N-octanoyltransferase	2.3.1.204	--

[denovogenes]_102122	89.18	2e-179	ehr:EHR_08650	K02036	pstB	phosphate transport system ATP-binding protein	3.6.3.27	Environmental Information Processing--Membrane transport--ABC transporters

[denovogenes]_102123	100.00	0.0	mmk:MU9_2283	K01695	trpA	tryptophan synthase alpha chain	4.2.1.20	Metabolism--Amino acid metabolism--Glycine, serine and threonine metabolism|Metabolism--Amino acid metabolism--Phenylalanine, tyrosine and tryptophan biosynthesis|Metabolism--Global and overview maps--Metabolic pathways|Metabolism--Global and overview maps--Biosynthesis of secondary metabolites|Metabolism--Global and overview maps--Biosynthesis of antibiotics|Metabolism--Global and overview maps--Biosynthesis of amino acids

[denovogenes]_102124	99.63	0.0	mmk:MU9_808	K02034	ABC.PE.P1	peptide/nickel transport system permease protein	--	Cellular Processes--Cellular community - prokaryotes--Quorum sensing

[denovogenes]_102125	100.00	0.0	bfs:BF9343_4079	K01092	E3.1.3.25, IMPA, suhB	myo-inositol-1(or 4)-monophosphatase	3.1.3.25	Metabolism--Biosynthesis of other secondary metabolites--Streptomycin biosynthesis|Metabolism--Carbohydrate metabolism--Inositol phosphate metabolism|Metabolism--Global and overview maps--Metabolic pathways|Environmental Information Processing--Signal transduction--Phosphatidylinositol signaling system

[denovogenes]_102128	70.08	4e-139	cbt:CLH_0097	K03424	--	--	--	--

[denovogenes]_102129	54.75	2e-108	cbk:CLL_A2469	K03442	mscS	small conductance mechanosensitive channel	--	--

[denovogenes]_102130	80.31	3e-159	lacy:A4V08_17715	K01151	nfo	deoxyribonuclease IV	3.1.21.2	Genetic Information Processing--Replication and repair--Base excision repair

[denovogenes]_102132	56.25	7e-107	emu:EMQU_0977	K02030	ABC.PA.S	polar amino acid transport system substrate-binding protein	--	--

[denovogenes]_102133	100.00	0.0	bfb:VU15_09750	K07001	K07001	NTE family protein	--	--

[denovogenes]_102134	56.22	1e-84	ehr:EHR_07940	K06872	K06872	uncharacterized protein	--	--

[denovogenes]_102135	70.90	3e-139	lacy:A4V08_30800	K02823	pyrDII	dihydroorotate dehydrogenase electron transfer subunit	--	Metabolism--Nucleotide metabolism--Pyrimidine metabolism|Metabolism--Global and overview maps--Metabolic pathways

[denovogenes]_102136	43.55	1e-22	ecoh:ECRM13516_5335	K02317	dnaT	DNA replication protein DnaT	--	Genetic Information Processing--Replication and repair--Homologous recombination

[denovogenes]_102139	71.10	9e-143	pdc:CDIF630_01614	K00796	folP	dihydropteroate synthase	2.5.1.15	Metabolism--Metabolism of cofactors and vitamins--Folate biosynthesis|Metabolism--Global and overview maps--Metabolic pathways

[denovogenes]_10214	95.53	0.0	bfg:BF638R_3531	K00937	ppk	polyphosphate kinase	2.7.4.1	Metabolism--Energy metabolism--Oxidative phosphorylation|Genetic Information Processing--Folding, sorting and degradation--RNA degradation

[denovogenes]_102140	99.63	0.0	bfb:VU15_17975	K06897	K06897	7,8-dihydropterin-6-yl-methyl-4-(beta-D-ribofuranosyl)aminobenzene 5'-phosphate synthase	2.5.1.105	--

[denovogenes]_102145	100.00	0.0	efi:OG1RF_10137	K02013	ABC.FEV.A	iron complex transport system ATP-binding protein	3.6.3.34	Environmental Information Processing--Membrane transport--ABC transporters

[denovogenes]_102148	56.77	4e-100	cdg:CDBI1_04190	K02020	modA	molybdate transport system substrate-binding protein	--	Environmental Information Processing--Membrane transport--ABC transporters

[denovogenes]_102150	68.80	1e-133	cdg:CDBI1_08400	K03148	thiF	sulfur carrier protein ThiS adenylyltransferase	2.7.7.73	Metabolism--Metabolism of cofactors and vitamins--Thiamine metabolism|Metabolism--Global and overview maps--Metabolic pathways|Genetic Information Processing--Folding, sorting and degradation--Sulfur relay system

[denovogenes]_102151	100.00	0.0	stb:SGPB_1298	K01992	ABC-2.P	ABC-2 type transport system permease protein	--	--

[denovogenes]_102152	74.72	8e-143	efm:M7W_1192	K00981	E2.7.7.41, CDS1, CDS2, cdsA	phosphatidate cytidylyltransferase	2.7.7.41	Metabolism--Lipid metabolism--Glycerophospholipid metabolism|Metabolism--Global and overview maps--Metabolic pathways|Metabolism--Global and overview maps--Biosynthesis of secondary metabolites|Environmental Information Processing--Signal transduction--Phosphatidylinositol signaling system

[denovogenes]_102160	50.37	2e-90	esi:Exig_2824	K06221	dkgA	2,5-diketo-D-gluconate reductase A	1.1.1.346	--

[denovogenes]_102161	76.40	6e-158	rho:RHOM_04105	K10190	lacG, araQ	lactose/L-arabinose transport system permease protein	--	Environmental Information Processing--Membrane transport--ABC transporters

[denovogenes]_102163	87.64	1e-172	cbz:Cbs_3760	K02013	ABC.FEV.A	iron complex transport system ATP-binding protein	3.6.3.34	Environmental Information Processing--Membrane transport--ABC transporters

[denovogenes]_102164	63.02	2e-107	clt:CM240_2756	K06409	spoVB	stage V sporulation protein B	--	--

[denovogenes]_102166	100.00	0.0	ene:ENT_18220	K11071	potB	spermidine/putrescine transport system permease protein	--	Environmental Information Processing--Membrane transport--ABC transporters

[denovogenes]_102168	99.25	0.0	elo:EC042_4389	K00068	srlD	sorbitol-6-phosphate 2-dehydrogenase	1.1.1.140	Metabolism--Carbohydrate metabolism--Fructose and mannose metabolism

[denovogenes]_102169	40.58	3e-19	ooe:OEOE_1003	K04763	xerD	integrase/recombinase XerD	--	--

[denovogenes]_102170	50.94	2e-94	rto:RTO_23090	K01776	murI	glutamate racemase	5.1.1.3	Metabolism--Metabolism of other amino acids--D-Glutamine and D-glutamate metabolism|Metabolism--Global and overview maps--Metabolic pathways

[denovogenes]_102171	99.25	0.0	cif:AL515_13050	K19230	sapF	cationic peptide transport system ATP-binding protein	--	Human Diseases--Drug resistance: Antimicrobial--Cationic antimicrobial peptide (CAMP) resistance|Environmental Information Processing--Membrane transport--ABC transporters

[denovogenes]_102172	67.92	2e-122	csb:CLSA_c14030	K03470	rnhB	ribonuclease HII	3.1.26.4	Genetic Information Processing--Replication and repair--DNA replication

[denovogenes]_102173	60.74	7e-118	llm:llmg_0412	K00784	rnz	ribonuclease Z	3.1.26.11	Genetic Information Processing--Translation--RNA transport

[denovogenes]_102174	99.63	0.0	cbut:ATN24_18285	K02795	PTS-Man-EIIC, manY	PTS system, mannose-specific IIC component	--	Metabolism--Carbohydrate metabolism--Fructose and mannose metabolism|Metabolism--Carbohydrate metabolism--Amino sugar and nucleotide sugar metabolism|Metabolism--Global and overview maps--Metabolic pathways|Environmental Information Processing--Membrane transport--Phosphotransferase system (PTS)

[denovogenes]_102176	100.00	0.0	pmib:BB2000_0288	K18890	mdlB, smdB	ATP-binding cassette, subfamily B, multidrug efflux pump	--	Environmental Information Processing--Membrane transport--ABC transporters

[denovogenes]_102177	68.01	3e-121	mhg:MHY_01180	K02040	pstS	phosphate transport system substrate-binding protein	--	Environmental Information Processing--Membrane transport--ABC transporters|Environmental Information Processing--Signal transduction--Two-component system|Human Diseases--Infectious diseases: Bacterial--Tuberculosis

[denovogenes]_102178	93.26	0.0	cfd:CFNIH1_02875	K02081	agaR	DeoR family transcriptional regulator, aga operon transcriptional repressor	--	--

[denovogenes]_102179	63.20	2e-126	cpy:Cphy_1451	K02042	phnE	phosphonate transport system permease protein	--	Environmental Information Processing--Membrane transport--ABC transporters

[denovogenes]_10218	99.57	0.0	cfd:CFNIH1_05915	K03655	recG	ATP-dependent DNA helicase RecG	3.6.4.12	Genetic Information Processing--Replication and repair--Homologous recombination

[denovogenes]_102181	78.36	4e-157	csb:CLSA_c28650	K01892	HARS, hisS	histidyl-tRNA synthetase	6.1.1.21	Genetic Information Processing--Translation--Aminoacyl-tRNA biosynthesis

[denovogenes]_102182	95.52	0.0	cfd:CFNIH1_18600	K19802	ycjG, ykfB	L-Ala-D/L-Glu epimerase	5.1.1.20	--

[denovogenes]_102184	97.01	0.0	cif:AL515_13195	K01695	trpA	tryptophan synthase alpha chain	4.2.1.20	Metabolism--Amino acid metabolism--Glycine, serine and threonine metabolism|Metabolism--Amino acid metabolism--Phenylalanine, tyrosine and tryptophan biosynthesis|Metabolism--Global and overview maps--Metabolic pathways|Metabolism--Global and overview maps--Biosynthesis of secondary metabolites|Metabolism--Global and overview maps--Biosynthesis of antibiotics|Metabolism--Global and overview maps--Biosynthesis of amino acids

[denovogenes]_102185	52.87	3e-97	eum:ECUMN_0354	K13244	yahA	c-di-GMP-specific phosphodiesterase	3.1.4.52	--

[denovogenes]_102187	79.55	9e-159	cpe:CPE2242	K09773	ppsR	[pyruvate, water dikinase]-phosphate phosphotransferase / [pyruvate, water dikinase] kinase	2.7.4.28 2.7.11.33	--

[denovogenes]_102188	48.46	9e-63	soz:Spy49_1506c	K02315	dnaC	DNA replication protein DnaC	--	--

[denovogenes]_102189	44.74	1e-77	csr:Cspa_c38380	K03426	E3.6.1.22, NUDT12, nudC	NAD+ diphosphatase	3.6.1.22	Metabolism--Metabolism of cofactors and vitamins--Nicotinate and nicotinamide metabolism|Metabolism--Global and overview maps--Metabolic pathways|Cellular Processes--Transport and catabolism--Peroxisome

[denovogenes]_102193	67.55	7e-128	bhu:bhn_I0749	K04485	radA, sms	DNA repair protein RadA/Sms	--	--

[denovogenes]_102194	99.25	0.0	pmib:BB2000_0355	K00606	panB	3-methyl-2-oxobutanoate hydroxymethyltransferase	2.1.2.11	Metabolism--Metabolism of cofactors and vitamins--Pantothenate and CoA biosynthesis|Metabolism--Global and overview maps--Metabolic pathways|Metabolism--Global and overview maps--Biosynthesis of secondary metabolites

[denovogenes]_102196	63.53	1e-122	ipo:Ilyop_0201	K02108	ATPF0A, atpB	F-type H+-transporting ATPase subunit a	--	Metabolism--Energy metabolism--Oxidative phosphorylation|Metabolism--Energy metabolism--Photosynthesis|Metabolism--Global and overview maps--Metabolic pathways

[denovogenes]_102198	98.51	0.0	cif:AL515_16205	K14261	alaC	alanine-synthesizing transaminase	2.6.1.-	--

[denovogenes]_102199	100.00	0.0	ecoh:ECRM13516_2046	K10680	nemA	N-ethylmaleimide reductase	1.-.-.-	Metabolism--Xenobiotics biodegradation and metabolism--Nitrotoluene degradation|Metabolism--Global and overview maps--Microbial metabolism in diverse environments

[denovogenes]_10220	83.69	0.0	pdi:BDI_3344	K12573	rnr, vacB	ribonuclease R	3.1.-.-	Genetic Information Processing--Folding, sorting and degradation--RNA degradation

[denovogenes]_102201	57.62	2e-110	ctet:BN906_00615	K19294	algI	alginate O-acetyltransferase complex protein AlgI	--	--

[denovogenes]_102202	60.22	3e-115	cpf:CPF_2652	K15770	cycB, ganO	arabinogalactan oligomer / maltooligosaccharide transport system substrate-binding protein	--	Environmental Information Processing--Membrane transport--ABC transporters

[denovogenes]_102203	79.39	4e-152	fhw:RN87_10135	K01876	DARS, aspS	aspartyl-tRNA synthetase	6.1.1.12	Genetic Information Processing--Translation--Aminoacyl-tRNA biosynthesis

[denovogenes]_102204	96.64	0.0	cbut:ATN24_13455	K00784	rnz	ribonuclease Z	3.1.26.11	Genetic Information Processing--Translation--RNA transport

[denovogenes]_102207	64.38	3e-106	tfo:BFO_0082	K06147	ABCB-BAC	ATP-binding cassette, subfamily B, bacterial	--	--

[denovogenes]_102208	98.88	0.0	cfd:CFNIH1_13775	K19268	glmE, mutE, mamB	methylaspartate mutase epsilon subunit	5.4.99.1	Metabolism--Carbohydrate metabolism--Glyoxylate and dicarboxylate metabolism|Metabolism--Carbohydrate metabolism--C5-Branched dibasic acid metabolism|Metabolism--Global and overview maps--Metabolic pathways|Metabolism--Global and overview maps--Carbon metabolism

[denovogenes]_102209	53.56	9e-93	cle:Clole_4269	K06346	jag	spoIIIJ-associated protein	--	--

[denovogenes]_102211	51.94	5e-89	amt:Amet_3518	K02592	nifN	nitrogenase molybdenum-iron protein NifN	--	--

[denovogenes]_102212	73.41	4e-142	fus:HMPREF0409_02272	K01443	nagA, AMDHD2	N-acetylglucosamine-6-phosphate deacetylase	3.5.1.25	Metabolism--Carbohydrate metabolism--Amino sugar and nucleotide sugar metabolism|Metabolism--Global and overview maps--Biosynthesis of antibiotics

[denovogenes]_102213	57.58	3e-105	spnn:T308_10360	K02796	PTS-Man-EIID, manZ	PTS system, mannose-specific IID component	--	Metabolism--Carbohydrate metabolism--Fructose and mannose metabolism|Metabolism--Carbohydrate metabolism--Amino sugar and nucleotide sugar metabolism|Metabolism--Global and overview maps--Metabolic pathways|Environmental Information Processing--Membrane transport--Phosphotransferase system (PTS)

[denovogenes]_102215	51.31	2e-86	csh:Closa_1148	K07483	K07483	transposase	--	--

[denovogenes]_102216	91.79	0.0	cbi:CLJ_B0343	K01232	glvA	maltose-6'-phosphate glucosidase	3.2.1.122	Metabolism--Carbohydrate metabolism--Starch and sucrose metabolism

[denovogenes]_102217	66.08	4e-113	cbl:CLK_0467	K01560	E3.8.1.2	2-haloacid dehalogenase	3.8.1.2	Metabolism--Xenobiotics biodegradation and metabolism--Chlorocyclohexane and chlorobenzene degradation|Metabolism--Xenobiotics biodegradation and metabolism--Chloroalkane and chloroalkene degradation|Metabolism--Global and overview maps--Metabolic pathways|Metabolism--Global and overview maps--Microbial metabolism in diverse environments

[denovogenes]_102220	40.52	3e-19	sgp:SpiGrapes_1044	K07720	yesN	two-component system, response regulator YesN	--	Environmental Information Processing--Signal transduction--Two-component system

[denovogenes]_102222	100.00	0.0	cbut:ATN24_03660	K06436	yabG	spore coat assemly protein	--	--

[denovogenes]_102225	42.69	6e-61	eli:ELI_13895	K12941	abgB	aminobenzoyl-glutamate utilization protein B	--	--

[denovogenes]_102226	95.00	2e-171	cfd:CFNIH1_14395	K10678	nfsA	nitroreductase	1.-.-.-	Metabolism--Xenobiotics biodegradation and metabolism--Nitrotoluene degradation|Metabolism--Global and overview maps--Microbial metabolism in diverse environments

[denovogenes]_102227	84.01	8e-170	cbt:CLH_1018	K00600	glyA, SHMT	glycine hydroxymethyltransferase	2.1.2.1	Metabolism--Amino acid metabolism--Glycine, serine and threonine metabolism|Metabolism--Metabolism of other amino acids--Cyanoamino acid metabolism|Metabolism--Carbohydrate metabolism--Glyoxylate and dicarboxylate metabolism|Metabolism--Metabolism of cofactors and vitamins--One carbon pool by folate|Metabolism--Energy metabolism--Methane metabolism|Metabolism--Global and overview maps--Metabolic pathways|Metabolism--Global and overview maps--Biosynthesis of secondary metabolites|Metabolism--Global and overview maps--Microbial metabolism in diverse environments|Metabolism--Global and overview maps--Biosynthesis of antibiotics|Metabolism--Global and overview maps--Carbon metabolism|Metabolism--Global and overview maps--Biosynthesis of amino acids|Human Diseases--Drug resistance: Antineoplastic--Antifolate resistance

[denovogenes]_102228	51.65	5e-83	bif:N288_04435	K07003	K07003	uncharacterized protein	--	--

[denovogenes]_102229	78.81	2e-157	psi:S70_11790	K18148	rtcB	release factor H-coupled RctB family protein	--	Human Diseases--Drug resistance: Antimicrobial--beta-Lactam resistance

[denovogenes]_10223	99.71	0.0	cfd:CFNIH1_03295	K07648	arcB	two-component system, OmpR family, aerobic respiration control sensor histidine kinase ArcB	2.7.13.3	Environmental Information Processing--Signal transduction--Two-component system|Cellular Processes--Cellular community - prokaryotes--Biofilm formation - Escherichia coli

[denovogenes]_102230	55.39	2e-95	bon:A361_24415	K02809	PTS-Scr-EIIB, scrA, sacP, sacX, ptsS	PTS system, sucrose-specific IIB component	2.7.1.211	Metabolism--Carbohydrate metabolism--Starch and sucrose metabolism|Environmental Information Processing--Membrane transport--Phosphotransferase system (PTS)

[denovogenes]_102230	55.39	2e-95	bon:A361_24415	K02810	PTS-Scr-EIIC, scrA, sacP, sacX, ptsS	PTS system, sucrose-specific IIC component	--	Metabolism--Carbohydrate metabolism--Starch and sucrose metabolism|Environmental Information Processing--Membrane transport--Phosphotransferase system (PTS)

[denovogenes]_102231	63.42	2e-92	cpro:CPRO_09320	K03406	mcp	methyl-accepting chemotaxis protein	--	Environmental Information Processing--Signal transduction--Two-component system|Cellular Processes--Cell motility--Bacterial chemotaxis

[denovogenes]_102233	66.67	2e-128	cbut:ATN24_09285	K04759	feoB	ferrous iron transport protein B	--	--

[denovogenes]_102234	78.81	2e-159	rix:RO1_18030	K07407	E3.2.1.22B, galA, rafA	alpha-galactosidase	3.2.1.22	Metabolism--Carbohydrate metabolism--Galactose metabolism|Metabolism--Lipid metabolism--Glycerolipid metabolism|Metabolism--Lipid metabolism--Sphingolipid metabolism|Metabolism--Glycan biosynthesis and metabolism--Glycosphingolipid biosynthesis - globo and isoglobo series

[denovogenes]_102235	83.64	2e-143	cbv:U729_2817	K03110	ftsY	fused signal recognition particle receptor	--	Cellular Processes--Cellular community - prokaryotes--Quorum sensing|Genetic Information Processing--Folding, sorting and degradation--Protein export|Environmental Information Processing--Membrane transport--Bacterial secretion system

[denovogenes]_102237	56.28	1e-88	ypj:CH55_3717	K08961	K08961	chondroitin-sulfate-ABC endolyase/exolyase	4.2.2.20 4.2.2.21	--

[denovogenes]_102238	84.76	8e-175	pdf:CD630DERM_35400	K01874	MARS, metG	methionyl-tRNA synthetase	6.1.1.10	Metabolism--Metabolism of other amino acids--Selenocompound metabolism|Genetic Information Processing--Translation--Aminoacyl-tRNA biosynthesis

[denovogenes]_102239	43.51	1e-32	dml:Dmul_20150	K07814	K07814	putative two-component system response regulator	--	--

[denovogenes]_10224	98.70	0.0	boa:Bovatus_04049	K02316	dnaG	DNA primase	2.7.7.-	Genetic Information Processing--Replication and repair--DNA replication

[denovogenes]_102240	68.80	3e-122	cay:CEA_G2722	K06158	ABCF3	ATP-binding cassette, subfamily F, member 3	--	--

[denovogenes]_102242	76.26	8e-144	bhl:Bache_2483	K00067	rfbD, rmlD	dTDP-4-dehydrorhamnose reductase	1.1.1.133	Metabolism--Biosynthesis of other secondary metabolites--Streptomycin biosynthesis|Metabolism--Metabolism of terpenoids and polyketides--Polyketide sugar unit biosynthesis|Metabolism--Global and overview maps--Biosynthesis of antibiotics

[denovogenes]_102244	85.87	7e-169	cle:Clole_0629	K01874	MARS, metG	methionyl-tRNA synthetase	6.1.1.10	Metabolism--Metabolism of other amino acids--Selenocompound metabolism|Genetic Information Processing--Translation--Aminoacyl-tRNA biosynthesis

[denovogenes]_102247	65.92	2e-129	afn:Acfer_1448	K09685	purR	purine operon repressor	--	--

[denovogenes]_102248	66.79	5e-130	pft:JBW_00609	K00677	lpxA	UDP-N-acetylglucosamine acyltransferase	2.3.1.129	Metabolism--Glycan biosynthesis and metabolism--Lipopolysaccharide biosynthesis|Metabolism--Global and overview maps--Metabolic pathways|Human Diseases--Drug resistance: Antimicrobial--Cationic antimicrobial peptide (CAMP) resistance

[denovogenes]_102249	89.88	1e-172	btho:Btheta7330_02631	K07001	K07001	NTE family protein	--	--

[denovogenes]_102250	85.34	1e-168	rto:RTO_24650	K14540	rbgA	ribosome biogenesis GTPase A	--	--

[denovogenes]_102251	62.69	3e-118	afn:Acfer_2046	K02006	cbiO	cobalt/nickel transport system ATP-binding protein	--	Environmental Information Processing--Membrane transport--ABC transporters

[denovogenes]_102252	58.21	1e-111	cct:CC1_24040	K06147	ABCB-BAC	ATP-binding cassette, subfamily B, bacterial	--	--

[denovogenes]_102254	94.16	5e-176	bhl:Bache_2700	K11071	potB	spermidine/putrescine transport system permease protein	--	Environmental Information Processing--Membrane transport--ABC transporters

[denovogenes]_10226	97.26	0.0	bth:BT_0105	K03169	topB	DNA topoisomerase III	5.99.1.2	--

[denovogenes]_102260	52.03	7e-38	pca:Pcar_1321	K08309	slt	soluble lytic murein transglycosylase	3.2.1.-	--

[denovogenes]_102261	54.61	1e-110	rto:RTO_17430	K04486	E3.1.3.15B	histidinol-phosphatase (PHP family)	3.1.3.15	Metabolism--Amino acid metabolism--Histidine metabolism|Metabolism--Global and overview maps--Metabolic pathways|Metabolism--Global and overview maps--Biosynthesis of secondary metabolites|Metabolism--Global and overview maps--Biosynthesis of amino acids

[denovogenes]_102263	70.33	2e-120	fpla:A4U99_05435	K03584	recO	DNA repair protein RecO (recombination protein O)	--	Genetic Information Processing--Replication and repair--Homologous recombination

[denovogenes]_102264	98.88	0.0	bxy:BXY_03430	K07001	K07001	NTE family protein	--	--

[denovogenes]_102268	95.52	0.0	boa:Bovatus_00851	K07001	K07001	NTE family protein	--	--

[denovogenes]_102269	78.16	5e-155	byl:A4V09_19315	K01567	pdaA	peptidoglycan-N-acetylmuramic acid deacetylase	3.5.1.-	--

[denovogenes]_102272	100.00	0.0	bvu:BVU_4104	K01448	E3.5.1.28B, amiA, amiB, amiC	N-acetylmuramoyl-L-alanine amidase	3.5.1.28	Human Diseases--Drug resistance: Antimicrobial--Cationic antimicrobial peptide (CAMP) resistance

[denovogenes]_102277	71.27	9e-140	bprs:CK3_22250	K00941	thiD	hydroxymethylpyrimidine/phosphomethylpyrimidine kinase	2.7.1.49 2.7.4.7	Metabolism--Metabolism of cofactors and vitamins--Thiamine metabolism|Metabolism--Global and overview maps--Metabolic pathways

[denovogenes]_102278	87.31	5e-179	cso:CLS_08360	K07699	spo0A	two-component system, response regulator, stage 0 sporulation protein A	--	Environmental Information Processing--Signal transduction--Two-component system|Cellular Processes--Cellular community - prokaryotes--Quorum sensing

[denovogenes]_10228	100.00	0.0	ecm:EcSMS35_3987	K03655	recG	ATP-dependent DNA helicase RecG	3.6.4.12	Genetic Information Processing--Replication and repair--Homologous recombination

[denovogenes]_102280	64.59	2e-106	fpla:A4U99_04135	K02190	cbiK	sirohydrochlorin cobaltochelatase	4.99.1.3	Metabolism--Metabolism of cofactors and vitamins--Porphyrin and chlorophyll metabolism|Metabolism--Global and overview maps--Metabolic pathways

[denovogenes]_102288	83.15	5e-161	fpla:A4U99_13765	K05833	ABC.X4.A	putative ABC transport system ATP-binding protein	--	--

[denovogenes]_102289	94.78	0.0	bxy:BXY_15910	K01991	ABC-2.OM, wza	polysaccharide biosynthesis/export protein	--	Cellular Processes--Cellular community - prokaryotes--Biofilm formation - Escherichia coli

[denovogenes]_10229	87.59	0.0	fhw:RN87_04040	K02355	fusA, GFM, EFG	elongation factor G	--	--

[denovogenes]_102290	50.76	3e-92	bcl:ABC1422	K01048	pldB	lysophospholipase	3.1.1.5	Metabolism--Lipid metabolism--Glycerophospholipid metabolism

[denovogenes]_102291	83.21	2e-165	rto:RTO_30250	K01265	map	methionyl aminopeptidase	3.4.11.18	--

[denovogenes]_102292	54.92	6e-108	clo:HMPREF0868_0012	K03337	iolB	5-deoxy-glucuronate isomerase	5.3.1.30	Metabolism--Carbohydrate metabolism--Inositol phosphate metabolism|Metabolism--Global and overview maps--Metabolic pathways|Metabolism--Global and overview maps--Microbial metabolism in diverse environments

[denovogenes]_102293	41.20	1e-55	puf:UFO1_4750	K17836	penP	beta-lactamase class A	3.5.2.6	Metabolism--Biosynthesis of other secondary metabolites--Penicillin and cephalosporin biosynthesis|Metabolism--Global and overview maps--Biosynthesis of antibiotics|Human Diseases--Drug resistance: Antimicrobial--beta-Lactam resistance

[denovogenes]_102294	42.95	6e-33	sag:SAG0555	K07741	antB	anti-repressor protein	--	--

[denovogenes]_102298	62.31	7e-55	rim:ROI_08810	K07720	yesN	two-component system, response regulator YesN	--	Environmental Information Processing--Signal transduction--Two-component system

[denovogenes]_1023	99.92	0.0	fpla:A4U99_17605	K03737	por, nifJ	pyruvate-ferredoxin/flavodoxin oxidoreductase	1.2.7.1 1.2.7.-	Metabolism--Carbohydrate metabolism--Glycolysis / Gluconeogenesis|Metabolism--Carbohydrate metabolism--Citrate cycle (TCA cycle)|Metabolism--Carbohydrate metabolism--Pyruvate metabolism|Metabolism--Carbohydrate metabolism--Butanoate metabolism|Metabolism--Energy metabolism--Carbon fixation pathways in prokaryotes|Metabolism--Global and overview maps--Metabolic pathways|Metabolism--Global and overview maps--Microbial metabolism in diverse environments|Metabolism--Global and overview maps--Biosynthesis of antibiotics|Metabolism--Global and overview maps--Carbon metabolism

[denovogenes]_102300	56.49	4e-106	cpy:Cphy_2914	K00286	proC	pyrroline-5-carboxylate reductase	1.5.1.2	Metabolism--Amino acid metabolism--Arginine and proline metabolism|Metabolism--Global and overview maps--Metabolic pathways|Metabolism--Global and overview maps--Biosynthesis of secondary metabolites|Metabolism--Global and overview maps--Biosynthesis of antibiotics|Metabolism--Global and overview maps--Biosynthesis of amino acids

[denovogenes]_102301	45.89	6e-55	sbu:SpiBuddy_2152	K11175	purN	phosphoribosylglycinamide formyltransferase 1	2.1.2.2	Metabolism--Nucleotide metabolism--Purine metabolism|Metabolism--Metabolism of cofactors and vitamins--One carbon pool by folate|Metabolism--Global and overview maps--Metabolic pathways|Metabolism--Global and overview maps--Biosynthesis of secondary metabolites|Metabolism--Global and overview maps--Biosynthesis of antibiotics

[denovogenes]_102303	62.45	5e-124	cpy:Cphy_1451	K02042	phnE	phosphonate transport system permease protein	--	Environmental Information Processing--Membrane transport--ABC transporters

[denovogenes]_102304	45.61	6e-70	cso:CLS_31800	K01104	E3.1.3.48	protein-tyrosine phosphatase	3.1.3.48	--

[denovogenes]_102305	88.10	6e-174	bdh:GV66_10545	K07137	K07137	uncharacterized protein	--	--

[denovogenes]_102307	74.07	5e-143	fpla:A4U99_08295	K16785	ecfT	energy-coupling factor transport system permease protein	--	Environmental Information Processing--Membrane transport--ABC transporters

[denovogenes]_102309	65.27	3e-121	ruk:A4V00_14925	K02410	fliG	flagellar motor switch protein FliG	--	Cellular Processes--Cell motility--Bacterial chemotaxis|Cellular Processes--Cell motility--Flagellar assembly

[denovogenes]_102312	71.64	1e-142	ibu:IB211_00427	K16203	dppA1	D-amino peptidase	3.4.11.-	--

[denovogenes]_102313	84.31	2e-157	bprl:CL2_17520	K03149	thiG	thiazole synthase	2.8.1.10	Metabolism--Metabolism of cofactors and vitamins--Thiamine metabolism|Metabolism--Global and overview maps--Metabolic pathways

[denovogenes]_102314	86.30	8e-157	lacy:A4V08_33250	K01449	E3.5.1.28C, cwlJ, sleB	N-acetylmuramoyl-L-alanine amidase	3.5.1.28	--

[denovogenes]_102315	84.38	4e-166	cso:CLS_35580	K00978	rfbF	glucose-1-phosphate cytidylyltransferase	2.7.7.33	Metabolism--Carbohydrate metabolism--Starch and sucrose metabolism|Metabolism--Carbohydrate metabolism--Amino sugar and nucleotide sugar metabolism|Metabolism--Global and overview maps--Metabolic pathways

[denovogenes]_102316	59.30	2e-112	brm:Bmur_2299	K11070	potC	spermidine/putrescine transport system permease protein	--	Environmental Information Processing--Membrane transport--ABC transporters

[denovogenes]_102319	50.55	2e-85	rus:RBI_I00980	K03798	ftsH, hflB	cell division protease FtsH	3.4.24.-	--

[denovogenes]_102320	57.92	3e-103	cbt:CLH_3076	K06183	rsuA	16S rRNA pseudouridine516 synthase	5.4.99.19	--

[denovogenes]_102323	66.79	2e-135	bprs:CK3_24230	K07045	K07045	uncharacterized protein	--	--

[denovogenes]_102324	67.16	4e-130	lacy:A4V08_01935	K11749	rseP	regulator of sigma E protease	3.4.24.-	Cellular Processes--Cellular community - prokaryotes--Quorum sensing|Cellular Processes--Cell growth and death--Cell cycle - Caulobacter

[denovogenes]_102326	47.13	6e-77	nth:Nther_1461	K01358	clpP, CLPP	ATP-dependent Clp protease, protease subunit	3.4.21.92	Cellular Processes--Cell growth and death--Cell cycle - Caulobacter|Organismal Systems--Aging--Longevity regulating pathway - worm

[denovogenes]_102327	100.00	0.0	esl:O3K_14045	K01695	trpA	tryptophan synthase alpha chain	4.2.1.20	Metabolism--Amino acid metabolism--Glycine, serine and threonine metabolism|Metabolism--Amino acid metabolism--Phenylalanine, tyrosine and tryptophan biosynthesis|Metabolism--Global and overview maps--Metabolic pathways|Metabolism--Global and overview maps--Biosynthesis of secondary metabolites|Metabolism--Global and overview maps--Biosynthesis of antibiotics|Metabolism--Global and overview maps--Biosynthesis of amino acids

[denovogenes]_102329	68.09	2e-123	hsd:SD1D_1262	K00941	thiD	hydroxymethylpyrimidine/phosphomethylpyrimidine kinase	2.7.1.49 2.7.4.7	Metabolism--Metabolism of cofactors and vitamins--Thiamine metabolism|Metabolism--Global and overview maps--Metabolic pathways

[denovogenes]_10233	61.23	0.0	cbv:U729_37	K07037	K07037	uncharacterized protein	--	--

[denovogenes]_102332	62.95	9e-112	tae:TepiRe1_2385	K01803	TPI, tpiA	triosephosphate isomerase (TIM)	5.3.1.1	Metabolism--Carbohydrate metabolism--Glycolysis / Gluconeogenesis|Metabolism--Carbohydrate metabolism--Fructose and mannose metabolism|Metabolism--Carbohydrate metabolism--Inositol phosphate metabolism|Metabolism--Energy metabolism--Carbon fixation in photosynthetic organisms|Metabolism--Global and overview maps--Metabolic pathways|Metabolism--Global and overview maps--Biosynthesis of secondary metabolites|Metabolism--Global and overview maps--Microbial metabolism in diverse environments|Metabolism--Global and overview maps--Biosynthesis of antibiotics|Metabolism--Global and overview maps--Carbon metabolism|Metabolism--Global and overview maps--Biosynthesis of amino acids

[denovogenes]_102334	41.72	2e-24	nth:Nther_2544	K01447	E3.5.1.28A, cwlA, xlyA, xlyB	N-acetylmuramoyl-L-alanine amidase	3.5.1.28	--

[denovogenes]_102335	46.31	3e-75	ahh:RY45_09040	K02444	glpR	DeoR family transcriptional regulator, glycerol-3-phosphate regulon repressor	--	--

[denovogenes]_102336	69.65	2e-127	rho:RHOM_15650	K03496	parA, soj	chromosome partitioning protein	--	--

[denovogenes]_10234	73.37	0.0	efc:EFAU004_01988	K01879	glyS	glycyl-tRNA synthetase beta chain	6.1.1.14	Genetic Information Processing--Translation--Aminoacyl-tRNA biosynthesis

[denovogenes]_102342	92.54	0.0	csh:Closa_2471	K07335	bmpA, bmpB, tmpC	basic membrane protein A and related proteins	--	--

[denovogenes]_102343	86.59	6e-155	lacy:A4V08_25920	K12410	npdA	NAD-dependent deacetylase	3.5.1.-	--

[denovogenes]_102344	59.15	8e-115	hsd:SD1D_0811	K13292	lgt, umpA	phosphatidylglycerol:prolipoprotein diacylglycerol transferase	2.-.-.-	--

[denovogenes]_102345	99.63	0.0	ecoa:APECO78_06635	K01169	rna	ribonuclease I (enterobacter ribonuclease)	3.1.27.6	--

[denovogenes]_102348	78.36	7e-147	fpr:FP2_26440	K05833	ABC.X4.A	putative ABC transport system ATP-binding protein	--	--

[denovogenes]_102349	43.06	1e-47	rlt:Rleg2_3341	K02483	K02483	two-component system, OmpR family, response regulator	--	--

[denovogenes]_10235	90.32	0.0	efq:DR75_649	K03168	topA	DNA topoisomerase I	5.99.1.2	--

[denovogenes]_102352	42.01	7e-54	fnu:FN0590	K01436	K01436	amidohydrolase	3.5.1.-	--

[denovogenes]_102354	99.25	0.0	cif:AL515_05255	K01924	murC	UDP-N-acetylmuramate--alanine ligase	6.3.2.8	Metabolism--Metabolism of other amino acids--D-Glutamine and D-glutamate metabolism|Metabolism--Glycan biosynthesis and metabolism--Peptidoglycan biosynthesis|Metabolism--Global and overview maps--Metabolic pathways

[denovogenes]_102356	98.88	0.0	fpla:A4U99_13765	K05833	ABC.X4.A	putative ABC transport system ATP-binding protein	--	--

[denovogenes]_102357	74.44	6e-148	blf:BLIF_1539	K01250	rihA	pyrimidine-specific ribonucleoside hydrolase	3.2.-.-	--

[denovogenes]_10236	72.80	0.0	cbv:U729_602	K03798	ftsH, hflB	cell division protease FtsH	3.4.24.-	--

[denovogenes]_102360	100.00	0.0	ecol:LY180_08825	K19709	ydiF	acetate CoA-transferase	2.8.3.8	Metabolism--Xenobiotics biodegradation and metabolism--Aminobenzoate degradation|Metabolism--Carbohydrate metabolism--Propanoate metabolism|Metabolism--Carbohydrate metabolism--Butanoate metabolism|Metabolism--Global and overview maps--Metabolic pathways|Metabolism--Global and overview maps--Microbial metabolism in diverse environments

[denovogenes]_102362	54.24	2e-102	hsd:SD1D_1769	K04486	E3.1.3.15B	histidinol-phosphatase (PHP family)	3.1.3.15	Metabolism--Amino acid metabolism--Histidine metabolism|Metabolism--Global and overview maps--Metabolic pathways|Metabolism--Global and overview maps--Biosynthesis of secondary metabolites|Metabolism--Global and overview maps--Biosynthesis of amino acids

[denovogenes]_102363	92.57	0.0	bdh:GV66_15370	K18929	lldF	L-lactate dehydrogenase complex protein LldF	--	--

[denovogenes]_102364	41.67	7e-46	rpe:RPE_0606	K04114	bcrA, badF	benzoyl-CoA reductase subunit A	1.3.7.8	Metabolism--Xenobiotics biodegradation and metabolism--Benzoate degradation|Metabolism--Global and overview maps--Metabolic pathways|Metabolism--Global and overview maps--Microbial metabolism in diverse environments|Metabolism--Global and overview maps--Degradation of aromatic compounds

[denovogenes]_102367	84.76	4e-171	bcel:BcellWH2_00367	K02013	ABC.FEV.A	iron complex transport system ATP-binding protein	3.6.3.34	Environmental Information Processing--Membrane transport--ABC transporters

[denovogenes]_102368	100.00	0.0	cfd:CFNIH1_10320	K02526	kdgT	2-keto-3-deoxygluconate permease	--	--

[denovogenes]_10237	100.00	0.0	stb:SGPB_1824	K02355	fusA, GFM, EFG	elongation factor G	--	--

[denovogenes]_102370	42.24	4e-76	gfe:Gferi_24765	K06987	K06987	uncharacterized protein	--	--

[denovogenes]_102371	67.18	1e-130	eha:Ethha_2656	K03856	AROA2, aroA	3-deoxy-7-phosphoheptulonate synthase	2.5.1.54	Metabolism--Amino acid metabolism--Phenylalanine, tyrosine and tryptophan biosynthesis|Metabolism--Global and overview maps--Metabolic pathways|Metabolism--Global and overview maps--Biosynthesis of secondary metabolites|Metabolism--Global and overview maps--Biosynthesis of antibiotics|Metabolism--Global and overview maps--Biosynthesis of amino acids

[denovogenes]_102372	99.26	0.0	eoc:CE10_0808	K07027	K07027	glycosyltransferase 2 family protein	--	--

[denovogenes]_102375	41.44	4e-60	esr:ES1_11960	K03437	spoU	RNA methyltransferase, TrmH family	--	--

[denovogenes]_102379	67.91	1e-137	dai:Desaci_0452	K01620	ltaE	threonine aldolase	4.1.2.48	Metabolism--Amino acid metabolism--Glycine, serine and threonine metabolism|Metabolism--Global and overview maps--Metabolic pathways|Metabolism--Global and overview maps--Biosynthesis of secondary metabolites|Metabolism--Global and overview maps--Microbial metabolism in diverse environments|Metabolism--Global and overview maps--Biosynthesis of antibiotics|Metabolism--Global and overview maps--Biosynthesis of amino acids

[denovogenes]_10238	100.00	0.0	efq:DR75_649	K03168	topA	DNA topoisomerase I	5.99.1.2	--

[denovogenes]_102380	95.93	6e-176	ent:Ent638_1149	K00433	cpo	non-heme chloroperoxidase	1.11.1.10	--

[denovogenes]_102382	90.67	0.0	bvu:BVU_2901	K04041	fbp3	fructose-1,6-bisphosphatase III	3.1.3.11	Metabolism--Carbohydrate metabolism--Glycolysis / Gluconeogenesis|Metabolism--Carbohydrate metabolism--Pentose phosphate pathway|Metabolism--Carbohydrate metabolism--Fructose and mannose metabolism|Metabolism--Energy metabolism--Methane metabolism|Metabolism--Energy metabolism--Carbon fixation in photosynthetic organisms|Metabolism--Global and overview maps--Metabolic pathways|Metabolism--Global and overview maps--Biosynthesis of secondary metabolites|Metabolism--Global and overview maps--Microbial metabolism in diverse environments|Metabolism--Global and overview maps--Biosynthesis of antibiotics|Metabolism--Global and overview maps--Carbon metabolism

[denovogenes]_102385	49.43	2e-93	bcf:bcf_25570	K12573	rnr, vacB	ribonuclease R	3.1.-.-	Genetic Information Processing--Folding, sorting and degradation--RNA degradation

[denovogenes]_102387	67.82	3e-124	byl:A4V09_00280	K01546	kdpA	K+-transporting ATPase ATPase A chain	3.6.3.12	Environmental Information Processing--Signal transduction--Two-component system

[denovogenes]_102389	66.67	6e-108	cno:NT01CX_2252	K06901	pbuG	putative MFS transporter, AGZA family, xanthine/uracil permease	--	--

[denovogenes]_10239	44.52	0.0	ral:Rumal_2244	K02004	ABC.CD.P	putative ABC transport system permease protein	--	--

[denovogenes]_102390	99.63	0.0	bcel:BcellWH2_05194	K02118	ATPVB, ntpB, atpB	V/A-type H+/Na+-transporting ATPase subunit B	--	Metabolism--Energy metabolism--Oxidative phosphorylation|Metabolism--Global and overview maps--Metabolic pathways

[denovogenes]_102392	100.00	0.0	pdi:BDI_0634	K18785	mp2	beta-1,4-mannooligosaccharide/beta-1,4-mannosyl-N-acetylglucosamine phosphorylase	2.4.1.319 2.4.1.320	--

[denovogenes]_102394	100.00	0.0	pdi:BDI_3533	K02600	nusA	N utilization substance protein A	--	--

[denovogenes]_102395	99.63	0.0	cfd:CFNIH1_24445	K03106	SRP54, ffh	signal recognition particle subunit SRP54	3.6.5.4	Cellular Processes--Cellular community - prokaryotes--Quorum sensing|Genetic Information Processing--Folding, sorting and degradation--Protein export|Environmental Information Processing--Membrane transport--Bacterial secretion system

[denovogenes]_102399	81.48	3e-159	csq:CSCA_1915	K03205	virD4, lvhD4	type IV secretion system protein VirD4	--	Environmental Information Processing--Membrane transport--Bacterial secretion system

[denovogenes]_10240	81.94	0.0	cbv:U729_2787	K02519	infB, MTIF2	translation initiation factor IF-2	--	--

[denovogenes]_102402	57.89	4e-109	dhd:Dhaf_2831	K00986	E2.7.7.49	RNA-directed DNA polymerase	2.7.7.49	--

[denovogenes]_102403	44.89	5e-70	ele:Elen_0991	K01451	hipO	hippurate hydrolase	3.5.1.32	Metabolism--Amino acid metabolism--Phenylalanine metabolism

[denovogenes]_102405	42.26	2e-45	cuv:CUREI_07155	K01906	bioW	6-carboxyhexanoate--CoA ligase	6.2.1.14	Metabolism--Metabolism of cofactors and vitamins--Biotin metabolism|Metabolism--Global and overview maps--Metabolic pathways

[denovogenes]_102407	92.40	9e-168	pfr:PFREUD_14840	K02013	ABC.FEV.A	iron complex transport system ATP-binding protein	3.6.3.34	Environmental Information Processing--Membrane transport--ABC transporters

[denovogenes]_102408	100.00	0.0	btho:Btheta7330_02397	K07001	K07001	NTE family protein	--	--

[denovogenes]_102409	72.80	2e-146	bsa:Bacsa_0489	K00571	E2.1.1.72	site-specific DNA-methyltransferase (adenine-specific)	2.1.1.72	--

[denovogenes]_102411	44.16	4e-66	pcc:PCC21_013470	K13002	wbyL	glycosyltransferase	2.4.1.-	--

[denovogenes]_102412	91.42	0.0	pfre:RM25_0727	K02428	rdgB	XTP/dITP diphosphohydrolase	3.6.1.66	Metabolism--Nucleotide metabolism--Purine metabolism

[denovogenes]_102413	83.58	2e-163	pdi:BDI_2250	K00286	proC	pyrroline-5-carboxylate reductase	1.5.1.2	Metabolism--Amino acid metabolism--Arginine and proline metabolism|Metabolism--Global and overview maps--Metabolic pathways|Metabolism--Global and overview maps--Biosynthesis of secondary metabolites|Metabolism--Global and overview maps--Biosynthesis of antibiotics|Metabolism--Global and overview maps--Biosynthesis of amino acids

[denovogenes]_102417	41.25	6e-70	bxy:BXY_28940	K03734	apbE	FAD:protein FMN transferase	2.7.1.180	--

[denovogenes]_10242	45.69	0.0	rbr:RBR_01000	K03169	topB	DNA topoisomerase III	5.99.1.2	--

[denovogenes]_102420	65.80	4e-126	bhl:Bache_1104	K03561	exbB	biopolymer transport protein ExbB	--	--

[denovogenes]_102421	48.48	8e-73	lir:LAW_00400	K00981	E2.7.7.41, CDS1, CDS2, cdsA	phosphatidate cytidylyltransferase	2.7.7.41	Metabolism--Lipid metabolism--Glycerophospholipid metabolism|Metabolism--Global and overview maps--Metabolic pathways|Metabolism--Global and overview maps--Biosynthesis of secondary metabolites|Environmental Information Processing--Signal transduction--Phosphatidylinositol signaling system

[denovogenes]_102422	74.72	8e-148	pdi:BDI_0528	K03814	mtgA	monofunctional biosynthetic peptidoglycan transglycosylase	2.4.1.-	Metabolism--Glycan biosynthesis and metabolism--Peptidoglycan biosynthesis

[denovogenes]_102423	69.48	3e-122	elm:ELI_3913	K02007	cbiM	cobalt/nickel transport system permease protein	--	Environmental Information Processing--Membrane transport--ABC transporters

[denovogenes]_102424	99.25	0.0	paeo:M801_5047	K02848	waaP, rfaP	heptose I phosphotransferase	2.7.1.-	Metabolism--Glycan biosynthesis and metabolism--Lipopolysaccharide biosynthesis|Metabolism--Global and overview maps--Metabolic pathways

[denovogenes]_102425	100.00	0.0	paeo:M801_218	K13933	mdcE	malonate decarboxylase gamma subunit	4.1.1.87	--

[denovogenes]_102426	100.00	0.0	stb:SGPB_0832	K02823	pyrDII	dihydroorotate dehydrogenase electron transfer subunit	--	Metabolism--Nucleotide metabolism--Pyrimidine metabolism|Metabolism--Global and overview maps--Metabolic pathways

[denovogenes]_102427	94.49	5e-175	eclx:LI66_22885	K12204	dotC, traI	defect in organelle trafficking protein DotC	--	--

[denovogenes]_102429	100.00	0.0	cfd:CFNIH1_18775	K19230	sapF	cationic peptide transport system ATP-binding protein	--	Human Diseases--Drug resistance: Antimicrobial--Cationic antimicrobial peptide (CAMP) resistance|Environmental Information Processing--Membrane transport--ABC transporters

[denovogenes]_10243	57.40	0.0	cbz:Cbs_4782	K03657	uvrD, pcrA	DNA helicase II / ATP-dependent DNA helicase PcrA	3.6.4.12	Genetic Information Processing--Replication and repair--Nucleotide excision repair|Genetic Information Processing--Replication and repair--Mismatch repair

[denovogenes]_102430	47.49	2e-85	kol:Kole_1453	K03593	mrp, NUBPL	ATP-binding protein involved in chromosome partitioning	--	--

[denovogenes]_102434	41.26	2e-74	ipo:Ilyop_2610	K15342	cas1	CRISP-associated protein Cas1	--	--

[denovogenes]_102435	100.00	0.0	paeg:AI22_00905	K02528	ksgA	16S rRNA (adenine1518-N6/adenine1519-N6)-dimethyltransferase	2.1.1.182	--

[denovogenes]_102437	53.56	6e-97	amt:Amet_4719	K15587	nikD	nickel transport system ATP-binding protein	3.6.3.24	Environmental Information Processing--Membrane transport--ABC transporters

[denovogenes]_102438	100.00	0.0	shq:A0259_03175	K06971	K06971	uncharacterized protein	--	--

[denovogenes]_102439	99.63	0.0	paeo:M801_1140	K02411	fliH	flagellar assembly protein FliH	--	Cellular Processes--Cell motility--Flagellar assembly

[denovogenes]_10244	76.51	0.0	rob:CK5_10000	K04079	HSP90A, htpG	molecular chaperone HtpG	--	Genetic Information Processing--Folding, sorting and degradation--Protein processing in endoplasmic reticulum|Environmental Information Processing--Signal transduction--PI3K-Akt signaling pathway|Organismal Systems--Immune system--Antigen processing and presentation|Organismal Systems--Immune system--NOD-like receptor signaling pathway|Organismal Systems--Environmental adaptation--Plant-pathogen interaction|Organismal Systems--Immune system--IL-17 signaling pathway|Organismal Systems--Immune system--Th17 cell differentiation|Organismal Systems--Endocrine system--Progesterone-mediated oocyte maturation|Organismal Systems--Endocrine system--Estrogen signaling pathway|Human Diseases--Cancers: Overview--Pathways in cancer|Human Diseases--Cancers: Specific types--Prostate cancer|Human Diseases--Cardiovascular diseases--Fluid shear stress and atherosclerosis

[denovogenes]_102440	41.11	5e-48	oho:Oweho_0986	K19294	algI	alginate O-acetyltransferase complex protein AlgI	--	--

[denovogenes]_102441	42.91	2e-50	mpq:ABA45_04145	K07090	K07090	uncharacterized protein	--	--

[denovogenes]_102443	100.00	0.0	paeo:M801_3463	K01713	pheC	cyclohexadienyl dehydratase	4.2.1.51 4.2.1.91	Metabolism--Amino acid metabolism--Phenylalanine, tyrosine and tryptophan biosynthesis|Metabolism--Global and overview maps--Metabolic pathways|Metabolism--Global and overview maps--Biosynthesis of secondary metabolites|Metabolism--Global and overview maps--Biosynthesis of antibiotics|Metabolism--Global and overview maps--Biosynthesis of amino acids

[denovogenes]_102444	68.08	2e-122	fpr:FP2_20670	K00931	proB	glutamate 5-kinase	2.7.2.11	Metabolism--Amino acid metabolism--Arginine and proline metabolism|Metabolism--Biosynthesis of other secondary metabolites--Carbapenem biosynthesis|Metabolism--Global and overview maps--Metabolic pathways|Metabolism--Global and overview maps--Biosynthesis of antibiotics|Metabolism--Global and overview maps--Biosynthesis of amino acids

[denovogenes]_102445	42.86	2e-31	gfe:Gferi_12180	K06196	ccdA	cytochrome c-type biogenesis protein	--	--

[denovogenes]_102446	100.00	0.0	paes:SCV20265_3789	K11209	yghU, yfcG	GSH-dependent disulfide-bond oxidoreductase	1.8.4.-	--

[denovogenes]_102447	93.28	0.0	bfs:BF9343_4079	K01092	E3.1.3.25, IMPA, suhB	myo-inositol-1(or 4)-monophosphatase	3.1.3.25	Metabolism--Biosynthesis of other secondary metabolites--Streptomycin biosynthesis|Metabolism--Carbohydrate metabolism--Inositol phosphate metabolism|Metabolism--Global and overview maps--Metabolic pathways|Environmental Information Processing--Signal transduction--Phosphatidylinositol signaling system

[denovogenes]_102448	71.59	5e-138	dvm:DvMF_2610	K01995	livG	branched-chain amino acid transport system ATP-binding protein	--	Environmental Information Processing--Membrane transport--ABC transporters|Cellular Processes--Cellular community - prokaryotes--Quorum sensing

[denovogenes]_102449	98.88	0.0	cfd:CFNIH1_18935	K01695	trpA	tryptophan synthase alpha chain	4.2.1.20	Metabolism--Amino acid metabolism--Glycine, serine and threonine metabolism|Metabolism--Amino acid metabolism--Phenylalanine, tyrosine and tryptophan biosynthesis|Metabolism--Global and overview maps--Metabolic pathways|Metabolism--Global and overview maps--Biosynthesis of secondary metabolites|Metabolism--Global and overview maps--Biosynthesis of antibiotics|Metabolism--Global and overview maps--Biosynthesis of amino acids

[denovogenes]_10245	97.40	0.0	boa:Bovatus_04049	K02316	dnaG	DNA primase	2.7.7.-	Genetic Information Processing--Replication and repair--DNA replication

[denovogenes]_102450	100.00	3e-165	cfd:CFNIH1_18975	K03832	tonB	periplasmic protein TonB	--	--

[denovogenes]_102451	48.28	2e-83	kol:Kole_1453	K03593	mrp, NUBPL	ATP-binding protein involved in chromosome partitioning	--	--

[denovogenes]_102454	40.80	3e-24	sagr:SAIL_14770	K07271	licD	lipopolysaccharide cholinephosphotransferase	2.7.8.-	--

[denovogenes]_102455	99.25	0.0	lacy:A4V08_08690	K00046	idnO	gluconate 5-dehydrogenase	1.1.1.69	--

[denovogenes]_102456	100.00	0.0	paeo:M801_2993	K07140	K07140	uncharacterized protein	--	--

[denovogenes]_102457	47.19	4e-89	sfc:Spiaf_1392	K02026	ABC.MS.P1	multiple sugar transport system permease protein	--	--

[denovogenes]_102458	93.98	0.0	cso:CLS_16260	K01040	gctB	glutaconate CoA-transferase, subunit B	2.8.3.12	Metabolism--Xenobiotics biodegradation and metabolism--Styrene degradation|Metabolism--Carbohydrate metabolism--Butanoate metabolism|Metabolism--Global and overview maps--Microbial metabolism in diverse environments

[denovogenes]_102459	80.87	7e-112	bprs:CK3_33660	K07793	tctA	putative tricarboxylic transport membrane protein	--	Environmental Information Processing--Signal transduction--Two-component system

[denovogenes]_102460	100.00	0.0	paeo:M801_3809	K01092	E3.1.3.25, IMPA, suhB	myo-inositol-1(or 4)-monophosphatase	3.1.3.25	Metabolism--Biosynthesis of other secondary metabolites--Streptomycin biosynthesis|Metabolism--Carbohydrate metabolism--Inositol phosphate metabolism|Metabolism--Global and overview maps--Metabolic pathways|Environmental Information Processing--Signal transduction--Phosphatidylinositol signaling system

[denovogenes]_102461	41.95	1e-40	pmf:P9303_05381	K00975	glgC	glucose-1-phosphate adenylyltransferase	2.7.7.27	Metabolism--Carbohydrate metabolism--Starch and sucrose metabolism|Metabolism--Carbohydrate metabolism--Amino sugar and nucleotide sugar metabolism|Metabolism--Global and overview maps--Metabolic pathways|Metabolism--Global and overview maps--Biosynthesis of secondary metabolites|Cellular Processes--Cellular community - prokaryotes--Biofilm formation - Escherichia coli

[denovogenes]_102462	100.00	0.0	paeo:M801_2276	K02030	ABC.PA.S	polar amino acid transport system substrate-binding protein	--	--

[denovogenes]_102463	64.35	1e-99	fpa:FPR_17520	K01814	hisA	phosphoribosylformimino-5-aminoimidazole carboxamide ribotide isomerase	5.3.1.16	Metabolism--Amino acid metabolism--Histidine metabolism|Metabolism--Global and overview maps--Metabolic pathways|Metabolism--Global and overview maps--Biosynthesis of secondary metabolites|Metabolism--Global and overview maps--Biosynthesis of amino acids

[denovogenes]_102465	92.16	0.0	bfb:VU15_17975	K06897	K06897	7,8-dihydropterin-6-yl-methyl-4-(beta-D-ribofuranosyl)aminobenzene 5'-phosphate synthase	2.5.1.105	--

[denovogenes]_102466	64.92	2e-118	dvm:DvMF_2685	K02030	ABC.PA.S	polar amino acid transport system substrate-binding protein	--	--

[denovogenes]_102467	100.00	0.0	paeu:BN889_00069	K01695	trpA	tryptophan synthase alpha chain	4.2.1.20	Metabolism--Amino acid metabolism--Glycine, serine and threonine metabolism|Metabolism--Amino acid metabolism--Phenylalanine, tyrosine and tryptophan biosynthesis|Metabolism--Global and overview maps--Metabolic pathways|Metabolism--Global and overview maps--Biosynthesis of secondary metabolites|Metabolism--Global and overview maps--Biosynthesis of antibiotics|Metabolism--Global and overview maps--Biosynthesis of amino acids

[denovogenes]_102468	100.00	0.0	paeo:M801_4126	K02510	hpaI, hpcH	4-hydroxy-2-oxoheptanedioate aldolase	4.1.2.52	Metabolism--Amino acid metabolism--Tyrosine metabolism|Metabolism--Global and overview maps--Microbial metabolism in diverse environments

[denovogenes]_10247	98.99	0.0	bxy:BXY_41940	K04084	dsbD	thiol:disulfide interchange protein DsbD	1.8.1.8	--

[denovogenes]_102470	99.25	0.0	cfd:CFNIH1_13225	K01169	rna	ribonuclease I (enterobacter ribonuclease)	3.1.27.6	--

[denovogenes]_102471	94.03	0.0	ent:Ent638_1149	K00433	cpo	non-heme chloroperoxidase	1.11.1.10	--

[denovogenes]_102472	89.68	5e-169	lacy:A4V08_33155	K03785	aroD	3-dehydroquinate dehydratase I	4.2.1.10	Metabolism--Amino acid metabolism--Phenylalanine, tyrosine and tryptophan biosynthesis|Metabolism--Global and overview maps--Metabolic pathways|Metabolism--Global and overview maps--Biosynthesis of secondary metabolites|Metabolism--Global and overview maps--Biosynthesis of antibiotics|Metabolism--Global and overview maps--Biosynthesis of amino acids

[denovogenes]_102473	98.88	0.0	bfb:VU15_09750	K07001	K07001	NTE family protein	--	--

[denovogenes]_102477	100.00	0.0	paeo:M801_916	K10022	aotJ	arginine/ornithine transport system substrate-binding protein	--	Environmental Information Processing--Membrane transport--ABC transporters

[denovogenes]_102478	99.25	0.0	fpla:A4U99_07595	K13292	lgt, umpA	phosphatidylglycerol:prolipoprotein diacylglycerol transferase	2.-.-.-	--

[denovogenes]_102479	42.47	5e-59	pnp:IJ22_49400	K02025	ABC.MS.P	multiple sugar transport system permease protein	--	--

[denovogenes]_10248	79.16	0.0	fpla:A4U99_03380	K02355	fusA, GFM, EFG	elongation factor G	--	--

[denovogenes]_102480	46.09	2e-65	amu:Amuc_1747	K00968	PCYT1	choline-phosphate cytidylyltransferase	2.7.7.15	Metabolism--Metabolism of other amino acids--Phosphonate and phosphinate metabolism|Metabolism--Lipid metabolism--Glycerophospholipid metabolism|Metabolism--Global and overview maps--Metabolic pathways|Human Diseases--Cancers: Overview--Choline metabolism in cancer

[denovogenes]_102481	93.28	0.0	lacy:A4V08_34990	K00278	nadB	L-aspartate oxidase	1.4.3.16	Metabolism--Amino acid metabolism--Alanine, aspartate and glutamate metabolism|Metabolism--Metabolism of cofactors and vitamins--Nicotinate and nicotinamide metabolism|Metabolism--Global and overview maps--Metabolic pathways

[denovogenes]_102482	79.10	3e-147	csh:Closa_3091	K02030	ABC.PA.S	polar amino acid transport system substrate-binding protein	--	--

[denovogenes]_102483	40.29	8e-14	pox:MB84_07075	K21029	moeB	molybdopterin-synthase adenylyltransferase	2.7.7.80	Genetic Information Processing--Folding, sorting and degradation--Sulfur relay system

[denovogenes]_102486	56.33	3e-93	cgo:Corgl_0207	K16785	ecfT	energy-coupling factor transport system permease protein	--	Environmental Information Processing--Membrane transport--ABC transporters

[denovogenes]_102487	78.73	2e-140	gpa:GPA_13180	K06147	ABCB-BAC	ATP-binding cassette, subfamily B, bacterial	--	--

[denovogenes]_102488	95.91	6e-175	lacy:A4V08_26450	K02653	pilC	type IV pilus assembly protein PilC	--	--

[denovogenes]_10249	100.00	0.0	bfb:VU15_10355	K02014	TC.FEV.OM	iron complex outermembrane recepter protein	--	--

[denovogenes]_102492	78.07	4e-148	pmuc:ING2E5A_1537	K01890	FARSB, pheT	phenylalanyl-tRNA synthetase beta chain	6.1.1.20	Genetic Information Processing--Translation--Aminoacyl-tRNA biosynthesis

[denovogenes]_102494	98.51	0.0	cif:AL515_02805	K00068	srlD	sorbitol-6-phosphate 2-dehydrogenase	1.1.1.140	Metabolism--Carbohydrate metabolism--Fructose and mannose metabolism

[denovogenes]_102498	98.13	0.0	pdi:BDI_2122	K03650	mnmE, trmE, MSS1	tRNA modification GTPase	3.6.-.-	--

[denovogenes]_1025	99.59	0.0	paes:SCV20265_4851	K03546	sbcC	exonuclease SbcC	--	--

[denovogenes]_102500	99.63	0.0	bbrc:B7019_1412	K02520	infC, MTIF3	translation initiation factor IF-3	--	--

[denovogenes]_102501	100.00	0.0	pdf:CD630DERM_07270	K15023	acsE	5-methyltetrahydrofolate corrinoid/iron sulfur protein methyltransferase	2.1.1.258	Metabolism--Energy metabolism--Carbon fixation pathways in prokaryotes|Metabolism--Global and overview maps--Microbial metabolism in diverse environments|Metabolism--Global and overview maps--Carbon metabolism

[denovogenes]_102503	94.80	0.0	lacy:A4V08_34315	K17758	nnr	ADP-dependent NAD(P)H-hydrate dehydratase	4.2.1.136	--

[denovogenes]_102503	94.80	0.0	lacy:A4V08_34315	K17759	AIBP, nnrE	NAD(P)H-hydrate epimerase	5.1.99.6	--

[denovogenes]_102504	86.09	3e-166	spq:SPAB_03966	K07666	qseB	two-component system, OmpR family, response regulator QseB	--	Environmental Information Processing--Signal transduction--Two-component system|Cellular Processes--Cellular community - prokaryotes--Quorum sensing

[denovogenes]_102507	100.00	0.0	pau:PA14_01690	K02057	ABC.SS.P	simple sugar transport system permease protein	--	--

[denovogenes]_102508	100.00	0.0	ecoh:ECRM13516_0044	K03521	fixA, etfB	electron transfer flavoprotein beta subunit	--	--

[denovogenes]_102509	62.45	2e-113	ndk:I601_3615	K02470	gyrB	DNA gyrase subunit B	5.99.1.3	--

[denovogenes]_10251	67.31	0.0	cso:CLS_01000	K01187	malZ	alpha-glucosidase	3.2.1.20	Metabolism--Carbohydrate metabolism--Galactose metabolism|Metabolism--Carbohydrate metabolism--Starch and sucrose metabolism|Metabolism--Global and overview maps--Metabolic pathways

[denovogenes]_102512	67.54	3e-127	shi:Shel_19800	K01950	E6.3.5.1, NADSYN1, QNS1, nadE	NAD+ synthase (glutamine-hydrolysing)	6.3.5.1	Metabolism--Metabolism of cofactors and vitamins--Nicotinate and nicotinamide metabolism|Metabolism--Global and overview maps--Metabolic pathways

[denovogenes]_102513	67.95	6e-132	rix:RO1_33020	K03496	parA, soj	chromosome partitioning protein	--	--

[denovogenes]_102519	84.29	5e-169	cct:CC1_08860	K00571	E2.1.1.72	site-specific DNA-methyltransferase (adenine-specific)	2.1.1.72	--

[denovogenes]_102520	95.52	0.0	bcel:BcellWH2_00677	K01897	ACSL, fadD	long-chain acyl-CoA synthetase	6.2.1.3	Metabolism--Lipid metabolism--Fatty acid biosynthesis|Metabolism--Lipid metabolism--Fatty acid degradation|Metabolism--Global and overview maps--Metabolic pathways|Metabolism--Global and overview maps--Fatty acid metabolism|Cellular Processes--Cellular community - prokaryotes--Quorum sensing|Organismal Systems--Endocrine system--PPAR signaling pathway|Cellular Processes--Transport and catabolism--Peroxisome|Organismal Systems--Endocrine system--Adipocytokine signaling pathway

[denovogenes]_102522	85.13	2e-175	pdi:BDI_1675	K01130	E3.1.6.1, aslA	arylsulfatase	3.1.6.1	Metabolism--Lipid metabolism--Steroid hormone biosynthesis|Metabolism--Lipid metabolism--Sphingolipid metabolism

[denovogenes]_102524	49.42	1e-88	tpt:Tpet_1037	K02566	nagD	NagD protein	--	--

[denovogenes]_102527	44.94	2e-73	bbf:BBB_0697	K09692	tagG	teichoic acid transport system permease protein	--	Environmental Information Processing--Membrane transport--ABC transporters

[denovogenes]_102528	61.60	1e-103	gmc:GY4MC1_2177	K17215	K17215	inositol transport system ATP-binding protein	--	Environmental Information Processing--Membrane transport--ABC transporters

[denovogenes]_10253	47.54	0.0	ibu:IB211_00143	K07462	recJ	single-stranded-DNA-specific exonuclease	3.1.-.-	Genetic Information Processing--Replication and repair--Base excision repair|Genetic Information Processing--Replication and repair--Mismatch repair|Genetic Information Processing--Replication and repair--Homologous recombination

[denovogenes]_102531	99.26	0.0	cfd:CFNIH1_09920	K03522	fixB, etfA	electron transfer flavoprotein alpha subunit	--	--

[denovogenes]_102532	61.51	3e-98	ols:Olsu_1201	K15739	vanB, vanA, vanD	D-alanine---(R)-lactate ligase	6.1.2.1	Human Diseases--Drug resistance: Antimicrobial--Vancomycin resistance|Environmental Information Processing--Signal transduction--Two-component system

[denovogenes]_102534	82.46	8e-174	bth:BT_3982	K01144	E3.1.11.5	exodeoxyribonuclease V	3.1.11.5	--

[denovogenes]_102536	84.01	8e-169	pvl:AOB99_03325	K00077	panE, apbA	2-dehydropantoate 2-reductase	1.1.1.169	Metabolism--Metabolism of cofactors and vitamins--Pantothenate and CoA biosynthesis|Metabolism--Global and overview maps--Metabolic pathways|Metabolism--Global and overview maps--Biosynthesis of secondary metabolites

[denovogenes]_102537	99.25	0.0	ecm:EcSMS35_3764	K10824	nikE	nickel transport system ATP-binding protein	3.6.3.24	Environmental Information Processing--Membrane transport--ABC transporters

[denovogenes]_102538	92.91	0.0	pvl:AOB99_05070	K02564	nagB, GNPDA	glucosamine-6-phosphate deaminase	3.5.99.6	Metabolism--Carbohydrate metabolism--Amino sugar and nucleotide sugar metabolism|Metabolism--Global and overview maps--Metabolic pathways

[denovogenes]_102542	96.28	0.0	pvl:AOB99_00985	K03732	rhlB	ATP-dependent RNA helicase RhlB	3.6.4.13	Genetic Information Processing--Folding, sorting and degradation--RNA degradation

[denovogenes]_102544	77.57	3e-144	rto:RTO_20290	K03616	rnfB	electron transport complex protein RnfB	--	--

[denovogenes]_102547	99.63	0.0	cfd:CFNIH1_16600	K16370	pfkB	6-phosphofructokinase 2	2.7.1.11	Metabolism--Carbohydrate metabolism--Glycolysis / Gluconeogenesis|Metabolism--Carbohydrate metabolism--Pentose phosphate pathway|Metabolism--Carbohydrate metabolism--Fructose and mannose metabolism|Metabolism--Carbohydrate metabolism--Galactose metabolism|Metabolism--Energy metabolism--Methane metabolism|Metabolism--Global and overview maps--Metabolic pathways|Metabolism--Global and overview maps--Biosynthesis of secondary metabolites|Metabolism--Global and overview maps--Microbial metabolism in diverse environments|Metabolism--Global and overview maps--Biosynthesis of antibiotics|Metabolism--Global and overview maps--Carbon metabolism|Metabolism--Global and overview maps--Biosynthesis of amino acids

[denovogenes]_102548	100.00	0.0	pdf:CD630DERM_00790	K02982	RP-S3, rpsC	small subunit ribosomal protein S3	--	Genetic Information Processing--Translation--Ribosome

[denovogenes]_102549	96.27	0.0	sfs:SFyv_3749	K05708	hcaE, hcaA1	3-phenylpropionate/trans-cinnamate dioxygenase subunit alpha	1.14.12.19	Metabolism--Amino acid metabolism--Phenylalanine metabolism|Metabolism--Global and overview maps--Microbial metabolism in diverse environments|Metabolism--Global and overview maps--Degradation of aromatic compounds

[denovogenes]_10255	99.28	0.0	bth:BT_0457	K05970	SIAE	sialate O-acetylesterase	3.1.1.53	--

[denovogenes]_102550	82.13	8e-160	bfr:BF0603	K07137	K07137	uncharacterized protein	--	--

[denovogenes]_102551	99.25	0.0	cfd:CFNIH1_19725	K11105	cvrA, nhaP2	cell volume regulation protein A	--	--

[denovogenes]_102552	100.00	0.0	bbrc:B7019_0745	K00800	aroA	3-phosphoshikimate 1-carboxyvinyltransferase	2.5.1.19	Metabolism--Amino acid metabolism--Phenylalanine, tyrosine and tryptophan biosynthesis|Metabolism--Global and overview maps--Metabolic pathways|Metabolism--Global and overview maps--Biosynthesis of secondary metabolites|Metabolism--Global and overview maps--Biosynthesis of antibiotics|Metabolism--Global and overview maps--Biosynthesis of amino acids

[denovogenes]_102553	94.42	0.0	cfd:CFNIH1_00235	K03606	wcaJ	putative colanic acid biosysnthesis UDP-glucose lipid carrier transferase	--	Cellular Processes--Cellular community - prokaryotes--Biofilm formation - Vibrio cholerae

[denovogenes]_102554	92.16	0.0	rto:RTO_00440	K02314	dnaB	replicative DNA helicase	3.6.4.12	Genetic Information Processing--Replication and repair--DNA replication|Cellular Processes--Cell growth and death--Cell cycle - Caulobacter

[denovogenes]_102555	79.55	6e-155	csh:Closa_0501	K03310	TC.AGCS	alanine or glycine:cation symporter, AGCS family	--	--

[denovogenes]_102556	99.63	0.0	cfd:CFNIH1_03300	K07139	K07139	uncharacterized protein	--	--

[denovogenes]_102558	88.43	3e-172	boa:Bovatus_02371	K01740	metY	O-acetylhomoserine (thiol)-lyase	2.5.1.49	Metabolism--Amino acid metabolism--Cysteine and methionine metabolism|Metabolism--Global and overview maps--Metabolic pathways

[denovogenes]_10256	99.57	0.0	fpla:A4U99_03380	K02355	fusA, GFM, EFG	elongation factor G	--	--

[denovogenes]_102564	55.93	6e-94	ibu:IB211_01346	K02035	ABC.PE.S	peptide/nickel transport system substrate-binding protein	--	Cellular Processes--Cellular community - prokaryotes--Quorum sensing

[denovogenes]_102565	57.78	4e-116	bmh:BMWSH_1486	K00259	ald	alanine dehydrogenase	1.4.1.1	Metabolism--Amino acid metabolism--Alanine, aspartate and glutamate metabolism|Metabolism--Metabolism of other amino acids--Taurine and hypotaurine metabolism|Metabolism--Global and overview maps--Metabolic pathways

[denovogenes]_102566	42.64	9e-69	arh:AHiyo8_26050	K07497	K07497	putative transposase	--	--

[denovogenes]_102567	93.68	0.0	boa:Bovatus_02913	K01887	RARS, argS	arginyl-tRNA synthetase	6.1.1.19	Genetic Information Processing--Translation--Aminoacyl-tRNA biosynthesis

[denovogenes]_102573	72.01	1e-142	slq:M495_16800	K01685	uxaA	altronate hydrolase	4.2.1.7	Metabolism--Carbohydrate metabolism--Pentose and glucuronate interconversions|Metabolism--Global and overview maps--Metabolic pathways

[denovogenes]_102575	94.05	0.0	afd:Alfi_1489	K03495	gidA, mnmG, MTO1	tRNA uridine 5-carboxymethylaminomethyl modification enzyme	--	--

[denovogenes]_102577	98.88	0.0	elr:ECO55CA74_10395	K06867	K06867	uncharacterized protein	--	--

[denovogenes]_102579	85.50	1e-174	pdi:BDI_0578	K00764	purF, PPAT	amidophosphoribosyltransferase	2.4.2.14	Metabolism--Nucleotide metabolism--Purine metabolism|Metabolism--Amino acid metabolism--Alanine, aspartate and glutamate metabolism|Metabolism--Global and overview maps--Metabolic pathways|Metabolism--Global and overview maps--Biosynthesis of secondary metabolites|Metabolism--Global and overview maps--Biosynthesis of antibiotics

[denovogenes]_10258	99.86	0.0	ssj:SSON53_06635	K02400	flhA	flagellar biosynthesis protein FlhA	--	Cellular Processes--Cell motility--Flagellar assembly

[denovogenes]_102581	40.08	2e-57	ipo:Ilyop_0113	K20447	ndhL	nicotinate dehydrogenase large molybdopterin subunit	1.17.1.5	Metabolism--Metabolism of cofactors and vitamins--Nicotinate and nicotinamide metabolism|Metabolism--Global and overview maps--Microbial metabolism in diverse environments

[denovogenes]_102582	57.46	2e-107	shi:Shel_00020	K02338	DPO3B, dnaN	DNA polymerase III subunit beta	2.7.7.7	Metabolism--Nucleotide metabolism--Purine metabolism|Metabolism--Nucleotide metabolism--Pyrimidine metabolism|Metabolism--Global and overview maps--Metabolic pathways|Genetic Information Processing--Replication and repair--DNA replication|Genetic Information Processing--Replication and repair--Mismatch repair|Genetic Information Processing--Replication and repair--Homologous recombination

[denovogenes]_102586	98.88	0.0	pvl:AOB99_12490	K02747	PTS-Aga-EIID, agaE	PTS system, N-acetylgalactosamine-specific IID component	--	Metabolism--Carbohydrate metabolism--Galactose metabolism|Environmental Information Processing--Membrane transport--Phosphotransferase system (PTS)

[denovogenes]_102587	59.46	2e-106	shi:Shel_00520	K04654	hypD	hydrogenase expression/formation protein HypD	--	--

[denovogenes]_102588	86.35	3e-169	elp:P12B_c0137	K02014	TC.FEV.OM	iron complex outermembrane recepter protein	--	--

[denovogenes]_102589	72.49	7e-139	rum:CK1_00620	K03168	topA	DNA topoisomerase I	5.99.1.2	--

[denovogenes]_102590	82.53	3e-159	bfi:CIY_05700	K03701	uvrA	excinuclease ABC subunit A	--	Genetic Information Processing--Replication and repair--Nucleotide excision repair

[denovogenes]_102593	98.88	0.0	fpla:A4U99_11245	K03310	TC.AGCS	alanine or glycine:cation symporter, AGCS family	--	--

[denovogenes]_102594	100.00	0.0	pdf:CD630DERM_07410	K00864	glpK, GK	glycerol kinase	2.7.1.30	Metabolism--Lipid metabolism--Glycerolipid metabolism|Metabolism--Global and overview maps--Metabolic pathways|Organismal Systems--Endocrine system--PPAR signaling pathway|Organismal Systems--Environmental adaptation--Plant-pathogen interaction

[denovogenes]_102597	40.43	2e-59	ava:Ava_2025	K00705	malQ	4-alpha-glucanotransferase	2.4.1.25	Metabolism--Carbohydrate metabolism--Starch and sucrose metabolism|Metabolism--Global and overview maps--Metabolic pathways

[denovogenes]_1026	99.09	0.0	bbrc:B7019_2029	K02004	ABC.CD.P	putative ABC transport system permease protein	--	--

[denovogenes]_102600	58.36	2e-100	csh:Closa_3080	K13542	cobA-hemD	uroporphyrinogen III methyltransferase / synthase	2.1.1.107 4.2.1.75	Metabolism--Metabolism of cofactors and vitamins--Porphyrin and chlorophyll metabolism|Metabolism--Global and overview maps--Metabolic pathways|Metabolism--Global and overview maps--Biosynthesis of secondary metabolites

[denovogenes]_102603	94.03	2e-171	bcel:BcellWH2_01530	K03561	exbB	biopolymer transport protein ExbB	--	--

[denovogenes]_102605	89.11	8e-171	btho:Btheta7330_02631	K07001	K07001	NTE family protein	--	--

[denovogenes]_102609	98.51	0.0	boa:Bovatus_00851	K07001	K07001	NTE family protein	--	--

[denovogenes]_10261	98.85	0.0	cfd:CFNIH1_15960	K00123	fdoG, fdfH	formate dehydrogenase major subunit	1.2.1.2	Metabolism--Carbohydrate metabolism--Glyoxylate and dicarboxylate metabolism|Metabolism--Energy metabolism--Methane metabolism|Metabolism--Global and overview maps--Metabolic pathways|Metabolism--Global and overview maps--Microbial metabolism in diverse environments|Metabolism--Global and overview maps--Carbon metabolism

[denovogenes]_102610	40.78	6e-62	rst:ATY39_15340	K02528	ksgA	16S rRNA (adenine1518-N6/adenine1519-N6)-dimethyltransferase	2.1.1.182	--

[denovogenes]_102611	76.49	2e-157	osp:Odosp_0790	K06223	dam	DNA adenine methylase	2.1.1.72	Genetic Information Processing--Replication and repair--Mismatch repair

[denovogenes]_102615	100.00	0.0	boa:Bovatus_00730	K03924	moxR	MoxR-like ATPase	3.6.3.-	--

[denovogenes]_102617	100.00	0.0	pvl:AOB99_05070	K02564	nagB, GNPDA	glucosamine-6-phosphate deaminase	3.5.99.6	Metabolism--Carbohydrate metabolism--Amino sugar and nucleotide sugar metabolism|Metabolism--Global and overview maps--Metabolic pathways

[denovogenes]_102618	64.66	1e-123	ipo:Ilyop_0201	K02108	ATPF0A, atpB	F-type H+-transporting ATPase subunit a	--	Metabolism--Energy metabolism--Oxidative phosphorylation|Metabolism--Energy metabolism--Photosynthesis|Metabolism--Global and overview maps--Metabolic pathways

[denovogenes]_102621	99.25	0.0	pvl:AOB99_10215	K02389	flgD	flagellar basal-body rod modification protein FlgD	--	Cellular Processes--Cell motility--Flagellar assembly

[denovogenes]_102623	73.78	3e-144	emu:EMQU_2576	K08602	pepF, pepB	oligoendopeptidase F	3.4.24.-	--

[denovogenes]_102626	77.19	4e-151	vpr:Vpar_0483	K00868	pdxK, pdxY	pyridoxine kinase	2.7.1.35	Metabolism--Metabolism of cofactors and vitamins--Vitamin B6 metabolism|Metabolism--Global and overview maps--Metabolic pathways

[denovogenes]_102630	89.43	8e-178	ega:AL523_03565	K00016	LDH, ldh	L-lactate dehydrogenase	1.1.1.27	Metabolism--Carbohydrate metabolism--Glycolysis / Gluconeogenesis|Metabolism--Amino acid metabolism--Cysteine and methionine metabolism|Metabolism--Carbohydrate metabolism--Pyruvate metabolism|Metabolism--Carbohydrate metabolism--Propanoate metabolism|Metabolism--Global and overview maps--Metabolic pathways|Metabolism--Global and overview maps--Biosynthesis of secondary metabolites|Metabolism--Global and overview maps--Microbial metabolism in diverse environments|Metabolism--Global and overview maps--Biosynthesis of antibiotics|Organismal Systems--Endocrine system--Glucagon signaling pathway

[denovogenes]_102633	88.01	0.0	bfb:VU15_03880	K06889	K06889	uncharacterized protein	--	--

[denovogenes]_102635	100.00	0.0	pvl:AOB99_03055	K04655	hypE	hydrogenase expression/formation protein HypE	--	--

[denovogenes]_102636	100.00	0.0	pdi:BDI_0325	K00266	gltD	glutamate synthase (NADPH/NADH) small chain	1.4.1.13 1.4.1.14	Metabolism--Amino acid metabolism--Alanine, aspartate and glutamate metabolism|Metabolism--Energy metabolism--Nitrogen metabolism|Metabolism--Global and overview maps--Metabolic pathways|Metabolism--Global and overview maps--Biosynthesis of secondary metabolites|Metabolism--Global and overview maps--Microbial metabolism in diverse environments|Metabolism--Global and overview maps--Biosynthesis of antibiotics|Metabolism--Global and overview maps--Biosynthesis of amino acids

[denovogenes]_102639	94.05	0.0	spen:107002698	K03527	ispH, lytB	4-hydroxy-3-methylbut-2-en-1-yl diphosphate reductase	1.17.7.4	Metabolism--Metabolism of terpenoids and polyketides--Terpenoid backbone biosynthesis|Metabolism--Global and overview maps--Metabolic pathways|Metabolism--Global and overview maps--Biosynthesis of secondary metabolites|Metabolism--Global and overview maps--Biosynthesis of antibiotics

[denovogenes]_102645	58.08	4e-96	cbt:CLH_0760	K00826	E2.6.1.42, ilvE	branched-chain amino acid aminotransferase	2.6.1.42	Metabolism--Amino acid metabolism--Cysteine and methionine metabolism|Metabolism--Amino acid metabolism--Valine, leucine and isoleucine degradation|Metabolism--Amino acid metabolism--Valine, leucine and isoleucine biosynthesis|Metabolism--Metabolism of cofactors and vitamins--Pantothenate and CoA biosynthesis|Metabolism--Global and overview maps--Metabolic pathways|Metabolism--Global and overview maps--Biosynthesis of secondary metabolites|Metabolism--Global and overview maps--Biosynthesis of antibiotics|Metabolism--Global and overview maps--2-Oxocarboxylic acid metabolism|Metabolism--Global and overview maps--Biosynthesis of amino acids

[denovogenes]_102647	75.00	7e-143	cbv:U729_2797	K00981	E2.7.7.41, CDS1, CDS2, cdsA	phosphatidate cytidylyltransferase	2.7.7.41	Metabolism--Lipid metabolism--Glycerophospholipid metabolism|Metabolism--Global and overview maps--Metabolic pathways|Metabolism--Global and overview maps--Biosynthesis of secondary metabolites|Environmental Information Processing--Signal transduction--Phosphatidylinositol signaling system

[denovogenes]_102650	100.00	0.0	mmk:MU9_19	K02073	metQ	D-methionine transport system substrate-binding protein	--	Environmental Information Processing--Membrane transport--ABC transporters

[denovogenes]_102651	100.00	0.0	mmk:MU9_3055	K03589	ftsQ	cell division protein FtsQ	--	Cellular Processes--Cell growth and death--Cell cycle - Caulobacter

[denovogenes]_102653	100.00	0.0	mmk:MU9_3350	K02065	mlaF, linL, mkl	phospholipid/cholesterol/gamma-HCH transport system ATP-binding protein	--	Environmental Information Processing--Membrane transport--ABC transporters

[denovogenes]_102656	73.96	7e-147	ene:ENT_18390	K00858	ppnK, NADK	NAD+ kinase	2.7.1.23	Metabolism--Metabolism of cofactors and vitamins--Nicotinate and nicotinamide metabolism|Metabolism--Global and overview maps--Metabolic pathways

[denovogenes]_102657	64.42	3e-123	llm:llmg_0412	K00784	rnz	ribonuclease Z	3.1.26.11	Genetic Information Processing--Translation--RNA transport

[denovogenes]_102658	100.00	0.0	bfb:VU15_11700	K05807	bamD	outer membrane protein assembly factor BamD	--	--

[denovogenes]_102659	64.66	2e-121	cbz:Cbs_0647	K03148	thiF	sulfur carrier protein ThiS adenylyltransferase	2.7.7.73	Metabolism--Metabolism of cofactors and vitamins--Thiamine metabolism|Metabolism--Global and overview maps--Metabolic pathways|Genetic Information Processing--Folding, sorting and degradation--Sulfur relay system

[denovogenes]_102660	100.00	0.0	bfb:VU15_04365	K01627	kdsA	2-dehydro-3-deoxyphosphooctonate aldolase (KDO 8-P synthase)	2.5.1.55	Metabolism--Glycan biosynthesis and metabolism--Lipopolysaccharide biosynthesis|Metabolism--Global and overview maps--Metabolic pathways

[denovogenes]_102661	74.16	2e-140	cbt:CLH_3272	K00784	rnz	ribonuclease Z	3.1.26.11	Genetic Information Processing--Translation--RNA transport

[denovogenes]_102665	54.41	4e-102	gym:GYMC10_6248	K16787	ecfA2	energy-coupling factor transport system ATP-binding protein	3.6.3.-	Environmental Information Processing--Membrane transport--ABC transporters

[denovogenes]_10267	100.00	0.0	paeo:M801_3303	K01114	plcC	phospholipase C	3.1.4.3	Metabolism--Carbohydrate metabolism--Inositol phosphate metabolism|Metabolism--Lipid metabolism--Glycerophospholipid metabolism|Metabolism--Lipid metabolism--Ether lipid metabolism|Metabolism--Global and overview maps--Metabolic pathways|Metabolism--Global and overview maps--Biosynthesis of secondary metabolites|Cellular Processes--Cellular community - prokaryotes--Quorum sensing|Organismal Systems--Endocrine system--Thyroid hormone signaling pathway

[denovogenes]_102670	40.61	8e-61	cmj:AFK66_014615	K11938	cof	HMP-PP phosphatase	3.6.1.-	--

[denovogenes]_102671	100.00	0.0	stb:SGPB_0302	K02795	PTS-Man-EIIC, manY	PTS system, mannose-specific IIC component	--	Metabolism--Carbohydrate metabolism--Fructose and mannose metabolism|Metabolism--Carbohydrate metabolism--Amino sugar and nucleotide sugar metabolism|Metabolism--Global and overview maps--Metabolic pathways|Environmental Information Processing--Membrane transport--Phosphotransferase system (PTS)

[denovogenes]_102672	100.00	0.0	ene:ENT_00740	K02795	PTS-Man-EIIC, manY	PTS system, mannose-specific IIC component	--	Metabolism--Carbohydrate metabolism--Fructose and mannose metabolism|Metabolism--Carbohydrate metabolism--Amino sugar and nucleotide sugar metabolism|Metabolism--Global and overview maps--Metabolic pathways|Environmental Information Processing--Membrane transport--Phosphotransferase system (PTS)

[denovogenes]_102673	62.92	3e-127	clt:CM240_2056	K09692	tagG	teichoic acid transport system permease protein	--	Environmental Information Processing--Membrane transport--ABC transporters

[denovogenes]_10268	63.62	0.0	fpr:FP2_23890	K02355	fusA, GFM, EFG	elongation factor G	--	--

[denovogenes]_102680	45.69	7e-79	cbk:CLL_A1189	K06383	spoIIGA	stage II sporulation protein GA (sporulation sigma-E factor processing peptidase)	3.4.23.-	--

[denovogenes]_102681	52.21	2e-95	thl:TEH_06500	K01778	dapF	diaminopimelate epimerase	5.1.1.7	Metabolism--Amino acid metabolism--Lysine biosynthesis|Metabolism--Global and overview maps--Metabolic pathways|Metabolism--Global and overview maps--Biosynthesis of secondary metabolites|Metabolism--Global and overview maps--Microbial metabolism in diverse environments|Metabolism--Global and overview maps--Biosynthesis of antibiotics|Metabolism--Global and overview maps--Biosynthesis of amino acids

[denovogenes]_102682	96.25	0.0	enl:A3UG_11395	K01607	pcaC	4-carboxymuconolactone decarboxylase	4.1.1.44	Metabolism--Xenobiotics biodegradation and metabolism--Benzoate degradation|Metabolism--Global and overview maps--Metabolic pathways|Metabolism--Global and overview maps--Microbial metabolism in diverse environments|Metabolism--Global and overview maps--Degradation of aromatic compounds

[denovogenes]_102685	98.50	0.0	sgt:SGGB_0121	K05833	ABC.X4.A	putative ABC transport system ATP-binding protein	--	--

[denovogenes]_102687	100.00	0.0	ene:ENT_26070	K06867	K06867	uncharacterized protein	--	--

[denovogenes]_102688	64.77	2e-125	cbv:U729_2797	K00981	E2.7.7.41, CDS1, CDS2, cdsA	phosphatidate cytidylyltransferase	2.7.7.41	Metabolism--Lipid metabolism--Glycerophospholipid metabolism|Metabolism--Global and overview maps--Metabolic pathways|Metabolism--Global and overview maps--Biosynthesis of secondary metabolites|Environmental Information Processing--Signal transduction--Phosphatidylinositol signaling system

[denovogenes]_102689	100.00	0.0	stb:SGPB_0372	K02029	ABC.PA.P	polar amino acid transport system permease protein	--	--

[denovogenes]_102690	77.31	1e-148	cbv:U729_1463	K01715	crt	enoyl-CoA hydratase	4.2.1.17	Metabolism--Carbohydrate metabolism--Butanoate metabolism|Metabolism--Global and overview maps--Carbon metabolism

[denovogenes]_102691	66.42	5e-123	efq:DR75_98	K21064	ycsE, yitU, ywtE	5-amino-6-(5-phospho-D-ribitylamino)uracil phosphatase	3.1.3.104	Metabolism--Metabolism of cofactors and vitamins--Riboflavin metabolism|Metabolism--Global and overview maps--Metabolic pathways|Metabolism--Global and overview maps--Biosynthesis of secondary metabolites

[denovogenes]_102692	100.00	0.0	bfb:VU15_17745	K01803	TPI, tpiA	triosephosphate isomerase (TIM)	5.3.1.1	Metabolism--Carbohydrate metabolism--Glycolysis / Gluconeogenesis|Metabolism--Carbohydrate metabolism--Fructose and mannose metabolism|Metabolism--Carbohydrate metabolism--Inositol phosphate metabolism|Metabolism--Energy metabolism--Carbon fixation in photosynthetic organisms|Metabolism--Global and overview maps--Metabolic pathways|Metabolism--Global and overview maps--Biosynthesis of secondary metabolites|Metabolism--Global and overview maps--Microbial metabolism in diverse environments|Metabolism--Global and overview maps--Biosynthesis of antibiotics|Metabolism--Global and overview maps--Carbon metabolism|Metabolism--Global and overview maps--Biosynthesis of amino acids

[denovogenes]_102696	77.65	1e-145	cbv:U729_2797	K00981	E2.7.7.41, CDS1, CDS2, cdsA	phosphatidate cytidylyltransferase	2.7.7.41	Metabolism--Lipid metabolism--Glycerophospholipid metabolism|Metabolism--Global and overview maps--Metabolic pathways|Metabolism--Global and overview maps--Biosynthesis of secondary metabolites|Environmental Information Processing--Signal transduction--Phosphatidylinositol signaling system

[denovogenes]_102697	42.11	2e-66	gpa:GPA_12330	K02654	pilD, pppA	leader peptidase (prepilin peptidase) / N-methyltransferase	3.4.23.43 2.1.1.-	--

[denovogenes]_10270	100.00	0.0	cfd:CFNIH1_20555	K02400	flhA	flagellar biosynthesis protein FlhA	--	Cellular Processes--Cell motility--Flagellar assembly

[denovogenes]_102700	100.00	0.0	tur:AT726_04000	K18236	lnuB_F, lin	lincosamide nucleotidyltransferase B/F	--	--

[denovogenes]_102702	99.63	0.0	efq:DR75_2445	K02795	PTS-Man-EIIC, manY	PTS system, mannose-specific IIC component	--	Metabolism--Carbohydrate metabolism--Fructose and mannose metabolism|Metabolism--Carbohydrate metabolism--Amino sugar and nucleotide sugar metabolism|Metabolism--Global and overview maps--Metabolic pathways|Environmental Information Processing--Membrane transport--Phosphotransferase system (PTS)

[denovogenes]_102703	67.42	3e-126	pdf:CD630DERM_14950	K00286	proC	pyrroline-5-carboxylate reductase	1.5.1.2	Metabolism--Amino acid metabolism--Arginine and proline metabolism|Metabolism--Global and overview maps--Metabolic pathways|Metabolism--Global and overview maps--Biosynthesis of secondary metabolites|Metabolism--Global and overview maps--Biosynthesis of antibiotics|Metabolism--Global and overview maps--Biosynthesis of amino acids

[denovogenes]_102704	70.66	5e-133	csr:Cspa_c11710	K01962	accA	acetyl-CoA carboxylase carboxyl transferase subunit alpha	6.4.1.2	Metabolism--Lipid metabolism--Fatty acid biosynthesis|Metabolism--Carbohydrate metabolism--Pyruvate metabolism|Metabolism--Carbohydrate metabolism--Propanoate metabolism|Metabolism--Energy metabolism--Carbon fixation pathways in prokaryotes|Metabolism--Global and overview maps--Metabolic pathways|Metabolism--Global and overview maps--Biosynthesis of secondary metabolites|Metabolism--Global and overview maps--Microbial metabolism in diverse environments|Metabolism--Global and overview maps--Biosynthesis of antibiotics|Metabolism--Global and overview maps--Carbon metabolism|Metabolism--Global and overview maps--Fatty acid metabolism

[denovogenes]_102705	99.63	0.0	pmib:BB2000_2142	K03589	ftsQ	cell division protein FtsQ	--	Cellular Processes--Cell growth and death--Cell cycle - Caulobacter

[denovogenes]_102706	99.25	0.0	cbut:ATN24_19155	K00662	aacC	aminoglycoside 3-N-acetyltransferase	2.3.1.81	--

[denovogenes]_102707	67.95	4e-127	cdg:CDBI1_07875	K00878	thiM	hydroxyethylthiazole kinase	2.7.1.50	Metabolism--Metabolism of cofactors and vitamins--Thiamine metabolism|Metabolism--Global and overview maps--Metabolic pathways

[denovogenes]_102708	92.22	9e-175	cbv:U729_2573	K03091	SIG3.4	RNA polymerase sporulation-specific sigma factor	--	--

[denovogenes]_102709	99.25	0.0	pmr:PMI3121	K03832	tonB	periplasmic protein TonB	--	--

[denovogenes]_10271	99.28	0.0	pdi:BDI_2189	K00937	ppk	polyphosphate kinase	2.7.4.1	Metabolism--Energy metabolism--Oxidative phosphorylation|Genetic Information Processing--Folding, sorting and degradation--RNA degradation

[denovogenes]_102712	77.24	2e-143	emu:EMQU_0342	K07459	ybjD	putative ATP-dependent endonuclease of the OLD family	--	--

[denovogenes]_102713	100.00	0.0	stb:SGPB_1005	K02036	pstB	phosphate transport system ATP-binding protein	3.6.3.27	Environmental Information Processing--Membrane transport--ABC transporters

[denovogenes]_102715	100.00	0.0	boa:Bovatus_03316	K00046	idnO	gluconate 5-dehydrogenase	1.1.1.69	--

[denovogenes]_102716	100.00	0.0	pvl:AOB99_14200	K02031	ABC.PE.A	peptide/nickel transport system ATP-binding protein	--	Cellular Processes--Cellular community - prokaryotes--Quorum sensing

[denovogenes]_102718	78.03	5e-151	cbv:U729_1379	K16785	ecfT	energy-coupling factor transport system permease protein	--	Environmental Information Processing--Membrane transport--ABC transporters

[denovogenes]_102719	88.39	6e-178	cfd:CFNIH1_12855	K06897	K06897	7,8-dihydropterin-6-yl-methyl-4-(beta-D-ribofuranosyl)aminobenzene 5'-phosphate synthase	2.5.1.105	--

[denovogenes]_10272	99.86	0.0	ecoh:ECRM13516_0339	K02011	afuB, fbpB	iron(III) transport system permease protein	--	Environmental Information Processing--Membrane transport--ABC transporters

[denovogenes]_102720	85.77	3e-171	ctet:BN906_01495	K15342	cas1	CRISP-associated protein Cas1	--	--

[denovogenes]_102721	68.42	1e-126	rto:RTO_14350	K02795	PTS-Man-EIIC, manY	PTS system, mannose-specific IIC component	--	Metabolism--Carbohydrate metabolism--Fructose and mannose metabolism|Metabolism--Carbohydrate metabolism--Amino sugar and nucleotide sugar metabolism|Metabolism--Global and overview maps--Metabolic pathways|Environmental Information Processing--Membrane transport--Phosphotransferase system (PTS)

[denovogenes]_102723	100.00	0.0	pvl:AOB99_16575	K02073	metQ	D-methionine transport system substrate-binding protein	--	Environmental Information Processing--Membrane transport--ABC transporters

[denovogenes]_102727	100.00	0.0	btho:Btheta7330_05071	K00046	idnO	gluconate 5-dehydrogenase	1.1.1.69	--

[denovogenes]_102728	79.10	3e-155	cbv:U729_1722	K03979	obgE, cgtA	GTPase	3.6.5.-	--

[denovogenes]_102729	91.01	0.0	cbut:ATN24_08710	K04486	E3.1.3.15B	histidinol-phosphatase (PHP family)	3.1.3.15	Metabolism--Amino acid metabolism--Histidine metabolism|Metabolism--Global and overview maps--Metabolic pathways|Metabolism--Global and overview maps--Biosynthesis of secondary metabolites|Metabolism--Global and overview maps--Biosynthesis of amino acids

[denovogenes]_10273	88.24	0.0	csh:Closa_1844	K03168	topA	DNA topoisomerase I	5.99.1.2	--

[denovogenes]_102730	77.15	2e-151	cbt:CLH_0270	K16785	ecfT	energy-coupling factor transport system permease protein	--	Environmental Information Processing--Membrane transport--ABC transporters

[denovogenes]_102731	100.00	0.0	pvl:AOB99_06445	K06222	dkgB	2,5-diketo-D-gluconate reductase B	1.1.1.346	--

[denovogenes]_102732	60.98	6e-80	csb:CLSA_c32410	K06872	K06872	uncharacterized protein	--	--

[denovogenes]_102734	83.71	3e-166	cpe:CPE2242	K09773	ppsR	[pyruvate, water dikinase]-phosphate phosphotransferase / [pyruvate, water dikinase] kinase	2.7.4.28 2.7.11.33	--

[denovogenes]_102735	100.00	0.0	pvl:AOB99_14500	K03521	fixA, etfB	electron transfer flavoprotein beta subunit	--	--

[denovogenes]_102739	94.38	0.0	cbut:ATN24_07750	K18682	rny	ribonucrease Y	3.1.-.-	Genetic Information Processing--Folding, sorting and degradation--RNA degradation

[denovogenes]_10274	93.65	0.0	pdi:BDI_0484	K03070	secA	preprotein translocase subunit SecA	--	Cellular Processes--Cellular community - prokaryotes--Quorum sensing|Genetic Information Processing--Folding, sorting and degradation--Protein export|Environmental Information Processing--Membrane transport--Bacterial secretion system

[denovogenes]_102740	79.78	5e-153	pdf:CD630DERM_01100	K18672	dacA	diadenylate cyclase	2.7.7.85	--

[denovogenes]_102741	69.29	1e-135	csr:Cspa_c42200	K17236	araQ	arabinosaccharide transport system permease protein	--	Environmental Information Processing--Membrane transport--ABC transporters

[denovogenes]_102742	65.40	1e-119	str:Sterm_2098	K02795	PTS-Man-EIIC, manY	PTS system, mannose-specific IIC component	--	Metabolism--Carbohydrate metabolism--Fructose and mannose metabolism|Metabolism--Carbohydrate metabolism--Amino sugar and nucleotide sugar metabolism|Metabolism--Global and overview maps--Metabolic pathways|Environmental Information Processing--Membrane transport--Phosphotransferase system (PTS)

[denovogenes]_102743	44.91	7e-70	csd:Clst_2290	K08974	K08974	putative membrane protein	--	--

[denovogenes]_102744	98.88	0.0	cbut:ATN24_11205	K01992	ABC-2.P	ABC-2 type transport system permease protein	--	--

[denovogenes]_102745	89.89	3e-180	bxy:BXY_20080	K05349	bglX	beta-glucosidase	3.2.1.21	Metabolism--Metabolism of other amino acids--Cyanoamino acid metabolism|Metabolism--Carbohydrate metabolism--Starch and sucrose metabolism|Metabolism--Biosynthesis of other secondary metabolites--Phenylpropanoid biosynthesis|Metabolism--Global and overview maps--Metabolic pathways|Metabolism--Global and overview maps--Biosynthesis of secondary metabolites

[denovogenes]_102746	80.30	8e-153	vvm:VVMO6_04472	K02822	PTS-Ula-EIIB, ulaB, sgaB	PTS system, ascorbate-specific IIB component	2.7.1.194	Metabolism--Carbohydrate metabolism--Ascorbate and aldarate metabolism|Metabolism--Global and overview maps--Metabolic pathways|Metabolism--Global and overview maps--Microbial metabolism in diverse environments|Environmental Information Processing--Membrane transport--Phosphotransferase system (PTS)

[denovogenes]_102746	80.30	8e-153	vvm:VVMO6_04472	K03475	PTS-Ula-EIIC, ulaA, sgaT	PTS system, ascorbate-specific IIC component	--	Metabolism--Carbohydrate metabolism--Ascorbate and aldarate metabolism|Metabolism--Global and overview maps--Metabolic pathways|Metabolism--Global and overview maps--Microbial metabolism in diverse environments|Environmental Information Processing--Membrane transport--Phosphotransferase system (PTS)

[denovogenes]_102747	94.01	0.0	cbz:Cbs_3371	K00046	idnO	gluconate 5-dehydrogenase	1.1.1.69	--

[denovogenes]_102750	51.69	4e-94	prg:RB151_031330	K04016	nrfE	cytochrome c-type biogenesis protein NrfE	--	--

[denovogenes]_102751	99.25	0.0	bxy:BXY_30140	K01992	ABC-2.P	ABC-2 type transport system permease protein	--	--

[denovogenes]_102752	90.57	7e-172	prg:RB151_003730	K06158	ABCF3	ATP-binding cassette, subfamily F, member 3	--	--

[denovogenes]_102753	95.51	0.0	cfd:CFNIH1_14275	K13051	iaaA, ASRGL1	beta-aspartyl-peptidase (threonine type)	3.4.19.5	--

[denovogenes]_102755	66.79	2e-126	cbz:Cbs_4931	K15582	oppC	oligopeptide transport system permease protein	--	Human Diseases--Drug resistance: Antimicrobial--beta-Lactam resistance|Environmental Information Processing--Membrane transport--ABC transporters|Cellular Processes--Cellular community - prokaryotes--Quorum sensing

[denovogenes]_102757	40.80	4e-17	ava:Ava_4797	K01452	E3.5.1.41	chitin deacetylase	3.5.1.41	Metabolism--Carbohydrate metabolism--Amino sugar and nucleotide sugar metabolism|Metabolism--Global and overview maps--Metabolic pathways

[denovogenes]_102758	97.75	0.0	bxy:BXY_19350	K01092	E3.1.3.25, IMPA, suhB	myo-inositol-1(or 4)-monophosphatase	3.1.3.25	Metabolism--Biosynthesis of other secondary metabolites--Streptomycin biosynthesis|Metabolism--Carbohydrate metabolism--Inositol phosphate metabolism|Metabolism--Global and overview maps--Metabolic pathways|Environmental Information Processing--Signal transduction--Phosphatidylinositol signaling system

[denovogenes]_102759	65.54	2e-126	pdf:CD630DERM_14950	K00286	proC	pyrroline-5-carboxylate reductase	1.5.1.2	Metabolism--Amino acid metabolism--Arginine and proline metabolism|Metabolism--Global and overview maps--Metabolic pathways|Metabolism--Global and overview maps--Biosynthesis of secondary metabolites|Metabolism--Global and overview maps--Biosynthesis of antibiotics|Metabolism--Global and overview maps--Biosynthesis of amino acids

[denovogenes]_10276	100.00	0.0	fpla:A4U99_05330	K03701	uvrA	excinuclease ABC subunit A	--	Genetic Information Processing--Replication and repair--Nucleotide excision repair

[denovogenes]_102761	67.29	3e-100	cay:CEA_G2828	K06901	pbuG	putative MFS transporter, AGZA family, xanthine/uracil permease	--	--

[denovogenes]_102763	85.07	3e-167	aoe:Clos_0436	K01848	E5.4.99.2A, mcmA1	methylmalonyl-CoA mutase, N-terminal domain	5.4.99.2	Metabolism--Amino acid metabolism--Valine, leucine and isoleucine degradation|Metabolism--Carbohydrate metabolism--Glyoxylate and dicarboxylate metabolism|Metabolism--Carbohydrate metabolism--Propanoate metabolism|Metabolism--Energy metabolism--Carbon fixation pathways in prokaryotes|Metabolism--Global and overview maps--Metabolic pathways|Metabolism--Global and overview maps--Microbial metabolism in diverse environments|Metabolism--Global and overview maps--Carbon metabolism

[denovogenes]_102764	81.34	7e-158	cle:Clole_3442	K06147	ABCB-BAC	ATP-binding cassette, subfamily B, bacterial	--	--

[denovogenes]_102766	52.96	3e-78	reh:H16_A3186	K00011	E1.1.1.21, AKR1	aldehyde reductase	1.1.1.21	Metabolism--Carbohydrate metabolism--Pentose and glucuronate interconversions|Metabolism--Carbohydrate metabolism--Fructose and mannose metabolism|Metabolism--Carbohydrate metabolism--Galactose metabolism|Metabolism--Lipid metabolism--Glycerolipid metabolism|Metabolism--Global and overview maps--Metabolic pathways

[denovogenes]_102767	98.13	0.0	cfd:CFNIH1_06525	K13629	dsdX	D-serine transporter	--	--

[denovogenes]_102768	88.06	4e-174	lba:Lebu_0237	K02358	tuf, TUFM	elongation factor Tu	--	--

[denovogenes]_102769	97.01	2e-171	cbut:ATN24_03120	K02761	PTS-Cel-EIIC, celB, chbC	PTS system, cellobiose-specific IIC component	--	Metabolism--Carbohydrate metabolism--Starch and sucrose metabolism|Environmental Information Processing--Membrane transport--Phosphotransferase system (PTS)

[denovogenes]_10277	98.41	0.0	bfb:VU15_01330	K02621	parC	topoisomerase IV subunit A	5.99.1.-	--

[denovogenes]_102770	43.38	1e-70	clt:CM240_0874	K00694	bcsA	cellulose synthase (UDP-forming)	2.4.1.12	Metabolism--Carbohydrate metabolism--Starch and sucrose metabolism|Metabolism--Global and overview maps--Metabolic pathways|Cellular Processes--Cellular community - prokaryotes--Biofilm formation - Escherichia coli

[denovogenes]_102771	42.16	1e-50	lsa:LCA_1414	K03281	TC.CIC	chloride channel protein, CIC family	--	--

[denovogenes]_102773	62.08	8e-109	mte:CCDC5079_0619	K03043	rpoB	DNA-directed RNA polymerase subunit beta	2.7.7.6	Metabolism--Nucleotide metabolism--Purine metabolism|Metabolism--Nucleotide metabolism--Pyrimidine metabolism|Metabolism--Global and overview maps--Metabolic pathways|Genetic Information Processing--Transcription--RNA polymerase

[denovogenes]_102775	73.88	6e-141	pdf:CD630DERM_05570	K00876	udk, UCK	uridine kinase	2.7.1.48	Metabolism--Nucleotide metabolism--Pyrimidine metabolism|Metabolism--Xenobiotics biodegradation and metabolism--Drug metabolism - other enzymes|Metabolism--Global and overview maps--Metabolic pathways

[denovogenes]_102776	72.86	1e-140	cbv:U729_322	K00266	gltD	glutamate synthase (NADPH/NADH) small chain	1.4.1.13 1.4.1.14	Metabolism--Amino acid metabolism--Alanine, aspartate and glutamate metabolism|Metabolism--Energy metabolism--Nitrogen metabolism|Metabolism--Global and overview maps--Metabolic pathways|Metabolism--Global and overview maps--Biosynthesis of secondary metabolites|Metabolism--Global and overview maps--Microbial metabolism in diverse environments|Metabolism--Global and overview maps--Biosynthesis of antibiotics|Metabolism--Global and overview maps--Biosynthesis of amino acids

[denovogenes]_102777	64.79	1e-128	lgy:T479_05245	K03763	DPO3A2, polC	DNA polymerase III subunit alpha, Gram-positive type	2.7.7.7	Metabolism--Nucleotide metabolism--Purine metabolism|Metabolism--Nucleotide metabolism--Pyrimidine metabolism|Metabolism--Global and overview maps--Metabolic pathways|Genetic Information Processing--Replication and repair--DNA replication|Genetic Information Processing--Replication and repair--Mismatch repair|Genetic Information Processing--Replication and repair--Homologous recombination

[denovogenes]_102778	91.42	1e-180	cbut:ATN24_08245	K02035	ABC.PE.S	peptide/nickel transport system substrate-binding protein	--	Cellular Processes--Cellular community - prokaryotes--Quorum sensing

[denovogenes]_102780	54.68	9e-99	ssu:SSU05_1444	K00688	PYG, glgP	glycogen phosphorylase	2.4.1.1	Metabolism--Carbohydrate metabolism--Starch and sucrose metabolism|Metabolism--Global and overview maps--Metabolic pathways|Metabolism--Global and overview maps--Biosynthesis of secondary metabolites|Cellular Processes--Cellular community - prokaryotes--Biofilm formation - Escherichia coli|Organismal Systems--Endocrine system--Insulin signaling pathway|Organismal Systems--Endocrine system--Glucagon signaling pathway|Human Diseases--Endocrine and metabolic diseases--Insulin resistance

[denovogenes]_102781	65.17	8e-122	dmt:DESME_03105	K00941	thiD	hydroxymethylpyrimidine/phosphomethylpyrimidine kinase	2.7.1.49 2.7.4.7	Metabolism--Metabolism of cofactors and vitamins--Thiamine metabolism|Metabolism--Global and overview maps--Metabolic pathways

[denovogenes]_102783	63.53	2e-123	afn:Acfer_0255	K06153	bacA	undecaprenyl-diphosphatase	3.6.1.27	Metabolism--Glycan biosynthesis and metabolism--Peptidoglycan biosynthesis

[denovogenes]_102784	64.53	5e-119	afn:Acfer_1687	K09949	K09949	uncharacterized protein	--	--

[denovogenes]_102785	56.20	4e-97	dau:Daud_1371	K09013	sufC	Fe-S cluster assembly ATP-binding protein	--	--

[denovogenes]_102786	56.54	8e-98	rto:RTO_00370	K08600	srtB	sortase B	3.4.22.70	--

[denovogenes]_102790	63.74	7e-116	rix:RO1_09830	K00981	E2.7.7.41, CDS1, CDS2, cdsA	phosphatidate cytidylyltransferase	2.7.7.41	Metabolism--Lipid metabolism--Glycerophospholipid metabolism|Metabolism--Global and overview maps--Metabolic pathways|Metabolism--Global and overview maps--Biosynthesis of secondary metabolites|Environmental Information Processing--Signal transduction--Phosphatidylinositol signaling system

[denovogenes]_102793	91.45	2e-172	boa:Bovatus_04352	K03118	tatC	sec-independent protein translocase protein TatC	--	Genetic Information Processing--Folding, sorting and degradation--Protein export|Environmental Information Processing--Membrane transport--Bacterial secretion system

[denovogenes]_102794	74.81	2e-143	cpy:Cphy_2370	K03609	minD	septum site-determining protein MinD	--	--

[denovogenes]_102795	93.61	9e-180	btho:Btheta7330_03778	K01627	kdsA	2-dehydro-3-deoxyphosphooctonate aldolase (KDO 8-P synthase)	2.5.1.55	Metabolism--Glycan biosynthesis and metabolism--Lipopolysaccharide biosynthesis|Metabolism--Global and overview maps--Metabolic pathways

[denovogenes]_102798	79.63	9e-163	rto:RTO_09100	K01628	fucA	L-fuculose-phosphate aldolase	4.1.2.17	Metabolism--Carbohydrate metabolism--Fructose and mannose metabolism|Metabolism--Global and overview maps--Microbial metabolism in diverse environments

[denovogenes]_1028	100.00	0.0	ecol:LY180_06230	K00370	narG, narZ, nxrA	nitrate reductase / nitrite oxidoreductase, alpha subunit	1.7.5.1 1.7.99.4	Metabolism--Energy metabolism--Nitrogen metabolism|Metabolism--Global and overview maps--Microbial metabolism in diverse environments|Environmental Information Processing--Signal transduction--Two-component system

[denovogenes]_10280	99.86	0.0	bfg:BF638R_3531	K00937	ppk	polyphosphate kinase	2.7.4.1	Metabolism--Energy metabolism--Oxidative phosphorylation|Genetic Information Processing--Folding, sorting and degradation--RNA degradation

[denovogenes]_102806	71.54	4e-138	cso:CLS_07230	K00941	thiD	hydroxymethylpyrimidine/phosphomethylpyrimidine kinase	2.7.1.49 2.7.4.7	Metabolism--Metabolism of cofactors and vitamins--Thiamine metabolism|Metabolism--Global and overview maps--Metabolic pathways

[denovogenes]_102808	95.88	0.0	bhl:Bache_0766	K00046	idnO	gluconate 5-dehydrogenase	1.1.1.69	--

[denovogenes]_102810	92.22	2e-177	bhl:Bache_2362	K03149	thiG	thiazole synthase	2.8.1.10	Metabolism--Metabolism of cofactors and vitamins--Thiamine metabolism|Metabolism--Global and overview maps--Metabolic pathways

[denovogenes]_102812	48.18	3e-82	lrz:BJI69_19725	K12308	bgaB, lacA	beta-galactosidase	3.2.1.23	Metabolism--Carbohydrate metabolism--Galactose metabolism

[denovogenes]_102814	100.00	0.0	bdh:GV66_06175	K00046	idnO	gluconate 5-dehydrogenase	1.1.1.69	--

[denovogenes]_102816	97.38	0.0	boa:Bovatus_02870	K01092	E3.1.3.25, IMPA, suhB	myo-inositol-1(or 4)-monophosphatase	3.1.3.25	Metabolism--Biosynthesis of other secondary metabolites--Streptomycin biosynthesis|Metabolism--Carbohydrate metabolism--Inositol phosphate metabolism|Metabolism--Global and overview maps--Metabolic pathways|Environmental Information Processing--Signal transduction--Phosphatidylinositol signaling system

[denovogenes]_102818	100.00	0.0	bxy:BXY_01360	K05807	bamD	outer membrane protein assembly factor BamD	--	--

[denovogenes]_10282	64.79	0.0	btho:Btheta7330_00947	K09955	K09955	uncharacterized protein	--	--

[denovogenes]_102820	53.70	1e-74	thf:MA03_03330	K00757	udp, UPP	uridine phosphorylase	2.4.2.3	Metabolism--Nucleotide metabolism--Pyrimidine metabolism|Metabolism--Xenobiotics biodegradation and metabolism--Drug metabolism - other enzymes|Metabolism--Global and overview maps--Metabolic pathways

[denovogenes]_102822	98.13	0.0	bxy:BXY_01360	K05807	bamD	outer membrane protein assembly factor BamD	--	--

[denovogenes]_102824	62.17	1e-116	ova:OBV_20930	K16785	ecfT	energy-coupling factor transport system permease protein	--	Environmental Information Processing--Membrane transport--ABC transporters

[denovogenes]_102825	99.25	0.0	bvu:BVU_2265	K07085	K07085	putative transport protein	--	--

[denovogenes]_102826	74.81	4e-128	csh:Closa_2509	K05896	scpA	segregation and condensation protein A	--	--

[denovogenes]_102827	43.41	3e-42	ag:CAA12249	K04114	bcrA, badF	benzoyl-CoA reductase subunit A	1.3.7.8	Metabolism--Xenobiotics biodegradation and metabolism--Benzoate degradation|Metabolism--Global and overview maps--Metabolic pathways|Metabolism--Global and overview maps--Microbial metabolism in diverse environments|Metabolism--Global and overview maps--Degradation of aromatic compounds

[denovogenes]_102828	40.48	7e-41	abao:BEQ56_03605	K03524	birA	BirA family transcriptional regulator, biotin operon repressor / biotin-[acetyl-CoA-carboxylase] ligase	6.3.4.15	Metabolism--Metabolism of cofactors and vitamins--Biotin metabolism|Metabolism--Global and overview maps--Metabolic pathways

[denovogenes]_10283	72.54	0.0	cbv:U729_601	K03657	uvrD, pcrA	DNA helicase II / ATP-dependent DNA helicase PcrA	3.6.4.12	Genetic Information Processing--Replication and repair--Nucleotide excision repair|Genetic Information Processing--Replication and repair--Mismatch repair

[denovogenes]_102832	50.94	5e-87	rto:RTO_00370	K08600	srtB	sortase B	3.4.22.70	--

[denovogenes]_102833	43.66	1e-65	ene:ENT_23080	K03930	estA	putative tributyrin esterase	3.1.1.-	--

[denovogenes]_102836	100.00	0.0	btho:Btheta7330_02079	K05807	bamD	outer membrane protein assembly factor BamD	--	--

[denovogenes]_102838	44.36	2e-74	gfe:Gferi_22780	K16203	dppA1	D-amino peptidase	3.4.11.-	--

[denovogenes]_102839	91.39	0.0	bcel:BcellWH2_00419	K05807	bamD	outer membrane protein assembly factor BamD	--	--

[denovogenes]_102840	94.34	1e-174	bdh:GV66_05790	K06153	bacA	undecaprenyl-diphosphatase	3.6.1.27	Metabolism--Glycan biosynthesis and metabolism--Peptidoglycan biosynthesis

[denovogenes]_102841	96.99	0.0	bvu:BVU_0523	K00625	E2.3.1.8, pta	phosphate acetyltransferase	2.3.1.8	Metabolism--Metabolism of other amino acids--Taurine and hypotaurine metabolism|Metabolism--Carbohydrate metabolism--Pyruvate metabolism|Metabolism--Carbohydrate metabolism--Propanoate metabolism|Metabolism--Energy metabolism--Methane metabolism|Metabolism--Energy metabolism--Carbon fixation pathways in prokaryotes|Metabolism--Global and overview maps--Metabolic pathways|Metabolism--Global and overview maps--Microbial metabolism in diverse environments|Metabolism--Global and overview maps--Carbon metabolism

[denovogenes]_102843	99.22	0.0	bxy:BXY_31270	K03787	surE	5'-nucleotidase	3.1.3.5	Metabolism--Nucleotide metabolism--Purine metabolism|Metabolism--Nucleotide metabolism--Pyrimidine metabolism|Metabolism--Metabolism of cofactors and vitamins--Nicotinate and nicotinamide metabolism|Metabolism--Global and overview maps--Metabolic pathways|Metabolism--Global and overview maps--Biosynthesis of secondary metabolites

[denovogenes]_102844	100.00	0.0	shq:A0259_16390	K01092	E3.1.3.25, IMPA, suhB	myo-inositol-1(or 4)-monophosphatase	3.1.3.25	Metabolism--Biosynthesis of other secondary metabolites--Streptomycin biosynthesis|Metabolism--Carbohydrate metabolism--Inositol phosphate metabolism|Metabolism--Global and overview maps--Metabolic pathways|Environmental Information Processing--Signal transduction--Phosphatidylinositol signaling system

[denovogenes]_102845	99.63	0.0	ece:Z4256	K11264	mmcD	methylmalonyl-CoA decarboxylase	4.1.1.41	Metabolism--Carbohydrate metabolism--Propanoate metabolism

[denovogenes]_102847	85.34	5e-171	ssui:T15_0737	K00046	idnO	gluconate 5-dehydrogenase	1.1.1.69	--

[denovogenes]_102849	58.27	2e-107	ibu:IB211_01690c	K06442	tlyA	23S rRNA (cytidine1920-2'-O)/16S rRNA (cytidine1409-2'-O)-methyltransferase	2.1.1.226 2.1.1.227	--

[denovogenes]_10285	57.33	0.0	aav:Aave_0676	K07459	ybjD	putative ATP-dependent endonuclease of the OLD family	--	--

[denovogenes]_102851	40.14	6e-66	tpi:TREPR_0088	K01126	E3.1.4.46, glpQ, ugpQ	glycerophosphoryl diester phosphodiesterase	3.1.4.46	Metabolism--Lipid metabolism--Glycerophospholipid metabolism

[denovogenes]_102853	100.00	0.0	btho:Btheta7330_04184	K02528	ksgA	16S rRNA (adenine1518-N6/adenine1519-N6)-dimethyltransferase	2.1.1.182	--

[denovogenes]_102855	44.64	9e-20	psj:PSJM300_05405	K13683	wcaE	putative colanic acid biosynthesis glycosyltransferase	2.4.-.-	--

[denovogenes]_102857	97.38	0.0	boa:Bovatus_03316	K00046	idnO	gluconate 5-dehydrogenase	1.1.1.69	--

[denovogenes]_102858	74.63	1e-146	lacy:A4V08_19020	K04477	ycdX	putative hydrolase	--	--

[denovogenes]_102859	100.00	0.0	fpla:A4U99_00115	K03497	parB, spo0J	chromosome partitioning protein, ParB family	--	--

[denovogenes]_10286	81.25	0.0	pdf:CD630DERM_12740	K03168	topA	DNA topoisomerase I	5.99.1.2	--

[denovogenes]_102860	60.30	5e-98	fpa:FPR_29500	K00286	proC	pyrroline-5-carboxylate reductase	1.5.1.2	Metabolism--Amino acid metabolism--Arginine and proline metabolism|Metabolism--Global and overview maps--Metabolic pathways|Metabolism--Global and overview maps--Biosynthesis of secondary metabolites|Metabolism--Global and overview maps--Biosynthesis of antibiotics|Metabolism--Global and overview maps--Biosynthesis of amino acids

[denovogenes]_102861	89.02	2e-173	bxy:BXY_31270	K03787	surE	5'-nucleotidase	3.1.3.5	Metabolism--Nucleotide metabolism--Purine metabolism|Metabolism--Nucleotide metabolism--Pyrimidine metabolism|Metabolism--Metabolism of cofactors and vitamins--Nicotinate and nicotinamide metabolism|Metabolism--Global and overview maps--Metabolic pathways|Metabolism--Global and overview maps--Biosynthesis of secondary metabolites

[denovogenes]_102862	59.50	2e-109	cpy:Cphy_2640	K04032	eutT	ethanolamine utilization cobalamin adenosyltransferase	2.5.1.17	Metabolism--Metabolism of cofactors and vitamins--Porphyrin and chlorophyll metabolism|Metabolism--Global and overview maps--Metabolic pathways

[denovogenes]_102863	65.17	6e-130	byl:A4V09_01235	K01992	ABC-2.P	ABC-2 type transport system permease protein	--	--

[denovogenes]_102864	40.89	3e-45	scc:Spico_0460	K00757	udp, UPP	uridine phosphorylase	2.4.2.3	Metabolism--Nucleotide metabolism--Pyrimidine metabolism|Metabolism--Xenobiotics biodegradation and metabolism--Drug metabolism - other enzymes|Metabolism--Global and overview maps--Metabolic pathways

[denovogenes]_102865	53.31	7e-90	fpr:FP2_05750	K13292	lgt, umpA	phosphatidylglycerol:prolipoprotein diacylglycerol transferase	2.-.-.-	--

[denovogenes]_102869	42.38	1e-24	nth:Nther_2544	K01447	E3.5.1.28A, cwlA, xlyA, xlyB	N-acetylmuramoyl-L-alanine amidase	3.5.1.28	--

[denovogenes]_102872	100.00	0.0	elw:ECW_m3646	K02507	hofQ	protein transport protein HofQ	--	--

[denovogenes]_102873	64.42	3e-111	rho:RHOM_10500	K19310	bcrB	bacitracin transport system permease protein	--	Environmental Information Processing--Membrane transport--ABC transporters

[denovogenes]_102876	87.64	8e-177	bxy:BXY_19350	K01092	E3.1.3.25, IMPA, suhB	myo-inositol-1(or 4)-monophosphatase	3.1.3.25	Metabolism--Biosynthesis of other secondary metabolites--Streptomycin biosynthesis|Metabolism--Carbohydrate metabolism--Inositol phosphate metabolism|Metabolism--Global and overview maps--Metabolic pathways|Environmental Information Processing--Signal transduction--Phosphatidylinositol signaling system

[denovogenes]_102877	71.16	7e-137	ibu:IB211_02891c	K03521	fixA, etfB	electron transfer flavoprotein beta subunit	--	--

[denovogenes]_10288	85.07	0.0	rto:RTO_04740	K02355	fusA, GFM, EFG	elongation factor G	--	--

[denovogenes]_102882	74.91	2e-139	byl:A4V09_21225	K00849	galK	galactokinase	2.7.1.6	Metabolism--Carbohydrate metabolism--Galactose metabolism|Metabolism--Carbohydrate metabolism--Amino sugar and nucleotide sugar metabolism|Metabolism--Global and overview maps--Metabolic pathways

[denovogenes]_102884	99.63	0.0	ecy:ECSE_0032	K01956	carA, CPA1	carbamoyl-phosphate synthase small subunit	6.3.5.5	Metabolism--Nucleotide metabolism--Pyrimidine metabolism|Metabolism--Amino acid metabolism--Alanine, aspartate and glutamate metabolism|Metabolism--Global and overview maps--Metabolic pathways

[denovogenes]_102886	97.38	0.0	fpla:A4U99_00170	K07402	xdhC	xanthine dehydrogenase accessory factor	--	--

[denovogenes]_102888	56.93	1e-103	ova:OBV_20670	K11069	potD	spermidine/putrescine transport system substrate-binding protein	--	Environmental Information Processing--Membrane transport--ABC transporters

[denovogenes]_102889	41.35	2e-50	fpa:FPR_17490	K04486	E3.1.3.15B	histidinol-phosphatase (PHP family)	3.1.3.15	Metabolism--Amino acid metabolism--Histidine metabolism|Metabolism--Global and overview maps--Metabolic pathways|Metabolism--Global and overview maps--Biosynthesis of secondary metabolites|Metabolism--Global and overview maps--Biosynthesis of amino acids

[denovogenes]_10289	93.49	0.0	boa:Bovatus_00313	K12373	HEXA_B	hexosaminidase	3.2.1.52	Metabolism--Glycan biosynthesis and metabolism--Other glycan degradation|Metabolism--Glycan biosynthesis and metabolism--Various types of N-glycan biosynthesis|Metabolism--Carbohydrate metabolism--Amino sugar and nucleotide sugar metabolism|Metabolism--Glycan biosynthesis and metabolism--Glycosaminoglycan degradation|Metabolism--Glycan biosynthesis and metabolism--Glycosphingolipid biosynthesis - globo and isoglobo series|Metabolism--Glycan biosynthesis and metabolism--Glycosphingolipid biosynthesis - ganglio series|Metabolism--Global and overview maps--Metabolic pathways|Cellular Processes--Transport and catabolism--Lysosome

[denovogenes]_102895	93.63	0.0	ibu:IB211_00079c	K10824	nikE	nickel transport system ATP-binding protein	3.6.3.24	Environmental Information Processing--Membrane transport--ABC transporters

[denovogenes]_1029	99.34	0.0	stb:SGPB_0665	K16898	addA	ATP-dependent helicase/nuclease subunit A	3.1.-.- 3.6.4.12	--

[denovogenes]_10290	58.49	0.0	rto:RTO_21540	K03572	mutL	DNA mismatch repair protein MutL	--	Genetic Information Processing--Replication and repair--Mismatch repair

[denovogenes]_102900	99.63	0.0	cfd:CFNIH1_05265	K11103	dctA	aerobic C4-dicarboxylate transport protein	--	Environmental Information Processing--Signal transduction--Two-component system

[denovogenes]_102901	67.08	5e-118	eha:Ethha_1215	K06442	tlyA	23S rRNA (cytidine1920-2'-O)/16S rRNA (cytidine1409-2'-O)-methyltransferase	2.1.1.226 2.1.1.227	--

[denovogenes]_102902	62.60	2e-119	csh:Closa_1599	K02823	pyrDII	dihydroorotate dehydrogenase electron transfer subunit	--	Metabolism--Nucleotide metabolism--Pyrimidine metabolism|Metabolism--Global and overview maps--Metabolic pathways

[denovogenes]_102903	52.51	5e-96	rim:ROI_17080	K00891	E2.7.1.71, aroK, aroL	shikimate kinase	2.7.1.71	Metabolism--Amino acid metabolism--Phenylalanine, tyrosine and tryptophan biosynthesis|Metabolism--Global and overview maps--Metabolic pathways|Metabolism--Global and overview maps--Biosynthesis of secondary metabolites|Metabolism--Global and overview maps--Biosynthesis of antibiotics|Metabolism--Global and overview maps--Biosynthesis of amino acids

[denovogenes]_102904	97.38	0.0	cif:AL515_05775	K06222	dkgB	2,5-diketo-D-gluconate reductase B	1.1.1.346	--

[denovogenes]_102907	65.40	7e-127	cpas:Clopa_4804	K01962	accA	acetyl-CoA carboxylase carboxyl transferase subunit alpha	6.4.1.2	Metabolism--Lipid metabolism--Fatty acid biosynthesis|Metabolism--Carbohydrate metabolism--Pyruvate metabolism|Metabolism--Carbohydrate metabolism--Propanoate metabolism|Metabolism--Energy metabolism--Carbon fixation pathways in prokaryotes|Metabolism--Global and overview maps--Metabolic pathways|Metabolism--Global and overview maps--Biosynthesis of secondary metabolites|Metabolism--Global and overview maps--Microbial metabolism in diverse environments|Metabolism--Global and overview maps--Biosynthesis of antibiotics|Metabolism--Global and overview maps--Carbon metabolism|Metabolism--Global and overview maps--Fatty acid metabolism

[denovogenes]_102908	91.39	0.0	bcel:BcellWH2_00419	K05807	bamD	outer membrane protein assembly factor BamD	--	--

[denovogenes]_102909	91.73	5e-180	erb:A4V01_12170	K00266	gltD	glutamate synthase (NADPH/NADH) small chain	1.4.1.13 1.4.1.14	Metabolism--Amino acid metabolism--Alanine, aspartate and glutamate metabolism|Metabolism--Energy metabolism--Nitrogen metabolism|Metabolism--Global and overview maps--Metabolic pathways|Metabolism--Global and overview maps--Biosynthesis of secondary metabolites|Metabolism--Global and overview maps--Microbial metabolism in diverse environments|Metabolism--Global and overview maps--Biosynthesis of antibiotics|Metabolism--Global and overview maps--Biosynthesis of amino acids

[denovogenes]_10291	62.07	0.0	ccel:CCDG5_0790	K01915	glnA, GLUL	glutamine synthetase	6.3.1.2	Metabolism--Amino acid metabolism--Arginine biosynthesis|Metabolism--Amino acid metabolism--Alanine, aspartate and glutamate metabolism|Metabolism--Carbohydrate metabolism--Glyoxylate and dicarboxylate metabolism|Metabolism--Energy metabolism--Nitrogen metabolism|Metabolism--Global and overview maps--Metabolic pathways|Metabolism--Global and overview maps--Microbial metabolism in diverse environments|Metabolism--Global and overview maps--Biosynthesis of amino acids|Environmental Information Processing--Signal transduction--Two-component system|Organismal Systems--Nervous system--Glutamatergic synapse|Organismal Systems--Nervous system--GABAergic synapse

[denovogenes]_102910	87.69	1e-170	bdh:GV66_18475	K02123	ATPVI, ntpI, atpI	V/A-type H+/Na+-transporting ATPase subunit I	--	Metabolism--Energy metabolism--Oxidative phosphorylation|Metabolism--Global and overview maps--Metabolic pathways

[denovogenes]_102911	76.78	2e-147	cle:Clole_3418	K00768	E2.4.2.21, cobU, cobT	nicotinate-nucleotide--dimethylbenzimidazole phosphoribosyltransferase	2.4.2.21	Metabolism--Metabolism of cofactors and vitamins--Porphyrin and chlorophyll metabolism|Metabolism--Global and overview maps--Metabolic pathways

[denovogenes]_102912	83.40	3e-167	hsd:SD1D_1078	K01626	E2.5.1.54, aroF, aroG, aroH	3-deoxy-7-phosphoheptulonate synthase	2.5.1.54	Metabolism--Amino acid metabolism--Phenylalanine, tyrosine and tryptophan biosynthesis|Metabolism--Global and overview maps--Metabolic pathways|Metabolism--Global and overview maps--Biosynthesis of secondary metabolites|Metabolism--Global and overview maps--Biosynthesis of antibiotics|Metabolism--Global and overview maps--Biosynthesis of amino acids|Cellular Processes--Cellular community - prokaryotes--Quorum sensing

[denovogenes]_102916	92.88	0.0	btho:Btheta7330_00338	K03733	xerC	integrase/recombinase XerC	--	--

[denovogenes]_102917	78.65	7e-154	stm:STM1353	K03522	fixB, etfA	electron transfer flavoprotein alpha subunit	--	--

[denovogenes]_102918	76.23	6e-154	bcel:BcellWH2_03225	K01092	E3.1.3.25, IMPA, suhB	myo-inositol-1(or 4)-monophosphatase	3.1.3.25	Metabolism--Biosynthesis of other secondary metabolites--Streptomycin biosynthesis|Metabolism--Carbohydrate metabolism--Inositol phosphate metabolism|Metabolism--Global and overview maps--Metabolic pathways|Environmental Information Processing--Signal transduction--Phosphatidylinositol signaling system

[denovogenes]_102924	83.60	2e-155	ruk:A4V00_02565	K01740	metY	O-acetylhomoserine (thiol)-lyase	2.5.1.49	Metabolism--Amino acid metabolism--Cysteine and methionine metabolism|Metabolism--Global and overview maps--Metabolic pathways

[denovogenes]_102926	77.61	5e-155	bhl:Bache_2855	K01082	cysQ, MET22, BPNT1	3'(2'), 5'-bisphosphate nucleotidase	3.1.3.7	Metabolism--Energy metabolism--Sulfur metabolism|Metabolism--Global and overview maps--Metabolic pathways|Metabolism--Global and overview maps--Microbial metabolism in diverse environments|Metabolism--Global and overview maps--Biosynthesis of antibiotics

[denovogenes]_102928	76.32	4e-148	bprm:CL3_30150	K00058	serA, PHGDH	D-3-phosphoglycerate dehydrogenase	1.1.1.95	Metabolism--Amino acid metabolism--Glycine, serine and threonine metabolism|Metabolism--Energy metabolism--Methane metabolism|Metabolism--Global and overview maps--Metabolic pathways|Metabolism--Global and overview maps--Microbial metabolism in diverse environments|Metabolism--Global and overview maps--Biosynthesis of antibiotics|Metabolism--Global and overview maps--Carbon metabolism|Metabolism--Global and overview maps--Biosynthesis of amino acids

[denovogenes]_102929	61.19	5e-113	blr:BRLA_c018300	K03311	TC.LIVCS	branched-chain amino acid:cation transporter, LIVCS family	--	--

[denovogenes]_102930	77.24	5e-155	bth:BT_3982	K01144	E3.1.11.5	exodeoxyribonuclease V	3.1.11.5	--

[denovogenes]_102933	63.43	1e-127	erb:A4V01_01055	K01223	E3.2.1.86B, bglA	6-phospho-beta-glucosidase	3.2.1.86	Metabolism--Carbohydrate metabolism--Glycolysis / Gluconeogenesis|Metabolism--Carbohydrate metabolism--Starch and sucrose metabolism

[denovogenes]_102934	75.37	6e-149	cpy:Cphy_3390	K00803	AGPS, agpS	alkyldihydroxyacetonephosphate synthase	2.5.1.26	Metabolism--Lipid metabolism--Ether lipid metabolism|Metabolism--Global and overview maps--Metabolic pathways|Cellular Processes--Transport and catabolism--Peroxisome

[denovogenes]_102936	63.06	5e-120	gsb:GSUB_14440	K01966	PCCB, pccB	propionyl-CoA carboxylase beta chain	6.4.1.3	Metabolism--Amino acid metabolism--Valine, leucine and isoleucine degradation|Metabolism--Carbohydrate metabolism--Glyoxylate and dicarboxylate metabolism|Metabolism--Carbohydrate metabolism--Propanoate metabolism|Metabolism--Global and overview maps--Metabolic pathways|Metabolism--Global and overview maps--Microbial metabolism in diverse environments|Metabolism--Global and overview maps--Biosynthesis of antibiotics|Metabolism--Global and overview maps--Carbon metabolism

[denovogenes]_102938	62.31	2e-120	bprs:CK3_16200	K08602	pepF, pepB	oligoendopeptidase F	3.4.24.-	--

[denovogenes]_102939	100.00	0.0	ecq:ECED1_2378	K00033	PGD, gnd, gntZ	6-phosphogluconate dehydrogenase	1.1.1.44 1.1.1.343	Metabolism--Carbohydrate metabolism--Pentose phosphate pathway|Metabolism--Metabolism of other amino acids--Glutathione metabolism|Metabolism--Global and overview maps--Metabolic pathways|Metabolism--Global and overview maps--Biosynthesis of secondary metabolites|Metabolism--Global and overview maps--Microbial metabolism in diverse environments|Metabolism--Global and overview maps--Biosynthesis of antibiotics|Metabolism--Global and overview maps--Carbon metabolism

[denovogenes]_10294	99.86	0.0	bvu:BVU_0463	K02316	dnaG	DNA primase	2.7.7.-	Genetic Information Processing--Replication and repair--DNA replication

[denovogenes]_102940	72.76	2e-143	cst:CLOST_1296	K00600	glyA, SHMT	glycine hydroxymethyltransferase	2.1.2.1	Metabolism--Amino acid metabolism--Glycine, serine and threonine metabolism|Metabolism--Metabolism of other amino acids--Cyanoamino acid metabolism|Metabolism--Carbohydrate metabolism--Glyoxylate and dicarboxylate metabolism|Metabolism--Metabolism of cofactors and vitamins--One carbon pool by folate|Metabolism--Energy metabolism--Methane metabolism|Metabolism--Global and overview maps--Metabolic pathways|Metabolism--Global and overview maps--Biosynthesis of secondary metabolites|Metabolism--Global and overview maps--Microbial metabolism in diverse environments|Metabolism--Global and overview maps--Biosynthesis of antibiotics|Metabolism--Global and overview maps--Carbon metabolism|Metabolism--Global and overview maps--Biosynthesis of amino acids|Human Diseases--Drug resistance: Antineoplastic--Antifolate resistance

[denovogenes]_102943	47.74	2e-83	arh:AHiyo8_34870	K02469	gyrA	DNA gyrase subunit A	5.99.1.3	--

[denovogenes]_102944	76.49	1e-142	ibu:IB211_02376	K00864	glpK, GK	glycerol kinase	2.7.1.30	Metabolism--Lipid metabolism--Glycerolipid metabolism|Metabolism--Global and overview maps--Metabolic pathways|Organismal Systems--Endocrine system--PPAR signaling pathway|Organismal Systems--Environmental adaptation--Plant-pathogen interaction

[denovogenes]_102945	51.14	2e-88	six:BSY16_4794	K02028	ABC.PA.A	polar amino acid transport system ATP-binding protein	3.6.3.21	--

[denovogenes]_102946	88.76	7e-170	pfr:PFREUD_23010	K01151	nfo	deoxyribonuclease IV	3.1.21.2	Genetic Information Processing--Replication and repair--Base excision repair

[denovogenes]_102947	90.08	2e-156	pfre:RM25_1828	K03744	lemA	LemA protein	--	--

[denovogenes]_102949	72.73	1e-130	pfre:RM25_1465	K05810	yfiH	laccase domain-containing protein	--	--

[denovogenes]_102953	88.76	4e-142	pdi:BDI_3148	K09816	znuB	zinc transport system permease protein	--	Environmental Information Processing--Membrane transport--ABC transporters

[denovogenes]_102956	99.25	0.0	btho:Btheta7330_04405	K07052	K07052	uncharacterized protein	--	--

[denovogenes]_102957	42.17	2e-51	prt:AUC31_17250	K00059	fabG	3-oxoacyl-[acyl-carrier protein] reductase	1.1.1.100	Metabolism--Lipid metabolism--Fatty acid biosynthesis|Metabolism--Metabolism of cofactors and vitamins--Biotin metabolism|Metabolism--Lipid metabolism--Biosynthesis of unsaturated fatty acids|Metabolism--Global and overview maps--Metabolic pathways|Metabolism--Global and overview maps--Fatty acid metabolism

[denovogenes]_102958	42.19	8e-27	but:X994_1379	K06952	K06952	uncharacterized protein	--	--

[denovogenes]_10296	47.57	0.0	dvl:Dvul_0060	K01533	copB	Cu2+-exporting ATPase	3.6.3.4	--

[denovogenes]_102960	49.06	9e-90	fpla:A4U99_16940	K19427	epsJ	glycosyltransferase EpsJ	2.4.-.-	--

[denovogenes]_102962	100.00	0.0	paec:M802_5241	K03118	tatC	sec-independent protein translocase protein TatC	--	Genetic Information Processing--Folding, sorting and degradation--Protein export|Environmental Information Processing--Membrane transport--Bacterial secretion system

[denovogenes]_102964	64.17	5e-109	cct:CC1_05870	K01126	E3.1.4.46, glpQ, ugpQ	glycerophosphoryl diester phosphodiesterase	3.1.4.46	Metabolism--Lipid metabolism--Glycerophospholipid metabolism

[denovogenes]_102966	40.66	5e-46	gjf:M493_05430	K01092	E3.1.3.25, IMPA, suhB	myo-inositol-1(or 4)-monophosphatase	3.1.3.25	Metabolism--Biosynthesis of other secondary metabolites--Streptomycin biosynthesis|Metabolism--Carbohydrate metabolism--Inositol phosphate metabolism|Metabolism--Global and overview maps--Metabolic pathways|Environmental Information Processing--Signal transduction--Phosphatidylinositol signaling system

[denovogenes]_102968	67.55	7e-125	fpr:FP2_25520	K03218	rlmB	23S rRNA (guanosine2251-2'-O)-methyltransferase	2.1.1.185	--

[denovogenes]_102969	41.50	3e-15	crg:105338916	K19719	COL2A	collagen, type II, alpha	--	Environmental Information Processing--Signal transduction--PI3K-Akt signaling pathway|Cellular Processes--Cellular community - eukaryotes--Focal adhesion|Environmental Information Processing--Signaling molecules and interaction--ECM-receptor interaction|Organismal Systems--Digestive system--Protein digestion and absorption

[denovogenes]_102971	99.63	0.0	paeu:BN889_03682	K02041	phnC	phosphonate transport system ATP-binding protein	3.6.3.28	Environmental Information Processing--Membrane transport--ABC transporters

[denovogenes]_102972	100.00	0.0	fpla:A4U99_03300	K02405	fliA	RNA polymerase sigma factor for flagellar operon FliA	--	Environmental Information Processing--Signal transduction--Two-component system|Cellular Processes--Cellular community - prokaryotes--Biofilm formation - Pseudomonas aeruginosa|Cellular Processes--Cellular community - prokaryotes--Biofilm formation - Escherichia coli|Cellular Processes--Cell motility--Flagellar assembly|Cellular Processes--Cellular community - prokaryotes--Biofilm formation - Vibrio cholerae

[denovogenes]_102974	41.41	6e-26	blq:L21SP5_03403	K02477	K02477	two-component system, LytT family, response regulator	--	--

[denovogenes]_102977	99.63	0.0	efq:DR75_98	K21064	ycsE, yitU, ywtE	5-amino-6-(5-phospho-D-ribitylamino)uracil phosphatase	3.1.3.104	Metabolism--Metabolism of cofactors and vitamins--Riboflavin metabolism|Metabolism--Global and overview maps--Metabolic pathways|Metabolism--Global and overview maps--Biosynthesis of secondary metabolites

[denovogenes]_102978	49.65	7e-88	gea:GARCT_02574	K00558	DNMT1, dcm	DNA (cytosine-5)-methyltransferase 1	2.1.1.37	Metabolism--Amino acid metabolism--Cysteine and methionine metabolism|Metabolism--Global and overview maps--Metabolic pathways|Human Diseases--Cancers: Overview--MicroRNAs in cancer

[denovogenes]_102979	87.97	2e-167	pdi:BDI_0285	K01627	kdsA	2-dehydro-3-deoxyphosphooctonate aldolase (KDO 8-P synthase)	2.5.1.55	Metabolism--Glycan biosynthesis and metabolism--Lipopolysaccharide biosynthesis|Metabolism--Global and overview maps--Metabolic pathways

[denovogenes]_10298	96.60	0.0	boa:Bovatus_00157	K11381	bkdA	2-oxoisovalerate dehydrogenase E1 component	1.2.4.4	Metabolism--Amino acid metabolism--Valine, leucine and isoleucine degradation|Metabolism--Carbohydrate metabolism--Propanoate metabolism|Metabolism--Global and overview maps--Metabolic pathways|Metabolism--Global and overview maps--Biosynthesis of secondary metabolites|Metabolism--Global and overview maps--Biosynthesis of antibiotics

[denovogenes]_102980	67.42	2e-125	dvl:Dvul_2149	K07056	rsmI	16S rRNA (cytidine1402-2'-O)-methyltransferase	2.1.1.198	--

[denovogenes]_102981	40.15	9e-49	kom:HR38_22055	K11392	rsmF	16S rRNA (cytosine1407-C5)-methyltransferase	2.1.1.178	--

[denovogenes]_102983	100.00	0.0	pael:T223_28570	K19803	lpxT	Kdo2-lipid A phosphotransferase	2.7.4.29	--

[denovogenes]_102988	93.58	0.0	lacy:A4V08_25040	K00286	proC	pyrroline-5-carboxylate reductase	1.5.1.2	Metabolism--Amino acid metabolism--Arginine and proline metabolism|Metabolism--Global and overview maps--Metabolic pathways|Metabolism--Global and overview maps--Biosynthesis of secondary metabolites|Metabolism--Global and overview maps--Biosynthesis of antibiotics|Metabolism--Global and overview maps--Biosynthesis of amino acids

[denovogenes]_10299	97.25	0.0	fpla:A4U99_05070	K03655	recG	ATP-dependent DNA helicase RecG	3.6.4.12	Genetic Information Processing--Replication and repair--Homologous recombination

[denovogenes]_102991	50.78	9e-75	cbut:ATN24_14995	K08974	K08974	putative membrane protein	--	--

[denovogenes]_102992	42.63	3e-32	mbl:AAX09_08705	K07126	K07126	uncharacterized protein	--	--

[denovogenes]_102993	99.25	0.0	cfd:CFNIH1_04465	K13933	mdcE	malonate decarboxylase gamma subunit	4.1.1.87	--

[denovogenes]_102995	100.00	0.0	paf:PAM18_1269	K18302	mexJ	membrane fusion protein, multidrug efflux system	--	--

[denovogenes]_102996	73.86	2e-143	csh:Closa_2360	K17074	lysX2	putative lysine transport system permease protein	--	Environmental Information Processing--Membrane transport--ABC transporters

[denovogenes]_102997	99.63	0.0	paes:SCV20265_2240	K03424	--	--	--	--

[denovogenes]_1030	43.05	0.0	rho:RHOM_03360	K03296	TC.HAE1	hydrophobic/amphiphilic exporter-1 (mainly G- bacteria), HAE1 family	--	--

[denovogenes]_103000	95.88	0.0	lacy:A4V08_23695	K00784	rnz	ribonuclease Z	3.1.26.11	Genetic Information Processing--Translation--RNA transport

[denovogenes]_103002	51.38	9e-82	sap:Sulac_2952	K01692	paaF, echA	enoyl-CoA hydratase	4.2.1.17	Metabolism--Lipid metabolism--Fatty acid degradation|Metabolism--Amino acid metabolism--Valine, leucine and isoleucine degradation|Metabolism--Metabolism of terpenoids and polyketides--Geraniol degradation|Metabolism--Amino acid metabolism--Lysine degradation|Metabolism--Amino acid metabolism--Phenylalanine metabolism|Metabolism--Xenobiotics biodegradation and metabolism--Benzoate degradation|Metabolism--Amino acid metabolism--Tryptophan metabolism|Metabolism--Metabolism of other amino acids--beta-Alanine metabolism|Metabolism--Xenobiotics biodegradation and metabolism--Aminobenzoate degradation|Metabolism--Carbohydrate metabolism--Propanoate metabolism|Metabolism--Carbohydrate metabolism--Butanoate metabolism|Metabolism--Metabolism of terpenoids and polyketides--Limonene and pinene degradation|Metabolism--Xenobiotics biodegradation and metabolism--Caprolactam degradation|Metabolism--Global and overview maps--Metabolic pathways|Metabolism--Global and overview maps--Biosynthesis of secondary metabolites|Metabolism--Global and overview maps--Microbial metabolism in diverse environments|Metabolism--Global and overview maps--Biosynthesis of antibiotics|Metabolism--Global and overview maps--Fatty acid metabolism

[denovogenes]_103003	100.00	1e-167	lacy:A4V08_21350	K02863	RP-L1, MRPL1, rplA	large subunit ribosomal protein L1	--	Genetic Information Processing--Translation--Ribosome

[denovogenes]_103004	97.38	0.0	bfb:VU15_11700	K05807	bamD	outer membrane protein assembly factor BamD	--	--

[denovogenes]_103005	66.04	2e-130	dvg:Deval_0421	K11645	fbaB	fructose-bisphosphate aldolase, class I	4.1.2.13	Metabolism--Carbohydrate metabolism--Glycolysis / Gluconeogenesis|Metabolism--Carbohydrate metabolism--Pentose phosphate pathway|Metabolism--Carbohydrate metabolism--Fructose and mannose metabolism|Metabolism--Energy metabolism--Methane metabolism|Metabolism--Energy metabolism--Carbon fixation in photosynthetic organisms|Metabolism--Global and overview maps--Metabolic pathways|Metabolism--Global and overview maps--Biosynthesis of secondary metabolites|Metabolism--Global and overview maps--Microbial metabolism in diverse environments|Metabolism--Global and overview maps--Biosynthesis of antibiotics|Metabolism--Global and overview maps--Carbon metabolism|Metabolism--Global and overview maps--Biosynthesis of amino acids

[denovogenes]_103008	60.90	3e-123	fjo:Fjoh_4045	K18910	dpe, lre	D-psicose/D-tagatose/L-ribulose 3-epimerase	5.1.3.30 5.1.3.31	--

[denovogenes]_10301	100.00	0.0	rbr:RBR_01000	K03169	topB	DNA topoisomerase III	5.99.1.2	--

[denovogenes]_103010	100.00	0.0	btho:Btheta7330_02044	K01778	dapF	diaminopimelate epimerase	5.1.1.7	Metabolism--Amino acid metabolism--Lysine biosynthesis|Metabolism--Global and overview maps--Metabolic pathways|Metabolism--Global and overview maps--Biosynthesis of secondary metabolites|Metabolism--Global and overview maps--Microbial metabolism in diverse environments|Metabolism--Global and overview maps--Biosynthesis of antibiotics|Metabolism--Global and overview maps--Biosynthesis of amino acids

[denovogenes]_103011	47.78	1e-59	dvg:Deval_2136	K07220	K07220	uncharacterized protein	--	--

[denovogenes]_103013	100.00	0.0	paeo:M801_4125	K02509	hpaH	2-oxo-hept-3-ene-1,7-dioate hydratase	4.2.1.-	Metabolism--Amino acid metabolism--Tyrosine metabolism|Metabolism--Global and overview maps--Microbial metabolism in diverse environments

[denovogenes]_103014	100.00	0.0	pdi:BDI_3148	K09816	znuB	zinc transport system permease protein	--	Environmental Information Processing--Membrane transport--ABC transporters

[denovogenes]_103015	82.03	1e-150	dvg:Deval_1946	K03149	thiG	thiazole synthase	2.8.1.10	Metabolism--Metabolism of cofactors and vitamins--Thiamine metabolism|Metabolism--Global and overview maps--Metabolic pathways

[denovogenes]_103016	40.52	1e-22	blq:L21SP5_03403	K02477	K02477	two-component system, LytT family, response regulator	--	--

[denovogenes]_103017	100.00	0.0	paf:PAM18_3872	K00563	rlmA1	23S rRNA (guanine745-N1)-methyltransferase	2.1.1.187	--

[denovogenes]_103019	87.17	2e-172	pdi:BDI_2584	K02528	ksgA	16S rRNA (adenine1518-N6/adenine1519-N6)-dimethyltransferase	2.1.1.182	--

[denovogenes]_10302	95.23	0.0	bdo:EL88_18705	K18138	acrB, mexB, adeJ, smeE, mtrD, cmeB	multidrug efflux pump	--	Human Diseases--Drug resistance: Antimicrobial--beta-Lactam resistance|Human Diseases--Drug resistance: Antimicrobial--Cationic antimicrobial peptide (CAMP) resistance

[denovogenes]_103021	99.25	0.0	cfd:CFNIH1_12855	K06897	K06897	7,8-dihydropterin-6-yl-methyl-4-(beta-D-ribofuranosyl)aminobenzene 5'-phosphate synthase	2.5.1.105	--

[denovogenes]_103023	75.00	7e-135	bvu:BVU_2951	K00991	ispD	2-C-methyl-D-erythritol 4-phosphate cytidylyltransferase	2.7.7.60	Metabolism--Metabolism of terpenoids and polyketides--Terpenoid backbone biosynthesis|Metabolism--Global and overview maps--Metabolic pathways|Metabolism--Global and overview maps--Biosynthesis of secondary metabolites|Metabolism--Global and overview maps--Biosynthesis of antibiotics

[denovogenes]_103025	95.88	0.0	bfg:BF638R_1000	K01627	kdsA	2-dehydro-3-deoxyphosphooctonate aldolase (KDO 8-P synthase)	2.5.1.55	Metabolism--Glycan biosynthesis and metabolism--Lipopolysaccharide biosynthesis|Metabolism--Global and overview maps--Metabolic pathways

[denovogenes]_103026	99.25	0.0	pdi:BDI_2250	K00286	proC	pyrroline-5-carboxylate reductase	1.5.1.2	Metabolism--Amino acid metabolism--Arginine and proline metabolism|Metabolism--Global and overview maps--Metabolic pathways|Metabolism--Global and overview maps--Biosynthesis of secondary metabolites|Metabolism--Global and overview maps--Biosynthesis of antibiotics|Metabolism--Global and overview maps--Biosynthesis of amino acids

[denovogenes]_103028	83.27	1e-164	lacy:A4V08_33385	K16951	--	--	--	Metabolism--Energy metabolism--Sulfur metabolism|Metabolism--Global and overview maps--Microbial metabolism in diverse environments

[denovogenes]_103030	99.63	0.0	bbrd:BBBR_1871	K07090	K07090	uncharacterized protein	--	--

[denovogenes]_103033	57.43	1e-93	fpla:A4U99_13425	K02034	ABC.PE.P1	peptide/nickel transport system permease protein	--	Cellular Processes--Cellular community - prokaryotes--Quorum sensing

[denovogenes]_103036	98.88	0.0	cfd:CFNIH1_09870	K01956	carA, CPA1	carbamoyl-phosphate synthase small subunit	6.3.5.5	Metabolism--Nucleotide metabolism--Pyrimidine metabolism|Metabolism--Amino acid metabolism--Alanine, aspartate and glutamate metabolism|Metabolism--Global and overview maps--Metabolic pathways

[denovogenes]_103038	100.00	0.0	pdi:BDI_2672	K01476	E3.5.3.1, rocF, arg	arginase	3.5.3.1	Metabolism--Amino acid metabolism--Arginine biosynthesis|Metabolism--Amino acid metabolism--Arginine and proline metabolism|Metabolism--Global and overview maps--Metabolic pathways|Metabolism--Global and overview maps--Biosynthesis of secondary metabolites|Metabolism--Global and overview maps--Biosynthesis of antibiotics|Metabolism--Global and overview maps--Biosynthesis of amino acids|Human Diseases--Infectious diseases: Parasitic--Amoebiasis

[denovogenes]_103048	42.29	8e-55	olo:ADJ70_06970	K17758	nnr	ADP-dependent NAD(P)H-hydrate dehydratase	4.2.1.136	--

[denovogenes]_103048	42.29	8e-55	olo:ADJ70_06970	K17759	AIBP, nnrE	NAD(P)H-hydrate epimerase	5.1.99.6	--

[denovogenes]_103051	40.00	6e-47	baci:B1NLA3E_01680	K07029	dagK	diacylglycerol kinase (ATP)	2.7.1.107	Metabolism--Lipid metabolism--Glycerolipid metabolism|Metabolism--Lipid metabolism--Glycerophospholipid metabolism|Metabolism--Global and overview maps--Metabolic pathways|Metabolism--Global and overview maps--Biosynthesis of secondary metabolites

[denovogenes]_103054	99.25	0.0	bbrd:BBBR_0080	K03799	htpX	heat shock protein HtpX	3.4.24.-	--

[denovogenes]_103057	97.75	0.0	cfd:CFNIH1_23700	K04032	eutT	ethanolamine utilization cobalamin adenosyltransferase	2.5.1.17	Metabolism--Metabolism of cofactors and vitamins--Porphyrin and chlorophyll metabolism|Metabolism--Global and overview maps--Metabolic pathways

[denovogenes]_103058	45.53	6e-70	ibu:IB211_02389	K00059	fabG	3-oxoacyl-[acyl-carrier protein] reductase	1.1.1.100	Metabolism--Lipid metabolism--Fatty acid biosynthesis|Metabolism--Metabolism of cofactors and vitamins--Biotin metabolism|Metabolism--Lipid metabolism--Biosynthesis of unsaturated fatty acids|Metabolism--Global and overview maps--Metabolic pathways|Metabolism--Global and overview maps--Fatty acid metabolism

[denovogenes]_103060	61.60	9e-113	shi:Shel_09670	K00655	plsC	1-acyl-sn-glycerol-3-phosphate acyltransferase	2.3.1.51	Metabolism--Lipid metabolism--Glycerolipid metabolism|Metabolism--Lipid metabolism--Glycerophospholipid metabolism|Metabolism--Global and overview maps--Metabolic pathways|Metabolism--Global and overview maps--Biosynthesis of secondary metabolites

[denovogenes]_103061	99.63	0.0	ecp:ECP_2969	K12519	papD	chaperone protein PapD	--	--

[denovogenes]_103062	43.52	2e-22	doi:FH5T_12030	K02477	K02477	two-component system, LytT family, response regulator	--	--

[denovogenes]_103063	100.00	0.0	paeo:M801_1357	K02298	cyoB	cytochrome o ubiquinol oxidase subunit I	1.10.3.10	Metabolism--Energy metabolism--Oxidative phosphorylation|Metabolism--Global and overview maps--Metabolic pathways

[denovogenes]_103065	97.75	0.0	cfd:CFNIH1_10710	K06222	dkgB	2,5-diketo-D-gluconate reductase B	1.1.1.346	--

[denovogenes]_103066	59.93	1e-116	csh:Closa_1232	K01624	FBA, fbaA	fructose-bisphosphate aldolase, class II	4.1.2.13	Metabolism--Carbohydrate metabolism--Glycolysis / Gluconeogenesis|Metabolism--Carbohydrate metabolism--Pentose phosphate pathway|Metabolism--Carbohydrate metabolism--Fructose and mannose metabolism|Metabolism--Energy metabolism--Methane metabolism|Metabolism--Energy metabolism--Carbon fixation in photosynthetic organisms|Metabolism--Global and overview maps--Metabolic pathways|Metabolism--Global and overview maps--Biosynthesis of secondary metabolites|Metabolism--Global and overview maps--Microbial metabolism in diverse environments|Metabolism--Global and overview maps--Biosynthesis of antibiotics|Metabolism--Global and overview maps--Carbon metabolism|Metabolism--Global and overview maps--Biosynthesis of amino acids

[denovogenes]_103067	64.18	9e-126	rho:RHOM_04020	K01465	URA4, pyrC	dihydroorotase	3.5.2.3	Metabolism--Nucleotide metabolism--Pyrimidine metabolism|Metabolism--Global and overview maps--Metabolic pathways

[denovogenes]_103069	56.12	5e-95	csh:Closa_2388	K10118	msmF	raffinose/stachyose/melibiose transport system permease protein	--	Environmental Information Processing--Membrane transport--ABC transporters

[denovogenes]_10307	99.86	0.0	paeo:M801_5390	K03655	recG	ATP-dependent DNA helicase RecG	3.6.4.12	Genetic Information Processing--Replication and repair--Homologous recombination

[denovogenes]_103070	78.36	2e-151	pdi:BDI_3573	K02005	ABC.CD.TX	HlyD family secretion protein	--	--

[denovogenes]_103072	51.69	2e-98	tde:TDE1273	K02035	ABC.PE.S	peptide/nickel transport system substrate-binding protein	--	Cellular Processes--Cellular community - prokaryotes--Quorum sensing

[denovogenes]_103073	71.54	5e-144	bmq:BMQ_1512	K06971	K06971	uncharacterized protein	--	--

[denovogenes]_103076	100.00	0.0	cdg:CDBI1_00575	K16785	ecfT	energy-coupling factor transport system permease protein	--	Environmental Information Processing--Membrane transport--ABC transporters

[denovogenes]_103078	100.00	0.0	cdl:CDR20291_1989	K12960	mtaD	5-methylthioadenosine/S-adenosylhomocysteine deaminase	3.5.4.31 3.5.4.28	Metabolism--Amino acid metabolism--Cysteine and methionine metabolism|Metabolism--Global and overview maps--Metabolic pathways

[denovogenes]_103079	61.35	4e-113	csr:Cspa_c21490	K04072	adhE	acetaldehyde dehydrogenase / alcohol dehydrogenase	1.2.1.10 1.1.1.1	Metabolism--Carbohydrate metabolism--Glycolysis / Gluconeogenesis|Metabolism--Lipid metabolism--Fatty acid degradation|Metabolism--Amino acid metabolism--Tyrosine metabolism|Metabolism--Carbohydrate metabolism--Pyruvate metabolism|Metabolism--Xenobiotics biodegradation and metabolism--Chloroalkane and chloroalkene degradation|Metabolism--Xenobiotics biodegradation and metabolism--Naphthalene degradation|Metabolism--Carbohydrate metabolism--Butanoate metabolism|Metabolism--Global and overview maps--Metabolic pathways|Metabolism--Global and overview maps--Biosynthesis of secondary metabolites|Metabolism--Global and overview maps--Microbial metabolism in diverse environments|Metabolism--Global and overview maps--Biosynthesis of antibiotics|Metabolism--Global and overview maps--Degradation of aromatic compounds

[denovogenes]_10308	99.42	0.0	fpla:A4U99_13225	K01547	kdpB	K+-transporting ATPase ATPase B chain	3.6.3.12	Environmental Information Processing--Signal transduction--Two-component system

[denovogenes]_103084	96.25	0.0	bhl:Bache_0766	K00046	idnO	gluconate 5-dehydrogenase	1.1.1.69	--

[denovogenes]_103086	61.39	4e-112	lla:L0472	K07497	K07497	putative transposase	--	--

[denovogenes]_103087	92.45	0.0	pvl:AOB99_01155	K01058	pldA	phospholipase A1	3.1.1.32 3.1.1.4	Metabolism--Lipid metabolism--Glycerophospholipid metabolism|Metabolism--Lipid metabolism--Ether lipid metabolism|Metabolism--Lipid metabolism--Arachidonic acid metabolism|Metabolism--Lipid metabolism--Linoleic acid metabolism|Metabolism--Lipid metabolism--alpha-Linolenic acid metabolism|Metabolism--Global and overview maps--Metabolic pathways|Metabolism--Global and overview maps--Biosynthesis of secondary metabolites

[denovogenes]_103088	100.00	0.0	pdf:CD630DERM_29890	K15553	ssuA	sulfonate transport system substrate-binding protein	--	Metabolism--Energy metabolism--Sulfur metabolism|Environmental Information Processing--Membrane transport--ABC transporters

[denovogenes]_10309	69.28	0.0	cpro:CPRO_03770	K04759	feoB	ferrous iron transport protein B	--	--

[denovogenes]_103091	45.98	6e-61	pnl:PNK_1762	K18138	acrB, mexB, adeJ, smeE, mtrD, cmeB	multidrug efflux pump	--	Human Diseases--Drug resistance: Antimicrobial--beta-Lactam resistance|Human Diseases--Drug resistance: Antimicrobial--Cationic antimicrobial peptide (CAMP) resistance

[denovogenes]_103092	93.66	0.0	bfs:BF9343_2442	K04720	cobD	threonine-phosphate decarboxylase	4.1.1.81	Metabolism--Metabolism of cofactors and vitamins--Porphyrin and chlorophyll metabolism

[denovogenes]_103093	82.40	5e-152	euu:ADJ67_03605	K02824	--	--	--	--

[denovogenes]_103094	84.33	1e-167	bfb:VU15_09520	K02013	ABC.FEV.A	iron complex transport system ATP-binding protein	3.6.3.34	Environmental Information Processing--Membrane transport--ABC transporters

[denovogenes]_103095	88.01	2e-171	cfd:CFNIH1_20660	K01087	otsB	trehalose 6-phosphate phosphatase	3.1.3.12	Metabolism--Carbohydrate metabolism--Starch and sucrose metabolism|Metabolism--Global and overview maps--Metabolic pathways

[denovogenes]_103097	43.12	2e-76	coo:CCU_25230	K07497	K07497	putative transposase	--	--

[denovogenes]_103098	100.00	0.0	pdf:CD630DERM_27050	K12960	mtaD	5-methylthioadenosine/S-adenosylhomocysteine deaminase	3.5.4.31 3.5.4.28	Metabolism--Amino acid metabolism--Cysteine and methionine metabolism|Metabolism--Global and overview maps--Metabolic pathways

[denovogenes]_103100	90.64	2e-163	afd:Alfi_1442	K01925	murD	UDP-N-acetylmuramoylalanine--D-glutamate ligase	6.3.2.9	Metabolism--Metabolism of other amino acids--D-Glutamine and D-glutamate metabolism|Metabolism--Glycan biosynthesis and metabolism--Peptidoglycan biosynthesis|Metabolism--Global and overview maps--Metabolic pathways

[denovogenes]_103101	49.47	5e-51	pmu:PM1682	K01138	K01138	uncharacterized sulfatase	3.1.6.-	--

[denovogenes]_103102	88.81	0.0	bfb:VU15_04210	K02564	nagB, GNPDA	glucosamine-6-phosphate deaminase	3.5.99.6	Metabolism--Carbohydrate metabolism--Amino sugar and nucleotide sugar metabolism|Metabolism--Global and overview maps--Metabolic pathways

[denovogenes]_103103	40.98	6e-19	spv:SPH_2087	K01447	E3.5.1.28A, cwlA, xlyA, xlyB	N-acetylmuramoyl-L-alanine amidase	3.5.1.28	--

[denovogenes]_103104	50.39	2e-76	bprl:CL2_22740	K04759	feoB	ferrous iron transport protein B	--	--

[denovogenes]_103105	40.74	2e-33	fnu:FN0028	K07487	K07487	transposase	--	--

[denovogenes]_103106	94.76	0.0	bcel:BcellWH2_05138	K03426	E3.6.1.22, NUDT12, nudC	NAD+ diphosphatase	3.6.1.22	Metabolism--Metabolism of cofactors and vitamins--Nicotinate and nicotinamide metabolism|Metabolism--Global and overview maps--Metabolic pathways|Cellular Processes--Transport and catabolism--Peroxisome

[denovogenes]_103109	54.28	5e-100	mja:MJ_0879	K02588	nifH	nitrogenase iron protein NifH	1.18.6.1	Metabolism--Xenobiotics biodegradation and metabolism--Chloroalkane and chloroalkene degradation|Metabolism--Energy metabolism--Nitrogen metabolism|Metabolism--Global and overview maps--Metabolic pathways|Metabolism--Global and overview maps--Microbial metabolism in diverse environments

[denovogenes]_10311	64.78	0.0	btho:Btheta7330_00947	K09955	K09955	uncharacterized protein	--	--

[denovogenes]_103111	88.76	3e-155	byl:A4V09_02685	K03588	ftsW, spoVE	cell division protein FtsW	--	Cellular Processes--Cell growth and death--Cell cycle - Caulobacter

[denovogenes]_103112	46.03	2e-65	serr:Ser39006_4040	K10710	frlD	fructoselysine 6-kinase	2.7.1.-	--

[denovogenes]_103119	97.49	1e-170	lacy:A4V08_14780	K10112	msmX, msmK, malK, sugC, ggtA, msiK	multiple sugar transport system ATP-binding protein	--	Environmental Information Processing--Membrane transport--ABC transporters

[denovogenes]_10312	99.86	0.0	paeo:M801_4996	K21025	fimX	multidomain signaling protein FimX	--	Cellular Processes--Cellular community - prokaryotes--Biofilm formation - Pseudomonas aeruginosa

[denovogenes]_103122	98.88	0.0	cfd:CFNIH1_10410	K01894	gluQ	glutamyl-Q tRNA(Asp) synthetase	6.1.1.-	--

[denovogenes]_103123	81.41	3e-151	serr:Ser39006_2308	K03694	clpA	ATP-dependent Clp protease ATP-binding subunit ClpA	--	--

[denovogenes]_103124	89.55	2e-177	bhl:Bache_1337	K07139	K07139	uncharacterized protein	--	--

[denovogenes]_103127	85.82	4e-170	afd:Alfi_1333	K03797	E3.4.21.102, prc, ctpA	carboxyl-terminal processing protease	3.4.21.102	--

[denovogenes]_103129	95.90	0.0	bhl:Bache_2503	K01937	pyrG, CTPS	CTP synthase	6.3.4.2	Metabolism--Nucleotide metabolism--Pyrimidine metabolism|Metabolism--Global and overview maps--Metabolic pathways

[denovogenes]_10313	58.95	0.0	aav:Aave_0676	K07459	ybjD	putative ATP-dependent endonuclease of the OLD family	--	--

[denovogenes]_103130	83.96	1e-164	eel:EUBELI_20383	K07137	K07137	uncharacterized protein	--	--

[denovogenes]_103133	99.63	0.0	spen:107023284	K06215	pdxS, pdx1	pyridoxal 5'-phosphate synthase pdxS subunit	4.3.3.6	Metabolism--Metabolism of cofactors and vitamins--Vitamin B6 metabolism

[denovogenes]_103134	92.54	0.0	bcel:BcellWH2_00876	K01868	TARS, thrS	threonyl-tRNA synthetase	6.1.1.3	Genetic Information Processing--Translation--Aminoacyl-tRNA biosynthesis

[denovogenes]_103139	71.64	3e-134	bfi:CIY_05700	K03701	uvrA	excinuclease ABC subunit A	--	Genetic Information Processing--Replication and repair--Nucleotide excision repair

[denovogenes]_10314	49.13	0.0	dvl:Dvul_0060	K01533	copB	Cu2+-exporting ATPase	3.6.3.4	--

[denovogenes]_103144	40.30	6e-40	hbi:HBZC1_04840	K01952	purL, PFAS	phosphoribosylformylglycinamidine synthase	6.3.5.3	Metabolism--Nucleotide metabolism--Purine metabolism|Metabolism--Global and overview maps--Metabolic pathways|Metabolism--Global and overview maps--Biosynthesis of secondary metabolites|Metabolism--Global and overview maps--Biosynthesis of antibiotics

[denovogenes]_103146	90.67	9e-162	csh:Closa_4194	K02123	ATPVI, ntpI, atpI	V/A-type H+/Na+-transporting ATPase subunit I	--	Metabolism--Energy metabolism--Oxidative phosphorylation|Metabolism--Global and overview maps--Metabolic pathways

[denovogenes]_103150	100.00	0.0	boa:Bovatus_01153	K05807	bamD	outer membrane protein assembly factor BamD	--	--

[denovogenes]_103151	98.88	0.0	bxy:BXY_03190	K01991	ABC-2.OM, wza	polysaccharide biosynthesis/export protein	--	Cellular Processes--Cellular community - prokaryotes--Biofilm formation - Escherichia coli

[denovogenes]_103157	58.52	7e-117	csh:Closa_3410	K13653	K13653	AraC family transcriptional regulator	--	--

[denovogenes]_103158	94.76	0.0	boa:Bovatus_02870	K01092	E3.1.3.25, IMPA, suhB	myo-inositol-1(or 4)-monophosphatase	3.1.3.25	Metabolism--Biosynthesis of other secondary metabolites--Streptomycin biosynthesis|Metabolism--Carbohydrate metabolism--Inositol phosphate metabolism|Metabolism--Global and overview maps--Metabolic pathways|Environmental Information Processing--Signal transduction--Phosphatidylinositol signaling system

[denovogenes]_10316	84.20	0.0	pdi:BDI_3793	K04084	dsbD	thiol:disulfide interchange protein DsbD	1.8.1.8	--

[denovogenes]_103162	98.88	0.0	boa:Bovatus_04509	K06173	truA, PUS1	tRNA pseudouridine38-40 synthase	5.4.99.12	--

[denovogenes]_103165	95.51	0.0	btho:Btheta7330_04450	K03561	exbB	biopolymer transport protein ExbB	--	--

[denovogenes]_103166	98.51	0.0	bvu:BVU_1364	K03931	ygjK	putative isomerase	--	--

[denovogenes]_103167	100.00	0.0	pvl:AOB99_13305	K00677	lpxA	UDP-N-acetylglucosamine acyltransferase	2.3.1.129	Metabolism--Glycan biosynthesis and metabolism--Lipopolysaccharide biosynthesis|Metabolism--Global and overview maps--Metabolic pathways|Human Diseases--Drug resistance: Antimicrobial--Cationic antimicrobial peptide (CAMP) resistance

[denovogenes]_103169	99.63	0.0	pdi:BDI_1037	K20074	prpC, phpP	PPM family protein phosphatase	3.1.3.16	--

[denovogenes]_10317	80.82	0.0	pdi:BDI_0207	K01284	dcp	peptidyl-dipeptidase Dcp	3.4.15.5	--

[denovogenes]_103170	61.92	3e-112	cpro:CPRO_25570	K00931	proB	glutamate 5-kinase	2.7.2.11	Metabolism--Amino acid metabolism--Arginine and proline metabolism|Metabolism--Biosynthesis of other secondary metabolites--Carbapenem biosynthesis|Metabolism--Global and overview maps--Metabolic pathways|Metabolism--Global and overview maps--Biosynthesis of antibiotics|Metabolism--Global and overview maps--Biosynthesis of amino acids

[denovogenes]_103171	60.90	6e-123	fjo:Fjoh_4045	K18910	dpe, lre	D-psicose/D-tagatose/L-ribulose 3-epimerase	5.1.3.30 5.1.3.31	--

[denovogenes]_103172	44.32	8e-83	elm:ELI_4378	K06987	K06987	uncharacterized protein	--	--

[denovogenes]_103174	100.00	0.0	ecol:LY180_22220	K02057	ABC.SS.P	simple sugar transport system permease protein	--	--

[denovogenes]_103175	49.63	5e-90	efm:M7W_2338	K07104	catE	catechol 2,3-dioxygenase	1.13.11.2	Metabolism--Xenobiotics biodegradation and metabolism--Chlorocyclohexane and chlorobenzene degradation|Metabolism--Xenobiotics biodegradation and metabolism--Benzoate degradation|Metabolism--Xenobiotics biodegradation and metabolism--Xylene degradation|Metabolism--Xenobiotics biodegradation and metabolism--Styrene degradation|Metabolism--Global and overview maps--Metabolic pathways|Metabolism--Global and overview maps--Microbial metabolism in diverse environments|Metabolism--Global and overview maps--Degradation of aromatic compounds

[denovogenes]_103177	84.47	1e-158	vpr:Vpar_0399	K04771	degP, htrA	serine protease Do	3.4.21.107	Human Diseases--Drug resistance: Antimicrobial--Cationic antimicrobial peptide (CAMP) resistance|Environmental Information Processing--Signal transduction--Two-component system

[denovogenes]_103178	49.44	1e-85	elp:P12B_c4402	K07347	fimD, fimC, mrkC, htrE, cssD	outer membrane usher protein	--	Human Diseases--Infectious diseases: Bacterial--Pertussis

[denovogenes]_10318	87.68	0.0	rto:RTO_04740	K02355	fusA, GFM, EFG	elongation factor G	--	--

[denovogenes]_103180	61.45	7e-116	ipo:Ilyop_0036	K02843	waaF, rfaF	heptosyltransferase II	2.4.-.-	Metabolism--Glycan biosynthesis and metabolism--Lipopolysaccharide biosynthesis|Metabolism--Global and overview maps--Metabolic pathways

[denovogenes]_103181	100.00	0.0	ecoi:ECOPMV1_02726	K02057	ABC.SS.P	simple sugar transport system permease protein	--	--

[denovogenes]_103182	89.93	0.0	efm:M7W_2228	K13252	ptcA	putrescine carbamoyltransferase	2.1.3.6	--

[denovogenes]_103183	87.60	5e-160	vpr:Vpar_0105	K10439	rbsB	ribose transport system substrate-binding protein	--	Environmental Information Processing--Membrane transport--ABC transporters|Cellular Processes--Cell motility--Bacterial chemotaxis

[denovogenes]_103185	99.63	0.0	pdi:BDI_0380	K03585	acrA, mexA, adeI, smeD, mtrC, cmeA	membrane fusion protein, multidrug efflux system	--	Human Diseases--Drug resistance: Antimicrobial--beta-Lactam resistance|Human Diseases--Drug resistance: Antimicrobial--Cationic antimicrobial peptide (CAMP) resistance

[denovogenes]_103186	63.43	2e-115	bpsc:BBPC_1497	K02027	ABC.MS.S	multiple sugar transport system substrate-binding protein	--	--

[denovogenes]_103187	48.51	3e-86	fjo:Fjoh_3113	K01209	abfA	alpha-N-arabinofuranosidase	3.2.1.55	Metabolism--Carbohydrate metabolism--Amino sugar and nucleotide sugar metabolism

[denovogenes]_103188	48.37	1e-39	cpi:Cpin_6252	K01179	E3.2.1.4	endoglucanase	3.2.1.4	Metabolism--Carbohydrate metabolism--Starch and sucrose metabolism|Metabolism--Global and overview maps--Metabolic pathways

[denovogenes]_103189	50.37	3e-87	pdf:CD630DERM_22620	K03305	TC.POT	proton-dependent oligopeptide transporter, POT family	--	--

[denovogenes]_10319	99.42	0.0	efn:DENG_00575	K01811	xylS, yicI	alpha-D-xyloside xylohydrolase	3.2.1.177	--

[denovogenes]_103192	91.95	4e-175	bcel:BcellWH2_04678	K06001	trpB	tryptophan synthase beta chain	4.2.1.20	Metabolism--Amino acid metabolism--Glycine, serine and threonine metabolism|Metabolism--Amino acid metabolism--Phenylalanine, tyrosine and tryptophan biosynthesis|Metabolism--Global and overview maps--Metabolic pathways|Metabolism--Global and overview maps--Biosynthesis of secondary metabolites|Metabolism--Global and overview maps--Biosynthesis of antibiotics|Metabolism--Global and overview maps--Biosynthesis of amino acids

[denovogenes]_103193	78.73	6e-159	fne:FSDG_01047	K00626	E2.3.1.9, atoB	acetyl-CoA C-acetyltransferase	2.3.1.9	Metabolism--Lipid metabolism--Fatty acid degradation|Metabolism--Lipid metabolism--Synthesis and degradation of ketone bodies|Metabolism--Amino acid metabolism--Valine, leucine and isoleucine degradation|Metabolism--Amino acid metabolism--Lysine degradation|Metabolism--Xenobiotics biodegradation and metabolism--Benzoate degradation|Metabolism--Amino acid metabolism--Tryptophan metabolism|Metabolism--Carbohydrate metabolism--Pyruvate metabolism|Metabolism--Carbohydrate metabolism--Glyoxylate and dicarboxylate metabolism|Metabolism--Carbohydrate metabolism--Propanoate metabolism|Metabolism--Carbohydrate metabolism--Butanoate metabolism|Metabolism--Energy metabolism--Carbon fixation pathways in prokaryotes|Metabolism--Metabolism of terpenoids and polyketides--Terpenoid backbone biosynthesis|Metabolism--Global and overview maps--Metabolic pathways|Metabolism--Global and overview maps--Biosynthesis of secondary metabolites|Metabolism--Global and overview maps--Microbial metabolism in diverse environments|Metabolism--Global and overview maps--Biosynthesis of antibiotics|Metabolism--Global and overview maps--Carbon metabolism|Metabolism--Global and overview maps--Fatty acid metabolism|Environmental Information Processing--Signal transduction--Two-component system

[denovogenes]_103194	73.51	4e-142	fhw:RN87_08615	K10540	mglB	methyl-galactoside transport system substrate-binding protein	--	Environmental Information Processing--Membrane transport--ABC transporters|Cellular Processes--Cell motility--Bacterial chemotaxis

[denovogenes]_1032	100.00	0.0	efq:DR75_1946	K03046	rpoC	DNA-directed RNA polymerase subunit beta'	2.7.7.6	Metabolism--Nucleotide metabolism--Purine metabolism|Metabolism--Nucleotide metabolism--Pyrimidine metabolism|Metabolism--Global and overview maps--Metabolic pathways|Genetic Information Processing--Transcription--RNA polymerase

[denovogenes]_10320	78.20	0.0	bxy:BXY_17650	K03657	uvrD, pcrA	DNA helicase II / ATP-dependent DNA helicase PcrA	3.6.4.12	Genetic Information Processing--Replication and repair--Nucleotide excision repair|Genetic Information Processing--Replication and repair--Mismatch repair

[denovogenes]_103201	98.87	0.0	mmk:MU9_2425	K02013	ABC.FEV.A	iron complex transport system ATP-binding protein	3.6.3.34	Environmental Information Processing--Membrane transport--ABC transporters

[denovogenes]_103202	100.00	0.0	mmk:MU9_2593	K07090	K07090	uncharacterized protein	--	--

[denovogenes]_103203	99.62	0.0	mmk:MU9_553	K00640	cysE	serine O-acetyltransferase	2.3.1.30	Metabolism--Amino acid metabolism--Cysteine and methionine metabolism|Metabolism--Energy metabolism--Sulfur metabolism|Metabolism--Global and overview maps--Metabolic pathways|Metabolism--Global and overview maps--Biosynthesis of secondary metabolites|Metabolism--Global and overview maps--Microbial metabolism in diverse environments|Metabolism--Global and overview maps--Carbon metabolism|Metabolism--Global and overview maps--Biosynthesis of amino acids|Cellular Processes--Cellular community - prokaryotes--Biofilm formation - Vibrio cholerae

[denovogenes]_103206	46.33	8e-80	eft:M395_00150	K02530	lacR	DeoR family transcriptional regulator, lactose phosphotransferase system repressor	--	--

[denovogenes]_103207	79.53	1e-141	cbv:U729_1453	K06182	rluF	23S rRNA pseudouridine2604 synthase	5.4.99.21	--

[denovogenes]_103208	100.00	0.0	bfb:VU15_05820	K07387	K07387	putative metalloprotease	3.4.24.-	--

[denovogenes]_103209	99.21	0.0	mmk:MU9_973	K03787	surE	5'-nucleotidase	3.1.3.5	Metabolism--Nucleotide metabolism--Purine metabolism|Metabolism--Nucleotide metabolism--Pyrimidine metabolism|Metabolism--Metabolism of cofactors and vitamins--Nicotinate and nicotinamide metabolism|Metabolism--Global and overview maps--Metabolic pathways|Metabolism--Global and overview maps--Biosynthesis of secondary metabolites

[denovogenes]_10321	100.00	0.0	paeo:M801_2600	K03406	mcp	methyl-accepting chemotaxis protein	--	Environmental Information Processing--Signal transduction--Two-component system|Cellular Processes--Cell motility--Bacterial chemotaxis

[denovogenes]_103211	100.00	0.0	bfs:BF9343_0067	K06153	bacA	undecaprenyl-diphosphatase	3.6.1.27	Metabolism--Glycan biosynthesis and metabolism--Peptidoglycan biosynthesis

[denovogenes]_103212	76.69	2e-152	pdf:CD630DERM_03450	K07816	E2.7.6.5X	putative GTP pyrophosphokinase	2.7.6.5	Metabolism--Nucleotide metabolism--Purine metabolism

[denovogenes]_103213	58.89	2e-105	cbk:CLL_A1097	K02030	ABC.PA.S	polar amino acid transport system substrate-binding protein	--	--

[denovogenes]_103214	97.74	0.0	ses:SARI_03136	K07090	K07090	uncharacterized protein	--	--

[denovogenes]_103215	71.48	8e-130	lacy:A4V08_31215	K03496	parA, soj	chromosome partitioning protein	--	--

[denovogenes]_103217	84.15	5e-164	csr:Cspa_c52320	K03609	minD	septum site-determining protein MinD	--	--

[denovogenes]_103218	82.71	5e-146	lacy:A4V08_27045	K05896	scpA	segregation and condensation protein A	--	--

[denovogenes]_103219	69.32	2e-135	csh:Closa_2360	K17074	lysX2	putative lysine transport system permease protein	--	Environmental Information Processing--Membrane transport--ABC transporters

[denovogenes]_103220	83.91	2e-141	lacy:A4V08_09060	K02003	ABC.CD.A	putative ABC transport system ATP-binding protein	--	--

[denovogenes]_103223	57.53	2e-106	ene:ENT_02590	K02013	ABC.FEV.A	iron complex transport system ATP-binding protein	3.6.3.34	Environmental Information Processing--Membrane transport--ABC transporters

[denovogenes]_103224	79.32	1e-138	lacy:A4V08_01945	K00981	E2.7.7.41, CDS1, CDS2, cdsA	phosphatidate cytidylyltransferase	2.7.7.41	Metabolism--Lipid metabolism--Glycerophospholipid metabolism|Metabolism--Global and overview maps--Metabolic pathways|Metabolism--Global and overview maps--Biosynthesis of secondary metabolites|Environmental Information Processing--Signal transduction--Phosphatidylinositol signaling system

[denovogenes]_103225	100.00	0.0	ene:ENT_18720	K03565	recX	regulatory protein	--	--

[denovogenes]_103226	78.79	1e-145	cbt:CLH_1096	K05833	ABC.X4.A	putative ABC transport system ATP-binding protein	--	--

[denovogenes]_103229	100.00	0.0	mmk:MU9_705	K02053	ABC.SP.P	putative spermidine/putrescine transport system permease protein	--	Cellular Processes--Cellular community - prokaryotes--Quorum sensing

[denovogenes]_103230	43.94	7e-24	lrl:LC705_00842	K07741	antB	anti-repressor protein	--	--

[denovogenes]_103232	70.93	2e-133	csb:CLSA_c18290	K07402	xdhC	xanthine dehydrogenase accessory factor	--	--

[denovogenes]_103233	71.43	5e-140	cbut:ATN24_01290	K00286	proC	pyrroline-5-carboxylate reductase	1.5.1.2	Metabolism--Amino acid metabolism--Arginine and proline metabolism|Metabolism--Global and overview maps--Metabolic pathways|Metabolism--Global and overview maps--Biosynthesis of secondary metabolites|Metabolism--Global and overview maps--Biosynthesis of antibiotics|Metabolism--Global and overview maps--Biosynthesis of amino acids

[denovogenes]_103234	82.89	1e-157	ehr:EHR_13350	K03706	codY	transcriptional pleiotropic repressor	--	--

[denovogenes]_103235	81.12	1e-135	lacy:A4V08_07630	K02020	modA	molybdate transport system substrate-binding protein	--	Environmental Information Processing--Membrane transport--ABC transporters

[denovogenes]_103236	40.81	8e-56	dai:Desaci_2391	K01673	cynT, can	carbonic anhydrase	4.2.1.1	Metabolism--Energy metabolism--Nitrogen metabolism

[denovogenes]_103237	57.52	2e-111	cst:CLOST_1938	K00858	ppnK, NADK	NAD+ kinase	2.7.1.23	Metabolism--Metabolism of cofactors and vitamins--Nicotinate and nicotinamide metabolism|Metabolism--Global and overview maps--Metabolic pathways

[denovogenes]_103239	56.23	3e-108	cbt:CLH_3266	K02015	ABC.FEV.P	iron complex transport system permease protein	--	Environmental Information Processing--Membrane transport--ABC transporters

[denovogenes]_103242	99.25	0.0	efq:DR75_1241	K00981	E2.7.7.41, CDS1, CDS2, cdsA	phosphatidate cytidylyltransferase	2.7.7.41	Metabolism--Lipid metabolism--Glycerophospholipid metabolism|Metabolism--Global and overview maps--Metabolic pathways|Metabolism--Global and overview maps--Biosynthesis of secondary metabolites|Environmental Information Processing--Signal transduction--Phosphatidylinositol signaling system

[denovogenes]_10325	98.99	0.0	boa:Bovatus_00313	K12373	HEXA_B	hexosaminidase	3.2.1.52	Metabolism--Glycan biosynthesis and metabolism--Other glycan degradation|Metabolism--Glycan biosynthesis and metabolism--Various types of N-glycan biosynthesis|Metabolism--Carbohydrate metabolism--Amino sugar and nucleotide sugar metabolism|Metabolism--Glycan biosynthesis and metabolism--Glycosaminoglycan degradation|Metabolism--Glycan biosynthesis and metabolism--Glycosphingolipid biosynthesis - globo and isoglobo series|Metabolism--Glycan biosynthesis and metabolism--Glycosphingolipid biosynthesis - ganglio series|Metabolism--Global and overview maps--Metabolic pathways|Cellular Processes--Transport and catabolism--Lysosome

[denovogenes]_103252	82.71	1e-166	lacy:A4V08_22260	K16785	ecfT	energy-coupling factor transport system permease protein	--	Environmental Information Processing--Membrane transport--ABC transporters

[denovogenes]_103253	63.67	4e-114	pdf:CD630DERM_17790	K06864	K06864	uncharacterized protein	--	--

[denovogenes]_103254	41.70	2e-58	dgi:Desgi_2715	K01715	crt	enoyl-CoA hydratase	4.2.1.17	Metabolism--Carbohydrate metabolism--Butanoate metabolism|Metabolism--Global and overview maps--Carbon metabolism

[denovogenes]_103255	100.00	0.0	efd:EFD32_1464	K03785	aroD	3-dehydroquinate dehydratase I	4.2.1.10	Metabolism--Amino acid metabolism--Phenylalanine, tyrosine and tryptophan biosynthesis|Metabolism--Global and overview maps--Metabolic pathways|Metabolism--Global and overview maps--Biosynthesis of secondary metabolites|Metabolism--Global and overview maps--Biosynthesis of antibiotics|Metabolism--Global and overview maps--Biosynthesis of amino acids

[denovogenes]_103257	86.79	1e-166	pdf:CD630DERM_11500	K03609	minD	septum site-determining protein MinD	--	--

[denovogenes]_10326	73.84	0.0	bco:Bcell_0113	K02355	fusA, GFM, EFG	elongation factor G	--	--

[denovogenes]_103260	80.30	1e-157	lacy:A4V08_25065	K04763	xerD	integrase/recombinase XerD	--	--

[denovogenes]_103261	63.91	2e-125	cbv:U729_1066	K00286	proC	pyrroline-5-carboxylate reductase	1.5.1.2	Metabolism--Amino acid metabolism--Arginine and proline metabolism|Metabolism--Global and overview maps--Metabolic pathways|Metabolism--Global and overview maps--Biosynthesis of secondary metabolites|Metabolism--Global and overview maps--Biosynthesis of antibiotics|Metabolism--Global and overview maps--Biosynthesis of amino acids

[denovogenes]_103262	100.00	0.0	pvl:AOB99_11020	K07090	K07090	uncharacterized protein	--	--

[denovogenes]_103264	99.25	0.0	cbut:ATN24_02615	K01677	E4.2.1.2AA, fumA	fumarate hydratase subunit alpha	4.2.1.2	Metabolism--Carbohydrate metabolism--Citrate cycle (TCA cycle)|Metabolism--Carbohydrate metabolism--Pyruvate metabolism|Metabolism--Energy metabolism--Carbon fixation pathways in prokaryotes|Metabolism--Global and overview maps--Metabolic pathways|Metabolism--Global and overview maps--Biosynthesis of secondary metabolites|Metabolism--Global and overview maps--Microbial metabolism in diverse environments|Metabolism--Global and overview maps--Biosynthesis of antibiotics|Metabolism--Global and overview maps--Carbon metabolism

[denovogenes]_103265	98.50	0.0	cbut:ATN24_17705	K02008	cbiQ	cobalt/nickel transport system permease protein	--	Environmental Information Processing--Membrane transport--ABC transporters

[denovogenes]_103266	100.00	0.0	cbut:ATN24_01290	K00286	proC	pyrroline-5-carboxylate reductase	1.5.1.2	Metabolism--Amino acid metabolism--Arginine and proline metabolism|Metabolism--Global and overview maps--Metabolic pathways|Metabolism--Global and overview maps--Biosynthesis of secondary metabolites|Metabolism--Global and overview maps--Biosynthesis of antibiotics|Metabolism--Global and overview maps--Biosynthesis of amino acids

[denovogenes]_103268	100.00	0.0	shq:A0259_13185	K02795	PTS-Man-EIIC, manY	PTS system, mannose-specific IIC component	--	Metabolism--Carbohydrate metabolism--Fructose and mannose metabolism|Metabolism--Carbohydrate metabolism--Amino sugar and nucleotide sugar metabolism|Metabolism--Global and overview maps--Metabolic pathways|Environmental Information Processing--Membrane transport--Phosphotransferase system (PTS)

[denovogenes]_10327	100.00	0.0	boa:Bovatus_04582	K01915	glnA, GLUL	glutamine synthetase	6.3.1.2	Metabolism--Amino acid metabolism--Arginine biosynthesis|Metabolism--Amino acid metabolism--Alanine, aspartate and glutamate metabolism|Metabolism--Carbohydrate metabolism--Glyoxylate and dicarboxylate metabolism|Metabolism--Energy metabolism--Nitrogen metabolism|Metabolism--Global and overview maps--Metabolic pathways|Metabolism--Global and overview maps--Microbial metabolism in diverse environments|Metabolism--Global and overview maps--Biosynthesis of amino acids|Environmental Information Processing--Signal transduction--Two-component system|Organismal Systems--Nervous system--Glutamatergic synapse|Organismal Systems--Nervous system--GABAergic synapse

[denovogenes]_103270	86.97	9e-169	clt:CM240_2983	K02013	ABC.FEV.A	iron complex transport system ATP-binding protein	3.6.3.34	Environmental Information Processing--Membrane transport--ABC transporters

[denovogenes]_103271	99.62	0.0	cbut:ATN24_04145	K03609	minD	septum site-determining protein MinD	--	--

[denovogenes]_103275	56.44	3e-106	mhg:MHY_02330	K00700	GBE1, glgB	1,4-alpha-glucan branching enzyme	2.4.1.18	Metabolism--Carbohydrate metabolism--Starch and sucrose metabolism|Metabolism--Global and overview maps--Metabolic pathways|Metabolism--Global and overview maps--Biosynthesis of secondary metabolites

[denovogenes]_103277	100.00	0.0	pvl:AOB99_08525	K01591	pyrF	orotidine-5'-phosphate decarboxylase	4.1.1.23	Metabolism--Nucleotide metabolism--Pyrimidine metabolism|Metabolism--Global and overview maps--Metabolic pathways

[denovogenes]_103278	100.00	0.0	pvl:AOB99_08205	K02073	metQ	D-methionine transport system substrate-binding protein	--	Environmental Information Processing--Membrane transport--ABC transporters

[denovogenes]_10328	86.67	0.0	fus:HMPREF0409_02037	K03043	rpoB	DNA-directed RNA polymerase subunit beta	2.7.7.6	Metabolism--Nucleotide metabolism--Purine metabolism|Metabolism--Nucleotide metabolism--Pyrimidine metabolism|Metabolism--Global and overview maps--Metabolic pathways|Genetic Information Processing--Transcription--RNA polymerase

[denovogenes]_103280	97.74	0.0	ecoi:ECOPMV1_02260	K07273	acm	lysozyme	--	--

[denovogenes]_103281	65.58	1e-98	coo:CCU_26810	K03529	smc	chromosome segregation protein	--	--

[denovogenes]_103282	67.19	4e-119	mhg:MHY_01160	K02038	pstA	phosphate transport system permease protein	--	Environmental Information Processing--Membrane transport--ABC transporters

[denovogenes]_103283	100.00	0.0	efe:EFER_1693	K13497	trpGD	anthranilate synthase/phosphoribosyltransferase	4.1.3.27 2.4.2.18	Metabolism--Amino acid metabolism--Phenylalanine, tyrosine and tryptophan biosynthesis|Metabolism--Global and overview maps--Metabolic pathways|Metabolism--Global and overview maps--Biosynthesis of secondary metabolites|Metabolism--Global and overview maps--Biosynthesis of antibiotics|Metabolism--Global and overview maps--Biosynthesis of amino acids

[denovogenes]_103287	50.19	1e-89	fne:FSDG_02410	K03733	xerC	integrase/recombinase XerC	--	--

[denovogenes]_103289	68.30	2e-135	csr:Cspa_c00330	K01613	psd, PISD	phosphatidylserine decarboxylase	4.1.1.65	Metabolism--Lipid metabolism--Glycerophospholipid metabolism|Metabolism--Global and overview maps--Metabolic pathways|Metabolism--Global and overview maps--Biosynthesis of secondary metabolites

[denovogenes]_103290	96.62	0.0	cif:AL515_15005	K00941	thiD	hydroxymethylpyrimidine/phosphomethylpyrimidine kinase	2.7.1.49 2.7.4.7	Metabolism--Metabolism of cofactors and vitamins--Thiamine metabolism|Metabolism--Global and overview maps--Metabolic pathways

[denovogenes]_103291	94.36	0.0	cpf:CPF_2876	K00864	glpK, GK	glycerol kinase	2.7.1.30	Metabolism--Lipid metabolism--Glycerolipid metabolism|Metabolism--Global and overview maps--Metabolic pathways|Organismal Systems--Endocrine system--PPAR signaling pathway|Organismal Systems--Environmental adaptation--Plant-pathogen interaction

[denovogenes]_103292	62.50	2e-121	csr:Cspa_c11710	K01962	accA	acetyl-CoA carboxylase carboxyl transferase subunit alpha	6.4.1.2	Metabolism--Lipid metabolism--Fatty acid biosynthesis|Metabolism--Carbohydrate metabolism--Pyruvate metabolism|Metabolism--Carbohydrate metabolism--Propanoate metabolism|Metabolism--Energy metabolism--Carbon fixation pathways in prokaryotes|Metabolism--Global and overview maps--Metabolic pathways|Metabolism--Global and overview maps--Biosynthesis of secondary metabolites|Metabolism--Global and overview maps--Microbial metabolism in diverse environments|Metabolism--Global and overview maps--Biosynthesis of antibiotics|Metabolism--Global and overview maps--Carbon metabolism|Metabolism--Global and overview maps--Fatty acid metabolism

[denovogenes]_103293	99.62	0.0	bxy:BXY_31350	K02528	ksgA	16S rRNA (adenine1518-N6/adenine1519-N6)-dimethyltransferase	2.1.1.182	--

[denovogenes]_103294	100.00	0.0	pvl:AOB99_10955	K01963	accD	acetyl-CoA carboxylase carboxyl transferase subunit beta	6.4.1.2	Metabolism--Lipid metabolism--Fatty acid biosynthesis|Metabolism--Carbohydrate metabolism--Pyruvate metabolism|Metabolism--Carbohydrate metabolism--Propanoate metabolism|Metabolism--Energy metabolism--Carbon fixation pathways in prokaryotes|Metabolism--Global and overview maps--Metabolic pathways|Metabolism--Global and overview maps--Biosynthesis of secondary metabolites|Metabolism--Global and overview maps--Microbial metabolism in diverse environments|Metabolism--Global and overview maps--Biosynthesis of antibiotics|Metabolism--Global and overview maps--Carbon metabolism|Metabolism--Global and overview maps--Fatty acid metabolism

[denovogenes]_103295	64.66	2e-130	pdf:CD630DERM_10490	K00858	ppnK, NADK	NAD+ kinase	2.7.1.23	Metabolism--Metabolism of cofactors and vitamins--Nicotinate and nicotinamide metabolism|Metabolism--Global and overview maps--Metabolic pathways

[denovogenes]_103296	100.00	0.0	cif:AL515_07990	K02564	nagB, GNPDA	glucosamine-6-phosphate deaminase	3.5.99.6	Metabolism--Carbohydrate metabolism--Amino sugar and nucleotide sugar metabolism|Metabolism--Global and overview maps--Metabolic pathways

[denovogenes]_103297	63.91	2e-121	ccb:Clocel_0470	K01992	ABC-2.P	ABC-2 type transport system permease protein	--	--

[denovogenes]_103298	53.76	3e-84	cle:Clole_2901	K01736	aroC	chorismate synthase	4.2.3.5	Metabolism--Amino acid metabolism--Phenylalanine, tyrosine and tryptophan biosynthesis|Metabolism--Global and overview maps--Metabolic pathways|Metabolism--Global and overview maps--Biosynthesis of secondary metabolites|Metabolism--Global and overview maps--Biosynthesis of antibiotics|Metabolism--Global and overview maps--Biosynthesis of amino acids

[denovogenes]_103299	99.63	0.0	cbut:ATN24_12000	K13789	GGPS	geranylgeranyl diphosphate synthase, type II	2.5.1.1 2.5.1.10 2.5.1.29	Metabolism--Metabolism of terpenoids and polyketides--Terpenoid backbone biosynthesis|Metabolism--Global and overview maps--Metabolic pathways|Metabolism--Global and overview maps--Biosynthesis of secondary metabolites|Metabolism--Global and overview maps--Biosynthesis of antibiotics

[denovogenes]_103300	65.38	2e-107	pjd:Pjdr2_2688	K16927	cbrT	energy-coupling factor transport system substrate-specific component	--	--

[denovogenes]_103301	100.00	0.0	stb:SGPB_1085	K00796	folP	dihydropteroate synthase	2.5.1.15	Metabolism--Metabolism of cofactors and vitamins--Folate biosynthesis|Metabolism--Global and overview maps--Metabolic pathways

[denovogenes]_103302	48.48	1e-84	cso:CLS_04130	K00286	proC	pyrroline-5-carboxylate reductase	1.5.1.2	Metabolism--Amino acid metabolism--Arginine and proline metabolism|Metabolism--Global and overview maps--Metabolic pathways|Metabolism--Global and overview maps--Biosynthesis of secondary metabolites|Metabolism--Global and overview maps--Biosynthesis of antibiotics|Metabolism--Global and overview maps--Biosynthesis of amino acids

[denovogenes]_103303	91.67	2e-179	sent:TY21A_21505	K02314	dnaB	replicative DNA helicase	3.6.4.12	Genetic Information Processing--Replication and repair--DNA replication|Cellular Processes--Cell growth and death--Cell cycle - Caulobacter

[denovogenes]_103305	100.00	0.0	pvl:AOB99_13605	K05801	djlA	DnaJ like chaperone protein	--	--

[denovogenes]_103306	100.00	0.0	pvl:AOB99_10005	K00563	rlmA1	23S rRNA (guanine745-N1)-methyltransferase	2.1.1.187	--

[denovogenes]_103307	58.33	2e-99	cpe:CPE0784	K18231	msrA, vmlR	macrolide transport system ATP-binding/permease protein	--	Environmental Information Processing--Membrane transport--ABC transporters

[denovogenes]_103308	92.86	2e-176	sdz:Asd1617_03511	K01972	E6.5.1.2, ligA, ligB	DNA ligase (NAD+)	6.5.1.2	Genetic Information Processing--Replication and repair--DNA replication|Genetic Information Processing--Replication and repair--Base excision repair|Genetic Information Processing--Replication and repair--Nucleotide excision repair|Genetic Information Processing--Replication and repair--Mismatch repair

[denovogenes]_10331	81.07	0.0	cbv:U729_2787	K02519	infB, MTIF2	translation initiation factor IF-2	--	--

[denovogenes]_103311	53.62	6e-96	cpr:CPR_1340	K02529	lacI, galR	LacI family transcriptional regulator	--	--

[denovogenes]_103312	99.63	0.0	pmib:BB2000_3423	K00135	gabD	succinate-semialdehyde dehydrogenase / glutarate-semialdehyde dehydrogenase	1.2.1.16 1.2.1.79 1.2.1.20	Metabolism--Amino acid metabolism--Alanine, aspartate and glutamate metabolism|Metabolism--Amino acid metabolism--Lysine degradation|Metabolism--Amino acid metabolism--Tyrosine metabolism|Metabolism--Carbohydrate metabolism--Butanoate metabolism|Metabolism--Metabolism of cofactors and vitamins--Nicotinate and nicotinamide metabolism|Metabolism--Global and overview maps--Metabolic pathways|Metabolism--Global and overview maps--Microbial metabolism in diverse environments

[denovogenes]_103313	99.25	0.0	ecz:ECS88_2145	K01840	manB	phosphomannomutase	5.4.2.8	Metabolism--Carbohydrate metabolism--Fructose and mannose metabolism|Metabolism--Carbohydrate metabolism--Amino sugar and nucleotide sugar metabolism|Metabolism--Global and overview maps--Metabolic pathways|Metabolism--Global and overview maps--Biosynthesis of secondary metabolites|Metabolism--Global and overview maps--Biosynthesis of antibiotics

[denovogenes]_103316	94.76	1e-170	psx:DR96_589	K12942	abgT	aminobenzoyl-glutamate transport protein	--	--

[denovogenes]_103318	64.89	7e-100	cbv:U729_2146	K07090	K07090	uncharacterized protein	--	--

[denovogenes]_103319	79.25	7e-155	cbut:ATN24_12210	K03588	ftsW, spoVE	cell division protein FtsW	--	Cellular Processes--Cell growth and death--Cell cycle - Caulobacter

[denovogenes]_10332	98.22	0.0	mmk:MU9_1918	K01354	ptrB	oligopeptidase B	3.4.21.83	Human Diseases--Infectious diseases: Parasitic--Chagas disease (American trypanosomiasis)|Human Diseases--Infectious diseases: Parasitic--African trypanosomiasis

[denovogenes]_103320	100.00	0.0	sft:NCTC1_04264	K07712	glnG, ntrC	two-component system, NtrC family, nitrogen regulation response regulator GlnG	--	Environmental Information Processing--Signal transduction--Two-component system

[denovogenes]_103321	97.75	0.0	stc:str0840	K01533	copB	Cu2+-exporting ATPase	3.6.3.4	--

[denovogenes]_103328	99.25	0.0	cif:AL515_05220	K03587	ftsI	cell division protein FtsI (penicillin-binding protein 3)	--	Metabolism--Glycan biosynthesis and metabolism--Peptidoglycan biosynthesis|Human Diseases--Drug resistance: Antimicrobial--beta-Lactam resistance

[denovogenes]_103329	55.97	7e-100	mst:Msp_0967	K00002	AKR1A1, adh	alcohol dehydrogenase (NADP+)	1.1.1.2	Metabolism--Carbohydrate metabolism--Glycolysis / Gluconeogenesis|Metabolism--Carbohydrate metabolism--Pentose and glucuronate interconversions|Metabolism--Lipid metabolism--Glycerolipid metabolism|Metabolism--Xenobiotics biodegradation and metabolism--Caprolactam degradation|Metabolism--Global and overview maps--Metabolic pathways|Metabolism--Global and overview maps--Biosynthesis of secondary metabolites|Metabolism--Global and overview maps--Microbial metabolism in diverse environments|Metabolism--Global and overview maps--Biosynthesis of antibiotics|Metabolism--Global and overview maps--Degradation of aromatic compounds

[denovogenes]_103331	64.18	5e-121	cbk:CLL_A0156	K02499	yabN	tetrapyrrole methylase family protein / MazG family protein	--	--

[denovogenes]_103332	74.53	2e-147	cld:CLSPO_c36110	K16787	ecfA2	energy-coupling factor transport system ATP-binding protein	3.6.3.-	Environmental Information Processing--Membrane transport--ABC transporters

[denovogenes]_103333	64.05	6e-108	fpa:FPR_28240	K01681	ACO, acnA	aconitate hydratase	4.2.1.3	Metabolism--Carbohydrate metabolism--Citrate cycle (TCA cycle)|Metabolism--Carbohydrate metabolism--Glyoxylate and dicarboxylate metabolism|Metabolism--Energy metabolism--Carbon fixation pathways in prokaryotes|Metabolism--Global and overview maps--Metabolic pathways|Metabolism--Global and overview maps--Biosynthesis of secondary metabolites|Metabolism--Global and overview maps--Microbial metabolism in diverse environments|Metabolism--Global and overview maps--Biosynthesis of antibiotics|Metabolism--Global and overview maps--Carbon metabolism|Metabolism--Global and overview maps--2-Oxocarboxylic acid metabolism|Metabolism--Global and overview maps--Biosynthesis of amino acids

[denovogenes]_103335	76.54	6e-140	xho:A9255_05570	K03615	rnfC	electron transport complex protein RnfC	--	--

[denovogenes]_103337	69.66	2e-128	fnc:HMPREF0946_01639	K02056	ABC.SS.A	simple sugar transport system ATP-binding protein	3.6.3.17	--

[denovogenes]_103339	47.17	8e-71	ssg:Selsp_0850	K05810	yfiH	laccase domain-containing protein	--	--

[denovogenes]_103345	61.87	6e-117	mhg:MHY_05270	K00941	thiD	hydroxymethylpyrimidine/phosphomethylpyrimidine kinase	2.7.1.49 2.7.4.7	Metabolism--Metabolism of cofactors and vitamins--Thiamine metabolism|Metabolism--Global and overview maps--Metabolic pathways

[denovogenes]_103346	41.09	1e-66	sli:Slin_2091	K02477	K02477	two-component system, LytT family, response regulator	--	--

[denovogenes]_10335	79.20	0.0	lacy:A4V08_20920	K17675	SUPV3L1, SUV3	ATP-dependent RNA helicase SUPV3L1/SUV3	3.6.4.13	--

[denovogenes]_103351	77.82	5e-154	bprm:CL3_14210	K16785	ecfT	energy-coupling factor transport system permease protein	--	Environmental Information Processing--Membrane transport--ABC transporters

[denovogenes]_103352	61.25	2e-105	rto:RTO_02520	K06346	jag	spoIIIJ-associated protein	--	--

[denovogenes]_103354	89.66	4e-173	rho:RHOM_04075	K00046	idnO	gluconate 5-dehydrogenase	1.1.1.69	--

[denovogenes]_103355	40.23	3e-71	csq:CSCA_0517	K02031	ABC.PE.A	peptide/nickel transport system ATP-binding protein	--	Cellular Processes--Cellular community - prokaryotes--Quorum sensing

[denovogenes]_103356	65.61	3e-117	csh:Closa_3439	K03496	parA, soj	chromosome partitioning protein	--	--

[denovogenes]_10336	99.86	0.0	bfs:BF9343_1727	K05970	SIAE	sialate O-acetylesterase	3.1.1.53	--

[denovogenes]_103360	93.98	2e-180	bxy:BXY_34340	K01627	kdsA	2-dehydro-3-deoxyphosphooctonate aldolase (KDO 8-P synthase)	2.5.1.55	Metabolism--Glycan biosynthesis and metabolism--Lipopolysaccharide biosynthesis|Metabolism--Global and overview maps--Metabolic pathways

[denovogenes]_103361	88.35	0.0	cle:Clole_0051	K00957	cysD	sulfate adenylyltransferase subunit 2	2.7.7.4	Metabolism--Nucleotide metabolism--Purine metabolism|Metabolism--Biosynthesis of other secondary metabolites--Monobactam biosynthesis|Metabolism--Metabolism of other amino acids--Selenocompound metabolism|Metabolism--Energy metabolism--Sulfur metabolism|Metabolism--Global and overview maps--Metabolic pathways|Metabolism--Global and overview maps--Microbial metabolism in diverse environments|Metabolism--Global and overview maps--Biosynthesis of antibiotics

[denovogenes]_103362	100.00	0.0	bvu:BVU_4028	K00978	rfbF	glucose-1-phosphate cytidylyltransferase	2.7.7.33	Metabolism--Carbohydrate metabolism--Starch and sucrose metabolism|Metabolism--Carbohydrate metabolism--Amino sugar and nucleotide sugar metabolism|Metabolism--Global and overview maps--Metabolic pathways

[denovogenes]_103363	54.44	5e-106	rto:RTO_17430	K04486	E3.1.3.15B	histidinol-phosphatase (PHP family)	3.1.3.15	Metabolism--Amino acid metabolism--Histidine metabolism|Metabolism--Global and overview maps--Metabolic pathways|Metabolism--Global and overview maps--Biosynthesis of secondary metabolites|Metabolism--Global and overview maps--Biosynthesis of amino acids

[denovogenes]_103367	100.00	0.0	bxy:BXY_34340	K01627	kdsA	2-dehydro-3-deoxyphosphooctonate aldolase (KDO 8-P synthase)	2.5.1.55	Metabolism--Glycan biosynthesis and metabolism--Lipopolysaccharide biosynthesis|Metabolism--Global and overview maps--Metabolic pathways

[denovogenes]_103368	56.55	5e-112	bcel:BcellWH2_04146	K06897	K06897	7,8-dihydropterin-6-yl-methyl-4-(beta-D-ribofuranosyl)aminobenzene 5'-phosphate synthase	2.5.1.105	--

[denovogenes]_103369	44.49	4e-71	tpe:Tpen_1240	K00757	udp, UPP	uridine phosphorylase	2.4.2.3	Metabolism--Nucleotide metabolism--Pyrimidine metabolism|Metabolism--Xenobiotics biodegradation and metabolism--Drug metabolism - other enzymes|Metabolism--Global and overview maps--Metabolic pathways

[denovogenes]_103373	57.49	2e-86	ova:OBV_14280	K07284	srtA	sortase A	3.4.22.70	--

[denovogenes]_103374	80.97	7e-142	bhl:Bache_2260	K02233	E2.7.8.26, cobS, cobV	adenosylcobinamide-GDP ribazoletransferase	2.7.8.26	Metabolism--Metabolism of cofactors and vitamins--Porphyrin and chlorophyll metabolism|Metabolism--Global and overview maps--Metabolic pathways

[denovogenes]_103375	63.89	3e-114	ova:OBV_46360	K03496	parA, soj	chromosome partitioning protein	--	--

[denovogenes]_103376	88.72	1e-158	lacy:A4V08_27045	K05896	scpA	segregation and condensation protein A	--	--

[denovogenes]_103379	45.45	5e-59	fro:AALO17_04160	K02030	ABC.PA.S	polar amino acid transport system substrate-binding protein	--	--

[denovogenes]_10338	99.42	0.0	stb:SGPB_1280	K19005	ltaS	lipoteichoic acid synthase	2.7.8.20	Metabolism--Lipid metabolism--Glycerolipid metabolism|Metabolism--Global and overview maps--Metabolic pathways

[denovogenes]_103383	89.81	8e-171	bdh:GV66_18580	K01627	kdsA	2-dehydro-3-deoxyphosphooctonate aldolase (KDO 8-P synthase)	2.5.1.55	Metabolism--Glycan biosynthesis and metabolism--Lipopolysaccharide biosynthesis|Metabolism--Global and overview maps--Metabolic pathways

[denovogenes]_103385	98.13	0.0	btho:Btheta7330_04827	K03734	apbE	FAD:protein FMN transferase	2.7.1.180	--

[denovogenes]_103386	41.47	2e-66	sli:Slin_2091	K02477	K02477	two-component system, LytT family, response regulator	--	--

[denovogenes]_103387	100.00	0.0	bvu:BVU_1171	K06153	bacA	undecaprenyl-diphosphatase	3.6.1.27	Metabolism--Glycan biosynthesis and metabolism--Peptidoglycan biosynthesis

[denovogenes]_103388	44.49	1e-70	euu:ADJ67_07810	K03616	rnfB	electron transport complex protein RnfB	--	--

[denovogenes]_103389	79.70	1e-147	cso:CLS_24350	K00981	E2.7.7.41, CDS1, CDS2, cdsA	phosphatidate cytidylyltransferase	2.7.7.41	Metabolism--Lipid metabolism--Glycerophospholipid metabolism|Metabolism--Global and overview maps--Metabolic pathways|Metabolism--Global and overview maps--Biosynthesis of secondary metabolites|Environmental Information Processing--Signal transduction--Phosphatidylinositol signaling system

[denovogenes]_10339	73.81	0.0	cbv:U729_2416	K12308	bgaB, lacA	beta-galactosidase	3.2.1.23	Metabolism--Carbohydrate metabolism--Galactose metabolism

[denovogenes]_103390	87.22	3e-173	lacy:A4V08_22260	K16785	ecfT	energy-coupling factor transport system permease protein	--	Environmental Information Processing--Membrane transport--ABC transporters

[denovogenes]_103391	100.00	0.0	bxy:BXY_47010	K01572	oadB	oxaloacetate decarboxylase, beta subunit	4.1.1.3	Metabolism--Carbohydrate metabolism--Pyruvate metabolism|Metabolism--Global and overview maps--Metabolic pathways

[denovogenes]_103393	100.00	0.0	bdh:GV66_11545	K01991	ABC-2.OM, wza	polysaccharide biosynthesis/export protein	--	Cellular Processes--Cellular community - prokaryotes--Biofilm formation - Escherichia coli

[denovogenes]_103395	63.53	3e-124	ag:AAA98217	K00561	ermC, ermA	23S rRNA (adenine-N6)-dimethyltransferase	2.1.1.184	--

[denovogenes]_103396	46.62	6e-82	ccb:Clocel_0470	K01992	ABC-2.P	ABC-2 type transport system permease protein	--	--

[denovogenes]_103398	100.00	0.0	btho:Btheta7330_03778	K01627	kdsA	2-dehydro-3-deoxyphosphooctonate aldolase (KDO 8-P synthase)	2.5.1.55	Metabolism--Glycan biosynthesis and metabolism--Lipopolysaccharide biosynthesis|Metabolism--Global and overview maps--Metabolic pathways

[denovogenes]_103399	40.87	5e-50	dfo:Dform_01342	K00059	fabG	3-oxoacyl-[acyl-carrier protein] reductase	1.1.1.100	Metabolism--Lipid metabolism--Fatty acid biosynthesis|Metabolism--Metabolism of cofactors and vitamins--Biotin metabolism|Metabolism--Lipid metabolism--Biosynthesis of unsaturated fatty acids|Metabolism--Global and overview maps--Metabolic pathways|Metabolism--Global and overview maps--Fatty acid metabolism

[denovogenes]_1034	94.43	0.0	ega:AL523_04570	K03043	rpoB	DNA-directed RNA polymerase subunit beta	2.7.7.6	Metabolism--Nucleotide metabolism--Purine metabolism|Metabolism--Nucleotide metabolism--Pyrimidine metabolism|Metabolism--Global and overview maps--Metabolic pathways|Genetic Information Processing--Transcription--RNA polymerase

[denovogenes]_10340	81.42	0.0	rto:RTO_15660	K03168	topA	DNA topoisomerase I	5.99.1.2	--

[denovogenes]_103400	55.35	2e-103	ova:OBV_40000	K01776	murI	glutamate racemase	5.1.1.3	Metabolism--Metabolism of other amino acids--D-Glutamine and D-glutamate metabolism|Metabolism--Global and overview maps--Metabolic pathways

[denovogenes]_103402	63.11	3e-103	ssg:Selsp_1352	K07455	recT	recombination protein RecT	--	--

[denovogenes]_103404	86.84	6e-168	bpb:bpr_I1923	K14623	dinD	DNA-damage-inducible protein D	--	--

[denovogenes]_103405	100.00	0.0	shq:A0259_07060	K02564	nagB, GNPDA	glucosamine-6-phosphate deaminase	3.5.99.6	Metabolism--Carbohydrate metabolism--Amino sugar and nucleotide sugar metabolism|Metabolism--Global and overview maps--Metabolic pathways

[denovogenes]_103406	88.41	2e-153	lacy:A4V08_00990	K03685	rnc, DROSHA, RNT1	ribonuclease III	3.1.26.3	Genetic Information Processing--Translation--Ribosome biogenesis in eukaryotes|Human Diseases--Cancers: Overview--Proteoglycans in cancer

[denovogenes]_103407	100.00	0.0	ssj:SSON53_00905	K06858	ABC.VB12.S1, btuF	vitamin B12 transport system substrate-binding protein	--	Environmental Information Processing--Membrane transport--ABC transporters

[denovogenes]_103408	50.40	2e-88	ecos:EC958_0828	K00046	idnO	gluconate 5-dehydrogenase	1.1.1.69	--

[denovogenes]_10341	78.92	0.0	afn:Acfer_0454	K00962	pnp, PNPT1	polyribonucleotide nucleotidyltransferase	2.7.7.8	Metabolism--Nucleotide metabolism--Purine metabolism|Metabolism--Nucleotide metabolism--Pyrimidine metabolism|Genetic Information Processing--Folding, sorting and degradation--RNA degradation

[denovogenes]_103411	100.00	0.0	sbc:SbBS512_E1135	K00941	thiD	hydroxymethylpyrimidine/phosphomethylpyrimidine kinase	2.7.1.49 2.7.4.7	Metabolism--Metabolism of cofactors and vitamins--Thiamine metabolism|Metabolism--Global and overview maps--Metabolic pathways

[denovogenes]_103412	96.15	0.0	bxy:BXY_31350	K02528	ksgA	16S rRNA (adenine1518-N6/adenine1519-N6)-dimethyltransferase	2.1.1.182	--

[denovogenes]_103416	54.98	2e-86	bsx:C663_2465	K02483	K02483	two-component system, OmpR family, response regulator	--	--

[denovogenes]_103417	85.77	3e-165	lacy:A4V08_05195	K09817	znuC	zinc transport system ATP-binding protein	3.6.3.-	Environmental Information Processing--Membrane transport--ABC transporters

[denovogenes]_103418	85.48	9e-158	lacy:A4V08_15935	K02564	nagB, GNPDA	glucosamine-6-phosphate deaminase	3.5.99.6	Metabolism--Carbohydrate metabolism--Amino sugar and nucleotide sugar metabolism|Metabolism--Global and overview maps--Metabolic pathways

[denovogenes]_103419	99.62	0.0	fpla:A4U99_07325	K02034	ABC.PE.P1	peptide/nickel transport system permease protein	--	Cellular Processes--Cellular community - prokaryotes--Quorum sensing

[denovogenes]_10342	65.22	0.0	csh:Closa_3448	K02400	flhA	flagellar biosynthesis protein FlhA	--	Cellular Processes--Cell motility--Flagellar assembly

[denovogenes]_103420	98.50	0.0	bth:BT_1902	K00286	proC	pyrroline-5-carboxylate reductase	1.5.1.2	Metabolism--Amino acid metabolism--Arginine and proline metabolism|Metabolism--Global and overview maps--Metabolic pathways|Metabolism--Global and overview maps--Biosynthesis of secondary metabolites|Metabolism--Global and overview maps--Biosynthesis of antibiotics|Metabolism--Global and overview maps--Biosynthesis of amino acids

[denovogenes]_103421	96.24	0.0	cama:F384_00140	K02795	PTS-Man-EIIC, manY	PTS system, mannose-specific IIC component	--	Metabolism--Carbohydrate metabolism--Fructose and mannose metabolism|Metabolism--Carbohydrate metabolism--Amino sugar and nucleotide sugar metabolism|Metabolism--Global and overview maps--Metabolic pathways|Environmental Information Processing--Membrane transport--Phosphotransferase system (PTS)

[denovogenes]_103423	72.73	2e-129	era:ERE_31960	K02030	ABC.PA.S	polar amino acid transport system substrate-binding protein	--	--

[denovogenes]_103424	40.11	3e-34	fsc:FSU_0999	K18475	fliB	lysine-N-methylase	2.1.1.-	--

[denovogenes]_103426	85.61	5e-166	rim:ROI_17080	K00891	E2.7.1.71, aroK, aroL	shikimate kinase	2.7.1.71	Metabolism--Amino acid metabolism--Phenylalanine, tyrosine and tryptophan biosynthesis|Metabolism--Global and overview maps--Metabolic pathways|Metabolism--Global and overview maps--Biosynthesis of secondary metabolites|Metabolism--Global and overview maps--Biosynthesis of antibiotics|Metabolism--Global and overview maps--Biosynthesis of amino acids

[denovogenes]_103427	40.38	3e-42	lhi:JP39_02205	K02042	phnE	phosphonate transport system permease protein	--	Environmental Information Processing--Membrane transport--ABC transporters

[denovogenes]_103428	41.22	3e-59	spe:Spro_1104	K11938	cof	HMP-PP phosphatase	3.6.1.-	--

[denovogenes]_103429	97.37	0.0	btho:Btheta7330_02881	K01991	ABC-2.OM, wza	polysaccharide biosynthesis/export protein	--	Cellular Processes--Cellular community - prokaryotes--Biofilm formation - Escherichia coli

[denovogenes]_103430	65.17	2e-127	hsd:SD1D_2008	K01990	ABC-2.A	ABC-2 type transport system ATP-binding protein	--	--

[denovogenes]_103431	99.62	0.0	cfd:CFNIH1_03225	K02065	mlaF, linL, mkl	phospholipid/cholesterol/gamma-HCH transport system ATP-binding protein	--	Environmental Information Processing--Membrane transport--ABC transporters

[denovogenes]_103432	53.18	9e-97	sdq:SDSE167_2101	K02335	DPO1, polA	DNA polymerase I	2.7.7.7	Metabolism--Nucleotide metabolism--Purine metabolism|Metabolism--Nucleotide metabolism--Pyrimidine metabolism|Metabolism--Global and overview maps--Metabolic pathways|Genetic Information Processing--Replication and repair--DNA replication|Genetic Information Processing--Replication and repair--Base excision repair|Genetic Information Processing--Replication and repair--Nucleotide excision repair|Genetic Information Processing--Replication and repair--Homologous recombination

[denovogenes]_103433	98.87	0.0	cfd:CFNIH1_20825	K02424	fliY	cystine transport system substrate-binding protein	--	Environmental Information Processing--Membrane transport--ABC transporters

[denovogenes]_103434	40.23	5e-59	sfo:Z042_18940	K11938	cof	HMP-PP phosphatase	3.6.1.-	--

[denovogenes]_103435	49.60	2e-73	cno:NT01CX_0219	K07124	K07124	uncharacterized protein	--	--

[denovogenes]_103437	99.25	0.0	cfd:CFNIH1_02675	K03776	aer	aerotaxis receptor	--	Environmental Information Processing--Signal transduction--Two-component system|Cellular Processes--Cell motility--Bacterial chemotaxis

[denovogenes]_103440	77.82	3e-152	ibu:IB211_01812	K21071	pfk, pfp	ATP-dependent phosphofructokinase / diphosphate-dependent phosphofructokinase	2.7.1.11 2.7.1.90	Metabolism--Carbohydrate metabolism--Glycolysis / Gluconeogenesis|Metabolism--Carbohydrate metabolism--Pentose phosphate pathway|Metabolism--Carbohydrate metabolism--Fructose and mannose metabolism|Metabolism--Carbohydrate metabolism--Galactose metabolism|Metabolism--Energy metabolism--Methane metabolism|Metabolism--Global and overview maps--Metabolic pathways|Metabolism--Global and overview maps--Biosynthesis of secondary metabolites|Metabolism--Global and overview maps--Microbial metabolism in diverse environments|Metabolism--Global and overview maps--Biosynthesis of antibiotics

[denovogenes]_103441	100.00	0.0	elx:CDCO157_0003	K00872	thrB1	homoserine kinase	2.7.1.39	Metabolism--Amino acid metabolism--Glycine, serine and threonine metabolism|Metabolism--Global and overview maps--Metabolic pathways|Metabolism--Global and overview maps--Biosynthesis of secondary metabolites|Metabolism--Global and overview maps--Microbial metabolism in diverse environments|Metabolism--Global and overview maps--Biosynthesis of amino acids

[denovogenes]_103442	73.52	1e-137	rum:CK1_34280	K03785	aroD	3-dehydroquinate dehydratase I	4.2.1.10	Metabolism--Amino acid metabolism--Phenylalanine, tyrosine and tryptophan biosynthesis|Metabolism--Global and overview maps--Metabolic pathways|Metabolism--Global and overview maps--Biosynthesis of secondary metabolites|Metabolism--Global and overview maps--Biosynthesis of antibiotics|Metabolism--Global and overview maps--Biosynthesis of amino acids

[denovogenes]_103445	43.78	1e-27	mif:Metin_0106	K03406	mcp	methyl-accepting chemotaxis protein	--	Environmental Information Processing--Signal transduction--Two-component system|Cellular Processes--Cell motility--Bacterial chemotaxis

[denovogenes]_103446	98.87	0.0	lacy:A4V08_31215	K03496	parA, soj	chromosome partitioning protein	--	--

[denovogenes]_103447	100.00	0.0	btho:Btheta7330_02881	K01991	ABC-2.OM, wza	polysaccharide biosynthesis/export protein	--	Cellular Processes--Cellular community - prokaryotes--Biofilm formation - Escherichia coli

[denovogenes]_10345	94.20	0.0	bxy:BXY_18740	K07037	K07037	uncharacterized protein	--	--

[denovogenes]_103450	95.49	1e-179	cfd:CFNIH1_15005	K08344	scsB	suppressor for copper-sensitivity B	--	--

[denovogenes]_103451	48.33	1e-73	saz:Sama_1319	K03309	TC.DAACS	dicarboxylate/amino acid:cation (Na+ or H+) symporter, DAACS family	--	--

[denovogenes]_103452	92.22	2e-170	bvu:BVU_1338	K00930	argB	acetylglutamate kinase	2.7.2.8	Metabolism--Amino acid metabolism--Arginine biosynthesis|Metabolism--Global and overview maps--Metabolic pathways|Metabolism--Global and overview maps--Biosynthesis of secondary metabolites|Metabolism--Global and overview maps--Biosynthesis of antibiotics|Metabolism--Global and overview maps--2-Oxocarboxylic acid metabolism|Metabolism--Global and overview maps--Biosynthesis of amino acids

[denovogenes]_103455	97.00	0.0	bcel:BcellWH2_04132	K01923	purC	phosphoribosylaminoimidazole-succinocarboxamide synthase	6.3.2.6	Metabolism--Nucleotide metabolism--Purine metabolism|Metabolism--Global and overview maps--Metabolic pathways|Metabolism--Global and overview maps--Biosynthesis of secondary metabolites|Metabolism--Global and overview maps--Biosynthesis of antibiotics

[denovogenes]_103456	100.00	0.0	pdi:BDI_1915	K00652	bioF	8-amino-7-oxononanoate synthase	2.3.1.47	Metabolism--Metabolism of cofactors and vitamins--Biotin metabolism|Metabolism--Global and overview maps--Metabolic pathways

[denovogenes]_103457	100.00	0.0	ssn:SSON_1027	K09023	rutD	aminoacrylate hydrolase	3.5.1.-	Metabolism--Nucleotide metabolism--Pyrimidine metabolism|Metabolism--Global and overview maps--Metabolic pathways

[denovogenes]_103458	54.02	1e-95	fpla:A4U99_13455	K02115	ATPF1G, atpG	F-type H+-transporting ATPase subunit gamma	--	Metabolism--Energy metabolism--Oxidative phosphorylation|Metabolism--Energy metabolism--Photosynthesis|Metabolism--Global and overview maps--Metabolic pathways

[denovogenes]_10346	67.17	0.0	csh:Closa_3423	K03407	cheA	two-component system, chemotaxis family, sensor kinase CheA	2.7.13.3	Environmental Information Processing--Signal transduction--Two-component system|Cellular Processes--Cell motility--Bacterial chemotaxis

[denovogenes]_103460	70.11	4e-137	bcel:BcellWH2_01376	K01448	E3.5.1.28B, amiA, amiB, amiC	N-acetylmuramoyl-L-alanine amidase	3.5.1.28	Human Diseases--Drug resistance: Antimicrobial--Cationic antimicrobial peptide (CAMP) resistance

[denovogenes]_103461	68.05	3e-136	cbh:CLC_2670	K19689	ampS, pepS, ampT	aminopeptidase	3.4.11.-	--

[denovogenes]_103463	79.40	7e-145	bhl:Bache_3221	K11749	rseP	regulator of sigma E protease	3.4.24.-	Cellular Processes--Cellular community - prokaryotes--Quorum sensing|Cellular Processes--Cell growth and death--Cell cycle - Caulobacter

[denovogenes]_103465	49.03	1e-77	pvu:PHAVU_010G142600g	K14664	ILR1	IAA-amino acid hydrolase	3.5.1.-	--

[denovogenes]_103469	68.03	2e-136	cle:Clole_2223	K03778	ldhA	D-lactate dehydrogenase	1.1.1.28	Metabolism--Carbohydrate metabolism--Pyruvate metabolism|Metabolism--Global and overview maps--Microbial metabolism in diverse environments

[denovogenes]_10347	90.07	0.0	lacy:A4V08_12320	K02355	fusA, GFM, EFG	elongation factor G	--	--

[denovogenes]_103472	77.18	3e-81	arh:AHiyo8_50560	K03043	rpoB	DNA-directed RNA polymerase subunit beta	2.7.7.6	Metabolism--Nucleotide metabolism--Purine metabolism|Metabolism--Nucleotide metabolism--Pyrimidine metabolism|Metabolism--Global and overview maps--Metabolic pathways|Genetic Information Processing--Transcription--RNA polymerase

[denovogenes]_103476	69.81	1e-128	hsd:SD1D_1974	K02071	metN	D-methionine transport system ATP-binding protein	--	Environmental Information Processing--Membrane transport--ABC transporters

[denovogenes]_103478	85.45	3e-154	bfi:CIY_11680	K00789	metK	S-adenosylmethionine synthetase	2.5.1.6	Metabolism--Amino acid metabolism--Cysteine and methionine metabolism|Metabolism--Global and overview maps--Metabolic pathways|Metabolism--Global and overview maps--Biosynthesis of secondary metabolites|Metabolism--Global and overview maps--Biosynthesis of amino acids

[denovogenes]_103479	90.64	3e-177	cle:Clole_1149	K01876	DARS, aspS	aspartyl-tRNA synthetase	6.1.1.12	Genetic Information Processing--Translation--Aminoacyl-tRNA biosynthesis

[denovogenes]_103480	58.95	8e-92	bprs:CK3_15540	K06409	spoVB	stage V sporulation protein B	--	--

[denovogenes]_103481	60.70	2e-103	slp:Slip_0605	K00602	purH	phosphoribosylaminoimidazolecarboxamide formyltransferase / IMP cyclohydrolase	2.1.2.3 3.5.4.10	Metabolism--Nucleotide metabolism--Purine metabolism|Metabolism--Metabolism of cofactors and vitamins--One carbon pool by folate|Metabolism--Global and overview maps--Metabolic pathways|Metabolism--Global and overview maps--Biosynthesis of secondary metabolites|Metabolism--Global and overview maps--Biosynthesis of antibiotics|Human Diseases--Drug resistance: Antineoplastic--Antifolate resistance

[denovogenes]_103482	72.28	2e-136	lacy:A4V08_05850	K01753	dsdA	D-serine dehydratase	4.3.1.18	Metabolism--Amino acid metabolism--Glycine, serine and threonine metabolism

[denovogenes]_103485	93.63	0.0	kpu:KP1_2270	K03781	katE, CAT, catB, srpA	catalase	1.11.1.6	Metabolism--Amino acid metabolism--Tryptophan metabolism|Metabolism--Carbohydrate metabolism--Glyoxylate and dicarboxylate metabolism|Metabolism--Global and overview maps--Biosynthesis of secondary metabolites|Metabolism--Global and overview maps--Biosynthesis of antibiotics|Metabolism--Global and overview maps--Carbon metabolism|Environmental Information Processing--Signal transduction--MAPK signaling pathway - yeast|Environmental Information Processing--Signal transduction--MAPK signaling pathway - plant|Environmental Information Processing--Signal transduction--FoxO signaling pathway|Cellular Processes--Transport and catabolism--Peroxisome|Organismal Systems--Aging--Longevity regulating pathway|Organismal Systems--Aging--Longevity regulating pathway - worm|Organismal Systems--Aging--Longevity regulating pathway - multiple species|Human Diseases--Neurodegenerative diseases--Amyotrophic lateral sclerosis (ALS)

[denovogenes]_103486	67.18	1e-108	cdg:CDBI1_12875	K03531	ftsZ	cell division protein FtsZ	--	Cellular Processes--Cell growth and death--Cell cycle - Caulobacter

[denovogenes]_103488	45.71	1e-48	pri:PRIO_4803	K05989	ramA	alpha-L-rhamnosidase	3.2.1.40	--

[denovogenes]_10349	96.82	0.0	bth:BT_2686	K03296	TC.HAE1	hydrophobic/amphiphilic exporter-1 (mainly G- bacteria), HAE1 family	--	--

[denovogenes]_103490	100.00	0.0	esl:O3K_18945	K10975	allP	allantoin permease	--	--

[denovogenes]_103492	68.05	1e-131	cbt:CLH_2741	K03547	sbcD	exonuclease SbcD	--	--

[denovogenes]_103498	66.79	6e-114	pfre:RM25_1973	K12510	tadB	tight adherence protein B	--	--

[denovogenes]_103499	66.67	1e-113	pfre:RM25_1206	K07670	mtrA	two-component system, OmpR family, response regulator MtrA	--	Environmental Information Processing--Signal transduction--Two-component system

[denovogenes]_1035	99.92	0.0	efa:EF3238	K03043	rpoB	DNA-directed RNA polymerase subunit beta	2.7.7.6	Metabolism--Nucleotide metabolism--Purine metabolism|Metabolism--Nucleotide metabolism--Pyrimidine metabolism|Metabolism--Global and overview maps--Metabolic pathways|Genetic Information Processing--Transcription--RNA polymerase

[denovogenes]_10350	70.29	0.0	cpro:CPRO_28390	K02400	flhA	flagellar biosynthesis protein FlhA	--	Cellular Processes--Cell motility--Flagellar assembly

[denovogenes]_103500	50.36	1e-38	pmf:P9303_05381	K00975	glgC	glucose-1-phosphate adenylyltransferase	2.7.7.27	Metabolism--Carbohydrate metabolism--Starch and sucrose metabolism|Metabolism--Carbohydrate metabolism--Amino sugar and nucleotide sugar metabolism|Metabolism--Global and overview maps--Metabolic pathways|Metabolism--Global and overview maps--Biosynthesis of secondary metabolites|Cellular Processes--Cellular community - prokaryotes--Biofilm formation - Escherichia coli

[denovogenes]_103501	79.70	1e-70	ckr:CKR_2358	K07741	antB	anti-repressor protein	--	--

[denovogenes]_103502	83.66	4e-139	pfre:RM25_1001	K01609	trpC	indole-3-glycerol phosphate synthase	4.1.1.48	Metabolism--Amino acid metabolism--Phenylalanine, tyrosine and tryptophan biosynthesis|Metabolism--Global and overview maps--Metabolic pathways|Metabolism--Global and overview maps--Biosynthesis of secondary metabolites|Metabolism--Global and overview maps--Biosynthesis of antibiotics|Metabolism--Global and overview maps--Biosynthesis of amino acids

[denovogenes]_103504	88.72	3e-173	btho:Btheta7330_02881	K01991	ABC-2.OM, wza	polysaccharide biosynthesis/export protein	--	Cellular Processes--Cellular community - prokaryotes--Biofilm formation - Escherichia coli

[denovogenes]_103505	45.08	8e-18	ham:HALO2299	K03286	TC.OOP	OmpA-OmpF porin, OOP family	--	--

[denovogenes]_103506	99.25	0.0	bth:BT_0314	K02687	prmA	ribosomal protein L11 methyltransferase	2.1.1.-	--

[denovogenes]_103508	87.92	5e-170	bcel:BcellWH2_05263	K11071	potB	spermidine/putrescine transport system permease protein	--	Environmental Information Processing--Membrane transport--ABC transporters

[denovogenes]_103509	87.59	9e-169	bfs:BF9343_0067	K06153	bacA	undecaprenyl-diphosphatase	3.6.1.27	Metabolism--Glycan biosynthesis and metabolism--Peptidoglycan biosynthesis

[denovogenes]_10351	44.70	0.0	eel:EUBELI_00110	K03217	yidC, spoIIIJ, OXA1, ccfA	YidC/Oxa1 family membrane protein insertase	--	Cellular Processes--Cellular community - prokaryotes--Quorum sensing|Genetic Information Processing--Folding, sorting and degradation--Protein export|Environmental Information Processing--Membrane transport--Bacterial secretion system

[denovogenes]_103513	65.28	2e-129	sph:MGAS10270_Spy1168	K01091	gph	phosphoglycolate phosphatase	3.1.3.18	Metabolism--Carbohydrate metabolism--Glyoxylate and dicarboxylate metabolism|Metabolism--Global and overview maps--Metabolic pathways|Metabolism--Global and overview maps--Biosynthesis of secondary metabolites|Metabolism--Global and overview maps--Biosynthesis of antibiotics

[denovogenes]_103517	100.00	0.0	ene:ENT_08520	K02020	modA	molybdate transport system substrate-binding protein	--	Environmental Information Processing--Membrane transport--ABC transporters

[denovogenes]_103518	100.00	0.0	paeo:M801_5114	K02030	ABC.PA.S	polar amino acid transport system substrate-binding protein	--	--

[denovogenes]_103519	99.25	0.0	paeo:M801_5129	K01479	hutG	formiminoglutamase	3.5.3.8	Metabolism--Amino acid metabolism--Histidine metabolism|Metabolism--Global and overview maps--Metabolic pathways

[denovogenes]_10352	99.57	0.0	bxy:BXY_18740	K07037	K07037	uncharacterized protein	--	--

[denovogenes]_103520	99.24	0.0	paec:M802_4804	K07346	fimC	fimbrial chaperone protein	--	--

[denovogenes]_103523	40.94	1e-14	psb:Psyr_1041	K07506	K07506	AraC family transcriptional regulator	--	--

[denovogenes]_103524	69.37	8e-135	cso:CLS_17370	K03437	spoU	RNA methyltransferase, TrmH family	--	--

[denovogenes]_103525	97.74	0.0	cfd:CFNIH1_10480	K06858	ABC.VB12.S1, btuF	vitamin B12 transport system substrate-binding protein	--	Environmental Information Processing--Membrane transport--ABC transporters

[denovogenes]_103526	98.12	0.0	bfs:BF9343_0067	K06153	bacA	undecaprenyl-diphosphatase	3.6.1.27	Metabolism--Glycan biosynthesis and metabolism--Peptidoglycan biosynthesis

[denovogenes]_103529	98.87	0.0	cfd:CFNIH1_21810	K00941	thiD	hydroxymethylpyrimidine/phosphomethylpyrimidine kinase	2.7.1.49 2.7.4.7	Metabolism--Metabolism of cofactors and vitamins--Thiamine metabolism|Metabolism--Global and overview maps--Metabolic pathways

[denovogenes]_10353	63.50	0.0	rch:RUM_12900	K03168	topA	DNA topoisomerase I	5.99.1.2	--

[denovogenes]_103530	44.57	2e-72	dfi:AXF13_03025	K00286	proC	pyrroline-5-carboxylate reductase	1.5.1.2	Metabolism--Amino acid metabolism--Arginine and proline metabolism|Metabolism--Global and overview maps--Metabolic pathways|Metabolism--Global and overview maps--Biosynthesis of secondary metabolites|Metabolism--Global and overview maps--Biosynthesis of antibiotics|Metabolism--Global and overview maps--Biosynthesis of amino acids

[denovogenes]_103531	55.95	3e-95	pft:JBW_01264	K02172	blaR1	bla regulator protein blaR1	--	Human Diseases--Drug resistance: Antimicrobial--beta-Lactam resistance

[denovogenes]_103532	95.06	0.0	lacy:A4V08_27000	K07720	yesN	two-component system, response regulator YesN	--	Environmental Information Processing--Signal transduction--Two-component system

[denovogenes]_103536	100.00	0.0	paeo:M801_4146	K03366	butA, budC	meso-butanediol dehydrogenase / (S,S)-butanediol dehydrogenase / diacetyl reductase	1.1.1.- 1.1.1.76 1.1.1.304	Metabolism--Carbohydrate metabolism--Butanoate metabolism

[denovogenes]_10354	73.88	0.0	ibu:IB211_00590	K02355	fusA, GFM, EFG	elongation factor G	--	--

[denovogenes]_103543	98.50	0.0	fpla:A4U99_05115	K04070	pflX	putative pyruvate formate lyase activating enzyme	1.97.1.4	--

[denovogenes]_103544	91.67	5e-178	bfs:BF9343_0777	K03118	tatC	sec-independent protein translocase protein TatC	--	Genetic Information Processing--Folding, sorting and degradation--Protein export|Environmental Information Processing--Membrane transport--Bacterial secretion system

[denovogenes]_103545	100.00	0.0	paec:M802_4891	K00606	panB	3-methyl-2-oxobutanoate hydroxymethyltransferase	2.1.2.11	Metabolism--Metabolism of cofactors and vitamins--Pantothenate and CoA biosynthesis|Metabolism--Global and overview maps--Metabolic pathways|Metabolism--Global and overview maps--Biosynthesis of secondary metabolites

[denovogenes]_103546	100.00	0.0	cfd:CFNIH1_20825	K02424	fliY	cystine transport system substrate-binding protein	--	Environmental Information Processing--Membrane transport--ABC transporters

[denovogenes]_103547	98.87	0.0	pdi:BDI_2584	K02528	ksgA	16S rRNA (adenine1518-N6/adenine1519-N6)-dimethyltransferase	2.1.1.182	--

[denovogenes]_103548	100.00	0.0	paeo:M801_350	K13292	lgt, umpA	phosphatidylglycerol:prolipoprotein diacylglycerol transferase	2.-.-.-	--

[denovogenes]_10355	70.22	0.0	cpy:Cphy_1909	K07003	K07003	uncharacterized protein	--	--

[denovogenes]_103550	85.71	9e-173	pdi:BDI_1320	K01629	rhaD	rhamnulose-1-phosphate aldolase	4.1.2.19	Metabolism--Carbohydrate metabolism--Pentose and glucuronate interconversions|Metabolism--Carbohydrate metabolism--Fructose and mannose metabolism|Metabolism--Global and overview maps--Microbial metabolism in diverse environments

[denovogenes]_103551	72.56	2e-149	dpg:DESPIGER_0261	K00175	korB, oorB, oforB	2-oxoglutarate/2-oxoacid ferredoxin oxidoreductase subunit beta	1.2.7.3 1.2.7.11	Metabolism--Carbohydrate metabolism--Glycolysis / Gluconeogenesis|Metabolism--Carbohydrate metabolism--Citrate cycle (TCA cycle)|Metabolism--Carbohydrate metabolism--Pyruvate metabolism|Metabolism--Carbohydrate metabolism--Butanoate metabolism|Metabolism--Energy metabolism--Carbon fixation pathways in prokaryotes|Metabolism--Global and overview maps--Metabolic pathways|Metabolism--Global and overview maps--Microbial metabolism in diverse environments|Metabolism--Global and overview maps--Biosynthesis of antibiotics|Metabolism--Global and overview maps--Carbon metabolism

[denovogenes]_103552	98.12	0.0	lacy:A4V08_01945	K00981	E2.7.7.41, CDS1, CDS2, cdsA	phosphatidate cytidylyltransferase	2.7.7.41	Metabolism--Lipid metabolism--Glycerophospholipid metabolism|Metabolism--Global and overview maps--Metabolic pathways|Metabolism--Global and overview maps--Biosynthesis of secondary metabolites|Environmental Information Processing--Signal transduction--Phosphatidylinositol signaling system

[denovogenes]_103554	100.00	0.0	cfd:CFNIH1_07680	K13641	iclR	IclR family transcriptional regulator, acetate operon repressor	--	--

[denovogenes]_103555	100.00	0.0	paeo:M801_3260	K07221	oprO_P	phosphate-selective porin OprO and OprP	--	--

[denovogenes]_10356	59.30	0.0	pgm:PGRAT_01265	K05349	bglX	beta-glucosidase	3.2.1.21	Metabolism--Metabolism of other amino acids--Cyanoamino acid metabolism|Metabolism--Carbohydrate metabolism--Starch and sucrose metabolism|Metabolism--Biosynthesis of other secondary metabolites--Phenylpropanoid biosynthesis|Metabolism--Global and overview maps--Metabolic pathways|Metabolism--Global and overview maps--Biosynthesis of secondary metabolites

[denovogenes]_103561	43.45	4e-70	lsl:LSL_1486	K01181	E3.2.1.8, xynA	endo-1,4-beta-xylanase	3.2.1.8	--

[denovogenes]_103563	99.25	0.0	pdi:BDI_2407	K06153	bacA	undecaprenyl-diphosphatase	3.6.1.27	Metabolism--Glycan biosynthesis and metabolism--Peptidoglycan biosynthesis

[denovogenes]_103565	79.70	5e-158	csh:Closa_1268	K01990	ABC-2.A	ABC-2 type transport system ATP-binding protein	--	--

[denovogenes]_103567	49.60	1e-82	awo:Awo_c22370	K02050	ABC.SN.P	NitT/TauT family transport system permease protein	--	--

[denovogenes]_103568	96.99	0.0	bfg:BF638R_1404	K07387	K07387	putative metalloprotease	3.4.24.-	--

[denovogenes]_103569	60.75	3e-121	cpf:CPF_1788	K16951	--	--	--	Metabolism--Energy metabolism--Sulfur metabolism|Metabolism--Global and overview maps--Microbial metabolism in diverse environments

[denovogenes]_10357	82.05	0.0	bdh:GV66_16625	K07001	K07001	NTE family protein	--	--

[denovogenes]_103571	78.87	8e-144	cbz:Cbs_0771	K10119	msmG	raffinose/stachyose/melibiose transport system permease protein	--	Environmental Information Processing--Membrane transport--ABC transporters

[denovogenes]_103572	95.86	0.0	lacy:A4V08_15460	K02119	ATPVC, ntpC, atpC	V/A-type H+/Na+-transporting ATPase subunit C	--	Metabolism--Energy metabolism--Oxidative phosphorylation|Metabolism--Global and overview maps--Metabolic pathways

[denovogenes]_103573	85.38	2e-165	csh:Closa_3732	K16785	ecfT	energy-coupling factor transport system permease protein	--	Environmental Information Processing--Membrane transport--ABC transporters

[denovogenes]_103574	41.70	1e-58	dgi:Desgi_2715	K01715	crt	enoyl-CoA hydratase	4.2.1.17	Metabolism--Carbohydrate metabolism--Butanoate metabolism|Metabolism--Global and overview maps--Carbon metabolism

[denovogenes]_103575	92.42	0.0	csh:Closa_0399	K00973	E2.7.7.24, rfbA, rffH	glucose-1-phosphate thymidylyltransferase	2.7.7.24	Metabolism--Biosynthesis of other secondary metabolites--Streptomycin biosynthesis|Metabolism--Metabolism of terpenoids and polyketides--Polyketide sugar unit biosynthesis|Metabolism--Biosynthesis of other secondary metabolites--Acarbose and validamycin biosynthesis|Metabolism--Global and overview maps--Biosynthesis of antibiotics

[denovogenes]_103577	52.36	2e-79	str:Sterm_3466	K15634	gpmB	probable phosphoglycerate mutase	5.4.2.12	Metabolism--Carbohydrate metabolism--Glycolysis / Gluconeogenesis|Metabolism--Amino acid metabolism--Glycine, serine and threonine metabolism|Metabolism--Energy metabolism--Methane metabolism|Metabolism--Global and overview maps--Metabolic pathways|Metabolism--Global and overview maps--Biosynthesis of secondary metabolites|Metabolism--Global and overview maps--Microbial metabolism in diverse environments|Metabolism--Global and overview maps--Biosynthesis of antibiotics|Metabolism--Global and overview maps--Carbon metabolism|Metabolism--Global and overview maps--Biosynthesis of amino acids

[denovogenes]_103579	89.10	2e-173	bfs:BF9343_0067	K06153	bacA	undecaprenyl-diphosphatase	3.6.1.27	Metabolism--Glycan biosynthesis and metabolism--Peptidoglycan biosynthesis

[denovogenes]_103580	94.74	3e-176	lacy:A4V08_24780	K07010	K07010	putative glutamine amidotransferase	--	--

[denovogenes]_103586	83.15	6e-166	bcel:BcellWH2_00693	K01119	cpdB	2',3'-cyclic-nucleotide 2'-phosphodiesterase / 3'-nucleotidase	3.1.4.16 3.1.3.6	Metabolism--Nucleotide metabolism--Purine metabolism|Metabolism--Nucleotide metabolism--Pyrimidine metabolism

[denovogenes]_103587	72.18	4e-139	lacy:A4V08_31185	K02392	flgG	flagellar basal-body rod protein FlgG	--	Cellular Processes--Cell motility--Flagellar assembly

[denovogenes]_103588	90.23	9e-177	csh:Closa_2360	K17074	lysX2	putative lysine transport system permease protein	--	Environmental Information Processing--Membrane transport--ABC transporters

[denovogenes]_103589	65.41	7e-127	afd:Alfi_3278	K00969	nadD	nicotinate-nucleotide adenylyltransferase	2.7.7.18	Metabolism--Metabolism of cofactors and vitamins--Nicotinate and nicotinamide metabolism|Metabolism--Global and overview maps--Metabolic pathways

[denovogenes]_10359	84.52	0.0	pfre:RM25_2098	K12954	ctpG	cation-transporting ATPase G	3.6.3.-	--

[denovogenes]_103596	47.35	3e-81	ccb:Clocel_0470	K01992	ABC-2.P	ABC-2 type transport system permease protein	--	--

[denovogenes]_103597	99.62	0.0	sbc:SbBS512_E1048	K02424	fliY	cystine transport system substrate-binding protein	--	Environmental Information Processing--Membrane transport--ABC transporters

[denovogenes]_10360	76.52	0.0	pfr:PFREUD_13050	K03722	dinG	ATP-dependent DNA helicase DinG	3.6.4.12	--

[denovogenes]_103600	88.26	4e-175	bsa:Bacsa_2429	K00046	idnO	gluconate 5-dehydrogenase	1.1.1.69	--

[denovogenes]_103601	73.76	3e-135	cgo:Corgl_0328	K00097	pdxA	4-hydroxythreonine-4-phosphate dehydrogenase	1.1.1.262	Metabolism--Metabolism of cofactors and vitamins--Vitamin B6 metabolism|Metabolism--Global and overview maps--Metabolic pathways

[denovogenes]_103602	73.81	8e-120	cpor:BED41_12075	K02015	ABC.FEV.P	iron complex transport system permease protein	--	Environmental Information Processing--Membrane transport--ABC transporters

[denovogenes]_103603	99.25	0.0	cfd:CFNIH1_04875	K05814	ugpA	sn-glycerol 3-phosphate transport system permease protein	--	Environmental Information Processing--Membrane transport--ABC transporters

[denovogenes]_103605	99.62	0.0	paeo:M801_1855	K03544	clpX, CLPX	ATP-dependent Clp protease ATP-binding subunit ClpX	--	Cellular Processes--Cell growth and death--Cell cycle - Caulobacter

[denovogenes]_103607	79.40	2e-155	ash:AL1_25180	K04075	tilS, mesJ	tRNA(Ile)-lysidine synthase	6.3.4.19	--

[denovogenes]_10361	82.59	0.0	pdi:BDI_3793	K04084	dsbD	thiol:disulfide interchange protein DsbD	1.8.1.8	--

[denovogenes]_103611	99.63	0.0	bbre:B12L_1386	K00674	dapD	2,3,4,5-tetrahydropyridine-2-carboxylate N-succinyltransferase	2.3.1.117	Metabolism--Amino acid metabolism--Lysine biosynthesis|Metabolism--Global and overview maps--Metabolic pathways|Metabolism--Global and overview maps--Microbial metabolism in diverse environments|Metabolism--Global and overview maps--Biosynthesis of amino acids

[denovogenes]_103612	41.75	2e-44	bacb:OY17_19085	K00903	epsB	protein-tyrosine kinase	2.7.10.2	--

[denovogenes]_103613	58.04	1e-103	plv:ERIC2_c30570	K09704	K09704	uncharacterized protein	--	--

[denovogenes]_103614	95.11	0.0	cfd:CFNIH1_10480	K06858	ABC.VB12.S1, btuF	vitamin B12 transport system substrate-binding protein	--	Environmental Information Processing--Membrane transport--ABC transporters

[denovogenes]_103615	63.12	7e-110	cgo:Corgl_0991	K06442	tlyA	23S rRNA (cytidine1920-2'-O)/16S rRNA (cytidine1409-2'-O)-methyltransferase	2.1.1.226 2.1.1.227	--

[denovogenes]_103616	51.70	1e-89	erb:A4V01_00410	K02796	PTS-Man-EIID, manZ	PTS system, mannose-specific IID component	--	Metabolism--Carbohydrate metabolism--Fructose and mannose metabolism|Metabolism--Carbohydrate metabolism--Amino sugar and nucleotide sugar metabolism|Metabolism--Global and overview maps--Metabolic pathways|Environmental Information Processing--Membrane transport--Phosphotransferase system (PTS)

[denovogenes]_103617	100.00	0.0	bbrd:BBBR_0708	K05794	terC	tellurite resistance protein TerC	--	--

[denovogenes]_103618	40.78	4e-29	kpt:VK055_0696	K21088	ydaM	diguanylate cyclase	2.7.7.65	Cellular Processes--Cellular community - prokaryotes--Biofilm formation - Escherichia coli

[denovogenes]_103619	83.46	2e-154	csh:Closa_1857	K00981	E2.7.7.41, CDS1, CDS2, cdsA	phosphatidate cytidylyltransferase	2.7.7.41	Metabolism--Lipid metabolism--Glycerophospholipid metabolism|Metabolism--Global and overview maps--Metabolic pathways|Metabolism--Global and overview maps--Biosynthesis of secondary metabolites|Environmental Information Processing--Signal transduction--Phosphatidylinositol signaling system

[denovogenes]_10362	100.00	0.0	btho:Btheta7330_00329	K07037	K07037	uncharacterized protein	--	--

[denovogenes]_103621	78.65	9e-142	cgo:Corgl_1159	K01938	fhs	formate--tetrahydrofolate ligase	6.3.4.3	Metabolism--Metabolism of cofactors and vitamins--One carbon pool by folate|Metabolism--Energy metabolism--Carbon fixation pathways in prokaryotes|Metabolism--Global and overview maps--Metabolic pathways|Metabolism--Global and overview maps--Microbial metabolism in diverse environments|Metabolism--Global and overview maps--Carbon metabolism

[denovogenes]_103624	75.66	2e-139	rum:CK1_03190	K03205	virD4, lvhD4	type IV secretion system protein VirD4	--	Environmental Information Processing--Membrane transport--Bacterial secretion system

[denovogenes]_103626	68.16	8e-126	pdi:BDI_0814	K02224	cobB-cbiA	cobyrinic acid a,c-diamide synthase	6.3.5.9 6.3.5.11	Metabolism--Metabolism of cofactors and vitamins--Porphyrin and chlorophyll metabolism|Metabolism--Global and overview maps--Metabolic pathways

[denovogenes]_103631	99.25	0.0	pdi:BDI_1664	K01785	galM, GALM	aldose 1-epimerase	5.1.3.3	Metabolism--Carbohydrate metabolism--Glycolysis / Gluconeogenesis|Metabolism--Carbohydrate metabolism--Galactose metabolism|Metabolism--Global and overview maps--Metabolic pathways|Metabolism--Global and overview maps--Biosynthesis of secondary metabolites|Metabolism--Global and overview maps--Microbial metabolism in diverse environments|Metabolism--Global and overview maps--Biosynthesis of antibiotics

[denovogenes]_103632	86.89	6e-178	pdi:BDI_1693	K06881	nrnA	bifunctional oligoribonuclease and PAP phosphatase NrnA	3.1.3.7 3.1.13.3	Metabolism--Energy metabolism--Sulfur metabolism|Metabolism--Global and overview maps--Metabolic pathways|Metabolism--Global and overview maps--Microbial metabolism in diverse environments

[denovogenes]_103634	48.84	7e-73	fpr:FP2_21840	K03497	parB, spo0J	chromosome partitioning protein, ParB family	--	--

[denovogenes]_103635	97.34	0.0	boa:Bovatus_04497	K06872	K06872	uncharacterized protein	--	--

[denovogenes]_103636	77.44	2e-158	eac:EAL2_808p03090	K00385	asrC	anaerobic sulfite reductase subunit C	--	Metabolism--Energy metabolism--Sulfur metabolism|Metabolism--Global and overview maps--Microbial metabolism in diverse environments

[denovogenes]_103637	88.85	4e-167	csh:Closa_0057	K01810	GPI, pgi	glucose-6-phosphate isomerase	5.3.1.9	Metabolism--Carbohydrate metabolism--Glycolysis / Gluconeogenesis|Metabolism--Carbohydrate metabolism--Pentose phosphate pathway|Metabolism--Carbohydrate metabolism--Starch and sucrose metabolism|Metabolism--Carbohydrate metabolism--Amino sugar and nucleotide sugar metabolism|Metabolism--Global and overview maps--Metabolic pathways|Metabolism--Global and overview maps--Biosynthesis of secondary metabolites|Metabolism--Global and overview maps--Microbial metabolism in diverse environments|Metabolism--Global and overview maps--Biosynthesis of antibiotics|Metabolism--Global and overview maps--Carbon metabolism

[denovogenes]_103639	90.98	4e-178	bhl:Bache_3189	K06926	K06926	uncharacterized protein	--	--

[denovogenes]_10364	82.65	0.0	lir:LAW_00515	K15987	hppA	K(+)-stimulated pyrophosphate-energized sodium pump	3.6.1.1	Metabolism--Energy metabolism--Oxidative phosphorylation

[denovogenes]_103640	49.07	1e-80	fjo:Fjoh_3113	K01209	abfA	alpha-N-arabinofuranosidase	3.2.1.55	Metabolism--Carbohydrate metabolism--Amino sugar and nucleotide sugar metabolism

[denovogenes]_103642	71.08	2e-126	cbut:ATN24_15425	K02031	ABC.PE.A	peptide/nickel transport system ATP-binding protein	--	Cellular Processes--Cellular community - prokaryotes--Quorum sensing

[denovogenes]_103646	71.70	2e-138	byl:A4V09_18600	K18345	vanSB, vanS, vanSD	two-component system, OmpR family, sensor histidine kinase VanS	2.7.13.3	Human Diseases--Drug resistance: Antimicrobial--Vancomycin resistance|Environmental Information Processing--Signal transduction--Two-component system

[denovogenes]_103649	71.71	3e-129	erb:A4V01_20385	K00336	nuoG	NADH-quinone oxidoreductase subunit G	1.6.5.3	Metabolism--Energy metabolism--Oxidative phosphorylation|Metabolism--Global and overview maps--Metabolic pathways

[denovogenes]_10365	100.00	0.0	btho:Btheta7330_03031	K00615	E2.2.1.1, tktA, tktB	transketolase	2.2.1.1	Metabolism--Carbohydrate metabolism--Pentose phosphate pathway|Metabolism--Energy metabolism--Carbon fixation in photosynthetic organisms|Metabolism--Metabolism of terpenoids and polyketides--Biosynthesis of ansamycins|Metabolism--Global and overview maps--Metabolic pathways|Metabolism--Global and overview maps--Biosynthesis of secondary metabolites|Metabolism--Global and overview maps--Microbial metabolism in diverse environments|Metabolism--Global and overview maps--Biosynthesis of antibiotics|Metabolism--Global and overview maps--Carbon metabolism|Metabolism--Global and overview maps--Biosynthesis of amino acids

[denovogenes]_103650	77.15	8e-122	stb:SGPB_0700	K01546	kdpA	K+-transporting ATPase ATPase A chain	3.6.3.12	Environmental Information Processing--Signal transduction--Two-component system

[denovogenes]_103651	55.81	4e-114	pff:PFLUOLIPICF720905	K00527	nrdD	ribonucleoside-triphosphate reductase	1.17.4.2	Metabolism--Nucleotide metabolism--Purine metabolism|Metabolism--Nucleotide metabolism--Pyrimidine metabolism|Metabolism--Global and overview maps--Metabolic pathways

[denovogenes]_103653	46.29	6e-72	mjl:Mjls_1438	K08100	E1.3.3.5	bilirubin oxidase	1.3.3.5	Metabolism--Metabolism of cofactors and vitamins--Porphyrin and chlorophyll metabolism|Metabolism--Global and overview maps--Biosynthesis of secondary metabolites

[denovogenes]_103657	57.09	6e-89	ols:Olsu_0998	K02316	dnaG	DNA primase	2.7.7.-	Genetic Information Processing--Replication and repair--DNA replication

[denovogenes]_103659	100.00	0.0	cif:AL515_20240	K06120	dhaB	glycerol dehydratase large subunit	4.2.1.30	Metabolism--Lipid metabolism--Glycerolipid metabolism

[denovogenes]_10366	53.27	0.0	eic:NT01EI_1050	K00219	fadH	2,4-dienoyl-CoA reductase (NADPH2)	1.3.1.34	--

[denovogenes]_103662	67.68	7e-128	csh:Closa_1594	K08602	pepF, pepB	oligoendopeptidase F	3.4.24.-	--

[denovogenes]_103663	93.23	3e-177	boa:Bovatus_01251	K16264	czcD, zitB	cobalt-zinc-cadmium efflux system protein	--	--

[denovogenes]_103665	42.62	9e-58	fsc:FSU_2094	K06921	K06921	uncharacterized protein	--	--

[denovogenes]_103666	77.90	2e-156	bfs:BF9343_2114	K01897	ACSL, fadD	long-chain acyl-CoA synthetase	6.2.1.3	Metabolism--Lipid metabolism--Fatty acid biosynthesis|Metabolism--Lipid metabolism--Fatty acid degradation|Metabolism--Global and overview maps--Metabolic pathways|Metabolism--Global and overview maps--Fatty acid metabolism|Cellular Processes--Cellular community - prokaryotes--Quorum sensing|Organismal Systems--Endocrine system--PPAR signaling pathway|Cellular Processes--Transport and catabolism--Peroxisome|Organismal Systems--Endocrine system--Adipocytokine signaling pathway

[denovogenes]_103669	98.88	0.0	cfd:CFNIH1_24245	K03100	lepB, TPP	signal peptidase I	3.4.21.89	Cellular Processes--Cellular community - prokaryotes--Quorum sensing|Genetic Information Processing--Folding, sorting and degradation--Protein export

[denovogenes]_10367	51.19	0.0	fpa:FPR_30230	K12132	prkC, stkP	eukaryotic-like serine/threonine-protein kinase	2.7.11.1	--

[denovogenes]_103671	46.24	2e-82	lacy:A4V08_16340	K07718	yesM	two-component system, sensor histidine kinase YesM	2.7.13.3	Environmental Information Processing--Signal transduction--Two-component system

[denovogenes]_103675	98.12	0.0	bxy:BXY_31350	K02528	ksgA	16S rRNA (adenine1518-N6/adenine1519-N6)-dimethyltransferase	2.1.1.182	--

[denovogenes]_103679	93.98	0.0	bhl:Bache_1077	K01265	map	methionyl aminopeptidase	3.4.11.18	--

[denovogenes]_10368	100.00	0.0	paeo:M801_1678	K01547	kdpB	K+-transporting ATPase ATPase B chain	3.6.3.12	Environmental Information Processing--Signal transduction--Two-component system

[denovogenes]_103681	55.68	3e-92	erb:A4V01_13950	K01992	ABC-2.P	ABC-2 type transport system permease protein	--	--

[denovogenes]_103682	60.53	9e-115	elm:ELI_2645	K00784	rnz	ribonuclease Z	3.1.26.11	Genetic Information Processing--Translation--RNA transport

[denovogenes]_103683	65.20	1e-122	btho:Btheta7330_02671	K03832	tonB	periplasmic protein TonB	--	--

[denovogenes]_103684	60.37	8e-113	btf:YBT020_20610	K06442	tlyA	23S rRNA (cytidine1920-2'-O)/16S rRNA (cytidine1409-2'-O)-methyltransferase	2.1.1.226 2.1.1.227	--

[denovogenes]_103685	54.96	6e-102	erb:A4V01_05655	K16785	ecfT	energy-coupling factor transport system permease protein	--	Environmental Information Processing--Membrane transport--ABC transporters

[denovogenes]_103686	45.25	6e-80	tbe:Trebr_0203	K06221	dkgA	2,5-diketo-D-gluconate reductase A	1.1.1.346	--

[denovogenes]_103687	95.04	0.0	bxy:BXY_45640	K03426	E3.6.1.22, NUDT12, nudC	NAD+ diphosphatase	3.6.1.22	Metabolism--Metabolism of cofactors and vitamins--Nicotinate and nicotinamide metabolism|Metabolism--Global and overview maps--Metabolic pathways|Cellular Processes--Transport and catabolism--Peroxisome

[denovogenes]_103689	53.53	5e-89	erl:AOC36_00590	K13292	lgt, umpA	phosphatidylglycerol:prolipoprotein diacylglycerol transferase	2.-.-.-	--

[denovogenes]_103690	98.50	0.0	bxy:BXY_34340	K01627	kdsA	2-dehydro-3-deoxyphosphooctonate aldolase (KDO 8-P synthase)	2.5.1.55	Metabolism--Glycan biosynthesis and metabolism--Lipopolysaccharide biosynthesis|Metabolism--Global and overview maps--Metabolic pathways

[denovogenes]_103691	61.45	7e-100	msi:Msm_1143	K01609	trpC	indole-3-glycerol phosphate synthase	4.1.1.48	Metabolism--Amino acid metabolism--Phenylalanine, tyrosine and tryptophan biosynthesis|Metabolism--Global and overview maps--Metabolic pathways|Metabolism--Global and overview maps--Biosynthesis of secondary metabolites|Metabolism--Global and overview maps--Biosynthesis of antibiotics|Metabolism--Global and overview maps--Biosynthesis of amino acids

[denovogenes]_103694	67.43	9e-123	erb:A4V01_05970	K00941	thiD	hydroxymethylpyrimidine/phosphomethylpyrimidine kinase	2.7.1.49 2.7.4.7	Metabolism--Metabolism of cofactors and vitamins--Thiamine metabolism|Metabolism--Global and overview maps--Metabolic pathways

[denovogenes]_103695	43.44	4e-64	ega:AL523_09125	K19337	hexR	RpiR family transcriptional regulator, carbohydrate utilization regulator	--	--

[denovogenes]_103696	57.52	2e-108	clt:CM240_2367	K00615	E2.2.1.1, tktA, tktB	transketolase	2.2.1.1	Metabolism--Carbohydrate metabolism--Pentose phosphate pathway|Metabolism--Energy metabolism--Carbon fixation in photosynthetic organisms|Metabolism--Metabolism of terpenoids and polyketides--Biosynthesis of ansamycins|Metabolism--Global and overview maps--Metabolic pathways|Metabolism--Global and overview maps--Biosynthesis of secondary metabolites|Metabolism--Global and overview maps--Microbial metabolism in diverse environments|Metabolism--Global and overview maps--Biosynthesis of antibiotics|Metabolism--Global and overview maps--Carbon metabolism|Metabolism--Global and overview maps--Biosynthesis of amino acids

[denovogenes]_103697	81.64	4e-154	bprl:CL2_10530	K01715	crt	enoyl-CoA hydratase	4.2.1.17	Metabolism--Carbohydrate metabolism--Butanoate metabolism|Metabolism--Global and overview maps--Carbon metabolism

[denovogenes]_10370	42.50	1e-171	obt:OPIT5_18450	K12308	bgaB, lacA	beta-galactosidase	3.2.1.23	Metabolism--Carbohydrate metabolism--Galactose metabolism

[denovogenes]_103702	77.56	2e-86	bcel:BcellWH2_04530	K03830	yafP	putative acetyltransferase	2.3.1.-	--

[denovogenes]_103707	45.15	9e-45	rva:Rvan_0039	K04114	bcrA, badF	benzoyl-CoA reductase subunit A	1.3.7.8	Metabolism--Xenobiotics biodegradation and metabolism--Benzoate degradation|Metabolism--Global and overview maps--Metabolic pathways|Metabolism--Global and overview maps--Microbial metabolism in diverse environments|Metabolism--Global and overview maps--Degradation of aromatic compounds

[denovogenes]_103708	99.63	0.0	edj:ECDH1ME8569_1260	K10112	msmX, msmK, malK, sugC, ggtA, msiK	multiple sugar transport system ATP-binding protein	--	Environmental Information Processing--Membrane transport--ABC transporters

[denovogenes]_103710	99.25	0.0	pdi:BDI_1320	K01629	rhaD	rhamnulose-1-phosphate aldolase	4.1.2.19	Metabolism--Carbohydrate metabolism--Pentose and glucuronate interconversions|Metabolism--Carbohydrate metabolism--Fructose and mannose metabolism|Metabolism--Global and overview maps--Microbial metabolism in diverse environments

[denovogenes]_103713	87.97	2e-173	vpr:Vpar_1463	K16785	ecfT	energy-coupling factor transport system permease protein	--	Environmental Information Processing--Membrane transport--ABC transporters

[denovogenes]_103719	42.18	4e-67	ppeo:ABE82_06205	K06158	ABCF3	ATP-binding cassette, subfamily F, member 3	--	--

[denovogenes]_10372	93.91	0.0	byl:A4V09_17825	K02355	fusA, GFM, EFG	elongation factor G	--	--

[denovogenes]_103720	86.52	2e-161	vpr:Vpar_1287	K03458	TC.NCS2	nucleobase:cation symporter-2, NCS2 family	--	--

[denovogenes]_103723	71.16	4e-137	euc:EC1_02260	K01006	ppdK	pyruvate, orthophosphate dikinase	2.7.9.1	Metabolism--Carbohydrate metabolism--Pyruvate metabolism|Metabolism--Energy metabolism--Carbon fixation in photosynthetic organisms|Metabolism--Energy metabolism--Carbon fixation pathways in prokaryotes|Metabolism--Global and overview maps--Metabolic pathways|Metabolism--Global and overview maps--Microbial metabolism in diverse environments|Metabolism--Global and overview maps--Carbon metabolism

[denovogenes]_103724	47.76	1e-72	mtr:MTR_8g013780	K01537	E3.6.3.8	Ca2+-transporting ATPase	3.6.3.8	--

[denovogenes]_103725	80.75	1e-145	cbt:CLH_0738	K09816	znuB	zinc transport system permease protein	--	Environmental Information Processing--Membrane transport--ABC transporters

[denovogenes]_103726	79.32	7e-145	cbk:CLL_A0994	K11070	potC	spermidine/putrescine transport system permease protein	--	Environmental Information Processing--Membrane transport--ABC transporters

[denovogenes]_103727	100.00	0.0	mmk:MU9_1935	K02795	PTS-Man-EIIC, manY	PTS system, mannose-specific IIC component	--	Metabolism--Carbohydrate metabolism--Fructose and mannose metabolism|Metabolism--Carbohydrate metabolism--Amino sugar and nucleotide sugar metabolism|Metabolism--Global and overview maps--Metabolic pathways|Environmental Information Processing--Membrane transport--Phosphotransferase system (PTS)

[denovogenes]_103731	45.10	3e-77	rpc:RPC_0779	K01092	E3.1.3.25, IMPA, suhB	myo-inositol-1(or 4)-monophosphatase	3.1.3.25	Metabolism--Biosynthesis of other secondary metabolites--Streptomycin biosynthesis|Metabolism--Carbohydrate metabolism--Inositol phosphate metabolism|Metabolism--Global and overview maps--Metabolic pathways|Environmental Information Processing--Signal transduction--Phosphatidylinositol signaling system

[denovogenes]_103732	99.20	0.0	mmk:MU9_1166	K01069	E3.1.2.6, gloB	hydroxyacylglutathione hydrolase	3.1.2.6	Metabolism--Carbohydrate metabolism--Pyruvate metabolism

[denovogenes]_103734	68.44	7e-132	ecas:ECBG_01046	K07124	K07124	uncharacterized protein	--	--

[denovogenes]_103735	99.25	0.0	mmk:MU9_500	K03832	tonB	periplasmic protein TonB	--	--

[denovogenes]_103736	68.94	6e-126	ess:ATZ33_13665	K06346	jag	spoIIIJ-associated protein	--	--

[denovogenes]_103737	99.25	0.0	mmk:MU9_3341	K15552	tauC	taurine transport system permease protein	--	Metabolism--Energy metabolism--Sulfur metabolism|Environmental Information Processing--Membrane transport--ABC transporters

[denovogenes]_10374	99.71	0.0	pdi:BDI_3408	K16089	TC.FEV.OM2, cirA, cfrA, hmuR	outer membrane receptor for ferrienterochelin and colicins	--	--

[denovogenes]_103740	99.62	0.0	bfb:VU15_12220	K11071	potB	spermidine/putrescine transport system permease protein	--	Environmental Information Processing--Membrane transport--ABC transporters

[denovogenes]_103741	99.62	0.0	mmk:MU9_809	K02031	ABC.PE.A	peptide/nickel transport system ATP-binding protein	--	Cellular Processes--Cellular community - prokaryotes--Quorum sensing

[denovogenes]_103742	71.15	3e-117	clt:CM240_1006	K08974	K08974	putative membrane protein	--	--

[denovogenes]_103743	85.94	1e-165	efq:DR75_2871	K03424	--	--	--	--

[denovogenes]_103747	80.00	1e-150	ehr:EHR_04405	K16785	ecfT	energy-coupling factor transport system permease protein	--	Environmental Information Processing--Membrane transport--ABC transporters

[denovogenes]_103749	85.28	5e-169	ega:AL523_06790	K09769	K09769	uncharacterized protein	--	--

[denovogenes]_10375	56.46	0.0	doi:FH5T_07695	K07407	E3.2.1.22B, galA, rafA	alpha-galactosidase	3.2.1.22	Metabolism--Carbohydrate metabolism--Galactose metabolism|Metabolism--Lipid metabolism--Glycerolipid metabolism|Metabolism--Lipid metabolism--Sphingolipid metabolism|Metabolism--Glycan biosynthesis and metabolism--Glycosphingolipid biosynthesis - globo and isoglobo series

[denovogenes]_103751	78.11	2e-150	cbt:CLH_1096	K05833	ABC.X4.A	putative ABC transport system ATP-binding protein	--	--

[denovogenes]_103755	86.04	3e-166	csb:CLSA_c06180	K03609	minD	septum site-determining protein MinD	--	--

[denovogenes]_103756	48.26	9e-82	pdc:CDIF630_03618	K02379	fdhD	FdhD protein	--	--

[denovogenes]_10376	61.46	0.0	cgo:Corgl_1074	K00705	malQ	4-alpha-glucanotransferase	2.4.1.25	Metabolism--Carbohydrate metabolism--Starch and sucrose metabolism|Metabolism--Global and overview maps--Metabolic pathways

[denovogenes]_103760	68.94	5e-128	cbv:U729_2505	K02796	PTS-Man-EIID, manZ	PTS system, mannose-specific IID component	--	Metabolism--Carbohydrate metabolism--Fructose and mannose metabolism|Metabolism--Carbohydrate metabolism--Amino sugar and nucleotide sugar metabolism|Metabolism--Global and overview maps--Metabolic pathways|Environmental Information Processing--Membrane transport--Phosphotransferase system (PTS)

[denovogenes]_103761	72.73	8e-138	cdg:CDBI1_03325	K03437	spoU	RNA methyltransferase, TrmH family	--	--

[denovogenes]_103762	100.00	0.0	bfb:VU15_19155	K01265	map	methionyl aminopeptidase	3.4.11.18	--

[denovogenes]_103763	84.47	6e-160	lacy:A4V08_18965	K11070	potC	spermidine/putrescine transport system permease protein	--	Environmental Information Processing--Membrane transport--ABC transporters

[denovogenes]_103765	41.76	2e-34	cpae:CPAST_c02260	K08600	srtB	sortase B	3.4.22.70	--

[denovogenes]_103766	100.00	0.0	mmk:MU9_933	K01265	map	methionyl aminopeptidase	3.4.11.18	--

[denovogenes]_103769	100.00	0.0	stb:SGPB_2004	K16785	ecfT	energy-coupling factor transport system permease protein	--	Environmental Information Processing--Membrane transport--ABC transporters

[denovogenes]_103770	100.00	0.0	ene:ENT_18390	K00858	ppnK, NADK	NAD+ kinase	2.7.1.23	Metabolism--Metabolism of cofactors and vitamins--Nicotinate and nicotinamide metabolism|Metabolism--Global and overview maps--Metabolic pathways

[denovogenes]_103771	100.00	0.0	ene:ENT_03060	K16785	ecfT	energy-coupling factor transport system permease protein	--	Environmental Information Processing--Membrane transport--ABC transporters

[denovogenes]_103774	100.00	0.0	efd:EFD32_1278	K03767	PPIA	peptidyl-prolyl cis-trans isomerase A (cyclophilin A)	5.2.1.8	Human Diseases--Drug resistance: Antimicrobial--Cationic antimicrobial peptide (CAMP) resistance

[denovogenes]_103775	69.80	1e-131	ctx:Clo1313_2769	K02003	ABC.CD.A	putative ABC transport system ATP-binding protein	--	--

[denovogenes]_103777	99.62	0.0	efq:DR75_1884	K09769	K09769	uncharacterized protein	--	--

[denovogenes]_103779	54.05	7e-101	pbd:PBOR_11875	K07720	yesN	two-component system, response regulator YesN	--	Environmental Information Processing--Signal transduction--Two-component system

[denovogenes]_10378	99.28	0.0	boa:Bovatus_02816	K07037	K07037	uncharacterized protein	--	--

[denovogenes]_103780	75.47	2e-144	csr:Cspa_c02210	K16785	ecfT	energy-coupling factor transport system permease protein	--	Environmental Information Processing--Membrane transport--ABC transporters

[denovogenes]_103781	85.61	9e-164	csb:CLSA_c06180	K03609	minD	septum site-determining protein MinD	--	--

[denovogenes]_103783	100.00	0.0	pvl:AOB99_03165	K08354	phsC	thiosulfate reductase cytochrome b subunit	--	Metabolism--Energy metabolism--Sulfur metabolism|Metabolism--Global and overview maps--Microbial metabolism in diverse environments

[denovogenes]_103784	40.16	9e-20	six:BSY16_4547	K18910	dpe, lre	D-psicose/D-tagatose/L-ribulose 3-epimerase	5.1.3.30 5.1.3.31	--

[denovogenes]_103786	77.10	6e-149	pdf:CD630DERM_15990	K00941	thiD	hydroxymethylpyrimidine/phosphomethylpyrimidine kinase	2.7.1.49 2.7.4.7	Metabolism--Metabolism of cofactors and vitamins--Thiamine metabolism|Metabolism--Global and overview maps--Metabolic pathways

[denovogenes]_10379	100.00	0.0	bfb:VU15_10885	K16089	TC.FEV.OM2, cirA, cfrA, hmuR	outer membrane receptor for ferrienterochelin and colicins	--	--

[denovogenes]_103790	51.35	7e-82	cbt:CLH_0760	K00826	E2.6.1.42, ilvE	branched-chain amino acid aminotransferase	2.6.1.42	Metabolism--Amino acid metabolism--Cysteine and methionine metabolism|Metabolism--Amino acid metabolism--Valine, leucine and isoleucine degradation|Metabolism--Amino acid metabolism--Valine, leucine and isoleucine biosynthesis|Metabolism--Metabolism of cofactors and vitamins--Pantothenate and CoA biosynthesis|Metabolism--Global and overview maps--Metabolic pathways|Metabolism--Global and overview maps--Biosynthesis of secondary metabolites|Metabolism--Global and overview maps--Biosynthesis of antibiotics|Metabolism--Global and overview maps--2-Oxocarboxylic acid metabolism|Metabolism--Global and overview maps--Biosynthesis of amino acids

[denovogenes]_103791	99.61	0.0	stb:SGPB_0952	K02003	ABC.CD.A	putative ABC transport system ATP-binding protein	--	--

[denovogenes]_103792	100.00	0.0	pvl:AOB99_02070	K07090	K07090	uncharacterized protein	--	--

[denovogenes]_103793	98.87	0.0	ecos:EC958_4472	K02814	PTS-Sor-EIIC, sorA	PTS system, sorbose-specific IIC component	--	Metabolism--Carbohydrate metabolism--Fructose and mannose metabolism|Environmental Information Processing--Membrane transport--Phosphotransferase system (PTS)

[denovogenes]_103794	95.83	0.0	cfd:CFNIH1_18830	K01591	pyrF	orotidine-5'-phosphate decarboxylase	4.1.1.23	Metabolism--Nucleotide metabolism--Pyrimidine metabolism|Metabolism--Global and overview maps--Metabolic pathways

[denovogenes]_103795	93.21	0.0	cif:AL515_11970	K11962	urtD	urea transport system ATP-binding protein	--	Environmental Information Processing--Membrane transport--ABC transporters

[denovogenes]_103798	98.87	0.0	cfd:CFNIH1_10445	K02013	ABC.FEV.A	iron complex transport system ATP-binding protein	3.6.3.34	Environmental Information Processing--Membrane transport--ABC transporters

[denovogenes]_103799	96.24	0.0	cif:AL515_12335	K06145	gntR	LacI family transcriptional regulator, gluconate utilization system Gnt-I transcriptional repressor	--	--

[denovogenes]_1038	91.21	0.0	pfr:PFREUD_17350	K03723	mfd	transcription-repair coupling factor (superfamily II helicase)	3.6.4.-	Genetic Information Processing--Replication and repair--Nucleotide excision repair

[denovogenes]_103800	98.87	0.0	stb:SGPB_1038	K00941	thiD	hydroxymethylpyrimidine/phosphomethylpyrimidine kinase	2.7.1.49 2.7.4.7	Metabolism--Metabolism of cofactors and vitamins--Thiamine metabolism|Metabolism--Global and overview maps--Metabolic pathways

[denovogenes]_103801	99.62	0.0	stb:SGPB_1037	K00878	thiM	hydroxyethylthiazole kinase	2.7.1.50	Metabolism--Metabolism of cofactors and vitamins--Thiamine metabolism|Metabolism--Global and overview maps--Metabolic pathways

[denovogenes]_103802	59.62	2e-106	yen:YE0252	K01104	E3.1.3.48	protein-tyrosine phosphatase	3.1.3.48	--

[denovogenes]_103804	98.87	0.0	cbut:ATN24_00645	K02029	ABC.PA.P	polar amino acid transport system permease protein	--	--

[denovogenes]_103804	98.87	0.0	cbut:ATN24_00645	K02030	ABC.PA.S	polar amino acid transport system substrate-binding protein	--	--

[denovogenes]_103805	78.71	1e-150	pdf:CD630DERM_15990	K00941	thiD	hydroxymethylpyrimidine/phosphomethylpyrimidine kinase	2.7.1.49 2.7.4.7	Metabolism--Metabolism of cofactors and vitamins--Thiamine metabolism|Metabolism--Global and overview maps--Metabolic pathways

[denovogenes]_103806	61.80	5e-112	gap:GAPWK_2630	K00558	DNMT1, dcm	DNA (cytosine-5)-methyltransferase 1	2.1.1.37	Metabolism--Amino acid metabolism--Cysteine and methionine metabolism|Metabolism--Global and overview maps--Metabolic pathways|Human Diseases--Cancers: Overview--MicroRNAs in cancer

[denovogenes]_103808	98.49	0.0	cfd:CFNIH1_13040	K02013	ABC.FEV.A	iron complex transport system ATP-binding protein	3.6.3.34	Environmental Information Processing--Membrane transport--ABC transporters

[denovogenes]_10381	59.91	0.0	erb:A4V01_11780	K03168	topA	DNA topoisomerase I	5.99.1.2	--

[denovogenes]_103810	93.58	0.0	cbut:ATN24_18620	K02594	nifV	homocitrate synthase NifV	2.3.3.14	Metabolism--Carbohydrate metabolism--Pyruvate metabolism

[denovogenes]_103811	99.22	0.0	cbut:ATN24_15160	K01752	E4.3.1.17, sdaA, sdaB, tdcG	L-serine dehydratase	4.3.1.17	Metabolism--Amino acid metabolism--Glycine, serine and threonine metabolism|Metabolism--Amino acid metabolism--Cysteine and methionine metabolism|Metabolism--Global and overview maps--Metabolic pathways|Metabolism--Global and overview maps--Biosynthesis of secondary metabolites|Metabolism--Global and overview maps--Biosynthesis of antibiotics|Metabolism--Global and overview maps--Carbon metabolism|Metabolism--Global and overview maps--Biosynthesis of amino acids

[denovogenes]_103812	99.25	0.0	pvl:AOB99_09920	K00941	thiD	hydroxymethylpyrimidine/phosphomethylpyrimidine kinase	2.7.1.49 2.7.4.7	Metabolism--Metabolism of cofactors and vitamins--Thiamine metabolism|Metabolism--Global and overview maps--Metabolic pathways

[denovogenes]_103819	80.38	1e-159	cdg:CDBI1_00575	K16785	ecfT	energy-coupling factor transport system permease protein	--	Environmental Information Processing--Membrane transport--ABC transporters

[denovogenes]_10382	41.56	7e-178	boa:Bovatus_03166	K01187	malZ	alpha-glucosidase	3.2.1.20	Metabolism--Carbohydrate metabolism--Galactose metabolism|Metabolism--Carbohydrate metabolism--Starch and sucrose metabolism|Metabolism--Global and overview maps--Metabolic pathways

[denovogenes]_103820	69.80	2e-118	ctc:CTC_02435	K00302	soxA	sarcosine oxidase, subunit alpha	1.5.3.1	Metabolism--Amino acid metabolism--Glycine, serine and threonine metabolism|Metabolism--Global and overview maps--Metabolic pathways

[denovogenes]_103821	99.62	0.0	cbut:ATN24_02830	K06896	mapP	maltose 6'-phosphate phosphatase	3.1.3.90	Metabolism--Carbohydrate metabolism--Starch and sucrose metabolism

[denovogenes]_103822	45.82	2e-86	sho:SHJGH_0503	K06221	dkgA	2,5-diketo-D-gluconate reductase A	1.1.1.346	--

[denovogenes]_103823	96.98	0.0	cfd:CFNIH1_05755	K02848	waaP, rfaP	heptose I phosphotransferase	2.7.1.-	Metabolism--Glycan biosynthesis and metabolism--Lipopolysaccharide biosynthesis|Metabolism--Global and overview maps--Metabolic pathways

[denovogenes]_103824	81.89	1e-165	cdg:CDBI1_00575	K16785	ecfT	energy-coupling factor transport system permease protein	--	Environmental Information Processing--Membrane transport--ABC transporters

[denovogenes]_103825	90.23	2e-171	prg:RB151_003120	K16345	xanP	xanthine permease XanP	--	--

[denovogenes]_103826	84.09	8e-165	cpae:CPAST_c10110	K07496	K07496	putative transposase	--	--

[denovogenes]_103828	42.62	2e-65	cle:Clole_2785	K02315	dnaC	DNA replication protein DnaC	--	--

[denovogenes]_10383	80.90	0.0	mhg:MHY_05010	K01547	kdpB	K+-transporting ATPase ATPase B chain	3.6.3.12	Environmental Information Processing--Signal transduction--Two-component system

[denovogenes]_103833	40.18	4e-15	eac:EAL2_c19650	K01358	clpP, CLPP	ATP-dependent Clp protease, protease subunit	3.4.21.92	Cellular Processes--Cell growth and death--Cell cycle - Caulobacter|Organismal Systems--Aging--Longevity regulating pathway - worm

[denovogenes]_103834	56.69	5e-96	cbk:CLL_A3589	K03442	mscS	small conductance mechanosensitive channel	--	--

[denovogenes]_103835	68.44	4e-132	gfe:Gferi_26055	K04024	eutJ	ethanolamine utilization protein EutJ	--	--

[denovogenes]_103838	100.00	0.0	ecoh:ECRM13516_2171	K01424	E3.5.1.1, ansA, ansB	L-asparaginase	3.5.1.1	Metabolism--Amino acid metabolism--Alanine, aspartate and glutamate metabolism|Metabolism--Metabolism of other amino acids--Cyanoamino acid metabolism|Metabolism--Global and overview maps--Metabolic pathways|Metabolism--Global and overview maps--Biosynthesis of secondary metabolites

[denovogenes]_103839	98.49	0.0	cbut:ATN24_09750	K15598	thiY	putative hydroxymethylpyrimidine transport system substrate-binding protein	--	Environmental Information Processing--Membrane transport--ABC transporters

[denovogenes]_103840	95.86	0.0	edj:ECDH1ME8569_3863	K07709	zraS, hydH	two-component system, NtrC family, sensor histidine kinase HydH	2.7.13.3	Environmental Information Processing--Signal transduction--Two-component system

[denovogenes]_103842	98.87	0.0	cpr:CPR_2648	K02314	dnaB	replicative DNA helicase	3.6.4.12	Genetic Information Processing--Replication and repair--DNA replication|Cellular Processes--Cell growth and death--Cell cycle - Caulobacter

[denovogenes]_103843	88.72	0.0	bld:BLi01200	K00364	E1.7.1.7, guaC	GMP reductase	1.7.1.7	Metabolism--Nucleotide metabolism--Purine metabolism

[denovogenes]_103844	57.89	2e-102	erl:AOC36_08840	K02010	afuC, fbpC	iron(III) transport system ATP-binding protein	3.6.3.30	Environmental Information Processing--Membrane transport--ABC transporters

[denovogenes]_103846	66.29	2e-128	cst:CLOST_0671	K01258	pepT	tripeptide aminopeptidase	3.4.11.4	--

[denovogenes]_103848	66.67	2e-128	cbn:CbC4_0062	K03742	pncC	nicotinamide-nucleotide amidase	3.5.1.42	Metabolism--Metabolism of cofactors and vitamins--Nicotinate and nicotinamide metabolism

[denovogenes]_103851	99.62	0.0	sens:Q786_22305	K03427	hsdM	type I restriction enzyme M protein	2.1.1.72	--

[denovogenes]_103853	86.19	4e-170	thj:104815606	K08916	--	--	--	Metabolism--Energy metabolism--Photosynthesis - antenna proteins|Metabolism--Global and overview maps--Metabolic pathways

[denovogenes]_103854	99.61	0.0	cbut:ATN24_05090	K02392	flgG	flagellar basal-body rod protein FlgG	--	Cellular Processes--Cell motility--Flagellar assembly

[denovogenes]_103855	99.25	0.0	cbut:ATN24_06740	K03524	birA	BirA family transcriptional regulator, biotin operon repressor / biotin-[acetyl-CoA-carboxylase] ligase	6.3.4.15	Metabolism--Metabolism of cofactors and vitamins--Biotin metabolism|Metabolism--Global and overview maps--Metabolic pathways

[denovogenes]_103857	100.00	0.0	elo:EC042_3422	K02081	agaR	DeoR family transcriptional regulator, aga operon transcriptional repressor	--	--

[denovogenes]_103858	98.11	0.0	cfd:CFNIH1_13265	K07700	dpiB, citA	two-component system, CitB family, cit operon sensor histidine kinase CitA	2.7.13.3	Environmental Information Processing--Signal transduction--Two-component system

[denovogenes]_10386	98.12	0.0	mmk:MU9_1760	K03407	cheA	two-component system, chemotaxis family, sensor kinase CheA	2.7.13.3	Environmental Information Processing--Signal transduction--Two-component system|Cellular Processes--Cell motility--Bacterial chemotaxis

[denovogenes]_103860	47.92	1e-69	lmob:BN419_2113	K03657	uvrD, pcrA	DNA helicase II / ATP-dependent DNA helicase PcrA	3.6.4.12	Genetic Information Processing--Replication and repair--Nucleotide excision repair|Genetic Information Processing--Replication and repair--Mismatch repair

[denovogenes]_103862	80.00	8e-160	vna:PN96_02885	K01206	FUCA	alpha-L-fucosidase	3.2.1.51	Metabolism--Glycan biosynthesis and metabolism--Other glycan degradation

[denovogenes]_103863	70.68	8e-135	mhg:MHY_13590	K03070	secA	preprotein translocase subunit SecA	--	Cellular Processes--Cellular community - prokaryotes--Quorum sensing|Genetic Information Processing--Folding, sorting and degradation--Protein export|Environmental Information Processing--Membrane transport--Bacterial secretion system

[denovogenes]_103864	76.98	8e-144	cle:Clole_2157	K01938	fhs	formate--tetrahydrofolate ligase	6.3.4.3	Metabolism--Metabolism of cofactors and vitamins--One carbon pool by folate|Metabolism--Energy metabolism--Carbon fixation pathways in prokaryotes|Metabolism--Global and overview maps--Metabolic pathways|Metabolism--Global and overview maps--Microbial metabolism in diverse environments|Metabolism--Global and overview maps--Carbon metabolism

[denovogenes]_103865	98.12	0.0	boa:Bovatus_02936	K01195	uidA, GUSB	beta-glucuronidase	3.2.1.31	Metabolism--Carbohydrate metabolism--Pentose and glucuronate interconversions|Metabolism--Glycan biosynthesis and metabolism--Glycosaminoglycan degradation|Metabolism--Metabolism of cofactors and vitamins--Porphyrin and chlorophyll metabolism|Metabolism--Biosynthesis of other secondary metabolites--Flavone and flavonol biosynthesis|Metabolism--Xenobiotics biodegradation and metabolism--Drug metabolism - other enzymes|Metabolism--Global and overview maps--Metabolic pathways|Metabolism--Global and overview maps--Biosynthesis of secondary metabolites|Cellular Processes--Transport and catabolism--Lysosome

[denovogenes]_103866	58.05	3e-109	cbut:ATN24_08185	K01200	pulA	pullulanase	3.2.1.41	Metabolism--Carbohydrate metabolism--Starch and sucrose metabolism|Metabolism--Global and overview maps--Metabolic pathways|Metabolism--Global and overview maps--Biosynthesis of secondary metabolites

[denovogenes]_103868	83.15	2e-161	pdf:CD630DERM_31730	K00927	PGK, pgk	phosphoglycerate kinase	2.7.2.3	Metabolism--Carbohydrate metabolism--Glycolysis / Gluconeogenesis|Metabolism--Energy metabolism--Carbon fixation in photosynthetic organisms|Metabolism--Global and overview maps--Metabolic pathways|Metabolism--Global and overview maps--Biosynthesis of secondary metabolites|Metabolism--Global and overview maps--Microbial metabolism in diverse environments|Metabolism--Global and overview maps--Biosynthesis of antibiotics|Metabolism--Global and overview maps--Carbon metabolism|Metabolism--Global and overview maps--Biosynthesis of amino acids

[denovogenes]_103872	82.33	6e-160	cpe:CPE1094	K01595	ppc	phosphoenolpyruvate carboxylase	4.1.1.31	Metabolism--Carbohydrate metabolism--Pyruvate metabolism|Metabolism--Energy metabolism--Methane metabolism|Metabolism--Energy metabolism--Carbon fixation in photosynthetic organisms|Metabolism--Energy metabolism--Carbon fixation pathways in prokaryotes|Metabolism--Global and overview maps--Metabolic pathways|Metabolism--Global and overview maps--Microbial metabolism in diverse environments|Metabolism--Global and overview maps--Carbon metabolism

[denovogenes]_103873	67.97	6e-122	sfe:SFxv_0973	K00656	E2.3.1.54, pflD	formate C-acetyltransferase	2.3.1.54	Metabolism--Carbohydrate metabolism--Pyruvate metabolism|Metabolism--Carbohydrate metabolism--Propanoate metabolism|Metabolism--Carbohydrate metabolism--Butanoate metabolism|Metabolism--Global and overview maps--Metabolic pathways

[denovogenes]_103875	100.00	0.0	cbut:ATN24_06350	K00266	gltD	glutamate synthase (NADPH/NADH) small chain	1.4.1.13 1.4.1.14	Metabolism--Amino acid metabolism--Alanine, aspartate and glutamate metabolism|Metabolism--Energy metabolism--Nitrogen metabolism|Metabolism--Global and overview maps--Metabolic pathways|Metabolism--Global and overview maps--Biosynthesis of secondary metabolites|Metabolism--Global and overview maps--Microbial metabolism in diverse environments|Metabolism--Global and overview maps--Biosynthesis of antibiotics|Metabolism--Global and overview maps--Biosynthesis of amino acids

[denovogenes]_103877	58.56	1e-105	cad:Curi_c18540	K00145	argC	N-acetyl-gamma-glutamyl-phosphate reductase	1.2.1.38	Metabolism--Amino acid metabolism--Arginine biosynthesis|Metabolism--Global and overview maps--Metabolic pathways|Metabolism--Global and overview maps--Biosynthesis of secondary metabolites|Metabolism--Global and overview maps--Biosynthesis of antibiotics|Metabolism--Global and overview maps--2-Oxocarboxylic acid metabolism|Metabolism--Global and overview maps--Biosynthesis of amino acids

[denovogenes]_103878	61.74	2e-110	afn:Acfer_0555	K03521	fixA, etfB	electron transfer flavoprotein beta subunit	--	--

[denovogenes]_103879	56.25	2e-100	chy:CHY_1441	K00772	mtaP, MTAP	5'-methylthioadenosine phosphorylase	2.4.2.28	Metabolism--Amino acid metabolism--Cysteine and methionine metabolism|Metabolism--Global and overview maps--Metabolic pathways

[denovogenes]_10388	100.00	0.0	mmk:MU9_630	K01879	glyS	glycyl-tRNA synthetase beta chain	6.1.1.14	Genetic Information Processing--Translation--Aminoacyl-tRNA biosynthesis

[denovogenes]_103880	71.32	5e-138	pdi:BDI_3293	K06859	pgi1	glucose-6-phosphate isomerase, archaeal	5.3.1.9	Metabolism--Carbohydrate metabolism--Glycolysis / Gluconeogenesis|Metabolism--Carbohydrate metabolism--Pentose phosphate pathway|Metabolism--Carbohydrate metabolism--Starch and sucrose metabolism|Metabolism--Carbohydrate metabolism--Amino sugar and nucleotide sugar metabolism|Metabolism--Global and overview maps--Metabolic pathways|Metabolism--Global and overview maps--Biosynthesis of secondary metabolites|Metabolism--Global and overview maps--Microbial metabolism in diverse environments|Metabolism--Global and overview maps--Biosynthesis of antibiotics|Metabolism--Global and overview maps--Carbon metabolism

[denovogenes]_103882	62.36	2e-108	baco:OXB_2280	K02050	ABC.SN.P	NitT/TauT family transport system permease protein	--	--

[denovogenes]_103886	100.00	0.0	bvu:BVU_3318	K02069	ABC.X2.P	putative ABC transport system permease protein	--	--

[denovogenes]_103887	75.85	3e-140	bfs:BF9343_2696	K01991	ABC-2.OM, wza	polysaccharide biosynthesis/export protein	--	Cellular Processes--Cellular community - prokaryotes--Biofilm formation - Escherichia coli

[denovogenes]_103889	69.58	2e-135	bsa:Bacsa_1526	K05306	phnX	phosphonoacetaldehyde hydrolase	3.11.1.1	Metabolism--Metabolism of other amino acids--Phosphonate and phosphinate metabolism|Metabolism--Global and overview maps--Metabolic pathways|Metabolism--Global and overview maps--Microbial metabolism in diverse environments

[denovogenes]_10389	99.85	0.0	mmk:MU9_2745	K16089	TC.FEV.OM2, cirA, cfrA, hmuR	outer membrane receptor for ferrienterochelin and colicins	--	--

[denovogenes]_103891	51.79	3e-82	mhg:MHY_00150	K02020	modA	molybdate transport system substrate-binding protein	--	Environmental Information Processing--Membrane transport--ABC transporters

[denovogenes]_103892	71.26	1e-132	rto:RTO_14150	K00655	plsC	1-acyl-sn-glycerol-3-phosphate acyltransferase	2.3.1.51	Metabolism--Lipid metabolism--Glycerolipid metabolism|Metabolism--Lipid metabolism--Glycerophospholipid metabolism|Metabolism--Global and overview maps--Metabolic pathways|Metabolism--Global and overview maps--Biosynthesis of secondary metabolites

[denovogenes]_103894	84.09	6e-171	lacy:A4V08_28595	K00615	E2.2.1.1, tktA, tktB	transketolase	2.2.1.1	Metabolism--Carbohydrate metabolism--Pentose phosphate pathway|Metabolism--Energy metabolism--Carbon fixation in photosynthetic organisms|Metabolism--Metabolism of terpenoids and polyketides--Biosynthesis of ansamycins|Metabolism--Global and overview maps--Metabolic pathways|Metabolism--Global and overview maps--Biosynthesis of secondary metabolites|Metabolism--Global and overview maps--Microbial metabolism in diverse environments|Metabolism--Global and overview maps--Biosynthesis of antibiotics|Metabolism--Global and overview maps--Carbon metabolism|Metabolism--Global and overview maps--Biosynthesis of amino acids

[denovogenes]_103895	67.55	3e-139	rob:CK5_22830	K02013	ABC.FEV.A	iron complex transport system ATP-binding protein	3.6.3.34	Environmental Information Processing--Membrane transport--ABC transporters

[denovogenes]_103897	68.20	3e-123	ibu:IB211_01598	K05896	scpA	segregation and condensation protein A	--	--

[denovogenes]_103898	69.43	5e-133	cct:CC1_16000	K02034	ABC.PE.P1	peptide/nickel transport system permease protein	--	Cellular Processes--Cellular community - prokaryotes--Quorum sensing

[denovogenes]_103899	75.44	2e-117	rto:RTO_03720	K02050	ABC.SN.P	NitT/TauT family transport system permease protein	--	--

[denovogenes]_10390	99.27	0.0	bfs:BF9343_4073	K00656	E2.3.1.54, pflD	formate C-acetyltransferase	2.3.1.54	Metabolism--Carbohydrate metabolism--Pyruvate metabolism|Metabolism--Carbohydrate metabolism--Propanoate metabolism|Metabolism--Carbohydrate metabolism--Butanoate metabolism|Metabolism--Global and overview maps--Metabolic pathways

[denovogenes]_103901	96.46	2e-178	btho:Btheta7330_02980	K01991	ABC-2.OM, wza	polysaccharide biosynthesis/export protein	--	Cellular Processes--Cellular community - prokaryotes--Biofilm formation - Escherichia coli

[denovogenes]_103902	100.00	0.0	bvu:BVU_0888	K07271	licD	lipopolysaccharide cholinephosphotransferase	2.7.8.-	--

[denovogenes]_103904	61.78	9e-110	bcel:BcellWH2_03282	K07052	K07052	uncharacterized protein	--	--

[denovogenes]_103906	99.25	0.0	bvu:BVU_2903	K03426	E3.6.1.22, NUDT12, nudC	NAD+ diphosphatase	3.6.1.22	Metabolism--Metabolism of cofactors and vitamins--Nicotinate and nicotinamide metabolism|Metabolism--Global and overview maps--Metabolic pathways|Cellular Processes--Transport and catabolism--Peroxisome

[denovogenes]_10391	61.79	0.0	btho:Btheta7330_00947	K09955	K09955	uncharacterized protein	--	--

[denovogenes]_103911	72.80	2e-133	bprm:CL3_26560	K05936	cobM, cbiF	precorrin-4/cobalt-precorrin-4 C11-methyltransferase	2.1.1.133 2.1.1.271	Metabolism--Metabolism of cofactors and vitamins--Porphyrin and chlorophyll metabolism|Metabolism--Global and overview maps--Metabolic pathways

[denovogenes]_103912	41.70	1e-46	erb:A4V01_08400	K06890	K06890	uncharacterized protein	--	--

[denovogenes]_103914	62.64	1e-116	esu:EUS_12600	K16785	ecfT	energy-coupling factor transport system permease protein	--	Environmental Information Processing--Membrane transport--ABC transporters

[denovogenes]_103915	53.79	9e-94	afd:Alfi_0829	K00558	DNMT1, dcm	DNA (cytosine-5)-methyltransferase 1	2.1.1.37	Metabolism--Amino acid metabolism--Cysteine and methionine metabolism|Metabolism--Global and overview maps--Metabolic pathways|Human Diseases--Cancers: Overview--MicroRNAs in cancer

[denovogenes]_103916	75.00	8e-137	fpa:FPR_28350	K00941	thiD	hydroxymethylpyrimidine/phosphomethylpyrimidine kinase	2.7.1.49 2.7.4.7	Metabolism--Metabolism of cofactors and vitamins--Thiamine metabolism|Metabolism--Global and overview maps--Metabolic pathways

[denovogenes]_103917	59.25	1e-105	puf:UFO1_3171	K03521	fixA, etfB	electron transfer flavoprotein beta subunit	--	--

[denovogenes]_103918	81.89	8e-160	bdo:EL88_13530	K01991	ABC-2.OM, wza	polysaccharide biosynthesis/export protein	--	Cellular Processes--Cellular community - prokaryotes--Biofilm formation - Escherichia coli

[denovogenes]_10392	79.71	0.0	lacy:A4V08_01680	K03169	topB	DNA topoisomerase III	5.99.1.2	--

[denovogenes]_103920	92.08	5e-180	rto:RTO_09860	K02886	RP-L2, MRPL2, rplB	large subunit ribosomal protein L2	--	Genetic Information Processing--Translation--Ribosome

[denovogenes]_103921	57.60	7e-96	lacy:A4V08_19270	K02654	pilD, pppA	leader peptidase (prepilin peptidase) / N-methyltransferase	3.4.23.43 2.1.1.-	--

[denovogenes]_103922	74.24	2e-145	csh:Closa_3615	K00286	proC	pyrroline-5-carboxylate reductase	1.5.1.2	Metabolism--Amino acid metabolism--Arginine and proline metabolism|Metabolism--Global and overview maps--Metabolic pathways|Metabolism--Global and overview maps--Biosynthesis of secondary metabolites|Metabolism--Global and overview maps--Biosynthesis of antibiotics|Metabolism--Global and overview maps--Biosynthesis of amino acids

[denovogenes]_103923	53.31	4e-83	ibu:IB211_03367	K03497	parB, spo0J	chromosome partitioning protein, ParB family	--	--

[denovogenes]_103925	100.00	0.0	bvu:BVU_3582	K01627	kdsA	2-dehydro-3-deoxyphosphooctonate aldolase (KDO 8-P synthase)	2.5.1.55	Metabolism--Glycan biosynthesis and metabolism--Lipopolysaccharide biosynthesis|Metabolism--Global and overview maps--Metabolic pathways

[denovogenes]_103927	66.80	4e-118	cso:CLS_03410	K01448	E3.5.1.28B, amiA, amiB, amiC	N-acetylmuramoyl-L-alanine amidase	3.5.1.28	Human Diseases--Drug resistance: Antimicrobial--Cationic antimicrobial peptide (CAMP) resistance

[denovogenes]_103928	42.17	4e-55	llm:llmg_0412	K00784	rnz	ribonuclease Z	3.1.26.11	Genetic Information Processing--Translation--RNA transport

[denovogenes]_10393	92.31	0.0	lacy:A4V08_12320	K02355	fusA, GFM, EFG	elongation factor G	--	--

[denovogenes]_103931	99.25	0.0	ecoh:ECRM13516_4417	K02848	waaP, rfaP	heptose I phosphotransferase	2.7.1.-	Metabolism--Glycan biosynthesis and metabolism--Lipopolysaccharide biosynthesis|Metabolism--Global and overview maps--Metabolic pathways

[denovogenes]_103932	40.48	5e-24	ckl:CKL_1926	K01447	E3.5.1.28A, cwlA, xlyA, xlyB	N-acetylmuramoyl-L-alanine amidase	3.5.1.28	--

[denovogenes]_103933	93.58	7e-180	lacy:A4V08_18965	K11070	potC	spermidine/putrescine transport system permease protein	--	Environmental Information Processing--Membrane transport--ABC transporters

[denovogenes]_103934	100.00	0.0	bxy:BXY_16590	K03561	exbB	biopolymer transport protein ExbB	--	--

[denovogenes]_103935	91.32	3e-178	lacy:A4V08_02665	K05833	ABC.X4.A	putative ABC transport system ATP-binding protein	--	--

[denovogenes]_103936	98.12	0.0	lacy:A4V08_31120	K02556	motA	chemotaxis protein MotA	--	Environmental Information Processing--Signal transduction--Two-component system|Cellular Processes--Cell motility--Bacterial chemotaxis|Cellular Processes--Cell motility--Flagellar assembly

[denovogenes]_103937	54.51	2e-95	bxy:BXY_38480	K01991	ABC-2.OM, wza	polysaccharide biosynthesis/export protein	--	Cellular Processes--Cellular community - prokaryotes--Biofilm formation - Escherichia coli

[denovogenes]_103938	82.63	7e-161	cle:Clole_3210	K02013	ABC.FEV.A	iron complex transport system ATP-binding protein	3.6.3.34	Environmental Information Processing--Membrane transport--ABC transporters

[denovogenes]_103939	65.61	1e-117	cle:Clole_3235	K13292	lgt, umpA	phosphatidylglycerol:prolipoprotein diacylglycerol transferase	2.-.-.-	--

[denovogenes]_10394	96.23	0.0	sif:Sinf_1793	K03205	virD4, lvhD4	type IV secretion system protein VirD4	--	Environmental Information Processing--Membrane transport--Bacterial secretion system

[denovogenes]_103940	100.00	0.0	sdy:SDY_2050	K03424	--	--	--	--

[denovogenes]_103944	81.13	4e-157	lacy:A4V08_02665	K05833	ABC.X4.A	putative ABC transport system ATP-binding protein	--	--

[denovogenes]_103948	58.23	2e-96	ibu:IB211_02500c	K07124	K07124	uncharacterized protein	--	--

[denovogenes]_103949	42.06	1e-59	pfl:PFL_3340	K03436	fruR2, fruR	DeoR family transcriptional regulator, fructose operon transcriptional repressor	--	--

[denovogenes]_10395	82.41	0.0	stb:SGPB_0701	K01547	kdpB	K+-transporting ATPase ATPase B chain	3.6.3.12	Environmental Information Processing--Signal transduction--Two-component system

[denovogenes]_103951	53.49	2e-82	byl:A4V09_08850	K01420	fnr	CRP/FNR family transcriptional regulator, anaerobic regulatory protein	--	--

[denovogenes]_103953	57.89	4e-100	ssg:Selsp_1352	K07455	recT	recombination protein RecT	--	--

[denovogenes]_103955	47.22	5e-79	fpe:Ferpe_1836	K02032	ABC.PE.A1	peptide/nickel transport system ATP-binding protein	--	Cellular Processes--Cellular community - prokaryotes--Quorum sensing

[denovogenes]_103956	100.00	0.0	btho:Btheta7330_04450	K03561	exbB	biopolymer transport protein ExbB	--	--

[denovogenes]_10396	99.57	0.0	cfd:CFNIH1_19375	K04072	adhE	acetaldehyde dehydrogenase / alcohol dehydrogenase	1.2.1.10 1.1.1.1	Metabolism--Carbohydrate metabolism--Glycolysis / Gluconeogenesis|Metabolism--Lipid metabolism--Fatty acid degradation|Metabolism--Amino acid metabolism--Tyrosine metabolism|Metabolism--Carbohydrate metabolism--Pyruvate metabolism|Metabolism--Xenobiotics biodegradation and metabolism--Chloroalkane and chloroalkene degradation|Metabolism--Xenobiotics biodegradation and metabolism--Naphthalene degradation|Metabolism--Carbohydrate metabolism--Butanoate metabolism|Metabolism--Global and overview maps--Metabolic pathways|Metabolism--Global and overview maps--Biosynthesis of secondary metabolites|Metabolism--Global and overview maps--Microbial metabolism in diverse environments|Metabolism--Global and overview maps--Biosynthesis of antibiotics|Metabolism--Global and overview maps--Degradation of aromatic compounds

[denovogenes]_103960	40.32	9e-49	bsm:BSM4216_1545	K00059	fabG	3-oxoacyl-[acyl-carrier protein] reductase	1.1.1.100	Metabolism--Lipid metabolism--Fatty acid biosynthesis|Metabolism--Metabolism of cofactors and vitamins--Biotin metabolism|Metabolism--Lipid metabolism--Biosynthesis of unsaturated fatty acids|Metabolism--Global and overview maps--Metabolic pathways|Metabolism--Global and overview maps--Fatty acid metabolism

[denovogenes]_103962	99.25	0.0	fpla:A4U99_04135	K02190	cbiK	sirohydrochlorin cobaltochelatase	4.99.1.3	Metabolism--Metabolism of cofactors and vitamins--Porphyrin and chlorophyll metabolism|Metabolism--Global and overview maps--Metabolic pathways

[denovogenes]_103963	74.62	1e-145	bprs:CK3_32030	K03437	spoU	RNA methyltransferase, TrmH family	--	--

[denovogenes]_103964	42.86	3e-41	dto:TOL2_C28120	K13954	yiaY	alcohol dehydrogenase	1.1.1.1	Metabolism--Carbohydrate metabolism--Glycolysis / Gluconeogenesis|Metabolism--Lipid metabolism--Fatty acid degradation|Metabolism--Amino acid metabolism--Tyrosine metabolism|Metabolism--Xenobiotics biodegradation and metabolism--Chloroalkane and chloroalkene degradation|Metabolism--Xenobiotics biodegradation and metabolism--Naphthalene degradation|Metabolism--Global and overview maps--Metabolic pathways|Metabolism--Global and overview maps--Biosynthesis of secondary metabolites|Metabolism--Global and overview maps--Microbial metabolism in diverse environments|Metabolism--Global and overview maps--Biosynthesis of antibiotics|Metabolism--Global and overview maps--Degradation of aromatic compounds

[denovogenes]_103968	97.37	0.0	bdh:GV66_04755	K01585	speA	arginine decarboxylase	4.1.1.19	Metabolism--Amino acid metabolism--Arginine and proline metabolism|Metabolism--Global and overview maps--Metabolic pathways

[denovogenes]_103969	40.00	2e-23	psab:PSAB_22770	K18967	dge1	diguanylate cyclase	2.7.7.65	--

[denovogenes]_10397	50.22	0.0	afn:Acfer_1239	K03527	ispH, lytB	4-hydroxy-3-methylbut-2-en-1-yl diphosphate reductase	1.17.7.4	Metabolism--Metabolism of terpenoids and polyketides--Terpenoid backbone biosynthesis|Metabolism--Global and overview maps--Metabolic pathways|Metabolism--Global and overview maps--Biosynthesis of secondary metabolites|Metabolism--Global and overview maps--Biosynthesis of antibiotics

[denovogenes]_10397	50.22	0.0	afn:Acfer_1239	K02945	RP-S1, rpsA	small subunit ribosomal protein S1	--	Genetic Information Processing--Translation--Ribosome

[denovogenes]_103970	48.94	1e-78	bal:BACI_c33990	K07273	acm	lysozyme	--	--

[denovogenes]_103971	63.52	2e-110	cst:CLOST_2489	K01990	ABC-2.A	ABC-2 type transport system ATP-binding protein	--	--

[denovogenes]_103972	94.72	0.0	cfd:CFNIH1_05975	K03292	TC.GPH	glycoside/pentoside/hexuronide:cation symporter, GPH family	--	--

[denovogenes]_103973	87.59	2e-168	lacy:A4V08_25590	K02687	prmA	ribosomal protein L11 methyltransferase	2.1.1.-	--

[denovogenes]_103974	78.87	1e-150	ibu:IB211_02706	K07444	ypsC	putative N6-adenine-specific DNA methylase	2.1.1.-	--

[denovogenes]_103975	50.76	3e-85	esu:EUS_21700	K02945	RP-S1, rpsA	small subunit ribosomal protein S1	--	Genetic Information Processing--Translation--Ribosome

[denovogenes]_103976	56.98	7e-108	csb:CLSA_c25150	K03148	thiF	sulfur carrier protein ThiS adenylyltransferase	2.7.7.73	Metabolism--Metabolism of cofactors and vitamins--Thiamine metabolism|Metabolism--Global and overview maps--Metabolic pathways|Genetic Information Processing--Folding, sorting and degradation--Sulfur relay system

[denovogenes]_103977	90.57	2e-173	bdh:GV66_17880	K02069	ABC.X2.P	putative ABC transport system permease protein	--	--

[denovogenes]_103978	40.23	3e-61	pmw:B2K_26165	K10709	frlC	protein FrlC	--	--

[denovogenes]_103981	46.96	6e-72	cso:CLS_31800	K01104	E3.1.3.48	protein-tyrosine phosphatase	3.1.3.48	--

[denovogenes]_103982	41.63	4e-55	llm:llmg_0412	K00784	rnz	ribonuclease Z	3.1.26.11	Genetic Information Processing--Translation--RNA transport

[denovogenes]_103987	57.31	1e-98	csh:Closa_0785	K06173	truA, PUS1	tRNA pseudouridine38-40 synthase	5.4.99.12	--

[denovogenes]_103988	47.50	1e-75	rus:RBI_I01479	K11105	cvrA, nhaP2	cell volume regulation protein A	--	--

[denovogenes]_103992	60.45	1e-102	ibu:IB211_02290c	K10540	mglB	methyl-galactoside transport system substrate-binding protein	--	Environmental Information Processing--Membrane transport--ABC transporters|Cellular Processes--Cell motility--Bacterial chemotaxis

[denovogenes]_103994	43.65	1e-43	hau:Haur_2699	K07814	K07814	putative two-component system response regulator	--	--

[denovogenes]_103996	96.59	0.0	cfd:CFNIH1_18830	K01591	pyrF	orotidine-5'-phosphate decarboxylase	4.1.1.23	Metabolism--Nucleotide metabolism--Pyrimidine metabolism|Metabolism--Global and overview maps--Metabolic pathways

[denovogenes]_103998	74.34	1e-146	bprs:CK3_09430	K01267	DNPEP	aspartyl aminopeptidase	3.4.11.21	--

[denovogenes]_103999	89.47	4e-176	cle:Clole_3655	K13542	cobA-hemD	uroporphyrinogen III methyltransferase / synthase	2.1.1.107 4.2.1.75	Metabolism--Metabolism of cofactors and vitamins--Porphyrin and chlorophyll metabolism|Metabolism--Global and overview maps--Metabolic pathways|Metabolism--Global and overview maps--Biosynthesis of secondary metabolites

[denovogenes]_10400	71.80	0.0	csh:Closa_3448	K02400	flhA	flagellar biosynthesis protein FlhA	--	Cellular Processes--Cell motility--Flagellar assembly

[denovogenes]_104000	83.65	6e-149	csh:Closa_4173	K09816	znuB	zinc transport system permease protein	--	Environmental Information Processing--Membrane transport--ABC transporters

[denovogenes]_104002	99.62	0.0	btho:Btheta7330_01939	K11070	potC	spermidine/putrescine transport system permease protein	--	Environmental Information Processing--Membrane transport--ABC transporters

[denovogenes]_104004	66.67	5e-122	ibu:IB211_01389c	K00705	malQ	4-alpha-glucanotransferase	2.4.1.25	Metabolism--Carbohydrate metabolism--Starch and sucrose metabolism|Metabolism--Global and overview maps--Metabolic pathways

[denovogenes]_104006	73.76	5e-137	ibu:IB211_02930c	K00975	glgC	glucose-1-phosphate adenylyltransferase	2.7.7.27	Metabolism--Carbohydrate metabolism--Starch and sucrose metabolism|Metabolism--Carbohydrate metabolism--Amino sugar and nucleotide sugar metabolism|Metabolism--Global and overview maps--Metabolic pathways|Metabolism--Global and overview maps--Biosynthesis of secondary metabolites|Cellular Processes--Cellular community - prokaryotes--Biofilm formation - Escherichia coli

[denovogenes]_104009	96.98	0.0	cfd:CFNIH1_03915	K01791	wecB	UDP-N-acetylglucosamine 2-epimerase (non-hydrolysing)	5.1.3.14	Metabolism--Carbohydrate metabolism--Amino sugar and nucleotide sugar metabolism|Metabolism--Global and overview maps--Metabolic pathways|Cellular Processes--Cellular community - prokaryotes--Biofilm formation - Vibrio cholerae

[denovogenes]_10401	88.45	0.0	bdh:GV66_01665	K00937	ppk	polyphosphate kinase	2.7.4.1	Metabolism--Energy metabolism--Oxidative phosphorylation|Genetic Information Processing--Folding, sorting and degradation--RNA degradation

[denovogenes]_104012	77.70	2e-67	ssil:SOLI23_02120	K02406	fliC	flagellin	--	Environmental Information Processing--Signal transduction--Two-component system|Cellular Processes--Cell motility--Flagellar assembly|Organismal Systems--Immune system--NOD-like receptor signaling pathway|Organismal Systems--Environmental adaptation--Plant-pathogen interaction|Human Diseases--Infectious diseases: Bacterial--Salmonella infection|Human Diseases--Infectious diseases: Bacterial--Legionellosis

[denovogenes]_104013	89.55	1e-136	bprs:CK3_29190	K03613	rnfE	electron transport complex protein RnfE	--	--

[denovogenes]_104016	71.97	1e-144	cct:CC1_16080	K02836	prfB	peptide chain release factor 2	--	--

[denovogenes]_104017	62.78	5e-111	ped:ING2D1G_1402	K03522	fixB, etfA	electron transfer flavoprotein alpha subunit	--	--

[denovogenes]_104018	92.86	1e-180	bhl:Bache_3334	K01847	MUT	methylmalonyl-CoA mutase	5.4.99.2	Metabolism--Amino acid metabolism--Valine, leucine and isoleucine degradation|Metabolism--Carbohydrate metabolism--Glyoxylate and dicarboxylate metabolism|Metabolism--Carbohydrate metabolism--Propanoate metabolism|Metabolism--Energy metabolism--Carbon fixation pathways in prokaryotes|Metabolism--Global and overview maps--Metabolic pathways|Metabolism--Global and overview maps--Microbial metabolism in diverse environments|Metabolism--Global and overview maps--Carbon metabolism

[denovogenes]_10402	68.36	0.0	rim:ROI_19270	K01811	xylS, yicI	alpha-D-xyloside xylohydrolase	3.2.1.177	--

[denovogenes]_104020	99.25	0.0	cif:AL515_04880	K03686	dnaJ	molecular chaperone DnaJ	--	--

[denovogenes]_104021	46.49	3e-74	ele:Elen_0991	K01451	hipO	hippurate hydrolase	3.5.1.32	Metabolism--Amino acid metabolism--Phenylalanine metabolism

[denovogenes]_104024	66.80	2e-118	rch:RUM_07780	K02314	dnaB	replicative DNA helicase	3.6.4.12	Genetic Information Processing--Replication and repair--DNA replication|Cellular Processes--Cell growth and death--Cell cycle - Caulobacter

[denovogenes]_104026	98.50	0.0	cif:AL515_18410	K06176	truD, PUS7	tRNA pseudouridine13 synthase	5.4.99.27	--

[denovogenes]_104028	54.23	2e-91	dec:DCF50_p2931	K00595	cobL	precorrin-6Y C5,15-methyltransferase (decarboxylating)	2.1.1.132	Metabolism--Metabolism of cofactors and vitamins--Porphyrin and chlorophyll metabolism|Metabolism--Global and overview maps--Metabolic pathways

[denovogenes]_104029	62.92	1e-114	ssu:SSU05_1444	K00688	PYG, glgP	glycogen phosphorylase	2.4.1.1	Metabolism--Carbohydrate metabolism--Starch and sucrose metabolism|Metabolism--Global and overview maps--Metabolic pathways|Metabolism--Global and overview maps--Biosynthesis of secondary metabolites|Cellular Processes--Cellular community - prokaryotes--Biofilm formation - Escherichia coli|Organismal Systems--Endocrine system--Insulin signaling pathway|Organismal Systems--Endocrine system--Glucagon signaling pathway|Human Diseases--Endocrine and metabolic diseases--Insulin resistance

[denovogenes]_10403	51.61	0.0	ccel:CCDG5_1288	K12132	prkC, stkP	eukaryotic-like serine/threonine-protein kinase	2.7.11.1	--

[denovogenes]_104030	100.00	0.0	mod:AS202_10870	K18221	tetX	tetracycline 11a-monooxygenase, tetracycline resistance protein	1.14.13.231	Metabolism--Metabolism of terpenoids and polyketides--Tetracycline biosynthesis|Metabolism--Global and overview maps--Biosynthesis of antibiotics

[denovogenes]_104033	79.32	5e-140	rum:CK1_25450	K06901	pbuG	putative MFS transporter, AGZA family, xanthine/uracil permease	--	--

[denovogenes]_104036	45.77	4e-69	cpy:Cphy_3850	K02406	fliC	flagellin	--	Environmental Information Processing--Signal transduction--Two-component system|Cellular Processes--Cell motility--Flagellar assembly|Organismal Systems--Immune system--NOD-like receptor signaling pathway|Organismal Systems--Environmental adaptation--Plant-pathogen interaction|Human Diseases--Infectious diseases: Bacterial--Salmonella infection|Human Diseases--Infectious diseases: Bacterial--Legionellosis

[denovogenes]_104038	100.00	0.0	ecr:ECIAI1_0701	K01903	sucC	succinyl-CoA synthetase beta subunit	6.2.1.5	Metabolism--Carbohydrate metabolism--Citrate cycle (TCA cycle)|Metabolism--Carbohydrate metabolism--Propanoate metabolism|Metabolism--Carbohydrate metabolism--C5-Branched dibasic acid metabolism|Metabolism--Energy metabolism--Carbon fixation pathways in prokaryotes|Metabolism--Global and overview maps--Metabolic pathways|Metabolism--Global and overview maps--Biosynthesis of secondary metabolites|Metabolism--Global and overview maps--Microbial metabolism in diverse environments|Metabolism--Global and overview maps--Biosynthesis of antibiotics|Metabolism--Global and overview maps--Carbon metabolism

[denovogenes]_104039	71.24	3e-113	rum:CK1_00610	K03168	topA	DNA topoisomerase I	5.99.1.2	--

[denovogenes]_104042	47.22	1e-42	ccel:CCDG5_0050	K05366	mrcA	penicillin-binding protein 1A	2.4.1.- 3.4.-.-	Metabolism--Glycan biosynthesis and metabolism--Peptidoglycan biosynthesis|Metabolism--Global and overview maps--Metabolic pathways|Human Diseases--Drug resistance: Antimicrobial--beta-Lactam resistance

[denovogenes]_104043	68.68	3e-129	pfre:RM25_1083	K01126	E3.1.4.46, glpQ, ugpQ	glycerophosphoryl diester phosphodiesterase	3.1.4.46	Metabolism--Lipid metabolism--Glycerophospholipid metabolism

[denovogenes]_104044	85.98	2e-162	pfre:RM25_1031	K01575	alsD, budA, aldC	acetolactate decarboxylase	4.1.1.5	Metabolism--Carbohydrate metabolism--Butanoate metabolism|Metabolism--Carbohydrate metabolism--C5-Branched dibasic acid metabolism

[denovogenes]_104047	74.72	4e-135	afn:Acfer_0336	K11070	potC	spermidine/putrescine transport system permease protein	--	Environmental Information Processing--Membrane transport--ABC transporters

[denovogenes]_104048	98.02	0.0	btho:Btheta7330_02631	K07001	K07001	NTE family protein	--	--

[denovogenes]_10405	62.04	0.0	ibu:IB211_03259c	K01186	NEU1	sialidase-1	3.2.1.18	Metabolism--Glycan biosynthesis and metabolism--Other glycan degradation|Metabolism--Lipid metabolism--Sphingolipid metabolism|Cellular Processes--Transport and catabolism--Lysosome

[denovogenes]_104052	71.48	5e-138	pdi:BDI_1822	K07387	K07387	putative metalloprotease	3.4.24.-	--

[denovogenes]_104053	47.72	4e-43	ddn:DND132_1518	K03561	exbB	biopolymer transport protein ExbB	--	--

[denovogenes]_104054	100.00	0.0	paec:M802_5637	K09690	ABC-2.LPSE.P	lipopolysaccharide transport system permease protein	--	Environmental Information Processing--Membrane transport--ABC transporters

[denovogenes]_104057	84.91	1e-165	bcel:BcellWH2_01795	K01991	ABC-2.OM, wza	polysaccharide biosynthesis/export protein	--	Cellular Processes--Cellular community - prokaryotes--Biofilm formation - Escherichia coli

[denovogenes]_10406	68.65	0.0	lacy:A4V08_06410	K02027	ABC.MS.S	multiple sugar transport system substrate-binding protein	--	--

[denovogenes]_104062	100.00	0.0	paes:SCV20265_0397	K03149	thiG	thiazole synthase	2.8.1.10	Metabolism--Metabolism of cofactors and vitamins--Thiamine metabolism|Metabolism--Global and overview maps--Metabolic pathways

[denovogenes]_104063	100.00	0.0	paeo:M801_3971	K00941	thiD	hydroxymethylpyrimidine/phosphomethylpyrimidine kinase	2.7.1.49 2.7.4.7	Metabolism--Metabolism of cofactors and vitamins--Thiamine metabolism|Metabolism--Global and overview maps--Metabolic pathways

[denovogenes]_104064	100.00	0.0	paec:M802_4999	K07124	K07124	uncharacterized protein	--	--

[denovogenes]_104065	50.45	3e-81	pku:AUO94_13020	K19082	braR, bceR	two-component system, OmpR family, response regulator protein BraR/BceR	--	Environmental Information Processing--Signal transduction--Two-component system

[denovogenes]_104067	49.79	1e-77	btw:BF38_996	K16698	tuaG	teichuronic acid biosynthesis glycosyltransferase TuaG	2.4.-.-	--

[denovogenes]_104068	42.11	5e-17	mph:MLP_04780	K18910	dpe, lre	D-psicose/D-tagatose/L-ribulose 3-epimerase	5.1.3.30 5.1.3.31	--

[denovogenes]_104069	95.85	1e-173	pdi:BDI_3681	K00528	fpr	ferredoxin--NADP+ reductase	1.18.1.2	--

[denovogenes]_10407	68.81	0.0	rto:RTO_21760	K03657	uvrD, pcrA	DNA helicase II / ATP-dependent DNA helicase PcrA	3.6.4.12	Genetic Information Processing--Replication and repair--Nucleotide excision repair|Genetic Information Processing--Replication and repair--Mismatch repair

[denovogenes]_104070	100.00	0.0	cfd:CFNIH1_10445	K02013	ABC.FEV.A	iron complex transport system ATP-binding protein	3.6.3.34	Environmental Information Processing--Membrane transport--ABC transporters

[denovogenes]_104072	100.00	0.0	paeo:M801_562	K01816	hyi, gip	hydroxypyruvate isomerase	5.3.1.22	Metabolism--Carbohydrate metabolism--Glyoxylate and dicarboxylate metabolism|Metabolism--Global and overview maps--Metabolic pathways

[denovogenes]_104074	47.89	1e-84	ccb:Clocel_0470	K01992	ABC-2.P	ABC-2 type transport system permease protein	--	--

[denovogenes]_104077	42.50	5e-54	dsa:Desal_1705	K20266	trbJ	type IV secretion system protein TrbJ	--	Cellular Processes--Cellular community - prokaryotes--Quorum sensing

[denovogenes]_104078	59.04	5e-100	dvg:Deval_1604	K00655	plsC	1-acyl-sn-glycerol-3-phosphate acyltransferase	2.3.1.51	Metabolism--Lipid metabolism--Glycerolipid metabolism|Metabolism--Lipid metabolism--Glycerophospholipid metabolism|Metabolism--Global and overview maps--Metabolic pathways|Metabolism--Global and overview maps--Biosynthesis of secondary metabolites

[denovogenes]_104079	63.64	1e-116	eha:Ethha_1215	K06442	tlyA	23S rRNA (cytidine1920-2'-O)/16S rRNA (cytidine1409-2'-O)-methyltransferase	2.1.1.226 2.1.1.227	--

[denovogenes]_104080	100.00	0.0	paeo:M801_5175	K02030	ABC.PA.S	polar amino acid transport system substrate-binding protein	--	--

[denovogenes]_104083	100.00	0.0	paec:M802_4288	K02013	ABC.FEV.A	iron complex transport system ATP-binding protein	3.6.3.34	Environmental Information Processing--Membrane transport--ABC transporters

[denovogenes]_104084	51.60	6e-75	bip:Bint_1873	K02050	ABC.SN.P	NitT/TauT family transport system permease protein	--	--

[denovogenes]_104085	63.08	2e-112	dvg:Deval_0007	K09729	K09729	uncharacterized protein	--	--

[denovogenes]_104086	75.00	7e-140	rho:RHOM_13425	K00941	thiD	hydroxymethylpyrimidine/phosphomethylpyrimidine kinase	2.7.1.49 2.7.4.7	Metabolism--Metabolism of cofactors and vitamins--Thiamine metabolism|Metabolism--Global and overview maps--Metabolic pathways

[denovogenes]_104090	100.00	0.0	cfd:CFNIH1_05755	K02848	waaP, rfaP	heptose I phosphotransferase	2.7.1.-	Metabolism--Glycan biosynthesis and metabolism--Lipopolysaccharide biosynthesis|Metabolism--Global and overview maps--Metabolic pathways

[denovogenes]_104091	40.32	2e-16	rgi:RGI145_22210	K18910	dpe, lre	D-psicose/D-tagatose/L-ribulose 3-epimerase	5.1.3.30 5.1.3.31	--

[denovogenes]_104092	41.29	7e-58	abo:ABO_1111	K01073	E3.1.2.20	acyl-CoA hydrolase	3.1.2.20	--

[denovogenes]_104093	66.79	2e-130	dvm:DvMF_0520	K02050	ABC.SN.P	NitT/TauT family transport system permease protein	--	--

[denovogenes]_104094	41.35	9e-22	lal:AT746_04170	K02477	K02477	two-component system, LytT family, response regulator	--	--

[denovogenes]_104095	91.76	5e-170	pdi:BDI_0488	K00930	argB	acetylglutamate kinase	2.7.2.8	Metabolism--Amino acid metabolism--Arginine biosynthesis|Metabolism--Global and overview maps--Metabolic pathways|Metabolism--Global and overview maps--Biosynthesis of secondary metabolites|Metabolism--Global and overview maps--Biosynthesis of antibiotics|Metabolism--Global and overview maps--2-Oxocarboxylic acid metabolism|Metabolism--Global and overview maps--Biosynthesis of amino acids

[denovogenes]_104096	99.25	0.0	cfd:CFNIH1_24120	K05817	hcaR	LysR family transcriptional regulator, hca operon transcriptional activator	--	--

[denovogenes]_104098	93.21	0.0	cif:AL515_11970	K11962	urtD	urea transport system ATP-binding protein	--	Environmental Information Processing--Membrane transport--ABC transporters

[denovogenes]_104099	98.87	0.0	fpla:A4U99_06700	K02588	nifH	nitrogenase iron protein NifH	1.18.6.1	Metabolism--Xenobiotics biodegradation and metabolism--Chloroalkane and chloroalkene degradation|Metabolism--Energy metabolism--Nitrogen metabolism|Metabolism--Global and overview maps--Metabolic pathways|Metabolism--Global and overview maps--Microbial metabolism in diverse environments

[denovogenes]_1041	88.55	0.0	pdf:CD630DERM_00660	K03043	rpoB	DNA-directed RNA polymerase subunit beta	2.7.7.6	Metabolism--Nucleotide metabolism--Purine metabolism|Metabolism--Nucleotide metabolism--Pyrimidine metabolism|Metabolism--Global and overview maps--Metabolic pathways|Genetic Information Processing--Transcription--RNA polymerase

[denovogenes]_10410	100.00	0.0	stb:SGPB_0701	K01547	kdpB	K+-transporting ATPase ATPase B chain	3.6.3.12	Environmental Information Processing--Signal transduction--Two-component system

[denovogenes]_104100	67.42	4e-135	dvg:Deval_1186	K00184	K00184	prokaryotic molybdopterin-containing oxidoreductase family, iron-sulfur binding subunit	--	--

[denovogenes]_104101	52.80	6e-86	pgt:PGTDC60_1558	K01991	ABC-2.OM, wza	polysaccharide biosynthesis/export protein	--	Cellular Processes--Cellular community - prokaryotes--Biofilm formation - Escherichia coli

[denovogenes]_104103	50.55	1e-84	eci:UTI89_C2305	K00786	E2.4.-.-	--	--	--

[denovogenes]_104105	49.06	1e-81	ste:STER_1359	K07497	K07497	putative transposase	--	--

[denovogenes]_104106	100.00	0.0	paeo:M801_4465	K02066	mlaE, linK	phospholipid/cholesterol/gamma-HCH transport system permease protein	--	Environmental Information Processing--Membrane transport--ABC transporters

[denovogenes]_104107	99.62	0.0	ecg:E2348C_3878	K02848	waaP, rfaP	heptose I phosphotransferase	2.7.1.-	Metabolism--Glycan biosynthesis and metabolism--Lipopolysaccharide biosynthesis|Metabolism--Global and overview maps--Metabolic pathways

[denovogenes]_104108	100.00	0.0	cfd:CFNIH1_13040	K02013	ABC.FEV.A	iron complex transport system ATP-binding protein	3.6.3.34	Environmental Information Processing--Membrane transport--ABC transporters

[denovogenes]_104109	94.72	0.0	ror:RORB6_15075	K02795	PTS-Man-EIIC, manY	PTS system, mannose-specific IIC component	--	Metabolism--Carbohydrate metabolism--Fructose and mannose metabolism|Metabolism--Carbohydrate metabolism--Amino sugar and nucleotide sugar metabolism|Metabolism--Global and overview maps--Metabolic pathways|Environmental Information Processing--Membrane transport--Phosphotransferase system (PTS)

[denovogenes]_10411	43.20	2e-84	dsy:DSY3456	K02688	prpR	transcriptional regulator, propionate catabolism operon regulatory protein	--	--

[denovogenes]_104112	69.72	1e-121	spng:HMPREF1038_02172	K02795	PTS-Man-EIIC, manY	PTS system, mannose-specific IIC component	--	Metabolism--Carbohydrate metabolism--Fructose and mannose metabolism|Metabolism--Carbohydrate metabolism--Amino sugar and nucleotide sugar metabolism|Metabolism--Global and overview maps--Metabolic pathways|Environmental Information Processing--Membrane transport--Phosphotransferase system (PTS)

[denovogenes]_104115	100.00	0.0	paeo:M801_2075	K13766	liuC	methylglutaconyl-CoA hydratase	4.2.1.18	Metabolism--Amino acid metabolism--Valine, leucine and isoleucine degradation|Metabolism--Global and overview maps--Metabolic pathways

[denovogenes]_104117	58.56	3e-91	dde:Dde_0751	K07090	K07090	uncharacterized protein	--	--

[denovogenes]_104119	100.00	0.0	paeo:M801_4040	K06858	ABC.VB12.S1, btuF	vitamin B12 transport system substrate-binding protein	--	Environmental Information Processing--Membrane transport--ABC transporters

[denovogenes]_104121	99.62	0.0	paeo:M801_1860	K00208	fabI	enoyl-[acyl-carrier protein] reductase I	1.3.1.9 1.3.1.10	Metabolism--Lipid metabolism--Fatty acid biosynthesis|Metabolism--Metabolism of cofactors and vitamins--Biotin metabolism|Metabolism--Global and overview maps--Metabolic pathways|Metabolism--Global and overview maps--Fatty acid metabolism

[denovogenes]_104122	70.94	7e-135	sek:SSPA1155	K03929	pnbA	para-nitrobenzyl esterase	3.1.1.-	--

[denovogenes]_104123	99.62	0.0	lacy:A4V08_18965	K11070	potC	spermidine/putrescine transport system permease protein	--	Environmental Information Processing--Membrane transport--ABC transporters

[denovogenes]_104124	100.00	0.0	cfd:CFNIH1_15505	K03424	--	--	--	--

[denovogenes]_104125	82.64	1e-146	lacy:A4V08_10105	K02038	pstA	phosphate transport system permease protein	--	Environmental Information Processing--Membrane transport--ABC transporters

[denovogenes]_104127	100.00	0.0	paeo:M801_4682	K01776	murI	glutamate racemase	5.1.1.3	Metabolism--Metabolism of other amino acids--D-Glutamine and D-glutamate metabolism|Metabolism--Global and overview maps--Metabolic pathways

[denovogenes]_104128	54.32	1e-85	fpla:A4U99_07745	K07284	srtA	sortase A	3.4.22.70	--

[denovogenes]_104129	74.72	1e-144	csh:Closa_4102	K00111	glpA, glpD	glycerol-3-phosphate dehydrogenase	1.1.5.3	Metabolism--Lipid metabolism--Glycerophospholipid metabolism|Metabolism--Global and overview maps--Biosynthesis of secondary metabolites

[denovogenes]_10413	55.92	0.0	dvl:Dvul_2794	K08352	phsA, psrA	thiosulfate reductase / polysulfide reductase chain A	1.8.5.5	Metabolism--Energy metabolism--Sulfur metabolism|Metabolism--Global and overview maps--Microbial metabolism in diverse environments

[denovogenes]_104130	100.00	9e-100	paeu:BN889_02362	K05343	treS	maltose alpha-D-glucosyltransferase / alpha-amylase	5.4.99.16 3.2.1.1	Metabolism--Carbohydrate metabolism--Starch and sucrose metabolism|Metabolism--Global and overview maps--Metabolic pathways

[denovogenes]_104131	74.81	6e-143	lacy:A4V08_31215	K03496	parA, soj	chromosome partitioning protein	--	--

[denovogenes]_104132	99.25	0.0	pdi:BDI_1647	K11071	potB	spermidine/putrescine transport system permease protein	--	Environmental Information Processing--Membrane transport--ABC transporters

[denovogenes]_104134	98.49	0.0	lacy:A4V08_02665	K05833	ABC.X4.A	putative ABC transport system ATP-binding protein	--	--

[denovogenes]_104135	100.00	0.0	paeo:M801_2367	K02050	ABC.SN.P	NitT/TauT family transport system permease protein	--	--

[denovogenes]_104136	98.49	0.0	fpla:A4U99_08330	K19118	csd2, cas7	CRISPR-associated protein Csd2	--	--

[denovogenes]_104137	42.17	1e-54	llm:llmg_0412	K00784	rnz	ribonuclease Z	3.1.26.11	Genetic Information Processing--Translation--RNA transport

[denovogenes]_104139	100.00	0.0	paeu:BN889_01538	K06872	K06872	uncharacterized protein	--	--

[denovogenes]_10414	58.60	0.0	efau:EFAU085_00720	K03169	topB	DNA topoisomerase III	5.99.1.2	--

[denovogenes]_104143	45.93	3e-68	ert:EUR_11320	K06400	spoIVCA	site-specific DNA recombinase	--	--

[denovogenes]_104146	41.38	2e-60	eyy:EGYY_01810	K01992	ABC-2.P	ABC-2 type transport system permease protein	--	--

[denovogenes]_10415	99.71	0.0	bth:BT_4262	K01284	dcp	peptidyl-dipeptidase Dcp	3.4.15.5	--

[denovogenes]_104150	100.00	0.0	cfd:CFNIH1_07470	K00867	coaA	type I pantothenate kinase	2.7.1.33	Metabolism--Metabolism of cofactors and vitamins--Pantothenate and CoA biosynthesis|Metabolism--Global and overview maps--Metabolic pathways

[denovogenes]_104152	100.00	0.0	bbrc:B7019_1733	K00560	thyA, TYMS	thymidylate synthase	2.1.1.45	Metabolism--Nucleotide metabolism--Pyrimidine metabolism|Metabolism--Metabolism of cofactors and vitamins--One carbon pool by folate|Metabolism--Global and overview maps--Metabolic pathways|Human Diseases--Drug resistance: Antineoplastic--Antifolate resistance

[denovogenes]_104155	99.62	0.0	cfd:CFNIH1_15690	K00884	NAGK, nagK	N-acetylglucosamine kinase	2.7.1.59	Metabolism--Carbohydrate metabolism--Amino sugar and nucleotide sugar metabolism|Metabolism--Global and overview maps--Metabolic pathways

[denovogenes]_104156	99.25	0.0	pdi:BDI_1822	K07387	K07387	putative metalloprotease	3.4.24.-	--

[denovogenes]_104157	69.14	6e-136	lir:LAW_00513	K01995	livG	branched-chain amino acid transport system ATP-binding protein	--	Environmental Information Processing--Membrane transport--ABC transporters|Cellular Processes--Cellular community - prokaryotes--Quorum sensing

[denovogenes]_104158	78.49	6e-157	tpi:TREPR_0210	K08678	UXS1, uxs	UDP-glucuronate decarboxylase	4.1.1.35	Metabolism--Carbohydrate metabolism--Amino sugar and nucleotide sugar metabolism|Metabolism--Global and overview maps--Metabolic pathways

[denovogenes]_104159	86.09	5e-165	rto:RTO_17810	K07478	ycaJ	putative ATPase	--	--

[denovogenes]_10416	91.29	0.0	bfs:BF9343_4073	K00656	E2.3.1.54, pflD	formate C-acetyltransferase	2.3.1.54	Metabolism--Carbohydrate metabolism--Pyruvate metabolism|Metabolism--Carbohydrate metabolism--Propanoate metabolism|Metabolism--Carbohydrate metabolism--Butanoate metabolism|Metabolism--Global and overview maps--Metabolic pathways

[denovogenes]_104162	86.42	3e-175	bcel:BcellWH2_04786	K07282	pgsA, capA	poly-gamma-glutamate synthesis protein (capsule biosynthesis protein)	--	--

[denovogenes]_104163	94.72	1e-173	pdi:BDI_3681	K00528	fpr	ferredoxin--NADP+ reductase	1.18.1.2	--

[denovogenes]_104164	53.31	7e-95	tde:TDE2371	K02574	napH	ferredoxin-type protein NapH	--	--

[denovogenes]_104165	97.74	0.0	pvl:AOB99_03400	K03544	clpX, CLPX	ATP-dependent Clp protease ATP-binding subunit ClpX	--	Cellular Processes--Cell growth and death--Cell cycle - Caulobacter

[denovogenes]_104166	57.58	1e-105	cgo:Corgl_0184	K02108	ATPF0A, atpB	F-type H+-transporting ATPase subunit a	--	Metabolism--Energy metabolism--Oxidative phosphorylation|Metabolism--Energy metabolism--Photosynthesis|Metabolism--Global and overview maps--Metabolic pathways

[denovogenes]_104167	78.41	3e-153	afd:Alfi_0877	K13378	nuoCD	NADH-quinone oxidoreductase subunit C/D	1.6.5.3	Metabolism--Energy metabolism--Oxidative phosphorylation|Metabolism--Global and overview maps--Metabolic pathways

[denovogenes]_104168	78.87	1e-154	csh:Closa_1594	K08602	pepF, pepB	oligoendopeptidase F	3.4.24.-	--

[denovogenes]_104169	98.87	0.0	cfd:CFNIH1_24390	K14170	pheA	chorismate mutase / prephenate dehydratase	5.4.99.5 4.2.1.51	Metabolism--Amino acid metabolism--Phenylalanine, tyrosine and tryptophan biosynthesis|Metabolism--Global and overview maps--Metabolic pathways|Metabolism--Global and overview maps--Biosynthesis of secondary metabolites|Metabolism--Global and overview maps--Biosynthesis of antibiotics|Metabolism--Global and overview maps--Biosynthesis of amino acids

[denovogenes]_10417	42.80	2e-145	gte:GTCCBUS3UF5_18460	K00666	K00666	fatty-acyl-CoA synthase	6.2.1.-	--

[denovogenes]_104170	53.08	2e-92	psts:E05_09040	K05349	bglX	beta-glucosidase	3.2.1.21	Metabolism--Metabolism of other amino acids--Cyanoamino acid metabolism|Metabolism--Carbohydrate metabolism--Starch and sucrose metabolism|Metabolism--Biosynthesis of other secondary metabolites--Phenylpropanoid biosynthesis|Metabolism--Global and overview maps--Metabolic pathways|Metabolism--Global and overview maps--Biosynthesis of secondary metabolites

[denovogenes]_104172	100.00	0.0	bbrc:B7019_2104	K03217	yidC, spoIIIJ, OXA1, ccfA	YidC/Oxa1 family membrane protein insertase	--	Cellular Processes--Cellular community - prokaryotes--Quorum sensing|Genetic Information Processing--Folding, sorting and degradation--Protein export|Environmental Information Processing--Membrane transport--Bacterial secretion system

[denovogenes]_104173	74.80	7e-137	cbi:CLJ_B1379	K00571	E2.1.1.72	site-specific DNA-methyltransferase (adenine-specific)	2.1.1.72	--

[denovogenes]_104177	74.15	2e-130	cgo:Corgl_1750	K10040	ABC.GLN1.P	putative glutamine transport system permease protein	--	Environmental Information Processing--Membrane transport--ABC transporters

[denovogenes]_104179	99.25	0.0	pdi:BDI_3681	K00528	fpr	ferredoxin--NADP+ reductase	1.18.1.2	--

[denovogenes]_10418	71.99	0.0	bfb:VU15_20305	K00656	E2.3.1.54, pflD	formate C-acetyltransferase	2.3.1.54	Metabolism--Carbohydrate metabolism--Pyruvate metabolism|Metabolism--Carbohydrate metabolism--Propanoate metabolism|Metabolism--Carbohydrate metabolism--Butanoate metabolism|Metabolism--Global and overview maps--Metabolic pathways

[denovogenes]_104180	74.52	5e-135	cgo:Corgl_1757	K00764	purF, PPAT	amidophosphoribosyltransferase	2.4.2.14	Metabolism--Nucleotide metabolism--Purine metabolism|Metabolism--Amino acid metabolism--Alanine, aspartate and glutamate metabolism|Metabolism--Global and overview maps--Metabolic pathways|Metabolism--Global and overview maps--Biosynthesis of secondary metabolites|Metabolism--Global and overview maps--Biosynthesis of antibiotics

[denovogenes]_104181	56.60	4e-107	oho:Oweho_3155	K00548	metH, MTR	5-methyltetrahydrofolate--homocysteine methyltransferase	2.1.1.13	Metabolism--Amino acid metabolism--Cysteine and methionine metabolism|Metabolism--Metabolism of other amino acids--Selenocompound metabolism|Metabolism--Metabolism of cofactors and vitamins--One carbon pool by folate|Metabolism--Global and overview maps--Metabolic pathways|Metabolism--Global and overview maps--Biosynthesis of secondary metabolites|Metabolism--Global and overview maps--Biosynthesis of amino acids

[denovogenes]_104182	50.00	2e-72	cbz:Cbs_3037	K03798	ftsH, hflB	cell division protease FtsH	3.4.24.-	--

[denovogenes]_104185	100.00	0.0	bbru:Bbr_1132	K03284	corA	magnesium transporter	--	--

[denovogenes]_104187	43.86	5e-59	llm:llmg_0412	K00784	rnz	ribonuclease Z	3.1.26.11	Genetic Information Processing--Translation--RNA transport

[denovogenes]_104188	48.29	4e-89	mhg:MHY_06720	K01214	ISA, treX	isoamylase	3.2.1.68	Metabolism--Carbohydrate metabolism--Starch and sucrose metabolism|Metabolism--Global and overview maps--Metabolic pathways|Metabolism--Global and overview maps--Biosynthesis of secondary metabolites

[denovogenes]_10419	97.59	0.0	lacy:A4V08_20920	K17675	SUPV3L1, SUV3	ATP-dependent RNA helicase SUPV3L1/SUV3	3.6.4.13	--

[denovogenes]_104190	77.27	9e-145	bfb:VU15_02705	K03585	acrA, mexA, adeI, smeD, mtrC, cmeA	membrane fusion protein, multidrug efflux system	--	Human Diseases--Drug resistance: Antimicrobial--beta-Lactam resistance|Human Diseases--Drug resistance: Antimicrobial--Cationic antimicrobial peptide (CAMP) resistance

[denovogenes]_104191	89.06	3e-167	csh:Closa_2142	K05833	ABC.X4.A	putative ABC transport system ATP-binding protein	--	--

[denovogenes]_104194	77.87	4e-139	shi:Shel_10900	K06180	rluD	23S rRNA pseudouridine1911/1915/1917 synthase	5.4.99.23	--

[denovogenes]_104198	95.47	0.0	bvu:BVU_1859	K01676	E4.2.1.2A, fumA, fumB	fumarate hydratase, class I	4.2.1.2	Metabolism--Carbohydrate metabolism--Citrate cycle (TCA cycle)|Metabolism--Carbohydrate metabolism--Pyruvate metabolism|Metabolism--Energy metabolism--Carbon fixation pathways in prokaryotes|Metabolism--Global and overview maps--Metabolic pathways|Metabolism--Global and overview maps--Biosynthesis of secondary metabolites|Metabolism--Global and overview maps--Microbial metabolism in diverse environments|Metabolism--Global and overview maps--Biosynthesis of antibiotics|Metabolism--Global and overview maps--Carbon metabolism

[denovogenes]_104199	79.62	4e-155	bcel:BcellWH2_03589	K07137	K07137	uncharacterized protein	--	--

[denovogenes]_10420	62.08	0.0	btho:Btheta7330_00947	K09955	K09955	uncharacterized protein	--	--

[denovogenes]_104200	100.00	0.0	pdf:CD630DERM_11500	K03609	minD	septum site-determining protein MinD	--	--

[denovogenes]_104204	98.49	0.0	bna:106437233	K03696	clpC	ATP-dependent Clp protease ATP-binding subunit ClpC	--	--

[denovogenes]_104207	95.85	0.0	afd:Alfi_1509	K07462	recJ	single-stranded-DNA-specific exonuclease	3.1.-.-	Genetic Information Processing--Replication and repair--Base excision repair|Genetic Information Processing--Replication and repair--Mismatch repair|Genetic Information Processing--Replication and repair--Homologous recombination

[denovogenes]_104208	82.64	4e-166	ols:Olsu_0780	K00262	E1.4.1.4, gdhA	glutamate dehydrogenase (NADP+)	1.4.1.4	Metabolism--Amino acid metabolism--Arginine biosynthesis|Metabolism--Amino acid metabolism--Alanine, aspartate and glutamate metabolism|Metabolism--Energy metabolism--Nitrogen metabolism|Metabolism--Global and overview maps--Metabolic pathways

[denovogenes]_104209	89.85	8e-178	pvl:AOB99_08640	K07053	E3.1.3.97	3',5'-nucleoside bisphosphate phosphatase	3.1.3.97	--

[denovogenes]_10421	99.33	0.0	lacy:A4V08_06850	K02519	infB, MTIF2	translation initiation factor IF-2	--	--

[denovogenes]_104211	81.37	3e-150	byl:A4V09_21255	K02037	pstC	phosphate transport system permease protein	--	Environmental Information Processing--Membrane transport--ABC transporters

[denovogenes]_104215	89.85	0.0	bfr:BF1602	K00652	bioF	8-amino-7-oxononanoate synthase	2.3.1.47	Metabolism--Metabolism of cofactors and vitamins--Biotin metabolism|Metabolism--Global and overview maps--Metabolic pathways

[denovogenes]_104219	46.24	2e-82	csr:Cspa_c09540	K02755	PTS-Bgl-EIIA, bglF, bglP	PTS system, beta-glucoside-specific IIA component	2.7.1.-	Environmental Information Processing--Membrane transport--Phosphotransferase system (PTS)

[denovogenes]_104219	46.24	2e-82	csr:Cspa_c09540	K02756	PTS-Bgl-EIIB, bglF, bglP	PTS system, beta-glucoside-specific IIB component	2.7.1.-	Environmental Information Processing--Membrane transport--Phosphotransferase system (PTS)

[denovogenes]_104219	46.24	2e-82	csr:Cspa_c09540	K02757	PTS-Bgl-EIIC, bglF, bglP	PTS system, beta-glucoside-specific IIC component	--	Environmental Information Processing--Membrane transport--Phosphotransferase system (PTS)

[denovogenes]_10422	85.51	0.0	pdi:BDI_2189	K00937	ppk	polyphosphate kinase	2.7.4.1	Metabolism--Energy metabolism--Oxidative phosphorylation|Genetic Information Processing--Folding, sorting and degradation--RNA degradation

[denovogenes]_104220	99.62	0.0	boa:Bovatus_03748	K08218	ampG	MFS transporter, PAT family, beta-lactamase induction signal transducer AmpG	--	Human Diseases--Drug resistance: Antimicrobial--beta-Lactam resistance

[denovogenes]_104221	63.02	9e-126	hsd:SD1D_1209	K21011	pelF	polysaccharide biosynthesis protein PelF	--	Cellular Processes--Cellular community - prokaryotes--Biofilm formation - Pseudomonas aeruginosa

[denovogenes]_104228	53.73	2e-85	btc:CT43_CH0229	K06959	tex	protein Tex	--	--

[denovogenes]_104229	44.84	3e-74	aoe:Clos_2162	K01534	zntA	Cd2+/Zn2+-exporting ATPase	3.6.3.3 3.6.3.5	--

[denovogenes]_10423	94.63	0.0	lacy:A4V08_07970	K12527	ygfK	putative selenate reductase	1.97.1.9	Metabolism--Metabolism of other amino acids--Selenocompound metabolism

[denovogenes]_104230	60.98	9e-108	ssl:SS1G_04454	K01955	carB, CPA2	carbamoyl-phosphate synthase large subunit	6.3.5.5	Metabolism--Nucleotide metabolism--Pyrimidine metabolism|Metabolism--Amino acid metabolism--Alanine, aspartate and glutamate metabolism|Metabolism--Global and overview maps--Metabolic pathways

[denovogenes]_104232	73.31	4e-138	shi:Shel_01760	K01933	purM	phosphoribosylformylglycinamidine cyclo-ligase	6.3.3.1	Metabolism--Nucleotide metabolism--Purine metabolism|Metabolism--Global and overview maps--Metabolic pathways|Metabolism--Global and overview maps--Biosynthesis of secondary metabolites|Metabolism--Global and overview maps--Biosynthesis of antibiotics

[denovogenes]_104235	99.25	0.0	bbrc:B7019_0784	K00240	sdhB, frdB	succinate dehydrogenase / fumarate reductase, iron-sulfur subunit	1.3.5.1 1.3.5.4	Metabolism--Carbohydrate metabolism--Citrate cycle (TCA cycle)|Metabolism--Energy metabolism--Oxidative phosphorylation|Metabolism--Carbohydrate metabolism--Butanoate metabolism|Metabolism--Energy metabolism--Carbon fixation pathways in prokaryotes|Metabolism--Global and overview maps--Metabolic pathways|Metabolism--Global and overview maps--Biosynthesis of secondary metabolites|Metabolism--Global and overview maps--Microbial metabolism in diverse environments|Metabolism--Global and overview maps--Biosynthesis of antibiotics|Metabolism--Global and overview maps--Carbon metabolism

[denovogenes]_104239	99.62	0.0	bcel:BcellWH2_04384	K00764	purF, PPAT	amidophosphoribosyltransferase	2.4.2.14	Metabolism--Nucleotide metabolism--Purine metabolism|Metabolism--Amino acid metabolism--Alanine, aspartate and glutamate metabolism|Metabolism--Global and overview maps--Metabolic pathways|Metabolism--Global and overview maps--Biosynthesis of secondary metabolites|Metabolism--Global and overview maps--Biosynthesis of antibiotics

[denovogenes]_104240	43.98	5e-49	byl:A4V09_15410	K03406	mcp	methyl-accepting chemotaxis protein	--	Environmental Information Processing--Signal transduction--Two-component system|Cellular Processes--Cell motility--Bacterial chemotaxis

[denovogenes]_104242	43.65	1e-38	ster:AOA14_04625	K01156	res	type III restriction enzyme	3.1.21.5	--

[denovogenes]_104245	84.15	4e-164	bcel:BcellWH2_01795	K01991	ABC-2.OM, wza	polysaccharide biosynthesis/export protein	--	Cellular Processes--Cellular community - prokaryotes--Biofilm formation - Escherichia coli

[denovogenes]_104249	58.61	6e-103	bvs:BARVI_00445	K05946	E2.4.1.187, tagA	N-acetylglucosaminyldiphosphoundecaprenol N-acetyl-beta-D-mannosaminyltransferase	2.4.1.187	Cellular Processes--Cellular community - prokaryotes--Biofilm formation - Vibrio cholerae

[denovogenes]_10425	99.57	0.0	bbrs:BS27_0010	K01190	lacZ	beta-galactosidase	3.2.1.23	Metabolism--Carbohydrate metabolism--Galactose metabolism|Metabolism--Glycan biosynthesis and metabolism--Other glycan degradation|Metabolism--Lipid metabolism--Sphingolipid metabolism|Metabolism--Global and overview maps--Metabolic pathways

[denovogenes]_104253	93.58	0.0	efe:EFER_3921	K02848	waaP, rfaP	heptose I phosphotransferase	2.7.1.-	Metabolism--Glycan biosynthesis and metabolism--Lipopolysaccharide biosynthesis|Metabolism--Global and overview maps--Metabolic pathways

[denovogenes]_104254	100.00	0.0	sbo:SBO_2368	K07320	prmB	ribosomal protein L3 glutamine methyltransferase	2.1.1.298	--

[denovogenes]_104258	88.59	2e-177	trm:JO41_09890	K01040	gctB	glutaconate CoA-transferase, subunit B	2.8.3.12	Metabolism--Xenobiotics biodegradation and metabolism--Styrene degradation|Metabolism--Carbohydrate metabolism--Butanoate metabolism|Metabolism--Global and overview maps--Microbial metabolism in diverse environments

[denovogenes]_104259	95.49	0.0	vpr:Vpar_1568	K01940	argG, ASS1	argininosuccinate synthase	6.3.4.5	Metabolism--Amino acid metabolism--Arginine biosynthesis|Metabolism--Amino acid metabolism--Alanine, aspartate and glutamate metabolism|Metabolism--Global and overview maps--Metabolic pathways|Metabolism--Global and overview maps--Biosynthesis of secondary metabolites|Metabolism--Global and overview maps--Biosynthesis of antibiotics|Metabolism--Global and overview maps--Biosynthesis of amino acids|Human Diseases--Cardiovascular diseases--Fluid shear stress and atherosclerosis

[denovogenes]_104261	100.00	0.0	eoc:CE10_4545	K01671	yihT	sulfofructosephosphate aldolase	4.1.2.57	--

[denovogenes]_104262	47.17	6e-70	azi:AzCIB_1425	K03546	sbcC	exonuclease SbcC	--	--

[denovogenes]_104268	69.50	1e-133	ipo:Ilyop_0596	K01258	pepT	tripeptide aminopeptidase	3.4.11.4	--

[denovogenes]_104269	84.53	2e-168	vpr:Vpar_1561	K02031	ABC.PE.A	peptide/nickel transport system ATP-binding protein	--	Cellular Processes--Cellular community - prokaryotes--Quorum sensing

[denovogenes]_104270	97.36	0.0	boa:Bovatus_02765	K01265	map	methionyl aminopeptidase	3.4.11.18	--

[denovogenes]_104272	100.00	0.0	boa:Bovatus_02765	K01265	map	methionyl aminopeptidase	3.4.11.18	--

[denovogenes]_104275	100.00	0.0	pmr:PMI3360	K18979	queG	epoxyqueuosine reductase	1.17.99.6	--

[denovogenes]_104277	63.74	1e-113	ctyk:CTK_C13440	K07402	xdhC	xanthine dehydrogenase accessory factor	--	--

[denovogenes]_10428	83.19	0.0	ain:Acin_2341	K02355	fusA, GFM, EFG	elongation factor G	--	--

[denovogenes]_104280	55.64	1e-97	med:MELS_0368	K02346	DPO4, dinB	DNA polymerase IV	2.7.7.7	--

[denovogenes]_104281	50.00	2e-92	bhy:BHWA1_02503	K00335	nuoF	NADH-quinone oxidoreductase subunit F	1.6.5.3	Metabolism--Energy metabolism--Oxidative phosphorylation|Metabolism--Global and overview maps--Metabolic pathways

[denovogenes]_104282	98.50	0.0	pdi:BDI_3694	K02493	hemK, prmC, HEMK	release factor glutamine methyltransferase	2.1.1.297	--

[denovogenes]_104285	64.53	2e-122	ipo:Ilyop_0749	K01809	manA, MPI	mannose-6-phosphate isomerase	5.3.1.8	Metabolism--Carbohydrate metabolism--Fructose and mannose metabolism|Metabolism--Carbohydrate metabolism--Amino sugar and nucleotide sugar metabolism|Metabolism--Global and overview maps--Metabolic pathways|Metabolism--Global and overview maps--Biosynthesis of secondary metabolites|Metabolism--Global and overview maps--Biosynthesis of antibiotics

[denovogenes]_104286	45.02	3e-70	ipo:Ilyop_0668	K02204	thrB2	homoserine kinase type II	2.7.1.39	Metabolism--Amino acid metabolism--Glycine, serine and threonine metabolism|Metabolism--Global and overview maps--Metabolic pathways|Metabolism--Global and overview maps--Biosynthesis of secondary metabolites|Metabolism--Global and overview maps--Microbial metabolism in diverse environments|Metabolism--Global and overview maps--Biosynthesis of amino acids

[denovogenes]_104287	100.00	0.0	cpf:CPF_2683	K16786	ecfA1	energy-coupling factor transport system ATP-binding protein	3.6.3.-	Environmental Information Processing--Membrane transport--ABC transporters

[denovogenes]_104288	50.94	3e-94	efa:EF0272	K01223	E3.2.1.86B, bglA	6-phospho-beta-glucosidase	3.2.1.86	Metabolism--Carbohydrate metabolism--Glycolysis / Gluconeogenesis|Metabolism--Carbohydrate metabolism--Starch and sucrose metabolism

[denovogenes]_104289	44.09	2e-57	pmuc:ING2E5A_0670	K05349	bglX	beta-glucosidase	3.2.1.21	Metabolism--Metabolism of other amino acids--Cyanoamino acid metabolism|Metabolism--Carbohydrate metabolism--Starch and sucrose metabolism|Metabolism--Biosynthesis of other secondary metabolites--Phenylpropanoid biosynthesis|Metabolism--Global and overview maps--Metabolic pathways|Metabolism--Global and overview maps--Biosynthesis of secondary metabolites

[denovogenes]_10429	99.85	0.0	bfb:VU15_11890	K00937	ppk	polyphosphate kinase	2.7.4.1	Metabolism--Energy metabolism--Oxidative phosphorylation|Genetic Information Processing--Folding, sorting and degradation--RNA degradation

[denovogenes]_104290	54.79	4e-93	csh:Closa_1271	K01534	zntA	Cd2+/Zn2+-exporting ATPase	3.6.3.3 3.6.3.5	--

[denovogenes]_104291	99.25	0.0	pmr:PMI3050	K03924	moxR	MoxR-like ATPase	3.6.3.-	--

[denovogenes]_104293	100.00	0.0	shq:A0259_07175	K01546	kdpA	K+-transporting ATPase ATPase A chain	3.6.3.12	Environmental Information Processing--Signal transduction--Two-component system

[denovogenes]_10430	99.70	0.0	bfs:BF9343_1243	K01187	malZ	alpha-glucosidase	3.2.1.20	Metabolism--Carbohydrate metabolism--Galactose metabolism|Metabolism--Carbohydrate metabolism--Starch and sucrose metabolism|Metabolism--Global and overview maps--Metabolic pathways

[denovogenes]_104302	99.62	0.0	mmk:MU9_3478	K15587	nikD	nickel transport system ATP-binding protein	3.6.3.24	Environmental Information Processing--Membrane transport--ABC transporters

[denovogenes]_104303	99.62	0.0	bfb:VU15_16715	K00560	thyA, TYMS	thymidylate synthase	2.1.1.45	Metabolism--Nucleotide metabolism--Pyrimidine metabolism|Metabolism--Metabolism of cofactors and vitamins--One carbon pool by folate|Metabolism--Global and overview maps--Metabolic pathways|Human Diseases--Drug resistance: Antineoplastic--Antifolate resistance

[denovogenes]_104304	97.35	0.0	mmk:MU9_1270	K00348	nqrC	Na+-transporting NADH:ubiquinone oxidoreductase subunit C	1.6.5.8	--

[denovogenes]_104305	99.62	0.0	bfb:VU15_03780	K03118	tatC	sec-independent protein translocase protein TatC	--	Genetic Information Processing--Folding, sorting and degradation--Protein export|Environmental Information Processing--Membrane transport--Bacterial secretion system

[denovogenes]_104306	68.97	6e-142	cpf:CPF_1692	K16951	--	--	--	Metabolism--Energy metabolism--Sulfur metabolism|Metabolism--Global and overview maps--Microbial metabolism in diverse environments

[denovogenes]_104307	100.00	0.0	mmk:MU9_792	K03424	--	--	--	--

[denovogenes]_104312	92.16	9e-170	ega:AL523_07555	K09013	sufC	Fe-S cluster assembly ATP-binding protein	--	--

[denovogenes]_104315	55.56	5e-101	ctet:BN906_01087	K03616	rnfB	electron transport complex protein RnfB	--	--

[denovogenes]_104317	83.27	3e-164	cbut:ATN24_15615	K07816	E2.7.6.5X	putative GTP pyrophosphokinase	2.7.6.5	Metabolism--Nucleotide metabolism--Purine metabolism

[denovogenes]_104318	71.49	3e-122	lacy:A4V08_21225	K03763	DPO3A2, polC	DNA polymerase III subunit alpha, Gram-positive type	2.7.7.7	Metabolism--Nucleotide metabolism--Purine metabolism|Metabolism--Nucleotide metabolism--Pyrimidine metabolism|Metabolism--Global and overview maps--Metabolic pathways|Genetic Information Processing--Replication and repair--DNA replication|Genetic Information Processing--Replication and repair--Mismatch repair|Genetic Information Processing--Replication and repair--Homologous recombination

[denovogenes]_10432	47.15	0.0	lsl:LSL_1018	K00694	bcsA	cellulose synthase (UDP-forming)	2.4.1.12	Metabolism--Carbohydrate metabolism--Starch and sucrose metabolism|Metabolism--Global and overview maps--Metabolic pathways|Cellular Processes--Cellular community - prokaryotes--Biofilm formation - Escherichia coli

[denovogenes]_104320	84.03	2e-167	cbut:ATN24_15615	K07816	E2.7.6.5X	putative GTP pyrophosphokinase	2.7.6.5	Metabolism--Nucleotide metabolism--Purine metabolism

[denovogenes]_104321	100.00	0.0	bfb:VU15_10515	K02069	ABC.X2.P	putative ABC transport system permease protein	--	--

[denovogenes]_104324	70.63	1e-130	lacy:A4V08_34485	K09861	K09861	uncharacterized protein	--	--

[denovogenes]_104326	69.02	1e-131	pdf:CD630DERM_34220	K02190	cbiK	sirohydrochlorin cobaltochelatase	4.99.1.3	Metabolism--Metabolism of cofactors and vitamins--Porphyrin and chlorophyll metabolism|Metabolism--Global and overview maps--Metabolic pathways

[denovogenes]_104329	55.17	2e-100	ctet:BN906_01087	K03616	rnfB	electron transport complex protein RnfB	--	--

[denovogenes]_10433	73.26	0.0	cdg:CDBI1_11810	K01879	glyS	glycyl-tRNA synthetase beta chain	6.1.1.14	Genetic Information Processing--Translation--Aminoacyl-tRNA biosynthesis

[denovogenes]_104331	99.62	0.0	stb:SGPB_0192	K00981	E2.7.7.41, CDS1, CDS2, cdsA	phosphatidate cytidylyltransferase	2.7.7.41	Metabolism--Lipid metabolism--Glycerophospholipid metabolism|Metabolism--Global and overview maps--Metabolic pathways|Metabolism--Global and overview maps--Biosynthesis of secondary metabolites|Environmental Information Processing--Signal transduction--Phosphatidylinositol signaling system

[denovogenes]_104332	74.10	9e-141	lacy:A4V08_19020	K04477	ycdX	putative hydrolase	--	--

[denovogenes]_104335	76.36	2e-141	cbv:U729_2803	K03706	codY	transcriptional pleiotropic repressor	--	--

[denovogenes]_104336	99.24	0.0	efi:OG1RF_11257	K05896	scpA	segregation and condensation protein A	--	--

[denovogenes]_104338	80.61	3e-156	cbut:ATN24_15615	K07816	E2.7.6.5X	putative GTP pyrophosphokinase	2.7.6.5	Metabolism--Nucleotide metabolism--Purine metabolism

[denovogenes]_10434	88.37	0.0	csr:Cspa_c01850	K02355	fusA, GFM, EFG	elongation factor G	--	--

[denovogenes]_104341	100.00	0.0	stb:SGPB_0714	K02795	PTS-Man-EIIC, manY	PTS system, mannose-specific IIC component	--	Metabolism--Carbohydrate metabolism--Fructose and mannose metabolism|Metabolism--Carbohydrate metabolism--Amino sugar and nucleotide sugar metabolism|Metabolism--Global and overview maps--Metabolic pathways|Environmental Information Processing--Membrane transport--Phosphotransferase system (PTS)

[denovogenes]_104345	100.00	0.0	elo:EC042_0135	K00606	panB	3-methyl-2-oxobutanoate hydroxymethyltransferase	2.1.2.11	Metabolism--Metabolism of cofactors and vitamins--Pantothenate and CoA biosynthesis|Metabolism--Global and overview maps--Metabolic pathways|Metabolism--Global and overview maps--Biosynthesis of secondary metabolites

[denovogenes]_104348	100.00	0.0	pmr:PMI1835	K02048	cysP, sbp	sulfate transport system substrate-binding protein	--	Metabolism--Energy metabolism--Sulfur metabolism|Environmental Information Processing--Membrane transport--ABC transporters

[denovogenes]_104349	91.29	1e-166	vpr:Vpar_0007	K02470	gyrB	DNA gyrase subunit B	5.99.1.3	--

[denovogenes]_10435	93.31	0.0	cbut:ATN24_02405	K02355	fusA, GFM, EFG	elongation factor G	--	--

[denovogenes]_104350	99.62	0.0	elh:ETEC_0806	K16076	nmpC, ompD	outer membrane porin protein LC	--	--

[denovogenes]_104351	71.26	3e-138	rob:CK5_27060	K07052	K07052	uncharacterized protein	--	--

[denovogenes]_104352	89.31	2e-178	trm:JO41_09890	K01040	gctB	glutaconate CoA-transferase, subunit B	2.8.3.12	Metabolism--Xenobiotics biodegradation and metabolism--Styrene degradation|Metabolism--Carbohydrate metabolism--Butanoate metabolism|Metabolism--Global and overview maps--Microbial metabolism in diverse environments

[denovogenes]_104353	100.00	0.0	pvl:AOB99_14620	K02013	ABC.FEV.A	iron complex transport system ATP-binding protein	3.6.3.34	Environmental Information Processing--Membrane transport--ABC transporters

[denovogenes]_104355	100.00	0.0	pmr:PMI1430	K02013	ABC.FEV.A	iron complex transport system ATP-binding protein	3.6.3.34	Environmental Information Processing--Membrane transport--ABC transporters

[denovogenes]_104358	98.48	0.0	cfd:CFNIH1_10220	K00364	E1.7.1.7, guaC	GMP reductase	1.7.1.7	Metabolism--Nucleotide metabolism--Purine metabolism

[denovogenes]_10436	87.94	0.0	csb:CLSA_c01980	K02355	fusA, GFM, EFG	elongation factor G	--	--

[denovogenes]_104360	60.69	2e-111	cpy:Cphy_1177	K01628	fucA	L-fuculose-phosphate aldolase	4.1.2.17	Metabolism--Carbohydrate metabolism--Fructose and mannose metabolism|Metabolism--Global and overview maps--Microbial metabolism in diverse environments

[denovogenes]_104362	86.38	2e-173	csr:Cspa_c54150	K00978	rfbF	glucose-1-phosphate cytidylyltransferase	2.7.7.33	Metabolism--Carbohydrate metabolism--Starch and sucrose metabolism|Metabolism--Carbohydrate metabolism--Amino sugar and nucleotide sugar metabolism|Metabolism--Global and overview maps--Metabolic pathways

[denovogenes]_104363	100.00	0.0	pvl:AOB99_03915	K02013	ABC.FEV.A	iron complex transport system ATP-binding protein	3.6.3.34	Environmental Information Processing--Membrane transport--ABC transporters

[denovogenes]_104364	68.38	5e-123	rto:RTO_25290	K03149	thiG	thiazole synthase	2.8.1.10	Metabolism--Metabolism of cofactors and vitamins--Thiamine metabolism|Metabolism--Global and overview maps--Metabolic pathways

[denovogenes]_104365	40.71	2e-57	gfe:Gferi_17770	K07053	E3.1.3.97	3',5'-nucleoside bisphosphate phosphatase	3.1.3.97	--

[denovogenes]_104366	100.00	0.0	cbut:ATN24_02110	K03525	coaX	type III pantothenate kinase	2.7.1.33	Metabolism--Metabolism of cofactors and vitamins--Pantothenate and CoA biosynthesis|Metabolism--Global and overview maps--Metabolic pathways

[denovogenes]_104368	99.62	0.0	pvl:AOB99_00435	K03591	ftsN	cell division protein FtsN	--	--

[denovogenes]_104369	97.73	0.0	cfd:CFNIH1_21120	K02190	cbiK	sirohydrochlorin cobaltochelatase	4.99.1.3	Metabolism--Metabolism of cofactors and vitamins--Porphyrin and chlorophyll metabolism|Metabolism--Global and overview maps--Metabolic pathways

[denovogenes]_10437	76.92	0.0	cbt:CLH_2077	K03737	por, nifJ	pyruvate-ferredoxin/flavodoxin oxidoreductase	1.2.7.1 1.2.7.-	Metabolism--Carbohydrate metabolism--Glycolysis / Gluconeogenesis|Metabolism--Carbohydrate metabolism--Citrate cycle (TCA cycle)|Metabolism--Carbohydrate metabolism--Pyruvate metabolism|Metabolism--Carbohydrate metabolism--Butanoate metabolism|Metabolism--Energy metabolism--Carbon fixation pathways in prokaryotes|Metabolism--Global and overview maps--Metabolic pathways|Metabolism--Global and overview maps--Microbial metabolism in diverse environments|Metabolism--Global and overview maps--Biosynthesis of antibiotics|Metabolism--Global and overview maps--Carbon metabolism

[denovogenes]_104370	40.28	2e-41	ssil:SOLI23_00255	K00059	fabG	3-oxoacyl-[acyl-carrier protein] reductase	1.1.1.100	Metabolism--Lipid metabolism--Fatty acid biosynthesis|Metabolism--Metabolism of cofactors and vitamins--Biotin metabolism|Metabolism--Lipid metabolism--Biosynthesis of unsaturated fatty acids|Metabolism--Global and overview maps--Metabolic pathways|Metabolism--Global and overview maps--Fatty acid metabolism

[denovogenes]_104371	69.70	1e-135	cbv:U729_2797	K00981	E2.7.7.41, CDS1, CDS2, cdsA	phosphatidate cytidylyltransferase	2.7.7.41	Metabolism--Lipid metabolism--Glycerophospholipid metabolism|Metabolism--Global and overview maps--Metabolic pathways|Metabolism--Global and overview maps--Biosynthesis of secondary metabolites|Environmental Information Processing--Signal transduction--Phosphatidylinositol signaling system

[denovogenes]_104372	100.00	0.0	rho:RHOM_01605	K19299	aph3-III	aminoglycoside 3'-phosphotransferase III	2.7.1.95	--

[denovogenes]_104374	84.47	4e-165	csb:CLSA_c06180	K03609	minD	septum site-determining protein MinD	--	--

[denovogenes]_104375	69.70	6e-128	cbv:U729_2797	K00981	E2.7.7.41, CDS1, CDS2, cdsA	phosphatidate cytidylyltransferase	2.7.7.41	Metabolism--Lipid metabolism--Glycerophospholipid metabolism|Metabolism--Global and overview maps--Metabolic pathways|Metabolism--Global and overview maps--Biosynthesis of secondary metabolites|Environmental Information Processing--Signal transduction--Phosphatidylinositol signaling system

[denovogenes]_104377	96.59	0.0	panp:PSNIH2_23050	K07497	K07497	putative transposase	--	--

[denovogenes]_104378	45.92	2e-49	aka:TKWG_15125	K03546	sbcC	exonuclease SbcC	--	--

[denovogenes]_104379	76.52	4e-142	cbk:CLL_A0322	K01703	leuC	3-isopropylmalate/(R)-2-methylmalate dehydratase large subunit	4.2.1.33 4.2.1.35	Metabolism--Amino acid metabolism--Valine, leucine and isoleucine biosynthesis|Metabolism--Carbohydrate metabolism--C5-Branched dibasic acid metabolism|Metabolism--Global and overview maps--Metabolic pathways|Metabolism--Global and overview maps--Biosynthesis of secondary metabolites|Metabolism--Global and overview maps--2-Oxocarboxylic acid metabolism|Metabolism--Global and overview maps--Biosynthesis of amino acids

[denovogenes]_104381	72.80	7e-130	cbt:CLH_2687	K03308	TC.NSS	neurotransmitter:Na+ symporter, NSS family	--	--

[denovogenes]_104382	40.62	7e-25	btf:YBT020_14255	K20742	ykfC	gamma-D-glutamyl-L-lysine dipeptidyl-peptidase	3.4.14.13	--

[denovogenes]_104383	99.62	0.0	elo:EC042_0918	K13892	gsiA	glutathione transport system ATP-binding protein	--	Environmental Information Processing--Membrane transport--ABC transporters

[denovogenes]_104384	95.08	0.0	cfd:CFNIH1_12570	K09973	K09973	uncharacterized protein	--	--

[denovogenes]_104386	99.62	0.0	ecoh:ECRM13516_1801	K02053	ABC.SP.P	putative spermidine/putrescine transport system permease protein	--	Cellular Processes--Cellular community - prokaryotes--Quorum sensing

[denovogenes]_104389	75.09	4e-142	pdf:CD630DERM_26950	K01914	asnA	aspartate--ammonia ligase	6.3.1.1	Metabolism--Amino acid metabolism--Alanine, aspartate and glutamate metabolism|Metabolism--Metabolism of other amino acids--Cyanoamino acid metabolism|Metabolism--Global and overview maps--Metabolic pathways|Metabolism--Global and overview maps--Biosynthesis of secondary metabolites|Metabolism--Global and overview maps--Biosynthesis of amino acids

[denovogenes]_10439	67.54	0.0	rto:RTO_15340	K01214	ISA, treX	isoamylase	3.2.1.68	Metabolism--Carbohydrate metabolism--Starch and sucrose metabolism|Metabolism--Global and overview maps--Metabolic pathways|Metabolism--Global and overview maps--Biosynthesis of secondary metabolites

[denovogenes]_104391	80.62	1e-152	pdf:CD630DERM_26300	K00057	gpsA	glycerol-3-phosphate dehydrogenase (NAD(P)+)	1.1.1.94	Metabolism--Lipid metabolism--Glycerophospholipid metabolism|Metabolism--Global and overview maps--Biosynthesis of secondary metabolites

[denovogenes]_104392	96.97	0.0	cfd:CFNIH1_05695	K19354	waaH	heptose III glucuronosyltransferase	2.4.1.-	--

[denovogenes]_104394	99.60	1e-177	cif:AL515_21885	K05814	ugpA	sn-glycerol 3-phosphate transport system permease protein	--	Environmental Information Processing--Membrane transport--ABC transporters

[denovogenes]_104396	80.75	5e-157	cbk:CLL_A2058	K01226	treC	trehalose-6-phosphate hydrolase	3.2.1.93	Metabolism--Carbohydrate metabolism--Starch and sucrose metabolism

[denovogenes]_104397	66.04	1e-123	ccel:CCDG5_2019	K02026	ABC.MS.P1	multiple sugar transport system permease protein	--	--

[denovogenes]_104399	74.81	2e-135	vpr:Vpar_0348	K02824	--	--	--	--

[denovogenes]_1044	99.59	0.0	bfb:VU15_01015	K01191	E3.2.1.24	alpha-mannosidase	3.2.1.24	Metabolism--Glycan biosynthesis and metabolism--Other glycan degradation

[denovogenes]_104400	62.26	2e-114	cbt:CLH_2210	K01928	murE	UDP-N-acetylmuramoyl-L-alanyl-D-glutamate--2,6-diaminopimelate ligase	6.3.2.13	Metabolism--Amino acid metabolism--Lysine biosynthesis|Metabolism--Glycan biosynthesis and metabolism--Peptidoglycan biosynthesis

[denovogenes]_104401	71.91	4e-129	fhw:RN87_05390	K12942	abgT	aminobenzoyl-glutamate transport protein	--	--

[denovogenes]_104402	98.48	0.0	cif:AL515_01175	K13636	dsdC	LysR family transcriptional regulator, D-serine deaminase activator	--	--

[denovogenes]_104403	99.62	0.0	cbut:ATN24_14815	K07718	yesM	two-component system, sensor histidine kinase YesM	2.7.13.3	Environmental Information Processing--Signal transduction--Two-component system

[denovogenes]_104406	95.85	0.0	bxy:BXY_31940	K01051	E3.1.1.11	pectinesterase	3.1.1.11	Metabolism--Carbohydrate metabolism--Pentose and glucuronate interconversions|Metabolism--Global and overview maps--Metabolic pathways

[denovogenes]_104407	41.67	2e-39	cac:CA_C0946	K02238	comEC	competence protein ComEC	--	--

[denovogenes]_104409	62.50	2e-104	vpr:Vpar_1200	K03742	pncC	nicotinamide-nucleotide amidase	3.5.1.42	Metabolism--Metabolism of cofactors and vitamins--Nicotinate and nicotinamide metabolism

[denovogenes]_10441	72.19	0.0	csh:Closa_3448	K02400	flhA	flagellar biosynthesis protein FlhA	--	Cellular Processes--Cell motility--Flagellar assembly

[denovogenes]_104411	100.00	0.0	cif:AL515_03565	K07791	dcuA	anaerobic C4-dicarboxylate transporter DcuA	--	--

[denovogenes]_104412	52.14	6e-93	cpas:Clopa_4099	K01486	ade	adenine deaminase	3.5.4.2	Metabolism--Nucleotide metabolism--Purine metabolism|Metabolism--Global and overview maps--Metabolic pathways

[denovogenes]_104414	75.47	1e-148	cck:Ccar_16080	K00123	fdoG, fdfH	formate dehydrogenase major subunit	1.2.1.2	Metabolism--Carbohydrate metabolism--Glyoxylate and dicarboxylate metabolism|Metabolism--Energy metabolism--Methane metabolism|Metabolism--Global and overview maps--Metabolic pathways|Metabolism--Global and overview maps--Microbial metabolism in diverse environments|Metabolism--Global and overview maps--Carbon metabolism

[denovogenes]_104415	100.00	0.0	sbc:SbBS512_E2831	K00615	E2.2.1.1, tktA, tktB	transketolase	2.2.1.1	Metabolism--Carbohydrate metabolism--Pentose phosphate pathway|Metabolism--Energy metabolism--Carbon fixation in photosynthetic organisms|Metabolism--Metabolism of terpenoids and polyketides--Biosynthesis of ansamycins|Metabolism--Global and overview maps--Metabolic pathways|Metabolism--Global and overview maps--Biosynthesis of secondary metabolites|Metabolism--Global and overview maps--Microbial metabolism in diverse environments|Metabolism--Global and overview maps--Biosynthesis of antibiotics|Metabolism--Global and overview maps--Carbon metabolism|Metabolism--Global and overview maps--Biosynthesis of amino acids

[denovogenes]_104416	64.94	1e-128	cbut:ATN24_09080	K00005	gldA	glycerol dehydrogenase	1.1.1.6	Metabolism--Lipid metabolism--Glycerolipid metabolism|Metabolism--Carbohydrate metabolism--Propanoate metabolism|Metabolism--Global and overview maps--Metabolic pathways

[denovogenes]_104417	98.11	0.0	cif:AL515_20705	K01305	iadA	beta-aspartyl-dipeptidase (metallo-type)	3.4.19.-	--

[denovogenes]_10442	100.00	0.0	bdh:GV66_15885	K00937	ppk	polyphosphate kinase	2.7.4.1	Metabolism--Energy metabolism--Oxidative phosphorylation|Genetic Information Processing--Folding, sorting and degradation--RNA degradation

[denovogenes]_104420	94.72	0.0	slq:M495_06880	K06970	rlmF	23S rRNA (adenine1618-N6)-methyltransferase	2.1.1.181	--

[denovogenes]_104421	88.30	4e-170	cbk:CLL_A2562	K01887	RARS, argS	arginyl-tRNA synthetase	6.1.1.19	Genetic Information Processing--Translation--Aminoacyl-tRNA biosynthesis

[denovogenes]_104424	41.18	2e-37	blep:AL038_14115	K07552	bcr	MFS transporter, DHA1 family, bicyclomycin/chloramphenicol resistance protein	--	--

[denovogenes]_104425	80.30	1e-158	vpr:Vpar_1422	K01845	hemL	glutamate-1-semialdehyde 2,1-aminomutase	5.4.3.8	Metabolism--Metabolism of cofactors and vitamins--Porphyrin and chlorophyll metabolism|Metabolism--Global and overview maps--Metabolic pathways|Metabolism--Global and overview maps--Biosynthesis of secondary metabolites

[denovogenes]_104428	81.75	3e-157	bhl:Bache_0941	K01092	E3.1.3.25, IMPA, suhB	myo-inositol-1(or 4)-monophosphatase	3.1.3.25	Metabolism--Biosynthesis of other secondary metabolites--Streptomycin biosynthesis|Metabolism--Carbohydrate metabolism--Inositol phosphate metabolism|Metabolism--Global and overview maps--Metabolic pathways|Environmental Information Processing--Signal transduction--Phosphatidylinositol signaling system

[denovogenes]_104431	81.75	3e-155	bth:BT_3966	K02477	K02477	two-component system, LytT family, response regulator	--	--

[denovogenes]_104432	42.63	4e-43	rei:IE4771_PB00164	K14659	nodB	chitooligosaccharide deacetylase	3.5.1.-	--

[denovogenes]_104434	71.97	6e-145	afn:Acfer_1633	K05833	ABC.X4.A	putative ABC transport system ATP-binding protein	--	--

[denovogenes]_104435	53.05	1e-86	coo:CCU_03980	K08600	srtB	sortase B	3.4.22.70	--

[denovogenes]_104437	81.30	6e-161	cso:CLS_12050	K01611	speD, AMD1	S-adenosylmethionine decarboxylase	4.1.1.50	Metabolism--Amino acid metabolism--Cysteine and methionine metabolism|Metabolism--Amino acid metabolism--Arginine and proline metabolism|Metabolism--Global and overview maps--Metabolic pathways

[denovogenes]_104438	64.23	7e-122	byl:A4V09_05610	K02006	cbiO	cobalt/nickel transport system ATP-binding protein	--	Environmental Information Processing--Membrane transport--ABC transporters

[denovogenes]_104439	92.37	0.0	bhl:Bache_1262	K02528	ksgA	16S rRNA (adenine1518-N6/adenine1519-N6)-dimethyltransferase	2.1.1.182	--

[denovogenes]_104444	69.32	1e-131	rto:RTO_05600	K00286	proC	pyrroline-5-carboxylate reductase	1.5.1.2	Metabolism--Amino acid metabolism--Arginine and proline metabolism|Metabolism--Global and overview maps--Metabolic pathways|Metabolism--Global and overview maps--Biosynthesis of secondary metabolites|Metabolism--Global and overview maps--Biosynthesis of antibiotics|Metabolism--Global and overview maps--Biosynthesis of amino acids

[denovogenes]_104448	40.91	1e-59	bacw:QR42_17170	K19548	--	--	--	Metabolism--Global and overview maps--Biosynthesis of antibiotics

[denovogenes]_104451	75.19	5e-147	awo:Awo_c00480	K02566	nagD	NagD protein	--	--

[denovogenes]_104452	78.41	1e-150	fpla:A4U99_05705	K05833	ABC.X4.A	putative ABC transport system ATP-binding protein	--	--

[denovogenes]_104454	100.00	0.0	bvu:BVU_1895	K01834	PGAM, gpmA	2,3-bisphosphoglycerate-dependent phosphoglycerate mutase	5.4.2.11	Metabolism--Carbohydrate metabolism--Glycolysis / Gluconeogenesis|Metabolism--Amino acid metabolism--Glycine, serine and threonine metabolism|Metabolism--Energy metabolism--Methane metabolism|Metabolism--Global and overview maps--Metabolic pathways|Metabolism--Global and overview maps--Biosynthesis of secondary metabolites|Metabolism--Global and overview maps--Microbial metabolism in diverse environments|Metabolism--Global and overview maps--Biosynthesis of antibiotics|Metabolism--Global and overview maps--Carbon metabolism|Metabolism--Global and overview maps--Biosynthesis of amino acids|Organismal Systems--Endocrine system--Glucagon signaling pathway|Human Diseases--Cancers: Overview--Central carbon metabolism in cancer

[denovogenes]_104455	52.69	2e-90	fpla:A4U99_04195	K04486	E3.1.3.15B	histidinol-phosphatase (PHP family)	3.1.3.15	Metabolism--Amino acid metabolism--Histidine metabolism|Metabolism--Global and overview maps--Metabolic pathways|Metabolism--Global and overview maps--Biosynthesis of secondary metabolites|Metabolism--Global and overview maps--Biosynthesis of amino acids

[denovogenes]_104456	41.90	5e-49	csz:CSSP291_15420	K09861	K09861	uncharacterized protein	--	--

[denovogenes]_104457	73.73	6e-118	rch:RUM_07810	K01990	ABC-2.A	ABC-2 type transport system ATP-binding protein	--	--

[denovogenes]_104458	40.15	2e-48	eha:Ethha_2180	K03525	coaX	type III pantothenate kinase	2.7.1.33	Metabolism--Metabolism of cofactors and vitamins--Pantothenate and CoA biosynthesis|Metabolism--Global and overview maps--Metabolic pathways

[denovogenes]_104460	96.59	0.0	bxy:BXY_12570	K00560	thyA, TYMS	thymidylate synthase	2.1.1.45	Metabolism--Nucleotide metabolism--Pyrimidine metabolism|Metabolism--Metabolism of cofactors and vitamins--One carbon pool by folate|Metabolism--Global and overview maps--Metabolic pathways|Human Diseases--Drug resistance: Antineoplastic--Antifolate resistance

[denovogenes]_104461	82.93	2e-144	fpla:A4U99_09780	K00554	trmD	tRNA (guanine37-N1)-methyltransferase	2.1.1.228	--

[denovogenes]_104462	72.61	4e-131	csh:Closa_2414	K05934	E2.1.1.131, cobJ, cbiH	precorrin-3B C17-methyltransferase	2.1.1.131	Metabolism--Metabolism of cofactors and vitamins--Porphyrin and chlorophyll metabolism|Metabolism--Global and overview maps--Metabolic pathways

[denovogenes]_104463	99.24	0.0	bxy:BXY_01200	K01956	carA, CPA1	carbamoyl-phosphate synthase small subunit	6.3.5.5	Metabolism--Nucleotide metabolism--Pyrimidine metabolism|Metabolism--Amino acid metabolism--Alanine, aspartate and glutamate metabolism|Metabolism--Global and overview maps--Metabolic pathways

[denovogenes]_104466	79.46	9e-149	bprs:CK3_17830	K01715	crt	enoyl-CoA hydratase	4.2.1.17	Metabolism--Carbohydrate metabolism--Butanoate metabolism|Metabolism--Global and overview maps--Carbon metabolism

[denovogenes]_104467	93.89	0.0	bhl:Bache_1522	K02428	rdgB	XTP/dITP diphosphohydrolase	3.6.1.66	Metabolism--Nucleotide metabolism--Purine metabolism

[denovogenes]_104468	73.11	3e-129	cle:Clole_1602	K00286	proC	pyrroline-5-carboxylate reductase	1.5.1.2	Metabolism--Amino acid metabolism--Arginine and proline metabolism|Metabolism--Global and overview maps--Metabolic pathways|Metabolism--Global and overview maps--Biosynthesis of secondary metabolites|Metabolism--Global and overview maps--Biosynthesis of antibiotics|Metabolism--Global and overview maps--Biosynthesis of amino acids

[denovogenes]_104469	79.83	1e-141	lacy:A4V08_28835	K03394	cobI-cbiL	precorrin-2/cobalt-factor-2 C20-methyltransferase	2.1.1.130 2.1.1.151	Metabolism--Metabolism of cofactors and vitamins--Porphyrin and chlorophyll metabolism|Metabolism--Global and overview maps--Metabolic pathways

[denovogenes]_10447	76.16	0.0	erb:A4V01_11420	K01915	glnA, GLUL	glutamine synthetase	6.3.1.2	Metabolism--Amino acid metabolism--Arginine biosynthesis|Metabolism--Amino acid metabolism--Alanine, aspartate and glutamate metabolism|Metabolism--Carbohydrate metabolism--Glyoxylate and dicarboxylate metabolism|Metabolism--Energy metabolism--Nitrogen metabolism|Metabolism--Global and overview maps--Metabolic pathways|Metabolism--Global and overview maps--Microbial metabolism in diverse environments|Metabolism--Global and overview maps--Biosynthesis of amino acids|Environmental Information Processing--Signal transduction--Two-component system|Organismal Systems--Nervous system--Glutamatergic synapse|Organismal Systems--Nervous system--GABAergic synapse

[denovogenes]_104472	48.08	2e-75	bgy:BGLY_0274	K03478	chbG	chitin disaccharide deacetylase	3.5.1.105	--

[denovogenes]_104475	99.62	0.0	btho:Btheta7330_05093	K06153	bacA	undecaprenyl-diphosphatase	3.6.1.27	Metabolism--Glycan biosynthesis and metabolism--Peptidoglycan biosynthesis

[denovogenes]_104476	96.21	0.0	boa:Bovatus_00768	K02069	ABC.X2.P	putative ABC transport system permease protein	--	--

[denovogenes]_104477	99.62	0.0	btho:Btheta7330_02509	K02069	ABC.X2.P	putative ABC transport system permease protein	--	--

[denovogenes]_104479	40.55	6e-53	eha:Ethha_0558	K02081	agaR	DeoR family transcriptional regulator, aga operon transcriptional repressor	--	--

[denovogenes]_10448	88.23	0.0	cdg:CDBI1_00395	K02355	fusA, GFM, EFG	elongation factor G	--	--

[denovogenes]_104480	95.45	0.0	btho:Btheta7330_02509	K02069	ABC.X2.P	putative ABC transport system permease protein	--	--

[denovogenes]_104484	49.62	9e-92	tur:AT726_05830	K01992	ABC-2.P	ABC-2 type transport system permease protein	--	--

[denovogenes]_104485	99.24	0.0	bxy:BXY_00230	K11070	potC	spermidine/putrescine transport system permease protein	--	Environmental Information Processing--Membrane transport--ABC transporters

[denovogenes]_104486	99.62	0.0	shq:A0259_04435	K01611	speD, AMD1	S-adenosylmethionine decarboxylase	4.1.1.50	Metabolism--Amino acid metabolism--Cysteine and methionine metabolism|Metabolism--Amino acid metabolism--Arginine and proline metabolism|Metabolism--Global and overview maps--Metabolic pathways

[denovogenes]_104487	99.62	0.0	bvu:BVU_1950	K00560	thyA, TYMS	thymidylate synthase	2.1.1.45	Metabolism--Nucleotide metabolism--Pyrimidine metabolism|Metabolism--Metabolism of cofactors and vitamins--One carbon pool by folate|Metabolism--Global and overview maps--Metabolic pathways|Human Diseases--Drug resistance: Antineoplastic--Antifolate resistance

[denovogenes]_104488	77.27	3e-148	bvu:BVU_0703	K01991	ABC-2.OM, wza	polysaccharide biosynthesis/export protein	--	Cellular Processes--Cellular community - prokaryotes--Biofilm formation - Escherichia coli

[denovogenes]_104494	74.62	3e-145	cay:CEA_G1529	K16951	--	--	--	Metabolism--Energy metabolism--Sulfur metabolism|Metabolism--Global and overview maps--Microbial metabolism in diverse environments

[denovogenes]_104497	76.52	8e-143	cle:Clole_4128	K05833	ABC.X4.A	putative ABC transport system ATP-binding protein	--	--

[denovogenes]_104498	67.68	7e-128	bprm:CL3_18160	K02053	ABC.SP.P	putative spermidine/putrescine transport system permease protein	--	Cellular Processes--Cellular community - prokaryotes--Quorum sensing

[denovogenes]_104499	75.00	8e-124	senn:SN31241_6890	K18320	--	--	--	--

[denovogenes]_1045	99.25	0.0	mmk:MU9_910	K03582	recB	exodeoxyribonuclease V beta subunit	3.1.11.5	Genetic Information Processing--Replication and repair--Homologous recombination

[denovogenes]_10450	75.18	0.0	csb:CLSA_c22210	K03737	por, nifJ	pyruvate-ferredoxin/flavodoxin oxidoreductase	1.2.7.1 1.2.7.-	Metabolism--Carbohydrate metabolism--Glycolysis / Gluconeogenesis|Metabolism--Carbohydrate metabolism--Citrate cycle (TCA cycle)|Metabolism--Carbohydrate metabolism--Pyruvate metabolism|Metabolism--Carbohydrate metabolism--Butanoate metabolism|Metabolism--Energy metabolism--Carbon fixation pathways in prokaryotes|Metabolism--Global and overview maps--Metabolic pathways|Metabolism--Global and overview maps--Microbial metabolism in diverse environments|Metabolism--Global and overview maps--Biosynthesis of antibiotics|Metabolism--Global and overview maps--Carbon metabolism

[denovogenes]_104500	85.98	6e-168	lacy:A4V08_13910	K01470	E3.5.2.10	creatinine amidohydrolase	3.5.2.10	Metabolism--Amino acid metabolism--Arginine and proline metabolism

[denovogenes]_104508	54.83	1e-97	ibu:IB211_00192	K03424	--	--	--	--

[denovogenes]_104509	93.56	3e-162	fpla:A4U99_03260	K02417	fliNY, fliN	flagellar motor switch protein FliN/FliY	--	Cellular Processes--Cell motility--Bacterial chemotaxis|Cellular Processes--Cell motility--Flagellar assembly

[denovogenes]_10451	68.80	0.0	pdi:BDI_2948	K05970	SIAE	sialate O-acetylesterase	3.1.1.53	--

[denovogenes]_104510	100.00	0.0	ecr:ECIAI1_0132	K00606	panB	3-methyl-2-oxobutanoate hydroxymethyltransferase	2.1.2.11	Metabolism--Metabolism of cofactors and vitamins--Pantothenate and CoA biosynthesis|Metabolism--Global and overview maps--Metabolic pathways|Metabolism--Global and overview maps--Biosynthesis of secondary metabolites

[denovogenes]_104512	40.31	3e-52	euc:EC1_06480	K08600	srtB	sortase B	3.4.22.70	--

[denovogenes]_104513	46.04	8e-86	esu:EUS_14700	K03284	corA	magnesium transporter	--	--

[denovogenes]_104515	99.62	0.0	bxy:BXY_12570	K00560	thyA, TYMS	thymidylate synthase	2.1.1.45	Metabolism--Nucleotide metabolism--Pyrimidine metabolism|Metabolism--Metabolism of cofactors and vitamins--One carbon pool by folate|Metabolism--Global and overview maps--Metabolic pathways|Human Diseases--Drug resistance: Antineoplastic--Antifolate resistance

[denovogenes]_104516	100.00	0.0	btho:Btheta7330_00998	K00560	thyA, TYMS	thymidylate synthase	2.1.1.45	Metabolism--Nucleotide metabolism--Pyrimidine metabolism|Metabolism--Metabolism of cofactors and vitamins--One carbon pool by folate|Metabolism--Global and overview maps--Metabolic pathways|Human Diseases--Drug resistance: Antineoplastic--Antifolate resistance

[denovogenes]_104517	96.59	6e-180	btho:Btheta7330_01939	K11070	potC	spermidine/putrescine transport system permease protein	--	Environmental Information Processing--Membrane transport--ABC transporters

[denovogenes]_10452	100.00	0.0	paeo:M801_4623	K06200	cstA	carbon starvation protein	--	--

[denovogenes]_104520	66.67	3e-135	cpf:CPF_1692	K16951	--	--	--	Metabolism--Energy metabolism--Sulfur metabolism|Metabolism--Global and overview maps--Microbial metabolism in diverse environments

[denovogenes]_104523	42.31	3e-56	ibu:IB211_02201c	K03437	spoU	RNA methyltransferase, TrmH family	--	--

[denovogenes]_104524	40.26	1e-34	lja:Lj1g3v4140640.1	K02470	gyrB	DNA gyrase subunit B	5.99.1.3	--

[denovogenes]_104525	65.92	3e-113	csh:Closa_4162	K07238	TC.ZIP, zupT, ZRT3, ZIP2	zinc transporter, ZIP family	--	--

[denovogenes]_104526	76.05	4e-136	csh:Closa_2988	K02050	ABC.SN.P	NitT/TauT family transport system permease protein	--	--

[denovogenes]_104529	94.70	0.0	bvu:BVU_1950	K00560	thyA, TYMS	thymidylate synthase	2.1.1.45	Metabolism--Nucleotide metabolism--Pyrimidine metabolism|Metabolism--Metabolism of cofactors and vitamins--One carbon pool by folate|Metabolism--Global and overview maps--Metabolic pathways|Human Diseases--Drug resistance: Antineoplastic--Antifolate resistance

[denovogenes]_104530	90.94	3e-177	bvu:BVU_2043	K08303	K08303	putative protease	3.4.-.-	Human Diseases--Infectious diseases: Bacterial--Epithelial cell signaling in Helicobacter pylori infection

[denovogenes]_104531	99.62	0.0	ecy:ECSE_0375	K02554	mhpD	2-keto-4-pentenoate hydratase	4.2.1.80	Metabolism--Amino acid metabolism--Phenylalanine metabolism|Metabolism--Xenobiotics biodegradation and metabolism--Benzoate degradation|Metabolism--Xenobiotics biodegradation and metabolism--Dioxin degradation|Metabolism--Xenobiotics biodegradation and metabolism--Xylene degradation|Metabolism--Global and overview maps--Metabolic pathways|Metabolism--Global and overview maps--Microbial metabolism in diverse environments|Metabolism--Global and overview maps--Degradation of aromatic compounds

[denovogenes]_104532	90.26	1e-156	lacy:A4V08_26960	K02012	afuA, fbpA	iron(III) transport system substrate-binding protein	--	Environmental Information Processing--Membrane transport--ABC transporters

[denovogenes]_104533	96.23	0.0	bvu:BVU_1762	K03499	trkA, ktrA	trk system potassium uptake protein	--	--

[denovogenes]_104537	76.14	4e-154	bdh:GV66_13790	K00784	rnz	ribonuclease Z	3.1.26.11	Genetic Information Processing--Translation--RNA transport

[denovogenes]_104540	89.35	6e-178	bcel:BcellWH2_02915	K07214	fes	enterochelin esterase and related enzymes	--	--

[denovogenes]_104542	100.00	0.0	cif:AL515_08200	K01902	sucD	succinyl-CoA synthetase alpha subunit	6.2.1.5	Metabolism--Carbohydrate metabolism--Citrate cycle (TCA cycle)|Metabolism--Carbohydrate metabolism--Propanoate metabolism|Metabolism--Carbohydrate metabolism--C5-Branched dibasic acid metabolism|Metabolism--Energy metabolism--Carbon fixation pathways in prokaryotes|Metabolism--Global and overview maps--Metabolic pathways|Metabolism--Global and overview maps--Biosynthesis of secondary metabolites|Metabolism--Global and overview maps--Microbial metabolism in diverse environments|Metabolism--Global and overview maps--Biosynthesis of antibiotics|Metabolism--Global and overview maps--Carbon metabolism

[denovogenes]_104543	69.41	1e-123	ova:OBV_20690	K11071	potB	spermidine/putrescine transport system permease protein	--	Environmental Information Processing--Membrane transport--ABC transporters

[denovogenes]_104544	100.00	0.0	elu:UM146_14940	K09973	K09973	uncharacterized protein	--	--

[denovogenes]_10455	76.45	0.0	dvm:DvMF_2931	K02355	fusA, GFM, EFG	elongation factor G	--	--

[denovogenes]_104550	61.69	2e-84	bcf:bcf_00645	K03046	rpoC	DNA-directed RNA polymerase subunit beta'	2.7.7.6	Metabolism--Nucleotide metabolism--Purine metabolism|Metabolism--Nucleotide metabolism--Pyrimidine metabolism|Metabolism--Global and overview maps--Metabolic pathways|Genetic Information Processing--Transcription--RNA polymerase

[denovogenes]_104552	96.97	0.0	cfd:CFNIH1_10520	K01265	map	methionyl aminopeptidase	3.4.11.18	--

[denovogenes]_104553	66.67	3e-111	efau:EFAU085_01575	K03292	TC.GPH	glycoside/pentoside/hexuronide:cation symporter, GPH family	--	--

[denovogenes]_104554	100.00	0.0	shq:A0259_04685	K01265	map	methionyl aminopeptidase	3.4.11.18	--

[denovogenes]_104557	42.47	8e-37	par:Psyc_1596	K07665	cusR, copR, silR	two-component system, OmpR family, copper resistance phosphate regulon response regulator CusR	--	Environmental Information Processing--Signal transduction--Two-component system

[denovogenes]_104559	85.28	4e-166	bdh:GV66_15445	K03631	recN	DNA repair protein RecN (Recombination protein N)	--	--

[denovogenes]_10456	94.04	0.0	bfb:VU15_11890	K00937	ppk	polyphosphate kinase	2.7.4.1	Metabolism--Energy metabolism--Oxidative phosphorylation|Genetic Information Processing--Folding, sorting and degradation--RNA degradation

[denovogenes]_104560	41.47	7e-58	tap:GZ22_03155	K07029	dagK	diacylglycerol kinase (ATP)	2.7.1.107	Metabolism--Lipid metabolism--Glycerolipid metabolism|Metabolism--Lipid metabolism--Glycerophospholipid metabolism|Metabolism--Global and overview maps--Metabolic pathways|Metabolism--Global and overview maps--Biosynthesis of secondary metabolites

[denovogenes]_104561	46.22	1e-26	doi:FH5T_12030	K02477	K02477	two-component system, LytT family, response regulator	--	--

[denovogenes]_104562	63.20	2e-115	rto:RTO_15120	K03785	aroD	3-dehydroquinate dehydratase I	4.2.1.10	Metabolism--Amino acid metabolism--Phenylalanine, tyrosine and tryptophan biosynthesis|Metabolism--Global and overview maps--Metabolic pathways|Metabolism--Global and overview maps--Biosynthesis of secondary metabolites|Metabolism--Global and overview maps--Biosynthesis of antibiotics|Metabolism--Global and overview maps--Biosynthesis of amino acids

[denovogenes]_104564	40.00	6e-50	bvu:BVU_0570	K07133	K07133	uncharacterized protein	--	--

[denovogenes]_104565	65.27	2e-120	tte:TTE1761	K00927	PGK, pgk	phosphoglycerate kinase	2.7.2.3	Metabolism--Carbohydrate metabolism--Glycolysis / Gluconeogenesis|Metabolism--Energy metabolism--Carbon fixation in photosynthetic organisms|Metabolism--Global and overview maps--Metabolic pathways|Metabolism--Global and overview maps--Biosynthesis of secondary metabolites|Metabolism--Global and overview maps--Microbial metabolism in diverse environments|Metabolism--Global and overview maps--Biosynthesis of antibiotics|Metabolism--Global and overview maps--Carbon metabolism|Metabolism--Global and overview maps--Biosynthesis of amino acids

[denovogenes]_104566	90.05	3e-146	cko:CKO_01829	K07821	torY	trimethylamine-N-oxide reductase (cytochrome c), cytochrome c-type subunit TorY	--	--

[denovogenes]_104569	79.55	4e-152	bprs:CK3_09000	K01625	eda	2-dehydro-3-deoxyphosphogluconate aldolase / (4S)-4-hydroxy-2-oxoglutarate aldolase	4.1.2.14 4.1.3.42	Metabolism--Carbohydrate metabolism--Pentose phosphate pathway|Metabolism--Carbohydrate metabolism--Glyoxylate and dicarboxylate metabolism|Metabolism--Global and overview maps--Metabolic pathways|Metabolism--Global and overview maps--Microbial metabolism in diverse environments|Metabolism--Global and overview maps--Carbon metabolism

[denovogenes]_10457	100.00	0.0	cfd:CFNIH1_23870	K00937	ppk	polyphosphate kinase	2.7.4.1	Metabolism--Energy metabolism--Oxidative phosphorylation|Genetic Information Processing--Folding, sorting and degradation--RNA degradation

[denovogenes]_104570	52.24	2e-87	cle:Clole_4052	K06148	ABCC-BAC	ATP-binding cassette, subfamily C, bacterial	--	--

[denovogenes]_104571	52.06	9e-89	csh:Closa_2302	K01426	E3.5.1.4, amiE	amidase	3.5.1.4	Metabolism--Amino acid metabolism--Arginine and proline metabolism|Metabolism--Amino acid metabolism--Phenylalanine metabolism|Metabolism--Amino acid metabolism--Tryptophan metabolism|Metabolism--Xenobiotics biodegradation and metabolism--Aminobenzoate degradation|Metabolism--Xenobiotics biodegradation and metabolism--Styrene degradation|Metabolism--Global and overview maps--Microbial metabolism in diverse environments

[denovogenes]_104575	61.74	1e-114	tpz:Tph_c21550	K00147	proA	glutamate-5-semialdehyde dehydrogenase	1.2.1.41	Metabolism--Amino acid metabolism--Arginine and proline metabolism|Metabolism--Biosynthesis of other secondary metabolites--Carbapenem biosynthesis|Metabolism--Global and overview maps--Metabolic pathways|Metabolism--Global and overview maps--Biosynthesis of secondary metabolites|Metabolism--Global and overview maps--Biosynthesis of antibiotics|Metabolism--Global and overview maps--Biosynthesis of amino acids

[denovogenes]_104577	59.92	4e-97	lmob:BN419_1563	K00962	pnp, PNPT1	polyribonucleotide nucleotidyltransferase	2.7.7.8	Metabolism--Nucleotide metabolism--Purine metabolism|Metabolism--Nucleotide metabolism--Pyrimidine metabolism|Genetic Information Processing--Folding, sorting and degradation--RNA degradation

[denovogenes]_104579	55.47	1e-98	csq:CSCA_2395	K03701	uvrA	excinuclease ABC subunit A	--	Genetic Information Processing--Replication and repair--Nucleotide excision repair

[denovogenes]_104582	70.57	2e-125	cle:Clole_2570	K06147	ABCB-BAC	ATP-binding cassette, subfamily B, bacterial	--	--

[denovogenes]_104583	69.52	1e-116	lacy:A4V08_33170	K00014	aroE	shikimate dehydrogenase	1.1.1.25	Metabolism--Amino acid metabolism--Phenylalanine, tyrosine and tryptophan biosynthesis|Metabolism--Global and overview maps--Metabolic pathways|Metabolism--Global and overview maps--Biosynthesis of secondary metabolites|Metabolism--Global and overview maps--Biosynthesis of antibiotics|Metabolism--Global and overview maps--Biosynthesis of amino acids

[denovogenes]_104584	55.51	3e-99	lmob:BN419_1087	K06959	tex	protein Tex	--	--

[denovogenes]_104587	90.94	6e-168	cle:Clole_3329	K00088	IMPDH, guaB	IMP dehydrogenase	1.1.1.205	Metabolism--Nucleotide metabolism--Purine metabolism|Metabolism--Xenobiotics biodegradation and metabolism--Drug metabolism - other enzymes|Metabolism--Global and overview maps--Metabolic pathways|Metabolism--Global and overview maps--Biosynthesis of secondary metabolites

[denovogenes]_104588	43.91	8e-64	pdf:CD630DERM_23830	K02688	prpR	transcriptional regulator, propionate catabolism operon regulatory protein	--	--

[denovogenes]_104589	95.09	1e-180	cle:Clole_0052	K00955	cysNC	bifunctional enzyme CysN/CysC	2.7.7.4 2.7.1.25	Metabolism--Nucleotide metabolism--Purine metabolism|Metabolism--Biosynthesis of other secondary metabolites--Monobactam biosynthesis|Metabolism--Metabolism of other amino acids--Selenocompound metabolism|Metabolism--Energy metabolism--Sulfur metabolism|Metabolism--Global and overview maps--Metabolic pathways|Metabolism--Global and overview maps--Microbial metabolism in diverse environments|Metabolism--Global and overview maps--Biosynthesis of antibiotics

[denovogenes]_10459	100.00	0.0	shq:A0259_16215	K00937	ppk	polyphosphate kinase	2.7.4.1	Metabolism--Energy metabolism--Oxidative phosphorylation|Genetic Information Processing--Folding, sorting and degradation--RNA degradation

[denovogenes]_104590	69.81	8e-138	sdz:Asd1617_06023	K01811	xylS, yicI	alpha-D-xyloside xylohydrolase	3.2.1.177	--

[denovogenes]_104591	94.34	0.0	bhl:Bache_1166	K01733	thrC	threonine synthase	4.2.3.1	Metabolism--Amino acid metabolism--Glycine, serine and threonine metabolism|Metabolism--Metabolism of cofactors and vitamins--Vitamin B6 metabolism|Metabolism--Global and overview maps--Metabolic pathways|Metabolism--Global and overview maps--Biosynthesis of secondary metabolites|Metabolism--Global and overview maps--Microbial metabolism in diverse environments|Metabolism--Global and overview maps--Biosynthesis of amino acids

[denovogenes]_104598	40.46	2e-54	elm:ELI_0497	K02004	ABC.CD.P	putative ABC transport system permease protein	--	--

[denovogenes]_104600	95.83	3e-178	pfre:RM25_2202	K02049	ABC.SN.A	NitT/TauT family transport system ATP-binding protein	--	--

[denovogenes]_104603	90.11	3e-167	pfre:RM25_1506	K03969	--	--	--	--

[denovogenes]_104604	99.62	0.0	stb:SGPB_1080	K11071	potB	spermidine/putrescine transport system permease protein	--	Environmental Information Processing--Membrane transport--ABC transporters

[denovogenes]_104605	85.99	1e-125	pfr:PFREUD_12240	K02233	E2.7.8.26, cobS, cobV	adenosylcobinamide-GDP ribazoletransferase	2.7.8.26	Metabolism--Metabolism of cofactors and vitamins--Porphyrin and chlorophyll metabolism|Metabolism--Global and overview maps--Metabolic pathways

[denovogenes]_104607	96.59	5e-177	pfre:RM25_0423	K02008	cbiQ	cobalt/nickel transport system permease protein	--	Environmental Information Processing--Membrane transport--ABC transporters

[denovogenes]_10461	80.35	0.0	cgo:Corgl_0061	K02355	fusA, GFM, EFG	elongation factor G	--	--

[denovogenes]_104610	56.61	4e-98	elm:ELI_0963	K02071	metN	D-methionine transport system ATP-binding protein	--	Environmental Information Processing--Membrane transport--ABC transporters

[denovogenes]_104611	42.58	1e-52	eha:Ethha_2289	K07238	TC.ZIP, zupT, ZRT3, ZIP2	zinc transporter, ZIP family	--	--

[denovogenes]_104613	46.30	3e-49	dvg:Deval_2349	K01420	fnr	CRP/FNR family transcriptional regulator, anaerobic regulatory protein	--	--

[denovogenes]_104614	88.15	3e-119	pael:T223_01000	K03832	tonB	periplasmic protein TonB	--	--

[denovogenes]_104615	100.00	0.0	paec:M802_426	K06599	chpD	AraC family transcriptional regulator, chemosensory pili system protein ChpD	--	--

[denovogenes]_104617	44.66	7e-53	ibu:IB211_00434c	K17836	penP	beta-lactamase class A	3.5.2.6	Metabolism--Biosynthesis of other secondary metabolites--Penicillin and cephalosporin biosynthesis|Metabolism--Global and overview maps--Biosynthesis of antibiotics|Human Diseases--Drug resistance: Antimicrobial--beta-Lactam resistance

[denovogenes]_104618	73.11	6e-139	cle:Clole_4128	K05833	ABC.X4.A	putative ABC transport system ATP-binding protein	--	--

[denovogenes]_104619	50.61	1e-79	fpr:FP2_06360	K01560	E3.8.1.2	2-haloacid dehalogenase	3.8.1.2	Metabolism--Xenobiotics biodegradation and metabolism--Chlorocyclohexane and chlorobenzene degradation|Metabolism--Xenobiotics biodegradation and metabolism--Chloroalkane and chloroalkene degradation|Metabolism--Global and overview maps--Metabolic pathways|Metabolism--Global and overview maps--Microbial metabolism in diverse environments

[denovogenes]_10462	87.55	0.0	pdi:BDI_1443	K16089	TC.FEV.OM2, cirA, cfrA, hmuR	outer membrane receptor for ferrienterochelin and colicins	--	--

[denovogenes]_104620	83.71	9e-154	dfi:AXF13_01520	K02050	ABC.SN.P	NitT/TauT family transport system permease protein	--	--

[denovogenes]_104621	91.67	0.0	pdi:BDI_3749	K00560	thyA, TYMS	thymidylate synthase	2.1.1.45	Metabolism--Nucleotide metabolism--Pyrimidine metabolism|Metabolism--Metabolism of cofactors and vitamins--One carbon pool by folate|Metabolism--Global and overview maps--Metabolic pathways|Human Diseases--Drug resistance: Antineoplastic--Antifolate resistance

[denovogenes]_104622	98.10	0.0	bfb:VU15_21625	K00046	idnO	gluconate 5-dehydrogenase	1.1.1.69	--

[denovogenes]_104623	66.94	1e-118	cml:BN424_1875	K06223	dam	DNA adenine methylase	2.1.1.72	Genetic Information Processing--Replication and repair--Mismatch repair

[denovogenes]_104627	99.62	0.0	fpla:A4U99_04195	K04486	E3.1.3.15B	histidinol-phosphatase (PHP family)	3.1.3.15	Metabolism--Amino acid metabolism--Histidine metabolism|Metabolism--Global and overview maps--Metabolic pathways|Metabolism--Global and overview maps--Biosynthesis of secondary metabolites|Metabolism--Global and overview maps--Biosynthesis of amino acids

[denovogenes]_104628	99.62	0.0	paeo:M801_668	K01611	speD, AMD1	S-adenosylmethionine decarboxylase	4.1.1.50	Metabolism--Amino acid metabolism--Cysteine and methionine metabolism|Metabolism--Amino acid metabolism--Arginine and proline metabolism|Metabolism--Global and overview maps--Metabolic pathways

[denovogenes]_104629	51.33	5e-75	nth:Nther_1461	K01358	clpP, CLPP	ATP-dependent Clp protease, protease subunit	3.4.21.92	Cellular Processes--Cell growth and death--Cell cycle - Caulobacter|Organismal Systems--Aging--Longevity regulating pathway - worm

[denovogenes]_104630	99.62	0.0	fpla:A4U99_10155	K17828	pyrDI	dihydroorotate dehydrogenase (NAD+) catalytic subunit	1.3.1.14	Metabolism--Nucleotide metabolism--Pyrimidine metabolism|Metabolism--Global and overview maps--Metabolic pathways

[denovogenes]_104631	40.86	3e-51	mtc:MT1753.1	K00065	kduD	2-deoxy-D-gluconate 3-dehydrogenase	1.1.1.125	Metabolism--Carbohydrate metabolism--Pentose and glucuronate interconversions

[denovogenes]_104632	98.11	0.0	cfd:CFNIH1_15925	K02031	ABC.PE.A	peptide/nickel transport system ATP-binding protein	--	Cellular Processes--Cellular community - prokaryotes--Quorum sensing

[denovogenes]_104634	71.37	3e-139	dvm:DvMF_0861	K02029	ABC.PA.P	polar amino acid transport system permease protein	--	--

[denovogenes]_104635	56.47	9e-101	lir:LAW_00537	K02528	ksgA	16S rRNA (adenine1518-N6/adenine1519-N6)-dimethyltransferase	2.1.1.182	--

[denovogenes]_10464	94.25	0.0	mmk:MU9_3207	K11021	tccC	insecticidal toxin complex protein TccC	--	--

[denovogenes]_104640	97.73	3e-178	fpla:A4U99_16520	K02051	ABC.SN.S	NitT/TauT family transport system substrate-binding protein	--	--

[denovogenes]_104642	100.00	0.0	cfd:CFNIH1_01095	K00560	thyA, TYMS	thymidylate synthase	2.1.1.45	Metabolism--Nucleotide metabolism--Pyrimidine metabolism|Metabolism--Metabolism of cofactors and vitamins--One carbon pool by folate|Metabolism--Global and overview maps--Metabolic pathways|Human Diseases--Drug resistance: Antineoplastic--Antifolate resistance

[denovogenes]_104645	100.00	0.0	paeo:M801_351	K00560	thyA, TYMS	thymidylate synthase	2.1.1.45	Metabolism--Nucleotide metabolism--Pyrimidine metabolism|Metabolism--Metabolism of cofactors and vitamins--One carbon pool by folate|Metabolism--Global and overview maps--Metabolic pathways|Human Diseases--Drug resistance: Antineoplastic--Antifolate resistance

[denovogenes]_104646	100.00	0.0	cfd:CFNIH1_01145	K02529	lacI, galR	LacI family transcriptional regulator	--	--

[denovogenes]_104647	100.00	0.0	efq:DR75_666	K00059	fabG	3-oxoacyl-[acyl-carrier protein] reductase	1.1.1.100	Metabolism--Lipid metabolism--Fatty acid biosynthesis|Metabolism--Metabolism of cofactors and vitamins--Biotin metabolism|Metabolism--Lipid metabolism--Biosynthesis of unsaturated fatty acids|Metabolism--Global and overview maps--Metabolic pathways|Metabolism--Global and overview maps--Fatty acid metabolism

[denovogenes]_104648	99.62	0.0	paeo:M801_3189	K02065	mlaF, linL, mkl	phospholipid/cholesterol/gamma-HCH transport system ATP-binding protein	--	Environmental Information Processing--Membrane transport--ABC transporters

[denovogenes]_104649	100.00	0.0	cfd:CFNIH1_00530	K00068	srlD	sorbitol-6-phosphate 2-dehydrogenase	1.1.1.140	Metabolism--Carbohydrate metabolism--Fructose and mannose metabolism

[denovogenes]_104652	100.00	0.0	cfd:CFNIH1_22765	K00526	E1.17.4.1B, nrdB, nrdF	ribonucleoside-diphosphate reductase beta chain	1.17.4.1	Metabolism--Nucleotide metabolism--Purine metabolism|Metabolism--Nucleotide metabolism--Pyrimidine metabolism|Metabolism--Global and overview maps--Metabolic pathways

[denovogenes]_104654	98.51	0.0	paeg:AI22_29390	K19300	aph3-II	aminoglycoside 3'-phosphotransferase II	2.7.1.95	--

[denovogenes]_104655	95.38	1e-180	pdi:BDI_1708	K06158	ABCF3	ATP-binding cassette, subfamily F, member 3	--	--

[denovogenes]_104656	61.39	1e-100	dvg:Deval_1260	K02190	cbiK	sirohydrochlorin cobaltochelatase	4.99.1.3	Metabolism--Metabolism of cofactors and vitamins--Porphyrin and chlorophyll metabolism|Metabolism--Global and overview maps--Metabolic pathways

[denovogenes]_104657	100.00	0.0	ene:ENT_14720	K09692	tagG	teichoic acid transport system permease protein	--	Environmental Information Processing--Membrane transport--ABC transporters

[denovogenes]_104659	91.67	1e-161	csh:Closa_2093	K01997	livH	branched-chain amino acid transport system permease protein	--	Environmental Information Processing--Membrane transport--ABC transporters|Cellular Processes--Cellular community - prokaryotes--Quorum sensing

[denovogenes]_104660	100.00	0.0	cfd:CFNIH1_21120	K02190	cbiK	sirohydrochlorin cobaltochelatase	4.99.1.3	Metabolism--Metabolism of cofactors and vitamins--Porphyrin and chlorophyll metabolism|Metabolism--Global and overview maps--Metabolic pathways

[denovogenes]_104661	100.00	0.0	pdi:BDI_3749	K00560	thyA, TYMS	thymidylate synthase	2.1.1.45	Metabolism--Nucleotide metabolism--Pyrimidine metabolism|Metabolism--Metabolism of cofactors and vitamins--One carbon pool by folate|Metabolism--Global and overview maps--Metabolic pathways|Human Diseases--Drug resistance: Antineoplastic--Antifolate resistance

[denovogenes]_104662	94.70	0.0	lacy:A4V08_30800	K02823	pyrDII	dihydroorotate dehydrogenase electron transfer subunit	--	Metabolism--Nucleotide metabolism--Pyrimidine metabolism|Metabolism--Global and overview maps--Metabolic pathways

[denovogenes]_104666	75.00	5e-146	fpla:A4U99_01100	K02049	ABC.SN.A	NitT/TauT family transport system ATP-binding protein	--	--

[denovogenes]_104667	41.73	2e-62	caw:Q783_09675	K03521	fixA, etfB	electron transfer flavoprotein beta subunit	--	--

[denovogenes]_104669	63.14	6e-123	erb:A4V01_04270	K02003	ABC.CD.A	putative ABC transport system ATP-binding protein	--	--

[denovogenes]_10467	73.55	0.0	efau:EFAU085_01677	K01972	E6.5.1.2, ligA, ligB	DNA ligase (NAD+)	6.5.1.2	Genetic Information Processing--Replication and repair--DNA replication|Genetic Information Processing--Replication and repair--Base excision repair|Genetic Information Processing--Replication and repair--Nucleotide excision repair|Genetic Information Processing--Replication and repair--Mismatch repair

[denovogenes]_104670	79.25	3e-157	csh:Closa_0612	K00384	trxB	thioredoxin reductase (NADPH)	1.8.1.9	Metabolism--Nucleotide metabolism--Pyrimidine metabolism|Metabolism--Metabolism of other amino acids--Selenocompound metabolism

[denovogenes]_104672	99.62	0.0	paec:M802_2420	K02073	metQ	D-methionine transport system substrate-binding protein	--	Environmental Information Processing--Membrane transport--ABC transporters

[denovogenes]_104673	67.05	2e-127	cama:F384_19950	K17467	PTS-Dga-EIID, dgaD	PTS system, D-glucosaminate-specific IID component	--	Metabolism--Carbohydrate metabolism--Pentose phosphate pathway|Metabolism--Global and overview maps--Microbial metabolism in diverse environments|Environmental Information Processing--Membrane transport--Phosphotransferase system (PTS)

[denovogenes]_104675	71.81	4e-132	rho:RHOM_13425	K00941	thiD	hydroxymethylpyrimidine/phosphomethylpyrimidine kinase	2.7.1.49 2.7.4.7	Metabolism--Metabolism of cofactors and vitamins--Thiamine metabolism|Metabolism--Global and overview maps--Metabolic pathways

[denovogenes]_104676	68.16	1e-123	pmib:BB2000_3135	K03832	tonB	periplasmic protein TonB	--	--

[denovogenes]_104677	85.61	4e-153	csh:Closa_2988	K02050	ABC.SN.P	NitT/TauT family transport system permease protein	--	--

[denovogenes]_104680	61.11	1e-97	rum:CK1_22890	K02029	ABC.PA.P	polar amino acid transport system permease protein	--	--

[denovogenes]_104680	61.11	1e-97	rum:CK1_22890	K02030	ABC.PA.S	polar amino acid transport system substrate-binding protein	--	--

[denovogenes]_104683	100.00	0.0	cfd:CFNIH1_05375	K02525	kdgR	LacI family transcriptional regulator, kdg operon repressor	--	--

[denovogenes]_104684	40.27	4e-53	amr:AM1_1261	K01126	E3.1.4.46, glpQ, ugpQ	glycerophosphoryl diester phosphodiesterase	3.1.4.46	Metabolism--Lipid metabolism--Glycerophospholipid metabolism

[denovogenes]_104685	100.00	0.0	paeo:M801_3830	K05833	ABC.X4.A	putative ABC transport system ATP-binding protein	--	--

[denovogenes]_104686	98.11	0.0	bfb:VU15_10600	K01714	dapA	4-hydroxy-tetrahydrodipicolinate synthase	4.3.3.7	Metabolism--Biosynthesis of other secondary metabolites--Monobactam biosynthesis|Metabolism--Amino acid metabolism--Lysine biosynthesis|Metabolism--Global and overview maps--Metabolic pathways|Metabolism--Global and overview maps--Biosynthesis of secondary metabolites|Metabolism--Global and overview maps--Microbial metabolism in diverse environments|Metabolism--Global and overview maps--Biosynthesis of antibiotics|Metabolism--Global and overview maps--Biosynthesis of amino acids

[denovogenes]_104687	58.04	5e-106	ccl:Clocl_3407	K00721	DPM1	dolichol-phosphate mannosyltransferase	2.4.1.83	Metabolism--Glycan biosynthesis and metabolism--N-Glycan biosynthesis|Metabolism--Global and overview maps--Metabolic pathways

[denovogenes]_104689	41.60	7e-74	marr:BKP56_10825	K02099	--	--	--	--

[denovogenes]_104692	48.02	3e-69	bprm:CL3_11490	K01868	TARS, thrS	threonyl-tRNA synthetase	6.1.1.3	Genetic Information Processing--Translation--Aminoacyl-tRNA biosynthesis

[denovogenes]_104693	80.47	1e-141	pdi:BDI_1805	K04652	hypB	hydrogenase nickel incorporation protein HypB	--	--

[denovogenes]_104694	56.34	4e-99	euu:ADJ67_01210	K06173	truA, PUS1	tRNA pseudouridine38-40 synthase	5.4.99.12	--

[denovogenes]_10470	100.00	0.0	bfb:VU15_04825	K16089	TC.FEV.OM2, cirA, cfrA, hmuR	outer membrane receptor for ferrienterochelin and colicins	--	--

[denovogenes]_104701	89.11	2e-176	csh:Closa_0494	K00978	rfbF	glucose-1-phosphate cytidylyltransferase	2.7.7.33	Metabolism--Carbohydrate metabolism--Starch and sucrose metabolism|Metabolism--Carbohydrate metabolism--Amino sugar and nucleotide sugar metabolism|Metabolism--Global and overview maps--Metabolic pathways

[denovogenes]_104702	92.80	0.0	pdi:BDI_3749	K00560	thyA, TYMS	thymidylate synthase	2.1.1.45	Metabolism--Nucleotide metabolism--Pyrimidine metabolism|Metabolism--Metabolism of cofactors and vitamins--One carbon pool by folate|Metabolism--Global and overview maps--Metabolic pathways|Human Diseases--Drug resistance: Antineoplastic--Antifolate resistance

[denovogenes]_104703	56.59	1e-97	eyy:EGYY_07670	K00666	K00666	fatty-acyl-CoA synthase	6.2.1.-	--

[denovogenes]_104705	64.71	2e-74	eyy:EGYY_20440	K06864	K06864	uncharacterized protein	--	--

[denovogenes]_104706	76.89	2e-135	afd:Alfi_3023	K12267	msrAB	peptide methionine sulfoxide reductase msrA/msrB	1.8.4.11 1.8.4.12	--

[denovogenes]_104707	100.00	0.0	cif:AL515_15670	K00526	E1.17.4.1B, nrdB, nrdF	ribonucleoside-diphosphate reductase beta chain	1.17.4.1	Metabolism--Nucleotide metabolism--Purine metabolism|Metabolism--Nucleotide metabolism--Pyrimidine metabolism|Metabolism--Global and overview maps--Metabolic pathways

[denovogenes]_104709	96.60	0.0	cfd:CFNIH1_13075	K01252	entB, dhbB, vibB, mxcF	bifunctional isochorismate lyase / aryl carrier protein	3.3.2.1 6.3.2.14	Metabolism--Metabolism of terpenoids and polyketides--Biosynthesis of siderophore group nonribosomal peptides|Metabolism--Global and overview maps--Biosynthesis of secondary metabolites|Metabolism--Global and overview maps--Biosynthesis of antibiotics

[denovogenes]_104710	64.91	1e-129	ccm:Ccan_18810	K01869	LARS, leuS	leucyl-tRNA synthetase	6.1.1.4	Genetic Information Processing--Translation--Aminoacyl-tRNA biosynthesis

[denovogenes]_104711	98.11	1e-180	rto:RTO_14350	K02795	PTS-Man-EIIC, manY	PTS system, mannose-specific IIC component	--	Metabolism--Carbohydrate metabolism--Fructose and mannose metabolism|Metabolism--Carbohydrate metabolism--Amino sugar and nucleotide sugar metabolism|Metabolism--Global and overview maps--Metabolic pathways|Environmental Information Processing--Membrane transport--Phosphotransferase system (PTS)

[denovogenes]_104716	100.00	0.0	bcel:BcellWH2_02925	K00046	idnO	gluconate 5-dehydrogenase	1.1.1.69	--

[denovogenes]_104719	67.83	1e-131	ele:Elen_2224	K03813	modD	molybdenum transport protein	2.4.2.-	--

[denovogenes]_10472	99.36	0.0	ene:ENT_09160	K20073	mapZ, locZ	mid-cell-anchored protein Z	--	--

[denovogenes]_104722	100.00	0.0	cfd:CFNIH1_04935	K09811	ftsX	cell division transport system permease protein	--	Environmental Information Processing--Membrane transport--ABC transporters

[denovogenes]_104723	70.44	5e-137	bvu:BVU_3992	K01155	E3.1.21.4	type II restriction enzyme	3.1.21.4	--

[denovogenes]_104724	100.00	0.0	bbrd:BBBR_0952	K00616	E2.2.1.2, talA, talB	transaldolase	2.2.1.2	Metabolism--Carbohydrate metabolism--Pentose phosphate pathway|Metabolism--Global and overview maps--Metabolic pathways|Metabolism--Global and overview maps--Biosynthesis of secondary metabolites|Metabolism--Global and overview maps--Microbial metabolism in diverse environments|Metabolism--Global and overview maps--Biosynthesis of antibiotics|Metabolism--Global and overview maps--Carbon metabolism|Metabolism--Global and overview maps--Biosynthesis of amino acids

[denovogenes]_104726	92.08	3e-139	pmr:PMI3424	K05592	deaD, cshA	ATP-dependent RNA helicase DeaD	3.6.4.13	Genetic Information Processing--Folding, sorting and degradation--RNA degradation

[denovogenes]_104727	96.97	0.0	bcel:BcellWH2_04578	K11927	rhlE	ATP-dependent RNA helicase RhlE	3.6.4.13	Genetic Information Processing--Folding, sorting and degradation--RNA degradation

[denovogenes]_104728	83.72	3e-161	cfd:CFNIH1_03515	K01674	cah	carbonic anhydrase	4.2.1.1	Metabolism--Energy metabolism--Nitrogen metabolism

[denovogenes]_10473	77.83	0.0	gfe:Gferi_10450	K01712	hutU, UROC1	urocanate hydratase	4.2.1.49	Metabolism--Amino acid metabolism--Histidine metabolism|Metabolism--Global and overview maps--Metabolic pathways

[denovogenes]_104731	52.87	3e-92	lacy:A4V08_19540	K10907	K10907	aminotransferase	2.6.1.-	--

[denovogenes]_104732	77.27	5e-143	pit:PIN17_A0458	K00962	pnp, PNPT1	polyribonucleotide nucleotidyltransferase	2.7.7.8	Metabolism--Nucleotide metabolism--Purine metabolism|Metabolism--Nucleotide metabolism--Pyrimidine metabolism|Genetic Information Processing--Folding, sorting and degradation--RNA degradation

[denovogenes]_104734	65.50	2e-123	cgo:Corgl_1223	K02031	ABC.PE.A	peptide/nickel transport system ATP-binding protein	--	Cellular Processes--Cellular community - prokaryotes--Quorum sensing

[denovogenes]_104736	88.30	2e-177	btho:Btheta7330_02107	K17716	capD	UDP-glucose 4-epimerase	5.1.3.2	Metabolism--Carbohydrate metabolism--Galactose metabolism|Metabolism--Carbohydrate metabolism--Amino sugar and nucleotide sugar metabolism|Metabolism--Global and overview maps--Metabolic pathways

[denovogenes]_104737	45.41	1e-32	rmg:Rhom172_1718	K02014	TC.FEV.OM	iron complex outermembrane recepter protein	--	--

[denovogenes]_104739	100.00	0.0	bbrs:BS27_0521	K02027	ABC.MS.S	multiple sugar transport system substrate-binding protein	--	--

[denovogenes]_10474	47.39	2e-152	ctc:CTC_01847	K17363	urdA	urocanate reductase	1.3.99.33	Metabolism--Amino acid metabolism--Histidine metabolism

[denovogenes]_104742	60.08	1e-105	ene:ENT_11230	K01955	carB, CPA2	carbamoyl-phosphate synthase large subunit	6.3.5.5	Metabolism--Nucleotide metabolism--Pyrimidine metabolism|Metabolism--Amino acid metabolism--Alanine, aspartate and glutamate metabolism|Metabolism--Global and overview maps--Metabolic pathways

[denovogenes]_104744	80.38	4e-159	cvr:CHLNCDRAFT_48385	K02355	fusA, GFM, EFG	elongation factor G	--	--

[denovogenes]_104746	44.44	3e-69	mhg:MHY_25410	K07456	mutS2	DNA mismatch repair protein MutS2	--	Genetic Information Processing--Replication and repair--Mismatch repair

[denovogenes]_104747	71.97	3e-142	pdi:BDI_1721	K05601	hcp	hydroxylamine reductase	1.7.99.1	Metabolism--Energy metabolism--Nitrogen metabolism

[denovogenes]_104749	60.46	3e-119	peo:AS203_07780	K12373	HEXA_B	hexosaminidase	3.2.1.52	Metabolism--Glycan biosynthesis and metabolism--Other glycan degradation|Metabolism--Glycan biosynthesis and metabolism--Various types of N-glycan biosynthesis|Metabolism--Carbohydrate metabolism--Amino sugar and nucleotide sugar metabolism|Metabolism--Glycan biosynthesis and metabolism--Glycosaminoglycan degradation|Metabolism--Glycan biosynthesis and metabolism--Glycosphingolipid biosynthesis - globo and isoglobo series|Metabolism--Glycan biosynthesis and metabolism--Glycosphingolipid biosynthesis - ganglio series|Metabolism--Global and overview maps--Metabolic pathways|Cellular Processes--Transport and catabolism--Lysosome

[denovogenes]_104750	40.94	6e-45	ath:AT2G01630	K19891	GN1_2_3	glucan endo-1,3-beta-glucosidase 1/2/3	3.2.1.39	Metabolism--Carbohydrate metabolism--Starch and sucrose metabolism

[denovogenes]_104754	99.62	0.0	rum:CK1_03890	K06147	ABCB-BAC	ATP-binding cassette, subfamily B, bacterial	--	--

[denovogenes]_104755	52.35	7e-87	gea:GARCT_02750	K04072	adhE	acetaldehyde dehydrogenase / alcohol dehydrogenase	1.2.1.10 1.1.1.1	Metabolism--Carbohydrate metabolism--Glycolysis / Gluconeogenesis|Metabolism--Lipid metabolism--Fatty acid degradation|Metabolism--Amino acid metabolism--Tyrosine metabolism|Metabolism--Carbohydrate metabolism--Pyruvate metabolism|Metabolism--Xenobiotics biodegradation and metabolism--Chloroalkane and chloroalkene degradation|Metabolism--Xenobiotics biodegradation and metabolism--Naphthalene degradation|Metabolism--Carbohydrate metabolism--Butanoate metabolism|Metabolism--Global and overview maps--Metabolic pathways|Metabolism--Global and overview maps--Biosynthesis of secondary metabolites|Metabolism--Global and overview maps--Microbial metabolism in diverse environments|Metabolism--Global and overview maps--Biosynthesis of antibiotics|Metabolism--Global and overview maps--Degradation of aromatic compounds

[denovogenes]_104756	64.79	1e-126	bpb:bpr_I2344	K02027	ABC.MS.S	multiple sugar transport system substrate-binding protein	--	--

[denovogenes]_104757	89.06	9e-171	sbi:SORBI_03g004320	K10999	CESA	cellulose synthase A	2.4.1.12	--

[denovogenes]_104758	40.74	3e-19	hhl:Halha_2321	K02821	PTS-Ula-EIIA, ulaC, sgaA	PTS system, ascorbate-specific IIA component	2.7.1.194	Metabolism--Carbohydrate metabolism--Ascorbate and aldarate metabolism|Metabolism--Global and overview maps--Metabolic pathways|Metabolism--Global and overview maps--Microbial metabolism in diverse environments|Environmental Information Processing--Membrane transport--Phosphotransferase system (PTS)

[denovogenes]_10476	100.00	0.0	cbut:ATN24_05060	K02400	flhA	flagellar biosynthesis protein FlhA	--	Cellular Processes--Cell motility--Flagellar assembly

[denovogenes]_104767	44.32	3e-64	sro:Sros_3086	K01845	hemL	glutamate-1-semialdehyde 2,1-aminomutase	5.4.3.8	Metabolism--Metabolism of cofactors and vitamins--Porphyrin and chlorophyll metabolism|Metabolism--Global and overview maps--Metabolic pathways|Metabolism--Global and overview maps--Biosynthesis of secondary metabolites

[denovogenes]_104768	43.02	3e-58	fpr:FP2_04210	K06147	ABCB-BAC	ATP-binding cassette, subfamily B, bacterial	--	--

[denovogenes]_10477	97.67	0.0	prg:RB151_003950	K03043	rpoB	DNA-directed RNA polymerase subunit beta	2.7.7.6	Metabolism--Nucleotide metabolism--Purine metabolism|Metabolism--Nucleotide metabolism--Pyrimidine metabolism|Metabolism--Global and overview maps--Metabolic pathways|Genetic Information Processing--Transcription--RNA polymerase

[denovogenes]_104772	85.31	1e-133	bcel:BcellWH2_02448	K03469	rnhA, RNASEH1	ribonuclease HI	3.1.26.4	Genetic Information Processing--Replication and repair--DNA replication

[denovogenes]_104774	93.18	0.0	bhl:Bache_3297	K00560	thyA, TYMS	thymidylate synthase	2.1.1.45	Metabolism--Nucleotide metabolism--Pyrimidine metabolism|Metabolism--Metabolism of cofactors and vitamins--One carbon pool by folate|Metabolism--Global and overview maps--Metabolic pathways|Human Diseases--Drug resistance: Antineoplastic--Antifolate resistance

[denovogenes]_104775	99.24	0.0	boa:Bovatus_00768	K02069	ABC.X2.P	putative ABC transport system permease protein	--	--

[denovogenes]_104776	96.30	0.0	bxy:BXY_01870	K03616	rnfB	electron transport complex protein RnfB	--	--

[denovogenes]_104779	100.00	0.0	boa:Bovatus_01301	K11070	potC	spermidine/putrescine transport system permease protein	--	Environmental Information Processing--Membrane transport--ABC transporters

[denovogenes]_10478	79.18	0.0	ain:Acin_0389	K02355	fusA, GFM, EFG	elongation factor G	--	--

[denovogenes]_104780	47.58	1e-72	vig:BKP57_18605	K07238	TC.ZIP, zupT, ZRT3, ZIP2	zinc transporter, ZIP family	--	--

[denovogenes]_104781	76.52	6e-146	euc:EC1_16030	K05833	ABC.X4.A	putative ABC transport system ATP-binding protein	--	--

[denovogenes]_104784	98.11	0.0	bxy:BXY_12570	K00560	thyA, TYMS	thymidylate synthase	2.1.1.45	Metabolism--Nucleotide metabolism--Pyrimidine metabolism|Metabolism--Metabolism of cofactors and vitamins--One carbon pool by folate|Metabolism--Global and overview maps--Metabolic pathways|Human Diseases--Drug resistance: Antineoplastic--Antifolate resistance

[denovogenes]_104785	49.58	1e-75	btw:BF38_996	K16698	tuaG	teichuronic acid biosynthesis glycosyltransferase TuaG	2.4.-.-	--

[denovogenes]_104787	69.32	5e-136	elm:ELI_2230	K16951	--	--	--	Metabolism--Energy metabolism--Sulfur metabolism|Metabolism--Global and overview maps--Microbial metabolism in diverse environments

[denovogenes]_104788	41.35	8e-54	csb:CLSA_c03190	K02598	nirC	nitrite transporter NirC	--	--

[denovogenes]_104789	100.00	0.0	eoc:CE10_0136	K00606	panB	3-methyl-2-oxobutanoate hydroxymethyltransferase	2.1.2.11	Metabolism--Metabolism of cofactors and vitamins--Pantothenate and CoA biosynthesis|Metabolism--Global and overview maps--Metabolic pathways|Metabolism--Global and overview maps--Biosynthesis of secondary metabolites

[denovogenes]_10479	60.55	0.0	ain:Acin_0387	K03655	recG	ATP-dependent DNA helicase RecG	3.6.4.12	Genetic Information Processing--Replication and repair--Homologous recombination

[denovogenes]_104790	53.64	8e-92	cbm:CBF_3750	K01776	murI	glutamate racemase	5.1.1.3	Metabolism--Metabolism of other amino acids--D-Glutamine and D-glutamate metabolism|Metabolism--Global and overview maps--Metabolic pathways

[denovogenes]_104791	100.00	0.0	ecoi:ECOPMV1_00971	K15554	ssuC	sulfonate transport system permease protein	--	Metabolism--Energy metabolism--Sulfur metabolism|Environmental Information Processing--Membrane transport--ABC transporters

[denovogenes]_104792	44.03	2e-34	ssk:SSUD12_1640	K01207	nagZ	beta-N-acetylhexosaminidase	3.2.1.52	Metabolism--Carbohydrate metabolism--Amino sugar and nucleotide sugar metabolism|Metabolism--Glycan biosynthesis and metabolism--Glycosaminoglycan degradation|Metabolism--Global and overview maps--Metabolic pathways|Human Diseases--Drug resistance: Antimicrobial--beta-Lactam resistance

[denovogenes]_104794	98.11	0.0	ecv:APECO1_3044	K02444	glpR	DeoR family transcriptional regulator, glycerol-3-phosphate regulon repressor	--	--

[denovogenes]_104796	99.61	0.0	pvl:AOB99_12515	K02081	agaR	DeoR family transcriptional regulator, aga operon transcriptional repressor	--	--

[denovogenes]_104797	63.02	4e-122	ain:Acin_1141	K14441	rimO	ribosomal protein S12 methylthiotransferase	2.8.4.4	--

[denovogenes]_104798	99.22	0.0	bvu:BVU_3887	K03612	rnfG	electron transport complex protein RnfG	--	--

[denovogenes]_104799	63.91	6e-119	efq:DR75_801	K02082	agaS	tagatose-6-phosphate ketose/aldose isomerase	5.-.-.-	--

[denovogenes]_1048	99.83	0.0	boa:Bovatus_02793	K03043	rpoB	DNA-directed RNA polymerase subunit beta	2.7.7.6	Metabolism--Nucleotide metabolism--Purine metabolism|Metabolism--Nucleotide metabolism--Pyrimidine metabolism|Metabolism--Global and overview maps--Metabolic pathways|Genetic Information Processing--Transcription--RNA polymerase

[denovogenes]_10480	56.59	0.0	bcel:BcellWH2_01185	K19049	cslA	chondroitin AC lyase	4.2.2.5	--

[denovogenes]_104801	44.35	3e-32	barc:AOA65_0262	K06015	E3.5.1.81	N-acyl-D-amino-acid deacylase	3.5.1.81	--

[denovogenes]_104802	40.64	1e-34	top:TOPB45_0498	K00124	fdoH	formate dehydrogenase iron-sulfur subunit	--	Metabolism--Carbohydrate metabolism--Glyoxylate and dicarboxylate metabolism|Metabolism--Energy metabolism--Methane metabolism|Metabolism--Global and overview maps--Metabolic pathways|Metabolism--Global and overview maps--Microbial metabolism in diverse environments|Metabolism--Global and overview maps--Carbon metabolism

[denovogenes]_104803	51.74	7e-91	csh:Closa_1417	K01971	ligD	bifunctional non-homologous end joining protein LigD	6.5.1.1	Genetic Information Processing--Replication and repair--Non-homologous end-joining

[denovogenes]_104806	52.51	1e-99	aar:Acear_0119	K05540	dusB	tRNA-dihydrouridine synthase B	1.-.-.-	--

[denovogenes]_104807	54.51	3e-96	bcel:BcellWH2_01448	K07098	K07098	uncharacterized protein	--	--

[denovogenes]_104819	85.32	3e-64	pper:PRUPE_ppa018016mg	K03671	trxA	thioredoxin 1	--	Organismal Systems--Immune system--NOD-like receptor signaling pathway|Human Diseases--Cardiovascular diseases--Fluid shear stress and atherosclerosis

[denovogenes]_10482	93.73	0.0	bhl:Bache_0872	K09181	yfiQ	acetyltransferase	--	--

[denovogenes]_104821	100.00	0.0	bfb:VU15_16545	K05306	phnX	phosphonoacetaldehyde hydrolase	3.11.1.1	Metabolism--Metabolism of other amino acids--Phosphonate and phosphinate metabolism|Metabolism--Global and overview maps--Metabolic pathways|Metabolism--Global and overview maps--Microbial metabolism in diverse environments

[denovogenes]_104822	100.00	1e-168	bfb:VU15_16780	K00945	cmk	CMP/dCMP kinase	2.7.4.25	Metabolism--Nucleotide metabolism--Pyrimidine metabolism|Metabolism--Global and overview maps--Metabolic pathways

[denovogenes]_104829	84.79	8e-157	lacy:A4V08_33230	K03613	rnfE	electron transport complex protein RnfE	--	--

[denovogenes]_104830	100.00	0.0	bfb:VU15_11940	K01695	trpA	tryptophan synthase alpha chain	4.2.1.20	Metabolism--Amino acid metabolism--Glycine, serine and threonine metabolism|Metabolism--Amino acid metabolism--Phenylalanine, tyrosine and tryptophan biosynthesis|Metabolism--Global and overview maps--Metabolic pathways|Metabolism--Global and overview maps--Biosynthesis of secondary metabolites|Metabolism--Global and overview maps--Biosynthesis of antibiotics|Metabolism--Global and overview maps--Biosynthesis of amino acids

[denovogenes]_104831	100.00	0.0	mmk:MU9_2743	K07090	K07090	uncharacterized protein	--	--

[denovogenes]_104833	80.99	5e-160	cpr:CPR_1426	K16951	--	--	--	Metabolism--Energy metabolism--Sulfur metabolism|Metabolism--Global and overview maps--Microbial metabolism in diverse environments

[denovogenes]_104835	100.00	0.0	mmk:MU9_336	K02039	phoU	phosphate transport system protein	--	--

[denovogenes]_104836	100.00	0.0	bfb:VU15_21625	K00046	idnO	gluconate 5-dehydrogenase	1.1.1.69	--

[denovogenes]_104838	79.09	3e-146	cbk:CLL_A0772	K09816	znuB	zinc transport system permease protein	--	Environmental Information Processing--Membrane transport--ABC transporters

[denovogenes]_104839	91.53	3e-168	lacy:A4V08_29120	K01803	TPI, tpiA	triosephosphate isomerase (TIM)	5.3.1.1	Metabolism--Carbohydrate metabolism--Glycolysis / Gluconeogenesis|Metabolism--Carbohydrate metabolism--Fructose and mannose metabolism|Metabolism--Carbohydrate metabolism--Inositol phosphate metabolism|Metabolism--Energy metabolism--Carbon fixation in photosynthetic organisms|Metabolism--Global and overview maps--Metabolic pathways|Metabolism--Global and overview maps--Biosynthesis of secondary metabolites|Metabolism--Global and overview maps--Microbial metabolism in diverse environments|Metabolism--Global and overview maps--Biosynthesis of antibiotics|Metabolism--Global and overview maps--Carbon metabolism|Metabolism--Global and overview maps--Biosynthesis of amino acids

[denovogenes]_10484	73.80	0.0	bfg:BF638R_1731	K05970	SIAE	sialate O-acetylesterase	3.1.1.53	--

[denovogenes]_104841	59.90	2e-78	csb:CLSA_c32410	K06872	K06872	uncharacterized protein	--	--

[denovogenes]_104842	92.02	3e-175	lacy:A4V08_00725	K03609	minD	septum site-determining protein MinD	--	--

[denovogenes]_104843	62.99	3e-109	psx:DR96_4025	K12057	traF	conjugal transfer pilus assembly protein TraF	--	--

[denovogenes]_104846	64.37	9e-125	cbz:Cbs_1572	K03437	spoU	RNA methyltransferase, TrmH family	--	--

[denovogenes]_104847	66.54	6e-127	txy:Thexy_2102	K01628	fucA	L-fuculose-phosphate aldolase	4.1.2.17	Metabolism--Carbohydrate metabolism--Fructose and mannose metabolism|Metabolism--Global and overview maps--Microbial metabolism in diverse environments

[denovogenes]_104848	41.35	9e-55	llm:llmg_0412	K00784	rnz	ribonuclease Z	3.1.26.11	Genetic Information Processing--Translation--RNA transport

[denovogenes]_104849	100.00	0.0	mmk:MU9_946	K00677	lpxA	UDP-N-acetylglucosamine acyltransferase	2.3.1.129	Metabolism--Glycan biosynthesis and metabolism--Lipopolysaccharide biosynthesis|Metabolism--Global and overview maps--Metabolic pathways|Human Diseases--Drug resistance: Antimicrobial--Cationic antimicrobial peptide (CAMP) resistance

[denovogenes]_104852	43.57	9e-61	llm:llmg_0412	K00784	rnz	ribonuclease Z	3.1.26.11	Genetic Information Processing--Translation--RNA transport

[denovogenes]_104854	76.43	8e-153	cbut:ATN24_00615	K00560	thyA, TYMS	thymidylate synthase	2.1.1.45	Metabolism--Nucleotide metabolism--Pyrimidine metabolism|Metabolism--Metabolism of cofactors and vitamins--One carbon pool by folate|Metabolism--Global and overview maps--Metabolic pathways|Human Diseases--Drug resistance: Antineoplastic--Antifolate resistance

[denovogenes]_104855	85.50	4e-168	clt:CM240_2983	K02013	ABC.FEV.A	iron complex transport system ATP-binding protein	3.6.3.34	Environmental Information Processing--Membrane transport--ABC transporters

[denovogenes]_104856	100.00	0.0	efq:DR75_1966	K00796	folP	dihydropteroate synthase	2.5.1.15	Metabolism--Metabolism of cofactors and vitamins--Folate biosynthesis|Metabolism--Global and overview maps--Metabolic pathways

[denovogenes]_104857	40.51	1e-57	llm:llmg_0412	K00784	rnz	ribonuclease Z	3.1.26.11	Genetic Information Processing--Translation--RNA transport

[denovogenes]_104858	49.42	4e-86	cbut:ATN24_15015	K03190	ureD, ureH	urease accessory protein	--	--

[denovogenes]_104859	100.00	0.0	ene:ENT_11220	K02823	pyrDII	dihydroorotate dehydrogenase electron transfer subunit	--	Metabolism--Nucleotide metabolism--Pyrimidine metabolism|Metabolism--Global and overview maps--Metabolic pathways

[denovogenes]_10486	61.10	0.0	rim:ROI_16030	K01187	malZ	alpha-glucosidase	3.2.1.20	Metabolism--Carbohydrate metabolism--Galactose metabolism|Metabolism--Carbohydrate metabolism--Starch and sucrose metabolism|Metabolism--Global and overview maps--Metabolic pathways

[denovogenes]_104861	77.95	6e-156	cbut:ATN24_00615	K00560	thyA, TYMS	thymidylate synthase	2.1.1.45	Metabolism--Nucleotide metabolism--Pyrimidine metabolism|Metabolism--Metabolism of cofactors and vitamins--One carbon pool by folate|Metabolism--Global and overview maps--Metabolic pathways|Human Diseases--Drug resistance: Antineoplastic--Antifolate resistance

[denovogenes]_104862	98.10	0.0	cfd:CFNIH1_10395	K00606	panB	3-methyl-2-oxobutanoate hydroxymethyltransferase	2.1.2.11	Metabolism--Metabolism of cofactors and vitamins--Pantothenate and CoA biosynthesis|Metabolism--Global and overview maps--Metabolic pathways|Metabolism--Global and overview maps--Biosynthesis of secondary metabolites

[denovogenes]_104864	74.14	2e-146	fpla:A4U99_01100	K02049	ABC.SN.A	NitT/TauT family transport system ATP-binding protein	--	--

[denovogenes]_104866	44.79	3e-79	edu:LIU_10275	K08600	srtB	sortase B	3.4.22.70	--

[denovogenes]_104867	100.00	0.0	ene:ENT_12940	K19508	PTS-Gfr-EIIC, gfrC	PTS system, fructoselysine/glucoselysine-specific IIC component	--	Environmental Information Processing--Membrane transport--Phosphotransferase system (PTS)

[denovogenes]_104868	41.02	3e-58	llm:llmg_0412	K00784	rnz	ribonuclease Z	3.1.26.11	Genetic Information Processing--Translation--RNA transport

[denovogenes]_10487	98.54	0.0	boa:Bovatus_02598	K03657	uvrD, pcrA	DNA helicase II / ATP-dependent DNA helicase PcrA	3.6.4.12	Genetic Information Processing--Replication and repair--Nucleotide excision repair|Genetic Information Processing--Replication and repair--Mismatch repair

[denovogenes]_104870	98.86	0.0	cbut:ATN24_19230	K01695	trpA	tryptophan synthase alpha chain	4.2.1.20	Metabolism--Amino acid metabolism--Glycine, serine and threonine metabolism|Metabolism--Amino acid metabolism--Phenylalanine, tyrosine and tryptophan biosynthesis|Metabolism--Global and overview maps--Metabolic pathways|Metabolism--Global and overview maps--Biosynthesis of secondary metabolites|Metabolism--Global and overview maps--Biosynthesis of antibiotics|Metabolism--Global and overview maps--Biosynthesis of amino acids

[denovogenes]_104871	75.10	4e-139	cbv:U729_2008	K02050	ABC.SN.P	NitT/TauT family transport system permease protein	--	--

[denovogenes]_104872	59.32	3e-93	cbz:Cbs_4313	K00826	E2.6.1.42, ilvE	branched-chain amino acid aminotransferase	2.6.1.42	Metabolism--Amino acid metabolism--Cysteine and methionine metabolism|Metabolism--Amino acid metabolism--Valine, leucine and isoleucine degradation|Metabolism--Amino acid metabolism--Valine, leucine and isoleucine biosynthesis|Metabolism--Metabolism of cofactors and vitamins--Pantothenate and CoA biosynthesis|Metabolism--Global and overview maps--Metabolic pathways|Metabolism--Global and overview maps--Biosynthesis of secondary metabolites|Metabolism--Global and overview maps--Biosynthesis of antibiotics|Metabolism--Global and overview maps--2-Oxocarboxylic acid metabolism|Metabolism--Global and overview maps--Biosynthesis of amino acids

[denovogenes]_104873	45.58	3e-37	pmf:P9303_05381	K00975	glgC	glucose-1-phosphate adenylyltransferase	2.7.7.27	Metabolism--Carbohydrate metabolism--Starch and sucrose metabolism|Metabolism--Carbohydrate metabolism--Amino sugar and nucleotide sugar metabolism|Metabolism--Global and overview maps--Metabolic pathways|Metabolism--Global and overview maps--Biosynthesis of secondary metabolites|Cellular Processes--Cellular community - prokaryotes--Biofilm formation - Escherichia coli

[denovogenes]_104875	63.95	1e-117	cbv:U729_2892	K03437	spoU	RNA methyltransferase, TrmH family	--	--

[denovogenes]_104878	100.00	0.0	efs:EFS1_0627	K03930	estA	putative tributyrin esterase	3.1.1.-	--

[denovogenes]_104879	99.62	0.0	cbut:ATN24_00615	K00560	thyA, TYMS	thymidylate synthase	2.1.1.45	Metabolism--Nucleotide metabolism--Pyrimidine metabolism|Metabolism--Metabolism of cofactors and vitamins--One carbon pool by folate|Metabolism--Global and overview maps--Metabolic pathways|Human Diseases--Drug resistance: Antineoplastic--Antifolate resistance

[denovogenes]_104880	99.24	0.0	pmib:BB2000_2205	K00065	kduD	2-deoxy-D-gluconate 3-dehydrogenase	1.1.1.125	Metabolism--Carbohydrate metabolism--Pentose and glucuronate interconversions

[denovogenes]_104881	63.10	2e-119	ccl:Clocl_0999	K09817	znuC	zinc transport system ATP-binding protein	3.6.3.-	Environmental Information Processing--Membrane transport--ABC transporters

[denovogenes]_104882	56.84	7e-88	rix:RO1_33930	K03798	ftsH, hflB	cell division protease FtsH	3.4.24.-	--

[denovogenes]_104883	96.20	4e-179	cif:AL515_09175	K15554	ssuC	sulfonate transport system permease protein	--	Metabolism--Energy metabolism--Sulfur metabolism|Environmental Information Processing--Membrane transport--ABC transporters

[denovogenes]_104884	69.58	8e-136	ipo:Ilyop_2233	K05833	ABC.X4.A	putative ABC transport system ATP-binding protein	--	--

[denovogenes]_104885	42.98	7e-61	vcy:IX92_16235	K11963	urtE	urea transport system ATP-binding protein	--	Environmental Information Processing--Membrane transport--ABC transporters

[denovogenes]_104886	100.00	0.0	elf:LF82_0394	K11105	cvrA, nhaP2	cell volume regulation protein A	--	--

[denovogenes]_104888	74.03	1e-147	cama:F384_26425	K12057	traF	conjugal transfer pilus assembly protein TraF	--	--

[denovogenes]_10489	61.36	0.0	ash:AL1_27120	K01187	malZ	alpha-glucosidase	3.2.1.20	Metabolism--Carbohydrate metabolism--Galactose metabolism|Metabolism--Carbohydrate metabolism--Starch and sucrose metabolism|Metabolism--Global and overview maps--Metabolic pathways

[denovogenes]_104890	62.78	1e-123	csq:CSCA_4177	K01962	accA	acetyl-CoA carboxylase carboxyl transferase subunit alpha	6.4.1.2	Metabolism--Lipid metabolism--Fatty acid biosynthesis|Metabolism--Carbohydrate metabolism--Pyruvate metabolism|Metabolism--Carbohydrate metabolism--Propanoate metabolism|Metabolism--Energy metabolism--Carbon fixation pathways in prokaryotes|Metabolism--Global and overview maps--Metabolic pathways|Metabolism--Global and overview maps--Biosynthesis of secondary metabolites|Metabolism--Global and overview maps--Microbial metabolism in diverse environments|Metabolism--Global and overview maps--Biosynthesis of antibiotics|Metabolism--Global and overview maps--Carbon metabolism|Metabolism--Global and overview maps--Fatty acid metabolism

[denovogenes]_104891	85.98	2e-167	cpr:CPR_0701	K10907	K10907	aminotransferase	2.6.1.-	--

[denovogenes]_104893	63.12	3e-122	cct:CC1_18920	K02026	ABC.MS.P1	multiple sugar transport system permease protein	--	--

[denovogenes]_104895	57.03	1e-95	cama:F384_09800	K03169	topB	DNA topoisomerase III	5.99.1.2	--

[denovogenes]_104897	81.75	1e-163	cbv:U729_379	K16951	--	--	--	Metabolism--Energy metabolism--Sulfur metabolism|Metabolism--Global and overview maps--Microbial metabolism in diverse environments

[denovogenes]_1049	91.00	0.0	lacy:A4V08_04670	K03737	por, nifJ	pyruvate-ferredoxin/flavodoxin oxidoreductase	1.2.7.1 1.2.7.-	Metabolism--Carbohydrate metabolism--Glycolysis / Gluconeogenesis|Metabolism--Carbohydrate metabolism--Citrate cycle (TCA cycle)|Metabolism--Carbohydrate metabolism--Pyruvate metabolism|Metabolism--Carbohydrate metabolism--Butanoate metabolism|Metabolism--Energy metabolism--Carbon fixation pathways in prokaryotes|Metabolism--Global and overview maps--Metabolic pathways|Metabolism--Global and overview maps--Microbial metabolism in diverse environments|Metabolism--Global and overview maps--Biosynthesis of antibiotics|Metabolism--Global and overview maps--Carbon metabolism

[denovogenes]_10490	77.52	0.0	bprm:CL3_24250	K03798	ftsH, hflB	cell division protease FtsH	3.4.24.-	--

[denovogenes]_104900	95.45	5e-176	cif:AL515_10705	K05777	ABC.VB1X.S	putative thiamine transport system substrate-binding protein	--	--

[denovogenes]_104902	98.48	0.0	cfd:CFNIH1_03885	K16693	wzxE	enterobacterial common antigen flippase	--	--

[denovogenes]_104903	43.09	1e-17	opo:DSM2777_04120	K03755	adiY	AraC family transcriptional regulator, transcriptional activator of adiA	--	--

[denovogenes]_104905	100.00	4e-180	cbut:ATN24_14500	K15552	tauC	taurine transport system permease protein	--	Metabolism--Energy metabolism--Sulfur metabolism|Environmental Information Processing--Membrane transport--ABC transporters

[denovogenes]_104906	40.08	4e-56	goy:GLS_c13890	K06192	pqiB	paraquat-inducible protein B	--	--

[denovogenes]_104908	99.62	0.0	cbut:ATN24_12855	K16951	--	--	--	Metabolism--Energy metabolism--Sulfur metabolism|Metabolism--Global and overview maps--Microbial metabolism in diverse environments

[denovogenes]_104909	99.62	0.0	elo:EC042_2636	K15835	murR	RpiR family transcriptional regulator, murPQ operon repressor	--	--

[denovogenes]_104910	84.09	1e-164	cle:Clole_2467	K03553	recA	recombination protein RecA	--	Genetic Information Processing--Replication and repair--Homologous recombination

[denovogenes]_104911	69.08	2e-125	cbk:CLL_A1196	K07636	phoR	two-component system, OmpR family, phosphate regulon sensor histidine kinase PhoR	2.7.13.3	Environmental Information Processing--Signal transduction--Two-component system

[denovogenes]_104912	67.98	8e-119	cle:Clole_1816	K02823	pyrDII	dihydroorotate dehydrogenase electron transfer subunit	--	Metabolism--Nucleotide metabolism--Pyrimidine metabolism|Metabolism--Global and overview maps--Metabolic pathways

[denovogenes]_104913	94.68	0.0	cif:AL515_08150	K05522	nei	endonuclease VIII	3.2.2.- 4.2.99.18	Genetic Information Processing--Replication and repair--Base excision repair

[denovogenes]_104914	76.89	6e-151	afn:Acfer_0243	K01745	hutH, HAL	histidine ammonia-lyase	4.3.1.3	Metabolism--Amino acid metabolism--Histidine metabolism|Metabolism--Global and overview maps--Metabolic pathways

[denovogenes]_104915	93.92	0.0	kin:AB182_21570	K01956	carA, CPA1	carbamoyl-phosphate synthase small subunit	6.3.5.5	Metabolism--Nucleotide metabolism--Pyrimidine metabolism|Metabolism--Amino acid metabolism--Alanine, aspartate and glutamate metabolism|Metabolism--Global and overview maps--Metabolic pathways

[denovogenes]_104916	99.62	0.0	cfd:CFNIH1_23470	K03815	xapA	xanthosine phosphorylase	2.4.2.-	Metabolism--Nucleotide metabolism--Purine metabolism|Metabolism--Global and overview maps--Metabolic pathways|Metabolism--Global and overview maps--Biosynthesis of secondary metabolites

[denovogenes]_104917	43.80	7e-70	ssm:Spirs_3522	K01101	E3.1.3.41	4-nitrophenyl phosphatase	3.1.3.41	Metabolism--Xenobiotics biodegradation and metabolism--Aminobenzoate degradation|Metabolism--Global and overview maps--Microbial metabolism in diverse environments

[denovogenes]_104918	100.00	0.0	sbc:SbBS512_E0271	K00121	frmA, ADH5, adhC	S-(hydroxymethyl)glutathione dehydrogenase / alcohol dehydrogenase	1.1.1.284 1.1.1.1	Metabolism--Carbohydrate metabolism--Glycolysis / Gluconeogenesis|Metabolism--Lipid metabolism--Fatty acid degradation|Metabolism--Amino acid metabolism--Tyrosine metabolism|Metabolism--Xenobiotics biodegradation and metabolism--Chloroalkane and chloroalkene degradation|Metabolism--Xenobiotics biodegradation and metabolism--Naphthalene degradation|Metabolism--Energy metabolism--Methane metabolism|Metabolism--Metabolism of cofactors and vitamins--Retinol metabolism|Metabolism--Xenobiotics biodegradation and metabolism--Metabolism of xenobiotics by cytochrome P450|Metabolism--Xenobiotics biodegradation and metabolism--Drug metabolism - cytochrome P450|Metabolism--Global and overview maps--Metabolic pathways|Metabolism--Global and overview maps--Biosynthesis of secondary metabolites|Metabolism--Global and overview maps--Microbial metabolism in diverse environments|Metabolism--Global and overview maps--Biosynthesis of antibiotics|Metabolism--Global and overview maps--Carbon metabolism|Metabolism--Global and overview maps--Degradation of aromatic compounds|Human Diseases--Cancers: Overview--Chemical carcinogenesis

[denovogenes]_104920	98.86	0.0	cbut:ATN24_11645	K02433	gatA, QRSL1	aspartyl-tRNA(Asn)/glutamyl-tRNA(Gln) amidotransferase subunit A	6.3.5.6 6.3.5.7	Genetic Information Processing--Translation--Aminoacyl-tRNA biosynthesis|Metabolism--Global and overview maps--Metabolic pathways

[denovogenes]_104921	100.00	0.0	cfd:CFNIH1_15790	K01756	purB, ADSL	adenylosuccinate lyase	4.3.2.2	Metabolism--Nucleotide metabolism--Purine metabolism|Metabolism--Amino acid metabolism--Alanine, aspartate and glutamate metabolism|Metabolism--Global and overview maps--Metabolic pathways|Metabolism--Global and overview maps--Biosynthesis of secondary metabolites|Metabolism--Global and overview maps--Biosynthesis of antibiotics

[denovogenes]_104922	100.00	0.0	cfd:CFNIH1_01200	K00626	E2.3.1.9, atoB	acetyl-CoA C-acetyltransferase	2.3.1.9	Metabolism--Lipid metabolism--Fatty acid degradation|Metabolism--Lipid metabolism--Synthesis and degradation of ketone bodies|Metabolism--Amino acid metabolism--Valine, leucine and isoleucine degradation|Metabolism--Amino acid metabolism--Lysine degradation|Metabolism--Xenobiotics biodegradation and metabolism--Benzoate degradation|Metabolism--Amino acid metabolism--Tryptophan metabolism|Metabolism--Carbohydrate metabolism--Pyruvate metabolism|Metabolism--Carbohydrate metabolism--Glyoxylate and dicarboxylate metabolism|Metabolism--Carbohydrate metabolism--Propanoate metabolism|Metabolism--Carbohydrate metabolism--Butanoate metabolism|Metabolism--Energy metabolism--Carbon fixation pathways in prokaryotes|Metabolism--Metabolism of terpenoids and polyketides--Terpenoid backbone biosynthesis|Metabolism--Global and overview maps--Metabolic pathways|Metabolism--Global and overview maps--Biosynthesis of secondary metabolites|Metabolism--Global and overview maps--Microbial metabolism in diverse environments|Metabolism--Global and overview maps--Biosynthesis of antibiotics|Metabolism--Global and overview maps--Carbon metabolism|Metabolism--Global and overview maps--Fatty acid metabolism|Environmental Information Processing--Signal transduction--Two-component system

[denovogenes]_104923	53.73	1e-96	sbb:Sbal175_2123	K04069	pflA, pflC, pflE	pyruvate formate lyase activating enzyme	1.97.1.4	--

[denovogenes]_104924	63.91	6e-125	cbk:CLL_A1402	K02016	ABC.FEV.S	iron complex transport system substrate-binding protein	--	Environmental Information Processing--Membrane transport--ABC transporters

[denovogenes]_104926	72.14	1e-131	vpr:Vpar_0295	K03431	glmM	phosphoglucosamine mutase	5.4.2.10	Metabolism--Carbohydrate metabolism--Amino sugar and nucleotide sugar metabolism|Metabolism--Global and overview maps--Metabolic pathways|Metabolism--Global and overview maps--Biosynthesis of antibiotics

[denovogenes]_104928	75.29	9e-152	med:MELS_1411	K00560	thyA, TYMS	thymidylate synthase	2.1.1.45	Metabolism--Nucleotide metabolism--Pyrimidine metabolism|Metabolism--Metabolism of cofactors and vitamins--One carbon pool by folate|Metabolism--Global and overview maps--Metabolic pathways|Human Diseases--Drug resistance: Antineoplastic--Antifolate resistance

[denovogenes]_104930	67.86	4e-131	cle:Clole_1665	K04079	HSP90A, htpG	molecular chaperone HtpG	--	Genetic Information Processing--Folding, sorting and degradation--Protein processing in endoplasmic reticulum|Environmental Information Processing--Signal transduction--PI3K-Akt signaling pathway|Organismal Systems--Immune system--Antigen processing and presentation|Organismal Systems--Immune system--NOD-like receptor signaling pathway|Organismal Systems--Environmental adaptation--Plant-pathogen interaction|Organismal Systems--Immune system--IL-17 signaling pathway|Organismal Systems--Immune system--Th17 cell differentiation|Organismal Systems--Endocrine system--Progesterone-mediated oocyte maturation|Organismal Systems--Endocrine system--Estrogen signaling pathway|Human Diseases--Cancers: Overview--Pathways in cancer|Human Diseases--Cancers: Specific types--Prostate cancer|Human Diseases--Cardiovascular diseases--Fluid shear stress and atherosclerosis

[denovogenes]_104931	80.45	3e-145	cle:Clole_2129	K04043	dnaK	molecular chaperone DnaK	--	Genetic Information Processing--Folding, sorting and degradation--RNA degradation|Organismal Systems--Aging--Longevity regulating pathway - worm|Human Diseases--Infectious diseases: Bacterial--Tuberculosis

[denovogenes]_104932	69.76	1e-126	cbn:CbC4_1935	K02071	metN	D-methionine transport system ATP-binding protein	--	Environmental Information Processing--Membrane transport--ABC transporters

[denovogenes]_104933	88.26	4e-171	cbk:CLL_A2888	K03544	clpX, CLPX	ATP-dependent Clp protease ATP-binding subunit ClpX	--	Cellular Processes--Cell growth and death--Cell cycle - Caulobacter

[denovogenes]_104936	80.30	1e-158	cbv:U729_1141	K00532	E1.12.7.2	ferredoxin hydrogenase	1.12.7.2	--

[denovogenes]_104937	99.24	0.0	cfd:CFNIH1_02655	K00574	cfa	cyclopropane-fatty-acyl-phospholipid synthase	2.1.1.79	--

[denovogenes]_104938	79.62	8e-144	cle:Clole_2417	K02600	nusA	N utilization substance protein A	--	--

[denovogenes]_104941	43.44	9e-24	synp:Syn7502_02473	K03496	parA, soj	chromosome partitioning protein	--	--

[denovogenes]_104942	54.09	2e-84	cdg:CDBI1_16020	K02040	pstS	phosphate transport system substrate-binding protein	--	Environmental Information Processing--Membrane transport--ABC transporters|Environmental Information Processing--Signal transduction--Two-component system|Human Diseases--Infectious diseases: Bacterial--Tuberculosis

[denovogenes]_104943	100.00	3e-126	pvl:AOB99_03340	K02301	cyoE	protoheme IX farnesyltransferase	2.5.1.-	Metabolism--Energy metabolism--Oxidative phosphorylation|Metabolism--Metabolism of cofactors and vitamins--Porphyrin and chlorophyll metabolism|Metabolism--Global and overview maps--Metabolic pathways|Metabolism--Global and overview maps--Biosynthesis of secondary metabolites

[denovogenes]_104944	100.00	0.0	pvl:AOB99_12255	K01703	leuC	3-isopropylmalate/(R)-2-methylmalate dehydratase large subunit	4.2.1.33 4.2.1.35	Metabolism--Amino acid metabolism--Valine, leucine and isoleucine biosynthesis|Metabolism--Carbohydrate metabolism--C5-Branched dibasic acid metabolism|Metabolism--Global and overview maps--Metabolic pathways|Metabolism--Global and overview maps--Biosynthesis of secondary metabolites|Metabolism--Global and overview maps--2-Oxocarboxylic acid metabolism|Metabolism--Global and overview maps--Biosynthesis of amino acids

[denovogenes]_104945	90.91	1e-171	clt:CM240_2556	K02818	PTS-Tre-EIIB, treB	PTS system, trehalose-specific IIB component	2.7.1.201	Metabolism--Carbohydrate metabolism--Starch and sucrose metabolism|Environmental Information Processing--Membrane transport--Phosphotransferase system (PTS)

[denovogenes]_104945	90.91	1e-171	clt:CM240_2556	K02819	PTS-Tre-EIIC, treB	PTS system, trehalose-specific IIC component	--	Metabolism--Carbohydrate metabolism--Starch and sucrose metabolism|Environmental Information Processing--Membrane transport--Phosphotransferase system (PTS)

[denovogenes]_104947	43.22	7e-54	cbz:Cbs_3405	K11527	K11527	two-component system, sensor histidine kinase and response regulator	2.7.13.3	--

[denovogenes]_104948	95.83	1e-176	cif:AL515_04580	K00041	uxaB	tagaturonate reductase	1.1.1.58	Metabolism--Carbohydrate metabolism--Pentose and glucuronate interconversions|Metabolism--Global and overview maps--Metabolic pathways

[denovogenes]_104949	99.24	0.0	cif:AL515_09540	K01620	ltaE	threonine aldolase	4.1.2.48	Metabolism--Amino acid metabolism--Glycine, serine and threonine metabolism|Metabolism--Global and overview maps--Metabolic pathways|Metabolism--Global and overview maps--Biosynthesis of secondary metabolites|Metabolism--Global and overview maps--Microbial metabolism in diverse environments|Metabolism--Global and overview maps--Biosynthesis of antibiotics|Metabolism--Global and overview maps--Biosynthesis of amino acids

[denovogenes]_104950	43.02	2e-70	pnl:PNK_1762	K18138	acrB, mexB, adeJ, smeE, mtrD, cmeB	multidrug efflux pump	--	Human Diseases--Drug resistance: Antimicrobial--beta-Lactam resistance|Human Diseases--Drug resistance: Antimicrobial--Cationic antimicrobial peptide (CAMP) resistance

[denovogenes]_104951	100.00	0.0	cif:AL515_02305	K03667	hslU	ATP-dependent HslUV protease ATP-binding subunit HslU	--	--

[denovogenes]_104952	99.24	0.0	boa:Bovatus_03103	K01960	pycB	pyruvate carboxylase subunit B	6.4.1.1	Metabolism--Carbohydrate metabolism--Citrate cycle (TCA cycle)|Metabolism--Carbohydrate metabolism--Pyruvate metabolism|Metabolism--Energy metabolism--Carbon fixation pathways in prokaryotes|Metabolism--Global and overview maps--Metabolic pathways|Metabolism--Global and overview maps--Microbial metabolism in diverse environments|Metabolism--Global and overview maps--Carbon metabolism|Metabolism--Global and overview maps--Biosynthesis of amino acids

[denovogenes]_104954	56.82	3e-104	vig:BKP57_12895	K00527	nrdD	ribonucleoside-triphosphate reductase	1.17.4.2	Metabolism--Nucleotide metabolism--Purine metabolism|Metabolism--Nucleotide metabolism--Pyrimidine metabolism|Metabolism--Global and overview maps--Metabolic pathways

[denovogenes]_104955	51.30	3e-93	fjo:Fjoh_0774	K01179	E3.2.1.4	endoglucanase	3.2.1.4	Metabolism--Carbohydrate metabolism--Starch and sucrose metabolism|Metabolism--Global and overview maps--Metabolic pathways

[denovogenes]_104957	57.07	2e-77	clt:CM240_2328	K01537	E3.6.3.8	Ca2+-transporting ATPase	3.6.3.8	--

[denovogenes]_104958	58.40	1e-104	afn:Acfer_0312	K09729	K09729	uncharacterized protein	--	--

[denovogenes]_104959	91.44	1e-167	boa:Bovatus_03532	K00930	argB	acetylglutamate kinase	2.7.2.8	Metabolism--Amino acid metabolism--Arginine biosynthesis|Metabolism--Global and overview maps--Metabolic pathways|Metabolism--Global and overview maps--Biosynthesis of secondary metabolites|Metabolism--Global and overview maps--Biosynthesis of antibiotics|Metabolism--Global and overview maps--2-Oxocarboxylic acid metabolism|Metabolism--Global and overview maps--Biosynthesis of amino acids

[denovogenes]_10496	50.98	5e-156	vpr:Vpar_1830	K00336	nuoG	NADH-quinone oxidoreductase subunit G	1.6.5.3	Metabolism--Energy metabolism--Oxidative phosphorylation|Metabolism--Global and overview maps--Metabolic pathways

[denovogenes]_104964	77.57	1e-152	cpy:Cphy_2370	K03609	minD	septum site-determining protein MinD	--	--

[denovogenes]_104965	59.00	5e-113	elm:ELI_4370	K02031	ABC.PE.A	peptide/nickel transport system ATP-binding protein	--	Cellular Processes--Cellular community - prokaryotes--Quorum sensing

[denovogenes]_104966	42.70	6e-40	ele:Elen_0226	K02042	phnE	phosphonate transport system permease protein	--	Environmental Information Processing--Membrane transport--ABC transporters

[denovogenes]_104967	71.86	1e-134	rho:RHOM_08335	K03616	rnfB	electron transport complex protein RnfB	--	--

[denovogenes]_104971	50.38	8e-81	byl:A4V09_05570	K02233	E2.7.8.26, cobS, cobV	adenosylcobinamide-GDP ribazoletransferase	2.7.8.26	Metabolism--Metabolism of cofactors and vitamins--Porphyrin and chlorophyll metabolism|Metabolism--Global and overview maps--Metabolic pathways

[denovogenes]_104973	100.00	0.0	bdh:GV66_17865	K07164	K07164	uncharacterized protein	--	--

[denovogenes]_104975	93.31	1e-178	btho:Btheta7330_02631	K07001	K07001	NTE family protein	--	--

[denovogenes]_104977	94.68	2e-175	bhl:Bache_2701	K11070	potC	spermidine/putrescine transport system permease protein	--	Environmental Information Processing--Membrane transport--ABC transporters

[denovogenes]_104978	54.33	7e-98	ava:Ava_2888	K05886	sdh	serine 3-dehydrogenase (NADP+)	1.1.1.276	--

[denovogenes]_104979	53.82	2e-99	bbay:A4V04_08065	K01713	pheC	cyclohexadienyl dehydratase	4.2.1.51 4.2.1.91	Metabolism--Amino acid metabolism--Phenylalanine, tyrosine and tryptophan biosynthesis|Metabolism--Global and overview maps--Metabolic pathways|Metabolism--Global and overview maps--Biosynthesis of secondary metabolites|Metabolism--Global and overview maps--Biosynthesis of antibiotics|Metabolism--Global and overview maps--Biosynthesis of amino acids

[denovogenes]_104982	70.50	2e-126	fpla:A4U99_00170	K07402	xdhC	xanthine dehydrogenase accessory factor	--	--

[denovogenes]_104983	45.38	8e-71	byl:A4V09_20805	K20074	prpC, phpP	PPM family protein phosphatase	3.1.3.16	--

[denovogenes]_104984	40.91	8e-38	fpl:Ferp_1187	K04114	bcrA, badF	benzoyl-CoA reductase subunit A	1.3.7.8	Metabolism--Xenobiotics biodegradation and metabolism--Benzoate degradation|Metabolism--Global and overview maps--Metabolic pathways|Metabolism--Global and overview maps--Microbial metabolism in diverse environments|Metabolism--Global and overview maps--Degradation of aromatic compounds

[denovogenes]_104985	74.43	6e-140	rto:RTO_20290	K03616	rnfB	electron transport complex protein RnfB	--	--

[denovogenes]_104986	72.90	3e-125	pbt:ING2E5B_1294	K07238	TC.ZIP, zupT, ZRT3, ZIP2	zinc transporter, ZIP family	--	--

[denovogenes]_104988	86.31	9e-175	boa:Bovatus_03697	K02564	nagB, GNPDA	glucosamine-6-phosphate deaminase	3.5.99.6	Metabolism--Carbohydrate metabolism--Amino sugar and nucleotide sugar metabolism|Metabolism--Global and overview maps--Metabolic pathways

[denovogenes]_10499	84.69	0.0	cpf:CPF_1211	K01547	kdpB	K+-transporting ATPase ATPase B chain	3.6.3.12	Environmental Information Processing--Signal transduction--Two-component system

[denovogenes]_104993	69.69	4e-126	cpy:Cphy_0713	K07238	TC.ZIP, zupT, ZRT3, ZIP2	zinc transporter, ZIP family	--	--

[denovogenes]_104994	96.96	0.0	bxy:BXY_08450	K08679	E5.1.3.6	UDP-glucuronate 4-epimerase	5.1.3.6	Metabolism--Carbohydrate metabolism--Amino sugar and nucleotide sugar metabolism|Metabolism--Global and overview maps--Metabolic pathways

[denovogenes]_104995	100.00	0.0	bvu:BVU_4120	K02564	nagB, GNPDA	glucosamine-6-phosphate deaminase	3.5.99.6	Metabolism--Carbohydrate metabolism--Amino sugar and nucleotide sugar metabolism|Metabolism--Global and overview maps--Metabolic pathways

[denovogenes]_104996	85.06	1e-170	rum:CK1_00260	K00757	udp, UPP	uridine phosphorylase	2.4.2.3	Metabolism--Nucleotide metabolism--Pyrimidine metabolism|Metabolism--Xenobiotics biodegradation and metabolism--Drug metabolism - other enzymes|Metabolism--Global and overview maps--Metabolic pathways

[denovogenes]_104998	42.44	8e-36	des:DSOUD_2952	K15460	yfiC, trmX	tRNA1Val (adenine37-N6)-methyltransferase	2.1.1.223	--

[denovogenes]_104999	71.15	6e-131	lacy:A4V08_11785	K02049	ABC.SN.A	NitT/TauT family transport system ATP-binding protein	--	--

[denovogenes]_105001	64.96	1e-102	rho:RHOM_10010	K06973	K06973	uncharacterized protein	--	--

[denovogenes]_105002	72.31	4e-135	rto:RTO_19810	K07699	spo0A	two-component system, response regulator, stage 0 sporulation protein A	--	Environmental Information Processing--Signal transduction--Two-component system|Cellular Processes--Cellular community - prokaryotes--Quorum sensing

[denovogenes]_105005	43.32	2e-67	avs:AWM76_07320	K19423	epsE	glycosyltransferase EpsE	2.4.-.-	--

[denovogenes]_105006	40.49	9e-35	afa:UZ73_11865	K00059	fabG	3-oxoacyl-[acyl-carrier protein] reductase	1.1.1.100	Metabolism--Lipid metabolism--Fatty acid biosynthesis|Metabolism--Metabolism of cofactors and vitamins--Biotin metabolism|Metabolism--Lipid metabolism--Biosynthesis of unsaturated fatty acids|Metabolism--Global and overview maps--Metabolic pathways|Metabolism--Global and overview maps--Fatty acid metabolism

[denovogenes]_105008	87.31	2e-168	boa:Bovatus_01563	K04486	E3.1.3.15B	histidinol-phosphatase (PHP family)	3.1.3.15	Metabolism--Amino acid metabolism--Histidine metabolism|Metabolism--Global and overview maps--Metabolic pathways|Metabolism--Global and overview maps--Biosynthesis of secondary metabolites|Metabolism--Global and overview maps--Biosynthesis of amino acids

[denovogenes]_10501	99.42	0.0	bvu:BVU_4140	K05970	SIAE	sialate O-acetylesterase	3.1.1.53	--

[denovogenes]_105010	80.17	7e-143	cso:CLS_03240	K02003	ABC.CD.A	putative ABC transport system ATP-binding protein	--	--

[denovogenes]_105012	81.32	2e-150	cct:CC1_00760	K01715	crt	enoyl-CoA hydratase	4.2.1.17	Metabolism--Carbohydrate metabolism--Butanoate metabolism|Metabolism--Global and overview maps--Carbon metabolism

[denovogenes]_105013	56.47	3e-102	ccel:CCDG5_0514	K00931	proB	glutamate 5-kinase	2.7.2.11	Metabolism--Amino acid metabolism--Arginine and proline metabolism|Metabolism--Biosynthesis of other secondary metabolites--Carbapenem biosynthesis|Metabolism--Global and overview maps--Metabolic pathways|Metabolism--Global and overview maps--Biosynthesis of antibiotics|Metabolism--Global and overview maps--Biosynthesis of amino acids

[denovogenes]_105015	100.00	0.0	bvu:BVU_3554	K11070	potC	spermidine/putrescine transport system permease protein	--	Environmental Information Processing--Membrane transport--ABC transporters

[denovogenes]_105018	71.09	2e-130	fpla:A4U99_13990	K07699	spo0A	two-component system, response regulator, stage 0 sporulation protein A	--	Environmental Information Processing--Signal transduction--Two-component system|Cellular Processes--Cellular community - prokaryotes--Quorum sensing

[denovogenes]_10502	100.00	0.0	bth:BT_0806	K01870	IARS, ileS	isoleucyl-tRNA synthetase	6.1.1.5	Genetic Information Processing--Translation--Aminoacyl-tRNA biosynthesis

[denovogenes]_105020	51.97	1e-85	ova:OBV_39500	K02421	fliR	flagellar biosynthetic protein FliR	--	Cellular Processes--Cell motility--Flagellar assembly

[denovogenes]_105021	67.43	2e-134	cpy:Cphy_2914	K00286	proC	pyrroline-5-carboxylate reductase	1.5.1.2	Metabolism--Amino acid metabolism--Arginine and proline metabolism|Metabolism--Global and overview maps--Metabolic pathways|Metabolism--Global and overview maps--Biosynthesis of secondary metabolites|Metabolism--Global and overview maps--Biosynthesis of antibiotics|Metabolism--Global and overview maps--Biosynthesis of amino acids

[denovogenes]_105023	93.10	8e-178	btho:Btheta7330_05093	K06153	bacA	undecaprenyl-diphosphatase	3.6.1.27	Metabolism--Glycan biosynthesis and metabolism--Peptidoglycan biosynthesis

[denovogenes]_105024	90.49	2e-175	cso:CLS_26130	K03609	minD	septum site-determining protein MinD	--	--

[denovogenes]_105028	41.18	2e-15	beo:BEH_04490	K07705	lytT, lytR	two-component system, LytT family, response regulator LytT	--	Environmental Information Processing--Signal transduction--Two-component system

[denovogenes]_10503	96.22	0.0	pfre:RM25_0195	K06147	ABCB-BAC	ATP-binding cassette, subfamily B, bacterial	--	--

[denovogenes]_105031	69.35	3e-130	lacy:A4V08_33240	K03616	rnfB	electron transport complex protein RnfB	--	--

[denovogenes]_105032	82.20	6e-164	bxy:BXY_17330	K01081	E3.1.3.5	5'-nucleotidase	3.1.3.5	Metabolism--Nucleotide metabolism--Purine metabolism|Metabolism--Nucleotide metabolism--Pyrimidine metabolism|Metabolism--Metabolism of cofactors and vitamins--Nicotinate and nicotinamide metabolism|Metabolism--Global and overview maps--Metabolic pathways|Metabolism--Global and overview maps--Biosynthesis of secondary metabolites

[denovogenes]_105035	69.13	2e-119	cpy:Cphy_3609	K09812	ftsE	cell division transport system ATP-binding protein	--	Environmental Information Processing--Membrane transport--ABC transporters

[denovogenes]_105037	85.93	1e-164	sezo:SeseC_00298	K02025	ABC.MS.P	multiple sugar transport system permease protein	--	--

[denovogenes]_105038	97.72	0.0	bvu:BVU_2675	K01991	ABC-2.OM, wza	polysaccharide biosynthesis/export protein	--	Cellular Processes--Cellular community - prokaryotes--Biofilm formation - Escherichia coli

[denovogenes]_105039	90.08	3e-167	bfs:BF9343_2651	K11070	potC	spermidine/putrescine transport system permease protein	--	Environmental Information Processing--Membrane transport--ABC transporters

[denovogenes]_105040	100.00	0.0	aaj:BOQ57_11320	K00984	aadA	streptomycin 3"-adenylyltransferase	2.7.7.47	--

[denovogenes]_105041	98.10	0.0	fpla:A4U99_02965	K16951	--	--	--	Metabolism--Energy metabolism--Sulfur metabolism|Metabolism--Global and overview maps--Microbial metabolism in diverse environments

[denovogenes]_105044	47.89	1e-70	ibu:IB211_00227c	K18979	queG	epoxyqueuosine reductase	1.17.99.6	--

[denovogenes]_105045	89.35	2e-180	btho:Btheta7330_03847	K04486	E3.1.3.15B	histidinol-phosphatase (PHP family)	3.1.3.15	Metabolism--Amino acid metabolism--Histidine metabolism|Metabolism--Global and overview maps--Metabolic pathways|Metabolism--Global and overview maps--Biosynthesis of secondary metabolites|Metabolism--Global and overview maps--Biosynthesis of amino acids

[denovogenes]_105046	95.06	0.0	boa:Bovatus_04524	K04486	E3.1.3.15B	histidinol-phosphatase (PHP family)	3.1.3.15	Metabolism--Amino acid metabolism--Histidine metabolism|Metabolism--Global and overview maps--Metabolic pathways|Metabolism--Global and overview maps--Biosynthesis of secondary metabolites|Metabolism--Global and overview maps--Biosynthesis of amino acids

[denovogenes]_105047	99.62	0.0	ssj:SSON53_02535	K01425	glsA, GLS	glutaminase	3.5.1.2	Metabolism--Amino acid metabolism--Arginine biosynthesis|Metabolism--Amino acid metabolism--Alanine, aspartate and glutamate metabolism|Metabolism--Metabolism of other amino acids--D-Glutamine and D-glutamate metabolism|Metabolism--Global and overview maps--Metabolic pathways|Organismal Systems--Nervous system--Glutamatergic synapse|Organismal Systems--Nervous system--GABAergic synapse|Organismal Systems--Excretory system--Proximal tubule bicarbonate reclamation|Human Diseases--Cancers: Overview--MicroRNAs in cancer|Human Diseases--Cancers: Overview--Central carbon metabolism in cancer

[denovogenes]_105049	81.68	1e-153	lacy:A4V08_27000	K07720	yesN	two-component system, response regulator YesN	--	Environmental Information Processing--Signal transduction--Two-component system

[denovogenes]_10505	76.71	0.0	gfe:Gferi_09750	K01473	hyuA	N-methylhydantoinase A	3.5.2.14	Metabolism--Amino acid metabolism--Arginine and proline metabolism|Metabolism--Global and overview maps--Metabolic pathways

[denovogenes]_105050	84.79	5e-169	lacy:A4V08_13910	K01470	E3.5.2.10	creatinine amidohydrolase	3.5.2.10	Metabolism--Amino acid metabolism--Arginine and proline metabolism

[denovogenes]_105053	79.39	1e-145	cso:CLS_31870	K03616	rnfB	electron transport complex protein RnfB	--	--

[denovogenes]_105054	82.58	8e-158	bvu:BVU_3864	K09808	lolC_E	lipoprotein-releasing system permease protein	--	Environmental Information Processing--Membrane transport--ABC transporters

[denovogenes]_105055	100.00	0.0	ese:ECSF_2393	K07715	glrR, qseF	two-component system, NtrC family, response regulator GlrR	--	Environmental Information Processing--Signal transduction--Two-component system|Cellular Processes--Cellular community - prokaryotes--Quorum sensing

[denovogenes]_105056	77.57	3e-157	vhl:BME96_15405	K02049	ABC.SN.A	NitT/TauT family transport system ATP-binding protein	--	--

[denovogenes]_105058	99.62	0.0	eck:EC55989_4510	K07074	K07074	uncharacterized protein	--	--

[denovogenes]_105059	99.62	0.0	ece:Z2874	K19333	kdgR	IclR family transcriptional regulator, KDG regulon repressor	--	--

[denovogenes]_10506	53.29	0.0	bvs:BARVI_03875	K07459	ybjD	putative ATP-dependent endonuclease of the OLD family	--	--

[denovogenes]_105060	41.02	4e-50	pha:PSHAa0410	K07484	K07484	transposase	--	--

[denovogenes]_105061	87.07	2e-172	lacy:A4V08_33385	K16951	--	--	--	Metabolism--Energy metabolism--Sulfur metabolism|Metabolism--Global and overview maps--Microbial metabolism in diverse environments

[denovogenes]_105063	99.61	0.0	pdf:CD630DERM_10950	K01992	ABC-2.P	ABC-2 type transport system permease protein	--	--

[denovogenes]_105066	78.33	3e-146	lacy:A4V08_08065	K03521	fixA, etfB	electron transfer flavoprotein beta subunit	--	--

[denovogenes]_105067	94.55	7e-174	cso:CLS_24200	K03706	codY	transcriptional pleiotropic repressor	--	--

[denovogenes]_105070	59.02	2e-109	esr:ES1_25750	K01962	accA	acetyl-CoA carboxylase carboxyl transferase subunit alpha	6.4.1.2	Metabolism--Lipid metabolism--Fatty acid biosynthesis|Metabolism--Carbohydrate metabolism--Pyruvate metabolism|Metabolism--Carbohydrate metabolism--Propanoate metabolism|Metabolism--Energy metabolism--Carbon fixation pathways in prokaryotes|Metabolism--Global and overview maps--Metabolic pathways|Metabolism--Global and overview maps--Biosynthesis of secondary metabolites|Metabolism--Global and overview maps--Microbial metabolism in diverse environments|Metabolism--Global and overview maps--Biosynthesis of antibiotics|Metabolism--Global and overview maps--Carbon metabolism|Metabolism--Global and overview maps--Fatty acid metabolism

[denovogenes]_105072	44.40	3e-71	hty:BN2458_PEG1751	K01953	asnB, ASNS	asparagine synthase (glutamine-hydrolysing)	6.3.5.4	Metabolism--Amino acid metabolism--Alanine, aspartate and glutamate metabolism|Metabolism--Global and overview maps--Metabolic pathways|Metabolism--Global and overview maps--Biosynthesis of secondary metabolites

[denovogenes]_105073	44.67	2e-30	bprs:CK3_32470	K03100	lepB, TPP	signal peptidase I	3.4.21.89	Cellular Processes--Cellular community - prokaryotes--Quorum sensing|Genetic Information Processing--Folding, sorting and degradation--Protein export

[denovogenes]_105076	98.86	0.0	bdh:GV66_21905	K02564	nagB, GNPDA	glucosamine-6-phosphate deaminase	3.5.99.6	Metabolism--Carbohydrate metabolism--Amino sugar and nucleotide sugar metabolism|Metabolism--Global and overview maps--Metabolic pathways

[denovogenes]_105077	82.82	1e-159	hsd:SD1D_1648	K00625	E2.3.1.8, pta	phosphate acetyltransferase	2.3.1.8	Metabolism--Metabolism of other amino acids--Taurine and hypotaurine metabolism|Metabolism--Carbohydrate metabolism--Pyruvate metabolism|Metabolism--Carbohydrate metabolism--Propanoate metabolism|Metabolism--Energy metabolism--Methane metabolism|Metabolism--Energy metabolism--Carbon fixation pathways in prokaryotes|Metabolism--Global and overview maps--Metabolic pathways|Metabolism--Global and overview maps--Microbial metabolism in diverse environments|Metabolism--Global and overview maps--Carbon metabolism

[denovogenes]_105078	55.23	1e-81	ruk:A4V00_06710	K06147	ABCB-BAC	ATP-binding cassette, subfamily B, bacterial	--	--

[denovogenes]_105079	93.16	3e-179	lacy:A4V08_00725	K03609	minD	septum site-determining protein MinD	--	--

[denovogenes]_10508	94.76	0.0	bfb:VU15_04825	K16089	TC.FEV.OM2, cirA, cfrA, hmuR	outer membrane receptor for ferrienterochelin and colicins	--	--

[denovogenes]_105083	86.09	7e-167	ere:EUBREC_2689	K03205	virD4, lvhD4	type IV secretion system protein VirD4	--	Environmental Information Processing--Membrane transport--Bacterial secretion system

[denovogenes]_105086	94.30	1e-174	bcel:BcellWH2_05262	K11070	potC	spermidine/putrescine transport system permease protein	--	Environmental Information Processing--Membrane transport--ABC transporters

[denovogenes]_105087	98.48	0.0	btho:Btheta7330_05055	K09817	znuC	zinc transport system ATP-binding protein	3.6.3.-	Environmental Information Processing--Membrane transport--ABC transporters

[denovogenes]_105088	90.76	5e-166	bprs:CK3_05150	K01835	pgm	phosphoglucomutase	5.4.2.2	Metabolism--Carbohydrate metabolism--Glycolysis / Gluconeogenesis|Metabolism--Carbohydrate metabolism--Pentose phosphate pathway|Metabolism--Carbohydrate metabolism--Galactose metabolism|Metabolism--Nucleotide metabolism--Purine metabolism|Metabolism--Carbohydrate metabolism--Starch and sucrose metabolism|Metabolism--Carbohydrate metabolism--Amino sugar and nucleotide sugar metabolism|Metabolism--Biosynthesis of other secondary metabolites--Streptomycin biosynthesis|Metabolism--Global and overview maps--Metabolic pathways|Metabolism--Global and overview maps--Biosynthesis of secondary metabolites|Metabolism--Global and overview maps--Microbial metabolism in diverse environments|Metabolism--Global and overview maps--Biosynthesis of antibiotics

[denovogenes]_10509	98.84	0.0	cfd:CFNIH1_04970	K01534	zntA	Cd2+/Zn2+-exporting ATPase	3.6.3.3 3.6.3.5	--

[denovogenes]_105091	70.99	2e-140	cbz:Cbs_0784	K16951	--	--	--	Metabolism--Energy metabolism--Sulfur metabolism|Metabolism--Global and overview maps--Microbial metabolism in diverse environments

[denovogenes]_105092	100.00	0.0	ssj:SSON53_00250	K05245	caiT	L-carnitine/gamma-butyrobetaine antiporter	--	--

[denovogenes]_105094	96.96	0.0	bdh:GV66_04755	K01585	speA	arginine decarboxylase	4.1.1.19	Metabolism--Amino acid metabolism--Arginine and proline metabolism|Metabolism--Global and overview maps--Metabolic pathways

[denovogenes]_105097	79.93	1e-153	csq:CSCA_1915	K03205	virD4, lvhD4	type IV secretion system protein VirD4	--	Environmental Information Processing--Membrane transport--Bacterial secretion system

[denovogenes]_105098	84.06	3e-139	fpla:A4U99_01345	K03218	rlmB	23S rRNA (guanosine2251-2'-O)-methyltransferase	2.1.1.185	--

[denovogenes]_105099	87.88	1e-162	cle:Clole_0916	K01997	livH	branched-chain amino acid transport system permease protein	--	Environmental Information Processing--Membrane transport--ABC transporters|Cellular Processes--Cellular community - prokaryotes--Quorum sensing

[denovogenes]_105101	98.86	0.0	cfd:CFNIH1_13530	K13795	citB, tcuB	citrate/tricarballylate utilization protein	--	--

[denovogenes]_105104	61.83	6e-121	cah:CAETHG_1373	K00058	serA, PHGDH	D-3-phosphoglycerate dehydrogenase	1.1.1.95	Metabolism--Amino acid metabolism--Glycine, serine and threonine metabolism|Metabolism--Energy metabolism--Methane metabolism|Metabolism--Global and overview maps--Metabolic pathways|Metabolism--Global and overview maps--Microbial metabolism in diverse environments|Metabolism--Global and overview maps--Biosynthesis of antibiotics|Metabolism--Global and overview maps--Carbon metabolism|Metabolism--Global and overview maps--Biosynthesis of amino acids

[denovogenes]_105105	71.92	5e-134	sek:SSPA1155	K03929	pnbA	para-nitrobenzyl esterase	3.1.1.-	--

[denovogenes]_105106	100.00	0.0	pdi:BDI_2803	K07391	comM	magnesium chelatase family protein	--	--

[denovogenes]_105107	100.00	0.0	shq:A0259_19445	K10986	PTS-Gam-EIID, agaD	PTS system, galactosamine-specific IID component	--	Metabolism--Carbohydrate metabolism--Galactose metabolism|Environmental Information Processing--Membrane transport--Phosphotransferase system (PTS)

[denovogenes]_105108	63.89	4e-118	clj:CLJU_c23050	K02588	nifH	nitrogenase iron protein NifH	1.18.6.1	Metabolism--Xenobiotics biodegradation and metabolism--Chloroalkane and chloroalkene degradation|Metabolism--Energy metabolism--Nitrogen metabolism|Metabolism--Global and overview maps--Metabolic pathways|Metabolism--Global and overview maps--Microbial metabolism in diverse environments

[denovogenes]_105111	62.92	3e-126	bcy:Bcer98_2716	K01182	IMA, malL	oligo-1,6-glucosidase	3.2.1.10	Metabolism--Carbohydrate metabolism--Galactose metabolism|Metabolism--Carbohydrate metabolism--Starch and sucrose metabolism|Metabolism--Global and overview maps--Metabolic pathways

[denovogenes]_105115	59.16	2e-108	erb:A4V01_05820	K01624	FBA, fbaA	fructose-bisphosphate aldolase, class II	4.1.2.13	Metabolism--Carbohydrate metabolism--Glycolysis / Gluconeogenesis|Metabolism--Carbohydrate metabolism--Pentose phosphate pathway|Metabolism--Carbohydrate metabolism--Fructose and mannose metabolism|Metabolism--Energy metabolism--Methane metabolism|Metabolism--Energy metabolism--Carbon fixation in photosynthetic organisms|Metabolism--Global and overview maps--Metabolic pathways|Metabolism--Global and overview maps--Biosynthesis of secondary metabolites|Metabolism--Global and overview maps--Microbial metabolism in diverse environments|Metabolism--Global and overview maps--Biosynthesis of antibiotics|Metabolism--Global and overview maps--Carbon metabolism|Metabolism--Global and overview maps--Biosynthesis of amino acids

[denovogenes]_105117	55.86	6e-100	ctet:BN906_02758	K17758	nnr	ADP-dependent NAD(P)H-hydrate dehydratase	4.2.1.136	--

[denovogenes]_105117	55.86	6e-100	ctet:BN906_02758	K17759	AIBP, nnrE	NAD(P)H-hydrate epimerase	5.1.99.6	--

[denovogenes]_105118	92.40	5e-174	bdh:GV66_17865	K07164	K07164	uncharacterized protein	--	--

[denovogenes]_105121	97.72	0.0	ecos:EC958_2380	K00067	rfbD, rmlD	dTDP-4-dehydrorhamnose reductase	1.1.1.133	Metabolism--Biosynthesis of other secondary metabolites--Streptomycin biosynthesis|Metabolism--Metabolism of terpenoids and polyketides--Polyketide sugar unit biosynthesis|Metabolism--Global and overview maps--Biosynthesis of antibiotics

[denovogenes]_105123	40.08	2e-43	fpla:A4U99_04135	K02190	cbiK	sirohydrochlorin cobaltochelatase	4.99.1.3	Metabolism--Metabolism of cofactors and vitamins--Porphyrin and chlorophyll metabolism|Metabolism--Global and overview maps--Metabolic pathways

[denovogenes]_10513	82.68	0.0	shi:Shel_19480	K03696	clpC	ATP-dependent Clp protease ATP-binding subunit ClpC	--	--

[denovogenes]_105131	53.03	1e-90	bck:BCO26_2647	K01972	E6.5.1.2, ligA, ligB	DNA ligase (NAD+)	6.5.1.2	Genetic Information Processing--Replication and repair--DNA replication|Genetic Information Processing--Replication and repair--Base excision repair|Genetic Information Processing--Replication and repair--Nucleotide excision repair|Genetic Information Processing--Replication and repair--Mismatch repair

[denovogenes]_105132	51.52	6e-97	tng:GSTEN00005371G001	K00688	PYG, glgP	glycogen phosphorylase	2.4.1.1	Metabolism--Carbohydrate metabolism--Starch and sucrose metabolism|Metabolism--Global and overview maps--Metabolic pathways|Metabolism--Global and overview maps--Biosynthesis of secondary metabolites|Cellular Processes--Cellular community - prokaryotes--Biofilm formation - Escherichia coli|Organismal Systems--Endocrine system--Insulin signaling pathway|Organismal Systems--Endocrine system--Glucagon signaling pathway|Human Diseases--Endocrine and metabolic diseases--Insulin resistance

[denovogenes]_105133	73.05	5e-140	bprs:CK3_28590	K00868	pdxK, pdxY	pyridoxine kinase	2.7.1.35	Metabolism--Metabolism of cofactors and vitamins--Vitamin B6 metabolism|Metabolism--Global and overview maps--Metabolic pathways

[denovogenes]_105134	57.64	8e-74	ttm:Tthe_1463	K04096	smf	DNA processing protein	--	--

[denovogenes]_105135	66.67	1e-121	bprs:CK3_28720	K01929	murF	UDP-N-acetylmuramoyl-tripeptide--D-alanyl-D-alanine ligase	6.3.2.10	Metabolism--Amino acid metabolism--Lysine biosynthesis|Metabolism--Glycan biosynthesis and metabolism--Peptidoglycan biosynthesis|Metabolism--Global and overview maps--Metabolic pathways|Human Diseases--Drug resistance: Antimicrobial--Vancomycin resistance

[denovogenes]_105138	99.24	0.0	pdi:BDI_0104	K02429	fucP	MFS transporter, FHS family, L-fucose permease	--	--

[denovogenes]_105139	51.70	4e-74	sagr:SAIL_6320	K01537	E3.6.3.8	Ca2+-transporting ATPase	3.6.3.8	--

[denovogenes]_105141	61.30	1e-108	bprl:CL2_29970	K07455	recT	recombination protein RecT	--	--

[denovogenes]_105144	92.02	1e-180	pfre:RM25_1091	K01995	livG	branched-chain amino acid transport system ATP-binding protein	--	Environmental Information Processing--Membrane transport--ABC transporters|Cellular Processes--Cellular community - prokaryotes--Quorum sensing

[denovogenes]_105145	93.54	2e-174	pfre:RM25_1382	K02440	GLPF	glycerol uptake facilitator protein	--	--

[denovogenes]_105146	93.54	9e-180	pfre:RM25_0993	K05833	ABC.X4.A	putative ABC transport system ATP-binding protein	--	--

[denovogenes]_105147	85.02	4e-155	pfre:RM25_0244	K00886	ppgK	polyphosphate glucokinase	2.7.1.63	Metabolism--Carbohydrate metabolism--Glycolysis / Gluconeogenesis|Metabolism--Carbohydrate metabolism--Amino sugar and nucleotide sugar metabolism|Metabolism--Global and overview maps--Metabolic pathways|Metabolism--Global and overview maps--Biosynthesis of secondary metabolites|Metabolism--Global and overview maps--Microbial metabolism in diverse environments|Metabolism--Global and overview maps--Biosynthesis of antibiotics|Metabolism--Global and overview maps--Carbon metabolism

[denovogenes]_105148	86.10	1e-169	pdi:BDI_2584	K02528	ksgA	16S rRNA (adenine1518-N6/adenine1519-N6)-dimethyltransferase	2.1.1.182	--

[denovogenes]_105152	57.25	1e-87	fpr:FP2_23060	K03616	rnfB	electron transport complex protein RnfB	--	--

[denovogenes]_105153	49.63	3e-86	bdh:GV66_14615	K01991	ABC-2.OM, wza	polysaccharide biosynthesis/export protein	--	Cellular Processes--Cellular community - prokaryotes--Biofilm formation - Escherichia coli

[denovogenes]_105154	55.56	3e-75	bprm:CL3_01240	K03424	--	--	--	--

[denovogenes]_105158	73.36	1e-130	dvg:Deval_1684	K00215	dapB	4-hydroxy-tetrahydrodipicolinate reductase	1.17.1.8	Metabolism--Biosynthesis of other secondary metabolites--Monobactam biosynthesis|Metabolism--Amino acid metabolism--Lysine biosynthesis|Metabolism--Global and overview maps--Metabolic pathways|Metabolism--Global and overview maps--Biosynthesis of secondary metabolites|Metabolism--Global and overview maps--Microbial metabolism in diverse environments|Metabolism--Global and overview maps--Biosynthesis of antibiotics|Metabolism--Global and overview maps--Biosynthesis of amino acids

[denovogenes]_105159	99.62	0.0	paeo:M801_237	K01055	pcaD	3-oxoadipate enol-lactonase	3.1.1.24	Metabolism--Xenobiotics biodegradation and metabolism--Benzoate degradation|Metabolism--Global and overview maps--Metabolic pathways|Metabolism--Global and overview maps--Microbial metabolism in diverse environments|Metabolism--Global and overview maps--Degradation of aromatic compounds

[denovogenes]_105164	60.23	1e-116	cpy:Cphy_2914	K00286	proC	pyrroline-5-carboxylate reductase	1.5.1.2	Metabolism--Amino acid metabolism--Arginine and proline metabolism|Metabolism--Global and overview maps--Metabolic pathways|Metabolism--Global and overview maps--Biosynthesis of secondary metabolites|Metabolism--Global and overview maps--Biosynthesis of antibiotics|Metabolism--Global and overview maps--Biosynthesis of amino acids

[denovogenes]_105166	40.37	1e-12	por:APT59_20755	K08083	algR	two-component system, LytT family, response regulator AlgR	--	Environmental Information Processing--Signal transduction--Two-component system

[denovogenes]_10517	99.85	0.0	mmk:MU9_34	K03230	yscV, sctV, hrcV	type III secretion protein V	--	Environmental Information Processing--Membrane transport--Bacterial secretion system

[denovogenes]_105170	79.09	5e-155	rho:RHOM_02780	K03521	fixA, etfB	electron transfer flavoprotein beta subunit	--	--

[denovogenes]_105171	100.00	0.0	paec:M802_3989	K10022	aotJ	arginine/ornithine transport system substrate-binding protein	--	Environmental Information Processing--Membrane transport--ABC transporters

[denovogenes]_105172	79.09	3e-147	pfre:RM25_0692	K03524	birA	BirA family transcriptional regulator, biotin operon repressor / biotin-[acetyl-CoA-carboxylase] ligase	6.3.4.15	Metabolism--Metabolism of cofactors and vitamins--Biotin metabolism|Metabolism--Global and overview maps--Metabolic pathways

[denovogenes]_105175	99.62	0.0	paeu:BN889_04371	K10831	tauB	taurine transport system ATP-binding protein	3.6.3.36	Metabolism--Energy metabolism--Sulfur metabolism|Environmental Information Processing--Membrane transport--ABC transporters

[denovogenes]_105179	57.43	2e-98	gfe:Gferi_20020	K10824	nikE	nickel transport system ATP-binding protein	3.6.3.24	Environmental Information Processing--Membrane transport--ABC transporters

[denovogenes]_105180	98.86	0.0	cfd:CFNIH1_13665	K05522	nei	endonuclease VIII	3.2.2.- 4.2.99.18	Genetic Information Processing--Replication and repair--Base excision repair

[denovogenes]_105181	68.70	4e-123	dvm:DvMF_1441	K06442	tlyA	23S rRNA (cytidine1920-2'-O)/16S rRNA (cytidine1409-2'-O)-methyltransferase	2.1.1.226 2.1.1.227	--

[denovogenes]_105182	81.30	4e-150	bxy:BXY_25280	K00558	DNMT1, dcm	DNA (cytosine-5)-methyltransferase 1	2.1.1.37	Metabolism--Amino acid metabolism--Cysteine and methionine metabolism|Metabolism--Global and overview maps--Metabolic pathways|Human Diseases--Cancers: Overview--MicroRNAs in cancer

[denovogenes]_105183	100.00	0.0	cfd:CFNIH1_14740	K15554	ssuC	sulfonate transport system permease protein	--	Metabolism--Energy metabolism--Sulfur metabolism|Environmental Information Processing--Membrane transport--ABC transporters

[denovogenes]_105185	67.33	1e-127	lir:LAW_01084	K02065	mlaF, linL, mkl	phospholipid/cholesterol/gamma-HCH transport system ATP-binding protein	--	Environmental Information Processing--Membrane transport--ABC transporters

[denovogenes]_105186	42.73	1e-55	rho:RHOM_02055	K13581	ccrM	modification methylase	2.1.1.72	Cellular Processes--Cell growth and death--Cell cycle - Caulobacter

[denovogenes]_105187	61.89	2e-111	fpa:FPR_30190	K00604	MTFMT, fmt	methionyl-tRNA formyltransferase	2.1.2.9	Metabolism--Metabolism of cofactors and vitamins--One carbon pool by folate|Genetic Information Processing--Translation--Aminoacyl-tRNA biosynthesis

[denovogenes]_10519	100.00	0.0	bfs:BF9343_4178	K09181	yfiQ	acetyltransferase	--	--

[denovogenes]_105190	91.25	5e-159	bmr:BMI_II363	K20266	trbJ	type IV secretion system protein TrbJ	--	Cellular Processes--Cellular community - prokaryotes--Quorum sensing

[denovogenes]_105191	100.00	0.0	paec:M802_3353	K03610	minC	septum site-determining protein MinC	--	--

[denovogenes]_105192	58.56	2e-114	ddl:Desdi_0204	K00784	rnz	ribonuclease Z	3.1.26.11	Genetic Information Processing--Translation--RNA transport

[denovogenes]_105193	49.79	3e-79	dat:HRM2_20040	K16203	dppA1	D-amino peptidase	3.4.11.-	--

[denovogenes]_105194	100.00	0.0	fpla:A4U99_01100	K02049	ABC.SN.A	NitT/TauT family transport system ATP-binding protein	--	--

[denovogenes]_105196	88.97	5e-165	bcel:BcellWH2_05262	K11070	potC	spermidine/putrescine transport system permease protein	--	Environmental Information Processing--Membrane transport--ABC transporters

[denovogenes]_105197	49.37	7e-74	aag:AaeL_AAEL011803	K17286	STOM	erythrocyte band 7 integral membrane protein	--	--

[denovogenes]_105199	98.48	0.0	lacy:A4V08_17735	K06346	jag	spoIIIJ-associated protein	--	--

[denovogenes]_1052	99.92	0.0	stb:SGPB_0084	K03046	rpoC	DNA-directed RNA polymerase subunit beta'	2.7.7.6	Metabolism--Nucleotide metabolism--Purine metabolism|Metabolism--Nucleotide metabolism--Pyrimidine metabolism|Metabolism--Global and overview maps--Metabolic pathways|Genetic Information Processing--Transcription--RNA polymerase

[denovogenes]_105202	99.62	0.0	paeu:BN889_05527	K15866	paaG	2-(1,2-epoxy-1,2-dihydrophenyl)acetyl-CoA isomerase	5.3.3.18	Metabolism--Amino acid metabolism--Phenylalanine metabolism|Metabolism--Global and overview maps--Microbial metabolism in diverse environments

[denovogenes]_105204	99.62	0.0	paeb:NCGM1900_2082	K02455	gspF	general secretion pathway protein F	--	Environmental Information Processing--Membrane transport--Bacterial secretion system|Cellular Processes--Cellular community - prokaryotes--Biofilm formation - Vibrio cholerae

[denovogenes]_105205	70.00	5e-131	fnu:FN0400	K02032	ABC.PE.A1	peptide/nickel transport system ATP-binding protein	--	Cellular Processes--Cellular community - prokaryotes--Quorum sensing

[denovogenes]_105206	100.00	0.0	pdi:BDI_0334	K07124	K07124	uncharacterized protein	--	--

[denovogenes]_105207	90.87	2e-172	csh:Closa_2040	K03609	minD	septum site-determining protein MinD	--	--

[denovogenes]_105208	80.58	2e-142	ele:Elen_0239	K02028	ABC.PA.A	polar amino acid transport system ATP-binding protein	3.6.3.21	--

[denovogenes]_105210	57.87	3e-99	paej:H70737_26930	K03784	deoD	purine-nucleoside phosphorylase	2.4.2.1	Metabolism--Nucleotide metabolism--Purine metabolism|Metabolism--Nucleotide metabolism--Pyrimidine metabolism|Metabolism--Metabolism of cofactors and vitamins--Nicotinate and nicotinamide metabolism|Metabolism--Global and overview maps--Metabolic pathways|Metabolism--Global and overview maps--Biosynthesis of secondary metabolites

[denovogenes]_105211	99.62	0.0	fpla:A4U99_01735	K07238	TC.ZIP, zupT, ZRT3, ZIP2	zinc transporter, ZIP family	--	--

[denovogenes]_105212	99.62	0.0	cfd:CFNIH1_00845	K04765	mazG	nucleoside triphosphate diphosphatase	3.6.1.9	Metabolism--Nucleotide metabolism--Purine metabolism|Metabolism--Nucleotide metabolism--Pyrimidine metabolism|Metabolism--Metabolism of cofactors and vitamins--Nicotinate and nicotinamide metabolism|Metabolism--Metabolism of cofactors and vitamins--Pantothenate and CoA biosynthesis|Metabolism--Global and overview maps--Metabolic pathways

[denovogenes]_105216	99.24	0.0	cfd:CFNIH1_10395	K00606	panB	3-methyl-2-oxobutanoate hydroxymethyltransferase	2.1.2.11	Metabolism--Metabolism of cofactors and vitamins--Pantothenate and CoA biosynthesis|Metabolism--Global and overview maps--Metabolic pathways|Metabolism--Global and overview maps--Biosynthesis of secondary metabolites

[denovogenes]_105217	44.10	2e-31	axx:ERS451415_03582	K18900	bpeT	LysR family transcriptional regulator, regulator for bpeEF and oprC	--	--

[denovogenes]_105218	50.97	2e-87	dmt:DESME_00710	K02053	ABC.SP.P	putative spermidine/putrescine transport system permease protein	--	Cellular Processes--Cellular community - prokaryotes--Quorum sensing

[denovogenes]_105219	99.62	0.0	paer:PA1R_gp1080	K02055	ABC.SP.S	putative spermidine/putrescine transport system substrate-binding protein	--	Cellular Processes--Cellular community - prokaryotes--Quorum sensing

[denovogenes]_10522	48.04	0.0	sri:SELR_27200	K02749	PTS-Glv-EIIB, glvB, malP, aglA	PTS system, alpha-glucoside-specific IIB component	2.7.1.208 2.7.1.-	Metabolism--Carbohydrate metabolism--Starch and sucrose metabolism|Environmental Information Processing--Membrane transport--Phosphotransferase system (PTS)

[denovogenes]_10522	48.04	0.0	sri:SELR_27200	K02750	PTS-Glv-EIIC, glvC, malP, aglA	PTS system, alpha-glucoside-specific IIC component	--	Metabolism--Carbohydrate metabolism--Starch and sucrose metabolism|Environmental Information Processing--Membrane transport--Phosphotransferase system (PTS)

[denovogenes]_105221	45.45	1e-66	bln:Blon_1528	K07741	antB	anti-repressor protein	--	--

[denovogenes]_105223	99.62	0.0	lacy:A4V08_00725	K03609	minD	septum site-determining protein MinD	--	--

[denovogenes]_105225	77.95	4e-151	paq:PAGR_g1099	K00058	serA, PHGDH	D-3-phosphoglycerate dehydrogenase	1.1.1.95	Metabolism--Amino acid metabolism--Glycine, serine and threonine metabolism|Metabolism--Energy metabolism--Methane metabolism|Metabolism--Global and overview maps--Metabolic pathways|Metabolism--Global and overview maps--Microbial metabolism in diverse environments|Metabolism--Global and overview maps--Biosynthesis of antibiotics|Metabolism--Global and overview maps--Carbon metabolism|Metabolism--Global and overview maps--Biosynthesis of amino acids

[denovogenes]_105226	100.00	0.0	efn:DENG_00841	K18891	patA, rscA, lmrC, satA	ATP-binding cassette, subfamily B, multidrug efflux pump	--	Environmental Information Processing--Membrane transport--ABC transporters

[denovogenes]_105229	41.94	7e-47	rmr:Rmar_2450	K00059	fabG	3-oxoacyl-[acyl-carrier protein] reductase	1.1.1.100	Metabolism--Lipid metabolism--Fatty acid biosynthesis|Metabolism--Metabolism of cofactors and vitamins--Biotin metabolism|Metabolism--Lipid metabolism--Biosynthesis of unsaturated fatty acids|Metabolism--Global and overview maps--Metabolic pathways|Metabolism--Global and overview maps--Fatty acid metabolism

[denovogenes]_10523	99.71	0.0	eum:ECUMN_3202	K03230	yscV, sctV, hrcV	type III secretion protein V	--	Environmental Information Processing--Membrane transport--Bacterial secretion system

[denovogenes]_105231	100.00	0.0	kpne:KU54_01050	K18825	sul3	dihydropteroate synthase type 3	2.5.1.15	Metabolism--Metabolism of cofactors and vitamins--Folate biosynthesis|Metabolism--Global and overview maps--Metabolic pathways

[denovogenes]_105233	62.74	1e-124	shi:Shel_11870	K02823	pyrDII	dihydroorotate dehydrogenase electron transfer subunit	--	Metabolism--Nucleotide metabolism--Pyrimidine metabolism|Metabolism--Global and overview maps--Metabolic pathways

[denovogenes]_105234	51.91	2e-90	gym:GYMC10_3804	K00059	fabG	3-oxoacyl-[acyl-carrier protein] reductase	1.1.1.100	Metabolism--Lipid metabolism--Fatty acid biosynthesis|Metabolism--Metabolism of cofactors and vitamins--Biotin metabolism|Metabolism--Lipid metabolism--Biosynthesis of unsaturated fatty acids|Metabolism--Global and overview maps--Metabolic pathways|Metabolism--Global and overview maps--Fatty acid metabolism

[denovogenes]_105235	100.00	4e-171	bbrd:BBBR_0418	K09157	K09157	uncharacterized protein	--	--

[denovogenes]_105236	44.25	4e-54	hje:HacjB3_09865	K01066	aes	acetyl esterase	3.1.1.-	--

[denovogenes]_105237	94.68	0.0	lacy:A4V08_33385	K16951	--	--	--	Metabolism--Energy metabolism--Sulfur metabolism|Metabolism--Global and overview maps--Microbial metabolism in diverse environments

[denovogenes]_105238	74.33	2e-146	rho:RHOM_08180	K10194	togN	oligogalacturonide transport system permease protein	--	Environmental Information Processing--Membrane transport--ABC transporters

[denovogenes]_10524	62.07	0.0	cbv:U729_37	K07037	K07037	uncharacterized protein	--	--

[denovogenes]_105240	95.63	0.0	bfb:VU15_11860	K01126	E3.1.4.46, glpQ, ugpQ	glycerophosphoryl diester phosphodiesterase	3.1.4.46	Metabolism--Lipid metabolism--Glycerophospholipid metabolism

[denovogenes]_105241	75.86	3e-152	rto:RTO_16120	K07124	K07124	uncharacterized protein	--	--

[denovogenes]_105244	93.54	1e-169	lacy:A4V08_33230	K03613	rnfE	electron transport complex protein RnfE	--	--

[denovogenes]_105245	58.26	6e-84	cgo:Corgl_0463	K01990	ABC-2.A	ABC-2 type transport system ATP-binding protein	--	--

[denovogenes]_105247	46.01	7e-74	med:MELS_0178	K03424	--	--	--	--

[denovogenes]_105249	95.45	0.0	lacy:A4V08_34950	K11755	hisIE	phosphoribosyl-ATP pyrophosphohydrolase / phosphoribosyl-AMP cyclohydrolase	3.6.1.31 3.5.4.19	Metabolism--Amino acid metabolism--Histidine metabolism|Metabolism--Global and overview maps--Metabolic pathways|Metabolism--Global and overview maps--Biosynthesis of secondary metabolites|Metabolism--Global and overview maps--Biosynthesis of amino acids

[denovogenes]_10525	99.85	0.0	efl:EF62_2184	K19005	ltaS	lipoteichoic acid synthase	2.7.8.20	Metabolism--Lipid metabolism--Glycerolipid metabolism|Metabolism--Global and overview maps--Metabolic pathways

[denovogenes]_105250	81.75	9e-143	pmr:PMI0168	K07312	ynfH	Tat-targeted selenate reductase subunit YnfH	--	--

[denovogenes]_105251	82.13	3e-156	lacy:A4V08_06850	K02519	infB, MTIF2	translation initiation factor IF-2	--	--

[denovogenes]_105252	62.21	8e-121	bxy:BXY_16850	K00929	E2.7.2.7, buk	butyrate kinase	2.7.2.7	Metabolism--Carbohydrate metabolism--Butanoate metabolism|Metabolism--Global and overview maps--Metabolic pathways

[denovogenes]_105254	82.89	3e-165	bcel:BcellWH2_03840	K03524	birA	BirA family transcriptional regulator, biotin operon repressor / biotin-[acetyl-CoA-carboxylase] ligase	6.3.4.15	Metabolism--Metabolism of cofactors and vitamins--Biotin metabolism|Metabolism--Global and overview maps--Metabolic pathways

[denovogenes]_105256	72.24	5e-136	cgo:Corgl_1455	K01915	glnA, GLUL	glutamine synthetase	6.3.1.2	Metabolism--Amino acid metabolism--Arginine biosynthesis|Metabolism--Amino acid metabolism--Alanine, aspartate and glutamate metabolism|Metabolism--Carbohydrate metabolism--Glyoxylate and dicarboxylate metabolism|Metabolism--Energy metabolism--Nitrogen metabolism|Metabolism--Global and overview maps--Metabolic pathways|Metabolism--Global and overview maps--Microbial metabolism in diverse environments|Metabolism--Global and overview maps--Biosynthesis of amino acids|Environmental Information Processing--Signal transduction--Two-component system|Organismal Systems--Nervous system--Glutamatergic synapse|Organismal Systems--Nervous system--GABAergic synapse

[denovogenes]_105257	60.53	1e-110	bbi:BBIF_0181	K03484	scrR	LacI family transcriptional regulator, sucrose operon repressor	--	--

[denovogenes]_105258	95.83	7e-175	eyy:EGYY_05950	K02945	RP-S1, rpsA	small subunit ribosomal protein S1	--	Genetic Information Processing--Translation--Ribosome

[denovogenes]_105259	83.53	2e-145	bprm:CL3_08380	K02010	afuC, fbpC	iron(III) transport system ATP-binding protein	3.6.3.30	Environmental Information Processing--Membrane transport--ABC transporters

[denovogenes]_105260	85.93	7e-172	bfb:VU15_08065	K03799	htpX	heat shock protein HtpX	3.4.24.-	--

[denovogenes]_105261	63.02	2e-102	ele:Elen_1646	K09761	rsmE	16S rRNA (uracil1498-N3)-methyltransferase	2.1.1.193	--

[denovogenes]_105262	40.12	4e-23	pacn:TIA1EST1_07685	K00059	fabG	3-oxoacyl-[acyl-carrier protein] reductase	1.1.1.100	Metabolism--Lipid metabolism--Fatty acid biosynthesis|Metabolism--Metabolism of cofactors and vitamins--Biotin metabolism|Metabolism--Lipid metabolism--Biosynthesis of unsaturated fatty acids|Metabolism--Global and overview maps--Metabolic pathways|Metabolism--Global and overview maps--Fatty acid metabolism

[denovogenes]_105266	88.26	8e-174	bcel:BcellWH2_00333	K04720	cobD	threonine-phosphate decarboxylase	4.1.1.81	Metabolism--Metabolism of cofactors and vitamins--Porphyrin and chlorophyll metabolism

[denovogenes]_105269	51.98	1e-87	emu:EMQU_1023	K02025	ABC.MS.P	multiple sugar transport system permease protein	--	--

[denovogenes]_10527	64.42	0.0	ipo:Ilyop_1697	K03655	recG	ATP-dependent DNA helicase RecG	3.6.4.12	Genetic Information Processing--Replication and repair--Homologous recombination

[denovogenes]_105270	71.97	4e-129	swo:Swol_0984	K07133	K07133	uncharacterized protein	--	--

[denovogenes]_105273	67.05	6e-126	lacy:A4V08_11155	K19294	algI	alginate O-acetyltransferase complex protein AlgI	--	--

[denovogenes]_105274	76.14	6e-147	rob:CK5_00540	K02031	ABC.PE.A	peptide/nickel transport system ATP-binding protein	--	Cellular Processes--Cellular community - prokaryotes--Quorum sensing

[denovogenes]_105276	100.00	0.0	bpsc:BBPC_1773	K18333	K18333	L-fucose dehydrogenase	--	Metabolism--Carbohydrate metabolism--Fructose and mannose metabolism|Metabolism--Global and overview maps--Microbial metabolism in diverse environments

[denovogenes]_105277	46.12	2e-66	pln:Plano_2697	K11690	dctM	C4-dicarboxylate transporter, DctM subunit	--	Environmental Information Processing--Signal transduction--Two-component system

[denovogenes]_105279	99.24	0.0	cama:F384_01480	K02012	afuA, fbpA	iron(III) transport system substrate-binding protein	--	Environmental Information Processing--Membrane transport--ABC transporters

[denovogenes]_10528	99.56	0.0	bxy:BXY_20740	K09181	yfiQ	acetyltransferase	--	--

[denovogenes]_105280	94.30	3e-179	csh:Closa_2190	K03086	SIG1, rpoD	RNA polymerase primary sigma factor	--	--

[denovogenes]_105283	56.27	4e-105	pta:HPL003_21840	K07406	melA	alpha-galactosidase	3.2.1.22	Metabolism--Carbohydrate metabolism--Galactose metabolism|Metabolism--Lipid metabolism--Glycerolipid metabolism|Metabolism--Lipid metabolism--Sphingolipid metabolism|Metabolism--Glycan biosynthesis and metabolism--Glycosphingolipid biosynthesis - globo and isoglobo series

[denovogenes]_105285	75.00	5e-139	shi:Shel_13520	K02346	DPO4, dinB	DNA polymerase IV	2.7.7.7	--

[denovogenes]_105287	82.95	5e-149	eyy:EGYY_01470	K03307	TC.SSS	solute:Na+ symporter, SSS family	--	--

[denovogenes]_105289	82.13	4e-157	cso:CLS_13850	K03306	TC.PIT	inorganic phosphate transporter, PiT family	--	--

[denovogenes]_105290	72.09	6e-141	erb:A4V01_08970	K03590	ftsA	cell division protein FtsA	--	Cellular Processes--Cell growth and death--Cell cycle - Caulobacter

[denovogenes]_105297	84.85	2e-161	sdz:Asd1617_03656	K05367	pbpC	penicillin-binding protein 1C	2.4.1.-	Metabolism--Glycan biosynthesis and metabolism--Peptidoglycan biosynthesis

[denovogenes]_105298	65.77	1e-124	clb:Clo1100_0149	K10118	msmF	raffinose/stachyose/melibiose transport system permease protein	--	Environmental Information Processing--Membrane transport--ABC transporters

[denovogenes]_105299	99.23	4e-176	cdg:CDBI1_01615	K10440	rbsC	ribose transport system permease protein	--	Environmental Information Processing--Membrane transport--ABC transporters

[denovogenes]_105300	85.61	2e-161	byl:A4V09_14460	K03308	TC.NSS	neurotransmitter:Na+ symporter, NSS family	--	--

[denovogenes]_105301	100.00	0.0	bbrs:BS27_1530	K00574	cfa	cyclopropane-fatty-acyl-phospholipid synthase	2.1.1.79	--

[denovogenes]_105302	81.86	2e-143	rto:RTO_09410	K07816	E2.7.6.5X	putative GTP pyrophosphokinase	2.7.6.5	Metabolism--Nucleotide metabolism--Purine metabolism

[denovogenes]_105303	94.30	1e-177	pmib:BB2000_2507	K02316	dnaG	DNA primase	2.7.7.-	Genetic Information Processing--Replication and repair--DNA replication

[denovogenes]_105304	64.39	5e-121	bdh:GV66_11705	K00075	murB	UDP-N-acetylmuramate dehydrogenase	1.3.1.98	Metabolism--Carbohydrate metabolism--Amino sugar and nucleotide sugar metabolism|Metabolism--Glycan biosynthesis and metabolism--Peptidoglycan biosynthesis|Metabolism--Global and overview maps--Metabolic pathways

[denovogenes]_105306	75.00	5e-141	cgo:Corgl_0044	K01835	pgm	phosphoglucomutase	5.4.2.2	Metabolism--Carbohydrate metabolism--Glycolysis / Gluconeogenesis|Metabolism--Carbohydrate metabolism--Pentose phosphate pathway|Metabolism--Carbohydrate metabolism--Galactose metabolism|Metabolism--Nucleotide metabolism--Purine metabolism|Metabolism--Carbohydrate metabolism--Starch and sucrose metabolism|Metabolism--Carbohydrate metabolism--Amino sugar and nucleotide sugar metabolism|Metabolism--Biosynthesis of other secondary metabolites--Streptomycin biosynthesis|Metabolism--Global and overview maps--Metabolic pathways|Metabolism--Global and overview maps--Biosynthesis of secondary metabolites|Metabolism--Global and overview maps--Microbial metabolism in diverse environments|Metabolism--Global and overview maps--Biosynthesis of antibiotics

[denovogenes]_105307	95.83	5e-175	afd:Alfi_1443	K03588	ftsW, spoVE	cell division protein FtsW	--	Cellular Processes--Cell growth and death--Cell cycle - Caulobacter

[denovogenes]_10531	91.89	0.0	bxy:BXY_48620	K07386	pepO	putative endopeptidase	3.4.24.-	--

[denovogenes]_105312	67.42	3e-134	bacc:BRDCF_p2077	K04041	fbp3	fructose-1,6-bisphosphatase III	3.1.3.11	Metabolism--Carbohydrate metabolism--Glycolysis / Gluconeogenesis|Metabolism--Carbohydrate metabolism--Pentose phosphate pathway|Metabolism--Carbohydrate metabolism--Fructose and mannose metabolism|Metabolism--Energy metabolism--Methane metabolism|Metabolism--Energy metabolism--Carbon fixation in photosynthetic organisms|Metabolism--Global and overview maps--Metabolic pathways|Metabolism--Global and overview maps--Biosynthesis of secondary metabolites|Metabolism--Global and overview maps--Microbial metabolism in diverse environments|Metabolism--Global and overview maps--Biosynthesis of antibiotics|Metabolism--Global and overview maps--Carbon metabolism

[denovogenes]_105315	96.59	0.0	pmib:BB2000_1932	K01885	EARS, gltX	glutamyl-tRNA synthetase	6.1.1.17	Metabolism--Metabolism of cofactors and vitamins--Porphyrin and chlorophyll metabolism|Genetic Information Processing--Translation--Aminoacyl-tRNA biosynthesis|Metabolism--Global and overview maps--Metabolic pathways|Metabolism--Global and overview maps--Biosynthesis of secondary metabolites

[denovogenes]_105324	100.00	0.0	cfd:CFNIH1_06765	K01914	asnA	aspartate--ammonia ligase	6.3.1.1	Metabolism--Amino acid metabolism--Alanine, aspartate and glutamate metabolism|Metabolism--Metabolism of other amino acids--Cyanoamino acid metabolism|Metabolism--Global and overview maps--Metabolic pathways|Metabolism--Global and overview maps--Biosynthesis of secondary metabolites|Metabolism--Global and overview maps--Biosynthesis of amino acids

[denovogenes]_105327	40.38	2e-63	mmn:midi_01256	K01873	VARS, valS	valyl-tRNA synthetase	6.1.1.9	Genetic Information Processing--Translation--Aminoacyl-tRNA biosynthesis

[denovogenes]_105331	42.70	3e-60	bado:BBMN23_0470	K05349	bglX	beta-glucosidase	3.2.1.21	Metabolism--Metabolism of other amino acids--Cyanoamino acid metabolism|Metabolism--Carbohydrate metabolism--Starch and sucrose metabolism|Metabolism--Biosynthesis of other secondary metabolites--Phenylpropanoid biosynthesis|Metabolism--Global and overview maps--Metabolic pathways|Metabolism--Global and overview maps--Biosynthesis of secondary metabolites

[denovogenes]_105332	79.55	3e-161	llr:llh_0475	K19068	wbjC	UDP-2-acetamido-2,6-beta-L-arabino-hexul-4-ose reductase	1.1.1.367	--

[denovogenes]_105336	80.15	1e-154	pdi:BDI_1664	K01785	galM, GALM	aldose 1-epimerase	5.1.3.3	Metabolism--Carbohydrate metabolism--Glycolysis / Gluconeogenesis|Metabolism--Carbohydrate metabolism--Galactose metabolism|Metabolism--Global and overview maps--Metabolic pathways|Metabolism--Global and overview maps--Biosynthesis of secondary metabolites|Metabolism--Global and overview maps--Microbial metabolism in diverse environments|Metabolism--Global and overview maps--Biosynthesis of antibiotics

[denovogenes]_105337	42.48	4e-67	mro:MROS_1490	K01187	malZ	alpha-glucosidase	3.2.1.20	Metabolism--Carbohydrate metabolism--Galactose metabolism|Metabolism--Carbohydrate metabolism--Starch and sucrose metabolism|Metabolism--Global and overview maps--Metabolic pathways

[denovogenes]_105338	90.91	8e-172	bcel:BcellWH2_04019	K00343	nuoN	NADH-quinone oxidoreductase subunit N	1.6.5.3	Metabolism--Energy metabolism--Oxidative phosphorylation|Metabolism--Global and overview maps--Metabolic pathways

[denovogenes]_105339	98.86	0.0	bcel:BcellWH2_05489	K00645	fabD	[acyl-carrier-protein] S-malonyltransferase	2.3.1.39	Metabolism--Lipid metabolism--Fatty acid biosynthesis|Metabolism--Global and overview maps--Metabolic pathways|Metabolism--Global and overview maps--Fatty acid metabolism

[denovogenes]_10534	97.81	0.0	bxy:BXY_20740	K09181	yfiQ	acetyltransferase	--	--

[denovogenes]_105340	51.76	7e-83	dpg:DESPIGER_0190	K03733	xerC	integrase/recombinase XerC	--	--

[denovogenes]_105342	45.45	8e-21	lrl:LC705_00842	K07741	antB	anti-repressor protein	--	--

[denovogenes]_105344	98.86	0.0	bxy:BXY_28970	K02564	nagB, GNPDA	glucosamine-6-phosphate deaminase	3.5.99.6	Metabolism--Carbohydrate metabolism--Amino sugar and nucleotide sugar metabolism|Metabolism--Global and overview maps--Metabolic pathways

[denovogenes]_105345	99.23	0.0	boa:Bovatus_01563	K04486	E3.1.3.15B	histidinol-phosphatase (PHP family)	3.1.3.15	Metabolism--Amino acid metabolism--Histidine metabolism|Metabolism--Global and overview maps--Metabolic pathways|Metabolism--Global and overview maps--Biosynthesis of secondary metabolites|Metabolism--Global and overview maps--Biosynthesis of amino acids

[denovogenes]_10535	100.00	0.0	bvu:BVU_3108	K16089	TC.FEV.OM2, cirA, cfrA, hmuR	outer membrane receptor for ferrienterochelin and colicins	--	--

[denovogenes]_105350	40.75	2e-65	erb:A4V01_02300	K04486	E3.1.3.15B	histidinol-phosphatase (PHP family)	3.1.3.15	Metabolism--Amino acid metabolism--Histidine metabolism|Metabolism--Global and overview maps--Metabolic pathways|Metabolism--Global and overview maps--Biosynthesis of secondary metabolites|Metabolism--Global and overview maps--Biosynthesis of amino acids

[denovogenes]_105354	44.49	4e-69	toc:Toce_0386	K06864	K06864	uncharacterized protein	--	--

[denovogenes]_105355	99.62	0.0	boa:Bovatus_04524	K04486	E3.1.3.15B	histidinol-phosphatase (PHP family)	3.1.3.15	Metabolism--Amino acid metabolism--Histidine metabolism|Metabolism--Global and overview maps--Metabolic pathways|Metabolism--Global and overview maps--Biosynthesis of secondary metabolites|Metabolism--Global and overview maps--Biosynthesis of amino acids

[denovogenes]_105357	53.88	9e-101	lacy:A4V08_33240	K03616	rnfB	electron transport complex protein RnfB	--	--

[denovogenes]_10536	91.68	0.0	boa:Bovatus_04529	K01284	dcp	peptidyl-dipeptidase Dcp	3.4.15.5	--

[denovogenes]_105361	81.85	2e-150	euc:EC1_01390	K00059	fabG	3-oxoacyl-[acyl-carrier protein] reductase	1.1.1.100	Metabolism--Lipid metabolism--Fatty acid biosynthesis|Metabolism--Metabolism of cofactors and vitamins--Biotin metabolism|Metabolism--Lipid metabolism--Biosynthesis of unsaturated fatty acids|Metabolism--Global and overview maps--Metabolic pathways|Metabolism--Global and overview maps--Fatty acid metabolism

[denovogenes]_105362	42.23	1e-61	cpe:CPE0197	K03481	glvR	RpiR family transcriptional regulator, glv operon transcriptional regulator	--	--

[denovogenes]_105363	53.05	2e-56	etr:ETAE_1677	K19303	mepH	murein DD-endopeptidase	3.4.-.-	--

[denovogenes]_105364	46.80	2e-80	aex:Astex_2725	K12308	bgaB, lacA	beta-galactosidase	3.2.1.23	Metabolism--Carbohydrate metabolism--Galactose metabolism

[denovogenes]_105365	79.17	1e-149	vpr:Vpar_1113	K02563	murG	UDP-N-acetylglucosamine--N-acetylmuramyl-(pentapeptide) pyrophosphoryl-undecaprenol N-acetylglucosamine transferase	2.4.1.227	Metabolism--Glycan biosynthesis and metabolism--Peptidoglycan biosynthesis|Metabolism--Global and overview maps--Metabolic pathways|Human Diseases--Drug resistance: Antimicrobial--Vancomycin resistance|Cellular Processes--Cell growth and death--Cell cycle - Caulobacter

[denovogenes]_105369	48.29	6e-85	fnu:FN1161	K01776	murI	glutamate racemase	5.1.1.3	Metabolism--Metabolism of other amino acids--D-Glutamine and D-glutamate metabolism|Metabolism--Global and overview maps--Metabolic pathways

[denovogenes]_10537	99.42	0.0	bxy:BXY_33460	K01284	dcp	peptidyl-dipeptidase Dcp	3.4.15.5	--

[denovogenes]_105370	100.00	0.0	eoi:ECO111_3639	K01223	E3.2.1.86B, bglA	6-phospho-beta-glucosidase	3.2.1.86	Metabolism--Carbohydrate metabolism--Glycolysis / Gluconeogenesis|Metabolism--Carbohydrate metabolism--Starch and sucrose metabolism

[denovogenes]_105372	50.38	4e-86	ace:Acel_0509	K00370	narG, narZ, nxrA	nitrate reductase / nitrite oxidoreductase, alpha subunit	1.7.5.1 1.7.99.4	Metabolism--Energy metabolism--Nitrogen metabolism|Metabolism--Global and overview maps--Microbial metabolism in diverse environments|Environmental Information Processing--Signal transduction--Two-component system

[denovogenes]_105373	75.95	2e-143	fus:HMPREF0409_01281	K03609	minD	septum site-determining protein MinD	--	--

[denovogenes]_105374	91.25	7e-174	vpr:Vpar_1562	K02035	ABC.PE.S	peptide/nickel transport system substrate-binding protein	--	Cellular Processes--Cellular community - prokaryotes--Quorum sensing

[denovogenes]_105375	50.78	7e-94	fhw:RN87_07120	K02013	ABC.FEV.A	iron complex transport system ATP-binding protein	3.6.3.34	Environmental Information Processing--Membrane transport--ABC transporters

[denovogenes]_105378	100.00	0.0	cpe:CPE2631	K02795	PTS-Man-EIIC, manY	PTS system, mannose-specific IIC component	--	Metabolism--Carbohydrate metabolism--Fructose and mannose metabolism|Metabolism--Carbohydrate metabolism--Amino sugar and nucleotide sugar metabolism|Metabolism--Global and overview maps--Metabolic pathways|Environmental Information Processing--Membrane transport--Phosphotransferase system (PTS)

[denovogenes]_105379	95.37	1e-179	ega:AL523_05240	K03553	recA	recombination protein RecA	--	Genetic Information Processing--Replication and repair--Homologous recombination

[denovogenes]_10538	46.41	0.0	rch:RUM_03890	K12573	rnr, vacB	ribonuclease R	3.1.-.-	Genetic Information Processing--Folding, sorting and degradation--RNA degradation

[denovogenes]_105380	100.00	0.0	bvu:BVU_3112	K07085	K07085	putative transport protein	--	--

[denovogenes]_105382	43.48	4e-71	fnu:FN1161	K01776	murI	glutamate racemase	5.1.1.3	Metabolism--Metabolism of other amino acids--D-Glutamine and D-glutamate metabolism|Metabolism--Global and overview maps--Metabolic pathways

[denovogenes]_105383	95.08	8e-176	vpr:Vpar_0576	K03106	SRP54, ffh	signal recognition particle subunit SRP54	3.6.5.4	Cellular Processes--Cellular community - prokaryotes--Quorum sensing|Genetic Information Processing--Folding, sorting and degradation--Protein export|Environmental Information Processing--Membrane transport--Bacterial secretion system

[denovogenes]_105384	99.24	0.0	ecol:LY180_02080	K11921	cynR	LysR family transcriptional regulator, cyn operon transcriptional activator	--	--

[denovogenes]_105386	71.15	1e-136	puf:UFO1_3232	K00175	korB, oorB, oforB	2-oxoglutarate/2-oxoacid ferredoxin oxidoreductase subunit beta	1.2.7.3 1.2.7.11	Metabolism--Carbohydrate metabolism--Glycolysis / Gluconeogenesis|Metabolism--Carbohydrate metabolism--Citrate cycle (TCA cycle)|Metabolism--Carbohydrate metabolism--Pyruvate metabolism|Metabolism--Carbohydrate metabolism--Butanoate metabolism|Metabolism--Energy metabolism--Carbon fixation pathways in prokaryotes|Metabolism--Global and overview maps--Metabolic pathways|Metabolism--Global and overview maps--Microbial metabolism in diverse environments|Metabolism--Global and overview maps--Biosynthesis of antibiotics|Metabolism--Global and overview maps--Carbon metabolism

[denovogenes]_105387	100.00	0.0	pvl:AOB99_09125	K06217	phoH, phoL	phosphate starvation-inducible protein PhoH and related proteins	--	--

[denovogenes]_105389	100.00	0.0	pvl:AOB99_07345	K01575	alsD, budA, aldC	acetolactate decarboxylase	4.1.1.5	Metabolism--Carbohydrate metabolism--Butanoate metabolism|Metabolism--Carbohydrate metabolism--C5-Branched dibasic acid metabolism

[denovogenes]_10539	65.40	0.0	amt:Amet_4520	K00527	nrdD	ribonucleoside-triphosphate reductase	1.17.4.2	Metabolism--Nucleotide metabolism--Purine metabolism|Metabolism--Nucleotide metabolism--Pyrimidine metabolism|Metabolism--Global and overview maps--Metabolic pathways

[denovogenes]_105390	53.44	4e-90	mhg:MHY_13590	K03070	secA	preprotein translocase subunit SecA	--	Cellular Processes--Cellular community - prokaryotes--Quorum sensing|Genetic Information Processing--Folding, sorting and degradation--Protein export|Environmental Information Processing--Membrane transport--Bacterial secretion system

[denovogenes]_105393	100.00	0.0	pvl:AOB99_14130	K03324	yjbB	phosphate:Na+ symporter	--	--

[denovogenes]_105396	100.00	0.0	pmr:PMI2117	K10716	kch, trkA, mthK, pch	voltage-gated potassium channel	--	--

[denovogenes]_105397	100.00	0.0	sbc:SbBS512_E4821	K01255	CARP, pepA	leucyl aminopeptidase	3.4.11.1	Metabolism--Metabolism of other amino acids--Glutathione metabolism|Metabolism--Global and overview maps--Metabolic pathways

[denovogenes]_1054	71.56	0.0	bfs:BF9343_0283	K01191	E3.2.1.24	alpha-mannosidase	3.2.1.24	Metabolism--Glycan biosynthesis and metabolism--Other glycan degradation

[denovogenes]_10540	52.88	2e-132	lmi:LMXM_16_0440	K05305	FUK	fucokinase	2.7.1.52	Metabolism--Carbohydrate metabolism--Fructose and mannose metabolism|Metabolism--Carbohydrate metabolism--Amino sugar and nucleotide sugar metabolism|Metabolism--Global and overview maps--Metabolic pathways

[denovogenes]_105401	68.99	5e-128	cbut:ATN24_08385	K03437	spoU	RNA methyltransferase, TrmH family	--	--

[denovogenes]_105403	99.62	0.0	mmk:MU9_1542	K06219	smtA	S-adenosylmethionine-dependent methyltransferase	--	--

[denovogenes]_105404	99.62	0.0	mmk:MU9_1598	K07124	K07124	uncharacterized protein	--	--

[denovogenes]_105405	98.24	1e-162	mmk:MU9_1736	K07165	fecR	transmembrane sensor	--	--

[denovogenes]_105407	99.62	0.0	mmk:MU9_2989	K02018	modB	molybdate transport system permease protein	--	Environmental Information Processing--Membrane transport--ABC transporters

[denovogenes]_105409	99.62	0.0	mmk:MU9_1160	K04765	mazG	nucleoside triphosphate diphosphatase	3.6.1.9	Metabolism--Nucleotide metabolism--Purine metabolism|Metabolism--Nucleotide metabolism--Pyrimidine metabolism|Metabolism--Metabolism of cofactors and vitamins--Nicotinate and nicotinamide metabolism|Metabolism--Metabolism of cofactors and vitamins--Pantothenate and CoA biosynthesis|Metabolism--Global and overview maps--Metabolic pathways

[denovogenes]_10541	99.42	0.0	btho:Btheta7330_00173	K09181	yfiQ	acetyltransferase	--	--

[denovogenes]_105411	100.00	0.0	boa:Bovatus_01060	K03496	parA, soj	chromosome partitioning protein	--	--

[denovogenes]_105413	87.36	1e-162	lacy:A4V08_33240	K03616	rnfB	electron transport complex protein RnfB	--	--

[denovogenes]_105414	99.24	0.0	mmk:MU9_2379	K00208	fabI	enoyl-[acyl-carrier protein] reductase I	1.3.1.9 1.3.1.10	Metabolism--Lipid metabolism--Fatty acid biosynthesis|Metabolism--Metabolism of cofactors and vitamins--Biotin metabolism|Metabolism--Global and overview maps--Metabolic pathways|Metabolism--Global and overview maps--Fatty acid metabolism

[denovogenes]_105416	40.31	2e-61	tfo:BFO_0548	K07271	licD	lipopolysaccharide cholinephosphotransferase	2.7.8.-	--

[denovogenes]_105417	100.00	0.0	bfb:VU15_12215	K11070	potC	spermidine/putrescine transport system permease protein	--	Environmental Information Processing--Membrane transport--ABC transporters

[denovogenes]_105419	86.64	1e-170	clt:CM240_2983	K02013	ABC.FEV.A	iron complex transport system ATP-binding protein	3.6.3.34	Environmental Information Processing--Membrane transport--ABC transporters

[denovogenes]_10542	99.71	0.0	bth:BT_0186	K04084	dsbD	thiol:disulfide interchange protein DsbD	1.8.1.8	--

[denovogenes]_105421	100.00	0.0	bfb:VU15_20120	K07052	K07052	uncharacterized protein	--	--

[denovogenes]_105422	100.00	0.0	bfb:VU15_12455	K01991	ABC-2.OM, wza	polysaccharide biosynthesis/export protein	--	Cellular Processes--Cellular community - prokaryotes--Biofilm formation - Escherichia coli

[denovogenes]_105423	99.62	0.0	mmk:MU9_1006	K05895	cobK-cbiJ	precorrin-6A/cobalt-precorrin-6A reductase	1.3.1.54 1.3.1.106	Metabolism--Metabolism of cofactors and vitamins--Porphyrin and chlorophyll metabolism|Metabolism--Global and overview maps--Metabolic pathways

[denovogenes]_105424	54.25	8e-89	ers:K210_08965	K19508	PTS-Gfr-EIIC, gfrC	PTS system, fructoselysine/glucoselysine-specific IIC component	--	Environmental Information Processing--Membrane transport--Phosphotransferase system (PTS)

[denovogenes]_105426	70.11	1e-128	lacy:A4V08_31185	K02392	flgG	flagellar basal-body rod protein FlgG	--	Cellular Processes--Cell motility--Flagellar assembly

[denovogenes]_105427	69.73	3e-131	cbv:U729_1449	K01243	mtnN, mtn, pfs	adenosylhomocysteine nucleosidase	3.2.2.9	Metabolism--Amino acid metabolism--Cysteine and methionine metabolism|Metabolism--Global and overview maps--Metabolic pathways|Metabolism--Global and overview maps--Biosynthesis of amino acids

[denovogenes]_105429	73.66	3e-145	cbv:U729_2010	K02051	ABC.SN.S	NitT/TauT family transport system substrate-binding protein	--	--

[denovogenes]_105431	100.00	0.0	stb:SGPB_0286	K18474	fabM	trans-2-decenoyl-[acyl-carrier protein] isomerase	5.3.3.14	Metabolism--Lipid metabolism--Fatty acid biosynthesis

[denovogenes]_105432	100.00	0.0	efq:DR75_2104	K09685	purR	purine operon repressor	--	--

[denovogenes]_105436	99.16	3e-168	efn:DENG_01488	K01992	ABC-2.P	ABC-2 type transport system permease protein	--	--

[denovogenes]_105438	73.60	2e-139	cad:Curi_c19980	K02049	ABC.SN.A	NitT/TauT family transport system ATP-binding protein	--	--

[denovogenes]_10544	100.00	0.0	bth:BT_1890	K03657	uvrD, pcrA	DNA helicase II / ATP-dependent DNA helicase PcrA	3.6.4.12	Genetic Information Processing--Replication and repair--Nucleotide excision repair|Genetic Information Processing--Replication and repair--Mismatch repair

[denovogenes]_105440	84.03	1e-150	cdg:CDBI1_17600	K01591	pyrF	orotidine-5'-phosphate decarboxylase	4.1.1.23	Metabolism--Nucleotide metabolism--Pyrimidine metabolism|Metabolism--Global and overview maps--Metabolic pathways

[denovogenes]_105441	52.14	6e-89	rus:RBI_I00525	K03616	rnfB	electron transport complex protein RnfB	--	--

[denovogenes]_105442	100.00	0.0	efd:EFD32_0358	K00046	idnO	gluconate 5-dehydrogenase	1.1.1.69	--

[denovogenes]_105443	100.00	0.0	stb:SGPB_1589	K04069	pflA, pflC, pflE	pyruvate formate lyase activating enzyme	1.97.1.4	--

[denovogenes]_105444	100.00	0.0	stb:SGPB_1591	K02013	ABC.FEV.A	iron complex transport system ATP-binding protein	3.6.3.34	Environmental Information Processing--Membrane transport--ABC transporters

[denovogenes]_105445	100.00	0.0	bfb:VU15_04095	K04486	E3.1.3.15B	histidinol-phosphatase (PHP family)	3.1.3.15	Metabolism--Amino acid metabolism--Histidine metabolism|Metabolism--Global and overview maps--Metabolic pathways|Metabolism--Global and overview maps--Biosynthesis of secondary metabolites|Metabolism--Global and overview maps--Biosynthesis of amino acids

[denovogenes]_105446	100.00	0.0	efd:EFD32_2480	K01962	accA	acetyl-CoA carboxylase carboxyl transferase subunit alpha	6.4.1.2	Metabolism--Lipid metabolism--Fatty acid biosynthesis|Metabolism--Carbohydrate metabolism--Pyruvate metabolism|Metabolism--Carbohydrate metabolism--Propanoate metabolism|Metabolism--Energy metabolism--Carbon fixation pathways in prokaryotes|Metabolism--Global and overview maps--Metabolic pathways|Metabolism--Global and overview maps--Biosynthesis of secondary metabolites|Metabolism--Global and overview maps--Microbial metabolism in diverse environments|Metabolism--Global and overview maps--Biosynthesis of antibiotics|Metabolism--Global and overview maps--Carbon metabolism|Metabolism--Global and overview maps--Fatty acid metabolism

[denovogenes]_105448	100.00	0.0	efi:OG1RF_11841	K03584	recO	DNA repair protein RecO (recombination protein O)	--	Genetic Information Processing--Replication and repair--Homologous recombination

[denovogenes]_105449	66.41	2e-133	ctc:CTC_00509	K03337	iolB	5-deoxy-glucuronate isomerase	5.3.1.30	Metabolism--Carbohydrate metabolism--Inositol phosphate metabolism|Metabolism--Global and overview maps--Metabolic pathways|Metabolism--Global and overview maps--Microbial metabolism in diverse environments

[denovogenes]_105450	99.62	0.0	ene:ENT_10990	K07124	K07124	uncharacterized protein	--	--

[denovogenes]_105452	54.79	2e-104	bha:BH3444	K07497	K07497	putative transposase	--	--

[denovogenes]_105453	94.27	2e-176	cfd:CFNIH1_12230	K07308	dmsC	anaerobic dimethyl sulfoxide reductase subunit C (DMSO reductase anchor subunit)	--	Metabolism--Energy metabolism--Sulfur metabolism

[denovogenes]_105454	69.92	3e-126	cbk:CLL_A1659	K00948	PRPS, prsA	ribose-phosphate pyrophosphokinase	2.7.6.1	Metabolism--Carbohydrate metabolism--Pentose phosphate pathway|Metabolism--Nucleotide metabolism--Purine metabolism|Metabolism--Global and overview maps--Metabolic pathways|Metabolism--Global and overview maps--Biosynthesis of secondary metabolites|Metabolism--Global and overview maps--Microbial metabolism in diverse environments|Metabolism--Global and overview maps--Biosynthesis of antibiotics|Metabolism--Global and overview maps--Carbon metabolism|Metabolism--Global and overview maps--Biosynthesis of amino acids

[denovogenes]_105457	59.30	5e-106	slr:L21SP2_0631	K01995	livG	branched-chain amino acid transport system ATP-binding protein	--	Environmental Information Processing--Membrane transport--ABC transporters|Cellular Processes--Cellular community - prokaryotes--Quorum sensing

[denovogenes]_105458	100.00	0.0	pvl:AOB99_01855	K07090	K07090	uncharacterized protein	--	--

[denovogenes]_10546	89.65	0.0	pdi:BDI_1804	K09181	yfiQ	acetyltransferase	--	--

[denovogenes]_105460	100.00	0.0	stb:SGPB_1079	K11070	potC	spermidine/putrescine transport system permease protein	--	Environmental Information Processing--Membrane transport--ABC transporters

[denovogenes]_105462	58.56	6e-106	cbv:U729_1279	K04075	tilS, mesJ	tRNA(Ile)-lysidine synthase	6.3.4.19	--

[denovogenes]_105463	40.89	4e-50	cag:Cagg_0630	K00059	fabG	3-oxoacyl-[acyl-carrier protein] reductase	1.1.1.100	Metabolism--Lipid metabolism--Fatty acid biosynthesis|Metabolism--Metabolism of cofactors and vitamins--Biotin metabolism|Metabolism--Lipid metabolism--Biosynthesis of unsaturated fatty acids|Metabolism--Global and overview maps--Metabolic pathways|Metabolism--Global and overview maps--Fatty acid metabolism

[denovogenes]_105464	62.69	4e-114	bhu:bhn_I1905	K01609	trpC	indole-3-glycerol phosphate synthase	4.1.1.48	Metabolism--Amino acid metabolism--Phenylalanine, tyrosine and tryptophan biosynthesis|Metabolism--Global and overview maps--Metabolic pathways|Metabolism--Global and overview maps--Biosynthesis of secondary metabolites|Metabolism--Global and overview maps--Biosynthesis of antibiotics|Metabolism--Global and overview maps--Biosynthesis of amino acids

[denovogenes]_105466	100.00	0.0	cbut:ATN24_03025	K03521	fixA, etfB	electron transfer flavoprotein beta subunit	--	--

[denovogenes]_105467	41.53	4e-51	yee:YE5303_10431	K07346	fimC	fimbrial chaperone protein	--	--

[denovogenes]_105468	63.57	7e-111	vpr:Vpar_1719	K00266	gltD	glutamate synthase (NADPH/NADH) small chain	1.4.1.13 1.4.1.14	Metabolism--Amino acid metabolism--Alanine, aspartate and glutamate metabolism|Metabolism--Energy metabolism--Nitrogen metabolism|Metabolism--Global and overview maps--Metabolic pathways|Metabolism--Global and overview maps--Biosynthesis of secondary metabolites|Metabolism--Global and overview maps--Microbial metabolism in diverse environments|Metabolism--Global and overview maps--Biosynthesis of antibiotics|Metabolism--Global and overview maps--Biosynthesis of amino acids

[denovogenes]_105471	99.62	0.0	cif:AL515_04720	K00758	deoA, TYMP	thymidine phosphorylase	2.4.2.4	Metabolism--Nucleotide metabolism--Pyrimidine metabolism|Metabolism--Xenobiotics biodegradation and metabolism--Drug metabolism - other enzymes|Metabolism--Global and overview maps--Metabolic pathways|Human Diseases--Cancers: Specific types--Bladder cancer

[denovogenes]_105472	72.24	2e-132	cbt:CLH_2096	K07258	dacC, dacA, dacD	D-alanyl-D-alanine carboxypeptidase (penicillin-binding protein 5/6)	3.4.16.4	Metabolism--Glycan biosynthesis and metabolism--Peptidoglycan biosynthesis|Metabolism--Global and overview maps--Metabolic pathways

[denovogenes]_105479	99.62	0.0	shq:A0259_04750	K00677	lpxA	UDP-N-acetylglucosamine acyltransferase	2.3.1.129	Metabolism--Glycan biosynthesis and metabolism--Lipopolysaccharide biosynthesis|Metabolism--Global and overview maps--Metabolic pathways|Human Diseases--Drug resistance: Antimicrobial--Cationic antimicrobial peptide (CAMP) resistance

[denovogenes]_10548	100.00	0.0	fpla:A4U99_12935	K02355	fusA, GFM, EFG	elongation factor G	--	--

[denovogenes]_105480	70.72	9e-127	clt:CM240_1224	K06901	pbuG	putative MFS transporter, AGZA family, xanthine/uracil permease	--	--

[denovogenes]_105482	100.00	0.0	cbut:ATN24_01380	K03497	parB, spo0J	chromosome partitioning protein, ParB family	--	--

[denovogenes]_105483	69.20	6e-134	vpr:Vpar_1222	K05837	rodA, mrdB	rod shape determining protein RodA	--	--

[denovogenes]_105484	94.66	0.0	prg:RB151_001270	K00262	E1.4.1.4, gdhA	glutamate dehydrogenase (NADP+)	1.4.1.4	Metabolism--Amino acid metabolism--Arginine biosynthesis|Metabolism--Amino acid metabolism--Alanine, aspartate and glutamate metabolism|Metabolism--Energy metabolism--Nitrogen metabolism|Metabolism--Global and overview maps--Metabolic pathways

[denovogenes]_105485	99.62	0.0	ecw:EcE24377A_0075	K01703	leuC	3-isopropylmalate/(R)-2-methylmalate dehydratase large subunit	4.2.1.33 4.2.1.35	Metabolism--Amino acid metabolism--Valine, leucine and isoleucine biosynthesis|Metabolism--Carbohydrate metabolism--C5-Branched dibasic acid metabolism|Metabolism--Global and overview maps--Metabolic pathways|Metabolism--Global and overview maps--Biosynthesis of secondary metabolites|Metabolism--Global and overview maps--2-Oxocarboxylic acid metabolism|Metabolism--Global and overview maps--Biosynthesis of amino acids

[denovogenes]_105486	59.22	1e-93	gfe:Gferi_24430	K02057	ABC.SS.P	simple sugar transport system permease protein	--	--

[denovogenes]_105488	91.25	2e-179	psx:DR96_1104	K00133	asd	aspartate-semialdehyde dehydrogenase	1.2.1.11	Metabolism--Amino acid metabolism--Glycine, serine and threonine metabolism|Metabolism--Biosynthesis of other secondary metabolites--Monobactam biosynthesis|Metabolism--Amino acid metabolism--Cysteine and methionine metabolism|Metabolism--Amino acid metabolism--Lysine biosynthesis|Metabolism--Global and overview maps--Metabolic pathways|Metabolism--Global and overview maps--Biosynthesis of secondary metabolites|Metabolism--Global and overview maps--Microbial metabolism in diverse environments|Metabolism--Global and overview maps--Biosynthesis of antibiotics|Metabolism--Global and overview maps--2-Oxocarboxylic acid metabolism|Metabolism--Global and overview maps--Biosynthesis of amino acids

[denovogenes]_105491	56.11	7e-104	paen:P40081_27580	K16248	gutA, gutP	probable glucitol transport protein GutA	--	--

[denovogenes]_105492	98.09	0.0	cfd:CFNIH1_09645	K03800	lplA, lplJ	lipoate---protein ligase	6.3.1.20	Metabolism--Metabolism of cofactors and vitamins--Lipoic acid metabolism|Metabolism--Global and overview maps--Metabolic pathways

[denovogenes]_105494	100.00	0.0	shq:A0259_14995	K00526	E1.17.4.1B, nrdB, nrdF	ribonucleoside-diphosphate reductase beta chain	1.17.4.1	Metabolism--Nucleotide metabolism--Purine metabolism|Metabolism--Nucleotide metabolism--Pyrimidine metabolism|Metabolism--Global and overview maps--Metabolic pathways

[denovogenes]_105496	68.32	2e-129	cle:Clole_1001	K19294	algI	alginate O-acetyltransferase complex protein AlgI	--	--

[denovogenes]_105497	41.15	3e-64	lci:LCK_00797	K03292	TC.GPH	glycoside/pentoside/hexuronide:cation symporter, GPH family	--	--

[denovogenes]_105499	100.00	0.0	shq:A0259_19945	K03568	tldD	TldD protein	--	--

[denovogenes]_10550	43.97	0.0	gel:IB49_09905	K06978	K06978	uncharacterized protein	--	--

[denovogenes]_105501	82.06	2e-158	cif:AL515_08750	K00984	aadA	streptomycin 3"-adenylyltransferase	2.7.7.47	--

[denovogenes]_105502	100.00	0.0	pmib:BB2000_3353	K00973	E2.7.7.24, rfbA, rffH	glucose-1-phosphate thymidylyltransferase	2.7.7.24	Metabolism--Biosynthesis of other secondary metabolites--Streptomycin biosynthesis|Metabolism--Metabolism of terpenoids and polyketides--Polyketide sugar unit biosynthesis|Metabolism--Biosynthesis of other secondary metabolites--Acarbose and validamycin biosynthesis|Metabolism--Global and overview maps--Biosynthesis of antibiotics

[denovogenes]_105503	77.95	2e-145	cbut:ATN24_18095	K03312	gltS	glutamate:Na+ symporter, ESS family	--	--

[denovogenes]_105504	51.72	2e-78	csb:CLSA_c30600	K00873	PK, pyk	pyruvate kinase	2.7.1.40	Metabolism--Carbohydrate metabolism--Glycolysis / Gluconeogenesis|Metabolism--Nucleotide metabolism--Purine metabolism|Metabolism--Carbohydrate metabolism--Pyruvate metabolism|Metabolism--Global and overview maps--Metabolic pathways|Metabolism--Global and overview maps--Biosynthesis of secondary metabolites|Metabolism--Global and overview maps--Microbial metabolism in diverse environments|Metabolism--Global and overview maps--Biosynthesis of antibiotics|Metabolism--Global and overview maps--Carbon metabolism|Metabolism--Global and overview maps--Biosynthesis of amino acids|Organismal Systems--Endocrine system--Glucagon signaling pathway|Human Diseases--Endocrine and metabolic diseases--Type II diabetes mellitus|Human Diseases--Cancers: Overview--Viral carcinogenesis|Human Diseases--Cancers: Overview--Central carbon metabolism in cancer

[denovogenes]_105505	100.00	0.0	cif:AL515_19235	K01480	speB	agmatinase	3.5.3.11	Metabolism--Amino acid metabolism--Arginine and proline metabolism|Metabolism--Global and overview maps--Metabolic pathways

[denovogenes]_105507	100.00	0.0	cbut:ATN24_15440	K02035	ABC.PE.S	peptide/nickel transport system substrate-binding protein	--	Cellular Processes--Cellular community - prokaryotes--Quorum sensing

[denovogenes]_105508	100.00	0.0	shq:A0259_18245	K01480	speB	agmatinase	3.5.3.11	Metabolism--Amino acid metabolism--Arginine and proline metabolism|Metabolism--Global and overview maps--Metabolic pathways

[denovogenes]_10551	63.81	0.0	bur:Bcep18194_B0574	K00219	fadH	2,4-dienoyl-CoA reductase (NADPH2)	1.3.1.34	--

[denovogenes]_105511	100.00	0.0	cbut:ATN24_15645	K01866	YARS, tyrS	tyrosyl-tRNA synthetase	6.1.1.1	Genetic Information Processing--Translation--Aminoacyl-tRNA biosynthesis

[denovogenes]_105512	75.48	8e-133	cpe:CPE0529	K07088	K07088	uncharacterized protein	--	--

[denovogenes]_105514	69.32	1e-123	cbn:CbC4_0023	K01338	lon	ATP-dependent Lon protease	3.4.21.53	Cellular Processes--Cell growth and death--Cell cycle - Caulobacter

[denovogenes]_105519	69.20	2e-131	cst:CLOST_2139	K02446	glpX	fructose-1,6-bisphosphatase II	3.1.3.11	Metabolism--Carbohydrate metabolism--Glycolysis / Gluconeogenesis|Metabolism--Carbohydrate metabolism--Pentose phosphate pathway|Metabolism--Carbohydrate metabolism--Fructose and mannose metabolism|Metabolism--Energy metabolism--Methane metabolism|Metabolism--Energy metabolism--Carbon fixation in photosynthetic organisms|Metabolism--Global and overview maps--Metabolic pathways|Metabolism--Global and overview maps--Biosynthesis of secondary metabolites|Metabolism--Global and overview maps--Microbial metabolism in diverse environments|Metabolism--Global and overview maps--Biosynthesis of antibiotics|Metabolism--Global and overview maps--Carbon metabolism

[denovogenes]_105523	99.24	0.0	cif:AL515_06550	K18988	ampH	D-alanyl-D-alanine-carboxypeptidase / D-alanyl-D-alanine-endopeptidase	3.4.-.-	--

[denovogenes]_105524	67.30	1e-119	clt:CM240_0950	K01255	CARP, pepA	leucyl aminopeptidase	3.4.11.1	Metabolism--Metabolism of other amino acids--Glutathione metabolism|Metabolism--Global and overview maps--Metabolic pathways

[denovogenes]_105525	72.95	1e-121	cbv:U729_1281	K03798	ftsH, hflB	cell division protease FtsH	3.4.24.-	--

[denovogenes]_105528	67.56	2e-135	ppol:X809_06425	K06909	xtmB	phage terminase large subunit	--	--

[denovogenes]_105529	59.35	7e-103	dor:Desor_2897	K09861	K09861	uncharacterized protein	--	--

[denovogenes]_10553	99.42	0.0	cfd:CFNIH1_05245	K07290	yhjG	AsmA family protein	--	--

[denovogenes]_105533	59.14	4e-116	pnp:IJ22_36040	K02049	ABC.SN.A	NitT/TauT family transport system ATP-binding protein	--	--

[denovogenes]_105534	88.93	4e-176	bcel:BcellWH2_01770	K03474	pdxJ	pyridoxine 5-phosphate synthase	2.6.99.2	Metabolism--Metabolism of cofactors and vitamins--Vitamin B6 metabolism|Metabolism--Global and overview maps--Metabolic pathways

[denovogenes]_105536	82.35	4e-149	lacy:A4V08_02340	K03706	codY	transcriptional pleiotropic repressor	--	--

[denovogenes]_105537	77.38	2e-144	rob:CK5_23990	K05936	cobM, cbiF	precorrin-4/cobalt-precorrin-4 C11-methyltransferase	2.1.1.133 2.1.1.271	Metabolism--Metabolism of cofactors and vitamins--Porphyrin and chlorophyll metabolism|Metabolism--Global and overview maps--Metabolic pathways

[denovogenes]_105538	99.62	0.0	bvu:BVU_0071	K06989	nadX	aspartate dehydrogenase	1.4.1.21	Metabolism--Metabolism of cofactors and vitamins--Nicotinate and nicotinamide metabolism|Metabolism--Global and overview maps--Metabolic pathways

[denovogenes]_105540	100.00	0.0	bvu:BVU_3325	K01092	E3.1.3.25, IMPA, suhB	myo-inositol-1(or 4)-monophosphatase	3.1.3.25	Metabolism--Biosynthesis of other secondary metabolites--Streptomycin biosynthesis|Metabolism--Carbohydrate metabolism--Inositol phosphate metabolism|Metabolism--Global and overview maps--Metabolic pathways|Environmental Information Processing--Signal transduction--Phosphatidylinositol signaling system

[denovogenes]_105541	57.53	2e-96	elm:ELI_1665	K01609	trpC	indole-3-glycerol phosphate synthase	4.1.1.48	Metabolism--Amino acid metabolism--Phenylalanine, tyrosine and tryptophan biosynthesis|Metabolism--Global and overview maps--Metabolic pathways|Metabolism--Global and overview maps--Biosynthesis of secondary metabolites|Metabolism--Global and overview maps--Biosynthesis of antibiotics|Metabolism--Global and overview maps--Biosynthesis of amino acids

[denovogenes]_105543	64.02	6e-116	rto:RTO_30380	K03437	spoU	RNA methyltransferase, TrmH family	--	--

[denovogenes]_105544	98.47	0.0	bxy:BXY_45640	K03426	E3.6.1.22, NUDT12, nudC	NAD+ diphosphatase	3.6.1.22	Metabolism--Metabolism of cofactors and vitamins--Nicotinate and nicotinamide metabolism|Metabolism--Global and overview maps--Metabolic pathways|Cellular Processes--Transport and catabolism--Peroxisome

[denovogenes]_105546	42.68	5e-57	lacy:A4V08_31165	K02421	fliR	flagellar biosynthetic protein FliR	--	Cellular Processes--Cell motility--Flagellar assembly

[denovogenes]_105548	68.77	8e-123	csh:Closa_3439	K03496	parA, soj	chromosome partitioning protein	--	--

[denovogenes]_105549	56.00	3e-104	csh:Closa_3447	K02405	fliA	RNA polymerase sigma factor for flagellar operon FliA	--	Environmental Information Processing--Signal transduction--Two-component system|Cellular Processes--Cellular community - prokaryotes--Biofilm formation - Pseudomonas aeruginosa|Cellular Processes--Cellular community - prokaryotes--Biofilm formation - Escherichia coli|Cellular Processes--Cell motility--Flagellar assembly|Cellular Processes--Cellular community - prokaryotes--Biofilm formation - Vibrio cholerae

[denovogenes]_10555	98.98	0.0	pdi:BDI_3779	K01277	DPP3	dipeptidyl-peptidase III	3.4.14.4	--

[denovogenes]_105550	87.02	6e-174	btho:Btheta7330_01524	K03426	E3.6.1.22, NUDT12, nudC	NAD+ diphosphatase	3.6.1.22	Metabolism--Metabolism of cofactors and vitamins--Nicotinate and nicotinamide metabolism|Metabolism--Global and overview maps--Metabolic pathways|Cellular Processes--Transport and catabolism--Peroxisome

[denovogenes]_105551	84.62	1e-159	fpla:A4U99_16715	K02499	yabN	tetrapyrrole methylase family protein / MazG family protein	--	--

[denovogenes]_105554	99.24	0.0	bdh:GV66_15970	K05306	phnX	phosphonoacetaldehyde hydrolase	3.11.1.1	Metabolism--Metabolism of other amino acids--Phosphonate and phosphinate metabolism|Metabolism--Global and overview maps--Metabolic pathways|Metabolism--Global and overview maps--Microbial metabolism in diverse environments

[denovogenes]_105559	78.93	1e-149	fpla:A4U99_04285	K02028	ABC.PA.A	polar amino acid transport system ATP-binding protein	3.6.3.21	--

[denovogenes]_10556	69.68	0.0	dvm:DvMF_1590	K01915	glnA, GLUL	glutamine synthetase	6.3.1.2	Metabolism--Amino acid metabolism--Arginine biosynthesis|Metabolism--Amino acid metabolism--Alanine, aspartate and glutamate metabolism|Metabolism--Carbohydrate metabolism--Glyoxylate and dicarboxylate metabolism|Metabolism--Energy metabolism--Nitrogen metabolism|Metabolism--Global and overview maps--Metabolic pathways|Metabolism--Global and overview maps--Microbial metabolism in diverse environments|Metabolism--Global and overview maps--Biosynthesis of amino acids|Environmental Information Processing--Signal transduction--Two-component system|Organismal Systems--Nervous system--Glutamatergic synapse|Organismal Systems--Nervous system--GABAergic synapse

[denovogenes]_105560	67.30	7e-128	rto:RTO_25600	K01814	hisA	phosphoribosylformimino-5-aminoimidazole carboxamide ribotide isomerase	5.3.1.16	Metabolism--Amino acid metabolism--Histidine metabolism|Metabolism--Global and overview maps--Metabolic pathways|Metabolism--Global and overview maps--Biosynthesis of secondary metabolites|Metabolism--Global and overview maps--Biosynthesis of amino acids

[denovogenes]_105561	73.73	2e-138	ibu:IB211_02608c	K07056	rsmI	16S rRNA (cytidine1402-2'-O)-methyltransferase	2.1.1.198	--

[denovogenes]_105562	44.88	2e-46	cly:Celly_0300	K05799	pdhR	GntR family transcriptional regulator, transcriptional repressor for pyruvate dehydrogenase complex	--	--

[denovogenes]_105563	72.90	8e-137	gfe:Gferi_09785	K00059	fabG	3-oxoacyl-[acyl-carrier protein] reductase	1.1.1.100	Metabolism--Lipid metabolism--Fatty acid biosynthesis|Metabolism--Metabolism of cofactors and vitamins--Biotin metabolism|Metabolism--Lipid metabolism--Biosynthesis of unsaturated fatty acids|Metabolism--Global and overview maps--Metabolic pathways|Metabolism--Global and overview maps--Fatty acid metabolism

[denovogenes]_10557	99.42	0.0	pdi:BDI_2739	K01206	FUCA	alpha-L-fucosidase	3.2.1.51	Metabolism--Glycan biosynthesis and metabolism--Other glycan degradation

[denovogenes]_105573	49.42	3e-74	lacy:A4V08_25725	K06608	iolR	DeoR family transcriptional regulator, myo-inositol catabolism operon repressor	--	--

[denovogenes]_105574	100.00	0.0	btho:Btheta7330_02546	K03924	moxR	MoxR-like ATPase	3.6.3.-	--

[denovogenes]_105576	56.30	2e-96	cpor:BED41_05565	K01630	garL	2-dehydro-3-deoxyglucarate aldolase	4.1.2.20	Metabolism--Carbohydrate metabolism--Ascorbate and aldarate metabolism

[denovogenes]_105577	75.77	2e-139	ova:OBV_23600	K06442	tlyA	23S rRNA (cytidine1920-2'-O)/16S rRNA (cytidine1409-2'-O)-methyltransferase	2.1.1.226 2.1.1.227	--

[denovogenes]_105578	56.75	3e-105	bip:Bint_1414	K15554	ssuC	sulfonate transport system permease protein	--	Metabolism--Energy metabolism--Sulfur metabolism|Environmental Information Processing--Membrane transport--ABC transporters

[denovogenes]_105579	100.00	0.0	esl:O3K_13500	K15866	paaG	2-(1,2-epoxy-1,2-dihydrophenyl)acetyl-CoA isomerase	5.3.3.18	Metabolism--Amino acid metabolism--Phenylalanine metabolism|Metabolism--Global and overview maps--Microbial metabolism in diverse environments

[denovogenes]_105582	65.22	7e-48	rob:CK5_12910	K03436	fruR2, fruR	DeoR family transcriptional regulator, fructose operon transcriptional repressor	--	--

[denovogenes]_105584	100.00	0.0	ecol:LY180_22815	K02510	hpaI, hpcH	4-hydroxy-2-oxoheptanedioate aldolase	4.1.2.52	Metabolism--Amino acid metabolism--Tyrosine metabolism|Metabolism--Global and overview maps--Microbial metabolism in diverse environments

[denovogenes]_105585	89.69	7e-173	fpla:A4U99_01820	K02886	RP-L2, MRPL2, rplB	large subunit ribosomal protein L2	--	Genetic Information Processing--Translation--Ribosome

[denovogenes]_105586	54.62	1e-98	fnt:HMPREF0405_01724	K17836	penP	beta-lactamase class A	3.5.2.6	Metabolism--Biosynthesis of other secondary metabolites--Penicillin and cephalosporin biosynthesis|Metabolism--Global and overview maps--Biosynthesis of antibiotics|Human Diseases--Drug resistance: Antimicrobial--beta-Lactam resistance

[denovogenes]_105588	55.34	1e-104	pft:JBW_04278	K02013	ABC.FEV.A	iron complex transport system ATP-binding protein	3.6.3.34	Environmental Information Processing--Membrane transport--ABC transporters

[denovogenes]_10559	71.82	0.0	pmuc:ING2E5A_2274	K05989	ramA	alpha-L-rhamnosidase	3.2.1.40	--

[denovogenes]_105590	71.19	1e-119	bprm:CL3_06240	K02028	ABC.PA.A	polar amino acid transport system ATP-binding protein	3.6.3.21	--

[denovogenes]_105592	40.00	5e-54	bco:Bcell_3649	K16698	tuaG	teichuronic acid biosynthesis glycosyltransferase TuaG	2.4.-.-	--

[denovogenes]_105593	41.96	3e-15	mph:MLP_04780	K18910	dpe, lre	D-psicose/D-tagatose/L-ribulose 3-epimerase	5.1.3.30 5.1.3.31	--

[denovogenes]_105596	78.33	1e-146	ibu:IB211_03042c	K02886	RP-L2, MRPL2, rplB	large subunit ribosomal protein L2	--	Genetic Information Processing--Translation--Ribosome

[denovogenes]_105597	100.00	0.0	fpla:A4U99_01820	K02886	RP-L2, MRPL2, rplB	large subunit ribosomal protein L2	--	Genetic Information Processing--Translation--Ribosome

[denovogenes]_1056	99.83	0.0	mmk:MU9_1521	K03466	ftsK, spoIIIE	DNA segregation ATPase FtsK/SpoIIIE, S-DNA-T family	--	--

[denovogenes]_105604	99.24	0.0	lacy:A4V08_02340	K03706	codY	transcriptional pleiotropic repressor	--	--

[denovogenes]_105605	43.02	4e-54	ral:Rumal_2214	K02493	hemK, prmC, HEMK	release factor glutamine methyltransferase	2.1.1.297	--

[denovogenes]_105609	47.29	7e-86	fpr:FP2_10620	K07720	yesN	two-component system, response regulator YesN	--	Environmental Information Processing--Signal transduction--Two-component system

[denovogenes]_105610	62.69	4e-107	bbru:Bbr_1430	K16785	ecfT	energy-coupling factor transport system permease protein	--	Environmental Information Processing--Membrane transport--ABC transporters

[denovogenes]_105612	74.62	4e-148	cpy:Cphy_3780	K01990	ABC-2.A	ABC-2 type transport system ATP-binding protein	--	--

[denovogenes]_105613	75.00	2e-125	cgo:Corgl_1283	K02795	PTS-Man-EIIC, manY	PTS system, mannose-specific IIC component	--	Metabolism--Carbohydrate metabolism--Fructose and mannose metabolism|Metabolism--Carbohydrate metabolism--Amino sugar and nucleotide sugar metabolism|Metabolism--Global and overview maps--Metabolic pathways|Environmental Information Processing--Membrane transport--Phosphotransferase system (PTS)

[denovogenes]_105615	64.62	2e-118	cpor:BED41_12195	K12942	abgT	aminobenzoyl-glutamate transport protein	--	--

[denovogenes]_105616	88.93	5e-174	koc:AB185_21650	K15866	paaG	2-(1,2-epoxy-1,2-dihydrophenyl)acetyl-CoA isomerase	5.3.3.18	Metabolism--Amino acid metabolism--Phenylalanine metabolism|Metabolism--Global and overview maps--Microbial metabolism in diverse environments

[denovogenes]_10562	98.25	0.0	bxy:BXY_20740	K09181	yfiQ	acetyltransferase	--	--

[denovogenes]_105621	98.47	0.0	cif:AL515_05650	K00677	lpxA	UDP-N-acetylglucosamine acyltransferase	2.3.1.129	Metabolism--Glycan biosynthesis and metabolism--Lipopolysaccharide biosynthesis|Metabolism--Global and overview maps--Metabolic pathways|Human Diseases--Drug resistance: Antimicrobial--Cationic antimicrobial peptide (CAMP) resistance

[denovogenes]_105622	99.24	0.0	btho:Btheta7330_03750	K02428	rdgB	XTP/dITP diphosphohydrolase	3.6.1.66	Metabolism--Nucleotide metabolism--Purine metabolism

[denovogenes]_105625	73.75	5e-150	twi:Thewi_0856	K18011	kamE	beta-lysine 5,6-aminomutase beta subunit	5.4.3.3	Metabolism--Amino acid metabolism--Lysine degradation

[denovogenes]_105626	45.77	3e-72	ccel:CCDG5_1744	K01992	ABC-2.P	ABC-2 type transport system permease protein	--	--

[denovogenes]_105629	72.09	3e-135	cpy:Cphy_2448	K00575	cheR	chemotaxis protein methyltransferase CheR	2.1.1.80	Environmental Information Processing--Signal transduction--Two-component system|Cellular Processes--Cell motility--Bacterial chemotaxis

[denovogenes]_105631	96.20	0.0	bvu:BVU_3337	K00609	pyrB, PYR2	aspartate carbamoyltransferase catalytic subunit	2.1.3.2	Metabolism--Nucleotide metabolism--Pyrimidine metabolism|Metabolism--Amino acid metabolism--Alanine, aspartate and glutamate metabolism|Metabolism--Global and overview maps--Metabolic pathways

[denovogenes]_105635	92.37	8e-178	fpla:A4U99_01820	K02886	RP-L2, MRPL2, rplB	large subunit ribosomal protein L2	--	Genetic Information Processing--Translation--Ribosome

[denovogenes]_105637	90.42	1e-178	cfd:CFNIH1_22845	K13014	arnD	undecaprenyl phosphate-alpha-L-ara4FN deformylase	3.5.1.-	Metabolism--Carbohydrate metabolism--Amino sugar and nucleotide sugar metabolism|Human Diseases--Drug resistance: Antimicrobial--Cationic antimicrobial peptide (CAMP) resistance

[denovogenes]_105638	100.00	0.0	sft:NCTC1_00572	K02019	modE	molybdate transport system regulatory protein	--	--

[denovogenes]_105639	65.00	4e-87	rum:CK1_02070	K04759	feoB	ferrous iron transport protein B	--	--

[denovogenes]_105646	56.27	4e-92	shi:Shel_16310	K15986	ppaC	manganese-dependent inorganic pyrophosphatase	3.6.1.1	Metabolism--Energy metabolism--Oxidative phosphorylation

[denovogenes]_105648	79.77	6e-153	ral:Rumal_0039	K02886	RP-L2, MRPL2, rplB	large subunit ribosomal protein L2	--	Genetic Information Processing--Translation--Ribosome

[denovogenes]_10565	94.02	0.0	bsa:Bacsa_2532	K03169	topB	DNA topoisomerase III	5.99.1.2	--

[denovogenes]_105651	60.41	1e-98	cct:CC1_11570	K03308	TC.NSS	neurotransmitter:Na+ symporter, NSS family	--	--

[denovogenes]_105652	98.46	0.0	fpla:A4U99_13280	K04032	eutT	ethanolamine utilization cobalamin adenosyltransferase	2.5.1.17	Metabolism--Metabolism of cofactors and vitamins--Porphyrin and chlorophyll metabolism|Metabolism--Global and overview maps--Metabolic pathways

[denovogenes]_105655	100.00	0.0	fpla:A4U99_02910	K18011	kamE	beta-lysine 5,6-aminomutase beta subunit	5.4.3.3	Metabolism--Amino acid metabolism--Lysine degradation

[denovogenes]_105656	48.89	4e-73	smw:SMWW4_v1c06200	K12519	papD	chaperone protein PapD	--	--

[denovogenes]_105657	71.37	2e-139	bprs:CK3_00450	K00757	udp, UPP	uridine phosphorylase	2.4.2.3	Metabolism--Nucleotide metabolism--Pyrimidine metabolism|Metabolism--Xenobiotics biodegradation and metabolism--Drug metabolism - other enzymes|Metabolism--Global and overview maps--Metabolic pathways

[denovogenes]_105659	98.85	0.0	cif:AL515_16685	K07223	K07223	putative iron-dependent peroxidase	--	--

[denovogenes]_10566	99.85	0.0	pmr:PMI2212	K07347	fimD, fimC, mrkC, htrE, cssD	outer membrane usher protein	--	Human Diseases--Infectious diseases: Bacterial--Pertussis

[denovogenes]_105661	83.27	2e-161	cbut:ATN24_12245	K03438	mraW, rsmH	16S rRNA (cytosine1402-N4)-methyltransferase	2.1.1.199	--

[denovogenes]_105662	63.04	9e-112	carc:NY10_1389	K10189	lacF, araP	lactose/L-arabinose transport system permease protein	--	Environmental Information Processing--Membrane transport--ABC transporters

[denovogenes]_105664	62.21	2e-121	cpy:Cphy_3719	K01839	deoB	phosphopentomutase	5.4.2.7	Metabolism--Carbohydrate metabolism--Pentose phosphate pathway|Metabolism--Nucleotide metabolism--Purine metabolism

[denovogenes]_105665	56.02	1e-91	erb:A4V01_15895	K01270	pepD	dipeptidase D	3.4.13.-	Metabolism--Metabolism of other amino acids--Glutathione metabolism|Metabolism--Global and overview maps--Metabolic pathways

[denovogenes]_105666	42.86	4e-14	nbr:O3I_002670	K16050	hsaD	4,5:9,10-diseco-3-hydroxy-5,9,17-trioxoandrosta-1(10),2-diene-4-oate hydrolase	3.7.1.17	Metabolism--Xenobiotics biodegradation and metabolism--Steroid degradation|Metabolism--Global and overview maps--Metabolic pathways|Metabolism--Global and overview maps--Microbial metabolism in diverse environments|Metabolism--Global and overview maps--Degradation of aromatic compounds

[denovogenes]_105668	100.00	0.0	ece:Z5403	K02495	hemN, hemZ	oxygen-independent coproporphyrinogen III oxidase	1.3.98.3	Metabolism--Metabolism of cofactors and vitamins--Porphyrin and chlorophyll metabolism|Metabolism--Global and overview maps--Metabolic pathways|Metabolism--Global and overview maps--Biosynthesis of secondary metabolites

[denovogenes]_10567	100.00	0.0	bsa:Bacsa_2532	K03169	topB	DNA topoisomerase III	5.99.1.2	--

[denovogenes]_105671	95.42	0.0	bhl:Bache_2185	K00606	panB	3-methyl-2-oxobutanoate hydroxymethyltransferase	2.1.2.11	Metabolism--Metabolism of cofactors and vitamins--Pantothenate and CoA biosynthesis|Metabolism--Global and overview maps--Metabolic pathways|Metabolism--Global and overview maps--Biosynthesis of secondary metabolites

[denovogenes]_105672	44.83	5e-81	byl:A4V09_21080	K00817	hisC	histidinol-phosphate aminotransferase	2.6.1.9	Metabolism--Amino acid metabolism--Histidine metabolism|Metabolism--Amino acid metabolism--Tyrosine metabolism|Metabolism--Amino acid metabolism--Phenylalanine metabolism|Metabolism--Amino acid metabolism--Phenylalanine, tyrosine and tryptophan biosynthesis|Metabolism--Biosynthesis of other secondary metabolites--Novobiocin biosynthesis|Metabolism--Biosynthesis of other secondary metabolites--Tropane, piperidine and pyridine alkaloid biosynthesis|Metabolism--Global and overview maps--Metabolic pathways|Metabolism--Global and overview maps--Biosynthesis of secondary metabolites|Metabolism--Global and overview maps--Biosynthesis of antibiotics|Metabolism--Global and overview maps--Biosynthesis of amino acids

[denovogenes]_105673	89.35	6e-171	byl:A4V09_05090	K01754	E4.3.1.19, ilvA, tdcB	threonine dehydratase	4.3.1.19	Metabolism--Amino acid metabolism--Glycine, serine and threonine metabolism|Metabolism--Amino acid metabolism--Valine, leucine and isoleucine biosynthesis|Metabolism--Global and overview maps--Metabolic pathways|Metabolism--Global and overview maps--Biosynthesis of secondary metabolites|Metabolism--Global and overview maps--Biosynthesis of antibiotics|Metabolism--Global and overview maps--Carbon metabolism|Metabolism--Global and overview maps--Biosynthesis of amino acids

[denovogenes]_105674	78.01	1e-144	ert:EUR_08670	K02564	nagB, GNPDA	glucosamine-6-phosphate deaminase	3.5.99.6	Metabolism--Carbohydrate metabolism--Amino sugar and nucleotide sugar metabolism|Metabolism--Global and overview maps--Metabolic pathways

[denovogenes]_105677	42.31	2e-70	deh:cbdbA122	K03605	hyaD, hybD	hydrogenase maturation protease	3.4.23.-	--

[denovogenes]_105678	93.49	9e-173	lacy:A4V08_33240	K03616	rnfB	electron transport complex protein RnfB	--	--

[denovogenes]_10568	41.17	1e-107	cpr:CPR_1353	K11060	entB	probable enterotoxin B	--	--

[denovogenes]_105680	84.56	5e-166	bxy:BXY_27970	K07133	K07133	uncharacterized protein	--	--

[denovogenes]_105682	68.32	3e-136	cle:Clole_2718	K00042	garR, glxR	2-hydroxy-3-oxopropionate reductase	1.1.1.60	Metabolism--Carbohydrate metabolism--Glyoxylate and dicarboxylate metabolism|Metabolism--Global and overview maps--Metabolic pathways

[denovogenes]_105683	50.00	2e-62	cck:Ccar_23800	K04771	degP, htrA	serine protease Do	3.4.21.107	Human Diseases--Drug resistance: Antimicrobial--Cationic antimicrobial peptide (CAMP) resistance|Environmental Information Processing--Signal transduction--Two-component system

[denovogenes]_105684	79.01	1e-148	bprs:CK3_09960	K01258	pepT	tripeptide aminopeptidase	3.4.11.4	--

[denovogenes]_105686	55.97	2e-104	lsj:LSJ_1341c	K01154	hsdS	type I restriction enzyme, S subunit	3.1.21.3	--

[denovogenes]_105687	86.21	3e-165	cle:Clole_1269	K00600	glyA, SHMT	glycine hydroxymethyltransferase	2.1.2.1	Metabolism--Amino acid metabolism--Glycine, serine and threonine metabolism|Metabolism--Metabolism of other amino acids--Cyanoamino acid metabolism|Metabolism--Carbohydrate metabolism--Glyoxylate and dicarboxylate metabolism|Metabolism--Metabolism of cofactors and vitamins--One carbon pool by folate|Metabolism--Energy metabolism--Methane metabolism|Metabolism--Global and overview maps--Metabolic pathways|Metabolism--Global and overview maps--Biosynthesis of secondary metabolites|Metabolism--Global and overview maps--Microbial metabolism in diverse environments|Metabolism--Global and overview maps--Biosynthesis of antibiotics|Metabolism--Global and overview maps--Carbon metabolism|Metabolism--Global and overview maps--Biosynthesis of amino acids|Human Diseases--Drug resistance: Antineoplastic--Antifolate resistance

[denovogenes]_105688	42.59	3e-60	rho:RHOM_09200	K08384	spoVD	stage V sporulation protein D (sporulation-specific penicillin-binding protein)	--	--

[denovogenes]_105689	41.63	5e-60	cpr:CPR_0339	K02337	DPO3A1, dnaE	DNA polymerase III subunit alpha	2.7.7.7	Metabolism--Nucleotide metabolism--Purine metabolism|Metabolism--Nucleotide metabolism--Pyrimidine metabolism|Metabolism--Global and overview maps--Metabolic pathways|Genetic Information Processing--Replication and repair--DNA replication|Genetic Information Processing--Replication and repair--Mismatch repair|Genetic Information Processing--Replication and repair--Homologous recombination

[denovogenes]_10569	99.71	0.0	bfr:BF1736	K12373	HEXA_B	hexosaminidase	3.2.1.52	Metabolism--Glycan biosynthesis and metabolism--Other glycan degradation|Metabolism--Glycan biosynthesis and metabolism--Various types of N-glycan biosynthesis|Metabolism--Carbohydrate metabolism--Amino sugar and nucleotide sugar metabolism|Metabolism--Glycan biosynthesis and metabolism--Glycosaminoglycan degradation|Metabolism--Glycan biosynthesis and metabolism--Glycosphingolipid biosynthesis - globo and isoglobo series|Metabolism--Glycan biosynthesis and metabolism--Glycosphingolipid biosynthesis - ganglio series|Metabolism--Global and overview maps--Metabolic pathways|Cellular Processes--Transport and catabolism--Lysosome

[denovogenes]_105690	97.34	1e-172	cle:Clole_2327	K03531	ftsZ	cell division protein FtsZ	--	Cellular Processes--Cell growth and death--Cell cycle - Caulobacter

[denovogenes]_105691	76.25	3e-146	lacy:A4V08_34710	K03742	pncC	nicotinamide-nucleotide amidase	3.5.1.42	Metabolism--Metabolism of cofactors and vitamins--Nicotinate and nicotinamide metabolism

[denovogenes]_105692	60.55	3e-114	cck:Ccar_18950	K01586	lysA	diaminopimelate decarboxylase	4.1.1.20	Metabolism--Amino acid metabolism--Lysine biosynthesis|Metabolism--Global and overview maps--Metabolic pathways|Metabolism--Global and overview maps--Biosynthesis of secondary metabolites|Metabolism--Global and overview maps--Microbial metabolism in diverse environments|Metabolism--Global and overview maps--Biosynthesis of antibiotics|Metabolism--Global and overview maps--Biosynthesis of amino acids

[denovogenes]_105693	45.21	7e-69	lpl:lp_3473	K05989	ramA	alpha-L-rhamnosidase	3.2.1.40	--

[denovogenes]_105698	100.00	0.0	pdi:BDI_1170	K17723	preA	dihydropyrimidine dehydrogenase (NAD+) subunit PreA	1.3.1.1	Metabolism--Nucleotide metabolism--Pyrimidine metabolism|Metabolism--Metabolism of other amino acids--beta-Alanine metabolism|Metabolism--Metabolism of cofactors and vitamins--Pantothenate and CoA biosynthesis|Metabolism--Global and overview maps--Metabolic pathways

[denovogenes]_10570	87.15	0.0	eft:M395_07230	K02622	parE	topoisomerase IV subunit B	5.99.1.-	--

[denovogenes]_105701	44.92	1e-43	ssil:SOLI23_06110	K02003	ABC.CD.A	putative ABC transport system ATP-binding protein	--	--

[denovogenes]_105704	94.30	0.0	cfd:CFNIH1_12180	K00946	thiL	thiamine-monophosphate kinase	2.7.4.16	Metabolism--Metabolism of cofactors and vitamins--Thiamine metabolism|Metabolism--Global and overview maps--Metabolic pathways

[denovogenes]_105705	88.97	5e-172	cbv:U729_1916	K02117	ATPVA, ntpA, atpA	V/A-type H+/Na+-transporting ATPase subunit A	3.6.3.14 3.6.3.15	Metabolism--Energy metabolism--Oxidative phosphorylation|Metabolism--Global and overview maps--Metabolic pathways

[denovogenes]_105707	89.69	6e-172	pfre:RM25_1255	K09817	znuC	zinc transport system ATP-binding protein	3.6.3.-	Environmental Information Processing--Membrane transport--ABC transporters

[denovogenes]_105708	40.94	1e-49	chx:102170657	K13703	ABHD11	abhydrolase domain-containing protein 11	--	--

[denovogenes]_105711	50.20	4e-68	pfre:RM25_1498	K02654	pilD, pppA	leader peptidase (prepilin peptidase) / N-methyltransferase	3.4.23.43 2.1.1.-	--

[denovogenes]_105712	96.95	0.0	pfr:PFREUD_15190	K06958	rapZ	RNase adapter protein RapZ	--	--

[denovogenes]_105713	90.84	2e-176	pfre:RM25_1431	K01803	TPI, tpiA	triosephosphate isomerase (TIM)	5.3.1.1	Metabolism--Carbohydrate metabolism--Glycolysis / Gluconeogenesis|Metabolism--Carbohydrate metabolism--Fructose and mannose metabolism|Metabolism--Carbohydrate metabolism--Inositol phosphate metabolism|Metabolism--Energy metabolism--Carbon fixation in photosynthetic organisms|Metabolism--Global and overview maps--Metabolic pathways|Metabolism--Global and overview maps--Biosynthesis of secondary metabolites|Metabolism--Global and overview maps--Microbial metabolism in diverse environments|Metabolism--Global and overview maps--Biosynthesis of antibiotics|Metabolism--Global and overview maps--Carbon metabolism|Metabolism--Global and overview maps--Biosynthesis of amino acids

[denovogenes]_105715	90.38	9e-165	bcel:BcellWH2_05262	K11070	potC	spermidine/putrescine transport system permease protein	--	Environmental Information Processing--Membrane transport--ABC transporters

[denovogenes]_105716	99.24	0.0	btho:Btheta7330_01524	K03426	E3.6.1.22, NUDT12, nudC	NAD+ diphosphatase	3.6.1.22	Metabolism--Metabolism of cofactors and vitamins--Nicotinate and nicotinamide metabolism|Metabolism--Global and overview maps--Metabolic pathways|Cellular Processes--Transport and catabolism--Peroxisome

[denovogenes]_105717	67.31	2e-127	dae:Dtox_1687	K01151	nfo	deoxyribonuclease IV	3.1.21.2	Genetic Information Processing--Replication and repair--Base excision repair

[denovogenes]_105718	100.00	0.0	kin:AB182_02485	K12067	traE	conjugal transfer pilus assembly protein TraE	--	--

[denovogenes]_10572	100.00	0.0	bfg:BF638R_4068	K07037	K07037	uncharacterized protein	--	--

[denovogenes]_105721	100.00	0.0	paeo:M801_5615	K03496	parA, soj	chromosome partitioning protein	--	--

[denovogenes]_105724	100.00	5e-177	paeo:M801_5553	K09816	znuB	zinc transport system permease protein	--	Environmental Information Processing--Membrane transport--ABC transporters

[denovogenes]_105725	100.00	0.0	ag:AAQ76277	K18973	blaOXA-50	beta-lactamase class D OXA-50	3.5.2.6	Human Diseases--Drug resistance: Antimicrobial--beta-Lactam resistance

[denovogenes]_105726	91.57	9e-179	pdi:BDI_2106	K00677	lpxA	UDP-N-acetylglucosamine acyltransferase	2.3.1.129	Metabolism--Glycan biosynthesis and metabolism--Lipopolysaccharide biosynthesis|Metabolism--Global and overview maps--Metabolic pathways|Human Diseases--Drug resistance: Antimicrobial--Cationic antimicrobial peptide (CAMP) resistance

[denovogenes]_105727	41.49	5e-53	hco:LOKO_01076	K12984	waaE, kdtX	(heptosyl)LPS beta-1,4-glucosyltransferase	2.4.1.-	--

[denovogenes]_105728	97.71	0.0	sgt:SGGB_2181	K15051	K15051, endA	DNA-entry nuclease	--	--

[denovogenes]_10573	99.85	0.0	bfg:BF638R_0944	K01284	dcp	peptidyl-dipeptidase Dcp	3.4.15.5	--

[denovogenes]_105730	54.13	1e-32	jde:Jden_2133	K07741	antB	anti-repressor protein	--	--

[denovogenes]_105731	100.00	0.0	cfd:CFNIH1_18785	K00208	fabI	enoyl-[acyl-carrier protein] reductase I	1.3.1.9 1.3.1.10	Metabolism--Lipid metabolism--Fatty acid biosynthesis|Metabolism--Metabolism of cofactors and vitamins--Biotin metabolism|Metabolism--Global and overview maps--Metabolic pathways|Metabolism--Global and overview maps--Fatty acid metabolism

[denovogenes]_105734	81.03	6e-151	lir:LAW_00844	K03496	parA, soj	chromosome partitioning protein	--	--

[denovogenes]_105735	100.00	0.0	fpla:A4U99_13590	K03337	iolB	5-deoxy-glucuronate isomerase	5.3.1.30	Metabolism--Carbohydrate metabolism--Inositol phosphate metabolism|Metabolism--Global and overview maps--Metabolic pathways|Metabolism--Global and overview maps--Microbial metabolism in diverse environments

[denovogenes]_105736	100.00	0.0	fpla:A4U99_16715	K02499	yabN	tetrapyrrole methylase family protein / MazG family protein	--	--

[denovogenes]_105738	41.30	3e-12	lrz:BJI69_03875	K02169	bioC	malonyl-CoA O-methyltransferase	2.1.1.197	Metabolism--Metabolism of cofactors and vitamins--Biotin metabolism|Metabolism--Global and overview maps--Metabolic pathways

[denovogenes]_105739	99.62	0.0	cfd:CFNIH1_09535	K13255	fhuF	ferric iron reductase protein FhuF	--	--

[denovogenes]_10574	71.05	0.0	cdg:CDBI1_12430	K03655	recG	ATP-dependent DNA helicase RecG	3.6.4.12	Genetic Information Processing--Replication and repair--Homologous recombination

[denovogenes]_105740	100.00	0.0	paeo:M801_332	K11070	potC	spermidine/putrescine transport system permease protein	--	Environmental Information Processing--Membrane transport--ABC transporters

[denovogenes]_105741	58.57	2e-97	dpg:DESPIGER_1292	K07053	E3.1.3.97	3',5'-nucleoside bisphosphate phosphatase	3.1.3.97	--

[denovogenes]_105746	40.23	2e-45	kcr:Kcr_0822	K00059	fabG	3-oxoacyl-[acyl-carrier protein] reductase	1.1.1.100	Metabolism--Lipid metabolism--Fatty acid biosynthesis|Metabolism--Metabolism of cofactors and vitamins--Biotin metabolism|Metabolism--Lipid metabolism--Biosynthesis of unsaturated fatty acids|Metabolism--Global and overview maps--Metabolic pathways|Metabolism--Global and overview maps--Fatty acid metabolism

[denovogenes]_105748	54.88	6e-88	fpr:FP2_22620	K06173	truA, PUS1	tRNA pseudouridine38-40 synthase	5.4.99.12	--

[denovogenes]_10575	99.85	0.0	efq:DR75_618	K02622	parE	topoisomerase IV subunit B	5.99.1.-	--

[denovogenes]_105751	94.30	1e-163	bfb:VU15_21395	K03321	TC.SULP	sulfate permease, SulP family	--	--

[denovogenes]_105753	99.62	0.0	cfd:CFNIH1_18000	K00675	nhoA	N-hydroxyarylamine O-acetyltransferase	2.3.1.118	--

[denovogenes]_105759	91.41	5e-171	lacy:A4V08_34850	K06183	rsuA	16S rRNA pseudouridine516 synthase	5.4.99.19	--

[denovogenes]_105763	50.00	6e-74	smw:SMWW4_v1c06200	K12519	papD	chaperone protein PapD	--	--

[denovogenes]_105764	100.00	0.0	paec:M802_1735	K03228	yscT, sctT, hrcT	type III secretion protein T	--	Environmental Information Processing--Membrane transport--Bacterial secretion system

[denovogenes]_105765	100.00	0.0	fpla:A4U99_06735	K02034	ABC.PE.P1	peptide/nickel transport system permease protein	--	Cellular Processes--Cellular community - prokaryotes--Quorum sensing

[denovogenes]_105768	71.19	4e-112	dfi:AXF13_15090	K02020	modA	molybdate transport system substrate-binding protein	--	Environmental Information Processing--Membrane transport--ABC transporters

[denovogenes]_105769	53.88	4e-97	cle:Clole_3995	K00984	aadA	streptomycin 3"-adenylyltransferase	2.7.7.47	--

[denovogenes]_10577	93.28	0.0	bhl:Bache_3272	K00937	ppk	polyphosphate kinase	2.7.4.1	Metabolism--Energy metabolism--Oxidative phosphorylation|Genetic Information Processing--Folding, sorting and degradation--RNA degradation

[denovogenes]_105771	98.09	0.0	cfd:CFNIH1_13730	K02303	cobA	uroporphyrin-III C-methyltransferase	2.1.1.107	Metabolism--Metabolism of cofactors and vitamins--Porphyrin and chlorophyll metabolism|Metabolism--Global and overview maps--Metabolic pathways|Metabolism--Global and overview maps--Biosynthesis of secondary metabolites

[denovogenes]_105772	45.05	1e-48	pen:PSEEN2869	K07782	sdiA	LuxR family transcriptional regulator, quorum-sensing system regulator SdiA	--	Environmental Information Processing--Signal transduction--Two-component system|Cellular Processes--Cellular community - prokaryotes--Quorum sensing|Cellular Processes--Cellular community - prokaryotes--Biofilm formation - Escherichia coli

[denovogenes]_105773	99.24	0.0	cfd:CFNIH1_23605	K07223	K07223	putative iron-dependent peroxidase	--	--

[denovogenes]_105774	98.47	0.0	cfd:CFNIH1_14270	K00984	aadA	streptomycin 3"-adenylyltransferase	2.7.7.47	--

[denovogenes]_105775	98.85	0.0	fpla:A4U99_09275	K03525	coaX	type III pantothenate kinase	2.7.1.33	Metabolism--Metabolism of cofactors and vitamins--Pantothenate and CoA biosynthesis|Metabolism--Global and overview maps--Metabolic pathways

[denovogenes]_105776	100.00	0.0	paeo:M801_3431	K15554	ssuC	sulfonate transport system permease protein	--	Metabolism--Energy metabolism--Sulfur metabolism|Environmental Information Processing--Membrane transport--ABC transporters

[denovogenes]_105777	88.93	3e-174	bfg:BF638R_4225	K07052	K07052	uncharacterized protein	--	--

[denovogenes]_10578	66.76	0.0	rto:RTO_20470	K03655	recG	ATP-dependent DNA helicase RecG	3.6.4.12	Genetic Information Processing--Replication and repair--Homologous recombination

[denovogenes]_105780	54.85	3e-91	bfi:CIY_15190	K01915	glnA, GLUL	glutamine synthetase	6.3.1.2	Metabolism--Amino acid metabolism--Arginine biosynthesis|Metabolism--Amino acid metabolism--Alanine, aspartate and glutamate metabolism|Metabolism--Carbohydrate metabolism--Glyoxylate and dicarboxylate metabolism|Metabolism--Energy metabolism--Nitrogen metabolism|Metabolism--Global and overview maps--Metabolic pathways|Metabolism--Global and overview maps--Microbial metabolism in diverse environments|Metabolism--Global and overview maps--Biosynthesis of amino acids|Environmental Information Processing--Signal transduction--Two-component system|Organismal Systems--Nervous system--Glutamatergic synapse|Organismal Systems--Nervous system--GABAergic synapse

[denovogenes]_105789	55.88	2e-95	lacy:A4V08_29480	K03465	thyX, thy1	thymidylate synthase (FAD)	2.1.1.148	Metabolism--Nucleotide metabolism--Pyrimidine metabolism|Metabolism--Metabolism of cofactors and vitamins--One carbon pool by folate|Metabolism--Global and overview maps--Metabolic pathways

[denovogenes]_105792	42.55	2e-50	fpr:FP2_31180	K01104	E3.1.3.48	protein-tyrosine phosphatase	3.1.3.48	--

[denovogenes]_105795	58.40	1e-106	csq:CSCA_2485	K03521	fixA, etfB	electron transfer flavoprotein beta subunit	--	--

[denovogenes]_105798	52.67	1e-97	csh:Closa_3577	K03496	parA, soj	chromosome partitioning protein	--	--

[denovogenes]_105799	98.09	0.0	sgt:SGGB_1831	K02073	metQ	D-methionine transport system substrate-binding protein	--	Environmental Information Processing--Membrane transport--ABC transporters

[denovogenes]_10580	79.51	0.0	byl:A4V09_00285	K01547	kdpB	K+-transporting ATPase ATPase B chain	3.6.3.12	Environmental Information Processing--Signal transduction--Two-component system

[denovogenes]_105800	48.45	3e-85	pdf:CD630DERM_13910	K06978	K06978	uncharacterized protein	--	--

[denovogenes]_105801	81.23	3e-153	pdi:BDI_1071	K07085	K07085	putative transport protein	--	--

[denovogenes]_105802	99.62	0.0	pdi:BDI_1646	K11070	potC	spermidine/putrescine transport system permease protein	--	Environmental Information Processing--Membrane transport--ABC transporters

[denovogenes]_105803	49.60	1e-78	rum:CK1_23800	K02315	dnaC	DNA replication protein DnaC	--	--

[denovogenes]_105804	100.00	0.0	efn:DENG_01053	K07455	recT	recombination protein RecT	--	--

[denovogenes]_105805	73.49	9e-136	bhu:bhn_I2632	K02041	phnC	phosphonate transport system ATP-binding protein	3.6.3.28	Environmental Information Processing--Membrane transport--ABC transporters

[denovogenes]_105806	97.33	0.0	bfg:BF638R_4535	K03517	nadA	quinolinate synthase	2.5.1.72	Metabolism--Metabolism of cofactors and vitamins--Nicotinate and nicotinamide metabolism|Metabolism--Global and overview maps--Metabolic pathways

[denovogenes]_105807	74.52	2e-128	cso:CLS_30580	K01752	E4.3.1.17, sdaA, sdaB, tdcG	L-serine dehydratase	4.3.1.17	Metabolism--Amino acid metabolism--Glycine, serine and threonine metabolism|Metabolism--Amino acid metabolism--Cysteine and methionine metabolism|Metabolism--Global and overview maps--Metabolic pathways|Metabolism--Global and overview maps--Biosynthesis of secondary metabolites|Metabolism--Global and overview maps--Biosynthesis of antibiotics|Metabolism--Global and overview maps--Carbon metabolism|Metabolism--Global and overview maps--Biosynthesis of amino acids

[denovogenes]_105808	53.39	3e-87	cgo:Corgl_0764	K03589	ftsQ	cell division protein FtsQ	--	Cellular Processes--Cell growth and death--Cell cycle - Caulobacter

[denovogenes]_105810	49.15	1e-62	ccu:Ccur_00510	K07308	dmsC	anaerobic dimethyl sulfoxide reductase subunit C (DMSO reductase anchor subunit)	--	Metabolism--Energy metabolism--Sulfur metabolism

[denovogenes]_105811	81.68	2e-164	bxy:BXY_25590	K06989	nadX	aspartate dehydrogenase	1.4.1.21	Metabolism--Metabolism of cofactors and vitamins--Nicotinate and nicotinamide metabolism|Metabolism--Global and overview maps--Metabolic pathways

[denovogenes]_105812	48.80	3e-86	sbu:SpiBuddy_0344	K02510	hpaI, hpcH	4-hydroxy-2-oxoheptanedioate aldolase	4.1.2.52	Metabolism--Amino acid metabolism--Tyrosine metabolism|Metabolism--Global and overview maps--Microbial metabolism in diverse environments

[denovogenes]_105813	55.09	4e-96	dsy:DSY3426	K06400	spoIVCA	site-specific DNA recombinase	--	--

[denovogenes]_105814	69.20	3e-135	dor:Desor_5129	K00973	E2.7.7.24, rfbA, rffH	glucose-1-phosphate thymidylyltransferase	2.7.7.24	Metabolism--Biosynthesis of other secondary metabolites--Streptomycin biosynthesis|Metabolism--Metabolism of terpenoids and polyketides--Polyketide sugar unit biosynthesis|Metabolism--Biosynthesis of other secondary metabolites--Acarbose and validamycin biosynthesis|Metabolism--Global and overview maps--Biosynthesis of antibiotics

[denovogenes]_105815	95.42	0.0	lacy:A4V08_09235	K09797	K09797	uncharacterized protein	--	--

[denovogenes]_105816	91.22	0.0	lacy:A4V08_02870	K14977	ylbA, UGHY	(S)-ureidoglycine aminohydrolase	3.5.3.26	Metabolism--Nucleotide metabolism--Purine metabolism|Metabolism--Global and overview maps--Microbial metabolism in diverse environments

[denovogenes]_105818	56.27	8e-94	daf:Desaf_3019	K06178	rluB	23S rRNA pseudouridine2605 synthase	5.4.99.22	--

[denovogenes]_10582	99.56	0.0	bdo:EL88_08105	K03657	uvrD, pcrA	DNA helicase II / ATP-dependent DNA helicase PcrA	3.6.4.12	Genetic Information Processing--Replication and repair--Nucleotide excision repair|Genetic Information Processing--Replication and repair--Mismatch repair

[denovogenes]_105821	49.01	4e-67	lacy:A4V08_20645	K07090	K07090	uncharacterized protein	--	--

[denovogenes]_105824	50.86	4e-28	fpla:A4U99_10000	K18349	vanRC, vanRE, vanRG	two-component system, OmpR family, response regulator VanR	--	Human Diseases--Drug resistance: Antimicrobial--Vancomycin resistance|Environmental Information Processing--Signal transduction--Two-component system

[denovogenes]_105828	62.04	5e-116	pdt:Prede_2711	K19286	nfrA2	FMN reductase [NAD(P)H]	1.5.1.39	Metabolism--Metabolism of cofactors and vitamins--Riboflavin metabolism|Metabolism--Global and overview maps--Metabolic pathways

[denovogenes]_105832	100.00	0.0	paeo:M801_1502	K03496	parA, soj	chromosome partitioning protein	--	--

[denovogenes]_105833	46.88	3e-73	bfs:BF9343_3562	K02477	K02477	two-component system, LytT family, response regulator	--	--

[denovogenes]_105835	51.37	1e-77	mit:OCO_09380	K03657	uvrD, pcrA	DNA helicase II / ATP-dependent DNA helicase PcrA	3.6.4.12	Genetic Information Processing--Replication and repair--Nucleotide excision repair|Genetic Information Processing--Replication and repair--Mismatch repair

[denovogenes]_105836	91.22	4e-176	pdi:BDI_0801	K01714	dapA	4-hydroxy-tetrahydrodipicolinate synthase	4.3.3.7	Metabolism--Biosynthesis of other secondary metabolites--Monobactam biosynthesis|Metabolism--Amino acid metabolism--Lysine biosynthesis|Metabolism--Global and overview maps--Metabolic pathways|Metabolism--Global and overview maps--Biosynthesis of secondary metabolites|Metabolism--Global and overview maps--Microbial metabolism in diverse environments|Metabolism--Global and overview maps--Biosynthesis of antibiotics|Metabolism--Global and overview maps--Biosynthesis of amino acids

[denovogenes]_105838	99.24	0.0	bbre:B12L_0544	K02069	ABC.X2.P	putative ABC transport system permease protein	--	--

[denovogenes]_105839	43.19	1e-65	opo:DSM2777_11670	K01250	rihA	pyrimidine-specific ribonucleoside hydrolase	3.2.-.-	--

[denovogenes]_105840	97.70	2e-167	lacy:A4V08_27335	K02050	ABC.SN.P	NitT/TauT family transport system permease protein	--	--

[denovogenes]_105842	80.15	2e-150	bfb:VU15_16020	K03310	TC.AGCS	alanine or glycine:cation symporter, AGCS family	--	--

[denovogenes]_105844	96.58	0.0	bcel:BcellWH2_01306	K01572	oadB	oxaloacetate decarboxylase, beta subunit	4.1.1.3	Metabolism--Carbohydrate metabolism--Pyruvate metabolism|Metabolism--Global and overview maps--Metabolic pathways

[denovogenes]_105849	61.28	8e-106	senb:BN855_11920	K10906	recE	exodeoxyribonuclease VIII	3.1.11.-	--

[denovogenes]_10585	93.70	0.0	bhl:Bache_2133	K03702	uvrB	excinuclease ABC subunit B	--	Genetic Information Processing--Replication and repair--Nucleotide excision repair

[denovogenes]_105850	91.98	2e-180	lcx:LCA12A_0277	K00016	LDH, ldh	L-lactate dehydrogenase	1.1.1.27	Metabolism--Carbohydrate metabolism--Glycolysis / Gluconeogenesis|Metabolism--Amino acid metabolism--Cysteine and methionine metabolism|Metabolism--Carbohydrate metabolism--Pyruvate metabolism|Metabolism--Carbohydrate metabolism--Propanoate metabolism|Metabolism--Global and overview maps--Metabolic pathways|Metabolism--Global and overview maps--Biosynthesis of secondary metabolites|Metabolism--Global and overview maps--Microbial metabolism in diverse environments|Metabolism--Global and overview maps--Biosynthesis of antibiotics|Organismal Systems--Endocrine system--Glucagon signaling pathway

[denovogenes]_105853	71.09	1e-136	cct:CC1_29100	K02013	ABC.FEV.A	iron complex transport system ATP-binding protein	3.6.3.34	Environmental Information Processing--Membrane transport--ABC transporters

[denovogenes]_105856	72.76	1e-133	byl:A4V09_08020	K00384	trxB	thioredoxin reductase (NADPH)	1.8.1.9	Metabolism--Nucleotide metabolism--Pyrimidine metabolism|Metabolism--Metabolism of other amino acids--Selenocompound metabolism

[denovogenes]_105858	49.62	1e-90	amt:Amet_0510	K00823	puuE	4-aminobutyrate aminotransferase	2.6.1.19	Metabolism--Amino acid metabolism--Alanine, aspartate and glutamate metabolism|Metabolism--Metabolism of other amino acids--beta-Alanine metabolism|Metabolism--Carbohydrate metabolism--Propanoate metabolism|Metabolism--Carbohydrate metabolism--Butanoate metabolism|Metabolism--Global and overview maps--Metabolic pathways|Metabolism--Global and overview maps--Microbial metabolism in diverse environments

[denovogenes]_10586	100.00	0.0	bvu:BVU_1474	K07037	K07037	uncharacterized protein	--	--

[denovogenes]_105860	99.62	0.0	cfd:CFNIH1_19955	K19333	kdgR	IclR family transcriptional regulator, KDG regulon repressor	--	--

[denovogenes]_105861	98.09	0.0	bcel:BcellWH2_04443	K02428	rdgB	XTP/dITP diphosphohydrolase	3.6.1.66	Metabolism--Nucleotide metabolism--Purine metabolism

[denovogenes]_105864	64.12	2e-125	byl:A4V09_12000	K02026	ABC.MS.P1	multiple sugar transport system permease protein	--	--

[denovogenes]_105866	48.08	4e-82	ccel:CCDG5_1744	K01992	ABC-2.P	ABC-2 type transport system permease protein	--	--

[denovogenes]_105867	97.89	2e-145	sdz:Asd1617_01219	K07647	torS	two-component system, OmpR family, sensor histidine kinase TorS	2.7.13.3	Environmental Information Processing--Signal transduction--Two-component system

[denovogenes]_105868	44.90	8e-79	cdl:CDR20291_2270	K02688	prpR	transcriptional regulator, propionate catabolism operon regulatory protein	--	--

[denovogenes]_105869	57.43	1e-98	cth:Cthe_1516	K06949	rsgA, engC	ribosome biogenesis GTPase / thiamine phosphate phosphatase	3.6.1.- 3.1.3.100	Metabolism--Metabolism of cofactors and vitamins--Thiamine metabolism|Metabolism--Global and overview maps--Metabolic pathways

[denovogenes]_105871	67.45	1e-134	ova:OBV_39060	K02510	hpaI, hpcH	4-hydroxy-2-oxoheptanedioate aldolase	4.1.2.52	Metabolism--Amino acid metabolism--Tyrosine metabolism|Metabolism--Global and overview maps--Microbial metabolism in diverse environments

[denovogenes]_105872	77.57	3e-153	cgo:Corgl_1378	K01883	CARS, cysS	cysteinyl-tRNA synthetase	6.1.1.16	Genetic Information Processing--Translation--Aminoacyl-tRNA biosynthesis

[denovogenes]_105874	99.62	0.0	eld:i02_4704	K03760	eptA	lipid A ethanolamine phosphotransferase	2.7.8.43	Human Diseases--Drug resistance: Antimicrobial--Cationic antimicrobial peptide (CAMP) resistance

[denovogenes]_105875	54.55	2e-92	bsa:Bacsa_1635	K05970	SIAE	sialate O-acetylesterase	3.1.1.53	--

[denovogenes]_105877	99.60	0.0	pdi:BDI_3092	K00640	cysE	serine O-acetyltransferase	2.3.1.30	Metabolism--Amino acid metabolism--Cysteine and methionine metabolism|Metabolism--Energy metabolism--Sulfur metabolism|Metabolism--Global and overview maps--Metabolic pathways|Metabolism--Global and overview maps--Biosynthesis of secondary metabolites|Metabolism--Global and overview maps--Microbial metabolism in diverse environments|Metabolism--Global and overview maps--Carbon metabolism|Metabolism--Global and overview maps--Biosynthesis of amino acids|Cellular Processes--Cellular community - prokaryotes--Biofilm formation - Vibrio cholerae

[denovogenes]_10588	90.07	0.0	bdo:EL88_02215	K07037	K07037	uncharacterized protein	--	--

[denovogenes]_105881	40.48	7e-62	thj:104824946	K20547	CHIB	basic endochitinase B	3.2.1.14	Metabolism--Carbohydrate metabolism--Amino sugar and nucleotide sugar metabolism|Metabolism--Global and overview maps--Metabolic pathways|Environmental Information Processing--Signal transduction--MAPK signaling pathway - plant

[denovogenes]_105882	51.54	2e-77	bprm:CL3_29340	K01308	yqgT	g-D-glutamyl-meso-diaminopimelate peptidase	3.4.19.11	--

[denovogenes]_105886	97.33	0.0	sot:102588569	K01807	rpiA	ribose 5-phosphate isomerase A	5.3.1.6	Metabolism--Carbohydrate metabolism--Pentose phosphate pathway|Metabolism--Energy metabolism--Carbon fixation in photosynthetic organisms|Metabolism--Global and overview maps--Metabolic pathways|Metabolism--Global and overview maps--Biosynthesis of secondary metabolites|Metabolism--Global and overview maps--Microbial metabolism in diverse environments|Metabolism--Global and overview maps--Biosynthesis of antibiotics|Metabolism--Global and overview maps--Carbon metabolism|Metabolism--Global and overview maps--Biosynthesis of amino acids

[denovogenes]_105887	96.56	0.0	spen:107031663	K09872	PIP	aquaporin PIP	--	--

[denovogenes]_105888	51.35	4e-79	psty:BFS30_00475	K01206	FUCA	alpha-L-fucosidase	3.2.1.51	Metabolism--Glycan biosynthesis and metabolism--Other glycan degradation

[denovogenes]_105889	56.80	1e-106	toc:Toce_2083	K05540	dusB	tRNA-dihydrouridine synthase B	1.-.-.-	--

[denovogenes]_10589	85.55	0.0	lacy:A4V08_34765	K03655	recG	ATP-dependent DNA helicase RecG	3.6.4.12	Genetic Information Processing--Replication and repair--Homologous recombination

[denovogenes]_105890	50.18	3e-83	ssm:Spirs_3381	K00135	gabD	succinate-semialdehyde dehydrogenase / glutarate-semialdehyde dehydrogenase	1.2.1.16 1.2.1.79 1.2.1.20	Metabolism--Amino acid metabolism--Alanine, aspartate and glutamate metabolism|Metabolism--Amino acid metabolism--Lysine degradation|Metabolism--Amino acid metabolism--Tyrosine metabolism|Metabolism--Carbohydrate metabolism--Butanoate metabolism|Metabolism--Metabolism of cofactors and vitamins--Nicotinate and nicotinamide metabolism|Metabolism--Global and overview maps--Metabolic pathways|Metabolism--Global and overview maps--Microbial metabolism in diverse environments

[denovogenes]_105891	62.95	4e-95	ash:AL1_19220	K03733	xerC	integrase/recombinase XerC	--	--

[denovogenes]_105893	98.48	0.0	cif:AL515_01130	K07783	uhpC	MFS transporter, OPA family, sugar phosphate sensor protein UhpC	--	Environmental Information Processing--Signal transduction--Two-component system

[denovogenes]_105897	79.01	5e-152	ash:AL1_15600	K01928	murE	UDP-N-acetylmuramoyl-L-alanyl-D-glutamate--2,6-diaminopimelate ligase	6.3.2.13	Metabolism--Amino acid metabolism--Lysine biosynthesis|Metabolism--Glycan biosynthesis and metabolism--Peptidoglycan biosynthesis

[denovogenes]_105899	51.29	7e-85	fgl:EM308_03175	K01206	FUCA	alpha-L-fucosidase	3.2.1.51	Metabolism--Glycan biosynthesis and metabolism--Other glycan degradation

[denovogenes]_105900	98.86	0.0	ecoh:ECRM13516_1086	K01093	appA	4-phytase / acid phosphatase	3.1.3.26 3.1.3.2	Metabolism--Carbohydrate metabolism--Inositol phosphate metabolism|Metabolism--Xenobiotics biodegradation and metabolism--Aminobenzoate degradation|Metabolism--Metabolism of cofactors and vitamins--Riboflavin metabolism|Metabolism--Global and overview maps--Metabolic pathways|Metabolism--Global and overview maps--Microbial metabolism in diverse environments

[denovogenes]_105901	92.37	5e-177	sdz:Asd1617_03575	K18324	acrD	multidrug efflux pump	--	Environmental Information Processing--Signal transduction--Two-component system

[denovogenes]_105902	99.24	0.0	cif:AL515_18520	K01706	gudD	glucarate dehydratase	4.2.1.40	Metabolism--Carbohydrate metabolism--Ascorbate and aldarate metabolism|Metabolism--Global and overview maps--Metabolic pathways

[denovogenes]_105903	43.51	6e-54	cbm:CBF_2196	K10797	enr	2-enoate reductase	1.3.1.31	Metabolism--Amino acid metabolism--Phenylalanine metabolism|Metabolism--Global and overview maps--Microbial metabolism in diverse environments

[denovogenes]_105904	94.27	4e-168	bcel:BcellWH2_01240	K07636	phoR	two-component system, OmpR family, phosphate regulon sensor histidine kinase PhoR	2.7.13.3	Environmental Information Processing--Signal transduction--Two-component system

[denovogenes]_105905	92.40	0.0	pdi:BDI_2489	K01925	murD	UDP-N-acetylmuramoylalanine--D-glutamate ligase	6.3.2.9	Metabolism--Metabolism of other amino acids--D-Glutamine and D-glutamate metabolism|Metabolism--Glycan biosynthesis and metabolism--Peptidoglycan biosynthesis|Metabolism--Global and overview maps--Metabolic pathways

[denovogenes]_105909	50.19	6e-92	bfi:CIY_13120	K03655	recG	ATP-dependent DNA helicase RecG	3.6.4.12	Genetic Information Processing--Replication and repair--Homologous recombination

[denovogenes]_105913	49.62	5e-85	gea:GARCT_02750	K04072	adhE	acetaldehyde dehydrogenase / alcohol dehydrogenase	1.2.1.10 1.1.1.1	Metabolism--Carbohydrate metabolism--Glycolysis / Gluconeogenesis|Metabolism--Lipid metabolism--Fatty acid degradation|Metabolism--Amino acid metabolism--Tyrosine metabolism|Metabolism--Carbohydrate metabolism--Pyruvate metabolism|Metabolism--Xenobiotics biodegradation and metabolism--Chloroalkane and chloroalkene degradation|Metabolism--Xenobiotics biodegradation and metabolism--Naphthalene degradation|Metabolism--Carbohydrate metabolism--Butanoate metabolism|Metabolism--Global and overview maps--Metabolic pathways|Metabolism--Global and overview maps--Biosynthesis of secondary metabolites|Metabolism--Global and overview maps--Microbial metabolism in diverse environments|Metabolism--Global and overview maps--Biosynthesis of antibiotics|Metabolism--Global and overview maps--Degradation of aromatic compounds

[denovogenes]_105916	66.54	9e-125	rob:CK5_30460	K13038	coaBC, dfp	phosphopantothenoylcysteine decarboxylase / phosphopantothenate--cysteine ligase	4.1.1.36 6.3.2.5	Metabolism--Metabolism of cofactors and vitamins--Pantothenate and CoA biosynthesis|Metabolism--Global and overview maps--Metabolic pathways

[denovogenes]_105917	79.47	3e-155	osp:Odosp_3322	K00864	glpK, GK	glycerol kinase	2.7.1.30	Metabolism--Lipid metabolism--Glycerolipid metabolism|Metabolism--Global and overview maps--Metabolic pathways|Organismal Systems--Endocrine system--PPAR signaling pathway|Organismal Systems--Environmental adaptation--Plant-pathogen interaction

[denovogenes]_10592	72.60	0.0	myv:G155_21020	K03427	hsdM	type I restriction enzyme M protein	2.1.1.72	--

[denovogenes]_105921	95.42	0.0	bxy:BXY_25590	K06989	nadX	aspartate dehydrogenase	1.4.1.21	Metabolism--Metabolism of cofactors and vitamins--Nicotinate and nicotinamide metabolism|Metabolism--Global and overview maps--Metabolic pathways

[denovogenes]_105922	85.11	4e-162	bth:BT_3966	K02477	K02477	two-component system, LytT family, response regulator	--	--

[denovogenes]_105924	66.26	1e-118	pdt:Prede_2711	K19286	nfrA2	FMN reductase [NAD(P)H]	1.5.1.39	Metabolism--Metabolism of cofactors and vitamins--Riboflavin metabolism|Metabolism--Global and overview maps--Metabolic pathways

[denovogenes]_105926	41.34	2e-29	lrf:LAR_1077	K07741	antB	anti-repressor protein	--	--

[denovogenes]_105927	54.92	5e-99	erb:A4V01_06665	K04486	E3.1.3.15B	histidinol-phosphatase (PHP family)	3.1.3.15	Metabolism--Amino acid metabolism--Histidine metabolism|Metabolism--Global and overview maps--Metabolic pathways|Metabolism--Global and overview maps--Biosynthesis of secondary metabolites|Metabolism--Global and overview maps--Biosynthesis of amino acids

[denovogenes]_105928	100.00	0.0	bvu:BVU_2927	K01599	hemE, UROD	uroporphyrinogen decarboxylase	4.1.1.37	Metabolism--Metabolism of cofactors and vitamins--Porphyrin and chlorophyll metabolism|Metabolism--Global and overview maps--Metabolic pathways|Metabolism--Global and overview maps--Biosynthesis of secondary metabolites

[denovogenes]_105929	93.51	0.0	boa:Bovatus_04541	K01918	panC	pantoate--beta-alanine ligase	6.3.2.1	Metabolism--Metabolism of other amino acids--beta-Alanine metabolism|Metabolism--Metabolism of cofactors and vitamins--Pantothenate and CoA biosynthesis|Metabolism--Global and overview maps--Metabolic pathways|Metabolism--Global and overview maps--Biosynthesis of secondary metabolites

[denovogenes]_105931	100.00	0.0	pvl:AOB99_08845	K00208	fabI	enoyl-[acyl-carrier protein] reductase I	1.3.1.9 1.3.1.10	Metabolism--Lipid metabolism--Fatty acid biosynthesis|Metabolism--Metabolism of cofactors and vitamins--Biotin metabolism|Metabolism--Global and overview maps--Metabolic pathways|Metabolism--Global and overview maps--Fatty acid metabolism

[denovogenes]_105932	88.99	2e-143	edu:LIU_13815	K03427	hsdM	type I restriction enzyme M protein	2.1.1.72	--

[denovogenes]_105935	76.60	1e-137	vpr:Vpar_1429	K00286	proC	pyrroline-5-carboxylate reductase	1.5.1.2	Metabolism--Amino acid metabolism--Arginine and proline metabolism|Metabolism--Global and overview maps--Metabolic pathways|Metabolism--Global and overview maps--Biosynthesis of secondary metabolites|Metabolism--Global and overview maps--Biosynthesis of antibiotics|Metabolism--Global and overview maps--Biosynthesis of amino acids

[denovogenes]_105936	62.31	2e-116	eyy:EGYY_27630	K05845	opuC	osmoprotectant transport system substrate-binding protein	--	Environmental Information Processing--Membrane transport--ABC transporters

[denovogenes]_105936	62.31	2e-116	eyy:EGYY_27630	K05846	opuBD	osmoprotectant transport system permease protein	--	Environmental Information Processing--Membrane transport--ABC transporters

[denovogenes]_105937	100.00	0.0	pvl:AOB99_07695	K00873	PK, pyk	pyruvate kinase	2.7.1.40	Metabolism--Carbohydrate metabolism--Glycolysis / Gluconeogenesis|Metabolism--Nucleotide metabolism--Purine metabolism|Metabolism--Carbohydrate metabolism--Pyruvate metabolism|Metabolism--Global and overview maps--Metabolic pathways|Metabolism--Global and overview maps--Biosynthesis of secondary metabolites|Metabolism--Global and overview maps--Microbial metabolism in diverse environments|Metabolism--Global and overview maps--Biosynthesis of antibiotics|Metabolism--Global and overview maps--Carbon metabolism|Metabolism--Global and overview maps--Biosynthesis of amino acids|Organismal Systems--Endocrine system--Glucagon signaling pathway|Human Diseases--Endocrine and metabolic diseases--Type II diabetes mellitus|Human Diseases--Cancers: Overview--Viral carcinogenesis|Human Diseases--Cancers: Overview--Central carbon metabolism in cancer

[denovogenes]_105938	84.73	5e-165	vpr:Vpar_1140	K05895	cobK-cbiJ	precorrin-6A/cobalt-precorrin-6A reductase	1.3.1.54 1.3.1.106	Metabolism--Metabolism of cofactors and vitamins--Porphyrin and chlorophyll metabolism|Metabolism--Global and overview maps--Metabolic pathways

[denovogenes]_10594	99.85	0.0	paec:M802_8	K01879	glyS	glycyl-tRNA synthetase beta chain	6.1.1.14	Genetic Information Processing--Translation--Aminoacyl-tRNA biosynthesis

[denovogenes]_105941	99.61	1e-180	pvl:AOB99_09640	K02018	modB	molybdate transport system permease protein	--	Environmental Information Processing--Membrane transport--ABC transporters

[denovogenes]_105942	77.19	2e-147	fus:HMPREF0409_02329	K01845	hemL	glutamate-1-semialdehyde 2,1-aminomutase	5.4.3.8	Metabolism--Metabolism of cofactors and vitamins--Porphyrin and chlorophyll metabolism|Metabolism--Global and overview maps--Metabolic pathways|Metabolism--Global and overview maps--Biosynthesis of secondary metabolites

[denovogenes]_105944	100.00	0.0	koe:A225_R1p0440	K00984	aadA	streptomycin 3"-adenylyltransferase	2.7.7.47	--

[denovogenes]_105945	67.05	6e-131	ehr:EHR_01975	K07566	tsaC, rimN, SUA5	L-threonylcarbamoyladenylate synthase	2.7.7.87	--

[denovogenes]_105946	100.00	0.0	ecm:EcSMS35_4913	K13255	fhuF	ferric iron reductase protein FhuF	--	--

[denovogenes]_105948	80.00	3e-149	asan:AWM72_05545	K02777	PTS-Glc-EIIA, crr	PTS system, sugar-specific IIA component	2.7.1.-	Metabolism--Carbohydrate metabolism--Glycolysis / Gluconeogenesis|Metabolism--Carbohydrate metabolism--Starch and sucrose metabolism|Metabolism--Carbohydrate metabolism--Amino sugar and nucleotide sugar metabolism|Cellular Processes--Cellular community - prokaryotes--Biofilm formation - Escherichia coli|Environmental Information Processing--Membrane transport--Phosphotransferase system (PTS)|Cellular Processes--Cellular community - prokaryotes--Biofilm formation - Vibrio cholerae

[denovogenes]_105948	80.00	3e-149	asan:AWM72_05545	K20107	PTS-Mal-EIIB, malT	PTS system, maltose-specific IIB component	2.7.1.208	Metabolism--Carbohydrate metabolism--Starch and sucrose metabolism|Environmental Information Processing--Membrane transport--Phosphotransferase system (PTS)

[denovogenes]_105948	80.00	3e-149	asan:AWM72_05545	K20108	PTS-Mal-EIIC, malT	PTS system, maltose-specific IIC component	--	Metabolism--Carbohydrate metabolism--Starch and sucrose metabolism|Environmental Information Processing--Membrane transport--Phosphotransferase system (PTS)

[denovogenes]_105949	56.54	3e-104	vpr:Vpar_0018	K09824	virK	uncharacterized protein	--	--

[denovogenes]_10595	98.54	0.0	fpla:A4U99_14790	K12573	rnr, vacB	ribonuclease R	3.1.-.-	Genetic Information Processing--Folding, sorting and degradation--RNA degradation

[denovogenes]_105950	100.00	0.0	eoi:ECO111_0370	K01659	prpC	2-methylcitrate synthase	2.3.3.5	Metabolism--Carbohydrate metabolism--Propanoate metabolism

[denovogenes]_105953	99.24	0.0	pvl:AOB99_07900	K03758	arcD, lysl, lysP	arginine:ornithine antiporter / lysine permease	--	--

[denovogenes]_105955	99.24	3e-180	elp:P12B_c0142	K02015	ABC.FEV.P	iron complex transport system permease protein	--	Environmental Information Processing--Membrane transport--ABC transporters

[denovogenes]_105956	60.56	4e-93	cro:ROD_19331	K05875	tar	methyl-accepting chemotaxis protein II, aspartate sensor receptor	--	Environmental Information Processing--Signal transduction--Two-component system|Cellular Processes--Cell motility--Bacterial chemotaxis

[denovogenes]_105958	40.25	3e-62	lok:Loa_01893	K21024	bifA	c-di-GMP phosphodiesterase	3.1.4.52	Cellular Processes--Cellular community - prokaryotes--Biofilm formation - Pseudomonas aeruginosa

[denovogenes]_105959	86.69	4e-160	emu:EMQU_0631	K03424	--	--	--	--

[denovogenes]_105960	98.86	0.0	cpr:CPR_0166	K01738	cysK	cysteine synthase A	2.5.1.47	Metabolism--Amino acid metabolism--Cysteine and methionine metabolism|Metabolism--Energy metabolism--Sulfur metabolism|Metabolism--Global and overview maps--Metabolic pathways|Metabolism--Global and overview maps--Biosynthesis of secondary metabolites|Metabolism--Global and overview maps--Microbial metabolism in diverse environments|Metabolism--Global and overview maps--Biosynthesis of antibiotics|Metabolism--Global and overview maps--Carbon metabolism|Metabolism--Global and overview maps--Biosynthesis of amino acids

[denovogenes]_105962	100.00	0.0	ecw:EcE24377A_4519	K02358	tuf, TUFM	elongation factor Tu	--	--

[denovogenes]_105963	100.00	0.0	shq:A0259_21805	K01042	selA	L-seryl-tRNA(Ser) seleniumtransferase	2.9.1.1	Metabolism--Metabolism of other amino acids--Selenocompound metabolism|Genetic Information Processing--Translation--Aminoacyl-tRNA biosynthesis

[denovogenes]_105965	100.00	0.0	pmib:BB2000_0253	K01662	dxs	1-deoxy-D-xylulose-5-phosphate synthase	2.2.1.7	Metabolism--Metabolism of cofactors and vitamins--Thiamine metabolism|Metabolism--Metabolism of terpenoids and polyketides--Terpenoid backbone biosynthesis|Metabolism--Global and overview maps--Metabolic pathways|Metabolism--Global and overview maps--Biosynthesis of secondary metabolites|Metabolism--Global and overview maps--Biosynthesis of antibiotics

[denovogenes]_105967	100.00	0.0	ecm:EcSMS35_0492	K18890	mdlB, smdB	ATP-binding cassette, subfamily B, multidrug efflux pump	--	Environmental Information Processing--Membrane transport--ABC transporters

[denovogenes]_105968	84.79	3e-170	bcel:BcellWH2_02318	K01897	ACSL, fadD	long-chain acyl-CoA synthetase	6.2.1.3	Metabolism--Lipid metabolism--Fatty acid biosynthesis|Metabolism--Lipid metabolism--Fatty acid degradation|Metabolism--Global and overview maps--Metabolic pathways|Metabolism--Global and overview maps--Fatty acid metabolism|Cellular Processes--Cellular community - prokaryotes--Quorum sensing|Organismal Systems--Endocrine system--PPAR signaling pathway|Cellular Processes--Transport and catabolism--Peroxisome|Organismal Systems--Endocrine system--Adipocytokine signaling pathway

[denovogenes]_10597	99.70	0.0	pdi:BDI_1668	K00615	E2.2.1.1, tktA, tktB	transketolase	2.2.1.1	Metabolism--Carbohydrate metabolism--Pentose phosphate pathway|Metabolism--Energy metabolism--Carbon fixation in photosynthetic organisms|Metabolism--Metabolism of terpenoids and polyketides--Biosynthesis of ansamycins|Metabolism--Global and overview maps--Metabolic pathways|Metabolism--Global and overview maps--Biosynthesis of secondary metabolites|Metabolism--Global and overview maps--Microbial metabolism in diverse environments|Metabolism--Global and overview maps--Biosynthesis of antibiotics|Metabolism--Global and overview maps--Carbon metabolism|Metabolism--Global and overview maps--Biosynthesis of amino acids

[denovogenes]_105970	57.79	4e-104	suh:SAMSHR1132_01410	K00128	--	--	--	Metabolism--Carbohydrate metabolism--Glycolysis / Gluconeogenesis|Metabolism--Carbohydrate metabolism--Ascorbate and aldarate metabolism|Metabolism--Lipid metabolism--Fatty acid degradation|Metabolism--Amino acid metabolism--Valine, leucine and isoleucine degradation|Metabolism--Amino acid metabolism--Lysine degradation|Metabolism--Amino acid metabolism--Arginine and proline metabolism|Metabolism--Amino acid metabolism--Histidine metabolism|Metabolism--Amino acid metabolism--Tryptophan metabolism|Metabolism--Metabolism of other amino acids--beta-Alanine metabolism|Metabolism--Lipid metabolism--Glycerolipid metabolism|Metabolism--Carbohydrate metabolism--Pyruvate metabolism|Metabolism--Xenobiotics biodegradation and metabolism--Chloroalkane and chloroalkene degradation|Metabolism--Metabolism of terpenoids and polyketides--Limonene and pinene degradation|Metabolism--Metabolism of terpenoids and polyketides--Insect hormone biosynthesis|Metabolism--Global and overview maps--Metabolic pathways|Metabolism--Global and overview maps--Biosynthesis of secondary metabolites|Metabolism--Global and overview maps--Microbial metabolism in diverse environments|Metabolism--Global and overview maps--Biosynthesis of antibiotics

[denovogenes]_105972	100.00	4e-180	mmk:MU9_1790	K02421	fliR	flagellar biosynthetic protein FliR	--	Cellular Processes--Cell motility--Flagellar assembly

[denovogenes]_105973	99.23	1e-180	mmk:MU9_2020	K09816	znuB	zinc transport system permease protein	--	Environmental Information Processing--Membrane transport--ABC transporters

[denovogenes]_105974	44.64	4e-12	msc:BN69_0478	K18199	E4.2.1.103, inhA	cyclohexyl-isocyanide hydratase	4.2.1.103	Metabolism--Xenobiotics biodegradation and metabolism--Caprolactam degradation

[denovogenes]_105975	99.62	0.0	mmk:MU9_1413	K02019	modE	molybdate transport system regulatory protein	--	--

[denovogenes]_105979	47.40	9e-42	spf:SpyM51316	K07741	antB	anti-repressor protein	--	--

[denovogenes]_105980	100.00	0.0	bfg:BF638R_0189	K00930	argB	acetylglutamate kinase	2.7.2.8	Metabolism--Amino acid metabolism--Arginine biosynthesis|Metabolism--Global and overview maps--Metabolic pathways|Metabolism--Global and overview maps--Biosynthesis of secondary metabolites|Metabolism--Global and overview maps--Biosynthesis of antibiotics|Metabolism--Global and overview maps--2-Oxocarboxylic acid metabolism|Metabolism--Global and overview maps--Biosynthesis of amino acids

[denovogenes]_105984	100.00	0.0	bfb:VU15_04495	K02428	rdgB	XTP/dITP diphosphohydrolase	3.6.1.66	Metabolism--Nucleotide metabolism--Purine metabolism

[denovogenes]_105985	100.00	0.0	bfg:BF638R_4262	K04069	pflA, pflC, pflE	pyruvate formate lyase activating enzyme	1.97.1.4	--

[denovogenes]_105989	100.00	0.0	mmk:MU9_1016	K02303	cobA	uroporphyrin-III C-methyltransferase	2.1.1.107	Metabolism--Metabolism of cofactors and vitamins--Porphyrin and chlorophyll metabolism|Metabolism--Global and overview maps--Metabolic pathways|Metabolism--Global and overview maps--Biosynthesis of secondary metabolites

[denovogenes]_10599	99.85	0.0	eun:UMNK88_3691	K07452	mcrB	5-methylcytosine-specific restriction enzyme B	3.1.21.-	--

[denovogenes]_105994	100.00	0.0	mmk:MU9_760	K03183	ubiE	demethylmenaquinone methyltransferase / 2-methoxy-6-polyprenyl-1,4-benzoquinol methylase	2.1.1.163 2.1.1.201	Metabolism--Metabolism of cofactors and vitamins--Ubiquinone and other terpenoid-quinone biosynthesis|Metabolism--Global and overview maps--Metabolic pathways|Metabolism--Global and overview maps--Biosynthesis of secondary metabolites

[denovogenes]_105995	81.85	4e-158	pdf:CD630DERM_07170	K07321	cooC	CO dehydrogenase maturation factor	--	--

[denovogenes]_105996	83.08	2e-165	cpr:CPR_0797	K02013	ABC.FEV.A	iron complex transport system ATP-binding protein	3.6.3.34	Environmental Information Processing--Membrane transport--ABC transporters

[denovogenes]_105999	54.47	5e-100	eha:Ethha_1878	K03496	parA, soj	chromosome partitioning protein	--	--

[denovogenes]_106	47.56	0.0	bbp:BBPR_0482	K01190	lacZ	beta-galactosidase	3.2.1.23	Metabolism--Carbohydrate metabolism--Galactose metabolism|Metabolism--Glycan biosynthesis and metabolism--Other glycan degradation|Metabolism--Lipid metabolism--Sphingolipid metabolism|Metabolism--Global and overview maps--Metabolic pathways

[denovogenes]_106001	80.08	1e-144	lacy:A4V08_15935	K02564	nagB, GNPDA	glucosamine-6-phosphate deaminase	3.5.99.6	Metabolism--Carbohydrate metabolism--Amino sugar and nucleotide sugar metabolism|Metabolism--Global and overview maps--Metabolic pathways

[denovogenes]_106002	95.02	2e-180	cfd:CFNIH1_11000	K02392	flgG	flagellar basal-body rod protein FlgG	--	Cellular Processes--Cell motility--Flagellar assembly

[denovogenes]_106003	88.46	7e-169	cfd:CFNIH1_10970	K02386	flgA	flagella basal body P-ring formation protein FlgA	--	Cellular Processes--Cell motility--Flagellar assembly

[denovogenes]_106006	75.48	2e-142	pdf:CD630DERM_36730	K03497	parB, spo0J	chromosome partitioning protein, ParB family	--	--

[denovogenes]_106007	58.53	3e-103	csr:Cspa_c58390	K03217	yidC, spoIIIJ, OXA1, ccfA	YidC/Oxa1 family membrane protein insertase	--	Cellular Processes--Cellular community - prokaryotes--Quorum sensing|Genetic Information Processing--Folding, sorting and degradation--Protein export|Environmental Information Processing--Membrane transport--Bacterial secretion system

[denovogenes]_106008	67.47	5e-119	lacy:A4V08_25595	K09761	rsmE	16S rRNA (uracil1498-N3)-methyltransferase	2.1.1.193	--

[denovogenes]_106009	41.80	8e-30	mgp:100551395	K00061	RDH5	11-cis-retinol dehydrogenase	1.1.1.315	Metabolism--Metabolism of cofactors and vitamins--Retinol metabolism

[denovogenes]_106010	71.10	6e-134	cbv:U729_1449	K01243	mtnN, mtn, pfs	adenosylhomocysteine nucleosidase	3.2.2.9	Metabolism--Amino acid metabolism--Cysteine and methionine metabolism|Metabolism--Global and overview maps--Metabolic pathways|Metabolism--Global and overview maps--Biosynthesis of amino acids

[denovogenes]_106011	72.37	1e-130	csb:CLSA_c18290	K07402	xdhC	xanthine dehydrogenase accessory factor	--	--

[denovogenes]_106012	62.45	9e-113	cbut:ATN24_15265	K04486	E3.1.3.15B	histidinol-phosphatase (PHP family)	3.1.3.15	Metabolism--Amino acid metabolism--Histidine metabolism|Metabolism--Global and overview maps--Metabolic pathways|Metabolism--Global and overview maps--Biosynthesis of secondary metabolites|Metabolism--Global and overview maps--Biosynthesis of amino acids

[denovogenes]_106013	47.06	1e-27	ebf:D782_2521	K07455	recT	recombination protein RecT	--	--

[denovogenes]_106014	99.23	0.0	stb:SGPB_0371	K02030	ABC.PA.S	polar amino acid transport system substrate-binding protein	--	--

[denovogenes]_106016	93.46	0.0	ega:AL523_10460	K02967	RP-S2, MRPS2, rpsB	small subunit ribosomal protein S2	--	Genetic Information Processing--Translation--Ribosome

[denovogenes]_106017	80.08	6e-158	efn:DENG_02465	K00806	uppS	undecaprenyl diphosphate synthase	2.5.1.31	Metabolism--Metabolism of terpenoids and polyketides--Terpenoid backbone biosynthesis|Metabolism--Global and overview maps--Biosynthesis of secondary metabolites

[denovogenes]_106018	70.88	2e-135	csr:Cspa_c00670	K03424	--	--	--	--

[denovogenes]_10602	78.55	0.0	pdi:BDI_0665	K12373	HEXA_B	hexosaminidase	3.2.1.52	Metabolism--Glycan biosynthesis and metabolism--Other glycan degradation|Metabolism--Glycan biosynthesis and metabolism--Various types of N-glycan biosynthesis|Metabolism--Carbohydrate metabolism--Amino sugar and nucleotide sugar metabolism|Metabolism--Glycan biosynthesis and metabolism--Glycosaminoglycan degradation|Metabolism--Glycan biosynthesis and metabolism--Glycosphingolipid biosynthesis - globo and isoglobo series|Metabolism--Glycan biosynthesis and metabolism--Glycosphingolipid biosynthesis - ganglio series|Metabolism--Global and overview maps--Metabolic pathways|Cellular Processes--Transport and catabolism--Lysosome

[denovogenes]_106020	81.60	5e-141	seec:CFSAN002050_07420	K15540	ecpD	chaperone protein EcpD	--	--

[denovogenes]_106021	99.62	0.0	ene:ENT_27400	K14977	ylbA, UGHY	(S)-ureidoglycine aminohydrolase	3.5.3.26	Metabolism--Nucleotide metabolism--Purine metabolism|Metabolism--Global and overview maps--Microbial metabolism in diverse environments

[denovogenes]_106023	42.19	2e-21	fro:AALO17_21580	K03478	chbG	chitin disaccharide deacetylase	3.5.1.105	--

[denovogenes]_106026	99.61	0.0	efa:EF1773	K00059	fabG	3-oxoacyl-[acyl-carrier protein] reductase	1.1.1.100	Metabolism--Lipid metabolism--Fatty acid biosynthesis|Metabolism--Metabolism of cofactors and vitamins--Biotin metabolism|Metabolism--Lipid metabolism--Biosynthesis of unsaturated fatty acids|Metabolism--Global and overview maps--Metabolic pathways|Metabolism--Global and overview maps--Fatty acid metabolism

[denovogenes]_106028	59.02	5e-107	pdf:CD630DERM_17740	K02030	ABC.PA.S	polar amino acid transport system substrate-binding protein	--	--

[denovogenes]_106029	55.47	2e-102	ppq:PPSQR21_048780	K00065	kduD	2-deoxy-D-gluconate 3-dehydrogenase	1.1.1.125	Metabolism--Carbohydrate metabolism--Pentose and glucuronate interconversions

[denovogenes]_106032	79.62	2e-148	pdf:CD630DERM_12750	K03706	codY	transcriptional pleiotropic repressor	--	--

[denovogenes]_106033	98.72	3e-167	cfd:CFNIH1_17860	K01028	E2.8.3.5A, scoA	3-oxoacid CoA-transferase subunit A	2.8.3.5	Metabolism--Lipid metabolism--Synthesis and degradation of ketone bodies|Metabolism--Amino acid metabolism--Valine, leucine and isoleucine degradation|Metabolism--Carbohydrate metabolism--Butanoate metabolism

[denovogenes]_106034	61.78	2e-113	cbk:CLL_A1277	K11753	ribF	riboflavin kinase / FMN adenylyltransferase	2.7.1.26 2.7.7.2	Metabolism--Metabolism of cofactors and vitamins--Riboflavin metabolism|Metabolism--Global and overview maps--Metabolic pathways|Metabolism--Global and overview maps--Biosynthesis of secondary metabolites

[denovogenes]_106037	100.00	0.0	pvl:AOB99_01970	K03118	tatC	sec-independent protein translocase protein TatC	--	Genetic Information Processing--Folding, sorting and degradation--Protein export|Environmental Information Processing--Membrane transport--Bacterial secretion system

[denovogenes]_106038	99.62	0.0	cbut:ATN24_01830	K03424	--	--	--	--

[denovogenes]_106039	85.82	3e-161	cbv:U729_1463	K01715	crt	enoyl-CoA hydratase	4.2.1.17	Metabolism--Carbohydrate metabolism--Butanoate metabolism|Metabolism--Global and overview maps--Carbon metabolism

[denovogenes]_106040	54.58	6e-94	cbv:U729_195	K04486	E3.1.3.15B	histidinol-phosphatase (PHP family)	3.1.3.15	Metabolism--Amino acid metabolism--Histidine metabolism|Metabolism--Global and overview maps--Metabolic pathways|Metabolism--Global and overview maps--Biosynthesis of secondary metabolites|Metabolism--Global and overview maps--Biosynthesis of amino acids

[denovogenes]_106041	88.12	1e-168	ecr:ECIAI1_4247	K07074	K07074	uncharacterized protein	--	--

[denovogenes]_106043	96.55	5e-170	cfd:CFNIH1_16950	K08354	phsC	thiosulfate reductase cytochrome b subunit	--	Metabolism--Energy metabolism--Sulfur metabolism|Metabolism--Global and overview maps--Microbial metabolism in diverse environments

[denovogenes]_106044	40.10	6e-32	xla:399015	K11154	RDH16	retinol dehydrogenase 16	1.1.1.-	Metabolism--Metabolism of cofactors and vitamins--Retinol metabolism|Metabolism--Global and overview maps--Metabolic pathways

[denovogenes]_106045	100.00	0.0	pmib:BB2000_2121	K05799	pdhR	GntR family transcriptional regulator, transcriptional repressor for pyruvate dehydrogenase complex	--	--

[denovogenes]_106046	100.00	0.0	ene:ENT_16120	K02967	RP-S2, MRPS2, rpsB	small subunit ribosomal protein S2	--	Genetic Information Processing--Translation--Ribosome

[denovogenes]_106047	99.22	0.0	sgg:SGGBAA2069_c14720	K01101	E3.1.3.41	4-nitrophenyl phosphatase	3.1.3.41	Metabolism--Xenobiotics biodegradation and metabolism--Aminobenzoate degradation|Metabolism--Global and overview maps--Microbial metabolism in diverse environments

[denovogenes]_106050	99.62	0.0	eoj:ECO26_4462	K10710	frlD	fructoselysine 6-kinase	2.7.1.-	--

[denovogenes]_106051	100.00	0.0	pvl:AOB99_15130	K03426	E3.6.1.22, NUDT12, nudC	NAD+ diphosphatase	3.6.1.22	Metabolism--Metabolism of cofactors and vitamins--Nicotinate and nicotinamide metabolism|Metabolism--Global and overview maps--Metabolic pathways|Cellular Processes--Transport and catabolism--Peroxisome

[denovogenes]_106052	100.00	0.0	pvl:AOB99_10680	K03834	tyrP	tyrosine-specific transport protein	--	--

[denovogenes]_106053	64.45	2e-119	ecas:ECBG_02258	K03930	estA	putative tributyrin esterase	3.1.1.-	--

[denovogenes]_106055	49.45	8e-83	gea:GARCT_02750	K04072	adhE	acetaldehyde dehydrogenase / alcohol dehydrogenase	1.2.1.10 1.1.1.1	Metabolism--Carbohydrate metabolism--Glycolysis / Gluconeogenesis|Metabolism--Lipid metabolism--Fatty acid degradation|Metabolism--Amino acid metabolism--Tyrosine metabolism|Metabolism--Carbohydrate metabolism--Pyruvate metabolism|Metabolism--Xenobiotics biodegradation and metabolism--Chloroalkane and chloroalkene degradation|Metabolism--Xenobiotics biodegradation and metabolism--Naphthalene degradation|Metabolism--Carbohydrate metabolism--Butanoate metabolism|Metabolism--Global and overview maps--Metabolic pathways|Metabolism--Global and overview maps--Biosynthesis of secondary metabolites|Metabolism--Global and overview maps--Microbial metabolism in diverse environments|Metabolism--Global and overview maps--Biosynthesis of antibiotics|Metabolism--Global and overview maps--Degradation of aromatic compounds

[denovogenes]_106056	100.00	0.0	pvl:AOB99_02055	K07160	K07160	UPF0271 protein	--	--

[denovogenes]_106060	96.93	2e-166	cfd:CFNIH1_20965	K02421	fliR	flagellar biosynthetic protein FliR	--	Cellular Processes--Cell motility--Flagellar assembly

[denovogenes]_106061	41.53	2e-62	lpr:LBP_cg0034	K04771	degP, htrA	serine protease Do	3.4.21.107	Human Diseases--Drug resistance: Antimicrobial--Cationic antimicrobial peptide (CAMP) resistance|Environmental Information Processing--Signal transduction--Two-component system

[denovogenes]_106062	55.73	5e-89	pdf:CD630DERM_26640	K01928	murE	UDP-N-acetylmuramoyl-L-alanyl-D-glutamate--2,6-diaminopimelate ligase	6.3.2.13	Metabolism--Amino acid metabolism--Lysine biosynthesis|Metabolism--Glycan biosynthesis and metabolism--Peptidoglycan biosynthesis

[denovogenes]_106064	99.62	0.0	pvl:AOB99_04495	K00348	nqrC	Na+-transporting NADH:ubiquinone oxidoreductase subunit C	1.6.5.8	--

[denovogenes]_106065	81.30	6e-153	cpr:CPR_0364	K18888	efrB	ATP-binding cassette, subfamily B, multidrug efflux pump	--	Environmental Information Processing--Membrane transport--ABC transporters

[denovogenes]_106066	78.38	2e-144	vpr:Vpar_0119	K16786	ecfA1	energy-coupling factor transport system ATP-binding protein	3.6.3.-	Environmental Information Processing--Membrane transport--ABC transporters

[denovogenes]_106066	78.38	2e-144	vpr:Vpar_0119	K16787	ecfA2	energy-coupling factor transport system ATP-binding protein	3.6.3.-	Environmental Information Processing--Membrane transport--ABC transporters

[denovogenes]_106068	76.25	1e-138	cck:Ccar_01425	K03521	fixA, etfB	electron transfer flavoprotein beta subunit	--	--

[denovogenes]_106069	100.00	0.0	pvl:AOB99_04895	K05515	mrdA	penicillin-binding protein 2	--	Metabolism--Glycan biosynthesis and metabolism--Peptidoglycan biosynthesis|Human Diseases--Drug resistance: Antimicrobial--beta-Lactam resistance

[denovogenes]_10607	98.69	0.0	boa:Bovatus_00079	K04084	dsbD	thiol:disulfide interchange protein DsbD	1.8.1.8	--

[denovogenes]_106070	85.82	2e-160	cif:AL515_11180	K02029	ABC.PA.P	polar amino acid transport system permease protein	--	--

[denovogenes]_106071	42.98	8e-15	cac:CA_C3166	K06933	K06933	uncharacterized protein	--	--

[denovogenes]_106072	87.55	3e-167	ecy:ECSE_P2-0073	K12057	traF	conjugal transfer pilus assembly protein TraF	--	--

[denovogenes]_106074	68.08	6e-124	fnt:HMPREF0405_01137	K12942	abgT	aminobenzoyl-glutamate transport protein	--	--

[denovogenes]_106075	100.00	0.0	sdz:Asd1617_03148	K06219	smtA	S-adenosylmethionine-dependent methyltransferase	--	--

[denovogenes]_106076	40.15	5e-20	lki:LKI_04015	K03148	thiF	sulfur carrier protein ThiS adenylyltransferase	2.7.7.73	Metabolism--Metabolism of cofactors and vitamins--Thiamine metabolism|Metabolism--Global and overview maps--Metabolic pathways|Genetic Information Processing--Folding, sorting and degradation--Sulfur relay system

[denovogenes]_106077	100.00	0.0	cbut:ATN24_03150	K01715	crt	enoyl-CoA hydratase	4.2.1.17	Metabolism--Carbohydrate metabolism--Butanoate metabolism|Metabolism--Global and overview maps--Carbon metabolism

[denovogenes]_106078	73.18	4e-132	cck:Ccar_22800	K03521	fixA, etfB	electron transfer flavoprotein beta subunit	--	--

[denovogenes]_106079	100.00	0.0	pvl:AOB99_05110	K01175	ybfF	esterase	3.1.-.-	--

[denovogenes]_10608	95.55	0.0	boa:Bovatus_03936	K06158	ABCF3	ATP-binding cassette, subfamily F, member 3	--	--

[denovogenes]_106080	92.34	3e-176	vpr:Vpar_0422	K02071	metN	D-methionine transport system ATP-binding protein	--	Environmental Information Processing--Membrane transport--ABC transporters

[denovogenes]_106083	78.38	9e-148	prg:RB151_012000	K07106	murQ	N-acetylmuramic acid 6-phosphate etherase	4.2.1.126	Metabolism--Carbohydrate metabolism--Amino sugar and nucleotide sugar metabolism|Metabolism--Global and overview maps--Metabolic pathways

[denovogenes]_106084	72.31	9e-135	cle:Clole_1593	K00767	nadC, QPRT	nicotinate-nucleotide pyrophosphorylase (carboxylating)	2.4.2.19	Metabolism--Metabolism of cofactors and vitamins--Nicotinate and nicotinamide metabolism|Metabolism--Global and overview maps--Metabolic pathways

[denovogenes]_106085	40.47	2e-52	eah:FA04_27225	K10439	rbsB	ribose transport system substrate-binding protein	--	Environmental Information Processing--Membrane transport--ABC transporters|Cellular Processes--Cell motility--Bacterial chemotaxis

[denovogenes]_106086	71.98	2e-135	cbut:ATN24_01830	K03424	--	--	--	--

[denovogenes]_106087	54.02	4e-94	clt:CM240_1151	K02233	E2.7.8.26, cobS, cobV	adenosylcobinamide-GDP ribazoletransferase	2.7.8.26	Metabolism--Metabolism of cofactors and vitamins--Porphyrin and chlorophyll metabolism|Metabolism--Global and overview maps--Metabolic pathways

[denovogenes]_106088	100.00	0.0	pvl:AOB99_13705	K15777	DOPA	4,5-DOPA dioxygenase extradiol	1.13.11.-	Metabolism--Biosynthesis of other secondary metabolites--Betalain biosynthesis

[denovogenes]_106090	59.47	2e-108	pow:IJ21_39050	K17320	lplC	putative aldouronate transport system permease protein	--	Environmental Information Processing--Membrane transport--ABC transporters

[denovogenes]_106091	100.00	0.0	pvl:AOB99_07670	K09816	znuB	zinc transport system permease protein	--	Environmental Information Processing--Membrane transport--ABC transporters

[denovogenes]_106092	75.58	9e-147	cbv:U729_2841	K06941	rlmN	23S rRNA (adenine2503-C2)-methyltransferase	2.1.1.192	--

[denovogenes]_106093	50.96	8e-87	cbv:U729_2504	K02795	PTS-Man-EIIC, manY	PTS system, mannose-specific IIC component	--	Metabolism--Carbohydrate metabolism--Fructose and mannose metabolism|Metabolism--Carbohydrate metabolism--Amino sugar and nucleotide sugar metabolism|Metabolism--Global and overview maps--Metabolic pathways|Environmental Information Processing--Membrane transport--Phosphotransferase system (PTS)

[denovogenes]_106094	98.09	0.0	cif:AL515_18995	K07462	recJ	single-stranded-DNA-specific exonuclease	3.1.-.-	Genetic Information Processing--Replication and repair--Base excision repair|Genetic Information Processing--Replication and repair--Mismatch repair|Genetic Information Processing--Replication and repair--Homologous recombination

[denovogenes]_106095	70.23	9e-133	cbut:ATN24_03750	K07566	tsaC, rimN, SUA5	L-threonylcarbamoyladenylate synthase	2.7.7.87	--

[denovogenes]_106102	77.31	2e-147	cpe:CPE2176	K01443	nagA, AMDHD2	N-acetylglucosamine-6-phosphate deacetylase	3.5.1.25	Metabolism--Carbohydrate metabolism--Amino sugar and nucleotide sugar metabolism|Metabolism--Global and overview maps--Biosynthesis of antibiotics

[denovogenes]_106104	73.26	2e-138	cpy:Cphy_0816	K02032	ABC.PE.A1	peptide/nickel transport system ATP-binding protein	--	Cellular Processes--Cellular community - prokaryotes--Quorum sensing

[denovogenes]_106105	65.25	1e-119	eac:EAL2_c07680	K02168	betT, betS	choline/glycine/proline betaine transport protein	--	--

[denovogenes]_106107	42.38	1e-68	tli:Tlie_1741	K01436	K01436	amidohydrolase	3.5.1.-	--

[denovogenes]_106108	100.00	0.0	eoc:CE10_2589	K09475	ompC	outer membrane pore protein C	--	Human Diseases--Drug resistance: Antimicrobial--beta-Lactam resistance|Environmental Information Processing--Signal transduction--Two-component system

[denovogenes]_106112	77.48	6e-154	pdf:CD630DERM_23810	K00179	iorA	indolepyruvate ferredoxin oxidoreductase, alpha subunit	1.2.7.8	--

[denovogenes]_106114	54.58	6e-99	bcef:BcrFT9_04105	K12573	rnr, vacB	ribonuclease R	3.1.-.-	Genetic Information Processing--Folding, sorting and degradation--RNA degradation

[denovogenes]_106115	100.00	0.0	eci:UTI89_C4157	K15633	gpmI	2,3-bisphosphoglycerate-independent phosphoglycerate mutase	5.4.2.12	Metabolism--Carbohydrate metabolism--Glycolysis / Gluconeogenesis|Metabolism--Amino acid metabolism--Glycine, serine and threonine metabolism|Metabolism--Energy metabolism--Methane metabolism|Metabolism--Global and overview maps--Metabolic pathways|Metabolism--Global and overview maps--Biosynthesis of secondary metabolites|Metabolism--Global and overview maps--Microbial metabolism in diverse environments|Metabolism--Global and overview maps--Biosynthesis of antibiotics|Metabolism--Global and overview maps--Carbon metabolism|Metabolism--Global and overview maps--Biosynthesis of amino acids

[denovogenes]_106116	60.84	5e-109	sft:NCTC1_00638	K07646	kdpD	two-component system, OmpR family, sensor histidine kinase KdpD	2.7.13.3	Environmental Information Processing--Signal transduction--Two-component system

[denovogenes]_106117	93.89	0.0	prg:RB151_010570	K05515	mrdA	penicillin-binding protein 2	--	Metabolism--Glycan biosynthesis and metabolism--Peptidoglycan biosynthesis|Human Diseases--Drug resistance: Antimicrobial--beta-Lactam resistance

[denovogenes]_106118	95.80	7e-179	prg:RB151_003980	K03326	TC.DCUC, dcuC, dcuD	C4-dicarboxylate transporter, DcuC family	--	--

[denovogenes]_10612	99.56	0.0	mmk:MU9_1573	K03658	helD	DNA helicase IV	3.6.4.12	--

[denovogenes]_106121	99.24	0.0	cif:AL515_08980	K07478	ycaJ	putative ATPase	--	--

[denovogenes]_106122	100.00	0.0	sdz:Asd1617_00574	K00892	gsk	inosine kinase	2.7.1.73	Metabolism--Nucleotide metabolism--Purine metabolism

[denovogenes]_106125	55.98	1e-85	cbk:CLL_A1196	K07636	phoR	two-component system, OmpR family, phosphate regulon sensor histidine kinase PhoR	2.7.13.3	Environmental Information Processing--Signal transduction--Two-component system

[denovogenes]_106126	48.29	3e-77	lca:LSEI_2166	K07484	K07484	transposase	--	--

[denovogenes]_10613	99.71	0.0	bfg:BF638R_3078	K03628	rho	transcription termination factor Rho	--	Genetic Information Processing--Folding, sorting and degradation--RNA degradation

[denovogenes]_106130	56.55	3e-103	gea:GARCT_00074	K03798	ftsH, hflB	cell division protease FtsH	3.4.24.-	--

[denovogenes]_106132	51.66	1e-50	rho:RHOM_07925	K02755	PTS-Bgl-EIIA, bglF, bglP	PTS system, beta-glucoside-specific IIA component	2.7.1.-	Environmental Information Processing--Membrane transport--Phosphotransferase system (PTS)

[denovogenes]_106132	51.66	1e-50	rho:RHOM_07925	K02756	PTS-Bgl-EIIB, bglF, bglP	PTS system, beta-glucoside-specific IIB component	2.7.1.-	Environmental Information Processing--Membrane transport--Phosphotransferase system (PTS)

[denovogenes]_106132	51.66	1e-50	rho:RHOM_07925	K02757	PTS-Bgl-EIIC, bglF, bglP	PTS system, beta-glucoside-specific IIC component	--	Environmental Information Processing--Membrane transport--Phosphotransferase system (PTS)

[denovogenes]_106134	55.16	1e-100	lgy:T479_13145	K02041	phnC	phosphonate transport system ATP-binding protein	3.6.3.28	Environmental Information Processing--Membrane transport--ABC transporters

[denovogenes]_106135	66.26	1e-119	pdt:Prede_2711	K19286	nfrA2	FMN reductase [NAD(P)H]	1.5.1.39	Metabolism--Metabolism of cofactors and vitamins--Riboflavin metabolism|Metabolism--Global and overview maps--Metabolic pathways

[denovogenes]_106137	87.36	1e-168	btho:Btheta7330_02980	K01991	ABC-2.OM, wza	polysaccharide biosynthesis/export protein	--	Cellular Processes--Cellular community - prokaryotes--Biofilm formation - Escherichia coli

[denovogenes]_10614	47.84	0.0	ctx:Clo1313_2770	K02004	ABC.CD.P	putative ABC transport system permease protein	--	--

[denovogenes]_106140	54.15	2e-87	anx:ACH33_05020	K07090	K07090	uncharacterized protein	--	--

[denovogenes]_106141	71.32	3e-131	dec:DCF50_p2941	K05936	cobM, cbiF	precorrin-4/cobalt-precorrin-4 C11-methyltransferase	2.1.1.133 2.1.1.271	Metabolism--Metabolism of cofactors and vitamins--Porphyrin and chlorophyll metabolism|Metabolism--Global and overview maps--Metabolic pathways

[denovogenes]_106143	63.28	5e-116	byl:A4V09_05730	K05896	scpA	segregation and condensation protein A	--	--

[denovogenes]_106144	77.52	2e-147	rum:CK1_37600	K01695	trpA	tryptophan synthase alpha chain	4.2.1.20	Metabolism--Amino acid metabolism--Glycine, serine and threonine metabolism|Metabolism--Amino acid metabolism--Phenylalanine, tyrosine and tryptophan biosynthesis|Metabolism--Global and overview maps--Metabolic pathways|Metabolism--Global and overview maps--Biosynthesis of secondary metabolites|Metabolism--Global and overview maps--Biosynthesis of antibiotics|Metabolism--Global and overview maps--Biosynthesis of amino acids

[denovogenes]_106146	73.73	4e-143	rto:RTO_09360	K03496	parA, soj	chromosome partitioning protein	--	--

[denovogenes]_106147	55.60	7e-89	csh:Closa_4162	K07238	TC.ZIP, zupT, ZRT3, ZIP2	zinc transporter, ZIP family	--	--

[denovogenes]_106148	64.90	9e-96	fpla:A4U99_16515	K02050	ABC.SN.P	NitT/TauT family transport system permease protein	--	--

[denovogenes]_106149	86.15	3e-135	fpla:A4U99_13480	K02108	ATPF0A, atpB	F-type H+-transporting ATPase subunit a	--	Metabolism--Energy metabolism--Oxidative phosphorylation|Metabolism--Energy metabolism--Photosynthesis|Metabolism--Global and overview maps--Metabolic pathways

[denovogenes]_10615	100.00	0.0	ene:ENT_09650	K03316	TC.CPA1	monovalent cation:H+ antiporter, CPA1 family	--	--

[denovogenes]_106150	66.27	3e-105	ibu:IB211_02442c	K06410	spoVFA	dipicolinate synthase subunit A	--	--

[denovogenes]_106156	84.50	3e-160	cso:CLS_36800	K01715	crt	enoyl-CoA hydratase	4.2.1.17	Metabolism--Carbohydrate metabolism--Butanoate metabolism|Metabolism--Global and overview maps--Carbon metabolism

[denovogenes]_106158	100.00	0.0	bvu:BVU_3990	K07001	K07001	NTE family protein	--	--

[denovogenes]_10616	99.85	0.0	ecg:E2348C_4612	K07452	mcrB	5-methylcytosine-specific restriction enzyme B	3.1.21.-	--

[denovogenes]_106162	74.59	3e-135	lacy:A4V08_05260	K12410	npdA	NAD-dependent deacetylase	3.5.1.-	--

[denovogenes]_106164	100.00	0.0	bvu:BVU_2782	K02169	bioC	malonyl-CoA O-methyltransferase	2.1.1.197	Metabolism--Metabolism of cofactors and vitamins--Biotin metabolism|Metabolism--Global and overview maps--Metabolic pathways

[denovogenes]_106167	48.33	4e-69	ash:AL1_15900	K12308	bgaB, lacA	beta-galactosidase	3.2.1.23	Metabolism--Carbohydrate metabolism--Galactose metabolism

[denovogenes]_106168	98.47	0.0	btho:Btheta7330_02546	K03924	moxR	MoxR-like ATPase	3.6.3.-	--

[denovogenes]_10617	66.72	0.0	elm:ELI_4379	K01534	zntA	Cd2+/Zn2+-exporting ATPase	3.6.3.3 3.6.3.5	--

[denovogenes]_106170	98.85	0.0	btho:Btheta7330_00179	K03734	apbE	FAD:protein FMN transferase	2.7.1.180	--

[denovogenes]_106171	49.62	1e-61	bpj:B2904_orf326	K07090	K07090	uncharacterized protein	--	--

[denovogenes]_106173	100.00	0.0	bth:BT_3587	K02564	nagB, GNPDA	glucosamine-6-phosphate deaminase	3.5.99.6	Metabolism--Carbohydrate metabolism--Amino sugar and nucleotide sugar metabolism|Metabolism--Global and overview maps--Metabolic pathways

[denovogenes]_106174	42.41	4e-69	sli:Slin_2091	K02477	K02477	two-component system, LytT family, response regulator	--	--

[denovogenes]_106175	81.23	1e-157	bprm:CL3_17070	K01151	nfo	deoxyribonuclease IV	3.1.21.2	Genetic Information Processing--Replication and repair--Base excision repair

[denovogenes]_106179	50.81	7e-79	cbut:ATN24_01005	K07090	K07090	uncharacterized protein	--	--

[denovogenes]_10618	99.71	0.0	bth:BT_2094	K02014	TC.FEV.OM	iron complex outermembrane recepter protein	--	--

[denovogenes]_106180	71.54	9e-131	lacy:A4V08_02635	K01929	murF	UDP-N-acetylmuramoyl-tripeptide--D-alanyl-D-alanine ligase	6.3.2.10	Metabolism--Amino acid metabolism--Lysine biosynthesis|Metabolism--Glycan biosynthesis and metabolism--Peptidoglycan biosynthesis|Metabolism--Global and overview maps--Metabolic pathways|Human Diseases--Drug resistance: Antimicrobial--Vancomycin resistance

[denovogenes]_106183	47.47	7e-75	dca:Desca_0984	K06173	truA, PUS1	tRNA pseudouridine38-40 synthase	5.4.99.12	--

[denovogenes]_106184	49.12	3e-64	ruk:A4V00_04575	K05896	scpA	segregation and condensation protein A	--	--

[denovogenes]_106186	40.53	2e-61	yel:LC20_03973	K00065	kduD	2-deoxy-D-gluconate 3-dehydrogenase	1.1.1.125	Metabolism--Carbohydrate metabolism--Pentose and glucuronate interconversions

[denovogenes]_106187	49.81	9e-94	pbd:PBOR_08995	K02026	ABC.MS.P1	multiple sugar transport system permease protein	--	--

[denovogenes]_106188	100.00	0.0	bxy:BXY_04580	K03924	moxR	MoxR-like ATPase	3.6.3.-	--

[denovogenes]_10619	42.92	0.0	rch:RUM_03890	K12573	rnr, vacB	ribonuclease R	3.1.-.-	Genetic Information Processing--Folding, sorting and degradation--RNA degradation

[denovogenes]_106190	68.63	7e-135	ibu:IB211_03182c	K02510	hpaI, hpcH	4-hydroxy-2-oxoheptanedioate aldolase	4.1.2.52	Metabolism--Amino acid metabolism--Tyrosine metabolism|Metabolism--Global and overview maps--Microbial metabolism in diverse environments

[denovogenes]_106192	66.40	2e-115	bprs:CK3_30440	K17733	cwlK	peptidoglycan L-alanyl-D-glutamate endopeptidase CwlK	3.4.-.-	--

[denovogenes]_106193	49.60	1e-90	bpw:WESB_1266	K06971	K06971	uncharacterized protein	--	--

[denovogenes]_106195	85.44	3e-165	bvu:BVU_4194	K02067	mlaD, linM	phospholipid/cholesterol/gamma-HCH transport system substrate-binding protein	--	Environmental Information Processing--Membrane transport--ABC transporters

[denovogenes]_106196	100.00	0.0	sbc:SbBS512_E1870	K08354	phsC	thiosulfate reductase cytochrome b subunit	--	Metabolism--Energy metabolism--Sulfur metabolism|Metabolism--Global and overview maps--Microbial metabolism in diverse environments

[denovogenes]_106198	99.62	0.0	fpla:A4U99_00110	K03496	parA, soj	chromosome partitioning protein	--	--

[denovogenes]_106199	99.23	0.0	bth:BT_1478	K04486	E3.1.3.15B	histidinol-phosphatase (PHP family)	3.1.3.15	Metabolism--Amino acid metabolism--Histidine metabolism|Metabolism--Global and overview maps--Metabolic pathways|Metabolism--Global and overview maps--Biosynthesis of secondary metabolites|Metabolism--Global and overview maps--Biosynthesis of amino acids

[denovogenes]_10620	77.56	0.0	fpla:A4U99_04265	K00700	GBE1, glgB	1,4-alpha-glucan branching enzyme	2.4.1.18	Metabolism--Carbohydrate metabolism--Starch and sucrose metabolism|Metabolism--Global and overview maps--Metabolic pathways|Metabolism--Global and overview maps--Biosynthesis of secondary metabolites

[denovogenes]_106200	52.61	2e-82	nth:Nther_1461	K01358	clpP, CLPP	ATP-dependent Clp protease, protease subunit	3.4.21.92	Cellular Processes--Cell growth and death--Cell cycle - Caulobacter|Organismal Systems--Aging--Longevity regulating pathway - worm

[denovogenes]_106201	69.35	4e-130	csh:Closa_3242	K06989	nadX	aspartate dehydrogenase	1.4.1.21	Metabolism--Metabolism of cofactors and vitamins--Nicotinate and nicotinamide metabolism|Metabolism--Global and overview maps--Metabolic pathways

[denovogenes]_106206	63.32	2e-113	rch:RUM_12800	K04083	hslO	molecular chaperone Hsp33	--	--

[denovogenes]_106208	96.56	0.0	bvu:BVU_3828	K00605	gcvT, AMT	aminomethyltransferase	2.1.2.10	Metabolism--Amino acid metabolism--Glycine, serine and threonine metabolism|Metabolism--Carbohydrate metabolism--Glyoxylate and dicarboxylate metabolism|Metabolism--Metabolism of cofactors and vitamins--One carbon pool by folate|Metabolism--Global and overview maps--Metabolic pathways|Metabolism--Global and overview maps--Biosynthesis of secondary metabolites|Metabolism--Global and overview maps--Biosynthesis of antibiotics|Metabolism--Global and overview maps--Carbon metabolism

[denovogenes]_106210	98.47	0.0	cdl:CDR20291_1751	K19310	bcrB	bacitracin transport system permease protein	--	Environmental Information Processing--Membrane transport--ABC transporters

[denovogenes]_106212	100.00	0.0	ecoi:ECOPMV1_02039	K02421	fliR	flagellar biosynthetic protein FliR	--	Cellular Processes--Cell motility--Flagellar assembly

[denovogenes]_106215	81.44	2e-161	bvu:BVU_0703	K01991	ABC-2.OM, wza	polysaccharide biosynthesis/export protein	--	Cellular Processes--Cellular community - prokaryotes--Biofilm formation - Escherichia coli

[denovogenes]_106217	77.78	2e-146	ere:EUBREC_2003	K02028	ABC.PA.A	polar amino acid transport system ATP-binding protein	3.6.3.21	--

[denovogenes]_106219	83.40	2e-157	rho:RHOM_15650	K03496	parA, soj	chromosome partitioning protein	--	--

[denovogenes]_10622	92.05	0.0	bxy:BXY_41940	K04084	dsbD	thiol:disulfide interchange protein DsbD	1.8.1.8	--

[denovogenes]_106224	64.14	3e-113	ibu:IB211_00871	K02340	DPO3D1, holA	DNA polymerase III subunit delta	2.7.7.7	Metabolism--Nucleotide metabolism--Purine metabolism|Metabolism--Nucleotide metabolism--Pyrimidine metabolism|Metabolism--Global and overview maps--Metabolic pathways|Genetic Information Processing--Replication and repair--DNA replication|Genetic Information Processing--Replication and repair--Mismatch repair|Genetic Information Processing--Replication and repair--Homologous recombination

[denovogenes]_106226	92.37	2e-165	bvu:BVU_3000	K02111	ATPF1A, atpA	F-type H+-transporting ATPase subunit alpha	3.6.3.14	Metabolism--Energy metabolism--Oxidative phosphorylation|Metabolism--Energy metabolism--Photosynthesis|Metabolism--Global and overview maps--Metabolic pathways

[denovogenes]_106227	98.82	0.0	sbc:SbBS512_E1900	K03521	fixA, etfB	electron transfer flavoprotein beta subunit	--	--

[denovogenes]_106229	92.25	6e-169	rto:RTO_10170	K06969	rlmI	23S rRNA (cytosine1962-C5)-methyltransferase	2.1.1.191	--

[denovogenes]_10623	100.00	0.0	bvu:BVU_4136	K12373	HEXA_B	hexosaminidase	3.2.1.52	Metabolism--Glycan biosynthesis and metabolism--Other glycan degradation|Metabolism--Glycan biosynthesis and metabolism--Various types of N-glycan biosynthesis|Metabolism--Carbohydrate metabolism--Amino sugar and nucleotide sugar metabolism|Metabolism--Glycan biosynthesis and metabolism--Glycosaminoglycan degradation|Metabolism--Glycan biosynthesis and metabolism--Glycosphingolipid biosynthesis - globo and isoglobo series|Metabolism--Glycan biosynthesis and metabolism--Glycosphingolipid biosynthesis - ganglio series|Metabolism--Global and overview maps--Metabolic pathways|Cellular Processes--Transport and catabolism--Lysosome

[denovogenes]_106230	46.71	2e-28	rum:CK1_16640	K12511	tadC	tight adherence protein C	--	--

[denovogenes]_106232	98.85	0.0	pdi:BDI_1387	K03322	mntH	manganese transport protein	--	--

[denovogenes]_106233	97.32	0.0	cif:AL515_16145	K06076	fadL	long-chain fatty acid transport protein	--	--

[denovogenes]_106234	40.16	5e-60	psab:PSAB_12505	K00046	idnO	gluconate 5-dehydrogenase	1.1.1.69	--

[denovogenes]_106235	58.78	3e-106	dor:Desor_2897	K09861	K09861	uncharacterized protein	--	--

[denovogenes]_106236	79.77	6e-150	lacy:A4V08_26790	K00375	K00375	GntR family transcriptional regulator / MocR family aminotransferase	--	--

[denovogenes]_106238	99.23	0.0	fpla:A4U99_08590	K14742	tsaB	tRNA threonylcarbamoyladenosine biosynthesis protein TsaB	--	--

[denovogenes]_106239	100.00	0.0	btho:Btheta7330_03214	K07387	K07387	putative metalloprotease	3.4.24.-	--

[denovogenes]_10624	65.79	0.0	csh:Closa_2050	K03572	mutL	DNA mismatch repair protein MutL	--	Genetic Information Processing--Replication and repair--Mismatch repair

[denovogenes]_106241	94.64	0.0	bprs:CK3_23950	K01615	E4.1.1.70	glutaconyl-CoA decarboxylase	4.1.1.70	Metabolism--Xenobiotics biodegradation and metabolism--Benzoate degradation|Metabolism--Carbohydrate metabolism--Butanoate metabolism|Metabolism--Global and overview maps--Microbial metabolism in diverse environments

[denovogenes]_106243	90.95	1e-163	cso:CLS_38210	K03091	SIG3.4	RNA polymerase sporulation-specific sigma factor	--	--

[denovogenes]_106244	81.99	1e-156	euc:EC1_04220	K03496	parA, soj	chromosome partitioning protein	--	--

[denovogenes]_106247	79.62	6e-153	cle:Clole_3046	K01963	accD	acetyl-CoA carboxylase carboxyl transferase subunit beta	6.4.1.2	Metabolism--Lipid metabolism--Fatty acid biosynthesis|Metabolism--Carbohydrate metabolism--Pyruvate metabolism|Metabolism--Carbohydrate metabolism--Propanoate metabolism|Metabolism--Energy metabolism--Carbon fixation pathways in prokaryotes|Metabolism--Global and overview maps--Metabolic pathways|Metabolism--Global and overview maps--Biosynthesis of secondary metabolites|Metabolism--Global and overview maps--Microbial metabolism in diverse environments|Metabolism--Global and overview maps--Biosynthesis of antibiotics|Metabolism--Global and overview maps--Carbon metabolism|Metabolism--Global and overview maps--Fatty acid metabolism

[denovogenes]_10625	92.44	0.0	bvu:BVU_3322	K00700	GBE1, glgB	1,4-alpha-glucan branching enzyme	2.4.1.18	Metabolism--Carbohydrate metabolism--Starch and sucrose metabolism|Metabolism--Global and overview maps--Metabolic pathways|Metabolism--Global and overview maps--Biosynthesis of secondary metabolites

[denovogenes]_106251	41.63	7e-70	csh:Closa_3659	K01992	ABC-2.P	ABC-2 type transport system permease protein	--	--

[denovogenes]_106252	87.69	2e-172	bhl:Bache_0455	K01438	argE	acetylornithine deacetylase	3.5.1.16	Metabolism--Amino acid metabolism--Arginine biosynthesis|Metabolism--Global and overview maps--Metabolic pathways|Metabolism--Global and overview maps--Biosynthesis of secondary metabolites|Metabolism--Global and overview maps--Biosynthesis of antibiotics|Metabolism--Global and overview maps--2-Oxocarboxylic acid metabolism|Metabolism--Global and overview maps--Biosynthesis of amino acids

[denovogenes]_106254	69.50	8e-121	csq:CSCA_3869	K01752	E4.3.1.17, sdaA, sdaB, tdcG	L-serine dehydratase	4.3.1.17	Metabolism--Amino acid metabolism--Glycine, serine and threonine metabolism|Metabolism--Amino acid metabolism--Cysteine and methionine metabolism|Metabolism--Global and overview maps--Metabolic pathways|Metabolism--Global and overview maps--Biosynthesis of secondary metabolites|Metabolism--Global and overview maps--Biosynthesis of antibiotics|Metabolism--Global and overview maps--Carbon metabolism|Metabolism--Global and overview maps--Biosynthesis of amino acids

[denovogenes]_106256	77.48	4e-142	lacy:A4V08_08340	K03631	recN	DNA repair protein RecN (Recombination protein N)	--	--

[denovogenes]_106257	43.70	2e-68	fro:AALO17_22380	K01104	E3.1.3.48	protein-tyrosine phosphatase	3.1.3.48	--

[denovogenes]_106258	67.42	6e-103	spk:MGAS9429_Spy0084	K03043	rpoB	DNA-directed RNA polymerase subunit beta	2.7.7.6	Metabolism--Nucleotide metabolism--Purine metabolism|Metabolism--Nucleotide metabolism--Pyrimidine metabolism|Metabolism--Global and overview maps--Metabolic pathways|Genetic Information Processing--Transcription--RNA polymerase

[denovogenes]_106259	51.34	2e-76	cle:Clole_3596	K02396	flgK	flagellar hook-associated protein 1 FlgK	--	Cellular Processes--Cell motility--Flagellar assembly

[denovogenes]_10626	100.00	0.0	ecoh:ECRM13516_1004	K03658	helD	DNA helicase IV	3.6.4.12	--

[denovogenes]_106260	68.18	1e-100	ers:K210_05860	K07335	bmpA, bmpB, tmpC	basic membrane protein A and related proteins	--	--

[denovogenes]_106262	93.46	8e-168	lacy:A4V08_33230	K03613	rnfE	electron transport complex protein RnfE	--	--

[denovogenes]_106268	68.70	5e-123	ele:Elen_3041	K20074	prpC, phpP	PPM family protein phosphatase	3.1.3.16	--

[denovogenes]_106269	100.00	0.0	sew:SeSA_B0002	K03502	DPO5C, umuC	DNA polymerase V	--	--

[denovogenes]_10627	99.85	0.0	bvu:BVU_0296	K00937	ppk	polyphosphate kinase	2.7.4.1	Metabolism--Energy metabolism--Oxidative phosphorylation|Genetic Information Processing--Folding, sorting and degradation--RNA degradation

[denovogenes]_106270	87.26	1e-168	rto:RTO_23930	K01892	HARS, hisS	histidyl-tRNA synthetase	6.1.1.21	Genetic Information Processing--Translation--Aminoacyl-tRNA biosynthesis

[denovogenes]_106271	80.08	2e-144	cbut:ATN24_20020	K02795	PTS-Man-EIIC, manY	PTS system, mannose-specific IIC component	--	Metabolism--Carbohydrate metabolism--Fructose and mannose metabolism|Metabolism--Carbohydrate metabolism--Amino sugar and nucleotide sugar metabolism|Metabolism--Global and overview maps--Metabolic pathways|Environmental Information Processing--Membrane transport--Phosphotransferase system (PTS)

[denovogenes]_106274	93.89	0.0	bcel:BcellWH2_01693	K01840	manB	phosphomannomutase	5.4.2.8	Metabolism--Carbohydrate metabolism--Fructose and mannose metabolism|Metabolism--Carbohydrate metabolism--Amino sugar and nucleotide sugar metabolism|Metabolism--Global and overview maps--Metabolic pathways|Metabolism--Global and overview maps--Biosynthesis of secondary metabolites|Metabolism--Global and overview maps--Biosynthesis of antibiotics

[denovogenes]_106275	79.01	2e-148	bhl:Bache_1551	K01534	zntA	Cd2+/Zn2+-exporting ATPase	3.6.3.3 3.6.3.5	--

[denovogenes]_106276	41.26	3e-53	bcai:K788_0001292	K12308	bgaB, lacA	beta-galactosidase	3.2.1.23	Metabolism--Carbohydrate metabolism--Galactose metabolism

[denovogenes]_106278	71.37	8e-145	cpas:Clopa_2874	K00100	bdhAB	butanol dehydrogenase	1.1.1.-	Metabolism--Carbohydrate metabolism--Butanoate metabolism

[denovogenes]_106279	46.92	9e-78	bwe:BcerKBAB4_5668	K13010	per, rfbE	perosamine synthetase	2.6.1.102	Metabolism--Carbohydrate metabolism--Amino sugar and nucleotide sugar metabolism

[denovogenes]_106280	69.17	2e-134	agn:AFK25_07630	K07137	K07137	uncharacterized protein	--	--

[denovogenes]_106283	63.12	2e-117	byl:A4V09_00140	K00036	G6PD, zwf	glucose-6-phosphate 1-dehydrogenase	1.1.1.49 1.1.1.363	Metabolism--Carbohydrate metabolism--Pentose phosphate pathway|Metabolism--Metabolism of other amino acids--Glutathione metabolism|Metabolism--Global and overview maps--Metabolic pathways|Metabolism--Global and overview maps--Biosynthesis of secondary metabolites|Metabolism--Global and overview maps--Microbial metabolism in diverse environments|Metabolism--Global and overview maps--Biosynthesis of antibiotics|Metabolism--Global and overview maps--Carbon metabolism|Human Diseases--Cancers: Overview--Central carbon metabolism in cancer

[denovogenes]_106286	48.28	1e-98	cbk:CLL_A0640	K02016	ABC.FEV.S	iron complex transport system substrate-binding protein	--	Environmental Information Processing--Membrane transport--ABC transporters

[denovogenes]_106287	55.43	2e-96	fgl:EM308_14465	K01686	uxuA	mannonate dehydratase	4.2.1.8	Metabolism--Carbohydrate metabolism--Pentose and glucuronate interconversions|Metabolism--Global and overview maps--Metabolic pathways

[denovogenes]_106290	100.00	0.0	elh:ETEC_4730	K02837	prfC	peptide chain release factor 3	--	--

[denovogenes]_106292	58.62	7e-106	mcl:MCCL_1885	K03696	clpC	ATP-dependent Clp protease ATP-binding subunit ClpC	--	--

[denovogenes]_106293	42.86	3e-32	rhz:RHPLAN_54490	K02050	ABC.SN.P	NitT/TauT family transport system permease protein	--	--

[denovogenes]_106294	87.30	2e-157	pfre:RM25_1491	K00655	plsC	1-acyl-sn-glycerol-3-phosphate acyltransferase	2.3.1.51	Metabolism--Lipid metabolism--Glycerolipid metabolism|Metabolism--Lipid metabolism--Glycerophospholipid metabolism|Metabolism--Global and overview maps--Metabolic pathways|Metabolism--Global and overview maps--Biosynthesis of secondary metabolites

[denovogenes]_106296	91.19	0.0	pdi:BDI_3792	K02428	rdgB	XTP/dITP diphosphohydrolase	3.6.1.66	Metabolism--Nucleotide metabolism--Purine metabolism

[denovogenes]_106297	78.16	4e-154	pdi:BDI_3735	K03424	--	--	--	--

[denovogenes]_1063	52.11	0.0	pfre:RM25_1186	K03583	recC	exodeoxyribonuclease V gamma subunit	3.1.11.5	Genetic Information Processing--Replication and repair--Homologous recombination

[denovogenes]_10630	64.68	0.0	fpla:A4U99_14790	K12573	rnr, vacB	ribonuclease R	3.1.-.-	Genetic Information Processing--Folding, sorting and degradation--RNA degradation

[denovogenes]_106300	100.00	0.0	bth:BT_1445	K02169	bioC	malonyl-CoA O-methyltransferase	2.1.1.197	Metabolism--Metabolism of cofactors and vitamins--Biotin metabolism|Metabolism--Global and overview maps--Metabolic pathways

[denovogenes]_106303	56.55	2e-101	fpa:FPR_26210	K03424	--	--	--	--

[denovogenes]_106306	100.00	0.0	cfd:CFNIH1_11000	K02392	flgG	flagellar basal-body rod protein FlgG	--	Cellular Processes--Cell motility--Flagellar assembly

[denovogenes]_106307	99.57	1e-169	cfd:CFNIH1_17860	K01028	E2.8.3.5A, scoA	3-oxoacid CoA-transferase subunit A	2.8.3.5	Metabolism--Lipid metabolism--Synthesis and degradation of ketone bodies|Metabolism--Amino acid metabolism--Valine, leucine and isoleucine degradation|Metabolism--Carbohydrate metabolism--Butanoate metabolism

[denovogenes]_106308	70.52	7e-124	dvg:Deval_2261	K00606	panB	3-methyl-2-oxobutanoate hydroxymethyltransferase	2.1.2.11	Metabolism--Metabolism of cofactors and vitamins--Pantothenate and CoA biosynthesis|Metabolism--Global and overview maps--Metabolic pathways|Metabolism--Global and overview maps--Biosynthesis of secondary metabolites

[denovogenes]_106309	99.22	0.0	fpla:A4U99_10505	K09817	znuC	zinc transport system ATP-binding protein	3.6.3.-	Environmental Information Processing--Membrane transport--ABC transporters

[denovogenes]_10631	61.16	0.0	cdg:CDBI1_02105	K03657	uvrD, pcrA	DNA helicase II / ATP-dependent DNA helicase PcrA	3.6.4.12	Genetic Information Processing--Replication and repair--Nucleotide excision repair|Genetic Information Processing--Replication and repair--Mismatch repair

[denovogenes]_106310	95.20	1e-164	bfr:BF0483	K03630	radC	DNA repair protein RadC	--	--

[denovogenes]_106313	63.22	3e-104	amu:Amuc_1533	K01609	trpC	indole-3-glycerol phosphate synthase	4.1.1.48	Metabolism--Amino acid metabolism--Phenylalanine, tyrosine and tryptophan biosynthesis|Metabolism--Global and overview maps--Metabolic pathways|Metabolism--Global and overview maps--Biosynthesis of secondary metabolites|Metabolism--Global and overview maps--Biosynthesis of antibiotics|Metabolism--Global and overview maps--Biosynthesis of amino acids

[denovogenes]_106315	40.08	5e-58	ccb:Clocel_0322	K03424	--	--	--	--

[denovogenes]_106316	100.00	0.0	cfd:CFNIH1_12720	K14977	ylbA, UGHY	(S)-ureidoglycine aminohydrolase	3.5.3.26	Metabolism--Nucleotide metabolism--Purine metabolism|Metabolism--Global and overview maps--Microbial metabolism in diverse environments

[denovogenes]_106317	100.00	0.0	stb:SGPB_1706	K03706	codY	transcriptional pleiotropic repressor	--	--

[denovogenes]_106318	56.11	8e-102	bbe:BBR47_08260	K12941	abgB	aminobenzoyl-glutamate utilization protein B	--	--

[denovogenes]_106319	100.00	0.0	paeo:M801_3646	K01265	map	methionyl aminopeptidase	3.4.11.18	--

[denovogenes]_106320	99.62	0.0	fpla:A4U99_15265	K04079	HSP90A, htpG	molecular chaperone HtpG	--	Genetic Information Processing--Folding, sorting and degradation--Protein processing in endoplasmic reticulum|Environmental Information Processing--Signal transduction--PI3K-Akt signaling pathway|Organismal Systems--Immune system--Antigen processing and presentation|Organismal Systems--Immune system--NOD-like receptor signaling pathway|Organismal Systems--Environmental adaptation--Plant-pathogen interaction|Organismal Systems--Immune system--IL-17 signaling pathway|Organismal Systems--Immune system--Th17 cell differentiation|Organismal Systems--Endocrine system--Progesterone-mediated oocyte maturation|Organismal Systems--Endocrine system--Estrogen signaling pathway|Human Diseases--Cancers: Overview--Pathways in cancer|Human Diseases--Cancers: Specific types--Prostate cancer|Human Diseases--Cardiovascular diseases--Fluid shear stress and atherosclerosis

[denovogenes]_106321	96.53	2e-171	fpla:A4U99_04150	K02008	cbiQ	cobalt/nickel transport system permease protein	--	Environmental Information Processing--Membrane transport--ABC transporters

[denovogenes]_106322	79.84	2e-144	dpg:DESPIGER_0623	K05781	phnK	putative phosphonate transport system ATP-binding protein	--	--

[denovogenes]_106324	55.20	3e-81	dvu:DVU1097	K09740	K09740	uncharacterized protein	--	--

[denovogenes]_106325	100.00	0.0	paeo:M801_1119	K02392	flgG	flagellar basal-body rod protein FlgG	--	Cellular Processes--Cell motility--Flagellar assembly

[denovogenes]_106327	80.65	2e-141	byl:A4V09_20255	K02010	afuC, fbpC	iron(III) transport system ATP-binding protein	3.6.3.30	Environmental Information Processing--Membrane transport--ABC transporters

[denovogenes]_106328	100.00	0.0	cfd:CFNIH1_20965	K02421	fliR	flagellar biosynthetic protein FliR	--	Cellular Processes--Cell motility--Flagellar assembly

[denovogenes]_106329	59.52	3e-103	bip:Bint_1413	K02050	ABC.SN.P	NitT/TauT family transport system permease protein	--	--

[denovogenes]_106331	72.55	7e-136	dvg:Deval_0123	K11070	potC	spermidine/putrescine transport system permease protein	--	Environmental Information Processing--Membrane transport--ABC transporters

[denovogenes]_106332	50.59	5e-84	seds:AAY24_10205	K01692	paaF, echA	enoyl-CoA hydratase	4.2.1.17	Metabolism--Lipid metabolism--Fatty acid degradation|Metabolism--Amino acid metabolism--Valine, leucine and isoleucine degradation|Metabolism--Metabolism of terpenoids and polyketides--Geraniol degradation|Metabolism--Amino acid metabolism--Lysine degradation|Metabolism--Amino acid metabolism--Phenylalanine metabolism|Metabolism--Xenobiotics biodegradation and metabolism--Benzoate degradation|Metabolism--Amino acid metabolism--Tryptophan metabolism|Metabolism--Metabolism of other amino acids--beta-Alanine metabolism|Metabolism--Xenobiotics biodegradation and metabolism--Aminobenzoate degradation|Metabolism--Carbohydrate metabolism--Propanoate metabolism|Metabolism--Carbohydrate metabolism--Butanoate metabolism|Metabolism--Metabolism of terpenoids and polyketides--Limonene and pinene degradation|Metabolism--Xenobiotics biodegradation and metabolism--Caprolactam degradation|Metabolism--Global and overview maps--Metabolic pathways|Metabolism--Global and overview maps--Biosynthesis of secondary metabolites|Metabolism--Global and overview maps--Microbial metabolism in diverse environments|Metabolism--Global and overview maps--Biosynthesis of antibiotics|Metabolism--Global and overview maps--Fatty acid metabolism

[denovogenes]_106334	88.51	1e-176	bfg:BF638R_4262	K04069	pflA, pflC, pflE	pyruvate formate lyase activating enzyme	1.97.1.4	--

[denovogenes]_106335	40.00	5e-59	bacc:BRDCF_p1645	K01470	E3.5.2.10	creatinine amidohydrolase	3.5.2.10	Metabolism--Amino acid metabolism--Arginine and proline metabolism

[denovogenes]_106336	44.03	6e-59	cdn:BN940_01441	K00059	fabG	3-oxoacyl-[acyl-carrier protein] reductase	1.1.1.100	Metabolism--Lipid metabolism--Fatty acid biosynthesis|Metabolism--Metabolism of cofactors and vitamins--Biotin metabolism|Metabolism--Lipid metabolism--Biosynthesis of unsaturated fatty acids|Metabolism--Global and overview maps--Metabolic pathways|Metabolism--Global and overview maps--Fatty acid metabolism

[denovogenes]_106337	100.00	0.0	fpla:A4U99_04285	K02028	ABC.PA.A	polar amino acid transport system ATP-binding protein	3.6.3.21	--

[denovogenes]_106338	100.00	0.0	cfd:CFNIH1_16965	K02029	ABC.PA.P	polar amino acid transport system permease protein	--	--

[denovogenes]_106339	99.23	0.0	cfd:CFNIH1_16950	K08354	phsC	thiosulfate reductase cytochrome b subunit	--	Metabolism--Energy metabolism--Sulfur metabolism|Metabolism--Global and overview maps--Microbial metabolism in diverse environments

[denovogenes]_10634	44.59	0.0	ccel:CCDG5_1596	K03572	mutL	DNA mismatch repair protein MutL	--	Genetic Information Processing--Replication and repair--Mismatch repair

[denovogenes]_106341	99.62	0.0	pdi:BDI_2106	K00677	lpxA	UDP-N-acetylglucosamine acyltransferase	2.3.1.129	Metabolism--Glycan biosynthesis and metabolism--Lipopolysaccharide biosynthesis|Metabolism--Global and overview maps--Metabolic pathways|Human Diseases--Drug resistance: Antimicrobial--Cationic antimicrobial peptide (CAMP) resistance

[denovogenes]_106343	92.72	2e-179	pdi:BDI_2106	K00677	lpxA	UDP-N-acetylglucosamine acyltransferase	2.3.1.129	Metabolism--Glycan biosynthesis and metabolism--Lipopolysaccharide biosynthesis|Metabolism--Global and overview maps--Metabolic pathways|Human Diseases--Drug resistance: Antimicrobial--Cationic antimicrobial peptide (CAMP) resistance

[denovogenes]_106345	95.02	0.0	bfb:VU15_04495	K02428	rdgB	XTP/dITP diphosphohydrolase	3.6.1.66	Metabolism--Nucleotide metabolism--Purine metabolism

[denovogenes]_106346	42.25	8e-63	erj:EJP617_33040	K16702	amsE	amylovoran biosynthesis glycosyltransferase AmsE	2.4.-.-	--

[denovogenes]_106347	53.07	2e-71	erl:AOC36_02605	K02029	ABC.PA.P	polar amino acid transport system permease protein	--	--

[denovogenes]_106348	90.42	4e-176	pdi:BDI_2359	K01265	map	methionyl aminopeptidase	3.4.11.18	--

[denovogenes]_106349	53.90	8e-97	byl:A4V09_03305	K03660	OGG1	N-glycosylase/DNA lyase	3.2.2.- 4.2.99.18	Genetic Information Processing--Replication and repair--Base excision repair

[denovogenes]_106351	100.00	0.0	prp:M062_00445	K11898	impE	type VI secretion system protein ImpE	--	--

[denovogenes]_106352	56.48	2e-74	sri:SELR_27710	K14084	mttC	trimethylamine corrinoid protein	--	Metabolism--Energy metabolism--Methane metabolism|Metabolism--Global and overview maps--Microbial metabolism in diverse environments|Metabolism--Global and overview maps--Carbon metabolism

[denovogenes]_106353	100.00	0.0	paeo:M801_2968	K00348	nqrC	Na+-transporting NADH:ubiquinone oxidoreductase subunit C	1.6.5.8	--

[denovogenes]_106357	100.00	0.0	pdi:BDI_3792	K02428	rdgB	XTP/dITP diphosphohydrolase	3.6.1.66	Metabolism--Nucleotide metabolism--Purine metabolism

[denovogenes]_106358	99.62	0.0	cfd:CFNIH1_01575	K11264	mmcD	methylmalonyl-CoA decarboxylase	4.1.1.41	Metabolism--Carbohydrate metabolism--Propanoate metabolism

[denovogenes]_10636	76.20	0.0	pdi:BDI_2739	K01206	FUCA	alpha-L-fucosidase	3.2.1.51	Metabolism--Glycan biosynthesis and metabolism--Other glycan degradation

[denovogenes]_106360	64.39	2e-125	dfi:AXF13_02730	K07045	K07045	uncharacterized protein	--	--

[denovogenes]_106361	60.08	2e-106	pfz:AV641_17020	K07046	K07046	L-fuconolactonase	3.1.1.-	Metabolism--Carbohydrate metabolism--Fructose and mannose metabolism|Metabolism--Global and overview maps--Microbial metabolism in diverse environments

[denovogenes]_106364	78.99	4e-144	csh:Closa_0982	K16785	ecfT	energy-coupling factor transport system permease protein	--	Environmental Information Processing--Membrane transport--ABC transporters

[denovogenes]_106366	100.00	0.0	paeg:AI22_26895	K15984	rsmJ	16S rRNA (guanine1516-N2)-methyltransferase	2.1.1.242	--

[denovogenes]_106367	44.91	4e-66	sde:Sde_2935	K01190	lacZ	beta-galactosidase	3.2.1.23	Metabolism--Carbohydrate metabolism--Galactose metabolism|Metabolism--Glycan biosynthesis and metabolism--Other glycan degradation|Metabolism--Lipid metabolism--Sphingolipid metabolism|Metabolism--Global and overview maps--Metabolic pathways

[denovogenes]_10637	83.60	0.0	lacy:A4V08_19705	K00656	E2.3.1.54, pflD	formate C-acetyltransferase	2.3.1.54	Metabolism--Carbohydrate metabolism--Pyruvate metabolism|Metabolism--Carbohydrate metabolism--Propanoate metabolism|Metabolism--Carbohydrate metabolism--Butanoate metabolism|Metabolism--Global and overview maps--Metabolic pathways

[denovogenes]_106370	99.61	0.0	paeo:M801_4170	K01142	E3.1.11.2, xthA	exodeoxyribonuclease III	3.1.11.2	Genetic Information Processing--Replication and repair--Base excision repair

[denovogenes]_106371	50.44	5e-67	bbe:BBR47_55660	K00757	udp, UPP	uridine phosphorylase	2.4.2.3	Metabolism--Nucleotide metabolism--Pyrimidine metabolism|Metabolism--Xenobiotics biodegradation and metabolism--Drug metabolism - other enzymes|Metabolism--Global and overview maps--Metabolic pathways

[denovogenes]_106372	76.13	2e-139	bprs:CK3_09030	K12410	npdA	NAD-dependent deacetylase	3.5.1.-	--

[denovogenes]_106374	40.33	2e-51	csr:Cspa_c02360	K18672	dacA	diadenylate cyclase	2.7.7.85	--

[denovogenes]_106375	100.00	0.0	efq:DR75_2510	K02795	PTS-Man-EIIC, manY	PTS system, mannose-specific IIC component	--	Metabolism--Carbohydrate metabolism--Fructose and mannose metabolism|Metabolism--Carbohydrate metabolism--Amino sugar and nucleotide sugar metabolism|Metabolism--Global and overview maps--Metabolic pathways|Environmental Information Processing--Membrane transport--Phosphotransferase system (PTS)

[denovogenes]_106377	47.54	4e-25	bpt:Bpet1882	K03286	TC.OOP	OmpA-OmpF porin, OOP family	--	--

[denovogenes]_106378	92.34	1e-172	lacy:A4V08_25555	K06989	nadX	aspartate dehydrogenase	1.4.1.21	Metabolism--Metabolism of cofactors and vitamins--Nicotinate and nicotinamide metabolism|Metabolism--Global and overview maps--Metabolic pathways

[denovogenes]_10638	60.62	0.0	esu:EUS_27010	K02400	flhA	flagellar biosynthesis protein FlhA	--	Cellular Processes--Cell motility--Flagellar assembly

[denovogenes]_106380	99.23	0.0	cfd:CFNIH1_21125	K05895	cobK-cbiJ	precorrin-6A/cobalt-precorrin-6A reductase	1.3.1.54 1.3.1.106	Metabolism--Metabolism of cofactors and vitamins--Porphyrin and chlorophyll metabolism|Metabolism--Global and overview maps--Metabolic pathways

[denovogenes]_106382	100.00	0.0	paeo:M801_2891	K10014	hisJ	histidine transport system substrate-binding protein	--	Environmental Information Processing--Membrane transport--ABC transporters

[denovogenes]_106383	70.65	3e-121	rto:RTO_02520	K06346	jag	spoIIIJ-associated protein	--	--

[denovogenes]_106384	90.42	4e-170	lacy:A4V08_04960	K06967	trmK	tRNA (adenine22-N1)-methyltransferase	2.1.1.217	--

[denovogenes]_106385	80.38	1e-159	dde:Dde_1256	K00245	frdB	fumarate reductase iron-sulfur subunit	1.3.5.4	Metabolism--Carbohydrate metabolism--Citrate cycle (TCA cycle)|Metabolism--Energy metabolism--Oxidative phosphorylation|Metabolism--Carbohydrate metabolism--Pyruvate metabolism|Metabolism--Carbohydrate metabolism--Butanoate metabolism|Metabolism--Energy metabolism--Carbon fixation pathways in prokaryotes|Metabolism--Global and overview maps--Metabolic pathways|Metabolism--Global and overview maps--Biosynthesis of secondary metabolites|Metabolism--Global and overview maps--Microbial metabolism in diverse environments|Metabolism--Global and overview maps--Biosynthesis of antibiotics|Metabolism--Global and overview maps--Carbon metabolism|Environmental Information Processing--Signal transduction--Two-component system

[denovogenes]_106386	99.62	0.0	cfd:CFNIH1_09890	K08299	caiD	crotonobetainyl-CoA hydratase	4.2.1.149	--

[denovogenes]_106387	99.62	0.0	cfd:CFNIH1_00500	K03553	recA	recombination protein RecA	--	Genetic Information Processing--Replication and repair--Homologous recombination

[denovogenes]_10639	98.98	0.0	cfd:CFNIH1_14845	K03658	helD	DNA helicase IV	3.6.4.12	--

[denovogenes]_106391	100.00	0.0	pdi:BDI_0179	K03525	coaX	type III pantothenate kinase	2.7.1.33	Metabolism--Metabolism of cofactors and vitamins--Pantothenate and CoA biosynthesis|Metabolism--Global and overview maps--Metabolic pathways

[denovogenes]_106392	61.30	7e-114	bxy:BXY_45290	K07133	K07133	uncharacterized protein	--	--

[denovogenes]_106393	73.28	1e-138	olo:ADJ70_10740	K01626	E2.5.1.54, aroF, aroG, aroH	3-deoxy-7-phosphoheptulonate synthase	2.5.1.54	Metabolism--Amino acid metabolism--Phenylalanine, tyrosine and tryptophan biosynthesis|Metabolism--Global and overview maps--Metabolic pathways|Metabolism--Global and overview maps--Biosynthesis of secondary metabolites|Metabolism--Global and overview maps--Biosynthesis of antibiotics|Metabolism--Global and overview maps--Biosynthesis of amino acids|Cellular Processes--Cellular community - prokaryotes--Quorum sensing

[denovogenes]_106394	40.16	2e-53	amt:Amet_4313	K18672	dacA	diadenylate cyclase	2.7.7.85	--

[denovogenes]_106395	48.63	3e-88	csh:Closa_1180	K02054	ABC.SP.P1	putative spermidine/putrescine transport system permease protein	--	Cellular Processes--Cellular community - prokaryotes--Quorum sensing

[denovogenes]_106396	51.41	3e-80	dvg:Deval_2144	K01057	PGLS, pgl, devB	6-phosphogluconolactonase	3.1.1.31	Metabolism--Carbohydrate metabolism--Pentose phosphate pathway|Metabolism--Global and overview maps--Metabolic pathways|Metabolism--Global and overview maps--Biosynthesis of secondary metabolites|Metabolism--Global and overview maps--Microbial metabolism in diverse environments|Metabolism--Global and overview maps--Biosynthesis of antibiotics|Metabolism--Global and overview maps--Carbon metabolism

[denovogenes]_106397	59.68	3e-104	brm:Bmur_0403	K02050	ABC.SN.P	NitT/TauT family transport system permease protein	--	--

[denovogenes]_1064	100.00	0.0	bth:BT_3774	K01191	E3.2.1.24	alpha-mannosidase	3.2.1.24	Metabolism--Glycan biosynthesis and metabolism--Other glycan degradation

[denovogenes]_10640	99.56	0.0	pdi:BDI_1854	K03798	ftsH, hflB	cell division protease FtsH	3.4.24.-	--

[denovogenes]_106401	62.03	8e-110	gfe:Gferi_21755	K01990	ABC-2.A	ABC-2 type transport system ATP-binding protein	--	--

[denovogenes]_106402	45.53	8e-66	zmo:ZMO1264	K01781	mdlA	mandelate racemase	5.1.2.2	Metabolism--Xenobiotics biodegradation and metabolism--Aminobenzoate degradation|Metabolism--Global and overview maps--Microbial metabolism in diverse environments

[denovogenes]_106403	58.59	2e-105	tbe:Trebr_1227	K02013	ABC.FEV.A	iron complex transport system ATP-binding protein	3.6.3.34	Environmental Information Processing--Membrane transport--ABC transporters

[denovogenes]_106404	55.98	6e-108	chu:CHU_3182	K07263	pqqL	zinc protease	3.4.24.-	--

[denovogenes]_106405	68.28	6e-111	stb:SGPB_0704	K07667	kdpE	two-component system, OmpR family, KDP operon response regulator KdpE	--	Environmental Information Processing--Signal transduction--Two-component system|Cellular Processes--Cellular community - prokaryotes--Quorum sensing

[denovogenes]_106406	100.00	0.0	pdi:BDI_1496	K00059	fabG	3-oxoacyl-[acyl-carrier protein] reductase	1.1.1.100	Metabolism--Lipid metabolism--Fatty acid biosynthesis|Metabolism--Metabolism of cofactors and vitamins--Biotin metabolism|Metabolism--Lipid metabolism--Biosynthesis of unsaturated fatty acids|Metabolism--Global and overview maps--Metabolic pathways|Metabolism--Global and overview maps--Fatty acid metabolism

[denovogenes]_106408	40.36	4e-42	kra:Krad_0012	K08253	E2.7.10.2	non-specific protein-tyrosine kinase	2.7.10.2	--

[denovogenes]_106409	45.88	3e-79	byl:A4V09_20550	K15552	tauC	taurine transport system permease protein	--	Metabolism--Energy metabolism--Sulfur metabolism|Environmental Information Processing--Membrane transport--ABC transporters

[denovogenes]_10641	99.85	0.0	pdi:BDI_0681	K04079	HSP90A, htpG	molecular chaperone HtpG	--	Genetic Information Processing--Folding, sorting and degradation--Protein processing in endoplasmic reticulum|Environmental Information Processing--Signal transduction--PI3K-Akt signaling pathway|Organismal Systems--Immune system--Antigen processing and presentation|Organismal Systems--Immune system--NOD-like receptor signaling pathway|Organismal Systems--Environmental adaptation--Plant-pathogen interaction|Organismal Systems--Immune system--IL-17 signaling pathway|Organismal Systems--Immune system--Th17 cell differentiation|Organismal Systems--Endocrine system--Progesterone-mediated oocyte maturation|Organismal Systems--Endocrine system--Estrogen signaling pathway|Human Diseases--Cancers: Overview--Pathways in cancer|Human Diseases--Cancers: Specific types--Prostate cancer|Human Diseases--Cardiovascular diseases--Fluid shear stress and atherosclerosis

[denovogenes]_106410	100.00	0.0	bbre:B12L_0168	K08483	PTS-EI.PTSI, ptsI	phosphotransferase system, enzyme I, PtsI	2.7.3.9	Environmental Information Processing--Membrane transport--Phosphotransferase system (PTS)

[denovogenes]_106413	99.62	0.0	cfd:CFNIH1_05385	K00874	kdgK	2-dehydro-3-deoxygluconokinase	2.7.1.45	Metabolism--Carbohydrate metabolism--Pentose phosphate pathway|Metabolism--Global and overview maps--Metabolic pathways|Metabolism--Global and overview maps--Microbial metabolism in diverse environments|Metabolism--Global and overview maps--Carbon metabolism

[denovogenes]_106414	48.73	4e-59	ahz:APS56_04630	K18581	ugl	unsaturated chondroitin disaccharide hydrolase	3.2.1.180	--

[denovogenes]_106418	43.63	5e-40	puf:UFO1_0280	K00266	gltD	glutamate synthase (NADPH/NADH) small chain	1.4.1.13 1.4.1.14	Metabolism--Amino acid metabolism--Alanine, aspartate and glutamate metabolism|Metabolism--Energy metabolism--Nitrogen metabolism|Metabolism--Global and overview maps--Metabolic pathways|Metabolism--Global and overview maps--Biosynthesis of secondary metabolites|Metabolism--Global and overview maps--Microbial metabolism in diverse environments|Metabolism--Global and overview maps--Biosynthesis of antibiotics|Metabolism--Global and overview maps--Biosynthesis of amino acids

[denovogenes]_106420	84.41	4e-162	bfb:VU15_16815	K00031	IDH1, IDH2, icd	isocitrate dehydrogenase	1.1.1.42	Metabolism--Carbohydrate metabolism--Citrate cycle (TCA cycle)|Metabolism--Metabolism of other amino acids--Glutathione metabolism|Metabolism--Energy metabolism--Carbon fixation pathways in prokaryotes|Metabolism--Global and overview maps--Metabolic pathways|Metabolism--Global and overview maps--Biosynthesis of secondary metabolites|Metabolism--Global and overview maps--Microbial metabolism in diverse environments|Metabolism--Global and overview maps--Biosynthesis of antibiotics|Metabolism--Global and overview maps--Carbon metabolism|Metabolism--Global and overview maps--2-Oxocarboxylic acid metabolism|Metabolism--Global and overview maps--Biosynthesis of amino acids|Cellular Processes--Transport and catabolism--Peroxisome

[denovogenes]_106424	77.99	9e-153	pdi:BDI_3735	K03424	--	--	--	--

[denovogenes]_106426	100.00	0.0	bbrc:B7019_0497	K02032	ABC.PE.A1	peptide/nickel transport system ATP-binding protein	--	Cellular Processes--Cellular community - prokaryotes--Quorum sensing

[denovogenes]_106427	80.60	1e-137	lacy:A4V08_12865	K01709	rfbG	CDP-glucose 4,6-dehydratase	4.2.1.45	Metabolism--Carbohydrate metabolism--Amino sugar and nucleotide sugar metabolism

[denovogenes]_106428	98.85	3e-179	bbrs:BS27_0138	K04043	dnaK	molecular chaperone DnaK	--	Genetic Information Processing--Folding, sorting and degradation--RNA degradation|Organismal Systems--Aging--Longevity regulating pathway - worm|Human Diseases--Infectious diseases: Bacterial--Tuberculosis

[denovogenes]_106429	62.79	7e-112	ele:Elen_1938	K02003	ABC.CD.A	putative ABC transport system ATP-binding protein	--	--

[denovogenes]_106430	86.64	3e-168	ash:AL1_01000	K01409	KAE1, tsaD, QRI7	N6-L-threonylcarbamoyladenine synthase	2.3.1.234	--

[denovogenes]_106433	96.95	0.0	bcel:BcellWH2_04867	K03385	nrfA	nitrite reductase (cytochrome c-552)	1.7.2.2	Metabolism--Energy metabolism--Nitrogen metabolism|Metabolism--Global and overview maps--Microbial metabolism in diverse environments|Human Diseases--Infectious diseases: Bacterial--Salmonella infection

[denovogenes]_106436	93.08	0.0	bcel:BcellWH2_03579	K00651	metA	homoserine O-succinyltransferase	2.3.1.46	Metabolism--Amino acid metabolism--Cysteine and methionine metabolism|Metabolism--Energy metabolism--Sulfur metabolism|Metabolism--Global and overview maps--Metabolic pathways|Metabolism--Global and overview maps--Biosynthesis of secondary metabolites|Metabolism--Global and overview maps--Biosynthesis of amino acids

[denovogenes]_106438	83.97	3e-157	rum:CK1_06590	K02232	cobQ, cbiP	adenosylcobyric acid synthase	6.3.5.10	Metabolism--Metabolism of cofactors and vitamins--Porphyrin and chlorophyll metabolism|Metabolism--Global and overview maps--Metabolic pathways

[denovogenes]_106439	98.85	0.0	ecl:EcolC_1797	K11392	rsmF	16S rRNA (cytosine1407-C5)-methyltransferase	2.1.1.178	--

[denovogenes]_10644	62.50	0.0	bpb:bpr_I1763	K10797	enr	2-enoate reductase	1.3.1.31	Metabolism--Amino acid metabolism--Phenylalanine metabolism|Metabolism--Global and overview maps--Microbial metabolism in diverse environments

[denovogenes]_106440	100.00	0.0	bbrc:B7019_2049	K10119	msmG	raffinose/stachyose/melibiose transport system permease protein	--	Environmental Information Processing--Membrane transport--ABC transporters

[denovogenes]_106443	99.62	0.0	eun:UMNK88_4504	K00883	dgoK	2-dehydro-3-deoxygalactonokinase	2.7.1.58	Metabolism--Carbohydrate metabolism--Galactose metabolism|Metabolism--Global and overview maps--Metabolic pathways

[denovogenes]_106445	67.05	5e-125	byl:A4V09_04280	K11072	potA	spermidine/putrescine transport system ATP-binding protein	3.6.3.31	Environmental Information Processing--Membrane transport--ABC transporters

[denovogenes]_106446	40.56	8e-56	eha:Ethha_1494	K03547	sbcD	exonuclease SbcD	--	--

[denovogenes]_106449	43.67	4e-49	gba:J421_3604	K01207	nagZ	beta-N-acetylhexosaminidase	3.2.1.52	Metabolism--Carbohydrate metabolism--Amino sugar and nucleotide sugar metabolism|Metabolism--Glycan biosynthesis and metabolism--Glycosaminoglycan degradation|Metabolism--Global and overview maps--Metabolic pathways|Human Diseases--Drug resistance: Antimicrobial--beta-Lactam resistance

[denovogenes]_10645	64.33	0.0	pdi:BDI_2739	K01206	FUCA	alpha-L-fucosidase	3.2.1.51	Metabolism--Glycan biosynthesis and metabolism--Other glycan degradation

[denovogenes]_106450	54.34	3e-86	dosa:Os01t0711400-01	K00281	GLDC, gcvP	glycine dehydrogenase	1.4.4.2	Metabolism--Amino acid metabolism--Glycine, serine and threonine metabolism|Metabolism--Carbohydrate metabolism--Glyoxylate and dicarboxylate metabolism|Metabolism--Global and overview maps--Metabolic pathways|Metabolism--Global and overview maps--Biosynthesis of secondary metabolites|Metabolism--Global and overview maps--Biosynthesis of antibiotics|Metabolism--Global and overview maps--Carbon metabolism

[denovogenes]_106454	72.41	6e-140	cso:CLS_12080	K01480	speB	agmatinase	3.5.3.11	Metabolism--Amino acid metabolism--Arginine and proline metabolism|Metabolism--Global and overview maps--Metabolic pathways

[denovogenes]_106456	54.44	5e-86	lsl:LSL_0805	K01185	E3.2.1.17	lysozyme	3.2.1.17	--

[denovogenes]_106459	98.85	0.0	boa:Bovatus_01882	K00156	poxB	pyruvate dehydrogenase (quinone)	1.2.5.1	Metabolism--Carbohydrate metabolism--Pyruvate metabolism

[denovogenes]_106460	100.00	0.0	ecos:EC958_0394	K02392	flgG	flagellar basal-body rod protein FlgG	--	Cellular Processes--Cell motility--Flagellar assembly

[denovogenes]_106462	80.53	8e-158	lacy:A4V08_25040	K00286	proC	pyrroline-5-carboxylate reductase	1.5.1.2	Metabolism--Amino acid metabolism--Arginine and proline metabolism|Metabolism--Global and overview maps--Metabolic pathways|Metabolism--Global and overview maps--Biosynthesis of secondary metabolites|Metabolism--Global and overview maps--Biosynthesis of antibiotics|Metabolism--Global and overview maps--Biosynthesis of amino acids

[denovogenes]_106463	41.43	1e-44	sze:AW14_06855	K07497	K07497	putative transposase	--	--

[denovogenes]_106464	74.81	2e-142	rto:RTO_23130	K00243	K00243	uncharacterized protein	--	--

[denovogenes]_106465	81.51	2e-135	pdi:BDI_0353	K05516	--	--	--	--

[denovogenes]_106469	93.10	3e-175	bcel:BcellWH2_04710	K03169	topB	DNA topoisomerase III	5.99.1.2	--

[denovogenes]_10647	100.00	0.0	mmk:MU9_2503	K00937	ppk	polyphosphate kinase	2.7.4.1	Metabolism--Energy metabolism--Oxidative phosphorylation|Genetic Information Processing--Folding, sorting and degradation--RNA degradation

[denovogenes]_106470	90.42	1e-176	lacy:A4V08_15120	K00700	GBE1, glgB	1,4-alpha-glucan branching enzyme	2.4.1.18	Metabolism--Carbohydrate metabolism--Starch and sucrose metabolism|Metabolism--Global and overview maps--Metabolic pathways|Metabolism--Global and overview maps--Biosynthesis of secondary metabolites

[denovogenes]_106472	74.33	1e-140	cbut:ATN24_19100	K02749	PTS-Glv-EIIB, glvB, malP, aglA	PTS system, alpha-glucoside-specific IIB component	2.7.1.208 2.7.1.-	Metabolism--Carbohydrate metabolism--Starch and sucrose metabolism|Environmental Information Processing--Membrane transport--Phosphotransferase system (PTS)

[denovogenes]_106472	74.33	1e-140	cbut:ATN24_19100	K02750	PTS-Glv-EIIC, glvC, malP, aglA	PTS system, alpha-glucoside-specific IIC component	--	Metabolism--Carbohydrate metabolism--Starch and sucrose metabolism|Environmental Information Processing--Membrane transport--Phosphotransferase system (PTS)

[denovogenes]_106474	59.70	8e-110	dor:Desor_4409	K00179	iorA	indolepyruvate ferredoxin oxidoreductase, alpha subunit	1.2.7.8	--

[denovogenes]_106475	85.88	3e-168	efe:EFER_2681	K06905	K06905	uncharacterized protein	--	--

[denovogenes]_106477	87.40	2e-162	byl:A4V09_11090	K03545	tig	trigger factor	--	--

[denovogenes]_106478	85.44	9e-169	bcel:BcellWH2_00922	K06113	abnA	arabinan endo-1,5-alpha-L-arabinosidase	3.2.1.99	--

[denovogenes]_10648	100.00	0.0	bfs:BF9343_0029	K01190	lacZ	beta-galactosidase	3.2.1.23	Metabolism--Carbohydrate metabolism--Galactose metabolism|Metabolism--Glycan biosynthesis and metabolism--Other glycan degradation|Metabolism--Lipid metabolism--Sphingolipid metabolism|Metabolism--Global and overview maps--Metabolic pathways

[denovogenes]_106480	86.26	7e-167	rto:RTO_02540	K03495	gidA, mnmG, MTO1	tRNA uridine 5-carboxymethylaminomethyl modification enzyme	--	--

[denovogenes]_106482	49.24	5e-89	csh:Closa_2960	K06015	E3.5.1.81	N-acyl-D-amino-acid deacylase	3.5.1.81	--

[denovogenes]_106487	100.00	0.0	bbrd:BBBR_1329	K00058	serA, PHGDH	D-3-phosphoglycerate dehydrogenase	1.1.1.95	Metabolism--Amino acid metabolism--Glycine, serine and threonine metabolism|Metabolism--Energy metabolism--Methane metabolism|Metabolism--Global and overview maps--Metabolic pathways|Metabolism--Global and overview maps--Microbial metabolism in diverse environments|Metabolism--Global and overview maps--Biosynthesis of antibiotics|Metabolism--Global and overview maps--Carbon metabolism|Metabolism--Global and overview maps--Biosynthesis of amino acids

[denovogenes]_10649	99.71	0.0	bfs:BF9343_3161	K01277	DPP3	dipeptidyl-peptidase III	3.4.14.4	--

[denovogenes]_106490	94.27	7e-178	byl:A4V09_22050	K01951	guaA, GMPS	GMP synthase (glutamine-hydrolysing)	6.3.5.2	Metabolism--Nucleotide metabolism--Purine metabolism|Metabolism--Xenobiotics biodegradation and metabolism--Drug metabolism - other enzymes|Metabolism--Global and overview maps--Metabolic pathways

[denovogenes]_106491	100.00	0.0	pdf:CD630DERM_28170	K18888	efrB	ATP-binding cassette, subfamily B, multidrug efflux pump	--	Environmental Information Processing--Membrane transport--ABC transporters

[denovogenes]_106495	50.00	7e-84	arb:A9P82_12135	K01190	lacZ	beta-galactosidase	3.2.1.23	Metabolism--Carbohydrate metabolism--Galactose metabolism|Metabolism--Glycan biosynthesis and metabolism--Other glycan degradation|Metabolism--Lipid metabolism--Sphingolipid metabolism|Metabolism--Global and overview maps--Metabolic pathways

[denovogenes]_106496	100.00	0.0	bbrc:B7019_1403	K03517	nadA	quinolinate synthase	2.5.1.72	Metabolism--Metabolism of cofactors and vitamins--Nicotinate and nicotinamide metabolism|Metabolism--Global and overview maps--Metabolic pathways

[denovogenes]_106497	40.62	5e-46	psts:E05_27080	K01952	purL, PFAS	phosphoribosylformylglycinamidine synthase	6.3.5.3	Metabolism--Nucleotide metabolism--Purine metabolism|Metabolism--Global and overview maps--Metabolic pathways|Metabolism--Global and overview maps--Biosynthesis of secondary metabolites|Metabolism--Global and overview maps--Biosynthesis of antibiotics

[denovogenes]_10650	77.08	0.0	pdf:CD630DERM_27440	K00951	relA	GTP pyrophosphokinase	2.7.6.5	Metabolism--Nucleotide metabolism--Purine metabolism

[denovogenes]_106500	98.47	0.0	pdc:CDIF630_00682	K07335	bmpA, bmpB, tmpC	basic membrane protein A and related proteins	--	--

[denovogenes]_106501	91.60	4e-180	bprs:CK3_13530	K02358	tuf, TUFM	elongation factor Tu	--	--

[denovogenes]_106502	87.01	4e-166	bfg:BF638R_3068	K03587	ftsI	cell division protein FtsI (penicillin-binding protein 3)	--	Metabolism--Glycan biosynthesis and metabolism--Peptidoglycan biosynthesis|Human Diseases--Drug resistance: Antimicrobial--beta-Lactam resistance

[denovogenes]_106503	98.47	0.0	boa:Bovatus_01971	K05306	phnX	phosphonoacetaldehyde hydrolase	3.11.1.1	Metabolism--Metabolism of other amino acids--Phosphonate and phosphinate metabolism|Metabolism--Global and overview maps--Metabolic pathways|Metabolism--Global and overview maps--Microbial metabolism in diverse environments

[denovogenes]_106504	98.46	0.0	bxy:BXY_41590	K07387	K07387	putative metalloprotease	3.4.24.-	--

[denovogenes]_106507	40.93	3e-57	lba:Lebu_1881	K01776	murI	glutamate racemase	5.1.1.3	Metabolism--Metabolism of other amino acids--D-Glutamine and D-glutamate metabolism|Metabolism--Global and overview maps--Metabolic pathways

[denovogenes]_106509	46.11	8e-40	pdi:BDI_0861	K00558	DNMT1, dcm	DNA (cytosine-5)-methyltransferase 1	2.1.1.37	Metabolism--Amino acid metabolism--Cysteine and methionine metabolism|Metabolism--Global and overview maps--Metabolic pathways|Human Diseases--Cancers: Overview--MicroRNAs in cancer

[denovogenes]_10651	100.00	0.0	pmib:BB2000_3019	K01414	prlC	oligopeptidase A	3.4.24.70	--

[denovogenes]_106510	43.97	3e-72	llr:llh_6685	K04486	E3.1.3.15B	histidinol-phosphatase (PHP family)	3.1.3.15	Metabolism--Amino acid metabolism--Histidine metabolism|Metabolism--Global and overview maps--Metabolic pathways|Metabolism--Global and overview maps--Biosynthesis of secondary metabolites|Metabolism--Global and overview maps--Biosynthesis of amino acids

[denovogenes]_106513	56.76	2e-110	elm:ELI_2329	K05306	phnX	phosphonoacetaldehyde hydrolase	3.11.1.1	Metabolism--Metabolism of other amino acids--Phosphonate and phosphinate metabolism|Metabolism--Global and overview maps--Metabolic pathways|Metabolism--Global and overview maps--Microbial metabolism in diverse environments

[denovogenes]_106516	100.00	0.0	ecoi:ECOPMV1_02696	K03977	engA, der	GTPase	--	--

[denovogenes]_106518	58.46	2e-106	lae:LBAT_1451	K07497	K07497	putative transposase	--	--

[denovogenes]_10652	83.16	0.0	cpf:CPF_2671	K00525	E1.17.4.1A, nrdA, nrdE	ribonucleoside-diphosphate reductase alpha chain	1.17.4.1	Metabolism--Nucleotide metabolism--Purine metabolism|Metabolism--Nucleotide metabolism--Pyrimidine metabolism|Metabolism--Global and overview maps--Metabolic pathways

[denovogenes]_106520	99.62	0.0	boa:Bovatus_03970	K00088	IMPDH, guaB	IMP dehydrogenase	1.1.1.205	Metabolism--Nucleotide metabolism--Purine metabolism|Metabolism--Xenobiotics biodegradation and metabolism--Drug metabolism - other enzymes|Metabolism--Global and overview maps--Metabolic pathways|Metabolism--Global and overview maps--Biosynthesis of secondary metabolites

[denovogenes]_106521	58.20	8e-111	rum:CK1_07980	K02031	ABC.PE.A	peptide/nickel transport system ATP-binding protein	--	Cellular Processes--Cellular community - prokaryotes--Quorum sensing

[denovogenes]_106522	100.00	0.0	boa:Bovatus_04019	K01992	ABC-2.P	ABC-2 type transport system permease protein	--	--

[denovogenes]_106523	100.00	0.0	pvl:AOB99_15855	K02170	bioH	pimeloyl-[acyl-carrier protein] methyl ester esterase	3.1.1.85	Metabolism--Metabolism of cofactors and vitamins--Biotin metabolism|Metabolism--Global and overview maps--Metabolic pathways

[denovogenes]_106525	76.98	2e-146	vpr:Vpar_1855	K06346	jag	spoIIIJ-associated protein	--	--

[denovogenes]_106526	100.00	0.0	pvl:AOB99_06770	K03424	--	--	--	--

[denovogenes]_10653	46.41	0.0	bif:N288_23445	K01361	E3.4.21.96	lactocepin	3.4.21.96	--

[denovogenes]_106530	100.00	0.0	eoc:CE10_0489	K14977	ylbA, UGHY	(S)-ureidoglycine aminohydrolase	3.5.3.26	Metabolism--Nucleotide metabolism--Purine metabolism|Metabolism--Global and overview maps--Microbial metabolism in diverse environments

[denovogenes]_106531	71.86	2e-134	vpr:Vpar_0239	K09817	znuC	zinc transport system ATP-binding protein	3.6.3.-	Environmental Information Processing--Membrane transport--ABC transporters

[denovogenes]_106532	84.29	1e-159	vpr:Vpar_0163	K01695	trpA	tryptophan synthase alpha chain	4.2.1.20	Metabolism--Amino acid metabolism--Glycine, serine and threonine metabolism|Metabolism--Amino acid metabolism--Phenylalanine, tyrosine and tryptophan biosynthesis|Metabolism--Global and overview maps--Metabolic pathways|Metabolism--Global and overview maps--Biosynthesis of secondary metabolites|Metabolism--Global and overview maps--Biosynthesis of antibiotics|Metabolism--Global and overview maps--Biosynthesis of amino acids

[denovogenes]_106534	85.82	2e-166	bpw:WESB_2383	K03521	fixA, etfB	electron transfer flavoprotein beta subunit	--	--

[denovogenes]_106536	100.00	0.0	cpf:CPF_0281	K07058	K07058	membrane protein	--	--

[denovogenes]_106537	59.70	3e-105	bip:Bint_0312	K00882	fruK	1-phosphofructokinase	2.7.1.56	Metabolism--Carbohydrate metabolism--Fructose and mannose metabolism

[denovogenes]_106539	89.27	1e-167	vpr:Vpar_1681	K02314	dnaB	replicative DNA helicase	3.6.4.12	Genetic Information Processing--Replication and repair--DNA replication|Cellular Processes--Cell growth and death--Cell cycle - Caulobacter

[denovogenes]_106541	100.00	0.0	pvl:AOB99_16610	K01735	aroB	3-dehydroquinate synthase	4.2.3.4	Metabolism--Amino acid metabolism--Phenylalanine, tyrosine and tryptophan biosynthesis|Metabolism--Global and overview maps--Metabolic pathways|Metabolism--Global and overview maps--Biosynthesis of secondary metabolites|Metabolism--Global and overview maps--Biosynthesis of antibiotics|Metabolism--Global and overview maps--Biosynthesis of amino acids

[denovogenes]_106542	52.67	1e-82	lmoq:LM6179_1359	K02020	modA	molybdate transport system substrate-binding protein	--	Environmental Information Processing--Membrane transport--ABC transporters

[denovogenes]_106544	52.67	6e-89	ecas:ECBG_02861	K01785	galM, GALM	aldose 1-epimerase	5.1.3.3	Metabolism--Carbohydrate metabolism--Glycolysis / Gluconeogenesis|Metabolism--Carbohydrate metabolism--Galactose metabolism|Metabolism--Global and overview maps--Metabolic pathways|Metabolism--Global and overview maps--Biosynthesis of secondary metabolites|Metabolism--Global and overview maps--Microbial metabolism in diverse environments|Metabolism--Global and overview maps--Biosynthesis of antibiotics

[denovogenes]_106547	82.51	2e-159	edu:LIU_09835	K01478	arcA	arginine deiminase	3.5.3.6	Metabolism--Amino acid metabolism--Arginine biosynthesis|Metabolism--Global and overview maps--Metabolic pathways|Metabolism--Global and overview maps--Biosynthesis of secondary metabolites|Metabolism--Global and overview maps--Biosynthesis of antibiotics

[denovogenes]_106548	77.48	3e-150	ecas:ECBG_02867	K00841	patA	aminotransferase	2.6.1.-	Metabolism--Amino acid metabolism--Lysine biosynthesis|Metabolism--Global and overview maps--Metabolic pathways|Metabolism--Global and overview maps--Biosynthesis of secondary metabolites|Metabolism--Global and overview maps--Biosynthesis of amino acids

[denovogenes]_106551	100.00	0.0	sdy:SDY_3532	K01580	E4.1.1.15, gadB, gadA, GAD	glutamate decarboxylase	4.1.1.15	Metabolism--Amino acid metabolism--Alanine, aspartate and glutamate metabolism|Metabolism--Metabolism of other amino acids--beta-Alanine metabolism|Metabolism--Metabolism of other amino acids--Taurine and hypotaurine metabolism|Metabolism--Carbohydrate metabolism--Butanoate metabolism|Metabolism--Global and overview maps--Metabolic pathways|Metabolism--Global and overview maps--Biosynthesis of secondary metabolites|Metabolism--Global and overview maps--Microbial metabolism in diverse environments|Cellular Processes--Cellular community - prokaryotes--Quorum sensing|Organismal Systems--Nervous system--GABAergic synapse|Human Diseases--Endocrine and metabolic diseases--Type I diabetes mellitus

[denovogenes]_106553	68.97	6e-129	ctet:BN906_02617	K01972	E6.5.1.2, ligA, ligB	DNA ligase (NAD+)	6.5.1.2	Genetic Information Processing--Replication and repair--DNA replication|Genetic Information Processing--Replication and repair--Base excision repair|Genetic Information Processing--Replication and repair--Nucleotide excision repair|Genetic Information Processing--Replication and repair--Mismatch repair

[denovogenes]_106554	99.24	0.0	eun:UMNK88_5139	K03476	ulaG	L-ascorbate 6-phosphate lactonase	3.1.1.-	Metabolism--Carbohydrate metabolism--Ascorbate and aldarate metabolism|Metabolism--Global and overview maps--Metabolic pathways|Metabolism--Global and overview maps--Microbial metabolism in diverse environments

[denovogenes]_106555	90.84	6e-158	ess:ATZ33_07605	K06213	mgtE	magnesium transporter	--	--

[denovogenes]_106556	47.13	1e-79	sagi:MSA_17940	K01006	ppdK	pyruvate, orthophosphate dikinase	2.7.9.1	Metabolism--Carbohydrate metabolism--Pyruvate metabolism|Metabolism--Energy metabolism--Carbon fixation in photosynthetic organisms|Metabolism--Energy metabolism--Carbon fixation pathways in prokaryotes|Metabolism--Global and overview maps--Metabolic pathways|Metabolism--Global and overview maps--Microbial metabolism in diverse environments|Metabolism--Global and overview maps--Carbon metabolism

[denovogenes]_106558	98.09	0.0	ecm:EcSMS35_0028	K12700	--	--	--	--

[denovogenes]_106559	100.00	0.0	ecm:EcSMS35_0028	K12700	--	--	--	--

[denovogenes]_106561	61.24	5e-112	cbt:CLH_2711	K07238	TC.ZIP, zupT, ZRT3, ZIP2	zinc transporter, ZIP family	--	--

[denovogenes]_106562	68.44	3e-124	cbv:U729_2878	K01567	pdaA	peptidoglycan-N-acetylmuramic acid deacetylase	3.5.1.-	--

[denovogenes]_106563	100.00	0.0	mmk:MU9_1782	K02392	flgG	flagellar basal-body rod protein FlgG	--	Cellular Processes--Cell motility--Flagellar assembly

[denovogenes]_106564	100.00	0.0	mmk:MU9_1463	K03748	sanA	SanA protein	--	--

[denovogenes]_106566	100.00	0.0	mmk:MU9_3448	K12144	hyfI	hydrogenase-4 component I	1.-.-.-	--

[denovogenes]_106567	100.00	0.0	mmk:MU9_47	K11070	potC	spermidine/putrescine transport system permease protein	--	Environmental Information Processing--Membrane transport--ABC transporters

[denovogenes]_106568	99.62	0.0	mmk:MU9_78	K07090	K07090	uncharacterized protein	--	--

[denovogenes]_106570	100.00	0.0	lmw:LMOSLCC2755_1741	K00789	metK	S-adenosylmethionine synthetase	2.5.1.6	Metabolism--Amino acid metabolism--Cysteine and methionine metabolism|Metabolism--Global and overview maps--Metabolic pathways|Metabolism--Global and overview maps--Biosynthesis of secondary metabolites|Metabolism--Global and overview maps--Biosynthesis of amino acids

[denovogenes]_106571	41.88	2e-13	pol:Bpro_1716	K01432	AFMID	arylformamidase	3.5.1.9	Metabolism--Amino acid metabolism--Tryptophan metabolism|Metabolism--Carbohydrate metabolism--Glyoxylate and dicarboxylate metabolism|Metabolism--Global and overview maps--Metabolic pathways

[denovogenes]_106572	46.67	4e-68	efd:EFD32_2456	K10716	kch, trkA, mthK, pch	voltage-gated potassium channel	--	--

[denovogenes]_106573	78.46	2e-136	fpp:FPB0191_01566	K02795	PTS-Man-EIIC, manY	PTS system, mannose-specific IIC component	--	Metabolism--Carbohydrate metabolism--Fructose and mannose metabolism|Metabolism--Carbohydrate metabolism--Amino sugar and nucleotide sugar metabolism|Metabolism--Global and overview maps--Metabolic pathways|Environmental Information Processing--Membrane transport--Phosphotransferase system (PTS)

[denovogenes]_106577	100.00	0.0	mmk:MU9_2340	K07274	mipA, ompV	MipA family protein	--	--

[denovogenes]_106578	100.00	0.0	mmk:MU9_839	K09861	K09861	uncharacterized protein	--	--

[denovogenes]_106579	75.38	3e-140	edu:LIU_04330	K02042	phnE	phosphonate transport system permease protein	--	Environmental Information Processing--Membrane transport--ABC transporters

[denovogenes]_106580	83.08	1e-160	lacy:A4V08_31460	K03521	fixA, etfB	electron transfer flavoprotein beta subunit	--	--

[denovogenes]_106581	100.00	0.0	mmk:MU9_1005	K02190	cbiK	sirohydrochlorin cobaltochelatase	4.99.1.3	Metabolism--Metabolism of cofactors and vitamins--Porphyrin and chlorophyll metabolism|Metabolism--Global and overview maps--Metabolic pathways

[denovogenes]_106582	90.77	3e-165	eft:M395_08275	K02795	PTS-Man-EIIC, manY	PTS system, mannose-specific IIC component	--	Metabolism--Carbohydrate metabolism--Fructose and mannose metabolism|Metabolism--Carbohydrate metabolism--Amino sugar and nucleotide sugar metabolism|Metabolism--Global and overview maps--Metabolic pathways|Environmental Information Processing--Membrane transport--Phosphotransferase system (PTS)

[denovogenes]_106583	72.37	5e-139	efd:EFD32_2480	K01962	accA	acetyl-CoA carboxylase carboxyl transferase subunit alpha	6.4.1.2	Metabolism--Lipid metabolism--Fatty acid biosynthesis|Metabolism--Carbohydrate metabolism--Pyruvate metabolism|Metabolism--Carbohydrate metabolism--Propanoate metabolism|Metabolism--Energy metabolism--Carbon fixation pathways in prokaryotes|Metabolism--Global and overview maps--Metabolic pathways|Metabolism--Global and overview maps--Biosynthesis of secondary metabolites|Metabolism--Global and overview maps--Microbial metabolism in diverse environments|Metabolism--Global and overview maps--Biosynthesis of antibiotics|Metabolism--Global and overview maps--Carbon metabolism|Metabolism--Global and overview maps--Fatty acid metabolism

[denovogenes]_106585	68.87	1e-126	cck:Ccar_08140	K01990	ABC-2.A	ABC-2 type transport system ATP-binding protein	--	--

[denovogenes]_106587	78.63	3e-146	lacy:A4V08_19695	K04069	pflA, pflC, pflE	pyruvate formate lyase activating enzyme	1.97.1.4	--

[denovogenes]_106588	56.60	1e-102	yen:YE0252	K01104	E3.1.3.48	protein-tyrosine phosphatase	3.1.3.48	--

[denovogenes]_106589	88.85	2e-174	cbv:U729_2510	K00065	kduD	2-deoxy-D-gluconate 3-dehydrogenase	1.1.1.125	Metabolism--Carbohydrate metabolism--Pentose and glucuronate interconversions

[denovogenes]_106590	50.00	1e-85	mpx:MPD5_0369	K00286	proC	pyrroline-5-carboxylate reductase	1.5.1.2	Metabolism--Amino acid metabolism--Arginine and proline metabolism|Metabolism--Global and overview maps--Metabolic pathways|Metabolism--Global and overview maps--Biosynthesis of secondary metabolites|Metabolism--Global and overview maps--Biosynthesis of antibiotics|Metabolism--Global and overview maps--Biosynthesis of amino acids

[denovogenes]_106591	76.13	2e-130	cbv:U729_1171	K09697	natA	sodium transport system ATP-binding protein	3.6.3.7	Environmental Information Processing--Membrane transport--ABC transporters|Environmental Information Processing--Signal transduction--Two-component system

[denovogenes]_106592	82.69	2e-160	lacy:A4V08_26080	K01448	E3.5.1.28B, amiA, amiB, amiC	N-acetylmuramoyl-L-alanine amidase	3.5.1.28	Human Diseases--Drug resistance: Antimicrobial--Cationic antimicrobial peptide (CAMP) resistance

[denovogenes]_106594	98.85	0.0	cif:AL515_05875	K02421	fliR	flagellar biosynthetic protein FliR	--	Cellular Processes--Cell motility--Flagellar assembly

[denovogenes]_106596	47.24	4e-76	ecas:ECBG_00010	K01104	E3.1.3.48	protein-tyrosine phosphatase	3.1.3.48	--

[denovogenes]_106597	100.00	0.0	mmk:MU9_709	K03426	E3.6.1.22, NUDT12, nudC	NAD+ diphosphatase	3.6.1.22	Metabolism--Metabolism of cofactors and vitamins--Nicotinate and nicotinamide metabolism|Metabolism--Global and overview maps--Metabolic pathways|Cellular Processes--Transport and catabolism--Peroxisome

[denovogenes]_106598	58.75	3e-103	csr:Cspa_c58390	K03217	yidC, spoIIIJ, OXA1, ccfA	YidC/Oxa1 family membrane protein insertase	--	Cellular Processes--Cellular community - prokaryotes--Quorum sensing|Genetic Information Processing--Folding, sorting and degradation--Protein export|Environmental Information Processing--Membrane transport--Bacterial secretion system

[denovogenes]_10660	85.99	0.0	bsca:BBSC_0871	K01547	kdpB	K+-transporting ATPase ATPase B chain	3.6.3.12	Environmental Information Processing--Signal transduction--Two-component system

[denovogenes]_106601	82.33	2e-141	lacy:A4V08_02410	K02003	ABC.CD.A	putative ABC transport system ATP-binding protein	--	--

[denovogenes]_106602	99.62	0.0	efa:EF2197	K07011	K07011	uncharacterized protein	--	--

[denovogenes]_106603	79.17	4e-143	cfd:CFNIH1_17050	K02020	modA	molybdate transport system substrate-binding protein	--	Environmental Information Processing--Membrane transport--ABC transporters

[denovogenes]_106605	70.63	1e-132	cbv:U729_2798	K00806	uppS	undecaprenyl diphosphate synthase	2.5.1.31	Metabolism--Metabolism of terpenoids and polyketides--Terpenoid backbone biosynthesis|Metabolism--Global and overview maps--Biosynthesis of secondary metabolites

[denovogenes]_106607	100.00	0.0	efs:EFS1_1986	K01092	E3.1.3.25, IMPA, suhB	myo-inositol-1(or 4)-monophosphatase	3.1.3.25	Metabolism--Biosynthesis of other secondary metabolites--Streptomycin biosynthesis|Metabolism--Carbohydrate metabolism--Inositol phosphate metabolism|Metabolism--Global and overview maps--Metabolic pathways|Environmental Information Processing--Signal transduction--Phosphatidylinositol signaling system

[denovogenes]_10661	86.60	0.0	rto:RTO_12480	K04759	feoB	ferrous iron transport protein B	--	--

[denovogenes]_106610	60.52	3e-96	cdl:CDR20291_0036	K00991	ispD	2-C-methyl-D-erythritol 4-phosphate cytidylyltransferase	2.7.7.60	Metabolism--Metabolism of terpenoids and polyketides--Terpenoid backbone biosynthesis|Metabolism--Global and overview maps--Metabolic pathways|Metabolism--Global and overview maps--Biosynthesis of secondary metabolites|Metabolism--Global and overview maps--Biosynthesis of antibiotics

[denovogenes]_106611	100.00	0.0	ene:ENT_10450	K03706	codY	transcriptional pleiotropic repressor	--	--

[denovogenes]_106613	59.62	1e-107	cdf:CD630_01330	K02242	comFC	competence protein ComFC	--	--

[denovogenes]_106614	45.80	5e-74	eac:EAL2_c20260	K01104	E3.1.3.48	protein-tyrosine phosphatase	3.1.3.48	--

[denovogenes]_106615	100.00	0.0	efd:EFD32_2041	K00286	proC	pyrroline-5-carboxylate reductase	1.5.1.2	Metabolism--Amino acid metabolism--Arginine and proline metabolism|Metabolism--Global and overview maps--Metabolic pathways|Metabolism--Global and overview maps--Biosynthesis of secondary metabolites|Metabolism--Global and overview maps--Biosynthesis of antibiotics|Metabolism--Global and overview maps--Biosynthesis of amino acids

[denovogenes]_106616	98.46	0.0	sgt:SGGB_1758	K02013	ABC.FEV.A	iron complex transport system ATP-binding protein	3.6.3.34	Environmental Information Processing--Membrane transport--ABC transporters

[denovogenes]_106617	72.40	2e-129	cpr:CPR_0339	K02337	DPO3A1, dnaE	DNA polymerase III subunit alpha	2.7.7.7	Metabolism--Nucleotide metabolism--Purine metabolism|Metabolism--Nucleotide metabolism--Pyrimidine metabolism|Metabolism--Global and overview maps--Metabolic pathways|Genetic Information Processing--Replication and repair--DNA replication|Genetic Information Processing--Replication and repair--Mismatch repair|Genetic Information Processing--Replication and repair--Homologous recombination

[denovogenes]_10662	49.07	0.0	dvl:Dvul_0060	K01533	copB	Cu2+-exporting ATPase	3.6.3.4	--

[denovogenes]_106621	100.00	0.0	vhl:BME96_08060	K18236	lnuB_F, lin	lincosamide nucleotidyltransferase B/F	--	--

[denovogenes]_106622	100.00	2e-177	gmx:3989377	K05573	ndhB	NAD(P)H-quinone oxidoreductase subunit 2	1.6.5.3	Metabolism--Energy metabolism--Oxidative phosphorylation|Metabolism--Global and overview maps--Metabolic pathways

[denovogenes]_106623	100.00	0.0	pvl:AOB99_14075	K03424	--	--	--	--

[denovogenes]_106626	96.54	0.0	cif:AL515_06830	K00077	panE, apbA	2-dehydropantoate 2-reductase	1.1.1.169	Metabolism--Metabolism of cofactors and vitamins--Pantothenate and CoA biosynthesis|Metabolism--Global and overview maps--Metabolic pathways|Metabolism--Global and overview maps--Biosynthesis of secondary metabolites

[denovogenes]_106627	100.00	0.0	stb:SGPB_1430	K13292	lgt, umpA	phosphatidylglycerol:prolipoprotein diacylglycerol transferase	2.-.-.-	--

[denovogenes]_106629	60.63	3e-109	pdf:CD630DERM_34220	K02190	cbiK	sirohydrochlorin cobaltochelatase	4.99.1.3	Metabolism--Metabolism of cofactors and vitamins--Porphyrin and chlorophyll metabolism|Metabolism--Global and overview maps--Metabolic pathways

[denovogenes]_106630	80.82	3e-144	pdf:CD630DERM_34820	K07566	tsaC, rimN, SUA5	L-threonylcarbamoyladenylate synthase	2.7.7.87	--

[denovogenes]_106632	80.08	6e-155	tae:TepiRe1_0196	K03046	rpoC	DNA-directed RNA polymerase subunit beta'	2.7.7.6	Metabolism--Nucleotide metabolism--Purine metabolism|Metabolism--Nucleotide metabolism--Pyrimidine metabolism|Metabolism--Global and overview maps--Metabolic pathways|Genetic Information Processing--Transcription--RNA polymerase

[denovogenes]_106634	63.92	1e-114	cbt:CLH_3304	K02556	motA	chemotaxis protein MotA	--	Environmental Information Processing--Signal transduction--Two-component system|Cellular Processes--Cell motility--Bacterial chemotaxis|Cellular Processes--Cell motility--Flagellar assembly

[denovogenes]_106638	59.07	6e-97	bon:A361_11245	K02746	PTS-Aga-EIIC, agaW	PTS system, N-acetylgalactosamine-specific IIC component	--	Metabolism--Carbohydrate metabolism--Galactose metabolism|Environmental Information Processing--Membrane transport--Phosphotransferase system (PTS)

[denovogenes]_106639	82.76	6e-159	clb:Clo1100_3837	K01875	SARS, serS	seryl-tRNA synthetase	6.1.1.11	Genetic Information Processing--Translation--Aminoacyl-tRNA biosynthesis

[denovogenes]_106641	62.55	3e-109	cck:Ccar_06270	K00215	dapB	4-hydroxy-tetrahydrodipicolinate reductase	1.17.1.8	Metabolism--Biosynthesis of other secondary metabolites--Monobactam biosynthesis|Metabolism--Amino acid metabolism--Lysine biosynthesis|Metabolism--Global and overview maps--Metabolic pathways|Metabolism--Global and overview maps--Biosynthesis of secondary metabolites|Metabolism--Global and overview maps--Microbial metabolism in diverse environments|Metabolism--Global and overview maps--Biosynthesis of antibiotics|Metabolism--Global and overview maps--Biosynthesis of amino acids

[denovogenes]_106644	81.54	2e-149	fnt:HMPREF0405_00589	K19508	PTS-Gfr-EIIC, gfrC	PTS system, fructoselysine/glucoselysine-specific IIC component	--	Environmental Information Processing--Membrane transport--Phosphotransferase system (PTS)

[denovogenes]_106645	67.95	1e-127	gst:HW35_12590	K06889	K06889	uncharacterized protein	--	--

[denovogenes]_106646	82.38	1e-155	cbv:U729_2175	K02026	ABC.MS.P1	multiple sugar transport system permease protein	--	--

[denovogenes]_106647	97.69	0.0	cif:AL515_20930	K01639	E4.1.3.3, nanA, NPL	N-acetylneuraminate lyase	4.1.3.3	Metabolism--Carbohydrate metabolism--Amino sugar and nucleotide sugar metabolism

[denovogenes]_106648	90.00	1e-177	cama:F384_00130	K06859	pgi1	glucose-6-phosphate isomerase, archaeal	5.3.1.9	Metabolism--Carbohydrate metabolism--Glycolysis / Gluconeogenesis|Metabolism--Carbohydrate metabolism--Pentose phosphate pathway|Metabolism--Carbohydrate metabolism--Starch and sucrose metabolism|Metabolism--Carbohydrate metabolism--Amino sugar and nucleotide sugar metabolism|Metabolism--Global and overview maps--Metabolic pathways|Metabolism--Global and overview maps--Biosynthesis of secondary metabolites|Metabolism--Global and overview maps--Microbial metabolism in diverse environments|Metabolism--Global and overview maps--Biosynthesis of antibiotics|Metabolism--Global and overview maps--Carbon metabolism

[denovogenes]_106649	96.54	0.0	esl:O3K_21655	K03424	--	--	--	--

[denovogenes]_10665	74.19	0.0	rto:RTO_24790	K00937	ppk	polyphosphate kinase	2.7.4.1	Metabolism--Energy metabolism--Oxidative phosphorylation|Genetic Information Processing--Folding, sorting and degradation--RNA degradation

[denovogenes]_106650	61.00	4e-101	clt:CM240_1821	K02030	ABC.PA.S	polar amino acid transport system substrate-binding protein	--	--

[denovogenes]_106651	99.62	0.0	sdz:Asd1617_02015	K01008	selD, SEPHS	selenide, water dikinase	2.7.9.3	Metabolism--Metabolism of other amino acids--Selenocompound metabolism|Metabolism--Global and overview maps--Metabolic pathways

[denovogenes]_106652	97.31	0.0	cif:AL515_18540	K06175	truC	tRNA pseudouridine65 synthase	5.4.99.26	--

[denovogenes]_106653	97.31	3e-168	ag:AAA20116	K18214	tetP_A, tet40	MFS transporter, DHA3 family, tetracycline resistance protein	--	--

[denovogenes]_106654	94.84	0.0	cfd:CFNIH1_13225	K01169	rna	ribonuclease I (enterobacter ribonuclease)	3.1.27.6	--

[denovogenes]_106656	63.14	2e-125	auh:AWM75_05085	K01482	DDAH, ddaH	dimethylargininase	3.5.3.18	--

[denovogenes]_106657	99.62	0.0	ecos:EC958_0107	K03424	--	--	--	--

[denovogenes]_106658	60.96	2e-109	ccb:Clocel_3447	K13292	lgt, umpA	phosphatidylglycerol:prolipoprotein diacylglycerol transferase	2.-.-.-	--

[denovogenes]_106660	88.80	9e-156	prg:RB151_039260	K02056	ABC.SS.A	simple sugar transport system ATP-binding protein	3.6.3.17	--

[denovogenes]_106661	88.00	2e-160	cle:Clole_0360	K02003	ABC.CD.A	putative ABC transport system ATP-binding protein	--	--

[denovogenes]_106662	70.36	8e-126	csb:CLSA_c26500	K11041	eta	exfoliative toxin A/B	--	Human Diseases--Infectious diseases: Bacterial--Staphylococcus aureus infection

[denovogenes]_106664	99.62	0.0	shq:A0259_09530	K02392	flgG	flagellar basal-body rod protein FlgG	--	Cellular Processes--Cell motility--Flagellar assembly

[denovogenes]_106665	46.90	2e-63	fjo:Fjoh_4232	K05970	SIAE	sialate O-acetylesterase	3.1.1.53	--

[denovogenes]_106666	98.04	0.0	cbut:ATN24_17595	K03481	glvR	RpiR family transcriptional regulator, glv operon transcriptional regulator	--	--

[denovogenes]_106667	80.77	3e-155	ibu:IB211_00771c	K01686	uxuA	mannonate dehydratase	4.2.1.8	Metabolism--Carbohydrate metabolism--Pentose and glucuronate interconversions|Metabolism--Global and overview maps--Metabolic pathways

[denovogenes]_106668	47.44	2e-59	mhg:MHY_22200	K02469	gyrA	DNA gyrase subunit A	5.99.1.3	--

[denovogenes]_10667	86.94	0.0	bdh:GV66_02520	K02316	dnaG	DNA primase	2.7.7.-	Genetic Information Processing--Replication and repair--DNA replication

[denovogenes]_106671	78.54	2e-151	csr:Cspa_c48140	K19075	cst2, cas7	CRISPR-associated protein Cst2	--	--

[denovogenes]_106672	87.74	7e-165	cbt:CLH_2342	K17828	pyrDI	dihydroorotate dehydrogenase (NAD+) catalytic subunit	1.3.1.14	Metabolism--Nucleotide metabolism--Pyrimidine metabolism|Metabolism--Global and overview maps--Metabolic pathways

[denovogenes]_106673	100.00	0.0	elp:P12B_c1054	K02483	K02483	two-component system, OmpR family, response regulator	--	--

[denovogenes]_106675	71.92	2e-134	cle:Clole_2885	K07478	ycaJ	putative ATPase	--	--

[denovogenes]_106676	81.23	6e-147	cdg:CDBI1_06505	K15738	uup	ATP-binding cassette, subfamily F, uup	--	--

[denovogenes]_106679	80.00	2e-153	pmib:BB2000_3152	K00375	K00375	GntR family transcriptional regulator / MocR family aminotransferase	--	--

[denovogenes]_106680	75.85	1e-140	cbv:U729_458	K10540	mglB	methyl-galactoside transport system substrate-binding protein	--	Environmental Information Processing--Membrane transport--ABC transporters|Cellular Processes--Cell motility--Bacterial chemotaxis

[denovogenes]_106681	87.36	2e-157	cbt:CLH_0374	K04077	groEL, HSPD1	chaperonin GroEL	--	Genetic Information Processing--Folding, sorting and degradation--RNA degradation|Organismal Systems--Aging--Longevity regulating pathway - worm|Human Diseases--Endocrine and metabolic diseases--Type I diabetes mellitus|Human Diseases--Infectious diseases: Bacterial--Legionellosis|Human Diseases--Infectious diseases: Bacterial--Tuberculosis

[denovogenes]_106682	77.25	3e-144	pdf:CD630DERM_11750	K00925	ackA	acetate kinase	2.7.2.1	Metabolism--Metabolism of other amino acids--Taurine and hypotaurine metabolism|Metabolism--Carbohydrate metabolism--Pyruvate metabolism|Metabolism--Carbohydrate metabolism--Propanoate metabolism|Metabolism--Energy metabolism--Methane metabolism|Metabolism--Energy metabolism--Carbon fixation pathways in prokaryotes|Metabolism--Global and overview maps--Metabolic pathways|Metabolism--Global and overview maps--Microbial metabolism in diverse environments|Metabolism--Global and overview maps--Carbon metabolism

[denovogenes]_106685	48.05	8e-82	has:Halsa_0319	K01436	K01436	amidohydrolase	3.5.1.-	--

[denovogenes]_106686	52.73	2e-81	sft:NCTC1_00638	K07646	kdpD	two-component system, OmpR family, sensor histidine kinase KdpD	2.7.13.3	Environmental Information Processing--Signal transduction--Two-component system

[denovogenes]_106687	47.77	2e-80	stb:SGPB_1292	K12999	rgpI	glucosyltransferase	2.4.1.-	--

[denovogenes]_10669	99.27	0.0	fpla:A4U99_00940	K01874	MARS, metG	methionyl-tRNA synthetase	6.1.1.10	Metabolism--Metabolism of other amino acids--Selenocompound metabolism|Genetic Information Processing--Translation--Aminoacyl-tRNA biosynthesis

[denovogenes]_106690	62.64	7e-107	cbt:CLH_2668	K05845	opuC	osmoprotectant transport system substrate-binding protein	--	Environmental Information Processing--Membrane transport--ABC transporters

[denovogenes]_106690	62.64	7e-107	cbt:CLH_2668	K05846	opuBD	osmoprotectant transport system permease protein	--	Environmental Information Processing--Membrane transport--ABC transporters

[denovogenes]_106694	61.48	6e-117	cle:Clole_2969	K00965	galT, GALT	UDPglucose--hexose-1-phosphate uridylyltransferase	2.7.7.12	Metabolism--Carbohydrate metabolism--Galactose metabolism|Metabolism--Carbohydrate metabolism--Amino sugar and nucleotide sugar metabolism|Metabolism--Global and overview maps--Metabolic pathways|Organismal Systems--Endocrine system--Prolactin signaling pathway

[denovogenes]_106695	70.50	1e-135	gfe:Gferi_06770	K06147	ABCB-BAC	ATP-binding cassette, subfamily B, bacterial	--	--

[denovogenes]_106696	69.35	9e-134	mhg:MHY_12980	K03737	por, nifJ	pyruvate-ferredoxin/flavodoxin oxidoreductase	1.2.7.1 1.2.7.-	Metabolism--Carbohydrate metabolism--Glycolysis / Gluconeogenesis|Metabolism--Carbohydrate metabolism--Citrate cycle (TCA cycle)|Metabolism--Carbohydrate metabolism--Pyruvate metabolism|Metabolism--Carbohydrate metabolism--Butanoate metabolism|Metabolism--Energy metabolism--Carbon fixation pathways in prokaryotes|Metabolism--Global and overview maps--Metabolic pathways|Metabolism--Global and overview maps--Microbial metabolism in diverse environments|Metabolism--Global and overview maps--Biosynthesis of antibiotics|Metabolism--Global and overview maps--Carbon metabolism

[denovogenes]_106699	64.86	6e-119	btt:HD73_5582	K03070	secA	preprotein translocase subunit SecA	--	Cellular Processes--Cellular community - prokaryotes--Quorum sensing|Genetic Information Processing--Folding, sorting and degradation--Protein export|Environmental Information Processing--Membrane transport--Bacterial secretion system

[denovogenes]_106702	57.63	2e-90	koe:A225_4576	K04656	hypF	hydrogenase maturation protein HypF	--	--

[denovogenes]_106703	98.47	0.0	ecm:EcSMS35_2394	K00112	glpB	glycerol-3-phosphate dehydrogenase subunit B	1.1.5.3	Metabolism--Lipid metabolism--Glycerophospholipid metabolism|Metabolism--Global and overview maps--Biosynthesis of secondary metabolites

[denovogenes]_106706	89.27	8e-167	prg:RB151_008710	K10441	rbsA	ribose transport system ATP-binding protein	3.6.3.17	Environmental Information Processing--Membrane transport--ABC transporters

[denovogenes]_106707	46.97	3e-76	euc:EC1_02250	K01006	ppdK	pyruvate, orthophosphate dikinase	2.7.9.1	Metabolism--Carbohydrate metabolism--Pyruvate metabolism|Metabolism--Energy metabolism--Carbon fixation in photosynthetic organisms|Metabolism--Energy metabolism--Carbon fixation pathways in prokaryotes|Metabolism--Global and overview maps--Metabolic pathways|Metabolism--Global and overview maps--Microbial metabolism in diverse environments|Metabolism--Global and overview maps--Carbon metabolism

[denovogenes]_106708	62.84	2e-111	clj:CLJU_c21230	K03572	mutL	DNA mismatch repair protein MutL	--	Genetic Information Processing--Replication and repair--Mismatch repair

[denovogenes]_106709	77.01	9e-145	cle:Clole_2832	K15633	gpmI	2,3-bisphosphoglycerate-independent phosphoglycerate mutase	5.4.2.12	Metabolism--Carbohydrate metabolism--Glycolysis / Gluconeogenesis|Metabolism--Amino acid metabolism--Glycine, serine and threonine metabolism|Metabolism--Energy metabolism--Methane metabolism|Metabolism--Global and overview maps--Metabolic pathways|Metabolism--Global and overview maps--Biosynthesis of secondary metabolites|Metabolism--Global and overview maps--Microbial metabolism in diverse environments|Metabolism--Global and overview maps--Biosynthesis of antibiotics|Metabolism--Global and overview maps--Carbon metabolism|Metabolism--Global and overview maps--Biosynthesis of amino acids

[denovogenes]_106712	66.67	4e-121	afn:Acfer_1196	K00215	dapB	4-hydroxy-tetrahydrodipicolinate reductase	1.17.1.8	Metabolism--Biosynthesis of other secondary metabolites--Monobactam biosynthesis|Metabolism--Amino acid metabolism--Lysine biosynthesis|Metabolism--Global and overview maps--Metabolic pathways|Metabolism--Global and overview maps--Biosynthesis of secondary metabolites|Metabolism--Global and overview maps--Microbial metabolism in diverse environments|Metabolism--Global and overview maps--Biosynthesis of antibiotics|Metabolism--Global and overview maps--Biosynthesis of amino acids

[denovogenes]_106713	64.62	7e-111	afn:Acfer_0649	K02030	ABC.PA.S	polar amino acid transport system substrate-binding protein	--	--

[denovogenes]_106716	100.00	0.0	bvu:BVU_0073	K05801	djlA	DnaJ like chaperone protein	--	--

[denovogenes]_106717	81.85	2e-149	rto:RTO_15670	K03706	codY	transcriptional pleiotropic repressor	--	--

[denovogenes]_106722	52.36	3e-97	pft:JBW_03381	K02013	ABC.FEV.A	iron complex transport system ATP-binding protein	3.6.3.34	Environmental Information Processing--Membrane transport--ABC transporters

[denovogenes]_106723	83.85	8e-160	bhl:Bache_1231	K05801	djlA	DnaJ like chaperone protein	--	--

[denovogenes]_106724	88.46	5e-168	boa:Bovatus_01220	K01609	trpC	indole-3-glycerol phosphate synthase	4.1.1.48	Metabolism--Amino acid metabolism--Phenylalanine, tyrosine and tryptophan biosynthesis|Metabolism--Global and overview maps--Metabolic pathways|Metabolism--Global and overview maps--Biosynthesis of secondary metabolites|Metabolism--Global and overview maps--Biosynthesis of antibiotics|Metabolism--Global and overview maps--Biosynthesis of amino acids

[denovogenes]_106725	70.93	7e-133	bfi:CIY_15040	K01609	trpC	indole-3-glycerol phosphate synthase	4.1.1.48	Metabolism--Amino acid metabolism--Phenylalanine, tyrosine and tryptophan biosynthesis|Metabolism--Global and overview maps--Metabolic pathways|Metabolism--Global and overview maps--Biosynthesis of secondary metabolites|Metabolism--Global and overview maps--Biosynthesis of antibiotics|Metabolism--Global and overview maps--Biosynthesis of amino acids

[denovogenes]_106729	46.88	4e-58	byl:A4V09_05300	K08600	srtB	sortase B	3.4.22.70	--

[denovogenes]_10673	60.23	0.0	hsd:SD1D_1105	K03529	smc	chromosome segregation protein	--	--

[denovogenes]_106731	82.24	2e-160	rto:RTO_05910	K02823	pyrDII	dihydroorotate dehydrogenase electron transfer subunit	--	Metabolism--Nucleotide metabolism--Pyrimidine metabolism|Metabolism--Global and overview maps--Metabolic pathways

[denovogenes]_106732	92.31	5e-180	bxy:BXY_41590	K07387	K07387	putative metalloprotease	3.4.24.-	--

[denovogenes]_106735	64.09	5e-121	tjr:TherJR_2666	K01995	livG	branched-chain amino acid transport system ATP-binding protein	--	Environmental Information Processing--Membrane transport--ABC transporters|Cellular Processes--Cellular community - prokaryotes--Quorum sensing

[denovogenes]_106737	53.01	1e-94	csh:Closa_3447	K02405	fliA	RNA polymerase sigma factor for flagellar operon FliA	--	Environmental Information Processing--Signal transduction--Two-component system|Cellular Processes--Cellular community - prokaryotes--Biofilm formation - Pseudomonas aeruginosa|Cellular Processes--Cellular community - prokaryotes--Biofilm formation - Escherichia coli|Cellular Processes--Cell motility--Flagellar assembly|Cellular Processes--Cellular community - prokaryotes--Biofilm formation - Vibrio cholerae

[denovogenes]_106741	86.92	2e-149	fpla:A4U99_01345	K03218	rlmB	23S rRNA (guanosine2251-2'-O)-methyltransferase	2.1.1.185	--

[denovogenes]_106742	65.12	1e-117	tae:TepiRe1_2385	K01803	TPI, tpiA	triosephosphate isomerase (TIM)	5.3.1.1	Metabolism--Carbohydrate metabolism--Glycolysis / Gluconeogenesis|Metabolism--Carbohydrate metabolism--Fructose and mannose metabolism|Metabolism--Carbohydrate metabolism--Inositol phosphate metabolism|Metabolism--Energy metabolism--Carbon fixation in photosynthetic organisms|Metabolism--Global and overview maps--Metabolic pathways|Metabolism--Global and overview maps--Biosynthesis of secondary metabolites|Metabolism--Global and overview maps--Microbial metabolism in diverse environments|Metabolism--Global and overview maps--Biosynthesis of antibiotics|Metabolism--Global and overview maps--Carbon metabolism|Metabolism--Global and overview maps--Biosynthesis of amino acids

[denovogenes]_106743	81.35	7e-153	cso:CLS_35440	K16961	yxeM	putative amino-acid transport system substrate-binding protein	--	Environmental Information Processing--Membrane transport--ABC transporters

[denovogenes]_106744	66.02	7e-114	ibu:IB211_01523	K02008	cbiQ	cobalt/nickel transport system permease protein	--	Environmental Information Processing--Membrane transport--ABC transporters

[denovogenes]_106746	99.62	0.0	bxy:BXY_44870	K04486	E3.1.3.15B	histidinol-phosphatase (PHP family)	3.1.3.15	Metabolism--Amino acid metabolism--Histidine metabolism|Metabolism--Global and overview maps--Metabolic pathways|Metabolism--Global and overview maps--Biosynthesis of secondary metabolites|Metabolism--Global and overview maps--Biosynthesis of amino acids

[denovogenes]_106747	99.51	2e-150	bvu:BVU_3160	K21063	K21063	5-amino-6-(5-phospho-D-ribitylamino)uracil phosphatase	3.1.3.104	Metabolism--Metabolism of cofactors and vitamins--Riboflavin metabolism|Metabolism--Global and overview maps--Metabolic pathways|Metabolism--Global and overview maps--Biosynthesis of secondary metabolites

[denovogenes]_106749	43.08	2e-65	gym:GYMC10_6252	K00662	aacC	aminoglycoside 3-N-acetyltransferase	2.3.1.81	--

[denovogenes]_106751	93.46	0.0	bxy:BXY_41590	K07387	K07387	putative metalloprotease	3.4.24.-	--

[denovogenes]_106754	89.02	2e-166	lacy:A4V08_17735	K06346	jag	spoIIIJ-associated protein	--	--

[denovogenes]_106756	75.71	4e-146	csh:Closa_0365	K02036	pstB	phosphate transport system ATP-binding protein	3.6.3.27	Environmental Information Processing--Membrane transport--ABC transporters

[denovogenes]_106757	98.08	0.0	bxy:BXY_00910	K01609	trpC	indole-3-glycerol phosphate synthase	4.1.1.48	Metabolism--Amino acid metabolism--Phenylalanine, tyrosine and tryptophan biosynthesis|Metabolism--Global and overview maps--Metabolic pathways|Metabolism--Global and overview maps--Biosynthesis of secondary metabolites|Metabolism--Global and overview maps--Biosynthesis of antibiotics|Metabolism--Global and overview maps--Biosynthesis of amino acids

[denovogenes]_106758	70.38	2e-133	edu:LIU_04330	K02042	phnE	phosphonate transport system permease protein	--	Environmental Information Processing--Membrane transport--ABC transporters

[denovogenes]_106759	88.62	2e-157	cso:CLS_38200	K03091	SIG3.4	RNA polymerase sporulation-specific sigma factor	--	--

[denovogenes]_106760	99.62	0.0	shq:A0259_15365	K10014	hisJ	histidine transport system substrate-binding protein	--	Environmental Information Processing--Membrane transport--ABC transporters

[denovogenes]_106761	100.00	0.0	shq:A0259_15370	K10013	ABC.ARG.S, argT	lysine/arginine/ornithine transport system substrate-binding protein	--	Environmental Information Processing--Membrane transport--ABC transporters

[denovogenes]_106763	65.00	9e-127	elm:ELI_2329	K05306	phnX	phosphonoacetaldehyde hydrolase	3.11.1.1	Metabolism--Metabolism of other amino acids--Phosphonate and phosphinate metabolism|Metabolism--Global and overview maps--Metabolic pathways|Metabolism--Global and overview maps--Microbial metabolism in diverse environments

[denovogenes]_106764	59.11	5e-108	bvs:BARVI_00805	K01778	dapF	diaminopimelate epimerase	5.1.1.7	Metabolism--Amino acid metabolism--Lysine biosynthesis|Metabolism--Global and overview maps--Metabolic pathways|Metabolism--Global and overview maps--Biosynthesis of secondary metabolites|Metabolism--Global and overview maps--Microbial metabolism in diverse environments|Metabolism--Global and overview maps--Biosynthesis of antibiotics|Metabolism--Global and overview maps--Biosynthesis of amino acids

[denovogenes]_106768	100.00	0.0	fpla:A4U99_17125	K07238	TC.ZIP, zupT, ZRT3, ZIP2	zinc transporter, ZIP family	--	--

[denovogenes]_10677	86.07	0.0	pdi:BDI_0681	K04079	HSP90A, htpG	molecular chaperone HtpG	--	Genetic Information Processing--Folding, sorting and degradation--Protein processing in endoplasmic reticulum|Environmental Information Processing--Signal transduction--PI3K-Akt signaling pathway|Organismal Systems--Immune system--Antigen processing and presentation|Organismal Systems--Immune system--NOD-like receptor signaling pathway|Organismal Systems--Environmental adaptation--Plant-pathogen interaction|Organismal Systems--Immune system--IL-17 signaling pathway|Organismal Systems--Immune system--Th17 cell differentiation|Organismal Systems--Endocrine system--Progesterone-mediated oocyte maturation|Organismal Systems--Endocrine system--Estrogen signaling pathway|Human Diseases--Cancers: Overview--Pathways in cancer|Human Diseases--Cancers: Specific types--Prostate cancer|Human Diseases--Cardiovascular diseases--Fluid shear stress and atherosclerosis

[denovogenes]_106770	55.73	3e-106	fpla:A4U99_06680	K02013	ABC.FEV.A	iron complex transport system ATP-binding protein	3.6.3.34	Environmental Information Processing--Membrane transport--ABC transporters

[denovogenes]_106771	100.00	0.0	ecol:LY180_14180	K06175	truC	tRNA pseudouridine65 synthase	5.4.99.26	--

[denovogenes]_106774	66.93	2e-124	ccel:CCDG5_1660	K03091	SIG3.4	RNA polymerase sporulation-specific sigma factor	--	--

[denovogenes]_106775	60.85	6e-105	erb:A4V01_12120	K00014	aroE	shikimate dehydrogenase	1.1.1.25	Metabolism--Amino acid metabolism--Phenylalanine, tyrosine and tryptophan biosynthesis|Metabolism--Global and overview maps--Metabolic pathways|Metabolism--Global and overview maps--Biosynthesis of secondary metabolites|Metabolism--Global and overview maps--Biosynthesis of antibiotics|Metabolism--Global and overview maps--Biosynthesis of amino acids

[denovogenes]_106776	100.00	0.0	bvu:BVU_1684	K05810	yfiH	laccase domain-containing protein	--	--

[denovogenes]_106777	100.00	0.0	shq:A0259_19720	K02066	mlaE, linK	phospholipid/cholesterol/gamma-HCH transport system permease protein	--	Environmental Information Processing--Membrane transport--ABC transporters

[denovogenes]_106778	88.08	4e-170	lacy:A4V08_26080	K01448	E3.5.1.28B, amiA, amiB, amiC	N-acetylmuramoyl-L-alanine amidase	3.5.1.28	Human Diseases--Drug resistance: Antimicrobial--Cationic antimicrobial peptide (CAMP) resistance

[denovogenes]_10678	100.00	0.0	cfd:CFNIH1_11875	K02011	afuB, fbpB	iron(III) transport system permease protein	--	Environmental Information Processing--Membrane transport--ABC transporters

[denovogenes]_106780	41.34	3e-61	ccel:CCDG5_0873	K03525	coaX	type III pantothenate kinase	2.7.1.33	Metabolism--Metabolism of cofactors and vitamins--Pantothenate and CoA biosynthesis|Metabolism--Global and overview maps--Metabolic pathways

[denovogenes]_106781	64.92	2e-116	ova:OBV_05310	K02003	ABC.CD.A	putative ABC transport system ATP-binding protein	--	--

[denovogenes]_106783	71.78	6e-130	rto:RTO_23790	K02564	nagB, GNPDA	glucosamine-6-phosphate deaminase	3.5.99.6	Metabolism--Carbohydrate metabolism--Amino sugar and nucleotide sugar metabolism|Metabolism--Global and overview maps--Metabolic pathways

[denovogenes]_106786	61.54	1e-111	cct:CC1_24220	K07238	TC.ZIP, zupT, ZRT3, ZIP2	zinc transporter, ZIP family	--	--

[denovogenes]_106789	69.41	5e-132	spyh:L897_08805	K01712	hutU, UROC1	urocanate hydratase	4.2.1.49	Metabolism--Amino acid metabolism--Histidine metabolism|Metabolism--Global and overview maps--Metabolic pathways

[denovogenes]_10679	83.21	0.0	bsca:BBSC_0871	K01547	kdpB	K+-transporting ATPase ATPase B chain	3.6.3.12	Environmental Information Processing--Signal transduction--Two-component system

[denovogenes]_106791	87.69	2e-164	cle:Clole_0248	K02026	ABC.MS.P1	multiple sugar transport system permease protein	--	--

[denovogenes]_106792	71.60	7e-133	cpy:Cphy_0433	K02034	ABC.PE.P1	peptide/nickel transport system permease protein	--	Cellular Processes--Cellular community - prokaryotes--Quorum sensing

[denovogenes]_106793	45.53	7e-78	aoe:Clos_0860	K07646	kdpD	two-component system, OmpR family, sensor histidine kinase KdpD	2.7.13.3	Environmental Information Processing--Signal transduction--Two-component system

[denovogenes]_106795	99.62	0.0	eal:EAKF1_ch3808	K07552	bcr	MFS transporter, DHA1 family, bicyclomycin/chloramphenicol resistance protein	--	--

[denovogenes]_106796	58.46	1e-99	cad:Curi_c11730	K01997	livH	branched-chain amino acid transport system permease protein	--	Environmental Information Processing--Membrane transport--ABC transporters|Cellular Processes--Cellular community - prokaryotes--Quorum sensing

[denovogenes]_106797	98.83	0.0	fpla:A4U99_02045	K01803	TPI, tpiA	triosephosphate isomerase (TIM)	5.3.1.1	Metabolism--Carbohydrate metabolism--Glycolysis / Gluconeogenesis|Metabolism--Carbohydrate metabolism--Fructose and mannose metabolism|Metabolism--Carbohydrate metabolism--Inositol phosphate metabolism|Metabolism--Energy metabolism--Carbon fixation in photosynthetic organisms|Metabolism--Global and overview maps--Metabolic pathways|Metabolism--Global and overview maps--Biosynthesis of secondary metabolites|Metabolism--Global and overview maps--Microbial metabolism in diverse environments|Metabolism--Global and overview maps--Biosynthesis of antibiotics|Metabolism--Global and overview maps--Carbon metabolism|Metabolism--Global and overview maps--Biosynthesis of amino acids

[denovogenes]_106798	47.04	5e-78	sri:SELR_03170	K00286	proC	pyrroline-5-carboxylate reductase	1.5.1.2	Metabolism--Amino acid metabolism--Arginine and proline metabolism|Metabolism--Global and overview maps--Metabolic pathways|Metabolism--Global and overview maps--Biosynthesis of secondary metabolites|Metabolism--Global and overview maps--Biosynthesis of antibiotics|Metabolism--Global and overview maps--Biosynthesis of amino acids

[denovogenes]_106799	81.23	2e-146	cpy:Cphy_0861	K17320	lplC	putative aldouronate transport system permease protein	--	Environmental Information Processing--Membrane transport--ABC transporters

[denovogenes]_10680	62.80	0.0	fpa:FPR_19830	K03655	recG	ATP-dependent DNA helicase RecG	3.6.4.12	Genetic Information Processing--Replication and repair--Homologous recombination

[denovogenes]_106800	100.00	9e-180	sbc:SbBS512_E4553	K03327	TC.MATE, SLC47A, norM, mdtK, dinF	multidrug resistance protein, MATE family	--	--

[denovogenes]_106801	98.85	0.0	cfd:CFNIH1_05460	K03796	bax	Bax protein	--	--

[denovogenes]_106802	100.00	0.0	fpla:A4U99_17040	K11534	deoR	DeoR family transcriptional regulator, deoxyribose operon repressor	--	--

[denovogenes]_106803	88.70	3e-158	lacy:A4V08_13525	K12410	npdA	NAD-dependent deacetylase	3.5.1.-	--

[denovogenes]_106804	83.20	3e-148	lacy:A4V08_25585	K07090	K07090	uncharacterized protein	--	--

[denovogenes]_106806	66.54	8e-119	fsc:FSU_3073	K02028	ABC.PA.A	polar amino acid transport system ATP-binding protein	3.6.3.21	--

[denovogenes]_106808	55.79	1e-95	hsd:SD1D_0630	K04517	tyrA2	prephenate dehydrogenase	1.3.1.12	Metabolism--Amino acid metabolism--Phenylalanine, tyrosine and tryptophan biosynthesis|Metabolism--Biosynthesis of other secondary metabolites--Novobiocin biosynthesis|Metabolism--Global and overview maps--Metabolic pathways|Metabolism--Global and overview maps--Biosynthesis of secondary metabolites|Metabolism--Global and overview maps--Biosynthesis of antibiotics|Metabolism--Global and overview maps--Biosynthesis of amino acids

[denovogenes]_106809	95.40	4e-175	lacy:A4V08_30760	K10557	lsrD	AI-2 transport system permease protein	--	Environmental Information Processing--Membrane transport--ABC transporters|Cellular Processes--Cellular community - prokaryotes--Quorum sensing

[denovogenes]_10681	92.39	0.0	bfg:BF638R_0029	K01190	lacZ	beta-galactosidase	3.2.1.23	Metabolism--Carbohydrate metabolism--Galactose metabolism|Metabolism--Glycan biosynthesis and metabolism--Other glycan degradation|Metabolism--Lipid metabolism--Sphingolipid metabolism|Metabolism--Global and overview maps--Metabolic pathways

[denovogenes]_106810	98.46	2e-180	lacy:A4V08_12485	K02053	ABC.SP.P	putative spermidine/putrescine transport system permease protein	--	Cellular Processes--Cellular community - prokaryotes--Quorum sensing

[denovogenes]_106815	63.18	5e-117	carc:NY10_2134	K02037	pstC	phosphate transport system permease protein	--	Environmental Information Processing--Membrane transport--ABC transporters

[denovogenes]_106816	100.00	0.0	elp:P12B_c0220	K02421	fliR	flagellar biosynthetic protein FliR	--	Cellular Processes--Cell motility--Flagellar assembly

[denovogenes]_106818	48.59	7e-73	awo:Awo_c02020	K02654	pilD, pppA	leader peptidase (prepilin peptidase) / N-methyltransferase	3.4.23.43 2.1.1.-	--

[denovogenes]_106819	71.71	3e-110	tsu:Tresu_0180	K07035	K07035	uncharacterized protein	--	--

[denovogenes]_106820	85.71	1e-163	cfd:CFNIH1_04640	K12288	hofM	pilus assembly protein HofM	--	--

[denovogenes]_106822	67.44	3e-129	lacy:A4V08_00555	K12267	msrAB	peptide methionine sulfoxide reductase msrA/msrB	1.8.4.11 1.8.4.12	--

[denovogenes]_106823	51.54	1e-96	lacy:A4V08_06015	K02013	ABC.FEV.A	iron complex transport system ATP-binding protein	3.6.3.34	Environmental Information Processing--Membrane transport--ABC transporters

[denovogenes]_106824	68.09	8e-127	rho:RHOM_15885	K00016	LDH, ldh	L-lactate dehydrogenase	1.1.1.27	Metabolism--Carbohydrate metabolism--Glycolysis / Gluconeogenesis|Metabolism--Amino acid metabolism--Cysteine and methionine metabolism|Metabolism--Carbohydrate metabolism--Pyruvate metabolism|Metabolism--Carbohydrate metabolism--Propanoate metabolism|Metabolism--Global and overview maps--Metabolic pathways|Metabolism--Global and overview maps--Biosynthesis of secondary metabolites|Metabolism--Global and overview maps--Microbial metabolism in diverse environments|Metabolism--Global and overview maps--Biosynthesis of antibiotics|Organismal Systems--Endocrine system--Glucagon signaling pathway

[denovogenes]_106825	99.62	0.0	cfd:CFNIH1_23100	K10013	ABC.ARG.S, argT	lysine/arginine/ornithine transport system substrate-binding protein	--	Environmental Information Processing--Membrane transport--ABC transporters

[denovogenes]_106826	95.00	0.0	bdh:GV66_08535	K00790	murA	UDP-N-acetylglucosamine 1-carboxyvinyltransferase	2.5.1.7	Metabolism--Carbohydrate metabolism--Amino sugar and nucleotide sugar metabolism|Metabolism--Glycan biosynthesis and metabolism--Peptidoglycan biosynthesis|Metabolism--Global and overview maps--Metabolic pathways

[denovogenes]_106827	77.82	3e-149	bprs:CK3_23130	K00757	udp, UPP	uridine phosphorylase	2.4.2.3	Metabolism--Nucleotide metabolism--Pyrimidine metabolism|Metabolism--Xenobiotics biodegradation and metabolism--Drug metabolism - other enzymes|Metabolism--Global and overview maps--Metabolic pathways

[denovogenes]_10683	95.17	0.0	bfb:VU15_15295	K01277	DPP3	dipeptidyl-peptidase III	3.4.14.4	--

[denovogenes]_106830	82.95	5e-158	lacy:A4V08_27260	K18369	adh2	alcohol dehydrogenase	1.1.1.-	Metabolism--Carbohydrate metabolism--Propanoate metabolism

[denovogenes]_106835	77.43	1e-151	bprs:CK3_15820	K03426	E3.6.1.22, NUDT12, nudC	NAD+ diphosphatase	3.6.1.22	Metabolism--Metabolism of cofactors and vitamins--Nicotinate and nicotinamide metabolism|Metabolism--Global and overview maps--Metabolic pathways|Cellular Processes--Transport and catabolism--Peroxisome

[denovogenes]_106836	46.72	1e-70	tae:TepiRe1_2545	K03294	TC.APA	basic amino acid/polyamine antiporter, APA family	--	--

[denovogenes]_106837	96.93	0.0	fpla:A4U99_01140	K01803	TPI, tpiA	triosephosphate isomerase (TIM)	5.3.1.1	Metabolism--Carbohydrate metabolism--Glycolysis / Gluconeogenesis|Metabolism--Carbohydrate metabolism--Fructose and mannose metabolism|Metabolism--Carbohydrate metabolism--Inositol phosphate metabolism|Metabolism--Energy metabolism--Carbon fixation in photosynthetic organisms|Metabolism--Global and overview maps--Metabolic pathways|Metabolism--Global and overview maps--Biosynthesis of secondary metabolites|Metabolism--Global and overview maps--Microbial metabolism in diverse environments|Metabolism--Global and overview maps--Biosynthesis of antibiotics|Metabolism--Global and overview maps--Carbon metabolism|Metabolism--Global and overview maps--Biosynthesis of amino acids

[denovogenes]_106839	98.85	0.0	cfd:CFNIH1_12720	K14977	ylbA, UGHY	(S)-ureidoglycine aminohydrolase	3.5.3.26	Metabolism--Nucleotide metabolism--Purine metabolism|Metabolism--Global and overview maps--Microbial metabolism in diverse environments

[denovogenes]_10684	99.85	0.0	paeo:M801_1343	K01354	ptrB	oligopeptidase B	3.4.21.83	Human Diseases--Infectious diseases: Parasitic--Chagas disease (American trypanosomiasis)|Human Diseases--Infectious diseases: Parasitic--African trypanosomiasis

[denovogenes]_106841	90.00	2e-176	bcel:BcellWH2_00917	K01785	galM, GALM	aldose 1-epimerase	5.1.3.3	Metabolism--Carbohydrate metabolism--Glycolysis / Gluconeogenesis|Metabolism--Carbohydrate metabolism--Galactose metabolism|Metabolism--Global and overview maps--Metabolic pathways|Metabolism--Global and overview maps--Biosynthesis of secondary metabolites|Metabolism--Global and overview maps--Microbial metabolism in diverse environments|Metabolism--Global and overview maps--Biosynthesis of antibiotics

[denovogenes]_106842	96.92	0.0	cfd:CFNIH1_03670	K03424	--	--	--	--

[denovogenes]_106843	58.78	2e-106	bprs:CK3_29020	K02034	ABC.PE.P1	peptide/nickel transport system permease protein	--	Cellular Processes--Cellular community - prokaryotes--Quorum sensing

[denovogenes]_106845	85.38	2e-164	rho:RHOM_02780	K03521	fixA, etfB	electron transfer flavoprotein beta subunit	--	--

[denovogenes]_106846	100.00	0.0	bxy:BXY_48120	K01940	argG, ASS1	argininosuccinate synthase	6.3.4.5	Metabolism--Amino acid metabolism--Arginine biosynthesis|Metabolism--Amino acid metabolism--Alanine, aspartate and glutamate metabolism|Metabolism--Global and overview maps--Metabolic pathways|Metabolism--Global and overview maps--Biosynthesis of secondary metabolites|Metabolism--Global and overview maps--Biosynthesis of antibiotics|Metabolism--Global and overview maps--Biosynthesis of amino acids|Human Diseases--Cardiovascular diseases--Fluid shear stress and atherosclerosis

[denovogenes]_106849	81.54	2e-157	erb:A4V01_00985	K02510	hpaI, hpcH	4-hydroxy-2-oxoheptanedioate aldolase	4.1.2.52	Metabolism--Amino acid metabolism--Tyrosine metabolism|Metabolism--Global and overview maps--Microbial metabolism in diverse environments

[denovogenes]_106850	97.32	0.0	cfd:CFNIH1_05255	K00874	kdgK	2-dehydro-3-deoxygluconokinase	2.7.1.45	Metabolism--Carbohydrate metabolism--Pentose phosphate pathway|Metabolism--Global and overview maps--Metabolic pathways|Metabolism--Global and overview maps--Microbial metabolism in diverse environments|Metabolism--Global and overview maps--Carbon metabolism

[denovogenes]_106853	57.47	1e-113	cpy:Cphy_0432	K15581	oppB	oligopeptide transport system permease protein	--	Human Diseases--Drug resistance: Antimicrobial--beta-Lactam resistance|Environmental Information Processing--Membrane transport--ABC transporters|Cellular Processes--Cellular community - prokaryotes--Quorum sensing

[denovogenes]_106855	80.08	6e-157	bprm:CL3_17880	K00282	gcvPA	glycine dehydrogenase subunit 1	1.4.4.2	Metabolism--Amino acid metabolism--Glycine, serine and threonine metabolism|Metabolism--Carbohydrate metabolism--Glyoxylate and dicarboxylate metabolism|Metabolism--Global and overview maps--Metabolic pathways|Metabolism--Global and overview maps--Biosynthesis of secondary metabolites|Metabolism--Global and overview maps--Biosynthesis of antibiotics|Metabolism--Global and overview maps--Carbon metabolism

[denovogenes]_106856	100.00	0.0	cif:AL515_14040	K00873	PK, pyk	pyruvate kinase	2.7.1.40	Metabolism--Carbohydrate metabolism--Glycolysis / Gluconeogenesis|Metabolism--Nucleotide metabolism--Purine metabolism|Metabolism--Carbohydrate metabolism--Pyruvate metabolism|Metabolism--Global and overview maps--Metabolic pathways|Metabolism--Global and overview maps--Biosynthesis of secondary metabolites|Metabolism--Global and overview maps--Microbial metabolism in diverse environments|Metabolism--Global and overview maps--Biosynthesis of antibiotics|Metabolism--Global and overview maps--Carbon metabolism|Metabolism--Global and overview maps--Biosynthesis of amino acids|Organismal Systems--Endocrine system--Glucagon signaling pathway|Human Diseases--Endocrine and metabolic diseases--Type II diabetes mellitus|Human Diseases--Cancers: Overview--Viral carcinogenesis|Human Diseases--Cancers: Overview--Central carbon metabolism in cancer

[denovogenes]_106858	92.72	0.0	bfs:BF9343_0882	K07405	E3.2.1.1A	alpha-amylase	3.2.1.1	Metabolism--Carbohydrate metabolism--Starch and sucrose metabolism|Metabolism--Global and overview maps--Metabolic pathways

[denovogenes]_10686	53.89	0.0	csh:Closa_0089	K03406	mcp	methyl-accepting chemotaxis protein	--	Environmental Information Processing--Signal transduction--Two-component system|Cellular Processes--Cell motility--Bacterial chemotaxis

[denovogenes]_106861	49.74	5e-57	eha:Ethha_1410	K01448	E3.5.1.28B, amiA, amiB, amiC	N-acetylmuramoyl-L-alanine amidase	3.5.1.28	Human Diseases--Drug resistance: Antimicrobial--Cationic antimicrobial peptide (CAMP) resistance

[denovogenes]_106862	42.37	2e-63	rba:RB7481	K01134	ARSA	arylsulfatase A	3.1.6.8	Metabolism--Lipid metabolism--Sphingolipid metabolism|Cellular Processes--Transport and catabolism--Lysosome

[denovogenes]_106863	48.82	4e-79	dru:Desru_2311	K03500	rsmB, sun	16S rRNA (cytosine967-C5)-methyltransferase	2.1.1.176	--

[denovogenes]_106864	49.19	2e-71	ttm:Tthe_1360	K00945	cmk	CMP/dCMP kinase	2.7.4.25	Metabolism--Nucleotide metabolism--Pyrimidine metabolism|Metabolism--Global and overview maps--Metabolic pathways

[denovogenes]_106868	65.02	2e-100	aeq:AEQU_1674	K06164	phnI	alpha-D-ribose 1-methylphosphonate 5-triphosphate synthase subunit PhnI	2.7.8.37	Metabolism--Metabolism of other amino acids--Phosphonate and phosphinate metabolism

[denovogenes]_10687	78.92	0.0	csh:Closa_1960	K03655	recG	ATP-dependent DNA helicase RecG	3.6.4.12	Genetic Information Processing--Replication and repair--Homologous recombination

[denovogenes]_106874	44.12	6e-64	ppm:PPSC2_28075	K00527	nrdD	ribonucleoside-triphosphate reductase	1.17.4.2	Metabolism--Nucleotide metabolism--Purine metabolism|Metabolism--Nucleotide metabolism--Pyrimidine metabolism|Metabolism--Global and overview maps--Metabolic pathways

[denovogenes]_106876	57.83	1e-98	ctc:CTC_01941	K00557	trmA	tRNA (uracil-5-)-methyltransferase	2.1.1.35	--

[denovogenes]_106877	72.20	4e-132	ova:OBV_01450	K00625	E2.3.1.8, pta	phosphate acetyltransferase	2.3.1.8	Metabolism--Metabolism of other amino acids--Taurine and hypotaurine metabolism|Metabolism--Carbohydrate metabolism--Pyruvate metabolism|Metabolism--Carbohydrate metabolism--Propanoate metabolism|Metabolism--Energy metabolism--Methane metabolism|Metabolism--Energy metabolism--Carbon fixation pathways in prokaryotes|Metabolism--Global and overview maps--Metabolic pathways|Metabolism--Global and overview maps--Microbial metabolism in diverse environments|Metabolism--Global and overview maps--Carbon metabolism

[denovogenes]_106878	70.88	2e-139	puf:UFO1_4587	K00057	gpsA	glycerol-3-phosphate dehydrogenase (NAD(P)+)	1.1.1.94	Metabolism--Lipid metabolism--Glycerophospholipid metabolism|Metabolism--Global and overview maps--Biosynthesis of secondary metabolites

[denovogenes]_106879	89.66	2e-174	bhl:Bache_2986	K00817	hisC	histidinol-phosphate aminotransferase	2.6.1.9	Metabolism--Amino acid metabolism--Histidine metabolism|Metabolism--Amino acid metabolism--Tyrosine metabolism|Metabolism--Amino acid metabolism--Phenylalanine metabolism|Metabolism--Amino acid metabolism--Phenylalanine, tyrosine and tryptophan biosynthesis|Metabolism--Biosynthesis of other secondary metabolites--Novobiocin biosynthesis|Metabolism--Biosynthesis of other secondary metabolites--Tropane, piperidine and pyridine alkaloid biosynthesis|Metabolism--Global and overview maps--Metabolic pathways|Metabolism--Global and overview maps--Biosynthesis of secondary metabolites|Metabolism--Global and overview maps--Biosynthesis of antibiotics|Metabolism--Global and overview maps--Biosynthesis of amino acids

[denovogenes]_106880	100.00	0.0	cfd:CFNIH1_23515	K08483	PTS-EI.PTSI, ptsI	phosphotransferase system, enzyme I, PtsI	2.7.3.9	Environmental Information Processing--Membrane transport--Phosphotransferase system (PTS)

[denovogenes]_106883	83.40	3e-160	pdi:BDI_2852	K05306	phnX	phosphonoacetaldehyde hydrolase	3.11.1.1	Metabolism--Metabolism of other amino acids--Phosphonate and phosphinate metabolism|Metabolism--Global and overview maps--Metabolic pathways|Metabolism--Global and overview maps--Microbial metabolism in diverse environments

[denovogenes]_106886	99.62	0.0	paec:M802_5690	K02073	metQ	D-methionine transport system substrate-binding protein	--	Environmental Information Processing--Membrane transport--ABC transporters

[denovogenes]_106887	99.62	0.0	pdk:PADK2_29360	K00059	fabG	3-oxoacyl-[acyl-carrier protein] reductase	1.1.1.100	Metabolism--Lipid metabolism--Fatty acid biosynthesis|Metabolism--Metabolism of cofactors and vitamins--Biotin metabolism|Metabolism--Lipid metabolism--Biosynthesis of unsaturated fatty acids|Metabolism--Global and overview maps--Metabolic pathways|Metabolism--Global and overview maps--Fatty acid metabolism

[denovogenes]_106888	99.23	0.0	fpla:A4U99_02780	K01470	E3.5.2.10	creatinine amidohydrolase	3.5.2.10	Metabolism--Amino acid metabolism--Arginine and proline metabolism

[denovogenes]_106889	100.00	0.0	cfd:CFNIH1_10865	K02421	fliR	flagellar biosynthetic protein FliR	--	Cellular Processes--Cell motility--Flagellar assembly

[denovogenes]_106890	97.69	0.0	cfd:CFNIH1_10970	K02386	flgA	flagella basal body P-ring formation protein FlgA	--	Cellular Processes--Cell motility--Flagellar assembly

[denovogenes]_106892	100.00	0.0	fpla:A4U99_15780	K00059	fabG	3-oxoacyl-[acyl-carrier protein] reductase	1.1.1.100	Metabolism--Lipid metabolism--Fatty acid biosynthesis|Metabolism--Metabolism of cofactors and vitamins--Biotin metabolism|Metabolism--Lipid metabolism--Biosynthesis of unsaturated fatty acids|Metabolism--Global and overview maps--Metabolic pathways|Metabolism--Global and overview maps--Fatty acid metabolism

[denovogenes]_106893	100.00	0.0	paeo:M801_233	K01040	gctB	glutaconate CoA-transferase, subunit B	2.8.3.12	Metabolism--Xenobiotics biodegradation and metabolism--Styrene degradation|Metabolism--Carbohydrate metabolism--Butanoate metabolism|Metabolism--Global and overview maps--Microbial metabolism in diverse environments

[denovogenes]_106894	100.00	0.0	paec:M802_229	K01628	fucA	L-fuculose-phosphate aldolase	4.1.2.17	Metabolism--Carbohydrate metabolism--Fructose and mannose metabolism|Metabolism--Global and overview maps--Microbial metabolism in diverse environments

[denovogenes]_106895	43.10	1e-23	cgt:cgR_1918	K07741	antB	anti-repressor protein	--	--

[denovogenes]_10690	59.53	0.0	elm:ELI_2095	K11751	ushA	5'-nucleotidase / UDP-sugar diphosphatase	3.1.3.5 3.6.1.45	Metabolism--Nucleotide metabolism--Purine metabolism|Metabolism--Nucleotide metabolism--Pyrimidine metabolism|Metabolism--Metabolism of cofactors and vitamins--Nicotinate and nicotinamide metabolism|Metabolism--Global and overview maps--Metabolic pathways|Metabolism--Global and overview maps--Biosynthesis of secondary metabolites

[denovogenes]_106903	99.62	0.0	fpla:A4U99_15250	K09769	K09769	uncharacterized protein	--	--

[denovogenes]_106906	100.00	2e-179	cfd:CFNIH1_01400	K12943	ygeR	lipoprotein YgeR	--	--

[denovogenes]_106908	100.00	0.0	paeo:M801_4439	K00413	CYC1, CYT1, petC	ubiquinol-cytochrome c reductase cytochrome c1 subunit	--	Metabolism--Energy metabolism--Oxidative phosphorylation|Metabolism--Global and overview maps--Metabolic pathways|Environmental Information Processing--Signal transduction--Two-component system|Organismal Systems--Circulatory system--Cardiac muscle contraction|Human Diseases--Endocrine and metabolic diseases--Non-alcoholic fatty liver disease (NAFLD)|Human Diseases--Neurodegenerative diseases--Alzheimer's disease|Human Diseases--Neurodegenerative diseases--Parkinson's disease|Human Diseases--Neurodegenerative diseases--Huntington's disease

[denovogenes]_106909	68.99	1e-122	dvl:Dvul_2948	K13292	lgt, umpA	phosphatidylglycerol:prolipoprotein diacylglycerol transferase	2.-.-.-	--

[denovogenes]_10691	48.22	4e-97	fbt:D770_21865	K07214	fes	enterochelin esterase and related enzymes	--	--

[denovogenes]_106913	40.17	8e-63	ova:OBV_03160	K07010	K07010	putative glutamine amidotransferase	--	--

[denovogenes]_106915	100.00	3e-160	paeu:BN889_00870	K01114	plcC	phospholipase C	3.1.4.3	Metabolism--Carbohydrate metabolism--Inositol phosphate metabolism|Metabolism--Lipid metabolism--Glycerophospholipid metabolism|Metabolism--Lipid metabolism--Ether lipid metabolism|Metabolism--Global and overview maps--Metabolic pathways|Metabolism--Global and overview maps--Biosynthesis of secondary metabolites|Cellular Processes--Cellular community - prokaryotes--Quorum sensing|Organismal Systems--Endocrine system--Thyroid hormone signaling pathway

[denovogenes]_106917	59.52	5e-91	bcel:BcellWH2_01990	K08717	utp	urea transporter	--	--

[denovogenes]_106918	40.40	7e-29	rmn:TK49_17095	K06989	nadX	aspartate dehydrogenase	1.4.1.21	Metabolism--Metabolism of cofactors and vitamins--Nicotinate and nicotinamide metabolism|Metabolism--Global and overview maps--Metabolic pathways

[denovogenes]_106919	41.71	4e-40	exm:U719_15585	K00019	E1.1.1.30, bdh	3-hydroxybutyrate dehydrogenase	1.1.1.30	Metabolism--Lipid metabolism--Synthesis and degradation of ketone bodies|Metabolism--Carbohydrate metabolism--Butanoate metabolism|Metabolism--Global and overview maps--Metabolic pathways

[denovogenes]_106920	100.00	0.0	paec:M802_3349	K04754	mlaA, vacJ	phospholipid-binding lipoprotein MlaA	--	--

[denovogenes]_106921	55.34	1e-99	eha:Ethha_1446	K00598	tam	trans-aconitate 2-methyltransferase	2.1.1.144	--

[denovogenes]_106922	57.65	6e-101	lir:LAW_00015	K09761	rsmE	16S rRNA (uracil1498-N3)-methyltransferase	2.1.1.193	--

[denovogenes]_106923	60.78	5e-111	elo:EC042_0332	K06221	dkgA	2,5-diketo-D-gluconate reductase A	1.1.1.346	--

[denovogenes]_106924	100.00	2e-175	pdk:PADK2_05455	K07090	K07090	uncharacterized protein	--	--

[denovogenes]_106925	96.92	0.0	lacy:A4V08_22670	K02049	ABC.SN.A	NitT/TauT family transport system ATP-binding protein	--	--

[denovogenes]_106928	100.00	2e-161	mmk:MU9_1019	K02302	cysG	uroporphyrin-III C-methyltransferase / precorrin-2 dehydrogenase / sirohydrochlorin ferrochelatase	2.1.1.107 1.3.1.76 4.99.1.4	Metabolism--Metabolism of cofactors and vitamins--Porphyrin and chlorophyll metabolism|Metabolism--Global and overview maps--Metabolic pathways|Metabolism--Global and overview maps--Biosynthesis of secondary metabolites

[denovogenes]_106929	96.93	0.0	bfb:VU15_03330	K16053	mscM	miniconductance mechanosensitive channel	--	--

[denovogenes]_10693	45.66	0.0	cct:CC1_19330	K02355	fusA, GFM, EFG	elongation factor G	--	--

[denovogenes]_106930	91.54	1e-179	cama:F384_00130	K06859	pgi1	glucose-6-phosphate isomerase, archaeal	5.3.1.9	Metabolism--Carbohydrate metabolism--Glycolysis / Gluconeogenesis|Metabolism--Carbohydrate metabolism--Pentose phosphate pathway|Metabolism--Carbohydrate metabolism--Starch and sucrose metabolism|Metabolism--Carbohydrate metabolism--Amino sugar and nucleotide sugar metabolism|Metabolism--Global and overview maps--Metabolic pathways|Metabolism--Global and overview maps--Biosynthesis of secondary metabolites|Metabolism--Global and overview maps--Microbial metabolism in diverse environments|Metabolism--Global and overview maps--Biosynthesis of antibiotics|Metabolism--Global and overview maps--Carbon metabolism

[denovogenes]_106931	43.70	4e-46	pse:NH8B_4087	K00874	kdgK	2-dehydro-3-deoxygluconokinase	2.7.1.45	Metabolism--Carbohydrate metabolism--Pentose phosphate pathway|Metabolism--Global and overview maps--Metabolic pathways|Metabolism--Global and overview maps--Microbial metabolism in diverse environments|Metabolism--Global and overview maps--Carbon metabolism

[denovogenes]_106934	100.00	0.0	eck:EC55989_4935	K11534	deoR	DeoR family transcriptional regulator, deoxyribose operon repressor	--	--

[denovogenes]_106935	72.94	1e-139	elm:ELI_2935	K02510	hpaI, hpcH	4-hydroxy-2-oxoheptanedioate aldolase	4.1.2.52	Metabolism--Amino acid metabolism--Tyrosine metabolism|Metabolism--Global and overview maps--Microbial metabolism in diverse environments

[denovogenes]_106938	47.67	1e-81	pln:Plano_2882	K00525	E1.17.4.1A, nrdA, nrdE	ribonucleoside-diphosphate reductase alpha chain	1.17.4.1	Metabolism--Nucleotide metabolism--Purine metabolism|Metabolism--Nucleotide metabolism--Pyrimidine metabolism|Metabolism--Global and overview maps--Metabolic pathways

[denovogenes]_106940	65.64	3e-123	vpe:Varpa_1438	K18333	K18333	L-fucose dehydrogenase	--	Metabolism--Carbohydrate metabolism--Fructose and mannose metabolism|Metabolism--Global and overview maps--Microbial metabolism in diverse environments

[denovogenes]_106941	99.23	0.0	cfd:CFNIH1_00890	K06175	truC	tRNA pseudouridine65 synthase	5.4.99.26	--

[denovogenes]_106942	54.26	7e-96	tae:TepiRe1_1939	K00046	idnO	gluconate 5-dehydrogenase	1.1.1.69	--

[denovogenes]_106943	59.76	4e-106	bth:BT_3587	K02564	nagB, GNPDA	glucosamine-6-phosphate deaminase	3.5.99.6	Metabolism--Carbohydrate metabolism--Amino sugar and nucleotide sugar metabolism|Metabolism--Global and overview maps--Metabolic pathways

[denovogenes]_106944	100.00	0.0	paeg:AI22_01280	K04748	norQ	nitric oxide reductase NorQ protein	--	--

[denovogenes]_106946	84.25	2e-165	csh:Closa_1681	K00757	udp, UPP	uridine phosphorylase	2.4.2.3	Metabolism--Nucleotide metabolism--Pyrimidine metabolism|Metabolism--Xenobiotics biodegradation and metabolism--Drug metabolism - other enzymes|Metabolism--Global and overview maps--Metabolic pathways

[denovogenes]_106947	100.00	0.0	paeo:M801_2700	K01265	map	methionyl aminopeptidase	3.4.11.18	--

[denovogenes]_106948	100.00	0.0	cfd:CFNIH1_23100	K10013	ABC.ARG.S, argT	lysine/arginine/ornithine transport system substrate-binding protein	--	Environmental Information Processing--Membrane transport--ABC transporters

[denovogenes]_106949	100.00	0.0	cif:AL515_15990	K10014	hisJ	histidine transport system substrate-binding protein	--	Environmental Information Processing--Membrane transport--ABC transporters

[denovogenes]_106953	41.31	7e-69	tme:Tmel_0509	K02805	wecE, rffA	dTDP-4-amino-4,6-dideoxygalactose transaminase	2.6.1.59	--

[denovogenes]_106956	85.77	6e-161	bprs:CK3_07460	K02053	ABC.SP.P	putative spermidine/putrescine transport system permease protein	--	Cellular Processes--Cellular community - prokaryotes--Quorum sensing

[denovogenes]_106959	54.86	2e-93	bha:BH3444	K07497	K07497	putative transposase	--	--

[denovogenes]_10696	85.21	0.0	vpr:Vpar_0976	K01879	glyS	glycyl-tRNA synthetase beta chain	6.1.1.14	Genetic Information Processing--Translation--Aminoacyl-tRNA biosynthesis

[denovogenes]_106962	40.71	8e-19	lpil:LIP_0400	K03710	K03710	GntR family transcriptional regulator	--	--

[denovogenes]_106963	99.62	0.0	esl:O3K_24660	K03424	--	--	--	--

[denovogenes]_106969	95.37	5e-159	lacy:A4V08_05215	K08974	K08974	putative membrane protein	--	--

[denovogenes]_10697	100.00	0.0	mmk:MU9_3394	K01547	kdpB	K+-transporting ATPase ATPase B chain	3.6.3.12	Environmental Information Processing--Signal transduction--Two-component system

[denovogenes]_106971	96.54	0.0	lacy:A4V08_26080	K01448	E3.5.1.28B, amiA, amiB, amiC	N-acetylmuramoyl-L-alanine amidase	3.5.1.28	Human Diseases--Drug resistance: Antimicrobial--Cationic antimicrobial peptide (CAMP) resistance

[denovogenes]_106972	68.40	2e-122	cpy:Cphy_0114	K09697	natA	sodium transport system ATP-binding protein	3.6.3.7	Environmental Information Processing--Membrane transport--ABC transporters|Environmental Information Processing--Signal transduction--Two-component system

[denovogenes]_106973	59.16	3e-103	toh:BCB71_11450	K03332	fruA	fructan beta-fructosidase	3.2.1.80	Metabolism--Carbohydrate metabolism--Fructose and mannose metabolism

[denovogenes]_106974	62.55	2e-114	ddt:AAY81_04480	K01992	ABC-2.P	ABC-2 type transport system permease protein	--	--

[denovogenes]_106976	100.00	0.0	sbc:SbBS512_E0642	K00239	sdhA, frdA	succinate dehydrogenase / fumarate reductase, flavoprotein subunit	1.3.5.1 1.3.5.4	Metabolism--Carbohydrate metabolism--Citrate cycle (TCA cycle)|Metabolism--Energy metabolism--Oxidative phosphorylation|Metabolism--Carbohydrate metabolism--Butanoate metabolism|Metabolism--Energy metabolism--Carbon fixation pathways in prokaryotes|Metabolism--Global and overview maps--Metabolic pathways|Metabolism--Global and overview maps--Biosynthesis of secondary metabolites|Metabolism--Global and overview maps--Microbial metabolism in diverse environments|Metabolism--Global and overview maps--Biosynthesis of antibiotics|Metabolism--Global and overview maps--Carbon metabolism|Human Diseases--Infectious diseases: Bacterial--Legionellosis

[denovogenes]_106978	52.46	5e-81	cls:CXIVA_15370	K00548	metH, MTR	5-methyltetrahydrofolate--homocysteine methyltransferase	2.1.1.13	Metabolism--Amino acid metabolism--Cysteine and methionine metabolism|Metabolism--Metabolism of other amino acids--Selenocompound metabolism|Metabolism--Metabolism of cofactors and vitamins--One carbon pool by folate|Metabolism--Global and overview maps--Metabolic pathways|Metabolism--Global and overview maps--Biosynthesis of secondary metabolites|Metabolism--Global and overview maps--Biosynthesis of amino acids

[denovogenes]_106979	75.57	4e-138	lacy:A4V08_31190	K02392	flgG	flagellar basal-body rod protein FlgG	--	Cellular Processes--Cell motility--Flagellar assembly

[denovogenes]_106980	56.86	5e-94	bdh:GV66_14615	K01991	ABC-2.OM, wza	polysaccharide biosynthesis/export protein	--	Cellular Processes--Cellular community - prokaryotes--Biofilm formation - Escherichia coli

[denovogenes]_106982	58.24	2e-110	btho:Btheta7330_03596	K10563	mutM, fpg	formamidopyrimidine-DNA glycosylase	3.2.2.23 4.2.99.18	Genetic Information Processing--Replication and repair--Base excision repair

[denovogenes]_106983	99.62	0.0	bbrc:B7019_0179	K08659	pepDA, pepDB	dipeptidase	3.4.-.-	--

[denovogenes]_106987	40.91	5e-49	bbp:BBPR_0016	K07317	K07317	adenine-specific DNA-methyltransferase	2.1.1.72	--

[denovogenes]_106989	47.35	2e-60	afd:Alfi_3061	K03760	eptA	lipid A ethanolamine phosphotransferase	2.7.8.43	Human Diseases--Drug resistance: Antimicrobial--Cationic antimicrobial peptide (CAMP) resistance

[denovogenes]_106991	99.62	0.0	cfd:CFNIH1_03670	K03424	--	--	--	--

[denovogenes]_106993	67.69	4e-119	elm:ELI_2735	K00052	leuB	3-isopropylmalate dehydrogenase	1.1.1.85	Metabolism--Amino acid metabolism--Valine, leucine and isoleucine biosynthesis|Metabolism--Carbohydrate metabolism--C5-Branched dibasic acid metabolism|Metabolism--Global and overview maps--Metabolic pathways|Metabolism--Global and overview maps--Biosynthesis of secondary metabolites|Metabolism--Global and overview maps--2-Oxocarboxylic acid metabolism|Metabolism--Global and overview maps--Biosynthesis of amino acids

[denovogenes]_106998	41.06	1e-56	cck:Ccar_13150	K01599	hemE, UROD	uroporphyrinogen decarboxylase	4.1.1.37	Metabolism--Metabolism of cofactors and vitamins--Porphyrin and chlorophyll metabolism|Metabolism--Global and overview maps--Metabolic pathways|Metabolism--Global and overview maps--Biosynthesis of secondary metabolites

[denovogenes]_106999	95.40	0.0	lacy:A4V08_34550	K00554	trmD	tRNA (guanine37-N1)-methyltransferase	2.1.1.228	--

[denovogenes]_1070	69.18	0.0	cpe:CPE0360	K02337	DPO3A1, dnaE	DNA polymerase III subunit alpha	2.7.7.7	Metabolism--Nucleotide metabolism--Purine metabolism|Metabolism--Nucleotide metabolism--Pyrimidine metabolism|Metabolism--Global and overview maps--Metabolic pathways|Genetic Information Processing--Replication and repair--DNA replication|Genetic Information Processing--Replication and repair--Mismatch repair|Genetic Information Processing--Replication and repair--Homologous recombination

[denovogenes]_10700	100.00	0.0	bfb:VU15_02065	K01547	kdpB	K+-transporting ATPase ATPase B chain	3.6.3.12	Environmental Information Processing--Signal transduction--Two-component system

[denovogenes]_107002	99.62	0.0	cfd:CFNIH1_05660	K00057	gpsA	glycerol-3-phosphate dehydrogenase (NAD(P)+)	1.1.1.94	Metabolism--Lipid metabolism--Glycerophospholipid metabolism|Metabolism--Global and overview maps--Biosynthesis of secondary metabolites

[denovogenes]_107003	67.18	3e-129	rix:RO1_33020	K03496	parA, soj	chromosome partitioning protein	--	--

[denovogenes]_107008	93.85	3e-175	bfb:VU15_18840	K00602	purH	phosphoribosylaminoimidazolecarboxamide formyltransferase / IMP cyclohydrolase	2.1.2.3 3.5.4.10	Metabolism--Nucleotide metabolism--Purine metabolism|Metabolism--Metabolism of cofactors and vitamins--One carbon pool by folate|Metabolism--Global and overview maps--Metabolic pathways|Metabolism--Global and overview maps--Biosynthesis of secondary metabolites|Metabolism--Global and overview maps--Biosynthesis of antibiotics|Human Diseases--Drug resistance: Antineoplastic--Antifolate resistance

[denovogenes]_10701	80.56	0.0	lacy:A4V08_34765	K03655	recG	ATP-dependent DNA helicase RecG	3.6.4.12	Genetic Information Processing--Replication and repair--Homologous recombination

[denovogenes]_107010	72.44	3e-121	shi:Shel_12880	K03575	mutY	A/G-specific adenine glycosylase	3.2.2.-	Genetic Information Processing--Replication and repair--Base excision repair

[denovogenes]_107014	98.85	0.0	pdi:BDI_1250	K00844	HK	hexokinase	2.7.1.1	Metabolism--Carbohydrate metabolism--Glycolysis / Gluconeogenesis|Metabolism--Carbohydrate metabolism--Fructose and mannose metabolism|Metabolism--Carbohydrate metabolism--Galactose metabolism|Metabolism--Carbohydrate metabolism--Starch and sucrose metabolism|Metabolism--Carbohydrate metabolism--Amino sugar and nucleotide sugar metabolism|Metabolism--Biosynthesis of other secondary metabolites--Streptomycin biosynthesis|Metabolism--Biosynthesis of other secondary metabolites--Neomycin, kanamycin and gentamicin biosynthesis|Metabolism--Global and overview maps--Metabolic pathways|Metabolism--Global and overview maps--Biosynthesis of secondary metabolites|Metabolism--Global and overview maps--Microbial metabolism in diverse environments|Metabolism--Global and overview maps--Biosynthesis of antibiotics|Metabolism--Global and overview maps--Carbon metabolism|Environmental Information Processing--Signal transduction--HIF-1 signaling pathway|Organismal Systems--Endocrine system--Insulin signaling pathway|Human Diseases--Endocrine and metabolic diseases--Type II diabetes mellitus|Organismal Systems--Digestive system--Carbohydrate digestion and absorption|Human Diseases--Cancers: Overview--Central carbon metabolism in cancer

[denovogenes]_107015	40.28	6e-44	mmy:MSC_0108	K00786	E2.4.-.-	--	--	--

[denovogenes]_107016	72.20	3e-132	euu:ADJ67_02215	K03431	glmM	phosphoglucosamine mutase	5.4.2.10	Metabolism--Carbohydrate metabolism--Amino sugar and nucleotide sugar metabolism|Metabolism--Global and overview maps--Metabolic pathways|Metabolism--Global and overview maps--Biosynthesis of antibiotics

[denovogenes]_107017	97.67	0.0	cfd:CFNIH1_05635	K14348	lldR	GntR family transcriptional regulator, L-lactate dehydrogenase operon regulator	--	--

[denovogenes]_107018	46.77	9e-75	cpra:CPter91_1817	K07497	K07497	putative transposase	--	--

[denovogenes]_10702	98.97	0.0	mmk:MU9_1355	K08344	scsB	suppressor for copper-sensitivity B	--	--

[denovogenes]_107020	41.83	1e-64	csq:CSCA_0667	K02016	ABC.FEV.S	iron complex transport system substrate-binding protein	--	Environmental Information Processing--Membrane transport--ABC transporters

[denovogenes]_107021	46.67	4e-27	cif:AL515_20315	K03931	ygjK	putative isomerase	--	--

[denovogenes]_107024	80.69	1e-151	boa:Bovatus_04937	K01911	menE	O-succinylbenzoic acid--CoA ligase	6.2.1.26	Metabolism--Metabolism of cofactors and vitamins--Ubiquinone and other terpenoid-quinone biosynthesis|Metabolism--Global and overview maps--Metabolic pathways|Metabolism--Global and overview maps--Biosynthesis of secondary metabolites

[denovogenes]_107025	98.08	0.0	pvl:AOB99_12160	K03590	ftsA	cell division protein FtsA	--	Cellular Processes--Cell growth and death--Cell cycle - Caulobacter

[denovogenes]_107026	45.87	3e-59	bfb:VU15_03420	K07011	K07011	uncharacterized protein	--	--

[denovogenes]_107027	88.84	2e-151	sot:102577524	K11294	NCL, NSR1	nucleolin	--	Human Diseases--Infectious diseases: Bacterial--Pathogenic Escherichia coli infection

[denovogenes]_107028	100.00	0.0	ecy:ECSE_1279	K00371	narH, narY, nxrB	nitrate reductase / nitrite oxidoreductase, beta subunit	1.7.5.1 1.7.99.4	Metabolism--Energy metabolism--Nitrogen metabolism|Metabolism--Global and overview maps--Microbial metabolism in diverse environments|Environmental Information Processing--Signal transduction--Two-component system

[denovogenes]_10703	48.57	0.0	bql:LL3_03375	K02334	dpo	DNA polymerase bacteriophage-type	2.7.7.7	--

[denovogenes]_107032	49.39	1e-74	stk:STP_1111	K04759	feoB	ferrous iron transport protein B	--	--

[denovogenes]_107034	99.62	0.0	cfd:CFNIH1_23790	K07287	bamC	outer membrane protein assembly factor BamC	--	--

[denovogenes]_107038	98.85	0.0	pdf:CD630DERM_02930	K01990	ABC-2.A	ABC-2 type transport system ATP-binding protein	--	--

[denovogenes]_10704	98.53	0.0	cif:AL515_13905	K03797	E3.4.21.102, prc, ctpA	carboxyl-terminal processing protease	3.4.21.102	--

[denovogenes]_107042	56.18	5e-99	euu:ADJ67_06295	K01439	dapE	succinyl-diaminopimelate desuccinylase	3.5.1.18	Metabolism--Amino acid metabolism--Lysine biosynthesis|Metabolism--Global and overview maps--Metabolic pathways|Metabolism--Global and overview maps--Microbial metabolism in diverse environments|Metabolism--Global and overview maps--Biosynthesis of amino acids

[denovogenes]_107043	88.80	2e-172	rto:RTO_19670	K02837	prfC	peptide chain release factor 3	--	--

[denovogenes]_107046	86.97	3e-169	boa:Bovatus_00803	K00041	uxaB	tagaturonate reductase	1.1.1.58	Metabolism--Carbohydrate metabolism--Pentose and glucuronate interconversions|Metabolism--Global and overview maps--Metabolic pathways

[denovogenes]_107048	99.62	0.0	cdl:CDR20291_3363	K02012	afuA, fbpA	iron(III) transport system substrate-binding protein	--	Environmental Information Processing--Membrane transport--ABC transporters

[denovogenes]_10705	92.52	0.0	bcel:BcellWH2_02015	K01547	kdpB	K+-transporting ATPase ATPase B chain	3.6.3.12	Environmental Information Processing--Signal transduction--Two-component system

[denovogenes]_107050	59.85	3e-105	blr:BRLA_c022480	K02381	fdrA	FdrA protein	--	--

[denovogenes]_107054	46.04	5e-75	bado:BBMN23_0470	K05349	bglX	beta-glucosidase	3.2.1.21	Metabolism--Metabolism of other amino acids--Cyanoamino acid metabolism|Metabolism--Carbohydrate metabolism--Starch and sucrose metabolism|Metabolism--Biosynthesis of other secondary metabolites--Phenylpropanoid biosynthesis|Metabolism--Global and overview maps--Metabolic pathways|Metabolism--Global and overview maps--Biosynthesis of secondary metabolites

[denovogenes]_107055	78.85	7e-149	aeq:AEQU_0570	K00963	UGP2, galU, galF	UTP--glucose-1-phosphate uridylyltransferase	2.7.7.9	Metabolism--Carbohydrate metabolism--Pentose and glucuronate interconversions|Metabolism--Carbohydrate metabolism--Galactose metabolism|Metabolism--Carbohydrate metabolism--Starch and sucrose metabolism|Metabolism--Carbohydrate metabolism--Amino sugar and nucleotide sugar metabolism|Metabolism--Global and overview maps--Metabolic pathways|Metabolism--Global and overview maps--Biosynthesis of antibiotics

[denovogenes]_107056	90.04	2e-170	shi:Shel_07970	K01571	oadA	oxaloacetate decarboxylase, alpha subunit	4.1.1.3	Metabolism--Carbohydrate metabolism--Pyruvate metabolism|Metabolism--Global and overview maps--Metabolic pathways

[denovogenes]_107059	99.23	0.0	eld:i02_1964	K01484	astB	succinylarginine dihydrolase	3.5.3.23	Metabolism--Amino acid metabolism--Arginine and proline metabolism|Metabolism--Global and overview maps--Metabolic pathways

[denovogenes]_107061	49.49	2e-57	bcel:BcellWH2_04814	K06076	fadL	long-chain fatty acid transport protein	--	--

[denovogenes]_107064	51.54	2e-85	ctx:Clo1313_0111	K13542	cobA-hemD	uroporphyrinogen III methyltransferase / synthase	2.1.1.107 4.2.1.75	Metabolism--Metabolism of cofactors and vitamins--Porphyrin and chlorophyll metabolism|Metabolism--Global and overview maps--Metabolic pathways|Metabolism--Global and overview maps--Biosynthesis of secondary metabolites

[denovogenes]_107065	77.01	1e-142	mmic:RN08_1813	K03701	uvrA	excinuclease ABC subunit A	--	Genetic Information Processing--Replication and repair--Nucleotide excision repair

[denovogenes]_107067	99.23	0.0	pvl:AOB99_09275	K10823	oppF	oligopeptide transport system ATP-binding protein	--	Human Diseases--Drug resistance: Antimicrobial--beta-Lactam resistance|Environmental Information Processing--Membrane transport--ABC transporters|Cellular Processes--Cellular community - prokaryotes--Quorum sensing

[denovogenes]_107070	88.12	2e-163	ag:AAA20116	K18214	tetP_A, tet40	MFS transporter, DHA3 family, tetracycline resistance protein	--	--

[denovogenes]_107071	83.91	1e-164	erb:A4V01_20840	K06023	hprK, ptsK	HPr kinase/phosphorylase	2.7.11.- 2.7.4.-	--

[denovogenes]_107072	100.00	0.0	ena:ECNA114_39121	K01754	E4.3.1.19, ilvA, tdcB	threonine dehydratase	4.3.1.19	Metabolism--Amino acid metabolism--Glycine, serine and threonine metabolism|Metabolism--Amino acid metabolism--Valine, leucine and isoleucine biosynthesis|Metabolism--Global and overview maps--Metabolic pathways|Metabolism--Global and overview maps--Biosynthesis of secondary metabolites|Metabolism--Global and overview maps--Biosynthesis of antibiotics|Metabolism--Global and overview maps--Carbon metabolism|Metabolism--Global and overview maps--Biosynthesis of amino acids

[denovogenes]_107073	70.88	2e-123	rum:CK1_32730	K15987	hppA	K(+)-stimulated pyrophosphate-energized sodium pump	3.6.1.1	Metabolism--Energy metabolism--Oxidative phosphorylation

[denovogenes]_107074	89.27	1e-174	bcel:BcellWH2_03642	K05970	SIAE	sialate O-acetylesterase	3.1.1.53	--

[denovogenes]_10708	90.91	0.0	bhl:Bache_2294	K04079	HSP90A, htpG	molecular chaperone HtpG	--	Genetic Information Processing--Folding, sorting and degradation--Protein processing in endoplasmic reticulum|Environmental Information Processing--Signal transduction--PI3K-Akt signaling pathway|Organismal Systems--Immune system--Antigen processing and presentation|Organismal Systems--Immune system--NOD-like receptor signaling pathway|Organismal Systems--Environmental adaptation--Plant-pathogen interaction|Organismal Systems--Immune system--IL-17 signaling pathway|Organismal Systems--Immune system--Th17 cell differentiation|Organismal Systems--Endocrine system--Progesterone-mediated oocyte maturation|Organismal Systems--Endocrine system--Estrogen signaling pathway|Human Diseases--Cancers: Overview--Pathways in cancer|Human Diseases--Cancers: Specific types--Prostate cancer|Human Diseases--Cardiovascular diseases--Fluid shear stress and atherosclerosis

[denovogenes]_107089	42.25	2e-34	ruk:A4V00_16655	K00981	E2.7.7.41, CDS1, CDS2, cdsA	phosphatidate cytidylyltransferase	2.7.7.41	Metabolism--Lipid metabolism--Glycerophospholipid metabolism|Metabolism--Global and overview maps--Metabolic pathways|Metabolism--Global and overview maps--Biosynthesis of secondary metabolites|Environmental Information Processing--Signal transduction--Phosphatidylinositol signaling system

[denovogenes]_10709	50.35	0.0	bfb:VU15_10885	K16089	TC.FEV.OM2, cirA, cfrA, hmuR	outer membrane receptor for ferrienterochelin and colicins	--	--

[denovogenes]_107091	98.85	0.0	boa:Bovatus_01220	K01609	trpC	indole-3-glycerol phosphate synthase	4.1.1.48	Metabolism--Amino acid metabolism--Phenylalanine, tyrosine and tryptophan biosynthesis|Metabolism--Global and overview maps--Metabolic pathways|Metabolism--Global and overview maps--Biosynthesis of secondary metabolites|Metabolism--Global and overview maps--Biosynthesis of antibiotics|Metabolism--Global and overview maps--Biosynthesis of amino acids

[denovogenes]_107092	53.67	2e-74	pary:A4V02_08670	K10716	kch, trkA, mthK, pch	voltage-gated potassium channel	--	--

[denovogenes]_107094	100.00	0.0	ecm:EcSMS35_4865	K01816	hyi, gip	hydroxypyruvate isomerase	5.3.1.22	Metabolism--Carbohydrate metabolism--Glyoxylate and dicarboxylate metabolism|Metabolism--Global and overview maps--Metabolic pathways

[denovogenes]_107095	42.59	2e-66	ptl:AOT13_07460	K00858	ppnK, NADK	NAD+ kinase	2.7.1.23	Metabolism--Metabolism of cofactors and vitamins--Nicotinate and nicotinamide metabolism|Metabolism--Global and overview maps--Metabolic pathways

[denovogenes]_107098	47.76	1e-80	erb:A4V01_02320	K02003	ABC.CD.A	putative ABC transport system ATP-binding protein	--	--

[denovogenes]_10710	89.59	0.0	bhl:Bache_3162	K01277	DPP3	dipeptidyl-peptidase III	3.4.14.4	--

[denovogenes]_107100	63.10	1e-113	erb:A4V01_02320	K02003	ABC.CD.A	putative ABC transport system ATP-binding protein	--	--

[denovogenes]_107103	56.69	1e-101	rbn:RBXJA2T_17586	K00656	E2.3.1.54, pflD	formate C-acetyltransferase	2.3.1.54	Metabolism--Carbohydrate metabolism--Pyruvate metabolism|Metabolism--Carbohydrate metabolism--Propanoate metabolism|Metabolism--Carbohydrate metabolism--Butanoate metabolism|Metabolism--Global and overview maps--Metabolic pathways

[denovogenes]_107107	100.00	0.0	pvl:AOB99_13410	K06175	truC	tRNA pseudouridine65 synthase	5.4.99.26	--

[denovogenes]_107108	100.00	0.0	pmib:BB2000_0354	K01918	panC	pantoate--beta-alanine ligase	6.3.2.1	Metabolism--Metabolism of other amino acids--beta-Alanine metabolism|Metabolism--Metabolism of cofactors and vitamins--Pantothenate and CoA biosynthesis|Metabolism--Global and overview maps--Metabolic pathways|Metabolism--Global and overview maps--Biosynthesis of secondary metabolites

[denovogenes]_107110	71.54	7e-132	vpr:Vpar_1110	K03589	ftsQ	cell division protein FtsQ	--	Cellular Processes--Cell growth and death--Cell cycle - Caulobacter

[denovogenes]_107112	82.24	2e-152	vpr:Vpar_0358	K02030	ABC.PA.S	polar amino acid transport system substrate-binding protein	--	--

[denovogenes]_107113	50.97	2e-83	ccl:Clocl_0049	K07114	yfbK	Ca-activated chloride channel homolog	--	--

[denovogenes]_107115	70.92	7e-124	fnc:HMPREF0946_00138	K02073	metQ	D-methionine transport system substrate-binding protein	--	Environmental Information Processing--Membrane transport--ABC transporters

[denovogenes]_107116	100.00	0.0	pvl:AOB99_14235	K12144	hyfI	hydrogenase-4 component I	1.-.-.-	--

[denovogenes]_107118	47.43	1e-79	jeo:JMA_22440	K06223	dam	DNA adenine methylase	2.1.1.72	Genetic Information Processing--Replication and repair--Mismatch repair

[denovogenes]_10712	94.43	0.0	bxy:BXY_14930	K01277	DPP3	dipeptidyl-peptidase III	3.4.14.4	--

[denovogenes]_107120	70.93	1e-132	fnu:FN0926	K00951	relA	GTP pyrophosphokinase	2.7.6.5	Metabolism--Nucleotide metabolism--Purine metabolism

[denovogenes]_107121	100.00	0.0	cpf:CPF_1937	K03177	truB, PUS4, TRUB1	tRNA pseudouridine55 synthase	5.4.99.25	--

[denovogenes]_107122	99.62	0.0	eln:NRG857_17165	K09811	ftsX	cell division transport system permease protein	--	Environmental Information Processing--Membrane transport--ABC transporters

[denovogenes]_107123	62.16	3e-114	btho:Btheta7330_02010	K16936	doxA	thiosulfate dehydrogenase [quinone] small subunit	1.8.5.2	Metabolism--Energy metabolism--Sulfur metabolism|Metabolism--Global and overview maps--Microbial metabolism in diverse environments

[denovogenes]_107123	62.16	3e-114	btho:Btheta7330_02010	K16937	doxD	thiosulfate dehydrogenase [quinone] large subunit	1.8.5.2	Metabolism--Energy metabolism--Sulfur metabolism|Metabolism--Global and overview maps--Microbial metabolism in diverse environments

[denovogenes]_107127	72.03	3e-123	ipo:Ilyop_1383	K18682	rny	ribonucrease Y	3.1.-.-	Genetic Information Processing--Folding, sorting and degradation--RNA degradation

[denovogenes]_107129	100.00	0.0	edj:ECDH1ME8569_0158	K07098	K07098	uncharacterized protein	--	--

[denovogenes]_107130	98.47	0.0	boa:Bovatus_01581	K08301	rng, cafA	ribonuclease G	3.1.26.-	--

[denovogenes]_107134	97.32	0.0	spen:107004561	K09503	DNAJA2	DnaJ homolog subfamily A member 2	--	Genetic Information Processing--Folding, sorting and degradation--Protein processing in endoplasmic reticulum

[denovogenes]_107136	44.35	2e-62	bvl:BF3285c1_0388	K18138	acrB, mexB, adeJ, smeE, mtrD, cmeB	multidrug efflux pump	--	Human Diseases--Drug resistance: Antimicrobial--beta-Lactam resistance|Human Diseases--Drug resistance: Antimicrobial--Cationic antimicrobial peptide (CAMP) resistance

[denovogenes]_107137	100.00	0.0	mmk:MU9_1373	K01175	ybfF	esterase	3.1.-.-	--

[denovogenes]_107139	99.22	0.0	mmk:MU9_1416	K02020	modA	molybdate transport system substrate-binding protein	--	Environmental Information Processing--Membrane transport--ABC transporters

[denovogenes]_107140	100.00	0.0	mmk:MU9_1483	K02500	hisF	cyclase	4.1.3.-	Metabolism--Amino acid metabolism--Histidine metabolism|Metabolism--Global and overview maps--Metabolic pathways|Metabolism--Global and overview maps--Biosynthesis of secondary metabolites|Metabolism--Global and overview maps--Biosynthesis of amino acids

[denovogenes]_107141	99.61	0.0	mmk:MU9_1621	K01470	E3.5.2.10	creatinine amidohydrolase	3.5.2.10	Metabolism--Amino acid metabolism--Arginine and proline metabolism

[denovogenes]_107142	44.90	1e-64	etd:ETAF_2037	K12519	papD	chaperone protein PapD	--	--

[denovogenes]_107143	62.45	1e-113	pdt:Prede_2711	K19286	nfrA2	FMN reductase [NAD(P)H]	1.5.1.39	Metabolism--Metabolism of cofactors and vitamins--Riboflavin metabolism|Metabolism--Global and overview maps--Metabolic pathways

[denovogenes]_107144	99.61	0.0	bfs:BF9343_3562	K02477	K02477	two-component system, LytT family, response regulator	--	--

[denovogenes]_107145	98.46	0.0	bfg:BF638R_3765	K07257	spsF	spore coat polysaccharide biosynthesis protein SpsF	--	--

[denovogenes]_107146	100.00	0.0	mmk:MU9_3144	K02746	PTS-Aga-EIIC, agaW	PTS system, N-acetylgalactosamine-specific IIC component	--	Metabolism--Carbohydrate metabolism--Galactose metabolism|Environmental Information Processing--Membrane transport--Phosphotransferase system (PTS)

[denovogenes]_107149	98.84	0.0	mmk:MU9_538	K12984	waaE, kdtX	(heptosyl)LPS beta-1,4-glucosyltransferase	2.4.1.-	--

[denovogenes]_10715	64.62	0.0	bfb:VU15_06680	K19049	cslA	chondroitin AC lyase	4.2.2.5	--

[denovogenes]_107150	44.94	1e-63	cml:BN424_418	K17076	K17076, lysY	putative lysine transport system ATP-binding protein	3.6.3.-	Environmental Information Processing--Membrane transport--ABC transporters

[denovogenes]_107154	99.61	0.0	mmk:MU9_872	K15777	DOPA	4,5-DOPA dioxygenase extradiol	1.13.11.-	Metabolism--Biosynthesis of other secondary metabolites--Betalain biosynthesis

[denovogenes]_107155	100.00	0.0	mmk:MU9_794	K01619	deoC, DERA	deoxyribose-phosphate aldolase	4.1.2.4	Metabolism--Carbohydrate metabolism--Pentose phosphate pathway

[denovogenes]_107156	100.00	0.0	mmk:MU9_2721	K03424	--	--	--	--

[denovogenes]_107158	48.65	2e-83	lhi:JP39_03790	K01992	ABC-2.P	ABC-2 type transport system permease protein	--	--

[denovogenes]_107159	74.03	4e-138	edu:LIU_04335	K02042	phnE	phosphonate transport system permease protein	--	Environmental Information Processing--Membrane transport--ABC transporters

[denovogenes]_107160	89.19	7e-168	csb:CLSA_c03580	K03521	fixA, etfB	electron transfer flavoprotein beta subunit	--	--

[denovogenes]_107161	78.33	4e-136	ecas:ECBG_02280	K01990	ABC-2.A	ABC-2 type transport system ATP-binding protein	--	--

[denovogenes]_107162	99.61	0.0	mmk:MU9_1009	K05936	cobM, cbiF	precorrin-4/cobalt-precorrin-4 C11-methyltransferase	2.1.1.133 2.1.1.271	Metabolism--Metabolism of cofactors and vitamins--Porphyrin and chlorophyll metabolism|Metabolism--Global and overview maps--Metabolic pathways

[denovogenes]_107164	62.16	1e-103	mpx:MPD5_1285	K19508	PTS-Gfr-EIIC, gfrC	PTS system, fructoselysine/glucoselysine-specific IIC component	--	Environmental Information Processing--Membrane transport--Phosphotransferase system (PTS)

[denovogenes]_107166	81.47	1e-155	cbv:U729_2023	K17836	penP	beta-lactamase class A	3.5.2.6	Metabolism--Biosynthesis of other secondary metabolites--Penicillin and cephalosporin biosynthesis|Metabolism--Global and overview maps--Biosynthesis of antibiotics|Human Diseases--Drug resistance: Antimicrobial--beta-Lactam resistance

[denovogenes]_107170	66.02	5e-122	lacy:A4V08_31190	K02392	flgG	flagellar basal-body rod protein FlgG	--	Cellular Processes--Cell motility--Flagellar assembly

[denovogenes]_107171	40.96	1e-54	tnp:Tnap_1082	K00059	fabG	3-oxoacyl-[acyl-carrier protein] reductase	1.1.1.100	Metabolism--Lipid metabolism--Fatty acid biosynthesis|Metabolism--Metabolism of cofactors and vitamins--Biotin metabolism|Metabolism--Lipid metabolism--Biosynthesis of unsaturated fatty acids|Metabolism--Global and overview maps--Metabolic pathways|Metabolism--Global and overview maps--Fatty acid metabolism

[denovogenes]_107174	62.11	1e-107	cpe:CPE1490	K07090	K07090	uncharacterized protein	--	--

[denovogenes]_107175	76.54	3e-138	cbut:ATN24_19030	K07238	TC.ZIP, zupT, ZRT3, ZIP2	zinc transporter, ZIP family	--	--

[denovogenes]_107178	93.00	7e-177	cbv:U729_2573	K03091	SIG3.4	RNA polymerase sporulation-specific sigma factor	--	--

[denovogenes]_10718	100.00	0.0	bvu:BVU_0992	K01284	dcp	peptidyl-dipeptidase Dcp	3.4.15.5	--

[denovogenes]_107180	77.99	4e-140	cbv:U729_1084	K03497	parB, spo0J	chromosome partitioning protein, ParB family	--	--

[denovogenes]_107181	73.08	9e-139	cbz:Cbs_5096	K03497	parB, spo0J	chromosome partitioning protein, ParB family	--	--

[denovogenes]_107183	71.94	7e-130	cbv:U729_944	K13292	lgt, umpA	phosphatidylglycerol:prolipoprotein diacylglycerol transferase	2.-.-.-	--

[denovogenes]_107186	80.31	1e-151	cbv:U729_1465	K03521	fixA, etfB	electron transfer flavoprotein beta subunit	--	--

[denovogenes]_107187	65.71	4e-89	cbz:Cbs_1895	K06872	K06872	uncharacterized protein	--	--

[denovogenes]_107188	81.61	4e-151	fpr:FP2_30980	K02747	PTS-Aga-EIID, agaE	PTS system, N-acetylgalactosamine-specific IID component	--	Metabolism--Carbohydrate metabolism--Galactose metabolism|Environmental Information Processing--Membrane transport--Phosphotransferase system (PTS)

[denovogenes]_107189	66.40	4e-111	csh:Closa_0624	K14742	tsaB	tRNA threonylcarbamoyladenosine biosynthesis protein TsaB	--	--

[denovogenes]_10719	87.24	0.0	bvu:BVU_0992	K01284	dcp	peptidyl-dipeptidase Dcp	3.4.15.5	--

[denovogenes]_107190	77.61	4e-145	cbv:U729_1283	K03525	coaX	type III pantothenate kinase	2.7.1.33	Metabolism--Metabolism of cofactors and vitamins--Pantothenate and CoA biosynthesis|Metabolism--Global and overview maps--Metabolic pathways

[denovogenes]_107191	71.20	8e-133	euc:EC1_04720	K03465	thyX, thy1	thymidylate synthase (FAD)	2.1.1.148	Metabolism--Nucleotide metabolism--Pyrimidine metabolism|Metabolism--Metabolism of cofactors and vitamins--One carbon pool by folate|Metabolism--Global and overview maps--Metabolic pathways

[denovogenes]_107192	59.76	5e-108	cbt:CLH_2711	K07238	TC.ZIP, zupT, ZRT3, ZIP2	zinc transporter, ZIP family	--	--

[denovogenes]_107193	99.61	0.0	efq:DR75_560	K00215	dapB	4-hydroxy-tetrahydrodipicolinate reductase	1.17.1.8	Metabolism--Biosynthesis of other secondary metabolites--Monobactam biosynthesis|Metabolism--Amino acid metabolism--Lysine biosynthesis|Metabolism--Global and overview maps--Metabolic pathways|Metabolism--Global and overview maps--Biosynthesis of secondary metabolites|Metabolism--Global and overview maps--Microbial metabolism in diverse environments|Metabolism--Global and overview maps--Biosynthesis of antibiotics|Metabolism--Global and overview maps--Biosynthesis of amino acids

[denovogenes]_107194	81.54	5e-156	cbut:ATN24_02110	K03525	coaX	type III pantothenate kinase	2.7.1.33	Metabolism--Metabolism of cofactors and vitamins--Pantothenate and CoA biosynthesis|Metabolism--Global and overview maps--Metabolic pathways

[denovogenes]_107195	57.20	2e-103	cbv:U729_195	K04486	E3.1.3.15B	histidinol-phosphatase (PHP family)	3.1.3.15	Metabolism--Amino acid metabolism--Histidine metabolism|Metabolism--Global and overview maps--Metabolic pathways|Metabolism--Global and overview maps--Biosynthesis of secondary metabolites|Metabolism--Global and overview maps--Biosynthesis of amino acids

[denovogenes]_107196	100.00	0.0	efq:DR75_283	K02053	ABC.SP.P	putative spermidine/putrescine transport system permease protein	--	Cellular Processes--Cellular community - prokaryotes--Quorum sensing

[denovogenes]_107197	81.85	1e-153	cbv:U729_1283	K03525	coaX	type III pantothenate kinase	2.7.1.33	Metabolism--Metabolism of cofactors and vitamins--Pantothenate and CoA biosynthesis|Metabolism--Global and overview maps--Metabolic pathways

[denovogenes]_107199	61.24	1e-125	elm:ELI_2329	K05306	phnX	phosphonoacetaldehyde hydrolase	3.11.1.1	Metabolism--Metabolism of other amino acids--Phosphonate and phosphinate metabolism|Metabolism--Global and overview maps--Metabolic pathways|Metabolism--Global and overview maps--Microbial metabolism in diverse environments

[denovogenes]_1072	77.69	0.0	dvg:Deval_2796	K03737	por, nifJ	pyruvate-ferredoxin/flavodoxin oxidoreductase	1.2.7.1 1.2.7.-	Metabolism--Carbohydrate metabolism--Glycolysis / Gluconeogenesis|Metabolism--Carbohydrate metabolism--Citrate cycle (TCA cycle)|Metabolism--Carbohydrate metabolism--Pyruvate metabolism|Metabolism--Carbohydrate metabolism--Butanoate metabolism|Metabolism--Energy metabolism--Carbon fixation pathways in prokaryotes|Metabolism--Global and overview maps--Metabolic pathways|Metabolism--Global and overview maps--Microbial metabolism in diverse environments|Metabolism--Global and overview maps--Biosynthesis of antibiotics|Metabolism--Global and overview maps--Carbon metabolism

[denovogenes]_10720	99.41	0.0	bxy:BXY_14930	K01277	DPP3	dipeptidyl-peptidase III	3.4.14.4	--

[denovogenes]_107200	76.65	9e-140	cbv:U729_1453	K06182	rluF	23S rRNA pseudouridine2604 synthase	5.4.99.21	--

[denovogenes]_107202	75.97	2e-143	csr:Cspa_c10850	K17836	penP	beta-lactamase class A	3.5.2.6	Metabolism--Biosynthesis of other secondary metabolites--Penicillin and cephalosporin biosynthesis|Metabolism--Global and overview maps--Biosynthesis of antibiotics|Human Diseases--Drug resistance: Antimicrobial--beta-Lactam resistance

[denovogenes]_107203	98.46	0.0	seeb:SEEB0189_000080	K12057	traF	conjugal transfer pilus assembly protein TraF	--	--

[denovogenes]_107204	40.16	4e-48	byl:A4V09_12980	K06987	K06987	uncharacterized protein	--	--

[denovogenes]_107205	100.00	0.0	pvl:AOB99_00180	K12984	waaE, kdtX	(heptosyl)LPS beta-1,4-glucosyltransferase	2.4.1.-	--

[denovogenes]_107207	86.05	2e-160	cld:CLSPO_c21800	K03149	thiG	thiazole synthase	2.8.1.10	Metabolism--Metabolism of cofactors and vitamins--Thiamine metabolism|Metabolism--Global and overview maps--Metabolic pathways

[denovogenes]_107208	98.56	6e-150	cfd:CFNIH1_11895	K20264	fusR	two-component system, NarL family, response regulator FusR	--	Environmental Information Processing--Signal transduction--Two-component system|Cellular Processes--Cellular community - prokaryotes--Quorum sensing

[denovogenes]_107212	88.02	8e-156	cbz:Cbs_0069	K02594	nifV	homocitrate synthase NifV	2.3.3.14	Metabolism--Carbohydrate metabolism--Pyruvate metabolism

[denovogenes]_107213	99.61	0.0	cfd:CFNIH1_02890	K02746	PTS-Aga-EIIC, agaW	PTS system, N-acetylgalactosamine-specific IIC component	--	Metabolism--Carbohydrate metabolism--Galactose metabolism|Environmental Information Processing--Membrane transport--Phosphotransferase system (PTS)

[denovogenes]_107214	72.48	2e-135	cfz:CSG_9300	K18333	K18333	L-fucose dehydrogenase	--	Metabolism--Carbohydrate metabolism--Fructose and mannose metabolism|Metabolism--Global and overview maps--Microbial metabolism in diverse environments

[denovogenes]_107215	67.74	6e-122	lacy:A4V08_10510	K02050	ABC.SN.P	NitT/TauT family transport system permease protein	--	--

[denovogenes]_107216	100.00	0.0	pvl:AOB99_14905	K11070	potC	spermidine/putrescine transport system permease protein	--	Environmental Information Processing--Membrane transport--ABC transporters

[denovogenes]_107217	43.96	2e-76	cct:CC1_06670	K04486	E3.1.3.15B	histidinol-phosphatase (PHP family)	3.1.3.15	Metabolism--Amino acid metabolism--Histidine metabolism|Metabolism--Global and overview maps--Metabolic pathways|Metabolism--Global and overview maps--Biosynthesis of secondary metabolites|Metabolism--Global and overview maps--Biosynthesis of amino acids

[denovogenes]_107218	100.00	0.0	pvl:AOB99_15160	K03149	thiG	thiazole synthase	2.8.1.10	Metabolism--Metabolism of cofactors and vitamins--Thiamine metabolism|Metabolism--Global and overview maps--Metabolic pathways

[denovogenes]_107219	100.00	0.0	pvl:AOB99_14065	K01619	deoC, DERA	deoxyribose-phosphate aldolase	4.1.2.4	Metabolism--Carbohydrate metabolism--Pentose phosphate pathway

[denovogenes]_107220	60.63	1e-103	cpe:CPE1490	K07090	K07090	uncharacterized protein	--	--

[denovogenes]_107222	99.61	0.0	stb:SGPB_0948	K03470	rnhB	ribonuclease HII	3.1.26.4	Genetic Information Processing--Replication and repair--DNA replication

[denovogenes]_107223	99.61	0.0	pvl:AOB99_02545	K02066	mlaE, linK	phospholipid/cholesterol/gamma-HCH transport system permease protein	--	Environmental Information Processing--Membrane transport--ABC transporters

[denovogenes]_107224	84.41	7e-150	cbv:U729_47	K04043	dnaK	molecular chaperone DnaK	--	Genetic Information Processing--Folding, sorting and degradation--RNA degradation|Organismal Systems--Aging--Longevity regulating pathway - worm|Human Diseases--Infectious diseases: Bacterial--Tuberculosis

[denovogenes]_107225	77.22	2e-147	cpr:CPR_2470	K03525	coaX	type III pantothenate kinase	2.7.1.33	Metabolism--Metabolism of cofactors and vitamins--Pantothenate and CoA biosynthesis|Metabolism--Global and overview maps--Metabolic pathways

[denovogenes]_107226	80.69	8e-152	cbv:U729_1283	K03525	coaX	type III pantothenate kinase	2.7.1.33	Metabolism--Metabolism of cofactors and vitamins--Pantothenate and CoA biosynthesis|Metabolism--Global and overview maps--Metabolic pathways

[denovogenes]_107228	93.44	0.0	cfd:CFNIH1_14120	K13574	ybiC	uncharacterized oxidoreductase	1.1.1.-	--

[denovogenes]_107230	78.52	1e-149	cle:Clole_3377	K03525	coaX	type III pantothenate kinase	2.7.1.33	Metabolism--Metabolism of cofactors and vitamins--Pantothenate and CoA biosynthesis|Metabolism--Global and overview maps--Metabolic pathways

[denovogenes]_107231	61.35	2e-108	cbt:CLH_2711	K07238	TC.ZIP, zupT, ZRT3, ZIP2	zinc transporter, ZIP family	--	--

[denovogenes]_107234	72.59	2e-139	cbn:CbC4_0167	K03525	coaX	type III pantothenate kinase	2.7.1.33	Metabolism--Metabolism of cofactors and vitamins--Pantothenate and CoA biosynthesis|Metabolism--Global and overview maps--Metabolic pathways

[denovogenes]_107238	74.62	1e-142	vpr:Vpar_0808	K09811	ftsX	cell division transport system permease protein	--	Environmental Information Processing--Membrane transport--ABC transporters

[denovogenes]_10724	69.16	0.0	cpy:Cphy_3524	K04759	feoB	ferrous iron transport protein B	--	--

[denovogenes]_107240	71.15	4e-136	sfo:Z042_09695	K08368	yaaU	MFS transporter, putative metabolite transport protein	--	--

[denovogenes]_107241	79.17	2e-128	cfd:CFNIH1_12830	K08088	fimY	fimbrial protein FimY	--	--

[denovogenes]_107242	47.79	2e-71	cth:Cthe_1671	K06400	spoIVCA	site-specific DNA recombinase	--	--

[denovogenes]_107243	99.23	0.0	eoc:CE10_1702	K10558	lsrA, ego	AI-2 transport system ATP-binding protein	--	Environmental Information Processing--Membrane transport--ABC transporters|Cellular Processes--Cellular community - prokaryotes--Quorum sensing

[denovogenes]_107244	87.64	4e-166	cpe:CPE2299	K03521	fixA, etfB	electron transfer flavoprotein beta subunit	--	--

[denovogenes]_107245	71.81	4e-123	cld:CLSPO_c30670	K01042	selA	L-seryl-tRNA(Ser) seleniumtransferase	2.9.1.1	Metabolism--Metabolism of other amino acids--Selenocompound metabolism|Genetic Information Processing--Translation--Aminoacyl-tRNA biosynthesis

[denovogenes]_107246	73.85	2e-138	faa:HMPREF0389_01061	K00088	IMPDH, guaB	IMP dehydrogenase	1.1.1.205	Metabolism--Nucleotide metabolism--Purine metabolism|Metabolism--Xenobiotics biodegradation and metabolism--Drug metabolism - other enzymes|Metabolism--Global and overview maps--Metabolic pathways|Metabolism--Global and overview maps--Biosynthesis of secondary metabolites

[denovogenes]_107247	53.36	1e-90	bha:BH3444	K07497	K07497	putative transposase	--	--

[denovogenes]_107248	100.00	0.0	elo:EC042_2648	K03736	eutC	ethanolamine ammonia-lyase small subunit	4.3.1.7	Metabolism--Lipid metabolism--Glycerophospholipid metabolism|Metabolism--Global and overview maps--Metabolic pathways

[denovogenes]_10725	95.12	0.0	bhl:Bache_1257	K03798	ftsH, hflB	cell division protease FtsH	3.4.24.-	--

[denovogenes]_107251	89.53	6e-156	cdl:CDR20291_1001	K03979	obgE, cgtA	GTPase	3.6.5.-	--

[denovogenes]_107253	64.00	1e-123	fpla:A4U99_06680	K02013	ABC.FEV.A	iron complex transport system ATP-binding protein	3.6.3.34	Environmental Information Processing--Membrane transport--ABC transporters

[denovogenes]_107254	68.36	5e-135	cbz:Cbs_0239	K01739	metB	cystathionine gamma-synthase	2.5.1.48	Metabolism--Amino acid metabolism--Cysteine and methionine metabolism|Metabolism--Metabolism of other amino acids--Selenocompound metabolism|Metabolism--Energy metabolism--Sulfur metabolism|Metabolism--Global and overview maps--Metabolic pathways|Metabolism--Global and overview maps--Biosynthesis of secondary metabolites|Metabolism--Global and overview maps--Biosynthesis of antibiotics|Metabolism--Global and overview maps--Biosynthesis of amino acids

[denovogenes]_107256	50.58	1e-80	cpe:CPE0391	K07098	K07098	uncharacterized protein	--	--

[denovogenes]_107260	100.00	0.0	cbut:ATN24_20120	K02529	lacI, galR	LacI family transcriptional regulator	--	--

[denovogenes]_107262	74.62	2e-143	csr:Cspa_c02800	K01652	E2.2.1.6L, ilvB, ilvG, ilvI	acetolactate synthase I/II/III large subunit	2.2.1.6	Metabolism--Amino acid metabolism--Valine, leucine and isoleucine biosynthesis|Metabolism--Carbohydrate metabolism--Butanoate metabolism|Metabolism--Carbohydrate metabolism--C5-Branched dibasic acid metabolism|Metabolism--Metabolism of cofactors and vitamins--Pantothenate and CoA biosynthesis|Metabolism--Global and overview maps--Metabolic pathways|Metabolism--Global and overview maps--Biosynthesis of secondary metabolites|Metabolism--Global and overview maps--Biosynthesis of antibiotics|Metabolism--Global and overview maps--2-Oxocarboxylic acid metabolism|Metabolism--Global and overview maps--Biosynthesis of amino acids

[denovogenes]_107264	100.00	0.0	shq:A0259_04270	K01000	mraY	phospho-N-acetylmuramoyl-pentapeptide-transferase	2.7.8.13	Metabolism--Glycan biosynthesis and metabolism--Peptidoglycan biosynthesis|Metabolism--Global and overview maps--Metabolic pathways|Human Diseases--Drug resistance: Antimicrobial--Vancomycin resistance

[denovogenes]_107266	100.00	0.0	boa:Bovatus_02855	K01610	E4.1.1.49, pckA	phosphoenolpyruvate carboxykinase (ATP)	4.1.1.49	Metabolism--Carbohydrate metabolism--Glycolysis / Gluconeogenesis|Metabolism--Carbohydrate metabolism--Citrate cycle (TCA cycle)|Metabolism--Carbohydrate metabolism--Pyruvate metabolism|Metabolism--Energy metabolism--Carbon fixation in photosynthetic organisms|Metabolism--Global and overview maps--Metabolic pathways|Metabolism--Global and overview maps--Biosynthesis of secondary metabolites|Metabolism--Global and overview maps--Microbial metabolism in diverse environments|Metabolism--Global and overview maps--Biosynthesis of antibiotics|Metabolism--Global and overview maps--Carbon metabolism

[denovogenes]_107267	79.23	1e-154	vpr:Vpar_1187	K06281	hyaB, hybC	hydrogenase large subunit	1.12.99.6	Metabolism--Xenobiotics biodegradation and metabolism--Nitrotoluene degradation|Metabolism--Global and overview maps--Microbial metabolism in diverse environments

[denovogenes]_107268	75.77	4e-130	cpr:CPR_1776	K00756	pdp	pyrimidine-nucleoside phosphorylase	2.4.2.2	Metabolism--Nucleotide metabolism--Pyrimidine metabolism|Metabolism--Global and overview maps--Metabolic pathways

[denovogenes]_107269	74.23	2e-141	cah:CAETHG_1885	K00820	glmS, GFPT	glucosamine--fructose-6-phosphate aminotransferase (isomerizing)	2.6.1.16	Metabolism--Amino acid metabolism--Alanine, aspartate and glutamate metabolism|Metabolism--Carbohydrate metabolism--Amino sugar and nucleotide sugar metabolism|Metabolism--Global and overview maps--Metabolic pathways|Metabolism--Global and overview maps--Biosynthesis of antibiotics|Human Diseases--Endocrine and metabolic diseases--Insulin resistance

[denovogenes]_10727	46.58	0.0	csq:CSCA_2386	K09181	yfiQ	acetyltransferase	--	--

[denovogenes]_107270	50.00	4e-90	lae:LBAT_1107	K02004	ABC.CD.P	putative ABC transport system permease protein	--	--

[denovogenes]_107271	79.68	5e-143	pdc:CDIF630_02804	K03475	PTS-Ula-EIIC, ulaA, sgaT	PTS system, ascorbate-specific IIC component	--	Metabolism--Carbohydrate metabolism--Ascorbate and aldarate metabolism|Metabolism--Global and overview maps--Metabolic pathways|Metabolism--Global and overview maps--Microbial metabolism in diverse environments|Environmental Information Processing--Membrane transport--Phosphotransferase system (PTS)

[denovogenes]_107272	84.62	5e-155	cbh:CLC_0813	K03325	TC.ACR3	arsenite transporter, ACR3 family	--	--

[denovogenes]_107273	45.38	1e-63	byl:A4V09_08855	K03292	TC.GPH	glycoside/pentoside/hexuronide:cation symporter, GPH family	--	--

[denovogenes]_107275	100.00	0.0	pvl:AOB99_11985	K00058	serA, PHGDH	D-3-phosphoglycerate dehydrogenase	1.1.1.95	Metabolism--Amino acid metabolism--Glycine, serine and threonine metabolism|Metabolism--Energy metabolism--Methane metabolism|Metabolism--Global and overview maps--Metabolic pathways|Metabolism--Global and overview maps--Microbial metabolism in diverse environments|Metabolism--Global and overview maps--Biosynthesis of antibiotics|Metabolism--Global and overview maps--Carbon metabolism|Metabolism--Global and overview maps--Biosynthesis of amino acids

[denovogenes]_107276	54.62	2e-80	cbi:CLJ_B3866	K06409	spoVB	stage V sporulation protein B	--	--

[denovogenes]_107279	65.77	1e-114	cle:Clole_2837	K03324	yjbB	phosphate:Na+ symporter	--	--

[denovogenes]_10728	58.62	0.0	fpla:A4U99_00895	K03798	ftsH, hflB	cell division protease FtsH	3.4.24.-	--

[denovogenes]_107282	97.69	0.0	vpr:Vpar_0831	K03527	ispH, lytB	4-hydroxy-3-methylbut-2-en-1-yl diphosphate reductase	1.17.7.4	Metabolism--Metabolism of terpenoids and polyketides--Terpenoid backbone biosynthesis|Metabolism--Global and overview maps--Metabolic pathways|Metabolism--Global and overview maps--Biosynthesis of secondary metabolites|Metabolism--Global and overview maps--Biosynthesis of antibiotics

[denovogenes]_107282	97.69	0.0	vpr:Vpar_0831	K02945	RP-S1, rpsA	small subunit ribosomal protein S1	--	Genetic Information Processing--Translation--Ribosome

[denovogenes]_107283	83.08	5e-162	boa:Bovatus_01529	K03427	hsdM	type I restriction enzyme M protein	2.1.1.72	--

[denovogenes]_107286	45.21	8e-78	med:MELS_1362	K15342	cas1	CRISP-associated protein Cas1	--	--

[denovogenes]_107289	43.20	1e-12	spv:SPH_2087	K01447	E3.5.1.28A, cwlA, xlyA, xlyB	N-acetylmuramoyl-L-alanine amidase	3.5.1.28	--

[denovogenes]_10729	73.44	0.0	bvu:BVU_0102	K01206	FUCA	alpha-L-fucosidase	3.2.1.51	Metabolism--Glycan biosynthesis and metabolism--Other glycan degradation

[denovogenes]_107291	42.31	1e-55	cbz:Cbs_3405	K11527	K11527	two-component system, sensor histidine kinase and response regulator	2.7.13.3	--

[denovogenes]_107292	67.69	2e-121	cbv:U729_49	K03705	hrcA	heat-inducible transcriptional repressor	--	--

[denovogenes]_107293	72.31	2e-141	cld:CLSPO_c32480	K04083	hslO	molecular chaperone Hsp33	--	--

[denovogenes]_107294	42.02	4e-44	marn:LN42_08850	K06871	K06871	uncharacterized protein	--	--

[denovogenes]_107296	84.23	7e-162	csr:Cspa_c57850	K00690	E2.4.1.7	sucrose phosphorylase	2.4.1.7	Metabolism--Carbohydrate metabolism--Starch and sucrose metabolism

[denovogenes]_107297	65.87	1e-122	ain:Acin_1457	K09007	GCH4, folE	GTP cyclohydrolase I	3.5.4.16	Metabolism--Metabolism of cofactors and vitamins--Folate biosynthesis|Metabolism--Global and overview maps--Metabolic pathways

[denovogenes]_10730	100.00	0.0	bth:BT_1846	K01277	DPP3	dipeptidyl-peptidase III	3.4.14.4	--

[denovogenes]_107300	78.60	3e-150	byl:A4V09_02790	K03091	SIG3.4	RNA polymerase sporulation-specific sigma factor	--	--

[denovogenes]_107303	65.75	5e-109	afn:Acfer_0649	K02030	ABC.PA.S	polar amino acid transport system substrate-binding protein	--	--

[denovogenes]_107307	56.64	4e-105	ain:Acin_0801	K02013	ABC.FEV.A	iron complex transport system ATP-binding protein	3.6.3.34	Environmental Information Processing--Membrane transport--ABC transporters

[denovogenes]_10731	100.00	0.0	shq:A0259_13260	K03797	E3.4.21.102, prc, ctpA	carboxyl-terminal processing protease	3.4.21.102	--

[denovogenes]_107312	100.00	0.0	bvu:BVU_3310	K02823	pyrDII	dihydroorotate dehydrogenase electron transfer subunit	--	Metabolism--Nucleotide metabolism--Pyrimidine metabolism|Metabolism--Global and overview maps--Metabolic pathways

[denovogenes]_107313	99.61	0.0	bxy:BXY_21000	K08641	vanX	D-alanyl-D-alanine dipeptidase	3.4.13.22	Human Diseases--Drug resistance: Antimicrobial--Vancomycin resistance|Environmental Information Processing--Signal transduction--Two-component system

[denovogenes]_107315	88.03	4e-172	bhl:Bache_3306	K03424	--	--	--	--

[denovogenes]_107316	65.64	1e-116	byl:A4V09_05865	K01695	trpA	tryptophan synthase alpha chain	4.2.1.20	Metabolism--Amino acid metabolism--Glycine, serine and threonine metabolism|Metabolism--Amino acid metabolism--Phenylalanine, tyrosine and tryptophan biosynthesis|Metabolism--Global and overview maps--Metabolic pathways|Metabolism--Global and overview maps--Biosynthesis of secondary metabolites|Metabolism--Global and overview maps--Biosynthesis of antibiotics|Metabolism--Global and overview maps--Biosynthesis of amino acids

[denovogenes]_10732	98.39	0.0	cfd:CFNIH1_13590	K01547	kdpB	K+-transporting ATPase ATPase B chain	3.6.3.12	Environmental Information Processing--Signal transduction--Two-component system

[denovogenes]_107323	100.00	0.0	btho:Btheta7330_00941	K03710	K03710	GntR family transcriptional regulator	--	--

[denovogenes]_107324	58.53	7e-106	rim:ROI_21800	K07258	dacC, dacA, dacD	D-alanyl-D-alanine carboxypeptidase (penicillin-binding protein 5/6)	3.4.16.4	Metabolism--Glycan biosynthesis and metabolism--Peptidoglycan biosynthesis|Metabolism--Global and overview maps--Metabolic pathways

[denovogenes]_107325	73.64	2e-142	csr:Cspa_c54150	K00978	rfbF	glucose-1-phosphate cytidylyltransferase	2.7.7.33	Metabolism--Carbohydrate metabolism--Starch and sucrose metabolism|Metabolism--Carbohydrate metabolism--Amino sugar and nucleotide sugar metabolism|Metabolism--Global and overview maps--Metabolic pathways

[denovogenes]_107326	99.61	0.0	bxy:BXY_23870	K02477	K02477	two-component system, LytT family, response regulator	--	--

[denovogenes]_107328	100.00	0.0	bxy:BXY_16900	K07052	K07052	uncharacterized protein	--	--

[denovogenes]_107330	77.43	1e-146	byl:A4V09_02790	K03091	SIG3.4	RNA polymerase sporulation-specific sigma factor	--	--

[denovogenes]_107331	42.92	2e-67	rum:CK1_17490	K01104	E3.1.3.48	protein-tyrosine phosphatase	3.1.3.48	--

[denovogenes]_107332	86.82	4e-167	bhl:Bache_2058	K01695	trpA	tryptophan synthase alpha chain	4.2.1.20	Metabolism--Amino acid metabolism--Glycine, serine and threonine metabolism|Metabolism--Amino acid metabolism--Phenylalanine, tyrosine and tryptophan biosynthesis|Metabolism--Global and overview maps--Metabolic pathways|Metabolism--Global and overview maps--Biosynthesis of secondary metabolites|Metabolism--Global and overview maps--Biosynthesis of antibiotics|Metabolism--Global and overview maps--Biosynthesis of amino acids

[denovogenes]_107333	75.10	1e-132	sew:SeSA_A0319	K11890	impM	type VI secretion system protein ImpM	--	Cellular Processes--Cellular community - prokaryotes--Biofilm formation - Pseudomonas aeruginosa

[denovogenes]_107334	50.62	4e-85	tpz:Tph_c25020	K02034	ABC.PE.P1	peptide/nickel transport system permease protein	--	Cellular Processes--Cellular community - prokaryotes--Quorum sensing

[denovogenes]_107337	71.60	1e-144	ssg:Selsp_0532	K00978	rfbF	glucose-1-phosphate cytidylyltransferase	2.7.7.33	Metabolism--Carbohydrate metabolism--Starch and sucrose metabolism|Metabolism--Carbohydrate metabolism--Amino sugar and nucleotide sugar metabolism|Metabolism--Global and overview maps--Metabolic pathways

[denovogenes]_107340	43.14	7e-66	ssm:Spirs_2739	K11645	fbaB	fructose-bisphosphate aldolase, class I	4.1.2.13	Metabolism--Carbohydrate metabolism--Glycolysis / Gluconeogenesis|Metabolism--Carbohydrate metabolism--Pentose phosphate pathway|Metabolism--Carbohydrate metabolism--Fructose and mannose metabolism|Metabolism--Energy metabolism--Methane metabolism|Metabolism--Energy metabolism--Carbon fixation in photosynthetic organisms|Metabolism--Global and overview maps--Metabolic pathways|Metabolism--Global and overview maps--Biosynthesis of secondary metabolites|Metabolism--Global and overview maps--Microbial metabolism in diverse environments|Metabolism--Global and overview maps--Biosynthesis of antibiotics|Metabolism--Global and overview maps--Carbon metabolism|Metabolism--Global and overview maps--Biosynthesis of amino acids

[denovogenes]_107342	50.20	2e-73	eha:Ethha_1020	K06173	truA, PUS1	tRNA pseudouridine38-40 synthase	5.4.99.12	--

[denovogenes]_107343	50.19	6e-91	ccel:CCDG5_0980	K07699	spo0A	two-component system, response regulator, stage 0 sporulation protein A	--	Environmental Information Processing--Signal transduction--Two-component system|Cellular Processes--Cellular community - prokaryotes--Quorum sensing

[denovogenes]_107345	64.45	2e-102	hsd:SD1D_2186	K02030	ABC.PA.S	polar amino acid transport system substrate-binding protein	--	--

[denovogenes]_107346	77.87	1e-149	bsa:Bacsa_0489	K00571	E2.1.1.72	site-specific DNA-methyltransferase (adenine-specific)	2.1.1.72	--

[denovogenes]_107347	44.96	6e-69	bprs:CK3_32460	K21023	mucR	diguanylate cyclase	2.7.7.65	Cellular Processes--Cellular community - prokaryotes--Biofilm formation - Pseudomonas aeruginosa

[denovogenes]_107348	98.07	0.0	bxy:BXY_43340	K02342	DPO3E, dnaQ	DNA polymerase III subunit epsilon	2.7.7.7	Metabolism--Nucleotide metabolism--Purine metabolism|Metabolism--Nucleotide metabolism--Pyrimidine metabolism|Metabolism--Global and overview maps--Metabolic pathways|Genetic Information Processing--Replication and repair--DNA replication|Genetic Information Processing--Replication and repair--Mismatch repair|Genetic Information Processing--Replication and repair--Homologous recombination

[denovogenes]_107350	69.77	1e-127	ess:ATZ33_10805	K02042	phnE	phosphonate transport system permease protein	--	Environmental Information Processing--Membrane transport--ABC transporters

[denovogenes]_107352	40.89	5e-54	tnp:Tnap_1082	K00059	fabG	3-oxoacyl-[acyl-carrier protein] reductase	1.1.1.100	Metabolism--Lipid metabolism--Fatty acid biosynthesis|Metabolism--Metabolism of cofactors and vitamins--Biotin metabolism|Metabolism--Lipid metabolism--Biosynthesis of unsaturated fatty acids|Metabolism--Global and overview maps--Metabolic pathways|Metabolism--Global and overview maps--Fatty acid metabolism

[denovogenes]_107356	40.24	2e-45	xho:A9255_02245	K18009	budC	meso-butanediol dehydrogenase / (S,S)-butanediol dehydrogenase / diacetyl reductase	1.1.1.- 1.1.1.76 1.1.1.304	Metabolism--Carbohydrate metabolism--Butanoate metabolism

[denovogenes]_107357	79.76	7e-142	byl:A4V09_20255	K02010	afuC, fbpC	iron(III) transport system ATP-binding protein	3.6.3.30	Environmental Information Processing--Membrane transport--ABC transporters

[denovogenes]_107359	61.32	3e-101	cpy:Cphy_0062	K13292	lgt, umpA	phosphatidylglycerol:prolipoprotein diacylglycerol transferase	2.-.-.-	--

[denovogenes]_107363	40.34	4e-51	clc:Calla_2203	K03436	fruR2, fruR	DeoR family transcriptional regulator, fructose operon transcriptional repressor	--	--

[denovogenes]_107364	99.23	0.0	btho:Btheta7330_04253	K03787	surE	5'-nucleotidase	3.1.3.5	Metabolism--Nucleotide metabolism--Purine metabolism|Metabolism--Nucleotide metabolism--Pyrimidine metabolism|Metabolism--Metabolism of cofactors and vitamins--Nicotinate and nicotinamide metabolism|Metabolism--Global and overview maps--Metabolic pathways|Metabolism--Global and overview maps--Biosynthesis of secondary metabolites

[denovogenes]_107366	49.43	3e-74	psab:PSAB_07185	K01250	rihA	pyrimidine-specific ribonucleoside hydrolase	3.2.-.-	--

[denovogenes]_107368	98.81	0.0	bhl:Bache_1251	K03496	parA, soj	chromosome partitioning protein	--	--

[denovogenes]_107369	100.00	0.0	bxy:BXY_43340	K02342	DPO3E, dnaQ	DNA polymerase III subunit epsilon	2.7.7.7	Metabolism--Nucleotide metabolism--Purine metabolism|Metabolism--Nucleotide metabolism--Pyrimidine metabolism|Metabolism--Global and overview maps--Metabolic pathways|Genetic Information Processing--Replication and repair--DNA replication|Genetic Information Processing--Replication and repair--Mismatch repair|Genetic Information Processing--Replication and repair--Homologous recombination

[denovogenes]_107370	71.76	6e-128	cpy:Cphy_0424	K03525	coaX	type III pantothenate kinase	2.7.1.33	Metabolism--Metabolism of cofactors and vitamins--Pantothenate and CoA biosynthesis|Metabolism--Global and overview maps--Metabolic pathways

[denovogenes]_107372	100.00	0.0	ecr:ECIAI1_0494	K02069	ABC.X2.P	putative ABC transport system permease protein	--	--

[denovogenes]_107374	62.45	4e-100	hsd:SD1D_2186	K02030	ABC.PA.S	polar amino acid transport system substrate-binding protein	--	--

[denovogenes]_107375	100.00	0.0	ssj:SSON53_17700	K12529	--	--	--	Metabolism--Metabolism of other amino acids--Selenocompound metabolism

[denovogenes]_107376	42.15	5e-44	tbo:Thebr_0613	K02444	glpR	DeoR family transcriptional regulator, glycerol-3-phosphate regulon repressor	--	--

[denovogenes]_107377	41.77	1e-55	tnp:Tnap_1082	K00059	fabG	3-oxoacyl-[acyl-carrier protein] reductase	1.1.1.100	Metabolism--Lipid metabolism--Fatty acid biosynthesis|Metabolism--Metabolism of cofactors and vitamins--Biotin metabolism|Metabolism--Lipid metabolism--Biosynthesis of unsaturated fatty acids|Metabolism--Global and overview maps--Metabolic pathways|Metabolism--Global and overview maps--Fatty acid metabolism

[denovogenes]_107378	42.01	2e-63	kcr:Kcr_1502	K00772	mtaP, MTAP	5'-methylthioadenosine phosphorylase	2.4.2.28	Metabolism--Amino acid metabolism--Cysteine and methionine metabolism|Metabolism--Global and overview maps--Metabolic pathways

[denovogenes]_107379	97.27	0.0	bdh:GV66_15455	K02342	DPO3E, dnaQ	DNA polymerase III subunit epsilon	2.7.7.7	Metabolism--Nucleotide metabolism--Purine metabolism|Metabolism--Nucleotide metabolism--Pyrimidine metabolism|Metabolism--Global and overview maps--Metabolic pathways|Genetic Information Processing--Replication and repair--DNA replication|Genetic Information Processing--Replication and repair--Mismatch repair|Genetic Information Processing--Replication and repair--Homologous recombination

[denovogenes]_10738	78.30	0.0	pdi:BDI_0207	K01284	dcp	peptidyl-dipeptidase Dcp	3.4.15.5	--

[denovogenes]_107381	80.40	4e-148	fpla:A4U99_01915	K01265	map	methionyl aminopeptidase	3.4.11.18	--

[denovogenes]_107383	53.74	3e-73	pary:A4V02_08670	K10716	kch, trkA, mthK, pch	voltage-gated potassium channel	--	--

[denovogenes]_107384	45.10	2e-74	tur:AT726_04910	K02049	ABC.SN.A	NitT/TauT family transport system ATP-binding protein	--	--

[denovogenes]_107387	67.19	6e-126	eha:Ethha_2791	K03496	parA, soj	chromosome partitioning protein	--	--

[denovogenes]_107389	97.28	0.0	lacy:A4V08_11130	K02027	ABC.MS.S	multiple sugar transport system substrate-binding protein	--	--

[denovogenes]_10739	100.00	0.0	paeu:BN889_00479	K02660	pilJ	twitching motility protein PilJ	--	Environmental Information Processing--Signal transduction--Two-component system|Cellular Processes--Cellular community - prokaryotes--Biofilm formation - Pseudomonas aeruginosa

[denovogenes]_107390	49.37	4e-78	shc:Shell_0463	K02032	ABC.PE.A1	peptide/nickel transport system ATP-binding protein	--	Cellular Processes--Cellular community - prokaryotes--Quorum sensing

[denovogenes]_107392	89.96	4e-174	bvu:BVU_3310	K02823	pyrDII	dihydroorotate dehydrogenase electron transfer subunit	--	Metabolism--Nucleotide metabolism--Pyrimidine metabolism|Metabolism--Global and overview maps--Metabolic pathways

[denovogenes]_107394	99.23	0.0	cdl:CDR20291_1752	K01992	ABC-2.P	ABC-2 type transport system permease protein	--	--

[denovogenes]_107396	41.00	1e-66	sli:Slin_2091	K02477	K02477	two-component system, LytT family, response regulator	--	--

[denovogenes]_107397	83.33	5e-161	bvu:BVU_4093	K01695	trpA	tryptophan synthase alpha chain	4.2.1.20	Metabolism--Amino acid metabolism--Glycine, serine and threonine metabolism|Metabolism--Amino acid metabolism--Phenylalanine, tyrosine and tryptophan biosynthesis|Metabolism--Global and overview maps--Metabolic pathways|Metabolism--Global and overview maps--Biosynthesis of secondary metabolites|Metabolism--Global and overview maps--Biosynthesis of antibiotics|Metabolism--Global and overview maps--Biosynthesis of amino acids

[denovogenes]_107398	70.87	2e-125	lacy:A4V08_19435	K06180	rluD	23S rRNA pseudouridine1911/1915/1917 synthase	5.4.99.23	--

[denovogenes]_107399	64.37	7e-110	csh:Closa_0624	K14742	tsaB	tRNA threonylcarbamoyladenosine biosynthesis protein TsaB	--	--

[denovogenes]_10740	99.71	0.0	cfd:CFNIH1_13590	K01547	kdpB	K+-transporting ATPase ATPase B chain	3.6.3.12	Environmental Information Processing--Signal transduction--Two-component system

[denovogenes]_107400	55.04	5e-78	lacy:A4V08_14720	K00878	thiM	hydroxyethylthiazole kinase	2.7.1.50	Metabolism--Metabolism of cofactors and vitamins--Thiamine metabolism|Metabolism--Global and overview maps--Metabolic pathways

[denovogenes]_107402	68.46	1e-129	lacy:A4V08_28590	K02026	ABC.MS.P1	multiple sugar transport system permease protein	--	--

[denovogenes]_107403	98.84	0.0	sdz:Asd1617_06116	K01619	deoC, DERA	deoxyribose-phosphate aldolase	4.1.2.4	Metabolism--Carbohydrate metabolism--Pentose phosphate pathway

[denovogenes]_107404	98.07	0.0	lacy:A4V08_31190	K02392	flgG	flagellar basal-body rod protein FlgG	--	Cellular Processes--Cell motility--Flagellar assembly

[denovogenes]_107407	40.96	3e-32	cin:100180086	K17286	STOM	erythrocyte band 7 integral membrane protein	--	--

[denovogenes]_107409	85.15	2e-143	lacy:A4V08_15010	K01243	mtnN, mtn, pfs	adenosylhomocysteine nucleosidase	3.2.2.9	Metabolism--Amino acid metabolism--Cysteine and methionine metabolism|Metabolism--Global and overview maps--Metabolic pathways|Metabolism--Global and overview maps--Biosynthesis of amino acids

[denovogenes]_107414	95.31	5e-164	fpla:A4U99_03285	K02421	fliR	flagellar biosynthetic protein FliR	--	Cellular Processes--Cell motility--Flagellar assembly

[denovogenes]_107415	93.46	1e-175	bvu:BVU_0450	K03977	engA, der	GTPase	--	--

[denovogenes]_107419	82.69	2e-162	bfs:BF9343_3824	K01802	E5.2.1.8	peptidylprolyl isomerase	5.2.1.8	--

[denovogenes]_10742	99.85	0.0	cfd:CFNIH1_15905	K16089	TC.FEV.OM2, cirA, cfrA, hmuR	outer membrane receptor for ferrienterochelin and colicins	--	--

[denovogenes]_107420	92.66	0.0	elp:P12B_c1690	K08303	K08303	putative protease	3.4.-.-	Human Diseases--Infectious diseases: Bacterial--Epithelial cell signaling in Helicobacter pylori infection

[denovogenes]_107424	66.54	2e-116	lacy:A4V08_22680	K02035	ABC.PE.S	peptide/nickel transport system substrate-binding protein	--	Cellular Processes--Cellular community - prokaryotes--Quorum sensing

[denovogenes]_107425	83.01	2e-155	ibu:IB211_00078c	K06987	K06987	uncharacterized protein	--	--

[denovogenes]_107427	98.08	0.0	cfd:CFNIH1_08235	K07643	basS	two-component system, OmpR family, sensor histidine kinase BasS	2.7.13.3	Human Diseases--Drug resistance: Antimicrobial--Cationic antimicrobial peptide (CAMP) resistance|Environmental Information Processing--Signal transduction--Two-component system

[denovogenes]_107429	51.64	3e-82	bmyc:DJ92_3932	K00128	--	--	--	Metabolism--Carbohydrate metabolism--Glycolysis / Gluconeogenesis|Metabolism--Carbohydrate metabolism--Ascorbate and aldarate metabolism|Metabolism--Lipid metabolism--Fatty acid degradation|Metabolism--Amino acid metabolism--Valine, leucine and isoleucine degradation|Metabolism--Amino acid metabolism--Lysine degradation|Metabolism--Amino acid metabolism--Arginine and proline metabolism|Metabolism--Amino acid metabolism--Histidine metabolism|Metabolism--Amino acid metabolism--Tryptophan metabolism|Metabolism--Metabolism of other amino acids--beta-Alanine metabolism|Metabolism--Lipid metabolism--Glycerolipid metabolism|Metabolism--Carbohydrate metabolism--Pyruvate metabolism|Metabolism--Xenobiotics biodegradation and metabolism--Chloroalkane and chloroalkene degradation|Metabolism--Metabolism of terpenoids and polyketides--Limonene and pinene degradation|Metabolism--Metabolism of terpenoids and polyketides--Insect hormone biosynthesis|Metabolism--Global and overview maps--Metabolic pathways|Metabolism--Global and overview maps--Biosynthesis of secondary metabolites|Metabolism--Global and overview maps--Microbial metabolism in diverse environments|Metabolism--Global and overview maps--Biosynthesis of antibiotics

[denovogenes]_107430	99.23	0.0	fpla:A4U99_17020	K02027	ABC.MS.S	multiple sugar transport system substrate-binding protein	--	--

[denovogenes]_107431	100.00	5e-180	shq:A0259_20940	K06156	gntU	Gnt-I system low-affinity gluconate transporter	--	--

[denovogenes]_107433	99.23	0.0	cfd:CFNIH1_01100	K13292	lgt, umpA	phosphatidylglycerol:prolipoprotein diacylglycerol transferase	2.-.-.-	--

[denovogenes]_107435	91.89	4e-175	lacy:A4V08_32645	K07058	K07058	membrane protein	--	--

[denovogenes]_107436	71.92	1e-118	cle:Clole_3045	K01962	accA	acetyl-CoA carboxylase carboxyl transferase subunit alpha	6.4.1.2	Metabolism--Lipid metabolism--Fatty acid biosynthesis|Metabolism--Carbohydrate metabolism--Pyruvate metabolism|Metabolism--Carbohydrate metabolism--Propanoate metabolism|Metabolism--Energy metabolism--Carbon fixation pathways in prokaryotes|Metabolism--Global and overview maps--Metabolic pathways|Metabolism--Global and overview maps--Biosynthesis of secondary metabolites|Metabolism--Global and overview maps--Microbial metabolism in diverse environments|Metabolism--Global and overview maps--Biosynthesis of antibiotics|Metabolism--Global and overview maps--Carbon metabolism|Metabolism--Global and overview maps--Fatty acid metabolism

[denovogenes]_107439	99.62	0.0	cfd:CFNIH1_08250	K03759	adiC	arginine:agmatine antiporter	--	--

[denovogenes]_107442	100.00	0.0	ecv:APECO1_2544	K02446	glpX	fructose-1,6-bisphosphatase II	3.1.3.11	Metabolism--Carbohydrate metabolism--Glycolysis / Gluconeogenesis|Metabolism--Carbohydrate metabolism--Pentose phosphate pathway|Metabolism--Carbohydrate metabolism--Fructose and mannose metabolism|Metabolism--Energy metabolism--Methane metabolism|Metabolism--Energy metabolism--Carbon fixation in photosynthetic organisms|Metabolism--Global and overview maps--Metabolic pathways|Metabolism--Global and overview maps--Biosynthesis of secondary metabolites|Metabolism--Global and overview maps--Microbial metabolism in diverse environments|Metabolism--Global and overview maps--Biosynthesis of antibiotics|Metabolism--Global and overview maps--Carbon metabolism

[denovogenes]_107443	100.00	0.0	pdi:BDI_2591	K03269	lpxH	UDP-2,3-diacylglucosamine hydrolase	3.6.1.54	Metabolism--Glycan biosynthesis and metabolism--Lipopolysaccharide biosynthesis|Metabolism--Global and overview maps--Metabolic pathways

[denovogenes]_107445	50.97	2e-81	cck:Ccar_01040	K03750	moeA	molybdopterin molybdotransferase	2.10.1.1	Metabolism--Metabolism of cofactors and vitamins--Folate biosynthesis|Metabolism--Global and overview maps--Metabolic pathways

[denovogenes]_107446	66.92	2e-130	fro:AALO17_03980	K00874	kdgK	2-dehydro-3-deoxygluconokinase	2.7.1.45	Metabolism--Carbohydrate metabolism--Pentose phosphate pathway|Metabolism--Global and overview maps--Metabolic pathways|Metabolism--Global and overview maps--Microbial metabolism in diverse environments|Metabolism--Global and overview maps--Carbon metabolism

[denovogenes]_107448	57.25	7e-98	cbk:CLL_A3042	K02598	nirC	nitrite transporter NirC	--	--

[denovogenes]_10745	96.77	0.0	bfb:VU15_02065	K01547	kdpB	K+-transporting ATPase ATPase B chain	3.6.3.12	Environmental Information Processing--Signal transduction--Two-component system

[denovogenes]_107453	95.08	0.0	spen:107025923	K08913	--	--	--	Metabolism--Energy metabolism--Photosynthesis - antenna proteins|Metabolism--Global and overview maps--Metabolic pathways

[denovogenes]_107455	56.98	3e-96	btho:Btheta7330_01639	K03455	TC.KEF	monovalent cation:H+ antiporter-2, CPA2 family	--	--

[denovogenes]_107456	55.43	5e-88	bprs:CK3_10760	K09807	K09807	uncharacterized protein	--	--

[denovogenes]_107457	54.62	1e-93	mbj:KQ51_01759	K00852	rbsK, RBKS	ribokinase	2.7.1.15	Metabolism--Carbohydrate metabolism--Pentose phosphate pathway

[denovogenes]_107458	70.77	7e-119	fpla:A4U99_17610	K07391	comM	magnesium chelatase family protein	--	--

[denovogenes]_107459	78.85	1e-145	bhl:Bache_1231	K05801	djlA	DnaJ like chaperone protein	--	--

[denovogenes]_107460	76.45	1e-147	cle:Clole_3506	K01151	nfo	deoxyribonuclease IV	3.1.21.2	Genetic Information Processing--Replication and repair--Base excision repair

[denovogenes]_107463	96.54	0.0	cfd:CFNIH1_24120	K05817	hcaR	LysR family transcriptional regulator, hca operon transcriptional activator	--	--

[denovogenes]_107464	55.77	7e-95	pob:LPB03_09725	K05970	SIAE	sialate O-acetylesterase	3.1.1.53	--

[denovogenes]_107465	40.00	2e-37	bfn:OI25_3596	K03566	gcvA	LysR family transcriptional regulator, glycine cleavage system transcriptional activator	--	Cellular Processes--Cellular community - prokaryotes--Biofilm formation - Escherichia coli

[denovogenes]_107468	71.54	8e-133	euc:EC1_01160	K01955	carB, CPA2	carbamoyl-phosphate synthase large subunit	6.3.5.5	Metabolism--Nucleotide metabolism--Pyrimidine metabolism|Metabolism--Amino acid metabolism--Alanine, aspartate and glutamate metabolism|Metabolism--Global and overview maps--Metabolic pathways

[denovogenes]_107469	82.79	4e-121	cfd:CFNIH1_10810	K13009	wzy	O-antigen polymerase	2.4.1.-	--

[denovogenes]_10747	99.56	0.0	cfd:CFNIH1_00220	K16554	exoP, vpsO	polysaccharide biosynthesis transport protein	--	Cellular Processes--Cellular community - prokaryotes--Biofilm formation - Vibrio cholerae

[denovogenes]_107470	85.38	1e-161	ibu:IB211_02689	K01950	E6.3.5.1, NADSYN1, QNS1, nadE	NAD+ synthase (glutamine-hydrolysing)	6.3.5.1	Metabolism--Metabolism of cofactors and vitamins--Nicotinate and nicotinamide metabolism|Metabolism--Global and overview maps--Metabolic pathways

[denovogenes]_107473	50.19	2e-82	saun:SAKOR_01178	K03168	topA	DNA topoisomerase I	5.99.1.2	--

[denovogenes]_107475	74.49	5e-132	lacy:A4V08_05385	K03575	mutY	A/G-specific adenine glycosylase	3.2.2.-	Genetic Information Processing--Replication and repair--Base excision repair

[denovogenes]_107478	48.94	2e-70	lacy:A4V08_12100	K09133	K09133	uncharacterized protein	--	--

[denovogenes]_107479	67.69	8e-134	cbt:CLH_1288	K01265	map	methionyl aminopeptidase	3.4.11.18	--

[denovogenes]_107480	57.69	4e-102	bah:BAMEG_4737	K01338	lon	ATP-dependent Lon protease	3.4.21.53	Cellular Processes--Cell growth and death--Cell cycle - Caulobacter

[denovogenes]_107481	63.08	2e-114	ova:OBV_24370	K02357	tsf, TSFM	elongation factor Ts	--	--

[denovogenes]_107483	44.40	5e-53	bant:A16_47930	K01890	FARSB, pheT	phenylalanyl-tRNA synthetase beta chain	6.1.1.20	Genetic Information Processing--Translation--Aminoacyl-tRNA biosynthesis

[denovogenes]_107489	93.12	2e-166	pfre:RM25_0070	K01990	ABC-2.A	ABC-2 type transport system ATP-binding protein	--	--

[denovogenes]_107490	97.68	0.0	pfre:RM25_1906	K02036	pstB	phosphate transport system ATP-binding protein	3.6.3.27	Environmental Information Processing--Membrane transport--ABC transporters

[denovogenes]_107491	77.35	5e-124	pfre:RM25_1234	K06997	yggS, PROSC	PLP dependent protein	--	--

[denovogenes]_107492	78.76	9e-143	pfr:PFREUD_10460	K02113	ATPF1D, atpH	F-type H+-transporting ATPase subunit delta	--	Metabolism--Energy metabolism--Oxidative phosphorylation|Metabolism--Energy metabolism--Photosynthesis|Metabolism--Global and overview maps--Metabolic pathways

[denovogenes]_107493	73.17	2e-84	pfre:RM25_1450	K00859	coaE	dephospho-CoA kinase	2.7.1.24	Metabolism--Metabolism of cofactors and vitamins--Pantothenate and CoA biosynthesis|Metabolism--Global and overview maps--Metabolic pathways

[denovogenes]_107494	44.40	3e-77	lae:LBAT_0590	K12410	npdA	NAD-dependent deacetylase	3.5.1.-	--

[denovogenes]_107496	57.83	1e-111	pgi:PG_0425	K01070	frmB, ESD, fghA	S-formylglutathione hydrolase	3.1.2.12	Metabolism--Energy metabolism--Methane metabolism|Metabolism--Global and overview maps--Microbial metabolism in diverse environments|Metabolism--Global and overview maps--Carbon metabolism

[denovogenes]_107497	40.16	3e-51	asb:RATSFB_1182	K18672	dacA	diadenylate cyclase	2.7.7.85	--

[denovogenes]_1075	90.76	0.0	pfre:RM25_0180	K16014	cydCD	ATP-binding cassette, subfamily C, bacterial CydCD	--	Environmental Information Processing--Membrane transport--ABC transporters

[denovogenes]_10750	99.41	0.0	ese:ECSF_0498	K11739	nfrA	bacteriophage N4 adsorption protein A	--	--

[denovogenes]_107500	100.00	0.0	paeo:M801_5537	K11066	E3.5.1.28D, amiD	N-acetylmuramoyl-L-alanine amidase	3.5.1.28	--

[denovogenes]_107501	63.57	1e-125	pdi:BDI_3476	K03426	E3.6.1.22, NUDT12, nudC	NAD+ diphosphatase	3.6.1.22	Metabolism--Metabolism of cofactors and vitamins--Nicotinate and nicotinamide metabolism|Metabolism--Global and overview maps--Metabolic pathways|Cellular Processes--Transport and catabolism--Peroxisome

[denovogenes]_107502	72.09	2e-139	pdi:BDI_2667	K01092	E3.1.3.25, IMPA, suhB	myo-inositol-1(or 4)-monophosphatase	3.1.3.25	Metabolism--Biosynthesis of other secondary metabolites--Streptomycin biosynthesis|Metabolism--Carbohydrate metabolism--Inositol phosphate metabolism|Metabolism--Global and overview maps--Metabolic pathways|Environmental Information Processing--Signal transduction--Phosphatidylinositol signaling system

[denovogenes]_107503	98.84	0.0	btho:Btheta7330_05045	K02477	K02477	two-component system, LytT family, response regulator	--	--

[denovogenes]_107504	48.54	1e-58	bpj:B2904_orf326	K07090	K07090	uncharacterized protein	--	--

[denovogenes]_107505	65.87	2e-97	fpa:FPR_29120	K02233	E2.7.8.26, cobS, cobV	adenosylcobinamide-GDP ribazoletransferase	2.7.8.26	Metabolism--Metabolism of cofactors and vitamins--Porphyrin and chlorophyll metabolism|Metabolism--Global and overview maps--Metabolic pathways

[denovogenes]_107506	100.00	0.0	paec:M802_4913	K00796	folP	dihydropteroate synthase	2.5.1.15	Metabolism--Metabolism of cofactors and vitamins--Folate biosynthesis|Metabolism--Global and overview maps--Metabolic pathways

[denovogenes]_107508	50.58	3e-87	csh:Closa_0633	K17836	penP	beta-lactamase class A	3.5.2.6	Metabolism--Biosynthesis of other secondary metabolites--Penicillin and cephalosporin biosynthesis|Metabolism--Global and overview maps--Biosynthesis of antibiotics|Human Diseases--Drug resistance: Antimicrobial--beta-Lactam resistance

[denovogenes]_10751	99.56	0.0	cfd:CFNIH1_04695	K06959	tex	protein Tex	--	--

[denovogenes]_107510	100.00	0.0	paeo:M801_5377	K01142	E3.1.11.2, xthA	exodeoxyribonuclease III	3.1.11.2	Genetic Information Processing--Replication and repair--Base excision repair

[denovogenes]_107511	83.20	8e-162	dfi:AXF13_01510	K02049	ABC.SN.A	NitT/TauT family transport system ATP-binding protein	--	--

[denovogenes]_107514	100.00	0.0	cfd:CFNIH1_09465	K19508	PTS-Gfr-EIIC, gfrC	PTS system, fructoselysine/glucoselysine-specific IIC component	--	Environmental Information Processing--Membrane transport--Phosphotransferase system (PTS)

[denovogenes]_107515	78.35	9e-145	sbc:SbBS512_E1900	K03521	fixA, etfB	electron transfer flavoprotein beta subunit	--	--

[denovogenes]_107516	58.17	2e-94	dgg:DGI_4025	K03496	parA, soj	chromosome partitioning protein	--	--

[denovogenes]_107517	100.00	0.0	paeg:AI22_26180	K09861	K09861	uncharacterized protein	--	--

[denovogenes]_107518	63.37	5e-113	lacy:A4V08_19695	K04069	pflA, pflC, pflE	pyruvate formate lyase activating enzyme	1.97.1.4	--

[denovogenes]_107519	99.61	0.0	cfd:CFNIH1_04350	K02654	pilD, pppA	leader peptidase (prepilin peptidase) / N-methyltransferase	3.4.23.43 2.1.1.-	--

[denovogenes]_107521	66.27	3e-126	brm:Bmur_0155	K15555	ssuB	sulfonate transport system ATP-binding protein	3.6.3.-	Metabolism--Energy metabolism--Sulfur metabolism|Environmental Information Processing--Membrane transport--ABC transporters

[denovogenes]_107522	43.63	2e-77	tae:TepiRe1_2387	K11534	deoR	DeoR family transcriptional regulator, deoxyribose operon repressor	--	--

[denovogenes]_107523	97.68	0.0	cfd:CFNIH1_16680	K02013	ABC.FEV.A	iron complex transport system ATP-binding protein	3.6.3.34	Environmental Information Processing--Membrane transport--ABC transporters

[denovogenes]_107524	47.47	5e-77	cso:CLS_02710	K00979	kdsB	3-deoxy-manno-octulosonate cytidylyltransferase (CMP-KDO synthetase)	2.7.7.38	Metabolism--Glycan biosynthesis and metabolism--Lipopolysaccharide biosynthesis|Metabolism--Global and overview maps--Metabolic pathways

[denovogenes]_107525	100.00	0.0	paeo:M801_3552	K03424	--	--	--	--

[denovogenes]_107526	55.91	4e-101	cpor:BED41_13925	K01995	livG	branched-chain amino acid transport system ATP-binding protein	--	Environmental Information Processing--Membrane transport--ABC transporters|Cellular Processes--Cellular community - prokaryotes--Quorum sensing

[denovogenes]_107528	96.14	1e-163	lacy:A4V08_18580	K02018	modB	molybdate transport system permease protein	--	Environmental Information Processing--Membrane transport--ABC transporters

[denovogenes]_107529	49.55	8e-66	pprc:PFLCHA0_c15010	K15540	ecpD	chaperone protein EcpD	--	--

[denovogenes]_107534	41.37	2e-54	tnp:Tnap_1082	K00059	fabG	3-oxoacyl-[acyl-carrier protein] reductase	1.1.1.100	Metabolism--Lipid metabolism--Fatty acid biosynthesis|Metabolism--Metabolism of cofactors and vitamins--Biotin metabolism|Metabolism--Lipid metabolism--Biosynthesis of unsaturated fatty acids|Metabolism--Global and overview maps--Metabolic pathways|Metabolism--Global and overview maps--Fatty acid metabolism

[denovogenes]_107535	98.46	1e-176	fpla:A4U99_09750	K02598	nirC	nitrite transporter NirC	--	--

[denovogenes]_107536	50.80	7e-82	atm:ANT_11860	K02566	nagD	NagD protein	--	--

[denovogenes]_107537	75.98	7e-139	pdi:BDI_0805	K02477	K02477	two-component system, LytT family, response regulator	--	--

[denovogenes]_107538	63.39	7e-111	doa:AXF15_07765	K02566	nagD	NagD protein	--	--

[denovogenes]_107545	80.69	3e-152	daf:Desaf_1449	K02500	hisF	cyclase	4.1.3.-	Metabolism--Amino acid metabolism--Histidine metabolism|Metabolism--Global and overview maps--Metabolic pathways|Metabolism--Global and overview maps--Biosynthesis of secondary metabolites|Metabolism--Global and overview maps--Biosynthesis of amino acids

[denovogenes]_107546	77.52	1e-139	cpy:Cphy_0713	K07238	TC.ZIP, zupT, ZRT3, ZIP2	zinc transporter, ZIP family	--	--

[denovogenes]_107548	100.00	0.0	fpla:A4U99_01095	K02050	ABC.SN.P	NitT/TauT family transport system permease protein	--	--

[denovogenes]_10755	95.75	0.0	boa:Bovatus_00864	K01190	lacZ	beta-galactosidase	3.2.1.23	Metabolism--Carbohydrate metabolism--Galactose metabolism|Metabolism--Glycan biosynthesis and metabolism--Other glycan degradation|Metabolism--Lipid metabolism--Sphingolipid metabolism|Metabolism--Global and overview maps--Metabolic pathways

[denovogenes]_107550	100.00	0.0	paeo:M801_1262	K00355	NQO1	NAD(P)H dehydrogenase (quinone)	1.6.5.2	Metabolism--Metabolism of cofactors and vitamins--Ubiquinone and other terpenoid-quinone biosynthesis|Metabolism--Global and overview maps--Biosynthesis of secondary metabolites|Human Diseases--Cardiovascular diseases--Fluid shear stress and atherosclerosis

[denovogenes]_107551	97.68	0.0	lacy:A4V08_26985	K07720	yesN	two-component system, response regulator YesN	--	Environmental Information Processing--Signal transduction--Two-component system

[denovogenes]_107555	100.00	0.0	sgi:SGRAN_1329	K20266	trbJ	type IV secretion system protein TrbJ	--	Cellular Processes--Cellular community - prokaryotes--Quorum sensing

[denovogenes]_107557	46.00	3e-72	csq:CSCA_3692	K01715	crt	enoyl-CoA hydratase	4.2.1.17	Metabolism--Carbohydrate metabolism--Butanoate metabolism|Metabolism--Global and overview maps--Carbon metabolism

[denovogenes]_10756	98.83	0.0	boa:Bovatus_01906	K01277	DPP3	dipeptidyl-peptidase III	3.4.14.4	--

[denovogenes]_107561	100.00	0.0	paer:PA1R_gp0632	K01992	ABC-2.P	ABC-2 type transport system permease protein	--	--

[denovogenes]_107566	95.75	0.0	cko:CKO_01189	K03603	fadR	GntR family transcriptional regulator, negative regulator for fad regulon and positive regulator of fabA	--	--

[denovogenes]_107567	100.00	0.0	paeg:AI22_29405	K05921	hpaG	5-oxopent-3-ene-1,2,5-tricarboxylate decarboxylase / 2-hydroxyhepta-2,4-diene-1,7-dioate isomerase	4.1.1.68 5.3.3.-	Metabolism--Amino acid metabolism--Tyrosine metabolism|Metabolism--Global and overview maps--Microbial metabolism in diverse environments|Metabolism--Global and overview maps--Degradation of aromatic compounds

[denovogenes]_107568	65.08	4e-123	clj:CLJU_c23050	K02588	nifH	nitrogenase iron protein NifH	1.18.6.1	Metabolism--Xenobiotics biodegradation and metabolism--Chloroalkane and chloroalkene degradation|Metabolism--Energy metabolism--Nitrogen metabolism|Metabolism--Global and overview maps--Metabolic pathways|Metabolism--Global and overview maps--Microbial metabolism in diverse environments

[denovogenes]_107569	100.00	0.0	pdk:PADK2_12335	K00453	E1.13.11.11, TDO2	tryptophan 2,3-dioxygenase	1.13.11.11	Metabolism--Amino acid metabolism--Tryptophan metabolism|Metabolism--Global and overview maps--Metabolic pathways

[denovogenes]_107570	56.25	2e-86	clc:Calla_1939	K02419	fliP	flagellar biosynthetic protein FliP	--	Cellular Processes--Cell motility--Flagellar assembly

[denovogenes]_107571	40.00	4e-63	sequ:Q426_07000	K02530	lacR	DeoR family transcriptional regulator, lactose phosphotransferase system repressor	--	--

[denovogenes]_107572	99.61	0.0	lacy:A4V08_25895	K00757	udp, UPP	uridine phosphorylase	2.4.2.3	Metabolism--Nucleotide metabolism--Pyrimidine metabolism|Metabolism--Xenobiotics biodegradation and metabolism--Drug metabolism - other enzymes|Metabolism--Global and overview maps--Metabolic pathways

[denovogenes]_107573	43.03	1e-34	hye:AM218_06300	K00566	mnmA, trmU	tRNA-uridine 2-sulfurtransferase	2.8.1.13	Genetic Information Processing--Folding, sorting and degradation--Sulfur relay system

[denovogenes]_107576	99.22	0.0	lacy:A4V08_13270	K02835	prfA, MTRF1, MRF1	peptide chain release factor 1	--	--

[denovogenes]_107577	42.23	9e-36	thu:AC731_008520	K00038	E1.1.1.53	3alpha(or 20beta)-hydroxysteroid dehydrogenase	1.1.1.53	Metabolism--Lipid metabolism--Steroid hormone biosynthesis|Metabolism--Global and overview maps--Metabolic pathways

[denovogenes]_107578	97.31	0.0	bfb:VU15_08065	K03799	htpX	heat shock protein HtpX	3.4.24.-	--

[denovogenes]_107580	100.00	0.0	efd:EFD32_0865	K02049	ABC.SN.A	NitT/TauT family transport system ATP-binding protein	--	--

[denovogenes]_107582	100.00	0.0	paec:M802_4058	K02073	metQ	D-methionine transport system substrate-binding protein	--	Environmental Information Processing--Membrane transport--ABC transporters

[denovogenes]_107583	70.38	1e-128	cgo:Corgl_1801	K03217	yidC, spoIIIJ, OXA1, ccfA	YidC/Oxa1 family membrane protein insertase	--	Cellular Processes--Cellular community - prokaryotes--Quorum sensing|Genetic Information Processing--Folding, sorting and degradation--Protein export|Environmental Information Processing--Membrane transport--Bacterial secretion system

[denovogenes]_107584	99.23	0.0	cfd:CFNIH1_04640	K12288	hofM	pilus assembly protein HofM	--	--

[denovogenes]_107585	86.87	1e-162	byl:A4V09_19035	K03525	coaX	type III pantothenate kinase	2.7.1.33	Metabolism--Metabolism of cofactors and vitamins--Pantothenate and CoA biosynthesis|Metabolism--Global and overview maps--Metabolic pathways

[denovogenes]_107586	57.63	9e-97	badl:BADO_1673	K01191	E3.2.1.24	alpha-mannosidase	3.2.1.24	Metabolism--Glycan biosynthesis and metabolism--Other glycan degradation

[denovogenes]_107587	70.66	2e-126	ehi:EHI_023110	K00002	AKR1A1, adh	alcohol dehydrogenase (NADP+)	1.1.1.2	Metabolism--Carbohydrate metabolism--Glycolysis / Gluconeogenesis|Metabolism--Carbohydrate metabolism--Pentose and glucuronate interconversions|Metabolism--Lipid metabolism--Glycerolipid metabolism|Metabolism--Xenobiotics biodegradation and metabolism--Caprolactam degradation|Metabolism--Global and overview maps--Metabolic pathways|Metabolism--Global and overview maps--Biosynthesis of secondary metabolites|Metabolism--Global and overview maps--Microbial metabolism in diverse environments|Metabolism--Global and overview maps--Biosynthesis of antibiotics|Metabolism--Global and overview maps--Degradation of aromatic compounds

[denovogenes]_107588	99.61	0.0	cfd:CFNIH1_20660	K01087	otsB	trehalose 6-phosphate phosphatase	3.1.3.12	Metabolism--Carbohydrate metabolism--Starch and sucrose metabolism|Metabolism--Global and overview maps--Metabolic pathways

[denovogenes]_107592	100.00	0.0	kin:AB182_00355	K12204	dotC, traI	defect in organelle trafficking protein DotC	--	--

[denovogenes]_107595	72.16	3e-122	bdh:GV66_13870	K08218	ampG	MFS transporter, PAT family, beta-lactamase induction signal transducer AmpG	--	Human Diseases--Drug resistance: Antimicrobial--beta-Lactam resistance

[denovogenes]_107598	100.00	0.0	bbrd:BBBR_0870	K09013	sufC	Fe-S cluster assembly ATP-binding protein	--	--

[denovogenes]_107599	79.51	5e-141	elm:ELI_3913	K02007	cbiM	cobalt/nickel transport system permease protein	--	Environmental Information Processing--Membrane transport--ABC transporters

[denovogenes]_1076	81.15	0.0	lacy:A4V08_14880	K03723	mfd	transcription-repair coupling factor (superfamily II helicase)	3.6.4.-	Genetic Information Processing--Replication and repair--Nucleotide excision repair

[denovogenes]_107600	86.46	2e-146	csh:Closa_1919	K03685	rnc, DROSHA, RNT1	ribonuclease III	3.1.26.3	Genetic Information Processing--Translation--Ribosome biogenesis in eukaryotes|Human Diseases--Cancers: Overview--Proteoglycans in cancer

[denovogenes]_107601	41.55	1e-41	tbo:Thebr_0613	K02444	glpR	DeoR family transcriptional regulator, glycerol-3-phosphate regulon repressor	--	--

[denovogenes]_107603	86.90	1e-147	byl:A4V09_17910	K02863	RP-L1, MRPL1, rplA	large subunit ribosomal protein L1	--	Genetic Information Processing--Translation--Ribosome

[denovogenes]_107604	78.95	7e-139	ele:Elen_2030	K02010	afuC, fbpC	iron(III) transport system ATP-binding protein	3.6.3.30	Environmental Information Processing--Membrane transport--ABC transporters

[denovogenes]_107607	72.69	9e-135	eyy:EGYY_17850	K00313	fixC	electron transfer flavoprotein-quinone oxidoreductase	1.5.5.-	--

[denovogenes]_107608	100.00	0.0	cfd:CFNIH1_17485	K04775	ydgD	protease YdgD	3.4.21.-	--

[denovogenes]_107609	42.02	3e-63	len:LEP3755_44500	K06338	cotSA	spore coat protein SA	--	--

[denovogenes]_10761	99.85	0.0	mmk:MU9_1949	K03797	E3.4.21.102, prc, ctpA	carboxyl-terminal processing protease	3.4.21.102	--

[denovogenes]_107610	100.00	0.0	pdi:BDI_2559	K03496	parA, soj	chromosome partitioning protein	--	--

[denovogenes]_107611	100.00	0.0	cfd:CFNIH1_10675	K02071	metN	D-methionine transport system ATP-binding protein	--	Environmental Information Processing--Membrane transport--ABC transporters

[denovogenes]_107613	99.61	0.0	cfd:CFNIH1_10155	K02563	murG	UDP-N-acetylglucosamine--N-acetylmuramyl-(pentapeptide) pyrophosphoryl-undecaprenol N-acetylglucosamine transferase	2.4.1.227	Metabolism--Glycan biosynthesis and metabolism--Peptidoglycan biosynthesis|Metabolism--Global and overview maps--Metabolic pathways|Human Diseases--Drug resistance: Antimicrobial--Vancomycin resistance|Cellular Processes--Cell growth and death--Cell cycle - Caulobacter

[denovogenes]_107616	58.40	4e-102	fpla:A4U99_09725	K02016	ABC.FEV.S	iron complex transport system substrate-binding protein	--	Environmental Information Processing--Membrane transport--ABC transporters

[denovogenes]_107618	44.84	2e-54	ccu:Ccur_05200	K03734	apbE	FAD:protein FMN transferase	2.7.1.180	--

[denovogenes]_107619	51.17	5e-95	rix:RO1_26250	K01092	E3.1.3.25, IMPA, suhB	myo-inositol-1(or 4)-monophosphatase	3.1.3.25	Metabolism--Biosynthesis of other secondary metabolites--Streptomycin biosynthesis|Metabolism--Carbohydrate metabolism--Inositol phosphate metabolism|Metabolism--Global and overview maps--Metabolic pathways|Environmental Information Processing--Signal transduction--Phosphatidylinositol signaling system

[denovogenes]_10762	99.85	0.0	bfb:VU15_10640	K04079	HSP90A, htpG	molecular chaperone HtpG	--	Genetic Information Processing--Folding, sorting and degradation--Protein processing in endoplasmic reticulum|Environmental Information Processing--Signal transduction--PI3K-Akt signaling pathway|Organismal Systems--Immune system--Antigen processing and presentation|Organismal Systems--Immune system--NOD-like receptor signaling pathway|Organismal Systems--Environmental adaptation--Plant-pathogen interaction|Organismal Systems--Immune system--IL-17 signaling pathway|Organismal Systems--Immune system--Th17 cell differentiation|Organismal Systems--Endocrine system--Progesterone-mediated oocyte maturation|Organismal Systems--Endocrine system--Estrogen signaling pathway|Human Diseases--Cancers: Overview--Pathways in cancer|Human Diseases--Cancers: Specific types--Prostate cancer|Human Diseases--Cardiovascular diseases--Fluid shear stress and atherosclerosis

[denovogenes]_107621	45.35	2e-66	loki:Lokiarch_41410	K01955	carB, CPA2	carbamoyl-phosphate synthase large subunit	6.3.5.5	Metabolism--Nucleotide metabolism--Pyrimidine metabolism|Metabolism--Amino acid metabolism--Alanine, aspartate and glutamate metabolism|Metabolism--Global and overview maps--Metabolic pathways

[denovogenes]_107622	100.00	0.0	cfd:CFNIH1_01610	K00927	PGK, pgk	phosphoglycerate kinase	2.7.2.3	Metabolism--Carbohydrate metabolism--Glycolysis / Gluconeogenesis|Metabolism--Energy metabolism--Carbon fixation in photosynthetic organisms|Metabolism--Global and overview maps--Metabolic pathways|Metabolism--Global and overview maps--Biosynthesis of secondary metabolites|Metabolism--Global and overview maps--Microbial metabolism in diverse environments|Metabolism--Global and overview maps--Biosynthesis of antibiotics|Metabolism--Global and overview maps--Carbon metabolism|Metabolism--Global and overview maps--Biosynthesis of amino acids

[denovogenes]_107623	66.15	4e-129	bxy:BXY_06120	K01903	sucC	succinyl-CoA synthetase beta subunit	6.2.1.5	Metabolism--Carbohydrate metabolism--Citrate cycle (TCA cycle)|Metabolism--Carbohydrate metabolism--Propanoate metabolism|Metabolism--Carbohydrate metabolism--C5-Branched dibasic acid metabolism|Metabolism--Energy metabolism--Carbon fixation pathways in prokaryotes|Metabolism--Global and overview maps--Metabolic pathways|Metabolism--Global and overview maps--Biosynthesis of secondary metabolites|Metabolism--Global and overview maps--Microbial metabolism in diverse environments|Metabolism--Global and overview maps--Biosynthesis of antibiotics|Metabolism--Global and overview maps--Carbon metabolism

[denovogenes]_107625	41.02	1e-63	ccb:Clocel_1517	K00852	rbsK, RBKS	ribokinase	2.7.1.15	Metabolism--Carbohydrate metabolism--Pentose phosphate pathway

[denovogenes]_107626	89.62	2e-173	bfb:VU15_04135	K01951	guaA, GMPS	GMP synthase (glutamine-hydrolysing)	6.3.5.2	Metabolism--Nucleotide metabolism--Purine metabolism|Metabolism--Xenobiotics biodegradation and metabolism--Drug metabolism - other enzymes|Metabolism--Global and overview maps--Metabolic pathways

[denovogenes]_107627	95.37	4e-178	pvl:AOB99_13730	K03272	gmhC, hldE, waaE, rfaE	D-beta-D-heptose 7-phosphate kinase / D-beta-D-heptose 1-phosphate adenosyltransferase	2.7.1.167 2.7.7.70	Metabolism--Glycan biosynthesis and metabolism--Lipopolysaccharide biosynthesis|Metabolism--Global and overview maps--Metabolic pathways

[denovogenes]_107632	95.75	0.0	pmib:BB2000_1894	K01963	accD	acetyl-CoA carboxylase carboxyl transferase subunit beta	6.4.1.2	Metabolism--Lipid metabolism--Fatty acid biosynthesis|Metabolism--Carbohydrate metabolism--Pyruvate metabolism|Metabolism--Carbohydrate metabolism--Propanoate metabolism|Metabolism--Energy metabolism--Carbon fixation pathways in prokaryotes|Metabolism--Global and overview maps--Metabolic pathways|Metabolism--Global and overview maps--Biosynthesis of secondary metabolites|Metabolism--Global and overview maps--Microbial metabolism in diverse environments|Metabolism--Global and overview maps--Biosynthesis of antibiotics|Metabolism--Global and overview maps--Carbon metabolism|Metabolism--Global and overview maps--Fatty acid metabolism

[denovogenes]_107633	59.07	2e-104	rbc:BN938_1068	K06147	ABCB-BAC	ATP-binding cassette, subfamily B, bacterial	--	--

[denovogenes]_107636	40.45	2e-38	tsi:TSIB_0632	K03308	TC.NSS	neurotransmitter:Na+ symporter, NSS family	--	--

[denovogenes]_107637	60.15	1e-105	tfo:BFO_0156	K02005	ABC.CD.TX	HlyD family secretion protein	--	--

[denovogenes]_10764	58.68	0.0	ega:AL523_10855	K03316	TC.CPA1	monovalent cation:H+ antiporter, CPA1 family	--	--

[denovogenes]_107640	98.01	0.0	boa:Bovatus_03338	K09815	znuA	zinc transport system substrate-binding protein	--	Environmental Information Processing--Membrane transport--ABC transporters

[denovogenes]_107645	50.44	2e-77	mfs:MFS40622_0186	K05936	cobM, cbiF	precorrin-4/cobalt-precorrin-4 C11-methyltransferase	2.1.1.133 2.1.1.271	Metabolism--Metabolism of cofactors and vitamins--Porphyrin and chlorophyll metabolism|Metabolism--Global and overview maps--Metabolic pathways

[denovogenes]_107647	83.52	6e-155	rum:CK1_35510	K01835	pgm	phosphoglucomutase	5.4.2.2	Metabolism--Carbohydrate metabolism--Glycolysis / Gluconeogenesis|Metabolism--Carbohydrate metabolism--Pentose phosphate pathway|Metabolism--Carbohydrate metabolism--Galactose metabolism|Metabolism--Nucleotide metabolism--Purine metabolism|Metabolism--Carbohydrate metabolism--Starch and sucrose metabolism|Metabolism--Carbohydrate metabolism--Amino sugar and nucleotide sugar metabolism|Metabolism--Biosynthesis of other secondary metabolites--Streptomycin biosynthesis|Metabolism--Global and overview maps--Metabolic pathways|Metabolism--Global and overview maps--Biosynthesis of secondary metabolites|Metabolism--Global and overview maps--Microbial metabolism in diverse environments|Metabolism--Global and overview maps--Biosynthesis of antibiotics

[denovogenes]_107651	61.09	1e-98	bfr:BF3231	K02343	DPO3G, dnaX	DNA polymerase III subunit gamma/tau	2.7.7.7	Metabolism--Nucleotide metabolism--Purine metabolism|Metabolism--Nucleotide metabolism--Pyrimidine metabolism|Metabolism--Global and overview maps--Metabolic pathways|Genetic Information Processing--Replication and repair--DNA replication|Genetic Information Processing--Replication and repair--Mismatch repair|Genetic Information Processing--Replication and repair--Homologous recombination

[denovogenes]_107654	59.61	1e-98	thx:Thet_0796	K18331	hndC	NADP-reducing hydrogenase subunit HndC	1.12.1.3	--

[denovogenes]_107657	96.54	0.0	bhl:Bache_3313	K01647	CS, gltA	citrate synthase	2.3.3.1	Metabolism--Carbohydrate metabolism--Citrate cycle (TCA cycle)|Metabolism--Carbohydrate metabolism--Glyoxylate and dicarboxylate metabolism|Metabolism--Global and overview maps--Metabolic pathways|Metabolism--Global and overview maps--Biosynthesis of secondary metabolites|Metabolism--Global and overview maps--Microbial metabolism in diverse environments|Metabolism--Global and overview maps--Biosynthesis of antibiotics|Metabolism--Global and overview maps--Carbon metabolism|Metabolism--Global and overview maps--2-Oxocarboxylic acid metabolism|Metabolism--Global and overview maps--Biosynthesis of amino acids

[denovogenes]_107659	74.81	8e-123	cgo:Corgl_0892	K03526	gcpE, ispG	(E)-4-hydroxy-3-methylbut-2-enyl-diphosphate synthase	1.17.7.1 1.17.7.3	Metabolism--Metabolism of terpenoids and polyketides--Terpenoid backbone biosynthesis|Metabolism--Global and overview maps--Metabolic pathways|Metabolism--Global and overview maps--Biosynthesis of secondary metabolites|Metabolism--Global and overview maps--Biosynthesis of antibiotics

[denovogenes]_107660	55.38	1e-102	ctc:CTC_01941	K00557	trmA	tRNA (uracil-5-)-methyltransferase	2.1.1.35	--

[denovogenes]_107662	44.40	7e-66	saz:Sama_1319	K03309	TC.DAACS	dicarboxylate/amino acid:cation (Na+ or H+) symporter, DAACS family	--	--

[denovogenes]_107663	45.53	2e-24	cak:Caul_4546	K07657	phoB	two-component system, OmpR family, phosphate regulon response regulator PhoB	--	Environmental Information Processing--Signal transduction--Two-component system

[denovogenes]_107664	63.32	2e-115	rmn:TK49_00210	K06020	E3.6.3.25	sulfate-transporting ATPase	3.6.3.25	--

[denovogenes]_107666	99.62	0.0	cfd:CFNIH1_19535	K02492	hemA	glutamyl-tRNA reductase	1.2.1.70	Metabolism--Metabolism of cofactors and vitamins--Porphyrin and chlorophyll metabolism|Metabolism--Global and overview maps--Metabolic pathways|Metabolism--Global and overview maps--Biosynthesis of secondary metabolites

[denovogenes]_10767	99.71	0.0	pvl:AOB99_05075	K02802	PTS-Nag-EIIA, nagE	PTS system, N-acetylglucosamine-specific IIA component	2.7.1.193	Metabolism--Carbohydrate metabolism--Amino sugar and nucleotide sugar metabolism|Environmental Information Processing--Membrane transport--Phosphotransferase system (PTS)

[denovogenes]_10767	99.71	0.0	pvl:AOB99_05075	K02803	PTS-Nag-EIIB, nagE	PTS system, N-acetylglucosamine-specific IIB component	2.7.1.193	Metabolism--Carbohydrate metabolism--Amino sugar and nucleotide sugar metabolism|Environmental Information Processing--Membrane transport--Phosphotransferase system (PTS)

[denovogenes]_10767	99.71	0.0	pvl:AOB99_05075	K02804	PTS-Nag-EIIC, nagE	PTS system, N-acetylglucosamine-specific IIC component	--	Metabolism--Carbohydrate metabolism--Amino sugar and nucleotide sugar metabolism|Environmental Information Processing--Membrane transport--Phosphotransferase system (PTS)

[denovogenes]_107675	85.00	2e-168	pdi:BDI_2651	K00784	rnz	ribonuclease Z	3.1.26.11	Genetic Information Processing--Translation--RNA transport

[denovogenes]_107676	80.31	4e-157	pec:W5S_2209	K03169	topB	DNA topoisomerase III	5.99.1.2	--

[denovogenes]_107677	45.53	4e-73	mpc:Mar181_0508	K03606	wcaJ	putative colanic acid biosysnthesis UDP-glucose lipid carrier transferase	--	Cellular Processes--Cellular community - prokaryotes--Biofilm formation - Vibrio cholerae

[denovogenes]_10768	89.88	0.0	cbv:U729_1339	K03043	rpoB	DNA-directed RNA polymerase subunit beta	2.7.7.6	Metabolism--Nucleotide metabolism--Purine metabolism|Metabolism--Nucleotide metabolism--Pyrimidine metabolism|Metabolism--Global and overview maps--Metabolic pathways|Genetic Information Processing--Transcription--RNA polymerase

[denovogenes]_107681	86.92	2e-173	bcel:BcellWH2_02030	K01179	E3.2.1.4	endoglucanase	3.2.1.4	Metabolism--Carbohydrate metabolism--Starch and sucrose metabolism|Metabolism--Global and overview maps--Metabolic pathways

[denovogenes]_107685	76.36	4e-154	shg:Sph21_4379	K02517	lpxL, htrB	Kdo2-lipid IVA lauroyltransferase	2.3.1.241	Metabolism--Glycan biosynthesis and metabolism--Lipopolysaccharide biosynthesis|Metabolism--Global and overview maps--Metabolic pathways

[denovogenes]_107686	100.00	0.0	boa:Bovatus_02686	K07052	K07052	uncharacterized protein	--	--

[denovogenes]_107687	46.69	2e-88	erb:A4V01_09940	K03609	minD	septum site-determining protein MinD	--	--

[denovogenes]_10769	100.00	0.0	pvl:AOB99_15220	K02355	fusA, GFM, EFG	elongation factor G	--	--

[denovogenes]_107693	59.46	7e-109	fro:AALO17_26720	K04486	E3.1.3.15B	histidinol-phosphatase (PHP family)	3.1.3.15	Metabolism--Amino acid metabolism--Histidine metabolism|Metabolism--Global and overview maps--Metabolic pathways|Metabolism--Global and overview maps--Biosynthesis of secondary metabolites|Metabolism--Global and overview maps--Biosynthesis of amino acids

[denovogenes]_107698	48.47	1e-75	rim:ROI_29960	K01448	E3.5.1.28B, amiA, amiB, amiC	N-acetylmuramoyl-L-alanine amidase	3.5.1.28	Human Diseases--Drug resistance: Antimicrobial--Cationic antimicrobial peptide (CAMP) resistance

[denovogenes]_107699	100.00	0.0	boa:Bovatus_01396	K02342	DPO3E, dnaQ	DNA polymerase III subunit epsilon	2.7.7.7	Metabolism--Nucleotide metabolism--Purine metabolism|Metabolism--Nucleotide metabolism--Pyrimidine metabolism|Metabolism--Global and overview maps--Metabolic pathways|Genetic Information Processing--Replication and repair--DNA replication|Genetic Information Processing--Replication and repair--Mismatch repair|Genetic Information Processing--Replication and repair--Homologous recombination

[denovogenes]_1077	99.67	0.0	efd:EFD32_0920	K16899	addB	ATP-dependent helicase/nuclease subunit B	3.1.-.- 3.6.4.12	--

[denovogenes]_10770	100.00	0.0	pvl:AOB99_10045	K13821	putA	RHH-type transcriptional regulator, proline utilization regulon repressor / proline dehydrogenase / delta 1-pyrroline-5-carboxylate dehydrogenase	1.5.5.2 1.2.1.88	Metabolism--Amino acid metabolism--Alanine, aspartate and glutamate metabolism|Metabolism--Amino acid metabolism--Arginine and proline metabolism|Metabolism--Global and overview maps--Metabolic pathways|Metabolism--Global and overview maps--Biosynthesis of secondary metabolites|Metabolism--Global and overview maps--Biosynthesis of antibiotics

[denovogenes]_107700	48.44	6e-89	bch:Bcen2424_0876	K09690	ABC-2.LPSE.P	lipopolysaccharide transport system permease protein	--	Environmental Information Processing--Membrane transport--ABC transporters

[denovogenes]_107702	99.14	1e-169	bvu:BVU_1428	K12410	npdA	NAD-dependent deacetylase	3.5.1.-	--

[denovogenes]_107703	100.00	0.0	pvl:AOB99_12285	K03366	butA, budC	meso-butanediol dehydrogenase / (S,S)-butanediol dehydrogenase / diacetyl reductase	1.1.1.- 1.1.1.76 1.1.1.304	Metabolism--Carbohydrate metabolism--Butanoate metabolism

[denovogenes]_107704	40.31	3e-68	ag:AAC27537	K18788	wbbD	UDP-Gal:alpha-D-GlcNAc-diphosphoundecaprenol beta-1,3-galactosyltransferase	2.4.1.303	--

[denovogenes]_107708	62.93	3e-115	emu:EMQU_2429	K19504	gfrE	glucoselysine-6-phosphate deglycase	--	--

[denovogenes]_107709	100.00	0.0	ecm:EcSMS35_2828	K00068	srlD	sorbitol-6-phosphate 2-dehydrogenase	1.1.1.140	Metabolism--Carbohydrate metabolism--Fructose and mannose metabolism

[denovogenes]_107710	65.37	5e-128	fne:FSDG_02048	K00648	fabH	3-oxoacyl-[acyl-carrier-protein] synthase III	2.3.1.180	Metabolism--Lipid metabolism--Fatty acid biosynthesis|Metabolism--Global and overview maps--Metabolic pathways|Metabolism--Global and overview maps--Fatty acid metabolism

[denovogenes]_107711	79.62	4e-152	fhw:RN87_02310	K01784	galE, GALE	UDP-glucose 4-epimerase	5.1.3.2	Metabolism--Carbohydrate metabolism--Galactose metabolism|Metabolism--Carbohydrate metabolism--Amino sugar and nucleotide sugar metabolism|Metabolism--Global and overview maps--Metabolic pathways

[denovogenes]_107714	100.00	0.0	pvl:AOB99_01840	K07106	murQ	N-acetylmuramic acid 6-phosphate etherase	4.2.1.126	Metabolism--Carbohydrate metabolism--Amino sugar and nucleotide sugar metabolism|Metabolism--Global and overview maps--Metabolic pathways

[denovogenes]_107715	76.08	6e-143	vpr:Vpar_1088	K01803	TPI, tpiA	triosephosphate isomerase (TIM)	5.3.1.1	Metabolism--Carbohydrate metabolism--Glycolysis / Gluconeogenesis|Metabolism--Carbohydrate metabolism--Fructose and mannose metabolism|Metabolism--Carbohydrate metabolism--Inositol phosphate metabolism|Metabolism--Energy metabolism--Carbon fixation in photosynthetic organisms|Metabolism--Global and overview maps--Metabolic pathways|Metabolism--Global and overview maps--Biosynthesis of secondary metabolites|Metabolism--Global and overview maps--Microbial metabolism in diverse environments|Metabolism--Global and overview maps--Biosynthesis of antibiotics|Metabolism--Global and overview maps--Carbon metabolism|Metabolism--Global and overview maps--Biosynthesis of amino acids

[denovogenes]_107716	53.88	3e-95	cth:Cthe_1671	K06400	spoIVCA	site-specific DNA recombinase	--	--

[denovogenes]_107717	50.40	2e-86	eft:M395_03700	K16869	lipL	octanoyl-[GcvH]:protein N-octanoyltransferase	2.3.1.204	--

[denovogenes]_107718	76.15	1e-148	ecas:ECBG_01363	K02529	lacI, galR	LacI family transcriptional regulator	--	--

[denovogenes]_107719	99.62	0.0	elx:CDCO157_4564	K11202	PTS-Fru2-EIIB	PTS system, fructose-specific IIB-like component	2.7.1.-	--

[denovogenes]_107719	99.62	0.0	elx:CDCO157_4564	K11203	PTS-Fru2-EIIC	PTS system, fructose-specific IIC-like component	--	--

[denovogenes]_10772	66.86	0.0	ruk:A4V00_12220	K04759	feoB	ferrous iron transport protein B	--	--

[denovogenes]_107720	68.58	9e-133	emu:EMQU_1721	K00965	galT, GALT	UDPglucose--hexose-1-phosphate uridylyltransferase	2.7.7.12	Metabolism--Carbohydrate metabolism--Galactose metabolism|Metabolism--Carbohydrate metabolism--Amino sugar and nucleotide sugar metabolism|Metabolism--Global and overview maps--Metabolic pathways|Organismal Systems--Endocrine system--Prolactin signaling pathway

[denovogenes]_107721	41.99	1e-50	csc:Csac_0361	K01213	E3.2.1.67	galacturan 1,4-alpha-galacturonidase	3.2.1.67	Metabolism--Carbohydrate metabolism--Pentose and glucuronate interconversions|Metabolism--Global and overview maps--Metabolic pathways

[denovogenes]_107724	81.40	2e-153	cbv:U729_2803	K03706	codY	transcriptional pleiotropic repressor	--	--

[denovogenes]_107726	100.00	0.0	mmk:MU9_1674	K01265	map	methionyl aminopeptidase	3.4.11.18	--

[denovogenes]_107728	100.00	0.0	mmk:MU9_3519	K03796	bax	Bax protein	--	--

[denovogenes]_107729	99.61	0.0	bfb:VU15_16760	K03424	--	--	--	--

[denovogenes]_10773	99.85	0.0	bvu:BVU_3300	K04079	HSP90A, htpG	molecular chaperone HtpG	--	Genetic Information Processing--Folding, sorting and degradation--Protein processing in endoplasmic reticulum|Environmental Information Processing--Signal transduction--PI3K-Akt signaling pathway|Organismal Systems--Immune system--Antigen processing and presentation|Organismal Systems--Immune system--NOD-like receptor signaling pathway|Organismal Systems--Environmental adaptation--Plant-pathogen interaction|Organismal Systems--Immune system--IL-17 signaling pathway|Organismal Systems--Immune system--Th17 cell differentiation|Organismal Systems--Endocrine system--Progesterone-mediated oocyte maturation|Organismal Systems--Endocrine system--Estrogen signaling pathway|Human Diseases--Cancers: Overview--Pathways in cancer|Human Diseases--Cancers: Specific types--Prostate cancer|Human Diseases--Cardiovascular diseases--Fluid shear stress and atherosclerosis

[denovogenes]_107733	99.59	1e-179	mmk:MU9_1255	K09761	rsmE	16S rRNA (uracil1498-N3)-methyltransferase	2.1.1.193	--

[denovogenes]_107735	99.61	0.0	bfb:VU15_11015	K03149	thiG	thiazole synthase	2.8.1.10	Metabolism--Metabolism of cofactors and vitamins--Thiamine metabolism|Metabolism--Global and overview maps--Metabolic pathways

[denovogenes]_107736	100.00	0.0	bfs:BF9343_2522	K00978	rfbF	glucose-1-phosphate cytidylyltransferase	2.7.7.33	Metabolism--Carbohydrate metabolism--Starch and sucrose metabolism|Metabolism--Carbohydrate metabolism--Amino sugar and nucleotide sugar metabolism|Metabolism--Global and overview maps--Metabolic pathways

[denovogenes]_107738	100.00	0.0	mmk:MU9_805	K03832	tonB	periplasmic protein TonB	--	--

[denovogenes]_107741	79.76	9e-143	cbz:Cbs_4558	K02795	PTS-Man-EIIC, manY	PTS system, mannose-specific IIC component	--	Metabolism--Carbohydrate metabolism--Fructose and mannose metabolism|Metabolism--Carbohydrate metabolism--Amino sugar and nucleotide sugar metabolism|Metabolism--Global and overview maps--Metabolic pathways|Environmental Information Processing--Membrane transport--Phosphotransferase system (PTS)

[denovogenes]_107742	64.96	6e-117	csb:CLSA_c00420	K07124	K07124	uncharacterized protein	--	--

[denovogenes]_107743	100.00	0.0	mmk:MU9_337	K02036	pstB	phosphate transport system ATP-binding protein	3.6.3.27	Environmental Information Processing--Membrane transport--ABC transporters

[denovogenes]_107744	80.62	5e-151	lacy:A4V08_31445	K01715	crt	enoyl-CoA hydratase	4.2.1.17	Metabolism--Carbohydrate metabolism--Butanoate metabolism|Metabolism--Global and overview maps--Carbon metabolism

[denovogenes]_107745	95.74	0.0	lacy:A4V08_32845	K03091	SIG3.4	RNA polymerase sporulation-specific sigma factor	--	--

[denovogenes]_107747	100.00	0.0	mmk:MU9_969	K00991	ispD	2-C-methyl-D-erythritol 4-phosphate cytidylyltransferase	2.7.7.60	Metabolism--Metabolism of terpenoids and polyketides--Terpenoid backbone biosynthesis|Metabolism--Global and overview maps--Metabolic pathways|Metabolism--Global and overview maps--Biosynthesis of secondary metabolites|Metabolism--Global and overview maps--Biosynthesis of antibiotics

[denovogenes]_107748	71.49	5e-132	lacy:A4V08_02555	K00868	pdxK, pdxY	pyridoxine kinase	2.7.1.35	Metabolism--Metabolism of cofactors and vitamins--Vitamin B6 metabolism|Metabolism--Global and overview maps--Metabolic pathways

[denovogenes]_107749	100.00	0.0	bfb:VU15_10565	K01241	amn	AMP nucleosidase	3.2.2.4	Metabolism--Nucleotide metabolism--Purine metabolism

[denovogenes]_107750	100.00	0.0	bfb:VU15_10580	K02823	pyrDII	dihydroorotate dehydrogenase electron transfer subunit	--	Metabolism--Nucleotide metabolism--Pyrimidine metabolism|Metabolism--Global and overview maps--Metabolic pathways

[denovogenes]_107751	55.06	1e-92	csb:CLSA_c24970	K19116	cas5h	CRISPR-associated protein Cas5h	--	--

[denovogenes]_107756	88.28	3e-176	lacy:A4V08_12875	K00978	rfbF	glucose-1-phosphate cytidylyltransferase	2.7.7.33	Metabolism--Carbohydrate metabolism--Starch and sucrose metabolism|Metabolism--Carbohydrate metabolism--Amino sugar and nucleotide sugar metabolism|Metabolism--Global and overview maps--Metabolic pathways

[denovogenes]_107757	58.70	1e-56	cth:Cthe_0331	K04750	phnB	PhnB protein	--	--

[denovogenes]_107759	100.00	0.0	efn:DENG_03224	K06346	jag	spoIIIJ-associated protein	--	--

[denovogenes]_10776	94.73	0.0	fpla:A4U99_03145	K03407	cheA	two-component system, chemotaxis family, sensor kinase CheA	2.7.13.3	Environmental Information Processing--Signal transduction--Two-component system|Cellular Processes--Cell motility--Bacterial chemotaxis

[denovogenes]_107762	43.53	3e-63	cten:CANTEDRAFT_119904	K17742	SOU1	sorbose reductase	1.1.1.289	Metabolism--Carbohydrate metabolism--Fructose and mannose metabolism

[denovogenes]_107763	99.61	0.0	efi:OG1RF_11168	K06967	trmK	tRNA (adenine22-N1)-methyltransferase	2.1.1.217	--

[denovogenes]_107764	50.00	2e-86	smar:SM39_pSMC2_52	K12057	traF	conjugal transfer pilus assembly protein TraF	--	--

[denovogenes]_107768	78.68	2e-150	ega:AL523_00250	K01995	livG	branched-chain amino acid transport system ATP-binding protein	--	Environmental Information Processing--Membrane transport--ABC transporters|Cellular Processes--Cellular community - prokaryotes--Quorum sensing

[denovogenes]_107769	100.00	5e-178	stb:SGPB_0362	K01990	ABC-2.A	ABC-2 type transport system ATP-binding protein	--	--

[denovogenes]_10777	99.85	0.0	btho:Btheta7330_02532	K04079	HSP90A, htpG	molecular chaperone HtpG	--	Genetic Information Processing--Folding, sorting and degradation--Protein processing in endoplasmic reticulum|Environmental Information Processing--Signal transduction--PI3K-Akt signaling pathway|Organismal Systems--Immune system--Antigen processing and presentation|Organismal Systems--Immune system--NOD-like receptor signaling pathway|Organismal Systems--Environmental adaptation--Plant-pathogen interaction|Organismal Systems--Immune system--IL-17 signaling pathway|Organismal Systems--Immune system--Th17 cell differentiation|Organismal Systems--Endocrine system--Progesterone-mediated oocyte maturation|Organismal Systems--Endocrine system--Estrogen signaling pathway|Human Diseases--Cancers: Overview--Pathways in cancer|Human Diseases--Cancers: Specific types--Prostate cancer|Human Diseases--Cardiovascular diseases--Fluid shear stress and atherosclerosis

[denovogenes]_107770	54.17	1e-91	cdg:CDBI1_14125	K03478	chbG	chitin disaccharide deacetylase	3.5.1.105	--

[denovogenes]_107773	99.22	0.0	stb:SGPB_2026	K03497	parB, spo0J	chromosome partitioning protein, ParB family	--	--

[denovogenes]_107776	90.20	2e-168	btm:MC28_1092	K02003	ABC.CD.A	putative ABC transport system ATP-binding protein	--	--

[denovogenes]_107777	42.52	2e-57	taz:TREAZ_0365	K03496	parA, soj	chromosome partitioning protein	--	--

[denovogenes]_107778	65.74	1e-118	cbut:ATN24_18135	K07053	E3.1.3.97	3',5'-nucleoside bisphosphate phosphatase	3.1.3.97	--

[denovogenes]_107781	53.60	2e-92	csh:Closa_0633	K17836	penP	beta-lactamase class A	3.5.2.6	Metabolism--Biosynthesis of other secondary metabolites--Penicillin and cephalosporin biosynthesis|Metabolism--Global and overview maps--Biosynthesis of antibiotics|Human Diseases--Drug resistance: Antimicrobial--beta-Lactam resistance

[denovogenes]_107782	100.00	0.0	ecoj:P423_17465	K19775	exuR	GntR family transcriptional regulator, hexuronate regulon transcriptional repressor	--	--

[denovogenes]_107783	40.86	4e-57	csb:CLSA_c16210	K10716	kch, trkA, mthK, pch	voltage-gated potassium channel	--	--

[denovogenes]_107785	81.01	4e-150	cbv:U729_2803	K03706	codY	transcriptional pleiotropic repressor	--	--

[denovogenes]_107786	46.67	4e-70	thl:TEH_08400	K02315	dnaC	DNA replication protein DnaC	--	--

[denovogenes]_107787	74.50	4e-143	drm:Dred_2234	K17074	lysX2	putative lysine transport system permease protein	--	Environmental Information Processing--Membrane transport--ABC transporters

[denovogenes]_107789	100.00	0.0	efn:DENG_02357	K03437	spoU	RNA methyltransferase, TrmH family	--	--

[denovogenes]_10779	99.85	0.0	esl:O3K_13535	K02618	paaZ	oxepin-CoA hydrolase / 3-oxo-5,6-dehydrosuberyl-CoA semialdehyde dehydrogenase	3.3.2.12 1.2.1.91	Metabolism--Amino acid metabolism--Phenylalanine metabolism|Metabolism--Global and overview maps--Microbial metabolism in diverse environments

[denovogenes]_107790	56.20	1e-90	vpr:Vpar_1847	K03497	parB, spo0J	chromosome partitioning protein, ParB family	--	--

[denovogenes]_107792	99.61	0.0	pvl:AOB99_01825	K11534	deoR	DeoR family transcriptional regulator, deoxyribose operon repressor	--	--

[denovogenes]_107793	99.60	2e-180	cbut:ATN24_00275	K03769	ppiC	peptidyl-prolyl cis-trans isomerase C	5.2.1.8	--

[denovogenes]_107794	73.75	1e-128	cbut:ATN24_01995	K06409	spoVB	stage V sporulation protein B	--	--

[denovogenes]_107795	87.55	5e-168	prg:RB151_006690	K09861	K09861	uncharacterized protein	--	--

[denovogenes]_107797	91.83	1e-175	cif:AL515_04700	K03424	--	--	--	--

[denovogenes]_107798	99.23	0.0	cif:AL515_13130	K13634	cysB	LysR family transcriptional regulator, cys regulon transcriptional activator	--	--

[denovogenes]_107800	77.22	2e-142	csq:CSCA_4131	K03495	gidA, mnmG, MTO1	tRNA uridine 5-carboxymethylaminomethyl modification enzyme	--	--

[denovogenes]_107801	97.29	0.0	pvl:AOB99_03970	K03472	epd	D-erythrose 4-phosphate dehydrogenase	1.2.1.72	Metabolism--Metabolism of cofactors and vitamins--Vitamin B6 metabolism|Metabolism--Global and overview maps--Metabolic pathways

[denovogenes]_107803	55.98	1e-102	erb:A4V01_18295	K01776	murI	glutamate racemase	5.1.1.3	Metabolism--Metabolism of other amino acids--D-Glutamine and D-glutamate metabolism|Metabolism--Global and overview maps--Metabolic pathways

[denovogenes]_107804	70.27	2e-132	cdg:CDBI1_04125	K15583	oppD	oligopeptide transport system ATP-binding protein	--	Human Diseases--Drug resistance: Antimicrobial--beta-Lactam resistance|Environmental Information Processing--Membrane transport--ABC transporters|Cellular Processes--Cellular community - prokaryotes--Quorum sensing

[denovogenes]_107805	99.22	0.0	ecv:APECO1_3785	K01816	hyi, gip	hydroxypyruvate isomerase	5.3.1.22	Metabolism--Carbohydrate metabolism--Glyoxylate and dicarboxylate metabolism|Metabolism--Global and overview maps--Metabolic pathways

[denovogenes]_107806	100.00	0.0	pvl:AOB99_12245	K01649	leuA	2-isopropylmalate synthase	2.3.3.13	Metabolism--Amino acid metabolism--Valine, leucine and isoleucine biosynthesis|Metabolism--Carbohydrate metabolism--Pyruvate metabolism|Metabolism--Global and overview maps--Metabolic pathways|Metabolism--Global and overview maps--Biosynthesis of secondary metabolites|Metabolism--Global and overview maps--2-Oxocarboxylic acid metabolism|Metabolism--Global and overview maps--Biosynthesis of amino acids

[denovogenes]_107807	46.97	8e-76	lam:LA2_07790	K02315	dnaC	DNA replication protein DnaC	--	--

[denovogenes]_107808	40.87	3e-56	pft:JBW_00787	K00354	E1.6.99.1	NADPH2 dehydrogenase	1.6.99.1	--

[denovogenes]_107809	53.53	1e-91	cdg:CDBI1_14125	K03478	chbG	chitin disaccharide deacetylase	3.5.1.105	--

[denovogenes]_107810	92.28	0.0	sgt:SGGB_2181	K15051	K15051, endA	DNA-entry nuclease	--	--

[denovogenes]_107811	100.00	0.0	shq:A0259_00745	K03118	tatC	sec-independent protein translocase protein TatC	--	Genetic Information Processing--Folding, sorting and degradation--Protein export|Environmental Information Processing--Membrane transport--Bacterial secretion system

[denovogenes]_107812	42.97	2e-68	aamy:GFC30_735	K07124	K07124	uncharacterized protein	--	--

[denovogenes]_107813	59.62	1e-90	cle:Clole_3526	K03076	secY	preprotein translocase subunit SecY	--	Cellular Processes--Cellular community - prokaryotes--Quorum sensing|Genetic Information Processing--Folding, sorting and degradation--Protein export|Environmental Information Processing--Membrane transport--Bacterial secretion system

[denovogenes]_107816	82.17	9e-160	cfd:CFNIH1_03515	K01674	cah	carbonic anhydrase	4.2.1.1	Metabolism--Energy metabolism--Nitrogen metabolism

[denovogenes]_107817	98.06	1e-180	elo:EC042_4334	K00930	argB	acetylglutamate kinase	2.7.2.8	Metabolism--Amino acid metabolism--Arginine biosynthesis|Metabolism--Global and overview maps--Metabolic pathways|Metabolism--Global and overview maps--Biosynthesis of secondary metabolites|Metabolism--Global and overview maps--Biosynthesis of antibiotics|Metabolism--Global and overview maps--2-Oxocarboxylic acid metabolism|Metabolism--Global and overview maps--Biosynthesis of amino acids

[denovogenes]_107818	55.29	7e-101	cbk:CLL_A2958	K07238	TC.ZIP, zupT, ZRT3, ZIP2	zinc transporter, ZIP family	--	--

[denovogenes]_107819	53.49	7e-89	bmyc:DJ92_5180	K16785	ecfT	energy-coupling factor transport system permease protein	--	Environmental Information Processing--Membrane transport--ABC transporters

[denovogenes]_10782	99.71	0.0	bxy:BXY_04700	K04079	HSP90A, htpG	molecular chaperone HtpG	--	Genetic Information Processing--Folding, sorting and degradation--Protein processing in endoplasmic reticulum|Environmental Information Processing--Signal transduction--PI3K-Akt signaling pathway|Organismal Systems--Immune system--Antigen processing and presentation|Organismal Systems--Immune system--NOD-like receptor signaling pathway|Organismal Systems--Environmental adaptation--Plant-pathogen interaction|Organismal Systems--Immune system--IL-17 signaling pathway|Organismal Systems--Immune system--Th17 cell differentiation|Organismal Systems--Endocrine system--Progesterone-mediated oocyte maturation|Organismal Systems--Endocrine system--Estrogen signaling pathway|Human Diseases--Cancers: Overview--Pathways in cancer|Human Diseases--Cancers: Specific types--Prostate cancer|Human Diseases--Cardiovascular diseases--Fluid shear stress and atherosclerosis

[denovogenes]_107820	96.53	0.0	cct:CC1_06180	K02315	dnaC	DNA replication protein DnaC	--	--

[denovogenes]_107823	42.06	1e-33	thx:Thet_0436	K03406	mcp	methyl-accepting chemotaxis protein	--	Environmental Information Processing--Signal transduction--Two-component system|Cellular Processes--Cell motility--Bacterial chemotaxis

[denovogenes]_107824	51.56	4e-86	cce:Ccel_2808	K00984	aadA	streptomycin 3"-adenylyltransferase	2.7.7.47	--

[denovogenes]_107826	100.00	0.0	ecoh:ECRM13516_0497	K01816	hyi, gip	hydroxypyruvate isomerase	5.3.1.22	Metabolism--Carbohydrate metabolism--Glyoxylate and dicarboxylate metabolism|Metabolism--Global and overview maps--Metabolic pathways

[denovogenes]_107828	69.48	5e-121	cle:Clole_4268	K03650	mnmE, trmE, MSS1	tRNA modification GTPase	3.6.-.-	--

[denovogenes]_107829	86.49	3e-167	cbt:CLH_1018	K00600	glyA, SHMT	glycine hydroxymethyltransferase	2.1.2.1	Metabolism--Amino acid metabolism--Glycine, serine and threonine metabolism|Metabolism--Metabolism of other amino acids--Cyanoamino acid metabolism|Metabolism--Carbohydrate metabolism--Glyoxylate and dicarboxylate metabolism|Metabolism--Metabolism of cofactors and vitamins--One carbon pool by folate|Metabolism--Energy metabolism--Methane metabolism|Metabolism--Global and overview maps--Metabolic pathways|Metabolism--Global and overview maps--Biosynthesis of secondary metabolites|Metabolism--Global and overview maps--Microbial metabolism in diverse environments|Metabolism--Global and overview maps--Biosynthesis of antibiotics|Metabolism--Global and overview maps--Carbon metabolism|Metabolism--Global and overview maps--Biosynthesis of amino acids|Human Diseases--Drug resistance: Antineoplastic--Antifolate resistance

[denovogenes]_107830	77.61	1e-143	cld:CLSPO_c01840	K02111	ATPF1A, atpA	F-type H+-transporting ATPase subunit alpha	3.6.3.14	Metabolism--Energy metabolism--Oxidative phosphorylation|Metabolism--Energy metabolism--Photosynthesis|Metabolism--Global and overview maps--Metabolic pathways

[denovogenes]_107831	99.23	0.0	cif:AL515_13130	K13634	cysB	LysR family transcriptional regulator, cys regulon transcriptional activator	--	--

[denovogenes]_107833	98.46	0.0	cfd:CFNIH1_13870	K03517	nadA	quinolinate synthase	2.5.1.72	Metabolism--Metabolism of cofactors and vitamins--Nicotinate and nicotinamide metabolism|Metabolism--Global and overview maps--Metabolic pathways

[denovogenes]_107834	100.00	0.0	elp:P12B_c4115	K07709	zraS, hydH	two-component system, NtrC family, sensor histidine kinase HydH	2.7.13.3	Environmental Information Processing--Signal transduction--Two-component system

[denovogenes]_107835	60.00	2e-99	pdf:CD630DERM_25930	K18231	msrA, vmlR	macrolide transport system ATP-binding/permease protein	--	Environmental Information Processing--Membrane transport--ABC transporters

[denovogenes]_107837	85.71	9e-160	pdf:CD630DERM_35150	K04042	glmU	bifunctional UDP-N-acetylglucosamine pyrophosphorylase / Glucosamine-1-phosphate N-acetyltransferase	2.7.7.23 2.3.1.157	Metabolism--Carbohydrate metabolism--Amino sugar and nucleotide sugar metabolism|Metabolism--Global and overview maps--Metabolic pathways|Metabolism--Global and overview maps--Biosynthesis of antibiotics

[denovogenes]_107838	100.00	0.0	cfd:CFNIH1_19325	K06131	clsA_B	cardiolipin synthase A/B	2.7.8.-	Metabolism--Lipid metabolism--Glycerophospholipid metabolism|Metabolism--Global and overview maps--Metabolic pathways

[denovogenes]_107839	98.06	0.0	cif:AL515_18995	K07462	recJ	single-stranded-DNA-specific exonuclease	3.1.-.-	Genetic Information Processing--Replication and repair--Base excision repair|Genetic Information Processing--Replication and repair--Mismatch repair|Genetic Information Processing--Replication and repair--Homologous recombination

[denovogenes]_10784	93.39	0.0	btho:Btheta7330_02532	K04079	HSP90A, htpG	molecular chaperone HtpG	--	Genetic Information Processing--Folding, sorting and degradation--Protein processing in endoplasmic reticulum|Environmental Information Processing--Signal transduction--PI3K-Akt signaling pathway|Organismal Systems--Immune system--Antigen processing and presentation|Organismal Systems--Immune system--NOD-like receptor signaling pathway|Organismal Systems--Environmental adaptation--Plant-pathogen interaction|Organismal Systems--Immune system--IL-17 signaling pathway|Organismal Systems--Immune system--Th17 cell differentiation|Organismal Systems--Endocrine system--Progesterone-mediated oocyte maturation|Organismal Systems--Endocrine system--Estrogen signaling pathway|Human Diseases--Cancers: Overview--Pathways in cancer|Human Diseases--Cancers: Specific types--Prostate cancer|Human Diseases--Cardiovascular diseases--Fluid shear stress and atherosclerosis

[denovogenes]_107840	66.28	7e-115	sgp:SpiGrapes_2627	K03299	TC.GNTP	gluconate:H+ symporter, GntP family	--	--

[denovogenes]_107841	81.01	7e-150	psx:DR96_3558	K07308	dmsC	anaerobic dimethyl sulfoxide reductase subunit C (DMSO reductase anchor subunit)	--	Metabolism--Energy metabolism--Sulfur metabolism

[denovogenes]_107842	97.28	0.0	prg:RB151_042300	K00826	E2.6.1.42, ilvE	branched-chain amino acid aminotransferase	2.6.1.42	Metabolism--Amino acid metabolism--Cysteine and methionine metabolism|Metabolism--Amino acid metabolism--Valine, leucine and isoleucine degradation|Metabolism--Amino acid metabolism--Valine, leucine and isoleucine biosynthesis|Metabolism--Metabolism of cofactors and vitamins--Pantothenate and CoA biosynthesis|Metabolism--Global and overview maps--Metabolic pathways|Metabolism--Global and overview maps--Biosynthesis of secondary metabolites|Metabolism--Global and overview maps--Biosynthesis of antibiotics|Metabolism--Global and overview maps--2-Oxocarboxylic acid metabolism|Metabolism--Global and overview maps--Biosynthesis of amino acids

[denovogenes]_107844	86.05	3e-161	cbut:ATN24_05910	K06350	kipA	antagonist of KipI	--	--

[denovogenes]_107845	100.00	6e-177	cbut:ATN24_02605	K03324	yjbB	phosphate:Na+ symporter	--	--

[denovogenes]_107846	67.59	3e-122	cle:Clole_2227	K09758	asdA	aspartate 4-decarboxylase	4.1.1.12	Metabolism--Amino acid metabolism--Alanine, aspartate and glutamate metabolism|Metabolism--Amino acid metabolism--Cysteine and methionine metabolism|Metabolism--Global and overview maps--Metabolic pathways|Metabolism--Global and overview maps--Biosynthesis of amino acids

[denovogenes]_107847	98.45	0.0	cfd:CFNIH1_16710	K01626	E2.5.1.54, aroF, aroG, aroH	3-deoxy-7-phosphoheptulonate synthase	2.5.1.54	Metabolism--Amino acid metabolism--Phenylalanine, tyrosine and tryptophan biosynthesis|Metabolism--Global and overview maps--Metabolic pathways|Metabolism--Global and overview maps--Biosynthesis of secondary metabolites|Metabolism--Global and overview maps--Biosynthesis of antibiotics|Metabolism--Global and overview maps--Biosynthesis of amino acids|Cellular Processes--Cellular community - prokaryotes--Quorum sensing

[denovogenes]_107848	90.70	5e-173	sind:105174728	K08915	--	--	--	Metabolism--Energy metabolism--Photosynthesis - antenna proteins|Metabolism--Global and overview maps--Metabolic pathways

[denovogenes]_107849	67.45	1e-125	cbv:U729_226	K03734	apbE	FAD:protein FMN transferase	2.7.1.180	--

[denovogenes]_107850	76.83	8e-145	pdf:CD630DERM_34060	K00335	nuoF	NADH-quinone oxidoreductase subunit F	1.6.5.3	Metabolism--Energy metabolism--Oxidative phosphorylation|Metabolism--Global and overview maps--Metabolic pathways

[denovogenes]_107852	77.99	6e-159	cad:Curi_c04240	K01466	allB	allantoinase	3.5.2.5	Metabolism--Nucleotide metabolism--Purine metabolism|Metabolism--Global and overview maps--Metabolic pathways|Metabolism--Global and overview maps--Microbial metabolism in diverse environments

[denovogenes]_107855	73.75	1e-129	sfs:SFyv_0844	K01547	kdpB	K+-transporting ATPase ATPase B chain	3.6.3.12	Environmental Information Processing--Signal transduction--Two-component system

[denovogenes]_107856	100.00	0.0	ecw:EcE24377A_3853	K01867	WARS, trpS	tryptophanyl-tRNA synthetase	6.1.1.2	Genetic Information Processing--Translation--Aminoacyl-tRNA biosynthesis

[denovogenes]_107857	46.15	4e-77	tto:Thethe_00429	K03484	scrR	LacI family transcriptional regulator, sucrose operon repressor	--	--

[denovogenes]_107858	40.23	3e-57	tra:Trad_0273	K03654	recQ	ATP-dependent DNA helicase RecQ	3.6.4.12	Genetic Information Processing--Folding, sorting and degradation--RNA degradation

[denovogenes]_107859	71.81	8e-137	cbz:Cbs_0130	K01881	PARS, proS	prolyl-tRNA synthetase	6.1.1.15	Genetic Information Processing--Translation--Aminoacyl-tRNA biosynthesis

[denovogenes]_10786	100.00	0.0	ecoh:ECRM13516_5125	K09800	tamB	translocation and assembly module TamB	--	--

[denovogenes]_107861	95.75	0.0	ssj:SSON53_15835	K12063	traC	conjugal transfer ATP-binding protein TraC	--	--

[denovogenes]_107866	99.61	0.0	cif:AL515_07100	K04079	HSP90A, htpG	molecular chaperone HtpG	--	Genetic Information Processing--Folding, sorting and degradation--Protein processing in endoplasmic reticulum|Environmental Information Processing--Signal transduction--PI3K-Akt signaling pathway|Organismal Systems--Immune system--Antigen processing and presentation|Organismal Systems--Immune system--NOD-like receptor signaling pathway|Organismal Systems--Environmental adaptation--Plant-pathogen interaction|Organismal Systems--Immune system--IL-17 signaling pathway|Organismal Systems--Immune system--Th17 cell differentiation|Organismal Systems--Endocrine system--Progesterone-mediated oocyte maturation|Organismal Systems--Endocrine system--Estrogen signaling pathway|Human Diseases--Cancers: Overview--Pathways in cancer|Human Diseases--Cancers: Specific types--Prostate cancer|Human Diseases--Cardiovascular diseases--Fluid shear stress and atherosclerosis

[denovogenes]_107867	66.93	3e-112	cbt:CLH_1484	K01738	cysK	cysteine synthase A	2.5.1.47	Metabolism--Amino acid metabolism--Cysteine and methionine metabolism|Metabolism--Energy metabolism--Sulfur metabolism|Metabolism--Global and overview maps--Metabolic pathways|Metabolism--Global and overview maps--Biosynthesis of secondary metabolites|Metabolism--Global and overview maps--Microbial metabolism in diverse environments|Metabolism--Global and overview maps--Biosynthesis of antibiotics|Metabolism--Global and overview maps--Carbon metabolism|Metabolism--Global and overview maps--Biosynthesis of amino acids

[denovogenes]_107868	47.44	7e-46	lsa:LCA_1414	K03281	TC.CIC	chloride channel protein, CIC family	--	--

[denovogenes]_107871	63.74	1e-109	vhr:AL538_22005	K02768	PTS-Fru-EIIA, fruB	PTS system, fructose-specific IIA component	2.7.1.202	Metabolism--Carbohydrate metabolism--Fructose and mannose metabolism|Metabolism--Global and overview maps--Metabolic pathways|Metabolism--Global and overview maps--Microbial metabolism in diverse environments|Environmental Information Processing--Membrane transport--Phosphotransferase system (PTS)

[denovogenes]_107871	63.74	1e-109	vhr:AL538_22005	K02769	PTS-Fru-EIIB, fruA	PTS system, fructose-specific IIB component	2.7.1.202	Metabolism--Carbohydrate metabolism--Fructose and mannose metabolism|Metabolism--Global and overview maps--Metabolic pathways|Metabolism--Global and overview maps--Microbial metabolism in diverse environments|Environmental Information Processing--Membrane transport--Phosphotransferase system (PTS)

[denovogenes]_107871	63.74	1e-109	vhr:AL538_22005	K02770	PTS-Fru-EIIC, fruA	PTS system, fructose-specific IIC component	--	Metabolism--Carbohydrate metabolism--Fructose and mannose metabolism|Metabolism--Global and overview maps--Metabolic pathways|Metabolism--Global and overview maps--Microbial metabolism in diverse environments|Environmental Information Processing--Membrane transport--Phosphotransferase system (PTS)

[denovogenes]_107872	41.30	8e-61	bcz:BCE33L0424	K01468	hutI, AMDHD1	imidazolonepropionase	3.5.2.7	Metabolism--Amino acid metabolism--Histidine metabolism|Metabolism--Global and overview maps--Metabolic pathways

[denovogenes]_107876	44.71	5e-68	bsm:BSM4216_1662	K15866	paaG	2-(1,2-epoxy-1,2-dihydrophenyl)acetyl-CoA isomerase	5.3.3.18	Metabolism--Amino acid metabolism--Phenylalanine metabolism|Metabolism--Global and overview maps--Microbial metabolism in diverse environments

[denovogenes]_107877	84.65	6e-145	bcel:BcellWH2_02188	K03630	radC	DNA repair protein RadC	--	--

[denovogenes]_107878	62.86	1e-109	cpf:CPF_1154	K00076	hdhA	7-alpha-hydroxysteroid dehydrogenase	1.1.1.159	--

[denovogenes]_107879	89.58	3e-172	bxy:BXY_23870	K02477	K02477	two-component system, LytT family, response regulator	--	--

[denovogenes]_107880	96.12	1e-179	bhl:Bache_1384	K00677	lpxA	UDP-N-acetylglucosamine acyltransferase	2.3.1.129	Metabolism--Glycan biosynthesis and metabolism--Lipopolysaccharide biosynthesis|Metabolism--Global and overview maps--Metabolic pathways|Human Diseases--Drug resistance: Antimicrobial--Cationic antimicrobial peptide (CAMP) resistance

[denovogenes]_107881	85.60	5e-169	rum:CK1_00260	K00757	udp, UPP	uridine phosphorylase	2.4.2.3	Metabolism--Nucleotide metabolism--Pyrimidine metabolism|Metabolism--Xenobiotics biodegradation and metabolism--Drug metabolism - other enzymes|Metabolism--Global and overview maps--Metabolic pathways

[denovogenes]_107884	49.80	2e-76	pft:JBW_03847	K06173	truA, PUS1	tRNA pseudouridine38-40 synthase	5.4.99.12	--

[denovogenes]_107885	63.18	5e-121	pth:PTH_0258	K03525	coaX	type III pantothenate kinase	2.7.1.33	Metabolism--Metabolism of cofactors and vitamins--Pantothenate and CoA biosynthesis|Metabolism--Global and overview maps--Metabolic pathways

[denovogenes]_107886	100.00	0.0	bvu:BVU_3313	K01241	amn	AMP nucleosidase	3.2.2.4	Metabolism--Nucleotide metabolism--Purine metabolism

[denovogenes]_10789	99.85	0.0	prp:M062_25415	K00219	fadH	2,4-dienoyl-CoA reductase (NADPH2)	1.3.1.34	--

[denovogenes]_107891	60.85	2e-105	boa:Bovatus_04431	K00674	dapD	2,3,4,5-tetrahydropyridine-2-carboxylate N-succinyltransferase	2.3.1.117	Metabolism--Amino acid metabolism--Lysine biosynthesis|Metabolism--Global and overview maps--Metabolic pathways|Metabolism--Global and overview maps--Microbial metabolism in diverse environments|Metabolism--Global and overview maps--Biosynthesis of amino acids

[denovogenes]_107892	63.28	7e-119	rob:CK5_16930	K00891	E2.7.1.71, aroK, aroL	shikimate kinase	2.7.1.71	Metabolism--Amino acid metabolism--Phenylalanine, tyrosine and tryptophan biosynthesis|Metabolism--Global and overview maps--Metabolic pathways|Metabolism--Global and overview maps--Biosynthesis of secondary metabolites|Metabolism--Global and overview maps--Biosynthesis of antibiotics|Metabolism--Global and overview maps--Biosynthesis of amino acids

[denovogenes]_107894	44.72	3e-32	pca:Pcar_1321	K08309	slt	soluble lytic murein transglycosylase	3.2.1.-	--

[denovogenes]_107898	43.75	9e-64	ppq:PPSQR21_032790	K02315	dnaC	DNA replication protein DnaC	--	--

[denovogenes]_107899	98.84	0.0	bhl:Bache_2303	K01241	amn	AMP nucleosidase	3.2.2.4	Metabolism--Nucleotide metabolism--Purine metabolism

[denovogenes]_1079	70.17	0.0	fpla:A4U99_11240	K16898	addA	ATP-dependent helicase/nuclease subunit A	3.1.-.- 3.6.4.12	--

[denovogenes]_10790	83.48	0.0	lir:LAW_00976	K02355	fusA, GFM, EFG	elongation factor G	--	--

[denovogenes]_107900	97.29	0.0	bhl:Bache_2300	K02823	pyrDII	dihydroorotate dehydrogenase electron transfer subunit	--	Metabolism--Nucleotide metabolism--Pyrimidine metabolism|Metabolism--Global and overview maps--Metabolic pathways

[denovogenes]_107901	84.88	7e-166	btho:Btheta7330_00988	K03424	--	--	--	--

[denovogenes]_107903	85.27	6e-160	fpla:A4U99_15055	K01715	crt	enoyl-CoA hydratase	4.2.1.17	Metabolism--Carbohydrate metabolism--Butanoate metabolism|Metabolism--Global and overview maps--Carbon metabolism

[denovogenes]_107909	100.00	0.0	bvu:BVU_0533	K09474	phoN	acid phosphatase (class A)	3.1.3.2	Metabolism--Xenobiotics biodegradation and metabolism--Aminobenzoate degradation|Metabolism--Metabolism of cofactors and vitamins--Riboflavin metabolism|Metabolism--Global and overview maps--Metabolic pathways|Metabolism--Global and overview maps--Microbial metabolism in diverse environments|Environmental Information Processing--Signal transduction--Two-component system

[denovogenes]_10791	96.92	0.0	bfb:VU15_10640	K04079	HSP90A, htpG	molecular chaperone HtpG	--	Genetic Information Processing--Folding, sorting and degradation--Protein processing in endoplasmic reticulum|Environmental Information Processing--Signal transduction--PI3K-Akt signaling pathway|Organismal Systems--Immune system--Antigen processing and presentation|Organismal Systems--Immune system--NOD-like receptor signaling pathway|Organismal Systems--Environmental adaptation--Plant-pathogen interaction|Organismal Systems--Immune system--IL-17 signaling pathway|Organismal Systems--Immune system--Th17 cell differentiation|Organismal Systems--Endocrine system--Progesterone-mediated oocyte maturation|Organismal Systems--Endocrine system--Estrogen signaling pathway|Human Diseases--Cancers: Overview--Pathways in cancer|Human Diseases--Cancers: Specific types--Prostate cancer|Human Diseases--Cardiovascular diseases--Fluid shear stress and atherosclerosis

[denovogenes]_107916	96.50	0.0	boa:Bovatus_00986	K03149	thiG	thiazole synthase	2.8.1.10	Metabolism--Metabolism of cofactors and vitamins--Thiamine metabolism|Metabolism--Global and overview maps--Metabolic pathways

[denovogenes]_107917	68.09	7e-130	cpe:CPE1537	K16951	--	--	--	Metabolism--Energy metabolism--Sulfur metabolism|Metabolism--Global and overview maps--Microbial metabolism in diverse environments

[denovogenes]_107918	85.49	2e-161	lacy:A4V08_19095	K02028	ABC.PA.A	polar amino acid transport system ATP-binding protein	3.6.3.21	--

[denovogenes]_107920	79.76	4e-142	cso:CLS_11480	K02010	afuC, fbpC	iron(III) transport system ATP-binding protein	3.6.3.30	Environmental Information Processing--Membrane transport--ABC transporters

[denovogenes]_107923	54.90	2e-100	erb:A4V01_05710	K05306	phnX	phosphonoacetaldehyde hydrolase	3.11.1.1	Metabolism--Metabolism of other amino acids--Phosphonate and phosphinate metabolism|Metabolism--Global and overview maps--Metabolic pathways|Metabolism--Global and overview maps--Microbial metabolism in diverse environments

[denovogenes]_107925	100.00	0.0	bdh:GV66_15455	K02342	DPO3E, dnaQ	DNA polymerase III subunit epsilon	2.7.7.7	Metabolism--Nucleotide metabolism--Purine metabolism|Metabolism--Nucleotide metabolism--Pyrimidine metabolism|Metabolism--Global and overview maps--Metabolic pathways|Genetic Information Processing--Replication and repair--DNA replication|Genetic Information Processing--Replication and repair--Mismatch repair|Genetic Information Processing--Replication and repair--Homologous recombination

[denovogenes]_107929	97.82	1e-165	fpla:A4U99_08805	K02967	RP-S2, MRPS2, rpsB	small subunit ribosomal protein S2	--	Genetic Information Processing--Translation--Ribosome

[denovogenes]_10793	99.85	0.0	paeg:AI22_29635	K16088	TC.FEV.OM1, fhuE, fpvA, fptA	outer-membrane receptor for ferric coprogen and ferric-rhodotorulic acid	--	--

[denovogenes]_107930	52.14	9e-99	paen:P40081_19225	K01438	argE	acetylornithine deacetylase	3.5.1.16	Metabolism--Amino acid metabolism--Arginine biosynthesis|Metabolism--Global and overview maps--Metabolic pathways|Metabolism--Global and overview maps--Biosynthesis of secondary metabolites|Metabolism--Global and overview maps--Biosynthesis of antibiotics|Metabolism--Global and overview maps--2-Oxocarboxylic acid metabolism|Metabolism--Global and overview maps--Biosynthesis of amino acids

[denovogenes]_107935	73.23	7e-127	csh:Closa_0982	K16785	ecfT	energy-coupling factor transport system permease protein	--	Environmental Information Processing--Membrane transport--ABC transporters

[denovogenes]_107936	76.61	1e-139	cle:Clole_3243	K02041	phnC	phosphonate transport system ATP-binding protein	3.6.3.28	Environmental Information Processing--Membrane transport--ABC transporters

[denovogenes]_10794	78.53	0.0	pdi:BDI_0915	K01533	copB	Cu2+-exporting ATPase	3.6.3.4	--

[denovogenes]_107940	86.82	2e-152	cpy:Cphy_1744	K00046	idnO	gluconate 5-dehydrogenase	1.1.1.69	--

[denovogenes]_107948	100.00	0.0	btho:Btheta7330_02523	K02823	pyrDII	dihydroorotate dehydrogenase electron transfer subunit	--	Metabolism--Nucleotide metabolism--Pyrimidine metabolism|Metabolism--Global and overview maps--Metabolic pathways

[denovogenes]_107950	98.06	0.0	boa:Bovatus_00755	K02823	pyrDII	dihydroorotate dehydrogenase electron transfer subunit	--	Metabolism--Nucleotide metabolism--Pyrimidine metabolism|Metabolism--Global and overview maps--Metabolic pathways

[denovogenes]_107952	95.74	0.0	boa:Bovatus_00755	K02823	pyrDII	dihydroorotate dehydrogenase electron transfer subunit	--	Metabolism--Nucleotide metabolism--Pyrimidine metabolism|Metabolism--Global and overview maps--Metabolic pathways

[denovogenes]_107954	54.88	3e-88	lacy:A4V08_31215	K03496	parA, soj	chromosome partitioning protein	--	--

[denovogenes]_107956	50.58	2e-93	pmw:B2K_12335	K02025	ABC.MS.P	multiple sugar transport system permease protein	--	--

[denovogenes]_10796	99.71	0.0	pdk:PADK2_07315	K19339	nosR	NosR/NirI family transcriptional regulator, nitrous oxide reductase regulator	--	--

[denovogenes]_107960	100.00	0.0	bvu:BVU_3855	K09812	ftsE	cell division transport system ATP-binding protein	--	Environmental Information Processing--Membrane transport--ABC transporters

[denovogenes]_107962	84.21	9e-136	lacy:A4V08_19025	K02945	RP-S1, rpsA	small subunit ribosomal protein S1	--	Genetic Information Processing--Translation--Ribosome

[denovogenes]_107963	41.92	1e-44	pao:Pat9b_4943	K02013	ABC.FEV.A	iron complex transport system ATP-binding protein	3.6.3.34	Environmental Information Processing--Membrane transport--ABC transporters

[denovogenes]_107964	52.16	5e-97	gvh:HMPREF9231_0297	K10119	msmG	raffinose/stachyose/melibiose transport system permease protein	--	Environmental Information Processing--Membrane transport--ABC transporters

[denovogenes]_107965	56.18	5e-98	cap:CLDAP_29810	K01995	livG	branched-chain amino acid transport system ATP-binding protein	--	Environmental Information Processing--Membrane transport--ABC transporters|Cellular Processes--Cellular community - prokaryotes--Quorum sensing

[denovogenes]_107966	41.07	1e-12	aai:AARI_01330	K03183	ubiE	demethylmenaquinone methyltransferase / 2-methoxy-6-polyprenyl-1,4-benzoquinol methylase	2.1.1.163 2.1.1.201	Metabolism--Metabolism of cofactors and vitamins--Ubiquinone and other terpenoid-quinone biosynthesis|Metabolism--Global and overview maps--Metabolic pathways|Metabolism--Global and overview maps--Biosynthesis of secondary metabolites

[denovogenes]_107968	62.35	5e-110	swo:Swol_0408	K02588	nifH	nitrogenase iron protein NifH	1.18.6.1	Metabolism--Xenobiotics biodegradation and metabolism--Chloroalkane and chloroalkene degradation|Metabolism--Energy metabolism--Nitrogen metabolism|Metabolism--Global and overview maps--Metabolic pathways|Metabolism--Global and overview maps--Microbial metabolism in diverse environments

[denovogenes]_107969	94.19	0.0	boa:Bovatus_02094	K03424	--	--	--	--

[denovogenes]_10797	99.56	0.0	cif:AL515_18500	K00951	relA	GTP pyrophosphokinase	2.7.6.5	Metabolism--Nucleotide metabolism--Purine metabolism

[denovogenes]_107970	53.85	1e-92	hsd:SD1D_0859	K02421	fliR	flagellar biosynthetic protein FliR	--	Cellular Processes--Cell motility--Flagellar assembly

[denovogenes]_107971	99.22	0.0	bfb:VU15_16425	K00978	rfbF	glucose-1-phosphate cytidylyltransferase	2.7.7.33	Metabolism--Carbohydrate metabolism--Starch and sucrose metabolism|Metabolism--Carbohydrate metabolism--Amino sugar and nucleotide sugar metabolism|Metabolism--Global and overview maps--Metabolic pathways

[denovogenes]_107973	51.78	1e-97	ccel:CCDG5_0500	K09769	K09769	uncharacterized protein	--	--

[denovogenes]_107974	40.00	3e-27	mhor:MSHOH_3871	K21029	moeB	molybdopterin-synthase adenylyltransferase	2.7.7.80	Genetic Information Processing--Folding, sorting and degradation--Sulfur relay system

[denovogenes]_107975	73.47	6e-131	tur:AT726_03940	K02028	ABC.PA.A	polar amino acid transport system ATP-binding protein	3.6.3.21	--

[denovogenes]_107976	94.57	0.0	bvu:BVU_3313	K01241	amn	AMP nucleosidase	3.2.2.4	Metabolism--Nucleotide metabolism--Purine metabolism

[denovogenes]_107977	99.61	0.0	esl:O3K_21510	K09861	K09861	uncharacterized protein	--	--

[denovogenes]_10798	98.68	0.0	lacy:A4V08_12395	K03043	rpoB	DNA-directed RNA polymerase subunit beta	2.7.7.6	Metabolism--Nucleotide metabolism--Purine metabolism|Metabolism--Nucleotide metabolism--Pyrimidine metabolism|Metabolism--Global and overview maps--Metabolic pathways|Genetic Information Processing--Transcription--RNA polymerase

[denovogenes]_107981	50.99	9e-92	cbt:CLH_3304	K02556	motA	chemotaxis protein MotA	--	Environmental Information Processing--Signal transduction--Two-component system|Cellular Processes--Cell motility--Bacterial chemotaxis|Cellular Processes--Cell motility--Flagellar assembly

[denovogenes]_107984	96.89	8e-178	lacy:A4V08_27365	K10440	rbsC	ribose transport system permease protein	--	Environmental Information Processing--Membrane transport--ABC transporters

[denovogenes]_107985	100.00	0.0	fpla:A4U99_15055	K01715	crt	enoyl-CoA hydratase	4.2.1.17	Metabolism--Carbohydrate metabolism--Butanoate metabolism|Metabolism--Global and overview maps--Carbon metabolism

[denovogenes]_107987	50.58	9e-80	gfe:Gferi_22335	K00065	kduD	2-deoxy-D-gluconate 3-dehydrogenase	1.1.1.125	Metabolism--Carbohydrate metabolism--Pentose and glucuronate interconversions

[denovogenes]_107988	99.22	0.0	btho:Btheta7330_00988	K03424	--	--	--	--

[denovogenes]_107989	94.19	7e-160	lacy:A4V08_28700	K02746	PTS-Aga-EIIC, agaW	PTS system, N-acetylgalactosamine-specific IIC component	--	Metabolism--Carbohydrate metabolism--Galactose metabolism|Environmental Information Processing--Membrane transport--Phosphotransferase system (PTS)

[denovogenes]_10799	85.19	0.0	bvu:BVU_0237	K03296	TC.HAE1	hydrophobic/amphiphilic exporter-1 (mainly G- bacteria), HAE1 family	--	--

[denovogenes]_107990	77.22	2e-149	fpla:A4U99_15250	K09769	K09769	uncharacterized protein	--	--

[denovogenes]_107991	79.84	3e-147	rum:CK1_28850	K03149	thiG	thiazole synthase	2.8.1.10	Metabolism--Metabolism of cofactors and vitamins--Thiamine metabolism|Metabolism--Global and overview maps--Metabolic pathways

[denovogenes]_107992	100.00	0.0	cif:AL515_05020	K00313	fixC	electron transfer flavoprotein-quinone oxidoreductase	1.5.5.-	--

[denovogenes]_107993	50.19	1e-87	bmq:BMQ_2270	K01990	ABC-2.A	ABC-2 type transport system ATP-binding protein	--	--

[denovogenes]_107994	53.57	3e-82	ccl:Clocl_1087	K03768	PPIB, ppiB	peptidyl-prolyl cis-trans isomerase B (cyclophilin B)	5.2.1.8	--

[denovogenes]_107995	42.35	7e-45	cml:BN424_1942	K03546	sbcC	exonuclease SbcC	--	--

[denovogenes]_107997	99.61	0.0	sbo:SBO_2237	K11930	torT	periplasmic protein TorT	--	--

[denovogenes]_107998	62.20	1e-104	ibu:IB211_02201c	K03437	spoU	RNA methyltransferase, TrmH family	--	--

[denovogenes]_108	61.28	0.0	ent:Ent638_0053	K15125	fhaB	filamentous hemagglutinin	--	Human Diseases--Infectious diseases: Bacterial--Pertussis

[denovogenes]_108001	41.06	2e-67	sgp:SpiGrapes_3204	K01695	trpA	tryptophan synthase alpha chain	4.2.1.20	Metabolism--Amino acid metabolism--Glycine, serine and threonine metabolism|Metabolism--Amino acid metabolism--Phenylalanine, tyrosine and tryptophan biosynthesis|Metabolism--Global and overview maps--Metabolic pathways|Metabolism--Global and overview maps--Biosynthesis of secondary metabolites|Metabolism--Global and overview maps--Biosynthesis of antibiotics|Metabolism--Global and overview maps--Biosynthesis of amino acids

[denovogenes]_108003	87.55	4e-152	lacy:A4V08_18800	K01892	HARS, hisS	histidyl-tRNA synthetase	6.1.1.21	Genetic Information Processing--Translation--Aminoacyl-tRNA biosynthesis

[denovogenes]_108004	65.22	2e-107	fpla:A4U99_16715	K02499	yabN	tetrapyrrole methylase family protein / MazG family protein	--	--

[denovogenes]_108006	41.20	1e-42	bprm:CL3_14780	K07720	yesN	two-component system, response regulator YesN	--	Environmental Information Processing--Signal transduction--Two-component system

[denovogenes]_108007	60.39	1e-103	tjr:TherJR_0047	K00132	E1.2.1.10	acetaldehyde dehydrogenase (acetylating)	1.2.1.10	Metabolism--Carbohydrate metabolism--Pyruvate metabolism|Metabolism--Carbohydrate metabolism--Butanoate metabolism|Metabolism--Global and overview maps--Metabolic pathways|Metabolism--Global and overview maps--Microbial metabolism in diverse environments

[denovogenes]_108009	98.47	0.0	elh:ETEC_2084	K05374	irp4, ybtT	yersiniabactin synthetase, thioesterase component	--	--

[denovogenes]_108013	99.22	0.0	cfd:CFNIH1_14320	K07258	dacC, dacA, dacD	D-alanyl-D-alanine carboxypeptidase (penicillin-binding protein 5/6)	3.4.16.4	Metabolism--Glycan biosynthesis and metabolism--Peptidoglycan biosynthesis|Metabolism--Global and overview maps--Metabolic pathways

[denovogenes]_108014	40.34	3e-60	eel:EUBELI_20321	K01104	E3.1.3.48	protein-tyrosine phosphatase	3.1.3.48	--

[denovogenes]_108015	44.35	2e-68	bip:Bint_1414	K15554	ssuC	sulfonate transport system permease protein	--	Metabolism--Energy metabolism--Sulfur metabolism|Environmental Information Processing--Membrane transport--ABC transporters

[denovogenes]_108016	56.37	2e-107	clj:CLJU_c07460	K01008	selD, SEPHS	selenide, water dikinase	2.7.9.3	Metabolism--Metabolism of other amino acids--Selenocompound metabolism|Metabolism--Global and overview maps--Metabolic pathways

[denovogenes]_108017	87.55	5e-169	rum:CK1_24050	K03147	--	--	--	Metabolism--Metabolism of cofactors and vitamins--Thiamine metabolism|Metabolism--Global and overview maps--Metabolic pathways

[denovogenes]_108019	95.35	8e-177	bvu:BVU_3166	K00652	bioF	8-amino-7-oxononanoate synthase	2.3.1.47	Metabolism--Metabolism of cofactors and vitamins--Biotin metabolism|Metabolism--Global and overview maps--Metabolic pathways

[denovogenes]_10802	100.00	0.0	pvl:AOB99_11050	K01782	fadJ	3-hydroxyacyl-CoA dehydrogenase / enoyl-CoA hydratase / 3-hydroxybutyryl-CoA epimerase	1.1.1.35 4.2.1.17 5.1.2.3	Metabolism--Lipid metabolism--Fatty acid degradation|Metabolism--Amino acid metabolism--Valine, leucine and isoleucine degradation|Metabolism--Metabolism of terpenoids and polyketides--Geraniol degradation|Metabolism--Amino acid metabolism--Lysine degradation|Metabolism--Xenobiotics biodegradation and metabolism--Benzoate degradation|Metabolism--Amino acid metabolism--Tryptophan metabolism|Metabolism--Metabolism of other amino acids--beta-Alanine metabolism|Metabolism--Carbohydrate metabolism--Propanoate metabolism|Metabolism--Carbohydrate metabolism--Butanoate metabolism|Metabolism--Metabolism of terpenoids and polyketides--Limonene and pinene degradation|Metabolism--Xenobiotics biodegradation and metabolism--Caprolactam degradation|Metabolism--Lipid metabolism--Biosynthesis of unsaturated fatty acids|Metabolism--Global and overview maps--Metabolic pathways|Metabolism--Global and overview maps--Biosynthesis of secondary metabolites|Metabolism--Global and overview maps--Microbial metabolism in diverse environments|Metabolism--Global and overview maps--Biosynthesis of antibiotics|Metabolism--Global and overview maps--Carbon metabolism|Metabolism--Global and overview maps--Fatty acid metabolism

[denovogenes]_108020	79.13	9e-155	rob:CK5_24710	K01586	lysA	diaminopimelate decarboxylase	4.1.1.20	Metabolism--Amino acid metabolism--Lysine biosynthesis|Metabolism--Global and overview maps--Metabolic pathways|Metabolism--Global and overview maps--Biosynthesis of secondary metabolites|Metabolism--Global and overview maps--Microbial metabolism in diverse environments|Metabolism--Global and overview maps--Biosynthesis of antibiotics|Metabolism--Global and overview maps--Biosynthesis of amino acids

[denovogenes]_108021	100.00	0.0	sdz:Asd1617_03466	K07088	K07088	uncharacterized protein	--	--

[denovogenes]_108022	51.72	4e-84	erb:A4V01_09955	K06402	spoIVFB	stage IV sporulation protein FB	3.4.24.-	--

[denovogenes]_108025	92.54	1e-37	ecl:EcolC_0727	K10939	acfD	accessory colonization factor AcfD	--	Cellular Processes--Cellular community - prokaryotes--Biofilm formation - Vibrio cholerae

[denovogenes]_108026	83.40	7e-156	fpla:A4U99_08855	K03526	gcpE, ispG	(E)-4-hydroxy-3-methylbut-2-enyl-diphosphate synthase	1.17.7.1 1.17.7.3	Metabolism--Metabolism of terpenoids and polyketides--Terpenoid backbone biosynthesis|Metabolism--Global and overview maps--Metabolic pathways|Metabolism--Global and overview maps--Biosynthesis of secondary metabolites|Metabolism--Global and overview maps--Biosynthesis of antibiotics

[denovogenes]_108028	77.52	1e-150	cama:F384_03225	K01816	hyi, gip	hydroxypyruvate isomerase	5.3.1.22	Metabolism--Carbohydrate metabolism--Glyoxylate and dicarboxylate metabolism|Metabolism--Global and overview maps--Metabolic pathways

[denovogenes]_10803	47.09	0.0	cbut:ATN24_20465	K07012	cas3	CRISPR-associated endonuclease/helicase Cas3	3.1.-.- 3.6.4.-	--

[denovogenes]_108030	99.61	0.0	ebe:B21_02658	K07497	K07497	putative transposase	--	--

[denovogenes]_108031	89.92	2e-152	bprs:CK3_00760	K07031	hddA	D-glycero-alpha-D-manno-heptose-7-phosphate kinase	2.7.1.168	Metabolism--Glycan biosynthesis and metabolism--Lipopolysaccharide biosynthesis

[denovogenes]_108032	76.83	1e-142	rob:CK5_16500	K02112	ATPF1B, atpD	F-type H+-transporting ATPase subunit beta	3.6.3.14	Metabolism--Energy metabolism--Oxidative phosphorylation|Metabolism--Energy metabolism--Photosynthesis|Metabolism--Global and overview maps--Metabolic pathways

[denovogenes]_108034	86.82	3e-155	ibu:IB211_01641	K00052	leuB	3-isopropylmalate dehydrogenase	1.1.1.85	Metabolism--Amino acid metabolism--Valine, leucine and isoleucine biosynthesis|Metabolism--Carbohydrate metabolism--C5-Branched dibasic acid metabolism|Metabolism--Global and overview maps--Metabolic pathways|Metabolism--Global and overview maps--Biosynthesis of secondary metabolites|Metabolism--Global and overview maps--2-Oxocarboxylic acid metabolism|Metabolism--Global and overview maps--Biosynthesis of amino acids

[denovogenes]_108035	50.19	7e-91	elm:ELI_3858	K01992	ABC-2.P	ABC-2 type transport system permease protein	--	--

[denovogenes]_108038	64.80	3e-109	eyy:EGYY_08300	K02030	ABC.PA.S	polar amino acid transport system substrate-binding protein	--	--

[denovogenes]_10804	100.00	0.0	cpf:CPF_2429	K03070	secA	preprotein translocase subunit SecA	--	Cellular Processes--Cellular community - prokaryotes--Quorum sensing|Genetic Information Processing--Folding, sorting and degradation--Protein export|Environmental Information Processing--Membrane transport--Bacterial secretion system

[denovogenes]_108040	86.87	3e-159	bdo:EL88_10465	K02005	ABC.CD.TX	HlyD family secretion protein	--	--

[denovogenes]_108041	51.94	5e-86	ccm:Ccan_04140	K00700	GBE1, glgB	1,4-alpha-glucan branching enzyme	2.4.1.18	Metabolism--Carbohydrate metabolism--Starch and sucrose metabolism|Metabolism--Global and overview maps--Metabolic pathways|Metabolism--Global and overview maps--Biosynthesis of secondary metabolites

[denovogenes]_108042	76.45	6e-149	pdf:CD630DERM_12790	K04487	iscS, NFS1	cysteine desulfurase	2.8.1.7	Metabolism--Metabolism of cofactors and vitamins--Thiamine metabolism|Metabolism--Global and overview maps--Metabolic pathways|Genetic Information Processing--Folding, sorting and degradation--Sulfur relay system

[denovogenes]_108046	59.85	6e-98	fpla:A4U99_09610	K03631	recN	DNA repair protein RecN (Recombination protein N)	--	--

[denovogenes]_108050	71.04	3e-132	pdf:CD630DERM_02240	K01945	purD	phosphoribosylamine--glycine ligase	6.3.4.13	Metabolism--Nucleotide metabolism--Purine metabolism|Metabolism--Global and overview maps--Metabolic pathways|Metabolism--Global and overview maps--Biosynthesis of secondary metabolites|Metabolism--Global and overview maps--Biosynthesis of antibiotics

[denovogenes]_108053	49.15	2e-95	eel:EUBELI_01267	K01448	E3.5.1.28B, amiA, amiB, amiC	N-acetylmuramoyl-L-alanine amidase	3.5.1.28	Human Diseases--Drug resistance: Antimicrobial--Cationic antimicrobial peptide (CAMP) resistance

[denovogenes]_108054	71.81	1e-131	rim:ROI_29380	K18640	parM	plasmid segregation protein ParM	--	--

[denovogenes]_108056	85.33	5e-159	cso:CLS_13180	K01745	hutH, HAL	histidine ammonia-lyase	4.3.1.3	Metabolism--Amino acid metabolism--Histidine metabolism|Metabolism--Global and overview maps--Metabolic pathways

[denovogenes]_108059	53.28	7e-88	pru:PRU_2412	K06921	K06921	uncharacterized protein	--	--

[denovogenes]_108060	100.00	0.0	boa:Bovatus_01262	K00874	kdgK	2-dehydro-3-deoxygluconokinase	2.7.1.45	Metabolism--Carbohydrate metabolism--Pentose phosphate pathway|Metabolism--Global and overview maps--Metabolic pathways|Metabolism--Global and overview maps--Microbial metabolism in diverse environments|Metabolism--Global and overview maps--Carbon metabolism

[denovogenes]_108061	71.54	2e-135	bprm:CL3_11490	K01868	TARS, thrS	threonyl-tRNA synthetase	6.1.1.3	Genetic Information Processing--Translation--Aminoacyl-tRNA biosynthesis

[denovogenes]_108063	50.98	1e-85	bon:A361_23270	K19421	epsC	polysaccharide biosynthesis protein EpsC	--	--

[denovogenes]_108064	64.34	2e-114	byl:A4V09_10090	K11105	cvrA, nhaP2	cell volume regulation protein A	--	--

[denovogenes]_108065	83.40	4e-154	cso:CLS_10100	K03147	--	--	--	Metabolism--Metabolism of cofactors and vitamins--Thiamine metabolism|Metabolism--Global and overview maps--Metabolic pathways

[denovogenes]_108067	94.57	4e-162	pfre:RM25_2043	K02020	modA	molybdate transport system substrate-binding protein	--	Environmental Information Processing--Membrane transport--ABC transporters

[denovogenes]_108068	40.50	3e-20	aka:TKWG_17130	K01092	E3.1.3.25, IMPA, suhB	myo-inositol-1(or 4)-monophosphatase	3.1.3.25	Metabolism--Biosynthesis of other secondary metabolites--Streptomycin biosynthesis|Metabolism--Carbohydrate metabolism--Inositol phosphate metabolism|Metabolism--Global and overview maps--Metabolic pathways|Environmental Information Processing--Signal transduction--Phosphatidylinositol signaling system

[denovogenes]_108070	100.00	0.0	btho:Btheta7330_02518	K01241	amn	AMP nucleosidase	3.2.2.4	Metabolism--Nucleotide metabolism--Purine metabolism

[denovogenes]_108071	83.52	2e-162	pdi:BDI_1113	K02823	pyrDII	dihydroorotate dehydrogenase electron transfer subunit	--	Metabolism--Nucleotide metabolism--Pyrimidine metabolism|Metabolism--Global and overview maps--Metabolic pathways

[denovogenes]_108072	87.60	7e-171	pdi:BDI_1116	K01241	amn	AMP nucleosidase	3.2.2.4	Metabolism--Nucleotide metabolism--Purine metabolism

[denovogenes]_108074	68.55	1e-106	pfr:PFREUD_07760	K05895	cobK-cbiJ	precorrin-6A/cobalt-precorrin-6A reductase	1.3.1.54 1.3.1.106	Metabolism--Metabolism of cofactors and vitamins--Porphyrin and chlorophyll metabolism|Metabolism--Global and overview maps--Metabolic pathways

[denovogenes]_108075	44.26	2e-40	bln:Blon_1528	K07741	antB	anti-repressor protein	--	--

[denovogenes]_108077	87.30	2e-160	pdi:BDI_2591	K03269	lpxH	UDP-2,3-diacylglucosamine hydrolase	3.6.1.54	Metabolism--Glycan biosynthesis and metabolism--Lipopolysaccharide biosynthesis|Metabolism--Global and overview maps--Metabolic pathways

[denovogenes]_108078	64.10	2e-111	pdi:BDI_0402	K01126	E3.1.4.46, glpQ, ugpQ	glycerophosphoryl diester phosphodiesterase	3.1.4.46	Metabolism--Lipid metabolism--Glycerophospholipid metabolism

[denovogenes]_108079	58.13	5e-94	dvm:DvMF_0345	K01092	E3.1.3.25, IMPA, suhB	myo-inositol-1(or 4)-monophosphatase	3.1.3.25	Metabolism--Biosynthesis of other secondary metabolites--Streptomycin biosynthesis|Metabolism--Carbohydrate metabolism--Inositol phosphate metabolism|Metabolism--Global and overview maps--Metabolic pathways|Environmental Information Processing--Signal transduction--Phosphatidylinositol signaling system

[denovogenes]_108081	100.00	0.0	paeo:M801_4632	K00528	fpr	ferredoxin--NADP+ reductase	1.18.1.2	--

[denovogenes]_108088	60.23	2e-117	ddn:DND132_1270	K14977	ylbA, UGHY	(S)-ureidoglycine aminohydrolase	3.5.3.26	Metabolism--Nucleotide metabolism--Purine metabolism|Metabolism--Global and overview maps--Microbial metabolism in diverse environments

[denovogenes]_108089	99.22	0.0	cfd:CFNIH1_12685	K01816	hyi, gip	hydroxypyruvate isomerase	5.3.1.22	Metabolism--Carbohydrate metabolism--Glyoxylate and dicarboxylate metabolism|Metabolism--Global and overview maps--Metabolic pathways

[denovogenes]_10809	70.56	0.0	cdg:CDBI1_03585	K03657	uvrD, pcrA	DNA helicase II / ATP-dependent DNA helicase PcrA	3.6.4.12	Genetic Information Processing--Replication and repair--Nucleotide excision repair|Genetic Information Processing--Replication and repair--Mismatch repair

[denovogenes]_108093	100.00	0.0	paeo:M801_3633	K00677	lpxA	UDP-N-acetylglucosamine acyltransferase	2.3.1.129	Metabolism--Glycan biosynthesis and metabolism--Lipopolysaccharide biosynthesis|Metabolism--Global and overview maps--Metabolic pathways|Human Diseases--Drug resistance: Antimicrobial--Cationic antimicrobial peptide (CAMP) resistance

[denovogenes]_108094	56.75	1e-100	pmuc:ING2E5A_1128	K02564	nagB, GNPDA	glucosamine-6-phosphate deaminase	3.5.99.6	Metabolism--Carbohydrate metabolism--Amino sugar and nucleotide sugar metabolism|Metabolism--Global and overview maps--Metabolic pathways

[denovogenes]_108096	53.75	2e-90	doa:AXF15_04700	K02050	ABC.SN.P	NitT/TauT family transport system permease protein	--	--

[denovogenes]_108097	49.06	8e-70	dvm:DvMF_1232	K02379	fdhD	FdhD protein	--	--

[denovogenes]_10810	99.12	0.0	efn:DENG_02455	K18149	pbp5, pbpC, pbp3	penicillin-binding protein	--	Human Diseases--Drug resistance: Antimicrobial--beta-Lactam resistance

[denovogenes]_108100	97.29	0.0	bfs:BF9343_2522	K00978	rfbF	glucose-1-phosphate cytidylyltransferase	2.7.7.33	Metabolism--Carbohydrate metabolism--Starch and sucrose metabolism|Metabolism--Carbohydrate metabolism--Amino sugar and nucleotide sugar metabolism|Metabolism--Global and overview maps--Metabolic pathways

[denovogenes]_108101	43.82	4e-64	ebf:D782_2535	K00065	kduD	2-deoxy-D-gluconate 3-dehydrogenase	1.1.1.125	Metabolism--Carbohydrate metabolism--Pentose and glucuronate interconversions

[denovogenes]_108102	99.61	0.0	fpla:A4U99_16575	K02072	metI	D-methionine transport system permease protein	--	Environmental Information Processing--Membrane transport--ABC transporters

[denovogenes]_108105	66.80	4e-121	fpr:FP2_05300	K03465	thyX, thy1	thymidylate synthase (FAD)	2.1.1.148	Metabolism--Nucleotide metabolism--Pyrimidine metabolism|Metabolism--Metabolism of cofactors and vitamins--One carbon pool by folate|Metabolism--Global and overview maps--Metabolic pathways

[denovogenes]_108107	92.25	3e-180	bfg:BF638R_2441	K02823	pyrDII	dihydroorotate dehydrogenase electron transfer subunit	--	Metabolism--Nucleotide metabolism--Pyrimidine metabolism|Metabolism--Global and overview maps--Metabolic pathways

[denovogenes]_108108	98.84	0.0	bfb:VU15_10565	K01241	amn	AMP nucleosidase	3.2.2.4	Metabolism--Nucleotide metabolism--Purine metabolism

[denovogenes]_10811	65.40	0.0	fnt:HMPREF0405_00501	K01915	glnA, GLUL	glutamine synthetase	6.3.1.2	Metabolism--Amino acid metabolism--Arginine biosynthesis|Metabolism--Amino acid metabolism--Alanine, aspartate and glutamate metabolism|Metabolism--Carbohydrate metabolism--Glyoxylate and dicarboxylate metabolism|Metabolism--Energy metabolism--Nitrogen metabolism|Metabolism--Global and overview maps--Metabolic pathways|Metabolism--Global and overview maps--Microbial metabolism in diverse environments|Metabolism--Global and overview maps--Biosynthesis of amino acids|Environmental Information Processing--Signal transduction--Two-component system|Organismal Systems--Nervous system--Glutamatergic synapse|Organismal Systems--Nervous system--GABAergic synapse

[denovogenes]_108110	50.98	6e-79	fpr:FP2_02170	K03437	spoU	RNA methyltransferase, TrmH family	--	--

[denovogenes]_108112	100.00	9e-180	ecoh:ECRM13516_4496	K03228	yscT, sctT, hrcT	type III secretion protein T	--	Environmental Information Processing--Membrane transport--Bacterial secretion system

[denovogenes]_108117	99.61	0.0	paeo:M801_2929	K03424	--	--	--	--

[denovogenes]_108119	100.00	4e-180	paeo:M801_4360	K00655	plsC	1-acyl-sn-glycerol-3-phosphate acyltransferase	2.3.1.51	Metabolism--Lipid metabolism--Glycerolipid metabolism|Metabolism--Lipid metabolism--Glycerophospholipid metabolism|Metabolism--Global and overview maps--Metabolic pathways|Metabolism--Global and overview maps--Biosynthesis of secondary metabolites

[denovogenes]_10812	100.00	0.0	pvl:AOB99_03495	K05802	kefA, bspA, aefA	potassium efflux system protein	--	--

[denovogenes]_108120	45.42	3e-49	dfi:AXF13_04515	K02574	napH	ferredoxin-type protein NapH	--	--

[denovogenes]_108121	100.00	0.0	paeo:M801_1867	K01069	E3.1.2.6, gloB	hydroxyacylglutathione hydrolase	3.1.2.6	Metabolism--Carbohydrate metabolism--Pyruvate metabolism

[denovogenes]_108122	66.06	1e-98	dvm:DvMF_2432	K03222	yscJ, sctJ, hrcJ	type III secretion protein J	--	Environmental Information Processing--Membrane transport--Bacterial secretion system

[denovogenes]_108123	58.75	3e-111	eha:Ethha_1446	K00598	tam	trans-aconitate 2-methyltransferase	2.1.1.144	--

[denovogenes]_108125	100.00	0.0	paeg:AI22_06970	K02030	ABC.PA.S	polar amino acid transport system substrate-binding protein	--	--

[denovogenes]_108127	100.00	0.0	paeo:M801_3382	K00528	fpr	ferredoxin--NADP+ reductase	1.18.1.2	--

[denovogenes]_108129	92.25	6e-178	bfb:VU15_16760	K03424	--	--	--	--

[denovogenes]_10813	93.97	0.0	bhl:Bache_0864	K01874	MARS, metG	methionyl-tRNA synthetase	6.1.1.10	Metabolism--Metabolism of other amino acids--Selenocompound metabolism|Genetic Information Processing--Translation--Aminoacyl-tRNA biosynthesis

[denovogenes]_108131	61.54	9e-103	pdi:BDI_3122	K00865	glxK, garK	glycerate 2-kinase	2.7.1.165	Metabolism--Amino acid metabolism--Glycine, serine and threonine metabolism|Metabolism--Lipid metabolism--Glycerolipid metabolism|Metabolism--Carbohydrate metabolism--Glyoxylate and dicarboxylate metabolism|Metabolism--Global and overview maps--Metabolic pathways|Metabolism--Global and overview maps--Microbial metabolism in diverse environments|Metabolism--Global and overview maps--Biosynthesis of antibiotics

[denovogenes]_108132	100.00	0.0	cfd:CFNIH1_21375	K02500	hisF	cyclase	4.1.3.-	Metabolism--Amino acid metabolism--Histidine metabolism|Metabolism--Global and overview maps--Metabolic pathways|Metabolism--Global and overview maps--Biosynthesis of secondary metabolites|Metabolism--Global and overview maps--Biosynthesis of amino acids

[denovogenes]_108133	99.61	0.0	cfd:CFNIH1_23860	K01933	purM	phosphoribosylformylglycinamidine cyclo-ligase	6.3.3.1	Metabolism--Nucleotide metabolism--Purine metabolism|Metabolism--Global and overview maps--Metabolic pathways|Metabolism--Global and overview maps--Biosynthesis of secondary metabolites|Metabolism--Global and overview maps--Biosynthesis of antibiotics

[denovogenes]_108135	99.61	0.0	cfd:CFNIH1_07150	K07640	cpxA	two-component system, OmpR family, sensor histidine kinase CpxA	2.7.13.3	Human Diseases--Drug resistance: Antimicrobial--Cationic antimicrobial peptide (CAMP) resistance|Environmental Information Processing--Signal transduction--Two-component system

[denovogenes]_108136	96.34	1e-176	lacy:A4V08_14760	K01126	E3.1.4.46, glpQ, ugpQ	glycerophosphoryl diester phosphodiesterase	3.1.4.46	Metabolism--Lipid metabolism--Glycerophospholipid metabolism

[denovogenes]_108138	77.52	2e-152	csh:Closa_1599	K02823	pyrDII	dihydroorotate dehydrogenase electron transfer subunit	--	Metabolism--Nucleotide metabolism--Pyrimidine metabolism|Metabolism--Global and overview maps--Metabolic pathways

[denovogenes]_108139	99.61	0.0	paeo:M801_3532	K16081	algE	alginate production protein	--	--

[denovogenes]_108140	44.31	2e-75	rso:RSp1110	K18900	bpeT	LysR family transcriptional regulator, regulator for bpeEF and oprC	--	--

[denovogenes]_108141	50.39	9e-75	bcel:BcellWH2_05172	K02004	ABC.CD.P	putative ABC transport system permease protein	--	--

[denovogenes]_108142	63.64	4e-98	cgo:Corgl_0773	K06997	yggS, PROSC	PLP dependent protein	--	--

[denovogenes]_108144	100.00	0.0	stb:SGPB_0409	K03565	recX	regulatory protein	--	--

[denovogenes]_108145	95.88	4e-174	lacy:A4V08_22285	K00059	fabG	3-oxoacyl-[acyl-carrier protein] reductase	1.1.1.100	Metabolism--Lipid metabolism--Fatty acid biosynthesis|Metabolism--Metabolism of cofactors and vitamins--Biotin metabolism|Metabolism--Lipid metabolism--Biosynthesis of unsaturated fatty acids|Metabolism--Global and overview maps--Metabolic pathways|Metabolism--Global and overview maps--Fatty acid metabolism

[denovogenes]_108146	98.84	0.0	cfd:CFNIH1_09605	K03424	--	--	--	--

[denovogenes]_108147	50.00	2e-75	nth:Nther_1461	K01358	clpP, CLPP	ATP-dependent Clp protease, protease subunit	3.4.21.92	Cellular Processes--Cell growth and death--Cell cycle - Caulobacter|Organismal Systems--Aging--Longevity regulating pathway - worm

[denovogenes]_108148	100.00	0.0	cfd:CFNIH1_02735	K19775	exuR	GntR family transcriptional regulator, hexuronate regulon transcriptional repressor	--	--

[denovogenes]_10815	77.30	0.0	rto:RTO_26980	K03169	topB	DNA topoisomerase III	5.99.1.2	--

[denovogenes]_108150	65.12	6e-116	cgo:Corgl_1253	K01803	TPI, tpiA	triosephosphate isomerase (TIM)	5.3.1.1	Metabolism--Carbohydrate metabolism--Glycolysis / Gluconeogenesis|Metabolism--Carbohydrate metabolism--Fructose and mannose metabolism|Metabolism--Carbohydrate metabolism--Inositol phosphate metabolism|Metabolism--Energy metabolism--Carbon fixation in photosynthetic organisms|Metabolism--Global and overview maps--Metabolic pathways|Metabolism--Global and overview maps--Biosynthesis of secondary metabolites|Metabolism--Global and overview maps--Microbial metabolism in diverse environments|Metabolism--Global and overview maps--Biosynthesis of antibiotics|Metabolism--Global and overview maps--Carbon metabolism|Metabolism--Global and overview maps--Biosynthesis of amino acids

[denovogenes]_108154	40.15	9e-14	scy:SCATT_38840	K04761	oxyR	LysR family transcriptional regulator, hydrogen peroxide-inducible genes activator	--	Cellular Processes--Cellular community - prokaryotes--Biofilm formation - Escherichia coli

[denovogenes]_108156	87.25	2e-165	pdi:BDI_2402	K02065	mlaF, linL, mkl	phospholipid/cholesterol/gamma-HCH transport system ATP-binding protein	--	Environmental Information Processing--Membrane transport--ABC transporters

[denovogenes]_108160	100.00	5e-178	paec:M802_1486	K02421	fliR	flagellar biosynthetic protein FliR	--	Cellular Processes--Cell motility--Flagellar assembly

[denovogenes]_108161	54.62	5e-87	ate:Athe_2157	K02419	fliP	flagellar biosynthetic protein FliP	--	Cellular Processes--Cell motility--Flagellar assembly

[denovogenes]_108162	79.84	4e-152	shi:Shel_14880	K01867	WARS, trpS	tryptophanyl-tRNA synthetase	6.1.1.2	Genetic Information Processing--Translation--Aminoacyl-tRNA biosynthesis

[denovogenes]_108163	83.08	1e-161	shi:Shel_21550	K00600	glyA, SHMT	glycine hydroxymethyltransferase	2.1.2.1	Metabolism--Amino acid metabolism--Glycine, serine and threonine metabolism|Metabolism--Metabolism of other amino acids--Cyanoamino acid metabolism|Metabolism--Carbohydrate metabolism--Glyoxylate and dicarboxylate metabolism|Metabolism--Metabolism of cofactors and vitamins--One carbon pool by folate|Metabolism--Energy metabolism--Methane metabolism|Metabolism--Global and overview maps--Metabolic pathways|Metabolism--Global and overview maps--Biosynthesis of secondary metabolites|Metabolism--Global and overview maps--Microbial metabolism in diverse environments|Metabolism--Global and overview maps--Biosynthesis of antibiotics|Metabolism--Global and overview maps--Carbon metabolism|Metabolism--Global and overview maps--Biosynthesis of amino acids|Human Diseases--Drug resistance: Antineoplastic--Antifolate resistance

[denovogenes]_108165	98.06	1e-176	lacy:A4V08_04830	K07238	TC.ZIP, zupT, ZRT3, ZIP2	zinc transporter, ZIP family	--	--

[denovogenes]_108166	98.84	0.0	elf:LF82_1250	K00880	lyxK	L-xylulokinase	2.7.1.53	Metabolism--Carbohydrate metabolism--Pentose and glucuronate interconversions|Metabolism--Carbohydrate metabolism--Ascorbate and aldarate metabolism

[denovogenes]_108167	82.56	1e-145	rbc:BN938_2810	K00076	hdhA	7-alpha-hydroxysteroid dehydrogenase	1.1.1.159	--

[denovogenes]_108168	57.48	4e-100	fpr:FP2_10620	K07720	yesN	two-component system, response regulator YesN	--	Environmental Information Processing--Signal transduction--Two-component system

[denovogenes]_108169	68.92	2e-128	lacy:A4V08_31180	K02405	fliA	RNA polymerase sigma factor for flagellar operon FliA	--	Environmental Information Processing--Signal transduction--Two-component system|Cellular Processes--Cellular community - prokaryotes--Biofilm formation - Pseudomonas aeruginosa|Cellular Processes--Cellular community - prokaryotes--Biofilm formation - Escherichia coli|Cellular Processes--Cell motility--Flagellar assembly|Cellular Processes--Cellular community - prokaryotes--Biofilm formation - Vibrio cholerae

[denovogenes]_108171	76.10	5e-138	byl:A4V09_10700	K01448	E3.5.1.28B, amiA, amiB, amiC	N-acetylmuramoyl-L-alanine amidase	3.5.1.28	Human Diseases--Drug resistance: Antimicrobial--Cationic antimicrobial peptide (CAMP) resistance

[denovogenes]_108174	77.13	5e-153	cpy:Cphy_3400	K02026	ABC.MS.P1	multiple sugar transport system permease protein	--	--

[denovogenes]_108175	55.82	4e-101	ctyk:CTK_C13930	K02013	ABC.FEV.A	iron complex transport system ATP-binding protein	3.6.3.34	Environmental Information Processing--Membrane transport--ABC transporters

[denovogenes]_108176	94.96	0.0	csh:Closa_2347	K03091	SIG3.4	RNA polymerase sporulation-specific sigma factor	--	--

[denovogenes]_108180	50.00	8e-87	pmq:PM3016_2536	K17241	aguE	alpha-1,4-digalacturonate transport system substrate-binding protein	--	Environmental Information Processing--Membrane transport--ABC transporters

[denovogenes]_108181	100.00	2e-178	pdf:CD630DERM_04800	K20492	nisG, spaG, cprC	lantibiotic transport system permease protein	--	Environmental Information Processing--Membrane transport--ABC transporters|Environmental Information Processing--Signal transduction--Two-component system|Cellular Processes--Cellular community - prokaryotes--Quorum sensing

[denovogenes]_108182	40.15	1e-18	fbt:D770_20225	K04763	xerD	integrase/recombinase XerD	--	--

[denovogenes]_108184	96.90	0.0	cfd:CFNIH1_16775	K05887	ydiB	quinate/shikimate dehydrogenase	1.1.1.282	Metabolism--Amino acid metabolism--Phenylalanine, tyrosine and tryptophan biosynthesis|Metabolism--Global and overview maps--Metabolic pathways|Metabolism--Global and overview maps--Biosynthesis of secondary metabolites|Metabolism--Global and overview maps--Biosynthesis of antibiotics

[denovogenes]_108187	73.23	1e-128	bhl:Bache_0210	K02343	DPO3G, dnaX	DNA polymerase III subunit gamma/tau	2.7.7.7	Metabolism--Nucleotide metabolism--Purine metabolism|Metabolism--Nucleotide metabolism--Pyrimidine metabolism|Metabolism--Global and overview maps--Metabolic pathways|Genetic Information Processing--Replication and repair--DNA replication|Genetic Information Processing--Replication and repair--Mismatch repair|Genetic Information Processing--Replication and repair--Homologous recombination

[denovogenes]_10819	61.71	0.0	hpy:HP1472	K00571	E2.1.1.72	site-specific DNA-methyltransferase (adenine-specific)	2.1.1.72	--

[denovogenes]_108190	99.61	0.0	pdi:BDI_1116	K01241	amn	AMP nucleosidase	3.2.2.4	Metabolism--Nucleotide metabolism--Purine metabolism

[denovogenes]_108191	99.22	0.0	cfd:CFNIH1_02430	K15777	DOPA	4,5-DOPA dioxygenase extradiol	1.13.11.-	Metabolism--Biosynthesis of other secondary metabolites--Betalain biosynthesis

[denovogenes]_108192	99.61	0.0	bcel:BcellWH2_05369	K01241	amn	AMP nucleosidase	3.2.2.4	Metabolism--Nucleotide metabolism--Purine metabolism

[denovogenes]_108194	78.99	2e-149	rto:RTO_20090	K00058	serA, PHGDH	D-3-phosphoglycerate dehydrogenase	1.1.1.95	Metabolism--Amino acid metabolism--Glycine, serine and threonine metabolism|Metabolism--Energy metabolism--Methane metabolism|Metabolism--Global and overview maps--Metabolic pathways|Metabolism--Global and overview maps--Microbial metabolism in diverse environments|Metabolism--Global and overview maps--Biosynthesis of antibiotics|Metabolism--Global and overview maps--Carbon metabolism|Metabolism--Global and overview maps--Biosynthesis of amino acids

[denovogenes]_108197	99.22	0.0	pvl:AOB99_15715	K02036	pstB	phosphate transport system ATP-binding protein	3.6.3.27	Environmental Information Processing--Membrane transport--ABC transporters

[denovogenes]_108199	41.10	1e-52	pbj:VN24_22905	K02026	ABC.MS.P1	multiple sugar transport system permease protein	--	--

[denovogenes]_108202	40.91	4e-65	ndi:NDAI_0A08090	K00697	otsA	trehalose 6-phosphate synthase	2.4.1.15	Metabolism--Carbohydrate metabolism--Starch and sucrose metabolism|Metabolism--Global and overview maps--Metabolic pathways

[denovogenes]_108209	97.29	0.0	cfd:CFNIH1_03515	K01674	cah	carbonic anhydrase	4.2.1.1	Metabolism--Energy metabolism--Nitrogen metabolism

[denovogenes]_108210	46.89	1e-73	rim:ROI_03480	K02529	lacI, galR	LacI family transcriptional regulator	--	--

[denovogenes]_108211	44.90	3e-62	fgl:EM308_12595	K02100	araE	MFS transporter, SP family, arabinose:H+ symporter	--	--

[denovogenes]_108212	99.61	0.0	sot:102599894	K02133	ATPeF1B, ATP5B, ATP2	F-type H+-transporting ATPase subunit beta	3.6.3.14	Metabolism--Energy metabolism--Oxidative phosphorylation|Metabolism--Global and overview maps--Metabolic pathways|Human Diseases--Neurodegenerative diseases--Alzheimer's disease|Human Diseases--Neurodegenerative diseases--Parkinson's disease|Human Diseases--Neurodegenerative diseases--Huntington's disease

[denovogenes]_108214	92.25	2e-169	pvl:AOB99_02225	K00005	gldA	glycerol dehydrogenase	1.1.1.6	Metabolism--Lipid metabolism--Glycerolipid metabolism|Metabolism--Carbohydrate metabolism--Propanoate metabolism|Metabolism--Global and overview maps--Metabolic pathways

[denovogenes]_108215	100.00	8e-180	pdf:CD630DERM_12600	K03311	TC.LIVCS	branched-chain amino acid:cation transporter, LIVCS family	--	--

[denovogenes]_108216	64.44	4e-112	soi:I872_03765	K01239	iunH	purine nucleosidase	3.2.2.1	Metabolism--Nucleotide metabolism--Purine metabolism|Metabolism--Metabolism of cofactors and vitamins--Nicotinate and nicotinamide metabolism|Metabolism--Global and overview maps--Metabolic pathways

[denovogenes]_108219	91.89	2e-175	pdi:BDI_3191	K08303	K08303	putative protease	3.4.-.-	Human Diseases--Infectious diseases: Bacterial--Epithelial cell signaling in Helicobacter pylori infection

[denovogenes]_10822	99.85	0.0	bvu:BVU_0882	K01874	MARS, metG	methionyl-tRNA synthetase	6.1.1.10	Metabolism--Metabolism of other amino acids--Selenocompound metabolism|Genetic Information Processing--Translation--Aminoacyl-tRNA biosynthesis

[denovogenes]_108221	80.16	3e-135	bxy:BXY_00590	K07085	K07085	putative transport protein	--	--

[denovogenes]_108222	79.46	5e-154	afd:Alfi_1494	K03593	mrp, NUBPL	ATP-binding protein involved in chromosome partitioning	--	--

[denovogenes]_108223	99.23	8e-177	pvl:AOB99_15910	K07793	tctA	putative tricarboxylic transport membrane protein	--	Environmental Information Processing--Signal transduction--Two-component system

[denovogenes]_108224	100.00	3e-177	pdf:CD630DERM_35250	K02012	afuA, fbpA	iron(III) transport system substrate-binding protein	--	Environmental Information Processing--Membrane transport--ABC transporters

[denovogenes]_108227	99.23	0.0	cif:AL515_01610	K01710	E4.2.1.46, rfbB, rffG	dTDP-glucose 4,6-dehydratase	4.2.1.46	Metabolism--Biosynthesis of other secondary metabolites--Streptomycin biosynthesis|Metabolism--Metabolism of terpenoids and polyketides--Polyketide sugar unit biosynthesis|Metabolism--Biosynthesis of other secondary metabolites--Acarbose and validamycin biosynthesis|Metabolism--Metabolism of terpenoids and polyketides--Biosynthesis of vancomycin group antibiotics|Metabolism--Global and overview maps--Biosynthesis of antibiotics

[denovogenes]_108228	40.89	3e-63	ecoo:ECRM13514_2076	K07310	ynfF	Tat-targeted selenate reductase subunit YnfF	1.97.1.9	Metabolism--Metabolism of other amino acids--Selenocompound metabolism

[denovogenes]_108229	91.50	3e-157	bdh:GV66_06040	K03321	TC.SULP	sulfate permease, SulP family	--	--

[denovogenes]_108234	96.14	0.0	bxy:BXY_34250	K01875	SARS, serS	seryl-tRNA synthetase	6.1.1.11	Genetic Information Processing--Translation--Aminoacyl-tRNA biosynthesis

[denovogenes]_108236	60.62	6e-114	clb:Clo1100_0966	K02227	cbiB, cobD	adenosylcobinamide-phosphate synthase	6.3.1.10	Metabolism--Metabolism of cofactors and vitamins--Porphyrin and chlorophyll metabolism|Metabolism--Global and overview maps--Metabolic pathways

[denovogenes]_108239	43.46	5e-59	btrm:SAMEA390648703285	K03762	proP	MFS transporter, MHS family, proline/betaine transporter	--	--

[denovogenes]_108240	45.06	8e-59	cpra:CPter91_2502	K03762	proP	MFS transporter, MHS family, proline/betaine transporter	--	--

[denovogenes]_108243	100.00	0.0	bbrn:B2258_1699	K02037	pstC	phosphate transport system permease protein	--	Environmental Information Processing--Membrane transport--ABC transporters

[denovogenes]_108245	49.19	8e-77	bon:A361_23270	K19421	epsC	polysaccharide biosynthesis protein EpsC	--	--

[denovogenes]_10825	51.67	0.0	ccel:CCDG5_0432	K03655	recG	ATP-dependent DNA helicase RecG	3.6.4.12	Genetic Information Processing--Replication and repair--Homologous recombination

[denovogenes]_108252	94.98	4e-168	sdz:Asd1617_05341	K03655	recG	ATP-dependent DNA helicase RecG	3.6.4.12	Genetic Information Processing--Replication and repair--Homologous recombination

[denovogenes]_108253	100.00	0.0	cfd:CFNIH1_14635	K03633	mukF	chromosome partition protein MukF	--	--

[denovogenes]_108254	79.07	8e-144	lmob:BN419_3177	K01547	kdpB	K+-transporting ATPase ATPase B chain	3.6.3.12	Environmental Information Processing--Signal transduction--Two-component system

[denovogenes]_108259	99.61	0.0	boa:Bovatus_03348	K02477	K02477	two-component system, LytT family, response regulator	--	--

[denovogenes]_108260	43.41	6e-71	cct:CC1_22890	K03091	SIG3.4	RNA polymerase sporulation-specific sigma factor	--	--

[denovogenes]_108261	46.85	1e-78	coo:CCU_21690	K00286	proC	pyrroline-5-carboxylate reductase	1.5.1.2	Metabolism--Amino acid metabolism--Arginine and proline metabolism|Metabolism--Global and overview maps--Metabolic pathways|Metabolism--Global and overview maps--Biosynthesis of secondary metabolites|Metabolism--Global and overview maps--Biosynthesis of antibiotics|Metabolism--Global and overview maps--Biosynthesis of amino acids

[denovogenes]_108263	99.61	0.0	boa:Bovatus_00755	K02823	pyrDII	dihydroorotate dehydrogenase electron transfer subunit	--	Metabolism--Nucleotide metabolism--Pyrimidine metabolism|Metabolism--Global and overview maps--Metabolic pathways

[denovogenes]_108264	99.61	0.0	boa:Bovatus_00758	K01241	amn	AMP nucleosidase	3.2.2.4	Metabolism--Nucleotide metabolism--Purine metabolism

[denovogenes]_108266	63.78	2e-120	tur:AT726_07485	K03525	coaX	type III pantothenate kinase	2.7.1.33	Metabolism--Metabolism of cofactors and vitamins--Pantothenate and CoA biosynthesis|Metabolism--Global and overview maps--Metabolic pathways

[denovogenes]_108267	75.58	1e-147	bfg:BF638R_0779	K00978	rfbF	glucose-1-phosphate cytidylyltransferase	2.7.7.33	Metabolism--Carbohydrate metabolism--Starch and sucrose metabolism|Metabolism--Carbohydrate metabolism--Amino sugar and nucleotide sugar metabolism|Metabolism--Global and overview maps--Metabolic pathways

[denovogenes]_10827	96.32	0.0	bvu:BVU_0882	K01874	MARS, metG	methionyl-tRNA synthetase	6.1.1.10	Metabolism--Metabolism of other amino acids--Selenocompound metabolism|Genetic Information Processing--Translation--Aminoacyl-tRNA biosynthesis

[denovogenes]_108270	53.52	7e-99	sub:SUB0424	K02013	ABC.FEV.A	iron complex transport system ATP-binding protein	3.6.3.34	Environmental Information Processing--Membrane transport--ABC transporters

[denovogenes]_108274	99.61	0.0	eld:i02_3378	K09688	ABC-2.CPSE.P	capsular polysaccharide transport system permease protein	--	Environmental Information Processing--Membrane transport--ABC transporters

[denovogenes]_10828	100.00	0.0	bvu:BVU_0302	K03931	ygjK	putative isomerase	--	--

[denovogenes]_108281	41.28	3e-38	euc:EC1_02720	K01104	E3.1.3.48	protein-tyrosine phosphatase	3.1.3.48	--

[denovogenes]_108282	98.84	0.0	boa:Bovatus_02094	K03424	--	--	--	--

[denovogenes]_108284	50.19	4e-81	sbc:SbBS512_E1900	K03521	fixA, etfB	electron transfer flavoprotein beta subunit	--	--

[denovogenes]_108285	100.00	0.0	pvl:AOB99_11510	K03100	lepB, TPP	signal peptidase I	3.4.21.89	Cellular Processes--Cellular community - prokaryotes--Quorum sensing|Genetic Information Processing--Folding, sorting and degradation--Protein export

[denovogenes]_108286	100.00	0.0	pvl:AOB99_10505	K07308	dmsC	anaerobic dimethyl sulfoxide reductase subunit C (DMSO reductase anchor subunit)	--	Metabolism--Energy metabolism--Sulfur metabolism

[denovogenes]_108287	58.59	2e-103	fpr:FP2_28950	K10824	nikE	nickel transport system ATP-binding protein	3.6.3.24	Environmental Information Processing--Membrane transport--ABC transporters

[denovogenes]_10829	98.53	0.0	cif:AL515_00120	K01414	prlC	oligopeptidase A	3.4.24.70	--

[denovogenes]_108292	100.00	0.0	elp:P12B_c4041	K00864	glpK, GK	glycerol kinase	2.7.1.30	Metabolism--Lipid metabolism--Glycerolipid metabolism|Metabolism--Global and overview maps--Metabolic pathways|Organismal Systems--Endocrine system--PPAR signaling pathway|Organismal Systems--Environmental adaptation--Plant-pathogen interaction

[denovogenes]_108293	72.69	1e-116	cfp:CR44_08110	K07791	dcuA	anaerobic C4-dicarboxylate transporter DcuA	--	--

[denovogenes]_108296	82.17	1e-160	vpr:Vpar_1235	K06180	rluD	23S rRNA pseudouridine1911/1915/1917 synthase	5.4.99.23	--

[denovogenes]_108297	72.59	5e-130	bsa:Bacsa_1788	K06158	ABCF3	ATP-binding cassette, subfamily F, member 3	--	--

[denovogenes]_108299	77.99	2e-146	vpr:Vpar_0618	K01524	ppx-gppA	exopolyphosphatase / guanosine-5'-triphosphate,3'-diphosphate pyrophosphatase	3.6.1.11 3.6.1.40	Metabolism--Nucleotide metabolism--Purine metabolism

[denovogenes]_10830	40.30	7e-108	byl:A4V09_15395	K03406	mcp	methyl-accepting chemotaxis protein	--	Environmental Information Processing--Signal transduction--Two-component system|Cellular Processes--Cell motility--Bacterial chemotaxis

[denovogenes]_108303	99.23	0.0	bvu:BVU_0332	K07568	queA	S-adenosylmethionine:tRNA ribosyltransferase-isomerase	2.4.99.17	--

[denovogenes]_108304	71.04	4e-133	vpr:Vpar_1022	K19405	mcsB	protein arginine kinase	2.7.14.1	--

[denovogenes]_108305	94.59	3e-177	sly:100736527	K09872	PIP	aquaporin PIP	--	--

[denovogenes]_108307	98.46	0.0	ssj:SSON53_20625	K03654	recQ	ATP-dependent DNA helicase RecQ	3.6.4.12	Genetic Information Processing--Folding, sorting and degradation--RNA degradation

[denovogenes]_108308	47.48	4e-75	cpo:COPRO5265_0182	K03299	TC.GNTP	gluconate:H+ symporter, GntP family	--	--

[denovogenes]_108310	75.29	9e-148	efa:EF1348	K01215	dexB	glucan 1,6-alpha-glucosidase	3.2.1.70	--

[denovogenes]_108312	93.00	7e-177	cbv:U729_2573	K03091	SIG3.4	RNA polymerase sporulation-specific sigma factor	--	--

[denovogenes]_108314	73.15	7e-141	cbv:U729_2798	K00806	uppS	undecaprenyl diphosphate synthase	2.5.1.31	Metabolism--Metabolism of terpenoids and polyketides--Terpenoid backbone biosynthesis|Metabolism--Global and overview maps--Biosynthesis of secondary metabolites

[denovogenes]_108315	99.61	0.0	mmk:MU9_1830	K02029	ABC.PA.P	polar amino acid transport system permease protein	--	--

[denovogenes]_108316	100.00	0.0	mmk:MU9_1455	K21029	moeB	molybdopterin-synthase adenylyltransferase	2.7.7.80	Genetic Information Processing--Folding, sorting and degradation--Sulfur relay system

[denovogenes]_108317	99.22	0.0	mmk:MU9_10	K02013	ABC.FEV.A	iron complex transport system ATP-binding protein	3.6.3.34	Environmental Information Processing--Membrane transport--ABC transporters

[denovogenes]_108319	99.22	0.0	mmk:MU9_2439	K06074	ABC.VB12.A, btuD	vitamin B12 transport system ATP-binding protein	3.6.3.33	Environmental Information Processing--Membrane transport--ABC transporters

[denovogenes]_10832	90.00	0.0	lacy:A4V08_13485	K01531	mgtA, mgtB	Mg2+-importing ATPase	3.6.3.2	--

[denovogenes]_108320	100.00	2e-149	mmk:MU9_2498	K00761	upp, UPRT	uracil phosphoribosyltransferase	2.4.2.9	Metabolism--Nucleotide metabolism--Pyrimidine metabolism|Metabolism--Global and overview maps--Metabolic pathways

[denovogenes]_108321	99.61	0.0	mmk:MU9_2541	K08680	menH	2-succinyl-6-hydroxy-2,4-cyclohexadiene-1-carboxylate synthase	4.2.99.20	Metabolism--Metabolism of cofactors and vitamins--Ubiquinone and other terpenoid-quinone biosynthesis|Metabolism--Global and overview maps--Metabolic pathways|Metabolism--Global and overview maps--Biosynthesis of secondary metabolites

[denovogenes]_108322	93.39	6e-180	prg:RB151_040270	K09968	K09968	uncharacterized protein	--	--

[denovogenes]_108323	100.00	0.0	mmk:MU9_599	K00930	argB	acetylglutamate kinase	2.7.2.8	Metabolism--Amino acid metabolism--Arginine biosynthesis|Metabolism--Global and overview maps--Metabolic pathways|Metabolism--Global and overview maps--Biosynthesis of secondary metabolites|Metabolism--Global and overview maps--Biosynthesis of antibiotics|Metabolism--Global and overview maps--2-Oxocarboxylic acid metabolism|Metabolism--Global and overview maps--Biosynthesis of amino acids

[denovogenes]_108324	98.83	0.0	mmk:MU9_3340	K10831	tauB	taurine transport system ATP-binding protein	3.6.3.36	Metabolism--Energy metabolism--Sulfur metabolism|Environmental Information Processing--Membrane transport--ABC transporters

[denovogenes]_108325	99.61	0.0	bfb:VU15_01830	K00286	proC	pyrroline-5-carboxylate reductase	1.5.1.2	Metabolism--Amino acid metabolism--Arginine and proline metabolism|Metabolism--Global and overview maps--Metabolic pathways|Metabolism--Global and overview maps--Biosynthesis of secondary metabolites|Metabolism--Global and overview maps--Biosynthesis of antibiotics|Metabolism--Global and overview maps--Biosynthesis of amino acids

[denovogenes]_108326	58.96	5e-110	edu:LIU_00765	K02013	ABC.FEV.A	iron complex transport system ATP-binding protein	3.6.3.34	Environmental Information Processing--Membrane transport--ABC transporters

[denovogenes]_108327	79.77	1e-151	lacy:A4V08_05195	K09817	znuC	zinc transport system ATP-binding protein	3.6.3.-	Environmental Information Processing--Membrane transport--ABC transporters

[denovogenes]_10833	97.79	0.0	pfre:RM25_0014	K02470	gyrB	DNA gyrase subunit B	5.99.1.3	--

[denovogenes]_108330	47.81	9e-85	dmt:DESME_03480	K19285	nfrA1	FMN reductase (NADPH)	1.5.1.38	Metabolism--Metabolism of cofactors and vitamins--Riboflavin metabolism|Metabolism--Global and overview maps--Metabolic pathways

[denovogenes]_108332	100.00	0.0	bfb:VU15_12815	K02342	DPO3E, dnaQ	DNA polymerase III subunit epsilon	2.7.7.7	Metabolism--Nucleotide metabolism--Purine metabolism|Metabolism--Nucleotide metabolism--Pyrimidine metabolism|Metabolism--Global and overview maps--Metabolic pathways|Genetic Information Processing--Replication and repair--DNA replication|Genetic Information Processing--Replication and repair--Mismatch repair|Genetic Information Processing--Replication and repair--Homologous recombination

[denovogenes]_108333	62.60	2e-116	csb:CLSA_c00420	K07124	K07124	uncharacterized protein	--	--

[denovogenes]_108334	100.00	0.0	mmk:MU9_383	K13637	uxuR	GntR family transcriptional regulator, uxu operon transcriptional repressor	--	--

[denovogenes]_108335	100.00	0.0	mmk:MU9_326	K02029	ABC.PA.P	polar amino acid transport system permease protein	--	--

[denovogenes]_108336	44.35	7e-20	blq:L21SP5_03403	K02477	K02477	two-component system, LytT family, response regulator	--	--

[denovogenes]_108340	100.00	0.0	mmk:MU9_1062	K12529	--	--	--	Metabolism--Metabolism of other amino acids--Selenocompound metabolism

[denovogenes]_108342	99.61	0.0	efn:DENG_02555	K03928	yvaK	carboxylesterase	3.1.1.1	--

[denovogenes]_108345	81.71	2e-159	lacy:A4V08_04855	K05833	ABC.X4.A	putative ABC transport system ATP-binding protein	--	--

[denovogenes]_108346	65.76	7e-116	cbv:U729_2504	K02795	PTS-Man-EIIC, manY	PTS system, mannose-specific IIC component	--	Metabolism--Carbohydrate metabolism--Fructose and mannose metabolism|Metabolism--Carbohydrate metabolism--Amino sugar and nucleotide sugar metabolism|Metabolism--Global and overview maps--Metabolic pathways|Environmental Information Processing--Membrane transport--Phosphotransferase system (PTS)

[denovogenes]_108347	57.81	8e-101	prg:RB151_P104615	K12066	traK	conjugal transfer pilus assembly protein TraK	--	--

[denovogenes]_10835	94.21	0.0	pfr:PFREUD_12440	K01868	TARS, thrS	threonyl-tRNA synthetase	6.1.1.3	Genetic Information Processing--Translation--Aminoacyl-tRNA biosynthesis

[denovogenes]_108350	100.00	0.0	stb:SGPB_0298	K01962	accA	acetyl-CoA carboxylase carboxyl transferase subunit alpha	6.4.1.2	Metabolism--Lipid metabolism--Fatty acid biosynthesis|Metabolism--Carbohydrate metabolism--Pyruvate metabolism|Metabolism--Carbohydrate metabolism--Propanoate metabolism|Metabolism--Energy metabolism--Carbon fixation pathways in prokaryotes|Metabolism--Global and overview maps--Metabolic pathways|Metabolism--Global and overview maps--Biosynthesis of secondary metabolites|Metabolism--Global and overview maps--Microbial metabolism in diverse environments|Metabolism--Global and overview maps--Biosynthesis of antibiotics|Metabolism--Global and overview maps--Carbon metabolism|Metabolism--Global and overview maps--Fatty acid metabolism

[denovogenes]_108351	99.61	0.0	mmk:MU9_939	K00806	uppS	undecaprenyl diphosphate synthase	2.5.1.31	Metabolism--Metabolism of terpenoids and polyketides--Terpenoid backbone biosynthesis|Metabolism--Global and overview maps--Biosynthesis of secondary metabolites

[denovogenes]_108353	99.61	0.0	efs:EFS1_2478	K02746	PTS-Aga-EIIC, agaW	PTS system, N-acetylgalactosamine-specific IIC component	--	Metabolism--Carbohydrate metabolism--Galactose metabolism|Environmental Information Processing--Membrane transport--Phosphotransferase system (PTS)

[denovogenes]_108354	81.30	3e-147	pdf:CD630DERM_36720	K03496	parA, soj	chromosome partitioning protein	--	--

[denovogenes]_108357	62.81	1e-106	cad:Curi_c23440	K00059	fabG	3-oxoacyl-[acyl-carrier protein] reductase	1.1.1.100	Metabolism--Lipid metabolism--Fatty acid biosynthesis|Metabolism--Metabolism of cofactors and vitamins--Biotin metabolism|Metabolism--Lipid metabolism--Biosynthesis of unsaturated fatty acids|Metabolism--Global and overview maps--Metabolic pathways|Metabolism--Global and overview maps--Fatty acid metabolism

[denovogenes]_108360	100.00	0.0	bfs:BF9343_3617	K00210	E1.3.1.12	prephenate dehydrogenase	1.3.1.12	Metabolism--Amino acid metabolism--Phenylalanine, tyrosine and tryptophan biosynthesis|Metabolism--Biosynthesis of other secondary metabolites--Novobiocin biosynthesis|Metabolism--Global and overview maps--Metabolic pathways|Metabolism--Global and overview maps--Biosynthesis of secondary metabolites|Metabolism--Global and overview maps--Biosynthesis of antibiotics|Metabolism--Global and overview maps--Biosynthesis of amino acids

[denovogenes]_108362	98.83	0.0	efi:OG1RF_10401	K06183	rsuA	16S rRNA pseudouridine516 synthase	5.4.99.19	--

[denovogenes]_108364	58.59	8e-103	csb:CLSA_c00420	K07124	K07124	uncharacterized protein	--	--

[denovogenes]_108365	58.66	3e-103	erb:A4V01_02320	K02003	ABC.CD.A	putative ABC transport system ATP-binding protein	--	--

[denovogenes]_108366	79.30	3e-149	cpe:CPE0122	K02003	ABC.CD.A	putative ABC transport system ATP-binding protein	--	--

[denovogenes]_108368	93.39	3e-176	pdf:CD630DERM_26420	K03091	SIG3.4	RNA polymerase sporulation-specific sigma factor	--	--

[denovogenes]_10837	87.87	0.0	pdi:BDI_1854	K03798	ftsH, hflB	cell division protease FtsH	3.4.24.-	--

[denovogenes]_108371	77.04	6e-150	lacy:A4V08_18560	K00065	kduD	2-deoxy-D-gluconate 3-dehydrogenase	1.1.1.125	Metabolism--Carbohydrate metabolism--Pentose and glucuronate interconversions

[denovogenes]_108373	99.61	0.0	stb:SGPB_0233	K07260	vanY	D-alanyl-D-alanine carboxypeptidase	3.4.16.4	Metabolism--Glycan biosynthesis and metabolism--Peptidoglycan biosynthesis|Human Diseases--Drug resistance: Antimicrobial--Vancomycin resistance|Environmental Information Processing--Signal transduction--Two-component system

[denovogenes]_108382	48.52	1e-68	cdg:CDBI1_07335	K07043	K07043	uncharacterized protein	--	--

[denovogenes]_108383	53.31	3e-89	pdf:CD630DERM_12770	K06976	K06976	uncharacterized protein	--	--

[denovogenes]_108384	94.94	4e-150	cama:F384_23000	K07308	dmsC	anaerobic dimethyl sulfoxide reductase subunit C (DMSO reductase anchor subunit)	--	Metabolism--Energy metabolism--Sulfur metabolism

[denovogenes]_108385	100.00	0.0	pvl:AOB99_00535	K00930	argB	acetylglutamate kinase	2.7.2.8	Metabolism--Amino acid metabolism--Arginine biosynthesis|Metabolism--Global and overview maps--Metabolic pathways|Metabolism--Global and overview maps--Biosynthesis of secondary metabolites|Metabolism--Global and overview maps--Biosynthesis of antibiotics|Metabolism--Global and overview maps--2-Oxocarboxylic acid metabolism|Metabolism--Global and overview maps--Biosynthesis of amino acids

[denovogenes]_108386	99.61	0.0	stb:SGPB_1188	K05847	opuA	osmoprotectant transport system ATP-binding protein	--	Environmental Information Processing--Membrane transport--ABC transporters

[denovogenes]_108387	53.70	9e-29	cbk:CLL_A0924	K07741	antB	anti-repressor protein	--	--

[denovogenes]_108388	100.00	0.0	efq:DR75_1356	K01922	PPCS, coaB	phosphopantothenate-cysteine ligase	6.3.2.5	Metabolism--Metabolism of cofactors and vitamins--Pantothenate and CoA biosynthesis|Metabolism--Global and overview maps--Metabolic pathways

[denovogenes]_108389	80.40	2e-154	cay:CEA_G0227	K01142	E3.1.11.2, xthA	exodeoxyribonuclease III	3.1.11.2	Genetic Information Processing--Replication and repair--Base excision repair

[denovogenes]_108390	99.61	0.0	cif:AL515_10945	K09773	ppsR	[pyruvate, water dikinase]-phosphate phosphotransferase / [pyruvate, water dikinase] kinase	2.7.4.28 2.7.11.33	--

[denovogenes]_108391	100.00	0.0	pvl:AOB99_14665	K03228	yscT, sctT, hrcT	type III secretion protein T	--	Environmental Information Processing--Membrane transport--Bacterial secretion system

[denovogenes]_108392	100.00	0.0	elo:EC042_1601	K16076	nmpC, ompD	outer membrane porin protein LC	--	--

[denovogenes]_108394	40.85	2e-53	puf:UFO1_1710	K06905	K06905	uncharacterized protein	--	--

[denovogenes]_108395	100.00	0.0	cbut:ATN24_18895	K04069	pflA, pflC, pflE	pyruvate formate lyase activating enzyme	1.97.1.4	--

[denovogenes]_108396	43.24	6e-34	plu:plu2266	K01066	aes	acetyl esterase	3.1.1.-	--

[denovogenes]_108397	99.22	0.0	eun:UMNK88_pEnt53	K12057	traF	conjugal transfer pilus assembly protein TraF	--	--

[denovogenes]_108400	60.87	9e-111	cbv:U729_944	K13292	lgt, umpA	phosphatidylglycerol:prolipoprotein diacylglycerol transferase	2.-.-.-	--

[denovogenes]_108402	100.00	0.0	ag:AAC36915	K00561	ermC, ermA	23S rRNA (adenine-N6)-dimethyltransferase	2.1.1.184	--

[denovogenes]_108403	62.40	5e-118	cpr:CPR_1552	K07335	bmpA, bmpB, tmpC	basic membrane protein A and related proteins	--	--

[denovogenes]_108405	78.35	1e-140	vpr:Vpar_1749	K01939	purA, ADSS	adenylosuccinate synthase	6.3.4.4	Metabolism--Nucleotide metabolism--Purine metabolism|Metabolism--Amino acid metabolism--Alanine, aspartate and glutamate metabolism|Metabolism--Global and overview maps--Metabolic pathways

[denovogenes]_108407	98.83	0.0	cif:AL515_04835	K09861	K09861	uncharacterized protein	--	--

[denovogenes]_108408	82.56	8e-163	csq:CSCA_2109	K00170	porB	pyruvate ferredoxin oxidoreductase beta subunit	1.2.7.1	Metabolism--Carbohydrate metabolism--Glycolysis / Gluconeogenesis|Metabolism--Carbohydrate metabolism--Citrate cycle (TCA cycle)|Metabolism--Carbohydrate metabolism--Pyruvate metabolism|Metabolism--Xenobiotics biodegradation and metabolism--Nitrotoluene degradation|Metabolism--Carbohydrate metabolism--Propanoate metabolism|Metabolism--Carbohydrate metabolism--Butanoate metabolism|Metabolism--Energy metabolism--Methane metabolism|Metabolism--Energy metabolism--Carbon fixation pathways in prokaryotes|Metabolism--Global and overview maps--Metabolic pathways|Metabolism--Global and overview maps--Microbial metabolism in diverse environments|Metabolism--Global and overview maps--Biosynthesis of antibiotics|Metabolism--Global and overview maps--Carbon metabolism

[denovogenes]_108409	60.23	9e-108	lmz:LMOSLCC2482_2057	K19508	PTS-Gfr-EIIC, gfrC	PTS system, fructoselysine/glucoselysine-specific IIC component	--	Environmental Information Processing--Membrane transport--Phosphotransferase system (PTS)

[denovogenes]_108411	48.64	5e-80	cdg:CDBI1_01445	K02392	flgG	flagellar basal-body rod protein FlgG	--	Cellular Processes--Cell motility--Flagellar assembly

[denovogenes]_108413	79.07	2e-150	cbk:CLL_A0897	K18707	mtaB	threonylcarbamoyladenosine tRNA methylthiotransferase MtaB	2.8.4.5	--

[denovogenes]_108415	84.05	2e-159	fnc:HMPREF0946_00125	K00677	lpxA	UDP-N-acetylglucosamine acyltransferase	2.3.1.129	Metabolism--Glycan biosynthesis and metabolism--Lipopolysaccharide biosynthesis|Metabolism--Global and overview maps--Metabolic pathways|Human Diseases--Drug resistance: Antimicrobial--Cationic antimicrobial peptide (CAMP) resistance

[denovogenes]_108417	99.22	0.0	ecoh:ECRM13516_0309	K03417	prpB	methylisocitrate lyase	4.1.3.30	Metabolism--Carbohydrate metabolism--Propanoate metabolism

[denovogenes]_108418	63.18	4e-123	ova:OBV_41780	K02334	dpo	DNA polymerase bacteriophage-type	2.7.7.7	--

[denovogenes]_108419	80.08	3e-147	pdf:CD630DERM_36720	K03496	parA, soj	chromosome partitioning protein	--	--

[denovogenes]_108420	99.61	0.0	cfd:CFNIH1_17460	K00324	pntA	NAD(P) transhydrogenase subunit alpha	1.6.1.2	Metabolism--Metabolism of cofactors and vitamins--Nicotinate and nicotinamide metabolism|Metabolism--Global and overview maps--Metabolic pathways

[denovogenes]_108421	99.22	0.0	prg:RB151_037560	K06958	rapZ	RNase adapter protein RapZ	--	--

[denovogenes]_108422	91.05	1e-174	cfd:CFNIH1_15975	K13069	E2.7.7.65	diguanylate cyclase	2.7.7.65	--

[denovogenes]_108424	61.87	5e-110	cbv:U729_1178	K01776	murI	glutamate racemase	5.1.1.3	Metabolism--Metabolism of other amino acids--D-Glutamine and D-glutamate metabolism|Metabolism--Global and overview maps--Metabolic pathways

[denovogenes]_108425	42.86	1e-69	cst:CLOST_2251	K02034	ABC.PE.P1	peptide/nickel transport system permease protein	--	Cellular Processes--Cellular community - prokaryotes--Quorum sensing

[denovogenes]_108426	60.76	4e-107	aoe:Clos_0304	K15555	ssuB	sulfonate transport system ATP-binding protein	3.6.3.-	Metabolism--Energy metabolism--Sulfur metabolism|Environmental Information Processing--Membrane transport--ABC transporters

[denovogenes]_108428	84.77	6e-162	bcel:BcellWH2_02108	K00286	proC	pyrroline-5-carboxylate reductase	1.5.1.2	Metabolism--Amino acid metabolism--Arginine and proline metabolism|Metabolism--Global and overview maps--Metabolic pathways|Metabolism--Global and overview maps--Biosynthesis of secondary metabolites|Metabolism--Global and overview maps--Biosynthesis of antibiotics|Metabolism--Global and overview maps--Biosynthesis of amino acids

[denovogenes]_108431	72.76	8e-136	cle:Clole_3629	K01791	wecB	UDP-N-acetylglucosamine 2-epimerase (non-hydrolysing)	5.1.3.14	Metabolism--Carbohydrate metabolism--Amino sugar and nucleotide sugar metabolism|Metabolism--Global and overview maps--Metabolic pathways|Cellular Processes--Cellular community - prokaryotes--Biofilm formation - Vibrio cholerae

[denovogenes]_108432	60.31	1e-107	sxo:SXYL_00257	K02761	PTS-Cel-EIIC, celB, chbC	PTS system, cellobiose-specific IIC component	--	Metabolism--Carbohydrate metabolism--Starch and sucrose metabolism|Environmental Information Processing--Membrane transport--Phosphotransferase system (PTS)

[denovogenes]_108435	95.65	3e-157	cpr:CPR_0805	K02795	PTS-Man-EIIC, manY	PTS system, mannose-specific IIC component	--	Metabolism--Carbohydrate metabolism--Fructose and mannose metabolism|Metabolism--Carbohydrate metabolism--Amino sugar and nucleotide sugar metabolism|Metabolism--Global and overview maps--Metabolic pathways|Environmental Information Processing--Membrane transport--Phosphotransferase system (PTS)

[denovogenes]_108436	72.43	2e-130	dai:Desaci_0247	K01647	CS, gltA	citrate synthase	2.3.3.1	Metabolism--Carbohydrate metabolism--Citrate cycle (TCA cycle)|Metabolism--Carbohydrate metabolism--Glyoxylate and dicarboxylate metabolism|Metabolism--Global and overview maps--Metabolic pathways|Metabolism--Global and overview maps--Biosynthesis of secondary metabolites|Metabolism--Global and overview maps--Microbial metabolism in diverse environments|Metabolism--Global and overview maps--Biosynthesis of antibiotics|Metabolism--Global and overview maps--Carbon metabolism|Metabolism--Global and overview maps--2-Oxocarboxylic acid metabolism|Metabolism--Global and overview maps--Biosynthesis of amino acids

[denovogenes]_108437	97.28	0.0	cif:AL515_20535	K16371	gatZ-kbaZ	D-tagatose-1,6-bisphosphate aldolase subunit GatZ/KbaZ	--	Metabolism--Carbohydrate metabolism--Galactose metabolism|Metabolism--Global and overview maps--Metabolic pathways

[denovogenes]_108438	65.12	6e-119	cso:CLS_20790	K01939	purA, ADSS	adenylosuccinate synthase	6.3.4.4	Metabolism--Nucleotide metabolism--Purine metabolism|Metabolism--Amino acid metabolism--Alanine, aspartate and glutamate metabolism|Metabolism--Global and overview maps--Metabolic pathways

[denovogenes]_108439	51.94	6e-80	cpy:Cphy_3492	K08974	K08974	putative membrane protein	--	--

[denovogenes]_10844	61.91	0.0	dpg:DESPIGER_0952	K02224	cobB-cbiA	cobyrinic acid a,c-diamide synthase	6.3.5.9 6.3.5.11	Metabolism--Metabolism of cofactors and vitamins--Porphyrin and chlorophyll metabolism|Metabolism--Global and overview maps--Metabolic pathways

[denovogenes]_108440	75.88	6e-140	csr:Cspa_c12730	K00057	gpsA	glycerol-3-phosphate dehydrogenase (NAD(P)+)	1.1.1.94	Metabolism--Lipid metabolism--Glycerophospholipid metabolism|Metabolism--Global and overview maps--Biosynthesis of secondary metabolites

[denovogenes]_108442	74.03	5e-140	cbv:U729_73	K15986	ppaC	manganese-dependent inorganic pyrophosphatase	3.6.1.1	Metabolism--Energy metabolism--Oxidative phosphorylation

[denovogenes]_108443	97.28	0.0	sdz:Asd1617_03791	K15460	yfiC, trmX	tRNA1Val (adenine37-N6)-methyltransferase	2.1.1.223	--

[denovogenes]_108444	69.17	4e-122	vpr:Vpar_0568	K17828	pyrDI	dihydroorotate dehydrogenase (NAD+) catalytic subunit	1.3.1.14	Metabolism--Nucleotide metabolism--Pyrimidine metabolism|Metabolism--Global and overview maps--Metabolic pathways

[denovogenes]_108446	76.80	1e-144	cbv:U729_2825	K00925	ackA	acetate kinase	2.7.2.1	Metabolism--Metabolism of other amino acids--Taurine and hypotaurine metabolism|Metabolism--Carbohydrate metabolism--Pyruvate metabolism|Metabolism--Carbohydrate metabolism--Propanoate metabolism|Metabolism--Energy metabolism--Methane metabolism|Metabolism--Energy metabolism--Carbon fixation pathways in prokaryotes|Metabolism--Global and overview maps--Metabolic pathways|Metabolism--Global and overview maps--Microbial metabolism in diverse environments|Metabolism--Global and overview maps--Carbon metabolism

[denovogenes]_108448	67.32	8e-132	cpf:CPF_1229	K04486	E3.1.3.15B	histidinol-phosphatase (PHP family)	3.1.3.15	Metabolism--Amino acid metabolism--Histidine metabolism|Metabolism--Global and overview maps--Metabolic pathways|Metabolism--Global and overview maps--Biosynthesis of secondary metabolites|Metabolism--Global and overview maps--Biosynthesis of amino acids

[denovogenes]_10845	40.81	3e-110	bgy:BGLY_3391	K07456	mutS2	DNA mismatch repair protein MutS2	--	Genetic Information Processing--Replication and repair--Mismatch repair

[denovogenes]_108452	74.43	1e-136	cbz:Cbs_4838	K02755	PTS-Bgl-EIIA, bglF, bglP	PTS system, beta-glucoside-specific IIA component	2.7.1.-	Environmental Information Processing--Membrane transport--Phosphotransferase system (PTS)

[denovogenes]_108452	74.43	1e-136	cbz:Cbs_4838	K02756	PTS-Bgl-EIIB, bglF, bglP	PTS system, beta-glucoside-specific IIB component	2.7.1.-	Environmental Information Processing--Membrane transport--Phosphotransferase system (PTS)

[denovogenes]_108452	74.43	1e-136	cbz:Cbs_4838	K02757	PTS-Bgl-EIIC, bglF, bglP	PTS system, beta-glucoside-specific IIC component	--	Environmental Information Processing--Membrane transport--Phosphotransferase system (PTS)

[denovogenes]_108453	54.33	5e-95	lacy:A4V08_05455	K06408	spoVAF	stage V sporulation protein AF	--	--

[denovogenes]_108454	73.75	4e-145	csb:CLSA_c13730	K06949	rsgA, engC	ribosome biogenesis GTPase / thiamine phosphate phosphatase	3.6.1.- 3.1.3.100	Metabolism--Metabolism of cofactors and vitamins--Thiamine metabolism|Metabolism--Global and overview maps--Metabolic pathways

[denovogenes]_108455	87.21	5e-171	vpr:Vpar_1614	K14170	pheA	chorismate mutase / prephenate dehydratase	5.4.99.5 4.2.1.51	Metabolism--Amino acid metabolism--Phenylalanine, tyrosine and tryptophan biosynthesis|Metabolism--Global and overview maps--Metabolic pathways|Metabolism--Global and overview maps--Biosynthesis of secondary metabolites|Metabolism--Global and overview maps--Biosynthesis of antibiotics|Metabolism--Global and overview maps--Biosynthesis of amino acids

[denovogenes]_108457	91.09	7e-172	cbz:Cbs_0598	K00927	PGK, pgk	phosphoglycerate kinase	2.7.2.3	Metabolism--Carbohydrate metabolism--Glycolysis / Gluconeogenesis|Metabolism--Energy metabolism--Carbon fixation in photosynthetic organisms|Metabolism--Global and overview maps--Metabolic pathways|Metabolism--Global and overview maps--Biosynthesis of secondary metabolites|Metabolism--Global and overview maps--Microbial metabolism in diverse environments|Metabolism--Global and overview maps--Biosynthesis of antibiotics|Metabolism--Global and overview maps--Carbon metabolism|Metabolism--Global and overview maps--Biosynthesis of amino acids

[denovogenes]_108458	88.76	1e-173	cbut:ATN24_18875	K01198	xynB	xylan 1,4-beta-xylosidase	3.2.1.37	Metabolism--Carbohydrate metabolism--Amino sugar and nucleotide sugar metabolism|Metabolism--Global and overview maps--Metabolic pathways

[denovogenes]_108459	80.23	2e-140	cbi:CLJ_B1746	K05845	opuC	osmoprotectant transport system substrate-binding protein	--	Environmental Information Processing--Membrane transport--ABC transporters

[denovogenes]_108459	80.23	2e-140	cbi:CLJ_B1746	K05846	opuBD	osmoprotectant transport system permease protein	--	Environmental Information Processing--Membrane transport--ABC transporters

[denovogenes]_108462	72.76	4e-133	ecv:APECO1_25	K02238	comEC	competence protein ComEC	--	--

[denovogenes]_108464	47.94	8e-73	cpae:CPAST_c17690	K02396	flgK	flagellar hook-associated protein 1 FlgK	--	Cellular Processes--Cell motility--Flagellar assembly

[denovogenes]_108465	100.00	0.0	cbut:ATN24_11480	K02529	lacI, galR	LacI family transcriptional regulator	--	--

[denovogenes]_108466	85.27	4e-165	pdf:CD630DERM_26510	K02563	murG	UDP-N-acetylglucosamine--N-acetylmuramyl-(pentapeptide) pyrophosphoryl-undecaprenol N-acetylglucosamine transferase	2.4.1.227	Metabolism--Glycan biosynthesis and metabolism--Peptidoglycan biosynthesis|Metabolism--Global and overview maps--Metabolic pathways|Human Diseases--Drug resistance: Antimicrobial--Vancomycin resistance|Cellular Processes--Cell growth and death--Cell cycle - Caulobacter

[denovogenes]_108467	100.00	0.0	pvl:AOB99_03010	K06282	hyaA, hybO	hydrogenase small subunit	1.12.99.6	Metabolism--Xenobiotics biodegradation and metabolism--Nitrotoluene degradation|Metabolism--Global and overview maps--Microbial metabolism in diverse environments

[denovogenes]_108469	99.22	0.0	ecol:LY180_16385	K01940	argG, ASS1	argininosuccinate synthase	6.3.4.5	Metabolism--Amino acid metabolism--Arginine biosynthesis|Metabolism--Amino acid metabolism--Alanine, aspartate and glutamate metabolism|Metabolism--Global and overview maps--Metabolic pathways|Metabolism--Global and overview maps--Biosynthesis of secondary metabolites|Metabolism--Global and overview maps--Biosynthesis of antibiotics|Metabolism--Global and overview maps--Biosynthesis of amino acids|Human Diseases--Cardiovascular diseases--Fluid shear stress and atherosclerosis

[denovogenes]_108470	69.77	1e-131	hsd:SD1D_1424	K02000	proV	glycine betaine/proline transport system ATP-binding protein	3.6.3.32	Environmental Information Processing--Membrane transport--ABC transporters

[denovogenes]_108471	69.26	3e-126	cbt:CLH_0440	K03592	pmbA	PmbA protein	--	--

[denovogenes]_108473	98.45	0.0	pvl:AOB99_14370	K01744	aspA	aspartate ammonia-lyase	4.3.1.1	Metabolism--Amino acid metabolism--Alanine, aspartate and glutamate metabolism|Metabolism--Global and overview maps--Metabolic pathways

[denovogenes]_108474	82.95	1e-152	elp:P12B_c3996	K18478	yihV	sulfofructose kinase	2.7.1.184	--

[denovogenes]_108475	59.43	2e-88	mcl:MCCL_1675	K01401	aur	aureolysin	3.4.24.29	Human Diseases--Drug resistance: Antimicrobial--Cationic antimicrobial peptide (CAMP) resistance|Human Diseases--Infectious diseases: Bacterial--Staphylococcus aureus infection

[denovogenes]_108477	100.00	0.0	pvl:AOB99_05630	K03593	mrp, NUBPL	ATP-binding protein involved in chromosome partitioning	--	--

[denovogenes]_10848	99.71	0.0	cfd:CFNIH1_20625	K03407	cheA	two-component system, chemotaxis family, sensor kinase CheA	2.7.13.3	Environmental Information Processing--Signal transduction--Two-component system|Cellular Processes--Cell motility--Bacterial chemotaxis

[denovogenes]_108480	40.08	4e-37	twi:Thewi_0505	K03406	mcp	methyl-accepting chemotaxis protein	--	Environmental Information Processing--Signal transduction--Two-component system|Cellular Processes--Cell motility--Bacterial chemotaxis

[denovogenes]_108482	63.89	2e-111	puf:UFO1_2586	K03706	codY	transcriptional pleiotropic repressor	--	--

[denovogenes]_108483	82.17	1e-153	rto:RTO_05910	K02823	pyrDII	dihydroorotate dehydrogenase electron transfer subunit	--	Metabolism--Nucleotide metabolism--Pyrimidine metabolism|Metabolism--Global and overview maps--Metabolic pathways

[denovogenes]_108486	56.92	2e-89	rob:CK5_21980	K13292	lgt, umpA	phosphatidylglycerol:prolipoprotein diacylglycerol transferase	2.-.-.-	--

[denovogenes]_108488	67.97	2e-129	hsd:SD1D_0177	K09697	natA	sodium transport system ATP-binding protein	3.6.3.7	Environmental Information Processing--Membrane transport--ABC transporters|Environmental Information Processing--Signal transduction--Two-component system

[denovogenes]_108489	96.50	0.0	bth:BT_3933	K00210	E1.3.1.12	prephenate dehydrogenase	1.3.1.12	Metabolism--Amino acid metabolism--Phenylalanine, tyrosine and tryptophan biosynthesis|Metabolism--Biosynthesis of other secondary metabolites--Novobiocin biosynthesis|Metabolism--Global and overview maps--Metabolic pathways|Metabolism--Global and overview maps--Biosynthesis of secondary metabolites|Metabolism--Global and overview maps--Biosynthesis of antibiotics|Metabolism--Global and overview maps--Biosynthesis of amino acids

[denovogenes]_108490	98.44	0.0	bth:BT_3933	K00210	E1.3.1.12	prephenate dehydrogenase	1.3.1.12	Metabolism--Amino acid metabolism--Phenylalanine, tyrosine and tryptophan biosynthesis|Metabolism--Biosynthesis of other secondary metabolites--Novobiocin biosynthesis|Metabolism--Global and overview maps--Metabolic pathways|Metabolism--Global and overview maps--Biosynthesis of secondary metabolites|Metabolism--Global and overview maps--Biosynthesis of antibiotics|Metabolism--Global and overview maps--Biosynthesis of amino acids

[denovogenes]_108491	56.08	6e-98	ain:Acin_1025	K02823	pyrDII	dihydroorotate dehydrogenase electron transfer subunit	--	Metabolism--Nucleotide metabolism--Pyrimidine metabolism|Metabolism--Global and overview maps--Metabolic pathways

[denovogenes]_108495	69.41	8e-132	fpla:A4U99_00915	K03424	--	--	--	--

[denovogenes]_108498	40.18	3e-34	fla:SY85_20955	K02477	K02477	two-component system, LytT family, response regulator	--	--

[denovogenes]_108499	90.98	6e-159	fpla:A4U99_03970	K02003	ABC.CD.A	putative ABC transport system ATP-binding protein	--	--

[denovogenes]_10850	100.00	0.0	elf:LF82_2234	K00656	E2.3.1.54, pflD	formate C-acetyltransferase	2.3.1.54	Metabolism--Carbohydrate metabolism--Pyruvate metabolism|Metabolism--Carbohydrate metabolism--Propanoate metabolism|Metabolism--Carbohydrate metabolism--Butanoate metabolism|Metabolism--Global and overview maps--Metabolic pathways

[denovogenes]_108500	80.93	5e-157	bhl:Bache_1210	K03524	birA	BirA family transcriptional regulator, biotin operon repressor / biotin-[acetyl-CoA-carboxylase] ligase	6.3.4.15	Metabolism--Metabolism of cofactors and vitamins--Biotin metabolism|Metabolism--Global and overview maps--Metabolic pathways

[denovogenes]_108505	71.02	4e-124	cct:CC1_24310	K00059	fabG	3-oxoacyl-[acyl-carrier protein] reductase	1.1.1.100	Metabolism--Lipid metabolism--Fatty acid biosynthesis|Metabolism--Metabolism of cofactors and vitamins--Biotin metabolism|Metabolism--Lipid metabolism--Biosynthesis of unsaturated fatty acids|Metabolism--Global and overview maps--Metabolic pathways|Metabolism--Global and overview maps--Fatty acid metabolism

[denovogenes]_108507	99.61	0.0	bvu:BVU_3542	K03149	thiG	thiazole synthase	2.8.1.10	Metabolism--Metabolism of cofactors and vitamins--Thiamine metabolism|Metabolism--Global and overview maps--Metabolic pathways

[denovogenes]_108508	82.49	3e-163	cso:CLS_38620	K05833	ABC.X4.A	putative ABC transport system ATP-binding protein	--	--

[denovogenes]_108509	51.83	2e-73	pary:A4V02_08670	K10716	kch, trkA, mthK, pch	voltage-gated potassium channel	--	--

[denovogenes]_108510	40.68	1e-12	csr:Cspa_c55380	K13018	wbpD, wlbB	UDP-2-acetamido-3-amino-2,3-dideoxy-glucuronate N-acetyltransferase	2.3.1.201	Metabolism--Carbohydrate metabolism--Amino sugar and nucleotide sugar metabolism

[denovogenes]_108514	70.43	3e-136	rto:RTO_23980	K05833	ABC.X4.A	putative ABC transport system ATP-binding protein	--	--

[denovogenes]_108520	100.00	0.0	bvu:BVU_0589	K00286	proC	pyrroline-5-carboxylate reductase	1.5.1.2	Metabolism--Amino acid metabolism--Arginine and proline metabolism|Metabolism--Global and overview maps--Metabolic pathways|Metabolism--Global and overview maps--Biosynthesis of secondary metabolites|Metabolism--Global and overview maps--Biosynthesis of antibiotics|Metabolism--Global and overview maps--Biosynthesis of amino acids

[denovogenes]_108522	56.86	1e-104	rus:RBI_I01490	K03525	coaX	type III pantothenate kinase	2.7.1.33	Metabolism--Metabolism of cofactors and vitamins--Pantothenate and CoA biosynthesis|Metabolism--Global and overview maps--Metabolic pathways

[denovogenes]_108523	56.18	1e-95	ava:Ava_2888	K05886	sdh	serine 3-dehydrogenase (NADP+)	1.1.1.276	--

[denovogenes]_108524	63.92	6e-117	byl:A4V09_19035	K03525	coaX	type III pantothenate kinase	2.7.1.33	Metabolism--Metabolism of cofactors and vitamins--Pantothenate and CoA biosynthesis|Metabolism--Global and overview maps--Metabolic pathways

[denovogenes]_108525	59.62	2e-109	rto:RTO_30380	K03437	spoU	RNA methyltransferase, TrmH family	--	--

[denovogenes]_108526	66.37	1e-109	paeq:R50912_17245	K01607	pcaC	4-carboxymuconolactone decarboxylase	4.1.1.44	Metabolism--Xenobiotics biodegradation and metabolism--Benzoate degradation|Metabolism--Global and overview maps--Metabolic pathways|Metabolism--Global and overview maps--Microbial metabolism in diverse environments|Metabolism--Global and overview maps--Degradation of aromatic compounds

[denovogenes]_108527	78.43	2e-146	dor:Desor_2389	K02003	ABC.CD.A	putative ABC transport system ATP-binding protein	--	--

[denovogenes]_108528	68.77	1e-132	cso:CLS_31830	K03424	--	--	--	--

[denovogenes]_108533	78.76	3e-155	bxy:BXY_21000	K08641	vanX	D-alanyl-D-alanine dipeptidase	3.4.13.22	Human Diseases--Drug resistance: Antimicrobial--Vancomycin resistance|Environmental Information Processing--Signal transduction--Two-component system

[denovogenes]_108534	99.61	0.0	bvu:BVU_2546	K06173	truA, PUS1	tRNA pseudouridine38-40 synthase	5.4.99.12	--

[denovogenes]_108535	51.33	6e-72	lsp:Bsph_4621	K11631	bceA, vraD	bacitracin transport system ATP-binding protein	--	Environmental Information Processing--Membrane transport--ABC transporters|Environmental Information Processing--Signal transduction--Two-component system

[denovogenes]_108536	85.99	3e-157	boa:Bovatus_02686	K07052	K07052	uncharacterized protein	--	--

[denovogenes]_108537	86.29	4e-165	csh:Closa_0365	K02036	pstB	phosphate transport system ATP-binding protein	3.6.3.27	Environmental Information Processing--Membrane transport--ABC transporters

[denovogenes]_108539	100.00	0.0	bxy:BXY_48080	K00286	proC	pyrroline-5-carboxylate reductase	1.5.1.2	Metabolism--Amino acid metabolism--Arginine and proline metabolism|Metabolism--Global and overview maps--Metabolic pathways|Metabolism--Global and overview maps--Biosynthesis of secondary metabolites|Metabolism--Global and overview maps--Biosynthesis of antibiotics|Metabolism--Global and overview maps--Biosynthesis of amino acids

[denovogenes]_108540	92.49	0.0	btho:Btheta7330_02144	K01142	E3.1.11.2, xthA	exodeoxyribonuclease III	3.1.11.2	Genetic Information Processing--Replication and repair--Base excision repair

[denovogenes]_108541	89.06	7e-173	btho:Btheta7330_00455	K09861	K09861	uncharacterized protein	--	--

[denovogenes]_108542	89.76	6e-171	ibu:IB211_01809c	K00757	udp, UPP	uridine phosphorylase	2.4.2.3	Metabolism--Nucleotide metabolism--Pyrimidine metabolism|Metabolism--Xenobiotics biodegradation and metabolism--Drug metabolism - other enzymes|Metabolism--Global and overview maps--Metabolic pathways

[denovogenes]_108544	56.57	2e-93	ova:OBV_20950	K06173	truA, PUS1	tRNA pseudouridine38-40 synthase	5.4.99.12	--

[denovogenes]_108545	56.35	5e-95	rch:RUM_07980	K03424	--	--	--	--

[denovogenes]_108546	69.53	4e-128	edu:LIU_04340	K02041	phnC	phosphonate transport system ATP-binding protein	3.6.3.28	Environmental Information Processing--Membrane transport--ABC transporters

[denovogenes]_108547	100.00	0.0	bvu:BVU_1916	K03787	surE	5'-nucleotidase	3.1.3.5	Metabolism--Nucleotide metabolism--Purine metabolism|Metabolism--Nucleotide metabolism--Pyrimidine metabolism|Metabolism--Metabolism of cofactors and vitamins--Nicotinate and nicotinamide metabolism|Metabolism--Global and overview maps--Metabolic pathways|Metabolism--Global and overview maps--Biosynthesis of secondary metabolites

[denovogenes]_108548	74.80	3e-137	bip:Bint_1415	K15555	ssuB	sulfonate transport system ATP-binding protein	3.6.3.-	Metabolism--Energy metabolism--Sulfur metabolism|Environmental Information Processing--Membrane transport--ABC transporters

[denovogenes]_108550	52.67	2e-91	csh:Closa_0133	K12510	tadB	tight adherence protein B	--	--

[denovogenes]_108551	99.22	0.0	bvu:BVU_1338	K00930	argB	acetylglutamate kinase	2.7.2.8	Metabolism--Amino acid metabolism--Arginine biosynthesis|Metabolism--Global and overview maps--Metabolic pathways|Metabolism--Global and overview maps--Biosynthesis of secondary metabolites|Metabolism--Global and overview maps--Biosynthesis of antibiotics|Metabolism--Global and overview maps--2-Oxocarboxylic acid metabolism|Metabolism--Global and overview maps--Biosynthesis of amino acids

[denovogenes]_108552	99.61	0.0	sbc:SbBS512_E2684	K10017	hisP	histidine transport system ATP-binding protein	3.6.3.21	Environmental Information Processing--Membrane transport--ABC transporters

[denovogenes]_108553	53.23	6e-94	cpro:CPRO_28380	K02405	fliA	RNA polymerase sigma factor for flagellar operon FliA	--	Environmental Information Processing--Signal transduction--Two-component system|Cellular Processes--Cellular community - prokaryotes--Biofilm formation - Pseudomonas aeruginosa|Cellular Processes--Cellular community - prokaryotes--Biofilm formation - Escherichia coli|Cellular Processes--Cell motility--Flagellar assembly|Cellular Processes--Cellular community - prokaryotes--Biofilm formation - Vibrio cholerae

[denovogenes]_108557	42.28	1e-19	blq:L21SP5_03403	K02477	K02477	two-component system, LytT family, response regulator	--	--

[denovogenes]_108558	76.19	2e-133	fpla:A4U99_07355	K07090	K07090	uncharacterized protein	--	--

[denovogenes]_108559	97.58	2e-170	boa:Bovatus_02150	K02233	E2.7.8.26, cobS, cobV	adenosylcobinamide-GDP ribazoletransferase	2.7.8.26	Metabolism--Metabolism of cofactors and vitamins--Porphyrin and chlorophyll metabolism|Metabolism--Global and overview maps--Metabolic pathways

[denovogenes]_108560	43.07	9e-47	bao:BAMF_3288	K00903	epsB	protein-tyrosine kinase	2.7.10.2	--

[denovogenes]_108561	48.00	3e-85	cbk:CLL_A3479	K07090	K07090	uncharacterized protein	--	--

[denovogenes]_108562	99.22	0.0	eun:UMNK88_4409	K07270	K07270	glycosyl transferase, family 25	--	--

[denovogenes]_108563	94.55	4e-178	bxy:BXY_48080	K00286	proC	pyrroline-5-carboxylate reductase	1.5.1.2	Metabolism--Amino acid metabolism--Arginine and proline metabolism|Metabolism--Global and overview maps--Metabolic pathways|Metabolism--Global and overview maps--Biosynthesis of secondary metabolites|Metabolism--Global and overview maps--Biosynthesis of antibiotics|Metabolism--Global and overview maps--Biosynthesis of amino acids

[denovogenes]_108566	75.42	9e-131	fpla:A4U99_08410	K01923	purC	phosphoribosylaminoimidazole-succinocarboxamide synthase	6.3.2.6	Metabolism--Nucleotide metabolism--Purine metabolism|Metabolism--Global and overview maps--Metabolic pathways|Metabolism--Global and overview maps--Biosynthesis of secondary metabolites|Metabolism--Global and overview maps--Biosynthesis of antibiotics

[denovogenes]_108567	60.00	5e-112	abao:BEQ56_08575	K11645	fbaB	fructose-bisphosphate aldolase, class I	4.1.2.13	Metabolism--Carbohydrate metabolism--Glycolysis / Gluconeogenesis|Metabolism--Carbohydrate metabolism--Pentose phosphate pathway|Metabolism--Carbohydrate metabolism--Fructose and mannose metabolism|Metabolism--Energy metabolism--Methane metabolism|Metabolism--Energy metabolism--Carbon fixation in photosynthetic organisms|Metabolism--Global and overview maps--Metabolic pathways|Metabolism--Global and overview maps--Biosynthesis of secondary metabolites|Metabolism--Global and overview maps--Microbial metabolism in diverse environments|Metabolism--Global and overview maps--Biosynthesis of antibiotics|Metabolism--Global and overview maps--Carbon metabolism|Metabolism--Global and overview maps--Biosynthesis of amino acids

[denovogenes]_108568	46.79	3e-53	cpy:Cphy_2517	K06397	spoIIIAH	stage III sporulation protein AH	--	--

[denovogenes]_108569	48.85	7e-76	rch:RUM_18740	K02823	pyrDII	dihydroorotate dehydrogenase electron transfer subunit	--	Metabolism--Nucleotide metabolism--Pyrimidine metabolism|Metabolism--Global and overview maps--Metabolic pathways

[denovogenes]_108570	73.54	2e-146	cso:CLS_38620	K05833	ABC.X4.A	putative ABC transport system ATP-binding protein	--	--

[denovogenes]_108571	40.11	1e-35	btw:BF38_5307	K07124	K07124	uncharacterized protein	--	--

[denovogenes]_108576	91.44	4e-176	lacy:A4V08_04855	K05833	ABC.X4.A	putative ABC transport system ATP-binding protein	--	--

[denovogenes]_108578	51.23	7e-82	awo:Awo_c22370	K02050	ABC.SN.P	NitT/TauT family transport system permease protein	--	--

[denovogenes]_108580	79.69	3e-147	byl:A4V09_13805	K01816	hyi, gip	hydroxypyruvate isomerase	5.3.1.22	Metabolism--Carbohydrate metabolism--Glyoxylate and dicarboxylate metabolism|Metabolism--Global and overview maps--Metabolic pathways

[denovogenes]_108583	59.76	5e-103	cce:Ccel_1227	K07718	yesM	two-component system, sensor histidine kinase YesM	2.7.13.3	Environmental Information Processing--Signal transduction--Two-component system

[denovogenes]_108584	94.19	1e-174	cpy:Cphy_0574	K10201	ABC.NGC.P	N-acetylglucosamine transport system permease protein	--	Environmental Information Processing--Membrane transport--ABC transporters

[denovogenes]_108585	70.82	2e-111	lacy:A4V08_08015	K09807	K09807	uncharacterized protein	--	--

[denovogenes]_108586	43.36	6e-20	blq:L21SP5_03403	K02477	K02477	two-component system, LytT family, response regulator	--	--

[denovogenes]_108588	66.00	1e-116	cpy:Cphy_1260	K01995	livG	branched-chain amino acid transport system ATP-binding protein	--	Environmental Information Processing--Membrane transport--ABC transporters|Cellular Processes--Cellular community - prokaryotes--Quorum sensing

[denovogenes]_108589	51.79	7e-95	boa:Bovatus_03697	K02564	nagB, GNPDA	glucosamine-6-phosphate deaminase	3.5.99.6	Metabolism--Carbohydrate metabolism--Amino sugar and nucleotide sugar metabolism|Metabolism--Global and overview maps--Metabolic pathways

[denovogenes]_10859	100.00	0.0	ecm:EcSMS35_2860	K03555	mutS	DNA mismatch repair protein MutS	--	Genetic Information Processing--Replication and repair--Mismatch repair

[denovogenes]_108590	88.67	2e-168	lacy:A4V08_14680	K01695	trpA	tryptophan synthase alpha chain	4.2.1.20	Metabolism--Amino acid metabolism--Glycine, serine and threonine metabolism|Metabolism--Amino acid metabolism--Phenylalanine, tyrosine and tryptophan biosynthesis|Metabolism--Global and overview maps--Metabolic pathways|Metabolism--Global and overview maps--Biosynthesis of secondary metabolites|Metabolism--Global and overview maps--Biosynthesis of antibiotics|Metabolism--Global and overview maps--Biosynthesis of amino acids

[denovogenes]_108591	94.55	0.0	fpla:A4U99_02045	K01803	TPI, tpiA	triosephosphate isomerase (TIM)	5.3.1.1	Metabolism--Carbohydrate metabolism--Glycolysis / Gluconeogenesis|Metabolism--Carbohydrate metabolism--Fructose and mannose metabolism|Metabolism--Carbohydrate metabolism--Inositol phosphate metabolism|Metabolism--Energy metabolism--Carbon fixation in photosynthetic organisms|Metabolism--Global and overview maps--Metabolic pathways|Metabolism--Global and overview maps--Biosynthesis of secondary metabolites|Metabolism--Global and overview maps--Microbial metabolism in diverse environments|Metabolism--Global and overview maps--Biosynthesis of antibiotics|Metabolism--Global and overview maps--Carbon metabolism|Metabolism--Global and overview maps--Biosynthesis of amino acids

[denovogenes]_108592	96.11	0.0	bvu:BVU_3542	K03149	thiG	thiazole synthase	2.8.1.10	Metabolism--Metabolism of cofactors and vitamins--Thiamine metabolism|Metabolism--Global and overview maps--Metabolic pathways

[denovogenes]_108593	98.05	0.0	lacy:A4V08_29825	K03079	ulaE, sgaU	L-ribulose-5-phosphate 3-epimerase	5.1.3.22	Metabolism--Carbohydrate metabolism--Ascorbate and aldarate metabolism|Metabolism--Global and overview maps--Metabolic pathways|Metabolism--Global and overview maps--Microbial metabolism in diverse environments

[denovogenes]_108595	99.22	0.0	boa:Bovatus_00986	K03149	thiG	thiazole synthase	2.8.1.10	Metabolism--Metabolism of cofactors and vitamins--Thiamine metabolism|Metabolism--Global and overview maps--Metabolic pathways

[denovogenes]_108599	66.93	1e-114	cso:CLS_19770	K01449	E3.5.1.28C, cwlJ, sleB	N-acetylmuramoyl-L-alanine amidase	3.5.1.28	--

[denovogenes]_1086	97.65	0.0	btho:Btheta7330_00849	K01190	lacZ	beta-galactosidase	3.2.1.23	Metabolism--Carbohydrate metabolism--Galactose metabolism|Metabolism--Glycan biosynthesis and metabolism--Other glycan degradation|Metabolism--Lipid metabolism--Sphingolipid metabolism|Metabolism--Global and overview maps--Metabolic pathways

[denovogenes]_10860	83.73	0.0	soi:I872_01195	K01712	hutU, UROC1	urocanate hydratase	4.2.1.49	Metabolism--Amino acid metabolism--Histidine metabolism|Metabolism--Global and overview maps--Metabolic pathways

[denovogenes]_108600	56.25	2e-70	cbz:Cbs_1826	K03093	SIGMA70	RNA polymerase sigma factor	--	--

[denovogenes]_108601	98.83	0.0	cif:AL515_02425	K00930	argB	acetylglutamate kinase	2.7.2.8	Metabolism--Amino acid metabolism--Arginine biosynthesis|Metabolism--Global and overview maps--Metabolic pathways|Metabolism--Global and overview maps--Biosynthesis of secondary metabolites|Metabolism--Global and overview maps--Biosynthesis of antibiotics|Metabolism--Global and overview maps--2-Oxocarboxylic acid metabolism|Metabolism--Global and overview maps--Biosynthesis of amino acids

[denovogenes]_108603	61.51	1e-107	bvs:BARVI_08885	K14379	ACP5	tartrate-resistant acid phosphatase type 5	3.1.3.2	Metabolism--Metabolism of cofactors and vitamins--Riboflavin metabolism|Metabolism--Global and overview maps--Metabolic pathways|Cellular Processes--Transport and catabolism--Lysosome|Organismal Systems--Development--Osteoclast differentiation|Human Diseases--Immune diseases--Rheumatoid arthritis

[denovogenes]_108604	98.83	0.0	cif:AL515_04390	K13637	uxuR	GntR family transcriptional regulator, uxu operon transcriptional repressor	--	--

[denovogenes]_108605	40.49	1e-40	agw:QT03_C0001G0299	K19954	adh1	alcohol dehydrogenase	1.1.1.-	--

[denovogenes]_108606	73.54	3e-143	bdo:EL88_17575	K03424	--	--	--	--

[denovogenes]_108609	100.00	0.0	shq:A0259_01570	K03426	E3.6.1.22, NUDT12, nudC	NAD+ diphosphatase	3.6.1.22	Metabolism--Metabolism of cofactors and vitamins--Nicotinate and nicotinamide metabolism|Metabolism--Global and overview maps--Metabolic pathways|Cellular Processes--Transport and catabolism--Peroxisome

[denovogenes]_10861	80.83	0.0	dno:DNO_0014	K01581	E4.1.1.17, ODC1, speC, speF	ornithine decarboxylase	4.1.1.17	Metabolism--Amino acid metabolism--Arginine and proline metabolism|Metabolism--Metabolism of other amino acids--Glutathione metabolism|Metabolism--Global and overview maps--Metabolic pathways|Metabolism--Global and overview maps--Biosynthesis of secondary metabolites|Metabolism--Global and overview maps--Biosynthesis of antibiotics

[denovogenes]_108610	99.16	1e-171	cif:AL515_19110	K01807	rpiA	ribose 5-phosphate isomerase A	5.3.1.6	Metabolism--Carbohydrate metabolism--Pentose phosphate pathway|Metabolism--Energy metabolism--Carbon fixation in photosynthetic organisms|Metabolism--Global and overview maps--Metabolic pathways|Metabolism--Global and overview maps--Biosynthesis of secondary metabolites|Metabolism--Global and overview maps--Microbial metabolism in diverse environments|Metabolism--Global and overview maps--Biosynthesis of antibiotics|Metabolism--Global and overview maps--Carbon metabolism|Metabolism--Global and overview maps--Biosynthesis of amino acids

[denovogenes]_108611	61.09	4e-113	cle:Clole_1991	K07238	TC.ZIP, zupT, ZRT3, ZIP2	zinc transporter, ZIP family	--	--

[denovogenes]_108612	89.88	2e-164	bcel:BcellWH2_02444	K00930	argB	acetylglutamate kinase	2.7.2.8	Metabolism--Amino acid metabolism--Arginine biosynthesis|Metabolism--Global and overview maps--Metabolic pathways|Metabolism--Global and overview maps--Biosynthesis of secondary metabolites|Metabolism--Global and overview maps--Biosynthesis of antibiotics|Metabolism--Global and overview maps--2-Oxocarboxylic acid metabolism|Metabolism--Global and overview maps--Biosynthesis of amino acids

[denovogenes]_108614	99.61	0.0	cif:AL515_10105	K05936	cobM, cbiF	precorrin-4/cobalt-precorrin-4 C11-methyltransferase	2.1.1.133 2.1.1.271	Metabolism--Metabolism of cofactors and vitamins--Porphyrin and chlorophyll metabolism|Metabolism--Global and overview maps--Metabolic pathways

[denovogenes]_108616	57.65	2e-103	rus:RBI_I00679	K02823	pyrDII	dihydroorotate dehydrogenase electron transfer subunit	--	Metabolism--Nucleotide metabolism--Pyrimidine metabolism|Metabolism--Global and overview maps--Metabolic pathways

[denovogenes]_108618	86.77	3e-165	lacy:A4V08_30840	K01933	purM	phosphoribosylformylglycinamidine cyclo-ligase	6.3.3.1	Metabolism--Nucleotide metabolism--Purine metabolism|Metabolism--Global and overview maps--Metabolic pathways|Metabolism--Global and overview maps--Biosynthesis of secondary metabolites|Metabolism--Global and overview maps--Biosynthesis of antibiotics

[denovogenes]_108619	72.09	4e-137	cbz:Cbs_2100	K14534	abfD	4-hydroxybutyryl-CoA dehydratase / vinylacetyl-CoA-Delta-isomerase	4.2.1.120 5.3.3.3	Metabolism--Carbohydrate metabolism--Butanoate metabolism|Metabolism--Energy metabolism--Carbon fixation pathways in prokaryotes|Metabolism--Global and overview maps--Metabolic pathways|Metabolism--Global and overview maps--Microbial metabolism in diverse environments|Metabolism--Global and overview maps--Carbon metabolism

[denovogenes]_10862	99.71	0.0	mmk:MU9_2104	K02014	TC.FEV.OM	iron complex outermembrane recepter protein	--	--

[denovogenes]_108620	45.02	2e-82	csq:CSCA_4584	K03088	SIG3.2, rpoE	RNA polymerase sigma-70 factor, ECF subfamily	--	--

[denovogenes]_108621	100.00	3e-178	sdz:Asd1617_05836	K04077	groEL, HSPD1	chaperonin GroEL	--	Genetic Information Processing--Folding, sorting and degradation--RNA degradation|Organismal Systems--Aging--Longevity regulating pathway - worm|Human Diseases--Endocrine and metabolic diseases--Type I diabetes mellitus|Human Diseases--Infectious diseases: Bacterial--Legionellosis|Human Diseases--Infectious diseases: Bacterial--Tuberculosis

[denovogenes]_108622	40.00	4e-08	paen:P40081_35055	K06386	spoIIQ	stage II sporulation protein Q	--	--

[denovogenes]_108623	57.25	2e-91	ctet:BN906_02535	K03312	gltS	glutamate:Na+ symporter, ESS family	--	--

[denovogenes]_108624	52.16	2e-85	ccel:CCDG5_0980	K07699	spo0A	two-component system, response regulator, stage 0 sporulation protein A	--	Environmental Information Processing--Signal transduction--Two-component system|Cellular Processes--Cellular community - prokaryotes--Quorum sensing

[denovogenes]_108626	40.57	2e-47	geb:GM18_1480	K07636	phoR	two-component system, OmpR family, phosphate regulon sensor histidine kinase PhoR	2.7.13.3	Environmental Information Processing--Signal transduction--Two-component system

[denovogenes]_108627	48.41	4e-63	fpla:A4U99_12210	K02406	fliC	flagellin	--	Environmental Information Processing--Signal transduction--Two-component system|Cellular Processes--Cell motility--Flagellar assembly|Organismal Systems--Immune system--NOD-like receptor signaling pathway|Organismal Systems--Environmental adaptation--Plant-pathogen interaction|Human Diseases--Infectious diseases: Bacterial--Salmonella infection|Human Diseases--Infectious diseases: Bacterial--Legionellosis

[denovogenes]_108628	67.06	1e-136	cpro:CPRO_12460	K02566	nagD	NagD protein	--	--

[denovogenes]_10863	100.00	0.0	mmk:MU9_2961	K16089	TC.FEV.OM2, cirA, cfrA, hmuR	outer membrane receptor for ferrienterochelin and colicins	--	--

[denovogenes]_108630	98.83	0.0	cif:AL515_04835	K09861	K09861	uncharacterized protein	--	--

[denovogenes]_108631	98.83	0.0	cif:AL515_15975	K10017	hisP	histidine transport system ATP-binding protein	3.6.3.21	Environmental Information Processing--Membrane transport--ABC transporters

[denovogenes]_108632	75.62	4e-137	bcel:BcellWH2_01448	K07098	K07098	uncharacterized protein	--	--

[denovogenes]_108633	66.54	8e-123	bdh:GV66_08050	K02474	wbpO	UDP-N-acetyl-D-galactosamine dehydrogenase	1.1.1.-	Metabolism--Carbohydrate metabolism--Amino sugar and nucleotide sugar metabolism

[denovogenes]_108634	99.61	0.0	cfd:CFNIH1_00540	K02468	srlR, gutR	DeoR family transcriptional regulator, glucitol operon repressor	--	--

[denovogenes]_108636	100.00	0.0	pdi:BDI_3476	K03426	E3.6.1.22, NUDT12, nudC	NAD+ diphosphatase	3.6.1.22	Metabolism--Metabolism of cofactors and vitamins--Nicotinate and nicotinamide metabolism|Metabolism--Global and overview maps--Metabolic pathways|Cellular Processes--Transport and catabolism--Peroxisome

[denovogenes]_108637	72.48	3e-136	eha:Ethha_2070	K03106	SRP54, ffh	signal recognition particle subunit SRP54	3.6.5.4	Cellular Processes--Cellular community - prokaryotes--Quorum sensing|Genetic Information Processing--Folding, sorting and degradation--Protein export|Environmental Information Processing--Membrane transport--Bacterial secretion system

[denovogenes]_108638	69.53	5e-134	bcel:BcellWH2_05260	K01126	E3.1.4.46, glpQ, ugpQ	glycerophosphoryl diester phosphodiesterase	3.1.4.46	Metabolism--Lipid metabolism--Glycerophospholipid metabolism

[denovogenes]_108639	99.61	0.0	btho:Btheta7330_04532	K00286	proC	pyrroline-5-carboxylate reductase	1.5.1.2	Metabolism--Amino acid metabolism--Arginine and proline metabolism|Metabolism--Global and overview maps--Metabolic pathways|Metabolism--Global and overview maps--Biosynthesis of secondary metabolites|Metabolism--Global and overview maps--Biosynthesis of antibiotics|Metabolism--Global and overview maps--Biosynthesis of amino acids

[denovogenes]_10864	100.00	0.0	bfb:VU15_20875	K01874	MARS, metG	methionyl-tRNA synthetase	6.1.1.10	Metabolism--Metabolism of other amino acids--Selenocompound metabolism|Genetic Information Processing--Translation--Aminoacyl-tRNA biosynthesis

[denovogenes]_108643	51.74	3e-76	fpla:A4U99_14060	K13963	SERPINB	serpin B	--	Human Diseases--Infectious diseases: Parasitic--Amoebiasis

[denovogenes]_108644	67.44	4e-128	cbt:CLH_1233	K00928	lysC	aspartate kinase	2.7.2.4	Metabolism--Amino acid metabolism--Glycine, serine and threonine metabolism|Metabolism--Biosynthesis of other secondary metabolites--Monobactam biosynthesis|Metabolism--Amino acid metabolism--Cysteine and methionine metabolism|Metabolism--Amino acid metabolism--Lysine biosynthesis|Metabolism--Global and overview maps--Metabolic pathways|Metabolism--Global and overview maps--Biosynthesis of secondary metabolites|Metabolism--Global and overview maps--Microbial metabolism in diverse environments|Metabolism--Global and overview maps--Biosynthesis of antibiotics|Metabolism--Global and overview maps--2-Oxocarboxylic acid metabolism|Metabolism--Global and overview maps--Biosynthesis of amino acids

[denovogenes]_108645	74.42	2e-144	cck:Ccar_04540	K10670	E1.21.4.2	glycine reductase	1.21.4.2	--

[denovogenes]_108646	46.49	3e-69	pth:PTH_1783	K03588	ftsW, spoVE	cell division protein FtsW	--	Cellular Processes--Cell growth and death--Cell cycle - Caulobacter

[denovogenes]_108651	42.34	6e-51	den:MHIR_DE00707	K03581	recD	exodeoxyribonuclease V alpha subunit	3.1.11.5	Genetic Information Processing--Replication and repair--Homologous recombination

[denovogenes]_108652	99.22	0.0	cfd:CFNIH1_18035	K02028	ABC.PA.A	polar amino acid transport system ATP-binding protein	3.6.3.21	--

[denovogenes]_108654	91.83	6e-162	sot:102577536	K12492	ARFGAP1	ADP-ribosylation factor GTPase-activating protein 1	--	Cellular Processes--Transport and catabolism--Endocytosis

[denovogenes]_108658	52.04	5e-90	byl:A4V09_00535	K01992	ABC-2.P	ABC-2 type transport system permease protein	--	--

[denovogenes]_10866	71.16	0.0	pdf:CD630DERM_07160	K00198	cooS, acsA	carbon-monoxide dehydrogenase catalytic subunit	1.2.7.4	Metabolism--Xenobiotics biodegradation and metabolism--Nitrotoluene degradation|Metabolism--Energy metabolism--Methane metabolism|Metabolism--Energy metabolism--Carbon fixation pathways in prokaryotes|Metabolism--Global and overview maps--Microbial metabolism in diverse environments|Metabolism--Global and overview maps--Carbon metabolism

[denovogenes]_108660	72.48	2e-141	tae:TepiRe1_1668	K00008	SORD, gutB	L-iditol 2-dehydrogenase	1.1.1.14	Metabolism--Carbohydrate metabolism--Pentose and glucuronate interconversions|Metabolism--Carbohydrate metabolism--Fructose and mannose metabolism|Metabolism--Global and overview maps--Metabolic pathways

[denovogenes]_108661	82.49	2e-152	rto:RTO_19750	K06442	tlyA	23S rRNA (cytidine1920-2'-O)/16S rRNA (cytidine1409-2'-O)-methyltransferase	2.1.1.226 2.1.1.227	--

[denovogenes]_108662	86.31	2e-155	rum:CK1_35000	K04043	dnaK	molecular chaperone DnaK	--	Genetic Information Processing--Folding, sorting and degradation--RNA degradation|Organismal Systems--Aging--Longevity regulating pathway - worm|Human Diseases--Infectious diseases: Bacterial--Tuberculosis

[denovogenes]_108663	84.50	3e-162	hsd:SD1D_2422	K03495	gidA, mnmG, MTO1	tRNA uridine 5-carboxymethylaminomethyl modification enzyme	--	--

[denovogenes]_108666	42.50	2e-70	mhg:MHY_23960	K00937	ppk	polyphosphate kinase	2.7.4.1	Metabolism--Energy metabolism--Oxidative phosphorylation|Genetic Information Processing--Folding, sorting and degradation--RNA degradation

[denovogenes]_108668	62.40	3e-113	tur:AT726_06150	K03495	gidA, mnmG, MTO1	tRNA uridine 5-carboxymethylaminomethyl modification enzyme	--	--

[denovogenes]_108669	63.57	2e-114	twi:Thewi_0167	K00259	ald	alanine dehydrogenase	1.4.1.1	Metabolism--Amino acid metabolism--Alanine, aspartate and glutamate metabolism|Metabolism--Metabolism of other amino acids--Taurine and hypotaurine metabolism|Metabolism--Global and overview maps--Metabolic pathways

[denovogenes]_10867	60.74	0.0	clt:CM240_1762	K07003	K07003	uncharacterized protein	--	--

[denovogenes]_108672	51.17	3e-83	cso:CLS_36730	K01534	zntA	Cd2+/Zn2+-exporting ATPase	3.6.3.3 3.6.3.5	--

[denovogenes]_108675	73.64	3e-137	rto:RTO_06820	K21071	pfk, pfp	ATP-dependent phosphofructokinase / diphosphate-dependent phosphofructokinase	2.7.1.11 2.7.1.90	Metabolism--Carbohydrate metabolism--Glycolysis / Gluconeogenesis|Metabolism--Carbohydrate metabolism--Pentose phosphate pathway|Metabolism--Carbohydrate metabolism--Fructose and mannose metabolism|Metabolism--Carbohydrate metabolism--Galactose metabolism|Metabolism--Energy metabolism--Methane metabolism|Metabolism--Global and overview maps--Metabolic pathways|Metabolism--Global and overview maps--Biosynthesis of secondary metabolites|Metabolism--Global and overview maps--Microbial metabolism in diverse environments|Metabolism--Global and overview maps--Biosynthesis of antibiotics

[denovogenes]_108676	61.69	2e-109	bfu:BC1G_00472	K01955	carB, CPA2	carbamoyl-phosphate synthase large subunit	6.3.5.5	Metabolism--Nucleotide metabolism--Pyrimidine metabolism|Metabolism--Amino acid metabolism--Alanine, aspartate and glutamate metabolism|Metabolism--Global and overview maps--Metabolic pathways

[denovogenes]_108677	86.82	1e-164	bhl:Bache_2877	K01950	E6.3.5.1, NADSYN1, QNS1, nadE	NAD+ synthase (glutamine-hydrolysing)	6.3.5.1	Metabolism--Metabolism of cofactors and vitamins--Nicotinate and nicotinamide metabolism|Metabolism--Global and overview maps--Metabolic pathways

[denovogenes]_10868	41.45	9e-89	dgi:Desgi_4530	K02334	dpo	DNA polymerase bacteriophage-type	2.7.7.7	--

[denovogenes]_108683	94.94	5e-178	pfre:RM25_1407	K00208	fabI	enoyl-[acyl-carrier protein] reductase I	1.3.1.9 1.3.1.10	Metabolism--Lipid metabolism--Fatty acid biosynthesis|Metabolism--Metabolism of cofactors and vitamins--Biotin metabolism|Metabolism--Global and overview maps--Metabolic pathways|Metabolism--Global and overview maps--Fatty acid metabolism

[denovogenes]_108684	76.89	3e-138	pfre:RM25_0885	K10008	gluA	glutamate transport system ATP-binding protein	3.6.3.-	Environmental Information Processing--Membrane transport--ABC transporters

[denovogenes]_108687	94.49	6e-168	pdi:BDI_1609	K03146	THI4, THI1	thiamine thiazole synthase	--	Metabolism--Metabolism of cofactors and vitamins--Thiamine metabolism|Metabolism--Global and overview maps--Metabolic pathways

[denovogenes]_108688	98.05	2e-179	pfre:RM25_0421	K02303	cobA	uroporphyrin-III C-methyltransferase	2.1.1.107	Metabolism--Metabolism of cofactors and vitamins--Porphyrin and chlorophyll metabolism|Metabolism--Global and overview maps--Metabolic pathways|Metabolism--Global and overview maps--Biosynthesis of secondary metabolites

[denovogenes]_10869	99.41	0.0	stb:SGPB_0351	K01879	glyS	glycyl-tRNA synthetase beta chain	6.1.1.14	Genetic Information Processing--Translation--Aminoacyl-tRNA biosynthesis

[denovogenes]_108690	89.72	8e-171	pdi:BDI_3054	K01719	hemD, UROS	uroporphyrinogen-III synthase	4.2.1.75	Metabolism--Metabolism of cofactors and vitamins--Porphyrin and chlorophyll metabolism|Metabolism--Global and overview maps--Metabolic pathways|Metabolism--Global and overview maps--Biosynthesis of secondary metabolites

[denovogenes]_108691	87.94	2e-159	pfre:RM25_0735	K03394	cobI-cbiL	precorrin-2/cobalt-factor-2 C20-methyltransferase	2.1.1.130 2.1.1.151	Metabolism--Metabolism of cofactors and vitamins--Porphyrin and chlorophyll metabolism|Metabolism--Global and overview maps--Metabolic pathways

[denovogenes]_108692	93.36	2e-173	pfre:RM25_0748	K07009	K07009	uncharacterized protein	--	--

[denovogenes]_108693	53.36	5e-85	bact:AB656_03995	K06904	K06904	uncharacterized protein	--	--

[denovogenes]_108694	81.32	2e-154	pdi:BDI_0586	K01695	trpA	tryptophan synthase alpha chain	4.2.1.20	Metabolism--Amino acid metabolism--Glycine, serine and threonine metabolism|Metabolism--Amino acid metabolism--Phenylalanine, tyrosine and tryptophan biosynthesis|Metabolism--Global and overview maps--Metabolic pathways|Metabolism--Global and overview maps--Biosynthesis of secondary metabolites|Metabolism--Global and overview maps--Biosynthesis of antibiotics|Metabolism--Global and overview maps--Biosynthesis of amino acids

[denovogenes]_108696	99.22	0.0	btho:Btheta7330_02233	K03149	thiG	thiazole synthase	2.8.1.10	Metabolism--Metabolism of cofactors and vitamins--Thiamine metabolism|Metabolism--Global and overview maps--Metabolic pathways

[denovogenes]_1087	74.06	0.0	btho:Btheta7330_00849	K01190	lacZ	beta-galactosidase	3.2.1.23	Metabolism--Carbohydrate metabolism--Galactose metabolism|Metabolism--Glycan biosynthesis and metabolism--Other glycan degradation|Metabolism--Lipid metabolism--Sphingolipid metabolism|Metabolism--Global and overview maps--Metabolic pathways

[denovogenes]_108701	100.00	0.0	btho:Btheta7330_04905	K00930	argB	acetylglutamate kinase	2.7.2.8	Metabolism--Amino acid metabolism--Arginine biosynthesis|Metabolism--Global and overview maps--Metabolic pathways|Metabolism--Global and overview maps--Biosynthesis of secondary metabolites|Metabolism--Global and overview maps--Biosynthesis of antibiotics|Metabolism--Global and overview maps--2-Oxocarboxylic acid metabolism|Metabolism--Global and overview maps--Biosynthesis of amino acids

[denovogenes]_108704	69.26	5e-131	bth:BT_3933	K00210	E1.3.1.12	prephenate dehydrogenase	1.3.1.12	Metabolism--Amino acid metabolism--Phenylalanine, tyrosine and tryptophan biosynthesis|Metabolism--Biosynthesis of other secondary metabolites--Novobiocin biosynthesis|Metabolism--Global and overview maps--Metabolic pathways|Metabolism--Global and overview maps--Biosynthesis of secondary metabolites|Metabolism--Global and overview maps--Biosynthesis of antibiotics|Metabolism--Global and overview maps--Biosynthesis of amino acids

[denovogenes]_108707	100.00	0.0	paeo:M801_5	K00655	plsC	1-acyl-sn-glycerol-3-phosphate acyltransferase	2.3.1.51	Metabolism--Lipid metabolism--Glycerolipid metabolism|Metabolism--Lipid metabolism--Glycerophospholipid metabolism|Metabolism--Global and overview maps--Metabolic pathways|Metabolism--Global and overview maps--Biosynthesis of secondary metabolites

[denovogenes]_108708	43.48	2e-64	sbv:N643_12020	K02468	srlR, gutR	DeoR family transcriptional regulator, glucitol operon repressor	--	--

[denovogenes]_10871	50.22	0.0	rus:RBI_I00662	K06147	ABCB-BAC	ATP-binding cassette, subfamily B, bacterial	--	--

[denovogenes]_108713	99.61	0.0	fpla:A4U99_10460	K00757	udp, UPP	uridine phosphorylase	2.4.2.3	Metabolism--Nucleotide metabolism--Pyrimidine metabolism|Metabolism--Xenobiotics biodegradation and metabolism--Drug metabolism - other enzymes|Metabolism--Global and overview maps--Metabolic pathways

[denovogenes]_108714	100.00	0.0	paeo:M801_4799	K05799	pdhR	GntR family transcriptional regulator, transcriptional repressor for pyruvate dehydrogenase complex	--	--

[denovogenes]_108716	49.59	2e-82	btrm:SAMEA390648701335	K02500	hisF	cyclase	4.1.3.-	Metabolism--Amino acid metabolism--Histidine metabolism|Metabolism--Global and overview maps--Metabolic pathways|Metabolism--Global and overview maps--Biosynthesis of secondary metabolites|Metabolism--Global and overview maps--Biosynthesis of amino acids

[denovogenes]_108717	74.31	3e-142	cvi:CV_2111	K06223	dam	DNA adenine methylase	2.1.1.72	Genetic Information Processing--Replication and repair--Mismatch repair

[denovogenes]_108721	80.40	1e-143	eclx:LI66_23410	K12211	icmG, dotF	intracellular multiplication protein IcmG	--	--

[denovogenes]_108722	100.00	0.0	fpla:A4U99_13650	K00046	idnO	gluconate 5-dehydrogenase	1.1.1.69	--

[denovogenes]_108723	100.00	0.0	cfd:CFNIH1_18035	K02028	ABC.PA.A	polar amino acid transport system ATP-binding protein	3.6.3.21	--

[denovogenes]_108727	100.00	0.0	paeo:M801_5173	K09798	K09798	uncharacterized protein	--	--

[denovogenes]_108728	99.61	0.0	fpla:A4U99_10250	K03525	coaX	type III pantothenate kinase	2.7.1.33	Metabolism--Metabolism of cofactors and vitamins--Pantothenate and CoA biosynthesis|Metabolism--Global and overview maps--Metabolic pathways

[denovogenes]_108729	100.00	0.0	paeo:M801_5193	K02028	ABC.PA.A	polar amino acid transport system ATP-binding protein	3.6.3.21	--

[denovogenes]_10873	52.36	0.0	rum:CK1_06550	K00595	cobL	precorrin-6Y C5,15-methyltransferase (decarboxylating)	2.1.1.132	Metabolism--Metabolism of cofactors and vitamins--Porphyrin and chlorophyll metabolism|Metabolism--Global and overview maps--Metabolic pathways

[denovogenes]_108730	42.62	2e-21	blq:L21SP5_03403	K02477	K02477	two-component system, LytT family, response regulator	--	--

[denovogenes]_108731	100.00	0.0	bth:BT_3933	K00210	E1.3.1.12	prephenate dehydrogenase	1.3.1.12	Metabolism--Amino acid metabolism--Phenylalanine, tyrosine and tryptophan biosynthesis|Metabolism--Biosynthesis of other secondary metabolites--Novobiocin biosynthesis|Metabolism--Global and overview maps--Metabolic pathways|Metabolism--Global and overview maps--Biosynthesis of secondary metabolites|Metabolism--Global and overview maps--Biosynthesis of antibiotics|Metabolism--Global and overview maps--Biosynthesis of amino acids

[denovogenes]_108732	67.57	2e-124	fpr:FP2_06420	K04083	hslO	molecular chaperone Hsp33	--	--

[denovogenes]_108733	57.37	6e-92	dhd:Dhaf_2613	K02050	ABC.SN.P	NitT/TauT family transport system permease protein	--	--

[denovogenes]_108738	99.22	0.0	cfd:CFNIH1_13935	K02020	modA	molybdate transport system substrate-binding protein	--	Environmental Information Processing--Membrane transport--ABC transporters

[denovogenes]_108739	98.83	0.0	bfb:VU15_12815	K02342	DPO3E, dnaQ	DNA polymerase III subunit epsilon	2.7.7.7	Metabolism--Nucleotide metabolism--Purine metabolism|Metabolism--Nucleotide metabolism--Pyrimidine metabolism|Metabolism--Global and overview maps--Metabolic pathways|Genetic Information Processing--Replication and repair--DNA replication|Genetic Information Processing--Replication and repair--Mismatch repair|Genetic Information Processing--Replication and repair--Homologous recombination

[denovogenes]_10874	99.85	0.0	bvu:BVU_0102	K01206	FUCA	alpha-L-fucosidase	3.2.1.51	Metabolism--Glycan biosynthesis and metabolism--Other glycan degradation

[denovogenes]_108741	100.00	0.0	cfd:CFNIH1_08160	K06163	phnJ	alpha-D-ribose 1-methylphosphonate 5-phosphate C-P lyase	4.7.1.1	Metabolism--Metabolism of other amino acids--Phosphonate and phosphinate metabolism

[denovogenes]_108743	100.00	1e-177	paec:M802_5584	K03521	fixA, etfB	electron transfer flavoprotein beta subunit	--	--

[denovogenes]_108745	95.33	2e-176	bfg:BF638R_0189	K00930	argB	acetylglutamate kinase	2.7.2.8	Metabolism--Amino acid metabolism--Arginine biosynthesis|Metabolism--Global and overview maps--Metabolic pathways|Metabolism--Global and overview maps--Biosynthesis of secondary metabolites|Metabolism--Global and overview maps--Biosynthesis of antibiotics|Metabolism--Global and overview maps--2-Oxocarboxylic acid metabolism|Metabolism--Global and overview maps--Biosynthesis of amino acids

[denovogenes]_108746	100.00	0.0	cfd:CFNIH1_09305	K13637	uxuR	GntR family transcriptional regulator, uxu operon transcriptional repressor	--	--

[denovogenes]_108747	99.61	0.0	paeo:M801_3808	K15396	trmJ	tRNA (cytidine32/uridine32-2'-O)-methyltransferase	2.1.1.200	--

[denovogenes]_108748	99.61	0.0	ecg:E2348C_3872	K12981	waaZ, rfaZ	KDO transferase III	2.4.99.-	Metabolism--Glycan biosynthesis and metabolism--Lipopolysaccharide biosynthesis

[denovogenes]_108749	100.00	0.0	cfd:CFNIH1_07585	K03426	E3.6.1.22, NUDT12, nudC	NAD+ diphosphatase	3.6.1.22	Metabolism--Metabolism of cofactors and vitamins--Nicotinate and nicotinamide metabolism|Metabolism--Global and overview maps--Metabolic pathways|Cellular Processes--Transport and catabolism--Peroxisome

[denovogenes]_10875	67.16	0.0	rto:RTO_21760	K03657	uvrD, pcrA	DNA helicase II / ATP-dependent DNA helicase PcrA	3.6.4.12	Genetic Information Processing--Replication and repair--Nucleotide excision repair|Genetic Information Processing--Replication and repair--Mismatch repair

[denovogenes]_108751	79.84	2e-142	cgo:Corgl_0408	K03710	K03710	GntR family transcriptional regulator	--	--

[denovogenes]_108752	95.72	7e-178	bfb:VU15_01830	K00286	proC	pyrroline-5-carboxylate reductase	1.5.1.2	Metabolism--Amino acid metabolism--Arginine and proline metabolism|Metabolism--Global and overview maps--Metabolic pathways|Metabolism--Global and overview maps--Biosynthesis of secondary metabolites|Metabolism--Global and overview maps--Biosynthesis of antibiotics|Metabolism--Global and overview maps--Biosynthesis of amino acids

[denovogenes]_108755	98.05	0.0	lacy:A4V08_32885	K03525	coaX	type III pantothenate kinase	2.7.1.33	Metabolism--Metabolism of cofactors and vitamins--Pantothenate and CoA biosynthesis|Metabolism--Global and overview maps--Metabolic pathways

[denovogenes]_108756	90.27	3e-169	bcel:BcellWH2_05263	K11071	potB	spermidine/putrescine transport system permease protein	--	Environmental Information Processing--Membrane transport--ABC transporters

[denovogenes]_108758	100.00	0.0	efs:EFS1_1716	K11635	yxdL	putative ABC transport system ATP-binding protein	--	Environmental Information Processing--Signal transduction--Two-component system

[denovogenes]_10876	100.00	0.0	bxy:BXY_20760	K01874	MARS, metG	methionyl-tRNA synthetase	6.1.1.10	Metabolism--Metabolism of other amino acids--Selenocompound metabolism|Genetic Information Processing--Translation--Aminoacyl-tRNA biosynthesis

[denovogenes]_108760	100.00	0.0	cfd:CFNIH1_00540	K02468	srlR, gutR	DeoR family transcriptional regulator, glucitol operon repressor	--	--

[denovogenes]_108763	99.61	0.0	cfd:CFNIH1_21140	K05936	cobM, cbiF	precorrin-4/cobalt-precorrin-4 C11-methyltransferase	2.1.1.133 2.1.1.271	Metabolism--Metabolism of cofactors and vitamins--Porphyrin and chlorophyll metabolism|Metabolism--Global and overview maps--Metabolic pathways

[denovogenes]_108767	61.69	1e-63	sth:STH926	K03386	PRDX2_4, ahpC	peroxiredoxin (alkyl hydroperoxide reductase subunit C)	1.11.1.15	Cellular Processes--Cell growth and death--Apoptosis - fly

[denovogenes]_10877	98.67	0.0	boa:Bovatus_03017	K01874	MARS, metG	methionyl-tRNA synthetase	6.1.1.10	Metabolism--Metabolism of other amino acids--Selenocompound metabolism|Genetic Information Processing--Translation--Aminoacyl-tRNA biosynthesis

[denovogenes]_108771	59.75	2e-95	dvg:Deval_2528	K02533	lasT	tRNA/rRNA methyltransferase	2.1.1.-	--

[denovogenes]_108773	97.67	7e-177	fpla:A4U99_04120	K04720	cobD	threonine-phosphate decarboxylase	4.1.1.81	Metabolism--Metabolism of cofactors and vitamins--Porphyrin and chlorophyll metabolism

[denovogenes]_108774	53.33	2e-70	eyy:EGYY_21130	K16927	cbrT	energy-coupling factor transport system substrate-specific component	--	--

[denovogenes]_108775	91.05	2e-176	bfg:BF638R_3870	K09861	K09861	uncharacterized protein	--	--

[denovogenes]_108776	52.16	1e-93	prg:RB151_007300	K08297	caiA	crotonobetainyl-CoA dehydrogenase	1.3.99.-	--

[denovogenes]_108778	98.05	0.0	lacy:A4V08_14680	K01695	trpA	tryptophan synthase alpha chain	4.2.1.20	Metabolism--Amino acid metabolism--Glycine, serine and threonine metabolism|Metabolism--Amino acid metabolism--Phenylalanine, tyrosine and tryptophan biosynthesis|Metabolism--Global and overview maps--Metabolic pathways|Metabolism--Global and overview maps--Biosynthesis of secondary metabolites|Metabolism--Global and overview maps--Biosynthesis of antibiotics|Metabolism--Global and overview maps--Biosynthesis of amino acids

[denovogenes]_108779	50.62	3e-80	cbt:CLH_1959	K03481	glvR	RpiR family transcriptional regulator, glv operon transcriptional regulator	--	--

[denovogenes]_108780	42.86	9e-44	lpil:LIP_0391	K10218	ligK, galC	4-hydroxy-4-methyl-2-oxoglutarate aldolase	4.1.3.17	Metabolism--Xenobiotics biodegradation and metabolism--Benzoate degradation|Metabolism--Carbohydrate metabolism--C5-Branched dibasic acid metabolism|Metabolism--Global and overview maps--Microbial metabolism in diverse environments

[denovogenes]_108781	100.00	0.0	cfd:CFNIH1_22915	K03415	cheV	two-component system, chemotaxis family, response regulator CheV	--	Environmental Information Processing--Signal transduction--Two-component system|Cellular Processes--Cell motility--Bacterial chemotaxis

[denovogenes]_108785	71.60	5e-142	afd:Alfi_1658	K03787	surE	5'-nucleotidase	3.1.3.5	Metabolism--Nucleotide metabolism--Purine metabolism|Metabolism--Nucleotide metabolism--Pyrimidine metabolism|Metabolism--Metabolism of cofactors and vitamins--Nicotinate and nicotinamide metabolism|Metabolism--Global and overview maps--Metabolic pathways|Metabolism--Global and overview maps--Biosynthesis of secondary metabolites

[denovogenes]_108786	100.00	0.0	cfd:CFNIH1_07390	K00930	argB	acetylglutamate kinase	2.7.2.8	Metabolism--Amino acid metabolism--Arginine biosynthesis|Metabolism--Global and overview maps--Metabolic pathways|Metabolism--Global and overview maps--Biosynthesis of secondary metabolites|Metabolism--Global and overview maps--Biosynthesis of antibiotics|Metabolism--Global and overview maps--2-Oxocarboxylic acid metabolism|Metabolism--Global and overview maps--Biosynthesis of amino acids

[denovogenes]_108788	98.83	0.0	cfd:CFNIH1_09750	K09861	K09861	uncharacterized protein	--	--

[denovogenes]_108795	68.53	5e-104	euu:ADJ67_04080	K00003	E1.1.1.3	homoserine dehydrogenase	1.1.1.3	Metabolism--Amino acid metabolism--Glycine, serine and threonine metabolism|Metabolism--Amino acid metabolism--Cysteine and methionine metabolism|Metabolism--Amino acid metabolism--Lysine biosynthesis|Metabolism--Global and overview maps--Metabolic pathways|Metabolism--Global and overview maps--Biosynthesis of secondary metabolites|Metabolism--Global and overview maps--Microbial metabolism in diverse environments|Metabolism--Global and overview maps--Biosynthesis of antibiotics|Metabolism--Global and overview maps--Biosynthesis of amino acids

[denovogenes]_108799	42.35	3e-46	tth:TT_P0220	K01190	lacZ	beta-galactosidase	3.2.1.23	Metabolism--Carbohydrate metabolism--Galactose metabolism|Metabolism--Glycan biosynthesis and metabolism--Other glycan degradation|Metabolism--Lipid metabolism--Sphingolipid metabolism|Metabolism--Global and overview maps--Metabolic pathways

[denovogenes]_1088	65.32	0.0	ecas:ECBG_01436	K03529	smc	chromosome segregation protein	--	--

[denovogenes]_10880	92.95	0.0	bhl:Bache_2373	K00700	GBE1, glgB	1,4-alpha-glucan branching enzyme	2.4.1.18	Metabolism--Carbohydrate metabolism--Starch and sucrose metabolism|Metabolism--Global and overview maps--Metabolic pathways|Metabolism--Global and overview maps--Biosynthesis of secondary metabolites

[denovogenes]_108806	60.08	3e-108	tped:TPE_1514	K02033	ABC.PE.P	peptide/nickel transport system permease protein	--	Cellular Processes--Cellular community - prokaryotes--Quorum sensing

[denovogenes]_108808	69.57	2e-130	rto:RTO_14990	K03424	--	--	--	--

[denovogenes]_108809	82.49	2e-159	pdi:BDI_0586	K01695	trpA	tryptophan synthase alpha chain	4.2.1.20	Metabolism--Amino acid metabolism--Glycine, serine and threonine metabolism|Metabolism--Amino acid metabolism--Phenylalanine, tyrosine and tryptophan biosynthesis|Metabolism--Global and overview maps--Metabolic pathways|Metabolism--Global and overview maps--Biosynthesis of secondary metabolites|Metabolism--Global and overview maps--Biosynthesis of antibiotics|Metabolism--Global and overview maps--Biosynthesis of amino acids

[denovogenes]_108810	98.01	1e-174	bcel:BcellWH2_03232	K03797	E3.4.21.102, prc, ctpA	carboxyl-terminal processing protease	3.4.21.102	--

[denovogenes]_108814	76.65	7e-144	csh:Closa_2893	K06381	spoIID	stage II sporulation protein D	--	--

[denovogenes]_108818	41.88	4e-16	ppw:PputW619_2736	K07506	K07506	AraC family transcriptional regulator	--	--

[denovogenes]_108820	98.44	0.0	bth:BT_3933	K00210	E1.3.1.12	prephenate dehydrogenase	1.3.1.12	Metabolism--Amino acid metabolism--Phenylalanine, tyrosine and tryptophan biosynthesis|Metabolism--Biosynthesis of other secondary metabolites--Novobiocin biosynthesis|Metabolism--Global and overview maps--Metabolic pathways|Metabolism--Global and overview maps--Biosynthesis of secondary metabolites|Metabolism--Global and overview maps--Biosynthesis of antibiotics|Metabolism--Global and overview maps--Biosynthesis of amino acids

[denovogenes]_108821	74.90	2e-146	csh:Closa_0799	K01620	ltaE	threonine aldolase	4.1.2.48	Metabolism--Amino acid metabolism--Glycine, serine and threonine metabolism|Metabolism--Global and overview maps--Metabolic pathways|Metabolism--Global and overview maps--Biosynthesis of secondary metabolites|Metabolism--Global and overview maps--Microbial metabolism in diverse environments|Metabolism--Global and overview maps--Biosynthesis of antibiotics|Metabolism--Global and overview maps--Biosynthesis of amino acids

[denovogenes]_108824	96.50	7e-165	bbrv:B689b_0723	K03631	recN	DNA repair protein RecN (Recombination protein N)	--	--

[denovogenes]_108826	99.61	2e-180	pae:PA4961	K07186	smp	membrane protein	--	--

[denovogenes]_108827	92.31	5e-169	bcel:BcellWH2_03943	K02528	ksgA	16S rRNA (adenine1518-N6/adenine1519-N6)-dimethyltransferase	2.1.1.182	--

[denovogenes]_108829	52.31	6e-87	mhg:MHY_06370	K03336	iolD	3D-(3,5/4)-trihydroxycyclohexane-1,2-dione acylhydrolase (decyclizing)	3.7.1.22	Metabolism--Carbohydrate metabolism--Inositol phosphate metabolism|Metabolism--Global and overview maps--Metabolic pathways|Metabolism--Global and overview maps--Microbial metabolism in diverse environments

[denovogenes]_10883	81.29	0.0	ruk:A4V00_07180	K02355	fusA, GFM, EFG	elongation factor G	--	--

[denovogenes]_108830	75.10	5e-137	csh:Closa_1574	K00215	dapB	4-hydroxy-tetrahydrodipicolinate reductase	1.17.1.8	Metabolism--Biosynthesis of other secondary metabolites--Monobactam biosynthesis|Metabolism--Amino acid metabolism--Lysine biosynthesis|Metabolism--Global and overview maps--Metabolic pathways|Metabolism--Global and overview maps--Biosynthesis of secondary metabolites|Metabolism--Global and overview maps--Microbial metabolism in diverse environments|Metabolism--Global and overview maps--Biosynthesis of antibiotics|Metabolism--Global and overview maps--Biosynthesis of amino acids

[denovogenes]_108831	66.42	5e-126	cct:CC1_25470	K15051	K15051, endA	DNA-entry nuclease	--	--

[denovogenes]_108832	97.64	0.0	bcel:BcellWH2_01039	K06871	K06871	uncharacterized protein	--	--

[denovogenes]_108834	100.00	0.0	bcel:BcellWH2_05263	K11071	potB	spermidine/putrescine transport system permease protein	--	Environmental Information Processing--Membrane transport--ABC transporters

[denovogenes]_108835	41.00	7e-59	shq:A0259_16330	K07497	K07497	putative transposase	--	--

[denovogenes]_108837	49.09	4e-23	ava:Ava_C0210	K08259	lytM	lysostaphin	3.4.24.75	--

[denovogenes]_108838	52.87	4e-87	byl:A4V09_06130	K07124	K07124	uncharacterized protein	--	--

[denovogenes]_108839	66.93	1e-120	rto:RTO_04040	K00768	E2.4.2.21, cobU, cobT	nicotinate-nucleotide--dimethylbenzimidazole phosphoribosyltransferase	2.4.2.21	Metabolism--Metabolism of cofactors and vitamins--Porphyrin and chlorophyll metabolism|Metabolism--Global and overview maps--Metabolic pathways

[denovogenes]_108841	88.24	3e-166	clt:CM240_1204	K02118	ATPVB, ntpB, atpB	V/A-type H+/Na+-transporting ATPase subunit B	--	Metabolism--Energy metabolism--Oxidative phosphorylation|Metabolism--Global and overview maps--Metabolic pathways

[denovogenes]_108842	41.82	1e-46	bur:Bcep18194_B1624	K00219	fadH	2,4-dienoyl-CoA reductase (NADPH2)	1.3.1.34	--

[denovogenes]_108843	45.35	3e-71	tae:TepiRe1_0041	K15580	oppA, mppA	oligopeptide transport system substrate-binding protein	--	Human Diseases--Drug resistance: Antimicrobial--beta-Lactam resistance|Environmental Information Processing--Membrane transport--ABC transporters|Cellular Processes--Cellular community - prokaryotes--Quorum sensing

[denovogenes]_108844	97.67	0.0	bcel:BcellWH2_05407	K02005	ABC.CD.TX	HlyD family secretion protein	--	--

[denovogenes]_108848	98.84	0.0	bcel:BcellWH2_05484	K08138	xylE	MFS transporter, SP family, xylose:H+ symportor	--	--

[denovogenes]_108851	96.50	2e-180	lacy:A4V08_07675	K07402	xdhC	xanthine dehydrogenase accessory factor	--	--

[denovogenes]_108852	59.67	5e-99	byl:A4V09_21945	K02119	ATPVC, ntpC, atpC	V/A-type H+/Na+-transporting ATPase subunit C	--	Metabolism--Energy metabolism--Oxidative phosphorylation|Metabolism--Global and overview maps--Metabolic pathways

[denovogenes]_108854	100.00	2e-179	cfd:CFNIH1_16535	K02761	PTS-Cel-EIIC, celB, chbC	PTS system, cellobiose-specific IIC component	--	Metabolism--Carbohydrate metabolism--Starch and sucrose metabolism|Environmental Information Processing--Membrane transport--Phosphotransferase system (PTS)

[denovogenes]_108856	98.04	0.0	cif:AL515_15820	K03415	cheV	two-component system, chemotaxis family, response regulator CheV	--	Environmental Information Processing--Signal transduction--Two-component system|Cellular Processes--Cell motility--Bacterial chemotaxis

[denovogenes]_108857	60.00	1e-106	crn:CAR_c22680	K10541	mglC	methyl-galactoside transport system permease protein	--	Environmental Information Processing--Membrane transport--ABC transporters

[denovogenes]_10886	86.58	0.0	bhl:Bache_1824	K03628	rho	transcription termination factor Rho	--	Genetic Information Processing--Folding, sorting and degradation--RNA degradation

[denovogenes]_108861	99.22	4e-180	bbrn:B2258_1011	K01696	trpB	tryptophan synthase beta chain	4.2.1.20	Metabolism--Amino acid metabolism--Glycine, serine and threonine metabolism|Metabolism--Amino acid metabolism--Phenylalanine, tyrosine and tryptophan biosynthesis|Metabolism--Global and overview maps--Metabolic pathways|Metabolism--Global and overview maps--Biosynthesis of secondary metabolites|Metabolism--Global and overview maps--Biosynthesis of antibiotics|Metabolism--Global and overview maps--Biosynthesis of amino acids

[denovogenes]_108862	83.14	1e-153	afd:Alfi_3218	K00791	miaA, TRIT1	tRNA dimethylallyltransferase	2.5.1.75	Metabolism--Metabolism of terpenoids and polyketides--Zeatin biosynthesis|Metabolism--Global and overview maps--Metabolic pathways|Metabolism--Global and overview maps--Biosynthesis of secondary metabolites

[denovogenes]_108863	68.60	3e-130	cbv:U729_2549	K04487	iscS, NFS1	cysteine desulfurase	2.8.1.7	Metabolism--Metabolism of cofactors and vitamins--Thiamine metabolism|Metabolism--Global and overview maps--Metabolic pathways|Genetic Information Processing--Folding, sorting and degradation--Sulfur relay system

[denovogenes]_108866	61.48	5e-115	efm:M7W_2359	K02026	ABC.MS.P1	multiple sugar transport system permease protein	--	--

[denovogenes]_108868	100.00	0.0	pdi:BDI_2687	K07214	fes	enterochelin esterase and related enzymes	--	--

[denovogenes]_10887	98.67	0.0	fpla:A4U99_00260	K00087	ygeS, xdhA	xanthine dehydrogenase molybdenum-binding subunit	1.17.1.4	Metabolism--Nucleotide metabolism--Purine metabolism|Metabolism--Global and overview maps--Metabolic pathways|Metabolism--Global and overview maps--Microbial metabolism in diverse environments

[denovogenes]_108870	92.64	1e-173	bcel:BcellWH2_01565	K01847	MUT	methylmalonyl-CoA mutase	5.4.99.2	Metabolism--Amino acid metabolism--Valine, leucine and isoleucine degradation|Metabolism--Carbohydrate metabolism--Glyoxylate and dicarboxylate metabolism|Metabolism--Carbohydrate metabolism--Propanoate metabolism|Metabolism--Energy metabolism--Carbon fixation pathways in prokaryotes|Metabolism--Global and overview maps--Metabolic pathways|Metabolism--Global and overview maps--Microbial metabolism in diverse environments|Metabolism--Global and overview maps--Carbon metabolism

[denovogenes]_108875	97.29	0.0	pvl:AOB99_11875	K02836	prfB	peptide chain release factor 2	--	--

[denovogenes]_108876	100.00	0.0	bbrv:B689b_0835	K04096	smf	DNA processing protein	--	--

[denovogenes]_108880	100.00	0.0	bbrd:BBBR_1836	K01874	MARS, metG	methionyl-tRNA synthetase	6.1.1.10	Metabolism--Metabolism of other amino acids--Selenocompound metabolism|Genetic Information Processing--Translation--Aminoacyl-tRNA biosynthesis

[denovogenes]_108882	100.00	0.0	bbrs:BS27_1270	K01443	nagA, AMDHD2	N-acetylglucosamine-6-phosphate deacetylase	3.5.1.25	Metabolism--Carbohydrate metabolism--Amino sugar and nucleotide sugar metabolism|Metabolism--Global and overview maps--Biosynthesis of antibiotics

[denovogenes]_108884	94.96	0.0	lro:LOCK900_0830	K03734	apbE	FAD:protein FMN transferase	2.7.1.180	--

[denovogenes]_108885	98.16	1e-153	bcel:BcellWH2_04251	K01875	SARS, serS	seryl-tRNA synthetase	6.1.1.11	Genetic Information Processing--Translation--Aminoacyl-tRNA biosynthesis

[denovogenes]_108886	46.72	6e-66	clj:CLJU_c28050	K02205	rocE, rocC	arginine/ornithine permease	--	--

[denovogenes]_108888	66.28	2e-126	tde:TDE1271	K02032	ABC.PE.A1	peptide/nickel transport system ATP-binding protein	--	Cellular Processes--Cellular community - prokaryotes--Quorum sensing

[denovogenes]_108891	41.63	4e-38	tle:Tlet_0987	K07636	phoR	two-component system, OmpR family, phosphate regulon sensor histidine kinase PhoR	2.7.13.3	Environmental Information Processing--Signal transduction--Two-component system

[denovogenes]_108892	94.19	9e-176	lrg:LRHM_0577	K18891	patA, rscA, lmrC, satA	ATP-binding cassette, subfamily B, multidrug efflux pump	--	Environmental Information Processing--Membrane transport--ABC transporters

[denovogenes]_108894	73.75	3e-137	rch:RUM_16500	K19955	adh2	alcohol dehydrogenase	1.1.1.-	--

[denovogenes]_108896	93.80	1e-174	pvl:AOB99_05890	K07478	ycaJ	putative ATPase	--	--

[denovogenes]_108897	78.33	5e-134	cjer:H730_04265	K01547	kdpB	K+-transporting ATPase ATPase B chain	3.6.3.12	Environmental Information Processing--Signal transduction--Two-component system

[denovogenes]_108898	40.87	5e-49	bacc:BRDCF_p614	K03657	uvrD, pcrA	DNA helicase II / ATP-dependent DNA helicase PcrA	3.6.4.12	Genetic Information Processing--Replication and repair--Nucleotide excision repair|Genetic Information Processing--Replication and repair--Mismatch repair

[denovogenes]_108899	90.35	7e-175	boa:Bovatus_03040	K08641	vanX	D-alanyl-D-alanine dipeptidase	3.4.13.22	Human Diseases--Drug resistance: Antimicrobial--Vancomycin resistance|Environmental Information Processing--Signal transduction--Two-component system

[denovogenes]_1089	77.41	0.0	cbv:U729_763	K02337	DPO3A1, dnaE	DNA polymerase III subunit alpha	2.7.7.7	Metabolism--Nucleotide metabolism--Purine metabolism|Metabolism--Nucleotide metabolism--Pyrimidine metabolism|Metabolism--Global and overview maps--Metabolic pathways|Genetic Information Processing--Replication and repair--DNA replication|Genetic Information Processing--Replication and repair--Mismatch repair|Genetic Information Processing--Replication and repair--Homologous recombination

[denovogenes]_10890	100.00	0.0	bvu:BVU_3844	K01190	lacZ	beta-galactosidase	3.2.1.23	Metabolism--Carbohydrate metabolism--Galactose metabolism|Metabolism--Glycan biosynthesis and metabolism--Other glycan degradation|Metabolism--Lipid metabolism--Sphingolipid metabolism|Metabolism--Global and overview maps--Metabolic pathways

[denovogenes]_108903	92.22	3e-174	boa:Bovatus_04431	K00674	dapD	2,3,4,5-tetrahydropyridine-2-carboxylate N-succinyltransferase	2.3.1.117	Metabolism--Amino acid metabolism--Lysine biosynthesis|Metabolism--Global and overview maps--Metabolic pathways|Metabolism--Global and overview maps--Microbial metabolism in diverse environments|Metabolism--Global and overview maps--Biosynthesis of amino acids

[denovogenes]_108904	98.05	0.0	boa:Bovatus_03872	K00286	proC	pyrroline-5-carboxylate reductase	1.5.1.2	Metabolism--Amino acid metabolism--Arginine and proline metabolism|Metabolism--Global and overview maps--Metabolic pathways|Metabolism--Global and overview maps--Biosynthesis of secondary metabolites|Metabolism--Global and overview maps--Biosynthesis of antibiotics|Metabolism--Global and overview maps--Biosynthesis of amino acids

[denovogenes]_108905	44.19	2e-69	sapi:SAPIS_v1c08100	K09769	K09769	uncharacterized protein	--	--

[denovogenes]_108907	99.61	0.0	boa:Bovatus_00986	K03149	thiG	thiazole synthase	2.8.1.10	Metabolism--Metabolism of cofactors and vitamins--Thiamine metabolism|Metabolism--Global and overview maps--Metabolic pathways

[denovogenes]_108914	95.72	6e-178	boa:Bovatus_03532	K00930	argB	acetylglutamate kinase	2.7.2.8	Metabolism--Amino acid metabolism--Arginine biosynthesis|Metabolism--Global and overview maps--Metabolic pathways|Metabolism--Global and overview maps--Biosynthesis of secondary metabolites|Metabolism--Global and overview maps--Biosynthesis of antibiotics|Metabolism--Global and overview maps--2-Oxocarboxylic acid metabolism|Metabolism--Global and overview maps--Biosynthesis of amino acids

[denovogenes]_108916	71.98	1e-130	cpy:Cphy_0713	K07238	TC.ZIP, zupT, ZRT3, ZIP2	zinc transporter, ZIP family	--	--

[denovogenes]_108919	93.39	4e-178	sut:SAT0131_02871	K04763	xerD	integrase/recombinase XerD	--	--

[denovogenes]_10892	46.33	0.0	ham:HALO0772	K03407	cheA	two-component system, chemotaxis family, sensor kinase CheA	2.7.13.3	Environmental Information Processing--Signal transduction--Two-component system|Cellular Processes--Cell motility--Bacterial chemotaxis

[denovogenes]_108920	100.00	0.0	pvl:AOB99_05695	K02500	hisF	cyclase	4.1.3.-	Metabolism--Amino acid metabolism--Histidine metabolism|Metabolism--Global and overview maps--Metabolic pathways|Metabolism--Global and overview maps--Biosynthesis of secondary metabolites|Metabolism--Global and overview maps--Biosynthesis of amino acids

[denovogenes]_108922	100.00	3e-158	pvl:AOB99_05635	K00876	udk, UCK	uridine kinase	2.7.1.48	Metabolism--Nucleotide metabolism--Pyrimidine metabolism|Metabolism--Xenobiotics biodegradation and metabolism--Drug metabolism - other enzymes|Metabolism--Global and overview maps--Metabolic pathways

[denovogenes]_108926	100.00	0.0	cpf:CPF_2767	K03284	corA	magnesium transporter	--	--

[denovogenes]_108927	98.84	2e-175	pdi:BDI_2434	K03977	engA, der	GTPase	--	--

[denovogenes]_108928	49.40	2e-87	ava:Ava_2888	K05886	sdh	serine 3-dehydrogenase (NADP+)	1.1.1.276	--

[denovogenes]_108929	64.31	6e-117	vpr:Vpar_1073	K00850	pfkA, PFK	6-phosphofructokinase 1	2.7.1.11	Metabolism--Carbohydrate metabolism--Glycolysis / Gluconeogenesis|Metabolism--Carbohydrate metabolism--Pentose phosphate pathway|Metabolism--Carbohydrate metabolism--Fructose and mannose metabolism|Metabolism--Carbohydrate metabolism--Galactose metabolism|Metabolism--Energy metabolism--Methane metabolism|Metabolism--Global and overview maps--Metabolic pathways|Metabolism--Global and overview maps--Biosynthesis of secondary metabolites|Metabolism--Global and overview maps--Microbial metabolism in diverse environments|Metabolism--Global and overview maps--Biosynthesis of antibiotics|Metabolism--Global and overview maps--Carbon metabolism|Metabolism--Global and overview maps--Biosynthesis of amino acids|Genetic Information Processing--Folding, sorting and degradation--RNA degradation|Environmental Information Processing--Signal transduction--AMPK signaling pathway|Human Diseases--Cancers: Overview--Central carbon metabolism in cancer

[denovogenes]_108930	79.30	2e-146	vpr:Vpar_0921	K13789	GGPS	geranylgeranyl diphosphate synthase, type II	2.5.1.1 2.5.1.10 2.5.1.29	Metabolism--Metabolism of terpenoids and polyketides--Terpenoid backbone biosynthesis|Metabolism--Global and overview maps--Metabolic pathways|Metabolism--Global and overview maps--Biosynthesis of secondary metabolites|Metabolism--Global and overview maps--Biosynthesis of antibiotics

[denovogenes]_108931	67.30	5e-119	ain:Acin_1548	K16960	tcyN	L-cystine transport system ATP-binding protein	3.6.3.-	Environmental Information Processing--Membrane transport--ABC transporters

[denovogenes]_108932	100.00	0.0	pvl:AOB99_16555	K04083	hslO	molecular chaperone Hsp33	--	--

[denovogenes]_108936	66.41	4e-118	ipo:Ilyop_1716	K01662	dxs	1-deoxy-D-xylulose-5-phosphate synthase	2.2.1.7	Metabolism--Metabolism of cofactors and vitamins--Thiamine metabolism|Metabolism--Metabolism of terpenoids and polyketides--Terpenoid backbone biosynthesis|Metabolism--Global and overview maps--Metabolic pathways|Metabolism--Global and overview maps--Biosynthesis of secondary metabolites|Metabolism--Global and overview maps--Biosynthesis of antibiotics

[denovogenes]_108938	79.03	5e-147	cbv:U729_2405	K01590	hdc, HDC	histidine decarboxylase	4.1.1.22	Metabolism--Amino acid metabolism--Histidine metabolism|Metabolism--Global and overview maps--Metabolic pathways|Metabolism--Global and overview maps--Biosynthesis of secondary metabolites

[denovogenes]_108939	57.71	3e-96	pcr:Pcryo_2061	K00076	hdhA	7-alpha-hydroxysteroid dehydrogenase	1.1.1.159	--

[denovogenes]_10894	62.50	0.0	shi:Shel_28440	K03722	dinG	ATP-dependent DNA helicase DinG	3.6.4.12	--

[denovogenes]_108940	73.71	2e-139	bxy:BXY_44460	K00652	bioF	8-amino-7-oxononanoate synthase	2.3.1.47	Metabolism--Metabolism of cofactors and vitamins--Biotin metabolism|Metabolism--Global and overview maps--Metabolic pathways

[denovogenes]_108941	99.61	0.0	pmib:BB2000_0156	K03832	tonB	periplasmic protein TonB	--	--

[denovogenes]_108943	65.77	5e-117	amt:Amet_4244	K03300	TC.CITMHS	citrate-Mg2+:H+ or citrate-Ca2+:H+ symporter, CitMHS family	--	--

[denovogenes]_108944	54.65	2e-91	sfn:SFy_6036	K07347	fimD, fimC, mrkC, htrE, cssD	outer membrane usher protein	--	Human Diseases--Infectious diseases: Bacterial--Pertussis

[denovogenes]_108945	66.02	2e-114	ipo:Ilyop_0329	K10440	rbsC	ribose transport system permease protein	--	Environmental Information Processing--Membrane transport--ABC transporters

[denovogenes]_108946	44.54	2e-55	fnu:FN0223	K02238	comEC	competence protein ComEC	--	--

[denovogenes]_108948	100.00	0.0	ecm:EcSMS35_0456	K01662	dxs	1-deoxy-D-xylulose-5-phosphate synthase	2.2.1.7	Metabolism--Metabolism of cofactors and vitamins--Thiamine metabolism|Metabolism--Metabolism of terpenoids and polyketides--Terpenoid backbone biosynthesis|Metabolism--Global and overview maps--Metabolic pathways|Metabolism--Global and overview maps--Biosynthesis of secondary metabolites|Metabolism--Global and overview maps--Biosynthesis of antibiotics

[denovogenes]_108949	55.65	2e-84	sao:SAOUHSC_00779	K03702	uvrB	excinuclease ABC subunit B	--	Genetic Information Processing--Replication and repair--Nucleotide excision repair

[denovogenes]_10895	99.71	0.0	btho:Btheta7330_00163	K01874	MARS, metG	methionyl-tRNA synthetase	6.1.1.10	Metabolism--Metabolism of other amino acids--Selenocompound metabolism|Genetic Information Processing--Translation--Aminoacyl-tRNA biosynthesis

[denovogenes]_108951	66.53	2e-111	ecas:ECBG_02433	K12373	HEXA_B	hexosaminidase	3.2.1.52	Metabolism--Glycan biosynthesis and metabolism--Other glycan degradation|Metabolism--Glycan biosynthesis and metabolism--Various types of N-glycan biosynthesis|Metabolism--Carbohydrate metabolism--Amino sugar and nucleotide sugar metabolism|Metabolism--Glycan biosynthesis and metabolism--Glycosaminoglycan degradation|Metabolism--Glycan biosynthesis and metabolism--Glycosphingolipid biosynthesis - globo and isoglobo series|Metabolism--Glycan biosynthesis and metabolism--Glycosphingolipid biosynthesis - ganglio series|Metabolism--Global and overview maps--Metabolic pathways|Cellular Processes--Transport and catabolism--Lysosome

[denovogenes]_108953	49.07	9e-78	eel:EUBELI_00226	K03581	recD	exodeoxyribonuclease V alpha subunit	3.1.11.5	Genetic Information Processing--Replication and repair--Homologous recombination

[denovogenes]_108957	100.00	0.0	ecr:ECIAI1_3413	K09970	aapQ, bztB	general L-amino acid transport system permease protein	--	Environmental Information Processing--Membrane transport--ABC transporters

[denovogenes]_108958	72.09	4e-138	bcef:BcrFT9_01563	K01990	ABC-2.A	ABC-2 type transport system ATP-binding protein	--	--

[denovogenes]_10896	95.73	0.0	pdi:BDI_2791	K01874	MARS, metG	methionyl-tRNA synthetase	6.1.1.10	Metabolism--Metabolism of other amino acids--Selenocompound metabolism|Genetic Information Processing--Translation--Aminoacyl-tRNA biosynthesis

[denovogenes]_108960	100.00	0.0	bvu:BVU_4178	K06889	K06889	uncharacterized protein	--	--

[denovogenes]_108963	99.61	0.0	cpf:CPF_2655	K00532	E1.12.7.2	ferredoxin hydrogenase	1.12.7.2	--

[denovogenes]_108965	100.00	0.0	eoc:CE10_4324	K01232	glvA	maltose-6'-phosphate glucosidase	3.2.1.122	Metabolism--Carbohydrate metabolism--Starch and sucrose metabolism

[denovogenes]_108968	40.52	7e-12	ppse:BN5_0407	K03183	ubiE	demethylmenaquinone methyltransferase / 2-methoxy-6-polyprenyl-1,4-benzoquinol methylase	2.1.1.163 2.1.1.201	Metabolism--Metabolism of cofactors and vitamins--Ubiquinone and other terpenoid-quinone biosynthesis|Metabolism--Global and overview maps--Metabolic pathways|Metabolism--Global and overview maps--Biosynthesis of secondary metabolites

[denovogenes]_10897	42.53	3e-91	tvo:TVG0998758	K13525	VCP, CDC48	transitional endoplasmic reticulum ATPase	--	Genetic Information Processing--Folding, sorting and degradation--Protein processing in endoplasmic reticulum|Human Diseases--Infectious diseases: Bacterial--Legionellosis

[denovogenes]_108972	100.00	0.0	mmk:MU9_2603	K04754	mlaA, vacJ	phospholipid-binding lipoprotein MlaA	--	--

[denovogenes]_108974	100.00	1e-179	mmk:MU9_1120	K01992	ABC-2.P	ABC-2 type transport system permease protein	--	--

[denovogenes]_108975	61.63	8e-121	efm:M7W_1862	K02823	pyrDII	dihydroorotate dehydrogenase electron transfer subunit	--	Metabolism--Nucleotide metabolism--Pyrimidine metabolism|Metabolism--Global and overview maps--Metabolic pathways

[denovogenes]_108976	100.00	0.0	mmk:MU9_676	K03772	fkpA	FKBP-type peptidyl-prolyl cis-trans isomerase FkpA	5.2.1.8	--

[denovogenes]_108977	87.11	2e-164	edu:LIU_01905	K01992	ABC-2.P	ABC-2 type transport system permease protein	--	--

[denovogenes]_108978	99.61	0.0	mmk:MU9_3400	K01995	livG	branched-chain amino acid transport system ATP-binding protein	--	Environmental Information Processing--Membrane transport--ABC transporters|Cellular Processes--Cellular community - prokaryotes--Quorum sensing

[denovogenes]_108979	100.00	0.0	bfb:VU15_00300	K00677	lpxA	UDP-N-acetylglucosamine acyltransferase	2.3.1.129	Metabolism--Glycan biosynthesis and metabolism--Lipopolysaccharide biosynthesis|Metabolism--Global and overview maps--Metabolic pathways|Human Diseases--Drug resistance: Antimicrobial--Cationic antimicrobial peptide (CAMP) resistance

[denovogenes]_10898	70.92	3e-176	dfi:AXF13_01670	K03406	mcp	methyl-accepting chemotaxis protein	--	Environmental Information Processing--Signal transduction--Two-component system|Cellular Processes--Cell motility--Bacterial chemotaxis

[denovogenes]_108980	53.75	2e-97	ava:Ava_2888	K05886	sdh	serine 3-dehydrogenase (NADP+)	1.1.1.276	--

[denovogenes]_108981	67.19	1e-131	ega:AL523_14880	K03584	recO	DNA repair protein RecO (recombination protein O)	--	Genetic Information Processing--Replication and repair--Homologous recombination

[denovogenes]_108982	63.14	4e-113	bhu:bhn_I1902	K01695	trpA	tryptophan synthase alpha chain	4.2.1.20	Metabolism--Amino acid metabolism--Glycine, serine and threonine metabolism|Metabolism--Amino acid metabolism--Phenylalanine, tyrosine and tryptophan biosynthesis|Metabolism--Global and overview maps--Metabolic pathways|Metabolism--Global and overview maps--Biosynthesis of secondary metabolites|Metabolism--Global and overview maps--Biosynthesis of antibiotics|Metabolism--Global and overview maps--Biosynthesis of amino acids

[denovogenes]_108984	69.64	9e-117	cbv:U729_1227	K13292	lgt, umpA	phosphatidylglycerol:prolipoprotein diacylglycerol transferase	2.-.-.-	--

[denovogenes]_108985	67.08	3e-116	csb:CLSA_c00800	K13292	lgt, umpA	phosphatidylglycerol:prolipoprotein diacylglycerol transferase	2.-.-.-	--

[denovogenes]_108986	99.61	0.0	mmk:MU9_348	K02170	bioH	pimeloyl-[acyl-carrier protein] methyl ester esterase	3.1.1.85	Metabolism--Metabolism of cofactors and vitamins--Biotin metabolism|Metabolism--Global and overview maps--Metabolic pathways

[denovogenes]_108988	77.65	1e-144	cbn:CbC4_1343	K02003	ABC.CD.A	putative ABC transport system ATP-binding protein	--	--

[denovogenes]_108989	86.33	5e-163	lacy:A4V08_17705	K03496	parA, soj	chromosome partitioning protein	--	--

[denovogenes]_108992	99.61	0.0	efq:DR75_2871	K03424	--	--	--	--

[denovogenes]_108993	100.00	0.0	ene:ENT_29580	K01992	ABC-2.P	ABC-2 type transport system permease protein	--	--

[denovogenes]_108994	41.36	9e-37	cow:Calow_0882	K01448	E3.5.1.28B, amiA, amiB, amiC	N-acetylmuramoyl-L-alanine amidase	3.5.1.28	Human Diseases--Drug resistance: Antimicrobial--Cationic antimicrobial peptide (CAMP) resistance

[denovogenes]_108995	99.22	0.0	mmk:MU9_697	K03149	thiG	thiazole synthase	2.8.1.10	Metabolism--Metabolism of cofactors and vitamins--Thiamine metabolism|Metabolism--Global and overview maps--Metabolic pathways

[denovogenes]_108996	100.00	0.0	bfb:VU15_18410	K02065	mlaF, linL, mkl	phospholipid/cholesterol/gamma-HCH transport system ATP-binding protein	--	Environmental Information Processing--Membrane transport--ABC transporters

[denovogenes]_108997	88.98	2e-152	lacy:A4V08_26440	K03218	rlmB	23S rRNA (guanosine2251-2'-O)-methyltransferase	2.1.1.185	--

[denovogenes]_108998	98.44	0.0	stb:SGPB_0099	K00286	proC	pyrroline-5-carboxylate reductase	1.5.1.2	Metabolism--Amino acid metabolism--Arginine and proline metabolism|Metabolism--Global and overview maps--Metabolic pathways|Metabolism--Global and overview maps--Biosynthesis of secondary metabolites|Metabolism--Global and overview maps--Biosynthesis of antibiotics|Metabolism--Global and overview maps--Biosynthesis of amino acids

[denovogenes]_108999	89.45	6e-167	pdf:CD630DERM_17040	K03149	thiG	thiazole synthase	2.8.1.10	Metabolism--Metabolism of cofactors and vitamins--Thiamine metabolism|Metabolism--Global and overview maps--Metabolic pathways

[denovogenes]_109001	99.22	0.0	efa:EF1188	K01101	E3.1.3.41	4-nitrophenyl phosphatase	3.1.3.41	Metabolism--Xenobiotics biodegradation and metabolism--Aminobenzoate degradation|Metabolism--Global and overview maps--Microbial metabolism in diverse environments

[denovogenes]_109002	100.00	0.0	stb:SGPB_0270	K09013	sufC	Fe-S cluster assembly ATP-binding protein	--	--

[denovogenes]_109003	99.61	0.0	cif:AL515_05435	K01992	ABC-2.P	ABC-2 type transport system permease protein	--	--

[denovogenes]_109004	83.20	8e-158	pdf:CD630DERM_35390	K03424	--	--	--	--

[denovogenes]_109005	100.00	0.0	ene:ENT_19580	K06889	K06889	uncharacterized protein	--	--

[denovogenes]_109006	47.83	3e-80	tur:AT726_10880	K09861	K09861	uncharacterized protein	--	--

[denovogenes]_109008	73.44	5e-140	cbv:U729_2798	K00806	uppS	undecaprenyl diphosphate synthase	2.5.1.31	Metabolism--Metabolism of terpenoids and polyketides--Terpenoid backbone biosynthesis|Metabolism--Global and overview maps--Biosynthesis of secondary metabolites

[denovogenes]_109009	98.05	0.0	smn:SMA_1931	K03424	--	--	--	--

[denovogenes]_109010	71.49	6e-135	cbt:CLH_0681	K02564	nagB, GNPDA	glucosamine-6-phosphate deaminase	3.5.99.6	Metabolism--Carbohydrate metabolism--Amino sugar and nucleotide sugar metabolism|Metabolism--Global and overview maps--Metabolic pathways

[denovogenes]_109012	99.61	0.0	stb:SGPB_0163	K02530	lacR	DeoR family transcriptional regulator, lactose phosphotransferase system repressor	--	--

[denovogenes]_109013	76.19	1e-128	senc:SEET0819_08360	K11890	impM	type VI secretion system protein ImpM	--	Cellular Processes--Cellular community - prokaryotes--Biofilm formation - Pseudomonas aeruginosa

[denovogenes]_109014	62.30	8e-109	cbv:U729_944	K13292	lgt, umpA	phosphatidylglycerol:prolipoprotein diacylglycerol transferase	2.-.-.-	--

[denovogenes]_109016	99.61	0.0	ecoj:P423_19525	K02013	ABC.FEV.A	iron complex transport system ATP-binding protein	3.6.3.34	Environmental Information Processing--Membrane transport--ABC transporters

[denovogenes]_109019	52.02	2e-85	str:Sterm_0942	K03710	K03710	GntR family transcriptional regulator	--	--

[denovogenes]_10902	100.00	0.0	paer:PA1R_gp5539	K02014	TC.FEV.OM	iron complex outermembrane recepter protein	--	--

[denovogenes]_109020	61.35	4e-110	csh:Closa_1181	K02053	ABC.SP.P	putative spermidine/putrescine transport system permease protein	--	Cellular Processes--Cellular community - prokaryotes--Quorum sensing

[denovogenes]_109021	100.00	3e-169	sga:GALLO_0968	K01591	pyrF	orotidine-5'-phosphate decarboxylase	4.1.1.23	Metabolism--Nucleotide metabolism--Pyrimidine metabolism|Metabolism--Global and overview maps--Metabolic pathways

[denovogenes]_109028	47.35	2e-73	ppu:PP_1289	K09861	K09861	uncharacterized protein	--	--

[denovogenes]_10903	98.53	0.0	bbrc:B7019_0142	K02343	DPO3G, dnaX	DNA polymerase III subunit gamma/tau	2.7.7.7	Metabolism--Nucleotide metabolism--Purine metabolism|Metabolism--Nucleotide metabolism--Pyrimidine metabolism|Metabolism--Global and overview maps--Metabolic pathways|Genetic Information Processing--Replication and repair--DNA replication|Genetic Information Processing--Replication and repair--Mismatch repair|Genetic Information Processing--Replication and repair--Homologous recombination

[denovogenes]_109030	100.00	0.0	pmib:BB2000_2906	K02036	pstB	phosphate transport system ATP-binding protein	3.6.3.27	Environmental Information Processing--Membrane transport--ABC transporters

[denovogenes]_109031	81.64	1e-152	cdg:CDBI1_17410	K03525	coaX	type III pantothenate kinase	2.7.1.33	Metabolism--Metabolism of cofactors and vitamins--Pantothenate and CoA biosynthesis|Metabolism--Global and overview maps--Metabolic pathways

[denovogenes]_109035	95.31	0.0	cama:F384_17225	K02081	agaR	DeoR family transcriptional regulator, aga operon transcriptional repressor	--	--

[denovogenes]_109037	40.16	5e-56	btra:F544_15010	K12984	waaE, kdtX	(heptosyl)LPS beta-1,4-glucosyltransferase	2.4.1.-	--

[denovogenes]_109038	99.22	0.0	cfd:CFNIH1_02860	K01630	garL	2-dehydro-3-deoxyglucarate aldolase	4.1.2.20	Metabolism--Carbohydrate metabolism--Ascorbate and aldarate metabolism

[denovogenes]_109039	100.00	0.0	pvl:AOB99_08345	K14155	patB, malY	cystathione beta-lyase	4.4.1.8	Metabolism--Amino acid metabolism--Cysteine and methionine metabolism|Metabolism--Metabolism of other amino acids--Selenocompound metabolism|Metabolism--Global and overview maps--Metabolic pathways|Metabolism--Global and overview maps--Biosynthesis of secondary metabolites|Metabolism--Global and overview maps--Biosynthesis of amino acids

[denovogenes]_109040	73.62	7e-140	fne:FSDG_00894	K03601	xseA	exodeoxyribonuclease VII large subunit	3.1.11.6	Genetic Information Processing--Replication and repair--Mismatch repair

[denovogenes]_109041	73.41	2e-133	cay:CEA_G3583	K02371	fabK	enoyl-[acyl-carrier protein] reductase II	1.3.1.9	Metabolism--Lipid metabolism--Fatty acid biosynthesis|Metabolism--Global and overview maps--Metabolic pathways|Metabolism--Global and overview maps--Fatty acid metabolism

[denovogenes]_109042	82.10	7e-143	cdg:CDBI1_06125	K01756	purB, ADSL	adenylosuccinate lyase	4.3.2.2	Metabolism--Nucleotide metabolism--Purine metabolism|Metabolism--Amino acid metabolism--Alanine, aspartate and glutamate metabolism|Metabolism--Global and overview maps--Metabolic pathways|Metabolism--Global and overview maps--Biosynthesis of secondary metabolites|Metabolism--Global and overview maps--Biosynthesis of antibiotics

[denovogenes]_109043	51.88	6e-82	cbk:CLL_A0574	K06402	spoIVFB	stage IV sporulation protein FB	3.4.24.-	--

[denovogenes]_109044	100.00	0.0	pvl:AOB99_07790	K02069	ABC.X2.P	putative ABC transport system permease protein	--	--

[denovogenes]_109046	85.94	1e-164	pdf:CD630DERM_35390	K03424	--	--	--	--

[denovogenes]_109047	96.50	1e-170	cif:AL515_12560	K03304	tehA	tellurite resistance protein	--	--

[denovogenes]_109048	69.92	8e-138	puf:UFO1_0740	K07079	K07079	uncharacterized protein	--	--

[denovogenes]_109049	63.92	9e-114	pdf:CD630DERM_14910	K02073	metQ	D-methionine transport system substrate-binding protein	--	Environmental Information Processing--Membrane transport--ABC transporters

[denovogenes]_109050	81.50	1e-149	bwe:BcerKBAB4_2433	K06147	ABCB-BAC	ATP-binding cassette, subfamily B, bacterial	--	--

[denovogenes]_109051	99.61	1e-179	cbut:ATN24_03465	K02746	PTS-Aga-EIIC, agaW	PTS system, N-acetylgalactosamine-specific IIC component	--	Metabolism--Carbohydrate metabolism--Galactose metabolism|Environmental Information Processing--Membrane transport--Phosphotransferase system (PTS)

[denovogenes]_109053	66.55	8e-129	cle:Clole_1347	K00640	cysE	serine O-acetyltransferase	2.3.1.30	Metabolism--Amino acid metabolism--Cysteine and methionine metabolism|Metabolism--Energy metabolism--Sulfur metabolism|Metabolism--Global and overview maps--Metabolic pathways|Metabolism--Global and overview maps--Biosynthesis of secondary metabolites|Metabolism--Global and overview maps--Microbial metabolism in diverse environments|Metabolism--Global and overview maps--Carbon metabolism|Metabolism--Global and overview maps--Biosynthesis of amino acids|Cellular Processes--Cellular community - prokaryotes--Biofilm formation - Vibrio cholerae

[denovogenes]_109054	100.00	8e-176	pvl:AOB99_14050	K03784	deoD	purine-nucleoside phosphorylase	2.4.2.1	Metabolism--Nucleotide metabolism--Purine metabolism|Metabolism--Nucleotide metabolism--Pyrimidine metabolism|Metabolism--Metabolism of cofactors and vitamins--Nicotinate and nicotinamide metabolism|Metabolism--Global and overview maps--Metabolic pathways|Metabolism--Global and overview maps--Biosynthesis of secondary metabolites

[denovogenes]_109055	70.04	1e-131	cst:CLOST_1121	K02057	ABC.SS.P	simple sugar transport system permease protein	--	--

[denovogenes]_109056	100.00	4e-177	pvl:AOB99_11680	K07088	K07088	uncharacterized protein	--	--

[denovogenes]_109057	50.19	5e-77	cbt:CLH_3111	K17758	nnr	ADP-dependent NAD(P)H-hydrate dehydratase	4.2.1.136	--

[denovogenes]_109057	50.19	5e-77	cbt:CLH_3111	K17759	AIBP, nnrE	NAD(P)H-hydrate epimerase	5.1.99.6	--

[denovogenes]_109058	99.22	0.0	cbut:ATN24_06640	K06942	ychF	ribosome-binding ATPase	--	--

[denovogenes]_109059	94.14	7e-171	cif:AL515_07155	K02013	ABC.FEV.A	iron complex transport system ATP-binding protein	3.6.3.34	Environmental Information Processing--Membrane transport--ABC transporters

[denovogenes]_109067	74.02	5e-137	cle:Clole_4263	K03496	parA, soj	chromosome partitioning protein	--	--

[denovogenes]_109068	98.05	0.0	bxy:BXY_22610	K02557	motB	chemotaxis protein MotB	--	Cellular Processes--Cell motility--Bacterial chemotaxis|Cellular Processes--Cell motility--Flagellar assembly

[denovogenes]_109070	89.88	2e-161	prg:RB151_013150	K00800	aroA	3-phosphoshikimate 1-carboxyvinyltransferase	2.5.1.19	Metabolism--Amino acid metabolism--Phenylalanine, tyrosine and tryptophan biosynthesis|Metabolism--Global and overview maps--Metabolic pathways|Metabolism--Global and overview maps--Biosynthesis of secondary metabolites|Metabolism--Global and overview maps--Biosynthesis of antibiotics|Metabolism--Global and overview maps--Biosynthesis of amino acids

[denovogenes]_109073	52.34	4e-89	cbz:Cbs_1755	K01695	trpA	tryptophan synthase alpha chain	4.2.1.20	Metabolism--Amino acid metabolism--Glycine, serine and threonine metabolism|Metabolism--Amino acid metabolism--Phenylalanine, tyrosine and tryptophan biosynthesis|Metabolism--Global and overview maps--Metabolic pathways|Metabolism--Global and overview maps--Biosynthesis of secondary metabolites|Metabolism--Global and overview maps--Biosynthesis of antibiotics|Metabolism--Global and overview maps--Biosynthesis of amino acids

[denovogenes]_109074	59.68	1e-110	let:O77CONTIG1_03336	K03695	clpB	ATP-dependent Clp protease ATP-binding subunit ClpB	--	Organismal Systems--Aging--Longevity regulating pathway - multiple species

[denovogenes]_109076	56.22	2e-84	ddh:Desde_0707	K00548	metH, MTR	5-methyltetrahydrofolate--homocysteine methyltransferase	2.1.1.13	Metabolism--Amino acid metabolism--Cysteine and methionine metabolism|Metabolism--Metabolism of other amino acids--Selenocompound metabolism|Metabolism--Metabolism of cofactors and vitamins--One carbon pool by folate|Metabolism--Global and overview maps--Metabolic pathways|Metabolism--Global and overview maps--Biosynthesis of secondary metabolites|Metabolism--Global and overview maps--Biosynthesis of amino acids

[denovogenes]_109077	100.00	0.0	pvl:AOB99_05380	K02020	modA	molybdate transport system substrate-binding protein	--	Environmental Information Processing--Membrane transport--ABC transporters

[denovogenes]_109079	75.10	1e-129	cbv:U729_1453	K06182	rluF	23S rRNA pseudouridine2604 synthase	5.4.99.21	--

[denovogenes]_109080	80.16	5e-152	prg:RB151_025290	K07637	phoQ	two-component system, OmpR family, sensor histidine kinase PhoQ	2.7.13.3	Human Diseases--Drug resistance: Antimicrobial--Cationic antimicrobial peptide (CAMP) resistance|Environmental Information Processing--Signal transduction--Two-component system

[denovogenes]_109081	72.76	5e-134	hsd:SD1D_0631	K03856	AROA2, aroA	3-deoxy-7-phosphoheptulonate synthase	2.5.1.54	Metabolism--Amino acid metabolism--Phenylalanine, tyrosine and tryptophan biosynthesis|Metabolism--Global and overview maps--Metabolic pathways|Metabolism--Global and overview maps--Biosynthesis of secondary metabolites|Metabolism--Global and overview maps--Biosynthesis of antibiotics|Metabolism--Global and overview maps--Biosynthesis of amino acids

[denovogenes]_109084	99.61	0.0	cfd:CFNIH1_12270	K02301	cyoE	protoheme IX farnesyltransferase	2.5.1.-	Metabolism--Energy metabolism--Oxidative phosphorylation|Metabolism--Metabolism of cofactors and vitamins--Porphyrin and chlorophyll metabolism|Metabolism--Global and overview maps--Metabolic pathways|Metabolism--Global and overview maps--Biosynthesis of secondary metabolites

[denovogenes]_109086	57.81	2e-101	cah:CAETHG_2023	K01776	murI	glutamate racemase	5.1.1.3	Metabolism--Metabolism of other amino acids--D-Glutamine and D-glutamate metabolism|Metabolism--Global and overview maps--Metabolic pathways

[denovogenes]_109087	97.67	0.0	cbut:ATN24_19415	K01308	yqgT	g-D-glutamyl-meso-diaminopimelate peptidase	3.4.19.11	--

[denovogenes]_109088	79.30	5e-147	cbt:CLH_3371	K00027	ME2, sfcA, maeA	malate dehydrogenase (oxaloacetate-decarboxylating)	1.1.1.38	Metabolism--Carbohydrate metabolism--Pyruvate metabolism|Metabolism--Global and overview maps--Carbon metabolism|Environmental Information Processing--Signal transduction--Two-component system

[denovogenes]_109092	68.35	5e-124	csb:CLSA_c04960	K03628	rho	transcription termination factor Rho	--	Genetic Information Processing--Folding, sorting and degradation--RNA degradation

[denovogenes]_109094	45.20	2e-71	xpo:XPG1_0871	K07448	mrr	restriction system protein	--	--

[denovogenes]_109095	51.75	7e-90	aoe:Clos_0311	K02015	ABC.FEV.P	iron complex transport system permease protein	--	Environmental Information Processing--Membrane transport--ABC transporters

[denovogenes]_109098	65.23	6e-112	mhg:MHY_22930	K01872	AARS, alaS	alanyl-tRNA synthetase	6.1.1.7	Genetic Information Processing--Translation--Aminoacyl-tRNA biosynthesis

[denovogenes]_10910	100.00	0.0	pvl:AOB99_11745	K16089	TC.FEV.OM2, cirA, cfrA, hmuR	outer membrane receptor for ferrienterochelin and colicins	--	--

[denovogenes]_109104	75.00	1e-138	ruk:A4V00_18345	K20461	nukG, mcdG, sboG	lantibiotic transport system permease protein	--	Environmental Information Processing--Membrane transport--ABC transporters

[denovogenes]_109105	97.27	0.0	boa:Bovatus_03455	K00677	lpxA	UDP-N-acetylglucosamine acyltransferase	2.3.1.129	Metabolism--Glycan biosynthesis and metabolism--Lipopolysaccharide biosynthesis|Metabolism--Global and overview maps--Metabolic pathways|Human Diseases--Drug resistance: Antimicrobial--Cationic antimicrobial peptide (CAMP) resistance

[denovogenes]_109106	55.38	3e-98	puf:UFO1_2814	K02030	ABC.PA.S	polar amino acid transport system substrate-binding protein	--	--

[denovogenes]_109107	84.58	4e-135	bhl:Bache_1331	K01091	gph	phosphoglycolate phosphatase	3.1.3.18	Metabolism--Carbohydrate metabolism--Glyoxylate and dicarboxylate metabolism|Metabolism--Global and overview maps--Metabolic pathways|Metabolism--Global and overview maps--Biosynthesis of secondary metabolites|Metabolism--Global and overview maps--Biosynthesis of antibiotics

[denovogenes]_109109	56.97	2e-100	rum:CK1_06180	K03525	coaX	type III pantothenate kinase	2.7.1.33	Metabolism--Metabolism of cofactors and vitamins--Pantothenate and CoA biosynthesis|Metabolism--Global and overview maps--Metabolic pathways

[denovogenes]_10911	100.00	0.0	pvl:AOB99_12125	K17686	copA, ATP7	Cu+-exporting ATPase	3.6.3.54	Human Diseases--Drug resistance: Antineoplastic--Platinum drug resistance|Environmental Information Processing--Signal transduction--MAPK signaling pathway - plant

[denovogenes]_109111	42.25	3e-53	hhl:Halha_2012	K07053	E3.1.3.97	3',5'-nucleoside bisphosphate phosphatase	3.1.3.97	--

[denovogenes]_109112	77.73	2e-146	bprl:CL2_17520	K03149	thiG	thiazole synthase	2.8.1.10	Metabolism--Metabolism of cofactors and vitamins--Thiamine metabolism|Metabolism--Global and overview maps--Metabolic pathways

[denovogenes]_109113	62.70	4e-114	pdt:Prede_2711	K19286	nfrA2	FMN reductase [NAD(P)H]	1.5.1.39	Metabolism--Metabolism of cofactors and vitamins--Riboflavin metabolism|Metabolism--Global and overview maps--Metabolic pathways

[denovogenes]_109114	53.85	3e-92	byl:A4V09_05615	K02008	cbiQ	cobalt/nickel transport system permease protein	--	Environmental Information Processing--Membrane transport--ABC transporters

[denovogenes]_109115	75.00	6e-135	bsa:Bacsa_0228	K12343	SRD5A1	3-oxo-5-alpha-steroid 4-dehydrogenase 1	1.3.1.22	Metabolism--Lipid metabolism--Steroid hormone biosynthesis

[denovogenes]_109116	81.42	8e-153	ain:Acin_0137	K01995	livG	branched-chain amino acid transport system ATP-binding protein	--	Environmental Information Processing--Membrane transport--ABC transporters|Cellular Processes--Cellular community - prokaryotes--Quorum sensing

[denovogenes]_109117	45.78	8e-71	pft:JBW_01388	K03524	birA	BirA family transcriptional regulator, biotin operon repressor / biotin-[acetyl-CoA-carboxylase] ligase	6.3.4.15	Metabolism--Metabolism of cofactors and vitamins--Biotin metabolism|Metabolism--Global and overview maps--Metabolic pathways

[denovogenes]_109120	95.70	0.0	bhl:Bache_1889	K02342	DPO3E, dnaQ	DNA polymerase III subunit epsilon	2.7.7.7	Metabolism--Nucleotide metabolism--Purine metabolism|Metabolism--Nucleotide metabolism--Pyrimidine metabolism|Metabolism--Global and overview maps--Metabolic pathways|Genetic Information Processing--Replication and repair--DNA replication|Genetic Information Processing--Replication and repair--Mismatch repair|Genetic Information Processing--Replication and repair--Homologous recombination

[denovogenes]_109121	93.75	3e-180	boa:Bovatus_01222	K01695	trpA	tryptophan synthase alpha chain	4.2.1.20	Metabolism--Amino acid metabolism--Glycine, serine and threonine metabolism|Metabolism--Amino acid metabolism--Phenylalanine, tyrosine and tryptophan biosynthesis|Metabolism--Global and overview maps--Metabolic pathways|Metabolism--Global and overview maps--Biosynthesis of secondary metabolites|Metabolism--Global and overview maps--Biosynthesis of antibiotics|Metabolism--Global and overview maps--Biosynthesis of amino acids

[denovogenes]_109122	76.08	4e-146	era:ERE_11680	K01695	trpA	tryptophan synthase alpha chain	4.2.1.20	Metabolism--Amino acid metabolism--Glycine, serine and threonine metabolism|Metabolism--Amino acid metabolism--Phenylalanine, tyrosine and tryptophan biosynthesis|Metabolism--Global and overview maps--Metabolic pathways|Metabolism--Global and overview maps--Biosynthesis of secondary metabolites|Metabolism--Global and overview maps--Biosynthesis of antibiotics|Metabolism--Global and overview maps--Biosynthesis of amino acids

[denovogenes]_109124	54.10	1e-86	ash:AL1_15900	K12308	bgaB, lacA	beta-galactosidase	3.2.1.23	Metabolism--Carbohydrate metabolism--Galactose metabolism

[denovogenes]_109127	45.34	2e-71	clt:CM240_1779	K01091	gph	phosphoglycolate phosphatase	3.1.3.18	Metabolism--Carbohydrate metabolism--Glyoxylate and dicarboxylate metabolism|Metabolism--Global and overview maps--Metabolic pathways|Metabolism--Global and overview maps--Biosynthesis of secondary metabolites|Metabolism--Global and overview maps--Biosynthesis of antibiotics

[denovogenes]_109129	44.49	8e-75	baco:OXB_1129	K01995	livG	branched-chain amino acid transport system ATP-binding protein	--	Environmental Information Processing--Membrane transport--ABC transporters|Cellular Processes--Cellular community - prokaryotes--Quorum sensing

[denovogenes]_10913	75.26	0.0	fro:AALO17_23070	K00656	E2.3.1.54, pflD	formate C-acetyltransferase	2.3.1.54	Metabolism--Carbohydrate metabolism--Pyruvate metabolism|Metabolism--Carbohydrate metabolism--Propanoate metabolism|Metabolism--Carbohydrate metabolism--Butanoate metabolism|Metabolism--Global and overview maps--Metabolic pathways

[denovogenes]_109133	45.82	9e-80	byl:A4V09_12955	K10823	oppF	oligopeptide transport system ATP-binding protein	--	Human Diseases--Drug resistance: Antimicrobial--beta-Lactam resistance|Environmental Information Processing--Membrane transport--ABC transporters|Cellular Processes--Cellular community - prokaryotes--Quorum sensing

[denovogenes]_109134	71.25	3e-117	fpla:A4U99_05690	K05946	E2.4.1.187, tagA	N-acetylglucosaminyldiphosphoundecaprenol N-acetyl-beta-D-mannosaminyltransferase	2.4.1.187	Cellular Processes--Cellular community - prokaryotes--Biofilm formation - Vibrio cholerae

[denovogenes]_109135	40.16	5e-65	bce:BC1788	K01048	pldB	lysophospholipase	3.1.1.5	Metabolism--Lipid metabolism--Glycerophospholipid metabolism

[denovogenes]_109137	44.09	2e-57	ibu:IB211_00044c	K06897	K06897	7,8-dihydropterin-6-yl-methyl-4-(beta-D-ribofuranosyl)aminobenzene 5'-phosphate synthase	2.5.1.105	--

[denovogenes]_109138	78.91	7e-155	bcel:BcellWH2_05260	K01126	E3.1.4.46, glpQ, ugpQ	glycerophosphoryl diester phosphodiesterase	3.1.4.46	Metabolism--Lipid metabolism--Glycerophospholipid metabolism

[denovogenes]_10914	99.56	0.0	pdi:BDI_3931	K01547	kdpB	K+-transporting ATPase ATPase B chain	3.6.3.12	Environmental Information Processing--Signal transduction--Two-component system

[denovogenes]_109142	53.15	1e-94	cpy:Cphy_1387	K02190	cbiK	sirohydrochlorin cobaltochelatase	4.99.1.3	Metabolism--Metabolism of cofactors and vitamins--Porphyrin and chlorophyll metabolism|Metabolism--Global and overview maps--Metabolic pathways

[denovogenes]_109144	75.40	2e-144	lacy:A4V08_14805	K03424	--	--	--	--

[denovogenes]_109145	47.06	2e-84	csh:Closa_3968	K06608	iolR	DeoR family transcriptional regulator, myo-inositol catabolism operon repressor	--	--

[denovogenes]_109146	65.46	2e-110	fpla:A4U99_12835	K03437	spoU	RNA methyltransferase, TrmH family	--	--

[denovogenes]_10915	100.00	0.0	ecm:EcSMS35_0019	K01811	xylS, yicI	alpha-D-xyloside xylohydrolase	3.2.1.177	--

[denovogenes]_109150	44.22	7e-73	eha:Ethha_2597	K03424	--	--	--	--

[denovogenes]_109151	53.75	1e-99	ava:Ava_2888	K05886	sdh	serine 3-dehydrogenase (NADP+)	1.1.1.276	--

[denovogenes]_109152	100.00	0.0	bxy:BXY_00890	K01695	trpA	tryptophan synthase alpha chain	4.2.1.20	Metabolism--Amino acid metabolism--Glycine, serine and threonine metabolism|Metabolism--Amino acid metabolism--Phenylalanine, tyrosine and tryptophan biosynthesis|Metabolism--Global and overview maps--Metabolic pathways|Metabolism--Global and overview maps--Biosynthesis of secondary metabolites|Metabolism--Global and overview maps--Biosynthesis of antibiotics|Metabolism--Global and overview maps--Biosynthesis of amino acids

[denovogenes]_109153	77.97	4e-132	csh:Closa_3382	K01990	ABC-2.A	ABC-2 type transport system ATP-binding protein	--	--

[denovogenes]_109154	71.60	6e-137	fpla:A4U99_09620	K00858	ppnK, NADK	NAD+ kinase	2.7.1.23	Metabolism--Metabolism of cofactors and vitamins--Nicotinate and nicotinamide metabolism|Metabolism--Global and overview maps--Metabolic pathways

[denovogenes]_109155	41.39	5e-48	cpy:Cphy_0022	K03574	mutT, NUDT15, MTH2	8-oxo-dGTP diphosphatase	3.6.1.55	--

[denovogenes]_109156	69.63	7e-96	rob:CK5_10190	K07023	K07023	putative hydrolases of HD superfamily	--	--

[denovogenes]_109157	84.38	5e-164	fpla:A4U99_07290	K03091	SIG3.4	RNA polymerase sporulation-specific sigma factor	--	--

[denovogenes]_109158	51.75	3e-93	ppq:PPSQR21_043040	K02013	ABC.FEV.A	iron complex transport system ATP-binding protein	3.6.3.34	Environmental Information Processing--Membrane transport--ABC transporters

[denovogenes]_10916	99.85	0.0	pvl:AOB99_10995	K15461	mnmC	tRNA 5-methylaminomethyl-2-thiouridine biosynthesis bifunctional protein	2.1.1.61 1.5.-.-	--

[denovogenes]_109161	72.47	3e-128	fpla:A4U99_03025	K03496	parA, soj	chromosome partitioning protein	--	--

[denovogenes]_109162	98.05	0.0	btho:Btheta7330_02025	K01695	trpA	tryptophan synthase alpha chain	4.2.1.20	Metabolism--Amino acid metabolism--Glycine, serine and threonine metabolism|Metabolism--Amino acid metabolism--Phenylalanine, tyrosine and tryptophan biosynthesis|Metabolism--Global and overview maps--Metabolic pathways|Metabolism--Global and overview maps--Biosynthesis of secondary metabolites|Metabolism--Global and overview maps--Biosynthesis of antibiotics|Metabolism--Global and overview maps--Biosynthesis of amino acids

[denovogenes]_109164	67.19	6e-131	rto:RTO_14990	K03424	--	--	--	--

[denovogenes]_109166	100.00	0.0	btho:Btheta7330_04964	K00677	lpxA	UDP-N-acetylglucosamine acyltransferase	2.3.1.129	Metabolism--Glycan biosynthesis and metabolism--Lipopolysaccharide biosynthesis|Metabolism--Global and overview maps--Metabolic pathways|Human Diseases--Drug resistance: Antimicrobial--Cationic antimicrobial peptide (CAMP) resistance

[denovogenes]_109167	100.00	0.0	shq:A0259_04475	K01992	ABC-2.P	ABC-2 type transport system permease protein	--	--

[denovogenes]_109169	67.05	2e-127	eha:Ethha_2791	K03496	parA, soj	chromosome partitioning protein	--	--

[denovogenes]_10917	100.00	0.0	pvl:AOB99_00975	K03656	rep	ATP-dependent DNA helicase Rep	3.6.4.12	--

[denovogenes]_109170	51.05	7e-86	deh:cbdbA122	K03605	hyaD, hybD	hydrogenase maturation protease	3.4.23.-	--

[denovogenes]_109174	56.18	2e-100	cay:CEA_G1513	K07074	K07074	uncharacterized protein	--	--

[denovogenes]_109175	99.61	0.0	fpla:A4U99_06680	K02013	ABC.FEV.A	iron complex transport system ATP-binding protein	3.6.3.34	Environmental Information Processing--Membrane transport--ABC transporters

[denovogenes]_109176	81.05	6e-153	ibu:IB211_02049	K06173	truA, PUS1	tRNA pseudouridine38-40 synthase	5.4.99.12	--

[denovogenes]_109179	95.70	0.0	bhl:Bache_0201	K02065	mlaF, linL, mkl	phospholipid/cholesterol/gamma-HCH transport system ATP-binding protein	--	Environmental Information Processing--Membrane transport--ABC transporters

[denovogenes]_109180	81.27	4e-151	lacy:A4V08_34865	K07124	K07124	uncharacterized protein	--	--

[denovogenes]_109181	66.53	8e-114	ert:EUR_09480	K03470	rnhB	ribonuclease HII	3.1.26.4	Genetic Information Processing--Replication and repair--DNA replication

[denovogenes]_109183	40.09	2e-45	cbut:ATN24_09745	K15599	thiX	putative hydroxymethylpyrimidine transport system permease protein	--	Environmental Information Processing--Membrane transport--ABC transporters

[denovogenes]_109184	89.80	1e-161	lacy:A4V08_27340	K02049	ABC.SN.A	NitT/TauT family transport system ATP-binding protein	--	--

[denovogenes]_109185	63.41	1e-118	bfr:BF1907	K01607	pcaC	4-carboxymuconolactone decarboxylase	4.1.1.44	Metabolism--Xenobiotics biodegradation and metabolism--Benzoate degradation|Metabolism--Global and overview maps--Metabolic pathways|Metabolism--Global and overview maps--Microbial metabolism in diverse environments|Metabolism--Global and overview maps--Degradation of aromatic compounds

[denovogenes]_109188	96.88	3e-180	spq:SPAB_05150	K03149	thiG	thiazole synthase	2.8.1.10	Metabolism--Metabolism of cofactors and vitamins--Thiamine metabolism|Metabolism--Global and overview maps--Metabolic pathways

[denovogenes]_109189	99.61	0.0	bxy:BXY_26170	K00677	lpxA	UDP-N-acetylglucosamine acyltransferase	2.3.1.129	Metabolism--Glycan biosynthesis and metabolism--Lipopolysaccharide biosynthesis|Metabolism--Global and overview maps--Metabolic pathways|Human Diseases--Drug resistance: Antimicrobial--Cationic antimicrobial peptide (CAMP) resistance

[denovogenes]_109190	70.70	2e-135	ibu:IB211_01535	K03091	SIG3.4	RNA polymerase sporulation-specific sigma factor	--	--

[denovogenes]_109193	74.51	4e-143	ert:EUR_08050	K01695	trpA	tryptophan synthase alpha chain	4.2.1.20	Metabolism--Amino acid metabolism--Glycine, serine and threonine metabolism|Metabolism--Amino acid metabolism--Phenylalanine, tyrosine and tryptophan biosynthesis|Metabolism--Global and overview maps--Metabolic pathways|Metabolism--Global and overview maps--Biosynthesis of secondary metabolites|Metabolism--Global and overview maps--Biosynthesis of antibiotics|Metabolism--Global and overview maps--Biosynthesis of amino acids

[denovogenes]_109194	99.61	0.0	btho:Btheta7330_00426	K02065	mlaF, linL, mkl	phospholipid/cholesterol/gamma-HCH transport system ATP-binding protein	--	Environmental Information Processing--Membrane transport--ABC transporters

[denovogenes]_109195	41.87	2e-38	ral:Rumal_0140	K06967	trmK	tRNA (adenine22-N1)-methyltransferase	2.1.1.217	--

[denovogenes]_109196	93.70	9e-163	cct:CC1_30270	K20461	nukG, mcdG, sboG	lantibiotic transport system permease protein	--	Environmental Information Processing--Membrane transport--ABC transporters

[denovogenes]_109197	99.61	0.0	boa:Bovatus_04541	K01918	panC	pantoate--beta-alanine ligase	6.3.2.1	Metabolism--Metabolism of other amino acids--beta-Alanine metabolism|Metabolism--Metabolism of cofactors and vitamins--Pantothenate and CoA biosynthesis|Metabolism--Global and overview maps--Metabolic pathways|Metabolism--Global and overview maps--Biosynthesis of secondary metabolites

[denovogenes]_109198	89.45	6e-154	cpy:Cphy_0575	K10202	ABC.NGC.P1	N-acetylglucosamine transport system permease protein	--	Environmental Information Processing--Membrane transport--ABC transporters

[denovogenes]_109199	87.71	1e-154	lacy:A4V08_19760	K01448	E3.5.1.28B, amiA, amiB, amiC	N-acetylmuramoyl-L-alanine amidase	3.5.1.28	Human Diseases--Drug resistance: Antimicrobial--Cationic antimicrobial peptide (CAMP) resistance

[denovogenes]_1092	98.49	0.0	fpla:A4U99_11240	K16898	addA	ATP-dependent helicase/nuclease subunit A	3.1.-.- 3.6.4.12	--

[denovogenes]_109201	73.55	7e-129	lacy:A4V08_14760	K01126	E3.1.4.46, glpQ, ugpQ	glycerophosphoryl diester phosphodiesterase	3.1.4.46	Metabolism--Lipid metabolism--Glycerophospholipid metabolism

[denovogenes]_109202	85.10	1e-162	bprm:CL3_18350	K03496	parA, soj	chromosome partitioning protein	--	--

[denovogenes]_109206	54.94	4e-100	ava:Ava_2888	K05886	sdh	serine 3-dehydrogenase (NADP+)	1.1.1.276	--

[denovogenes]_10921	77.93	0.0	ega:AL523_06580	K03655	recG	ATP-dependent DNA helicase RecG	3.6.4.12	Genetic Information Processing--Replication and repair--Homologous recombination

[denovogenes]_109211	56.80	5e-98	eha:Ethha_2597	K03424	--	--	--	--

[denovogenes]_109212	95.70	0.0	cif:AL515_12205	K00019	E1.1.1.30, bdh	3-hydroxybutyrate dehydrogenase	1.1.1.30	Metabolism--Lipid metabolism--Synthesis and degradation of ketone bodies|Metabolism--Carbohydrate metabolism--Butanoate metabolism|Metabolism--Global and overview maps--Metabolic pathways

[denovogenes]_109213	100.00	0.0	bvu:BVU_2946	K01791	wecB	UDP-N-acetylglucosamine 2-epimerase (non-hydrolysing)	5.1.3.14	Metabolism--Carbohydrate metabolism--Amino sugar and nucleotide sugar metabolism|Metabolism--Global and overview maps--Metabolic pathways|Cellular Processes--Cellular community - prokaryotes--Biofilm formation - Vibrio cholerae

[denovogenes]_109214	43.75	1e-76	aoe:Clos_0323	K02190	cbiK	sirohydrochlorin cobaltochelatase	4.99.1.3	Metabolism--Metabolism of cofactors and vitamins--Porphyrin and chlorophyll metabolism|Metabolism--Global and overview maps--Metabolic pathways

[denovogenes]_109215	43.95	3e-55	psu:Psesu_2895	K03670	mdoG	periplasmic glucans biosynthesis protein	--	--

[denovogenes]_109219	92.97	7e-176	bvu:BVU_0547	K01921	ddl	D-alanine-D-alanine ligase	6.3.2.4	Metabolism--Metabolism of other amino acids--D-Alanine metabolism|Metabolism--Glycan biosynthesis and metabolism--Peptidoglycan biosynthesis|Metabolism--Global and overview maps--Metabolic pathways|Human Diseases--Drug resistance: Antimicrobial--Vancomycin resistance

[denovogenes]_109220	97.27	6e-165	fpla:A4U99_01765	K01736	aroC	chorismate synthase	4.2.3.5	Metabolism--Amino acid metabolism--Phenylalanine, tyrosine and tryptophan biosynthesis|Metabolism--Global and overview maps--Metabolic pathways|Metabolism--Global and overview maps--Biosynthesis of secondary metabolites|Metabolism--Global and overview maps--Biosynthesis of antibiotics|Metabolism--Global and overview maps--Biosynthesis of amino acids

[denovogenes]_109221	49.01	2e-81	cct:CC1_06700	K01092	E3.1.3.25, IMPA, suhB	myo-inositol-1(or 4)-monophosphatase	3.1.3.25	Metabolism--Biosynthesis of other secondary metabolites--Streptomycin biosynthesis|Metabolism--Carbohydrate metabolism--Inositol phosphate metabolism|Metabolism--Global and overview maps--Metabolic pathways|Environmental Information Processing--Signal transduction--Phosphatidylinositol signaling system

[denovogenes]_109222	54.38	8e-87	fpla:A4U99_07745	K07284	srtA	sortase A	3.4.22.70	--

[denovogenes]_109224	75.00	3e-135	bprs:CK3_08670	K15554	ssuC	sulfonate transport system permease protein	--	Metabolism--Energy metabolism--Sulfur metabolism|Environmental Information Processing--Membrane transport--ABC transporters

[denovogenes]_109226	85.60	7e-169	sdz:Asd1617_04348	K12111	ebgA	evolved beta-galactosidase subunit alpha	3.2.1.23	Metabolism--Carbohydrate metabolism--Galactose metabolism|Metabolism--Glycan biosynthesis and metabolism--Other glycan degradation|Metabolism--Global and overview maps--Metabolic pathways

[denovogenes]_109227	100.00	0.0	cif:AL515_05435	K01992	ABC-2.P	ABC-2 type transport system permease protein	--	--

[denovogenes]_109228	98.05	3e-173	fpla:A4U99_07355	K07090	K07090	uncharacterized protein	--	--

[denovogenes]_10923	100.00	0.0	efa:EF3113	K03655	recG	ATP-dependent DNA helicase RecG	3.6.4.12	Genetic Information Processing--Replication and repair--Homologous recombination

[denovogenes]_109231	71.76	5e-129	cbz:Cbs_0957	K19508	PTS-Gfr-EIIC, gfrC	PTS system, fructoselysine/glucoselysine-specific IIC component	--	Environmental Information Processing--Membrane transport--Phosphotransferase system (PTS)

[denovogenes]_109232	94.96	1e-168	bcel:BcellWH2_02053	K02003	ABC.CD.A	putative ABC transport system ATP-binding protein	--	--

[denovogenes]_109233	99.22	0.0	cfd:CFNIH1_00910	K06879	queF	7-cyano-7-deazaguanine reductase	1.7.1.13	Metabolism--Metabolism of cofactors and vitamins--Folate biosynthesis|Metabolism--Global and overview maps--Metabolic pathways

[denovogenes]_109234	65.99	8e-128	bfr:BF1907	K01607	pcaC	4-carboxymuconolactone decarboxylase	4.1.1.44	Metabolism--Xenobiotics biodegradation and metabolism--Benzoate degradation|Metabolism--Global and overview maps--Metabolic pathways|Metabolism--Global and overview maps--Microbial metabolism in diverse environments|Metabolism--Global and overview maps--Degradation of aromatic compounds

[denovogenes]_109237	100.00	0.0	ecoa:APECO78_20850	K02170	bioH	pimeloyl-[acyl-carrier protein] methyl ester esterase	3.1.1.85	Metabolism--Metabolism of cofactors and vitamins--Biotin metabolism|Metabolism--Global and overview maps--Metabolic pathways

[denovogenes]_109239	53.85	7e-90	cso:CLS_00950	K06382	spoIIE	stage II sporulation protein E	3.1.3.16	--

[denovogenes]_10924	72.27	0.0	cbv:U729_2832	K03655	recG	ATP-dependent DNA helicase RecG	3.6.4.12	Genetic Information Processing--Replication and repair--Homologous recombination

[denovogenes]_109241	99.61	0.0	ecv:APECO1_1507	K01608	gcl	tartronate-semialdehyde synthase	4.1.1.47	Metabolism--Carbohydrate metabolism--Glyoxylate and dicarboxylate metabolism|Metabolism--Global and overview maps--Metabolic pathways

[denovogenes]_109242	98.44	0.0	cif:AL515_21735	K02170	bioH	pimeloyl-[acyl-carrier protein] methyl ester esterase	3.1.1.85	Metabolism--Metabolism of cofactors and vitamins--Biotin metabolism|Metabolism--Global and overview maps--Metabolic pathways

[denovogenes]_109243	51.36	7e-86	thl:TEH_04730	K01785	galM, GALM	aldose 1-epimerase	5.1.3.3	Metabolism--Carbohydrate metabolism--Glycolysis / Gluconeogenesis|Metabolism--Carbohydrate metabolism--Galactose metabolism|Metabolism--Global and overview maps--Metabolic pathways|Metabolism--Global and overview maps--Biosynthesis of secondary metabolites|Metabolism--Global and overview maps--Microbial metabolism in diverse environments|Metabolism--Global and overview maps--Biosynthesis of antibiotics

[denovogenes]_109244	90.20	1e-163	sdz:Asd1617_00524	K02004	ABC.CD.P	putative ABC transport system permease protein	--	--

[denovogenes]_109245	100.00	1e-177	fpla:A4U99_03285	K02421	fliR	flagellar biosynthetic protein FliR	--	Cellular Processes--Cell motility--Flagellar assembly

[denovogenes]_109247	96.09	0.0	sly:543915	K08235	E2.4.1.207	xyloglucan:xyloglucosyl transferase	2.4.1.207	--

[denovogenes]_10925	94.69	0.0	cif:AL515_02015	K15922	yihQ	sulfoquinovosidase	3.2.1.199	--

[denovogenes]_109250	98.83	0.0	cfd:CFNIH1_15460	K00648	fabH	3-oxoacyl-[acyl-carrier-protein] synthase III	2.3.1.180	Metabolism--Lipid metabolism--Fatty acid biosynthesis|Metabolism--Global and overview maps--Metabolic pathways|Metabolism--Global and overview maps--Fatty acid metabolism

[denovogenes]_109251	99.61	0.0	pdi:BDI_2828	K03685	rnc, DROSHA, RNT1	ribonuclease III	3.1.26.3	Genetic Information Processing--Translation--Ribosome biogenesis in eukaryotes|Human Diseases--Cancers: Overview--Proteoglycans in cancer

[denovogenes]_109252	52.73	5e-92	ibu:IB211_02698c	K01803	TPI, tpiA	triosephosphate isomerase (TIM)	5.3.1.1	Metabolism--Carbohydrate metabolism--Glycolysis / Gluconeogenesis|Metabolism--Carbohydrate metabolism--Fructose and mannose metabolism|Metabolism--Carbohydrate metabolism--Inositol phosphate metabolism|Metabolism--Energy metabolism--Carbon fixation in photosynthetic organisms|Metabolism--Global and overview maps--Metabolic pathways|Metabolism--Global and overview maps--Biosynthesis of secondary metabolites|Metabolism--Global and overview maps--Microbial metabolism in diverse environments|Metabolism--Global and overview maps--Biosynthesis of antibiotics|Metabolism--Global and overview maps--Carbon metabolism|Metabolism--Global and overview maps--Biosynthesis of amino acids

[denovogenes]_109253	65.23	3e-119	cpf:CPF_2854	K01262	pepP	Xaa-Pro aminopeptidase	3.4.11.9	--

[denovogenes]_109254	43.75	5e-71	byl:A4V09_08590	K01992	ABC-2.P	ABC-2 type transport system permease protein	--	--

[denovogenes]_109256	93.75	0.0	bhl:Bache_1538	K06941	rlmN	23S rRNA (adenine2503-C2)-methyltransferase	2.1.1.192	--

[denovogenes]_109257	64.80	2e-116	rch:RUM_09910	K01924	murC	UDP-N-acetylmuramate--alanine ligase	6.3.2.8	Metabolism--Metabolism of other amino acids--D-Glutamine and D-glutamate metabolism|Metabolism--Glycan biosynthesis and metabolism--Peptidoglycan biosynthesis|Metabolism--Global and overview maps--Metabolic pathways

[denovogenes]_109258	82.81	8e-135	cbv:U729_565	K02761	PTS-Cel-EIIC, celB, chbC	PTS system, cellobiose-specific IIC component	--	Metabolism--Carbohydrate metabolism--Starch and sucrose metabolism|Environmental Information Processing--Membrane transport--Phosphotransferase system (PTS)

[denovogenes]_109259	58.53	7e-109	byl:A4V09_03610	K13479	ygeT, xdhB	xanthine dehydrogenase FAD-binding subunit	1.17.1.4	Metabolism--Nucleotide metabolism--Purine metabolism|Metabolism--Global and overview maps--Metabolic pathways|Metabolism--Global and overview maps--Microbial metabolism in diverse environments

[denovogenes]_10926	71.02	0.0	cbv:U729_898	K00700	GBE1, glgB	1,4-alpha-glucan branching enzyme	2.4.1.18	Metabolism--Carbohydrate metabolism--Starch and sucrose metabolism|Metabolism--Global and overview maps--Metabolic pathways|Metabolism--Global and overview maps--Biosynthesis of secondary metabolites

[denovogenes]_109260	92.58	1e-169	lacy:A4V08_13395	K02033	ABC.PE.P	peptide/nickel transport system permease protein	--	Cellular Processes--Cellular community - prokaryotes--Quorum sensing

[denovogenes]_109261	97.66	0.0	cif:AL515_18495	K04765	mazG	nucleoside triphosphate diphosphatase	3.6.1.9	Metabolism--Nucleotide metabolism--Purine metabolism|Metabolism--Nucleotide metabolism--Pyrimidine metabolism|Metabolism--Metabolism of cofactors and vitamins--Nicotinate and nicotinamide metabolism|Metabolism--Metabolism of cofactors and vitamins--Pantothenate and CoA biosynthesis|Metabolism--Global and overview maps--Metabolic pathways

[denovogenes]_109262	66.80	1e-126	baci:B1NLA3E_20295	K01677	E4.2.1.2AA, fumA	fumarate hydratase subunit alpha	4.2.1.2	Metabolism--Carbohydrate metabolism--Citrate cycle (TCA cycle)|Metabolism--Carbohydrate metabolism--Pyruvate metabolism|Metabolism--Energy metabolism--Carbon fixation pathways in prokaryotes|Metabolism--Global and overview maps--Metabolic pathways|Metabolism--Global and overview maps--Biosynthesis of secondary metabolites|Metabolism--Global and overview maps--Microbial metabolism in diverse environments|Metabolism--Global and overview maps--Biosynthesis of antibiotics|Metabolism--Global and overview maps--Carbon metabolism

[denovogenes]_109264	94.94	2e-170	cfd:CFNIH1_14065	K06131	clsA_B	cardiolipin synthase A/B	2.7.8.-	Metabolism--Lipid metabolism--Glycerophospholipid metabolism|Metabolism--Global and overview maps--Metabolic pathways

[denovogenes]_109265	53.52	2e-94	rus:RBI_I01969	K01892	HARS, hisS	histidyl-tRNA synthetase	6.1.1.21	Genetic Information Processing--Translation--Aminoacyl-tRNA biosynthesis

[denovogenes]_109267	42.80	2e-68	dba:Dbac_2248	K00459	ncd2, npd	nitronate monooxygenase	1.13.12.16	Metabolism--Energy metabolism--Nitrogen metabolism

[denovogenes]_109268	59.06	8e-104	cbut:ATN24_15465	K03785	aroD	3-dehydroquinate dehydratase I	4.2.1.10	Metabolism--Amino acid metabolism--Phenylalanine, tyrosine and tryptophan biosynthesis|Metabolism--Global and overview maps--Metabolic pathways|Metabolism--Global and overview maps--Biosynthesis of secondary metabolites|Metabolism--Global and overview maps--Biosynthesis of antibiotics|Metabolism--Global and overview maps--Biosynthesis of amino acids

[denovogenes]_109269	100.00	7e-177	ecoa:APECO78_11315	K10556	lsrC	AI-2 transport system permease protein	--	Environmental Information Processing--Membrane transport--ABC transporters|Cellular Processes--Cellular community - prokaryotes--Quorum sensing

[denovogenes]_10927	99.85	0.0	pvl:AOB99_04960	K08344	scsB	suppressor for copper-sensitivity B	--	--

[denovogenes]_109271	66.15	2e-126	bacc:BRDCF_p614	K03657	uvrD, pcrA	DNA helicase II / ATP-dependent DNA helicase PcrA	3.6.4.12	Genetic Information Processing--Replication and repair--Nucleotide excision repair|Genetic Information Processing--Replication and repair--Mismatch repair

[denovogenes]_109272	41.76	1e-23	pls:VT03_15890	K17758	nnr	ADP-dependent NAD(P)H-hydrate dehydratase	4.2.1.136	--

[denovogenes]_109272	41.76	1e-23	pls:VT03_15890	K17759	AIBP, nnrE	NAD(P)H-hydrate epimerase	5.1.99.6	--

[denovogenes]_109277	42.80	5e-66	dhd:Dhaf_1798	K01465	URA4, pyrC	dihydroorotase	3.5.2.3	Metabolism--Nucleotide metabolism--Pyrimidine metabolism|Metabolism--Global and overview maps--Metabolic pathways

[denovogenes]_10928	100.00	0.0	ecy:ECSE_4161	K15922	yihQ	sulfoquinovosidase	3.2.1.199	--

[denovogenes]_109282	68.48	3e-127	ctc:CTC_00926	K06016	pydC	beta-ureidopropionase / N-carbamoyl-L-amino-acid hydrolase	3.5.1.6 3.5.1.87	Metabolism--Nucleotide metabolism--Pyrimidine metabolism|Metabolism--Global and overview maps--Metabolic pathways

[denovogenes]_109283	67.44	2e-113	cso:CLS_14620	K02035	ABC.PE.S	peptide/nickel transport system substrate-binding protein	--	Cellular Processes--Cellular community - prokaryotes--Quorum sensing

[denovogenes]_109289	76.77	5e-127	pfr:PFREUD_11800	K07150	K07150	uncharacterized protein	--	--

[denovogenes]_10929	97.73	0.0	cfd:CFNIH1_22065	K16089	TC.FEV.OM2, cirA, cfrA, hmuR	outer membrane receptor for ferrienterochelin and colicins	--	--

[denovogenes]_109294	100.00	2e-169	btho:Btheta7330_01038	K14742	tsaB	tRNA threonylcarbamoyladenosine biosynthesis protein TsaB	--	--

[denovogenes]_109295	84.21	5e-132	bcel:BcellWH2_04773	K09702	K09702	uncharacterized protein	--	--

[denovogenes]_109297	40.00	6e-14	aol:S58_31130	K07506	K07506	AraC family transcriptional regulator	--	--

[denovogenes]_109298	85.83	1e-157	dvg:Deval_0805	K02967	RP-S2, MRPS2, rpsB	small subunit ribosomal protein S2	--	Genetic Information Processing--Translation--Ribosome

[denovogenes]_109299	99.22	0.0	cfd:CFNIH1_17885	K00019	E1.1.1.30, bdh	3-hydroxybutyrate dehydrogenase	1.1.1.30	Metabolism--Lipid metabolism--Synthesis and degradation of ketone bodies|Metabolism--Carbohydrate metabolism--Butanoate metabolism|Metabolism--Global and overview maps--Metabolic pathways

[denovogenes]_1093	57.53	0.0	bcel:BcellWH2_03316	K01190	lacZ	beta-galactosidase	3.2.1.23	Metabolism--Carbohydrate metabolism--Galactose metabolism|Metabolism--Glycan biosynthesis and metabolism--Other glycan degradation|Metabolism--Lipid metabolism--Sphingolipid metabolism|Metabolism--Global and overview maps--Metabolic pathways

[denovogenes]_109301	46.09	7e-87	lir:LAW_00311	K03584	recO	DNA repair protein RecO (recombination protein O)	--	Genetic Information Processing--Replication and repair--Homologous recombination

[denovogenes]_109302	100.00	0.0	paeo:M801_5101	K03183	ubiE	demethylmenaquinone methyltransferase / 2-methoxy-6-polyprenyl-1,4-benzoquinol methylase	2.1.1.163 2.1.1.201	Metabolism--Metabolism of cofactors and vitamins--Ubiquinone and other terpenoid-quinone biosynthesis|Metabolism--Global and overview maps--Metabolic pathways|Metabolism--Global and overview maps--Biosynthesis of secondary metabolites

[denovogenes]_109303	41.25	5e-59	dhd:Dhaf_2073	K02510	hpaI, hpcH	4-hydroxy-2-oxoheptanedioate aldolase	4.1.2.52	Metabolism--Amino acid metabolism--Tyrosine metabolism|Metabolism--Global and overview maps--Microbial metabolism in diverse environments

[denovogenes]_109305	100.00	0.0	paeo:M801_4789	K00215	dapB	4-hydroxy-tetrahydrodipicolinate reductase	1.17.1.8	Metabolism--Biosynthesis of other secondary metabolites--Monobactam biosynthesis|Metabolism--Amino acid metabolism--Lysine biosynthesis|Metabolism--Global and overview maps--Metabolic pathways|Metabolism--Global and overview maps--Biosynthesis of secondary metabolites|Metabolism--Global and overview maps--Microbial metabolism in diverse environments|Metabolism--Global and overview maps--Biosynthesis of antibiotics|Metabolism--Global and overview maps--Biosynthesis of amino acids

[denovogenes]_109307	64.68	1e-115	dvg:Deval_0432	K01695	trpA	tryptophan synthase alpha chain	4.2.1.20	Metabolism--Amino acid metabolism--Glycine, serine and threonine metabolism|Metabolism--Amino acid metabolism--Phenylalanine, tyrosine and tryptophan biosynthesis|Metabolism--Global and overview maps--Metabolic pathways|Metabolism--Global and overview maps--Biosynthesis of secondary metabolites|Metabolism--Global and overview maps--Biosynthesis of antibiotics|Metabolism--Global and overview maps--Biosynthesis of amino acids

[denovogenes]_109308	98.44	2e-173	fpla:A4U99_01150	K07090	K07090	uncharacterized protein	--	--

[denovogenes]_109309	51.25	6e-78	cjz:M635_06380	K06152	E1.1.99.3G	gluconate 2-dehydrogenase gamma chain	1.1.99.3	Metabolism--Carbohydrate metabolism--Pentose phosphate pathway|Metabolism--Global and overview maps--Metabolic pathways|Metabolism--Global and overview maps--Microbial metabolism in diverse environments

[denovogenes]_10931	51.18	0.0	fnu:FN0580	K05367	pbpC	penicillin-binding protein 1C	2.4.1.-	Metabolism--Glycan biosynthesis and metabolism--Peptidoglycan biosynthesis

[denovogenes]_109311	99.61	3e-174	paec:M802_3434	K02042	phnE	phosphonate transport system permease protein	--	Environmental Information Processing--Membrane transport--ABC transporters

[denovogenes]_109313	89.45	3e-176	pdi:BDI_0695	K02342	DPO3E, dnaQ	DNA polymerase III subunit epsilon	2.7.7.7	Metabolism--Nucleotide metabolism--Purine metabolism|Metabolism--Nucleotide metabolism--Pyrimidine metabolism|Metabolism--Global and overview maps--Metabolic pathways|Genetic Information Processing--Replication and repair--DNA replication|Genetic Information Processing--Replication and repair--Mismatch repair|Genetic Information Processing--Replication and repair--Homologous recombination

[denovogenes]_109314	54.15	2e-99	ava:Ava_2888	K05886	sdh	serine 3-dehydrogenase (NADP+)	1.1.1.276	--

[denovogenes]_109318	100.00	0.0	paeo:M801_322	K02424	fliY	cystine transport system substrate-binding protein	--	Environmental Information Processing--Membrane transport--ABC transporters

[denovogenes]_109320	100.00	0.0	paeo:M801_5178	K02500	hisF	cyclase	4.1.3.-	Metabolism--Amino acid metabolism--Histidine metabolism|Metabolism--Global and overview maps--Metabolic pathways|Metabolism--Global and overview maps--Biosynthesis of secondary metabolites|Metabolism--Global and overview maps--Biosynthesis of amino acids

[denovogenes]_109322	50.78	1e-84	fpr:FP2_15400	K02823	pyrDII	dihydroorotate dehydrogenase electron transfer subunit	--	Metabolism--Nucleotide metabolism--Pyrimidine metabolism|Metabolism--Global and overview maps--Metabolic pathways

[denovogenes]_109323	43.58	2e-51	tmt:Tmath_0507	K02025	ABC.MS.P	multiple sugar transport system permease protein	--	--

[denovogenes]_109324	48.57	5e-65	rhl:LPU83_pLPU83d1873	K10831	tauB	taurine transport system ATP-binding protein	3.6.3.36	Metabolism--Energy metabolism--Sulfur metabolism|Environmental Information Processing--Membrane transport--ABC transporters

[denovogenes]_109326	98.44	2e-178	fpla:A4U99_14320	K04771	degP, htrA	serine protease Do	3.4.21.107	Human Diseases--Drug resistance: Antimicrobial--Cationic antimicrobial peptide (CAMP) resistance|Environmental Information Processing--Signal transduction--Two-component system

[denovogenes]_109329	100.00	0.0	paeo:M801_1235	K12410	npdA	NAD-dependent deacetylase	3.5.1.-	--

[denovogenes]_109330	82.03	2e-154	dvg:Deval_0308	K00979	kdsB	3-deoxy-manno-octulosonate cytidylyltransferase (CMP-KDO synthetase)	2.7.7.38	Metabolism--Glycan biosynthesis and metabolism--Lipopolysaccharide biosynthesis|Metabolism--Global and overview maps--Metabolic pathways

[denovogenes]_109331	100.00	1e-178	paeu:BN889_03935	K02768	PTS-Fru-EIIA, fruB	PTS system, fructose-specific IIA component	2.7.1.202	Metabolism--Carbohydrate metabolism--Fructose and mannose metabolism|Metabolism--Global and overview maps--Metabolic pathways|Metabolism--Global and overview maps--Microbial metabolism in diverse environments|Environmental Information Processing--Membrane transport--Phosphotransferase system (PTS)

[denovogenes]_109331	100.00	1e-178	paeu:BN889_03935	K08483	PTS-EI.PTSI, ptsI	phosphotransferase system, enzyme I, PtsI	2.7.3.9	Environmental Information Processing--Membrane transport--Phosphotransferase system (PTS)

[denovogenes]_109331	100.00	1e-178	paeu:BN889_03935	K11183	PTS-HPR.FRUB, fruB, fpr	phosphocarrier protein FPr	--	Environmental Information Processing--Membrane transport--Phosphotransferase system (PTS)

[denovogenes]_109332	96.48	0.0	bfb:VU15_00300	K00677	lpxA	UDP-N-acetylglucosamine acyltransferase	2.3.1.129	Metabolism--Glycan biosynthesis and metabolism--Lipopolysaccharide biosynthesis|Metabolism--Global and overview maps--Metabolic pathways|Human Diseases--Drug resistance: Antimicrobial--Cationic antimicrobial peptide (CAMP) resistance

[denovogenes]_109334	63.98	2e-114	clt:CM240_2883	K01990	ABC-2.A	ABC-2 type transport system ATP-binding protein	--	--

[denovogenes]_109336	100.00	0.0	pdi:BDI_0695	K02342	DPO3E, dnaQ	DNA polymerase III subunit epsilon	2.7.7.7	Metabolism--Nucleotide metabolism--Purine metabolism|Metabolism--Nucleotide metabolism--Pyrimidine metabolism|Metabolism--Global and overview maps--Metabolic pathways|Genetic Information Processing--Replication and repair--DNA replication|Genetic Information Processing--Replication and repair--Mismatch repair|Genetic Information Processing--Replication and repair--Homologous recombination

[denovogenes]_109337	44.02	1e-60	aac:Aaci_0337	K05921	hpaG	5-oxopent-3-ene-1,2,5-tricarboxylate decarboxylase / 2-hydroxyhepta-2,4-diene-1,7-dioate isomerase	4.1.1.68 5.3.3.-	Metabolism--Amino acid metabolism--Tyrosine metabolism|Metabolism--Global and overview maps--Microbial metabolism in diverse environments|Metabolism--Global and overview maps--Degradation of aromatic compounds

[denovogenes]_109338	60.08	1e-94	dvl:Dvul_2581	K09688	ABC-2.CPSE.P	capsular polysaccharide transport system permease protein	--	Environmental Information Processing--Membrane transport--ABC transporters

[denovogenes]_10934	99.85	0.0	boa:Bovatus_03937	K07386	pepO	putative endopeptidase	3.4.24.-	--

[denovogenes]_109340	73.05	3e-142	dba:Dbac_3139	K02009	cbiN	cobalt/nickel transport protein	--	Environmental Information Processing--Membrane transport--ABC transporters

[denovogenes]_109341	54.96	7e-88	apo:Arcpr_1847	K01995	livG	branched-chain amino acid transport system ATP-binding protein	--	Environmental Information Processing--Membrane transport--ABC transporters|Cellular Processes--Cellular community - prokaryotes--Quorum sensing

[denovogenes]_109342	99.22	0.0	cfd:CFNIH1_07555	K03149	thiG	thiazole synthase	2.8.1.10	Metabolism--Metabolism of cofactors and vitamins--Thiamine metabolism|Metabolism--Global and overview maps--Metabolic pathways

[denovogenes]_109343	69.09	5e-75	spj:MGAS2096_Spy1232	K02598	nirC	nitrite transporter NirC	--	--

[denovogenes]_109345	46.40	3e-72	nko:Niako_6782	K13002	wbyL	glycosyltransferase	2.4.1.-	--

[denovogenes]_109346	100.00	0.0	cfd:CFNIH1_10370	K01992	ABC-2.P	ABC-2 type transport system permease protein	--	--

[denovogenes]_109347	99.22	0.0	paem:U769_07940	K06167	phnP	phosphoribosyl 1,2-cyclic phosphate phosphodiesterase	3.1.4.55	Metabolism--Metabolism of other amino acids--Phosphonate and phosphinate metabolism

[denovogenes]_109348	99.61	0.0	paeo:M801_2065	K00019	E1.1.1.30, bdh	3-hydroxybutyrate dehydrogenase	1.1.1.30	Metabolism--Lipid metabolism--Synthesis and degradation of ketone bodies|Metabolism--Carbohydrate metabolism--Butanoate metabolism|Metabolism--Global and overview maps--Metabolic pathways

[denovogenes]_109349	69.90	3e-99	ols:Olsu_0038	K11175	purN	phosphoribosylglycinamide formyltransferase 1	2.1.2.2	Metabolism--Nucleotide metabolism--Purine metabolism|Metabolism--Metabolism of cofactors and vitamins--One carbon pool by folate|Metabolism--Global and overview maps--Metabolic pathways|Metabolism--Global and overview maps--Biosynthesis of secondary metabolites|Metabolism--Global and overview maps--Biosynthesis of antibiotics

[denovogenes]_109350	100.00	0.0	paf:PAM18_3036	K00451	HGD, hmgA	homogentisate 1,2-dioxygenase	1.13.11.5	Metabolism--Amino acid metabolism--Tyrosine metabolism|Metabolism--Xenobiotics biodegradation and metabolism--Styrene degradation|Metabolism--Global and overview maps--Metabolic pathways|Metabolism--Global and overview maps--Microbial metabolism in diverse environments

[denovogenes]_109351	100.00	0.0	paeo:M801_2305	K20987	pslD	polysaccharide biosynthesis/export protein PslD	--	Cellular Processes--Cellular community - prokaryotes--Biofilm formation - Pseudomonas aeruginosa

[denovogenes]_109352	99.55	2e-159	fpla:A4U99_01050	K02029	ABC.PA.P	polar amino acid transport system permease protein	--	--

[denovogenes]_109354	41.03	5e-17	fbt:D770_07925	K03183	ubiE	demethylmenaquinone methyltransferase / 2-methoxy-6-polyprenyl-1,4-benzoquinol methylase	2.1.1.163 2.1.1.201	Metabolism--Metabolism of cofactors and vitamins--Ubiquinone and other terpenoid-quinone biosynthesis|Metabolism--Global and overview maps--Metabolic pathways|Metabolism--Global and overview maps--Biosynthesis of secondary metabolites

[denovogenes]_109356	99.61	0.0	bfb:VU15_18410	K02065	mlaF, linL, mkl	phospholipid/cholesterol/gamma-HCH transport system ATP-binding protein	--	Environmental Information Processing--Membrane transport--ABC transporters

[denovogenes]_109357	93.75	0.0	lacy:A4V08_19565	K07160	K07160	UPF0271 protein	--	--

[denovogenes]_109358	99.22	0.0	lacy:A4V08_20690	K03784	deoD	purine-nucleoside phosphorylase	2.4.2.1	Metabolism--Nucleotide metabolism--Purine metabolism|Metabolism--Nucleotide metabolism--Pyrimidine metabolism|Metabolism--Metabolism of cofactors and vitamins--Nicotinate and nicotinamide metabolism|Metabolism--Global and overview maps--Metabolic pathways|Metabolism--Global and overview maps--Biosynthesis of secondary metabolites

[denovogenes]_109359	48.83	6e-71	bja:bll1082	K00065	kduD	2-deoxy-D-gluconate 3-dehydrogenase	1.1.1.125	Metabolism--Carbohydrate metabolism--Pentose and glucuronate interconversions

[denovogenes]_109360	40.86	8e-56	spe:Spro_1104	K11938	cof	HMP-PP phosphatase	3.6.1.-	--

[denovogenes]_109361	94.14	1e-177	lacy:A4V08_34485	K09861	K09861	uncharacterized protein	--	--

[denovogenes]_109364	100.00	0.0	pdi:BDI_3366	K01929	murF	UDP-N-acetylmuramoyl-tripeptide--D-alanyl-D-alanine ligase	6.3.2.10	Metabolism--Amino acid metabolism--Lysine biosynthesis|Metabolism--Glycan biosynthesis and metabolism--Peptidoglycan biosynthesis|Metabolism--Global and overview maps--Metabolic pathways|Human Diseases--Drug resistance: Antimicrobial--Vancomycin resistance

[denovogenes]_109365	55.64	2e-80	gpa:GPA_29370	K02035	ABC.PE.S	peptide/nickel transport system substrate-binding protein	--	Cellular Processes--Cellular community - prokaryotes--Quorum sensing

[denovogenes]_109366	84.38	5e-165	dvg:Deval_0100	K00978	rfbF	glucose-1-phosphate cytidylyltransferase	2.7.7.33	Metabolism--Carbohydrate metabolism--Starch and sucrose metabolism|Metabolism--Carbohydrate metabolism--Amino sugar and nucleotide sugar metabolism|Metabolism--Global and overview maps--Metabolic pathways

[denovogenes]_109367	95.31	1e-178	ruk:A4V00_15665	K03496	parA, soj	chromosome partitioning protein	--	--

[denovogenes]_109368	79.69	4e-146	cgo:Corgl_1133	K09013	sufC	Fe-S cluster assembly ATP-binding protein	--	--

[denovogenes]_109369	100.00	0.0	paec:M802_2544	K03805	dsbG	thiol:disulfide interchange protein DsbG	--	--

[denovogenes]_10937	99.56	0.0	bxy:BXY_15220	K11381	bkdA	2-oxoisovalerate dehydrogenase E1 component	1.2.4.4	Metabolism--Amino acid metabolism--Valine, leucine and isoleucine degradation|Metabolism--Carbohydrate metabolism--Propanoate metabolism|Metabolism--Global and overview maps--Metabolic pathways|Metabolism--Global and overview maps--Biosynthesis of secondary metabolites|Metabolism--Global and overview maps--Biosynthesis of antibiotics

[denovogenes]_109370	49.58	2e-75	lfu:HR49_22030	K00571	E2.1.1.72	site-specific DNA-methyltransferase (adenine-specific)	2.1.1.72	--

[denovogenes]_109371	66.93	2e-129	gpa:GPA_12800	K03217	yidC, spoIIIJ, OXA1, ccfA	YidC/Oxa1 family membrane protein insertase	--	Cellular Processes--Cellular community - prokaryotes--Quorum sensing|Genetic Information Processing--Folding, sorting and degradation--Protein export|Environmental Information Processing--Membrane transport--Bacterial secretion system

[denovogenes]_109372	79.92	8e-155	pdi:BDI_0384	K00677	lpxA	UDP-N-acetylglucosamine acyltransferase	2.3.1.129	Metabolism--Glycan biosynthesis and metabolism--Lipopolysaccharide biosynthesis|Metabolism--Global and overview maps--Metabolic pathways|Human Diseases--Drug resistance: Antimicrobial--Cationic antimicrobial peptide (CAMP) resistance

[denovogenes]_109373	84.05	2e-160	pdi:BDI_1113	K02823	pyrDII	dihydroorotate dehydrogenase electron transfer subunit	--	Metabolism--Nucleotide metabolism--Pyrimidine metabolism|Metabolism--Global and overview maps--Metabolic pathways

[denovogenes]_109374	96.48	7e-180	lacy:A4V08_26430	K01883	CARS, cysS	cysteinyl-tRNA synthetase	6.1.1.16	Genetic Information Processing--Translation--Aminoacyl-tRNA biosynthesis

[denovogenes]_109375	92.83	4e-175	bfb:VU15_11940	K01695	trpA	tryptophan synthase alpha chain	4.2.1.20	Metabolism--Amino acid metabolism--Glycine, serine and threonine metabolism|Metabolism--Amino acid metabolism--Phenylalanine, tyrosine and tryptophan biosynthesis|Metabolism--Global and overview maps--Metabolic pathways|Metabolism--Global and overview maps--Biosynthesis of secondary metabolites|Metabolism--Global and overview maps--Biosynthesis of antibiotics|Metabolism--Global and overview maps--Biosynthesis of amino acids

[denovogenes]_109377	40.71	1e-50	fpsz:AMR72_15635	K18009	budC	meso-butanediol dehydrogenase / (S,S)-butanediol dehydrogenase / diacetyl reductase	1.1.1.- 1.1.1.76 1.1.1.304	Metabolism--Carbohydrate metabolism--Butanoate metabolism

[denovogenes]_109378	51.00	6e-87	cbt:CLH_1958	K03481	glvR	RpiR family transcriptional regulator, glv operon transcriptional regulator	--	--

[denovogenes]_10938	79.74	0.0	csh:Closa_2493	K00611	OTC, argF, argI	ornithine carbamoyltransferase	2.1.3.3	Metabolism--Amino acid metabolism--Arginine biosynthesis|Metabolism--Global and overview maps--Metabolic pathways|Metabolism--Global and overview maps--Biosynthesis of secondary metabolites|Metabolism--Global and overview maps--Biosynthesis of antibiotics|Metabolism--Global and overview maps--Biosynthesis of amino acids

[denovogenes]_109382	100.00	0.0	cfd:CFNIH1_23205	K07320	prmB	ribosomal protein L3 glutamine methyltransferase	2.1.1.298	--

[denovogenes]_109385	41.14	3e-32	dmc:btf_1027	K07658	phoB1, phoP	two-component system, OmpR family, alkaline phosphatase synthesis response regulator PhoP	--	Environmental Information Processing--Signal transduction--Two-component system

[denovogenes]_109390	100.00	1e-180	paeo:M801_3413	K01692	paaF, echA	enoyl-CoA hydratase	4.2.1.17	Metabolism--Lipid metabolism--Fatty acid degradation|Metabolism--Amino acid metabolism--Valine, leucine and isoleucine degradation|Metabolism--Metabolism of terpenoids and polyketides--Geraniol degradation|Metabolism--Amino acid metabolism--Lysine degradation|Metabolism--Amino acid metabolism--Phenylalanine metabolism|Metabolism--Xenobiotics biodegradation and metabolism--Benzoate degradation|Metabolism--Amino acid metabolism--Tryptophan metabolism|Metabolism--Metabolism of other amino acids--beta-Alanine metabolism|Metabolism--Xenobiotics biodegradation and metabolism--Aminobenzoate degradation|Metabolism--Carbohydrate metabolism--Propanoate metabolism|Metabolism--Carbohydrate metabolism--Butanoate metabolism|Metabolism--Metabolism of terpenoids and polyketides--Limonene and pinene degradation|Metabolism--Xenobiotics biodegradation and metabolism--Caprolactam degradation|Metabolism--Global and overview maps--Metabolic pathways|Metabolism--Global and overview maps--Biosynthesis of secondary metabolites|Metabolism--Global and overview maps--Microbial metabolism in diverse environments|Metabolism--Global and overview maps--Biosynthesis of antibiotics|Metabolism--Global and overview maps--Fatty acid metabolism

[denovogenes]_109391	79.28	2e-150	pdi:BDI_0046	K07011	K07011	uncharacterized protein	--	--

[denovogenes]_109395	54.96	1e-90	lir:LAW_00056	K02049	ABC.SN.A	NitT/TauT family transport system ATP-binding protein	--	--

[denovogenes]_109397	63.42	5e-103	cls:CXIVA_20710	K05568	mnhD, mrpD	multicomponent Na+:H+ antiporter subunit D	--	--

[denovogenes]_109398	98.83	0.0	cfd:CFNIH1_04720	K02170	bioH	pimeloyl-[acyl-carrier protein] methyl ester esterase	3.1.1.85	Metabolism--Metabolism of cofactors and vitamins--Biotin metabolism|Metabolism--Global and overview maps--Metabolic pathways

[denovogenes]_10940	92.18	0.0	bcel:BcellWH2_03587	K11381	bkdA	2-oxoisovalerate dehydrogenase E1 component	1.2.4.4	Metabolism--Amino acid metabolism--Valine, leucine and isoleucine degradation|Metabolism--Carbohydrate metabolism--Propanoate metabolism|Metabolism--Global and overview maps--Metabolic pathways|Metabolism--Global and overview maps--Biosynthesis of secondary metabolites|Metabolism--Global and overview maps--Biosynthesis of antibiotics

[denovogenes]_109403	80.16	2e-149	ere:EUBREC_2884	K17318	K17318, lplA	putative aldouronate transport system substrate-binding protein	--	Environmental Information Processing--Membrane transport--ABC transporters

[denovogenes]_109405	88.24	2e-162	boa:Bovatus_03532	K00930	argB	acetylglutamate kinase	2.7.2.8	Metabolism--Amino acid metabolism--Arginine biosynthesis|Metabolism--Global and overview maps--Metabolic pathways|Metabolism--Global and overview maps--Biosynthesis of secondary metabolites|Metabolism--Global and overview maps--Biosynthesis of antibiotics|Metabolism--Global and overview maps--2-Oxocarboxylic acid metabolism|Metabolism--Global and overview maps--Biosynthesis of amino acids

[denovogenes]_109406	79.69	8e-152	coo:CCU_03990	K00789	metK	S-adenosylmethionine synthetase	2.5.1.6	Metabolism--Amino acid metabolism--Cysteine and methionine metabolism|Metabolism--Global and overview maps--Metabolic pathways|Metabolism--Global and overview maps--Biosynthesis of secondary metabolites|Metabolism--Global and overview maps--Biosynthesis of amino acids

[denovogenes]_109407	100.00	0.0	cif:AL515_13160	K06178	rluB	23S rRNA pseudouridine2605 synthase	5.4.99.22	--

[denovogenes]_109408	57.14	5e-91	psa:PST_2542	K10943	flrC, fleR	two-component system, response regulator FlrC	--	Environmental Information Processing--Signal transduction--Two-component system|Cellular Processes--Cellular community - prokaryotes--Biofilm formation - Vibrio cholerae

[denovogenes]_109409	63.60	5e-109	cpro:CPRO_02520	K03321	TC.SULP	sulfate permease, SulP family	--	--

[denovogenes]_10941	99.71	0.0	bvu:BVU_2033	K07386	pepO	putative endopeptidase	3.4.24.-	--

[denovogenes]_109410	100.00	0.0	pdf:CD630DERM_04790	K20491	nisE, spaE, cprB, epiE	lantibiotic transport system permease protein	--	Environmental Information Processing--Membrane transport--ABC transporters|Environmental Information Processing--Signal transduction--Two-component system|Cellular Processes--Cellular community - prokaryotes--Quorum sensing

[denovogenes]_109411	99.61	0.0	sbo:SBO_2991	K01630	garL	2-dehydro-3-deoxyglucarate aldolase	4.1.2.20	Metabolism--Carbohydrate metabolism--Ascorbate and aldarate metabolism

[denovogenes]_109412	77.82	2e-146	byl:A4V09_16865	K02529	lacI, galR	LacI family transcriptional regulator	--	--

[denovogenes]_109413	99.61	0.0	cfd:CFNIH1_23070	K07071	K07071	uncharacterized protein	--	--

[denovogenes]_109414	65.88	3e-120	eyy:EGYY_02770	K01482	DDAH, ddaH	dimethylargininase	3.5.3.18	--

[denovogenes]_109415	70.38	2e-128	bcel:BcellWH2_01510	K13963	SERPINB	serpin B	--	Human Diseases--Infectious diseases: Parasitic--Amoebiasis

[denovogenes]_109416	47.47	3e-76	ruk:A4V00_04340	K03525	coaX	type III pantothenate kinase	2.7.1.33	Metabolism--Metabolism of cofactors and vitamins--Pantothenate and CoA biosynthesis|Metabolism--Global and overview maps--Metabolic pathways

[denovogenes]_109417	40.32	4e-67	coo:CCU_22130	K00595	cobL	precorrin-6Y C5,15-methyltransferase (decarboxylating)	2.1.1.132	Metabolism--Metabolism of cofactors and vitamins--Porphyrin and chlorophyll metabolism|Metabolism--Global and overview maps--Metabolic pathways

[denovogenes]_109418	100.00	0.0	pdi:BDI_1609	K03146	THI4, THI1	thiamine thiazole synthase	--	Metabolism--Metabolism of cofactors and vitamins--Thiamine metabolism|Metabolism--Global and overview maps--Metabolic pathways

[denovogenes]_109420	68.36	2e-129	shi:Shel_16050	K00133	asd	aspartate-semialdehyde dehydrogenase	1.2.1.11	Metabolism--Amino acid metabolism--Glycine, serine and threonine metabolism|Metabolism--Biosynthesis of other secondary metabolites--Monobactam biosynthesis|Metabolism--Amino acid metabolism--Cysteine and methionine metabolism|Metabolism--Amino acid metabolism--Lysine biosynthesis|Metabolism--Global and overview maps--Metabolic pathways|Metabolism--Global and overview maps--Biosynthesis of secondary metabolites|Metabolism--Global and overview maps--Microbial metabolism in diverse environments|Metabolism--Global and overview maps--Biosynthesis of antibiotics|Metabolism--Global and overview maps--2-Oxocarboxylic acid metabolism|Metabolism--Global and overview maps--Biosynthesis of amino acids

[denovogenes]_109421	81.25	2e-154	pdi:BDI_1244	K03284	corA	magnesium transporter	--	--

[denovogenes]_109422	83.46	7e-156	cls:CXIVA_17290	K03149	thiG	thiazole synthase	2.8.1.10	Metabolism--Metabolism of cofactors and vitamins--Thiamine metabolism|Metabolism--Global and overview maps--Metabolic pathways

[denovogenes]_109426	78.52	2e-148	pdi:BDI_1822	K07387	K07387	putative metalloprotease	3.4.24.-	--

[denovogenes]_109427	92.58	6e-173	csh:Closa_4291	K03496	parA, soj	chromosome partitioning protein	--	--

[denovogenes]_109431	99.61	0.0	etw:ECSP_4969	K02856	rhaT	L-rhamnose-H+ transport protein	--	--

[denovogenes]_109432	70.87	2e-129	shi:Shel_10130	K01803	TPI, tpiA	triosephosphate isomerase (TIM)	5.3.1.1	Metabolism--Carbohydrate metabolism--Glycolysis / Gluconeogenesis|Metabolism--Carbohydrate metabolism--Fructose and mannose metabolism|Metabolism--Carbohydrate metabolism--Inositol phosphate metabolism|Metabolism--Energy metabolism--Carbon fixation in photosynthetic organisms|Metabolism--Global and overview maps--Metabolic pathways|Metabolism--Global and overview maps--Biosynthesis of secondary metabolites|Metabolism--Global and overview maps--Microbial metabolism in diverse environments|Metabolism--Global and overview maps--Biosynthesis of antibiotics|Metabolism--Global and overview maps--Carbon metabolism|Metabolism--Global and overview maps--Biosynthesis of amino acids

[denovogenes]_109433	81.71	1e-148	bsa:Bacsa_3602	K02371	fabK	enoyl-[acyl-carrier protein] reductase II	1.3.1.9	Metabolism--Lipid metabolism--Fatty acid biosynthesis|Metabolism--Global and overview maps--Metabolic pathways|Metabolism--Global and overview maps--Fatty acid metabolism

[denovogenes]_109438	95.72	0.0	ash:AL1_24060	K00134	GAPDH, gapA	glyceraldehyde 3-phosphate dehydrogenase	1.2.1.12	Metabolism--Carbohydrate metabolism--Glycolysis / Gluconeogenesis|Metabolism--Energy metabolism--Carbon fixation in photosynthetic organisms|Metabolism--Global and overview maps--Metabolic pathways|Metabolism--Global and overview maps--Biosynthesis of secondary metabolites|Metabolism--Global and overview maps--Microbial metabolism in diverse environments|Metabolism--Global and overview maps--Biosynthesis of antibiotics|Metabolism--Global and overview maps--Carbon metabolism|Metabolism--Global and overview maps--Biosynthesis of amino acids|Environmental Information Processing--Signal transduction--HIF-1 signaling pathway|Human Diseases--Neurodegenerative diseases--Alzheimer's disease

[denovogenes]_10944	76.00	0.0	cpy:Cphy_0326	K03798	ftsH, hflB	cell division protease FtsH	3.4.24.-	--

[denovogenes]_109442	87.45	6e-160	csh:Closa_0085	K15986	ppaC	manganese-dependent inorganic pyrophosphatase	3.6.1.1	Metabolism--Energy metabolism--Oxidative phosphorylation

[denovogenes]_109444	63.39	4e-114	cjy:QZ67_00504	K02355	fusA, GFM, EFG	elongation factor G	--	--

[denovogenes]_109445	82.42	2e-159	csh:Closa_0444	K01424	E3.5.1.1, ansA, ansB	L-asparaginase	3.5.1.1	Metabolism--Amino acid metabolism--Alanine, aspartate and glutamate metabolism|Metabolism--Metabolism of other amino acids--Cyanoamino acid metabolism|Metabolism--Global and overview maps--Metabolic pathways|Metabolism--Global and overview maps--Biosynthesis of secondary metabolites

[denovogenes]_109446	54.62	4e-85	csh:Closa_2155	K02005	ABC.CD.TX	HlyD family secretion protein	--	--

[denovogenes]_109447	98.05	0.0	cfd:CFNIH1_03890	K02805	wecE, rffA	dTDP-4-amino-4,6-dideoxygalactose transaminase	2.6.1.59	--

[denovogenes]_109448	96.89	0.0	bbrd:BBBR_0805	K00688	PYG, glgP	glycogen phosphorylase	2.4.1.1	Metabolism--Carbohydrate metabolism--Starch and sucrose metabolism|Metabolism--Global and overview maps--Metabolic pathways|Metabolism--Global and overview maps--Biosynthesis of secondary metabolites|Cellular Processes--Cellular community - prokaryotes--Biofilm formation - Escherichia coli|Organismal Systems--Endocrine system--Insulin signaling pathway|Organismal Systems--Endocrine system--Glucagon signaling pathway|Human Diseases--Endocrine and metabolic diseases--Insulin resistance

[denovogenes]_10945	87.61	0.0	bdh:GV66_10460	K07386	pepO	putative endopeptidase	3.4.24.-	--

[denovogenes]_109451	100.00	8e-180	cfd:CFNIH1_04910	K01997	livH	branched-chain amino acid transport system permease protein	--	Environmental Information Processing--Membrane transport--ABC transporters|Cellular Processes--Cellular community - prokaryotes--Quorum sensing

[denovogenes]_109452	67.26	2e-100	olo:ADJ70_11585	K03654	recQ	ATP-dependent DNA helicase RecQ	3.6.4.12	Genetic Information Processing--Folding, sorting and degradation--RNA degradation

[denovogenes]_109453	86.51	1e-157	cpy:Cphy_0553	K01876	DARS, aspS	aspartyl-tRNA synthetase	6.1.1.12	Genetic Information Processing--Translation--Aminoacyl-tRNA biosynthesis

[denovogenes]_109454	100.00	0.0	cfd:CFNIH1_10105	K03435	fruR1, fruR	LacI family transcriptional regulator, fructose operon transcriptional repressor	--	--

[denovogenes]_109456	82.75	2e-148	bhl:Bache_2256	K02227	cbiB, cobD	adenosylcobinamide-phosphate synthase	6.3.1.10	Metabolism--Metabolism of cofactors and vitamins--Porphyrin and chlorophyll metabolism|Metabolism--Global and overview maps--Metabolic pathways

[denovogenes]_109459	43.43	7e-41	aah:CF65_00932	K10439	rbsB	ribose transport system substrate-binding protein	--	Environmental Information Processing--Membrane transport--ABC transporters|Cellular Processes--Cell motility--Bacterial chemotaxis

[denovogenes]_10946	50.97	0.0	sli:Slin_4849	K01338	lon	ATP-dependent Lon protease	3.4.21.53	Cellular Processes--Cell growth and death--Cell cycle - Caulobacter

[denovogenes]_109463	99.61	0.0	bbrj:B7017_0146	K00059	fabG	3-oxoacyl-[acyl-carrier protein] reductase	1.1.1.100	Metabolism--Lipid metabolism--Fatty acid biosynthesis|Metabolism--Metabolism of cofactors and vitamins--Biotin metabolism|Metabolism--Lipid metabolism--Biosynthesis of unsaturated fatty acids|Metabolism--Global and overview maps--Metabolic pathways|Metabolism--Global and overview maps--Fatty acid metabolism

[denovogenes]_109464	70.63	2e-122	rim:ROI_16870	K17234	--	--	--	Environmental Information Processing--Membrane transport--ABC transporters

[denovogenes]_109465	40.34	2e-34	hro:HELRODRAFT_86412	K17286	STOM	erythrocyte band 7 integral membrane protein	--	--

[denovogenes]_109469	95.33	0.0	pdi:BDI_3565	K03340	dapdh	diaminopimelate dehydrogenase	1.4.1.16	Metabolism--Amino acid metabolism--Lysine biosynthesis|Metabolism--Global and overview maps--Metabolic pathways|Metabolism--Global and overview maps--Biosynthesis of secondary metabolites|Metabolism--Global and overview maps--Biosynthesis of amino acids

[denovogenes]_10947	99.71	0.0	btho:Btheta7330_03065	K11381	bkdA	2-oxoisovalerate dehydrogenase E1 component	1.2.4.4	Metabolism--Amino acid metabolism--Valine, leucine and isoleucine degradation|Metabolism--Carbohydrate metabolism--Propanoate metabolism|Metabolism--Global and overview maps--Metabolic pathways|Metabolism--Global and overview maps--Biosynthesis of secondary metabolites|Metabolism--Global and overview maps--Biosynthesis of antibiotics

[denovogenes]_109471	100.00	0.0	cfd:CFNIH1_11625	K09470	puuA	gamma-glutamylputrescine synthase	6.3.1.11	Metabolism--Amino acid metabolism--Arginine and proline metabolism|Metabolism--Global and overview maps--Metabolic pathways

[denovogenes]_109472	100.00	0.0	pdf:CD630DERM_01000	K16786	ecfA1	energy-coupling factor transport system ATP-binding protein	3.6.3.-	Environmental Information Processing--Membrane transport--ABC transporters

[denovogenes]_109473	68.59	7e-136	mhg:MHY_16560	K19115	csh2	CRISPR-associated protein Csh2	--	--

[denovogenes]_109474	99.61	0.0	cfd:CFNIH1_07220	K02446	glpX	fructose-1,6-bisphosphatase II	3.1.3.11	Metabolism--Carbohydrate metabolism--Glycolysis / Gluconeogenesis|Metabolism--Carbohydrate metabolism--Pentose phosphate pathway|Metabolism--Carbohydrate metabolism--Fructose and mannose metabolism|Metabolism--Energy metabolism--Methane metabolism|Metabolism--Energy metabolism--Carbon fixation in photosynthetic organisms|Metabolism--Global and overview maps--Metabolic pathways|Metabolism--Global and overview maps--Biosynthesis of secondary metabolites|Metabolism--Global and overview maps--Microbial metabolism in diverse environments|Metabolism--Global and overview maps--Biosynthesis of antibiotics|Metabolism--Global and overview maps--Carbon metabolism

[denovogenes]_109476	41.63	5e-65	pef:A7E78_06365	K04069	pflA, pflC, pflE	pyruvate formate lyase activating enzyme	1.97.1.4	--

[denovogenes]_109478	100.00	0.0	cdg:CDBI1_16480	K00873	PK, pyk	pyruvate kinase	2.7.1.40	Metabolism--Carbohydrate metabolism--Glycolysis / Gluconeogenesis|Metabolism--Nucleotide metabolism--Purine metabolism|Metabolism--Carbohydrate metabolism--Pyruvate metabolism|Metabolism--Global and overview maps--Metabolic pathways|Metabolism--Global and overview maps--Biosynthesis of secondary metabolites|Metabolism--Global and overview maps--Microbial metabolism in diverse environments|Metabolism--Global and overview maps--Biosynthesis of antibiotics|Metabolism--Global and overview maps--Carbon metabolism|Metabolism--Global and overview maps--Biosynthesis of amino acids|Organismal Systems--Endocrine system--Glucagon signaling pathway|Human Diseases--Endocrine and metabolic diseases--Type II diabetes mellitus|Human Diseases--Cancers: Overview--Viral carcinogenesis|Human Diseases--Cancers: Overview--Central carbon metabolism in cancer

[denovogenes]_10948	100.00	0.0	btho:Btheta7330_04477	K07386	pepO	putative endopeptidase	3.4.24.-	--

[denovogenes]_109480	70.04	9e-131	pdi:BDI_1052	K15727	czcB	membrane fusion protein, cobalt-zinc-cadmium efflux system	--	--

[denovogenes]_109483	75.38	1e-107	cso:CLS_28060	K02469	gyrA	DNA gyrase subunit A	5.99.1.3	--

[denovogenes]_109484	97.67	0.0	pmib:BB2000_0211	K01119	cpdB	2',3'-cyclic-nucleotide 2'-phosphodiesterase / 3'-nucleotidase	3.1.4.16 3.1.3.6	Metabolism--Nucleotide metabolism--Purine metabolism|Metabolism--Nucleotide metabolism--Pyrimidine metabolism

[denovogenes]_109485	61.09	1e-92	cbac:JI75_08755	K01835	pgm	phosphoglucomutase	5.4.2.2	Metabolism--Carbohydrate metabolism--Glycolysis / Gluconeogenesis|Metabolism--Carbohydrate metabolism--Pentose phosphate pathway|Metabolism--Carbohydrate metabolism--Galactose metabolism|Metabolism--Nucleotide metabolism--Purine metabolism|Metabolism--Carbohydrate metabolism--Starch and sucrose metabolism|Metabolism--Carbohydrate metabolism--Amino sugar and nucleotide sugar metabolism|Metabolism--Biosynthesis of other secondary metabolites--Streptomycin biosynthesis|Metabolism--Global and overview maps--Metabolic pathways|Metabolism--Global and overview maps--Biosynthesis of secondary metabolites|Metabolism--Global and overview maps--Microbial metabolism in diverse environments|Metabolism--Global and overview maps--Biosynthesis of antibiotics

[denovogenes]_109486	68.53	2e-110	toh:BCB71_05950	K02188	cbiD	cobalt-precorrin-5B (C1)-methyltransferase	2.1.1.195	Metabolism--Metabolism of cofactors and vitamins--Porphyrin and chlorophyll metabolism|Metabolism--Global and overview maps--Metabolic pathways

[denovogenes]_109487	100.00	0.0	esl:O3K_00240	K03291	MFS.SET	MFS transporter, SET family, sugar efflux transporter	--	--

[denovogenes]_109491	44.62	4e-66	ibu:IB211_02362c	K04019	eutA	ethanolamine utilization protein EutA	--	Metabolism--Lipid metabolism--Glycerophospholipid metabolism|Metabolism--Global and overview maps--Metabolic pathways

[denovogenes]_109493	56.47	3e-100	bacc:BRDCF_p831	K09001	anmK	anhydro-N-acetylmuramic acid kinase	2.7.1.170	--

[denovogenes]_109495	91.05	2e-166	rto:RTO_15710	K00088	IMPDH, guaB	IMP dehydrogenase	1.1.1.205	Metabolism--Nucleotide metabolism--Purine metabolism|Metabolism--Xenobiotics biodegradation and metabolism--Drug metabolism - other enzymes|Metabolism--Global and overview maps--Metabolic pathways|Metabolism--Global and overview maps--Biosynthesis of secondary metabolites

[denovogenes]_109496	89.52	6e-159	lacy:A4V08_06725	K03750	moeA	molybdopterin molybdotransferase	2.10.1.1	Metabolism--Metabolism of cofactors and vitamins--Folate biosynthesis|Metabolism--Global and overview maps--Metabolic pathways

[denovogenes]_109497	45.08	5e-64	azi:AzCIB_3530	K07014	K07014	uncharacterized protein	--	--

[denovogenes]_109498	50.43	2e-30	cace:CACET_c35380	K06374	spmB	spore maturation protein B	--	--

[denovogenes]_1095	77.82	0.0	cbv:U729_763	K02337	DPO3A1, dnaE	DNA polymerase III subunit alpha	2.7.7.7	Metabolism--Nucleotide metabolism--Purine metabolism|Metabolism--Nucleotide metabolism--Pyrimidine metabolism|Metabolism--Global and overview maps--Metabolic pathways|Genetic Information Processing--Replication and repair--DNA replication|Genetic Information Processing--Replication and repair--Mismatch repair|Genetic Information Processing--Replication and repair--Homologous recombination

[denovogenes]_109503	99.22	0.0	bcel:BcellWH2_02308	K02836	prfB	peptide chain release factor 2	--	--

[denovogenes]_109505	89.49	2e-170	bhl:Bache_2857	K11927	rhlE	ATP-dependent RNA helicase RhlE	3.6.4.13	Genetic Information Processing--Folding, sorting and degradation--RNA degradation

[denovogenes]_109508	94.93	1e-141	pvl:AOB99_15605	K06182	rluF	23S rRNA pseudouridine2604 synthase	5.4.99.21	--

[denovogenes]_109510	68.87	5e-124	dsa:Desal_1388	K11690	dctM	C4-dicarboxylate transporter, DctM subunit	--	Environmental Information Processing--Signal transduction--Two-component system

[denovogenes]_109511	89.11	4e-163	pmr:PMI1588	K07642	baeS, smeS	two-component system, OmpR family, sensor histidine kinase BaeS	2.7.13.3	Environmental Information Processing--Signal transduction--Two-component system

[denovogenes]_109512	50.00	9e-88	bur:Bcep18194_B1321	K05882	E1.1.1.91	aryl-alcohol dehydrogenase (NADP+)	1.1.1.91	--

[denovogenes]_109514	56.27	6e-93	cdg:CDBI1_14930	K02755	PTS-Bgl-EIIA, bglF, bglP	PTS system, beta-glucoside-specific IIA component	2.7.1.-	Environmental Information Processing--Membrane transport--Phosphotransferase system (PTS)

[denovogenes]_109514	56.27	6e-93	cdg:CDBI1_14930	K02756	PTS-Bgl-EIIB, bglF, bglP	PTS system, beta-glucoside-specific IIB component	2.7.1.-	Environmental Information Processing--Membrane transport--Phosphotransferase system (PTS)

[denovogenes]_109514	56.27	6e-93	cdg:CDBI1_14930	K02757	PTS-Bgl-EIIC, bglF, bglP	PTS system, beta-glucoside-specific IIC component	--	Environmental Information Processing--Membrane transport--Phosphotransferase system (PTS)

[denovogenes]_109517	91.80	1e-172	bth:BT_3129	K02557	motB	chemotaxis protein MotB	--	Cellular Processes--Cell motility--Bacterial chemotaxis|Cellular Processes--Cell motility--Flagellar assembly

[denovogenes]_109518	48.25	2e-73	boa:Bovatus_04431	K00674	dapD	2,3,4,5-tetrahydropyridine-2-carboxylate N-succinyltransferase	2.3.1.117	Metabolism--Amino acid metabolism--Lysine biosynthesis|Metabolism--Global and overview maps--Metabolic pathways|Metabolism--Global and overview maps--Microbial metabolism in diverse environments|Metabolism--Global and overview maps--Biosynthesis of amino acids

[denovogenes]_10952	77.94	0.0	lir:LAW_00249	K03702	uvrB	excinuclease ABC subunit B	--	Genetic Information Processing--Replication and repair--Nucleotide excision repair

[denovogenes]_109520	41.31	2e-60	cpi:Cpin_6102	K02477	K02477	two-component system, LytT family, response regulator	--	--

[denovogenes]_109522	100.00	0.0	boa:Bovatus_01222	K01695	trpA	tryptophan synthase alpha chain	4.2.1.20	Metabolism--Amino acid metabolism--Glycine, serine and threonine metabolism|Metabolism--Amino acid metabolism--Phenylalanine, tyrosine and tryptophan biosynthesis|Metabolism--Global and overview maps--Metabolic pathways|Metabolism--Global and overview maps--Biosynthesis of secondary metabolites|Metabolism--Global and overview maps--Biosynthesis of antibiotics|Metabolism--Global and overview maps--Biosynthesis of amino acids

[denovogenes]_109523	44.92	5e-60	axl:AXY_18070	K06153	bacA	undecaprenyl-diphosphatase	3.6.1.27	Metabolism--Glycan biosynthesis and metabolism--Peptidoglycan biosynthesis

[denovogenes]_109525	51.00	1e-79	exu:ESP131_05005	K00215	dapB	4-hydroxy-tetrahydrodipicolinate reductase	1.17.1.8	Metabolism--Biosynthesis of other secondary metabolites--Monobactam biosynthesis|Metabolism--Amino acid metabolism--Lysine biosynthesis|Metabolism--Global and overview maps--Metabolic pathways|Metabolism--Global and overview maps--Biosynthesis of secondary metabolites|Metabolism--Global and overview maps--Microbial metabolism in diverse environments|Metabolism--Global and overview maps--Biosynthesis of antibiotics|Metabolism--Global and overview maps--Biosynthesis of amino acids

[denovogenes]_109527	55.34	1e-100	ava:Ava_2888	K05886	sdh	serine 3-dehydrogenase (NADP+)	1.1.1.276	--

[denovogenes]_109529	53.36	1e-90	ctyk:CTK_C11240	K06223	dam	DNA adenine methylase	2.1.1.72	Genetic Information Processing--Replication and repair--Mismatch repair

[denovogenes]_109530	99.61	0.0	boa:Bovatus_03963	K02065	mlaF, linL, mkl	phospholipid/cholesterol/gamma-HCH transport system ATP-binding protein	--	Environmental Information Processing--Membrane transport--ABC transporters

[denovogenes]_109531	51.92	2e-89	xcv:XCVc0009	K07493	K07493	putative transposase	--	--

[denovogenes]_109532	62.30	5e-113	euc:EC1_00730	K03436	fruR2, fruR	DeoR family transcriptional regulator, fructose operon transcriptional repressor	--	--

[denovogenes]_109535	43.87	9e-71	sri:SELR_06510	K02444	glpR	DeoR family transcriptional regulator, glycerol-3-phosphate regulon repressor	--	--

[denovogenes]_109536	84.38	4e-158	ipo:Ilyop_1183	K03149	thiG	thiazole synthase	2.8.1.10	Metabolism--Metabolism of cofactors and vitamins--Thiamine metabolism|Metabolism--Global and overview maps--Metabolic pathways

[denovogenes]_109537	96.48	0.0	vpr:Vpar_1472	K01265	map	methionyl aminopeptidase	3.4.11.18	--

[denovogenes]_109538	100.00	0.0	pdi:BDI_2402	K02065	mlaF, linL, mkl	phospholipid/cholesterol/gamma-HCH transport system ATP-binding protein	--	Environmental Information Processing--Membrane transport--ABC transporters

[denovogenes]_10954	98.53	0.0	bfs:BF9343_1555	K11381	bkdA	2-oxoisovalerate dehydrogenase E1 component	1.2.4.4	Metabolism--Amino acid metabolism--Valine, leucine and isoleucine degradation|Metabolism--Carbohydrate metabolism--Propanoate metabolism|Metabolism--Global and overview maps--Metabolic pathways|Metabolism--Global and overview maps--Biosynthesis of secondary metabolites|Metabolism--Global and overview maps--Biosynthesis of antibiotics

[denovogenes]_109540	41.98	9e-58	btrh:F543_8610	K12984	waaE, kdtX	(heptosyl)LPS beta-1,4-glucosyltransferase	2.4.1.-	--

[denovogenes]_109542	66.80	4e-122	awo:Awo_c32360	K09121	K09121	uncharacterized protein	--	--

[denovogenes]_109544	96.88	0.0	bxy:BXY_47290	K02065	mlaF, linL, mkl	phospholipid/cholesterol/gamma-HCH transport system ATP-binding protein	--	Environmental Information Processing--Membrane transport--ABC transporters

[denovogenes]_109545	100.00	0.0	pvl:AOB99_07925	K01420	fnr	CRP/FNR family transcriptional regulator, anaerobic regulatory protein	--	--

[denovogenes]_109548	57.74	4e-102	ipo:Ilyop_0153	K04069	pflA, pflC, pflE	pyruvate formate lyase activating enzyme	1.97.1.4	--

[denovogenes]_109549	53.05	3e-89	eac:EAL2_c07820	K07238	TC.ZIP, zupT, ZRT3, ZIP2	zinc transporter, ZIP family	--	--

[denovogenes]_109551	84.82	6e-164	bth:BT_0602	K02472	wecC	UDP-N-acetyl-D-mannosaminuronic acid dehydrogenase	1.1.1.336	Metabolism--Carbohydrate metabolism--Amino sugar and nucleotide sugar metabolism|Cellular Processes--Cellular community - prokaryotes--Biofilm formation - Vibrio cholerae

[denovogenes]_109553	78.21	9e-151	btho:Btheta7330_02946	K07082	K07082	UPF0755 protein	--	--

[denovogenes]_109554	91.44	2e-159	vpr:Vpar_0997	K02433	gatA, QRSL1	aspartyl-tRNA(Asn)/glutamyl-tRNA(Gln) amidotransferase subunit A	6.3.5.6 6.3.5.7	Genetic Information Processing--Translation--Aminoacyl-tRNA biosynthesis|Metabolism--Global and overview maps--Metabolic pathways

[denovogenes]_109558	47.24	1e-75	fae:FAES_5045	K01874	MARS, metG	methionyl-tRNA synthetase	6.1.1.10	Metabolism--Metabolism of other amino acids--Selenocompound metabolism|Genetic Information Processing--Translation--Aminoacyl-tRNA biosynthesis

[denovogenes]_109560	46.62	1e-42	pmf:P9303_05381	K00975	glgC	glucose-1-phosphate adenylyltransferase	2.7.7.27	Metabolism--Carbohydrate metabolism--Starch and sucrose metabolism|Metabolism--Carbohydrate metabolism--Amino sugar and nucleotide sugar metabolism|Metabolism--Global and overview maps--Metabolic pathways|Metabolism--Global and overview maps--Biosynthesis of secondary metabolites|Cellular Processes--Cellular community - prokaryotes--Biofilm formation - Escherichia coli

[denovogenes]_109562	99.61	0.0	mmk:MU9_1831	K02030	ABC.PA.S	polar amino acid transport system substrate-binding protein	--	--

[denovogenes]_109563	99.61	0.0	mmk:MU9_1426	K02169	bioC	malonyl-CoA O-methyltransferase	2.1.1.197	Metabolism--Metabolism of cofactors and vitamins--Biotin metabolism|Metabolism--Global and overview maps--Metabolic pathways

[denovogenes]_109564	73.73	5e-131	efm:M7W_1233	K01779	racD	aspartate racemase	5.1.1.13	Metabolism--Amino acid metabolism--Alanine, aspartate and glutamate metabolism|Metabolism--Metabolism of terpenoids and polyketides--Nonribosomal peptide structures

[denovogenes]_109565	100.00	0.0	bfb:VU15_01760	K00806	uppS	undecaprenyl diphosphate synthase	2.5.1.31	Metabolism--Metabolism of terpenoids and polyketides--Terpenoid backbone biosynthesis|Metabolism--Global and overview maps--Biosynthesis of secondary metabolites

[denovogenes]_109567	72.94	3e-140	edu:LIU_04980	K02003	ABC.CD.A	putative ABC transport system ATP-binding protein	--	--

[denovogenes]_109568	45.75	3e-68	str:Sterm_3662	K03481	glvR	RpiR family transcriptional regulator, glv operon transcriptional regulator	--	--

[denovogenes]_109569	100.00	0.0	bfb:VU15_03835	K00677	lpxA	UDP-N-acetylglucosamine acyltransferase	2.3.1.129	Metabolism--Glycan biosynthesis and metabolism--Lipopolysaccharide biosynthesis|Metabolism--Global and overview maps--Metabolic pathways|Human Diseases--Drug resistance: Antimicrobial--Cationic antimicrobial peptide (CAMP) resistance

[denovogenes]_10957	99.71	0.0	cfd:CFNIH1_06935	K15922	yihQ	sulfoquinovosidase	3.2.1.199	--

[denovogenes]_109570	64.49	2e-104	lacy:A4V08_04960	K06967	trmK	tRNA (adenine22-N1)-methyltransferase	2.1.1.217	--

[denovogenes]_109571	100.00	0.0	bfg:BF638R_0739	K03496	parA, soj	chromosome partitioning protein	--	--

[denovogenes]_109573	40.27	2e-42	bso:BSNT_09854	K02020	modA	molybdate transport system substrate-binding protein	--	Environmental Information Processing--Membrane transport--ABC transporters

[denovogenes]_109574	79.18	9e-139	ecas:ECBG_03122	K02041	phnC	phosphonate transport system ATP-binding protein	3.6.3.28	Environmental Information Processing--Membrane transport--ABC transporters

[denovogenes]_109577	55.60	1e-99	lrc:LOCK908_0463	K03478	chbG	chitin disaccharide deacetylase	3.5.1.105	--

[denovogenes]_109578	100.00	0.0	mmk:MU9_997	K02233	E2.7.8.26, cobS, cobV	adenosylcobinamide-GDP ribazoletransferase	2.7.8.26	Metabolism--Metabolism of cofactors and vitamins--Porphyrin and chlorophyll metabolism|Metabolism--Global and overview maps--Metabolic pathways

[denovogenes]_109579	66.01	2e-115	pdf:CD630DERM_34370	K02233	E2.7.8.26, cobS, cobV	adenosylcobinamide-GDP ribazoletransferase	2.7.8.26	Metabolism--Metabolism of cofactors and vitamins--Porphyrin and chlorophyll metabolism|Metabolism--Global and overview maps--Metabolic pathways

[denovogenes]_10958	62.76	0.0	pmy:Pmen_3239	K08282	E2.7.11.1	non-specific serine/threonine protein kinase	2.7.11.1	--

[denovogenes]_109580	75.98	1e-143	lacy:A4V08_14805	K03424	--	--	--	--

[denovogenes]_109582	77.17	9e-143	clt:CM240_1813	K02003	ABC.CD.A	putative ABC transport system ATP-binding protein	--	--

[denovogenes]_109587	76.89	5e-116	lacy:A4V08_27050	K06024	scpB	segregation and condensation protein B	--	--

[denovogenes]_109591	79.22	1e-147	cck:Ccar_03205	K02003	ABC.CD.A	putative ABC transport system ATP-binding protein	--	--

[denovogenes]_109593	98.43	0.0	mmk:MU9_922	K01146	xni	protein Xni	--	--

[denovogenes]_109595	100.00	0.0	ene:ENT_19160	K02003	ABC.CD.A	putative ABC transport system ATP-binding protein	--	--

[denovogenes]_109597	100.00	0.0	stb:SGPB_0481	K04074	divIVA	cell division initiation protein	--	--

[denovogenes]_109599	89.41	2e-167	cld:CLSPO_c21870	K02003	ABC.CD.A	putative ABC transport system ATP-binding protein	--	--

[denovogenes]_1096	99.83	0.0	efn:DENG_02985	K03529	smc	chromosome segregation protein	--	--

[denovogenes]_10960	99.56	0.0	elp:P12B_c3266	K07121	K07121	uncharacterized protein	--	--

[denovogenes]_109600	79.45	1e-151	cbz:Cbs_5095	K03496	parA, soj	chromosome partitioning protein	--	--

[denovogenes]_109601	61.18	6e-108	thl:TEH_15700	K03470	rnhB	ribonuclease HII	3.1.26.4	Genetic Information Processing--Replication and repair--DNA replication

[denovogenes]_109602	80.78	2e-148	cdg:CDBI1_17410	K03525	coaX	type III pantothenate kinase	2.7.1.33	Metabolism--Metabolism of cofactors and vitamins--Pantothenate and CoA biosynthesis|Metabolism--Global and overview maps--Metabolic pathways

[denovogenes]_109603	74.21	1e-137	efa:EF1188	K01101	E3.1.3.41	4-nitrophenyl phosphatase	3.1.3.41	Metabolism--Xenobiotics biodegradation and metabolism--Aminobenzoate degradation|Metabolism--Global and overview maps--Microbial metabolism in diverse environments

[denovogenes]_109605	46.50	7e-71	baco:OXB_2218	K15600	thiZ	putative hydroxymethylpyrimidine transport system ATP-binding protein	--	Environmental Information Processing--Membrane transport--ABC transporters

[denovogenes]_109606	100.00	0.0	efd:EFD32_1375	K02013	ABC.FEV.A	iron complex transport system ATP-binding protein	3.6.3.34	Environmental Information Processing--Membrane transport--ABC transporters

[denovogenes]_109607	100.00	0.0	efq:DR75_652	K03470	rnhB	ribonuclease HII	3.1.26.4	Genetic Information Processing--Replication and repair--DNA replication

[denovogenes]_109608	74.12	2e-139	coo:CCU_28510	K02003	ABC.CD.A	putative ABC transport system ATP-binding protein	--	--

[denovogenes]_109609	100.00	0.0	stb:SGPB_0240	K00868	pdxK, pdxY	pyridoxine kinase	2.7.1.35	Metabolism--Metabolism of cofactors and vitamins--Vitamin B6 metabolism|Metabolism--Global and overview maps--Metabolic pathways

[denovogenes]_109611	77.02	3e-135	rho:RHOM_11530	K10118	msmF	raffinose/stachyose/melibiose transport system permease protein	--	Environmental Information Processing--Membrane transport--ABC transporters

[denovogenes]_109612	99.61	0.0	stb:SGPB_1880	K09765	K09765	uncharacterized protein	--	--

[denovogenes]_109613	100.00	0.0	sgt:SGGB_1297	K00215	dapB	4-hydroxy-tetrahydrodipicolinate reductase	1.17.1.8	Metabolism--Biosynthesis of other secondary metabolites--Monobactam biosynthesis|Metabolism--Amino acid metabolism--Lysine biosynthesis|Metabolism--Global and overview maps--Metabolic pathways|Metabolism--Global and overview maps--Biosynthesis of secondary metabolites|Metabolism--Global and overview maps--Microbial metabolism in diverse environments|Metabolism--Global and overview maps--Biosynthesis of antibiotics|Metabolism--Global and overview maps--Biosynthesis of amino acids

[denovogenes]_109614	76.86	2e-143	pdf:CD630DERM_12620	K03470	rnhB	ribonuclease HII	3.1.26.4	Genetic Information Processing--Replication and repair--DNA replication

[denovogenes]_109616	92.94	2e-172	cif:AL515_06515	K10831	tauB	taurine transport system ATP-binding protein	3.6.3.36	Metabolism--Energy metabolism--Sulfur metabolism|Environmental Information Processing--Membrane transport--ABC transporters

[denovogenes]_109617	63.39	2e-121	cbk:CLL_A2632	K02049	ABC.SN.A	NitT/TauT family transport system ATP-binding protein	--	--

[denovogenes]_109618	100.00	0.0	ene:ENT_06880	K05833	ABC.X4.A	putative ABC transport system ATP-binding protein	--	--

[denovogenes]_109619	99.61	7e-180	stb:SGPB_1890	K00674	dapD	2,3,4,5-tetrahydropyridine-2-carboxylate N-succinyltransferase	2.3.1.117	Metabolism--Amino acid metabolism--Lysine biosynthesis|Metabolism--Global and overview maps--Metabolic pathways|Metabolism--Global and overview maps--Microbial metabolism in diverse environments|Metabolism--Global and overview maps--Biosynthesis of amino acids

[denovogenes]_10962	94.09	0.0	afd:Alfi_1701	K02355	fusA, GFM, EFG	elongation factor G	--	--

[denovogenes]_109620	40.48	6e-61	eal:EAKF1_ch1096c	K13244	yahA	c-di-GMP-specific phosphodiesterase	3.1.4.52	--

[denovogenes]_109622	71.43	1e-131	cbz:Cbs_5096	K03497	parB, spo0J	chromosome partitioning protein, ParB family	--	--

[denovogenes]_109623	96.86	0.0	sbc:SbBS512_E2426	K04069	pflA, pflC, pflE	pyruvate formate lyase activating enzyme	1.97.1.4	--

[denovogenes]_109624	78.40	3e-148	cpe:soj	K03496	parA, soj	chromosome partitioning protein	--	--

[denovogenes]_109625	98.43	3e-177	cif:AL515_09170	K15555	ssuB	sulfonate transport system ATP-binding protein	3.6.3.-	Metabolism--Energy metabolism--Sulfur metabolism|Environmental Information Processing--Membrane transport--ABC transporters

[denovogenes]_109627	99.60	1e-180	cbut:ATN24_05145	K03091	SIG3.4	RNA polymerase sporulation-specific sigma factor	--	--

[denovogenes]_109628	99.61	0.0	elo:EC042_3062	K03228	yscT, sctT, hrcT	type III secretion protein T	--	Environmental Information Processing--Membrane transport--Bacterial secretion system

[denovogenes]_109629	100.00	0.0	pvl:AOB99_05420	K02169	bioC	malonyl-CoA O-methyltransferase	2.1.1.197	Metabolism--Metabolism of cofactors and vitamins--Biotin metabolism|Metabolism--Global and overview maps--Metabolic pathways

[denovogenes]_109631	85.88	2e-153	lacy:A4V08_27340	K02049	ABC.SN.A	NitT/TauT family transport system ATP-binding protein	--	--

[denovogenes]_109632	86.11	9e-149	cpr:CPR_1571	K03149	thiG	thiazole synthase	2.8.1.10	Metabolism--Metabolism of cofactors and vitamins--Thiamine metabolism|Metabolism--Global and overview maps--Metabolic pathways

[denovogenes]_109634	42.02	2e-52	bpm:BURPS1710b_A2009	K00423	E1.10.3.3	L-ascorbate oxidase	1.10.3.3	Metabolism--Carbohydrate metabolism--Ascorbate and aldarate metabolism|Metabolism--Global and overview maps--Metabolic pathways

[denovogenes]_109635	71.37	3e-136	erb:A4V01_04270	K02003	ABC.CD.A	putative ABC transport system ATP-binding protein	--	--

[denovogenes]_109637	99.61	0.0	sgg:SGGBAA2069_c06360	K00930	argB	acetylglutamate kinase	2.7.2.8	Metabolism--Amino acid metabolism--Arginine biosynthesis|Metabolism--Global and overview maps--Metabolic pathways|Metabolism--Global and overview maps--Biosynthesis of secondary metabolites|Metabolism--Global and overview maps--Biosynthesis of antibiotics|Metabolism--Global and overview maps--2-Oxocarboxylic acid metabolism|Metabolism--Global and overview maps--Biosynthesis of amino acids

[denovogenes]_109639	100.00	0.0	pvl:AOB99_05575	K21029	moeB	molybdopterin-synthase adenylyltransferase	2.7.7.80	Genetic Information Processing--Folding, sorting and degradation--Sulfur relay system

[denovogenes]_109641	53.72	7e-82	fhw:RN87_01030	K07090	K07090	uncharacterized protein	--	--

[denovogenes]_109642	100.00	0.0	pvl:AOB99_10710	K08680	menH	2-succinyl-6-hydroxy-2,4-cyclohexadiene-1-carboxylate synthase	4.2.99.20	Metabolism--Metabolism of cofactors and vitamins--Ubiquinone and other terpenoid-quinone biosynthesis|Metabolism--Global and overview maps--Metabolic pathways|Metabolism--Global and overview maps--Biosynthesis of secondary metabolites

[denovogenes]_109643	57.48	4e-101	ppx:T1E_0545	K03169	topB	DNA topoisomerase III	5.99.1.2	--

[denovogenes]_109644	100.00	0.0	efe:EFER_0465	K00554	trmD	tRNA (guanine37-N1)-methyltransferase	2.1.1.228	--

[denovogenes]_109647	85.49	1e-159	cpe:CPE0850	K02003	ABC.CD.A	putative ABC transport system ATP-binding protein	--	--

[denovogenes]_109649	100.00	0.0	sbc:SbBS512_E2426	K04069	pflA, pflC, pflE	pyruvate formate lyase activating enzyme	1.97.1.4	--

[denovogenes]_10965	77.23	0.0	csh:Closa_2939	K03654	recQ	ATP-dependent DNA helicase RecQ	3.6.4.12	Genetic Information Processing--Folding, sorting and degradation--RNA degradation

[denovogenes]_109650	81.03	2e-147	asu:Asuc_1303	K03300	TC.CITMHS	citrate-Mg2+:H+ or citrate-Ca2+:H+ symporter, CitMHS family	--	--

[denovogenes]_109652	40.00	3e-41	fpl:Ferp_1187	K04114	bcrA, badF	benzoyl-CoA reductase subunit A	1.3.7.8	Metabolism--Xenobiotics biodegradation and metabolism--Benzoate degradation|Metabolism--Global and overview maps--Metabolic pathways|Metabolism--Global and overview maps--Microbial metabolism in diverse environments|Metabolism--Global and overview maps--Degradation of aromatic compounds

[denovogenes]_109653	43.67	3e-67	cml:BN424_1792	K19285	nfrA1	FMN reductase (NADPH)	1.5.1.38	Metabolism--Metabolism of cofactors and vitamins--Riboflavin metabolism|Metabolism--Global and overview maps--Metabolic pathways

[denovogenes]_109654	100.00	0.0	cbut:ATN24_14870	K02103	araR	GntR family transcriptional regulator, arabinose operon transcriptional repressor	--	--

[denovogenes]_109655	81.20	3e-152	csr:Cspa_c51270	K01921	ddl	D-alanine-D-alanine ligase	6.3.2.4	Metabolism--Metabolism of other amino acids--D-Alanine metabolism|Metabolism--Glycan biosynthesis and metabolism--Peptidoglycan biosynthesis|Metabolism--Global and overview maps--Metabolic pathways|Human Diseases--Drug resistance: Antimicrobial--Vancomycin resistance

[denovogenes]_109657	54.69	1e-88	csb:CLSA_c45490	K03217	yidC, spoIIIJ, OXA1, ccfA	YidC/Oxa1 family membrane protein insertase	--	Cellular Processes--Cellular community - prokaryotes--Quorum sensing|Genetic Information Processing--Folding, sorting and degradation--Protein export|Environmental Information Processing--Membrane transport--Bacterial secretion system

[denovogenes]_109658	40.32	3e-39	bacc:BRDCF_p614	K03657	uvrD, pcrA	DNA helicase II / ATP-dependent DNA helicase PcrA	3.6.4.12	Genetic Information Processing--Replication and repair--Nucleotide excision repair|Genetic Information Processing--Replication and repair--Mismatch repair

[denovogenes]_109663	46.22	5e-74	yel:LC20_02973	K01890	FARSB, pheT	phenylalanyl-tRNA synthetase beta chain	6.1.1.20	Genetic Information Processing--Translation--Aminoacyl-tRNA biosynthesis

[denovogenes]_109664	100.00	0.0	prg:RB151_042780	K02886	RP-L2, MRPL2, rplB	large subunit ribosomal protein L2	--	Genetic Information Processing--Translation--Ribosome

[denovogenes]_109665	66.80	9e-120	bbay:A4V04_08305	K07138	K07138	uncharacterized protein	--	--

[denovogenes]_109666	68.63	8e-128	cld:CLSPO_c26120	K06398	spoIVA	stage IV sporulation protein A	--	--

[denovogenes]_109668	100.00	0.0	shq:A0259_00090	K02036	pstB	phosphate transport system ATP-binding protein	3.6.3.27	Environmental Information Processing--Membrane transport--ABC transporters

[denovogenes]_109669	74.02	1e-142	cbt:CLH_0067	K07139	K07139	uncharacterized protein	--	--

[denovogenes]_109670	98.83	4e-179	cko:CKO_03045	K11203	PTS-Fru2-EIIC	PTS system, fructose-specific IIC-like component	--	--

[denovogenes]_109671	98.05	0.0	cfd:CFNIH1_21450	K19997	gnu	GlcNAc-P-P-Und epimerase	5.1.3.26	--

[denovogenes]_109673	78.82	7e-142	cbz:Cbs_1505	K03322	mntH	manganese transport protein	--	--

[denovogenes]_109674	41.77	1e-58	eac:EAL2_c02570	K07029	dagK	diacylglycerol kinase (ATP)	2.7.1.107	Metabolism--Lipid metabolism--Glycerolipid metabolism|Metabolism--Lipid metabolism--Glycerophospholipid metabolism|Metabolism--Global and overview maps--Metabolic pathways|Metabolism--Global and overview maps--Biosynthesis of secondary metabolites

[denovogenes]_109676	75.49	2e-136	cno:NT01CX_0523	K03569	mreB	rod shape-determining protein MreB and related proteins	--	--

[denovogenes]_109677	71.88	4e-134	ctet:BN906_00978	K01465	URA4, pyrC	dihydroorotase	3.5.2.3	Metabolism--Nucleotide metabolism--Pyrimidine metabolism|Metabolism--Global and overview maps--Metabolic pathways

[denovogenes]_10968	58.40	0.0	pdi:BDI_0535	K01187	malZ	alpha-glucosidase	3.2.1.20	Metabolism--Carbohydrate metabolism--Galactose metabolism|Metabolism--Carbohydrate metabolism--Starch and sucrose metabolism|Metabolism--Global and overview maps--Metabolic pathways

[denovogenes]_109680	66.93	1e-125	mas:Mahau_0523	K01874	MARS, metG	methionyl-tRNA synthetase	6.1.1.10	Metabolism--Metabolism of other amino acids--Selenocompound metabolism|Genetic Information Processing--Translation--Aminoacyl-tRNA biosynthesis

[denovogenes]_109681	79.07	1e-148	cbv:U729_3055	K11072	potA	spermidine/putrescine transport system ATP-binding protein	3.6.3.31	Environmental Information Processing--Membrane transport--ABC transporters

[denovogenes]_109682	89.45	1e-177	cso:CLS_27000	K01875	SARS, serS	seryl-tRNA synthetase	6.1.1.11	Genetic Information Processing--Translation--Aminoacyl-tRNA biosynthesis

[denovogenes]_109683	40.17	2e-44	dfd:Desfe_0370	K05825	LYSN	2-aminoadipate transaminase	2.6.1.-	Metabolism--Amino acid metabolism--Lysine biosynthesis|Metabolism--Global and overview maps--Metabolic pathways|Metabolism--Global and overview maps--Biosynthesis of antibiotics|Metabolism--Global and overview maps--2-Oxocarboxylic acid metabolism|Metabolism--Global and overview maps--Biosynthesis of amino acids

[denovogenes]_109688	99.61	0.0	cif:AL515_03900	K11737	cycA	D-serine/D-alanine/glycine transporter	--	--

[denovogenes]_109689	52.16	4e-101	cdg:CDBI1_10810	K16950	asrA	anaerobic sulfite reductase subunit A	--	Metabolism--Energy metabolism--Sulfur metabolism|Metabolism--Global and overview maps--Microbial metabolism in diverse environments

[denovogenes]_10969	99.26	0.0	boa:Bovatus_00157	K11381	bkdA	2-oxoisovalerate dehydrogenase E1 component	1.2.4.4	Metabolism--Amino acid metabolism--Valine, leucine and isoleucine degradation|Metabolism--Carbohydrate metabolism--Propanoate metabolism|Metabolism--Global and overview maps--Metabolic pathways|Metabolism--Global and overview maps--Biosynthesis of secondary metabolites|Metabolism--Global and overview maps--Biosynthesis of antibiotics

[denovogenes]_109690	100.00	0.0	eum:ECUMN_0088	K01925	murD	UDP-N-acetylmuramoylalanine--D-glutamate ligase	6.3.2.9	Metabolism--Metabolism of other amino acids--D-Glutamine and D-glutamate metabolism|Metabolism--Glycan biosynthesis and metabolism--Peptidoglycan biosynthesis|Metabolism--Global and overview maps--Metabolic pathways

[denovogenes]_109692	97.66	2e-177	cbut:ATN24_09370	K03307	TC.SSS	solute:Na+ symporter, SSS family	--	--

[denovogenes]_109693	56.32	1e-97	csr:Cspa_c46650	K07720	yesN	two-component system, response regulator YesN	--	Environmental Information Processing--Signal transduction--Two-component system

[denovogenes]_109694	42.91	1e-66	sbh:SBI_02579	K07407	E3.2.1.22B, galA, rafA	alpha-galactosidase	3.2.1.22	Metabolism--Carbohydrate metabolism--Galactose metabolism|Metabolism--Lipid metabolism--Glycerolipid metabolism|Metabolism--Lipid metabolism--Sphingolipid metabolism|Metabolism--Glycan biosynthesis and metabolism--Glycosphingolipid biosynthesis - globo and isoglobo series

[denovogenes]_109699	83.53	3e-162	tur:AT726_10180	K01698	hemB, ALAD	porphobilinogen synthase	4.2.1.24	Metabolism--Metabolism of cofactors and vitamins--Porphyrin and chlorophyll metabolism|Metabolism--Global and overview maps--Metabolic pathways|Metabolism--Global and overview maps--Biosynthesis of secondary metabolites

[denovogenes]_10970	97.94	0.0	boa:Bovatus_03937	K07386	pepO	putative endopeptidase	3.4.24.-	--

[denovogenes]_109700	40.57	1e-49	lhk:LHK_00428	K03294	TC.APA	basic amino acid/polyamine antiporter, APA family	--	--

[denovogenes]_109701	78.91	1e-143	clt:CM240_0245	K02015	ABC.FEV.P	iron complex transport system permease protein	--	Environmental Information Processing--Membrane transport--ABC transporters

[denovogenes]_109702	46.96	2e-66	slp:Slip_1187	K02654	pilD, pppA	leader peptidase (prepilin peptidase) / N-methyltransferase	3.4.23.43 2.1.1.-	--

[denovogenes]_109703	87.16	9e-168	bhl:Bache_0569	K00677	lpxA	UDP-N-acetylglucosamine acyltransferase	2.3.1.129	Metabolism--Glycan biosynthesis and metabolism--Lipopolysaccharide biosynthesis|Metabolism--Global and overview maps--Metabolic pathways|Human Diseases--Drug resistance: Antimicrobial--Cationic antimicrobial peptide (CAMP) resistance

[denovogenes]_109704	86.57	5e-167	bth:BT_3248	K09817	znuC	zinc transport system ATP-binding protein	3.6.3.-	Environmental Information Processing--Membrane transport--ABC transporters

[denovogenes]_109705	57.87	3e-103	aoe:Clos_0031	K10795	prdD	D-proline reductase (dithiol) PrdD	1.21.4.1	Metabolism--Amino acid metabolism--Arginine and proline metabolism

[denovogenes]_109706	72.69	8e-136	ssg:Selsp_0700	K01142	E3.1.11.2, xthA	exodeoxyribonuclease III	3.1.11.2	Genetic Information Processing--Replication and repair--Base excision repair

[denovogenes]_109709	68.38	3e-117	byl:A4V09_04375	K02049	ABC.SN.A	NitT/TauT family transport system ATP-binding protein	--	--

[denovogenes]_109711	100.00	0.0	bvu:BVU_0099	K00677	lpxA	UDP-N-acetylglucosamine acyltransferase	2.3.1.129	Metabolism--Glycan biosynthesis and metabolism--Lipopolysaccharide biosynthesis|Metabolism--Global and overview maps--Metabolic pathways|Human Diseases--Drug resistance: Antimicrobial--Cationic antimicrobial peptide (CAMP) resistance

[denovogenes]_109713	61.83	1e-96	fpla:A4U99_17740	K04074	divIVA	cell division initiation protein	--	--

[denovogenes]_109714	50.97	6e-80	ain:Acin_2450	K06346	jag	spoIIIJ-associated protein	--	--

[denovogenes]_109715	94.90	0.0	bxy:BXY_44480	K02169	bioC	malonyl-CoA O-methyltransferase	2.1.1.197	Metabolism--Metabolism of cofactors and vitamins--Biotin metabolism|Metabolism--Global and overview maps--Metabolic pathways

[denovogenes]_109716	53.94	3e-88	csq:CSCA_5145	K15554	ssuC	sulfonate transport system permease protein	--	Metabolism--Energy metabolism--Sulfur metabolism|Environmental Information Processing--Membrane transport--ABC transporters

[denovogenes]_109717	46.61	3e-78	sgy:Sgly_2889	K05895	cobK-cbiJ	precorrin-6A/cobalt-precorrin-6A reductase	1.3.1.54 1.3.1.106	Metabolism--Metabolism of cofactors and vitamins--Porphyrin and chlorophyll metabolism|Metabolism--Global and overview maps--Metabolic pathways

[denovogenes]_109718	89.33	4e-169	bxy:BXY_01140	K01126	E3.1.4.46, glpQ, ugpQ	glycerophosphoryl diester phosphodiesterase	3.1.4.46	Metabolism--Lipid metabolism--Glycerophospholipid metabolism

[denovogenes]_109719	80.16	1e-146	ddh:Desde_0492	K02003	ABC.CD.A	putative ABC transport system ATP-binding protein	--	--

[denovogenes]_109722	62.11	3e-111	byl:A4V09_19035	K03525	coaX	type III pantothenate kinase	2.7.1.33	Metabolism--Metabolism of cofactors and vitamins--Pantothenate and CoA biosynthesis|Metabolism--Global and overview maps--Metabolic pathways

[denovogenes]_109723	83.53	8e-159	fpa:FPR_24090	K02003	ABC.CD.A	putative ABC transport system ATP-binding protein	--	--

[denovogenes]_109725	57.83	2e-98	rto:RTO_20570	K07124	K07124	uncharacterized protein	--	--

[denovogenes]_109726	72.76	3e-135	bxy:BXY_28940	K03734	apbE	FAD:protein FMN transferase	2.7.1.180	--

[denovogenes]_109727	55.65	1e-98	csh:Closa_3450	K02421	fliR	flagellar biosynthetic protein FliR	--	Cellular Processes--Cell motility--Flagellar assembly

[denovogenes]_10973	72.75	0.0	cbv:U729_2832	K03655	recG	ATP-dependent DNA helicase RecG	3.6.4.12	Genetic Information Processing--Replication and repair--Homologous recombination

[denovogenes]_109730	80.08	1e-154	rto:RTO_02570	K03496	parA, soj	chromosome partitioning protein	--	--

[denovogenes]_109731	67.73	1e-116	byl:A4V09_04375	K02049	ABC.SN.A	NitT/TauT family transport system ATP-binding protein	--	--

[denovogenes]_109732	67.45	4e-115	bth:BT_0060	K01991	ABC-2.OM, wza	polysaccharide biosynthesis/export protein	--	Cellular Processes--Cellular community - prokaryotes--Biofilm formation - Escherichia coli

[denovogenes]_109733	68.13	6e-124	cpy:Cphy_0114	K09697	natA	sodium transport system ATP-binding protein	3.6.3.7	Environmental Information Processing--Membrane transport--ABC transporters|Environmental Information Processing--Signal transduction--Two-component system

[denovogenes]_109734	90.20	8e-171	boa:Bovatus_00817	K03437	spoU	RNA methyltransferase, TrmH family	--	--

[denovogenes]_109735	64.40	6e-115	bcel:BcellWH2_00536	K00059	fabG	3-oxoacyl-[acyl-carrier protein] reductase	1.1.1.100	Metabolism--Lipid metabolism--Fatty acid biosynthesis|Metabolism--Metabolism of cofactors and vitamins--Biotin metabolism|Metabolism--Lipid metabolism--Biosynthesis of unsaturated fatty acids|Metabolism--Global and overview maps--Metabolic pathways|Metabolism--Global and overview maps--Fatty acid metabolism

[denovogenes]_109736	95.47	2e-168	bhl:Bache_2311	K02069	ABC.X2.P	putative ABC transport system permease protein	--	--

[denovogenes]_10974	99.85	0.0	mmk:MU9_1367	K02802	PTS-Nag-EIIA, nagE	PTS system, N-acetylglucosamine-specific IIA component	2.7.1.193	Metabolism--Carbohydrate metabolism--Amino sugar and nucleotide sugar metabolism|Environmental Information Processing--Membrane transport--Phosphotransferase system (PTS)

[denovogenes]_10974	99.85	0.0	mmk:MU9_1367	K02803	PTS-Nag-EIIB, nagE	PTS system, N-acetylglucosamine-specific IIB component	2.7.1.193	Metabolism--Carbohydrate metabolism--Amino sugar and nucleotide sugar metabolism|Environmental Information Processing--Membrane transport--Phosphotransferase system (PTS)

[denovogenes]_10974	99.85	0.0	mmk:MU9_1367	K02804	PTS-Nag-EIIC, nagE	PTS system, N-acetylglucosamine-specific IIC component	--	Metabolism--Carbohydrate metabolism--Amino sugar and nucleotide sugar metabolism|Environmental Information Processing--Membrane transport--Phosphotransferase system (PTS)

[denovogenes]_109740	41.60	2e-25	dor:Desor_3049	K17865	croR	3-hydroxybutyryl-CoA dehydratase	4.2.1.55	Metabolism--Carbohydrate metabolism--Glyoxylate and dicarboxylate metabolism|Metabolism--Carbohydrate metabolism--Butanoate metabolism|Metabolism--Global and overview maps--Microbial metabolism in diverse environments|Metabolism--Global and overview maps--Carbon metabolism

[denovogenes]_109741	40.71	3e-50	ped:ING2D1G_1454	K07668	vicR	two-component system, OmpR family, response regulator VicR	--	Environmental Information Processing--Signal transduction--Two-component system

[denovogenes]_109744	60.00	3e-97	rto:RTO_29330	K00991	ispD	2-C-methyl-D-erythritol 4-phosphate cytidylyltransferase	2.7.7.60	Metabolism--Metabolism of terpenoids and polyketides--Terpenoid backbone biosynthesis|Metabolism--Global and overview maps--Metabolic pathways|Metabolism--Global and overview maps--Biosynthesis of secondary metabolites|Metabolism--Global and overview maps--Biosynthesis of antibiotics

[denovogenes]_109745	60.63	2e-99	cso:CLS_23960	K02233	E2.7.8.26, cobS, cobV	adenosylcobinamide-GDP ribazoletransferase	2.7.8.26	Metabolism--Metabolism of cofactors and vitamins--Porphyrin and chlorophyll metabolism|Metabolism--Global and overview maps--Metabolic pathways

[denovogenes]_109746	45.60	3e-18	ham:HALO2299	K03286	TC.OOP	OmpA-OmpF porin, OOP family	--	--

[denovogenes]_109747	45.85	4e-69	cce:Ccel_1276	K02304	MET8	precorrin-2 dehydrogenase / sirohydrochlorin ferrochelatase	1.3.1.76 4.99.1.4	Metabolism--Metabolism of cofactors and vitamins--Porphyrin and chlorophyll metabolism|Metabolism--Global and overview maps--Metabolic pathways|Metabolism--Global and overview maps--Biosynthesis of secondary metabolites

[denovogenes]_109748	56.97	5e-108	rho:RHOM_02195	K04069	pflA, pflC, pflE	pyruvate formate lyase activating enzyme	1.97.1.4	--

[denovogenes]_10975	99.56	0.0	mmk:MU9_1468	K01874	MARS, metG	methionyl-tRNA synthetase	6.1.1.10	Metabolism--Metabolism of other amino acids--Selenocompound metabolism|Genetic Information Processing--Translation--Aminoacyl-tRNA biosynthesis

[denovogenes]_109751	100.00	0.0	bvu:BVU_1102	K09817	znuC	zinc transport system ATP-binding protein	3.6.3.-	Environmental Information Processing--Membrane transport--ABC transporters

[denovogenes]_109752	98.04	0.0	bxy:BXY_44480	K02169	bioC	malonyl-CoA O-methyltransferase	2.1.1.197	Metabolism--Metabolism of cofactors and vitamins--Biotin metabolism|Metabolism--Global and overview maps--Metabolic pathways

[denovogenes]_109753	79.30	7e-149	rto:RTO_02570	K03496	parA, soj	chromosome partitioning protein	--	--

[denovogenes]_109754	40.91	1e-13	ksa:C813_23445	K13694	spr	lipoprotein Spr	--	--

[denovogenes]_109757	100.00	0.0	bvu:BVU_1915	K03496	parA, soj	chromosome partitioning protein	--	--

[denovogenes]_109759	99.61	0.0	ecoa:APECO78_10700	K01692	paaF, echA	enoyl-CoA hydratase	4.2.1.17	Metabolism--Lipid metabolism--Fatty acid degradation|Metabolism--Amino acid metabolism--Valine, leucine and isoleucine degradation|Metabolism--Metabolism of terpenoids and polyketides--Geraniol degradation|Metabolism--Amino acid metabolism--Lysine degradation|Metabolism--Amino acid metabolism--Phenylalanine metabolism|Metabolism--Xenobiotics biodegradation and metabolism--Benzoate degradation|Metabolism--Amino acid metabolism--Tryptophan metabolism|Metabolism--Metabolism of other amino acids--beta-Alanine metabolism|Metabolism--Xenobiotics biodegradation and metabolism--Aminobenzoate degradation|Metabolism--Carbohydrate metabolism--Propanoate metabolism|Metabolism--Carbohydrate metabolism--Butanoate metabolism|Metabolism--Metabolism of terpenoids and polyketides--Limonene and pinene degradation|Metabolism--Xenobiotics biodegradation and metabolism--Caprolactam degradation|Metabolism--Global and overview maps--Metabolic pathways|Metabolism--Global and overview maps--Biosynthesis of secondary metabolites|Metabolism--Global and overview maps--Microbial metabolism in diverse environments|Metabolism--Global and overview maps--Biosynthesis of antibiotics|Metabolism--Global and overview maps--Fatty acid metabolism

[denovogenes]_10976	100.00	0.0	bfg:BF638R_2636	K03702	uvrB	excinuclease ABC subunit B	--	Genetic Information Processing--Replication and repair--Nucleotide excision repair

[denovogenes]_109760	72.83	4e-129	esu:EUS_22210	K01803	TPI, tpiA	triosephosphate isomerase (TIM)	5.3.1.1	Metabolism--Carbohydrate metabolism--Glycolysis / Gluconeogenesis|Metabolism--Carbohydrate metabolism--Fructose and mannose metabolism|Metabolism--Carbohydrate metabolism--Inositol phosphate metabolism|Metabolism--Energy metabolism--Carbon fixation in photosynthetic organisms|Metabolism--Global and overview maps--Metabolic pathways|Metabolism--Global and overview maps--Biosynthesis of secondary metabolites|Metabolism--Global and overview maps--Microbial metabolism in diverse environments|Metabolism--Global and overview maps--Biosynthesis of antibiotics|Metabolism--Global and overview maps--Carbon metabolism|Metabolism--Global and overview maps--Biosynthesis of amino acids

[denovogenes]_109761	99.61	0.0	bvu:BVU_2890	K03218	rlmB	23S rRNA (guanosine2251-2'-O)-methyltransferase	2.1.1.185	--

[denovogenes]_109762	98.82	0.0	boa:Bovatus_04487	K00677	lpxA	UDP-N-acetylglucosamine acyltransferase	2.3.1.129	Metabolism--Glycan biosynthesis and metabolism--Lipopolysaccharide biosynthesis|Metabolism--Global and overview maps--Metabolic pathways|Human Diseases--Drug resistance: Antimicrobial--Cationic antimicrobial peptide (CAMP) resistance

[denovogenes]_109765	91.37	3e-174	bvu:BVU_0241	K00677	lpxA	UDP-N-acetylglucosamine acyltransferase	2.3.1.129	Metabolism--Glycan biosynthesis and metabolism--Lipopolysaccharide biosynthesis|Metabolism--Global and overview maps--Metabolic pathways|Human Diseases--Drug resistance: Antimicrobial--Cationic antimicrobial peptide (CAMP) resistance

[denovogenes]_109766	42.31	1e-09	mts:MTES_2353	K03183	ubiE	demethylmenaquinone methyltransferase / 2-methoxy-6-polyprenyl-1,4-benzoquinol methylase	2.1.1.163 2.1.1.201	Metabolism--Metabolism of cofactors and vitamins--Ubiquinone and other terpenoid-quinone biosynthesis|Metabolism--Global and overview maps--Metabolic pathways|Metabolism--Global and overview maps--Biosynthesis of secondary metabolites

[denovogenes]_109769	41.08	1e-41	trm:JO41_03270	K07335	bmpA, bmpB, tmpC	basic membrane protein A and related proteins	--	--

[denovogenes]_10977	100.00	0.0	bfb:VU15_03240	K03798	ftsH, hflB	cell division protease FtsH	3.4.24.-	--

[denovogenes]_109771	47.37	7e-84	ckr:CKR_2282	K07124	K07124	uncharacterized protein	--	--

[denovogenes]_109774	100.00	0.0	bxy:BXY_33060	K00677	lpxA	UDP-N-acetylglucosamine acyltransferase	2.3.1.129	Metabolism--Glycan biosynthesis and metabolism--Lipopolysaccharide biosynthesis|Metabolism--Global and overview maps--Metabolic pathways|Human Diseases--Drug resistance: Antimicrobial--Cationic antimicrobial peptide (CAMP) resistance

[denovogenes]_109775	93.73	0.0	bvu:BVU_0525	K03269	lpxH	UDP-2,3-diacylglucosamine hydrolase	3.6.1.54	Metabolism--Glycan biosynthesis and metabolism--Lipopolysaccharide biosynthesis|Metabolism--Global and overview maps--Metabolic pathways

[denovogenes]_109776	99.22	0.0	bxy:BXY_01140	K01126	E3.1.4.46, glpQ, ugpQ	glycerophosphoryl diester phosphodiesterase	3.1.4.46	Metabolism--Lipid metabolism--Glycerophospholipid metabolism

[denovogenes]_109777	99.61	0.0	bvu:BVU_0413	K09861	K09861	uncharacterized protein	--	--

[denovogenes]_109780	74.50	6e-140	hsd:SD1D_1737	K06390	spoIIIAA	stage III sporulation protein AA	--	--

[denovogenes]_109781	43.36	3e-64	amt:Amet_2066	K17836	penP	beta-lactamase class A	3.5.2.6	Metabolism--Biosynthesis of other secondary metabolites--Penicillin and cephalosporin biosynthesis|Metabolism--Global and overview maps--Biosynthesis of antibiotics|Human Diseases--Drug resistance: Antimicrobial--beta-Lactam resistance

[denovogenes]_109782	76.38	2e-136	fpr:FP2_09410	K03205	virD4, lvhD4	type IV secretion system protein VirD4	--	Environmental Information Processing--Membrane transport--Bacterial secretion system

[denovogenes]_109784	40.00	1e-55	ag:AAF01818	K14250	oxyN, snoaM, dpsY, aknW, mtmY	cyclase	--	Metabolism--Metabolism of terpenoids and polyketides--Tetracycline biosynthesis|Metabolism--Metabolism of terpenoids and polyketides--Biosynthesis of type II polyketide products|Metabolism--Global and overview maps--Biosynthesis of antibiotics

[denovogenes]_109786	41.52	5e-50	paa:Paes_0622	K02050	ABC.SN.P	NitT/TauT family transport system permease protein	--	--

[denovogenes]_109787	57.87	3e-94	fpla:A4U99_07460	K02119	ATPVC, ntpC, atpC	V/A-type H+/Na+-transporting ATPase subunit C	--	Metabolism--Energy metabolism--Oxidative phosphorylation|Metabolism--Global and overview maps--Metabolic pathways

[denovogenes]_109788	43.51	2e-75	edu:LIU_10275	K08600	srtB	sortase B	3.4.22.70	--

[denovogenes]_109790	94.29	2e-170	fpla:A4U99_03250	K02557	motB	chemotaxis protein MotB	--	Cellular Processes--Cell motility--Bacterial chemotaxis|Cellular Processes--Cell motility--Flagellar assembly

[denovogenes]_109791	75.00	2e-130	ctc:CTC_02435	K00302	soxA	sarcosine oxidase, subunit alpha	1.5.3.1	Metabolism--Amino acid metabolism--Glycine, serine and threonine metabolism|Metabolism--Global and overview maps--Metabolic pathways

[denovogenes]_109793	100.00	0.0	btho:Btheta7330_00424	K06861	lptB	lipopolysaccharide export system ATP-binding protein	3.6.3.-	Environmental Information Processing--Membrane transport--ABC transporters

[denovogenes]_109794	41.32	5e-48	banm:EN10_07275	K01239	iunH	purine nucleosidase	3.2.2.1	Metabolism--Nucleotide metabolism--Purine metabolism|Metabolism--Metabolism of cofactors and vitamins--Nicotinate and nicotinamide metabolism|Metabolism--Global and overview maps--Metabolic pathways

[denovogenes]_109795	73.68	1e-122	ova:OBV_38560	K01779	racD	aspartate racemase	5.1.1.13	Metabolism--Amino acid metabolism--Alanine, aspartate and glutamate metabolism|Metabolism--Metabolism of terpenoids and polyketides--Nonribosomal peptide structures

[denovogenes]_109798	100.00	0.0	bvu:BVU_0525	K03269	lpxH	UDP-2,3-diacylglucosamine hydrolase	3.6.1.54	Metabolism--Glycan biosynthesis and metabolism--Lipopolysaccharide biosynthesis|Metabolism--Global and overview maps--Metabolic pathways

[denovogenes]_109799	75.29	2e-137	fpla:A4U99_14805	K02003	ABC.CD.A	putative ABC transport system ATP-binding protein	--	--

[denovogenes]_10980	46.65	0.0	ngk:NGK_1090	K12071	traD	conjugal transfer pilus assembly protein TraD	--	--

[denovogenes]_109804	98.39	9e-178	cfd:CFNIH1_01590	K09807	K09807	uncharacterized protein	--	--

[denovogenes]_109806	87.84	6e-162	kvq:SP68_00735	K01692	paaF, echA	enoyl-CoA hydratase	4.2.1.17	Metabolism--Lipid metabolism--Fatty acid degradation|Metabolism--Amino acid metabolism--Valine, leucine and isoleucine degradation|Metabolism--Metabolism of terpenoids and polyketides--Geraniol degradation|Metabolism--Amino acid metabolism--Lysine degradation|Metabolism--Amino acid metabolism--Phenylalanine metabolism|Metabolism--Xenobiotics biodegradation and metabolism--Benzoate degradation|Metabolism--Amino acid metabolism--Tryptophan metabolism|Metabolism--Metabolism of other amino acids--beta-Alanine metabolism|Metabolism--Xenobiotics biodegradation and metabolism--Aminobenzoate degradation|Metabolism--Carbohydrate metabolism--Propanoate metabolism|Metabolism--Carbohydrate metabolism--Butanoate metabolism|Metabolism--Metabolism of terpenoids and polyketides--Limonene and pinene degradation|Metabolism--Xenobiotics biodegradation and metabolism--Caprolactam degradation|Metabolism--Global and overview maps--Metabolic pathways|Metabolism--Global and overview maps--Biosynthesis of secondary metabolites|Metabolism--Global and overview maps--Microbial metabolism in diverse environments|Metabolism--Global and overview maps--Biosynthesis of antibiotics|Metabolism--Global and overview maps--Fatty acid metabolism

[denovogenes]_109807	62.45	8e-113	eel:EUBELI_01151	K01735	aroB	3-dehydroquinate synthase	4.2.3.4	Metabolism--Amino acid metabolism--Phenylalanine, tyrosine and tryptophan biosynthesis|Metabolism--Global and overview maps--Metabolic pathways|Metabolism--Global and overview maps--Biosynthesis of secondary metabolites|Metabolism--Global and overview maps--Biosynthesis of antibiotics|Metabolism--Global and overview maps--Biosynthesis of amino acids

[denovogenes]_109809	99.61	0.0	elf:LF82_0973	K00076	hdhA	7-alpha-hydroxysteroid dehydrogenase	1.1.1.159	--

[denovogenes]_109811	93.33	4e-170	fpla:A4U99_03205	K02411	fliH	flagellar assembly protein FliH	--	Cellular Processes--Cell motility--Flagellar assembly

[denovogenes]_109813	76.49	5e-138	ibu:IB211_01186c	K09817	znuC	zinc transport system ATP-binding protein	3.6.3.-	Environmental Information Processing--Membrane transport--ABC transporters

[denovogenes]_109814	72.05	4e-136	awo:Awo_c30350	K02003	ABC.CD.A	putative ABC transport system ATP-binding protein	--	--

[denovogenes]_109816	100.00	0.0	fpla:A4U99_01345	K03218	rlmB	23S rRNA (guanosine2251-2'-O)-methyltransferase	2.1.1.185	--

[denovogenes]_109817	99.61	0.0	sbc:SbBS512_E3864	K01999	livK	branched-chain amino acid transport system substrate-binding protein	--	Environmental Information Processing--Membrane transport--ABC transporters|Cellular Processes--Cellular community - prokaryotes--Quorum sensing

[denovogenes]_109819	56.35	3e-103	mas:Mahau_2745	K07045	K07045	uncharacterized protein	--	--

[denovogenes]_10982	100.00	0.0	efi:OG1RF_10657	K01874	MARS, metG	methionyl-tRNA synthetase	6.1.1.10	Metabolism--Metabolism of other amino acids--Selenocompound metabolism|Genetic Information Processing--Translation--Aminoacyl-tRNA biosynthesis

[denovogenes]_109820	56.13	1e-101	bprs:CK3_17600	K03525	coaX	type III pantothenate kinase	2.7.1.33	Metabolism--Metabolism of cofactors and vitamins--Pantothenate and CoA biosynthesis|Metabolism--Global and overview maps--Metabolic pathways

[denovogenes]_109821	54.28	2e-84	cpro:CPRO_14940	K02030	ABC.PA.S	polar amino acid transport system substrate-binding protein	--	--

[denovogenes]_109823	99.61	3e-177	fpla:A4U99_04100	K02233	E2.7.8.26, cobS, cobV	adenosylcobinamide-GDP ribazoletransferase	2.7.8.26	Metabolism--Metabolism of cofactors and vitamins--Porphyrin and chlorophyll metabolism|Metabolism--Global and overview maps--Metabolic pathways

[denovogenes]_109825	82.59	1e-156	lacy:A4V08_19695	K04069	pflA, pflC, pflE	pyruvate formate lyase activating enzyme	1.97.1.4	--

[denovogenes]_109826	41.77	3e-66	tur:AT726_09380	K07027	K07027	glycosyltransferase 2 family protein	--	--

[denovogenes]_109828	72.55	3e-135	bdh:GV66_02775	K01929	murF	UDP-N-acetylmuramoyl-tripeptide--D-alanyl-D-alanine ligase	6.3.2.10	Metabolism--Amino acid metabolism--Lysine biosynthesis|Metabolism--Glycan biosynthesis and metabolism--Peptidoglycan biosynthesis|Metabolism--Global and overview maps--Metabolic pathways|Human Diseases--Drug resistance: Antimicrobial--Vancomycin resistance

[denovogenes]_109829	52.83	5e-28	bsu:BSU20670	K07741	antB	anti-repressor protein	--	--

[denovogenes]_10983	99.85	0.0	ene:ENT_25810	K03483	mtlR	mannitol operon transcriptional antiterminator	--	--

[denovogenes]_109830	91.76	2e-162	lacy:A4V08_32885	K03525	coaX	type III pantothenate kinase	2.7.1.33	Metabolism--Metabolism of cofactors and vitamins--Pantothenate and CoA biosynthesis|Metabolism--Global and overview maps--Metabolic pathways

[denovogenes]_109833	95.31	4e-167	fpla:A4U99_07260	K01885	EARS, gltX	glutamyl-tRNA synthetase	6.1.1.17	Metabolism--Metabolism of cofactors and vitamins--Porphyrin and chlorophyll metabolism|Genetic Information Processing--Translation--Aminoacyl-tRNA biosynthesis|Metabolism--Global and overview maps--Metabolic pathways|Metabolism--Global and overview maps--Biosynthesis of secondary metabolites

[denovogenes]_109834	84.31	4e-155	ova:OBV_07690	K07160	K07160	UPF0271 protein	--	--

[denovogenes]_109837	83.86	2e-155	lacy:A4V08_33315	K11358	yhdR	aspartate aminotransferase	2.6.1.1	Metabolism--Amino acid metabolism--Arginine biosynthesis|Metabolism--Amino acid metabolism--Alanine, aspartate and glutamate metabolism|Metabolism--Amino acid metabolism--Cysteine and methionine metabolism|Metabolism--Amino acid metabolism--Arginine and proline metabolism|Metabolism--Amino acid metabolism--Tyrosine metabolism|Metabolism--Amino acid metabolism--Phenylalanine metabolism|Metabolism--Amino acid metabolism--Phenylalanine, tyrosine and tryptophan biosynthesis|Metabolism--Biosynthesis of other secondary metabolites--Novobiocin biosynthesis|Metabolism--Biosynthesis of other secondary metabolites--Isoquinoline alkaloid biosynthesis|Metabolism--Biosynthesis of other secondary metabolites--Tropane, piperidine and pyridine alkaloid biosynthesis|Metabolism--Global and overview maps--Metabolic pathways|Metabolism--Global and overview maps--Biosynthesis of secondary metabolites|Metabolism--Global and overview maps--Biosynthesis of antibiotics|Metabolism--Global and overview maps--2-Oxocarboxylic acid metabolism|Metabolism--Global and overview maps--Biosynthesis of amino acids

[denovogenes]_109838	59.68	4e-99	hsd:SD1D_0636	K03768	PPIB, ppiB	peptidyl-prolyl cis-trans isomerase B (cyclophilin B)	5.2.1.8	--

[denovogenes]_109839	54.84	4e-97	psim:KR76_11610	K01995	livG	branched-chain amino acid transport system ATP-binding protein	--	Environmental Information Processing--Membrane transport--ABC transporters|Cellular Processes--Cellular community - prokaryotes--Quorum sensing

[denovogenes]_10984	100.00	0.0	bfr:BF4102	K07386	pepO	putative endopeptidase	3.4.24.-	--

[denovogenes]_109842	70.83	8e-114	shi:Shel_09640	K06178	rluB	23S rRNA pseudouridine2605 synthase	5.4.99.22	--

[denovogenes]_109843	88.58	7e-161	cls:CXIVA_17290	K03149	thiG	thiazole synthase	2.8.1.10	Metabolism--Metabolism of cofactors and vitamins--Thiamine metabolism|Metabolism--Global and overview maps--Metabolic pathways

[denovogenes]_109845	100.00	4e-180	ecr:ECIAI1_0974	K15555	ssuB	sulfonate transport system ATP-binding protein	3.6.3.-	Metabolism--Energy metabolism--Sulfur metabolism|Environmental Information Processing--Membrane transport--ABC transporters

[denovogenes]_109846	95.80	1e-159	fpla:A4U99_05850	K01990	ABC-2.A	ABC-2 type transport system ATP-binding protein	--	--

[denovogenes]_109847	100.00	0.0	shq:A0259_06750	K05966	citG	triphosphoribosyl-dephospho-CoA synthase	2.4.2.52	Environmental Information Processing--Signal transduction--Two-component system

[denovogenes]_10985	94.68	0.0	seeb:SEEB0189_000225	K03497	parB, spo0J	chromosome partitioning protein, ParB family	--	--

[denovogenes]_109851	92.55	1e-172	cfd:CFNIH1_19570	K00928	lysC	aspartate kinase	2.7.2.4	Metabolism--Amino acid metabolism--Glycine, serine and threonine metabolism|Metabolism--Biosynthesis of other secondary metabolites--Monobactam biosynthesis|Metabolism--Amino acid metabolism--Cysteine and methionine metabolism|Metabolism--Amino acid metabolism--Lysine biosynthesis|Metabolism--Global and overview maps--Metabolic pathways|Metabolism--Global and overview maps--Biosynthesis of secondary metabolites|Metabolism--Global and overview maps--Microbial metabolism in diverse environments|Metabolism--Global and overview maps--Biosynthesis of antibiotics|Metabolism--Global and overview maps--2-Oxocarboxylic acid metabolism|Metabolism--Global and overview maps--Biosynthesis of amino acids

[denovogenes]_109852	73.73	6e-129	ccel:CCDG5_0209	K03040	rpoA	DNA-directed RNA polymerase subunit alpha	2.7.7.6	Metabolism--Nucleotide metabolism--Purine metabolism|Metabolism--Nucleotide metabolism--Pyrimidine metabolism|Metabolism--Global and overview maps--Metabolic pathways|Genetic Information Processing--Transcription--RNA polymerase

[denovogenes]_109853	70.08	9e-134	cck:Ccar_03205	K02003	ABC.CD.A	putative ABC transport system ATP-binding protein	--	--

[denovogenes]_109855	89.80	5e-158	bvu:BVU_4178	K06889	K06889	uncharacterized protein	--	--

[denovogenes]_109857	92.94	4e-172	bvu:BVU_0099	K00677	lpxA	UDP-N-acetylglucosamine acyltransferase	2.3.1.129	Metabolism--Glycan biosynthesis and metabolism--Lipopolysaccharide biosynthesis|Metabolism--Global and overview maps--Metabolic pathways|Human Diseases--Drug resistance: Antimicrobial--Cationic antimicrobial peptide (CAMP) resistance

[denovogenes]_109859	100.00	0.0	fpla:A4U99_14805	K02003	ABC.CD.A	putative ABC transport system ATP-binding protein	--	--

[denovogenes]_10986	99.26	0.0	cbut:ATN24_07425	K03655	recG	ATP-dependent DNA helicase RecG	3.6.4.12	Genetic Information Processing--Replication and repair--Homologous recombination

[denovogenes]_109862	58.59	8e-115	clb:Clo1100_0242	K01990	ABC-2.A	ABC-2 type transport system ATP-binding protein	--	--

[denovogenes]_109863	40.30	9e-50	yee:YE5303_13101	K16089	TC.FEV.OM2, cirA, cfrA, hmuR	outer membrane receptor for ferrienterochelin and colicins	--	--

[denovogenes]_109865	72.66	1e-133	lacy:A4V08_02665	K05833	ABC.X4.A	putative ABC transport system ATP-binding protein	--	--

[denovogenes]_109866	67.06	7e-118	anx:ACH33_12915	K16201	dppC	dipeptide transport system permease protein	--	Environmental Information Processing--Membrane transport--ABC transporters

[denovogenes]_109867	83.07	1e-153	ova:OBV_40750	K03521	fixA, etfB	electron transfer flavoprotein beta subunit	--	--

[denovogenes]_109869	50.20	3e-79	ibu:IB211_02587	K07699	spo0A	two-component system, response regulator, stage 0 sporulation protein A	--	Environmental Information Processing--Signal transduction--Two-component system|Cellular Processes--Cellular community - prokaryotes--Quorum sensing

[denovogenes]_10987	59.89	0.0	cbt:CLH_0781	K02407	fliD	flagellar hook-associated protein 2	--	Cellular Processes--Cell motility--Flagellar assembly

[denovogenes]_109870	84.40	9e-154	cpf:CPF_2496	K01409	KAE1, tsaD, QRI7	N6-L-threonylcarbamoyladenine synthase	2.3.1.234	--

[denovogenes]_109872	57.58	2e-92	fpa:FPR_05180	K00962	pnp, PNPT1	polyribonucleotide nucleotidyltransferase	2.7.7.8	Metabolism--Nucleotide metabolism--Purine metabolism|Metabolism--Nucleotide metabolism--Pyrimidine metabolism|Genetic Information Processing--Folding, sorting and degradation--RNA degradation

[denovogenes]_109876	45.38	2e-62	btb:BMB171_C2229	K18149	pbp5, pbpC, pbp3	penicillin-binding protein	--	Human Diseases--Drug resistance: Antimicrobial--beta-Lactam resistance

[denovogenes]_109878	99.61	0.0	cif:AL515_18175	K12266	norR	anaerobic nitric oxide reductase transcription regulator	--	Human Diseases--Infectious diseases: Bacterial--Salmonella infection

[denovogenes]_10988	54.12	0.0	bcel:BcellWH2_01938	K07001	K07001	NTE family protein	--	--

[denovogenes]_109881	45.10	4e-62	pdf:CD630DERM_24930	K03833	selB, EEFSEC	selenocysteine-specific elongation factor	--	--

[denovogenes]_109882	87.11	2e-167	eoc:CE10_1182	K16088	TC.FEV.OM1, fhuE, fpvA, fptA	outer-membrane receptor for ferric coprogen and ferric-rhodotorulic acid	--	--

[denovogenes]_109883	70.70	2e-124	cle:Clole_2330	K03588	ftsW, spoVE	cell division protein FtsW	--	Cellular Processes--Cell growth and death--Cell cycle - Caulobacter

[denovogenes]_109884	75.00	4e-132	erb:A4V01_18805	K02761	PTS-Cel-EIIC, celB, chbC	PTS system, cellobiose-specific IIC component	--	Metabolism--Carbohydrate metabolism--Starch and sucrose metabolism|Environmental Information Processing--Membrane transport--Phosphotransferase system (PTS)

[denovogenes]_109886	59.32	4e-94	awo:Awo_c01990	K02669	pilT	twitching motility protein PilT	--	--

[denovogenes]_109887	63.32	7e-105	gfe:Gferi_16225	K06714	rocR	arginine utilization regulatory protein	--	--

[denovogenes]_10989	75.00	0.0	ain:Acin_0095	K03702	uvrB	excinuclease ABC subunit B	--	Genetic Information Processing--Replication and repair--Nucleotide excision repair

[denovogenes]_109890	65.76	5e-116	clo:HMPREF0868_0592	K13607	fldA	cinnamoyl-CoA:phenyllactate CoA-transferase	2.8.3.17	Metabolism--Biosynthesis of other secondary metabolites--Tropane, piperidine and pyridine alkaloid biosynthesis

[denovogenes]_109892	63.53	1e-103	sfo:Z042_09695	K08368	yaaU	MFS transporter, putative metabolite transport protein	--	--

[denovogenes]_109894	46.03	1e-70	kok:KONIH1_22645	K07347	fimD, fimC, mrkC, htrE, cssD	outer membrane usher protein	--	Human Diseases--Infectious diseases: Bacterial--Pertussis

[denovogenes]_109895	82.42	1e-159	lacy:A4V08_02415	K07406	melA	alpha-galactosidase	3.2.1.22	Metabolism--Carbohydrate metabolism--Galactose metabolism|Metabolism--Lipid metabolism--Glycerolipid metabolism|Metabolism--Lipid metabolism--Sphingolipid metabolism|Metabolism--Glycan biosynthesis and metabolism--Glycosphingolipid biosynthesis - globo and isoglobo series

[denovogenes]_109896	92.97	4e-174	cle:Clole_0464	K01999	livK	branched-chain amino acid transport system substrate-binding protein	--	Environmental Information Processing--Membrane transport--ABC transporters|Cellular Processes--Cellular community - prokaryotes--Quorum sensing

[denovogenes]_109897	55.56	9e-77	mla:Mlab_0592	K12267	msrAB	peptide methionine sulfoxide reductase msrA/msrB	1.8.4.11 1.8.4.12	--

[denovogenes]_109900	94.51	8e-177	pfre:RM25_2215	K01126	E3.1.4.46, glpQ, ugpQ	glycerophosphoryl diester phosphodiesterase	3.1.4.46	Metabolism--Lipid metabolism--Glycerophospholipid metabolism

[denovogenes]_109901	93.68	4e-167	pfr:PFREUD_24000	K02013	ABC.FEV.A	iron complex transport system ATP-binding protein	3.6.3.34	Environmental Information Processing--Membrane transport--ABC transporters

[denovogenes]_109902	98.80	3e-180	pfre:RM25_1248	K00241	sdhC, frdC	succinate dehydrogenase / fumarate reductase, cytochrome b subunit	--	Metabolism--Carbohydrate metabolism--Citrate cycle (TCA cycle)|Metabolism--Energy metabolism--Oxidative phosphorylation|Metabolism--Carbohydrate metabolism--Butanoate metabolism|Metabolism--Energy metabolism--Carbon fixation pathways in prokaryotes|Metabolism--Global and overview maps--Metabolic pathways|Metabolism--Global and overview maps--Biosynthesis of secondary metabolites|Metabolism--Global and overview maps--Microbial metabolism in diverse environments|Metabolism--Global and overview maps--Biosynthesis of antibiotics|Metabolism--Global and overview maps--Carbon metabolism

[denovogenes]_109903	69.65	9e-115	pfre:RM25_1062	K03432	psmA, prcA	proteasome alpha subunit	3.4.25.1	Genetic Information Processing--Folding, sorting and degradation--Proteasome

[denovogenes]_109904	90.98	1e-173	pfre:RM25_0478	K00332	nuoC	NADH-quinone oxidoreductase subunit C	1.6.5.3	Metabolism--Energy metabolism--Oxidative phosphorylation|Metabolism--Global and overview maps--Metabolic pathways

[denovogenes]_109906	52.02	4e-61	mio:AOA12_07565	K00941	thiD	hydroxymethylpyrimidine/phosphomethylpyrimidine kinase	2.7.1.49 2.7.4.7	Metabolism--Metabolism of cofactors and vitamins--Thiamine metabolism|Metabolism--Global and overview maps--Metabolic pathways

[denovogenes]_109907	40.81	7e-52	rti:DC20_20765	K05946	E2.4.1.187, tagA	N-acetylglucosaminyldiphosphoundecaprenol N-acetyl-beta-D-mannosaminyltransferase	2.4.1.187	Cellular Processes--Cellular community - prokaryotes--Biofilm formation - Vibrio cholerae

[denovogenes]_109909	76.59	1e-136	pfre:RM25_0279	K00065	kduD	2-deoxy-D-gluconate 3-dehydrogenase	1.1.1.125	Metabolism--Carbohydrate metabolism--Pentose and glucuronate interconversions

[denovogenes]_10991	100.00	0.0	bth:BT_2424	K01547	kdpB	K+-transporting ATPase ATPase B chain	3.6.3.12	Environmental Information Processing--Signal transduction--Two-component system

[denovogenes]_109911	40.00	2e-19	btm:MC28_2716	K21029	moeB	molybdopterin-synthase adenylyltransferase	2.7.7.80	Genetic Information Processing--Folding, sorting and degradation--Sulfur relay system

[denovogenes]_109913	99.61	0.0	btho:Btheta7330_03903	K00677	lpxA	UDP-N-acetylglucosamine acyltransferase	2.3.1.129	Metabolism--Glycan biosynthesis and metabolism--Lipopolysaccharide biosynthesis|Metabolism--Global and overview maps--Metabolic pathways|Human Diseases--Drug resistance: Antimicrobial--Cationic antimicrobial peptide (CAMP) resistance

[denovogenes]_109915	83.00	2e-157	pdi:BDI_0452	K03437	spoU	RNA methyltransferase, TrmH family	--	--

[denovogenes]_109917	84.86	2e-166	pdi:BDI_1111	K01142	E3.1.11.2, xthA	exodeoxyribonuclease III	3.1.11.2	Genetic Information Processing--Replication and repair--Base excision repair

[denovogenes]_109918	46.59	2e-35	palk:PSAKL28_46660	K21021	tpbB	diguanylate cyclase	2.7.7.65	Cellular Processes--Cellular community - prokaryotes--Biofilm formation - Pseudomonas aeruginosa

[denovogenes]_109920	69.80	2e-125	tpi:TREPR_0033	K00772	mtaP, MTAP	5'-methylthioadenosine phosphorylase	2.4.2.28	Metabolism--Amino acid metabolism--Cysteine and methionine metabolism|Metabolism--Global and overview maps--Metabolic pathways

[denovogenes]_109921	65.70	8e-114	lir:LAW_00462	K07164	K07164	uncharacterized protein	--	--

[denovogenes]_109922	73.41	8e-138	cah:CAETHG_3506	K02003	ABC.CD.A	putative ABC transport system ATP-binding protein	--	--

[denovogenes]_109926	99.22	0.0	fpla:A4U99_03205	K02411	fliH	flagellar assembly protein FliH	--	Cellular Processes--Cell motility--Flagellar assembly

[denovogenes]_109927	100.00	0.0	fpla:A4U99_03250	K02557	motB	chemotaxis protein MotB	--	Cellular Processes--Cell motility--Bacterial chemotaxis|Cellular Processes--Cell motility--Flagellar assembly

[denovogenes]_109928	97.60	1e-180	fpla:A4U99_01915	K01265	map	methionyl aminopeptidase	3.4.11.18	--

[denovogenes]_109929	41.39	9e-63	pgi:PG_0113	K00662	aacC	aminoglycoside 3-N-acetyltransferase	2.3.1.81	--

[denovogenes]_10993	51.10	3e-153	dhd:Dhaf_4621	K00336	nuoG	NADH-quinone oxidoreductase subunit G	1.6.5.3	Metabolism--Energy metabolism--Oxidative phosphorylation|Metabolism--Global and overview maps--Metabolic pathways

[denovogenes]_109930	98.82	0.0	cfd:CFNIH1_21310	K08354	phsC	thiosulfate reductase cytochrome b subunit	--	Metabolism--Energy metabolism--Sulfur metabolism|Metabolism--Global and overview maps--Microbial metabolism in diverse environments

[denovogenes]_109931	98.82	0.0	fpla:A4U99_00915	K03424	--	--	--	--

[denovogenes]_109932	47.81	7e-76	ccb:Clocel_2195	K18431	legF, ptmB	CMP-N,N'-diacetyllegionaminic acid synthase	2.7.7.82	Metabolism--Carbohydrate metabolism--Amino sugar and nucleotide sugar metabolism

[denovogenes]_109934	100.00	0.0	paeo:M801_5066	K03496	parA, soj	chromosome partitioning protein	--	--

[denovogenes]_109935	100.00	0.0	paeo:M801_1108	K01995	livG	branched-chain amino acid transport system ATP-binding protein	--	Environmental Information Processing--Membrane transport--ABC transporters|Cellular Processes--Cellular community - prokaryotes--Quorum sensing

[denovogenes]_109936	87.06	4e-165	koc:AB185_13100	K00046	idnO	gluconate 5-dehydrogenase	1.1.1.69	--

[denovogenes]_109938	100.00	4e-177	paeo:M801_4734	K02013	ABC.FEV.A	iron complex transport system ATP-binding protein	3.6.3.34	Environmental Information Processing--Membrane transport--ABC transporters

[denovogenes]_109939	96.08	0.0	bfb:VU15_03220	K03787	surE	5'-nucleotidase	3.1.3.5	Metabolism--Nucleotide metabolism--Purine metabolism|Metabolism--Nucleotide metabolism--Pyrimidine metabolism|Metabolism--Metabolism of cofactors and vitamins--Nicotinate and nicotinamide metabolism|Metabolism--Global and overview maps--Metabolic pathways|Metabolism--Global and overview maps--Biosynthesis of secondary metabolites

[denovogenes]_109940	100.00	0.0	bfg:BF638R_0739	K03496	parA, soj	chromosome partitioning protein	--	--

[denovogenes]_109941	66.95	9e-109	dpr:Despr_0246	K09817	znuC	zinc transport system ATP-binding protein	3.6.3.-	Environmental Information Processing--Membrane transport--ABC transporters

[denovogenes]_109944	48.62	5e-80	dfi:AXF13_09695	K01779	racD	aspartate racemase	5.1.1.13	Metabolism--Amino acid metabolism--Alanine, aspartate and glutamate metabolism|Metabolism--Metabolism of terpenoids and polyketides--Nonribosomal peptide structures

[denovogenes]_109945	99.22	0.0	fpla:A4U99_11135	K02013	ABC.FEV.A	iron complex transport system ATP-binding protein	3.6.3.34	Environmental Information Processing--Membrane transport--ABC transporters

[denovogenes]_109946	96.86	2e-174	cfd:CFNIH1_14735	K15555	ssuB	sulfonate transport system ATP-binding protein	3.6.3.-	Metabolism--Energy metabolism--Sulfur metabolism|Environmental Information Processing--Membrane transport--ABC transporters

[denovogenes]_109947	63.89	6e-115	dda:Dd703_0033	K15373	isfD	sulfoacetaldehyde reductase	1.1.1.313	Metabolism--Metabolism of other amino acids--Taurine and hypotaurine metabolism|Metabolism--Global and overview maps--Metabolic pathways

[denovogenes]_10995	92.10	0.0	bhl:Bache_1090	K01712	hutU, UROC1	urocanate hydratase	4.2.1.49	Metabolism--Amino acid metabolism--Histidine metabolism|Metabolism--Global and overview maps--Metabolic pathways

[denovogenes]_109955	99.22	0.0	cfd:CFNIH1_11910	K10831	tauB	taurine transport system ATP-binding protein	3.6.3.36	Metabolism--Energy metabolism--Sulfur metabolism|Environmental Information Processing--Membrane transport--ABC transporters

[denovogenes]_109957	46.99	3e-78	pri:PRIO_1155	K00662	aacC	aminoglycoside 3-N-acetyltransferase	2.3.1.81	--

[denovogenes]_109958	81.96	6e-162	dvg:Deval_0637	K00184	K00184	prokaryotic molybdopterin-containing oxidoreductase family, iron-sulfur binding subunit	--	--

[denovogenes]_109959	99.61	0.0	fpla:A4U99_09595	K00215	dapB	4-hydroxy-tetrahydrodipicolinate reductase	1.17.1.8	Metabolism--Biosynthesis of other secondary metabolites--Monobactam biosynthesis|Metabolism--Amino acid metabolism--Lysine biosynthesis|Metabolism--Global and overview maps--Metabolic pathways|Metabolism--Global and overview maps--Biosynthesis of secondary metabolites|Metabolism--Global and overview maps--Microbial metabolism in diverse environments|Metabolism--Global and overview maps--Biosynthesis of antibiotics|Metabolism--Global and overview maps--Biosynthesis of amino acids

[denovogenes]_109960	40.68	1e-61	rum:CK1_02070	K04759	feoB	ferrous iron transport protein B	--	--

[denovogenes]_109962	99.61	0.0	cfd:CFNIH1_09105	K00046	idnO	gluconate 5-dehydrogenase	1.1.1.69	--

[denovogenes]_109963	100.00	0.0	cif:AL515_18230	K15832	hycG	formate hydrogenlyase subunit 7	--	--

[denovogenes]_109964	47.27	1e-57	tpt:Tpet_0904	K00059	fabG	3-oxoacyl-[acyl-carrier protein] reductase	1.1.1.100	Metabolism--Lipid metabolism--Fatty acid biosynthesis|Metabolism--Metabolism of cofactors and vitamins--Biotin metabolism|Metabolism--Lipid metabolism--Biosynthesis of unsaturated fatty acids|Metabolism--Global and overview maps--Metabolic pathways|Metabolism--Global and overview maps--Fatty acid metabolism

[denovogenes]_109966	85.88	1e-159	lacy:A4V08_20955	K06206	sfsA	sugar fermentation stimulation protein A	--	--

[denovogenes]_109968	100.00	2e-173	fpla:A4U99_07290	K03091	SIG3.4	RNA polymerase sporulation-specific sigma factor	--	--

[denovogenes]_10997	100.00	0.0	bvu:BVU_2092	K04084	dsbD	thiol:disulfide interchange protein DsbD	1.8.1.8	--

[denovogenes]_109970	100.00	0.0	paeo:M801_5315	K02030	ABC.PA.S	polar amino acid transport system substrate-binding protein	--	--

[denovogenes]_109973	43.37	2e-71	nko:Niako_6782	K13002	wbyL	glycosyltransferase	2.4.1.-	--

[denovogenes]_109977	41.94	7e-59	ttk:TST_1353	K00059	fabG	3-oxoacyl-[acyl-carrier protein] reductase	1.1.1.100	Metabolism--Lipid metabolism--Fatty acid biosynthesis|Metabolism--Metabolism of cofactors and vitamins--Biotin metabolism|Metabolism--Lipid metabolism--Biosynthesis of unsaturated fatty acids|Metabolism--Global and overview maps--Metabolic pathways|Metabolism--Global and overview maps--Fatty acid metabolism

[denovogenes]_109978	43.04	9e-53	gct:GC56T3_2040	K16164	K16164	acylpyruvate hydrolase	3.7.1.5	Metabolism--Amino acid metabolism--Tyrosine metabolism|Metabolism--Global and overview maps--Metabolic pathways|Metabolism--Global and overview maps--Microbial metabolism in diverse environments

[denovogenes]_109979	75.49	2e-130	fpla:A4U99_07355	K07090	K07090	uncharacterized protein	--	--

[denovogenes]_10998	96.45	0.0	bxy:BXY_09360	K01547	kdpB	K+-transporting ATPase ATPase B chain	3.6.3.12	Environmental Information Processing--Signal transduction--Two-component system

[denovogenes]_109981	57.09	2e-98	ccel:CCDG5_1308	K00757	udp, UPP	uridine phosphorylase	2.4.2.3	Metabolism--Nucleotide metabolism--Pyrimidine metabolism|Metabolism--Xenobiotics biodegradation and metabolism--Drug metabolism - other enzymes|Metabolism--Global and overview maps--Metabolic pathways

[denovogenes]_109982	81.10	3e-156	rix:RO1_23550	K02470	gyrB	DNA gyrase subunit B	5.99.1.3	--

[denovogenes]_109984	67.07	2e-122	pdi:BDI_2563	K03524	birA	BirA family transcriptional regulator, biotin operon repressor / biotin-[acetyl-CoA-carboxylase] ligase	6.3.4.15	Metabolism--Metabolism of cofactors and vitamins--Biotin metabolism|Metabolism--Global and overview maps--Metabolic pathways

[denovogenes]_109985	96.08	2e-166	pdi:BDI_1811	K06282	hyaA, hybO	hydrogenase small subunit	1.12.99.6	Metabolism--Xenobiotics biodegradation and metabolism--Nitrotoluene degradation|Metabolism--Global and overview maps--Microbial metabolism in diverse environments

[denovogenes]_109986	48.61	7e-82	boc:BG90_3759	K00065	kduD	2-deoxy-D-gluconate 3-dehydrogenase	1.1.1.125	Metabolism--Carbohydrate metabolism--Pentose and glucuronate interconversions

[denovogenes]_109990	99.61	0.0	paeu:BN889_03267	K10017	hisP	histidine transport system ATP-binding protein	3.6.3.21	Environmental Information Processing--Membrane transport--ABC transporters

[denovogenes]_109991	100.00	3e-176	pael:T223_20635	K02224	cobB-cbiA	cobyrinic acid a,c-diamide synthase	6.3.5.9 6.3.5.11	Metabolism--Metabolism of cofactors and vitamins--Porphyrin and chlorophyll metabolism|Metabolism--Global and overview maps--Metabolic pathways

[denovogenes]_109992	81.97	4e-144	cso:CLS_28360	K02003	ABC.CD.A	putative ABC transport system ATP-binding protein	--	--

[denovogenes]_109994	62.06	3e-107	scc:Spico_0110	K01995	livG	branched-chain amino acid transport system ATP-binding protein	--	Environmental Information Processing--Membrane transport--ABC transporters|Cellular Processes--Cellular community - prokaryotes--Quorum sensing

[denovogenes]_109997	93.73	2e-176	lacy:A4V08_13910	K01470	E3.5.2.10	creatinine amidohydrolase	3.5.2.10	Metabolism--Amino acid metabolism--Arginine and proline metabolism

[denovogenes]_109999	100.00	0.0	sgi:SGRAN_1322	K11811	arsH	arsenical resistance protein ArsH	--	--

[denovogenes]_11	91.60	0.0	mmk:MU9_60	K13735	yeeJ	adhesin/invasin	--	Human Diseases--Infectious diseases: Bacterial--Bacterial invasion of epithelial cells

[denovogenes]_11000	100.00	0.0	btho:Btheta7330_02076	K03702	uvrB	excinuclease ABC subunit B	--	Genetic Information Processing--Replication and repair--Nucleotide excision repair

[denovogenes]_110000	88.35	2e-165	pdi:BDI_2591	K03269	lpxH	UDP-2,3-diacylglucosamine hydrolase	3.6.1.54	Metabolism--Glycan biosynthesis and metabolism--Lipopolysaccharide biosynthesis|Metabolism--Global and overview maps--Metabolic pathways

[denovogenes]_110004	68.62	2e-118	ddl:Desdi_0700	K19285	nfrA1	FMN reductase (NADPH)	1.5.1.38	Metabolism--Metabolism of cofactors and vitamins--Riboflavin metabolism|Metabolism--Global and overview maps--Metabolic pathways

[denovogenes]_110006	45.29	2e-37	brs:S23_50970	K00058	serA, PHGDH	D-3-phosphoglycerate dehydrogenase	1.1.1.95	Metabolism--Amino acid metabolism--Glycine, serine and threonine metabolism|Metabolism--Energy metabolism--Methane metabolism|Metabolism--Global and overview maps--Metabolic pathways|Metabolism--Global and overview maps--Microbial metabolism in diverse environments|Metabolism--Global and overview maps--Biosynthesis of antibiotics|Metabolism--Global and overview maps--Carbon metabolism|Metabolism--Global and overview maps--Biosynthesis of amino acids

[denovogenes]_110007	100.00	0.0	bbrd:BBBR_1742	K07046	K07046	L-fuconolactonase	3.1.1.-	Metabolism--Carbohydrate metabolism--Fructose and mannose metabolism|Metabolism--Global and overview maps--Microbial metabolism in diverse environments

[denovogenes]_110008	43.53	3e-67	ctc:CTC_01441	K00132	E1.2.1.10	acetaldehyde dehydrogenase (acetylating)	1.2.1.10	Metabolism--Carbohydrate metabolism--Pyruvate metabolism|Metabolism--Carbohydrate metabolism--Butanoate metabolism|Metabolism--Global and overview maps--Metabolic pathways|Metabolism--Global and overview maps--Microbial metabolism in diverse environments

[denovogenes]_11001	99.70	0.0	bxy:BXY_09360	K01547	kdpB	K+-transporting ATPase ATPase B chain	3.6.3.12	Environmental Information Processing--Signal transduction--Two-component system

[denovogenes]_110011	100.00	0.0	shq:A0259_03090	K02013	ABC.FEV.A	iron complex transport system ATP-binding protein	3.6.3.34	Environmental Information Processing--Membrane transport--ABC transporters

[denovogenes]_110013	52.16	6e-99	tpi:TREPR_0935	K02034	ABC.PE.P1	peptide/nickel transport system permease protein	--	Cellular Processes--Cellular community - prokaryotes--Quorum sensing

[denovogenes]_110014	48.81	4e-86	fpla:A4U99_00720	K19333	kdgR	IclR family transcriptional regulator, KDG regulon repressor	--	--

[denovogenes]_110015	40.33	1e-43	gps:C427_4833	K02014	TC.FEV.OM	iron complex outermembrane recepter protein	--	--

[denovogenes]_110018	83.92	2e-141	csh:Closa_2987	K02049	ABC.SN.A	NitT/TauT family transport system ATP-binding protein	--	--

[denovogenes]_110020	100.00	0.0	pdf:CD630DERM_32400	K10795	prdD	D-proline reductase (dithiol) PrdD	1.21.4.1	Metabolism--Amino acid metabolism--Arginine and proline metabolism

[denovogenes]_110023	99.61	0.0	cfd:CFNIH1_13525	K03288	citA, tcuC	MFS transporter, MHS family, citrate/tricarballylate:H+ symporter	--	--

[denovogenes]_110024	77.56	4e-143	shi:Shel_23250	K03525	coaX	type III pantothenate kinase	2.7.1.33	Metabolism--Metabolism of cofactors and vitamins--Pantothenate and CoA biosynthesis|Metabolism--Global and overview maps--Metabolic pathways

[denovogenes]_110025	42.13	1e-53	sab:SAB0544c	K00520	merA	mercuric reductase	1.16.1.1	--

[denovogenes]_110026	66.27	1e-109	gpa:GPA_15570	K01491	folD	methylenetetrahydrofolate dehydrogenase (NADP+) / methenyltetrahydrofolate cyclohydrolase	1.5.1.5 3.5.4.9	Metabolism--Metabolism of cofactors and vitamins--One carbon pool by folate|Metabolism--Energy metabolism--Carbon fixation pathways in prokaryotes|Metabolism--Global and overview maps--Metabolic pathways|Metabolism--Global and overview maps--Microbial metabolism in diverse environments|Metabolism--Global and overview maps--Carbon metabolism

[denovogenes]_110027	46.22	9e-62	cml:BN424_1792	K19285	nfrA1	FMN reductase (NADPH)	1.5.1.38	Metabolism--Metabolism of cofactors and vitamins--Riboflavin metabolism|Metabolism--Global and overview maps--Metabolic pathways

[denovogenes]_110028	89.84	6e-149	bfb:VU15_19450	K07791	dcuA	anaerobic C4-dicarboxylate transporter DcuA	--	--

[denovogenes]_110033	46.64	7e-68	pfn:HZ99_07450	K07165	fecR	transmembrane sensor	--	--

[denovogenes]_110034	100.00	0.0	pdi:BDI_2635	K03787	surE	5'-nucleotidase	3.1.3.5	Metabolism--Nucleotide metabolism--Purine metabolism|Metabolism--Nucleotide metabolism--Pyrimidine metabolism|Metabolism--Metabolism of cofactors and vitamins--Nicotinate and nicotinamide metabolism|Metabolism--Global and overview maps--Metabolic pathways|Metabolism--Global and overview maps--Biosynthesis of secondary metabolites

[denovogenes]_110035	57.32	2e-98	ag:AAS55722	K20680	fdtB	dTDP-3-amino-3,6-dideoxy-alpha-D-galactopyranose transaminase	2.6.1.90	Metabolism--Metabolism of terpenoids and polyketides--Polyketide sugar unit biosynthesis|Metabolism--Global and overview maps--Biosynthesis of antibiotics

[denovogenes]_110038	40.57	5e-17	bxh:BAXH7_04219	K13530	adaA	AraC family transcriptional regulator, regulatory protein of adaptative response / methylphosphotriester-DNA alkyltransferase methyltransferase	2.1.1.-	--

[denovogenes]_11004	77.22	0.0	rho:RHOM_07045	K02400	flhA	flagellar biosynthesis protein FlhA	--	Cellular Processes--Cell motility--Flagellar assembly

[denovogenes]_110040	91.76	7e-180	csh:Closa_2216	K01889	FARSA, pheS	phenylalanyl-tRNA synthetase alpha chain	6.1.1.20	Genetic Information Processing--Translation--Aminoacyl-tRNA biosynthesis

[denovogenes]_110043	58.96	2e-93	ral:Rumal_0708	K00558	DNMT1, dcm	DNA (cytosine-5)-methyltransferase 1	2.1.1.37	Metabolism--Amino acid metabolism--Cysteine and methionine metabolism|Metabolism--Global and overview maps--Metabolic pathways|Human Diseases--Cancers: Overview--MicroRNAs in cancer

[denovogenes]_110044	66.93	7e-123	hsd:SD1D_0177	K09697	natA	sodium transport system ATP-binding protein	3.6.3.7	Environmental Information Processing--Membrane transport--ABC transporters|Environmental Information Processing--Signal transduction--Two-component system

[denovogenes]_110047	72.37	2e-142	byl:A4V09_21605	K01915	glnA, GLUL	glutamine synthetase	6.3.1.2	Metabolism--Amino acid metabolism--Arginine biosynthesis|Metabolism--Amino acid metabolism--Alanine, aspartate and glutamate metabolism|Metabolism--Carbohydrate metabolism--Glyoxylate and dicarboxylate metabolism|Metabolism--Energy metabolism--Nitrogen metabolism|Metabolism--Global and overview maps--Metabolic pathways|Metabolism--Global and overview maps--Microbial metabolism in diverse environments|Metabolism--Global and overview maps--Biosynthesis of amino acids|Environmental Information Processing--Signal transduction--Two-component system|Organismal Systems--Nervous system--Glutamatergic synapse|Organismal Systems--Nervous system--GABAergic synapse

[denovogenes]_11005	57.69	0.0	lacy:A4V08_15835	K02755	PTS-Bgl-EIIA, bglF, bglP	PTS system, beta-glucoside-specific IIA component	2.7.1.-	Environmental Information Processing--Membrane transport--Phosphotransferase system (PTS)

[denovogenes]_11005	57.69	0.0	lacy:A4V08_15835	K02756	PTS-Bgl-EIIB, bglF, bglP	PTS system, beta-glucoside-specific IIB component	2.7.1.-	Environmental Information Processing--Membrane transport--Phosphotransferase system (PTS)

[denovogenes]_11005	57.69	0.0	lacy:A4V08_15835	K02757	PTS-Bgl-EIIC, bglF, bglP	PTS system, beta-glucoside-specific IIC component	--	Environmental Information Processing--Membrane transport--Phosphotransferase system (PTS)

[denovogenes]_110051	64.73	9e-106	rim:ROI_25190	K06182	rluF	23S rRNA pseudouridine2604 synthase	5.4.99.21	--

[denovogenes]_110054	48.34	1e-34	ag:AAK37619	K18218	tet35	tetracycline resistance efflux pump	--	--

[denovogenes]_110055	70.70	9e-132	csh:Closa_1148	K07483	K07483	transposase	--	--

[denovogenes]_110057	98.82	0.0	eci:UTI89_C4057	K21086	yhjH	c-di-GMP phosphodiesterase	3.1.4.52	Cellular Processes--Cellular community - prokaryotes--Biofilm formation - Escherichia coli

[denovogenes]_110058	80.31	4e-152	byl:A4V09_20780	K03424	--	--	--	--

[denovogenes]_110059	77.56	1e-140	lacy:A4V08_06850	K02519	infB, MTIF2	translation initiation factor IF-2	--	--

[denovogenes]_11006	54.71	0.0	bcel:BcellWH2_01185	K19049	cslA	chondroitin AC lyase	4.2.2.5	--

[denovogenes]_110060	100.00	0.0	cif:AL515_00920	K01624	FBA, fbaA	fructose-bisphosphate aldolase, class II	4.1.2.13	Metabolism--Carbohydrate metabolism--Glycolysis / Gluconeogenesis|Metabolism--Carbohydrate metabolism--Pentose phosphate pathway|Metabolism--Carbohydrate metabolism--Fructose and mannose metabolism|Metabolism--Energy metabolism--Methane metabolism|Metabolism--Energy metabolism--Carbon fixation in photosynthetic organisms|Metabolism--Global and overview maps--Metabolic pathways|Metabolism--Global and overview maps--Biosynthesis of secondary metabolites|Metabolism--Global and overview maps--Microbial metabolism in diverse environments|Metabolism--Global and overview maps--Biosynthesis of antibiotics|Metabolism--Global and overview maps--Carbon metabolism|Metabolism--Global and overview maps--Biosynthesis of amino acids

[denovogenes]_110061	51.52	2e-54	pri:PRIO_4803	K05989	ramA	alpha-L-rhamnosidase	3.2.1.40	--

[denovogenes]_110063	65.62	2e-117	bhu:bhn_I2738	K10112	msmX, msmK, malK, sugC, ggtA, msiK	multiple sugar transport system ATP-binding protein	--	Environmental Information Processing--Membrane transport--ABC transporters

[denovogenes]_110064	55.29	6e-91	byl:A4V09_01370	K10439	rbsB	ribose transport system substrate-binding protein	--	Environmental Information Processing--Membrane transport--ABC transporters|Cellular Processes--Cell motility--Bacterial chemotaxis

[denovogenes]_110065	97.25	4e-179	cfd:CFNIH1_13525	K03288	citA, tcuC	MFS transporter, MHS family, citrate/tricarballylate:H+ symporter	--	--

[denovogenes]_110067	82.75	2e-146	afd:Alfi_0170	K00800	aroA	3-phosphoshikimate 1-carboxyvinyltransferase	2.5.1.19	Metabolism--Amino acid metabolism--Phenylalanine, tyrosine and tryptophan biosynthesis|Metabolism--Global and overview maps--Metabolic pathways|Metabolism--Global and overview maps--Biosynthesis of secondary metabolites|Metabolism--Global and overview maps--Biosynthesis of antibiotics|Metabolism--Global and overview maps--Biosynthesis of amino acids

[denovogenes]_110069	57.21	1e-79	gym:GYMC10_0359	K02034	ABC.PE.P1	peptide/nickel transport system permease protein	--	Cellular Processes--Cellular community - prokaryotes--Quorum sensing

[denovogenes]_11007	88.92	0.0	bdh:GV66_10775	K04084	dsbD	thiol:disulfide interchange protein DsbD	1.8.1.8	--

[denovogenes]_110070	88.24	5e-167	csh:Closa_3899	K11069	potD	spermidine/putrescine transport system substrate-binding protein	--	Environmental Information Processing--Membrane transport--ABC transporters

[denovogenes]_110071	98.05	0.0	cif:AL515_01175	K13636	dsdC	LysR family transcriptional regulator, D-serine deaminase activator	--	--

[denovogenes]_110072	75.29	2e-134	lacy:A4V08_25450	K03654	recQ	ATP-dependent DNA helicase RecQ	3.6.4.12	Genetic Information Processing--Folding, sorting and degradation--RNA degradation

[denovogenes]_110073	52.38	4e-79	csh:Closa_2490	K03575	mutY	A/G-specific adenine glycosylase	3.2.2.-	Genetic Information Processing--Replication and repair--Base excision repair

[denovogenes]_110074	85.83	2e-161	serr:Ser39006_2309	K03694	clpA	ATP-dependent Clp protease ATP-binding subunit ClpA	--	--

[denovogenes]_110075	93.98	5e-171	lacy:A4V08_18805	K01876	DARS, aspS	aspartyl-tRNA synthetase	6.1.1.12	Genetic Information Processing--Translation--Aminoacyl-tRNA biosynthesis

[denovogenes]_110076	100.00	0.0	elp:P12B_c1565	K11216	lsrK	autoinducer-2 kinase	2.7.1.189	Cellular Processes--Cellular community - prokaryotes--Quorum sensing

[denovogenes]_110078	100.00	5e-176	paeo:M801_1825	K02303	cobA	uroporphyrin-III C-methyltransferase	2.1.1.107	Metabolism--Metabolism of cofactors and vitamins--Porphyrin and chlorophyll metabolism|Metabolism--Global and overview maps--Metabolic pathways|Metabolism--Global and overview maps--Biosynthesis of secondary metabolites

[denovogenes]_110080	77.87	3e-148	bcel:BcellWH2_04076	K07139	K07139	uncharacterized protein	--	--

[denovogenes]_110081	51.17	6e-83	rix:RO1_36780	K03723	mfd	transcription-repair coupling factor (superfamily II helicase)	3.6.4.-	Genetic Information Processing--Replication and repair--Nucleotide excision repair

[denovogenes]_110084	58.59	6e-106	eao:BD94_3762	K07456	mutS2	DNA mismatch repair protein MutS2	--	Genetic Information Processing--Replication and repair--Mismatch repair

[denovogenes]_110085	47.43	9e-76	pdi:BDI_0336	K03296	TC.HAE1	hydrophobic/amphiphilic exporter-1 (mainly G- bacteria), HAE1 family	--	--

[denovogenes]_110087	68.75	9e-125	csh:Closa_1859	K11749	rseP	regulator of sigma E protease	3.4.24.-	Cellular Processes--Cellular community - prokaryotes--Quorum sensing|Cellular Processes--Cell growth and death--Cell cycle - Caulobacter

[denovogenes]_110089	69.92	3e-116	eclg:EC036_23000	K14062	ompN	outer membrane protein N	--	--

[denovogenes]_110090	86.33	1e-162	ypd:YPD4_0330	K12573	rnr, vacB	ribonuclease R	3.1.-.-	Genetic Information Processing--Folding, sorting and degradation--RNA degradation

[denovogenes]_110098	94.53	0.0	cama:F384_03285	K04835	mal	methylaspartate ammonia-lyase	4.3.1.2	Metabolism--Carbohydrate metabolism--Glyoxylate and dicarboxylate metabolism|Metabolism--Carbohydrate metabolism--C5-Branched dibasic acid metabolism|Metabolism--Global and overview maps--Metabolic pathways|Metabolism--Global and overview maps--Carbon metabolism

[denovogenes]_1101	88.07	0.0	lacy:A4V08_14880	K03723	mfd	transcription-repair coupling factor (superfamily II helicase)	3.6.4.-	Genetic Information Processing--Replication and repair--Nucleotide excision repair

[denovogenes]_110101	63.81	6e-111	csh:Closa_3774	K09963	K09963	uncharacterized protein	--	--

[denovogenes]_110102	98.44	0.0	cif:AL515_03695	K06949	rsgA, engC	ribosome biogenesis GTPase / thiamine phosphate phosphatase	3.6.1.- 3.1.3.100	Metabolism--Metabolism of cofactors and vitamins--Thiamine metabolism|Metabolism--Global and overview maps--Metabolic pathways

[denovogenes]_110107	97.66	0.0	bxy:BXY_33090	K06885	K06885	uncharacterized protein	--	--

[denovogenes]_110109	75.20	4e-138	erb:A4V01_08945	K00876	udk, UCK	uridine kinase	2.7.1.48	Metabolism--Nucleotide metabolism--Pyrimidine metabolism|Metabolism--Xenobiotics biodegradation and metabolism--Drug metabolism - other enzymes|Metabolism--Global and overview maps--Metabolic pathways

[denovogenes]_11011	93.65	0.0	bdh:GV66_17620	K01547	kdpB	K+-transporting ATPase ATPase B chain	3.6.3.12	Environmental Information Processing--Signal transduction--Two-component system

[denovogenes]_110110	48.09	4e-78	axl:AXY_05280	K02123	ATPVI, ntpI, atpI	V/A-type H+/Na+-transporting ATPase subunit I	--	Metabolism--Energy metabolism--Oxidative phosphorylation|Metabolism--Global and overview maps--Metabolic pathways

[denovogenes]_110117	72.05	4e-134	cbn:CbC4_0468	K02003	ABC.CD.A	putative ABC transport system ATP-binding protein	--	--

[denovogenes]_110119	50.00	1e-87	bprl:CL2_23100	K02013	ABC.FEV.A	iron complex transport system ATP-binding protein	3.6.3.34	Environmental Information Processing--Membrane transport--ABC transporters

[denovogenes]_11012	85.82	0.0	pdi:BDI_0687	K11381	bkdA	2-oxoisovalerate dehydrogenase E1 component	1.2.4.4	Metabolism--Amino acid metabolism--Valine, leucine and isoleucine degradation|Metabolism--Carbohydrate metabolism--Propanoate metabolism|Metabolism--Global and overview maps--Metabolic pathways|Metabolism--Global and overview maps--Biosynthesis of secondary metabolites|Metabolism--Global and overview maps--Biosynthesis of antibiotics

[denovogenes]_110120	99.61	0.0	boa:Bovatus_01198	K01126	E3.1.4.46, glpQ, ugpQ	glycerophosphoryl diester phosphodiesterase	3.1.4.46	Metabolism--Lipid metabolism--Glycerophospholipid metabolism

[denovogenes]_110126	98.43	0.0	bxy:BXY_33060	K00677	lpxA	UDP-N-acetylglucosamine acyltransferase	2.3.1.129	Metabolism--Glycan biosynthesis and metabolism--Lipopolysaccharide biosynthesis|Metabolism--Global and overview maps--Metabolic pathways|Human Diseases--Drug resistance: Antimicrobial--Cationic antimicrobial peptide (CAMP) resistance

[denovogenes]_110129	73.33	3e-140	coo:CCU_28510	K02003	ABC.CD.A	putative ABC transport system ATP-binding protein	--	--

[denovogenes]_110130	63.05	5e-116	mru:mru_0214	K01695	trpA	tryptophan synthase alpha chain	4.2.1.20	Metabolism--Amino acid metabolism--Glycine, serine and threonine metabolism|Metabolism--Amino acid metabolism--Phenylalanine, tyrosine and tryptophan biosynthesis|Metabolism--Global and overview maps--Metabolic pathways|Metabolism--Global and overview maps--Biosynthesis of secondary metabolites|Metabolism--Global and overview maps--Biosynthesis of antibiotics|Metabolism--Global and overview maps--Biosynthesis of amino acids

[denovogenes]_110131	52.99	2e-82	ers:K210_06910	K03497	parB, spo0J	chromosome partitioning protein, ParB family	--	--

[denovogenes]_110132	100.00	2e-180	ecm:EcSMS35_2186	K15555	ssuB	sulfonate transport system ATP-binding protein	3.6.3.-	Metabolism--Energy metabolism--Sulfur metabolism|Environmental Information Processing--Membrane transport--ABC transporters

[denovogenes]_110133	40.67	2e-44	ddl:Desdi_0609	K02008	cbiQ	cobalt/nickel transport system permease protein	--	Environmental Information Processing--Membrane transport--ABC transporters

[denovogenes]_110134	64.66	2e-121	efq:DR75_615	K04069	pflA, pflC, pflE	pyruvate formate lyase activating enzyme	1.97.1.4	--

[denovogenes]_110138	89.80	2e-159	vpr:Vpar_0567	K02823	pyrDII	dihydroorotate dehydrogenase electron transfer subunit	--	Metabolism--Nucleotide metabolism--Pyrimidine metabolism|Metabolism--Global and overview maps--Metabolic pathways

[denovogenes]_110139	91.34	6e-161	vpr:Vpar_1649	K06956	K06956	uncharacterized protein	--	--

[denovogenes]_11014	99.70	0.0	paeo:M801_3470	K01874	MARS, metG	methionyl-tRNA synthetase	6.1.1.10	Metabolism--Metabolism of other amino acids--Selenocompound metabolism|Genetic Information Processing--Translation--Aminoacyl-tRNA biosynthesis

[denovogenes]_110142	100.00	0.0	pmib:BB2000_0427	K12538	hasF, prtF	outer membrane protein, protease secretion system	--	--

[denovogenes]_110143	100.00	0.0	pmib:BB2000_2645	K05245	caiT	L-carnitine/gamma-butyrobetaine antiporter	--	--

[denovogenes]_110145	99.61	0.0	cpr:CPR_0337	K09762	K09762	uncharacterized protein	--	--

[denovogenes]_110146	99.61	0.0	bth:BT_2707	K03076	secY	preprotein translocase subunit SecY	--	Cellular Processes--Cellular community - prokaryotes--Quorum sensing|Genetic Information Processing--Folding, sorting and degradation--Protein export|Environmental Information Processing--Membrane transport--Bacterial secretion system

[denovogenes]_110147	100.00	4e-174	ecm:EcSMS35_3794	K03893	arsB	arsenical pump membrane protein	--	--

[denovogenes]_110148	89.84	6e-174	vpr:Vpar_0950	K04083	hslO	molecular chaperone Hsp33	--	--

[denovogenes]_110149	100.00	0.0	pvl:AOB99_06575	K07552	bcr	MFS transporter, DHA1 family, bicyclomycin/chloramphenicol resistance protein	--	--

[denovogenes]_110152	68.75	6e-125	fnu:FN0347	K01613	psd, PISD	phosphatidylserine decarboxylase	4.1.1.65	Metabolism--Lipid metabolism--Glycerophospholipid metabolism|Metabolism--Global and overview maps--Metabolic pathways|Metabolism--Global and overview maps--Biosynthesis of secondary metabolites

[denovogenes]_110153	99.61	1e-173	bvu:BVU_1627	K06178	rluB	23S rRNA pseudouridine2605 synthase	5.4.99.22	--

[denovogenes]_110154	69.02	2e-120	ehr:EHR_13455	K15738	uup	ATP-binding cassette, subfamily F, uup	--	--

[denovogenes]_110156	42.86	8e-69	gea:GARCT_02750	K04072	adhE	acetaldehyde dehydrogenase / alcohol dehydrogenase	1.2.1.10 1.1.1.1	Metabolism--Carbohydrate metabolism--Glycolysis / Gluconeogenesis|Metabolism--Lipid metabolism--Fatty acid degradation|Metabolism--Amino acid metabolism--Tyrosine metabolism|Metabolism--Carbohydrate metabolism--Pyruvate metabolism|Metabolism--Xenobiotics biodegradation and metabolism--Chloroalkane and chloroalkene degradation|Metabolism--Xenobiotics biodegradation and metabolism--Naphthalene degradation|Metabolism--Carbohydrate metabolism--Butanoate metabolism|Metabolism--Global and overview maps--Metabolic pathways|Metabolism--Global and overview maps--Biosynthesis of secondary metabolites|Metabolism--Global and overview maps--Microbial metabolism in diverse environments|Metabolism--Global and overview maps--Biosynthesis of antibiotics|Metabolism--Global and overview maps--Degradation of aromatic compounds

[denovogenes]_110157	94.49	6e-166	btho:Btheta7330_01286	K02837	prfC	peptide chain release factor 3	--	--

[denovogenes]_110158	81.25	3e-153	ecas:ECBG_01609	K11717	sufS	cysteine desulfurase / selenocysteine lyase	2.8.1.7 4.4.1.16	Metabolism--Metabolism of other amino acids--Selenocompound metabolism|Metabolism--Global and overview maps--Metabolic pathways

[denovogenes]_11016	46.58	0.0	ecp:ECP_2970	K12518	papC	outer membrane usher protein PapC	--	--

[denovogenes]_110166	71.48	6e-133	sfo:Z042_09695	K08368	yaaU	MFS transporter, putative metabolite transport protein	--	--

[denovogenes]_110168	100.00	0.0	cpe:CPE1774	K06207	typA, bipA	GTP-binding protein	--	--

[denovogenes]_11017	98.52	0.0	bfg:BF638R_2636	K03702	uvrB	excinuclease ABC subunit B	--	Genetic Information Processing--Replication and repair--Nucleotide excision repair

[denovogenes]_110170	82.08	8e-149	ess:ATZ33_16205	K01223	E3.2.1.86B, bglA	6-phospho-beta-glucosidase	3.2.1.86	Metabolism--Carbohydrate metabolism--Glycolysis / Gluconeogenesis|Metabolism--Carbohydrate metabolism--Starch and sucrose metabolism

[denovogenes]_110172	100.00	0.0	mmk:MU9_2011	K15256	cmoA	tRNA (cmo5U34)-methyltransferase	2.1.1.-	--

[denovogenes]_110173	42.80	8e-65	nko:Niako_6782	K13002	wbyL	glycosyltransferase	2.4.1.-	--

[denovogenes]_110175	69.05	8e-128	ecas:ECBG_00977	K05833	ABC.X4.A	putative ABC transport system ATP-binding protein	--	--

[denovogenes]_110177	45.49	4e-61	asb:RATSFB_1182	K18672	dacA	diadenylate cyclase	2.7.7.85	--

[denovogenes]_110178	100.00	0.0	bfb:VU15_01650	K03269	lpxH	UDP-2,3-diacylglucosamine hydrolase	3.6.1.54	Metabolism--Glycan biosynthesis and metabolism--Lipopolysaccharide biosynthesis|Metabolism--Global and overview maps--Metabolic pathways

[denovogenes]_110180	87.40	1e-169	ehr:EHR_00225	K01265	map	methionyl aminopeptidase	3.4.11.18	--

[denovogenes]_110183	78.57	1e-135	clt:CM240_2382	K03149	thiG	thiazole synthase	2.8.1.10	Metabolism--Metabolism of cofactors and vitamins--Thiamine metabolism|Metabolism--Global and overview maps--Metabolic pathways

[denovogenes]_110184	84.38	3e-151	cbut:ATN24_03465	K02746	PTS-Aga-EIIC, agaW	PTS system, N-acetylgalactosamine-specific IIC component	--	Metabolism--Carbohydrate metabolism--Galactose metabolism|Environmental Information Processing--Membrane transport--Phosphotransferase system (PTS)

[denovogenes]_110185	45.28	2e-70	cbh:CLC_1012	K02598	nirC	nitrite transporter NirC	--	--

[denovogenes]_110186	79.53	9e-151	ega:AL523_16110	K01104	E3.1.3.48	protein-tyrosine phosphatase	3.1.3.48	--

[denovogenes]_110188	62.65	2e-116	cbz:Cbs_0032	K01776	murI	glutamate racemase	5.1.1.3	Metabolism--Metabolism of other amino acids--D-Glutamine and D-glutamate metabolism|Metabolism--Global and overview maps--Metabolic pathways

[denovogenes]_110191	100.00	0.0	mmk:MU9_1035	K04030	eutQ	ethanolamine utilization protein EutQ	--	--

[denovogenes]_110192	60.08	1e-105	csb:CLSA_c42400	K01104	E3.1.3.48	protein-tyrosine phosphatase	3.1.3.48	--

[denovogenes]_110194	82.66	6e-144	cbv:U729_1453	K06182	rluF	23S rRNA pseudouridine2604 synthase	5.4.99.21	--

[denovogenes]_110195	67.73	5e-131	cpr:CPR_1117	K02013	ABC.FEV.A	iron complex transport system ATP-binding protein	3.6.3.34	Environmental Information Processing--Membrane transport--ABC transporters

[denovogenes]_110199	100.00	0.0	bfb:VU15_15455	K03783	punA, PNP	purine-nucleoside phosphorylase	2.4.2.1	Metabolism--Nucleotide metabolism--Purine metabolism|Metabolism--Nucleotide metabolism--Pyrimidine metabolism|Metabolism--Metabolism of cofactors and vitamins--Nicotinate and nicotinamide metabolism|Metabolism--Global and overview maps--Metabolic pathways|Metabolism--Global and overview maps--Biosynthesis of secondary metabolites

[denovogenes]_11020	82.30	0.0	pdi:BDI_3779	K01277	DPP3	dipeptidyl-peptidase III	3.4.14.4	--

[denovogenes]_110201	53.69	6e-72	cml:BN424_2781	K14742	tsaB	tRNA threonylcarbamoyladenosine biosynthesis protein TsaB	--	--

[denovogenes]_110203	49.00	2e-85	cpe:CPE0197	K03481	glvR	RpiR family transcriptional regulator, glv operon transcriptional regulator	--	--

[denovogenes]_110204	46.46	2e-78	pdi:BDI_0805	K02477	K02477	two-component system, LytT family, response regulator	--	--

[denovogenes]_110205	73.02	1e-134	cbt:CLH_0667	K03785	aroD	3-dehydroquinate dehydratase I	4.2.1.10	Metabolism--Amino acid metabolism--Phenylalanine, tyrosine and tryptophan biosynthesis|Metabolism--Global and overview maps--Metabolic pathways|Metabolism--Global and overview maps--Biosynthesis of secondary metabolites|Metabolism--Global and overview maps--Biosynthesis of antibiotics|Metabolism--Global and overview maps--Biosynthesis of amino acids

[denovogenes]_110207	99.61	0.0	efq:DR75_227	K03710	K03710	GntR family transcriptional regulator	--	--

[denovogenes]_110208	78.66	3e-150	cbz:Cbs_5095	K03496	parA, soj	chromosome partitioning protein	--	--

[denovogenes]_110209	66.54	3e-123	ega:AL523_12015	K01092	E3.1.3.25, IMPA, suhB	myo-inositol-1(or 4)-monophosphatase	3.1.3.25	Metabolism--Biosynthesis of other secondary metabolites--Streptomycin biosynthesis|Metabolism--Carbohydrate metabolism--Inositol phosphate metabolism|Metabolism--Global and overview maps--Metabolic pathways|Environmental Information Processing--Signal transduction--Phosphatidylinositol signaling system

[denovogenes]_11021	94.39	0.0	lacy:A4V08_02580	K12132	prkC, stkP	eukaryotic-like serine/threonine-protein kinase	2.7.11.1	--

[denovogenes]_110210	76.89	6e-118	lacy:A4V08_19025	K02945	RP-S1, rpsA	small subunit ribosomal protein S1	--	Genetic Information Processing--Translation--Ribosome

[denovogenes]_110211	100.00	0.0	ene:ENT_14850	K01265	map	methionyl aminopeptidase	3.4.11.18	--

[denovogenes]_110212	51.24	1e-80	ppen:T256_00290	K03811	pnuC	nicotinamide mononucleotide transporter	--	--

[denovogenes]_110213	96.06	1e-180	cama:F384_15680	K00059	fabG	3-oxoacyl-[acyl-carrier protein] reductase	1.1.1.100	Metabolism--Lipid metabolism--Fatty acid biosynthesis|Metabolism--Metabolism of cofactors and vitamins--Biotin metabolism|Metabolism--Lipid metabolism--Biosynthesis of unsaturated fatty acids|Metabolism--Global and overview maps--Metabolic pathways|Metabolism--Global and overview maps--Fatty acid metabolism

[denovogenes]_110214	40.93	5e-57	bww:bwei_1528	K07260	vanY	D-alanyl-D-alanine carboxypeptidase	3.4.16.4	Metabolism--Glycan biosynthesis and metabolism--Peptidoglycan biosynthesis|Human Diseases--Drug resistance: Antimicrobial--Vancomycin resistance|Environmental Information Processing--Signal transduction--Two-component system

[denovogenes]_110216	96.46	2e-172	sgg:SGGBAA2069_c00170	K03570	mreC	rod shape-determining protein MreC	--	--

[denovogenes]_110217	51.42	7e-81	tap:GZ22_12240	K02530	lacR	DeoR family transcriptional regulator, lactose phosphotransferase system repressor	--	--

[denovogenes]_11022	96.31	0.0	pmr:PMI3110	K00123	fdoG, fdfH	formate dehydrogenase major subunit	1.2.1.2	Metabolism--Carbohydrate metabolism--Glyoxylate and dicarboxylate metabolism|Metabolism--Energy metabolism--Methane metabolism|Metabolism--Global and overview maps--Metabolic pathways|Metabolism--Global and overview maps--Microbial metabolism in diverse environments|Metabolism--Global and overview maps--Carbon metabolism

[denovogenes]_110221	99.61	0.0	stb:SGPB_0386	K03366	butA, budC	meso-butanediol dehydrogenase / (S,S)-butanediol dehydrogenase / diacetyl reductase	1.1.1.- 1.1.1.76 1.1.1.304	Metabolism--Carbohydrate metabolism--Butanoate metabolism

[denovogenes]_110224	100.00	0.0	efq:DR75_615	K04069	pflA, pflC, pflE	pyruvate formate lyase activating enzyme	1.97.1.4	--

[denovogenes]_110225	100.00	0.0	stb:SGPB_1567	K01995	livG	branched-chain amino acid transport system ATP-binding protein	--	Environmental Information Processing--Membrane transport--ABC transporters|Cellular Processes--Cellular community - prokaryotes--Quorum sensing

[denovogenes]_110227	69.69	2e-124	cbt:CLH_0863	K09761	rsmE	16S rRNA (uracil1498-N3)-methyltransferase	2.1.1.193	--

[denovogenes]_110228	77.95	1e-147	cbut:ATN24_04280	K00978	rfbF	glucose-1-phosphate cytidylyltransferase	2.7.7.33	Metabolism--Carbohydrate metabolism--Starch and sucrose metabolism|Metabolism--Carbohydrate metabolism--Amino sugar and nucleotide sugar metabolism|Metabolism--Global and overview maps--Metabolic pathways

[denovogenes]_110229	90.56	2e-115	era:ERE_07380	K03496	parA, soj	chromosome partitioning protein	--	--

[denovogenes]_11023	86.72	0.0	pdi:BDI_1854	K03798	ftsH, hflB	cell division protease FtsH	3.4.24.-	--

[denovogenes]_110231	96.44	2e-180	cfd:CFNIH1_21045	K09933	mtfA	MtfA peptidase	--	--

[denovogenes]_110233	99.61	0.0	stb:SGPB_1450	K10041	ABC.GLN1.A	putative glutamine transport system ATP-binding protein	3.6.3.-	Environmental Information Processing--Membrane transport--ABC transporters

[denovogenes]_110234	68.11	3e-130	lacy:A4V08_06710	K06173	truA, PUS1	tRNA pseudouridine38-40 synthase	5.4.99.12	--

[denovogenes]_110235	60.63	3e-105	aoe:Clos_0031	K10795	prdD	D-proline reductase (dithiol) PrdD	1.21.4.1	Metabolism--Amino acid metabolism--Arginine and proline metabolism

[denovogenes]_110236	81.50	4e-149	pdf:CD630DERM_07720	K03091	SIG3.4	RNA polymerase sporulation-specific sigma factor	--	--

[denovogenes]_110239	99.61	0.0	ecp:ECP_4515	K00046	idnO	gluconate 5-dehydrogenase	1.1.1.69	--

[denovogenes]_11024	97.79	0.0	cfd:CFNIH1_21600	K07788	mdtB	multidrug efflux pump	--	Environmental Information Processing--Signal transduction--Two-component system

[denovogenes]_110241	83.33	1e-156	slq:M495_16790	K00046	idnO	gluconate 5-dehydrogenase	1.1.1.69	--

[denovogenes]_110242	84.96	3e-157	cbt:CLH_1151	K02036	pstB	phosphate transport system ATP-binding protein	3.6.3.27	Environmental Information Processing--Membrane transport--ABC transporters

[denovogenes]_110246	57.80	5e-33	ene:ENT_05800	K02086	dnaD	DNA replication protein	--	--

[denovogenes]_110250	42.72	5e-52	cso:CLS_25440	K06183	rsuA	16S rRNA pseudouridine516 synthase	5.4.99.19	--

[denovogenes]_110251	44.49	2e-68	ctc:CTC_01849	K07720	yesN	two-component system, response regulator YesN	--	Environmental Information Processing--Signal transduction--Two-component system

[denovogenes]_110253	50.00	3e-70	lja:Lj0g3v0129059.2	K00264	GLT1	glutamate synthase (NADPH/NADH)	1.4.1.13 1.4.1.14	Metabolism--Amino acid metabolism--Alanine, aspartate and glutamate metabolism|Metabolism--Energy metabolism--Nitrogen metabolism|Metabolism--Global and overview maps--Metabolic pathways|Metabolism--Global and overview maps--Biosynthesis of secondary metabolites|Metabolism--Global and overview maps--Microbial metabolism in diverse environments|Metabolism--Global and overview maps--Biosynthesis of antibiotics|Metabolism--Global and overview maps--Biosynthesis of amino acids

[denovogenes]_110254	42.19	1e-56	cay:CEA_G1331	K10716	kch, trkA, mthK, pch	voltage-gated potassium channel	--	--

[denovogenes]_110255	96.08	1e-171	psx:DR96_467	K02065	mlaF, linL, mkl	phospholipid/cholesterol/gamma-HCH transport system ATP-binding protein	--	Environmental Information Processing--Membrane transport--ABC transporters

[denovogenes]_110256	43.25	9e-65	scc:Spico_0496	K04477	ycdX	putative hydrolase	--	--

[denovogenes]_110259	77.29	3e-143	cle:Clole_3243	K02041	phnC	phosphonate transport system ATP-binding protein	3.6.3.28	Environmental Information Processing--Membrane transport--ABC transporters

[denovogenes]_110260	70.54	6e-129	cbt:CLH_0458	K06436	yabG	spore coat assemly protein	--	--

[denovogenes]_110261	99.21	5e-178	cbut:ATN24_00890	K02746	PTS-Aga-EIIC, agaW	PTS system, N-acetylgalactosamine-specific IIC component	--	Metabolism--Carbohydrate metabolism--Galactose metabolism|Environmental Information Processing--Membrane transport--Phosphotransferase system (PTS)

[denovogenes]_110263	100.00	0.0	pvl:AOB99_07185	K11604	sitA	manganese/iron transport system substrate-binding protein	--	Environmental Information Processing--Membrane transport--ABC transporters

[denovogenes]_110264	61.42	1e-107	aoe:Clos_0031	K10795	prdD	D-proline reductase (dithiol) PrdD	1.21.4.1	Metabolism--Amino acid metabolism--Arginine and proline metabolism

[denovogenes]_110265	100.00	0.0	ecos:EC958_0255	K05799	pdhR	GntR family transcriptional regulator, transcriptional repressor for pyruvate dehydrogenase complex	--	--

[denovogenes]_110266	47.88	2e-74	etd:ETAF_2037	K12519	papD	chaperone protein PapD	--	--

[denovogenes]_110267	54.94	7e-92	tsh:Tsac_0081	K10907	K10907	aminotransferase	2.6.1.-	--

[denovogenes]_110268	82.00	2e-150	ipo:Ilyop_2189	K02036	pstB	phosphate transport system ATP-binding protein	3.6.3.27	Environmental Information Processing--Membrane transport--ABC transporters

[denovogenes]_110270	41.33	3e-38	cace:CACET_c39140	K02315	dnaC	DNA replication protein DnaC	--	--

[denovogenes]_110272	97.65	0.0	senj:CFSAN001992_10090	K01918	panC	pantoate--beta-alanine ligase	6.3.2.1	Metabolism--Metabolism of other amino acids--beta-Alanine metabolism|Metabolism--Metabolism of cofactors and vitamins--Pantothenate and CoA biosynthesis|Metabolism--Global and overview maps--Metabolic pathways|Metabolism--Global and overview maps--Biosynthesis of secondary metabolites

[denovogenes]_110273	99.61	0.0	elf:LF82_1559	K03286	TC.OOP	OmpA-OmpF porin, OOP family	--	--

[denovogenes]_110274	92.16	2e-178	prg:RB151_006480	K06211	nadR	HTH-type transcriptional regulator, transcriptional repressor of NAD biosynthesis genes	2.7.7.1 2.7.1.22	Metabolism--Metabolism of cofactors and vitamins--Nicotinate and nicotinamide metabolism|Metabolism--Global and overview maps--Metabolic pathways

[denovogenes]_110275	87.45	2e-161	cah:CAETHG_2195	K19309	bcrA	bacitracin transport system ATP-binding protein	--	Environmental Information Processing--Membrane transport--ABC transporters

[denovogenes]_110276	99.61	0.0	elo:EC042_3263	K11474	glcC	GntR family transcriptional regulator, glc operon transcriptional activator	--	--

[denovogenes]_110278	99.61	0.0	cbut:ATN24_18555	K02020	modA	molybdate transport system substrate-binding protein	--	Environmental Information Processing--Membrane transport--ABC transporters

[denovogenes]_110279	92.13	2e-177	btho:Btheta7330_04599	K03269	lpxH	UDP-2,3-diacylglucosamine hydrolase	3.6.1.54	Metabolism--Glycan biosynthesis and metabolism--Lipopolysaccharide biosynthesis|Metabolism--Global and overview maps--Metabolic pathways

[denovogenes]_110281	99.22	2e-177	cbut:ATN24_03715	K03628	rho	transcription termination factor Rho	--	Genetic Information Processing--Folding, sorting and degradation--RNA degradation

[denovogenes]_110282	100.00	0.0	ecol:LY180_06610	K09473	puuD	gamma-glutamyl-gamma-aminobutyrate hydrolase	3.5.1.94	Metabolism--Amino acid metabolism--Arginine and proline metabolism|Metabolism--Global and overview maps--Metabolic pathways

[denovogenes]_110283	55.60	2e-95	gfe:Gferi_15540	K02564	nagB, GNPDA	glucosamine-6-phosphate deaminase	3.5.99.6	Metabolism--Carbohydrate metabolism--Amino sugar and nucleotide sugar metabolism|Metabolism--Global and overview maps--Metabolic pathways

[denovogenes]_110284	100.00	0.0	pvl:AOB99_12490	K02747	PTS-Aga-EIID, agaE	PTS system, N-acetylgalactosamine-specific IID component	--	Metabolism--Carbohydrate metabolism--Galactose metabolism|Environmental Information Processing--Membrane transport--Phosphotransferase system (PTS)

[denovogenes]_110286	52.31	2e-76	pse:NH8B_0416	K13017	wbpE, wlbC	UDP-2-acetamido-2-deoxy-ribo-hexuluronate aminotransferase	2.6.1.98	Metabolism--Carbohydrate metabolism--Amino sugar and nucleotide sugar metabolism

[denovogenes]_110288	98.82	0.0	cbut:ATN24_15895	K01222	E3.2.1.86A, celF	6-phospho-beta-glucosidase	3.2.1.86	Metabolism--Carbohydrate metabolism--Glycolysis / Gluconeogenesis|Metabolism--Carbohydrate metabolism--Starch and sucrose metabolism

[denovogenes]_110289	69.92	3e-122	cle:Clole_1904	K07137	K07137	uncharacterized protein	--	--

[denovogenes]_11029	98.52	0.0	bxy:BXY_09360	K01547	kdpB	K+-transporting ATPase ATPase B chain	3.6.3.12	Environmental Information Processing--Signal transduction--Two-component system

[denovogenes]_110290	100.00	0.0	ssj:SSON53_24750	K02042	phnE	phosphonate transport system permease protein	--	Environmental Information Processing--Membrane transport--ABC transporters

[denovogenes]_110295	86.27	2e-142	cbv:U729_1722	K03979	obgE, cgtA	GTPase	3.6.5.-	--

[denovogenes]_110297	64.57	2e-118	cct:CC1_18920	K02026	ABC.MS.P1	multiple sugar transport system permease protein	--	--

[denovogenes]_1103	46.22	0.0	afn:Acfer_1506	K03529	smc	chromosome segregation protein	--	--

[denovogenes]_110300	85.04	2e-158	csb:CLSA_c06180	K03609	minD	septum site-determining protein MinD	--	--

[denovogenes]_110303	51.60	2e-77	tex:Teth514_0528	K01436	K01436	amidohydrolase	3.5.1.-	--

[denovogenes]_110304	50.00	1e-31	ava:Ava_C0210	K08259	lytM	lysostaphin	3.4.24.75	--

[denovogenes]_110305	100.00	0.0	ecw:EcE24377A_2647	K00847	E2.7.1.4, scrK	fructokinase	2.7.1.4	Metabolism--Carbohydrate metabolism--Fructose and mannose metabolism|Metabolism--Carbohydrate metabolism--Starch and sucrose metabolism|Metabolism--Carbohydrate metabolism--Amino sugar and nucleotide sugar metabolism|Metabolism--Global and overview maps--Metabolic pathways

[denovogenes]_110306	46.12	3e-83	blf:BLIF_1546	K00852	rbsK, RBKS	ribokinase	2.7.1.15	Metabolism--Carbohydrate metabolism--Pentose phosphate pathway

[denovogenes]_110307	98.82	0.0	sft:NCTC1_04586	K07497	K07497	putative transposase	--	--

[denovogenes]_110309	53.73	8e-88	ers:K210_07600	K06147	ABCB-BAC	ATP-binding cassette, subfamily B, bacterial	--	--

[denovogenes]_110310	68.63	4e-116	spj:MGAS2096_Spy0014	K03798	ftsH, hflB	cell division protease FtsH	3.4.24.-	--

[denovogenes]_110313	61.78	5e-114	cdg:CDBI1_10950	K01262	pepP	Xaa-Pro aminopeptidase	3.4.11.9	--

[denovogenes]_110315	52.78	1e-75	bon:A361_14585	K01028	E2.8.3.5A, scoA	3-oxoacid CoA-transferase subunit A	2.8.3.5	Metabolism--Lipid metabolism--Synthesis and degradation of ketone bodies|Metabolism--Amino acid metabolism--Valine, leucine and isoleucine degradation|Metabolism--Carbohydrate metabolism--Butanoate metabolism

[denovogenes]_110317	44.53	3e-59	asb:RATSFB_1182	K18672	dacA	diadenylate cyclase	2.7.7.85	--

[denovogenes]_110318	90.48	9e-171	bhl:Bache_0586	K00215	dapB	4-hydroxy-tetrahydrodipicolinate reductase	1.17.1.8	Metabolism--Biosynthesis of other secondary metabolites--Monobactam biosynthesis|Metabolism--Amino acid metabolism--Lysine biosynthesis|Metabolism--Global and overview maps--Metabolic pathways|Metabolism--Global and overview maps--Biosynthesis of secondary metabolites|Metabolism--Global and overview maps--Microbial metabolism in diverse environments|Metabolism--Global and overview maps--Biosynthesis of antibiotics|Metabolism--Global and overview maps--Biosynthesis of amino acids

[denovogenes]_110319	90.55	1e-174	bxy:BXY_25810	K00215	dapB	4-hydroxy-tetrahydrodipicolinate reductase	1.17.1.8	Metabolism--Biosynthesis of other secondary metabolites--Monobactam biosynthesis|Metabolism--Amino acid metabolism--Lysine biosynthesis|Metabolism--Global and overview maps--Metabolic pathways|Metabolism--Global and overview maps--Biosynthesis of secondary metabolites|Metabolism--Global and overview maps--Microbial metabolism in diverse environments|Metabolism--Global and overview maps--Biosynthesis of antibiotics|Metabolism--Global and overview maps--Biosynthesis of amino acids

[denovogenes]_11032	100.00	0.0	pvl:AOB99_10045	K13821	putA	RHH-type transcriptional regulator, proline utilization regulon repressor / proline dehydrogenase / delta 1-pyrroline-5-carboxylate dehydrogenase	1.5.5.2 1.2.1.88	Metabolism--Amino acid metabolism--Alanine, aspartate and glutamate metabolism|Metabolism--Amino acid metabolism--Arginine and proline metabolism|Metabolism--Global and overview maps--Metabolic pathways|Metabolism--Global and overview maps--Biosynthesis of secondary metabolites|Metabolism--Global and overview maps--Biosynthesis of antibiotics

[denovogenes]_110321	65.56	4e-108	rim:ROI_25190	K06182	rluF	23S rRNA pseudouridine2604 synthase	5.4.99.21	--

[denovogenes]_110325	53.36	8e-86	rto:RTO_20570	K07124	K07124	uncharacterized protein	--	--

[denovogenes]_110326	67.06	6e-118	rto:RTO_24670	K03470	rnhB	ribonuclease HII	3.1.26.4	Genetic Information Processing--Replication and repair--DNA replication

[denovogenes]_110327	96.46	0.0	bxy:BXY_29830	K03269	lpxH	UDP-2,3-diacylglucosamine hydrolase	3.6.1.54	Metabolism--Glycan biosynthesis and metabolism--Lipopolysaccharide biosynthesis|Metabolism--Global and overview maps--Metabolic pathways

[denovogenes]_110328	43.48	6e-62	eac:EAL2_c18460	K18672	dacA	diadenylate cyclase	2.7.7.85	--

[denovogenes]_110329	99.21	0.0	bxy:BXY_25810	K00215	dapB	4-hydroxy-tetrahydrodipicolinate reductase	1.17.1.8	Metabolism--Biosynthesis of other secondary metabolites--Monobactam biosynthesis|Metabolism--Amino acid metabolism--Lysine biosynthesis|Metabolism--Global and overview maps--Metabolic pathways|Metabolism--Global and overview maps--Biosynthesis of secondary metabolites|Metabolism--Global and overview maps--Microbial metabolism in diverse environments|Metabolism--Global and overview maps--Biosynthesis of antibiotics|Metabolism--Global and overview maps--Biosynthesis of amino acids

[denovogenes]_11033	99.56	0.0	mmk:MU9_260	K03656	rep	ATP-dependent DNA helicase Rep	3.6.4.12	--

[denovogenes]_110333	85.43	8e-165	bhl:Bache_1885	K06167	phnP	phosphoribosyl 1,2-cyclic phosphate phosphodiesterase	3.1.4.55	Metabolism--Metabolism of other amino acids--Phosphonate and phosphinate metabolism

[denovogenes]_110335	57.66	2e-100	rum:CK1_25910	K04720	cobD	threonine-phosphate decarboxylase	4.1.1.81	Metabolism--Metabolism of cofactors and vitamins--Porphyrin and chlorophyll metabolism

[denovogenes]_110336	74.60	9e-138	cct:CC1_11080	K02028	ABC.PA.A	polar amino acid transport system ATP-binding protein	3.6.3.21	--

[denovogenes]_110337	72.22	1e-130	rto:RTO_24670	K03470	rnhB	ribonuclease HII	3.1.26.4	Genetic Information Processing--Replication and repair--DNA replication

[denovogenes]_110339	77.87	2e-147	bprm:CL3_16200	K00868	pdxK, pdxY	pyridoxine kinase	2.7.1.35	Metabolism--Metabolism of cofactors and vitamins--Vitamin B6 metabolism|Metabolism--Global and overview maps--Metabolic pathways

[denovogenes]_110340	100.00	0.0	bvu:BVU_1877	K03524	birA	BirA family transcriptional regulator, biotin operon repressor / biotin-[acetyl-CoA-carboxylase] ligase	6.3.4.15	Metabolism--Metabolism of cofactors and vitamins--Biotin metabolism|Metabolism--Global and overview maps--Metabolic pathways

[denovogenes]_110341	100.00	0.0	bvu:BVU_1964	K12343	SRD5A1	3-oxo-5-alpha-steroid 4-dehydrogenase 1	1.3.1.22	Metabolism--Lipid metabolism--Steroid hormone biosynthesis

[denovogenes]_110342	53.31	1e-84	tto:Thethe_01836	K02510	hpaI, hpcH	4-hydroxy-2-oxoheptanedioate aldolase	4.1.2.52	Metabolism--Amino acid metabolism--Tyrosine metabolism|Metabolism--Global and overview maps--Microbial metabolism in diverse environments

[denovogenes]_110345	99.61	0.0	bvu:BVU_2311	K00175	korB, oorB, oforB	2-oxoglutarate/2-oxoacid ferredoxin oxidoreductase subunit beta	1.2.7.3 1.2.7.11	Metabolism--Carbohydrate metabolism--Glycolysis / Gluconeogenesis|Metabolism--Carbohydrate metabolism--Citrate cycle (TCA cycle)|Metabolism--Carbohydrate metabolism--Pyruvate metabolism|Metabolism--Carbohydrate metabolism--Butanoate metabolism|Metabolism--Energy metabolism--Carbon fixation pathways in prokaryotes|Metabolism--Global and overview maps--Metabolic pathways|Metabolism--Global and overview maps--Microbial metabolism in diverse environments|Metabolism--Global and overview maps--Biosynthesis of antibiotics|Metabolism--Global and overview maps--Carbon metabolism

[denovogenes]_110346	98.82	0.0	btho:Btheta7330_03750	K02428	rdgB	XTP/dITP diphosphohydrolase	3.6.1.66	Metabolism--Nucleotide metabolism--Purine metabolism

[denovogenes]_110347	92.13	0.0	bhl:Bache_2576	K01142	E3.1.11.2, xthA	exodeoxyribonuclease III	3.1.11.2	Genetic Information Processing--Replication and repair--Base excision repair

[denovogenes]_110349	100.00	0.0	bvu:BVU_3800	K02477	K02477	two-component system, LytT family, response regulator	--	--

[denovogenes]_110351	98.43	0.0	bxy:BXY_17750	K03783	punA, PNP	purine-nucleoside phosphorylase	2.4.2.1	Metabolism--Nucleotide metabolism--Purine metabolism|Metabolism--Nucleotide metabolism--Pyrimidine metabolism|Metabolism--Metabolism of cofactors and vitamins--Nicotinate and nicotinamide metabolism|Metabolism--Global and overview maps--Metabolic pathways|Metabolism--Global and overview maps--Biosynthesis of secondary metabolites

[denovogenes]_110352	67.48	5e-118	rto:RTO_10660	K20074	prpC, phpP	PPM family protein phosphatase	3.1.3.16	--

[denovogenes]_110353	62.87	5e-86	rum:CK1_02070	K04759	feoB	ferrous iron transport protein B	--	--

[denovogenes]_110354	48.10	7e-77	bce:BC3441	K07273	acm	lysozyme	--	--

[denovogenes]_110355	51.78	2e-97	tbe:Trebr_0838	K07124	K07124	uncharacterized protein	--	--

[denovogenes]_110357	99.61	0.0	bxy:BXY_29830	K03269	lpxH	UDP-2,3-diacylglucosamine hydrolase	3.6.1.54	Metabolism--Glycan biosynthesis and metabolism--Lipopolysaccharide biosynthesis|Metabolism--Global and overview maps--Metabolic pathways

[denovogenes]_110358	68.27	3e-127	rto:RTO_17900	K01163	K01163	uncharacterized protein	--	--

[denovogenes]_110359	74.01	9e-115	rto:RTO_29840	K02003	ABC.CD.A	putative ABC transport system ATP-binding protein	--	--

[denovogenes]_11036	99.85	0.0	efi:OG1RF_10459	K01972	E6.5.1.2, ligA, ligB	DNA ligase (NAD+)	6.5.1.2	Genetic Information Processing--Replication and repair--DNA replication|Genetic Information Processing--Replication and repair--Base excision repair|Genetic Information Processing--Replication and repair--Nucleotide excision repair|Genetic Information Processing--Replication and repair--Mismatch repair

[denovogenes]_110362	69.80	2e-112	lacy:A4V08_25475	K07090	K07090	uncharacterized protein	--	--

[denovogenes]_110363	42.15	1e-16	cpro:CPRO_15970	K03148	thiF	sulfur carrier protein ThiS adenylyltransferase	2.7.7.73	Metabolism--Metabolism of cofactors and vitamins--Thiamine metabolism|Metabolism--Global and overview maps--Metabolic pathways|Genetic Information Processing--Folding, sorting and degradation--Sulfur relay system

[denovogenes]_110365	83.86	3e-161	cso:CLS_19380	K02823	pyrDII	dihydroorotate dehydrogenase electron transfer subunit	--	Metabolism--Nucleotide metabolism--Pyrimidine metabolism|Metabolism--Global and overview maps--Metabolic pathways

[denovogenes]_110366	100.00	0.0	btho:Btheta7330_02144	K01142	E3.1.11.2, xthA	exodeoxyribonuclease III	3.1.11.2	Genetic Information Processing--Replication and repair--Base excision repair

[denovogenes]_110368	92.71	5e-173	bfg:BF638R_3662	K00571	E2.1.1.72	site-specific DNA-methyltransferase (adenine-specific)	2.1.1.72	--

[denovogenes]_110369	91.09	3e-170	bcel:BcellWH2_00052	K00979	kdsB	3-deoxy-manno-octulosonate cytidylyltransferase (CMP-KDO synthetase)	2.7.7.38	Metabolism--Glycan biosynthesis and metabolism--Lipopolysaccharide biosynthesis|Metabolism--Global and overview maps--Metabolic pathways

[denovogenes]_11037	97.49	0.0	cif:AL515_21980	K01534	zntA	Cd2+/Zn2+-exporting ATPase	3.6.3.3 3.6.3.5	--

[denovogenes]_110370	64.90	1e-121	cpf:CPF_0751	K01470	E3.5.2.10	creatinine amidohydrolase	3.5.2.10	Metabolism--Amino acid metabolism--Arginine and proline metabolism

[denovogenes]_110371	70.63	2e-133	elm:ELI_3480	K02003	ABC.CD.A	putative ABC transport system ATP-binding protein	--	--

[denovogenes]_110372	100.00	0.0	bxy:BXY_02010	K01142	E3.1.11.2, xthA	exodeoxyribonuclease III	3.1.11.2	Genetic Information Processing--Replication and repair--Base excision repair

[denovogenes]_110373	73.14	3e-129	cso:CLS_33590	K03710	K03710	GntR family transcriptional regulator	--	--

[denovogenes]_110374	100.00	0.0	bvu:BVU_0449	K02065	mlaF, linL, mkl	phospholipid/cholesterol/gamma-HCH transport system ATP-binding protein	--	Environmental Information Processing--Membrane transport--ABC transporters

[denovogenes]_110375	93.28	1e-178	lacy:A4V08_18660	K02036	pstB	phosphate transport system ATP-binding protein	3.6.3.27	Environmental Information Processing--Membrane transport--ABC transporters

[denovogenes]_110377	98.43	0.0	bxy:BXY_05680	K03437	spoU	RNA methyltransferase, TrmH family	--	--

[denovogenes]_110378	65.60	2e-115	fpla:A4U99_01915	K01265	map	methionyl aminopeptidase	3.4.11.18	--

[denovogenes]_110379	99.61	0.0	boa:Bovatus_00178	K00175	korB, oorB, oforB	2-oxoglutarate/2-oxoacid ferredoxin oxidoreductase subunit beta	1.2.7.3 1.2.7.11	Metabolism--Carbohydrate metabolism--Glycolysis / Gluconeogenesis|Metabolism--Carbohydrate metabolism--Citrate cycle (TCA cycle)|Metabolism--Carbohydrate metabolism--Pyruvate metabolism|Metabolism--Carbohydrate metabolism--Butanoate metabolism|Metabolism--Energy metabolism--Carbon fixation pathways in prokaryotes|Metabolism--Global and overview maps--Metabolic pathways|Metabolism--Global and overview maps--Microbial metabolism in diverse environments|Metabolism--Global and overview maps--Biosynthesis of antibiotics|Metabolism--Global and overview maps--Carbon metabolism

[denovogenes]_110380	70.28	5e-128	csh:Closa_0484	K00757	udp, UPP	uridine phosphorylase	2.4.2.3	Metabolism--Nucleotide metabolism--Pyrimidine metabolism|Metabolism--Xenobiotics biodegradation and metabolism--Drug metabolism - other enzymes|Metabolism--Global and overview maps--Metabolic pathways

[denovogenes]_110381	52.03	4e-87	bpw:WESB_0260	K03436	fruR2, fruR	DeoR family transcriptional regulator, fructose operon transcriptional repressor	--	--

[denovogenes]_110384	88.89	2e-170	bdo:EL88_09975	K00979	kdsB	3-deoxy-manno-octulosonate cytidylyltransferase (CMP-KDO synthetase)	2.7.7.38	Metabolism--Glycan biosynthesis and metabolism--Lipopolysaccharide biosynthesis|Metabolism--Global and overview maps--Metabolic pathways

[denovogenes]_110387	99.61	0.0	bxy:BXY_34760	K02428	rdgB	XTP/dITP diphosphohydrolase	3.6.1.66	Metabolism--Nucleotide metabolism--Purine metabolism

[denovogenes]_110389	44.08	2e-61	eac:EAL2_c18460	K18672	dacA	diadenylate cyclase	2.7.7.85	--

[denovogenes]_11039	85.03	0.0	fus:HMPREF0409_00792	K01712	hutU, UROC1	urocanate hydratase	4.2.1.49	Metabolism--Amino acid metabolism--Histidine metabolism|Metabolism--Global and overview maps--Metabolic pathways

[denovogenes]_110391	89.76	7e-174	bhl:Bache_1253	K03787	surE	5'-nucleotidase	3.1.3.5	Metabolism--Nucleotide metabolism--Purine metabolism|Metabolism--Nucleotide metabolism--Pyrimidine metabolism|Metabolism--Metabolism of cofactors and vitamins--Nicotinate and nicotinamide metabolism|Metabolism--Global and overview maps--Metabolic pathways|Metabolism--Global and overview maps--Biosynthesis of secondary metabolites

[denovogenes]_110392	76.44	1e-114	ccel:CCDG5_0188	K02982	RP-S3, rpsC	small subunit ribosomal protein S3	--	Genetic Information Processing--Translation--Ribosome

[denovogenes]_110393	54.43	4e-88	cpro:CPRO_22430	K03218	rlmB	23S rRNA (guanosine2251-2'-O)-methyltransferase	2.1.1.185	--

[denovogenes]_110395	48.03	3e-84	byl:A4V09_07920	K00575	cheR	chemotaxis protein methyltransferase CheR	2.1.1.80	Environmental Information Processing--Signal transduction--Two-component system|Cellular Processes--Cell motility--Bacterial chemotaxis

[denovogenes]_110396	85.49	3e-157	ibu:IB211_01862	K02034	ABC.PE.P1	peptide/nickel transport system permease protein	--	Cellular Processes--Cellular community - prokaryotes--Quorum sensing

[denovogenes]_110397	66.54	3e-111	csh:Closa_2346	K03525	coaX	type III pantothenate kinase	2.7.1.33	Metabolism--Metabolism of cofactors and vitamins--Pantothenate and CoA biosynthesis|Metabolism--Global and overview maps--Metabolic pathways

[denovogenes]_11040	70.74	0.0	cle:Clole_1271	K02355	fusA, GFM, EFG	elongation factor G	--	--

[denovogenes]_110400	66.67	7e-58	byl:A4V09_22815	K07720	yesN	two-component system, response regulator YesN	--	Environmental Information Processing--Signal transduction--Two-component system

[denovogenes]_110403	89.76	3e-166	bvu:BVU_1925	K02528	ksgA	16S rRNA (adenine1518-N6/adenine1519-N6)-dimethyltransferase	2.1.1.182	--

[denovogenes]_110407	96.46	0.0	bvu:BVU_2311	K00175	korB, oorB, oforB	2-oxoglutarate/2-oxoacid ferredoxin oxidoreductase subunit beta	1.2.7.3 1.2.7.11	Metabolism--Carbohydrate metabolism--Glycolysis / Gluconeogenesis|Metabolism--Carbohydrate metabolism--Citrate cycle (TCA cycle)|Metabolism--Carbohydrate metabolism--Pyruvate metabolism|Metabolism--Carbohydrate metabolism--Butanoate metabolism|Metabolism--Energy metabolism--Carbon fixation pathways in prokaryotes|Metabolism--Global and overview maps--Metabolic pathways|Metabolism--Global and overview maps--Microbial metabolism in diverse environments|Metabolism--Global and overview maps--Biosynthesis of antibiotics|Metabolism--Global and overview maps--Carbon metabolism

[denovogenes]_110408	64.57	1e-124	paea:R70723_17250	K02003	ABC.CD.A	putative ABC transport system ATP-binding protein	--	--

[denovogenes]_110409	66.67	6e-110	ibu:IB211_01428	K07273	acm	lysozyme	--	--

[denovogenes]_11041	61.39	0.0	rim:ROI_16030	K01187	malZ	alpha-glucosidase	3.2.1.20	Metabolism--Carbohydrate metabolism--Galactose metabolism|Metabolism--Carbohydrate metabolism--Starch and sucrose metabolism|Metabolism--Global and overview maps--Metabolic pathways

[denovogenes]_110410	97.24	2e-179	cct:CC1_30250	K20459	nukF, mcdF, sboF	lantibiotic transport system ATP-binding protein	--	Environmental Information Processing--Membrane transport--ABC transporters

[denovogenes]_110411	86.22	4e-159	bvu:BVU_1964	K12343	SRD5A1	3-oxo-5-alpha-steroid 4-dehydrogenase 1	1.3.1.22	Metabolism--Lipid metabolism--Steroid hormone biosynthesis

[denovogenes]_110415	54.55	2e-45	ere:EUBREC_0647	K18816	aac6-I, aacA7	aminoglycoside 6'-N-acetyltransferase I	2.3.1.82	--

[denovogenes]_110417	61.20	6e-109	lot:AXF11_04340	K01995	livG	branched-chain amino acid transport system ATP-binding protein	--	Environmental Information Processing--Membrane transport--ABC transporters|Cellular Processes--Cellular community - prokaryotes--Quorum sensing

[denovogenes]_110418	52.07	3e-36	pca:Pcar_1321	K08309	slt	soluble lytic murein transglycosylase	3.2.1.-	--

[denovogenes]_110419	75.10	1e-142	ddh:Desde_0492	K02003	ABC.CD.A	putative ABC transport system ATP-binding protein	--	--

[denovogenes]_110420	55.56	2e-89	lsi:HN6_01035	K06959	tex	protein Tex	--	--

[denovogenes]_110421	58.27	7e-108	cbv:U729_301	K02444	glpR	DeoR family transcriptional regulator, glycerol-3-phosphate regulon repressor	--	--

[denovogenes]_110422	60.32	6e-119	cdl:CDR20291_3391	K05540	dusB	tRNA-dihydrouridine synthase B	1.-.-.-	--

[denovogenes]_110423	46.72	3e-73	bha:BH3661	K16698	tuaG	teichuronic acid biosynthesis glycosyltransferase TuaG	2.4.-.-	--

[denovogenes]_110425	100.00	0.0	shq:A0259_10500	K01096	pgpB	phosphatidylglycerophosphatase B	3.1.3.27 3.1.3.81 3.1.3.4 3.6.1.27	Metabolism--Lipid metabolism--Glycerophospholipid metabolism|Metabolism--Global and overview maps--Metabolic pathways

[denovogenes]_110427	48.77	4e-79	cpy:Cphy_0033	K12510	tadB	tight adherence protein B	--	--

[denovogenes]_110429	45.28	4e-78	paea:R70723_16735	K01992	ABC-2.P	ABC-2 type transport system permease protein	--	--

[denovogenes]_110431	86.61	2e-162	bvu:BVU_0199	K00243	K00243	uncharacterized protein	--	--

[denovogenes]_110432	100.00	0.0	bvu:BVU_2980	K07322	ytfE, scdA	regulator of cell morphogenesis and NO signaling	--	--

[denovogenes]_110433	46.72	1e-66	txy:Thexy_1387	K20074	prpC, phpP	PPM family protein phosphatase	3.1.3.16	--

[denovogenes]_110434	74.31	2e-143	ddh:Desde_0492	K02003	ABC.CD.A	putative ABC transport system ATP-binding protein	--	--

[denovogenes]_110436	64.57	4e-119	eel:EUBELI_01646	K07321	cooC	CO dehydrogenase maturation factor	--	--

[denovogenes]_110437	64.13	2e-92	cpro:CPRO_19300	K00558	DNMT1, dcm	DNA (cytosine-5)-methyltransferase 1	2.1.1.37	Metabolism--Amino acid metabolism--Cysteine and methionine metabolism|Metabolism--Global and overview maps--Metabolic pathways|Human Diseases--Cancers: Overview--MicroRNAs in cancer

[denovogenes]_110438	43.08	1e-61	eac:EAL2_c18460	K18672	dacA	diadenylate cyclase	2.7.7.85	--

[denovogenes]_110439	99.61	0.0	shq:A0259_07110	K01175	ybfF	esterase	3.1.-.-	--

[denovogenes]_11044	86.27	0.0	boa:Bovatus_02659	K01534	zntA	Cd2+/Zn2+-exporting ATPase	3.6.3.3 3.6.3.5	--

[denovogenes]_110441	46.05	3e-37	efe:EFER_2931	K11920	envY	AraC family transcriptional regulator	--	--

[denovogenes]_110442	99.21	0.0	cfd:CFNIH1_11620	K09473	puuD	gamma-glutamyl-gamma-aminobutyrate hydrolase	3.5.1.94	Metabolism--Amino acid metabolism--Arginine and proline metabolism|Metabolism--Global and overview maps--Metabolic pathways

[denovogenes]_110444	70.42	2e-125	lacy:A4V08_21225	K03763	DPO3A2, polC	DNA polymerase III subunit alpha, Gram-positive type	2.7.7.7	Metabolism--Nucleotide metabolism--Purine metabolism|Metabolism--Nucleotide metabolism--Pyrimidine metabolism|Metabolism--Global and overview maps--Metabolic pathways|Genetic Information Processing--Replication and repair--DNA replication|Genetic Information Processing--Replication and repair--Mismatch repair|Genetic Information Processing--Replication and repair--Homologous recombination

[denovogenes]_110445	51.01	1e-80	eha:Ethha_1020	K06173	truA, PUS1	tRNA pseudouridine38-40 synthase	5.4.99.12	--

[denovogenes]_110447	92.13	5e-179	boa:Bovatus_03802	K03269	lpxH	UDP-2,3-diacylglucosamine hydrolase	3.6.1.54	Metabolism--Glycan biosynthesis and metabolism--Lipopolysaccharide biosynthesis|Metabolism--Global and overview maps--Metabolic pathways

[denovogenes]_110449	68.50	2e-129	csh:Closa_0159	K03424	--	--	--	--

[denovogenes]_110450	95.29	6e-160	cif:AL515_18700	K03581	recD	exodeoxyribonuclease V alpha subunit	3.1.11.5	Genetic Information Processing--Replication and repair--Homologous recombination

[denovogenes]_110451	88.03	8e-150	bcel:BcellWH2_04286	K01990	ABC-2.A	ABC-2 type transport system ATP-binding protein	--	--

[denovogenes]_110452	99.61	0.0	fpla:A4U99_07605	K02346	DPO4, dinB	DNA polymerase IV	2.7.7.7	--

[denovogenes]_110453	98.04	0.0	cfd:CFNIH1_03320	K01485	codA	cytosine deaminase	3.5.4.1	Metabolism--Nucleotide metabolism--Pyrimidine metabolism|Metabolism--Amino acid metabolism--Arginine and proline metabolism|Metabolism--Global and overview maps--Metabolic pathways

[denovogenes]_110455	100.00	0.0	eih:ECOK1_0795	K01992	ABC-2.P	ABC-2 type transport system permease protein	--	--

[denovogenes]_110456	44.66	3e-44	ag:BAC80215	K19960	chnA	cyclohexanol dehydrogenase	1.1.1.245	Metabolism--Xenobiotics biodegradation and metabolism--Caprolactam degradation

[denovogenes]_110458	43.15	2e-69	rus:RBI_II00005	K00878	thiM	hydroxyethylthiazole kinase	2.7.1.50	Metabolism--Metabolism of cofactors and vitamins--Thiamine metabolism|Metabolism--Global and overview maps--Metabolic pathways

[denovogenes]_110459	76.02	7e-143	ibu:IB211_01472c	K01448	E3.5.1.28B, amiA, amiB, amiC	N-acetylmuramoyl-L-alanine amidase	3.5.1.28	Human Diseases--Drug resistance: Antimicrobial--Cationic antimicrobial peptide (CAMP) resistance

[denovogenes]_11046	75.64	0.0	pft:JBW_00473	K01547	kdpB	K+-transporting ATPase ATPase B chain	3.6.3.12	Environmental Information Processing--Signal transduction--Two-component system

[denovogenes]_110460	97.25	0.0	sens:Q786_00955	K01918	panC	pantoate--beta-alanine ligase	6.3.2.1	Metabolism--Metabolism of other amino acids--beta-Alanine metabolism|Metabolism--Metabolism of cofactors and vitamins--Pantothenate and CoA biosynthesis|Metabolism--Global and overview maps--Metabolic pathways|Metabolism--Global and overview maps--Biosynthesis of secondary metabolites

[denovogenes]_110461	85.33	2e-159	bhl:Bache_0775	K09817	znuC	zinc transport system ATP-binding protein	3.6.3.-	Environmental Information Processing--Membrane transport--ABC transporters

[denovogenes]_110462	100.00	1e-180	elh:ETEC_3497	K00024	mdh	malate dehydrogenase	1.1.1.37	Metabolism--Carbohydrate metabolism--Citrate cycle (TCA cycle)|Metabolism--Amino acid metabolism--Cysteine and methionine metabolism|Metabolism--Carbohydrate metabolism--Pyruvate metabolism|Metabolism--Carbohydrate metabolism--Glyoxylate and dicarboxylate metabolism|Metabolism--Energy metabolism--Methane metabolism|Metabolism--Energy metabolism--Carbon fixation in photosynthetic organisms|Metabolism--Energy metabolism--Carbon fixation pathways in prokaryotes|Metabolism--Global and overview maps--Metabolic pathways|Metabolism--Global and overview maps--Biosynthesis of secondary metabolites|Metabolism--Global and overview maps--Microbial metabolism in diverse environments|Metabolism--Global and overview maps--Biosynthesis of antibiotics|Metabolism--Global and overview maps--Carbon metabolism

[denovogenes]_110463	91.34	1e-143	fpla:A4U99_08560	K01992	ABC-2.P	ABC-2 type transport system permease protein	--	--

[denovogenes]_110464	86.61	1e-164	ebf:D782_3479	K00046	idnO	gluconate 5-dehydrogenase	1.1.1.69	--

[denovogenes]_110465	84.65	3e-155	bvu:BVU_3213	K10716	kch, trkA, mthK, pch	voltage-gated potassium channel	--	--

[denovogenes]_110466	100.00	0.0	sbc:SbBS512_E3506	K07229	yqjH	ferric-chelate reductase (NADPH)	1.16.1.9	--

[denovogenes]_110468	85.26	2e-158	cso:CLS_35300	K07160	K07160	UPF0271 protein	--	--

[denovogenes]_110469	79.92	2e-142	rto:RTO_16230	K02357	tsf, TSFM	elongation factor Ts	--	--

[denovogenes]_11047	41.01	1e-178	erb:A4V01_16550	K02123	ATPVI, ntpI, atpI	V/A-type H+/Na+-transporting ATPase subunit I	--	Metabolism--Energy metabolism--Oxidative phosphorylation|Metabolism--Global and overview maps--Metabolic pathways

[denovogenes]_110472	75.00	9e-144	bpb:bpr_I0929	K01815	kduI	4-deoxy-L-threo-5-hexosulose-uronate ketol-isomerase	5.3.1.17	Metabolism--Carbohydrate metabolism--Pentose and glucuronate interconversions

[denovogenes]_110473	97.23	0.0	bhl:Bache_2873	K00175	korB, oorB, oforB	2-oxoglutarate/2-oxoacid ferredoxin oxidoreductase subunit beta	1.2.7.3 1.2.7.11	Metabolism--Carbohydrate metabolism--Glycolysis / Gluconeogenesis|Metabolism--Carbohydrate metabolism--Citrate cycle (TCA cycle)|Metabolism--Carbohydrate metabolism--Pyruvate metabolism|Metabolism--Carbohydrate metabolism--Butanoate metabolism|Metabolism--Energy metabolism--Carbon fixation pathways in prokaryotes|Metabolism--Global and overview maps--Metabolic pathways|Metabolism--Global and overview maps--Microbial metabolism in diverse environments|Metabolism--Global and overview maps--Biosynthesis of antibiotics|Metabolism--Global and overview maps--Carbon metabolism

[denovogenes]_110475	49.80	5e-64	cpro:CPRO_14600	K20492	nisG, spaG, cprC	lantibiotic transport system permease protein	--	Environmental Information Processing--Membrane transport--ABC transporters|Environmental Information Processing--Signal transduction--Two-component system|Cellular Processes--Cellular community - prokaryotes--Quorum sensing

[denovogenes]_110476	81.89	2e-145	bprs:CK3_00370	K07335	bmpA, bmpB, tmpC	basic membrane protein A and related proteins	--	--

[denovogenes]_110480	97.24	0.0	cfd:CFNIH1_13770	K04835	mal	methylaspartate ammonia-lyase	4.3.1.2	Metabolism--Carbohydrate metabolism--Glyoxylate and dicarboxylate metabolism|Metabolism--Carbohydrate metabolism--C5-Branched dibasic acid metabolism|Metabolism--Global and overview maps--Metabolic pathways|Metabolism--Global and overview maps--Carbon metabolism

[denovogenes]_110484	42.52	2e-59	eac:EAL2_c18460	K18672	dacA	diadenylate cyclase	2.7.7.85	--

[denovogenes]_110486	76.80	5e-132	fpla:A4U99_11845	K01961	accC	acetyl-CoA carboxylase, biotin carboxylase subunit	6.4.1.2 6.3.4.14	Metabolism--Lipid metabolism--Fatty acid biosynthesis|Metabolism--Carbohydrate metabolism--Pyruvate metabolism|Metabolism--Carbohydrate metabolism--Propanoate metabolism|Metabolism--Energy metabolism--Carbon fixation pathways in prokaryotes|Metabolism--Global and overview maps--Metabolic pathways|Metabolism--Global and overview maps--Biosynthesis of secondary metabolites|Metabolism--Global and overview maps--Microbial metabolism in diverse environments|Metabolism--Global and overview maps--Biosynthesis of antibiotics|Metabolism--Global and overview maps--Carbon metabolism|Metabolism--Global and overview maps--Fatty acid metabolism

[denovogenes]_110487	60.94	7e-107	rto:RTO_30380	K03437	spoU	RNA methyltransferase, TrmH family	--	--

[denovogenes]_110490	40.24	1e-51	csc:Csac_2442	K03436	fruR2, fruR	DeoR family transcriptional regulator, fructose operon transcriptional repressor	--	--

[denovogenes]_110492	92.91	2e-175	bprs:CK3_09230	K02314	dnaB	replicative DNA helicase	3.6.4.12	Genetic Information Processing--Replication and repair--DNA replication|Cellular Processes--Cell growth and death--Cell cycle - Caulobacter

[denovogenes]_110494	47.54	5e-80	ddh:Desde_0335	K04069	pflA, pflC, pflE	pyruvate formate lyase activating enzyme	1.97.1.4	--

[denovogenes]_110495	68.11	3e-129	rho:RHOM_04150	K18581	ugl	unsaturated chondroitin disaccharide hydrolase	3.2.1.180	--

[denovogenes]_110496	46.59	8e-45	bdh:GV66_13410	K03587	ftsI	cell division protein FtsI (penicillin-binding protein 3)	--	Metabolism--Glycan biosynthesis and metabolism--Peptidoglycan biosynthesis|Human Diseases--Drug resistance: Antimicrobial--beta-Lactam resistance

[denovogenes]_110499	56.97	2e-83	cbi:CLJ_B3866	K06409	spoVB	stage V sporulation protein B	--	--

[denovogenes]_1105	94.62	0.0	pfre:RM25_1008	K02337	DPO3A1, dnaE	DNA polymerase III subunit alpha	2.7.7.7	Metabolism--Nucleotide metabolism--Purine metabolism|Metabolism--Nucleotide metabolism--Pyrimidine metabolism|Metabolism--Global and overview maps--Metabolic pathways|Genetic Information Processing--Replication and repair--DNA replication|Genetic Information Processing--Replication and repair--Mismatch repair|Genetic Information Processing--Replication and repair--Homologous recombination

[denovogenes]_11050	99.70	0.0	ecol:LY180_18360	K01176	AMY, amyA, malS	alpha-amylase	3.2.1.1	Metabolism--Carbohydrate metabolism--Starch and sucrose metabolism|Metabolism--Global and overview maps--Metabolic pathways|Organismal Systems--Digestive system--Carbohydrate digestion and absorption

[denovogenes]_110504	71.26	7e-129	clt:CM240_1749	K01486	ade	adenine deaminase	3.5.4.2	Metabolism--Nucleotide metabolism--Purine metabolism|Metabolism--Global and overview maps--Metabolic pathways

[denovogenes]_110508	87.45	8e-168	cle:Clole_4239	K09759	aspC	nondiscriminating aspartyl-tRNA synthetase	6.1.1.23	Genetic Information Processing--Translation--Aminoacyl-tRNA biosynthesis

[denovogenes]_110510	74.80	6e-137	gea:GARCT_02750	K04072	adhE	acetaldehyde dehydrogenase / alcohol dehydrogenase	1.2.1.10 1.1.1.1	Metabolism--Carbohydrate metabolism--Glycolysis / Gluconeogenesis|Metabolism--Lipid metabolism--Fatty acid degradation|Metabolism--Amino acid metabolism--Tyrosine metabolism|Metabolism--Carbohydrate metabolism--Pyruvate metabolism|Metabolism--Xenobiotics biodegradation and metabolism--Chloroalkane and chloroalkene degradation|Metabolism--Xenobiotics biodegradation and metabolism--Naphthalene degradation|Metabolism--Carbohydrate metabolism--Butanoate metabolism|Metabolism--Global and overview maps--Metabolic pathways|Metabolism--Global and overview maps--Biosynthesis of secondary metabolites|Metabolism--Global and overview maps--Microbial metabolism in diverse environments|Metabolism--Global and overview maps--Biosynthesis of antibiotics|Metabolism--Global and overview maps--Degradation of aromatic compounds

[denovogenes]_110511	83.14	2e-145	rch:RUM_06210	K09157	K09157	uncharacterized protein	--	--

[denovogenes]_110512	74.80	5e-139	mhg:MHY_12980	K03737	por, nifJ	pyruvate-ferredoxin/flavodoxin oxidoreductase	1.2.7.1 1.2.7.-	Metabolism--Carbohydrate metabolism--Glycolysis / Gluconeogenesis|Metabolism--Carbohydrate metabolism--Citrate cycle (TCA cycle)|Metabolism--Carbohydrate metabolism--Pyruvate metabolism|Metabolism--Carbohydrate metabolism--Butanoate metabolism|Metabolism--Energy metabolism--Carbon fixation pathways in prokaryotes|Metabolism--Global and overview maps--Metabolic pathways|Metabolism--Global and overview maps--Microbial metabolism in diverse environments|Metabolism--Global and overview maps--Biosynthesis of antibiotics|Metabolism--Global and overview maps--Carbon metabolism

[denovogenes]_110513	87.06	8e-155	cle:Clole_3217	K03458	TC.NCS2	nucleobase:cation symporter-2, NCS2 family	--	--

[denovogenes]_110518	70.52	1e-125	shi:Shel_01760	K01933	purM	phosphoribosylformylglycinamidine cyclo-ligase	6.3.3.1	Metabolism--Nucleotide metabolism--Purine metabolism|Metabolism--Global and overview maps--Metabolic pathways|Metabolism--Global and overview maps--Biosynthesis of secondary metabolites|Metabolism--Global and overview maps--Biosynthesis of antibiotics

[denovogenes]_110519	99.61	9e-175	elp:P12B_c3562	K03110	ftsY	fused signal recognition particle receptor	--	Cellular Processes--Cellular community - prokaryotes--Quorum sensing|Genetic Information Processing--Folding, sorting and degradation--Protein export|Environmental Information Processing--Membrane transport--Bacterial secretion system

[denovogenes]_110520	43.84	2e-54	bsu:BSU38910	K02479	K02479	two-component system, NarL family, response regulator	--	--

[denovogenes]_110521	42.03	6e-13	aaci:ASQ49_07140	K03975	dedA	membrane-associated protein	--	--

[denovogenes]_110522	97.24	0.0	pfre:RM25_1350	K00240	sdhB, frdB	succinate dehydrogenase / fumarate reductase, iron-sulfur subunit	1.3.5.1 1.3.5.4	Metabolism--Carbohydrate metabolism--Citrate cycle (TCA cycle)|Metabolism--Energy metabolism--Oxidative phosphorylation|Metabolism--Carbohydrate metabolism--Butanoate metabolism|Metabolism--Energy metabolism--Carbon fixation pathways in prokaryotes|Metabolism--Global and overview maps--Metabolic pathways|Metabolism--Global and overview maps--Biosynthesis of secondary metabolites|Metabolism--Global and overview maps--Microbial metabolism in diverse environments|Metabolism--Global and overview maps--Biosynthesis of antibiotics|Metabolism--Global and overview maps--Carbon metabolism

[denovogenes]_110523	99.21	0.0	btho:Btheta7330_04599	K03269	lpxH	UDP-2,3-diacylglucosamine hydrolase	3.6.1.54	Metabolism--Glycan biosynthesis and metabolism--Lipopolysaccharide biosynthesis|Metabolism--Global and overview maps--Metabolic pathways

[denovogenes]_110524	43.87	3e-63	eac:EAL2_c18460	K18672	dacA	diadenylate cyclase	2.7.7.85	--

[denovogenes]_110526	92.49	1e-169	pfre:RM25_1281	K02500	hisF	cyclase	4.1.3.-	Metabolism--Amino acid metabolism--Histidine metabolism|Metabolism--Global and overview maps--Metabolic pathways|Metabolism--Global and overview maps--Biosynthesis of secondary metabolites|Metabolism--Global and overview maps--Biosynthesis of amino acids

[denovogenes]_110527	100.00	0.0	btho:Btheta7330_04980	K00215	dapB	4-hydroxy-tetrahydrodipicolinate reductase	1.17.1.8	Metabolism--Biosynthesis of other secondary metabolites--Monobactam biosynthesis|Metabolism--Amino acid metabolism--Lysine biosynthesis|Metabolism--Global and overview maps--Metabolic pathways|Metabolism--Global and overview maps--Biosynthesis of secondary metabolites|Metabolism--Global and overview maps--Microbial metabolism in diverse environments|Metabolism--Global and overview maps--Biosynthesis of antibiotics|Metabolism--Global and overview maps--Biosynthesis of amino acids

[denovogenes]_110528	45.10	4e-68	afd:Alfi_0014	K07052	K07052	uncharacterized protein	--	--

[denovogenes]_11053	73.60	0.0	rto:RTO_07410	K12257	secDF	SecD/SecF fusion protein	--	Cellular Processes--Cellular community - prokaryotes--Quorum sensing|Genetic Information Processing--Folding, sorting and degradation--Protein export|Environmental Information Processing--Membrane transport--Bacterial secretion system

[denovogenes]_110530	100.00	0.0	btho:Btheta7330_03048	K00175	korB, oorB, oforB	2-oxoglutarate/2-oxoacid ferredoxin oxidoreductase subunit beta	1.2.7.3 1.2.7.11	Metabolism--Carbohydrate metabolism--Glycolysis / Gluconeogenesis|Metabolism--Carbohydrate metabolism--Citrate cycle (TCA cycle)|Metabolism--Carbohydrate metabolism--Pyruvate metabolism|Metabolism--Carbohydrate metabolism--Butanoate metabolism|Metabolism--Energy metabolism--Carbon fixation pathways in prokaryotes|Metabolism--Global and overview maps--Metabolic pathways|Metabolism--Global and overview maps--Microbial metabolism in diverse environments|Metabolism--Global and overview maps--Biosynthesis of antibiotics|Metabolism--Global and overview maps--Carbon metabolism

[denovogenes]_110532	93.31	0.0	pdi:BDI_1392	K00175	korB, oorB, oforB	2-oxoglutarate/2-oxoacid ferredoxin oxidoreductase subunit beta	1.2.7.3 1.2.7.11	Metabolism--Carbohydrate metabolism--Glycolysis / Gluconeogenesis|Metabolism--Carbohydrate metabolism--Citrate cycle (TCA cycle)|Metabolism--Carbohydrate metabolism--Pyruvate metabolism|Metabolism--Carbohydrate metabolism--Butanoate metabolism|Metabolism--Energy metabolism--Carbon fixation pathways in prokaryotes|Metabolism--Global and overview maps--Metabolic pathways|Metabolism--Global and overview maps--Microbial metabolism in diverse environments|Metabolism--Global and overview maps--Biosynthesis of antibiotics|Metabolism--Global and overview maps--Carbon metabolism

[denovogenes]_110533	90.16	1e-164	pdi:BDI_1400	K07164	K07164	uncharacterized protein	--	--

[denovogenes]_110534	69.05	2e-127	scc:Spico_1577	K01995	livG	branched-chain amino acid transport system ATP-binding protein	--	Environmental Information Processing--Membrane transport--ABC transporters|Cellular Processes--Cellular community - prokaryotes--Quorum sensing

[denovogenes]_110535	73.23	4e-134	pdi:BDI_0805	K02477	K02477	two-component system, LytT family, response regulator	--	--

[denovogenes]_110536	53.46	3e-78	pary:A4V02_08670	K10716	kch, trkA, mthK, pch	voltage-gated potassium channel	--	--

[denovogenes]_110537	99.61	0.0	stb:SGPB_0718	K03481	glvR	RpiR family transcriptional regulator, glv operon transcriptional regulator	--	--

[denovogenes]_11054	40.51	2e-121	cpel:CPEL_0513	K07317	K07317	adenine-specific DNA-methyltransferase	2.1.1.72	--

[denovogenes]_110540	81.10	3e-144	pdi:BDI_1805	K04652	hypB	hydrogenase nickel incorporation protein HypB	--	--

[denovogenes]_110542	97.64	0.0	bfb:VU15_01650	K03269	lpxH	UDP-2,3-diacylglucosamine hydrolase	3.6.1.54	Metabolism--Glycan biosynthesis and metabolism--Lipopolysaccharide biosynthesis|Metabolism--Global and overview maps--Metabolic pathways

[denovogenes]_110543	42.74	2e-62	elm:ELI_0774	K02190	cbiK	sirohydrochlorin cobaltochelatase	4.99.1.3	Metabolism--Metabolism of cofactors and vitamins--Porphyrin and chlorophyll metabolism|Metabolism--Global and overview maps--Metabolic pathways

[denovogenes]_110544	100.00	3e-177	paep:PA1S_00950	K00059	fabG	3-oxoacyl-[acyl-carrier protein] reductase	1.1.1.100	Metabolism--Lipid metabolism--Fatty acid biosynthesis|Metabolism--Metabolism of cofactors and vitamins--Biotin metabolism|Metabolism--Lipid metabolism--Biosynthesis of unsaturated fatty acids|Metabolism--Global and overview maps--Metabolic pathways|Metabolism--Global and overview maps--Fatty acid metabolism

[denovogenes]_110546	62.95	2e-102	dvl:Dvul_0267	K00979	kdsB	3-deoxy-manno-octulosonate cytidylyltransferase (CMP-KDO synthetase)	2.7.7.38	Metabolism--Glycan biosynthesis and metabolism--Lipopolysaccharide biosynthesis|Metabolism--Global and overview maps--Metabolic pathways

[denovogenes]_110547	48.61	4e-72	eba:p2B9	K20266	trbJ	type IV secretion system protein TrbJ	--	Cellular Processes--Cellular community - prokaryotes--Quorum sensing

[denovogenes]_110549	84.65	9e-159	pdi:BDI_1570	K03768	PPIB, ppiB	peptidyl-prolyl cis-trans isomerase B (cyclophilin B)	5.2.1.8	--

[denovogenes]_110550	73.71	6e-133	rim:ROI_07880	K01995	livG	branched-chain amino acid transport system ATP-binding protein	--	Environmental Information Processing--Membrane transport--ABC transporters|Cellular Processes--Cellular community - prokaryotes--Quorum sensing

[denovogenes]_110553	91.73	2e-170	pdi:BDI_2559	K03496	parA, soj	chromosome partitioning protein	--	--

[denovogenes]_110554	40.23	5e-49	fpl:Ferp_1187	K04114	bcrA, badF	benzoyl-CoA reductase subunit A	1.3.7.8	Metabolism--Xenobiotics biodegradation and metabolism--Benzoate degradation|Metabolism--Global and overview maps--Metabolic pathways|Metabolism--Global and overview maps--Microbial metabolism in diverse environments|Metabolism--Global and overview maps--Degradation of aromatic compounds

[denovogenes]_110555	43.44	2e-57	dpg:DESPIGER_1457	K07090	K07090	uncharacterized protein	--	--

[denovogenes]_110556	56.69	2e-96	sfw:WN53_25780	K00065	kduD	2-deoxy-D-gluconate 3-dehydrogenase	1.1.1.125	Metabolism--Carbohydrate metabolism--Pentose and glucuronate interconversions

[denovogenes]_110557	78.35	8e-148	dvm:DvMF_2816	K00208	fabI	enoyl-[acyl-carrier protein] reductase I	1.3.1.9 1.3.1.10	Metabolism--Lipid metabolism--Fatty acid biosynthesis|Metabolism--Metabolism of cofactors and vitamins--Biotin metabolism|Metabolism--Global and overview maps--Metabolic pathways|Metabolism--Global and overview maps--Fatty acid metabolism

[denovogenes]_11056	92.32	0.0	lacy:A4V08_14645	K01537	E3.6.3.8	Ca2+-transporting ATPase	3.6.3.8	--

[denovogenes]_110561	56.22	7e-99	fpla:A4U99_06680	K02013	ABC.FEV.A	iron complex transport system ATP-binding protein	3.6.3.34	Environmental Information Processing--Membrane transport--ABC transporters

[denovogenes]_110563	59.29	2e-110	har:HEAR1136	K00978	rfbF	glucose-1-phosphate cytidylyltransferase	2.7.7.33	Metabolism--Carbohydrate metabolism--Starch and sucrose metabolism|Metabolism--Carbohydrate metabolism--Amino sugar and nucleotide sugar metabolism|Metabolism--Global and overview maps--Metabolic pathways

[denovogenes]_110564	61.02	2e-106	sbc:SbBS512_E1900	K03521	fixA, etfB	electron transfer flavoprotein beta subunit	--	--

[denovogenes]_110565	42.80	1e-64	nko:Niako_6782	K13002	wbyL	glycosyltransferase	2.4.1.-	--

[denovogenes]_110566	100.00	0.0	paeo:M801_1233	K01482	DDAH, ddaH	dimethylargininase	3.5.3.18	--

[denovogenes]_110567	45.60	2e-71	nko:Niako_6782	K13002	wbyL	glycosyltransferase	2.4.1.-	--

[denovogenes]_110570	93.70	5e-176	lacy:A4V08_34865	K07124	K07124	uncharacterized protein	--	--

[denovogenes]_110571	40.35	2e-47	pen:PSEEN2139	K01071	MCH	medium-chain acyl-[acyl-carrier-protein] hydrolase	3.1.2.21	Metabolism--Lipid metabolism--Fatty acid biosynthesis|Metabolism--Global and overview maps--Metabolic pathways

[denovogenes]_110572	98.82	0.0	cfd:CFNIH1_18845	K01096	pgpB	phosphatidylglycerophosphatase B	3.1.3.27 3.1.3.81 3.1.3.4 3.6.1.27	Metabolism--Lipid metabolism--Glycerophospholipid metabolism|Metabolism--Global and overview maps--Metabolic pathways

[denovogenes]_110573	40.48	2e-25	bprm:CL3_14780	K07720	yesN	two-component system, response regulator YesN	--	Environmental Information Processing--Signal transduction--Two-component system

[denovogenes]_110577	96.85	0.0	lacy:A4V08_19095	K02028	ABC.PA.A	polar amino acid transport system ATP-binding protein	3.6.3.21	--

[denovogenes]_110578	100.00	7e-174	paeo:M801_2853	K07052	K07052	uncharacterized protein	--	--

[denovogenes]_110579	88.84	5e-166	csh:Closa_3745	K01265	map	methionyl aminopeptidase	3.4.11.18	--

[denovogenes]_11058	76.60	0.0	dvm:DvMF_2576	K01547	kdpB	K+-transporting ATPase ATPase B chain	3.6.3.12	Environmental Information Processing--Signal transduction--Two-component system

[denovogenes]_110581	94.09	2e-179	lacy:A4V08_22710	K09121	K09121	uncharacterized protein	--	--

[denovogenes]_110582	79.84	2e-135	dfi:AXF13_12040	K07793	tctA	putative tricarboxylic transport membrane protein	--	Environmental Information Processing--Signal transduction--Two-component system

[denovogenes]_110583	99.61	0.0	paeg:AI22_23370	K00979	kdsB	3-deoxy-manno-octulosonate cytidylyltransferase (CMP-KDO synthetase)	2.7.7.38	Metabolism--Glycan biosynthesis and metabolism--Lipopolysaccharide biosynthesis|Metabolism--Global and overview maps--Metabolic pathways

[denovogenes]_110584	99.61	0.0	bfg:BF638R_3308	K03783	punA, PNP	purine-nucleoside phosphorylase	2.4.2.1	Metabolism--Nucleotide metabolism--Purine metabolism|Metabolism--Nucleotide metabolism--Pyrimidine metabolism|Metabolism--Metabolism of cofactors and vitamins--Nicotinate and nicotinamide metabolism|Metabolism--Global and overview maps--Metabolic pathways|Metabolism--Global and overview maps--Biosynthesis of secondary metabolites

[denovogenes]_110587	65.34	6e-108	csh:Closa_2384	K02233	E2.7.8.26, cobS, cobV	adenosylcobinamide-GDP ribazoletransferase	2.7.8.26	Metabolism--Metabolism of cofactors and vitamins--Porphyrin and chlorophyll metabolism|Metabolism--Global and overview maps--Metabolic pathways

[denovogenes]_110588	100.00	0.0	paeo:M801_920	K10025	aotP	arginine/ornithine transport system ATP-binding protein	3.6.3.-	Environmental Information Processing--Membrane transport--ABC transporters

[denovogenes]_110589	84.25	1e-155	lacy:A4V08_15920	K11991	tadA	tRNA(adenine34) deaminase	3.5.4.33	--

[denovogenes]_110593	45.29	9e-49	pen:PSEEN2139	K01071	MCH	medium-chain acyl-[acyl-carrier-protein] hydrolase	3.1.2.21	Metabolism--Lipid metabolism--Fatty acid biosynthesis|Metabolism--Global and overview maps--Metabolic pathways

[denovogenes]_110596	93.70	4e-179	lacy:A4V08_14805	K03424	--	--	--	--

[denovogenes]_110597	80.78	3e-142	lacy:A4V08_24915	K00615	E2.2.1.1, tktA, tktB	transketolase	2.2.1.1	Metabolism--Carbohydrate metabolism--Pentose phosphate pathway|Metabolism--Energy metabolism--Carbon fixation in photosynthetic organisms|Metabolism--Metabolism of terpenoids and polyketides--Biosynthesis of ansamycins|Metabolism--Global and overview maps--Metabolic pathways|Metabolism--Global and overview maps--Biosynthesis of secondary metabolites|Metabolism--Global and overview maps--Microbial metabolism in diverse environments|Metabolism--Global and overview maps--Biosynthesis of antibiotics|Metabolism--Global and overview maps--Carbon metabolism|Metabolism--Global and overview maps--Biosynthesis of amino acids

[denovogenes]_110599	93.69	5e-143	lacy:A4V08_30600	K06889	K06889	uncharacterized protein	--	--

[denovogenes]_1106	99.83	0.0	stb:SGPB_0083	K03043	rpoB	DNA-directed RNA polymerase subunit beta	2.7.7.6	Metabolism--Nucleotide metabolism--Purine metabolism|Metabolism--Nucleotide metabolism--Pyrimidine metabolism|Metabolism--Global and overview maps--Metabolic pathways|Genetic Information Processing--Transcription--RNA polymerase

[denovogenes]_110606	51.37	1e-89	csb:CLSA_c26500	K11041	eta	exfoliative toxin A/B	--	Human Diseases--Infectious diseases: Bacterial--Staphylococcus aureus infection

[denovogenes]_110607	62.30	4e-105	cgo:Corgl_0212	K06898	K06898	uncharacterized protein	--	--

[denovogenes]_110609	99.61	0.0	bbrd:BBBR_0132	K00928	lysC	aspartate kinase	2.7.2.4	Metabolism--Amino acid metabolism--Glycine, serine and threonine metabolism|Metabolism--Biosynthesis of other secondary metabolites--Monobactam biosynthesis|Metabolism--Amino acid metabolism--Cysteine and methionine metabolism|Metabolism--Amino acid metabolism--Lysine biosynthesis|Metabolism--Global and overview maps--Metabolic pathways|Metabolism--Global and overview maps--Biosynthesis of secondary metabolites|Metabolism--Global and overview maps--Microbial metabolism in diverse environments|Metabolism--Global and overview maps--Biosynthesis of antibiotics|Metabolism--Global and overview maps--2-Oxocarboxylic acid metabolism|Metabolism--Global and overview maps--Biosynthesis of amino acids

[denovogenes]_11061	100.00	0.0	psg:G655_02920	K02316	dnaG	DNA primase	2.7.7.-	Genetic Information Processing--Replication and repair--DNA replication

[denovogenes]_110610	84.19	5e-167	pdi:BDI_1111	K01142	E3.1.11.2, xthA	exodeoxyribonuclease III	3.1.11.2	Genetic Information Processing--Replication and repair--Base excision repair

[denovogenes]_110611	77.25	5e-142	cso:CLS_36590	K03431	glmM	phosphoglucosamine mutase	5.4.2.10	Metabolism--Carbohydrate metabolism--Amino sugar and nucleotide sugar metabolism|Metabolism--Global and overview maps--Metabolic pathways|Metabolism--Global and overview maps--Biosynthesis of antibiotics

[denovogenes]_110614	40.96	1e-47	pfe:PSF113_4443	K07637	phoQ	two-component system, OmpR family, sensor histidine kinase PhoQ	2.7.13.3	Human Diseases--Drug resistance: Antimicrobial--Cationic antimicrobial peptide (CAMP) resistance|Environmental Information Processing--Signal transduction--Two-component system

[denovogenes]_110615	90.94	1e-172	csh:Closa_0365	K02036	pstB	phosphate transport system ATP-binding protein	3.6.3.27	Environmental Information Processing--Membrane transport--ABC transporters

[denovogenes]_110617	45.49	4e-61	asb:RATSFB_1182	K18672	dacA	diadenylate cyclase	2.7.7.85	--

[denovogenes]_110620	100.00	0.0	paeo:M801_2962	K00322	sthA, udhA	NAD(P) transhydrogenase	1.6.1.1	Metabolism--Metabolism of cofactors and vitamins--Nicotinate and nicotinamide metabolism|Metabolism--Global and overview maps--Metabolic pathways

[denovogenes]_110623	55.88	1e-98	bpw:WESB_1273	K07045	K07045	uncharacterized protein	--	--

[denovogenes]_110624	77.11	6e-144	pdi:BDI_1147	K06167	phnP	phosphoribosyl 1,2-cyclic phosphate phosphodiesterase	3.1.4.55	Metabolism--Metabolism of other amino acids--Phosphonate and phosphinate metabolism

[denovogenes]_110625	100.00	0.0	eoh:ECO103_1988	K16135	--	--	--	--

[denovogenes]_110629	90.94	1e-176	bcel:BcellWH2_02190	K03269	lpxH	UDP-2,3-diacylglucosamine hydrolase	3.6.1.54	Metabolism--Glycan biosynthesis and metabolism--Lipopolysaccharide biosynthesis|Metabolism--Global and overview maps--Metabolic pathways

[denovogenes]_11063	98.96	0.0	cfd:CFNIH1_05465	K01176	AMY, amyA, malS	alpha-amylase	3.2.1.1	Metabolism--Carbohydrate metabolism--Starch and sucrose metabolism|Metabolism--Global and overview maps--Metabolic pathways|Organismal Systems--Digestive system--Carbohydrate digestion and absorption

[denovogenes]_110631	77.47	2e-152	pdi:BDI_3702	K01776	murI	glutamate racemase	5.1.1.3	Metabolism--Metabolism of other amino acids--D-Glutamine and D-glutamate metabolism|Metabolism--Global and overview maps--Metabolic pathways

[denovogenes]_110633	98.03	0.0	pdi:BDI_1570	K03768	PPIB, ppiB	peptidyl-prolyl cis-trans isomerase B (cyclophilin B)	5.2.1.8	--

[denovogenes]_110634	77.02	2e-133	ele:Elen_2049	K00973	E2.7.7.24, rfbA, rffH	glucose-1-phosphate thymidylyltransferase	2.7.7.24	Metabolism--Biosynthesis of other secondary metabolites--Streptomycin biosynthesis|Metabolism--Metabolism of terpenoids and polyketides--Polyketide sugar unit biosynthesis|Metabolism--Biosynthesis of other secondary metabolites--Acarbose and validamycin biosynthesis|Metabolism--Global and overview maps--Biosynthesis of antibiotics

[denovogenes]_110635	90.04	2e-161	csh:Closa_2184	K02003	ABC.CD.A	putative ABC transport system ATP-binding protein	--	--

[denovogenes]_110636	100.00	0.0	paeo:M801_4023	K02030	ABC.PA.S	polar amino acid transport system substrate-binding protein	--	--

[denovogenes]_110637	45.38	2e-69	ols:Olsu_0609	K07271	licD	lipopolysaccharide cholinephosphotransferase	2.7.8.-	--

[denovogenes]_110638	87.80	2e-151	csh:Closa_2346	K03525	coaX	type III pantothenate kinase	2.7.1.33	Metabolism--Metabolism of cofactors and vitamins--Pantothenate and CoA biosynthesis|Metabolism--Global and overview maps--Metabolic pathways

[denovogenes]_110640	75.29	2e-136	cgo:Corgl_0276	K02071	metN	D-methionine transport system ATP-binding protein	--	Environmental Information Processing--Membrane transport--ABC transporters

[denovogenes]_110641	57.26	2e-35	arh:AHiyo8_45800	K01007	pps, ppsA	pyruvate, water dikinase	2.7.9.2	Metabolism--Carbohydrate metabolism--Pyruvate metabolism|Metabolism--Energy metabolism--Methane metabolism|Metabolism--Energy metabolism--Carbon fixation pathways in prokaryotes|Metabolism--Global and overview maps--Metabolic pathways|Metabolism--Global and overview maps--Microbial metabolism in diverse environments|Metabolism--Global and overview maps--Carbon metabolism

[denovogenes]_110643	87.84	3e-161	lacy:A4V08_31090	K02412	fliI	flagellum-specific ATP synthase	3.6.3.14	Cellular Processes--Cell motility--Flagellar assembly

[denovogenes]_110645	59.26	2e-95	afd:Alfi_3057	K18139	oprM, emhC, ttgC, cusC, adeK, smeF, mtrE, cmeC, gesC	outer membrane protein, multidrug efflux system	--	Human Diseases--Drug resistance: Antimicrobial--beta-Lactam resistance|Cellular Processes--Cellular community - prokaryotes--Quorum sensing

[denovogenes]_110649	41.95	3e-56	mhg:MHY_13590	K03070	secA	preprotein translocase subunit SecA	--	Cellular Processes--Cellular community - prokaryotes--Quorum sensing|Genetic Information Processing--Folding, sorting and degradation--Protein export|Environmental Information Processing--Membrane transport--Bacterial secretion system

[denovogenes]_110651	47.22	4e-77	pln:Plano_2882	K00525	E1.17.4.1A, nrdA, nrdE	ribonucleoside-diphosphate reductase alpha chain	1.17.4.1	Metabolism--Nucleotide metabolism--Purine metabolism|Metabolism--Nucleotide metabolism--Pyrimidine metabolism|Metabolism--Global and overview maps--Metabolic pathways

[denovogenes]_110654	72.44	8e-138	ova:OBV_04200	K02003	ABC.CD.A	putative ABC transport system ATP-binding protein	--	--

[denovogenes]_110655	84.98	7e-162	csh:Closa_0959	K03527	ispH, lytB	4-hydroxy-3-methylbut-2-en-1-yl diphosphate reductase	1.17.7.4	Metabolism--Metabolism of terpenoids and polyketides--Terpenoid backbone biosynthesis|Metabolism--Global and overview maps--Metabolic pathways|Metabolism--Global and overview maps--Biosynthesis of secondary metabolites|Metabolism--Global and overview maps--Biosynthesis of antibiotics

[denovogenes]_110656	79.75	3e-135	pdi:BDI_3169	K03092	SIG54, rpoN	RNA polymerase sigma-54 factor	--	Environmental Information Processing--Signal transduction--Two-component system|Cellular Processes--Cellular community - prokaryotes--Biofilm formation - Vibrio cholerae

[denovogenes]_110657	97.61	1e-179	bcel:BcellWH2_03926	K03496	parA, soj	chromosome partitioning protein	--	--

[denovogenes]_110660	72.33	1e-129	csh:Closa_0989	K00375	K00375	GntR family transcriptional regulator / MocR family aminotransferase	--	--

[denovogenes]_110661	67.59	8e-117	cgo:Corgl_0871	K00554	trmD	tRNA (guanine37-N1)-methyltransferase	2.1.1.228	--

[denovogenes]_110662	100.00	2e-180	pdf:CD630DERM_25790	K20074	prpC, phpP	PPM family protein phosphatase	3.1.3.16	--

[denovogenes]_110664	40.31	9e-60	sagr:SAIL_6330	K01537	E3.6.3.8	Ca2+-transporting ATPase	3.6.3.8	--

[denovogenes]_110665	83.14	9e-162	pdi:BDI_3155	K00876	udk, UCK	uridine kinase	2.7.1.48	Metabolism--Nucleotide metabolism--Pyrimidine metabolism|Metabolism--Xenobiotics biodegradation and metabolism--Drug metabolism - other enzymes|Metabolism--Global and overview maps--Metabolic pathways

[denovogenes]_110666	96.08	1e-164	cfd:CFNIH1_21595	K07799	mdtA	membrane fusion protein, multidrug efflux system	--	Environmental Information Processing--Signal transduction--Two-component system

[denovogenes]_110668	82.28	8e-144	csh:Closa_1296	K01883	CARS, cysS	cysteinyl-tRNA synthetase	6.1.1.16	Genetic Information Processing--Translation--Aminoacyl-tRNA biosynthesis

[denovogenes]_11067	82.89	0.0	pdi:BDI_2290	K07037	K07037	uncharacterized protein	--	--

[denovogenes]_110670	55.64	2e-89	byl:A4V09_23075	K02598	nirC	nitrite transporter NirC	--	--

[denovogenes]_110671	100.00	1e-180	cdl:CDR20291_2510	K09157	K09157	uncharacterized protein	--	--

[denovogenes]_110674	100.00	7e-176	ele:Elen_3045	K07090	K07090	uncharacterized protein	--	--

[denovogenes]_110676	40.74	3e-40	ssw:SSGZ1_0480	K08600	srtB	sortase B	3.4.22.70	--

[denovogenes]_110679	86.61	8e-169	bfb:VU15_06235	K03426	E3.6.1.22, NUDT12, nudC	NAD+ diphosphatase	3.6.1.22	Metabolism--Metabolism of cofactors and vitamins--Nicotinate and nicotinamide metabolism|Metabolism--Global and overview maps--Metabolic pathways|Cellular Processes--Transport and catabolism--Peroxisome

[denovogenes]_110680	76.49	1e-125	cgo:Corgl_0882	K04094	trmFO, gid	methylenetetrahydrofolate--tRNA-(uracil-5-)-methyltransferase	2.1.1.74	--

[denovogenes]_110681	100.00	0.0	cfd:CFNIH1_01615	K03472	epd	D-erythrose 4-phosphate dehydrogenase	1.2.1.72	Metabolism--Metabolism of cofactors and vitamins--Vitamin B6 metabolism|Metabolism--Global and overview maps--Metabolic pathways

[denovogenes]_110682	78.43	9e-137	ega:AL523_00255	K01998	livM	branched-chain amino acid transport system permease protein	--	Environmental Information Processing--Membrane transport--ABC transporters|Cellular Processes--Cellular community - prokaryotes--Quorum sensing

[denovogenes]_110684	95.69	0.0	btho:Btheta7330_01076	K03781	katE, CAT, catB, srpA	catalase	1.11.1.6	Metabolism--Amino acid metabolism--Tryptophan metabolism|Metabolism--Carbohydrate metabolism--Glyoxylate and dicarboxylate metabolism|Metabolism--Global and overview maps--Biosynthesis of secondary metabolites|Metabolism--Global and overview maps--Biosynthesis of antibiotics|Metabolism--Global and overview maps--Carbon metabolism|Environmental Information Processing--Signal transduction--MAPK signaling pathway - yeast|Environmental Information Processing--Signal transduction--MAPK signaling pathway - plant|Environmental Information Processing--Signal transduction--FoxO signaling pathway|Cellular Processes--Transport and catabolism--Peroxisome|Organismal Systems--Aging--Longevity regulating pathway|Organismal Systems--Aging--Longevity regulating pathway - worm|Organismal Systems--Aging--Longevity regulating pathway - multiple species|Human Diseases--Neurodegenerative diseases--Amyotrophic lateral sclerosis (ALS)

[denovogenes]_110686	99.61	0.0	pdi:BDI_3727	K01893	NARS, asnS	asparaginyl-tRNA synthetase	6.1.1.22	Genetic Information Processing--Translation--Aminoacyl-tRNA biosynthesis

[denovogenes]_110689	98.82	0.0	pvl:AOB99_08445	K01679	E4.2.1.2B, fumC	fumarate hydratase, class II	4.2.1.2	Metabolism--Carbohydrate metabolism--Citrate cycle (TCA cycle)|Metabolism--Carbohydrate metabolism--Pyruvate metabolism|Metabolism--Energy metabolism--Carbon fixation pathways in prokaryotes|Metabolism--Global and overview maps--Metabolic pathways|Metabolism--Global and overview maps--Biosynthesis of secondary metabolites|Metabolism--Global and overview maps--Microbial metabolism in diverse environments|Metabolism--Global and overview maps--Biosynthesis of antibiotics|Metabolism--Global and overview maps--Carbon metabolism|Human Diseases--Cancers: Overview--Pathways in cancer|Human Diseases--Cancers: Specific types--Renal cell carcinoma

[denovogenes]_110690	95.29	3e-170	bfs:BF9343_0465	K20265	gadC	glutamate:GABA antiporter	--	Cellular Processes--Cellular community - prokaryotes--Quorum sensing

[denovogenes]_110693	41.94	3e-45	mmh:Mmah_0608	K02198	ccmF	cytochrome c-type biogenesis protein CcmF	--	--

[denovogenes]_110694	61.20	7e-104	ibu:IB211_00101	K00384	trxB	thioredoxin reductase (NADPH)	1.8.1.9	Metabolism--Nucleotide metabolism--Pyrimidine metabolism|Metabolism--Metabolism of other amino acids--Selenocompound metabolism

[denovogenes]_110695	66.81	3e-110	stu:STH8232_1720	K03702	uvrB	excinuclease ABC subunit B	--	Genetic Information Processing--Replication and repair--Nucleotide excision repair

[denovogenes]_110698	69.83	5e-117	afd:Alfi_1390	K02687	prmA	ribosomal protein L11 methyltransferase	2.1.1.-	--

[denovogenes]_11070	84.02	0.0	ert:EUR_07600	K03169	topB	DNA topoisomerase III	5.99.1.2	--

[denovogenes]_110701	68.63	4e-128	clt:CM240_1749	K01486	ade	adenine deaminase	3.5.4.2	Metabolism--Nucleotide metabolism--Purine metabolism|Metabolism--Global and overview maps--Metabolic pathways

[denovogenes]_110703	70.75	1e-131	cbz:Cbs_4909	K00700	GBE1, glgB	1,4-alpha-glucan branching enzyme	2.4.1.18	Metabolism--Carbohydrate metabolism--Starch and sucrose metabolism|Metabolism--Global and overview maps--Metabolic pathways|Metabolism--Global and overview maps--Biosynthesis of secondary metabolites

[denovogenes]_110704	42.41	1e-60	dsy:DSY3417	K07484	K07484	transposase	--	--

[denovogenes]_110712	77.25	1e-140	erb:A4V01_07245	K00013	hisD	histidinol dehydrogenase	1.1.1.23	Metabolism--Amino acid metabolism--Histidine metabolism|Metabolism--Global and overview maps--Metabolic pathways|Metabolism--Global and overview maps--Biosynthesis of secondary metabolites|Metabolism--Global and overview maps--Biosynthesis of amino acids

[denovogenes]_110713	88.63	3e-169	lacy:A4V08_00885	K01706	gudD	glucarate dehydratase	4.2.1.40	Metabolism--Carbohydrate metabolism--Ascorbate and aldarate metabolism|Metabolism--Global and overview maps--Metabolic pathways

[denovogenes]_110714	50.97	2e-86	has:Halsa_1077	K01207	nagZ	beta-N-acetylhexosaminidase	3.2.1.52	Metabolism--Carbohydrate metabolism--Amino sugar and nucleotide sugar metabolism|Metabolism--Glycan biosynthesis and metabolism--Glycosaminoglycan degradation|Metabolism--Global and overview maps--Metabolic pathways|Human Diseases--Drug resistance: Antimicrobial--beta-Lactam resistance

[denovogenes]_110719	56.47	9e-103	ecoo:ECRM13514_1203	K11931	pgaB	biofilm PGA synthesis lipoprotein PgaB	3.-.-.-	Cellular Processes--Cellular community - prokaryotes--Biofilm formation - Escherichia coli

[denovogenes]_11072	99.56	0.0	boa:Bovatus_01156	K03702	uvrB	excinuclease ABC subunit B	--	Genetic Information Processing--Replication and repair--Nucleotide excision repair

[denovogenes]_110722	46.46	8e-71	olo:ADJ70_12655	K00014	aroE	shikimate dehydrogenase	1.1.1.25	Metabolism--Amino acid metabolism--Phenylalanine, tyrosine and tryptophan biosynthesis|Metabolism--Global and overview maps--Metabolic pathways|Metabolism--Global and overview maps--Biosynthesis of secondary metabolites|Metabolism--Global and overview maps--Biosynthesis of antibiotics|Metabolism--Global and overview maps--Biosynthesis of amino acids

[denovogenes]_110723	99.22	0.0	cif:AL515_13840	K03587	ftsI	cell division protein FtsI (penicillin-binding protein 3)	--	Metabolism--Glycan biosynthesis and metabolism--Peptidoglycan biosynthesis|Human Diseases--Drug resistance: Antimicrobial--beta-Lactam resistance

[denovogenes]_110724	100.00	0.0	sdz:Asd1617_02045	K00673	astA	arginine N-succinyltransferase	2.3.1.109	Metabolism--Amino acid metabolism--Arginine and proline metabolism|Metabolism--Global and overview maps--Metabolic pathways

[denovogenes]_110725	100.00	0.0	cfd:CFNIH1_10125	K03587	ftsI	cell division protein FtsI (penicillin-binding protein 3)	--	Metabolism--Glycan biosynthesis and metabolism--Peptidoglycan biosynthesis|Human Diseases--Drug resistance: Antimicrobial--beta-Lactam resistance

[denovogenes]_110726	96.86	2e-171	afd:Alfi_1195	K03310	TC.AGCS	alanine or glycine:cation symporter, AGCS family	--	--

[denovogenes]_110728	63.92	2e-106	lmob:BN419_1563	K00962	pnp, PNPT1	polyribonucleotide nucleotidyltransferase	2.7.7.8	Metabolism--Nucleotide metabolism--Purine metabolism|Metabolism--Nucleotide metabolism--Pyrimidine metabolism|Genetic Information Processing--Folding, sorting and degradation--RNA degradation

[denovogenes]_110729	89.02	1e-170	yel:LC20_01211	K02337	DPO3A1, dnaE	DNA polymerase III subunit alpha	2.7.7.7	Metabolism--Nucleotide metabolism--Purine metabolism|Metabolism--Nucleotide metabolism--Pyrimidine metabolism|Metabolism--Global and overview maps--Metabolic pathways|Genetic Information Processing--Replication and repair--DNA replication|Genetic Information Processing--Replication and repair--Mismatch repair|Genetic Information Processing--Replication and repair--Homologous recombination

[denovogenes]_110731	98.25	6e-167	boa:Bovatus_01962	K14742	tsaB	tRNA threonylcarbamoyladenosine biosynthesis protein TsaB	--	--

[denovogenes]_110733	48.25	1e-76	boa:Bovatus_04431	K00674	dapD	2,3,4,5-tetrahydropyridine-2-carboxylate N-succinyltransferase	2.3.1.117	Metabolism--Amino acid metabolism--Lysine biosynthesis|Metabolism--Global and overview maps--Metabolic pathways|Metabolism--Global and overview maps--Microbial metabolism in diverse environments|Metabolism--Global and overview maps--Biosynthesis of amino acids

[denovogenes]_110735	99.61	0.0	boa:Bovatus_04286	K03496	parA, soj	chromosome partitioning protein	--	--

[denovogenes]_110736	94.88	0.0	bxy:BXY_05680	K03437	spoU	RNA methyltransferase, TrmH family	--	--

[denovogenes]_110737	99.61	0.0	boa:Bovatus_03802	K03269	lpxH	UDP-2,3-diacylglucosamine hydrolase	3.6.1.54	Metabolism--Glycan biosynthesis and metabolism--Lipopolysaccharide biosynthesis|Metabolism--Global and overview maps--Metabolic pathways

[denovogenes]_110738	74.21	5e-135	llx:NCDO2118_1224	K02500	hisF	cyclase	4.1.3.-	Metabolism--Amino acid metabolism--Histidine metabolism|Metabolism--Global and overview maps--Metabolic pathways|Metabolism--Global and overview maps--Biosynthesis of secondary metabolites|Metabolism--Global and overview maps--Biosynthesis of amino acids

[denovogenes]_110742	42.06	2e-57	ctet:BN906_02358	K04032	eutT	ethanolamine utilization cobalamin adenosyltransferase	2.5.1.17	Metabolism--Metabolism of cofactors and vitamins--Porphyrin and chlorophyll metabolism|Metabolism--Global and overview maps--Metabolic pathways

[denovogenes]_110744	99.21	0.0	boa:Bovatus_04594	K02428	rdgB	XTP/dITP diphosphohydrolase	3.6.1.66	Metabolism--Nucleotide metabolism--Purine metabolism

[denovogenes]_110745	98.82	0.0	boa:Bovatus_02098	K00945	cmk	CMP/dCMP kinase	2.7.4.25	Metabolism--Nucleotide metabolism--Pyrimidine metabolism|Metabolism--Global and overview maps--Metabolic pathways

[denovogenes]_110748	44.27	7e-64	eac:EAL2_c18460	K18672	dacA	diadenylate cyclase	2.7.7.85	--

[denovogenes]_110749	51.79	1e-83	cbz:Cbs_4525	K01223	E3.2.1.86B, bglA	6-phospho-beta-glucosidase	3.2.1.86	Metabolism--Carbohydrate metabolism--Glycolysis / Gluconeogenesis|Metabolism--Carbohydrate metabolism--Starch and sucrose metabolism

[denovogenes]_11075	99.85	0.0	pdi:BDI_0002	K00525	E1.17.4.1A, nrdA, nrdE	ribonucleoside-diphosphate reductase alpha chain	1.17.4.1	Metabolism--Nucleotide metabolism--Purine metabolism|Metabolism--Nucleotide metabolism--Pyrimidine metabolism|Metabolism--Global and overview maps--Metabolic pathways

[denovogenes]_110750	64.03	3e-108	siz:SI82_03645	K02069	ABC.X2.P	putative ABC transport system permease protein	--	--

[denovogenes]_110751	100.00	0.0	pdi:BDI_1392	K00175	korB, oorB, oforB	2-oxoglutarate/2-oxoacid ferredoxin oxidoreductase subunit beta	1.2.7.3 1.2.7.11	Metabolism--Carbohydrate metabolism--Glycolysis / Gluconeogenesis|Metabolism--Carbohydrate metabolism--Citrate cycle (TCA cycle)|Metabolism--Carbohydrate metabolism--Pyruvate metabolism|Metabolism--Carbohydrate metabolism--Butanoate metabolism|Metabolism--Energy metabolism--Carbon fixation pathways in prokaryotes|Metabolism--Global and overview maps--Metabolic pathways|Metabolism--Global and overview maps--Microbial metabolism in diverse environments|Metabolism--Global and overview maps--Biosynthesis of antibiotics|Metabolism--Global and overview maps--Carbon metabolism

[denovogenes]_110752	100.00	0.0	shq:A0259_21155	K15587	nikD	nickel transport system ATP-binding protein	3.6.3.24	Environmental Information Processing--Membrane transport--ABC transporters

[denovogenes]_110753	100.00	0.0	pvl:AOB99_15310	K15835	murR	RpiR family transcriptional regulator, murPQ operon repressor	--	--

[denovogenes]_110754	100.00	0.0	pvl:AOB99_13545	K00560	thyA, TYMS	thymidylate synthase	2.1.1.45	Metabolism--Nucleotide metabolism--Pyrimidine metabolism|Metabolism--Metabolism of cofactors and vitamins--One carbon pool by folate|Metabolism--Global and overview maps--Metabolic pathways|Human Diseases--Drug resistance: Antineoplastic--Antifolate resistance

[denovogenes]_110756	100.00	0.0	pvl:AOB99_11260	K00228	CPOX, hemF	coproporphyrinogen III oxidase	1.3.3.3	Metabolism--Metabolism of cofactors and vitamins--Porphyrin and chlorophyll metabolism|Metabolism--Global and overview maps--Metabolic pathways|Metabolism--Global and overview maps--Biosynthesis of secondary metabolites

[denovogenes]_110758	98.43	0.0	pdi:BDI_1226	K09861	K09861	uncharacterized protein	--	--

[denovogenes]_110760	85.43	2e-157	vpr:Vpar_0791	K00891	E2.7.1.71, aroK, aroL	shikimate kinase	2.7.1.71	Metabolism--Amino acid metabolism--Phenylalanine, tyrosine and tryptophan biosynthesis|Metabolism--Global and overview maps--Metabolic pathways|Metabolism--Global and overview maps--Biosynthesis of secondary metabolites|Metabolism--Global and overview maps--Biosynthesis of antibiotics|Metabolism--Global and overview maps--Biosynthesis of amino acids

[denovogenes]_110761	100.00	0.0	pdi:BDI_1113	K02823	pyrDII	dihydroorotate dehydrogenase electron transfer subunit	--	Metabolism--Nucleotide metabolism--Pyrimidine metabolism|Metabolism--Global and overview maps--Metabolic pathways

[denovogenes]_110764	75.69	6e-139	ipo:Ilyop_2488	K17828	pyrDI	dihydroorotate dehydrogenase (NAD+) catalytic subunit	1.3.1.14	Metabolism--Nucleotide metabolism--Pyrimidine metabolism|Metabolism--Global and overview maps--Metabolic pathways

[denovogenes]_110766	56.47	8e-106	pcm:AY601_3559	K07497	K07497	putative transposase	--	--

[denovogenes]_110767	42.47	3e-53	ega:AL523_04695	K07001	K07001	NTE family protein	--	--

[denovogenes]_110768	100.00	0.0	bvu:BVU_2058	K06889	K06889	uncharacterized protein	--	--

[denovogenes]_110773	100.00	0.0	ecm:EcSMS35_1784	K12940	abgA	aminobenzoyl-glutamate utilization protein A	--	--

[denovogenes]_110775	40.42	6e-47	pre:PCA10_44700	K07678	barA, gacS, varS	two-component system, NarL family, sensor histidine kinase BarA	2.7.13.3	Environmental Information Processing--Signal transduction--Two-component system|Cellular Processes--Cellular community - prokaryotes--Biofilm formation - Pseudomonas aeruginosa|Cellular Processes--Cellular community - prokaryotes--Biofilm formation - Escherichia coli|Cellular Processes--Cellular community - prokaryotes--Biofilm formation - Vibrio cholerae

[denovogenes]_110778	84.71	4e-155	btho:Btheta7330_00261	K03387	ahpF	alkyl hydroperoxide reductase subunit F	1.6.4.-	--

[denovogenes]_110779	100.00	2e-175	ese:ECSF_4120	K02057	ABC.SS.P	simple sugar transport system permease protein	--	--

[denovogenes]_110783	100.00	2e-179	elo:EC042_2100	K02412	fliI	flagellum-specific ATP synthase	3.6.3.14	Cellular Processes--Cell motility--Flagellar assembly

[denovogenes]_110786	66.54	7e-119	bcee:V568_100716	K13926	rbbA	ribosome-dependent ATPase	--	--

[denovogenes]_110789	100.00	1e-171	mmk:MU9_1857	K07126	K07126	uncharacterized protein	--	--

[denovogenes]_11079	42.90	0.0	cdg:CDBI1_06430	K06978	K06978	uncharacterized protein	--	--

[denovogenes]_110790	99.60	4e-178	mmk:MU9_26	K03228	yscT, sctT, hrcT	type III secretion protein T	--	Environmental Information Processing--Membrane transport--Bacterial secretion system

[denovogenes]_110791	100.00	0.0	bfb:VU15_16805	K12343	SRD5A1	3-oxo-5-alpha-steroid 4-dehydrogenase 1	1.3.1.22	Metabolism--Lipid metabolism--Steroid hormone biosynthesis

[denovogenes]_110792	100.00	0.0	mmk:MU9_2990	K02020	modA	molybdate transport system substrate-binding protein	--	Environmental Information Processing--Membrane transport--ABC transporters

[denovogenes]_110795	64.00	1e-113	efu:HMPREF0351_11050	K02530	lacR	DeoR family transcriptional regulator, lactose phosphotransferase system repressor	--	--

[denovogenes]_110796	98.42	0.0	bfr:BF0555	K03088	SIG3.2, rpoE	RNA polymerase sigma-70 factor, ECF subfamily	--	--

[denovogenes]_110797	100.00	0.0	bfb:VU15_11435	K01142	E3.1.11.2, xthA	exodeoxyribonuclease III	3.1.11.2	Genetic Information Processing--Replication and repair--Base excision repair

[denovogenes]_110798	100.00	0.0	bfb:VU15_06950	K00175	korB, oorB, oforB	2-oxoglutarate/2-oxoacid ferredoxin oxidoreductase subunit beta	1.2.7.3 1.2.7.11	Metabolism--Carbohydrate metabolism--Glycolysis / Gluconeogenesis|Metabolism--Carbohydrate metabolism--Citrate cycle (TCA cycle)|Metabolism--Carbohydrate metabolism--Pyruvate metabolism|Metabolism--Carbohydrate metabolism--Butanoate metabolism|Metabolism--Energy metabolism--Carbon fixation pathways in prokaryotes|Metabolism--Global and overview maps--Metabolic pathways|Metabolism--Global and overview maps--Microbial metabolism in diverse environments|Metabolism--Global and overview maps--Biosynthesis of antibiotics|Metabolism--Global and overview maps--Carbon metabolism

[denovogenes]_110799	73.31	5e-127	ega:AL523_09425	K04074	divIVA	cell division initiation protein	--	--

[denovogenes]_11080	42.12	6e-53	pzu:PHZ_c2681	K07716	pleC	two-component system, cell cycle sensor histidine kinase PleC	2.7.13.3	Environmental Information Processing--Signal transduction--Two-component system|Cellular Processes--Cell growth and death--Cell cycle - Caulobacter

[denovogenes]_110801	100.00	0.0	mmk:MU9_2395	K06177	rluA	tRNA pseudouridine32 synthase / 23S rRNA pseudouridine746 synthase	5.4.99.28 5.4.99.29	--

[denovogenes]_110802	100.00	0.0	bfb:VU15_11860	K01126	E3.1.4.46, glpQ, ugpQ	glycerophosphoryl diester phosphodiesterase	3.1.4.46	Metabolism--Lipid metabolism--Glycerophospholipid metabolism

[denovogenes]_110804	44.92	5e-70	deh:cbdbA122	K03605	hyaD, hybD	hydrogenase maturation protease	3.4.23.-	--

[denovogenes]_110805	100.00	0.0	bfb:VU15_12390	K02036	pstB	phosphate transport system ATP-binding protein	3.6.3.27	Environmental Information Processing--Membrane transport--ABC transporters

[denovogenes]_110807	50.65	2e-74	nth:Nther_1461	K01358	clpP, CLPP	ATP-dependent Clp protease, protease subunit	3.4.21.92	Cellular Processes--Cell growth and death--Cell cycle - Caulobacter|Organismal Systems--Aging--Longevity regulating pathway - worm

[denovogenes]_110809	40.00	1e-51	cac:CA_P0051	K07124	K07124	uncharacterized protein	--	--

[denovogenes]_11081	61.84	0.0	cpas:Clopa_0561	K02334	dpo	DNA polymerase bacteriophage-type	2.7.7.7	--

[denovogenes]_110810	99.60	0.0	bfb:VU15_09895	K03437	spoU	RNA methyltransferase, TrmH family	--	--

[denovogenes]_110811	81.07	4e-144	csr:Cspa_c42660	K01834	PGAM, gpmA	2,3-bisphosphoglycerate-dependent phosphoglycerate mutase	5.4.2.11	Metabolism--Carbohydrate metabolism--Glycolysis / Gluconeogenesis|Metabolism--Amino acid metabolism--Glycine, serine and threonine metabolism|Metabolism--Energy metabolism--Methane metabolism|Metabolism--Global and overview maps--Metabolic pathways|Metabolism--Global and overview maps--Biosynthesis of secondary metabolites|Metabolism--Global and overview maps--Microbial metabolism in diverse environments|Metabolism--Global and overview maps--Biosynthesis of antibiotics|Metabolism--Global and overview maps--Carbon metabolism|Metabolism--Global and overview maps--Biosynthesis of amino acids|Organismal Systems--Endocrine system--Glucagon signaling pathway|Human Diseases--Cancers: Overview--Central carbon metabolism in cancer

[denovogenes]_110813	100.00	0.0	bfb:VU15_07705	K01470	E3.5.2.10	creatinine amidohydrolase	3.5.2.10	Metabolism--Amino acid metabolism--Arginine and proline metabolism

[denovogenes]_110814	98.81	0.0	mmk:MU9_244	K02852	wecG, rffM	UDP-N-acetyl-D-mannosaminouronate:lipid I N-acetyl-D-mannosaminouronosyltransferase	2.4.1.180	--

[denovogenes]_110816	50.00	5e-73	erb:A4V01_07730	K18349	vanRC, vanRE, vanRG	two-component system, OmpR family, response regulator VanR	--	Human Diseases--Drug resistance: Antimicrobial--Vancomycin resistance|Environmental Information Processing--Signal transduction--Two-component system

[denovogenes]_110819	57.32	2e-60	etr:ETAE_1677	K19303	mepH	murein DD-endopeptidase	3.4.-.-	--

[denovogenes]_11082	46.33	0.0	dvl:Dvul_0060	K01533	copB	Cu2+-exporting ATPase	3.6.3.4	--

[denovogenes]_110823	41.26	4e-21	meq:MSWHS_0180	K21029	moeB	molybdopterin-synthase adenylyltransferase	2.7.7.80	Genetic Information Processing--Folding, sorting and degradation--Sulfur relay system

[denovogenes]_110824	64.29	1e-121	csb:CLSA_c41550	K03481	glvR	RpiR family transcriptional regulator, glv operon transcriptional regulator	--	--

[denovogenes]_110825	56.18	1e-93	csb:CLSA_c41540	K03481	glvR	RpiR family transcriptional regulator, glv operon transcriptional regulator	--	--

[denovogenes]_110828	95.60	1e-178	lacy:A4V08_02335	K02967	RP-S2, MRPS2, rpsB	small subunit ribosomal protein S2	--	Genetic Information Processing--Translation--Ribosome

[denovogenes]_110829	100.00	0.0	ene:ENT_00210	K03496	parA, soj	chromosome partitioning protein	--	--

[denovogenes]_11083	100.00	0.0	bvu:BVU_3322	K00700	GBE1, glgB	1,4-alpha-glucan branching enzyme	2.4.1.18	Metabolism--Carbohydrate metabolism--Starch and sucrose metabolism|Metabolism--Global and overview maps--Metabolic pathways|Metabolism--Global and overview maps--Biosynthesis of secondary metabolites

[denovogenes]_110830	65.35	2e-108	pdf:CD630DERM_24350	K03584	recO	DNA repair protein RecO (recombination protein O)	--	Genetic Information Processing--Replication and repair--Homologous recombination

[denovogenes]_110831	60.08	1e-106	cbt:CLH_2711	K07238	TC.ZIP, zupT, ZRT3, ZIP2	zinc transporter, ZIP family	--	--

[denovogenes]_110832	51.60	7e-83	cpas:Clopa_2085	K03481	glvR	RpiR family transcriptional regulator, glv operon transcriptional regulator	--	--

[denovogenes]_110835	94.00	3e-176	cbv:U729_3023	K00773	tgt, QTRT1	queuine tRNA-ribosyltransferase	2.4.2.29	--

[denovogenes]_110836	89.72	5e-172	lacy:A4V08_18660	K02036	pstB	phosphate transport system ATP-binding protein	3.6.3.27	Environmental Information Processing--Membrane transport--ABC transporters

[denovogenes]_110838	100.00	0.0	mmk:MU9_2851	K00059	fabG	3-oxoacyl-[acyl-carrier protein] reductase	1.1.1.100	Metabolism--Lipid metabolism--Fatty acid biosynthesis|Metabolism--Metabolism of cofactors and vitamins--Biotin metabolism|Metabolism--Lipid metabolism--Biosynthesis of unsaturated fatty acids|Metabolism--Global and overview maps--Metabolic pathways|Metabolism--Global and overview maps--Fatty acid metabolism

[denovogenes]_110840	40.50	2e-54	cbv:U729_752	K08600	srtB	sortase B	3.4.22.70	--

[denovogenes]_110841	47.42	1e-62	sdc:SDSE_0700	K01838	pgmB	beta-phosphoglucomutase	5.4.2.6	Metabolism--Carbohydrate metabolism--Starch and sucrose metabolism

[denovogenes]_110842	59.76	2e-89	cby:CLM_2188	K16785	ecfT	energy-coupling factor transport system permease protein	--	Environmental Information Processing--Membrane transport--ABC transporters

[denovogenes]_110843	55.78	1e-95	cpae:CPAST_c10890	K06608	iolR	DeoR family transcriptional regulator, myo-inositol catabolism operon repressor	--	--

[denovogenes]_110844	78.31	1e-142	lacy:A4V08_34865	K07124	K07124	uncharacterized protein	--	--

[denovogenes]_110845	76.02	5e-144	lacy:A4V08_22265	K06173	truA, PUS1	tRNA pseudouridine38-40 synthase	5.4.99.12	--

[denovogenes]_110846	100.00	0.0	pvl:AOB99_15990	K03478	chbG	chitin disaccharide deacetylase	3.5.1.105	--

[denovogenes]_110849	45.67	3e-73	mtt:Ftrac_0723	K09861	K09861	uncharacterized protein	--	--

[denovogenes]_110850	40.16	2e-51	eba:ebA5287	K04114	bcrA, badF	benzoyl-CoA reductase subunit A	1.3.7.8	Metabolism--Xenobiotics biodegradation and metabolism--Benzoate degradation|Metabolism--Global and overview maps--Metabolic pathways|Metabolism--Global and overview maps--Microbial metabolism in diverse environments|Metabolism--Global and overview maps--Degradation of aromatic compounds

[denovogenes]_110854	86.96	2e-157	cif:AL515_13610	K02013	ABC.FEV.A	iron complex transport system ATP-binding protein	3.6.3.34	Environmental Information Processing--Membrane transport--ABC transporters

[denovogenes]_110855	58.86	5e-60	spf:SpyM51316	K07741	antB	anti-repressor protein	--	--

[denovogenes]_110856	99.60	5e-179	cbut:ATN24_02865	K02530	lacR	DeoR family transcriptional regulator, lactose phosphotransferase system repressor	--	--

[denovogenes]_110859	100.00	0.0	sdy:SDY_4097	K03755	adiY	AraC family transcriptional regulator, transcriptional activator of adiA	--	--

[denovogenes]_11086	99.39	0.0	bth:BT_2512	K01534	zntA	Cd2+/Zn2+-exporting ATPase	3.6.3.3 3.6.3.5	--

[denovogenes]_110860	46.51	5e-83	ere:EUBREC_1113	K20074	prpC, phpP	PPM family protein phosphatase	3.1.3.16	--

[denovogenes]_110861	100.00	0.0	ecl:EcolC_3086	K11920	envY	AraC family transcriptional regulator	--	--

[denovogenes]_110865	59.06	6e-108	bfi:CIY_07320	K02026	ABC.MS.P1	multiple sugar transport system permease protein	--	--

[denovogenes]_110866	100.00	0.0	ecy:ECSE_0220	K11892	impK, ompA, vasF, dotU	type VI secretion system protein ImpK	--	Environmental Information Processing--Membrane transport--Bacterial secretion system

[denovogenes]_110868	100.00	0.0	pvl:AOB99_06000	K00979	kdsB	3-deoxy-manno-octulosonate cytidylyltransferase (CMP-KDO synthetase)	2.7.7.38	Metabolism--Glycan biosynthesis and metabolism--Lipopolysaccharide biosynthesis|Metabolism--Global and overview maps--Metabolic pathways

[denovogenes]_110869	61.67	1e-105	sanc:SANR_2031	K02315	dnaC	DNA replication protein DnaC	--	--

[denovogenes]_110870	80.71	3e-128	cbut:ATN24_17750	K06147	ABCB-BAC	ATP-binding cassette, subfamily B, bacterial	--	--

[denovogenes]_110871	51.18	2e-81	csq:CSCA_5077	K16651	pduX	L-threonine kinase	2.7.1.177	Metabolism--Metabolism of cofactors and vitamins--Porphyrin and chlorophyll metabolism

[denovogenes]_110874	69.57	7e-135	cbv:U729_2798	K00806	uppS	undecaprenyl diphosphate synthase	2.5.1.31	Metabolism--Metabolism of terpenoids and polyketides--Terpenoid backbone biosynthesis|Metabolism--Global and overview maps--Biosynthesis of secondary metabolites

[denovogenes]_110875	64.88	7e-103	vpr:Vpar_0703	K03685	rnc, DROSHA, RNT1	ribonuclease III	3.1.26.3	Genetic Information Processing--Translation--Ribosome biogenesis in eukaryotes|Human Diseases--Cancers: Overview--Proteoglycans in cancer

[denovogenes]_110876	48.06	1e-78	gym:GYMC10_6046	K01183	E3.2.1.14	chitinase	3.2.1.14	Metabolism--Carbohydrate metabolism--Amino sugar and nucleotide sugar metabolism|Metabolism--Global and overview maps--Metabolic pathways

[denovogenes]_110877	74.21	3e-129	cbk:CLL_A0896	K09761	rsmE	16S rRNA (uracil1498-N3)-methyltransferase	2.1.1.193	--

[denovogenes]_110878	88.58	1e-165	prg:RB151_037590	K03592	pmbA	PmbA protein	--	--

[denovogenes]_110880	100.00	0.0	pvl:AOB99_12390	K16868	tehB	tellurite methyltransferase	2.1.1.265	--

[denovogenes]_110882	100.00	0.0	cbut:ATN24_07130	K00566	mnmA, trmU	tRNA-uridine 2-sulfurtransferase	2.8.1.13	Genetic Information Processing--Folding, sorting and degradation--Sulfur relay system

[denovogenes]_110883	97.63	0.0	sent:TY21A_00940	K00606	panB	3-methyl-2-oxobutanoate hydroxymethyltransferase	2.1.2.11	Metabolism--Metabolism of cofactors and vitamins--Pantothenate and CoA biosynthesis|Metabolism--Global and overview maps--Metabolic pathways|Metabolism--Global and overview maps--Biosynthesis of secondary metabolites

[denovogenes]_110884	98.81	0.0	cbut:ATN24_08255	K10823	oppF	oligopeptide transport system ATP-binding protein	--	Human Diseases--Drug resistance: Antimicrobial--beta-Lactam resistance|Environmental Information Processing--Membrane transport--ABC transporters|Cellular Processes--Cellular community - prokaryotes--Quorum sensing

[denovogenes]_110885	99.21	0.0	cif:AL515_06460	K01666	mhpE	4-hydroxy 2-oxovalerate aldolase	4.1.3.39	Metabolism--Amino acid metabolism--Phenylalanine metabolism|Metabolism--Xenobiotics biodegradation and metabolism--Benzoate degradation|Metabolism--Xenobiotics biodegradation and metabolism--Dioxin degradation|Metabolism--Xenobiotics biodegradation and metabolism--Xylene degradation|Metabolism--Global and overview maps--Metabolic pathways|Metabolism--Global and overview maps--Microbial metabolism in diverse environments|Metabolism--Global and overview maps--Degradation of aromatic compounds

[denovogenes]_110886	46.46	4e-75	mtt:Ftrac_0723	K09861	K09861	uncharacterized protein	--	--

[denovogenes]_110887	95.92	7e-170	prg:RB151_002480	K03762	proP	MFS transporter, MHS family, proline/betaine transporter	--	--

[denovogenes]_110888	99.15	4e-167	cbut:ATN24_04515	K02071	metN	D-methionine transport system ATP-binding protein	--	Environmental Information Processing--Membrane transport--ABC transporters

[denovogenes]_110889	44.87	9e-36	cle:Clole_2119	K06298	gerM	germination protein M	--	--

[denovogenes]_110890	79.92	3e-132	clt:CM240_0413	K11105	cvrA, nhaP2	cell volume regulation protein A	--	--

[denovogenes]_110892	100.00	0.0	ecos:EC958_0547	K03074	secF	preprotein translocase subunit SecF	--	Genetic Information Processing--Folding, sorting and degradation--Protein export|Environmental Information Processing--Membrane transport--Bacterial secretion system

[denovogenes]_110893	42.69	2e-63	caby:Cabys_111	K01966	PCCB, pccB	propionyl-CoA carboxylase beta chain	6.4.1.3	Metabolism--Amino acid metabolism--Valine, leucine and isoleucine degradation|Metabolism--Carbohydrate metabolism--Glyoxylate and dicarboxylate metabolism|Metabolism--Carbohydrate metabolism--Propanoate metabolism|Metabolism--Global and overview maps--Metabolic pathways|Metabolism--Global and overview maps--Microbial metabolism in diverse environments|Metabolism--Global and overview maps--Biosynthesis of antibiotics|Metabolism--Global and overview maps--Carbon metabolism

[denovogenes]_110894	45.28	4e-60	clt:CM240_2329	K01537	E3.6.3.8	Ca2+-transporting ATPase	3.6.3.8	--

[denovogenes]_110895	97.23	0.0	cfd:CFNIH1_13385	K01250	rihA	pyrimidine-specific ribonucleoside hydrolase	3.2.-.-	--

[denovogenes]_11090	92.12	0.0	pdi:BDI_0282	K03702	uvrB	excinuclease ABC subunit B	--	Genetic Information Processing--Replication and repair--Nucleotide excision repair

[denovogenes]_110902	62.75	3e-122	ssj:SSON53_21685	K01811	xylS, yicI	alpha-D-xyloside xylohydrolase	3.2.1.177	--

[denovogenes]_110903	51.02	4e-77	tit:Thit_0579	K01436	K01436	amidohydrolase	3.5.1.-	--

[denovogenes]_110904	99.61	0.0	elf:LF82_0723	K11754	folC	dihydrofolate synthase / folylpolyglutamate synthase	6.3.2.12 6.3.2.17	Metabolism--Metabolism of cofactors and vitamins--Folate biosynthesis|Metabolism--Global and overview maps--Metabolic pathways

[denovogenes]_110905	88.14	4e-157	fhw:RN87_06150	K03307	TC.SSS	solute:Na+ symporter, SSS family	--	--

[denovogenes]_110906	76.10	1e-142	cbk:CLL_A3292	K11358	yhdR	aspartate aminotransferase	2.6.1.1	Metabolism--Amino acid metabolism--Arginine biosynthesis|Metabolism--Amino acid metabolism--Alanine, aspartate and glutamate metabolism|Metabolism--Amino acid metabolism--Cysteine and methionine metabolism|Metabolism--Amino acid metabolism--Arginine and proline metabolism|Metabolism--Amino acid metabolism--Tyrosine metabolism|Metabolism--Amino acid metabolism--Phenylalanine metabolism|Metabolism--Amino acid metabolism--Phenylalanine, tyrosine and tryptophan biosynthesis|Metabolism--Biosynthesis of other secondary metabolites--Novobiocin biosynthesis|Metabolism--Biosynthesis of other secondary metabolites--Isoquinoline alkaloid biosynthesis|Metabolism--Biosynthesis of other secondary metabolites--Tropane, piperidine and pyridine alkaloid biosynthesis|Metabolism--Global and overview maps--Metabolic pathways|Metabolism--Global and overview maps--Biosynthesis of secondary metabolites|Metabolism--Global and overview maps--Biosynthesis of antibiotics|Metabolism--Global and overview maps--2-Oxocarboxylic acid metabolism|Metabolism--Global and overview maps--Biosynthesis of amino acids

[denovogenes]_110907	100.00	0.0	plz:S4054249_10315	K19272	aph3-I	aminoglycoside 3'-phosphotransferase I	2.7.1.95	--

[denovogenes]_11091	48.95	1e-100	csh:Closa_1606	K03406	mcp	methyl-accepting chemotaxis protein	--	Environmental Information Processing--Signal transduction--Two-component system|Cellular Processes--Cell motility--Bacterial chemotaxis

[denovogenes]_110910	68.38	2e-124	cle:Clole_1164	K02015	ABC.FEV.P	iron complex transport system permease protein	--	Environmental Information Processing--Membrane transport--ABC transporters

[denovogenes]_110911	99.21	0.0	cbut:ATN24_20230	K06147	ABCB-BAC	ATP-binding cassette, subfamily B, bacterial	--	--

[denovogenes]_110912	40.86	9e-37	hau:Haur_0374	K01912	paaK	phenylacetate-CoA ligase	6.2.1.30	Metabolism--Amino acid metabolism--Phenylalanine metabolism|Metabolism--Global and overview maps--Microbial metabolism in diverse environments|Cellular Processes--Cellular community - prokaryotes--Biofilm formation - Vibrio cholerae

[denovogenes]_110913	58.47	1e-95	llw:kw2_0786	K00757	udp, UPP	uridine phosphorylase	2.4.2.3	Metabolism--Nucleotide metabolism--Pyrimidine metabolism|Metabolism--Xenobiotics biodegradation and metabolism--Drug metabolism - other enzymes|Metabolism--Global and overview maps--Metabolic pathways

[denovogenes]_110914	99.60	0.0	cfd:CFNIH1_15700	K11069	potD	spermidine/putrescine transport system substrate-binding protein	--	Environmental Information Processing--Membrane transport--ABC transporters

[denovogenes]_110915	48.40	4e-68	cbt:CLH_0852	K06385	spoIIP	stage II sporulation protein P	--	--

[denovogenes]_110919	100.00	2e-179	pmib:BB2000_0345	K13629	dsdX	D-serine transporter	--	--

[denovogenes]_11092	85.93	0.0	pdi:BDI_1668	K00615	E2.2.1.1, tktA, tktB	transketolase	2.2.1.1	Metabolism--Carbohydrate metabolism--Pentose phosphate pathway|Metabolism--Energy metabolism--Carbon fixation in photosynthetic organisms|Metabolism--Metabolism of terpenoids and polyketides--Biosynthesis of ansamycins|Metabolism--Global and overview maps--Metabolic pathways|Metabolism--Global and overview maps--Biosynthesis of secondary metabolites|Metabolism--Global and overview maps--Microbial metabolism in diverse environments|Metabolism--Global and overview maps--Biosynthesis of antibiotics|Metabolism--Global and overview maps--Carbon metabolism|Metabolism--Global and overview maps--Biosynthesis of amino acids

[denovogenes]_110922	93.31	3e-174	cfd:CFNIH1_21455	K16711	wcaM	colanic acid biosynthesis protein WcaM	--	--

[denovogenes]_110923	56.49	3e-93	bcf:bcf_25570	K12573	rnr, vacB	ribonuclease R	3.1.-.-	Genetic Information Processing--Folding, sorting and degradation--RNA degradation

[denovogenes]_110925	85.43	1e-153	cbm:CBF_3708	K03798	ftsH, hflB	cell division protease FtsH	3.4.24.-	--

[denovogenes]_110926	92.52	2e-174	cfd:CFNIH1_07460	K00075	murB	UDP-N-acetylmuramate dehydrogenase	1.3.1.98	Metabolism--Carbohydrate metabolism--Amino sugar and nucleotide sugar metabolism|Metabolism--Glycan biosynthesis and metabolism--Peptidoglycan biosynthesis|Metabolism--Global and overview maps--Metabolic pathways

[denovogenes]_110927	90.16	1e-172	yru:BD65_213	K06415	spoVR	stage V sporulation protein R	--	--

[denovogenes]_11093	99.85	0.0	paeg:AI22_09265	K19713	tsdA	thiosulfate dehydrogenase	1.8.2.2	--

[denovogenes]_110933	70.47	2e-116	cace:CACET_c03930	K00335	nuoF	NADH-quinone oxidoreductase subunit F	1.6.5.3	Metabolism--Energy metabolism--Oxidative phosphorylation|Metabolism--Global and overview maps--Metabolic pathways

[denovogenes]_110935	98.43	0.0	cfd:CFNIH1_16125	K01424	E3.5.1.1, ansA, ansB	L-asparaginase	3.5.1.1	Metabolism--Amino acid metabolism--Alanine, aspartate and glutamate metabolism|Metabolism--Metabolism of other amino acids--Cyanoamino acid metabolism|Metabolism--Global and overview maps--Metabolic pathways|Metabolism--Global and overview maps--Biosynthesis of secondary metabolites

[denovogenes]_110937	62.45	6e-117	bbe:BBR47_08260	K12941	abgB	aminobenzoyl-glutamate utilization protein B	--	--

[denovogenes]_110938	55.60	8e-93	lmob:BN419_1087	K06959	tex	protein Tex	--	--

[denovogenes]_110939	53.72	9e-77	mhg:MHY_27760	K12573	rnr, vacB	ribonuclease R	3.1.-.-	Genetic Information Processing--Folding, sorting and degradation--RNA degradation

[denovogenes]_11094	97.64	0.0	bfb:VU15_03240	K03798	ftsH, hflB	cell division protease FtsH	3.4.24.-	--

[denovogenes]_110940	65.99	1e-124	ctc:CTC_01531	K03783	punA, PNP	purine-nucleoside phosphorylase	2.4.2.1	Metabolism--Nucleotide metabolism--Purine metabolism|Metabolism--Nucleotide metabolism--Pyrimidine metabolism|Metabolism--Metabolism of cofactors and vitamins--Nicotinate and nicotinamide metabolism|Metabolism--Global and overview maps--Metabolic pathways|Metabolism--Global and overview maps--Biosynthesis of secondary metabolites

[denovogenes]_110944	66.12	1e-113	sri:SELR_15800	K02028	ABC.PA.A	polar amino acid transport system ATP-binding protein	3.6.3.21	--

[denovogenes]_110945	98.81	0.0	bhl:Bache_2873	K00175	korB, oorB, oforB	2-oxoglutarate/2-oxoacid ferredoxin oxidoreductase subunit beta	1.2.7.3 1.2.7.11	Metabolism--Carbohydrate metabolism--Glycolysis / Gluconeogenesis|Metabolism--Carbohydrate metabolism--Citrate cycle (TCA cycle)|Metabolism--Carbohydrate metabolism--Pyruvate metabolism|Metabolism--Carbohydrate metabolism--Butanoate metabolism|Metabolism--Energy metabolism--Carbon fixation pathways in prokaryotes|Metabolism--Global and overview maps--Metabolic pathways|Metabolism--Global and overview maps--Microbial metabolism in diverse environments|Metabolism--Global and overview maps--Biosynthesis of antibiotics|Metabolism--Global and overview maps--Carbon metabolism

[denovogenes]_110951	49.14	5e-71	rto:RTO_06150	K07260	vanY	D-alanyl-D-alanine carboxypeptidase	3.4.16.4	Metabolism--Glycan biosynthesis and metabolism--Peptidoglycan biosynthesis|Human Diseases--Drug resistance: Antimicrobial--Vancomycin resistance|Environmental Information Processing--Signal transduction--Two-component system

[denovogenes]_110952	71.31	4e-127	elm:ELI_3913	K02007	cbiM	cobalt/nickel transport system permease protein	--	Environmental Information Processing--Membrane transport--ABC transporters

[denovogenes]_110953	81.03	2e-148	cct:CC1_29430	K03785	aroD	3-dehydroquinate dehydratase I	4.2.1.10	Metabolism--Amino acid metabolism--Phenylalanine, tyrosine and tryptophan biosynthesis|Metabolism--Global and overview maps--Metabolic pathways|Metabolism--Global and overview maps--Biosynthesis of secondary metabolites|Metabolism--Global and overview maps--Biosynthesis of antibiotics|Metabolism--Global and overview maps--Biosynthesis of amino acids

[denovogenes]_110954	65.86	4e-118	bprs:CK3_22270	K15600	thiZ	putative hydroxymethylpyrimidine transport system ATP-binding protein	--	Environmental Information Processing--Membrane transport--ABC transporters

[denovogenes]_110956	41.73	3e-57	aad:TC41_0457	K05921	hpaG	5-oxopent-3-ene-1,2,5-tricarboxylate decarboxylase / 2-hydroxyhepta-2,4-diene-1,7-dioate isomerase	4.1.1.68 5.3.3.-	Metabolism--Amino acid metabolism--Tyrosine metabolism|Metabolism--Global and overview maps--Microbial metabolism in diverse environments|Metabolism--Global and overview maps--Degradation of aromatic compounds

[denovogenes]_110958	82.07	8e-153	ibu:IB211_03368	K03496	parA, soj	chromosome partitioning protein	--	--

[denovogenes]_110959	66.93	3e-128	rto:RTO_14990	K03424	--	--	--	--

[denovogenes]_11096	41.70	4e-95	dfi:AXF13_12055	K02688	prpR	transcriptional regulator, propionate catabolism operon regulatory protein	--	--

[denovogenes]_110961	86.51	6e-156	rim:ROI_10130	K02500	hisF	cyclase	4.1.3.-	Metabolism--Amino acid metabolism--Histidine metabolism|Metabolism--Global and overview maps--Metabolic pathways|Metabolism--Global and overview maps--Biosynthesis of secondary metabolites|Metabolism--Global and overview maps--Biosynthesis of amino acids

[denovogenes]_110962	88.93	3e-165	bcel:BcellWH2_05466	K03437	spoU	RNA methyltransferase, TrmH family	--	--

[denovogenes]_110963	63.41	9e-108	rho:RHOM_08875	K06182	rluF	23S rRNA pseudouridine2604 synthase	5.4.99.21	--

[denovogenes]_110965	40.13	4e-16	mah:MEALZ_3479	K01501	E3.5.5.1	nitrilase	3.5.5.1	Metabolism--Amino acid metabolism--Tryptophan metabolism|Metabolism--Metabolism of other amino acids--Cyanoamino acid metabolism|Metabolism--Xenobiotics biodegradation and metabolism--Aminobenzoate degradation|Metabolism--Xenobiotics biodegradation and metabolism--Styrene degradation|Metabolism--Energy metabolism--Nitrogen metabolism|Metabolism--Global and overview maps--Microbial metabolism in diverse environments

[denovogenes]_110967	63.03	4e-105	abao:BEQ56_07000	K06201	cutC	copper homeostasis protein	--	--

[denovogenes]_110968	90.04	1e-166	boa:Bovatus_01971	K05306	phnX	phosphonoacetaldehyde hydrolase	3.11.1.1	Metabolism--Metabolism of other amino acids--Phosphonate and phosphinate metabolism|Metabolism--Global and overview maps--Metabolic pathways|Metabolism--Global and overview maps--Microbial metabolism in diverse environments

[denovogenes]_110969	57.31	2e-103	lacy:A4V08_11775	K02050	ABC.SN.P	NitT/TauT family transport system permease protein	--	--

[denovogenes]_11097	55.12	0.0	byl:A4V09_07890	K03407	cheA	two-component system, chemotaxis family, sensor kinase CheA	2.7.13.3	Environmental Information Processing--Signal transduction--Two-component system|Cellular Processes--Cell motility--Bacterial chemotaxis

[denovogenes]_110970	48.62	5e-76	bdo:EL88_02575	K03496	parA, soj	chromosome partitioning protein	--	--

[denovogenes]_110971	100.00	0.0	bvu:BVU_1634	K02967	RP-S2, MRPS2, rpsB	small subunit ribosomal protein S2	--	Genetic Information Processing--Translation--Ribosome

[denovogenes]_110972	92.43	6e-172	boa:Bovatus_02155	K02013	ABC.FEV.A	iron complex transport system ATP-binding protein	3.6.3.34	Environmental Information Processing--Membrane transport--ABC transporters

[denovogenes]_110974	40.26	5e-51	eyy:EGYY_06610	K18349	vanRC, vanRE, vanRG	two-component system, OmpR family, response regulator VanR	--	Human Diseases--Drug resistance: Antimicrobial--Vancomycin resistance|Environmental Information Processing--Signal transduction--Two-component system

[denovogenes]_110975	69.04	7e-120	csr:Cspa_c08670	K17076	K17076, lysY	putative lysine transport system ATP-binding protein	3.6.3.-	Environmental Information Processing--Membrane transport--ABC transporters

[denovogenes]_110976	99.60	0.0	boa:Bovatus_02113	K12343	SRD5A1	3-oxo-5-alpha-steroid 4-dehydrogenase 1	1.3.1.22	Metabolism--Lipid metabolism--Steroid hormone biosynthesis

[denovogenes]_110980	40.51	3e-37	nmx:NMA510612_1295	K00571	E2.1.1.72	site-specific DNA-methyltransferase (adenine-specific)	2.1.1.72	--

[denovogenes]_110981	52.00	9e-83	tmt:Tmath_1330	K00059	fabG	3-oxoacyl-[acyl-carrier protein] reductase	1.1.1.100	Metabolism--Lipid metabolism--Fatty acid biosynthesis|Metabolism--Metabolism of cofactors and vitamins--Biotin metabolism|Metabolism--Lipid metabolism--Biosynthesis of unsaturated fatty acids|Metabolism--Global and overview maps--Metabolic pathways|Metabolism--Global and overview maps--Fatty acid metabolism

[denovogenes]_110984	49.17	2e-88	asf:SFBM_0780	K04477	ycdX	putative hydrolase	--	--

[denovogenes]_110987	65.02	5e-114	cls:CXIVA_11480	K15460	yfiC, trmX	tRNA1Val (adenine37-N6)-methyltransferase	2.1.1.223	--

[denovogenes]_11099	99.70	0.0	ecg:E2348C_3949	K03230	yscV, sctV, hrcV	type III secretion protein V	--	Environmental Information Processing--Membrane transport--Bacterial secretion system

[denovogenes]_110990	40.24	3e-58	sjp:SJA_C1-05590	K15237	linC	2,5-dichloro-2,5-cyclohexadiene-1,4-diol dehydrogenase 1	1.3.1.-	Metabolism--Xenobiotics biodegradation and metabolism--Chlorocyclohexane and chlorobenzene degradation|Metabolism--Global and overview maps--Metabolic pathways|Metabolism--Global and overview maps--Microbial metabolism in diverse environments

[denovogenes]_110991	73.49	7e-137	ruk:A4V00_05365	K02003	ABC.CD.A	putative ABC transport system ATP-binding protein	--	--

[denovogenes]_110992	86.17	7e-164	fpla:A4U99_14700	K02036	pstB	phosphate transport system ATP-binding protein	3.6.3.27	Environmental Information Processing--Membrane transport--ABC transporters

[denovogenes]_110994	76.38	5e-146	bdh:GV66_19615	K02477	K02477	two-component system, LytT family, response regulator	--	--

[denovogenes]_110998	70.90	2e-124	gfe:Gferi_21755	K01990	ABC-2.A	ABC-2 type transport system ATP-binding protein	--	--

[denovogenes]_110999	98.42	1e-175	fpla:A4U99_00010	K03525	coaX	type III pantothenate kinase	2.7.1.33	Metabolism--Metabolism of cofactors and vitamins--Pantothenate and CoA biosynthesis|Metabolism--Global and overview maps--Metabolic pathways

[denovogenes]_1110	68.15	0.0	bfr:BF0736	K01190	lacZ	beta-galactosidase	3.2.1.23	Metabolism--Carbohydrate metabolism--Galactose metabolism|Metabolism--Glycan biosynthesis and metabolism--Other glycan degradation|Metabolism--Lipid metabolism--Sphingolipid metabolism|Metabolism--Global and overview maps--Metabolic pathways

[denovogenes]_111001	51.95	1e-86	cpy:Cphy_0115	K09696	natB	sodium transport system permease protein	--	Environmental Information Processing--Membrane transport--ABC transporters|Environmental Information Processing--Signal transduction--Two-component system

[denovogenes]_111002	70.16	2e-123	lacy:A4V08_34850	K06183	rsuA	16S rRNA pseudouridine516 synthase	5.4.99.19	--

[denovogenes]_111003	50.00	2e-78	bpip:BPP43_04570	K04477	ycdX	putative hydrolase	--	--

[denovogenes]_111004	100.00	0.0	ecol:LY180_12420	K06203	cysZ	CysZ protein	--	--

[denovogenes]_111007	63.71	3e-112	fpla:A4U99_14250	K04042	glmU	bifunctional UDP-N-acetylglucosamine pyrophosphorylase / Glucosamine-1-phosphate N-acetyltransferase	2.7.7.23 2.3.1.157	Metabolism--Carbohydrate metabolism--Amino sugar and nucleotide sugar metabolism|Metabolism--Global and overview maps--Metabolic pathways|Metabolism--Global and overview maps--Biosynthesis of antibiotics

[denovogenes]_111008	100.00	0.0	sbo:SBO_2734	K00065	kduD	2-deoxy-D-gluconate 3-dehydrogenase	1.1.1.125	Metabolism--Carbohydrate metabolism--Pentose and glucuronate interconversions

[denovogenes]_11101	100.00	0.0	paeo:M801_1981	K00527	nrdD	ribonucleoside-triphosphate reductase	1.17.4.2	Metabolism--Nucleotide metabolism--Purine metabolism|Metabolism--Nucleotide metabolism--Pyrimidine metabolism|Metabolism--Global and overview maps--Metabolic pathways

[denovogenes]_111012	96.05	2e-176	bll:BLJ_1243	K03205	virD4, lvhD4	type IV secretion system protein VirD4	--	Environmental Information Processing--Membrane transport--Bacterial secretion system

[denovogenes]_111015	98.02	1e-180	bvu:BVU_3905	K03596	lepA	GTP-binding protein LepA	--	Human Diseases--Infectious diseases: Bacterial--Legionellosis

[denovogenes]_111017	90.16	6e-168	bvu:BVU_2070	K03654	recQ	ATP-dependent DNA helicase RecQ	3.6.4.12	Genetic Information Processing--Folding, sorting and degradation--RNA degradation

[denovogenes]_111019	100.00	0.0	bvu:BVU_3213	K10716	kch, trkA, mthK, pch	voltage-gated potassium channel	--	--

[denovogenes]_11102	86.83	0.0	pdi:BDI_1668	K00615	E2.2.1.1, tktA, tktB	transketolase	2.2.1.1	Metabolism--Carbohydrate metabolism--Pentose phosphate pathway|Metabolism--Energy metabolism--Carbon fixation in photosynthetic organisms|Metabolism--Metabolism of terpenoids and polyketides--Biosynthesis of ansamycins|Metabolism--Global and overview maps--Metabolic pathways|Metabolism--Global and overview maps--Biosynthesis of secondary metabolites|Metabolism--Global and overview maps--Microbial metabolism in diverse environments|Metabolism--Global and overview maps--Biosynthesis of antibiotics|Metabolism--Global and overview maps--Carbon metabolism|Metabolism--Global and overview maps--Biosynthesis of amino acids

[denovogenes]_111020	99.60	0.0	fpla:A4U99_14700	K02036	pstB	phosphate transport system ATP-binding protein	3.6.3.27	Environmental Information Processing--Membrane transport--ABC transporters

[denovogenes]_111021	43.62	7e-56	boa:Bovatus_03429	K03655	recG	ATP-dependent DNA helicase RecG	3.6.4.12	Genetic Information Processing--Replication and repair--Homologous recombination

[denovogenes]_111024	100.00	0.0	bxy:BXY_47270	K06861	lptB	lipopolysaccharide export system ATP-binding protein	3.6.3.-	Environmental Information Processing--Membrane transport--ABC transporters

[denovogenes]_111025	98.03	1e-179	sdz:Asd1617_05986	K20543	bcsC	cellulose synthase operon protein C	--	--

[denovogenes]_111027	84.32	3e-148	lacy:A4V08_28835	K03394	cobI-cbiL	precorrin-2/cobalt-factor-2 C20-methyltransferase	2.1.1.130 2.1.1.151	Metabolism--Metabolism of cofactors and vitamins--Porphyrin and chlorophyll metabolism|Metabolism--Global and overview maps--Metabolic pathways

[denovogenes]_111028	99.21	0.0	boa:Bovatus_03961	K06861	lptB	lipopolysaccharide export system ATP-binding protein	3.6.3.-	Environmental Information Processing--Membrane transport--ABC transporters

[denovogenes]_11103	61.84	0.0	dvl:Dvul_2595	K07266	kpsC, lipA	capsular polysaccharide export protein	--	--

[denovogenes]_111031	40.65	3e-46	tpz:Tph_c20420	K06714	rocR	arginine utilization regulatory protein	--	--

[denovogenes]_111032	66.93	5e-120	cso:CLS_12200	K00065	kduD	2-deoxy-D-gluconate 3-dehydrogenase	1.1.1.125	Metabolism--Carbohydrate metabolism--Pentose and glucuronate interconversions

[denovogenes]_111033	60.96	3e-104	ibu:IB211_02806	K03169	topB	DNA topoisomerase III	5.99.1.2	--

[denovogenes]_111035	64.40	1e-92	cpy:Cphy_2241	K10541	mglC	methyl-galactoside transport system permease protein	--	Environmental Information Processing--Membrane transport--ABC transporters

[denovogenes]_111036	99.18	0.0	btho:Btheta7330_02046	K01126	E3.1.4.46, glpQ, ugpQ	glycerophosphoryl diester phosphodiesterase	3.1.4.46	Metabolism--Lipid metabolism--Glycerophospholipid metabolism

[denovogenes]_111037	79.53	1e-151	sdz:Asd1617_04899	K01176	AMY, amyA, malS	alpha-amylase	3.2.1.1	Metabolism--Carbohydrate metabolism--Starch and sucrose metabolism|Metabolism--Global and overview maps--Metabolic pathways|Organismal Systems--Digestive system--Carbohydrate digestion and absorption

[denovogenes]_111038	75.10	3e-139	bprm:CL3_17350	K02049	ABC.SN.A	NitT/TauT family transport system ATP-binding protein	--	--

[denovogenes]_111039	97.60	4e-179	fpla:A4U99_14250	K04042	glmU	bifunctional UDP-N-acetylglucosamine pyrophosphorylase / Glucosamine-1-phosphate N-acetyltransferase	2.7.7.23 2.3.1.157	Metabolism--Carbohydrate metabolism--Amino sugar and nucleotide sugar metabolism|Metabolism--Global and overview maps--Metabolic pathways|Metabolism--Global and overview maps--Biosynthesis of antibiotics

[denovogenes]_111040	100.00	0.0	pdi:BDI_3082	K09817	znuC	zinc transport system ATP-binding protein	3.6.3.-	Environmental Information Processing--Membrane transport--ABC transporters

[denovogenes]_111041	40.62	4e-21	eal:EAKF1_ch1745	K03755	adiY	AraC family transcriptional regulator, transcriptional activator of adiA	--	--

[denovogenes]_111042	63.49	2e-117	dai:Desaci_2132	K02049	ABC.SN.A	NitT/TauT family transport system ATP-binding protein	--	--

[denovogenes]_111043	98.42	5e-180	fpla:A4U99_18050	K06346	jag	spoIIIJ-associated protein	--	--

[denovogenes]_111044	64.98	1e-116	fpla:A4U99_13990	K07699	spo0A	two-component system, response regulator, stage 0 sporulation protein A	--	Environmental Information Processing--Signal transduction--Two-component system|Cellular Processes--Cellular community - prokaryotes--Quorum sensing

[denovogenes]_111046	41.06	2e-66	marm:YQ22_14885	K00059	fabG	3-oxoacyl-[acyl-carrier protein] reductase	1.1.1.100	Metabolism--Lipid metabolism--Fatty acid biosynthesis|Metabolism--Metabolism of cofactors and vitamins--Biotin metabolism|Metabolism--Lipid metabolism--Biosynthesis of unsaturated fatty acids|Metabolism--Global and overview maps--Metabolic pathways|Metabolism--Global and overview maps--Fatty acid metabolism

[denovogenes]_111047	100.00	0.0	cfd:CFNIH1_01190	K00065	kduD	2-deoxy-D-gluconate 3-dehydrogenase	1.1.1.125	Metabolism--Carbohydrate metabolism--Pentose and glucuronate interconversions

[denovogenes]_111048	51.98	2e-89	ccel:CCDG5_1599	K06168	miaB	tRNA-2-methylthio-N6-dimethylallyladenosine synthase	2.8.4.3	--

[denovogenes]_111050	85.38	4e-161	cpy:Cphy_2840	K02500	hisF	cyclase	4.1.3.-	Metabolism--Amino acid metabolism--Histidine metabolism|Metabolism--Global and overview maps--Metabolic pathways|Metabolism--Global and overview maps--Biosynthesis of secondary metabolites|Metabolism--Global and overview maps--Biosynthesis of amino acids

[denovogenes]_111051	41.08	4e-48	pdf:CD630DERM_28940	K01448	E3.5.1.28B, amiA, amiB, amiC	N-acetylmuramoyl-L-alanine amidase	3.5.1.28	Human Diseases--Drug resistance: Antimicrobial--Cationic antimicrobial peptide (CAMP) resistance

[denovogenes]_111052	100.00	0.0	pdi:BDI_3054	K01719	hemD, UROS	uroporphyrinogen-III synthase	4.2.1.75	Metabolism--Metabolism of cofactors and vitamins--Porphyrin and chlorophyll metabolism|Metabolism--Global and overview maps--Metabolic pathways|Metabolism--Global and overview maps--Biosynthesis of secondary metabolites

[denovogenes]_111054	98.81	0.0	fpla:A4U99_13405	K06898	K06898	uncharacterized protein	--	--

[denovogenes]_111055	91.73	4e-175	bcel:BcellWH2_02110	K01895	ACSS, acs	acetyl-CoA synthetase	6.2.1.1	Metabolism--Carbohydrate metabolism--Glycolysis / Gluconeogenesis|Metabolism--Carbohydrate metabolism--Pyruvate metabolism|Metabolism--Carbohydrate metabolism--Propanoate metabolism|Metabolism--Energy metabolism--Methane metabolism|Metabolism--Energy metabolism--Carbon fixation pathways in prokaryotes|Metabolism--Global and overview maps--Metabolic pathways|Metabolism--Global and overview maps--Biosynthesis of secondary metabolites|Metabolism--Global and overview maps--Microbial metabolism in diverse environments|Metabolism--Global and overview maps--Biosynthesis of antibiotics|Metabolism--Global and overview maps--Carbon metabolism

[denovogenes]_111057	49.80	3e-83	ckr:CKR_2282	K07124	K07124	uncharacterized protein	--	--

[denovogenes]_111058	86.11	6e-157	ova:OBV_40730	K14534	abfD	4-hydroxybutyryl-CoA dehydratase / vinylacetyl-CoA-Delta-isomerase	4.2.1.120 5.3.3.3	Metabolism--Carbohydrate metabolism--Butanoate metabolism|Metabolism--Energy metabolism--Carbon fixation pathways in prokaryotes|Metabolism--Global and overview maps--Metabolic pathways|Metabolism--Global and overview maps--Microbial metabolism in diverse environments|Metabolism--Global and overview maps--Carbon metabolism

[denovogenes]_111059	40.15	7e-49	fpl:Ferp_1187	K04114	bcrA, badF	benzoyl-CoA reductase subunit A	1.3.7.8	Metabolism--Xenobiotics biodegradation and metabolism--Benzoate degradation|Metabolism--Global and overview maps--Metabolic pathways|Metabolism--Global and overview maps--Microbial metabolism in diverse environments|Metabolism--Global and overview maps--Degradation of aromatic compounds

[denovogenes]_111060	41.73	1e-64	bprs:CK3_32830	K07080	K07080	uncharacterized protein	--	--

[denovogenes]_111062	56.52	5e-94	cpro:CPRO_06350	K03498	trkH, trkG, ktrB	trk system potassium uptake protein	--	--

[denovogenes]_111064	52.38	3e-96	rto:RTO_26190	K11749	rseP	regulator of sigma E protease	3.4.24.-	Cellular Processes--Cellular community - prokaryotes--Quorum sensing|Cellular Processes--Cell growth and death--Cell cycle - Caulobacter

[denovogenes]_111065	71.54	1e-128	tsh:Tsac_2218	K06016	pydC	beta-ureidopropionase / N-carbamoyl-L-amino-acid hydrolase	3.5.1.6 3.5.1.87	Metabolism--Nucleotide metabolism--Pyrimidine metabolism|Metabolism--Global and overview maps--Metabolic pathways

[denovogenes]_111067	69.49	6e-124	fpla:A4U99_07745	K07284	srtA	sortase A	3.4.22.70	--

[denovogenes]_111069	82.94	2e-146	bcel:BcellWH2_02382	K02563	murG	UDP-N-acetylglucosamine--N-acetylmuramyl-(pentapeptide) pyrophosphoryl-undecaprenol N-acetylglucosamine transferase	2.4.1.227	Metabolism--Glycan biosynthesis and metabolism--Peptidoglycan biosynthesis|Metabolism--Global and overview maps--Metabolic pathways|Human Diseases--Drug resistance: Antimicrobial--Vancomycin resistance|Cellular Processes--Cell growth and death--Cell cycle - Caulobacter

[denovogenes]_111071	94.86	3e-178	bhl:Bache_0201	K02065	mlaF, linL, mkl	phospholipid/cholesterol/gamma-HCH transport system ATP-binding protein	--	Environmental Information Processing--Membrane transport--ABC transporters

[denovogenes]_111073	99.21	0.0	cfd:CFNIH1_01785	K08306	mltC	membrane-bound lytic murein transglycosylase C	--	--

[denovogenes]_111074	98.42	0.0	cif:AL515_21615	K06223	dam	DNA adenine methylase	2.1.1.72	Genetic Information Processing--Replication and repair--Mismatch repair

[denovogenes]_111075	40.89	5e-54	rca:Rcas_3668	K08253	E2.7.10.2	non-specific protein-tyrosine kinase	2.7.10.2	--

[denovogenes]_111076	58.70	6e-87	ova:OBV_21420	K05946	E2.4.1.187, tagA	N-acetylglucosaminyldiphosphoundecaprenol N-acetyl-beta-D-mannosaminyltransferase	2.4.1.187	Cellular Processes--Cellular community - prokaryotes--Biofilm formation - Vibrio cholerae

[denovogenes]_111079	85.32	4e-145	byl:A4V09_14800	K03308	TC.NSS	neurotransmitter:Na+ symporter, NSS family	--	--

[denovogenes]_11108	96.44	0.0	ecc:c3690	K07266	kpsC, lipA	capsular polysaccharide export protein	--	--

[denovogenes]_111080	92.09	3e-172	bhl:Bache_0747	K09811	ftsX	cell division transport system permease protein	--	Environmental Information Processing--Membrane transport--ABC transporters

[denovogenes]_111081	98.02	0.0	bcel:BcellWH2_05212	K00874	kdgK	2-dehydro-3-deoxygluconokinase	2.7.1.45	Metabolism--Carbohydrate metabolism--Pentose phosphate pathway|Metabolism--Global and overview maps--Metabolic pathways|Metabolism--Global and overview maps--Microbial metabolism in diverse environments|Metabolism--Global and overview maps--Carbon metabolism

[denovogenes]_111083	100.00	0.0	brp:103839752	K08770	UBC	ubiquitin C	--	Organismal Systems--Endocrine system--PPAR signaling pathway

[denovogenes]_111084	76.10	4e-135	cle:Clole_3007	K12942	abgT	aminobenzoyl-glutamate transport protein	--	--

[denovogenes]_111085	44.94	2e-54	bcel:BcellWH2_04829	K18581	ugl	unsaturated chondroitin disaccharide hydrolase	3.2.1.180	--

[denovogenes]_111086	69.69	3e-135	byl:A4V09_00090	K01206	FUCA	alpha-L-fucosidase	3.2.1.51	Metabolism--Glycan biosynthesis and metabolism--Other glycan degradation

[denovogenes]_11109	99.70	0.0	bfg:BF638R_1599	K19049	cslA	chondroitin AC lyase	4.2.2.5	--

[denovogenes]_111093	92.91	3e-172	byl:A4V09_05450	K00198	cooS, acsA	carbon-monoxide dehydrogenase catalytic subunit	1.2.7.4	Metabolism--Xenobiotics biodegradation and metabolism--Nitrotoluene degradation|Metabolism--Energy metabolism--Methane metabolism|Metabolism--Energy metabolism--Carbon fixation pathways in prokaryotes|Metabolism--Global and overview maps--Microbial metabolism in diverse environments|Metabolism--Global and overview maps--Carbon metabolism

[denovogenes]_111095	69.23	9e-73	bprm:CL3_05240	K07497	K07497	putative transposase	--	--

[denovogenes]_111096	40.54	3e-46	tbc:A0O31_01812	K00525	E1.17.4.1A, nrdA, nrdE	ribonucleoside-diphosphate reductase alpha chain	1.17.4.1	Metabolism--Nucleotide metabolism--Purine metabolism|Metabolism--Nucleotide metabolism--Pyrimidine metabolism|Metabolism--Global and overview maps--Metabolic pathways

[denovogenes]_1111	45.07	0.0	ccel:CCDG5_0470	K03529	smc	chromosome segregation protein	--	--

[denovogenes]_111108	42.11	2e-47	wcb:AO080_02200	K07090	K07090	uncharacterized protein	--	--

[denovogenes]_11111	99.70	0.0	stb:SGPB_1592	K03316	TC.CPA1	monovalent cation:H+ antiporter, CPA1 family	--	--

[denovogenes]_111110	94.02	3e-174	pdi:BDI_2695	K01803	TPI, tpiA	triosephosphate isomerase (TIM)	5.3.1.1	Metabolism--Carbohydrate metabolism--Glycolysis / Gluconeogenesis|Metabolism--Carbohydrate metabolism--Fructose and mannose metabolism|Metabolism--Carbohydrate metabolism--Inositol phosphate metabolism|Metabolism--Energy metabolism--Carbon fixation in photosynthetic organisms|Metabolism--Global and overview maps--Metabolic pathways|Metabolism--Global and overview maps--Biosynthesis of secondary metabolites|Metabolism--Global and overview maps--Microbial metabolism in diverse environments|Metabolism--Global and overview maps--Biosynthesis of antibiotics|Metabolism--Global and overview maps--Carbon metabolism|Metabolism--Global and overview maps--Biosynthesis of amino acids

[denovogenes]_111111	71.65	2e-138	pdi:BDI_1226	K09861	K09861	uncharacterized protein	--	--

[denovogenes]_111112	52.57	4e-94	pdi:BDI_1917	K02169	bioC	malonyl-CoA O-methyltransferase	2.1.1.197	Metabolism--Metabolism of cofactors and vitamins--Biotin metabolism|Metabolism--Global and overview maps--Metabolic pathways

[denovogenes]_111113	53.94	6e-96	ava:Ava_2888	K05886	sdh	serine 3-dehydrogenase (NADP+)	1.1.1.276	--

[denovogenes]_111116	57.93	9e-61	etr:ETAE_1677	K19303	mepH	murein DD-endopeptidase	3.4.-.-	--

[denovogenes]_111117	67.19	5e-124	bcel:BcellWH2_01542	K12343	SRD5A1	3-oxo-5-alpha-steroid 4-dehydrogenase 1	1.3.1.22	Metabolism--Lipid metabolism--Steroid hormone biosynthesis

[denovogenes]_111119	63.09	3e-95	dfi:AXF13_00500	K03183	ubiE	demethylmenaquinone methyltransferase / 2-methoxy-6-polyprenyl-1,4-benzoquinol methylase	2.1.1.163 2.1.1.201	Metabolism--Metabolism of cofactors and vitamins--Ubiquinone and other terpenoid-quinone biosynthesis|Metabolism--Global and overview maps--Metabolic pathways|Metabolism--Global and overview maps--Biosynthesis of secondary metabolites

[denovogenes]_11112	75.53	0.0	aoe:Clos_2310	K03798	ftsH, hflB	cell division protease FtsH	3.4.24.-	--

[denovogenes]_111120	69.46	9e-120	csr:Cspa_c08670	K17076	K17076, lysY	putative lysine transport system ATP-binding protein	3.6.3.-	Environmental Information Processing--Membrane transport--ABC transporters

[denovogenes]_111129	40.18	1e-15	llu:AKJ09_05231	K00568	ubiG	2-polyprenyl-6-hydroxyphenyl methylase / 3-demethylubiquinone-9 3-methyltransferase	2.1.1.222 2.1.1.64	Metabolism--Metabolism of cofactors and vitamins--Ubiquinone and other terpenoid-quinone biosynthesis|Metabolism--Global and overview maps--Metabolic pathways|Metabolism--Global and overview maps--Biosynthesis of secondary metabolites

[denovogenes]_11113	77.00	0.0	cbut:ATN24_04495	K15987	hppA	K(+)-stimulated pyrophosphate-energized sodium pump	3.6.1.1	Metabolism--Energy metabolism--Oxidative phosphorylation

[denovogenes]_111130	73.50	5e-126	cso:CLS_19250	K07816	E2.7.6.5X	putative GTP pyrophosphokinase	2.7.6.5	Metabolism--Nucleotide metabolism--Purine metabolism

[denovogenes]_111132	40.61	1e-31	ruk:A4V00_15930	K08600	srtB	sortase B	3.4.22.70	--

[denovogenes]_111134	42.86	4e-63	bacy:QF06_17300	K19548	--	--	--	Metabolism--Global and overview maps--Biosynthesis of antibiotics

[denovogenes]_111135	83.47	2e-153	smu:SMU_621c	K07461	K07461	putative endonuclease	--	--

[denovogenes]_111136	75.89	7e-148	fpla:A4U99_14700	K02036	pstB	phosphate transport system ATP-binding protein	3.6.3.27	Environmental Information Processing--Membrane transport--ABC transporters

[denovogenes]_111137	73.81	8e-136	dvg:Deval_0658	K01995	livG	branched-chain amino acid transport system ATP-binding protein	--	Environmental Information Processing--Membrane transport--ABC transporters|Cellular Processes--Cellular community - prokaryotes--Quorum sensing

[denovogenes]_111138	100.00	0.0	paem:U769_05965	K08641	vanX	D-alanyl-D-alanine dipeptidase	3.4.13.22	Human Diseases--Drug resistance: Antimicrobial--Vancomycin resistance|Environmental Information Processing--Signal transduction--Two-component system

[denovogenes]_111140	98.42	0.0	bfb:VU15_11435	K01142	E3.1.11.2, xthA	exodeoxyribonuclease III	3.1.11.2	Genetic Information Processing--Replication and repair--Base excision repair

[denovogenes]_111142	41.90	7e-66	asr:WL1483_1114	K02444	glpR	DeoR family transcriptional regulator, glycerol-3-phosphate regulon repressor	--	--

[denovogenes]_111143	64.66	2e-119	dfi:AXF13_01510	K02049	ABC.SN.A	NitT/TauT family transport system ATP-binding protein	--	--

[denovogenes]_111144	95.44	2e-169	lacy:A4V08_28980	K03710	K03710	GntR family transcriptional regulator	--	--

[denovogenes]_111145	42.31	8e-54	kphy:AOZ06_02580	K03710	K03710	GntR family transcriptional regulator	--	--

[denovogenes]_111146	97.63	2e-179	lacy:A4V08_08775	K02500	hisF	cyclase	4.1.3.-	Metabolism--Amino acid metabolism--Histidine metabolism|Metabolism--Global and overview maps--Metabolic pathways|Metabolism--Global and overview maps--Biosynthesis of secondary metabolites|Metabolism--Global and overview maps--Biosynthesis of amino acids

[denovogenes]_111148	78.57	4e-147	lip:LI0060	K02444	glpR	DeoR family transcriptional regulator, glycerol-3-phosphate regulon repressor	--	--

[denovogenes]_111149	99.60	0.0	paeo:M801_3241	K03773	fklB	FKBP-type peptidyl-prolyl cis-trans isomerase FklB	5.2.1.8	--

[denovogenes]_11115	62.57	0.0	tur:AT726_04855	K04759	feoB	ferrous iron transport protein B	--	--

[denovogenes]_111150	73.91	2e-137	lir:LAW_00348	K01995	livG	branched-chain amino acid transport system ATP-binding protein	--	Environmental Information Processing--Membrane transport--ABC transporters|Cellular Processes--Cellular community - prokaryotes--Quorum sensing

[denovogenes]_111151	40.60	4e-19	vsc:VSVS12_02083	K02169	bioC	malonyl-CoA O-methyltransferase	2.1.1.197	Metabolism--Metabolism of cofactors and vitamins--Biotin metabolism|Metabolism--Global and overview maps--Metabolic pathways

[denovogenes]_111153	100.00	0.0	fpla:A4U99_08000	K02564	nagB, GNPDA	glucosamine-6-phosphate deaminase	3.5.99.6	Metabolism--Carbohydrate metabolism--Amino sugar and nucleotide sugar metabolism|Metabolism--Global and overview maps--Metabolic pathways

[denovogenes]_111154	100.00	0.0	paeo:M801_2451	K05783	benD-xylL	dihydroxycyclohexadiene carboxylate dehydrogenase	1.3.1.25 1.3.1.-	Metabolism--Xenobiotics biodegradation and metabolism--Benzoate degradation|Metabolism--Xenobiotics biodegradation and metabolism--Fluorobenzoate degradation|Metabolism--Xenobiotics biodegradation and metabolism--Xylene degradation|Metabolism--Global and overview maps--Metabolic pathways|Metabolism--Global and overview maps--Microbial metabolism in diverse environments|Metabolism--Global and overview maps--Degradation of aromatic compounds

[denovogenes]_111155	40.24	3e-46	sch:Sphch_0591	K15866	paaG	2-(1,2-epoxy-1,2-dihydrophenyl)acetyl-CoA isomerase	5.3.3.18	Metabolism--Amino acid metabolism--Phenylalanine metabolism|Metabolism--Global and overview maps--Microbial metabolism in diverse environments

[denovogenes]_111156	58.63	1e-107	coo:CCU_22700	K02003	ABC.CD.A	putative ABC transport system ATP-binding protein	--	--

[denovogenes]_111157	71.83	7e-128	cso:CLS_29160	K07090	K07090	uncharacterized protein	--	--

[denovogenes]_111159	41.15	3e-67	sgp:SpiGrapes_1826	K07720	yesN	two-component system, response regulator YesN	--	Environmental Information Processing--Signal transduction--Two-component system

[denovogenes]_11116	84.40	0.0	cbv:U729_29	K01006	ppdK	pyruvate, orthophosphate dikinase	2.7.9.1	Metabolism--Carbohydrate metabolism--Pyruvate metabolism|Metabolism--Energy metabolism--Carbon fixation in photosynthetic organisms|Metabolism--Energy metabolism--Carbon fixation pathways in prokaryotes|Metabolism--Global and overview maps--Metabolic pathways|Metabolism--Global and overview maps--Microbial metabolism in diverse environments|Metabolism--Global and overview maps--Carbon metabolism

[denovogenes]_111160	98.81	0.0	bfb:VU15_12390	K02036	pstB	phosphate transport system ATP-binding protein	3.6.3.27	Environmental Information Processing--Membrane transport--ABC transporters

[denovogenes]_111165	45.38	2e-24	byl:A4V09_22815	K07720	yesN	two-component system, response regulator YesN	--	Environmental Information Processing--Signal transduction--Two-component system

[denovogenes]_111167	99.21	0.0	bfb:VU15_06950	K00175	korB, oorB, oforB	2-oxoglutarate/2-oxoacid ferredoxin oxidoreductase subunit beta	1.2.7.3 1.2.7.11	Metabolism--Carbohydrate metabolism--Glycolysis / Gluconeogenesis|Metabolism--Carbohydrate metabolism--Citrate cycle (TCA cycle)|Metabolism--Carbohydrate metabolism--Pyruvate metabolism|Metabolism--Carbohydrate metabolism--Butanoate metabolism|Metabolism--Energy metabolism--Carbon fixation pathways in prokaryotes|Metabolism--Global and overview maps--Metabolic pathways|Metabolism--Global and overview maps--Microbial metabolism in diverse environments|Metabolism--Global and overview maps--Biosynthesis of antibiotics|Metabolism--Global and overview maps--Carbon metabolism

[denovogenes]_11117	100.00	0.0	pmr:PMI1659	K02400	flhA	flagellar biosynthesis protein FlhA	--	Cellular Processes--Cell motility--Flagellar assembly

[denovogenes]_111171	99.60	1e-177	paeo:M801_1881	K00059	fabG	3-oxoacyl-[acyl-carrier protein] reductase	1.1.1.100	Metabolism--Lipid metabolism--Fatty acid biosynthesis|Metabolism--Metabolism of cofactors and vitamins--Biotin metabolism|Metabolism--Lipid metabolism--Biosynthesis of unsaturated fatty acids|Metabolism--Global and overview maps--Metabolic pathways|Metabolism--Global and overview maps--Fatty acid metabolism

[denovogenes]_111172	60.40	2e-99	dgg:DGI_0332	K01803	TPI, tpiA	triosephosphate isomerase (TIM)	5.3.1.1	Metabolism--Carbohydrate metabolism--Glycolysis / Gluconeogenesis|Metabolism--Carbohydrate metabolism--Fructose and mannose metabolism|Metabolism--Carbohydrate metabolism--Inositol phosphate metabolism|Metabolism--Energy metabolism--Carbon fixation in photosynthetic organisms|Metabolism--Global and overview maps--Metabolic pathways|Metabolism--Global and overview maps--Biosynthesis of secondary metabolites|Metabolism--Global and overview maps--Microbial metabolism in diverse environments|Metabolism--Global and overview maps--Biosynthesis of antibiotics|Metabolism--Global and overview maps--Carbon metabolism|Metabolism--Global and overview maps--Biosynthesis of amino acids

[denovogenes]_111173	100.00	0.0	paeo:M801_1809	K06992	K06992	uncharacterized protein	--	--

[denovogenes]_111174	98.81	0.0	cfd:CFNIH1_16580	K00065	kduD	2-deoxy-D-gluconate 3-dehydrogenase	1.1.1.125	Metabolism--Carbohydrate metabolism--Pentose and glucuronate interconversions

[denovogenes]_111175	66.40	2e-122	bxy:BXY_12340	K12343	SRD5A1	3-oxo-5-alpha-steroid 4-dehydrogenase 1	1.3.1.22	Metabolism--Lipid metabolism--Steroid hormone biosynthesis

[denovogenes]_111178	57.14	2e-97	pft:JBW_01152	K01715	crt	enoyl-CoA hydratase	4.2.1.17	Metabolism--Carbohydrate metabolism--Butanoate metabolism|Metabolism--Global and overview maps--Carbon metabolism

[denovogenes]_111179	42.20	3e-29	ckl:CKL_1926	K01447	E3.5.1.28A, cwlA, xlyA, xlyB	N-acetylmuramoyl-L-alanine amidase	3.5.1.28	--

[denovogenes]_11118	91.10	0.0	bhl:Bache_3003	K04084	dsbD	thiol:disulfide interchange protein DsbD	1.8.1.8	--

[denovogenes]_111181	50.79	5e-88	csh:Closa_1926	K02027	ABC.MS.S	multiple sugar transport system substrate-binding protein	--	--

[denovogenes]_111182	94.47	1e-176	lacy:A4V08_08190	K07719	ycbB, glnL	two-component system, response regulator YcbB	--	Environmental Information Processing--Signal transduction--Two-component system

[denovogenes]_111186	48.39	1e-75	llr:llh_12875	K07493	K07493	putative transposase	--	--

[denovogenes]_111188	98.42	0.0	lacy:A4V08_18660	K02036	pstB	phosphate transport system ATP-binding protein	3.6.3.27	Environmental Information Processing--Membrane transport--ABC transporters

[denovogenes]_111189	87.35	1e-164	cso:CLS_12050	K01611	speD, AMD1	S-adenosylmethionine decarboxylase	4.1.1.50	Metabolism--Amino acid metabolism--Cysteine and methionine metabolism|Metabolism--Amino acid metabolism--Arginine and proline metabolism|Metabolism--Global and overview maps--Metabolic pathways

[denovogenes]_111190	42.80	9e-61	strm:M444_35575	K19548	--	--	--	Metabolism--Global and overview maps--Biosynthesis of antibiotics

[denovogenes]_111191	93.28	7e-173	lacy:A4V08_33320	K00817	hisC	histidinol-phosphate aminotransferase	2.6.1.9	Metabolism--Amino acid metabolism--Histidine metabolism|Metabolism--Amino acid metabolism--Tyrosine metabolism|Metabolism--Amino acid metabolism--Phenylalanine metabolism|Metabolism--Amino acid metabolism--Phenylalanine, tyrosine and tryptophan biosynthesis|Metabolism--Biosynthesis of other secondary metabolites--Novobiocin biosynthesis|Metabolism--Biosynthesis of other secondary metabolites--Tropane, piperidine and pyridine alkaloid biosynthesis|Metabolism--Global and overview maps--Metabolic pathways|Metabolism--Global and overview maps--Biosynthesis of secondary metabolites|Metabolism--Global and overview maps--Biosynthesis of antibiotics|Metabolism--Global and overview maps--Biosynthesis of amino acids

[denovogenes]_111192	43.70	2e-62	lsl:LSL_1486	K01181	E3.2.1.8, xynA	endo-1,4-beta-xylanase	3.2.1.8	--

[denovogenes]_111193	69.17	1e-127	rde:RD1_0979	K00937	ppk	polyphosphate kinase	2.7.4.1	Metabolism--Energy metabolism--Oxidative phosphorylation|Genetic Information Processing--Folding, sorting and degradation--RNA degradation

[denovogenes]_111194	99.60	0.0	ecos:EC958_3015	K03787	surE	5'-nucleotidase	3.1.3.5	Metabolism--Nucleotide metabolism--Purine metabolism|Metabolism--Nucleotide metabolism--Pyrimidine metabolism|Metabolism--Metabolism of cofactors and vitamins--Nicotinate and nicotinamide metabolism|Metabolism--Global and overview maps--Metabolic pathways|Metabolism--Global and overview maps--Biosynthesis of secondary metabolites

[denovogenes]_111195	100.00	0.0	pap:PSPA7_2568	K13378	nuoCD	NADH-quinone oxidoreductase subunit C/D	1.6.5.3	Metabolism--Energy metabolism--Oxidative phosphorylation|Metabolism--Global and overview maps--Metabolic pathways

[denovogenes]_111197	50.40	4e-77	puf:UFO1_0101	K00615	E2.2.1.1, tktA, tktB	transketolase	2.2.1.1	Metabolism--Carbohydrate metabolism--Pentose phosphate pathway|Metabolism--Energy metabolism--Carbon fixation in photosynthetic organisms|Metabolism--Metabolism of terpenoids and polyketides--Biosynthesis of ansamycins|Metabolism--Global and overview maps--Metabolic pathways|Metabolism--Global and overview maps--Biosynthesis of secondary metabolites|Metabolism--Global and overview maps--Microbial metabolism in diverse environments|Metabolism--Global and overview maps--Biosynthesis of antibiotics|Metabolism--Global and overview maps--Carbon metabolism|Metabolism--Global and overview maps--Biosynthesis of amino acids

[denovogenes]_111198	53.94	1e-88	rum:CK1_36700	K16899	addB	ATP-dependent helicase/nuclease subunit B	3.1.-.- 3.6.4.12	--

[denovogenes]_1112	71.91	0.0	lir:LAW_00024	K01007	pps, ppsA	pyruvate, water dikinase	2.7.9.2	Metabolism--Carbohydrate metabolism--Pyruvate metabolism|Metabolism--Energy metabolism--Methane metabolism|Metabolism--Energy metabolism--Carbon fixation pathways in prokaryotes|Metabolism--Global and overview maps--Metabolic pathways|Metabolism--Global and overview maps--Microbial metabolism in diverse environments|Metabolism--Global and overview maps--Carbon metabolism

[denovogenes]_111200	99.60	0.0	btho:Btheta7330_01349	K01889	FARSA, pheS	phenylalanyl-tRNA synthetase alpha chain	6.1.1.20	Genetic Information Processing--Translation--Aminoacyl-tRNA biosynthesis

[denovogenes]_111201	98.81	0.0	cif:AL515_18405	K03787	surE	5'-nucleotidase	3.1.3.5	Metabolism--Nucleotide metabolism--Purine metabolism|Metabolism--Nucleotide metabolism--Pyrimidine metabolism|Metabolism--Metabolism of cofactors and vitamins--Nicotinate and nicotinamide metabolism|Metabolism--Global and overview maps--Metabolic pathways|Metabolism--Global and overview maps--Biosynthesis of secondary metabolites

[denovogenes]_111202	98.81	0.0	eas:Entas_4245	K00757	udp, UPP	uridine phosphorylase	2.4.2.3	Metabolism--Nucleotide metabolism--Pyrimidine metabolism|Metabolism--Xenobiotics biodegradation and metabolism--Drug metabolism - other enzymes|Metabolism--Global and overview maps--Metabolic pathways

[denovogenes]_111204	100.00	0.0	cfd:CFNIH1_06685	K02040	pstS	phosphate transport system substrate-binding protein	--	Environmental Information Processing--Membrane transport--ABC transporters|Environmental Information Processing--Signal transduction--Two-component system|Human Diseases--Infectious diseases: Bacterial--Tuberculosis

[denovogenes]_111205	67.87	2e-121	ccu:Ccur_13310	K02028	ABC.PA.A	polar amino acid transport system ATP-binding protein	3.6.3.21	--

[denovogenes]_111206	73.91	3e-134	pdi:BDI_0690	K00382	DLD, lpd, pdhD	dihydrolipoamide dehydrogenase	1.8.1.4	Metabolism--Carbohydrate metabolism--Glycolysis / Gluconeogenesis|Metabolism--Carbohydrate metabolism--Citrate cycle (TCA cycle)|Metabolism--Amino acid metabolism--Glycine, serine and threonine metabolism|Metabolism--Amino acid metabolism--Valine, leucine and isoleucine degradation|Metabolism--Carbohydrate metabolism--Pyruvate metabolism|Metabolism--Carbohydrate metabolism--Glyoxylate and dicarboxylate metabolism|Metabolism--Carbohydrate metabolism--Propanoate metabolism|Metabolism--Global and overview maps--Metabolic pathways|Metabolism--Global and overview maps--Biosynthesis of secondary metabolites|Metabolism--Global and overview maps--Microbial metabolism in diverse environments|Metabolism--Global and overview maps--Biosynthesis of antibiotics|Metabolism--Global and overview maps--Carbon metabolism

[denovogenes]_111209	47.81	5e-74	pdf:CD630DERM_02940	K01992	ABC-2.P	ABC-2 type transport system permease protein	--	--

[denovogenes]_11121	40.63	1e-138	cpro:CPRO_10490	K03406	mcp	methyl-accepting chemotaxis protein	--	Environmental Information Processing--Signal transduction--Two-component system|Cellular Processes--Cell motility--Bacterial chemotaxis

[denovogenes]_111210	41.60	2e-55	caby:Cabys_1689	K02081	agaR	DeoR family transcriptional regulator, aga operon transcriptional repressor	--	--

[denovogenes]_111211	56.00	2e-92	csh:Closa_2136	K09138	K09138	uncharacterized protein	--	--

[denovogenes]_111212	100.00	2e-180	paf:PAM18_2286	K19610	pfeR, pirR	two-component system, OmpR family, response regulator PfeR	--	Environmental Information Processing--Signal transduction--Two-component system

[denovogenes]_111215	68.67	3e-118	cpy:Cphy_1927	K03498	trkH, trkG, ktrB	trk system potassium uptake protein	--	--

[denovogenes]_111219	49.42	4e-76	bfs:BF9343_3562	K02477	K02477	two-component system, LytT family, response regulator	--	--

[denovogenes]_111224	44.84	4e-65	fjo:Fjoh_1841	K01113	phoD	alkaline phosphatase D	3.1.3.1	Metabolism--Xenobiotics biodegradation and metabolism--Aminobenzoate degradation|Metabolism--Metabolism of cofactors and vitamins--Folate biosynthesis|Metabolism--Global and overview maps--Metabolic pathways|Metabolism--Global and overview maps--Microbial metabolism in diverse environments|Environmental Information Processing--Signal transduction--Two-component system

[denovogenes]_111226	98.42	0.0	enc:ECL_04407	K06907	K06907	uncharacterized protein	--	--

[denovogenes]_111227	86.08	3e-121	blx:GS08_01055	K03646	tolA	colicin import membrane protein	--	--

[denovogenes]_111228	45.02	1e-58	mst:Msp_0683	K00788	thiE	thiamine-phosphate pyrophosphorylase	2.5.1.3	Metabolism--Metabolism of cofactors and vitamins--Thiamine metabolism|Metabolism--Global and overview maps--Metabolic pathways

[denovogenes]_111229	100.00	0.0	bfb:VU15_18400	K06861	lptB	lipopolysaccharide export system ATP-binding protein	3.6.3.-	Environmental Information Processing--Membrane transport--ABC transporters

[denovogenes]_111230	45.38	5e-65	ols:Olsu_0622	K03710	K03710	GntR family transcriptional regulator	--	--

[denovogenes]_111231	98.81	0.0	ecg:E2348C_0292	K01659	prpC	2-methylcitrate synthase	2.3.3.5	Metabolism--Carbohydrate metabolism--Propanoate metabolism

[denovogenes]_111238	98.03	0.0	pmr:PMI1111	K01876	DARS, aspS	aspartyl-tRNA synthetase	6.1.1.12	Genetic Information Processing--Translation--Aminoacyl-tRNA biosynthesis

[denovogenes]_111239	54.58	3e-102	cbl:CLK_1484	K01271	pepQ	Xaa-Pro dipeptidase	3.4.13.9	--

[denovogenes]_11124	55.11	0.0	eha:Ethha_1458	K02945	RP-S1, rpsA	small subunit ribosomal protein S1	--	Genetic Information Processing--Translation--Ribosome

[denovogenes]_11124	55.11	0.0	eha:Ethha_1458	K03527	ispH, lytB	4-hydroxy-3-methylbut-2-en-1-yl diphosphate reductase	1.17.7.4	Metabolism--Metabolism of terpenoids and polyketides--Terpenoid backbone biosynthesis|Metabolism--Global and overview maps--Metabolic pathways|Metabolism--Global and overview maps--Biosynthesis of secondary metabolites|Metabolism--Global and overview maps--Biosynthesis of antibiotics

[denovogenes]_111241	63.71	3e-96	shi:Shel_28160	K00215	dapB	4-hydroxy-tetrahydrodipicolinate reductase	1.17.1.8	Metabolism--Biosynthesis of other secondary metabolites--Monobactam biosynthesis|Metabolism--Amino acid metabolism--Lysine biosynthesis|Metabolism--Global and overview maps--Metabolic pathways|Metabolism--Global and overview maps--Biosynthesis of secondary metabolites|Metabolism--Global and overview maps--Microbial metabolism in diverse environments|Metabolism--Global and overview maps--Biosynthesis of antibiotics|Metabolism--Global and overview maps--Biosynthesis of amino acids

[denovogenes]_111242	40.61	4e-50	efa:EF1211	K05910	npr	NADH peroxidase	1.11.1.1	--

[denovogenes]_111243	97.23	3e-172	lacy:A4V08_30030	K02056	ABC.SS.A	simple sugar transport system ATP-binding protein	3.6.3.17	--

[denovogenes]_111244	95.28	3e-176	bhl:Bache_0626	K03551	ruvB	holliday junction DNA helicase RuvB	3.6.4.12	Genetic Information Processing--Replication and repair--Homologous recombination

[denovogenes]_111247	66.67	3e-125	cbac:JI75_00340	K05847	opuA	osmoprotectant transport system ATP-binding protein	--	Environmental Information Processing--Membrane transport--ABC transporters

[denovogenes]_111248	68.78	2e-117	cld:CLSPO_c01670	K07566	tsaC, rimN, SUA5	L-threonylcarbamoyladenylate synthase	2.7.7.87	--

[denovogenes]_111249	84.39	3e-122	afd:Alfi_3274	K00058	serA, PHGDH	D-3-phosphoglycerate dehydrogenase	1.1.1.95	Metabolism--Amino acid metabolism--Glycine, serine and threonine metabolism|Metabolism--Energy metabolism--Methane metabolism|Metabolism--Global and overview maps--Metabolic pathways|Metabolism--Global and overview maps--Microbial metabolism in diverse environments|Metabolism--Global and overview maps--Biosynthesis of antibiotics|Metabolism--Global and overview maps--Carbon metabolism|Metabolism--Global and overview maps--Biosynthesis of amino acids

[denovogenes]_11125	85.61	0.0	cso:CLS_13150	K01712	hutU, UROC1	urocanate hydratase	4.2.1.49	Metabolism--Amino acid metabolism--Histidine metabolism|Metabolism--Global and overview maps--Metabolic pathways

[denovogenes]_111250	59.76	2e-106	gme:Gmet_1910	K00525	E1.17.4.1A, nrdA, nrdE	ribonucleoside-diphosphate reductase alpha chain	1.17.4.1	Metabolism--Nucleotide metabolism--Purine metabolism|Metabolism--Nucleotide metabolism--Pyrimidine metabolism|Metabolism--Global and overview maps--Metabolic pathways

[denovogenes]_111251	100.00	0.0	pdf:CD630DERM_03950	K20882	hadA	(R)-2-hydroxy-4-methylpentanoate CoA-transferase	2.8.3.24	--

[denovogenes]_111252	94.86	4e-175	pvl:AOB99_12220	K03438	mraW, rsmH	16S rRNA (cytosine1402-N4)-methyltransferase	2.1.1.199	--

[denovogenes]_111254	47.01	1e-69	coo:CCU_04790	K03722	dinG	ATP-dependent DNA helicase DinG	3.6.4.12	--

[denovogenes]_111257	99.61	5e-171	paeb:NCGM1900_4839	K21023	mucR	diguanylate cyclase	2.7.7.65	Cellular Processes--Cellular community - prokaryotes--Biofilm formation - Pseudomonas aeruginosa

[denovogenes]_11126	41.58	5e-81	etc:ETAC_09195	K07714	atoC	two-component system, NtrC family, response regulator AtoC	--	Environmental Information Processing--Signal transduction--Two-component system

[denovogenes]_111260	68.77	4e-109	cgo:Corgl_0090	K06147	ABCB-BAC	ATP-binding cassette, subfamily B, bacterial	--	--

[denovogenes]_111263	64.98	5e-112	byl:A4V09_04090	K06409	spoVB	stage V sporulation protein B	--	--

[denovogenes]_111264	98.82	0.0	pdi:BDI_1650	K01679	E4.2.1.2B, fumC	fumarate hydratase, class II	4.2.1.2	Metabolism--Carbohydrate metabolism--Citrate cycle (TCA cycle)|Metabolism--Carbohydrate metabolism--Pyruvate metabolism|Metabolism--Energy metabolism--Carbon fixation pathways in prokaryotes|Metabolism--Global and overview maps--Metabolic pathways|Metabolism--Global and overview maps--Biosynthesis of secondary metabolites|Metabolism--Global and overview maps--Microbial metabolism in diverse environments|Metabolism--Global and overview maps--Biosynthesis of antibiotics|Metabolism--Global and overview maps--Carbon metabolism|Human Diseases--Cancers: Overview--Pathways in cancer|Human Diseases--Cancers: Specific types--Renal cell carcinoma

[denovogenes]_111265	97.13	5e-178	lacy:A4V08_09140	K01755	argH, ASL	argininosuccinate lyase	4.3.2.1	Metabolism--Amino acid metabolism--Arginine biosynthesis|Metabolism--Amino acid metabolism--Alanine, aspartate and glutamate metabolism|Metabolism--Global and overview maps--Metabolic pathways|Metabolism--Global and overview maps--Biosynthesis of secondary metabolites|Metabolism--Global and overview maps--Biosynthesis of antibiotics|Metabolism--Global and overview maps--Biosynthesis of amino acids

[denovogenes]_11127	63.45	0.0	csb:CLSA_c10070	K20116	PTS-Glc1-EIIA, ptsG, glcA, glcB	PTS system, glucose-specific IIA component	2.7.1.199	Metabolism--Carbohydrate metabolism--Glycolysis / Gluconeogenesis|Metabolism--Carbohydrate metabolism--Amino sugar and nucleotide sugar metabolism|Environmental Information Processing--Membrane transport--Phosphotransferase system (PTS)

[denovogenes]_11127	63.45	0.0	csb:CLSA_c10070	K20117	PTS-Glc1-EIIB, ptsG, glcA, glcB	PTS system, glucose-specific IIB component	2.7.1.199	Metabolism--Carbohydrate metabolism--Glycolysis / Gluconeogenesis|Metabolism--Carbohydrate metabolism--Amino sugar and nucleotide sugar metabolism|Environmental Information Processing--Membrane transport--Phosphotransferase system (PTS)

[denovogenes]_11127	63.45	0.0	csb:CLSA_c10070	K20118	PTS-Glc1-EIIC, ptsG, glcA, glcB	PTS system, glucose-specific IIC component	--	Metabolism--Carbohydrate metabolism--Glycolysis / Gluconeogenesis|Metabolism--Carbohydrate metabolism--Amino sugar and nucleotide sugar metabolism|Environmental Information Processing--Membrane transport--Phosphotransferase system (PTS)

[denovogenes]_111271	62.20	8e-101	byl:A4V09_03505	K03588	ftsW, spoVE	cell division protein FtsW	--	Cellular Processes--Cell growth and death--Cell cycle - Caulobacter

[denovogenes]_111272	98.02	2e-180	bcel:BcellWH2_05134	K00259	ald	alanine dehydrogenase	1.4.1.1	Metabolism--Amino acid metabolism--Alanine, aspartate and glutamate metabolism|Metabolism--Metabolism of other amino acids--Taurine and hypotaurine metabolism|Metabolism--Global and overview maps--Metabolic pathways

[denovogenes]_111274	90.55	4e-149	pvl:AOB99_13920	K05792	terA	tellurite resistance protein TerA	--	--

[denovogenes]_111276	47.06	1e-73	bsa:Bacsa_0280	K02551	menD	2-succinyl-5-enolpyruvyl-6-hydroxy-3-cyclohexene-1-carboxylate synthase	2.2.1.9	Metabolism--Metabolism of cofactors and vitamins--Ubiquinone and other terpenoid-quinone biosynthesis|Metabolism--Global and overview maps--Metabolic pathways|Metabolism--Global and overview maps--Biosynthesis of secondary metabolites

[denovogenes]_111278	91.73	6e-170	bfs:BF9343_3781	K03572	mutL	DNA mismatch repair protein MutL	--	Genetic Information Processing--Replication and repair--Mismatch repair

[denovogenes]_11128	68.63	0.0	elm:ELI_3949	K03549	kup	KUP system potassium uptake protein	--	--

[denovogenes]_111281	66.94	2e-117	ccm:Ccan_04140	K00700	GBE1, glgB	1,4-alpha-glucan branching enzyme	2.4.1.18	Metabolism--Carbohydrate metabolism--Starch and sucrose metabolism|Metabolism--Global and overview maps--Metabolic pathways|Metabolism--Global and overview maps--Biosynthesis of secondary metabolites

[denovogenes]_111283	94.09	1e-175	byl:A4V09_21870	K01810	GPI, pgi	glucose-6-phosphate isomerase	5.3.1.9	Metabolism--Carbohydrate metabolism--Glycolysis / Gluconeogenesis|Metabolism--Carbohydrate metabolism--Pentose phosphate pathway|Metabolism--Carbohydrate metabolism--Starch and sucrose metabolism|Metabolism--Carbohydrate metabolism--Amino sugar and nucleotide sugar metabolism|Metabolism--Global and overview maps--Metabolic pathways|Metabolism--Global and overview maps--Biosynthesis of secondary metabolites|Metabolism--Global and overview maps--Microbial metabolism in diverse environments|Metabolism--Global and overview maps--Biosynthesis of antibiotics|Metabolism--Global and overview maps--Carbon metabolism

[denovogenes]_111284	85.89	2e-144	pdi:BDI_0784	K08138	xylE	MFS transporter, SP family, xylose:H+ symportor	--	--

[denovogenes]_111286	76.38	2e-141	cgo:Corgl_1448	K01223	E3.2.1.86B, bglA	6-phospho-beta-glucosidase	3.2.1.86	Metabolism--Carbohydrate metabolism--Glycolysis / Gluconeogenesis|Metabolism--Carbohydrate metabolism--Starch and sucrose metabolism

[denovogenes]_111287	99.21	0.0	pdf:CD630DERM_00140	K01875	SARS, serS	seryl-tRNA synthetase	6.1.1.11	Genetic Information Processing--Translation--Aminoacyl-tRNA biosynthesis

[denovogenes]_111288	78.74	3e-138	rto:RTO_24090	K06213	mgtE	magnesium transporter	--	--

[denovogenes]_111290	64.86	2e-103	cgo:Corgl_0670	K00147	proA	glutamate-5-semialdehyde dehydrogenase	1.2.1.41	Metabolism--Amino acid metabolism--Arginine and proline metabolism|Metabolism--Biosynthesis of other secondary metabolites--Carbapenem biosynthesis|Metabolism--Global and overview maps--Metabolic pathways|Metabolism--Global and overview maps--Biosynthesis of secondary metabolites|Metabolism--Global and overview maps--Biosynthesis of antibiotics|Metabolism--Global and overview maps--Biosynthesis of amino acids

[denovogenes]_111292	40.57	5e-33	ade:Adeh_4008	K07636	phoR	two-component system, OmpR family, phosphate regulon sensor histidine kinase PhoR	2.7.13.3	Environmental Information Processing--Signal transduction--Two-component system

[denovogenes]_111293	99.21	4e-176	cfd:CFNIH1_22935	K00341	nuoL	NADH-quinone oxidoreductase subunit L	1.6.5.3	Metabolism--Energy metabolism--Oxidative phosphorylation|Metabolism--Global and overview maps--Metabolic pathways

[denovogenes]_111296	75.69	5e-143	csh:Closa_2129	K01928	murE	UDP-N-acetylmuramoyl-L-alanyl-D-glutamate--2,6-diaminopimelate ligase	6.3.2.13	Metabolism--Amino acid metabolism--Lysine biosynthesis|Metabolism--Glycan biosynthesis and metabolism--Peptidoglycan biosynthesis

[denovogenes]_111297	96.44	2e-179	bxy:BXY_12340	K12343	SRD5A1	3-oxo-5-alpha-steroid 4-dehydrogenase 1	1.3.1.22	Metabolism--Lipid metabolism--Steroid hormone biosynthesis

[denovogenes]_111299	55.60	2e-93	csh:Closa_0385	K19285	nfrA1	FMN reductase (NADPH)	1.5.1.38	Metabolism--Metabolism of cofactors and vitamins--Riboflavin metabolism|Metabolism--Global and overview maps--Metabolic pathways

[denovogenes]_1113	99.75	0.0	efi:OG1RF_10201	K03723	mfd	transcription-repair coupling factor (superfamily II helicase)	3.6.4.-	Genetic Information Processing--Replication and repair--Nucleotide excision repair

[denovogenes]_11130	76.30	0.0	ibu:IB211_00109c	K01972	E6.5.1.2, ligA, ligB	DNA ligase (NAD+)	6.5.1.2	Genetic Information Processing--Replication and repair--DNA replication|Genetic Information Processing--Replication and repair--Base excision repair|Genetic Information Processing--Replication and repair--Nucleotide excision repair|Genetic Information Processing--Replication and repair--Mismatch repair

[denovogenes]_111301	99.60	0.0	boa:Bovatus_01080	K01142	E3.1.11.2, xthA	exodeoxyribonuclease III	3.1.11.2	Genetic Information Processing--Replication and repair--Base excision repair

[denovogenes]_111308	100.00	0.0	sem:STMDT12_C39050	K00348	nqrC	Na+-transporting NADH:ubiquinone oxidoreductase subunit C	1.6.5.8	--

[denovogenes]_11131	92.15	0.0	bdh:GV66_16645	K03702	uvrB	excinuclease ABC subunit B	--	Genetic Information Processing--Replication and repair--Nucleotide excision repair

[denovogenes]_111315	100.00	3e-174	pvl:AOB99_11710	K00782	lldG	L-lactate dehydrogenase complex protein LldG	--	--

[denovogenes]_111316	41.44	7e-19	mve:X875_6670	K21029	moeB	molybdopterin-synthase adenylyltransferase	2.7.7.80	Genetic Information Processing--Folding, sorting and degradation--Sulfur relay system

[denovogenes]_111317	98.42	0.0	ese:ECSF_4319	K03800	lplA, lplJ	lipoate---protein ligase	6.3.1.20	Metabolism--Metabolism of cofactors and vitamins--Lipoic acid metabolism|Metabolism--Global and overview maps--Metabolic pathways

[denovogenes]_11132	75.30	0.0	csh:Closa_1111	K01972	E6.5.1.2, ligA, ligB	DNA ligase (NAD+)	6.5.1.2	Genetic Information Processing--Replication and repair--DNA replication|Genetic Information Processing--Replication and repair--Base excision repair|Genetic Information Processing--Replication and repair--Nucleotide excision repair|Genetic Information Processing--Replication and repair--Mismatch repair

[denovogenes]_111320	49.43	3e-78	rob:CK5_29000	K01154	hsdS	type I restriction enzyme, S subunit	3.1.21.3	--

[denovogenes]_111324	65.82	4e-112	vpr:Vpar_0919	K03601	xseA	exodeoxyribonuclease VII large subunit	3.1.11.6	Genetic Information Processing--Replication and repair--Mismatch repair

[denovogenes]_111327	43.27	4e-60	ccz:CCALI_00269	K00034	gdh	glucose 1-dehydrogenase	1.1.1.47	Metabolism--Carbohydrate metabolism--Pentose phosphate pathway|Metabolism--Global and overview maps--Microbial metabolism in diverse environments|Metabolism--Global and overview maps--Carbon metabolism

[denovogenes]_111329	75.88	4e-135	ipo:Ilyop_0576	K03544	clpX, CLPX	ATP-dependent Clp protease ATP-binding subunit ClpX	--	Cellular Processes--Cell growth and death--Cell cycle - Caulobacter

[denovogenes]_11133	77.81	0.0	pfre:RM25_1051	K04066	priA	primosomal protein N' (replication factor Y) (superfamily II helicase)	3.6.4.-	Genetic Information Processing--Replication and repair--Homologous recombination

[denovogenes]_111330	41.34	1e-55	tep:TepRe1_0059	K01436	K01436	amidohydrolase	3.5.1.-	--

[denovogenes]_111332	63.78	3e-110	ipo:Ilyop_1277	K03215	rumA	23S rRNA (uracil1939-C5)-methyltransferase	2.1.1.190	--

[denovogenes]_111333	100.00	0.0	pvl:AOB99_08775	K03721	tyrR	transcriptional regulator of aroF, aroG, tyrA and aromatic amino acid transport	--	--

[denovogenes]_111335	100.00	0.0	eoc:CE10_0484	K00042	garR, glxR	2-hydroxy-3-oxopropionate reductase	1.1.1.60	Metabolism--Carbohydrate metabolism--Glyoxylate and dicarboxylate metabolism|Metabolism--Global and overview maps--Metabolic pathways

[denovogenes]_111338	59.17	3e-93	eft:M395_00960	K06183	rsuA	16S rRNA pseudouridine516 synthase	5.4.99.19	--

[denovogenes]_111339	51.20	2e-88	gfe:Gferi_24825	K07053	E3.1.3.97	3',5'-nucleoside bisphosphate phosphatase	3.1.3.97	--

[denovogenes]_111340	80.31	2e-144	fne:FSDG_00997	K01753	dsdA	D-serine dehydratase	4.3.1.18	Metabolism--Amino acid metabolism--Glycine, serine and threonine metabolism

[denovogenes]_111342	91.70	2e-173	sot:102593938	K08235	E2.4.1.207	xyloglucan:xyloglucosyl transferase	2.4.1.207	--

[denovogenes]_111344	99.60	5e-179	ssj:SSON53_06385	K02406	fliC	flagellin	--	Environmental Information Processing--Signal transduction--Two-component system|Cellular Processes--Cell motility--Flagellar assembly|Organismal Systems--Immune system--NOD-like receptor signaling pathway|Organismal Systems--Environmental adaptation--Plant-pathogen interaction|Human Diseases--Infectious diseases: Bacterial--Salmonella infection|Human Diseases--Infectious diseases: Bacterial--Legionellosis

[denovogenes]_111345	46.18	3e-74	sdc:SDSE_2041	K00885	nanK	N-acylmannosamine kinase	2.7.1.60	Metabolism--Carbohydrate metabolism--Amino sugar and nucleotide sugar metabolism|Metabolism--Global and overview maps--Metabolic pathways

[denovogenes]_111347	42.19	1e-59	afd:Alfi_1074	K12373	HEXA_B	hexosaminidase	3.2.1.52	Metabolism--Glycan biosynthesis and metabolism--Other glycan degradation|Metabolism--Glycan biosynthesis and metabolism--Various types of N-glycan biosynthesis|Metabolism--Carbohydrate metabolism--Amino sugar and nucleotide sugar metabolism|Metabolism--Glycan biosynthesis and metabolism--Glycosaminoglycan degradation|Metabolism--Glycan biosynthesis and metabolism--Glycosphingolipid biosynthesis - globo and isoglobo series|Metabolism--Glycan biosynthesis and metabolism--Glycosphingolipid biosynthesis - ganglio series|Metabolism--Global and overview maps--Metabolic pathways|Cellular Processes--Transport and catabolism--Lysosome

[denovogenes]_111348	61.29	4e-103	bcel:BcellWH2_05226	K17680	PEO1	twinkle protein	3.6.4.12	--

[denovogenes]_11135	90.62	0.0	pfre:RM25_0495	K00174	korA, oorA, oforA	2-oxoglutarate/2-oxoacid ferredoxin oxidoreductase subunit alpha	1.2.7.3 1.2.7.11	Metabolism--Carbohydrate metabolism--Glycolysis / Gluconeogenesis|Metabolism--Carbohydrate metabolism--Citrate cycle (TCA cycle)|Metabolism--Carbohydrate metabolism--Pyruvate metabolism|Metabolism--Carbohydrate metabolism--Butanoate metabolism|Metabolism--Energy metabolism--Carbon fixation pathways in prokaryotes|Metabolism--Global and overview maps--Metabolic pathways|Metabolism--Global and overview maps--Microbial metabolism in diverse environments|Metabolism--Global and overview maps--Biosynthesis of antibiotics|Metabolism--Global and overview maps--Carbon metabolism

[denovogenes]_111350	46.36	1e-68	deh:cbdbA122	K03605	hyaD, hybD	hydrogenase maturation protease	3.4.23.-	--

[denovogenes]_111352	81.97	2e-144	cbv:U729_2811	K00554	trmD	tRNA (guanine37-N1)-methyltransferase	2.1.1.228	--

[denovogenes]_111353	100.00	0.0	mmk:MU9_2123	K01420	fnr	CRP/FNR family transcriptional regulator, anaerobic regulatory protein	--	--

[denovogenes]_111354	100.00	0.0	mmk:MU9_1662	K03584	recO	DNA repair protein RecO (recombination protein O)	--	Genetic Information Processing--Replication and repair--Homologous recombination

[denovogenes]_111355	51.07	5e-76	etc:ETAC_10675	K12519	papD	chaperone protein PapD	--	--

[denovogenes]_111356	50.40	5e-83	smw:SMWW4_v1c06200	K12519	papD	chaperone protein PapD	--	--

[denovogenes]_11136	98.52	0.0	bth:BT_3163	K01187	malZ	alpha-glucosidase	3.2.1.20	Metabolism--Carbohydrate metabolism--Galactose metabolism|Metabolism--Carbohydrate metabolism--Starch and sucrose metabolism|Metabolism--Global and overview maps--Metabolic pathways

[denovogenes]_111360	48.48	1e-60	spi:MGAS10750_Spy1437	K01271	pepQ	Xaa-Pro dipeptidase	3.4.13.9	--

[denovogenes]_111361	88.49	1e-166	ehr:EHR_08655	K02036	pstB	phosphate transport system ATP-binding protein	3.6.3.27	Environmental Information Processing--Membrane transport--ABC transporters

[denovogenes]_111363	100.00	0.0	bfr:BF0925	K06173	truA, PUS1	tRNA pseudouridine38-40 synthase	5.4.99.12	--

[denovogenes]_111364	81.38	9e-144	cbt:CLH_2343	K02823	pyrDII	dihydroorotate dehydrogenase electron transfer subunit	--	Metabolism--Nucleotide metabolism--Pyrimidine metabolism|Metabolism--Global and overview maps--Metabolic pathways

[denovogenes]_111367	73.62	4e-137	ess:ATZ33_10025	K01990	ABC-2.A	ABC-2 type transport system ATP-binding protein	--	--

[denovogenes]_111368	100.00	0.0	bfb:VU15_12835	K06167	phnP	phosphoribosyl 1,2-cyclic phosphate phosphodiesterase	3.1.4.55	Metabolism--Metabolism of other amino acids--Phosphonate and phosphinate metabolism

[denovogenes]_111369	65.35	1e-117	cbv:U729_1178	K01776	murI	glutamate racemase	5.1.1.3	Metabolism--Metabolism of other amino acids--D-Glutamine and D-glutamate metabolism|Metabolism--Global and overview maps--Metabolic pathways

[denovogenes]_111370	99.60	0.0	mmk:MU9_438	K15984	rsmJ	16S rRNA (guanine1516-N2)-methyltransferase	2.1.1.242	--

[denovogenes]_111371	99.60	0.0	mmk:MU9_354	K02444	glpR	DeoR family transcriptional regulator, glycerol-3-phosphate regulon repressor	--	--

[denovogenes]_111372	80.00	2e-144	lacy:A4V08_34255	K00215	dapB	4-hydroxy-tetrahydrodipicolinate reductase	1.17.1.8	Metabolism--Biosynthesis of other secondary metabolites--Monobactam biosynthesis|Metabolism--Amino acid metabolism--Lysine biosynthesis|Metabolism--Global and overview maps--Metabolic pathways|Metabolism--Global and overview maps--Biosynthesis of secondary metabolites|Metabolism--Global and overview maps--Microbial metabolism in diverse environments|Metabolism--Global and overview maps--Biosynthesis of antibiotics|Metabolism--Global and overview maps--Biosynthesis of amino acids

[denovogenes]_111374	41.26	1e-51	pod:PODO_12765	K15599	thiX	putative hydroxymethylpyrimidine transport system permease protein	--	Environmental Information Processing--Membrane transport--ABC transporters

[denovogenes]_111378	81.35	5e-152	csr:Cspa_c57620	K01995	livG	branched-chain amino acid transport system ATP-binding protein	--	Environmental Information Processing--Membrane transport--ABC transporters|Cellular Processes--Cellular community - prokaryotes--Quorum sensing

[denovogenes]_111380	87.70	1e-164	lacy:A4V08_34630	K01995	livG	branched-chain amino acid transport system ATP-binding protein	--	Environmental Information Processing--Membrane transport--ABC transporters|Cellular Processes--Cellular community - prokaryotes--Quorum sensing

[denovogenes]_111381	100.00	0.0	bfr:BF3098	K07273	acm	lysozyme	--	--

[denovogenes]_111382	74.50	2e-139	cbut:ATN24_06495	K02049	ABC.SN.A	NitT/TauT family transport system ATP-binding protein	--	--

[denovogenes]_111383	42.04	8e-54	rix:RO1_19950	K07271	licD	lipopolysaccharide cholinephosphotransferase	2.7.8.-	--

[denovogenes]_111384	100.00	0.0	bfb:VU15_17970	K03439	trmB, METTL1	tRNA (guanine-N7-)-methyltransferase	2.1.1.33	--

[denovogenes]_111385	78.17	7e-142	lsj:LSJ_1725c	K02069	ABC.X2.P	putative ABC transport system permease protein	--	--

[denovogenes]_111387	98.41	0.0	mmk:MU9_927	K06175	truC	tRNA pseudouridine65 synthase	5.4.99.26	--

[denovogenes]_111388	98.41	3e-176	cif:AL515_05885	K02419	fliP	flagellar biosynthetic protein FliP	--	Cellular Processes--Cell motility--Flagellar assembly

[denovogenes]_11139	68.88	0.0	fpr:FP2_14400	K00700	GBE1, glgB	1,4-alpha-glucan branching enzyme	2.4.1.18	Metabolism--Carbohydrate metabolism--Starch and sucrose metabolism|Metabolism--Global and overview maps--Metabolic pathways|Metabolism--Global and overview maps--Biosynthesis of secondary metabolites

[denovogenes]_111391	98.81	0.0	mmk:MU9_699	K03148	thiF	sulfur carrier protein ThiS adenylyltransferase	2.7.7.73	Metabolism--Metabolism of cofactors and vitamins--Thiamine metabolism|Metabolism--Global and overview maps--Metabolic pathways|Genetic Information Processing--Folding, sorting and degradation--Sulfur relay system

[denovogenes]_111395	100.00	0.0	efi:OG1RF_10456	K03436	fruR2, fruR	DeoR family transcriptional regulator, fructose operon transcriptional repressor	--	--

[denovogenes]_111396	73.09	2e-137	cbut:ATN24_06495	K02049	ABC.SN.A	NitT/TauT family transport system ATP-binding protein	--	--

[denovogenes]_111397	62.60	3e-115	cbv:U729_1178	K01776	murI	glutamate racemase	5.1.1.3	Metabolism--Metabolism of other amino acids--D-Glutamine and D-glutamate metabolism|Metabolism--Global and overview maps--Metabolic pathways

[denovogenes]_111398	83.81	1e-153	pdf:CD630DERM_32610	K02036	pstB	phosphate transport system ATP-binding protein	3.6.3.27	Environmental Information Processing--Membrane transport--ABC transporters

[denovogenes]_111399	62.14	2e-107	pdf:CD630DERM_36080	K09013	sufC	Fe-S cluster assembly ATP-binding protein	--	--

[denovogenes]_11140	83.53	0.0	pfre:RM25_0767	K01284	dcp	peptidyl-dipeptidase Dcp	3.4.15.5	--

[denovogenes]_111400	43.36	5e-24	meq:MSWHS_0180	K21029	moeB	molybdopterin-synthase adenylyltransferase	2.7.7.80	Genetic Information Processing--Folding, sorting and degradation--Sulfur relay system

[denovogenes]_111401	81.67	7e-148	cbt:CLH_0843	K03091	SIG3.4	RNA polymerase sporulation-specific sigma factor	--	--

[denovogenes]_111402	50.79	4e-86	eac:EAL2_c12640	K03787	surE	5'-nucleotidase	3.1.3.5	Metabolism--Nucleotide metabolism--Purine metabolism|Metabolism--Nucleotide metabolism--Pyrimidine metabolism|Metabolism--Metabolism of cofactors and vitamins--Nicotinate and nicotinamide metabolism|Metabolism--Global and overview maps--Metabolic pathways|Metabolism--Global and overview maps--Biosynthesis of secondary metabolites

[denovogenes]_111405	57.37	8e-92	csr:Cspa_c58390	K03217	yidC, spoIIIJ, OXA1, ccfA	YidC/Oxa1 family membrane protein insertase	--	Cellular Processes--Cellular community - prokaryotes--Quorum sensing|Genetic Information Processing--Folding, sorting and degradation--Protein export|Environmental Information Processing--Membrane transport--Bacterial secretion system

[denovogenes]_111409	100.00	0.0	efq:DR75_753	K02036	pstB	phosphate transport system ATP-binding protein	3.6.3.27	Environmental Information Processing--Membrane transport--ABC transporters

[denovogenes]_111410	99.60	0.0	sgt:SGGB_1461	K02500	hisF	cyclase	4.1.3.-	Metabolism--Amino acid metabolism--Histidine metabolism|Metabolism--Global and overview maps--Metabolic pathways|Metabolism--Global and overview maps--Biosynthesis of secondary metabolites|Metabolism--Global and overview maps--Biosynthesis of amino acids

[denovogenes]_111411	99.60	0.0	stb:SGPB_0723	K05833	ABC.X4.A	putative ABC transport system ATP-binding protein	--	--

[denovogenes]_111413	85.83	2e-152	pdf:CD630DERM_01170	K00175	korB, oorB, oforB	2-oxoglutarate/2-oxoacid ferredoxin oxidoreductase subunit beta	1.2.7.3 1.2.7.11	Metabolism--Carbohydrate metabolism--Glycolysis / Gluconeogenesis|Metabolism--Carbohydrate metabolism--Citrate cycle (TCA cycle)|Metabolism--Carbohydrate metabolism--Pyruvate metabolism|Metabolism--Carbohydrate metabolism--Butanoate metabolism|Metabolism--Energy metabolism--Carbon fixation pathways in prokaryotes|Metabolism--Global and overview maps--Metabolic pathways|Metabolism--Global and overview maps--Microbial metabolism in diverse environments|Metabolism--Global and overview maps--Biosynthesis of antibiotics|Metabolism--Global and overview maps--Carbon metabolism

[denovogenes]_111414	100.00	0.0	sgt:SGGB_1266	K16960	tcyN	L-cystine transport system ATP-binding protein	3.6.3.-	Environmental Information Processing--Membrane transport--ABC transporters

[denovogenes]_111415	100.00	0.0	ebf:D782_4171	K05781	phnK	putative phosphonate transport system ATP-binding protein	--	--

[denovogenes]_111416	98.41	0.0	eoc:CE10_4809	K06167	phnP	phosphoribosyl 1,2-cyclic phosphate phosphodiesterase	3.1.4.55	Metabolism--Metabolism of other amino acids--Phosphonate and phosphinate metabolism

[denovogenes]_111417	100.00	1e-177	ene:ENT_19210	K02069	ABC.X2.P	putative ABC transport system permease protein	--	--

[denovogenes]_111418	99.60	0.0	elo:EC042_1250	K02013	ABC.FEV.A	iron complex transport system ATP-binding protein	3.6.3.34	Environmental Information Processing--Membrane transport--ABC transporters

[denovogenes]_11142	97.77	0.0	lacy:A4V08_05290	K04079	HSP90A, htpG	molecular chaperone HtpG	--	Genetic Information Processing--Folding, sorting and degradation--Protein processing in endoplasmic reticulum|Environmental Information Processing--Signal transduction--PI3K-Akt signaling pathway|Organismal Systems--Immune system--Antigen processing and presentation|Organismal Systems--Immune system--NOD-like receptor signaling pathway|Organismal Systems--Environmental adaptation--Plant-pathogen interaction|Organismal Systems--Immune system--IL-17 signaling pathway|Organismal Systems--Immune system--Th17 cell differentiation|Organismal Systems--Endocrine system--Progesterone-mediated oocyte maturation|Organismal Systems--Endocrine system--Estrogen signaling pathway|Human Diseases--Cancers: Overview--Pathways in cancer|Human Diseases--Cancers: Specific types--Prostate cancer|Human Diseases--Cardiovascular diseases--Fluid shear stress and atherosclerosis

[denovogenes]_111420	90.48	6e-170	cif:AL515_12095	K00598	tam	trans-aconitate 2-methyltransferase	2.1.1.144	--

[denovogenes]_111421	95.24	0.0	cif:AL515_03225	K06167	phnP	phosphoribosyl 1,2-cyclic phosphate phosphodiesterase	3.1.4.55	Metabolism--Metabolism of other amino acids--Phosphonate and phosphinate metabolism

[denovogenes]_111423	97.20	3e-178	bth:BT_p548231	K03496	parA, soj	chromosome partitioning protein	--	--

[denovogenes]_111424	72.22	6e-133	cle:Clole_2956	K00215	dapB	4-hydroxy-tetrahydrodipicolinate reductase	1.17.1.8	Metabolism--Biosynthesis of other secondary metabolites--Monobactam biosynthesis|Metabolism--Amino acid metabolism--Lysine biosynthesis|Metabolism--Global and overview maps--Metabolic pathways|Metabolism--Global and overview maps--Biosynthesis of secondary metabolites|Metabolism--Global and overview maps--Microbial metabolism in diverse environments|Metabolism--Global and overview maps--Biosynthesis of antibiotics|Metabolism--Global and overview maps--Biosynthesis of amino acids

[denovogenes]_111425	98.81	0.0	sgt:SGGB_0797	K07124	K07124	uncharacterized protein	--	--

[denovogenes]_111427	83.27	1e-157	cbv:U729_2551	K00566	mnmA, trmU	tRNA-uridine 2-sulfurtransferase	2.8.1.13	Genetic Information Processing--Folding, sorting and degradation--Sulfur relay system

[denovogenes]_111428	52.00	1e-82	etc:ETAC_10675	K12519	papD	chaperone protein PapD	--	--

[denovogenes]_111429	55.43	4e-95	csb:CLSA_c06840	K14170	pheA	chorismate mutase / prephenate dehydratase	5.4.99.5 4.2.1.51	Metabolism--Amino acid metabolism--Phenylalanine, tyrosine and tryptophan biosynthesis|Metabolism--Global and overview maps--Metabolic pathways|Metabolism--Global and overview maps--Biosynthesis of secondary metabolites|Metabolism--Global and overview maps--Biosynthesis of antibiotics|Metabolism--Global and overview maps--Biosynthesis of amino acids

[denovogenes]_11143	99.26	0.0	cfd:CFNIH1_17300	K03615	rnfC	electron transport complex protein RnfC	--	--

[denovogenes]_111430	100.00	0.0	stb:SGPB_1004	K02036	pstB	phosphate transport system ATP-binding protein	3.6.3.27	Environmental Information Processing--Membrane transport--ABC transporters

[denovogenes]_111431	99.60	0.0	pvl:AOB99_13340	K00806	uppS	undecaprenyl diphosphate synthase	2.5.1.31	Metabolism--Metabolism of terpenoids and polyketides--Terpenoid backbone biosynthesis|Metabolism--Global and overview maps--Biosynthesis of secondary metabolites

[denovogenes]_111432	100.00	1e-150	pvl:AOB99_13745	K07184	ygiM	SH3 domain protein	--	--

[denovogenes]_111433	60.16	4e-100	ipo:Ilyop_1900	K02428	rdgB	XTP/dITP diphosphohydrolase	3.6.1.66	Metabolism--Nucleotide metabolism--Purine metabolism

[denovogenes]_111434	79.37	1e-146	cbt:CLH_1959	K03481	glvR	RpiR family transcriptional regulator, glv operon transcriptional regulator	--	--

[denovogenes]_111435	99.60	0.0	pvl:AOB99_15880	K02444	glpR	DeoR family transcriptional regulator, glycerol-3-phosphate regulon repressor	--	--

[denovogenes]_111436	100.00	0.0	pvl:AOB99_01925	K00757	udp, UPP	uridine phosphorylase	2.4.2.3	Metabolism--Nucleotide metabolism--Pyrimidine metabolism|Metabolism--Xenobiotics biodegradation and metabolism--Drug metabolism - other enzymes|Metabolism--Global and overview maps--Metabolic pathways

[denovogenes]_111437	64.31	1e-102	pmw:B2K_30030	K10440	rbsC	ribose transport system permease protein	--	Environmental Information Processing--Membrane transport--ABC transporters

[denovogenes]_111439	52.82	1e-86	rto:RTO_15120	K03785	aroD	3-dehydroquinate dehydratase I	4.2.1.10	Metabolism--Amino acid metabolism--Phenylalanine, tyrosine and tryptophan biosynthesis|Metabolism--Global and overview maps--Metabolic pathways|Metabolism--Global and overview maps--Biosynthesis of secondary metabolites|Metabolism--Global and overview maps--Biosynthesis of antibiotics|Metabolism--Global and overview maps--Biosynthesis of amino acids

[denovogenes]_11144	99.55	0.0	cfd:CFNIH1_03950	K03656	rep	ATP-dependent DNA helicase Rep	3.6.4.12	--

[denovogenes]_111440	54.98	5e-85	dor:Desor_2143	K05020	opuD, betL	glycine betaine transporter	--	--

[denovogenes]_111441	40.74	4e-60	ctc:CTC_00119	K01071	MCH	medium-chain acyl-[acyl-carrier-protein] hydrolase	3.1.2.21	Metabolism--Lipid metabolism--Fatty acid biosynthesis|Metabolism--Global and overview maps--Metabolic pathways

[denovogenes]_111442	61.24	6e-56	etd:ETAF_1516	K19303	mepH	murein DD-endopeptidase	3.4.-.-	--

[denovogenes]_111445	74.19	8e-137	vpr:Vpar_0460	K07160	K07160	UPF0271 protein	--	--

[denovogenes]_111446	72.76	2e-130	aoe:Clos_0309	K02588	nifH	nitrogenase iron protein NifH	1.18.6.1	Metabolism--Xenobiotics biodegradation and metabolism--Chloroalkane and chloroalkene degradation|Metabolism--Energy metabolism--Nitrogen metabolism|Metabolism--Global and overview maps--Metabolic pathways|Metabolism--Global and overview maps--Microbial metabolism in diverse environments

[denovogenes]_111447	98.42	0.0	cif:AL515_18570	K01752	E4.3.1.17, sdaA, sdaB, tdcG	L-serine dehydratase	4.3.1.17	Metabolism--Amino acid metabolism--Glycine, serine and threonine metabolism|Metabolism--Amino acid metabolism--Cysteine and methionine metabolism|Metabolism--Global and overview maps--Metabolic pathways|Metabolism--Global and overview maps--Biosynthesis of secondary metabolites|Metabolism--Global and overview maps--Biosynthesis of antibiotics|Metabolism--Global and overview maps--Carbon metabolism|Metabolism--Global and overview maps--Biosynthesis of amino acids

[denovogenes]_111450	100.00	0.0	esl:O3K_23410	K06167	phnP	phosphoribosyl 1,2-cyclic phosphate phosphodiesterase	3.1.4.55	Metabolism--Metabolism of other amino acids--Phosphonate and phosphinate metabolism

[denovogenes]_111451	99.20	1e-176	tur:AT726_04715	K19310	bcrB	bacitracin transport system permease protein	--	Environmental Information Processing--Membrane transport--ABC transporters

[denovogenes]_111452	93.65	6e-179	cif:AL515_10795	K03478	chbG	chitin disaccharide deacetylase	3.5.1.105	--

[denovogenes]_111453	77.29	7e-144	cpf:CPF_2682	K16787	ecfA2	energy-coupling factor transport system ATP-binding protein	3.6.3.-	Environmental Information Processing--Membrane transport--ABC transporters

[denovogenes]_111456	64.66	2e-117	cdl:CDR20291_1199	K03769	ppiC	peptidyl-prolyl cis-trans isomerase C	5.2.1.8	--

[denovogenes]_111458	99.60	0.0	pmib:BB2000_0062	K01126	E3.1.4.46, glpQ, ugpQ	glycerophosphoryl diester phosphodiesterase	3.1.4.46	Metabolism--Lipid metabolism--Glycerophospholipid metabolism

[denovogenes]_111459	78.57	8e-144	cle:Clole_3343	K01409	KAE1, tsaD, QRI7	N6-L-threonylcarbamoyladenine synthase	2.3.1.234	--

[denovogenes]_111460	47.23	3e-64	bcl:ABC3165	K16698	tuaG	teichuronic acid biosynthesis glycosyltransferase TuaG	2.4.-.-	--

[denovogenes]_111461	54.44	5e-56	ctx:Clo1313_2373	K07451	mcrA	5-methylcytosine-specific restriction enzyme A	3.1.21.-	--

[denovogenes]_111462	76.28	2e-135	csb:CLSA_c31290	K02500	hisF	cyclase	4.1.3.-	Metabolism--Amino acid metabolism--Histidine metabolism|Metabolism--Global and overview maps--Metabolic pathways|Metabolism--Global and overview maps--Biosynthesis of secondary metabolites|Metabolism--Global and overview maps--Biosynthesis of amino acids

[denovogenes]_111464	99.60	6e-173	cbut:ATN24_19960	K02057	ABC.SS.P	simple sugar transport system permease protein	--	--

[denovogenes]_111467	79.84	4e-145	csr:Cspa_c51390	K09786	K09786	uncharacterized protein	--	--

[denovogenes]_111468	50.88	7e-79	cace:CACET_c30480	K07098	K07098	uncharacterized protein	--	--

[denovogenes]_111470	60.34	2e-102	eac:EAL2_c09600	K09758	asdA	aspartate 4-decarboxylase	4.1.1.12	Metabolism--Amino acid metabolism--Alanine, aspartate and glutamate metabolism|Metabolism--Amino acid metabolism--Cysteine and methionine metabolism|Metabolism--Global and overview maps--Metabolic pathways|Metabolism--Global and overview maps--Biosynthesis of amino acids

[denovogenes]_111473	94.86	6e-180	cfd:CFNIH1_05750	K03276	waaR, waaT, rfaJ	UDP-glucose/galactose:(glucosyl)LPS alpha-1,2-glucosyl/galactosyltransferase	2.4.1.-	Metabolism--Glycan biosynthesis and metabolism--Lipopolysaccharide biosynthesis|Metabolism--Global and overview maps--Metabolic pathways

[denovogenes]_111474	93.65	1e-173	prg:RB151_002600	K02444	glpR	DeoR family transcriptional regulator, glycerol-3-phosphate regulon repressor	--	--

[denovogenes]_111475	71.89	9e-129	cbut:ATN24_01945	K09685	purR	purine operon repressor	--	--

[denovogenes]_111476	80.95	3e-148	pdf:CD630DERM_36730	K03497	parB, spo0J	chromosome partitioning protein, ParB family	--	--

[denovogenes]_111477	100.00	0.0	shq:A0259_04715	K00806	uppS	undecaprenyl diphosphate synthase	2.5.1.31	Metabolism--Metabolism of terpenoids and polyketides--Terpenoid backbone biosynthesis|Metabolism--Global and overview maps--Biosynthesis of secondary metabolites

[denovogenes]_111478	46.61	1e-78	ava:Ava_2888	K05886	sdh	serine 3-dehydrogenase (NADP+)	1.1.1.276	--

[denovogenes]_111479	82.66	4e-149	clt:CM240_0122	K02028	ABC.PA.A	polar amino acid transport system ATP-binding protein	3.6.3.21	--

[denovogenes]_11148	67.25	0.0	bxy:BXY_12870	K01322	PREP	prolyl oligopeptidase	3.4.21.26	Organismal Systems--Endocrine system--Renin-angiotensin system

[denovogenes]_111480	57.87	4e-96	eno:ECENHK_04190	K07346	fimC	fimbrial chaperone protein	--	--

[denovogenes]_111482	55.51	6e-91	mhg:MHY_22610	K01338	lon	ATP-dependent Lon protease	3.4.21.53	Cellular Processes--Cell growth and death--Cell cycle - Caulobacter

[denovogenes]_111483	73.41	9e-137	vpr:Vpar_0304	K02028	ABC.PA.A	polar amino acid transport system ATP-binding protein	3.6.3.21	--

[denovogenes]_111484	40.51	2e-60	str:Sterm_1070	K02444	glpR	DeoR family transcriptional regulator, glycerol-3-phosphate regulon repressor	--	--

[denovogenes]_111485	61.25	4e-103	pdf:CD630DERM_36080	K09013	sufC	Fe-S cluster assembly ATP-binding protein	--	--

[denovogenes]_111486	88.84	2e-146	cif:AL515_11865	K07312	ynfH	Tat-targeted selenate reductase subunit YnfH	--	--

[denovogenes]_111488	76.40	7e-139	cbv:U729_1434	K03151	thiI	thiamine biosynthesis protein ThiI	--	Metabolism--Metabolism of cofactors and vitamins--Thiamine metabolism|Metabolism--Global and overview maps--Metabolic pathways|Genetic Information Processing--Folding, sorting and degradation--Sulfur relay system

[denovogenes]_111489	77.47	2e-137	fne:FSDG_02008	K13922	--	--	--	Metabolism--Carbohydrate metabolism--Propanoate metabolism

[denovogenes]_11149	99.85	0.0	pdi:BDI_2264	K03043	rpoB	DNA-directed RNA polymerase subunit beta	2.7.7.6	Metabolism--Nucleotide metabolism--Purine metabolism|Metabolism--Nucleotide metabolism--Pyrimidine metabolism|Metabolism--Global and overview maps--Metabolic pathways|Genetic Information Processing--Transcription--RNA polymerase

[denovogenes]_111490	75.82	3e-130	dsy:DSY0105	K09816	znuB	zinc transport system permease protein	--	Environmental Information Processing--Membrane transport--ABC transporters

[denovogenes]_111491	75.00	1e-134	vpr:Vpar_0129	K02500	hisF	cyclase	4.1.3.-	Metabolism--Amino acid metabolism--Histidine metabolism|Metabolism--Global and overview maps--Metabolic pathways|Metabolism--Global and overview maps--Biosynthesis of secondary metabolites|Metabolism--Global and overview maps--Biosynthesis of amino acids

[denovogenes]_111493	48.25	3e-66	cni:Calni_0665	K07462	recJ	single-stranded-DNA-specific exonuclease	3.1.-.-	Genetic Information Processing--Replication and repair--Base excision repair|Genetic Information Processing--Replication and repair--Mismatch repair|Genetic Information Processing--Replication and repair--Homologous recombination

[denovogenes]_111494	86.06	2e-162	fne:FSDG_00506	K05936	cobM, cbiF	precorrin-4/cobalt-precorrin-4 C11-methyltransferase	2.1.1.133 2.1.1.271	Metabolism--Metabolism of cofactors and vitamins--Porphyrin and chlorophyll metabolism|Metabolism--Global and overview maps--Metabolic pathways

[denovogenes]_111497	98.41	0.0	cfd:CFNIH1_21965	K00480	E1.14.13.1	salicylate hydroxylase	1.14.13.1	Metabolism--Xenobiotics biodegradation and metabolism--Dioxin degradation|Metabolism--Xenobiotics biodegradation and metabolism--Polycyclic aromatic hydrocarbon degradation|Metabolism--Xenobiotics biodegradation and metabolism--Naphthalene degradation|Metabolism--Global and overview maps--Metabolic pathways|Metabolism--Global and overview maps--Microbial metabolism in diverse environments|Metabolism--Global and overview maps--Degradation of aromatic compounds

[denovogenes]_111498	76.40	1e-145	prg:RB151_002860	K01467	ampC	beta-lactamase class C	3.5.2.6	Human Diseases--Drug resistance: Antimicrobial--beta-Lactam resistance|Environmental Information Processing--Signal transduction--Two-component system

[denovogenes]_11150	66.97	0.0	paeq:R50912_05515	K01811	xylS, yicI	alpha-D-xyloside xylohydrolase	3.2.1.177	--

[denovogenes]_111500	44.49	6e-70	deh:cbdbA122	K03605	hyaD, hybD	hydrogenase maturation protease	3.4.23.-	--

[denovogenes]_111501	59.35	2e-92	cpsb:B595_0534	K03230	yscV, sctV, hrcV	type III secretion protein V	--	Environmental Information Processing--Membrane transport--Bacterial secretion system

[denovogenes]_111504	100.00	0.0	pvl:AOB99_08745	K14058	ttcA	tRNA 2-thiocytidine biosynthesis protein TtcA	--	--

[denovogenes]_111505	71.54	1e-120	cbt:CLH_3071	K06148	ABCC-BAC	ATP-binding cassette, subfamily C, bacterial	--	--

[denovogenes]_111509	75.00	6e-142	csr:Cspa_c52680	K01223	E3.2.1.86B, bglA	6-phospho-beta-glucosidase	3.2.1.86	Metabolism--Carbohydrate metabolism--Glycolysis / Gluconeogenesis|Metabolism--Carbohydrate metabolism--Starch and sucrose metabolism

[denovogenes]_111512	78.26	5e-143	fnt:HMPREF0405_00161	K03076	secY	preprotein translocase subunit SecY	--	Cellular Processes--Cellular community - prokaryotes--Quorum sensing|Genetic Information Processing--Folding, sorting and degradation--Protein export|Environmental Information Processing--Membrane transport--Bacterial secretion system

[denovogenes]_111514	83.33	6e-157	cbt:CLH_2556	K10254	ohyA, sph	oleate hydratase	4.2.1.53	--

[denovogenes]_111517	66.01	2e-121	mbf:BHV42_02250	K00833	bioA	adenosylmethionine---8-amino-7-oxononanoate aminotransferase	2.6.1.62	Metabolism--Metabolism of cofactors and vitamins--Biotin metabolism|Metabolism--Global and overview maps--Metabolic pathways

[denovogenes]_111518	98.81	1e-180	ecoh:ECRM13516_2940	K00111	glpA, glpD	glycerol-3-phosphate dehydrogenase	1.1.5.3	Metabolism--Lipid metabolism--Glycerophospholipid metabolism|Metabolism--Global and overview maps--Biosynthesis of secondary metabolites

[denovogenes]_111523	98.42	2e-180	cbut:ATN24_14125	K13542	cobA-hemD	uroporphyrinogen III methyltransferase / synthase	2.1.1.107 4.2.1.75	Metabolism--Metabolism of cofactors and vitamins--Porphyrin and chlorophyll metabolism|Metabolism--Global and overview maps--Metabolic pathways|Metabolism--Global and overview maps--Biosynthesis of secondary metabolites

[denovogenes]_111524	99.21	0.0	cfd:CFNIH1_04430	K11747	kefB	glutathione-regulated potassium-efflux system protein KefB	--	--

[denovogenes]_111525	84.58	4e-158	cbut:ATN24_15415	K01639	E4.1.3.3, nanA, NPL	N-acetylneuraminate lyase	4.1.3.3	Metabolism--Carbohydrate metabolism--Amino sugar and nucleotide sugar metabolism

[denovogenes]_111527	90.91	1e-161	cpf:CPF_2573	K04077	groEL, HSPD1	chaperonin GroEL	--	Genetic Information Processing--Folding, sorting and degradation--RNA degradation|Organismal Systems--Aging--Longevity regulating pathway - worm|Human Diseases--Endocrine and metabolic diseases--Type I diabetes mellitus|Human Diseases--Infectious diseases: Bacterial--Legionellosis|Human Diseases--Infectious diseases: Bacterial--Tuberculosis

[denovogenes]_111529	80.24	6e-143	cbv:U729_905	K02803	PTS-Nag-EIIB, nagE	PTS system, N-acetylglucosamine-specific IIB component	2.7.1.193	Metabolism--Carbohydrate metabolism--Amino sugar and nucleotide sugar metabolism|Environmental Information Processing--Membrane transport--Phosphotransferase system (PTS)

[denovogenes]_111529	80.24	6e-143	cbv:U729_905	K02804	PTS-Nag-EIIC, nagE	PTS system, N-acetylglucosamine-specific IIC component	--	Metabolism--Carbohydrate metabolism--Amino sugar and nucleotide sugar metabolism|Environmental Information Processing--Membrane transport--Phosphotransferase system (PTS)

[denovogenes]_111530	92.09	1e-155	esl:O3K_04885	K01847	MUT	methylmalonyl-CoA mutase	5.4.99.2	Metabolism--Amino acid metabolism--Valine, leucine and isoleucine degradation|Metabolism--Carbohydrate metabolism--Glyoxylate and dicarboxylate metabolism|Metabolism--Carbohydrate metabolism--Propanoate metabolism|Metabolism--Energy metabolism--Carbon fixation pathways in prokaryotes|Metabolism--Global and overview maps--Metabolic pathways|Metabolism--Global and overview maps--Microbial metabolism in diverse environments|Metabolism--Global and overview maps--Carbon metabolism

[denovogenes]_111536	88.10	2e-170	bfs:BF9343_2897	K00655	plsC	1-acyl-sn-glycerol-3-phosphate acyltransferase	2.3.1.51	Metabolism--Lipid metabolism--Glycerolipid metabolism|Metabolism--Lipid metabolism--Glycerophospholipid metabolism|Metabolism--Global and overview maps--Metabolic pathways|Metabolism--Global and overview maps--Biosynthesis of secondary metabolites

[denovogenes]_111538	87.70	3e-168	bfb:VU15_17970	K03439	trmB, METTL1	tRNA (guanine-N7-)-methyltransferase	2.1.1.33	--

[denovogenes]_111539	100.00	0.0	bxy:BXY_30400	K01803	TPI, tpiA	triosephosphate isomerase (TIM)	5.3.1.1	Metabolism--Carbohydrate metabolism--Glycolysis / Gluconeogenesis|Metabolism--Carbohydrate metabolism--Fructose and mannose metabolism|Metabolism--Carbohydrate metabolism--Inositol phosphate metabolism|Metabolism--Energy metabolism--Carbon fixation in photosynthetic organisms|Metabolism--Global and overview maps--Metabolic pathways|Metabolism--Global and overview maps--Biosynthesis of secondary metabolites|Metabolism--Global and overview maps--Microbial metabolism in diverse environments|Metabolism--Global and overview maps--Biosynthesis of antibiotics|Metabolism--Global and overview maps--Carbon metabolism|Metabolism--Global and overview maps--Biosynthesis of amino acids

[denovogenes]_111541	60.42	3e-102	afn:Acfer_1700	K02065	mlaF, linL, mkl	phospholipid/cholesterol/gamma-HCH transport system ATP-binding protein	--	Environmental Information Processing--Membrane transport--ABC transporters

[denovogenes]_111543	69.26	3e-134	rto:RTO_24620	K00554	trmD	tRNA (guanine37-N1)-methyltransferase	2.1.1.228	--

[denovogenes]_111544	77.29	2e-141	sri:SELR_15790	K02500	hisF	cyclase	4.1.3.-	Metabolism--Amino acid metabolism--Histidine metabolism|Metabolism--Global and overview maps--Metabolic pathways|Metabolism--Global and overview maps--Biosynthesis of secondary metabolites|Metabolism--Global and overview maps--Biosynthesis of amino acids

[denovogenes]_111546	59.31	5e-97	rob:CK5_17790	K04477	ycdX	putative hydrolase	--	--

[denovogenes]_111547	53.44	1e-81	cls:CXIVA_17720	K03436	fruR2, fruR	DeoR family transcriptional regulator, fructose operon transcriptional repressor	--	--

[denovogenes]_111548	99.60	0.0	bvu:BVU_3328	K00979	kdsB	3-deoxy-manno-octulosonate cytidylyltransferase (CMP-KDO synthetase)	2.7.7.38	Metabolism--Glycan biosynthesis and metabolism--Lipopolysaccharide biosynthesis|Metabolism--Global and overview maps--Metabolic pathways

[denovogenes]_111549	40.37	2e-10	cbx:Cenrod_0909	K03286	TC.OOP	OmpA-OmpF porin, OOP family	--	--

[denovogenes]_111550	96.83	0.0	bxy:BXY_42370	K00655	plsC	1-acyl-sn-glycerol-3-phosphate acyltransferase	2.3.1.51	Metabolism--Lipid metabolism--Glycerolipid metabolism|Metabolism--Lipid metabolism--Glycerophospholipid metabolism|Metabolism--Global and overview maps--Metabolic pathways|Metabolism--Global and overview maps--Biosynthesis of secondary metabolites

[denovogenes]_111551	96.39	0.0	bvu:BVU_1577	K03427	hsdM	type I restriction enzyme M protein	2.1.1.72	--

[denovogenes]_111552	85.43	9e-156	bhl:Bache_1563	K06201	cutC	copper homeostasis protein	--	--

[denovogenes]_111553	76.31	2e-143	fpla:A4U99_18005	K01142	E3.1.11.2, xthA	exodeoxyribonuclease III	3.1.11.2	Genetic Information Processing--Replication and repair--Base excision repair

[denovogenes]_111556	89.64	7e-164	bprs:CK3_14050	K07160	K07160	UPF0271 protein	--	--

[denovogenes]_111558	96.83	0.0	bxy:BXY_10700	K03524	birA	BirA family transcriptional regulator, biotin operon repressor / biotin-[acetyl-CoA-carboxylase] ligase	6.3.4.15	Metabolism--Metabolism of cofactors and vitamins--Biotin metabolism|Metabolism--Global and overview maps--Metabolic pathways

[denovogenes]_111559	58.73	8e-98	csh:Closa_3458	K02557	motB	chemotaxis protein MotB	--	Cellular Processes--Cell motility--Bacterial chemotaxis|Cellular Processes--Cell motility--Flagellar assembly

[denovogenes]_11156	97.92	0.0	boa:Bovatus_04529	K01284	dcp	peptidyl-dipeptidase Dcp	3.4.15.5	--

[denovogenes]_111560	80.16	7e-146	bprm:CL3_08650	K06898	K06898	uncharacterized protein	--	--

[denovogenes]_111561	98.81	0.0	boa:Bovatus_01340	K02036	pstB	phosphate transport system ATP-binding protein	3.6.3.27	Environmental Information Processing--Membrane transport--ABC transporters

[denovogenes]_111563	99.60	0.0	btho:Btheta7330_00522	K01803	TPI, tpiA	triosephosphate isomerase (TIM)	5.3.1.1	Metabolism--Carbohydrate metabolism--Glycolysis / Gluconeogenesis|Metabolism--Carbohydrate metabolism--Fructose and mannose metabolism|Metabolism--Carbohydrate metabolism--Inositol phosphate metabolism|Metabolism--Energy metabolism--Carbon fixation in photosynthetic organisms|Metabolism--Global and overview maps--Metabolic pathways|Metabolism--Global and overview maps--Biosynthesis of secondary metabolites|Metabolism--Global and overview maps--Microbial metabolism in diverse environments|Metabolism--Global and overview maps--Biosynthesis of antibiotics|Metabolism--Global and overview maps--Carbon metabolism|Metabolism--Global and overview maps--Biosynthesis of amino acids

[denovogenes]_111565	76.80	1e-141	era:ERE_08050	K00215	dapB	4-hydroxy-tetrahydrodipicolinate reductase	1.17.1.8	Metabolism--Biosynthesis of other secondary metabolites--Monobactam biosynthesis|Metabolism--Amino acid metabolism--Lysine biosynthesis|Metabolism--Global and overview maps--Metabolic pathways|Metabolism--Global and overview maps--Biosynthesis of secondary metabolites|Metabolism--Global and overview maps--Microbial metabolism in diverse environments|Metabolism--Global and overview maps--Biosynthesis of antibiotics|Metabolism--Global and overview maps--Biosynthesis of amino acids

[denovogenes]_111566	55.38	3e-101	ava:Ava_2888	K05886	sdh	serine 3-dehydrogenase (NADP+)	1.1.1.276	--

[denovogenes]_111567	87.34	2e-148	rto:RTO_23810	K01788	nanE	N-acylglucosamine-6-phosphate 2-epimerase	5.1.3.9	Metabolism--Carbohydrate metabolism--Amino sugar and nucleotide sugar metabolism

[denovogenes]_111569	100.00	0.0	bvu:BVU_0267	K03439	trmB, METTL1	tRNA (guanine-N7-)-methyltransferase	2.1.1.33	--

[denovogenes]_11157	97.33	0.0	bdh:GV66_05875	K08676	tri	tricorn protease	3.4.21.-	--

[denovogenes]_111570	46.40	1e-29	tau:Tola_2365	K00992	murU	MurNAc alpha-1-phosphate uridylyltransferase	2.7.7.-	Metabolism--Carbohydrate metabolism--Amino sugar and nucleotide sugar metabolism|Metabolism--Global and overview maps--Metabolic pathways

[denovogenes]_111571	49.39	2e-77	cpy:Cphy_1107	K02233	E2.7.8.26, cobS, cobV	adenosylcobinamide-GDP ribazoletransferase	2.7.8.26	Metabolism--Metabolism of cofactors and vitamins--Porphyrin and chlorophyll metabolism|Metabolism--Global and overview maps--Metabolic pathways

[denovogenes]_111572	100.00	0.0	ash:AL1_19730	K02013	ABC.FEV.A	iron complex transport system ATP-binding protein	3.6.3.34	Environmental Information Processing--Membrane transport--ABC transporters

[denovogenes]_111573	51.60	2e-87	rto:RTO_03950	K03394	cobI-cbiL	precorrin-2/cobalt-factor-2 C20-methyltransferase	2.1.1.130 2.1.1.151	Metabolism--Metabolism of cofactors and vitamins--Porphyrin and chlorophyll metabolism|Metabolism--Global and overview maps--Metabolic pathways

[denovogenes]_111574	64.37	1e-109	csq:CSCA_0563	K02007	cbiM	cobalt/nickel transport system permease protein	--	Environmental Information Processing--Membrane transport--ABC transporters

[denovogenes]_111575	66.67	5e-121	cle:Clole_0360	K02003	ABC.CD.A	putative ABC transport system ATP-binding protein	--	--

[denovogenes]_111576	96.83	0.0	bxy:BXY_30080	K03439	trmB, METTL1	tRNA (guanine-N7-)-methyltransferase	2.1.1.33	--

[denovogenes]_111577	62.67	1e-104	rbr:RBR_09990	K00554	trmD	tRNA (guanine37-N1)-methyltransferase	2.1.1.228	--

[denovogenes]_111578	50.00	6e-81	gtn:GTNG_0300	K11635	yxdL	putative ABC transport system ATP-binding protein	--	Environmental Information Processing--Signal transduction--Two-component system

[denovogenes]_111579	85.32	3e-158	bprm:CL3_31910	K01995	livG	branched-chain amino acid transport system ATP-binding protein	--	Environmental Information Processing--Membrane transport--ABC transporters|Cellular Processes--Cellular community - prokaryotes--Quorum sensing

[denovogenes]_11158	58.23	0.0	cbut:ATN24_07385	K12132	prkC, stkP	eukaryotic-like serine/threonine-protein kinase	2.7.11.1	--

[denovogenes]_111581	44.58	3e-74	cpe:CPE0084	K06608	iolR	DeoR family transcriptional regulator, myo-inositol catabolism operon repressor	--	--

[denovogenes]_111582	90.48	9e-171	boa:Bovatus_01400	K06167	phnP	phosphoribosyl 1,2-cyclic phosphate phosphodiesterase	3.1.4.55	Metabolism--Metabolism of other amino acids--Phosphonate and phosphinate metabolism

[denovogenes]_111583	64.92	1e-112	elm:ELI_2815	K02010	afuC, fbpC	iron(III) transport system ATP-binding protein	3.6.3.30	Environmental Information Processing--Membrane transport--ABC transporters

[denovogenes]_111586	100.00	0.0	bxy:BXY_30080	K03439	trmB, METTL1	tRNA (guanine-N7-)-methyltransferase	2.1.1.33	--

[denovogenes]_111587	56.18	1e-90	aoe:Clos_0031	K10795	prdD	D-proline reductase (dithiol) PrdD	1.21.4.1	Metabolism--Amino acid metabolism--Arginine and proline metabolism

[denovogenes]_111588	99.21	0.0	boa:Bovatus_01340	K02036	pstB	phosphate transport system ATP-binding protein	3.6.3.27	Environmental Information Processing--Membrane transport--ABC transporters

[denovogenes]_111589	100.00	0.0	bvu:BVU_2255	K00655	plsC	1-acyl-sn-glycerol-3-phosphate acyltransferase	2.3.1.51	Metabolism--Lipid metabolism--Glycerolipid metabolism|Metabolism--Lipid metabolism--Glycerophospholipid metabolism|Metabolism--Global and overview maps--Metabolic pathways|Metabolism--Global and overview maps--Biosynthesis of secondary metabolites

[denovogenes]_11159	99.55	0.0	mmk:MU9_2867	K01972	E6.5.1.2, ligA, ligB	DNA ligase (NAD+)	6.5.1.2	Genetic Information Processing--Replication and repair--DNA replication|Genetic Information Processing--Replication and repair--Base excision repair|Genetic Information Processing--Replication and repair--Nucleotide excision repair|Genetic Information Processing--Replication and repair--Mismatch repair

[denovogenes]_111591	100.00	0.0	bvu:BVU_2613	K01142	E3.1.11.2, xthA	exodeoxyribonuclease III	3.1.11.2	Genetic Information Processing--Replication and repair--Base excision repair

[denovogenes]_111593	94.44	9e-179	bvu:BVU_0267	K03439	trmB, METTL1	tRNA (guanine-N7-)-methyltransferase	2.1.1.33	--

[denovogenes]_111594	99.60	0.0	ecol:LY180_04425	K11534	deoR	DeoR family transcriptional regulator, deoxyribose operon repressor	--	--

[denovogenes]_111596	53.47	2e-89	rim:ROI_36120	K06173	truA, PUS1	tRNA pseudouridine38-40 synthase	5.4.99.12	--

[denovogenes]_111597	71.19	2e-125	cpy:Cphy_3742	K02108	ATPF0A, atpB	F-type H+-transporting ATPase subunit a	--	Metabolism--Energy metabolism--Oxidative phosphorylation|Metabolism--Energy metabolism--Photosynthesis|Metabolism--Global and overview maps--Metabolic pathways

[denovogenes]_11160	76.84	0.0	ega:AL523_05265	K02817	PTS-Tre-EIIA, treP	PTS system, trehalose-specific IIA component	2.7.1.201	Metabolism--Carbohydrate metabolism--Starch and sucrose metabolism|Environmental Information Processing--Membrane transport--Phosphotransferase system (PTS)

[denovogenes]_11160	76.84	0.0	ega:AL523_05265	K02818	PTS-Tre-EIIB, treB	PTS system, trehalose-specific IIB component	2.7.1.201	Metabolism--Carbohydrate metabolism--Starch and sucrose metabolism|Environmental Information Processing--Membrane transport--Phosphotransferase system (PTS)

[denovogenes]_11160	76.84	0.0	ega:AL523_05265	K02819	PTS-Tre-EIIC, treB	PTS system, trehalose-specific IIC component	--	Metabolism--Carbohydrate metabolism--Starch and sucrose metabolism|Environmental Information Processing--Membrane transport--Phosphotransferase system (PTS)

[denovogenes]_111600	99.21	0.0	btho:Btheta7330_01900	K02036	pstB	phosphate transport system ATP-binding protein	3.6.3.27	Environmental Information Processing--Membrane transport--ABC transporters

[denovogenes]_111601	95.24	2e-180	boa:Bovatus_01400	K06167	phnP	phosphoribosyl 1,2-cyclic phosphate phosphodiesterase	3.1.4.55	Metabolism--Metabolism of other amino acids--Phosphonate and phosphinate metabolism

[denovogenes]_111602	73.79	3e-132	esr:ES1_17500	K03702	uvrB	excinuclease ABC subunit B	--	Genetic Information Processing--Replication and repair--Nucleotide excision repair

[denovogenes]_111607	60.57	6e-96	abao:BEQ56_08555	K02795	PTS-Man-EIIC, manY	PTS system, mannose-specific IIC component	--	Metabolism--Carbohydrate metabolism--Fructose and mannose metabolism|Metabolism--Carbohydrate metabolism--Amino sugar and nucleotide sugar metabolism|Metabolism--Global and overview maps--Metabolic pathways|Environmental Information Processing--Membrane transport--Phosphotransferase system (PTS)

[denovogenes]_111608	49.37	7e-76	nth:Nther_1461	K01358	clpP, CLPP	ATP-dependent Clp protease, protease subunit	3.4.21.92	Cellular Processes--Cell growth and death--Cell cycle - Caulobacter|Organismal Systems--Aging--Longevity regulating pathway - worm

[denovogenes]_111609	76.19	4e-121	csh:Closa_3799	K15669	--	--	--	Metabolism--Glycan biosynthesis and metabolism--Lipopolysaccharide biosynthesis

[denovogenes]_11161	45.91	0.0	bce:BC3480	K07456	mutS2	DNA mismatch repair protein MutS2	--	Genetic Information Processing--Replication and repair--Mismatch repair

[denovogenes]_111610	100.00	0.0	bxy:BXY_42370	K00655	plsC	1-acyl-sn-glycerol-3-phosphate acyltransferase	2.3.1.51	Metabolism--Lipid metabolism--Glycerolipid metabolism|Metabolism--Lipid metabolism--Glycerophospholipid metabolism|Metabolism--Global and overview maps--Metabolic pathways|Metabolism--Global and overview maps--Biosynthesis of secondary metabolites

[denovogenes]_111611	64.68	2e-116	paeq:R50912_13525	K02003	ABC.CD.A	putative ABC transport system ATP-binding protein	--	--

[denovogenes]_111613	85.37	1e-153	fpla:A4U99_01915	K01265	map	methionyl aminopeptidase	3.4.11.18	--

[denovogenes]_111615	96.03	6e-179	spq:SPAB_05152	K03148	thiF	sulfur carrier protein ThiS adenylyltransferase	2.7.7.73	Metabolism--Metabolism of cofactors and vitamins--Thiamine metabolism|Metabolism--Global and overview maps--Metabolic pathways|Genetic Information Processing--Folding, sorting and degradation--Sulfur relay system

[denovogenes]_111616	56.80	4e-100	ava:Ava_2888	K05886	sdh	serine 3-dehydrogenase (NADP+)	1.1.1.276	--

[denovogenes]_111617	55.60	2e-75	sbu:SpiBuddy_2174	K00865	glxK, garK	glycerate 2-kinase	2.7.1.165	Metabolism--Amino acid metabolism--Glycine, serine and threonine metabolism|Metabolism--Lipid metabolism--Glycerolipid metabolism|Metabolism--Carbohydrate metabolism--Glyoxylate and dicarboxylate metabolism|Metabolism--Global and overview maps--Metabolic pathways|Metabolism--Global and overview maps--Microbial metabolism in diverse environments|Metabolism--Global and overview maps--Biosynthesis of antibiotics

[denovogenes]_11162	87.12	0.0	eum:ECUMN_1305	K10906	recE	exodeoxyribonuclease VIII	3.1.11.-	--

[denovogenes]_111620	82.87	3e-150	sans:DK43_09675	K02025	ABC.MS.P	multiple sugar transport system permease protein	--	--

[denovogenes]_111623	73.41	8e-145	clb:Clo1100_2510	K02036	pstB	phosphate transport system ATP-binding protein	3.6.3.27	Environmental Information Processing--Membrane transport--ABC transporters

[denovogenes]_111624	43.03	1e-64	clj:CLJU_c37420	K20491	nisE, spaE, cprB, epiE	lantibiotic transport system permease protein	--	Environmental Information Processing--Membrane transport--ABC transporters|Environmental Information Processing--Signal transduction--Two-component system|Cellular Processes--Cellular community - prokaryotes--Quorum sensing

[denovogenes]_111628	100.00	0.0	cif:AL515_14215	K02557	motB	chemotaxis protein MotB	--	Cellular Processes--Cell motility--Bacterial chemotaxis|Cellular Processes--Cell motility--Flagellar assembly

[denovogenes]_11163	74.85	0.0	cdg:CDBI1_16230	K01972	E6.5.1.2, ligA, ligB	DNA ligase (NAD+)	6.5.1.2	Genetic Information Processing--Replication and repair--DNA replication|Genetic Information Processing--Replication and repair--Base excision repair|Genetic Information Processing--Replication and repair--Nucleotide excision repair|Genetic Information Processing--Replication and repair--Mismatch repair

[denovogenes]_111631	58.80	6e-96	ddh:Desde_3912	K02050	ABC.SN.P	NitT/TauT family transport system permease protein	--	--

[denovogenes]_111633	61.70	1e-106	rob:CK5_22060	K12410	npdA	NAD-dependent deacetylase	3.5.1.-	--

[denovogenes]_111634	60.80	1e-116	amt:Amet_3994	K03496	parA, soj	chromosome partitioning protein	--	--

[denovogenes]_111635	99.60	0.0	cif:AL515_04210	K06169	miaE	tRNA-(ms[2]io[6]A)-hydroxylase	1.-.-.-	--

[denovogenes]_111636	66.67	1e-119	paeh:H70357_22465	K02028	ABC.PA.A	polar amino acid transport system ATP-binding protein	3.6.3.21	--

[denovogenes]_111639	70.42	5e-126	sbr:SY1_19380	K00175	korB, oorB, oforB	2-oxoglutarate/2-oxoacid ferredoxin oxidoreductase subunit beta	1.2.7.3 1.2.7.11	Metabolism--Carbohydrate metabolism--Glycolysis / Gluconeogenesis|Metabolism--Carbohydrate metabolism--Citrate cycle (TCA cycle)|Metabolism--Carbohydrate metabolism--Pyruvate metabolism|Metabolism--Carbohydrate metabolism--Butanoate metabolism|Metabolism--Energy metabolism--Carbon fixation pathways in prokaryotes|Metabolism--Global and overview maps--Metabolic pathways|Metabolism--Global and overview maps--Microbial metabolism in diverse environments|Metabolism--Global and overview maps--Biosynthesis of antibiotics|Metabolism--Global and overview maps--Carbon metabolism

[denovogenes]_11164	55.26	0.0	cbz:Cbs_1900	K18149	pbp5, pbpC, pbp3	penicillin-binding protein	--	Human Diseases--Drug resistance: Antimicrobial--beta-Lactam resistance

[denovogenes]_111641	80.65	3e-153	eel:EUBELI_00471	K02036	pstB	phosphate transport system ATP-binding protein	3.6.3.27	Environmental Information Processing--Membrane transport--ABC transporters

[denovogenes]_111642	42.17	4e-60	csh:Closa_0046	K03710	K03710	GntR family transcriptional regulator	--	--

[denovogenes]_111643	40.71	1e-50	dhd:Dhaf_3871	K20460	nukE, mcdE, sboE	lantibiotic transport system permease protein	--	Environmental Information Processing--Membrane transport--ABC transporters

[denovogenes]_111645	41.96	2e-11	ova:OBV_03840	K07726	K07726	putative transcriptional regulator	--	--

[denovogenes]_111646	98.41	0.0	cfd:CFNIH1_10550	K00806	uppS	undecaprenyl diphosphate synthase	2.5.1.31	Metabolism--Metabolism of terpenoids and polyketides--Terpenoid backbone biosynthesis|Metabolism--Global and overview maps--Biosynthesis of secondary metabolites

[denovogenes]_111647	62.00	2e-109	sbr:SY1_06100	K00059	fabG	3-oxoacyl-[acyl-carrier protein] reductase	1.1.1.100	Metabolism--Lipid metabolism--Fatty acid biosynthesis|Metabolism--Metabolism of cofactors and vitamins--Biotin metabolism|Metabolism--Lipid metabolism--Biosynthesis of unsaturated fatty acids|Metabolism--Global and overview maps--Metabolic pathways|Metabolism--Global and overview maps--Fatty acid metabolism

[denovogenes]_111648	75.30	5e-135	fpla:A4U99_00110	K03496	parA, soj	chromosome partitioning protein	--	--

[denovogenes]_111649	100.00	4e-178	sdz:Asd1617_05343	K16345	xanP	xanthine permease XanP	--	--

[denovogenes]_11165	88.26	0.0	csb:CLSA_c01980	K02355	fusA, GFM, EFG	elongation factor G	--	--

[denovogenes]_111650	85.89	1e-154	fpla:A4U99_04060	K05936	cobM, cbiF	precorrin-4/cobalt-precorrin-4 C11-methyltransferase	2.1.1.133 2.1.1.271	Metabolism--Metabolism of cofactors and vitamins--Porphyrin and chlorophyll metabolism|Metabolism--Global and overview maps--Metabolic pathways

[denovogenes]_111651	93.25	7e-160	csr:Cspa_c18730	K18214	tetP_A, tet40	MFS transporter, DHA3 family, tetracycline resistance protein	--	--

[denovogenes]_111652	43.22	7e-35	hiz:R2866_0269	K09968	K09968	uncharacterized protein	--	--

[denovogenes]_111653	82.89	5e-144	ova:OBV_24380	K02967	RP-S2, MRPS2, rpsB	small subunit ribosomal protein S2	--	Genetic Information Processing--Translation--Ribosome

[denovogenes]_111656	40.27	3e-42	bbro:BAU06_23710	K14058	ttcA	tRNA 2-thiocytidine biosynthesis protein TtcA	--	--

[denovogenes]_111658	100.00	0.0	sbc:SbBS512_E1967	K07283	ydiY	putative salt-induced outer membrane protein	--	--

[denovogenes]_111663	68.25	9e-130	ere:EUBREC_0501	K01142	E3.1.11.2, xthA	exodeoxyribonuclease III	3.1.11.2	Genetic Information Processing--Replication and repair--Base excision repair

[denovogenes]_111665	57.20	3e-101	cno:NT01CX_1742	K15582	oppC	oligopeptide transport system permease protein	--	Human Diseases--Drug resistance: Antimicrobial--beta-Lactam resistance|Environmental Information Processing--Membrane transport--ABC transporters|Cellular Processes--Cellular community - prokaryotes--Quorum sensing

[denovogenes]_111666	43.08	2e-62	rbr:RBR_17410	K08600	srtB	sortase B	3.4.22.70	--

[denovogenes]_111667	99.60	0.0	cif:AL515_03250	K05781	phnK	putative phosphonate transport system ATP-binding protein	--	--

[denovogenes]_111668	50.75	2e-35	lacy:A4V08_00705	K07258	dacC, dacA, dacD	D-alanyl-D-alanine carboxypeptidase (penicillin-binding protein 5/6)	3.4.16.4	Metabolism--Glycan biosynthesis and metabolism--Peptidoglycan biosynthesis|Metabolism--Global and overview maps--Metabolic pathways

[denovogenes]_11167	99.85	0.0	eld:i02_0826	K03702	uvrB	excinuclease ABC subunit B	--	Genetic Information Processing--Replication and repair--Nucleotide excision repair

[denovogenes]_111670	66.80	2e-109	csh:Closa_0116	K03311	TC.LIVCS	branched-chain amino acid:cation transporter, LIVCS family	--	--

[denovogenes]_111672	92.06	3e-174	lacy:A4V08_34630	K01995	livG	branched-chain amino acid transport system ATP-binding protein	--	Environmental Information Processing--Membrane transport--ABC transporters|Cellular Processes--Cellular community - prokaryotes--Quorum sensing

[denovogenes]_111673	100.00	0.0	bth:BT_0811	K03437	spoU	RNA methyltransferase, TrmH family	--	--

[denovogenes]_111675	40.54	1e-44	cpo:COPRO5265_0262	K05995	pepE	dipeptidase E	3.4.13.21	--

[denovogenes]_111676	100.00	0.0	ecol:LY180_08820	K03785	aroD	3-dehydroquinate dehydratase I	4.2.1.10	Metabolism--Amino acid metabolism--Phenylalanine, tyrosine and tryptophan biosynthesis|Metabolism--Global and overview maps--Metabolic pathways|Metabolism--Global and overview maps--Biosynthesis of secondary metabolites|Metabolism--Global and overview maps--Biosynthesis of antibiotics|Metabolism--Global and overview maps--Biosynthesis of amino acids

[denovogenes]_111679	96.43	0.0	cfd:CFNIH1_14325	K11534	deoR	DeoR family transcriptional regulator, deoxyribose operon repressor	--	--

[denovogenes]_111680	79.28	1e-143	fpr:FP2_30640	K00789	metK	S-adenosylmethionine synthetase	2.5.1.6	Metabolism--Amino acid metabolism--Cysteine and methionine metabolism|Metabolism--Global and overview maps--Metabolic pathways|Metabolism--Global and overview maps--Biosynthesis of secondary metabolites|Metabolism--Global and overview maps--Biosynthesis of amino acids

[denovogenes]_111681	93.65	9e-178	cif:AL515_03225	K06167	phnP	phosphoribosyl 1,2-cyclic phosphate phosphodiesterase	3.1.4.55	Metabolism--Metabolism of other amino acids--Phosphonate and phosphinate metabolism

[denovogenes]_111682	40.38	2e-34	pdf:CD630DERM_27180	K08600	srtB	sortase B	3.4.22.70	--

[denovogenes]_111684	46.15	1e-74	lao:AOX59_18555	K04940	odh	opine dehydrogenase	1.5.1.28	--

[denovogenes]_111685	75.29	8e-134	rto:RTO_02530	K03650	mnmE, trmE, MSS1	tRNA modification GTPase	3.6.-.-	--

[denovogenes]_111687	59.60	3e-117	ssm:Spirs_3014	K01470	E3.5.2.10	creatinine amidohydrolase	3.5.2.10	Metabolism--Amino acid metabolism--Arginine and proline metabolism

[denovogenes]_111688	98.02	2e-171	cif:AL515_02950	K03327	TC.MATE, SLC47A, norM, mdtK, dinF	multidrug resistance protein, MATE family	--	--

[denovogenes]_111689	100.00	0.0	bth:BT_1367	K06167	phnP	phosphoribosyl 1,2-cyclic phosphate phosphodiesterase	3.1.4.55	Metabolism--Metabolism of other amino acids--Phosphonate and phosphinate metabolism

[denovogenes]_11169	55.29	0.0	afn:Acfer_0119	K03587	ftsI	cell division protein FtsI (penicillin-binding protein 3)	--	Metabolism--Glycan biosynthesis and metabolism--Peptidoglycan biosynthesis|Human Diseases--Drug resistance: Antimicrobial--beta-Lactam resistance

[denovogenes]_111690	69.71	8e-120	rob:CK5_20950	K07009	K07009	uncharacterized protein	--	--

[denovogenes]_111691	89.29	2e-173	bdh:GV66_13965	K01142	E3.1.11.2, xthA	exodeoxyribonuclease III	3.1.11.2	Genetic Information Processing--Replication and repair--Base excision repair

[denovogenes]_111694	66.00	6e-120	erb:A4V01_04350	K07124	K07124	uncharacterized protein	--	--

[denovogenes]_111696	72.22	4e-129	bhl:Bache_0319	K02493	hemK, prmC, HEMK	release factor glutamine methyltransferase	2.1.1.297	--

[denovogenes]_111697	95.63	0.0	cif:AL515_19355	K03575	mutY	A/G-specific adenine glycosylase	3.2.2.-	Genetic Information Processing--Replication and repair--Base excision repair

[denovogenes]_111698	100.00	6e-179	sbo:SBO_2856	K11735	gabP	GABA permease	--	--

[denovogenes]_111699	73.90	6e-127	lacy:A4V08_33205	K06147	ABCB-BAC	ATP-binding cassette, subfamily B, bacterial	--	--

[denovogenes]_1117	61.64	0.0	csb:CLSA_c13930	K03529	smc	chromosome segregation protein	--	--

[denovogenes]_11170	74.89	0.0	ctet:BN906_00407	K15987	hppA	K(+)-stimulated pyrophosphate-energized sodium pump	3.6.1.1	Metabolism--Energy metabolism--Oxidative phosphorylation

[denovogenes]_111700	72.47	2e-128	elm:ELI_2444	K01751	E4.3.1.15	diaminopropionate ammonia-lyase	4.3.1.15	--

[denovogenes]_111701	88.49	1e-169	bprs:CK3_13590	K12941	abgB	aminobenzoyl-glutamate utilization protein B	--	--

[denovogenes]_111702	79.01	9e-147	rum:CK1_00620	K03168	topA	DNA topoisomerase I	5.99.1.2	--

[denovogenes]_111707	44.62	2e-63	erj:EJP617_33040	K16702	amsE	amylovoran biosynthesis glycosyltransferase AmsE	2.4.-.-	--

[denovogenes]_111708	78.57	1e-147	cpf:CPF_2496	K01409	KAE1, tsaD, QRI7	N6-L-threonylcarbamoyladenine synthase	2.3.1.234	--

[denovogenes]_111711	66.01	2e-114	fpla:A4U99_00500	K18122	cat2, abfT	4-hydroxybutyrate CoA-transferase	2.8.3.-	Metabolism--Carbohydrate metabolism--Butanoate metabolism|Metabolism--Global and overview maps--Metabolic pathways|Metabolism--Global and overview maps--Carbon metabolism

[denovogenes]_111712	99.60	0.0	ecoa:APECO78_19525	K02082	agaS	tagatose-6-phosphate ketose/aldose isomerase	5.-.-.-	--

[denovogenes]_111713	58.63	2e-103	lpq:AF91_10605	K00425	cydA	cytochrome d ubiquinol oxidase subunit I	1.10.3.14	Metabolism--Energy metabolism--Oxidative phosphorylation|Metabolism--Global and overview maps--Metabolic pathways|Environmental Information Processing--Signal transduction--Two-component system

[denovogenes]_111714	43.80	2e-63	paeq:R50912_10270	K01208	cd, ma, nplT	cyclomaltodextrinase / maltogenic alpha-amylase / neopullulanase	3.2.1.54 3.2.1.133 3.2.1.135	Metabolism--Carbohydrate metabolism--Starch and sucrose metabolism|Metabolism--Global and overview maps--Metabolic pathways

[denovogenes]_111716	71.83	8e-129	hsd:SD1D_0052	K00864	glpK, GK	glycerol kinase	2.7.1.30	Metabolism--Lipid metabolism--Glycerolipid metabolism|Metabolism--Global and overview maps--Metabolic pathways|Organismal Systems--Endocrine system--PPAR signaling pathway|Organismal Systems--Environmental adaptation--Plant-pathogen interaction

[denovogenes]_111719	64.68	5e-114	sdq:SDSE167_0862	K01955	carB, CPA2	carbamoyl-phosphate synthase large subunit	6.3.5.5	Metabolism--Nucleotide metabolism--Pyrimidine metabolism|Metabolism--Amino acid metabolism--Alanine, aspartate and glutamate metabolism|Metabolism--Global and overview maps--Metabolic pathways

[denovogenes]_111722	72.76	9e-125	erb:A4V01_21090	K08963	mtnA	methylthioribose-1-phosphate isomerase	5.3.1.23	Metabolism--Amino acid metabolism--Cysteine and methionine metabolism|Metabolism--Global and overview maps--Metabolic pathways

[denovogenes]_111724	47.11	5e-66	cct:CC1_23020	K08384	spoVD	stage V sporulation protein D (sporulation-specific penicillin-binding protein)	--	--

[denovogenes]_111725	49.46	1e-59	cbn:CbC4_0889	K00336	nuoG	NADH-quinone oxidoreductase subunit G	1.6.5.3	Metabolism--Energy metabolism--Oxidative phosphorylation|Metabolism--Global and overview maps--Metabolic pathways

[denovogenes]_111727	96.03	0.0	pfr:PFREUD_09260	K00240	sdhB, frdB	succinate dehydrogenase / fumarate reductase, iron-sulfur subunit	1.3.5.1 1.3.5.4	Metabolism--Carbohydrate metabolism--Citrate cycle (TCA cycle)|Metabolism--Energy metabolism--Oxidative phosphorylation|Metabolism--Carbohydrate metabolism--Butanoate metabolism|Metabolism--Energy metabolism--Carbon fixation pathways in prokaryotes|Metabolism--Global and overview maps--Metabolic pathways|Metabolism--Global and overview maps--Biosynthesis of secondary metabolites|Metabolism--Global and overview maps--Microbial metabolism in diverse environments|Metabolism--Global and overview maps--Biosynthesis of antibiotics|Metabolism--Global and overview maps--Carbon metabolism

[denovogenes]_111728	92.06	2e-158	pfr:PFREUD_09290	K02069	ABC.X2.P	putative ABC transport system permease protein	--	--

[denovogenes]_111729	99.60	0.0	stb:SGPB_1016	K01092	E3.1.3.25, IMPA, suhB	myo-inositol-1(or 4)-monophosphatase	3.1.3.25	Metabolism--Biosynthesis of other secondary metabolites--Streptomycin biosynthesis|Metabolism--Carbohydrate metabolism--Inositol phosphate metabolism|Metabolism--Global and overview maps--Metabolic pathways|Environmental Information Processing--Signal transduction--Phosphatidylinositol signaling system

[denovogenes]_111733	87.95	8e-167	pdi:BDI_1302	K00721	DPM1	dolichol-phosphate mannosyltransferase	2.4.1.83	Metabolism--Glycan biosynthesis and metabolism--N-Glycan biosynthesis|Metabolism--Global and overview maps--Metabolic pathways

[denovogenes]_111734	82.94	2e-153	pdi:BDI_3082	K09817	znuC	zinc transport system ATP-binding protein	3.6.3.-	Environmental Information Processing--Membrane transport--ABC transporters

[denovogenes]_111737	44.44	2e-70	cpi:Cpin_6102	K02477	K02477	two-component system, LytT family, response regulator	--	--

[denovogenes]_111738	100.00	0.0	stb:SGPB_1406	K01803	TPI, tpiA	triosephosphate isomerase (TIM)	5.3.1.1	Metabolism--Carbohydrate metabolism--Glycolysis / Gluconeogenesis|Metabolism--Carbohydrate metabolism--Fructose and mannose metabolism|Metabolism--Carbohydrate metabolism--Inositol phosphate metabolism|Metabolism--Energy metabolism--Carbon fixation in photosynthetic organisms|Metabolism--Global and overview maps--Metabolic pathways|Metabolism--Global and overview maps--Biosynthesis of secondary metabolites|Metabolism--Global and overview maps--Microbial metabolism in diverse environments|Metabolism--Global and overview maps--Biosynthesis of antibiotics|Metabolism--Global and overview maps--Carbon metabolism|Metabolism--Global and overview maps--Biosynthesis of amino acids

[denovogenes]_111739	99.60	1e-177	cfd:CFNIH1_10875	K02419	fliP	flagellar biosynthetic protein FliP	--	Cellular Processes--Cell motility--Flagellar assembly

[denovogenes]_111740	98.02	0.0	cfd:CFNIH1_17745	K00598	tam	trans-aconitate 2-methyltransferase	2.1.1.144	--

[denovogenes]_111741	99.60	0.0	paec:M802_282	K07387	K07387	putative metalloprotease	3.4.24.-	--

[denovogenes]_111742	47.92	2e-57	paej:H70737_20350	K07507	mgtC	putative Mg2+ transporter-C (MgtC) family protein	--	--

[denovogenes]_111744	49.38	2e-72	dds:Ddes_0626	K07090	K07090	uncharacterized protein	--	--

[denovogenes]_111745	99.60	0.0	bth:BT_3894	K03439	trmB, METTL1	tRNA (guanine-N7-)-methyltransferase	2.1.1.33	--

[denovogenes]_111746	40.23	5e-45	maq:Maqu_1230	K04032	eutT	ethanolamine utilization cobalamin adenosyltransferase	2.5.1.17	Metabolism--Metabolism of cofactors and vitamins--Porphyrin and chlorophyll metabolism|Metabolism--Global and overview maps--Metabolic pathways

[denovogenes]_111747	71.49	2e-117	dvg:Deval_0134	K10036	glnH	glutamine transport system substrate-binding protein	--	Environmental Information Processing--Membrane transport--ABC transporters

[denovogenes]_111748	51.79	3e-86	dps:DP0378	K02056	ABC.SS.A	simple sugar transport system ATP-binding protein	3.6.3.17	--

[denovogenes]_111750	99.60	0.0	cfd:CFNIH1_16770	K03785	aroD	3-dehydroquinate dehydratase I	4.2.1.10	Metabolism--Amino acid metabolism--Phenylalanine, tyrosine and tryptophan biosynthesis|Metabolism--Global and overview maps--Metabolic pathways|Metabolism--Global and overview maps--Biosynthesis of secondary metabolites|Metabolism--Global and overview maps--Biosynthesis of antibiotics|Metabolism--Global and overview maps--Biosynthesis of amino acids

[denovogenes]_111751	41.90	6e-68	etr:ETAE_p006	K03203	virB8, lvhB8	type IV secretion system protein VirB8	--	Environmental Information Processing--Membrane transport--Bacterial secretion system

[denovogenes]_111752	63.56	2e-109	rbr:RBR_08200	K01265	map	methionyl aminopeptidase	3.4.11.18	--

[denovogenes]_111753	100.00	0.0	stb:SGPB_0674	K07124	K07124	uncharacterized protein	--	--

[denovogenes]_111754	99.60	4e-172	paeo:M801_613	K05801	djlA	DnaJ like chaperone protein	--	--

[denovogenes]_111755	98.41	0.0	fpla:A4U99_10160	K02823	pyrDII	dihydroorotate dehydrogenase electron transfer subunit	--	Metabolism--Nucleotide metabolism--Pyrimidine metabolism|Metabolism--Global and overview maps--Metabolic pathways

[denovogenes]_111757	67.25	3e-108	bfg:BF638R_1446	K15669	--	--	--	Metabolism--Glycan biosynthesis and metabolism--Lipopolysaccharide biosynthesis

[denovogenes]_111758	98.41	0.0	fpla:A4U99_07055	K09861	K09861	uncharacterized protein	--	--

[denovogenes]_11176	43.18	5e-86	mbs:MRBBS_2256	K02688	prpR	transcriptional regulator, propionate catabolism operon regulatory protein	--	--

[denovogenes]_111760	53.75	1e-89	bbay:A4V04_12840	K02013	ABC.FEV.A	iron complex transport system ATP-binding protein	3.6.3.34	Environmental Information Processing--Membrane transport--ABC transporters

[denovogenes]_111762	40.46	2e-50	seds:AAY24_03050	K04032	eutT	ethanolamine utilization cobalamin adenosyltransferase	2.5.1.17	Metabolism--Metabolism of cofactors and vitamins--Porphyrin and chlorophyll metabolism|Metabolism--Global and overview maps--Metabolic pathways

[denovogenes]_111764	61.92	2e-100	srz:AXX16_4149	K00757	udp, UPP	uridine phosphorylase	2.4.2.3	Metabolism--Nucleotide metabolism--Pyrimidine metabolism|Metabolism--Xenobiotics biodegradation and metabolism--Drug metabolism - other enzymes|Metabolism--Global and overview maps--Metabolic pathways

[denovogenes]_111765	65.57	2e-113	boa:Bovatus_01450	K19286	nfrA2	FMN reductase [NAD(P)H]	1.5.1.39	Metabolism--Metabolism of cofactors and vitamins--Riboflavin metabolism|Metabolism--Global and overview maps--Metabolic pathways

[denovogenes]_111766	100.00	1e-179	paec:M802_2433	K11892	impK, ompA, vasF, dotU	type VI secretion system protein ImpK	--	Environmental Information Processing--Membrane transport--Bacterial secretion system

[denovogenes]_111769	91.90	2e-170	bxy:BXY_03260	K00979	kdsB	3-deoxy-manno-octulosonate cytidylyltransferase (CMP-KDO synthetase)	2.7.7.38	Metabolism--Glycan biosynthesis and metabolism--Lipopolysaccharide biosynthesis|Metabolism--Global and overview maps--Metabolic pathways

[denovogenes]_11177	90.31	0.0	lacy:A4V08_14820	K01874	MARS, metG	methionyl-tRNA synthetase	6.1.1.10	Metabolism--Metabolism of other amino acids--Selenocompound metabolism|Genetic Information Processing--Translation--Aminoacyl-tRNA biosynthesis

[denovogenes]_111770	52.77	1e-85	dsy:DSY1140	K02017	modC	molybdate transport system ATP-binding protein	3.6.3.29	Environmental Information Processing--Membrane transport--ABC transporters

[denovogenes]_111771	100.00	4e-178	fpla:A4U99_10620	K02500	hisF	cyclase	4.1.3.-	Metabolism--Amino acid metabolism--Histidine metabolism|Metabolism--Global and overview maps--Metabolic pathways|Metabolism--Global and overview maps--Biosynthesis of secondary metabolites|Metabolism--Global and overview maps--Biosynthesis of amino acids

[denovogenes]_111772	99.21	0.0	cfd:CFNIH1_22885	K08680	menH	2-succinyl-6-hydroxy-2,4-cyclohexadiene-1-carboxylate synthase	4.2.99.20	Metabolism--Metabolism of cofactors and vitamins--Ubiquinone and other terpenoid-quinone biosynthesis|Metabolism--Global and overview maps--Metabolic pathways|Metabolism--Global and overview maps--Biosynthesis of secondary metabolites

[denovogenes]_111773	94.84	1e-179	bfr:BF2984	K06167	phnP	phosphoribosyl 1,2-cyclic phosphate phosphodiesterase	3.1.4.55	Metabolism--Metabolism of other amino acids--Phosphonate and phosphinate metabolism

[denovogenes]_111774	100.00	0.0	paeo:M801_3732	K00554	trmD	tRNA (guanine37-N1)-methyltransferase	2.1.1.228	--

[denovogenes]_111776	48.00	2e-80	pphr:APZ00_07075	K00046	idnO	gluconate 5-dehydrogenase	1.1.1.69	--

[denovogenes]_111777	100.00	0.0	cfd:CFNIH1_08155	K05781	phnK	putative phosphonate transport system ATP-binding protein	--	--

[denovogenes]_111778	98.81	0.0	cfd:CFNIH1_08130	K06167	phnP	phosphoribosyl 1,2-cyclic phosphate phosphodiesterase	3.1.4.55	Metabolism--Metabolism of other amino acids--Phosphonate and phosphinate metabolism

[denovogenes]_111779	100.00	5e-135	fpla:A4U99_00970	K01246	tag	DNA-3-methyladenine glycosylase I	3.2.2.20	Genetic Information Processing--Replication and repair--Base excision repair

[denovogenes]_111781	100.00	0.0	paeo:M801_801	K15256	cmoA	tRNA (cmo5U34)-methyltransferase	2.1.1.-	--

[denovogenes]_111782	52.96	8e-91	lpr:LBP_cg0676	K11741	sugE	quaternary ammonium compound-resistance protein SugE	--	--

[denovogenes]_111783	99.60	0.0	cfd:CFNIH1_03575	K09972	aapP, bztD	general L-amino acid transport system ATP-binding protein	3.6.3.-	Environmental Information Processing--Membrane transport--ABC transporters

[denovogenes]_111784	99.60	0.0	cfd:CFNIH1_14325	K11534	deoR	DeoR family transcriptional regulator, deoxyribose operon repressor	--	--

[denovogenes]_111785	59.92	7e-97	tee:Tel_05505	K02500	hisF	cyclase	4.1.3.-	Metabolism--Amino acid metabolism--Histidine metabolism|Metabolism--Global and overview maps--Metabolic pathways|Metabolism--Global and overview maps--Biosynthesis of secondary metabolites|Metabolism--Global and overview maps--Biosynthesis of amino acids

[denovogenes]_111786	40.16	2e-52	cpas:Clopa_4260	K00059	fabG	3-oxoacyl-[acyl-carrier protein] reductase	1.1.1.100	Metabolism--Lipid metabolism--Fatty acid biosynthesis|Metabolism--Metabolism of cofactors and vitamins--Biotin metabolism|Metabolism--Lipid metabolism--Biosynthesis of unsaturated fatty acids|Metabolism--Global and overview maps--Metabolic pathways|Metabolism--Global and overview maps--Fatty acid metabolism

[denovogenes]_111787	95.22	7e-166	lacy:A4V08_32475	K06182	rluF	23S rRNA pseudouridine2604 synthase	5.4.99.21	--

[denovogenes]_111788	100.00	0.0	paec:M802_3928	K02656	pilF	type IV pilus assembly protein PilF	--	--

[denovogenes]_111791	98.41	0.0	lacy:A4V08_33250	K01449	E3.5.1.28C, cwlJ, sleB	N-acetylmuramoyl-L-alanine amidase	3.5.1.28	--

[denovogenes]_111792	96.05	2e-160	bfb:VU15_16780	K00945	cmk	CMP/dCMP kinase	2.7.4.25	Metabolism--Nucleotide metabolism--Pyrimidine metabolism|Metabolism--Global and overview maps--Metabolic pathways

[denovogenes]_111794	99.21	0.0	cfd:CFNIH1_07565	K03148	thiF	sulfur carrier protein ThiS adenylyltransferase	2.7.7.73	Metabolism--Metabolism of cofactors and vitamins--Thiamine metabolism|Metabolism--Global and overview maps--Metabolic pathways|Genetic Information Processing--Folding, sorting and degradation--Sulfur relay system

[denovogenes]_111796	52.36	5e-85	awo:Awo_c34920	K06223	dam	DNA adenine methylase	2.1.1.72	Genetic Information Processing--Replication and repair--Mismatch repair

[denovogenes]_111797	42.69	1e-56	rha:RHA1_ro00912	K01757	E4.3.3.2	strictosidine synthase	4.3.3.2	Metabolism--Biosynthesis of other secondary metabolites--Indole alkaloid biosynthesis|Metabolism--Global and overview maps--Metabolic pathways|Metabolism--Global and overview maps--Biosynthesis of secondary metabolites

[denovogenes]_111798	96.03	1e-180	lacy:A4V08_18510	K02010	afuC, fbpC	iron(III) transport system ATP-binding protein	3.6.3.30	Environmental Information Processing--Membrane transport--ABC transporters

[denovogenes]_111799	98.81	0.0	pdi:BDI_3651	K03439	trmB, METTL1	tRNA (guanine-N7-)-methyltransferase	2.1.1.33	--

[denovogenes]_1118	47.12	0.0	efm:M7W_1788	K16899	addB	ATP-dependent helicase/nuclease subunit B	3.1.-.- 3.6.4.12	--

[denovogenes]_11180	59.56	0.0	byl:A4V09_22900	K09955	K09955	uncharacterized protein	--	--

[denovogenes]_111800	99.60	0.0	bfb:VU15_17970	K03439	trmB, METTL1	tRNA (guanine-N7-)-methyltransferase	2.1.1.33	--

[denovogenes]_111802	100.00	0.0	cfd:CFNIH1_04595	K01091	gph	phosphoglycolate phosphatase	3.1.3.18	Metabolism--Carbohydrate metabolism--Glyoxylate and dicarboxylate metabolism|Metabolism--Global and overview maps--Metabolic pathways|Metabolism--Global and overview maps--Biosynthesis of secondary metabolites|Metabolism--Global and overview maps--Biosynthesis of antibiotics

[denovogenes]_111803	96.83	2e-170	fpla:A4U99_12835	K03437	spoU	RNA methyltransferase, TrmH family	--	--

[denovogenes]_111804	100.00	0.0	paeo:M801_4399	K00059	fabG	3-oxoacyl-[acyl-carrier protein] reductase	1.1.1.100	Metabolism--Lipid metabolism--Fatty acid biosynthesis|Metabolism--Metabolism of cofactors and vitamins--Biotin metabolism|Metabolism--Lipid metabolism--Biosynthesis of unsaturated fatty acids|Metabolism--Global and overview maps--Metabolic pathways|Metabolism--Global and overview maps--Fatty acid metabolism

[denovogenes]_111805	100.00	0.0	pdi:BDI_3596	K07315	rsbU_P	phosphoserine phosphatase RsbU/P	3.1.3.3	--

[denovogenes]_111806	100.00	0.0	cif:AL515_21770	K02444	glpR	DeoR family transcriptional regulator, glycerol-3-phosphate regulon repressor	--	--

[denovogenes]_111807	92.46	4e-177	pdi:BDI_3651	K03439	trmB, METTL1	tRNA (guanine-N7-)-methyltransferase	2.1.1.33	--

[denovogenes]_11181	99.41	0.0	fpla:A4U99_04265	K00700	GBE1, glgB	1,4-alpha-glucan branching enzyme	2.4.1.18	Metabolism--Carbohydrate metabolism--Starch and sucrose metabolism|Metabolism--Global and overview maps--Metabolic pathways|Metabolism--Global and overview maps--Biosynthesis of secondary metabolites

[denovogenes]_111810	99.21	0.0	stb:SGPB_1357	K07009	K07009	uncharacterized protein	--	--

[denovogenes]_111811	100.00	0.0	cfd:CFNIH1_05300	K12371	dppD	dipeptide transport system ATP-binding protein	--	Environmental Information Processing--Membrane transport--ABC transporters

[denovogenes]_111812	42.52	6e-67	ypf:BZ19_369	K13002	wbyL	glycosyltransferase	2.4.1.-	--

[denovogenes]_111814	96.83	1e-180	lacy:A4V08_09990	K02028	ABC.PA.A	polar amino acid transport system ATP-binding protein	3.6.3.21	--

[denovogenes]_111815	55.13	7e-87	cgo:Corgl_1555	K01243	mtnN, mtn, pfs	adenosylhomocysteine nucleosidase	3.2.2.9	Metabolism--Amino acid metabolism--Cysteine and methionine metabolism|Metabolism--Global and overview maps--Metabolic pathways|Metabolism--Global and overview maps--Biosynthesis of amino acids

[denovogenes]_111816	90.87	1e-176	pdi:BDI_3651	K03439	trmB, METTL1	tRNA (guanine-N7-)-methyltransferase	2.1.1.33	--

[denovogenes]_111817	99.60	6e-177	paec:M802_4816	K21029	moeB	molybdopterin-synthase adenylyltransferase	2.7.7.80	Genetic Information Processing--Folding, sorting and degradation--Sulfur relay system

[denovogenes]_111818	94.44	1e-173	lacy:A4V08_34255	K00215	dapB	4-hydroxy-tetrahydrodipicolinate reductase	1.17.1.8	Metabolism--Biosynthesis of other secondary metabolites--Monobactam biosynthesis|Metabolism--Amino acid metabolism--Lysine biosynthesis|Metabolism--Global and overview maps--Metabolic pathways|Metabolism--Global and overview maps--Biosynthesis of secondary metabolites|Metabolism--Global and overview maps--Microbial metabolism in diverse environments|Metabolism--Global and overview maps--Biosynthesis of antibiotics|Metabolism--Global and overview maps--Biosynthesis of amino acids

[denovogenes]_11182	88.11	0.0	cle:Clole_3166	K15987	hppA	K(+)-stimulated pyrophosphate-energized sodium pump	3.6.1.1	Metabolism--Energy metabolism--Oxidative phosphorylation

[denovogenes]_111820	67.33	2e-125	ddl:Desdi_0748	K13653	K13653	AraC family transcriptional regulator	--	--

[denovogenes]_111822	90.48	8e-164	csh:Closa_2091	K01995	livG	branched-chain amino acid transport system ATP-binding protein	--	Environmental Information Processing--Membrane transport--ABC transporters|Cellular Processes--Cellular community - prokaryotes--Quorum sensing

[denovogenes]_111824	100.00	0.0	cfd:CFNIH1_16555	K03478	chbG	chitin disaccharide deacetylase	3.5.1.105	--

[denovogenes]_111825	99.60	0.0	cfd:CFNIH1_05125	K15984	rsmJ	16S rRNA (guanine1516-N2)-methyltransferase	2.1.1.242	--

[denovogenes]_111826	99.21	0.0	cfd:CFNIH1_09050	K06169	miaE	tRNA-(ms[2]io[6]A)-hydroxylase	1.-.-.-	--

[denovogenes]_111829	61.54	6e-110	lir:LAW_00055	K02050	ABC.SN.P	NitT/TauT family transport system permease protein	--	--

[denovogenes]_111830	96.83	7e-180	lacy:A4V08_22625	K02049	ABC.SN.A	NitT/TauT family transport system ATP-binding protein	--	--

[denovogenes]_111831	98.41	0.0	bbrc:B7019_2064	K06999	K06999	phospholipase/carboxylesterase	--	--

[denovogenes]_111832	99.60	0.0	lacy:A4V08_34630	K01995	livG	branched-chain amino acid transport system ATP-binding protein	--	Environmental Information Processing--Membrane transport--ABC transporters|Cellular Processes--Cellular community - prokaryotes--Quorum sensing

[denovogenes]_111834	88.10	3e-169	cso:CLS_12050	K01611	speD, AMD1	S-adenosylmethionine decarboxylase	4.1.1.50	Metabolism--Amino acid metabolism--Cysteine and methionine metabolism|Metabolism--Amino acid metabolism--Arginine and proline metabolism|Metabolism--Global and overview maps--Metabolic pathways

[denovogenes]_111835	61.63	3e-109	cpf:CPF_1154	K00076	hdhA	7-alpha-hydroxysteroid dehydrogenase	1.1.1.159	--

[denovogenes]_111837	87.70	3e-158	csh:Closa_0263	K02041	phnC	phosphonate transport system ATP-binding protein	3.6.3.28	Environmental Information Processing--Membrane transport--ABC transporters

[denovogenes]_111838	100.00	0.0	boa:Bovatus_01340	K02036	pstB	phosphate transport system ATP-binding protein	3.6.3.27	Environmental Information Processing--Membrane transport--ABC transporters

[denovogenes]_111839	78.57	7e-148	pdi:BDI_3082	K09817	znuC	zinc transport system ATP-binding protein	3.6.3.-	Environmental Information Processing--Membrane transport--ABC transporters

[denovogenes]_11184	89.78	0.0	bcel:BcellWH2_01613	K00705	malQ	4-alpha-glucanotransferase	2.4.1.25	Metabolism--Carbohydrate metabolism--Starch and sucrose metabolism|Metabolism--Global and overview maps--Metabolic pathways

[denovogenes]_111841	42.80	1e-59	csr:Cspa_c09540	K02755	PTS-Bgl-EIIA, bglF, bglP	PTS system, beta-glucoside-specific IIA component	2.7.1.-	Environmental Information Processing--Membrane transport--Phosphotransferase system (PTS)

[denovogenes]_111841	42.80	1e-59	csr:Cspa_c09540	K02756	PTS-Bgl-EIIB, bglF, bglP	PTS system, beta-glucoside-specific IIB component	2.7.1.-	Environmental Information Processing--Membrane transport--Phosphotransferase system (PTS)

[denovogenes]_111841	42.80	1e-59	csr:Cspa_c09540	K02757	PTS-Bgl-EIIC, bglF, bglP	PTS system, beta-glucoside-specific IIC component	--	Environmental Information Processing--Membrane transport--Phosphotransferase system (PTS)

[denovogenes]_111843	99.59	1e-177	cfd:CFNIH1_01590	K09807	K09807	uncharacterized protein	--	--

[denovogenes]_111846	92.06	2e-169	lacy:A4V08_19570	K00261	GLUD1_2, gdhA	glutamate dehydrogenase (NAD(P)+)	1.4.1.3	Metabolism--Amino acid metabolism--Arginine biosynthesis|Metabolism--Amino acid metabolism--Alanine, aspartate and glutamate metabolism|Metabolism--Metabolism of other amino acids--D-Glutamine and D-glutamate metabolism|Metabolism--Energy metabolism--Nitrogen metabolism|Metabolism--Global and overview maps--Metabolic pathways|Metabolism--Global and overview maps--Carbon metabolism|Organismal Systems--Excretory system--Proximal tubule bicarbonate reclamation

[denovogenes]_111847	99.21	0.0	lacy:A4V08_00745	K03570	mreC	rod shape-determining protein MreC	--	--

[denovogenes]_11185	97.18	0.0	pfre:RM25_1351	K00239	sdhA, frdA	succinate dehydrogenase / fumarate reductase, flavoprotein subunit	1.3.5.1 1.3.5.4	Metabolism--Carbohydrate metabolism--Citrate cycle (TCA cycle)|Metabolism--Energy metabolism--Oxidative phosphorylation|Metabolism--Carbohydrate metabolism--Butanoate metabolism|Metabolism--Energy metabolism--Carbon fixation pathways in prokaryotes|Metabolism--Global and overview maps--Metabolic pathways|Metabolism--Global and overview maps--Biosynthesis of secondary metabolites|Metabolism--Global and overview maps--Microbial metabolism in diverse environments|Metabolism--Global and overview maps--Biosynthesis of antibiotics|Metabolism--Global and overview maps--Carbon metabolism|Human Diseases--Infectious diseases: Bacterial--Legionellosis

[denovogenes]_111855	66.53	5e-108	shi:Shel_26650	K07090	K07090	uncharacterized protein	--	--

[denovogenes]_111856	40.10	1e-36	ere:EUBREC_1730	K00945	cmk	CMP/dCMP kinase	2.7.4.25	Metabolism--Nucleotide metabolism--Pyrimidine metabolism|Metabolism--Global and overview maps--Metabolic pathways

[denovogenes]_111859	94.86	9e-175	lacy:A4V08_32825	K06383	spoIIGA	stage II sporulation protein GA (sporulation sigma-E factor processing peptidase)	3.4.23.-	--

[denovogenes]_11186	99.26	0.0	btho:Btheta7330_05148	K07407	E3.2.1.22B, galA, rafA	alpha-galactosidase	3.2.1.22	Metabolism--Carbohydrate metabolism--Galactose metabolism|Metabolism--Lipid metabolism--Glycerolipid metabolism|Metabolism--Lipid metabolism--Sphingolipid metabolism|Metabolism--Glycan biosynthesis and metabolism--Glycosphingolipid biosynthesis - globo and isoglobo series

[denovogenes]_111861	91.30	3e-176	pdi:BDI_1490	K00651	metA	homoserine O-succinyltransferase	2.3.1.46	Metabolism--Amino acid metabolism--Cysteine and methionine metabolism|Metabolism--Energy metabolism--Sulfur metabolism|Metabolism--Global and overview maps--Metabolic pathways|Metabolism--Global and overview maps--Biosynthesis of secondary metabolites|Metabolism--Global and overview maps--Biosynthesis of amino acids

[denovogenes]_111862	41.33	4e-55	ngg:RG540_CH13770	K02335	DPO1, polA	DNA polymerase I	2.7.7.7	Metabolism--Nucleotide metabolism--Purine metabolism|Metabolism--Nucleotide metabolism--Pyrimidine metabolism|Metabolism--Global and overview maps--Metabolic pathways|Genetic Information Processing--Replication and repair--DNA replication|Genetic Information Processing--Replication and repair--Base excision repair|Genetic Information Processing--Replication and repair--Nucleotide excision repair|Genetic Information Processing--Replication and repair--Homologous recombination

[denovogenes]_111864	61.57	1e-101	rim:ROI_25190	K06182	rluF	23S rRNA pseudouridine2604 synthase	5.4.99.21	--

[denovogenes]_111865	83.33	8e-157	bhl:Bache_0523	K07214	fes	enterochelin esterase and related enzymes	--	--

[denovogenes]_111867	100.00	0.0	cfd:CFNIH1_01660	K07387	K07387	putative metalloprotease	3.4.24.-	--

[denovogenes]_111868	40.48	6e-54	dtn:DTL3_0333	K02026	ABC.MS.P1	multiple sugar transport system permease protein	--	--

[denovogenes]_111869	61.11	1e-107	shi:Shel_08880	K03589	ftsQ	cell division protein FtsQ	--	Cellular Processes--Cell growth and death--Cell cycle - Caulobacter

[denovogenes]_111870	99.19	0.0	mmk:MU9_2214	K02013	ABC.FEV.A	iron complex transport system ATP-binding protein	3.6.3.34	Environmental Information Processing--Membrane transport--ABC transporters

[denovogenes]_111873	42.52	6e-45	aci:ACIAD3431	K01154	hsdS	type I restriction enzyme, S subunit	3.1.21.3	--

[denovogenes]_111874	90.55	2e-171	pvl:AOB99_13655	K04753	sufI	suppressor of ftsI	--	--

[denovogenes]_111875	43.75	1e-60	pmib:BB2000_2822	K15835	murR	RpiR family transcriptional regulator, murPQ operon repressor	--	--

[denovogenes]_111876	44.73	7e-60	ctx:Clo1313_1852	K09121	K09121	uncharacterized protein	--	--

[denovogenes]_111878	92.46	9e-168	pdi:BDI_0477	K00790	murA	UDP-N-acetylglucosamine 1-carboxyvinyltransferase	2.5.1.7	Metabolism--Carbohydrate metabolism--Amino sugar and nucleotide sugar metabolism|Metabolism--Glycan biosynthesis and metabolism--Peptidoglycan biosynthesis|Metabolism--Global and overview maps--Metabolic pathways

[denovogenes]_111879	99.60	0.0	eoc:CE10_4814	K05781	phnK	putative phosphonate transport system ATP-binding protein	--	--

[denovogenes]_11188	84.10	0.0	bfb:VU15_06680	K19049	cslA	chondroitin AC lyase	4.2.2.5	--

[denovogenes]_111880	49.19	2e-79	fpla:A4U99_10685	K06714	rocR	arginine utilization regulatory protein	--	--

[denovogenes]_111881	71.90	2e-103	ele:Elen_1450	K03311	TC.LIVCS	branched-chain amino acid:cation transporter, LIVCS family	--	--

[denovogenes]_111884	55.77	3e-101	fae:FAES_2681	K01869	LARS, leuS	leucyl-tRNA synthetase	6.1.1.4	Genetic Information Processing--Translation--Aminoacyl-tRNA biosynthesis

[denovogenes]_11189	99.55	0.0	pael:T223_25015	K03406	mcp	methyl-accepting chemotaxis protein	--	Environmental Information Processing--Signal transduction--Two-component system|Cellular Processes--Cell motility--Bacterial chemotaxis

[denovogenes]_111891	77.38	2e-113	eyy:EGYY_17870	K03521	fixA, etfB	electron transfer flavoprotein beta subunit	--	--

[denovogenes]_111892	100.00	0.0	prp:M062_20315	K06970	rlmF	23S rRNA (adenine1618-N6)-methyltransferase	2.1.1.181	--

[denovogenes]_111894	65.59	1e-117	bfu:BC1G_00472	K01955	carB, CPA2	carbamoyl-phosphate synthase large subunit	6.3.5.5	Metabolism--Nucleotide metabolism--Pyrimidine metabolism|Metabolism--Amino acid metabolism--Alanine, aspartate and glutamate metabolism|Metabolism--Global and overview maps--Metabolic pathways

[denovogenes]_111896	96.84	0.0	cif:AL515_11600	K00868	pdxK, pdxY	pyridoxine kinase	2.7.1.35	Metabolism--Metabolism of cofactors and vitamins--Vitamin B6 metabolism|Metabolism--Global and overview maps--Metabolic pathways

[denovogenes]_111898	44.44	4e-27	nmv:NITMOv2_0715	K07658	phoB1, phoP	two-component system, OmpR family, alkaline phosphatase synthesis response regulator PhoP	--	Environmental Information Processing--Signal transduction--Two-component system

[denovogenes]_111899	70.26	2e-101	abao:BEQ56_12890	K10117	msmE	raffinose/stachyose/melibiose transport system substrate-binding protein	--	Environmental Information Processing--Membrane transport--ABC transporters

[denovogenes]_1119	60.20	0.0	csb:CLSA_c13930	K03529	smc	chromosome segregation protein	--	--

[denovogenes]_11190	99.26	0.0	cfd:CFNIH1_01295	K11931	pgaB	biofilm PGA synthesis lipoprotein PgaB	3.-.-.-	Cellular Processes--Cellular community - prokaryotes--Biofilm formation - Escherichia coli

[denovogenes]_111905	90.12	9e-172	lrc:LOCK908_1724	K01262	pepP	Xaa-Pro aminopeptidase	3.4.11.9	--

[denovogenes]_111906	88.89	2e-169	bth:BT_3894	K03439	trmB, METTL1	tRNA (guanine-N7-)-methyltransferase	2.1.1.33	--

[denovogenes]_111907	100.00	0.0	bbrs:BS27_0681	K01889	FARSA, pheS	phenylalanyl-tRNA synthetase alpha chain	6.1.1.20	Genetic Information Processing--Translation--Aminoacyl-tRNA biosynthesis

[denovogenes]_111908	66.11	6e-112	ash:AL1_23190	K03524	birA	BirA family transcriptional regulator, biotin operon repressor / biotin-[acetyl-CoA-carboxylase] ligase	6.3.4.15	Metabolism--Metabolism of cofactors and vitamins--Biotin metabolism|Metabolism--Global and overview maps--Metabolic pathways

[denovogenes]_111909	96.77	0.0	lacy:A4V08_24335	K00097	pdxA	4-hydroxythreonine-4-phosphate dehydrogenase	1.1.1.262	Metabolism--Metabolism of cofactors and vitamins--Vitamin B6 metabolism|Metabolism--Global and overview maps--Metabolic pathways

[denovogenes]_111910	70.87	4e-129	pdn:HMPREF9137_0369	K01338	lon	ATP-dependent Lon protease	3.4.21.53	Cellular Processes--Cell growth and death--Cell cycle - Caulobacter

[denovogenes]_111911	94.05	3e-172	pvl:AOB99_03285	K10441	rbsA	ribose transport system ATP-binding protein	3.6.3.17	Environmental Information Processing--Membrane transport--ABC transporters

[denovogenes]_111912	94.86	4e-177	pvl:AOB99_11570	K03631	recN	DNA repair protein RecN (Recombination protein N)	--	--

[denovogenes]_111913	76.40	2e-144	bfb:VU15_19385	K03733	xerC	integrase/recombinase XerC	--	--

[denovogenes]_111914	100.00	0.0	boa:Bovatus_03999	K02357	tsf, TSFM	elongation factor Ts	--	--

[denovogenes]_111915	57.75	1e-102	erb:A4V01_13970	K01928	murE	UDP-N-acetylmuramoyl-L-alanyl-D-glutamate--2,6-diaminopimelate ligase	6.3.2.13	Metabolism--Amino acid metabolism--Lysine biosynthesis|Metabolism--Glycan biosynthesis and metabolism--Peptidoglycan biosynthesis

[denovogenes]_111917	100.00	0.0	bbrj:B7017_1941	K02003	ABC.CD.A	putative ABC transport system ATP-binding protein	--	--

[denovogenes]_111918	73.02	5e-137	dmi:Desmer_2413	K03502	DPO5C, umuC	DNA polymerase V	--	--

[denovogenes]_111919	42.48	2e-53	sulr:B649_05450	K07133	K07133	uncharacterized protein	--	--

[denovogenes]_11192	56.73	0.0	bcel:BcellWH2_02284	K05989	ramA	alpha-L-rhamnosidase	3.2.1.40	--

[denovogenes]_111920	84.58	3e-153	cgo:Corgl_1746	K00266	gltD	glutamate synthase (NADPH/NADH) small chain	1.4.1.13 1.4.1.14	Metabolism--Amino acid metabolism--Alanine, aspartate and glutamate metabolism|Metabolism--Energy metabolism--Nitrogen metabolism|Metabolism--Global and overview maps--Metabolic pathways|Metabolism--Global and overview maps--Biosynthesis of secondary metabolites|Metabolism--Global and overview maps--Microbial metabolism in diverse environments|Metabolism--Global and overview maps--Biosynthesis of antibiotics|Metabolism--Global and overview maps--Biosynthesis of amino acids

[denovogenes]_111921	99.21	0.0	cfd:CFNIH1_15635	K03885	ndh	NADH dehydrogenase	1.6.99.3	Metabolism--Energy metabolism--Oxidative phosphorylation

[denovogenes]_111923	97.63	0.0	cfd:CFNIH1_15910	K02035	ABC.PE.S	peptide/nickel transport system substrate-binding protein	--	Cellular Processes--Cellular community - prokaryotes--Quorum sensing

[denovogenes]_111924	93.68	3e-174	pmib:BB2000_2447	K01448	E3.5.1.28B, amiA, amiB, amiC	N-acetylmuramoyl-L-alanine amidase	3.5.1.28	Human Diseases--Drug resistance: Antimicrobial--Cationic antimicrobial peptide (CAMP) resistance

[denovogenes]_111926	64.82	1e-118	rim:ROI_04030	K03292	TC.GPH	glycoside/pentoside/hexuronide:cation symporter, GPH family	--	--

[denovogenes]_111927	89.24	5e-165	shi:Shel_10740	K18682	rny	ribonucrease Y	3.1.-.-	Genetic Information Processing--Folding, sorting and degradation--RNA degradation

[denovogenes]_111928	60.25	4e-92	olo:ADJ70_04360	K02031	ABC.PE.A	peptide/nickel transport system ATP-binding protein	--	Cellular Processes--Cellular community - prokaryotes--Quorum sensing

[denovogenes]_11193	67.26	0.0	rto:RTO_16960	K08384	spoVD	stage V sporulation protein D (sporulation-specific penicillin-binding protein)	--	--

[denovogenes]_111930	79.92	2e-143	sdq:SDSE167_1774	K01916	nadE	NAD+ synthase	6.3.1.5	Metabolism--Metabolism of cofactors and vitamins--Nicotinate and nicotinamide metabolism|Metabolism--Global and overview maps--Metabolic pathways

[denovogenes]_111933	91.24	5e-169	ele:Elen_2150	K01714	dapA	4-hydroxy-tetrahydrodipicolinate synthase	4.3.3.7	Metabolism--Biosynthesis of other secondary metabolites--Monobactam biosynthesis|Metabolism--Amino acid metabolism--Lysine biosynthesis|Metabolism--Global and overview maps--Metabolic pathways|Metabolism--Global and overview maps--Biosynthesis of secondary metabolites|Metabolism--Global and overview maps--Microbial metabolism in diverse environments|Metabolism--Global and overview maps--Biosynthesis of antibiotics|Metabolism--Global and overview maps--Biosynthesis of amino acids

[denovogenes]_111934	65.61	9e-118	bcel:BcellWH2_03232	K03797	E3.4.21.102, prc, ctpA	carboxyl-terminal processing protease	3.4.21.102	--

[denovogenes]_111943	64.71	1e-109	olo:ADJ70_12595	K03215	rumA	23S rRNA (uracil1939-C5)-methyltransferase	2.1.1.190	--

[denovogenes]_111945	88.14	8e-165	emu:EMQU_0620	K15583	oppD	oligopeptide transport system ATP-binding protein	--	Human Diseases--Drug resistance: Antimicrobial--beta-Lactam resistance|Environmental Information Processing--Membrane transport--ABC transporters|Cellular Processes--Cellular community - prokaryotes--Quorum sensing

[denovogenes]_111946	95.65	7e-174	sdz:Asd1617_03574	K18324	acrD	multidrug efflux pump	--	Environmental Information Processing--Signal transduction--Two-component system

[denovogenes]_111947	59.18	2e-102	ebt:EBL_c36870	K06182	rluF	23S rRNA pseudouridine2604 synthase	5.4.99.21	--

[denovogenes]_111949	46.06	4e-69	gfe:Gferi_04105	K02057	ABC.SS.P	simple sugar transport system permease protein	--	--

[denovogenes]_111951	76.89	8e-120	bbp:BBPR_1566	K01214	ISA, treX	isoamylase	3.2.1.68	Metabolism--Carbohydrate metabolism--Starch and sucrose metabolism|Metabolism--Global and overview maps--Metabolic pathways|Metabolism--Global and overview maps--Biosynthesis of secondary metabolites

[denovogenes]_111954	78.37	7e-134	lacy:A4V08_30200	K03292	TC.GPH	glycoside/pentoside/hexuronide:cation symporter, GPH family	--	--

[denovogenes]_111955	66.40	9e-125	xor:XOC_1783	K00548	metH, MTR	5-methyltetrahydrofolate--homocysteine methyltransferase	2.1.1.13	Metabolism--Amino acid metabolism--Cysteine and methionine metabolism|Metabolism--Metabolism of other amino acids--Selenocompound metabolism|Metabolism--Metabolism of cofactors and vitamins--One carbon pool by folate|Metabolism--Global and overview maps--Metabolic pathways|Metabolism--Global and overview maps--Biosynthesis of secondary metabolites|Metabolism--Global and overview maps--Biosynthesis of amino acids

[denovogenes]_111959	82.47	8e-153	bcel:BcellWH2_05173	K02004	ABC.CD.P	putative ABC transport system permease protein	--	--

[denovogenes]_111963	50.59	2e-80	cgt:cgR_1383	K00688	PYG, glgP	glycogen phosphorylase	2.4.1.1	Metabolism--Carbohydrate metabolism--Starch and sucrose metabolism|Metabolism--Global and overview maps--Metabolic pathways|Metabolism--Global and overview maps--Biosynthesis of secondary metabolites|Cellular Processes--Cellular community - prokaryotes--Biofilm formation - Escherichia coli|Organismal Systems--Endocrine system--Insulin signaling pathway|Organismal Systems--Endocrine system--Glucagon signaling pathway|Human Diseases--Endocrine and metabolic diseases--Insulin resistance

[denovogenes]_111967	99.58	3e-176	boa:Bovatus_00467	K03814	mtgA	monofunctional biosynthetic peptidoglycan transglycosylase	2.4.1.-	Metabolism--Glycan biosynthesis and metabolism--Peptidoglycan biosynthesis

[denovogenes]_111968	99.10	1e-158	boa:Bovatus_04700	K07507	mgtC	putative Mg2+ transporter-C (MgtC) family protein	--	--

[denovogenes]_111969	43.25	3e-65	bco:Bcell_2853	K03785	aroD	3-dehydroquinate dehydratase I	4.2.1.10	Metabolism--Amino acid metabolism--Phenylalanine, tyrosine and tryptophan biosynthesis|Metabolism--Global and overview maps--Metabolic pathways|Metabolism--Global and overview maps--Biosynthesis of secondary metabolites|Metabolism--Global and overview maps--Biosynthesis of antibiotics|Metabolism--Global and overview maps--Biosynthesis of amino acids

[denovogenes]_11197	96.73	0.0	vpr:Vpar_1501	K02168	betT, betS	choline/glycine/proline betaine transport protein	--	--

[denovogenes]_111970	89.29	3e-166	boa:Bovatus_02302	K03524	birA	BirA family transcriptional regulator, biotin operon repressor / biotin-[acetyl-CoA-carboxylase] ligase	6.3.4.15	Metabolism--Metabolism of cofactors and vitamins--Biotin metabolism|Metabolism--Global and overview maps--Metabolic pathways

[denovogenes]_111971	98.81	0.0	boa:Bovatus_04013	K03439	trmB, METTL1	tRNA (guanine-N7-)-methyltransferase	2.1.1.33	--

[denovogenes]_111972	54.33	4e-96	bon:A361_00260	K03424	--	--	--	--

[denovogenes]_111973	100.00	0.0	boa:Bovatus_00124	K00655	plsC	1-acyl-sn-glycerol-3-phosphate acyltransferase	2.3.1.51	Metabolism--Lipid metabolism--Glycerolipid metabolism|Metabolism--Lipid metabolism--Glycerophospholipid metabolism|Metabolism--Global and overview maps--Metabolic pathways|Metabolism--Global and overview maps--Biosynthesis of secondary metabolites

[denovogenes]_111978	47.20	9e-75	cpas:Clopa_2085	K03481	glvR	RpiR family transcriptional regulator, glv operon transcriptional regulator	--	--

[denovogenes]_11198	100.00	0.0	shq:A0259_09255	K11931	pgaB	biofilm PGA synthesis lipoprotein PgaB	3.-.-.-	Cellular Processes--Cellular community - prokaryotes--Biofilm formation - Escherichia coli

[denovogenes]_111982	100.00	0.0	pvl:AOB99_11100	K08354	phsC	thiosulfate reductase cytochrome b subunit	--	Metabolism--Energy metabolism--Sulfur metabolism|Metabolism--Global and overview maps--Microbial metabolism in diverse environments

[denovogenes]_111983	86.45	2e-162	fne:FSDG_00506	K05936	cobM, cbiF	precorrin-4/cobalt-precorrin-4 C11-methyltransferase	2.1.1.133 2.1.1.271	Metabolism--Metabolism of cofactors and vitamins--Porphyrin and chlorophyll metabolism|Metabolism--Global and overview maps--Metabolic pathways

[denovogenes]_111984	44.53	4e-75	cbz:Cbs_4702	K03481	glvR	RpiR family transcriptional regulator, glv operon transcriptional regulator	--	--

[denovogenes]_111985	100.00	0.0	pdi:BDI_1841	K00991	ispD	2-C-methyl-D-erythritol 4-phosphate cytidylyltransferase	2.7.7.60	Metabolism--Metabolism of terpenoids and polyketides--Terpenoid backbone biosynthesis|Metabolism--Global and overview maps--Metabolic pathways|Metabolism--Global and overview maps--Biosynthesis of secondary metabolites|Metabolism--Global and overview maps--Biosynthesis of antibiotics

[denovogenes]_111986	100.00	0.0	eoh:ECO103_3297	K15342	cas1	CRISP-associated protein Cas1	--	--

[denovogenes]_111989	100.00	0.0	pvl:AOB99_11540	K15460	yfiC, trmX	tRNA1Val (adenine37-N6)-methyltransferase	2.1.1.223	--

[denovogenes]_11199	100.00	0.0	pvl:AOB99_04720	K03695	clpB	ATP-dependent Clp protease ATP-binding subunit ClpB	--	Organismal Systems--Aging--Longevity regulating pathway - multiple species

[denovogenes]_111993	66.53	3e-125	fnc:HMPREF0946_00736	K02013	ABC.FEV.A	iron complex transport system ATP-binding protein	3.6.3.34	Environmental Information Processing--Membrane transport--ABC transporters

[denovogenes]_111994	100.00	0.0	bvu:BVU_1961	K01647	CS, gltA	citrate synthase	2.3.3.1	Metabolism--Carbohydrate metabolism--Citrate cycle (TCA cycle)|Metabolism--Carbohydrate metabolism--Glyoxylate and dicarboxylate metabolism|Metabolism--Global and overview maps--Metabolic pathways|Metabolism--Global and overview maps--Biosynthesis of secondary metabolites|Metabolism--Global and overview maps--Microbial metabolism in diverse environments|Metabolism--Global and overview maps--Biosynthesis of antibiotics|Metabolism--Global and overview maps--Carbon metabolism|Metabolism--Global and overview maps--2-Oxocarboxylic acid metabolism|Metabolism--Global and overview maps--Biosynthesis of amino acids

[denovogenes]_111996	75.10	5e-133	mhg:MHY_01180	K02040	pstS	phosphate transport system substrate-binding protein	--	Environmental Information Processing--Membrane transport--ABC transporters|Environmental Information Processing--Signal transduction--Two-component system|Human Diseases--Infectious diseases: Bacterial--Tuberculosis

[denovogenes]_111997	84.98	8e-149	boa:Bovatus_01551	K02005	ABC.CD.TX	HlyD family secretion protein	--	--

[denovogenes]_111998	86.06	2e-155	vpr:Vpar_0369	K01933	purM	phosphoribosylformylglycinamidine cyclo-ligase	6.3.3.1	Metabolism--Nucleotide metabolism--Purine metabolism|Metabolism--Global and overview maps--Metabolic pathways|Metabolism--Global and overview maps--Biosynthesis of secondary metabolites|Metabolism--Global and overview maps--Biosynthesis of antibiotics

[denovogenes]_111999	100.00	0.0	cpf:CPF_2202	K07568	queA	S-adenosylmethionine:tRNA ribosyltransferase-isomerase	2.4.99.17	--

[denovogenes]_112001	100.00	0.0	eoc:CE10_2864	K12144	hyfI	hydrogenase-4 component I	1.-.-.-	--

[denovogenes]_112005	88.40	4e-161	ecas:ECBG_02256	K00789	metK	S-adenosylmethionine synthetase	2.5.1.6	Metabolism--Amino acid metabolism--Cysteine and methionine metabolism|Metabolism--Global and overview maps--Metabolic pathways|Metabolism--Global and overview maps--Biosynthesis of secondary metabolites|Metabolism--Global and overview maps--Biosynthesis of amino acids

[denovogenes]_112006	51.48	6e-79	pnl:PNK_1762	K18138	acrB, mexB, adeJ, smeE, mtrD, cmeB	multidrug efflux pump	--	Human Diseases--Drug resistance: Antimicrobial--beta-Lactam resistance|Human Diseases--Drug resistance: Antimicrobial--Cationic antimicrobial peptide (CAMP) resistance

[denovogenes]_112007	43.40	1e-59	bge:BC1002_0870	K07404	pgl	6-phosphogluconolactonase	3.1.1.31	Metabolism--Carbohydrate metabolism--Pentose phosphate pathway|Metabolism--Global and overview maps--Metabolic pathways|Metabolism--Global and overview maps--Biosynthesis of secondary metabolites|Metabolism--Global and overview maps--Microbial metabolism in diverse environments|Metabolism--Global and overview maps--Biosynthesis of antibiotics|Metabolism--Global and overview maps--Carbon metabolism

[denovogenes]_112008	83.33	7e-151	vpr:Vpar_0523	K02065	mlaF, linL, mkl	phospholipid/cholesterol/gamma-HCH transport system ATP-binding protein	--	Environmental Information Processing--Membrane transport--ABC transporters

[denovogenes]_112010	75.70	1e-141	ipo:Ilyop_2316	K00973	E2.7.7.24, rfbA, rffH	glucose-1-phosphate thymidylyltransferase	2.7.7.24	Metabolism--Biosynthesis of other secondary metabolites--Streptomycin biosynthesis|Metabolism--Metabolism of terpenoids and polyketides--Polyketide sugar unit biosynthesis|Metabolism--Biosynthesis of other secondary metabolites--Acarbose and validamycin biosynthesis|Metabolism--Global and overview maps--Biosynthesis of antibiotics

[denovogenes]_112011	100.00	0.0	pmr:PMI3576	K12975	eptB	KDO II ethanolaminephosphotransferase	2.7.8.42	Metabolism--Glycan biosynthesis and metabolism--Lipopolysaccharide biosynthesis|Human Diseases--Drug resistance: Antimicrobial--Cationic antimicrobial peptide (CAMP) resistance

[denovogenes]_112012	57.49	2e-86	banr:A16R_01130	K03046	rpoC	DNA-directed RNA polymerase subunit beta'	2.7.7.6	Metabolism--Nucleotide metabolism--Purine metabolism|Metabolism--Nucleotide metabolism--Pyrimidine metabolism|Metabolism--Global and overview maps--Metabolic pathways|Genetic Information Processing--Transcription--RNA polymerase

[denovogenes]_112015	98.81	0.0	cpe:CPE2148	K01270	pepD	dipeptidase D	3.4.13.-	Metabolism--Metabolism of other amino acids--Glutathione metabolism|Metabolism--Global and overview maps--Metabolic pathways

[denovogenes]_112017	99.21	0.0	eck:EC55989_3395	K11720	lptG	lipopolysaccharide export system permease protein	--	Environmental Information Processing--Membrane transport--ABC transporters

[denovogenes]_112018	75.10	2e-140	bcel:BcellWH2_01214	K01729	algL	poly(beta-D-mannuronate) lyase	4.2.2.3	Metabolism--Carbohydrate metabolism--Fructose and mannose metabolism

[denovogenes]_112022	47.04	6e-80	abg:Asbog_01754	K13013	wbqV	O-antigen biosynthesis protein WbqV	--	--

[denovogenes]_112023	84.46	1e-154	cbk:CLL_A0876	K03091	SIG3.4	RNA polymerase sporulation-specific sigma factor	--	--

[denovogenes]_112024	72.44	3e-131	cbz:Cbs_0835	K09761	rsmE	16S rRNA (uracil1498-N3)-methyltransferase	2.1.1.193	--

[denovogenes]_112025	99.60	0.0	mmk:MU9_1781	K02391	flgF	flagellar basal-body rod protein FlgF	--	Cellular Processes--Cell motility--Flagellar assembly

[denovogenes]_112028	82.47	8e-152	ecas:ECBG_01055	K00208	fabI	enoyl-[acyl-carrier protein] reductase I	1.3.1.9 1.3.1.10	Metabolism--Lipid metabolism--Fatty acid biosynthesis|Metabolism--Metabolism of cofactors and vitamins--Biotin metabolism|Metabolism--Global and overview maps--Metabolic pathways|Metabolism--Global and overview maps--Fatty acid metabolism

[denovogenes]_112029	62.55	2e-101	ehr:EHR_10735	K03436	fruR2, fruR	DeoR family transcriptional regulator, fructose operon transcriptional repressor	--	--

[denovogenes]_112032	56.05	2e-83	emu:EMQU_2105	K01922	PPCS, coaB	phosphopantothenate-cysteine ligase	6.3.2.5	Metabolism--Metabolism of cofactors and vitamins--Pantothenate and CoA biosynthesis|Metabolism--Global and overview maps--Metabolic pathways

[denovogenes]_112033	90.44	5e-168	ene:ENT_13020	K01803	TPI, tpiA	triosephosphate isomerase (TIM)	5.3.1.1	Metabolism--Carbohydrate metabolism--Glycolysis / Gluconeogenesis|Metabolism--Carbohydrate metabolism--Fructose and mannose metabolism|Metabolism--Carbohydrate metabolism--Inositol phosphate metabolism|Metabolism--Energy metabolism--Carbon fixation in photosynthetic organisms|Metabolism--Global and overview maps--Metabolic pathways|Metabolism--Global and overview maps--Biosynthesis of secondary metabolites|Metabolism--Global and overview maps--Microbial metabolism in diverse environments|Metabolism--Global and overview maps--Biosynthesis of antibiotics|Metabolism--Global and overview maps--Carbon metabolism|Metabolism--Global and overview maps--Biosynthesis of amino acids

[denovogenes]_112034	100.00	0.0	bfb:VU15_16145	K06201	cutC	copper homeostasis protein	--	--

[denovogenes]_112035	42.80	6e-67	nko:Niako_6782	K13002	wbyL	glycosyltransferase	2.4.1.-	--

[denovogenes]_112036	100.00	0.0	bfg:BF638R_0675	K03524	birA	BirA family transcriptional regulator, biotin operon repressor / biotin-[acetyl-CoA-carboxylase] ligase	6.3.4.15	Metabolism--Metabolism of cofactors and vitamins--Biotin metabolism|Metabolism--Global and overview maps--Metabolic pathways

[denovogenes]_112037	100.00	9e-175	mmk:MU9_2728	K00059	fabG	3-oxoacyl-[acyl-carrier protein] reductase	1.1.1.100	Metabolism--Lipid metabolism--Fatty acid biosynthesis|Metabolism--Metabolism of cofactors and vitamins--Biotin metabolism|Metabolism--Lipid metabolism--Biosynthesis of unsaturated fatty acids|Metabolism--Global and overview maps--Metabolic pathways|Metabolism--Global and overview maps--Fatty acid metabolism

[denovogenes]_112038	69.72	1e-132	cpr:CPR_1117	K02013	ABC.FEV.A	iron complex transport system ATP-binding protein	3.6.3.34	Environmental Information Processing--Membrane transport--ABC transporters

[denovogenes]_112039	60.16	2e-103	csb:CLSA_c12820	K02050	ABC.SN.P	NitT/TauT family transport system permease protein	--	--

[denovogenes]_11204	83.95	0.0	btho:Btheta7330_02902	K12373	HEXA_B	hexosaminidase	3.2.1.52	Metabolism--Glycan biosynthesis and metabolism--Other glycan degradation|Metabolism--Glycan biosynthesis and metabolism--Various types of N-glycan biosynthesis|Metabolism--Carbohydrate metabolism--Amino sugar and nucleotide sugar metabolism|Metabolism--Glycan biosynthesis and metabolism--Glycosaminoglycan degradation|Metabolism--Glycan biosynthesis and metabolism--Glycosphingolipid biosynthesis - globo and isoglobo series|Metabolism--Glycan biosynthesis and metabolism--Glycosphingolipid biosynthesis - ganglio series|Metabolism--Global and overview maps--Metabolic pathways|Cellular Processes--Transport and catabolism--Lysosome

[denovogenes]_112042	82.07	6e-159	bprs:CK3_00480	K01142	E3.1.11.2, xthA	exodeoxyribonuclease III	3.1.11.2	Genetic Information Processing--Replication and repair--Base excision repair

[denovogenes]_112045	43.61	2e-58	pku:AUO94_13515	K16785	ecfT	energy-coupling factor transport system permease protein	--	Environmental Information Processing--Membrane transport--ABC transporters

[denovogenes]_112046	63.97	3e-108	eft:M395_00685	K00059	fabG	3-oxoacyl-[acyl-carrier protein] reductase	1.1.1.100	Metabolism--Lipid metabolism--Fatty acid biosynthesis|Metabolism--Metabolism of cofactors and vitamins--Biotin metabolism|Metabolism--Lipid metabolism--Biosynthesis of unsaturated fatty acids|Metabolism--Global and overview maps--Metabolic pathways|Metabolism--Global and overview maps--Fatty acid metabolism

[denovogenes]_112049	73.71	3e-134	cbl:CLK_0494	K02013	ABC.FEV.A	iron complex transport system ATP-binding protein	3.6.3.34	Environmental Information Processing--Membrane transport--ABC transporters

[denovogenes]_112050	62.65	1e-111	lacy:A4V08_31165	K02421	fliR	flagellar biosynthetic protein FliR	--	Cellular Processes--Cell motility--Flagellar assembly

[denovogenes]_112051	79.60	2e-153	csh:Closa_4122	K01142	E3.1.11.2, xthA	exodeoxyribonuclease III	3.1.11.2	Genetic Information Processing--Replication and repair--Base excision repair

[denovogenes]_112053	100.00	0.0	mmk:MU9_756	K00757	udp, UPP	uridine phosphorylase	2.4.2.3	Metabolism--Nucleotide metabolism--Pyrimidine metabolism|Metabolism--Xenobiotics biodegradation and metabolism--Drug metabolism - other enzymes|Metabolism--Global and overview maps--Metabolic pathways

[denovogenes]_112054	100.00	4e-180	mmk:MU9_765	K03118	tatC	sec-independent protein translocase protein TatC	--	Genetic Information Processing--Folding, sorting and degradation--Protein export|Environmental Information Processing--Membrane transport--Bacterial secretion system

[denovogenes]_112055	56.02	2e-82	cbv:U729_1481	K02654	pilD, pppA	leader peptidase (prepilin peptidase) / N-methyltransferase	3.4.23.43 2.1.1.-	--

[denovogenes]_112056	74.19	3e-143	cbut:ATN24_08260	K00215	dapB	4-hydroxy-tetrahydrodipicolinate reductase	1.17.1.8	Metabolism--Biosynthesis of other secondary metabolites--Monobactam biosynthesis|Metabolism--Amino acid metabolism--Lysine biosynthesis|Metabolism--Global and overview maps--Metabolic pathways|Metabolism--Global and overview maps--Biosynthesis of secondary metabolites|Metabolism--Global and overview maps--Microbial metabolism in diverse environments|Metabolism--Global and overview maps--Biosynthesis of antibiotics|Metabolism--Global and overview maps--Biosynthesis of amino acids

[denovogenes]_112058	51.95	4e-87	csr:Cspa_c11930	K01104	E3.1.3.48	protein-tyrosine phosphatase	3.1.3.48	--

[denovogenes]_112059	100.00	0.0	bfb:VU15_21135	K00240	sdhB, frdB	succinate dehydrogenase / fumarate reductase, iron-sulfur subunit	1.3.5.1 1.3.5.4	Metabolism--Carbohydrate metabolism--Citrate cycle (TCA cycle)|Metabolism--Energy metabolism--Oxidative phosphorylation|Metabolism--Carbohydrate metabolism--Butanoate metabolism|Metabolism--Energy metabolism--Carbon fixation pathways in prokaryotes|Metabolism--Global and overview maps--Metabolic pathways|Metabolism--Global and overview maps--Biosynthesis of secondary metabolites|Metabolism--Global and overview maps--Microbial metabolism in diverse environments|Metabolism--Global and overview maps--Biosynthesis of antibiotics|Metabolism--Global and overview maps--Carbon metabolism

[denovogenes]_11206	41.47	8e-154	pft:JBW_00429	K00705	malQ	4-alpha-glucanotransferase	2.4.1.25	Metabolism--Carbohydrate metabolism--Starch and sucrose metabolism|Metabolism--Global and overview maps--Metabolic pathways

[denovogenes]_112060	99.60	0.0	ene:ENT_18990	K01142	E3.1.11.2, xthA	exodeoxyribonuclease III	3.1.11.2	Genetic Information Processing--Replication and repair--Base excision repair

[denovogenes]_112061	99.18	0.0	efn:DENG_00352	K01071	MCH	medium-chain acyl-[acyl-carrier-protein] hydrolase	3.1.2.21	Metabolism--Lipid metabolism--Fatty acid biosynthesis|Metabolism--Global and overview maps--Metabolic pathways

[denovogenes]_112063	65.74	5e-124	cbb:CLD_3510	K02013	ABC.FEV.A	iron complex transport system ATP-binding protein	3.6.3.34	Environmental Information Processing--Membrane transport--ABC transporters

[denovogenes]_112064	80.16	4e-145	pdf:CD630DERM_31720	K01803	TPI, tpiA	triosephosphate isomerase (TIM)	5.3.1.1	Metabolism--Carbohydrate metabolism--Glycolysis / Gluconeogenesis|Metabolism--Carbohydrate metabolism--Fructose and mannose metabolism|Metabolism--Carbohydrate metabolism--Inositol phosphate metabolism|Metabolism--Energy metabolism--Carbon fixation in photosynthetic organisms|Metabolism--Global and overview maps--Metabolic pathways|Metabolism--Global and overview maps--Biosynthesis of secondary metabolites|Metabolism--Global and overview maps--Microbial metabolism in diverse environments|Metabolism--Global and overview maps--Biosynthesis of antibiotics|Metabolism--Global and overview maps--Carbon metabolism|Metabolism--Global and overview maps--Biosynthesis of amino acids

[denovogenes]_112065	84.46	2e-154	cbt:CLH_0843	K03091	SIG3.4	RNA polymerase sporulation-specific sigma factor	--	--

[denovogenes]_112066	71.60	4e-127	lacy:A4V08_25585	K07090	K07090	uncharacterized protein	--	--

[denovogenes]_112067	100.00	0.0	bfb:VU15_00070	K02477	K02477	two-component system, LytT family, response regulator	--	--

[denovogenes]_112068	86.06	7e-167	lacy:A4V08_19120	K01142	E3.1.11.2, xthA	exodeoxyribonuclease III	3.1.11.2	Genetic Information Processing--Replication and repair--Base excision repair

[denovogenes]_112069	100.00	0.0	ses:SARI_01486	K07043	K07043	uncharacterized protein	--	--

[denovogenes]_11207	93.42	0.0	lacy:A4V08_13000	K02538	manR	activator of the mannose operon, transcriptional antiterminator	--	--

[denovogenes]_112070	73.06	3e-133	cdg:CDBI1_03825	K01916	nadE	NAD+ synthase	6.3.1.5	Metabolism--Metabolism of cofactors and vitamins--Nicotinate and nicotinamide metabolism|Metabolism--Global and overview maps--Metabolic pathways

[denovogenes]_112072	41.09	5e-22	mve:X875_6670	K21029	moeB	molybdopterin-synthase adenylyltransferase	2.7.7.80	Genetic Information Processing--Folding, sorting and degradation--Sulfur relay system

[denovogenes]_112073	68.92	9e-129	cbj:H04402_01124	K02013	ABC.FEV.A	iron complex transport system ATP-binding protein	3.6.3.34	Environmental Information Processing--Membrane transport--ABC transporters

[denovogenes]_112074	49.38	9e-71	sgo:SGO_0221	K01409	KAE1, tsaD, QRI7	N6-L-threonylcarbamoyladenine synthase	2.3.1.234	--

[denovogenes]_112075	99.20	0.0	stb:SGPB_0024	K03584	recO	DNA repair protein RecO (recombination protein O)	--	Genetic Information Processing--Replication and repair--Homologous recombination

[denovogenes]_112077	43.31	1e-61	cba:CLB_2447	K01358	clpP, CLPP	ATP-dependent Clp protease, protease subunit	3.4.21.92	Cellular Processes--Cell growth and death--Cell cycle - Caulobacter|Organismal Systems--Aging--Longevity regulating pathway - worm

[denovogenes]_112078	99.60	0.0	stb:SGPB_0268	K16511	mecA1_2	adapter protein MecA 1/2	--	--

[denovogenes]_112079	71.85	6e-125	ega:AL523_04970	K03501	gidB, rsmG	16S rRNA (guanine527-N7)-methyltransferase	2.1.1.170	--

[denovogenes]_112081	51.63	4e-88	ega:AL523_09810	K03785	aroD	3-dehydroquinate dehydratase I	4.2.1.10	Metabolism--Amino acid metabolism--Phenylalanine, tyrosine and tryptophan biosynthesis|Metabolism--Global and overview maps--Metabolic pathways|Metabolism--Global and overview maps--Biosynthesis of secondary metabolites|Metabolism--Global and overview maps--Biosynthesis of antibiotics|Metabolism--Global and overview maps--Biosynthesis of amino acids

[denovogenes]_112083	60.80	3e-113	cbk:CLL_A1404	K02013	ABC.FEV.A	iron complex transport system ATP-binding protein	3.6.3.34	Environmental Information Processing--Membrane transport--ABC transporters

[denovogenes]_112084	60.40	1e-110	tpi:TREPR_0540	K02013	ABC.FEV.A	iron complex transport system ATP-binding protein	3.6.3.34	Environmental Information Processing--Membrane transport--ABC transporters

[denovogenes]_112087	64.14	2e-116	csq:CSCA_2214	K02003	ABC.CD.A	putative ABC transport system ATP-binding protein	--	--

[denovogenes]_112088	67.87	7e-116	clb:Clo1100_2641	K06898	K06898	uncharacterized protein	--	--

[denovogenes]_11209	93.26	0.0	fpla:A4U99_17715	K03572	mutL	DNA mismatch repair protein MutL	--	Genetic Information Processing--Replication and repair--Mismatch repair

[denovogenes]_112090	72.65	5e-142	cdg:CDBI1_14915	K07010	K07010	putative glutamine amidotransferase	--	--

[denovogenes]_112093	67.07	6e-121	cbut:ATN24_07055	K00059	fabG	3-oxoacyl-[acyl-carrier protein] reductase	1.1.1.100	Metabolism--Lipid metabolism--Fatty acid biosynthesis|Metabolism--Metabolism of cofactors and vitamins--Biotin metabolism|Metabolism--Lipid metabolism--Biosynthesis of unsaturated fatty acids|Metabolism--Global and overview maps--Metabolic pathways|Metabolism--Global and overview maps--Fatty acid metabolism

[denovogenes]_112094	76.89	1e-134	sags:SaSA20_0911	K00625	E2.3.1.8, pta	phosphate acetyltransferase	2.3.1.8	Metabolism--Metabolism of other amino acids--Taurine and hypotaurine metabolism|Metabolism--Carbohydrate metabolism--Pyruvate metabolism|Metabolism--Carbohydrate metabolism--Propanoate metabolism|Metabolism--Energy metabolism--Methane metabolism|Metabolism--Energy metabolism--Carbon fixation pathways in prokaryotes|Metabolism--Global and overview maps--Metabolic pathways|Metabolism--Global and overview maps--Microbial metabolism in diverse environments|Metabolism--Global and overview maps--Carbon metabolism

[denovogenes]_112095	53.88	9e-83	tap:GZ22_03265	K03489	yydK	GntR family transcriptional regulator, transcriptional regulator of bglA	--	--

[denovogenes]_112097	69.60	7e-124	cct:CC1_29430	K03785	aroD	3-dehydroquinate dehydratase I	4.2.1.10	Metabolism--Amino acid metabolism--Phenylalanine, tyrosine and tryptophan biosynthesis|Metabolism--Global and overview maps--Metabolic pathways|Metabolism--Global and overview maps--Biosynthesis of secondary metabolites|Metabolism--Global and overview maps--Biosynthesis of antibiotics|Metabolism--Global and overview maps--Biosynthesis of amino acids

[denovogenes]_112098	100.00	0.0	ene:ENT_13020	K01803	TPI, tpiA	triosephosphate isomerase (TIM)	5.3.1.1	Metabolism--Carbohydrate metabolism--Glycolysis / Gluconeogenesis|Metabolism--Carbohydrate metabolism--Fructose and mannose metabolism|Metabolism--Carbohydrate metabolism--Inositol phosphate metabolism|Metabolism--Energy metabolism--Carbon fixation in photosynthetic organisms|Metabolism--Global and overview maps--Metabolic pathways|Metabolism--Global and overview maps--Biosynthesis of secondary metabolites|Metabolism--Global and overview maps--Microbial metabolism in diverse environments|Metabolism--Global and overview maps--Biosynthesis of antibiotics|Metabolism--Global and overview maps--Carbon metabolism|Metabolism--Global and overview maps--Biosynthesis of amino acids

[denovogenes]_112099	53.31	1e-84	etd:ETAF_2037	K12519	papD	chaperone protein PapD	--	--

[denovogenes]_112101	100.00	0.0	ene:ENT_28320	K02013	ABC.FEV.A	iron complex transport system ATP-binding protein	3.6.3.34	Environmental Information Processing--Membrane transport--ABC transporters

[denovogenes]_112102	91.24	1e-172	cpe:CPE0757	K01470	E3.5.2.10	creatinine amidohydrolase	3.5.2.10	Metabolism--Amino acid metabolism--Arginine and proline metabolism

[denovogenes]_112107	100.00	0.0	pvl:AOB99_15795	K00059	fabG	3-oxoacyl-[acyl-carrier protein] reductase	1.1.1.100	Metabolism--Lipid metabolism--Fatty acid biosynthesis|Metabolism--Metabolism of cofactors and vitamins--Biotin metabolism|Metabolism--Lipid metabolism--Biosynthesis of unsaturated fatty acids|Metabolism--Global and overview maps--Metabolic pathways|Metabolism--Global and overview maps--Fatty acid metabolism

[denovogenes]_112108	93.60	3e-164	cfd:CFNIH1_14010	K02169	bioC	malonyl-CoA O-methyltransferase	2.1.1.197	Metabolism--Metabolism of cofactors and vitamins--Biotin metabolism|Metabolism--Global and overview maps--Metabolic pathways

[denovogenes]_112109	93.20	4e-153	cfd:CFNIH1_15020	K07090	K07090	uncharacterized protein	--	--

[denovogenes]_11211	100.00	0.0	pdi:BDI_1726	K00700	GBE1, glgB	1,4-alpha-glucan branching enzyme	2.4.1.18	Metabolism--Carbohydrate metabolism--Starch and sucrose metabolism|Metabolism--Global and overview maps--Metabolic pathways|Metabolism--Global and overview maps--Biosynthesis of secondary metabolites

[denovogenes]_112112	59.11	9e-89	amt:Amet_1442	K00655	plsC	1-acyl-sn-glycerol-3-phosphate acyltransferase	2.3.1.51	Metabolism--Lipid metabolism--Glycerolipid metabolism|Metabolism--Lipid metabolism--Glycerophospholipid metabolism|Metabolism--Global and overview maps--Metabolic pathways|Metabolism--Global and overview maps--Biosynthesis of secondary metabolites

[denovogenes]_112113	49.38	2e-85	cls:CXIVA_11480	K15460	yfiC, trmX	tRNA1Val (adenine37-N6)-methyltransferase	2.1.1.223	--

[denovogenes]_112114	100.00	0.0	pvl:AOB99_10205	K02391	flgF	flagellar basal-body rod protein FlgF	--	Cellular Processes--Cell motility--Flagellar assembly

[denovogenes]_112115	58.40	9e-99	cbt:CLH_2219	K05794	terC	tellurite resistance protein TerC	--	--

[denovogenes]_112116	48.37	3e-82	amt:Amet_0090	K15460	yfiC, trmX	tRNA1Val (adenine37-N6)-methyltransferase	2.1.1.223	--

[denovogenes]_112117	55.69	1e-96	dhd:Dhaf_0239	K09861	K09861	uncharacterized protein	--	--

[denovogenes]_112118	100.00	0.0	pmib:BB2000_3220	K00059	fabG	3-oxoacyl-[acyl-carrier protein] reductase	1.1.1.100	Metabolism--Lipid metabolism--Fatty acid biosynthesis|Metabolism--Metabolism of cofactors and vitamins--Biotin metabolism|Metabolism--Lipid metabolism--Biosynthesis of unsaturated fatty acids|Metabolism--Global and overview maps--Metabolic pathways|Metabolism--Global and overview maps--Fatty acid metabolism

[denovogenes]_112119	75.30	8e-141	vpr:Vpar_0002	K02338	DPO3B, dnaN	DNA polymerase III subunit beta	2.7.7.7	Metabolism--Nucleotide metabolism--Purine metabolism|Metabolism--Nucleotide metabolism--Pyrimidine metabolism|Metabolism--Global and overview maps--Metabolic pathways|Genetic Information Processing--Replication and repair--DNA replication|Genetic Information Processing--Replication and repair--Mismatch repair|Genetic Information Processing--Replication and repair--Homologous recombination

[denovogenes]_112122	95.22	2e-179	cfd:CFNIH1_00930	K01146	xni	protein Xni	--	--

[denovogenes]_112126	100.00	0.0	pvl:AOB99_05580	K03750	moeA	molybdopterin molybdotransferase	2.10.1.1	Metabolism--Metabolism of cofactors and vitamins--Folate biosynthesis|Metabolism--Global and overview maps--Metabolic pathways

[denovogenes]_112127	100.00	0.0	pvl:AOB99_09645	K02020	modA	molybdate transport system substrate-binding protein	--	Environmental Information Processing--Membrane transport--ABC transporters

[denovogenes]_112128	100.00	0.0	cbut:ATN24_05350	K03086	SIG1, rpoD	RNA polymerase primary sigma factor	--	--

[denovogenes]_112129	100.00	0.0	aby:ABAYE3619	K18792	blaOXA-10	beta-lactamase class D OXA-10	3.5.2.6	Human Diseases--Drug resistance: Antimicrobial--beta-Lactam resistance

[denovogenes]_112134	84.06	1e-149	pdf:CD630DERM_01170	K00175	korB, oorB, oforB	2-oxoglutarate/2-oxoacid ferredoxin oxidoreductase subunit beta	1.2.7.3 1.2.7.11	Metabolism--Carbohydrate metabolism--Glycolysis / Gluconeogenesis|Metabolism--Carbohydrate metabolism--Citrate cycle (TCA cycle)|Metabolism--Carbohydrate metabolism--Pyruvate metabolism|Metabolism--Carbohydrate metabolism--Butanoate metabolism|Metabolism--Energy metabolism--Carbon fixation pathways in prokaryotes|Metabolism--Global and overview maps--Metabolic pathways|Metabolism--Global and overview maps--Microbial metabolism in diverse environments|Metabolism--Global and overview maps--Biosynthesis of antibiotics|Metabolism--Global and overview maps--Carbon metabolism

[denovogenes]_112136	99.60	0.0	pmib:BB2000_3161	K00989	rph	ribonuclease PH	2.7.7.56	--

[denovogenes]_112137	100.00	0.0	pmib:BB2000_3437	K03841	FBP, fbp	fructose-1,6-bisphosphatase I	3.1.3.11	Metabolism--Carbohydrate metabolism--Glycolysis / Gluconeogenesis|Metabolism--Carbohydrate metabolism--Pentose phosphate pathway|Metabolism--Carbohydrate metabolism--Fructose and mannose metabolism|Metabolism--Energy metabolism--Methane metabolism|Metabolism--Energy metabolism--Carbon fixation in photosynthetic organisms|Metabolism--Global and overview maps--Metabolic pathways|Metabolism--Global and overview maps--Biosynthesis of secondary metabolites|Metabolism--Global and overview maps--Microbial metabolism in diverse environments|Metabolism--Global and overview maps--Biosynthesis of antibiotics|Metabolism--Global and overview maps--Carbon metabolism|Environmental Information Processing--Signal transduction--AMPK signaling pathway|Organismal Systems--Endocrine system--Insulin signaling pathway

[denovogenes]_112138	93.64	1e-159	eclc:ECR091_01650	K05775	malM	maltose operon periplasmic protein	--	--

[denovogenes]_112139	99.20	0.0	cfd:CFNIH1_08585	K03477	ulaR	DeoR family transcriptional regulator, ulaG and ulaABCDEF operon transcriptional repressor	--	--

[denovogenes]_112141	99.20	0.0	bhl:Bache_0805	K00240	sdhB, frdB	succinate dehydrogenase / fumarate reductase, iron-sulfur subunit	1.3.5.1 1.3.5.4	Metabolism--Carbohydrate metabolism--Citrate cycle (TCA cycle)|Metabolism--Energy metabolism--Oxidative phosphorylation|Metabolism--Carbohydrate metabolism--Butanoate metabolism|Metabolism--Energy metabolism--Carbon fixation pathways in prokaryotes|Metabolism--Global and overview maps--Metabolic pathways|Metabolism--Global and overview maps--Biosynthesis of secondary metabolites|Metabolism--Global and overview maps--Microbial metabolism in diverse environments|Metabolism--Global and overview maps--Biosynthesis of antibiotics|Metabolism--Global and overview maps--Carbon metabolism

[denovogenes]_112142	82.14	2e-152	cbz:Cbs_4359	K07406	melA	alpha-galactosidase	3.2.1.22	Metabolism--Carbohydrate metabolism--Galactose metabolism|Metabolism--Lipid metabolism--Glycerolipid metabolism|Metabolism--Lipid metabolism--Sphingolipid metabolism|Metabolism--Glycan biosynthesis and metabolism--Glycosphingolipid biosynthesis - globo and isoglobo series

[denovogenes]_112144	100.00	0.0	ecoh:ECRM13516_1717	K19802	ycjG, ykfB	L-Ala-D/L-Glu epimerase	5.1.1.20	--

[denovogenes]_112145	85.31	4e-159	cad:Curi_c18560	K01886	QARS, glnS	glutaminyl-tRNA synthetase	6.1.1.18	Genetic Information Processing--Translation--Aminoacyl-tRNA biosynthesis|Metabolism--Global and overview maps--Metabolic pathways

[denovogenes]_112147	99.60	0.0	efe:EFER_0241	K01069	E3.1.2.6, gloB	hydroxyacylglutathione hydrolase	3.1.2.6	Metabolism--Carbohydrate metabolism--Pyruvate metabolism

[denovogenes]_112148	70.52	1e-129	pdf:CD630DERM_16490	K02013	ABC.FEV.A	iron complex transport system ATP-binding protein	3.6.3.34	Environmental Information Processing--Membrane transport--ABC transporters

[denovogenes]_112149	93.23	6e-169	cfd:CFNIH1_18915	K01657	trpE	anthranilate synthase component I	4.1.3.27	Metabolism--Amino acid metabolism--Phenylalanine, tyrosine and tryptophan biosynthesis|Metabolism--Global and overview maps--Metabolic pathways|Metabolism--Global and overview maps--Biosynthesis of secondary metabolites|Metabolism--Global and overview maps--Biosynthesis of antibiotics|Metabolism--Global and overview maps--Biosynthesis of amino acids|Cellular Processes--Cellular community - prokaryotes--Quorum sensing|Cellular Processes--Cellular community - prokaryotes--Biofilm formation - Pseudomonas aeruginosa

[denovogenes]_11215	90.18	0.0	pdi:BDI_1726	K00700	GBE1, glgB	1,4-alpha-glucan branching enzyme	2.4.1.18	Metabolism--Carbohydrate metabolism--Starch and sucrose metabolism|Metabolism--Global and overview maps--Metabolic pathways|Metabolism--Global and overview maps--Biosynthesis of secondary metabolites

[denovogenes]_112150	86.45	3e-163	vpr:Vpar_1553	K01142	E3.1.11.2, xthA	exodeoxyribonuclease III	3.1.11.2	Genetic Information Processing--Replication and repair--Base excision repair

[denovogenes]_112151	70.52	1e-136	cbk:CLL_A1404	K02013	ABC.FEV.A	iron complex transport system ATP-binding protein	3.6.3.34	Environmental Information Processing--Membrane transport--ABC transporters

[denovogenes]_112153	45.54	1e-13	aam:106496646	K19721	COL5AS	collagen, type V/XI/XXIV/XXVII, alpha	--	Organismal Systems--Digestive system--Protein digestion and absorption

[denovogenes]_112154	52.96	2e-91	cbt:CLH_0489	K02115	ATPF1G, atpG	F-type H+-transporting ATPase subunit gamma	--	Metabolism--Energy metabolism--Oxidative phosphorylation|Metabolism--Energy metabolism--Photosynthesis|Metabolism--Global and overview maps--Metabolic pathways

[denovogenes]_112155	50.21	3e-80	nth:Nther_1461	K01358	clpP, CLPP	ATP-dependent Clp protease, protease subunit	3.4.21.92	Cellular Processes--Cell growth and death--Cell cycle - Caulobacter|Organismal Systems--Aging--Longevity regulating pathway - worm

[denovogenes]_112156	100.00	0.0	pvl:AOB99_08140	K01754	E4.3.1.19, ilvA, tdcB	threonine dehydratase	4.3.1.19	Metabolism--Amino acid metabolism--Glycine, serine and threonine metabolism|Metabolism--Amino acid metabolism--Valine, leucine and isoleucine biosynthesis|Metabolism--Global and overview maps--Metabolic pathways|Metabolism--Global and overview maps--Biosynthesis of secondary metabolites|Metabolism--Global and overview maps--Biosynthesis of antibiotics|Metabolism--Global and overview maps--Carbon metabolism|Metabolism--Global and overview maps--Biosynthesis of amino acids

[denovogenes]_11216	52.57	0.0	dfi:AXF13_07900	K03404	chlD, bchD	magnesium chelatase subunit D	6.6.1.1	Metabolism--Metabolism of cofactors and vitamins--Porphyrin and chlorophyll metabolism|Metabolism--Global and overview maps--Metabolic pathways|Metabolism--Global and overview maps--Biosynthesis of secondary metabolites

[denovogenes]_112160	40.82	2e-56	puf:UFO1_1025	K09815	znuA	zinc transport system substrate-binding protein	--	Environmental Information Processing--Membrane transport--ABC transporters

[denovogenes]_112161	89.29	6e-165	prg:RB151_007210	K00946	thiL	thiamine-monophosphate kinase	2.7.4.16	Metabolism--Metabolism of cofactors and vitamins--Thiamine metabolism|Metabolism--Global and overview maps--Metabolic pathways

[denovogenes]_112163	100.00	0.0	pmib:BB2000_3475	K01992	ABC-2.P	ABC-2 type transport system permease protein	--	--

[denovogenes]_112164	91.63	5e-167	btho:Btheta7330_04025	K03496	parA, soj	chromosome partitioning protein	--	--

[denovogenes]_112165	62.30	3e-109	pdf:CD630DERM_31750	K05311	cggR	central glycolytic genes regulator	--	--

[denovogenes]_112166	88.05	9e-152	clt:CM240_2972	K15554	ssuC	sulfonate transport system permease protein	--	Metabolism--Energy metabolism--Sulfur metabolism|Environmental Information Processing--Membrane transport--ABC transporters

[denovogenes]_112169	92.77	8e-170	psx:DR96_3505	K06179	rluC	23S rRNA pseudouridine955/2504/2580 synthase	5.4.99.24	--

[denovogenes]_112170	90.44	3e-158	sbz:A464_3848	K03445	nepI	MFS transporter, DHA1 family, purine ribonucleoside efflux pump	--	--

[denovogenes]_112172	54.00	3e-88	clt:CM240_1151	K02233	E2.7.8.26, cobS, cobV	adenosylcobinamide-GDP ribazoletransferase	2.7.8.26	Metabolism--Metabolism of cofactors and vitamins--Porphyrin and chlorophyll metabolism|Metabolism--Global and overview maps--Metabolic pathways

[denovogenes]_112173	40.94	3e-55	mtc:MT1753.1	K00065	kduD	2-deoxy-D-gluconate 3-dehydrogenase	1.1.1.125	Metabolism--Carbohydrate metabolism--Pentose and glucuronate interconversions

[denovogenes]_112174	97.22	2e-178	prg:RB151_003030	K06207	typA, bipA	GTP-binding protein	--	--

[denovogenes]_112175	80.95	1e-135	clt:CM240_2981	K02015	ABC.FEV.P	iron complex transport system permease protein	--	Environmental Information Processing--Membrane transport--ABC transporters

[denovogenes]_112176	81.67	2e-144	psi:S70_13605	K00112	glpB	glycerol-3-phosphate dehydrogenase subunit B	1.1.5.3	Metabolism--Lipid metabolism--Glycerophospholipid metabolism|Metabolism--Global and overview maps--Biosynthesis of secondary metabolites

[denovogenes]_112177	50.60	1e-79	lra:LRHK_1854	K04759	feoB	ferrous iron transport protein B	--	--

[denovogenes]_112178	98.41	0.0	cfd:CFNIH1_11625	K09470	puuA	gamma-glutamylputrescine synthase	6.3.1.11	Metabolism--Amino acid metabolism--Arginine and proline metabolism|Metabolism--Global and overview maps--Metabolic pathways

[denovogenes]_112182	100.00	0.0	ecoh:ECRM13516_0822	K01992	ABC-2.P	ABC-2 type transport system permease protein	--	--

[denovogenes]_112183	96.43	0.0	psx:DR96_3424	K08303	K08303	putative protease	3.4.-.-	Human Diseases--Infectious diseases: Bacterial--Epithelial cell signaling in Helicobacter pylori infection

[denovogenes]_112185	99.21	0.0	ecp:ECP_2359	K03473	pdxB	erythronate-4-phosphate dehydrogenase	1.1.1.290	Metabolism--Metabolism of cofactors and vitamins--Vitamin B6 metabolism|Metabolism--Global and overview maps--Metabolic pathways

[denovogenes]_112187	51.59	1e-93	amt:Amet_3295	K02055	ABC.SP.S	putative spermidine/putrescine transport system substrate-binding protein	--	Cellular Processes--Cellular community - prokaryotes--Quorum sensing

[denovogenes]_112189	73.41	2e-135	cdg:CDBI1_12920	K01929	murF	UDP-N-acetylmuramoyl-tripeptide--D-alanyl-D-alanine ligase	6.3.2.10	Metabolism--Amino acid metabolism--Lysine biosynthesis|Metabolism--Glycan biosynthesis and metabolism--Peptidoglycan biosynthesis|Metabolism--Global and overview maps--Metabolic pathways|Human Diseases--Drug resistance: Antimicrobial--Vancomycin resistance

[denovogenes]_11219	99.40	0.0	paf:PAM18_3516	K02343	DPO3G, dnaX	DNA polymerase III subunit gamma/tau	2.7.7.7	Metabolism--Nucleotide metabolism--Purine metabolism|Metabolism--Nucleotide metabolism--Pyrimidine metabolism|Metabolism--Global and overview maps--Metabolic pathways|Genetic Information Processing--Replication and repair--DNA replication|Genetic Information Processing--Replication and repair--Mismatch repair|Genetic Information Processing--Replication and repair--Homologous recombination

[denovogenes]_112191	40.52	1e-33	mhg:MHY_04830	K08384	spoVD	stage V sporulation protein D (sporulation-specific penicillin-binding protein)	--	--

[denovogenes]_112192	48.41	7e-78	pdm:ADU72_0128	K07497	K07497	putative transposase	--	--

[denovogenes]_112193	69.44	7e-128	aoe:Clos_0626	K00128	--	--	--	Metabolism--Carbohydrate metabolism--Glycolysis / Gluconeogenesis|Metabolism--Carbohydrate metabolism--Ascorbate and aldarate metabolism|Metabolism--Lipid metabolism--Fatty acid degradation|Metabolism--Amino acid metabolism--Valine, leucine and isoleucine degradation|Metabolism--Amino acid metabolism--Lysine degradation|Metabolism--Amino acid metabolism--Arginine and proline metabolism|Metabolism--Amino acid metabolism--Histidine metabolism|Metabolism--Amino acid metabolism--Tryptophan metabolism|Metabolism--Metabolism of other amino acids--beta-Alanine metabolism|Metabolism--Lipid metabolism--Glycerolipid metabolism|Metabolism--Carbohydrate metabolism--Pyruvate metabolism|Metabolism--Xenobiotics biodegradation and metabolism--Chloroalkane and chloroalkene degradation|Metabolism--Metabolism of terpenoids and polyketides--Limonene and pinene degradation|Metabolism--Metabolism of terpenoids and polyketides--Insect hormone biosynthesis|Metabolism--Global and overview maps--Metabolic pathways|Metabolism--Global and overview maps--Biosynthesis of secondary metabolites|Metabolism--Global and overview maps--Microbial metabolism in diverse environments|Metabolism--Global and overview maps--Biosynthesis of antibiotics

[denovogenes]_112194	76.98	3e-145	bfi:CIY_15190	K01915	glnA, GLUL	glutamine synthetase	6.3.1.2	Metabolism--Amino acid metabolism--Arginine biosynthesis|Metabolism--Amino acid metabolism--Alanine, aspartate and glutamate metabolism|Metabolism--Carbohydrate metabolism--Glyoxylate and dicarboxylate metabolism|Metabolism--Energy metabolism--Nitrogen metabolism|Metabolism--Global and overview maps--Metabolic pathways|Metabolism--Global and overview maps--Microbial metabolism in diverse environments|Metabolism--Global and overview maps--Biosynthesis of amino acids|Environmental Information Processing--Signal transduction--Two-component system|Organismal Systems--Nervous system--Glutamatergic synapse|Organismal Systems--Nervous system--GABAergic synapse

[denovogenes]_112196	100.00	0.0	pvl:AOB99_03610	K14588	cueO	blue copper oxidase	--	--

[denovogenes]_112197	44.14	1e-59	bdo:EL88_16755	K05349	bglX	beta-glucosidase	3.2.1.21	Metabolism--Metabolism of other amino acids--Cyanoamino acid metabolism|Metabolism--Carbohydrate metabolism--Starch and sucrose metabolism|Metabolism--Biosynthesis of other secondary metabolites--Phenylpropanoid biosynthesis|Metabolism--Global and overview maps--Metabolic pathways|Metabolism--Global and overview maps--Biosynthesis of secondary metabolites

[denovogenes]_112198	62.75	2e-112	puf:UFO1_2230	K06898	K06898	uncharacterized protein	--	--

[denovogenes]_112199	95.56	6e-176	bhl:Bache_0514	K09013	sufC	Fe-S cluster assembly ATP-binding protein	--	--

[denovogenes]_1122	76.56	0.0	lacy:A4V08_00985	K03529	smc	chromosome segregation protein	--	--

[denovogenes]_11220	98.96	0.0	ecol:LY180_12730	K12137	hyfB	hydrogenase-4 component B	1.-.-.-	--

[denovogenes]_112200	75.21	7e-131	rob:CK5_20950	K07009	K07009	uncharacterized protein	--	--

[denovogenes]_112202	62.25	4e-112	bal:BACI_c31330	K02003	ABC.CD.A	putative ABC transport system ATP-binding protein	--	--

[denovogenes]_112203	86.06	4e-156	ruk:A4V00_18335	K20459	nukF, mcdF, sboF	lantibiotic transport system ATP-binding protein	--	Environmental Information Processing--Membrane transport--ABC transporters

[denovogenes]_112205	95.62	2e-176	bcel:BcellWH2_01760	K01803	TPI, tpiA	triosephosphate isomerase (TIM)	5.3.1.1	Metabolism--Carbohydrate metabolism--Glycolysis / Gluconeogenesis|Metabolism--Carbohydrate metabolism--Fructose and mannose metabolism|Metabolism--Carbohydrate metabolism--Inositol phosphate metabolism|Metabolism--Energy metabolism--Carbon fixation in photosynthetic organisms|Metabolism--Global and overview maps--Metabolic pathways|Metabolism--Global and overview maps--Biosynthesis of secondary metabolites|Metabolism--Global and overview maps--Microbial metabolism in diverse environments|Metabolism--Global and overview maps--Biosynthesis of antibiotics|Metabolism--Global and overview maps--Carbon metabolism|Metabolism--Global and overview maps--Biosynthesis of amino acids

[denovogenes]_112206	46.75	9e-75	oih:OB2925	K16698	tuaG	teichuronic acid biosynthesis glycosyltransferase TuaG	2.4.-.-	--

[denovogenes]_112207	53.48	1e-75	pft:JBW_00779	K06872	K06872	uncharacterized protein	--	--

[denovogenes]_112209	100.00	0.0	boa:Bovatus_02155	K02013	ABC.FEV.A	iron complex transport system ATP-binding protein	3.6.3.34	Environmental Information Processing--Membrane transport--ABC transporters

[denovogenes]_11221	90.18	0.0	pdi:BDI_1726	K00700	GBE1, glgB	1,4-alpha-glucan branching enzyme	2.4.1.18	Metabolism--Carbohydrate metabolism--Starch and sucrose metabolism|Metabolism--Global and overview maps--Metabolic pathways|Metabolism--Global and overview maps--Biosynthesis of secondary metabolites

[denovogenes]_112212	73.77	8e-124	bprs:CK3_22280	K15599	thiX	putative hydroxymethylpyrimidine transport system permease protein	--	Environmental Information Processing--Membrane transport--ABC transporters

[denovogenes]_112213	94.82	0.0	boa:Bovatus_00643	K01470	E3.5.2.10	creatinine amidohydrolase	3.5.2.10	Metabolism--Amino acid metabolism--Arginine and proline metabolism

[denovogenes]_112216	93.20	2e-171	bcel:BcellWH2_05052	K02500	hisF	cyclase	4.1.3.-	Metabolism--Amino acid metabolism--Histidine metabolism|Metabolism--Global and overview maps--Metabolic pathways|Metabolism--Global and overview maps--Biosynthesis of secondary metabolites|Metabolism--Global and overview maps--Biosynthesis of amino acids

[denovogenes]_112219	99.60	0.0	btho:Btheta7330_00944	K02013	ABC.FEV.A	iron complex transport system ATP-binding protein	3.6.3.34	Environmental Information Processing--Membrane transport--ABC transporters

[denovogenes]_112221	64.94	2e-96	fpla:A4U99_00175	K07402	xdhC	xanthine dehydrogenase accessory factor	--	--

[denovogenes]_112222	76.28	7e-130	fpla:A4U99_18050	K06346	jag	spoIIIJ-associated protein	--	--

[denovogenes]_112223	88.00	7e-167	boa:Bovatus_04509	K06173	truA, PUS1	tRNA pseudouridine38-40 synthase	5.4.99.12	--

[denovogenes]_112225	87.45	3e-164	byl:A4V09_21245	K02036	pstB	phosphate transport system ATP-binding protein	3.6.3.27	Environmental Information Processing--Membrane transport--ABC transporters

[denovogenes]_112227	40.70	1e-37	nma:NMA1465	K14058	ttcA	tRNA 2-thiocytidine biosynthesis protein TtcA	--	--

[denovogenes]_112228	90.36	4e-168	btho:Btheta7330_00976	K12343	SRD5A1	3-oxo-5-alpha-steroid 4-dehydrogenase 1	1.3.1.22	Metabolism--Lipid metabolism--Steroid hormone biosynthesis

[denovogenes]_112229	50.25	8e-61	csh:Closa_3467	K02411	fliH	flagellar assembly protein FliH	--	Cellular Processes--Cell motility--Flagellar assembly

[denovogenes]_11223	66.02	0.0	rix:RO1_12810	K12308	bgaB, lacA	beta-galactosidase	3.2.1.23	Metabolism--Carbohydrate metabolism--Galactose metabolism

[denovogenes]_112230	53.57	5e-88	csh:Closa_3458	K02557	motB	chemotaxis protein MotB	--	Cellular Processes--Cell motility--Bacterial chemotaxis|Cellular Processes--Cell motility--Flagellar assembly

[denovogenes]_112231	72.73	3e-119	fpla:A4U99_17540	K00059	fabG	3-oxoacyl-[acyl-carrier protein] reductase	1.1.1.100	Metabolism--Lipid metabolism--Fatty acid biosynthesis|Metabolism--Metabolism of cofactors and vitamins--Biotin metabolism|Metabolism--Lipid metabolism--Biosynthesis of unsaturated fatty acids|Metabolism--Global and overview maps--Metabolic pathways|Metabolism--Global and overview maps--Fatty acid metabolism

[denovogenes]_112232	49.81	1e-80	byl:A4V09_05300	K08600	srtB	sortase B	3.4.22.70	--

[denovogenes]_112233	77.42	4e-113	ibu:IB211_00816c	K02072	metI	D-methionine transport system permease protein	--	Environmental Information Processing--Membrane transport--ABC transporters

[denovogenes]_112234	99.20	0.0	bvu:BVU_3064	K03437	spoU	RNA methyltransferase, TrmH family	--	--

[denovogenes]_112235	70.45	1e-122	bprs:CK3_22270	K15600	thiZ	putative hydroxymethylpyrimidine transport system ATP-binding protein	--	Environmental Information Processing--Membrane transport--ABC transporters

[denovogenes]_112237	40.73	1e-38	byl:A4V09_19045	K03524	birA	BirA family transcriptional regulator, biotin operon repressor / biotin-[acetyl-CoA-carboxylase] ligase	6.3.4.15	Metabolism--Metabolism of cofactors and vitamins--Biotin metabolism|Metabolism--Global and overview maps--Metabolic pathways

[denovogenes]_112240	74.00	2e-136	rto:RTO_03960	K05936	cobM, cbiF	precorrin-4/cobalt-precorrin-4 C11-methyltransferase	2.1.1.133 2.1.1.271	Metabolism--Metabolism of cofactors and vitamins--Porphyrin and chlorophyll metabolism|Metabolism--Global and overview maps--Metabolic pathways

[denovogenes]_112241	74.70	1e-132	csh:Closa_3745	K01265	map	methionyl aminopeptidase	3.4.11.18	--

[denovogenes]_112246	55.24	3e-98	ccel:CCDG5_0500	K09769	K09769	uncharacterized protein	--	--

[denovogenes]_112247	81.71	2e-154	ruk:A4V00_02325	K00175	korB, oorB, oforB	2-oxoglutarate/2-oxoacid ferredoxin oxidoreductase subunit beta	1.2.7.3 1.2.7.11	Metabolism--Carbohydrate metabolism--Glycolysis / Gluconeogenesis|Metabolism--Carbohydrate metabolism--Citrate cycle (TCA cycle)|Metabolism--Carbohydrate metabolism--Pyruvate metabolism|Metabolism--Carbohydrate metabolism--Butanoate metabolism|Metabolism--Energy metabolism--Carbon fixation pathways in prokaryotes|Metabolism--Global and overview maps--Metabolic pathways|Metabolism--Global and overview maps--Microbial metabolism in diverse environments|Metabolism--Global and overview maps--Biosynthesis of antibiotics|Metabolism--Global and overview maps--Carbon metabolism

[denovogenes]_112249	75.21	1e-132	fpla:A4U99_12655	K12410	npdA	NAD-dependent deacetylase	3.5.1.-	--

[denovogenes]_11225	46.95	0.0	erb:A4V01_09015	K03655	recG	ATP-dependent DNA helicase RecG	3.6.4.12	Genetic Information Processing--Replication and repair--Homologous recombination

[denovogenes]_112250	42.17	5e-55	caby:Cabys_1689	K02081	agaR	DeoR family transcriptional regulator, aga operon transcriptional repressor	--	--

[denovogenes]_112251	40.37	1e-11	bts:Btus_3212	K06386	spoIIQ	stage II sporulation protein Q	--	--

[denovogenes]_112252	75.81	1e-141	byl:A4V09_15485	K01265	map	methionyl aminopeptidase	3.4.11.18	--

[denovogenes]_112253	74.40	4e-135	csh:Closa_3745	K01265	map	methionyl aminopeptidase	3.4.11.18	--

[denovogenes]_112254	44.22	4e-71	rto:RTO_02380	K12510	tadB	tight adherence protein B	--	--

[denovogenes]_112255	100.00	0.0	bvu:BVU_0466	K01803	TPI, tpiA	triosephosphate isomerase (TIM)	5.3.1.1	Metabolism--Carbohydrate metabolism--Glycolysis / Gluconeogenesis|Metabolism--Carbohydrate metabolism--Fructose and mannose metabolism|Metabolism--Carbohydrate metabolism--Inositol phosphate metabolism|Metabolism--Energy metabolism--Carbon fixation in photosynthetic organisms|Metabolism--Global and overview maps--Metabolic pathways|Metabolism--Global and overview maps--Biosynthesis of secondary metabolites|Metabolism--Global and overview maps--Microbial metabolism in diverse environments|Metabolism--Global and overview maps--Biosynthesis of antibiotics|Metabolism--Global and overview maps--Carbon metabolism|Metabolism--Global and overview maps--Biosynthesis of amino acids

[denovogenes]_112256	92.83	2e-178	lacy:A4V08_19120	K01142	E3.1.11.2, xthA	exodeoxyribonuclease III	3.1.11.2	Genetic Information Processing--Replication and repair--Base excision repair

[denovogenes]_112257	85.48	6e-154	rto:RTO_18110	K03218	rlmB	23S rRNA (guanosine2251-2'-O)-methyltransferase	2.1.1.185	--

[denovogenes]_112259	41.25	2e-36	ran:Riean_1954	K07741	antB	anti-repressor protein	--	--

[denovogenes]_11226	92.46	0.0	vpr:Vpar_0299	K00820	glmS, GFPT	glucosamine--fructose-6-phosphate aminotransferase (isomerizing)	2.6.1.16	Metabolism--Amino acid metabolism--Alanine, aspartate and glutamate metabolism|Metabolism--Carbohydrate metabolism--Amino sugar and nucleotide sugar metabolism|Metabolism--Global and overview maps--Metabolic pathways|Metabolism--Global and overview maps--Biosynthesis of antibiotics|Human Diseases--Endocrine and metabolic diseases--Insulin resistance

[denovogenes]_112260	64.76	4e-100	dgi:Desgi_1057	K01935	bioD	dethiobiotin synthetase	6.3.3.3	Metabolism--Metabolism of cofactors and vitamins--Biotin metabolism|Metabolism--Global and overview maps--Metabolic pathways

[denovogenes]_112265	45.89	9e-27	cvi:CV_0727	K01185	E3.2.1.17	lysozyme	3.2.1.17	--

[denovogenes]_112269	43.43	3e-72	rto:RTO_02380	K12510	tadB	tight adherence protein B	--	--

[denovogenes]_112271	80.08	1e-152	bxy:BXY_10700	K03524	birA	BirA family transcriptional regulator, biotin operon repressor / biotin-[acetyl-CoA-carboxylase] ligase	6.3.4.15	Metabolism--Metabolism of cofactors and vitamins--Biotin metabolism|Metabolism--Global and overview maps--Metabolic pathways

[denovogenes]_112272	99.20	0.0	bvu:BVU_0466	K01803	TPI, tpiA	triosephosphate isomerase (TIM)	5.3.1.1	Metabolism--Carbohydrate metabolism--Glycolysis / Gluconeogenesis|Metabolism--Carbohydrate metabolism--Fructose and mannose metabolism|Metabolism--Carbohydrate metabolism--Inositol phosphate metabolism|Metabolism--Energy metabolism--Carbon fixation in photosynthetic organisms|Metabolism--Global and overview maps--Metabolic pathways|Metabolism--Global and overview maps--Biosynthesis of secondary metabolites|Metabolism--Global and overview maps--Microbial metabolism in diverse environments|Metabolism--Global and overview maps--Biosynthesis of antibiotics|Metabolism--Global and overview maps--Carbon metabolism|Metabolism--Global and overview maps--Biosynthesis of amino acids

[denovogenes]_112273	87.25	2e-165	bdh:GV66_05270	K00215	dapB	4-hydroxy-tetrahydrodipicolinate reductase	1.17.1.8	Metabolism--Biosynthesis of other secondary metabolites--Monobactam biosynthesis|Metabolism--Amino acid metabolism--Lysine biosynthesis|Metabolism--Global and overview maps--Metabolic pathways|Metabolism--Global and overview maps--Biosynthesis of secondary metabolites|Metabolism--Global and overview maps--Microbial metabolism in diverse environments|Metabolism--Global and overview maps--Biosynthesis of antibiotics|Metabolism--Global and overview maps--Biosynthesis of amino acids

[denovogenes]_112274	52.13	1e-61	cbb:CLD_2060	K01358	clpP, CLPP	ATP-dependent Clp protease, protease subunit	3.4.21.92	Cellular Processes--Cell growth and death--Cell cycle - Caulobacter|Organismal Systems--Aging--Longevity regulating pathway - worm

[denovogenes]_112276	98.80	2e-177	fpla:A4U99_06655	K09817	znuC	zinc transport system ATP-binding protein	3.6.3.-	Environmental Information Processing--Membrane transport--ABC transporters

[denovogenes]_112278	71.60	2e-127	drs:DEHRE_14520	K05936	cobM, cbiF	precorrin-4/cobalt-precorrin-4 C11-methyltransferase	2.1.1.133 2.1.1.271	Metabolism--Metabolism of cofactors and vitamins--Porphyrin and chlorophyll metabolism|Metabolism--Global and overview maps--Metabolic pathways

[denovogenes]_112279	78.86	6e-149	lacy:A4V08_22265	K06173	truA, PUS1	tRNA pseudouridine38-40 synthase	5.4.99.12	--

[denovogenes]_11228	100.00	0.0	mmk:MU9_3455	K12137	hyfB	hydrogenase-4 component B	1.-.-.-	--

[denovogenes]_112280	99.20	0.0	bcel:BcellWH2_01908	K06861	lptB	lipopolysaccharide export system ATP-binding protein	3.6.3.-	Environmental Information Processing--Membrane transport--ABC transporters

[denovogenes]_112281	82.87	4e-149	ova:OBV_42040	K02500	hisF	cyclase	4.1.3.-	Metabolism--Amino acid metabolism--Histidine metabolism|Metabolism--Global and overview maps--Metabolic pathways|Metabolism--Global and overview maps--Biosynthesis of secondary metabolites|Metabolism--Global and overview maps--Biosynthesis of amino acids

[denovogenes]_112283	86.06	2e-168	bprm:CL3_14740	K01142	E3.1.11.2, xthA	exodeoxyribonuclease III	3.1.11.2	Genetic Information Processing--Replication and repair--Base excision repair

[denovogenes]_112286	44.96	1e-65	bbe:BBR47_31000	K15600	thiZ	putative hydroxymethylpyrimidine transport system ATP-binding protein	--	Environmental Information Processing--Membrane transport--ABC transporters

[denovogenes]_112287	58.60	6e-64	ruk:A4V00_06850	K03088	SIG3.2, rpoE	RNA polymerase sigma-70 factor, ECF subfamily	--	--

[denovogenes]_112288	61.85	2e-107	bvu:BVU_2653	K13002	wbyL	glycosyltransferase	2.4.1.-	--

[denovogenes]_112289	40.00	8e-53	smw:SMWW4_v1c06200	K12519	papD	chaperone protein PapD	--	--

[denovogenes]_11229	66.35	0.0	lacy:A4V08_01995	K06158	ABCF3	ATP-binding cassette, subfamily F, member 3	--	--

[denovogenes]_112290	100.00	0.0	bdh:GV66_05270	K00215	dapB	4-hydroxy-tetrahydrodipicolinate reductase	1.17.1.8	Metabolism--Biosynthesis of other secondary metabolites--Monobactam biosynthesis|Metabolism--Amino acid metabolism--Lysine biosynthesis|Metabolism--Global and overview maps--Metabolic pathways|Metabolism--Global and overview maps--Biosynthesis of secondary metabolites|Metabolism--Global and overview maps--Microbial metabolism in diverse environments|Metabolism--Global and overview maps--Biosynthesis of antibiotics|Metabolism--Global and overview maps--Biosynthesis of amino acids

[denovogenes]_112292	62.23	6e-102	amt:Amet_1663	K19119	cas5d	CRISPR-associated protein Cas5d	--	--

[denovogenes]_112294	100.00	0.0	sdy:SDY_3910	K03183	ubiE	demethylmenaquinone methyltransferase / 2-methoxy-6-polyprenyl-1,4-benzoquinol methylase	2.1.1.163 2.1.1.201	Metabolism--Metabolism of cofactors and vitamins--Ubiquinone and other terpenoid-quinone biosynthesis|Metabolism--Global and overview maps--Metabolic pathways|Metabolism--Global and overview maps--Biosynthesis of secondary metabolites

[denovogenes]_112295	100.00	0.0	bvu:BVU_3853	K02500	hisF	cyclase	4.1.3.-	Metabolism--Amino acid metabolism--Histidine metabolism|Metabolism--Global and overview maps--Metabolic pathways|Metabolism--Global and overview maps--Biosynthesis of secondary metabolites|Metabolism--Global and overview maps--Biosynthesis of amino acids

[denovogenes]_112297	44.44	1e-66	baco:OXB_2218	K15600	thiZ	putative hydroxymethylpyrimidine transport system ATP-binding protein	--	Environmental Information Processing--Membrane transport--ABC transporters

[denovogenes]_112298	40.25	2e-63	tro:trd_0598	K15599	thiX	putative hydroxymethylpyrimidine transport system permease protein	--	Environmental Information Processing--Membrane transport--ABC transporters

[denovogenes]_112299	80.08	1e-144	ibu:IB211_01556	K02500	hisF	cyclase	4.1.3.-	Metabolism--Amino acid metabolism--Histidine metabolism|Metabolism--Global and overview maps--Metabolic pathways|Metabolism--Global and overview maps--Biosynthesis of secondary metabolites|Metabolism--Global and overview maps--Biosynthesis of amino acids

[denovogenes]_112303	70.52	4e-129	lacy:A4V08_34255	K00215	dapB	4-hydroxy-tetrahydrodipicolinate reductase	1.17.1.8	Metabolism--Biosynthesis of other secondary metabolites--Monobactam biosynthesis|Metabolism--Amino acid metabolism--Lysine biosynthesis|Metabolism--Global and overview maps--Metabolic pathways|Metabolism--Global and overview maps--Biosynthesis of secondary metabolites|Metabolism--Global and overview maps--Microbial metabolism in diverse environments|Metabolism--Global and overview maps--Biosynthesis of antibiotics|Metabolism--Global and overview maps--Biosynthesis of amino acids

[denovogenes]_112304	70.68	7e-129	cct:CC1_11080	K02028	ABC.PA.A	polar amino acid transport system ATP-binding protein	3.6.3.21	--

[denovogenes]_112305	81.35	1e-148	cpy:Cphy_1237	K06147	ABCB-BAC	ATP-binding cassette, subfamily B, bacterial	--	--

[denovogenes]_112306	90.04	2e-166	ror:RORB6_07540	K02611	--	--	--	Metabolism--Amino acid metabolism--Phenylalanine metabolism|Metabolism--Global and overview maps--Microbial metabolism in diverse environments

[denovogenes]_112308	87.65	2e-160	lacy:A4V08_14770	K05815	ugpE	sn-glycerol 3-phosphate transport system permease protein	--	Environmental Information Processing--Membrane transport--ABC transporters

[denovogenes]_112310	75.71	2e-136	cso:CLS_11480	K02010	afuC, fbpC	iron(III) transport system ATP-binding protein	3.6.3.30	Environmental Information Processing--Membrane transport--ABC transporters

[denovogenes]_112311	100.00	0.0	bfg:BF638R_3662	K00571	E2.1.1.72	site-specific DNA-methyltransferase (adenine-specific)	2.1.1.72	--

[denovogenes]_112312	69.20	4e-122	elm:ELI_3913	K02007	cbiM	cobalt/nickel transport system permease protein	--	Environmental Information Processing--Membrane transport--ABC transporters

[denovogenes]_112314	71.20	3e-135	erb:A4V01_14305	K01142	E3.1.11.2, xthA	exodeoxyribonuclease III	3.1.11.2	Genetic Information Processing--Replication and repair--Base excision repair

[denovogenes]_112315	98.01	0.0	bvu:BVU_0761	K01470	E3.5.2.10	creatinine amidohydrolase	3.5.2.10	Metabolism--Amino acid metabolism--Arginine and proline metabolism

[denovogenes]_11232	57.82	0.0	bmyc:DJ92_411	K02004	ABC.CD.P	putative ABC transport system permease protein	--	--

[denovogenes]_112320	74.80	9e-139	bvu:BVU_2782	K02169	bioC	malonyl-CoA O-methyltransferase	2.1.1.197	Metabolism--Metabolism of cofactors and vitamins--Biotin metabolism|Metabolism--Global and overview maps--Metabolic pathways

[denovogenes]_112321	97.61	6e-155	fpla:A4U99_12135	K11068	hlyIII	hemolysin III	--	--

[denovogenes]_112322	50.40	5e-77	seu:SEQ_1274	K03497	parB, spo0J	chromosome partitioning protein, ParB family	--	--

[denovogenes]_112323	71.43	2e-128	lacy:A4V08_00620	K06201	cutC	copper homeostasis protein	--	--

[denovogenes]_112324	66.00	9e-129	faa:HMPREF0389_01218	K01142	E3.1.11.2, xthA	exodeoxyribonuclease III	3.1.11.2	Genetic Information Processing--Replication and repair--Base excision repair

[denovogenes]_112327	40.26	6e-19	acj:ACAM_1000	K21029	moeB	molybdopterin-synthase adenylyltransferase	2.7.7.80	Genetic Information Processing--Folding, sorting and degradation--Sulfur relay system

[denovogenes]_11233	99.40	0.0	sga:GALLO_1885	K03655	recG	ATP-dependent DNA helicase RecG	3.6.4.12	Genetic Information Processing--Replication and repair--Homologous recombination

[denovogenes]_112331	58.14	2e-88	pary:A4V02_08670	K10716	kch, trkA, mthK, pch	voltage-gated potassium channel	--	--

[denovogenes]_112332	64.37	5e-105	fpla:A4U99_13410	K06864	K06864	uncharacterized protein	--	--

[denovogenes]_112333	100.00	0.0	cif:AL515_01815	K03183	ubiE	demethylmenaquinone methyltransferase / 2-methoxy-6-polyprenyl-1,4-benzoquinol methylase	2.1.1.163 2.1.1.201	Metabolism--Metabolism of cofactors and vitamins--Ubiquinone and other terpenoid-quinone biosynthesis|Metabolism--Global and overview maps--Metabolic pathways|Metabolism--Global and overview maps--Biosynthesis of secondary metabolites

[denovogenes]_112335	42.48	3e-24	ana:all7002	K07492	K07492	putative transposase	--	--

[denovogenes]_112336	96.81	2e-148	fpla:A4U99_00175	K07402	xdhC	xanthine dehydrogenase accessory factor	--	--

[denovogenes]_112337	90.28	5e-163	bvu:BVU_0589	K00286	proC	pyrroline-5-carboxylate reductase	1.5.1.2	Metabolism--Amino acid metabolism--Arginine and proline metabolism|Metabolism--Global and overview maps--Metabolic pathways|Metabolism--Global and overview maps--Biosynthesis of secondary metabolites|Metabolism--Global and overview maps--Biosynthesis of antibiotics|Metabolism--Global and overview maps--Biosynthesis of amino acids

[denovogenes]_112339	100.00	0.0	pdi:BDI_3537	K09013	sufC	Fe-S cluster assembly ATP-binding protein	--	--

[denovogenes]_11234	100.00	0.0	pvl:AOB99_14270	K12137	hyfB	hydrogenase-4 component B	1.-.-.-	--

[denovogenes]_112341	80.79	2e-137	rto:RTO_18080	K00640	cysE	serine O-acetyltransferase	2.3.1.30	Metabolism--Amino acid metabolism--Cysteine and methionine metabolism|Metabolism--Energy metabolism--Sulfur metabolism|Metabolism--Global and overview maps--Metabolic pathways|Metabolism--Global and overview maps--Biosynthesis of secondary metabolites|Metabolism--Global and overview maps--Microbial metabolism in diverse environments|Metabolism--Global and overview maps--Carbon metabolism|Metabolism--Global and overview maps--Biosynthesis of amino acids|Cellular Processes--Cellular community - prokaryotes--Biofilm formation - Vibrio cholerae

[denovogenes]_112342	90.84	7e-164	rto:RTO_27340	K03977	engA, der	GTPase	--	--

[denovogenes]_112344	96.02	6e-177	bfb:VU15_17745	K01803	TPI, tpiA	triosephosphate isomerase (TIM)	5.3.1.1	Metabolism--Carbohydrate metabolism--Glycolysis / Gluconeogenesis|Metabolism--Carbohydrate metabolism--Fructose and mannose metabolism|Metabolism--Carbohydrate metabolism--Inositol phosphate metabolism|Metabolism--Energy metabolism--Carbon fixation in photosynthetic organisms|Metabolism--Global and overview maps--Metabolic pathways|Metabolism--Global and overview maps--Biosynthesis of secondary metabolites|Metabolism--Global and overview maps--Microbial metabolism in diverse environments|Metabolism--Global and overview maps--Biosynthesis of antibiotics|Metabolism--Global and overview maps--Carbon metabolism|Metabolism--Global and overview maps--Biosynthesis of amino acids

[denovogenes]_112346	99.60	0.0	cif:AL515_16150	K04754	mlaA, vacJ	phospholipid-binding lipoprotein MlaA	--	--

[denovogenes]_112349	95.26	7e-165	cso:CLS_16410	K02967	RP-S2, MRPS2, rpsB	small subunit ribosomal protein S2	--	Genetic Information Processing--Translation--Ribosome

[denovogenes]_11235	52.09	0.0	cbv:U729_2338	K18149	pbp5, pbpC, pbp3	penicillin-binding protein	--	Human Diseases--Drug resistance: Antimicrobial--beta-Lactam resistance

[denovogenes]_112350	59.04	1e-100	afn:Acfer_1636	K01989	ABC.X4.S	putative ABC transport system substrate-binding protein	--	--

[denovogenes]_112351	61.54	3e-102	ruk:A4V00_08005	K09861	K09861	uncharacterized protein	--	--

[denovogenes]_112352	57.55	6e-95	bprs:CK3_28460	K02233	E2.7.8.26, cobS, cobV	adenosylcobinamide-GDP ribazoletransferase	2.7.8.26	Metabolism--Metabolism of cofactors and vitamins--Porphyrin and chlorophyll metabolism|Metabolism--Global and overview maps--Metabolic pathways

[denovogenes]_112353	91.27	3e-154	bvu:BVU_0260	K01993	ABC-2.TX	HlyD family secretion protein	--	--

[denovogenes]_112355	99.60	0.0	fpla:A4U99_09045	K05896	scpA	segregation and condensation protein A	--	--

[denovogenes]_112358	91.24	3e-172	bvu:BVU_3853	K02500	hisF	cyclase	4.1.3.-	Metabolism--Amino acid metabolism--Histidine metabolism|Metabolism--Global and overview maps--Metabolic pathways|Metabolism--Global and overview maps--Biosynthesis of secondary metabolites|Metabolism--Global and overview maps--Biosynthesis of amino acids

[denovogenes]_112359	70.52	4e-132	cbut:ATN24_09390	K02013	ABC.FEV.A	iron complex transport system ATP-binding protein	3.6.3.34	Environmental Information Processing--Membrane transport--ABC transporters

[denovogenes]_112361	100.00	0.0	shq:A0259_02595	K03477	ulaR	DeoR family transcriptional regulator, ulaG and ulaABCDEF operon transcriptional repressor	--	--

[denovogenes]_112362	100.00	0.0	pdi:BDI_3208	K00240	sdhB, frdB	succinate dehydrogenase / fumarate reductase, iron-sulfur subunit	1.3.5.1 1.3.5.4	Metabolism--Carbohydrate metabolism--Citrate cycle (TCA cycle)|Metabolism--Energy metabolism--Oxidative phosphorylation|Metabolism--Carbohydrate metabolism--Butanoate metabolism|Metabolism--Energy metabolism--Carbon fixation pathways in prokaryotes|Metabolism--Global and overview maps--Metabolic pathways|Metabolism--Global and overview maps--Biosynthesis of secondary metabolites|Metabolism--Global and overview maps--Microbial metabolism in diverse environments|Metabolism--Global and overview maps--Biosynthesis of antibiotics|Metabolism--Global and overview maps--Carbon metabolism

[denovogenes]_112366	46.37	4e-65	pms:KNP414_01241	K19169	dndB	DNA sulfur modification protein DndB	--	--

[denovogenes]_112367	61.07	1e-106	bprs:CK3_29030	K02033	ABC.PE.P	peptide/nickel transport system permease protein	--	Cellular Processes--Cellular community - prokaryotes--Quorum sensing

[denovogenes]_112369	81.82	3e-133	bcel:BcellWH2_01607	K03453	TC.BASS	bile acid:Na+ symporter, BASS family	--	--

[denovogenes]_112370	54.07	2e-89	rto:RTO_26470	K06298	gerM	germination protein M	--	--

[denovogenes]_112373	43.10	2e-46	yak:ACZ76_14455	K07497	K07497	putative transposase	--	--

[denovogenes]_112376	69.20	2e-125	bprm:CL3_11490	K01868	TARS, thrS	threonyl-tRNA synthetase	6.1.1.3	Genetic Information Processing--Translation--Aminoacyl-tRNA biosynthesis

[denovogenes]_112378	99.60	9e-180	cfd:CFNIH1_15385	K02391	flgF	flagellar basal-body rod protein FlgF	--	Cellular Processes--Cell motility--Flagellar assembly

[denovogenes]_112379	62.30	6e-108	fpla:A4U99_08130	K00003	E1.1.1.3	homoserine dehydrogenase	1.1.1.3	Metabolism--Amino acid metabolism--Glycine, serine and threonine metabolism|Metabolism--Amino acid metabolism--Cysteine and methionine metabolism|Metabolism--Amino acid metabolism--Lysine biosynthesis|Metabolism--Global and overview maps--Metabolic pathways|Metabolism--Global and overview maps--Biosynthesis of secondary metabolites|Metabolism--Global and overview maps--Microbial metabolism in diverse environments|Metabolism--Global and overview maps--Biosynthesis of antibiotics|Metabolism--Global and overview maps--Biosynthesis of amino acids

[denovogenes]_11238	70.90	0.0	fpla:A4U99_01380	K07462	recJ	single-stranded-DNA-specific exonuclease	3.1.-.-	Genetic Information Processing--Replication and repair--Base excision repair|Genetic Information Processing--Replication and repair--Mismatch repair|Genetic Information Processing--Replication and repair--Homologous recombination

[denovogenes]_112382	95.22	1e-171	euc:EC1_10400	K03205	virD4, lvhD4	type IV secretion system protein VirD4	--	Environmental Information Processing--Membrane transport--Bacterial secretion system

[denovogenes]_112384	100.00	0.0	sbo:SBO_1175	K09817	znuC	zinc transport system ATP-binding protein	3.6.3.-	Environmental Information Processing--Membrane transport--ABC transporters

[denovogenes]_112385	66.93	5e-118	bcel:BcellWH2_02656	K03734	apbE	FAD:protein FMN transferase	2.7.1.180	--

[denovogenes]_112386	73.02	7e-126	cle:Clole_1894	K01752	E4.3.1.17, sdaA, sdaB, tdcG	L-serine dehydratase	4.3.1.17	Metabolism--Amino acid metabolism--Glycine, serine and threonine metabolism|Metabolism--Amino acid metabolism--Cysteine and methionine metabolism|Metabolism--Global and overview maps--Metabolic pathways|Metabolism--Global and overview maps--Biosynthesis of secondary metabolites|Metabolism--Global and overview maps--Biosynthesis of antibiotics|Metabolism--Global and overview maps--Carbon metabolism|Metabolism--Global and overview maps--Biosynthesis of amino acids

[denovogenes]_112388	47.41	8e-67	cpy:Cphy_3004	K01163	K01163	uncharacterized protein	--	--

[denovogenes]_112389	58.80	1e-101	cno:NT01CX_0917	K02315	dnaC	DNA replication protein DnaC	--	--

[denovogenes]_11239	59.58	0.0	cpro:CPRO_03770	K04759	feoB	ferrous iron transport protein B	--	--

[denovogenes]_112390	70.52	8e-122	bprs:CK3_33560	K18707	mtaB	threonylcarbamoyladenosine tRNA methylthiotransferase MtaB	2.8.4.5	--

[denovogenes]_112391	100.00	4e-179	cif:AL515_16270	K11535	nupC	nucleoside transport protein	--	--

[denovogenes]_112392	48.46	9e-67	cad:Curi_c17910	K15600	thiZ	putative hydroxymethylpyrimidine transport system ATP-binding protein	--	Environmental Information Processing--Membrane transport--ABC transporters

[denovogenes]_112393	59.44	1e-108	rch:RUM_07200	K01939	purA, ADSS	adenylosuccinate synthase	6.3.4.4	Metabolism--Nucleotide metabolism--Purine metabolism|Metabolism--Amino acid metabolism--Alanine, aspartate and glutamate metabolism|Metabolism--Global and overview maps--Metabolic pathways

[denovogenes]_112394	79.12	1e-145	bprs:CK3_25230	K00931	proB	glutamate 5-kinase	2.7.2.11	Metabolism--Amino acid metabolism--Arginine and proline metabolism|Metabolism--Biosynthesis of other secondary metabolites--Carbapenem biosynthesis|Metabolism--Global and overview maps--Metabolic pathways|Metabolism--Global and overview maps--Biosynthesis of antibiotics|Metabolism--Global and overview maps--Biosynthesis of amino acids

[denovogenes]_112396	50.99	1e-76	rum:CK1_00530	K04041	fbp3	fructose-1,6-bisphosphatase III	3.1.3.11	Metabolism--Carbohydrate metabolism--Glycolysis / Gluconeogenesis|Metabolism--Carbohydrate metabolism--Pentose phosphate pathway|Metabolism--Carbohydrate metabolism--Fructose and mannose metabolism|Metabolism--Energy metabolism--Methane metabolism|Metabolism--Energy metabolism--Carbon fixation in photosynthetic organisms|Metabolism--Global and overview maps--Metabolic pathways|Metabolism--Global and overview maps--Biosynthesis of secondary metabolites|Metabolism--Global and overview maps--Microbial metabolism in diverse environments|Metabolism--Global and overview maps--Biosynthesis of antibiotics|Metabolism--Global and overview maps--Carbon metabolism

[denovogenes]_112397	40.00	4e-24	cbz:Cbs_4279	K17865	croR	3-hydroxybutyryl-CoA dehydratase	4.2.1.55	Metabolism--Carbohydrate metabolism--Glyoxylate and dicarboxylate metabolism|Metabolism--Carbohydrate metabolism--Butanoate metabolism|Metabolism--Global and overview maps--Microbial metabolism in diverse environments|Metabolism--Global and overview maps--Carbon metabolism

[denovogenes]_112398	79.92	7e-146	rho:RHOM_11240	K00566	mnmA, trmU	tRNA-uridine 2-sulfurtransferase	2.8.1.13	Genetic Information Processing--Folding, sorting and degradation--Sulfur relay system

[denovogenes]_1124	67.82	0.0	rto:RTO_17740	K03529	smc	chromosome segregation protein	--	--

[denovogenes]_112401	73.02	3e-137	csh:Closa_1083	K03703	uvrC	excinuclease ABC subunit C	--	Genetic Information Processing--Replication and repair--Nucleotide excision repair

[denovogenes]_112405	77.78	2e-143	cle:Clole_1578	K00336	nuoG	NADH-quinone oxidoreductase subunit G	1.6.5.3	Metabolism--Energy metabolism--Oxidative phosphorylation|Metabolism--Global and overview maps--Metabolic pathways

[denovogenes]_112407	96.83	5e-178	cif:AL515_16610	K06203	cysZ	CysZ protein	--	--

[denovogenes]_112410	89.52	1e-164	erb:A4V01_12445	K02837	prfC	peptide chain release factor 3	--	--

[denovogenes]_112417	77.91	1e-146	rob:CK5_00560	K01758	CTH	cystathionine gamma-lyase	4.4.1.1	Metabolism--Amino acid metabolism--Glycine, serine and threonine metabolism|Metabolism--Amino acid metabolism--Cysteine and methionine metabolism|Metabolism--Metabolism of other amino acids--Selenocompound metabolism|Metabolism--Global and overview maps--Metabolic pathways|Metabolism--Global and overview maps--Biosynthesis of antibiotics|Metabolism--Global and overview maps--Biosynthesis of amino acids

[denovogenes]_11242	55.48	3e-170	cso:CLS_23930	K02224	cobB-cbiA	cobyrinic acid a,c-diamide synthase	6.3.5.9 6.3.5.11	Metabolism--Metabolism of cofactors and vitamins--Porphyrin and chlorophyll metabolism|Metabolism--Global and overview maps--Metabolic pathways

[denovogenes]_112422	80.88	1e-140	pfr:PFREUD_20230	K03785	aroD	3-dehydroquinate dehydratase I	4.2.1.10	Metabolism--Amino acid metabolism--Phenylalanine, tyrosine and tryptophan biosynthesis|Metabolism--Global and overview maps--Metabolic pathways|Metabolism--Global and overview maps--Biosynthesis of secondary metabolites|Metabolism--Global and overview maps--Biosynthesis of antibiotics|Metabolism--Global and overview maps--Biosynthesis of amino acids

[denovogenes]_112424	92.03	2e-162	pfr:PFREUD_06140	K16927	cbrT	energy-coupling factor transport system substrate-specific component	--	--

[denovogenes]_112425	93.23	8e-170	pfre:RM25_0640	K02013	ABC.FEV.A	iron complex transport system ATP-binding protein	3.6.3.34	Environmental Information Processing--Membrane transport--ABC transporters

[denovogenes]_112426	41.24	8e-37	pct:PC1_1316	K13002	wbyL	glycosyltransferase	2.4.1.-	--

[denovogenes]_112428	40.42	2e-52	scn:Solca_0478	K03574	mutT, NUDT15, MTH2	8-oxo-dGTP diphosphatase	3.6.1.55	--

[denovogenes]_112429	95.22	6e-158	pfr:PFREUD_02480	K03521	fixA, etfB	electron transfer flavoprotein beta subunit	--	--

[denovogenes]_11243	64.83	0.0	cso:CLS_10170	K02123	ATPVI, ntpI, atpI	V/A-type H+/Na+-transporting ATPase subunit I	--	Metabolism--Energy metabolism--Oxidative phosphorylation|Metabolism--Global and overview maps--Metabolic pathways

[denovogenes]_112431	59.69	2e-102	bfg:BF638R_3765	K07257	spsF	spore coat polysaccharide biosynthesis protein SpsF	--	--

[denovogenes]_112434	43.20	2e-58	mym:A176_002122	K00059	fabG	3-oxoacyl-[acyl-carrier protein] reductase	1.1.1.100	Metabolism--Lipid metabolism--Fatty acid biosynthesis|Metabolism--Metabolism of cofactors and vitamins--Biotin metabolism|Metabolism--Lipid metabolism--Biosynthesis of unsaturated fatty acids|Metabolism--Global and overview maps--Metabolic pathways|Metabolism--Global and overview maps--Fatty acid metabolism

[denovogenes]_112435	75.70	1e-140	pdi:BDI_1147	K06167	phnP	phosphoribosyl 1,2-cyclic phosphate phosphodiesterase	3.1.4.55	Metabolism--Metabolism of other amino acids--Phosphonate and phosphinate metabolism

[denovogenes]_112438	94.02	1e-172	pdi:BDI_3208	K00240	sdhB, frdB	succinate dehydrogenase / fumarate reductase, iron-sulfur subunit	1.3.5.1 1.3.5.4	Metabolism--Carbohydrate metabolism--Citrate cycle (TCA cycle)|Metabolism--Energy metabolism--Oxidative phosphorylation|Metabolism--Carbohydrate metabolism--Butanoate metabolism|Metabolism--Energy metabolism--Carbon fixation pathways in prokaryotes|Metabolism--Global and overview maps--Metabolic pathways|Metabolism--Global and overview maps--Biosynthesis of secondary metabolites|Metabolism--Global and overview maps--Microbial metabolism in diverse environments|Metabolism--Global and overview maps--Biosynthesis of antibiotics|Metabolism--Global and overview maps--Carbon metabolism

[denovogenes]_112439	88.45	5e-163	pdi:BDI_2018	K02500	hisF	cyclase	4.1.3.-	Metabolism--Amino acid metabolism--Histidine metabolism|Metabolism--Global and overview maps--Metabolic pathways|Metabolism--Global and overview maps--Biosynthesis of secondary metabolites|Metabolism--Global and overview maps--Biosynthesis of amino acids

[denovogenes]_11244	70.09	8e-154	bfr:BF1158	K07316	mod	adenine-specific DNA-methyltransferase	2.1.1.72	--

[denovogenes]_112441	44.94	7e-76	wfu:AXE80_13480	K02477	K02477	two-component system, LytT family, response regulator	--	--

[denovogenes]_112442	42.98	5e-63	ert:EUR_08670	K02564	nagB, GNPDA	glucosamine-6-phosphate deaminase	3.5.99.6	Metabolism--Carbohydrate metabolism--Amino sugar and nucleotide sugar metabolism|Metabolism--Global and overview maps--Metabolic pathways

[denovogenes]_112444	98.01	0.0	fpla:A4U99_04060	K05936	cobM, cbiF	precorrin-4/cobalt-precorrin-4 C11-methyltransferase	2.1.1.133 2.1.1.271	Metabolism--Metabolism of cofactors and vitamins--Porphyrin and chlorophyll metabolism|Metabolism--Global and overview maps--Metabolic pathways

[denovogenes]_112445	85.43	3e-161	pdi:BDI_0522	K00979	kdsB	3-deoxy-manno-octulosonate cytidylyltransferase (CMP-KDO synthetase)	2.7.7.38	Metabolism--Glycan biosynthesis and metabolism--Lipopolysaccharide biosynthesis|Metabolism--Global and overview maps--Metabolic pathways

[denovogenes]_112446	83.47	5e-158	pdi:BDI_1936	K06173	truA, PUS1	tRNA pseudouridine38-40 synthase	5.4.99.12	--

[denovogenes]_112447	40.00	2e-72	hsd:SD1D_1303	K02013	ABC.FEV.A	iron complex transport system ATP-binding protein	3.6.3.34	Environmental Information Processing--Membrane transport--ABC transporters

[denovogenes]_112448	75.30	2e-139	fpa:FPR_29200	K05936	cobM, cbiF	precorrin-4/cobalt-precorrin-4 C11-methyltransferase	2.1.1.133 2.1.1.271	Metabolism--Metabolism of cofactors and vitamins--Porphyrin and chlorophyll metabolism|Metabolism--Global and overview maps--Metabolic pathways

[denovogenes]_112449	99.60	7e-178	paeo:M801_4778	K01803	TPI, tpiA	triosephosphate isomerase (TIM)	5.3.1.1	Metabolism--Carbohydrate metabolism--Glycolysis / Gluconeogenesis|Metabolism--Carbohydrate metabolism--Fructose and mannose metabolism|Metabolism--Carbohydrate metabolism--Inositol phosphate metabolism|Metabolism--Energy metabolism--Carbon fixation in photosynthetic organisms|Metabolism--Global and overview maps--Metabolic pathways|Metabolism--Global and overview maps--Biosynthesis of secondary metabolites|Metabolism--Global and overview maps--Microbial metabolism in diverse environments|Metabolism--Global and overview maps--Biosynthesis of antibiotics|Metabolism--Global and overview maps--Carbon metabolism|Metabolism--Global and overview maps--Biosynthesis of amino acids

[denovogenes]_11245	48.52	0.0	cso:CLS_10010	K01163	K01163	uncharacterized protein	--	--

[denovogenes]_112450	60.80	8e-105	dvg:Deval_0331	K05810	yfiH	laccase domain-containing protein	--	--

[denovogenes]_112451	100.00	0.0	cfd:CFNIH1_23240	K04754	mlaA, vacJ	phospholipid-binding lipoprotein MlaA	--	--

[denovogenes]_112452	88.84	1e-166	dfi:AXF13_01525	K02049	ABC.SN.A	NitT/TauT family transport system ATP-binding protein	--	--

[denovogenes]_112455	40.18	1e-14	bao:BAMF_0545	K07741	antB	anti-repressor protein	--	--

[denovogenes]_112457	99.60	0.0	btho:Btheta7330_01843	K02500	hisF	cyclase	4.1.3.-	Metabolism--Amino acid metabolism--Histidine metabolism|Metabolism--Global and overview maps--Metabolic pathways|Metabolism--Global and overview maps--Biosynthesis of secondary metabolites|Metabolism--Global and overview maps--Biosynthesis of amino acids

[denovogenes]_112458	100.00	0.0	paeo:M801_3641	K00806	uppS	undecaprenyl diphosphate synthase	2.5.1.31	Metabolism--Metabolism of terpenoids and polyketides--Terpenoid backbone biosynthesis|Metabolism--Global and overview maps--Biosynthesis of secondary metabolites

[denovogenes]_112463	99.20	2e-176	paec:M802_5437	K01719	hemD, UROS	uroporphyrinogen-III synthase	4.2.1.75	Metabolism--Metabolism of cofactors and vitamins--Porphyrin and chlorophyll metabolism|Metabolism--Global and overview maps--Metabolic pathways|Metabolism--Global and overview maps--Biosynthesis of secondary metabolites

[denovogenes]_112464	97.61	9e-179	bfb:VU15_17745	K01803	TPI, tpiA	triosephosphate isomerase (TIM)	5.3.1.1	Metabolism--Carbohydrate metabolism--Glycolysis / Gluconeogenesis|Metabolism--Carbohydrate metabolism--Fructose and mannose metabolism|Metabolism--Carbohydrate metabolism--Inositol phosphate metabolism|Metabolism--Energy metabolism--Carbon fixation in photosynthetic organisms|Metabolism--Global and overview maps--Metabolic pathways|Metabolism--Global and overview maps--Biosynthesis of secondary metabolites|Metabolism--Global and overview maps--Microbial metabolism in diverse environments|Metabolism--Global and overview maps--Biosynthesis of antibiotics|Metabolism--Global and overview maps--Carbon metabolism|Metabolism--Global and overview maps--Biosynthesis of amino acids

[denovogenes]_112466	69.48	3e-129	eha:Ethha_2735	K01142	E3.1.11.2, xthA	exodeoxyribonuclease III	3.1.11.2	Genetic Information Processing--Replication and repair--Base excision repair

[denovogenes]_112468	96.02	2e-178	bfs:BF9343_0100	K02477	K02477	two-component system, LytT family, response regulator	--	--

[denovogenes]_112470	48.89	7e-69	eha:Ethha_1340	K04477	ycdX	putative hydrolase	--	--

[denovogenes]_112472	44.80	8e-68	fpa:FPR_20830	K01104	E3.1.3.48	protein-tyrosine phosphatase	3.1.3.48	--

[denovogenes]_112475	98.41	0.0	cfd:CFNIH1_00930	K01146	xni	protein Xni	--	--

[denovogenes]_112477	57.55	7e-98	dvm:DvMF_0940	K04486	E3.1.3.15B	histidinol-phosphatase (PHP family)	3.1.3.15	Metabolism--Amino acid metabolism--Histidine metabolism|Metabolism--Global and overview maps--Metabolic pathways|Metabolism--Global and overview maps--Biosynthesis of secondary metabolites|Metabolism--Global and overview maps--Biosynthesis of amino acids

[denovogenes]_112479	97.21	9e-179	cfd:CFNIH1_13080	K00216	entA	2,3-dihydro-2,3-dihydroxybenzoate dehydrogenase	1.3.1.28	Metabolism--Metabolism of terpenoids and polyketides--Biosynthesis of siderophore group nonribosomal peptides|Metabolism--Global and overview maps--Biosynthesis of secondary metabolites|Metabolism--Global and overview maps--Biosynthesis of antibiotics

[denovogenes]_11248	99.85	0.0	bth:BT_0683	K01187	malZ	alpha-glucosidase	3.2.1.20	Metabolism--Carbohydrate metabolism--Galactose metabolism|Metabolism--Carbohydrate metabolism--Starch and sucrose metabolism|Metabolism--Global and overview maps--Metabolic pathways

[denovogenes]_112480	73.31	2e-135	dfi:AXF13_01525	K02049	ABC.SN.A	NitT/TauT family transport system ATP-binding protein	--	--

[denovogenes]_112483	44.35	3e-69	ped:ING2D1G_1479	K03093	SIGMA70	RNA polymerase sigma factor	--	--

[denovogenes]_112489	53.93	5e-84	dvm:DvMF_2477	K02017	modC	molybdate transport system ATP-binding protein	3.6.3.29	Environmental Information Processing--Membrane transport--ABC transporters

[denovogenes]_11249	79.10	0.0	lacy:A4V08_18825	K01534	zntA	Cd2+/Zn2+-exporting ATPase	3.6.3.3 3.6.3.5	--

[denovogenes]_112492	42.57	2e-64	bfr:BF3365	K02477	K02477	two-component system, LytT family, response regulator	--	--

[denovogenes]_112493	41.50	3e-57	cpi:Cpin_6102	K02477	K02477	two-component system, LytT family, response regulator	--	--

[denovogenes]_112494	73.31	6e-136	lba:Lebu_1224	K02500	hisF	cyclase	4.1.3.-	Metabolism--Amino acid metabolism--Histidine metabolism|Metabolism--Global and overview maps--Metabolic pathways|Metabolism--Global and overview maps--Biosynthesis of secondary metabolites|Metabolism--Global and overview maps--Biosynthesis of amino acids

[denovogenes]_112496	100.00	2e-174	paeo:M801_4227	K12242	--	--	--	--

[denovogenes]_112498	68.53	5e-128	bfr:BF4382	K04069	pflA, pflC, pflE	pyruvate formate lyase activating enzyme	1.97.1.4	--

[denovogenes]_11250	94.49	0.0	rum:CK1_31700	K01972	E6.5.1.2, ligA, ligB	DNA ligase (NAD+)	6.5.1.2	Genetic Information Processing--Replication and repair--DNA replication|Genetic Information Processing--Replication and repair--Base excision repair|Genetic Information Processing--Replication and repair--Nucleotide excision repair|Genetic Information Processing--Replication and repair--Mismatch repair

[denovogenes]_112500	100.00	3e-180	paeo:M801_5401	K11474	glcC	GntR family transcriptional regulator, glc operon transcriptional activator	--	--

[denovogenes]_112502	100.00	0.0	paf:PAM18_1402	K02444	glpR	DeoR family transcriptional regulator, glycerol-3-phosphate regulon repressor	--	--

[denovogenes]_112504	99.20	0.0	cfd:CFNIH1_10740	K01069	E3.1.2.6, gloB	hydroxyacylglutathione hydrolase	3.1.2.6	Metabolism--Carbohydrate metabolism--Pyruvate metabolism

[denovogenes]_112508	66.93	4e-128	fpa:FPR_25660	K06889	K06889	uncharacterized protein	--	--

[denovogenes]_112509	58.68	6e-99	cpas:Clopa_1148	K15555	ssuB	sulfonate transport system ATP-binding protein	3.6.3.-	Metabolism--Energy metabolism--Sulfur metabolism|Environmental Information Processing--Membrane transport--ABC transporters

[denovogenes]_112510	98.41	0.0	cfd:CFNIH1_14010	K02169	bioC	malonyl-CoA O-methyltransferase	2.1.1.197	Metabolism--Metabolism of cofactors and vitamins--Biotin metabolism|Metabolism--Global and overview maps--Metabolic pathways

[denovogenes]_112511	96.81	1e-178	pdi:BDI_3537	K09013	sufC	Fe-S cluster assembly ATP-binding protein	--	--

[denovogenes]_112512	91.63	2e-169	pdi:BDI_2018	K02500	hisF	cyclase	4.1.3.-	Metabolism--Amino acid metabolism--Histidine metabolism|Metabolism--Global and overview maps--Metabolic pathways|Metabolism--Global and overview maps--Biosynthesis of secondary metabolites|Metabolism--Global and overview maps--Biosynthesis of amino acids

[denovogenes]_112513	98.80	0.0	pdi:BDI_0522	K00979	kdsB	3-deoxy-manno-octulosonate cytidylyltransferase (CMP-KDO synthetase)	2.7.7.38	Metabolism--Glycan biosynthesis and metabolism--Lipopolysaccharide biosynthesis|Metabolism--Global and overview maps--Metabolic pathways

[denovogenes]_112514	100.00	0.0	pdi:BDI_3208	K00240	sdhB, frdB	succinate dehydrogenase / fumarate reductase, iron-sulfur subunit	1.3.5.1 1.3.5.4	Metabolism--Carbohydrate metabolism--Citrate cycle (TCA cycle)|Metabolism--Energy metabolism--Oxidative phosphorylation|Metabolism--Carbohydrate metabolism--Butanoate metabolism|Metabolism--Energy metabolism--Carbon fixation pathways in prokaryotes|Metabolism--Global and overview maps--Metabolic pathways|Metabolism--Global and overview maps--Biosynthesis of secondary metabolites|Metabolism--Global and overview maps--Microbial metabolism in diverse environments|Metabolism--Global and overview maps--Biosynthesis of antibiotics|Metabolism--Global and overview maps--Carbon metabolism

[denovogenes]_112516	100.00	4e-175	paec:M802_4234	K09929	K09929	uncharacterized protein	--	--

[denovogenes]_112517	81.60	4e-140	ppn:Palpr_2778	K00853	araB	L-ribulokinase	2.7.1.16	Metabolism--Carbohydrate metabolism--Pentose and glucuronate interconversions|Metabolism--Global and overview maps--Metabolic pathways

[denovogenes]_112519	64.40	6e-116	sda:GGS_0822	K02530	lacR	DeoR family transcriptional regulator, lactose phosphotransferase system repressor	--	--

[denovogenes]_112521	54.58	1e-93	jeu:BJP62_17605	K16066	ydfG	3-hydroxy acid dehydrogenase / malonic semialdehyde reductase	1.1.1.381 1.1.1.-	Metabolism--Nucleotide metabolism--Pyrimidine metabolism|Metabolism--Amino acid metabolism--Glycine, serine and threonine metabolism|Metabolism--Global and overview maps--Metabolic pathways

[denovogenes]_112522	100.00	0.0	paes:SCV20265_3945	K02195	ccmC	heme exporter protein C	--	Environmental Information Processing--Membrane transport--ABC transporters

[denovogenes]_112523	95.22	9e-175	pdi:BDI_3208	K00240	sdhB, frdB	succinate dehydrogenase / fumarate reductase, iron-sulfur subunit	1.3.5.1 1.3.5.4	Metabolism--Carbohydrate metabolism--Citrate cycle (TCA cycle)|Metabolism--Energy metabolism--Oxidative phosphorylation|Metabolism--Carbohydrate metabolism--Butanoate metabolism|Metabolism--Energy metabolism--Carbon fixation pathways in prokaryotes|Metabolism--Global and overview maps--Metabolic pathways|Metabolism--Global and overview maps--Biosynthesis of secondary metabolites|Metabolism--Global and overview maps--Microbial metabolism in diverse environments|Metabolism--Global and overview maps--Biosynthesis of antibiotics|Metabolism--Global and overview maps--Carbon metabolism

[denovogenes]_112524	97.21	0.0	lacy:A4V08_19120	K01142	E3.1.11.2, xthA	exodeoxyribonuclease III	3.1.11.2	Genetic Information Processing--Replication and repair--Base excision repair

[denovogenes]_11253	100.00	0.0	ecol:LY180_12410	K01972	E6.5.1.2, ligA, ligB	DNA ligase (NAD+)	6.5.1.2	Genetic Information Processing--Replication and repair--DNA replication|Genetic Information Processing--Replication and repair--Base excision repair|Genetic Information Processing--Replication and repair--Nucleotide excision repair|Genetic Information Processing--Replication and repair--Mismatch repair

[denovogenes]_112531	44.94	7e-75	wfu:AXE80_13480	K02477	K02477	two-component system, LytT family, response regulator	--	--

[denovogenes]_112532	100.00	0.0	pdi:BDI_1147	K06167	phnP	phosphoribosyl 1,2-cyclic phosphate phosphodiesterase	3.1.4.55	Metabolism--Metabolism of other amino acids--Phosphonate and phosphinate metabolism

[denovogenes]_112533	88.21	3e-161	csh:Closa_3089	K02028	ABC.PA.A	polar amino acid transport system ATP-binding protein	3.6.3.21	--

[denovogenes]_112534	41.07	1e-27	vau:VANGNB10_cII0928	K18009	budC	meso-butanediol dehydrogenase / (S,S)-butanediol dehydrogenase / diacetyl reductase	1.1.1.- 1.1.1.76 1.1.1.304	Metabolism--Carbohydrate metabolism--Butanoate metabolism

[denovogenes]_112535	100.00	0.0	cfd:CFNIH1_15385	K02391	flgF	flagellar basal-body rod protein FlgF	--	Cellular Processes--Cell motility--Flagellar assembly

[denovogenes]_112536	100.00	0.0	cfd:CFNIH1_08585	K03477	ulaR	DeoR family transcriptional regulator, ulaG and ulaABCDEF operon transcriptional repressor	--	--

[denovogenes]_112537	100.00	0.0	paec:M802_1919	K02020	modA	molybdate transport system substrate-binding protein	--	Environmental Information Processing--Membrane transport--ABC transporters

[denovogenes]_112538	73.81	5e-140	csq:CSCA_2754	K00372	nasA	assimilatory nitrate reductase catalytic subunit	1.7.99.4	Metabolism--Energy metabolism--Nitrogen metabolism|Metabolism--Global and overview maps--Microbial metabolism in diverse environments

[denovogenes]_112539	100.00	2e-180	paeo:M801_4522	K07160	K07160	UPF0271 protein	--	--

[denovogenes]_11254	70.82	0.0	ert:EUR_10730	K18149	pbp5, pbpC, pbp3	penicillin-binding protein	--	Human Diseases--Drug resistance: Antimicrobial--beta-Lactam resistance

[denovogenes]_112541	98.80	0.0	pdi:BDI_2018	K02500	hisF	cyclase	4.1.3.-	Metabolism--Amino acid metabolism--Histidine metabolism|Metabolism--Global and overview maps--Metabolic pathways|Metabolism--Global and overview maps--Biosynthesis of secondary metabolites|Metabolism--Global and overview maps--Biosynthesis of amino acids

[denovogenes]_112542	40.74	4e-61	cck:Ccar_14310	K20373	rgg2	HTH-type transcriptional regulator, SHP2-responsive activator	--	Cellular Processes--Cellular community - prokaryotes--Quorum sensing

[denovogenes]_112543	100.00	5e-179	paeo:M801_3230	K02053	ABC.SP.P	putative spermidine/putrescine transport system permease protein	--	Cellular Processes--Cellular community - prokaryotes--Quorum sensing

[denovogenes]_112544	99.60	5e-180	bbrc:B7019_0461	K00215	dapB	4-hydroxy-tetrahydrodipicolinate reductase	1.17.1.8	Metabolism--Biosynthesis of other secondary metabolites--Monobactam biosynthesis|Metabolism--Amino acid metabolism--Lysine biosynthesis|Metabolism--Global and overview maps--Metabolic pathways|Metabolism--Global and overview maps--Biosynthesis of secondary metabolites|Metabolism--Global and overview maps--Microbial metabolism in diverse environments|Metabolism--Global and overview maps--Biosynthesis of antibiotics|Metabolism--Global and overview maps--Biosynthesis of amino acids

[denovogenes]_112546	73.79	3e-132	rho:RHOM_04720	K06147	ABCB-BAC	ATP-binding cassette, subfamily B, bacterial	--	--

[denovogenes]_112551	99.60	3e-179	bbrv:B689b_1932	K02528	ksgA	16S rRNA (adenine1518-N6/adenine1519-N6)-dimethyltransferase	2.1.1.182	--

[denovogenes]_112553	56.28	3e-85	rho:RHOM_05915	K06147	ABCB-BAC	ATP-binding cassette, subfamily B, bacterial	--	--

[denovogenes]_112555	93.60	4e-173	bcel:BcellWH2_05052	K02500	hisF	cyclase	4.1.3.-	Metabolism--Amino acid metabolism--Histidine metabolism|Metabolism--Global and overview maps--Metabolic pathways|Metabolism--Global and overview maps--Biosynthesis of secondary metabolites|Metabolism--Global and overview maps--Biosynthesis of amino acids

[denovogenes]_112556	99.60	5e-168	pae:PA4223	K06148	ABCC-BAC	ATP-binding cassette, subfamily C, bacterial	--	--

[denovogenes]_112557	100.00	0.0	pdi:BDI_2026	K00812	aspB	aspartate aminotransferase	2.6.1.1	Metabolism--Amino acid metabolism--Arginine biosynthesis|Metabolism--Amino acid metabolism--Alanine, aspartate and glutamate metabolism|Metabolism--Amino acid metabolism--Cysteine and methionine metabolism|Metabolism--Amino acid metabolism--Arginine and proline metabolism|Metabolism--Amino acid metabolism--Tyrosine metabolism|Metabolism--Amino acid metabolism--Phenylalanine metabolism|Metabolism--Amino acid metabolism--Phenylalanine, tyrosine and tryptophan biosynthesis|Metabolism--Biosynthesis of other secondary metabolites--Novobiocin biosynthesis|Metabolism--Biosynthesis of other secondary metabolites--Isoquinoline alkaloid biosynthesis|Metabolism--Biosynthesis of other secondary metabolites--Tropane, piperidine and pyridine alkaloid biosynthesis|Metabolism--Global and overview maps--Metabolic pathways|Metabolism--Global and overview maps--Biosynthesis of secondary metabolites|Metabolism--Global and overview maps--Biosynthesis of antibiotics|Metabolism--Global and overview maps--2-Oxocarboxylic acid metabolism|Metabolism--Global and overview maps--Biosynthesis of amino acids

[denovogenes]_112559	47.24	1e-77	pdi:BDI_0805	K02477	K02477	two-component system, LytT family, response regulator	--	--

[denovogenes]_112560	40.78	3e-46	dto:TOL2_C03820	K00059	fabG	3-oxoacyl-[acyl-carrier protein] reductase	1.1.1.100	Metabolism--Lipid metabolism--Fatty acid biosynthesis|Metabolism--Metabolism of cofactors and vitamins--Biotin metabolism|Metabolism--Lipid metabolism--Biosynthesis of unsaturated fatty acids|Metabolism--Global and overview maps--Metabolic pathways|Metabolism--Global and overview maps--Fatty acid metabolism

[denovogenes]_112561	99.20	0.0	cfd:CFNIH1_08760	K02058	ABC.SS.S	simple sugar transport system substrate-binding protein	--	--

[denovogenes]_112563	55.56	5e-99	paci:A4V11_00290	K00065	kduD	2-deoxy-D-gluconate 3-dehydrogenase	1.1.1.125	Metabolism--Carbohydrate metabolism--Pentose and glucuronate interconversions

[denovogenes]_112565	66.40	2e-119	sgp:SpiGrapes_1815	K01937	pyrG, CTPS	CTP synthase	6.3.4.2	Metabolism--Nucleotide metabolism--Pyrimidine metabolism|Metabolism--Global and overview maps--Metabolic pathways

[denovogenes]_112568	100.00	0.0	paeu:BN889_03081	K09471	puuB, ordL	gamma-glutamylputrescine oxidase	1.4.3.-	Metabolism--Amino acid metabolism--Arginine and proline metabolism|Metabolism--Global and overview maps--Metabolic pathways

[denovogenes]_112569	81.78	3e-148	ash:AL1_29730	K00979	kdsB	3-deoxy-manno-octulosonate cytidylyltransferase (CMP-KDO synthetase)	2.7.7.38	Metabolism--Glycan biosynthesis and metabolism--Lipopolysaccharide biosynthesis|Metabolism--Global and overview maps--Metabolic pathways

[denovogenes]_11257	79.76	0.0	bdo:EL88_16270	K12373	HEXA_B	hexosaminidase	3.2.1.52	Metabolism--Glycan biosynthesis and metabolism--Other glycan degradation|Metabolism--Glycan biosynthesis and metabolism--Various types of N-glycan biosynthesis|Metabolism--Carbohydrate metabolism--Amino sugar and nucleotide sugar metabolism|Metabolism--Glycan biosynthesis and metabolism--Glycosaminoglycan degradation|Metabolism--Glycan biosynthesis and metabolism--Glycosphingolipid biosynthesis - globo and isoglobo series|Metabolism--Glycan biosynthesis and metabolism--Glycosphingolipid biosynthesis - ganglio series|Metabolism--Global and overview maps--Metabolic pathways|Cellular Processes--Transport and catabolism--Lysosome

[denovogenes]_112571	70.20	3e-133	eel:EUBELI_01707	K15772	ganQ	arabinogalactan oligomer / maltooligosaccharide transport system permease protein	--	Environmental Information Processing--Membrane transport--ABC transporters

[denovogenes]_112572	41.67	2e-62	bpw:WESB_1268	K07045	K07045	uncharacterized protein	--	--

[denovogenes]_112573	98.41	0.0	pmib:BB2000_3161	K00989	rph	ribonuclease PH	2.7.7.56	--

[denovogenes]_112575	48.61	3e-73	ruk:A4V00_16080	K04023	eutH	ethanolamine transporter	--	--

[denovogenes]_112576	94.38	2e-174	bfb:VU15_16145	K06201	cutC	copper homeostasis protein	--	--

[denovogenes]_112577	96.02	3e-180	lacy:A4V08_05640	K01813	rhaA	L-rhamnose isomerase	5.3.1.14	Metabolism--Carbohydrate metabolism--Fructose and mannose metabolism|Metabolism--Global and overview maps--Microbial metabolism in diverse environments

[denovogenes]_112578	43.46	6e-45	ppm:PPSC2_28180	K02335	DPO1, polA	DNA polymerase I	2.7.7.7	Metabolism--Nucleotide metabolism--Purine metabolism|Metabolism--Nucleotide metabolism--Pyrimidine metabolism|Metabolism--Global and overview maps--Metabolic pathways|Genetic Information Processing--Replication and repair--DNA replication|Genetic Information Processing--Replication and repair--Base excision repair|Genetic Information Processing--Replication and repair--Nucleotide excision repair|Genetic Information Processing--Replication and repair--Homologous recombination

[denovogenes]_112580	91.13	2e-167	pdi:BDI_0708	K03218	rlmB	23S rRNA (guanosine2251-2'-O)-methyltransferase	2.1.1.185	--

[denovogenes]_112582	49.57	6e-27	bprl:CL2_09420	K07720	yesN	two-component system, response regulator YesN	--	Environmental Information Processing--Signal transduction--Two-component system

[denovogenes]_112584	80.63	4e-145	bprs:CK3_21100	K13891	gsiD	glutathione transport system permease protein	--	Environmental Information Processing--Membrane transport--ABC transporters

[denovogenes]_112585	50.80	2e-86	ecos:EC958_3104	K11891	impL, vasK, icmF	type VI secretion system protein ImpL	--	Cellular Processes--Cellular community - prokaryotes--Biofilm formation - Pseudomonas aeruginosa|Environmental Information Processing--Membrane transport--Bacterial secretion system

[denovogenes]_112586	74.37	5e-131	rob:CK5_20950	K07009	K07009	uncharacterized protein	--	--

[denovogenes]_112587	99.20	0.0	bfb:VU15_15060	K03521	fixA, etfB	electron transfer flavoprotein beta subunit	--	--

[denovogenes]_112589	42.11	3e-60	sjp:SJA_C1-05590	K15237	linC	2,5-dichloro-2,5-cyclohexadiene-1,4-diol dehydrogenase 1	1.3.1.-	Metabolism--Xenobiotics biodegradation and metabolism--Chlorocyclohexane and chlorobenzene degradation|Metabolism--Global and overview maps--Metabolic pathways|Metabolism--Global and overview maps--Microbial metabolism in diverse environments

[denovogenes]_11259	97.32	0.0	bhl:Bache_1050	K03046	rpoC	DNA-directed RNA polymerase subunit beta'	2.7.7.6	Metabolism--Nucleotide metabolism--Purine metabolism|Metabolism--Nucleotide metabolism--Pyrimidine metabolism|Metabolism--Global and overview maps--Metabolic pathways|Genetic Information Processing--Transcription--RNA polymerase

[denovogenes]_112592	95.97	1e-175	pmib:BB2000_2505	K01409	KAE1, tsaD, QRI7	N6-L-threonylcarbamoyladenine synthase	2.3.1.234	--

[denovogenes]_112593	90.04	4e-166	ror:RORB6_07540	K02611	--	--	--	Metabolism--Amino acid metabolism--Phenylalanine metabolism|Metabolism--Global and overview maps--Microbial metabolism in diverse environments

[denovogenes]_112594	55.16	2e-82	cgo:Corgl_0658	K07313	pphA	serine/threonine protein phosphatase 1	3.1.3.16	--

[denovogenes]_112595	71.65	1e-131	bhl:Bache_0777	K09815	znuA	zinc transport system substrate-binding protein	--	Environmental Information Processing--Membrane transport--ABC transporters

[denovogenes]_112597	70.95	3e-122	cso:CLS_30790	K06147	ABCB-BAC	ATP-binding cassette, subfamily B, bacterial	--	--

[denovogenes]_112598	53.20	1e-85	bbe:BBR47_58350	K14415	RTCB, rtcB	tRNA-splicing ligase RtcB	6.5.1.3	--

[denovogenes]_1126	63.49	0.0	rto:RTO_17740	K03529	smc	chromosome segregation protein	--	--

[denovogenes]_11260	67.41	0.0	bdn:BBDE_1590	K05349	bglX	beta-glucosidase	3.2.1.21	Metabolism--Metabolism of other amino acids--Cyanoamino acid metabolism|Metabolism--Carbohydrate metabolism--Starch and sucrose metabolism|Metabolism--Biosynthesis of other secondary metabolites--Phenylpropanoid biosynthesis|Metabolism--Global and overview maps--Metabolic pathways|Metabolism--Global and overview maps--Biosynthesis of secondary metabolites

[denovogenes]_112600	43.60	1e-57	pen:PSEEN0118	K03566	gcvA	LysR family transcriptional regulator, glycine cleavage system transcriptional activator	--	Cellular Processes--Cellular community - prokaryotes--Biofilm formation - Escherichia coli

[denovogenes]_112603	99.20	7e-178	bbrc:B7019_2046	K01182	IMA, malL	oligo-1,6-glucosidase	3.2.1.10	Metabolism--Carbohydrate metabolism--Galactose metabolism|Metabolism--Carbohydrate metabolism--Starch and sucrose metabolism|Metabolism--Global and overview maps--Metabolic pathways

[denovogenes]_112604	87.30	1e-160	pdi:BDI_3941	K00946	thiL	thiamine-monophosphate kinase	2.7.4.16	Metabolism--Metabolism of cofactors and vitamins--Thiamine metabolism|Metabolism--Global and overview maps--Metabolic pathways

[denovogenes]_112606	98.02	0.0	pdi:BDI_3293	K06859	pgi1	glucose-6-phosphate isomerase, archaeal	5.3.1.9	Metabolism--Carbohydrate metabolism--Glycolysis / Gluconeogenesis|Metabolism--Carbohydrate metabolism--Pentose phosphate pathway|Metabolism--Carbohydrate metabolism--Starch and sucrose metabolism|Metabolism--Carbohydrate metabolism--Amino sugar and nucleotide sugar metabolism|Metabolism--Global and overview maps--Metabolic pathways|Metabolism--Global and overview maps--Biosynthesis of secondary metabolites|Metabolism--Global and overview maps--Microbial metabolism in diverse environments|Metabolism--Global and overview maps--Biosynthesis of antibiotics|Metabolism--Global and overview maps--Carbon metabolism

[denovogenes]_112607	84.92	5e-154	boa:Bovatus_04562	K11537	xapB	MFS transporter, NHS family, xanthosine permease	--	--

[denovogenes]_112609	44.89	2e-60	kra:Krad_0424	K01420	fnr	CRP/FNR family transcriptional regulator, anaerobic regulatory protein	--	--

[denovogenes]_112610	49.01	3e-74	psn:Pedsa_3755	K18581	ugl	unsaturated chondroitin disaccharide hydrolase	3.2.1.180	--

[denovogenes]_112611	58.26	3e-99	eft:M395_08275	K02795	PTS-Man-EIIC, manY	PTS system, mannose-specific IIC component	--	Metabolism--Carbohydrate metabolism--Fructose and mannose metabolism|Metabolism--Carbohydrate metabolism--Amino sugar and nucleotide sugar metabolism|Metabolism--Global and overview maps--Metabolic pathways|Environmental Information Processing--Membrane transport--Phosphotransferase system (PTS)

[denovogenes]_112616	70.24	3e-130	cbv:U729_1608	K01937	pyrG, CTPS	CTP synthase	6.3.4.2	Metabolism--Nucleotide metabolism--Pyrimidine metabolism|Metabolism--Global and overview maps--Metabolic pathways

[denovogenes]_112617	50.98	2e-84	lmoe:BN418_2078	K00265	gltB	glutamate synthase (NADPH/NADH) large chain	1.4.1.13 1.4.1.14	Metabolism--Amino acid metabolism--Alanine, aspartate and glutamate metabolism|Metabolism--Energy metabolism--Nitrogen metabolism|Metabolism--Global and overview maps--Metabolic pathways|Metabolism--Global and overview maps--Biosynthesis of secondary metabolites|Metabolism--Global and overview maps--Microbial metabolism in diverse environments|Metabolism--Global and overview maps--Biosynthesis of antibiotics|Metabolism--Global and overview maps--Biosynthesis of amino acids

[denovogenes]_11262	73.30	0.0	dvg:Deval_2820	K06158	ABCF3	ATP-binding cassette, subfamily F, member 3	--	--

[denovogenes]_112623	83.26	5e-135	byl:A4V09_15130	K03686	dnaJ	molecular chaperone DnaJ	--	--

[denovogenes]_112627	78.97	2e-145	cgo:Corgl_1461	K01876	DARS, aspS	aspartyl-tRNA synthetase	6.1.1.12	Genetic Information Processing--Translation--Aminoacyl-tRNA biosynthesis

[denovogenes]_112628	78.17	3e-140	rto:RTO_24000	K00003	E1.1.1.3	homoserine dehydrogenase	1.1.1.3	Metabolism--Amino acid metabolism--Glycine, serine and threonine metabolism|Metabolism--Amino acid metabolism--Cysteine and methionine metabolism|Metabolism--Amino acid metabolism--Lysine biosynthesis|Metabolism--Global and overview maps--Metabolic pathways|Metabolism--Global and overview maps--Biosynthesis of secondary metabolites|Metabolism--Global and overview maps--Microbial metabolism in diverse environments|Metabolism--Global and overview maps--Biosynthesis of antibiotics|Metabolism--Global and overview maps--Biosynthesis of amino acids

[denovogenes]_11263	99.11	0.0	bth:BT_0757	K01190	lacZ	beta-galactosidase	3.2.1.23	Metabolism--Carbohydrate metabolism--Galactose metabolism|Metabolism--Glycan biosynthesis and metabolism--Other glycan degradation|Metabolism--Lipid metabolism--Sphingolipid metabolism|Metabolism--Global and overview maps--Metabolic pathways

[denovogenes]_112632	99.60	0.0	cdg:CDBI1_08160	K00283	gcvPB	glycine dehydrogenase subunit 2	1.4.4.2	Metabolism--Amino acid metabolism--Glycine, serine and threonine metabolism|Metabolism--Carbohydrate metabolism--Glyoxylate and dicarboxylate metabolism|Metabolism--Global and overview maps--Metabolic pathways|Metabolism--Global and overview maps--Biosynthesis of secondary metabolites|Metabolism--Global and overview maps--Biosynthesis of antibiotics|Metabolism--Global and overview maps--Carbon metabolism

[denovogenes]_112633	50.79	9e-78	saz:Sama_1319	K03309	TC.DAACS	dicarboxylate/amino acid:cation (Na+ or H+) symporter, DAACS family	--	--

[denovogenes]_112634	100.00	0.0	eoc:CE10_1668	K00027	ME2, sfcA, maeA	malate dehydrogenase (oxaloacetate-decarboxylating)	1.1.1.38	Metabolism--Carbohydrate metabolism--Pyruvate metabolism|Metabolism--Global and overview maps--Carbon metabolism|Environmental Information Processing--Signal transduction--Two-component system

[denovogenes]_112635	58.33	4e-96	eel:EUBELI_01005	K03733	xerC	integrase/recombinase XerC	--	--

[denovogenes]_112637	81.35	5e-147	afd:Alfi_1639	K03590	ftsA	cell division protein FtsA	--	Cellular Processes--Cell growth and death--Cell cycle - Caulobacter

[denovogenes]_112638	73.02	3e-134	aeq:AEQU_1769	K01951	guaA, GMPS	GMP synthase (glutamine-hydrolysing)	6.3.5.2	Metabolism--Nucleotide metabolism--Purine metabolism|Metabolism--Xenobiotics biodegradation and metabolism--Drug metabolism - other enzymes|Metabolism--Global and overview maps--Metabolic pathways

[denovogenes]_112639	94.65	2e-160	byl:A4V09_03450	K11690	dctM	C4-dicarboxylate transporter, DctM subunit	--	Environmental Information Processing--Signal transduction--Two-component system

[denovogenes]_11264	100.00	0.0	paeo:M801_5539	K13590	dgcB	diguanylate cyclase	2.7.7.65	Cellular Processes--Cell growth and death--Cell cycle - Caulobacter

[denovogenes]_112641	52.22	2e-87	psr:PSTAA_2161	K01971	ligD	bifunctional non-homologous end joining protein LigD	6.5.1.1	Genetic Information Processing--Replication and repair--Non-homologous end-joining

[denovogenes]_112642	64.20	2e-101	cbb:CLD_2618	K06901	pbuG	putative MFS transporter, AGZA family, xanthine/uracil permease	--	--

[denovogenes]_112643	76.59	2e-135	ypx:YPD8_1595	K01874	MARS, metG	methionyl-tRNA synthetase	6.1.1.10	Metabolism--Metabolism of other amino acids--Selenocompound metabolism|Genetic Information Processing--Translation--Aminoacyl-tRNA biosynthesis

[denovogenes]_112644	45.83	2e-56	lmob:BN419_2424	K01191	E3.2.1.24	alpha-mannosidase	3.2.1.24	Metabolism--Glycan biosynthesis and metabolism--Other glycan degradation

[denovogenes]_112645	98.02	0.0	pvl:AOB99_00010	K02338	DPO3B, dnaN	DNA polymerase III subunit beta	2.7.7.7	Metabolism--Nucleotide metabolism--Purine metabolism|Metabolism--Nucleotide metabolism--Pyrimidine metabolism|Metabolism--Global and overview maps--Metabolic pathways|Genetic Information Processing--Replication and repair--DNA replication|Genetic Information Processing--Replication and repair--Mismatch repair|Genetic Information Processing--Replication and repair--Homologous recombination

[denovogenes]_112647	99.60	0.0	btho:Btheta7330_00063	K00240	sdhB, frdB	succinate dehydrogenase / fumarate reductase, iron-sulfur subunit	1.3.5.1 1.3.5.4	Metabolism--Carbohydrate metabolism--Citrate cycle (TCA cycle)|Metabolism--Energy metabolism--Oxidative phosphorylation|Metabolism--Carbohydrate metabolism--Butanoate metabolism|Metabolism--Energy metabolism--Carbon fixation pathways in prokaryotes|Metabolism--Global and overview maps--Metabolic pathways|Metabolism--Global and overview maps--Biosynthesis of secondary metabolites|Metabolism--Global and overview maps--Microbial metabolism in diverse environments|Metabolism--Global and overview maps--Biosynthesis of antibiotics|Metabolism--Global and overview maps--Carbon metabolism

[denovogenes]_11265	81.23	0.0	pdi:BDI_3671	K04041	fbp3	fructose-1,6-bisphosphatase III	3.1.3.11	Metabolism--Carbohydrate metabolism--Glycolysis / Gluconeogenesis|Metabolism--Carbohydrate metabolism--Pentose phosphate pathway|Metabolism--Carbohydrate metabolism--Fructose and mannose metabolism|Metabolism--Energy metabolism--Methane metabolism|Metabolism--Energy metabolism--Carbon fixation in photosynthetic organisms|Metabolism--Global and overview maps--Metabolic pathways|Metabolism--Global and overview maps--Biosynthesis of secondary metabolites|Metabolism--Global and overview maps--Microbial metabolism in diverse environments|Metabolism--Global and overview maps--Biosynthesis of antibiotics|Metabolism--Global and overview maps--Carbon metabolism

[denovogenes]_112651	41.88	6e-40	erb:A4V01_11860	K06385	spoIIP	stage II sporulation protein P	--	--

[denovogenes]_112652	92.80	3e-177	bfg:BF638R_3662	K00571	E2.1.1.72	site-specific DNA-methyltransferase (adenine-specific)	2.1.1.72	--

[denovogenes]_112654	44.09	3e-75	pdi:BDI_0805	K02477	K02477	two-component system, LytT family, response regulator	--	--

[denovogenes]_112658	56.52	2e-95	saur:SABB_00246	K00806	uppS	undecaprenyl diphosphate synthase	2.5.1.31	Metabolism--Metabolism of terpenoids and polyketides--Terpenoid backbone biosynthesis|Metabolism--Global and overview maps--Biosynthesis of secondary metabolites

[denovogenes]_112659	93.20	8e-177	bfg:BF638R_3662	K00571	E2.1.1.72	site-specific DNA-methyltransferase (adenine-specific)	2.1.1.72	--

[denovogenes]_112660	50.20	4e-82	bld:BLi01773	K02823	pyrDII	dihydroorotate dehydrogenase electron transfer subunit	--	Metabolism--Nucleotide metabolism--Pyrimidine metabolism|Metabolism--Global and overview maps--Metabolic pathways

[denovogenes]_112661	78.00	5e-148	lacy:A4V08_18660	K02036	pstB	phosphate transport system ATP-binding protein	3.6.3.27	Environmental Information Processing--Membrane transport--ABC transporters

[denovogenes]_112662	45.78	2e-55	bty:Btoyo_2412	K03478	chbG	chitin disaccharide deacetylase	3.5.1.105	--

[denovogenes]_112665	96.81	2e-178	boa:Bovatus_04665	K06201	cutC	copper homeostasis protein	--	--

[denovogenes]_112671	73.20	6e-139	erb:A4V01_14305	K01142	E3.1.11.2, xthA	exodeoxyribonuclease III	3.1.11.2	Genetic Information Processing--Replication and repair--Base excision repair

[denovogenes]_112672	76.49	4e-133	byl:A4V09_06180	K02003	ABC.CD.A	putative ABC transport system ATP-binding protein	--	--

[denovogenes]_112677	99.60	0.0	btho:Btheta7330_00063	K00240	sdhB, frdB	succinate dehydrogenase / fumarate reductase, iron-sulfur subunit	1.3.5.1 1.3.5.4	Metabolism--Carbohydrate metabolism--Citrate cycle (TCA cycle)|Metabolism--Energy metabolism--Oxidative phosphorylation|Metabolism--Carbohydrate metabolism--Butanoate metabolism|Metabolism--Energy metabolism--Carbon fixation pathways in prokaryotes|Metabolism--Global and overview maps--Metabolic pathways|Metabolism--Global and overview maps--Biosynthesis of secondary metabolites|Metabolism--Global and overview maps--Microbial metabolism in diverse environments|Metabolism--Global and overview maps--Biosynthesis of antibiotics|Metabolism--Global and overview maps--Carbon metabolism

[denovogenes]_112678	47.35	3e-65	gel:IB49_04580	K01243	mtnN, mtn, pfs	adenosylhomocysteine nucleosidase	3.2.2.9	Metabolism--Amino acid metabolism--Cysteine and methionine metabolism|Metabolism--Global and overview maps--Metabolic pathways|Metabolism--Global and overview maps--Biosynthesis of amino acids

[denovogenes]_112680	48.39	6e-76	bha:BH3661	K16698	tuaG	teichuronic acid biosynthesis glycosyltransferase TuaG	2.4.-.-	--

[denovogenes]_112681	75.60	1e-140	ipo:Ilyop_1517	K01803	TPI, tpiA	triosephosphate isomerase (TIM)	5.3.1.1	Metabolism--Carbohydrate metabolism--Glycolysis / Gluconeogenesis|Metabolism--Carbohydrate metabolism--Fructose and mannose metabolism|Metabolism--Carbohydrate metabolism--Inositol phosphate metabolism|Metabolism--Energy metabolism--Carbon fixation in photosynthetic organisms|Metabolism--Global and overview maps--Metabolic pathways|Metabolism--Global and overview maps--Biosynthesis of secondary metabolites|Metabolism--Global and overview maps--Microbial metabolism in diverse environments|Metabolism--Global and overview maps--Biosynthesis of antibiotics|Metabolism--Global and overview maps--Carbon metabolism|Metabolism--Global and overview maps--Biosynthesis of amino acids

[denovogenes]_112682	76.83	1e-134	csr:Cspa_c22770	K19267	qorB	NAD(P)H dehydrogenase (quinone)	1.6.5.2	Metabolism--Metabolism of cofactors and vitamins--Ubiquinone and other terpenoid-quinone biosynthesis|Metabolism--Global and overview maps--Biosynthesis of secondary metabolites

[denovogenes]_112686	84.06	2e-154	bcel:BcellWH2_00536	K00059	fabG	3-oxoacyl-[acyl-carrier protein] reductase	1.1.1.100	Metabolism--Lipid metabolism--Fatty acid biosynthesis|Metabolism--Metabolism of cofactors and vitamins--Biotin metabolism|Metabolism--Lipid metabolism--Biosynthesis of unsaturated fatty acids|Metabolism--Global and overview maps--Metabolic pathways|Metabolism--Global and overview maps--Fatty acid metabolism

[denovogenes]_112688	100.00	0.0	pvl:AOB99_03885	K01069	E3.1.2.6, gloB	hydroxyacylglutathione hydrolase	3.1.2.6	Metabolism--Carbohydrate metabolism--Pyruvate metabolism

[denovogenes]_112689	43.03	3e-63	csb:CLSA_c41550	K03481	glvR	RpiR family transcriptional regulator, glv operon transcriptional regulator	--	--

[denovogenes]_112691	56.68	6e-96	ipo:Ilyop_2533	K07053	E3.1.3.97	3',5'-nucleoside bisphosphate phosphatase	3.1.3.97	--

[denovogenes]_112694	41.59	8e-44	pmf:P9303_05381	K00975	glgC	glucose-1-phosphate adenylyltransferase	2.7.7.27	Metabolism--Carbohydrate metabolism--Starch and sucrose metabolism|Metabolism--Carbohydrate metabolism--Amino sugar and nucleotide sugar metabolism|Metabolism--Global and overview maps--Metabolic pathways|Metabolism--Global and overview maps--Biosynthesis of secondary metabolites|Cellular Processes--Cellular community - prokaryotes--Biofilm formation - Escherichia coli

[denovogenes]_112695	88.80	3e-166	vpr:Vpar_0869	K06168	miaB	tRNA-2-methylthio-N6-dimethylallyladenosine synthase	2.8.4.3	--

[denovogenes]_112698	91.24	2e-166	fhw:RN87_04575	K01951	guaA, GMPS	GMP synthase (glutamine-hydrolysing)	6.3.5.2	Metabolism--Nucleotide metabolism--Purine metabolism|Metabolism--Xenobiotics biodegradation and metabolism--Drug metabolism - other enzymes|Metabolism--Global and overview maps--Metabolic pathways

[denovogenes]_112699	96.41	2e-173	vpr:Vpar_1054	K00088	IMPDH, guaB	IMP dehydrogenase	1.1.1.205	Metabolism--Nucleotide metabolism--Purine metabolism|Metabolism--Xenobiotics biodegradation and metabolism--Drug metabolism - other enzymes|Metabolism--Global and overview maps--Metabolic pathways|Metabolism--Global and overview maps--Biosynthesis of secondary metabolites

[denovogenes]_1127	81.86	0.0	lacy:A4V08_00985	K03529	smc	chromosome segregation protein	--	--

[denovogenes]_112701	61.69	5e-114	tsh:Tsac_2178	K00175	korB, oorB, oforB	2-oxoglutarate/2-oxoacid ferredoxin oxidoreductase subunit beta	1.2.7.3 1.2.7.11	Metabolism--Carbohydrate metabolism--Glycolysis / Gluconeogenesis|Metabolism--Carbohydrate metabolism--Citrate cycle (TCA cycle)|Metabolism--Carbohydrate metabolism--Pyruvate metabolism|Metabolism--Carbohydrate metabolism--Butanoate metabolism|Metabolism--Energy metabolism--Carbon fixation pathways in prokaryotes|Metabolism--Global and overview maps--Metabolic pathways|Metabolism--Global and overview maps--Microbial metabolism in diverse environments|Metabolism--Global and overview maps--Biosynthesis of antibiotics|Metabolism--Global and overview maps--Carbon metabolism

[denovogenes]_112702	41.18	8e-43	pst:PSPTO_1235	K19294	algI	alginate O-acetyltransferase complex protein AlgI	--	--

[denovogenes]_112703	100.00	0.0	sfe:SFxv_4525	K03788	aphA	acid phosphatase (class B)	3.1.3.2	Metabolism--Xenobiotics biodegradation and metabolism--Aminobenzoate degradation|Metabolism--Metabolism of cofactors and vitamins--Riboflavin metabolism|Metabolism--Global and overview maps--Metabolic pathways|Metabolism--Global and overview maps--Microbial metabolism in diverse environments

[denovogenes]_112704	78.57	1e-144	bpw:WESB_1300	K00048	fucO	lactaldehyde reductase	1.1.1.77	Metabolism--Carbohydrate metabolism--Glyoxylate and dicarboxylate metabolism|Metabolism--Carbohydrate metabolism--Propanoate metabolism|Metabolism--Global and overview maps--Microbial metabolism in diverse environments

[denovogenes]_112706	87.66	3e-152	fhw:RN87_02600	K02863	RP-L1, MRPL1, rplA	large subunit ribosomal protein L1	--	Genetic Information Processing--Translation--Ribosome

[denovogenes]_112707	81.27	1e-149	eft:M395_10495	K00057	gpsA	glycerol-3-phosphate dehydrogenase (NAD(P)+)	1.1.1.94	Metabolism--Lipid metabolism--Glycerophospholipid metabolism|Metabolism--Global and overview maps--Biosynthesis of secondary metabolites

[denovogenes]_112708	77.82	5e-132	ecas:ECBG_01453	K01647	CS, gltA	citrate synthase	2.3.3.1	Metabolism--Carbohydrate metabolism--Citrate cycle (TCA cycle)|Metabolism--Carbohydrate metabolism--Glyoxylate and dicarboxylate metabolism|Metabolism--Global and overview maps--Metabolic pathways|Metabolism--Global and overview maps--Biosynthesis of secondary metabolites|Metabolism--Global and overview maps--Microbial metabolism in diverse environments|Metabolism--Global and overview maps--Biosynthesis of antibiotics|Metabolism--Global and overview maps--Carbon metabolism|Metabolism--Global and overview maps--2-Oxocarboxylic acid metabolism|Metabolism--Global and overview maps--Biosynthesis of amino acids

[denovogenes]_112710	41.54	9e-59	jab:VN23_05645	K01126	E3.1.4.46, glpQ, ugpQ	glycerophosphoryl diester phosphodiesterase	3.1.4.46	Metabolism--Lipid metabolism--Glycerophospholipid metabolism

[denovogenes]_112712	86.85	3e-147	fhw:RN87_07155	K03596	lepA	GTP-binding protein LepA	--	Human Diseases--Infectious diseases: Bacterial--Legionellosis

[denovogenes]_112713	60.24	2e-100	ecas:ECBG_02972	K09815	znuA	zinc transport system substrate-binding protein	--	Environmental Information Processing--Membrane transport--ABC transporters

[denovogenes]_112715	100.00	0.0	cpf:CPF_0751	K01470	E3.5.2.10	creatinine amidohydrolase	3.5.2.10	Metabolism--Amino acid metabolism--Arginine and proline metabolism

[denovogenes]_112718	44.18	6e-57	toc:Toce_0214	K00001	E1.1.1.1, adh	alcohol dehydrogenase	1.1.1.1	Metabolism--Carbohydrate metabolism--Glycolysis / Gluconeogenesis|Metabolism--Lipid metabolism--Fatty acid degradation|Metabolism--Amino acid metabolism--Tyrosine metabolism|Metabolism--Xenobiotics biodegradation and metabolism--Chloroalkane and chloroalkene degradation|Metabolism--Xenobiotics biodegradation and metabolism--Naphthalene degradation|Metabolism--Metabolism of cofactors and vitamins--Retinol metabolism|Metabolism--Xenobiotics biodegradation and metabolism--Metabolism of xenobiotics by cytochrome P450|Metabolism--Xenobiotics biodegradation and metabolism--Drug metabolism - cytochrome P450|Metabolism--Global and overview maps--Metabolic pathways|Metabolism--Global and overview maps--Biosynthesis of secondary metabolites|Metabolism--Global and overview maps--Microbial metabolism in diverse environments|Metabolism--Global and overview maps--Biosynthesis of antibiotics|Metabolism--Global and overview maps--Degradation of aromatic compounds

[denovogenes]_112719	67.33	1e-119	ipo:Ilyop_2196	K01945	purD	phosphoribosylamine--glycine ligase	6.3.4.13	Metabolism--Nucleotide metabolism--Purine metabolism|Metabolism--Global and overview maps--Metabolic pathways|Metabolism--Global and overview maps--Biosynthesis of secondary metabolites|Metabolism--Global and overview maps--Biosynthesis of antibiotics

[denovogenes]_11272	42.47	3e-169	ssm:Spirs_2729	K00087	ygeS, xdhA	xanthine dehydrogenase molybdenum-binding subunit	1.17.1.4	Metabolism--Nucleotide metabolism--Purine metabolism|Metabolism--Global and overview maps--Metabolic pathways|Metabolism--Global and overview maps--Microbial metabolism in diverse environments

[denovogenes]_112721	47.24	8e-64	mrs:Murru_3399	K11105	cvrA, nhaP2	cell volume regulation protein A	--	--

[denovogenes]_112722	99.54	2e-150	pdi:BDI_2301	K09680	coaW	type II pantothenate kinase	2.7.1.33	Metabolism--Metabolism of cofactors and vitamins--Pantothenate and CoA biosynthesis|Metabolism--Global and overview maps--Metabolic pathways

[denovogenes]_112725	52.50	9e-72	pgm:PGRAT_23745	K01198	xynB	xylan 1,4-beta-xylosidase	3.2.1.37	Metabolism--Carbohydrate metabolism--Amino sugar and nucleotide sugar metabolism|Metabolism--Global and overview maps--Metabolic pathways

[denovogenes]_112727	77.20	4e-147	cbz:Cbs_1795	K00215	dapB	4-hydroxy-tetrahydrodipicolinate reductase	1.17.1.8	Metabolism--Biosynthesis of other secondary metabolites--Monobactam biosynthesis|Metabolism--Amino acid metabolism--Lysine biosynthesis|Metabolism--Global and overview maps--Metabolic pathways|Metabolism--Global and overview maps--Biosynthesis of secondary metabolites|Metabolism--Global and overview maps--Microbial metabolism in diverse environments|Metabolism--Global and overview maps--Biosynthesis of antibiotics|Metabolism--Global and overview maps--Biosynthesis of amino acids

[denovogenes]_112729	100.00	0.0	mmk:MU9_2008	K06201	cutC	copper homeostasis protein	--	--

[denovogenes]_11273	56.50	0.0	lir:LAW_01167	K17686	copA, ATP7	Cu+-exporting ATPase	3.6.3.54	Human Diseases--Drug resistance: Antineoplastic--Platinum drug resistance|Environmental Information Processing--Signal transduction--MAPK signaling pathway - plant

[denovogenes]_112730	99.20	0.0	mmk:MU9_2019	K01951	guaA, GMPS	GMP synthase (glutamine-hydrolysing)	6.3.5.2	Metabolism--Nucleotide metabolism--Purine metabolism|Metabolism--Xenobiotics biodegradation and metabolism--Drug metabolism - other enzymes|Metabolism--Global and overview maps--Metabolic pathways

[denovogenes]_112731	100.00	0.0	mmk:MU9_1409	K01834	PGAM, gpmA	2,3-bisphosphoglycerate-dependent phosphoglycerate mutase	5.4.2.11	Metabolism--Carbohydrate metabolism--Glycolysis / Gluconeogenesis|Metabolism--Amino acid metabolism--Glycine, serine and threonine metabolism|Metabolism--Energy metabolism--Methane metabolism|Metabolism--Global and overview maps--Metabolic pathways|Metabolism--Global and overview maps--Biosynthesis of secondary metabolites|Metabolism--Global and overview maps--Microbial metabolism in diverse environments|Metabolism--Global and overview maps--Biosynthesis of antibiotics|Metabolism--Global and overview maps--Carbon metabolism|Metabolism--Global and overview maps--Biosynthesis of amino acids|Organismal Systems--Endocrine system--Glucagon signaling pathway|Human Diseases--Cancers: Overview--Central carbon metabolism in cancer

[denovogenes]_112732	41.37	7e-63	sjp:SJA_C1-05590	K15237	linC	2,5-dichloro-2,5-cyclohexadiene-1,4-diol dehydrogenase 1	1.3.1.-	Metabolism--Xenobiotics biodegradation and metabolism--Chlorocyclohexane and chlorobenzene degradation|Metabolism--Global and overview maps--Metabolic pathways|Metabolism--Global and overview maps--Microbial metabolism in diverse environments

[denovogenes]_112733	100.00	0.0	bfg:BF638R_2971	K00721	DPM1	dolichol-phosphate mannosyltransferase	2.4.1.83	Metabolism--Glycan biosynthesis and metabolism--N-Glycan biosynthesis|Metabolism--Global and overview maps--Metabolic pathways

[denovogenes]_112734	42.51	4e-59	ag:BAC80215	K19960	chnA	cyclohexanol dehydrogenase	1.1.1.245	Metabolism--Xenobiotics biodegradation and metabolism--Caprolactam degradation

[denovogenes]_112735	100.00	6e-180	mmk:MU9_2869	K06203	cysZ	CysZ protein	--	--

[denovogenes]_112736	100.00	0.0	mmk:MU9_3046	K18778	zapD	cell division protein ZapD	--	--

[denovogenes]_112737	100.00	0.0	mmk:MU9_1299	K00554	trmD	tRNA (guanine37-N1)-methyltransferase	2.1.1.228	--

[denovogenes]_112738	99.60	0.0	mmk:MU9_572	K01803	TPI, tpiA	triosephosphate isomerase (TIM)	5.3.1.1	Metabolism--Carbohydrate metabolism--Glycolysis / Gluconeogenesis|Metabolism--Carbohydrate metabolism--Fructose and mannose metabolism|Metabolism--Carbohydrate metabolism--Inositol phosphate metabolism|Metabolism--Energy metabolism--Carbon fixation in photosynthetic organisms|Metabolism--Global and overview maps--Metabolic pathways|Metabolism--Global and overview maps--Biosynthesis of secondary metabolites|Metabolism--Global and overview maps--Microbial metabolism in diverse environments|Metabolism--Global and overview maps--Biosynthesis of antibiotics|Metabolism--Global and overview maps--Carbon metabolism|Metabolism--Global and overview maps--Biosynthesis of amino acids

[denovogenes]_11274	77.10	0.0	pdi:BDI_1398	K03628	rho	transcription termination factor Rho	--	Genetic Information Processing--Folding, sorting and degradation--RNA degradation

[denovogenes]_112740	100.00	2e-174	mmk:MU9_2773	K07090	K07090	uncharacterized protein	--	--

[denovogenes]_112741	58.57	9e-99	cbv:U729_244	K02233	E2.7.8.26, cobS, cobV	adenosylcobinamide-GDP ribazoletransferase	2.7.8.26	Metabolism--Metabolism of cofactors and vitamins--Porphyrin and chlorophyll metabolism|Metabolism--Global and overview maps--Metabolic pathways

[denovogenes]_112743	50.20	1e-87	euc:EC1_00910	K10711	frlR	GntR family transcriptional regulator, frlABCD operon transcriptional regulator	--	--

[denovogenes]_112744	99.60	0.0	bfb:VU15_12960	K02500	hisF	cyclase	4.1.3.-	Metabolism--Amino acid metabolism--Histidine metabolism|Metabolism--Global and overview maps--Metabolic pathways|Metabolism--Global and overview maps--Biosynthesis of secondary metabolites|Metabolism--Global and overview maps--Biosynthesis of amino acids

[denovogenes]_112745	73.49	7e-138	vte:BHY08_05045	K02041	phnC	phosphonate transport system ATP-binding protein	3.6.3.28	Environmental Information Processing--Membrane transport--ABC transporters

[denovogenes]_112747	100.00	0.0	mmk:MU9_993	K07387	K07387	putative metalloprotease	3.4.24.-	--

[denovogenes]_112750	52.28	4e-84	csr:Cspa_c03210	K02619	pabC	4-amino-4-deoxychorismate lyase	4.1.3.38	Metabolism--Metabolism of cofactors and vitamins--Folate biosynthesis

[denovogenes]_112751	99.60	0.0	bfb:VU15_21585	K01719	hemD, UROS	uroporphyrinogen-III synthase	4.2.1.75	Metabolism--Metabolism of cofactors and vitamins--Porphyrin and chlorophyll metabolism|Metabolism--Global and overview maps--Metabolic pathways|Metabolism--Global and overview maps--Biosynthesis of secondary metabolites

[denovogenes]_112752	55.78	2e-96	cbv:U729_244	K02233	E2.7.8.26, cobS, cobV	adenosylcobinamide-GDP ribazoletransferase	2.7.8.26	Metabolism--Metabolism of cofactors and vitamins--Porphyrin and chlorophyll metabolism|Metabolism--Global and overview maps--Metabolic pathways

[denovogenes]_112754	70.75	3e-131	cpf:CPF_1154	K00076	hdhA	7-alpha-hydroxysteroid dehydrogenase	1.1.1.159	--

[denovogenes]_112755	89.92	3e-163	cbv:U729_1515	K00989	rph	ribonuclease PH	2.7.7.56	--

[denovogenes]_112756	67.47	2e-121	ecas:ECBG_01813	K05896	scpA	segregation and condensation protein A	--	--

[denovogenes]_112757	84.80	6e-158	pdf:CD630DERM_34260	K05936	cobM, cbiF	precorrin-4/cobalt-precorrin-4 C11-methyltransferase	2.1.1.133 2.1.1.271	Metabolism--Metabolism of cofactors and vitamins--Porphyrin and chlorophyll metabolism|Metabolism--Global and overview maps--Metabolic pathways

[denovogenes]_11276	59.73	0.0	lir:LAW_00727	K03703	uvrC	excinuclease ABC subunit C	--	Genetic Information Processing--Replication and repair--Nucleotide excision repair

[denovogenes]_112760	100.00	0.0	ene:ENT_26490	K00208	fabI	enoyl-[acyl-carrier protein] reductase I	1.3.1.9 1.3.1.10	Metabolism--Lipid metabolism--Fatty acid biosynthesis|Metabolism--Metabolism of cofactors and vitamins--Biotin metabolism|Metabolism--Global and overview maps--Metabolic pathways|Metabolism--Global and overview maps--Fatty acid metabolism

[denovogenes]_112763	86.40	2e-161	fpla:A4U99_09720	K02013	ABC.FEV.A	iron complex transport system ATP-binding protein	3.6.3.34	Environmental Information Processing--Membrane transport--ABC transporters

[denovogenes]_112765	68.27	1e-124	ecas:ECBG_02554	K00215	dapB	4-hydroxy-tetrahydrodipicolinate reductase	1.17.1.8	Metabolism--Biosynthesis of other secondary metabolites--Monobactam biosynthesis|Metabolism--Amino acid metabolism--Lysine biosynthesis|Metabolism--Global and overview maps--Metabolic pathways|Metabolism--Global and overview maps--Biosynthesis of secondary metabolites|Metabolism--Global and overview maps--Microbial metabolism in diverse environments|Metabolism--Global and overview maps--Biosynthesis of antibiotics|Metabolism--Global and overview maps--Biosynthesis of amino acids

[denovogenes]_112766	85.60	4e-156	cif:AL515_11265	K02013	ABC.FEV.A	iron complex transport system ATP-binding protein	3.6.3.34	Environmental Information Processing--Membrane transport--ABC transporters

[denovogenes]_112770	99.15	1e-170	stb:SGPB_0027	K01923	purC	phosphoribosylaminoimidazole-succinocarboxamide synthase	6.3.2.6	Metabolism--Nucleotide metabolism--Purine metabolism|Metabolism--Global and overview maps--Metabolic pathways|Metabolism--Global and overview maps--Biosynthesis of secondary metabolites|Metabolism--Global and overview maps--Biosynthesis of antibiotics

[denovogenes]_112772	71.89	4e-132	efa:EF1839	K02530	lacR	DeoR family transcriptional regulator, lactose phosphotransferase system repressor	--	--

[denovogenes]_112773	97.20	3e-176	ibu:IB211_03274	K03205	virD4, lvhD4	type IV secretion system protein VirD4	--	Environmental Information Processing--Membrane transport--Bacterial secretion system

[denovogenes]_112774	80.80	2e-148	cle:Clole_0360	K02003	ABC.CD.A	putative ABC transport system ATP-binding protein	--	--

[denovogenes]_112777	60.00	1e-99	cbv:U729_2068	K02050	ABC.SN.P	NitT/TauT family transport system permease protein	--	--

[denovogenes]_112778	100.00	0.0	efi:OG1RF_10586	K01104	E3.1.3.48	protein-tyrosine phosphatase	3.1.3.48	--

[denovogenes]_112780	74.00	1e-121	pdf:CD630DERM_24490	K09761	rsmE	16S rRNA (uracil1498-N3)-methyltransferase	2.1.1.193	--

[denovogenes]_112782	51.79	1e-75	dau:Daud_1897	K07775	resD	two-component system, OmpR family, response regulator ResD	--	Environmental Information Processing--Signal transduction--Two-component system

[denovogenes]_112783	100.00	0.0	pvl:AOB99_15950	K10824	nikE	nickel transport system ATP-binding protein	3.6.3.24	Environmental Information Processing--Membrane transport--ABC transporters

[denovogenes]_112784	48.16	1e-83	nth:Nther_1461	K01358	clpP, CLPP	ATP-dependent Clp protease, protease subunit	3.4.21.92	Cellular Processes--Cell growth and death--Cell cycle - Caulobacter|Organismal Systems--Aging--Longevity regulating pathway - worm

[denovogenes]_112785	64.92	2e-108	cld:CLSPO_c17520	K03436	fruR2, fruR	DeoR family transcriptional regulator, fructose operon transcriptional repressor	--	--

[denovogenes]_112788	100.00	7e-154	cif:AL515_12990	K03969	--	--	--	--

[denovogenes]_112789	66.08	4e-120	cbut:ATN24_11400	K02768	PTS-Fru-EIIA, fruB	PTS system, fructose-specific IIA component	2.7.1.202	Metabolism--Carbohydrate metabolism--Fructose and mannose metabolism|Metabolism--Global and overview maps--Metabolic pathways|Metabolism--Global and overview maps--Microbial metabolism in diverse environments|Environmental Information Processing--Membrane transport--Phosphotransferase system (PTS)

[denovogenes]_112789	66.08	4e-120	cbut:ATN24_11400	K02769	PTS-Fru-EIIB, fruA	PTS system, fructose-specific IIB component	2.7.1.202	Metabolism--Carbohydrate metabolism--Fructose and mannose metabolism|Metabolism--Global and overview maps--Metabolic pathways|Metabolism--Global and overview maps--Microbial metabolism in diverse environments|Environmental Information Processing--Membrane transport--Phosphotransferase system (PTS)

[denovogenes]_112789	66.08	4e-120	cbut:ATN24_11400	K02770	PTS-Fru-EIIC, fruA	PTS system, fructose-specific IIC component	--	Metabolism--Carbohydrate metabolism--Fructose and mannose metabolism|Metabolism--Global and overview maps--Metabolic pathways|Metabolism--Global and overview maps--Microbial metabolism in diverse environments|Environmental Information Processing--Membrane transport--Phosphotransferase system (PTS)

[denovogenes]_112792	95.20	1e-179	bhl:Bache_0760	K01719	hemD, UROS	uroporphyrinogen-III synthase	4.2.1.75	Metabolism--Metabolism of cofactors and vitamins--Porphyrin and chlorophyll metabolism|Metabolism--Global and overview maps--Metabolic pathways|Metabolism--Global and overview maps--Biosynthesis of secondary metabolites

[denovogenes]_112793	67.20	5e-127	cbk:CLL_A2487	K00215	dapB	4-hydroxy-tetrahydrodipicolinate reductase	1.17.1.8	Metabolism--Biosynthesis of other secondary metabolites--Monobactam biosynthesis|Metabolism--Amino acid metabolism--Lysine biosynthesis|Metabolism--Global and overview maps--Metabolic pathways|Metabolism--Global and overview maps--Biosynthesis of secondary metabolites|Metabolism--Global and overview maps--Microbial metabolism in diverse environments|Metabolism--Global and overview maps--Biosynthesis of antibiotics|Metabolism--Global and overview maps--Biosynthesis of amino acids

[denovogenes]_112794	100.00	0.0	pvl:AOB99_04670	K00554	trmD	tRNA (guanine37-N1)-methyltransferase	2.1.1.228	--

[denovogenes]_112795	71.43	2e-130	csb:CLSA_c12490	K02823	pyrDII	dihydroorotate dehydrogenase electron transfer subunit	--	Metabolism--Nucleotide metabolism--Pyrimidine metabolism|Metabolism--Global and overview maps--Metabolic pathways

[denovogenes]_112797	44.74	7e-64	pmr:PMI0300	K12519	papD	chaperone protein PapD	--	--

[denovogenes]_112798	97.60	8e-177	pmib:BB2000_3468	K07126	K07126	uncharacterized protein	--	--

[denovogenes]_1128	68.21	0.0	lacy:A4V08_00985	K03529	smc	chromosome segregation protein	--	--

[denovogenes]_11280	98.81	0.0	bvu:BVU_0620	K01187	malZ	alpha-glucosidase	3.2.1.20	Metabolism--Carbohydrate metabolism--Galactose metabolism|Metabolism--Carbohydrate metabolism--Starch and sucrose metabolism|Metabolism--Global and overview maps--Metabolic pathways

[denovogenes]_112803	51.57	1e-72	tte:TTE2474	K02483	K02483	two-component system, OmpR family, response regulator	--	--

[denovogenes]_112804	99.20	3e-177	cbut:ATN24_01860	K13292	lgt, umpA	phosphatidylglycerol:prolipoprotein diacylglycerol transferase	2.-.-.-	--

[denovogenes]_112805	57.72	2e-97	camp:CFT03427_1044	K16850	uxaA2	altronate dehydratase large subunit	4.2.1.7	Metabolism--Carbohydrate metabolism--Pentose and glucuronate interconversions|Metabolism--Global and overview maps--Metabolic pathways

[denovogenes]_112807	99.60	0.0	pvl:AOB99_02320	K07387	K07387	putative metalloprotease	3.4.24.-	--

[denovogenes]_112808	42.29	5e-55	cpf:CPF_2806	K07533	prsA	foldase protein PrsA	5.2.1.8	--

[denovogenes]_112809	100.00	0.0	pvl:AOB99_05330	K01834	PGAM, gpmA	2,3-bisphosphoglycerate-dependent phosphoglycerate mutase	5.4.2.11	Metabolism--Carbohydrate metabolism--Glycolysis / Gluconeogenesis|Metabolism--Amino acid metabolism--Glycine, serine and threonine metabolism|Metabolism--Energy metabolism--Methane metabolism|Metabolism--Global and overview maps--Metabolic pathways|Metabolism--Global and overview maps--Biosynthesis of secondary metabolites|Metabolism--Global and overview maps--Microbial metabolism in diverse environments|Metabolism--Global and overview maps--Biosynthesis of antibiotics|Metabolism--Global and overview maps--Carbon metabolism|Metabolism--Global and overview maps--Biosynthesis of amino acids|Organismal Systems--Endocrine system--Glucagon signaling pathway|Human Diseases--Cancers: Overview--Central carbon metabolism in cancer

[denovogenes]_11281	100.00	0.0	mmk:MU9_1428	K03702	uvrB	excinuclease ABC subunit B	--	Genetic Information Processing--Replication and repair--Nucleotide excision repair

[denovogenes]_112810	99.19	6e-172	cbut:ATN24_08540	K07720	yesN	two-component system, response regulator YesN	--	Environmental Information Processing--Signal transduction--Two-component system

[denovogenes]_112811	73.09	3e-141	cls:CXIVA_24490	K01142	E3.1.11.2, xthA	exodeoxyribonuclease III	3.1.11.2	Genetic Information Processing--Replication and repair--Base excision repair

[denovogenes]_112812	80.40	1e-155	ccb:Clocel_4116	K01142	E3.1.11.2, xthA	exodeoxyribonuclease III	3.1.11.2	Genetic Information Processing--Replication and repair--Base excision repair

[denovogenes]_112813	99.60	2e-174	cbut:ATN24_06255	K02654	pilD, pppA	leader peptidase (prepilin peptidase) / N-methyltransferase	3.4.23.43 2.1.1.-	--

[denovogenes]_112814	43.72	8e-63	ag:BAC80215	K19960	chnA	cyclohexanol dehydrogenase	1.1.1.245	Metabolism--Xenobiotics biodegradation and metabolism--Caprolactam degradation

[denovogenes]_112815	100.00	0.0	pvl:AOB99_12100	K18778	zapD	cell division protein ZapD	--	--

[denovogenes]_112817	99.60	0.0	elf:LF82_1690	K11076	potG	putrescine transport system ATP-binding protein	--	Environmental Information Processing--Membrane transport--ABC transporters

[denovogenes]_112818	45.63	9e-77	teu:TEU_09770	K15830	hycE	formate hydrogenlyase subunit 5	--	--

[denovogenes]_112819	46.18	2e-64	cle:Clole_3020	K17758	nnr	ADP-dependent NAD(P)H-hydrate dehydratase	4.2.1.136	--

[denovogenes]_112819	46.18	2e-64	cle:Clole_3020	K17759	AIBP, nnrE	NAD(P)H-hydrate epimerase	5.1.99.6	--

[denovogenes]_112820	98.38	0.0	cif:AL515_14115	K15256	cmoA	tRNA (cmo5U34)-methyltransferase	2.1.1.-	--

[denovogenes]_112821	97.20	1e-176	cbut:ATN24_09740	K15600	thiZ	putative hydroxymethylpyrimidine transport system ATP-binding protein	--	Environmental Information Processing--Membrane transport--ABC transporters

[denovogenes]_112822	99.60	0.0	cbut:ATN24_07595	K00806	uppS	undecaprenyl diphosphate synthase	2.5.1.31	Metabolism--Metabolism of terpenoids and polyketides--Terpenoid backbone biosynthesis|Metabolism--Global and overview maps--Biosynthesis of secondary metabolites

[denovogenes]_112823	97.60	0.0	boa:Bovatus_03433	K00215	dapB	4-hydroxy-tetrahydrodipicolinate reductase	1.17.1.8	Metabolism--Biosynthesis of other secondary metabolites--Monobactam biosynthesis|Metabolism--Amino acid metabolism--Lysine biosynthesis|Metabolism--Global and overview maps--Metabolic pathways|Metabolism--Global and overview maps--Biosynthesis of secondary metabolites|Metabolism--Global and overview maps--Microbial metabolism in diverse environments|Metabolism--Global and overview maps--Biosynthesis of antibiotics|Metabolism--Global and overview maps--Biosynthesis of amino acids

[denovogenes]_112824	55.02	2e-90	cbut:ATN24_06255	K02654	pilD, pppA	leader peptidase (prepilin peptidase) / N-methyltransferase	3.4.23.43 2.1.1.-	--

[denovogenes]_112826	80.48	2e-149	cdg:CDBI1_07005	K03086	SIG1, rpoD	RNA polymerase primary sigma factor	--	--

[denovogenes]_112828	83.53	2e-159	cck:Ccar_08265	K01142	E3.1.11.2, xthA	exodeoxyribonuclease III	3.1.11.2	Genetic Information Processing--Replication and repair--Base excision repair

[denovogenes]_112829	63.56	4e-107	cbv:U729_1649	K03436	fruR2, fruR	DeoR family transcriptional regulator, fructose operon transcriptional repressor	--	--

[denovogenes]_11283	100.00	0.0	bfs:BF9343_2253	K00700	GBE1, glgB	1,4-alpha-glucan branching enzyme	2.4.1.18	Metabolism--Carbohydrate metabolism--Starch and sucrose metabolism|Metabolism--Global and overview maps--Metabolic pathways|Metabolism--Global and overview maps--Biosynthesis of secondary metabolites

[denovogenes]_112835	100.00	0.0	pvl:AOB99_14735	K03222	yscJ, sctJ, hrcJ	type III secretion protein J	--	Environmental Information Processing--Membrane transport--Bacterial secretion system

[denovogenes]_112836	100.00	0.0	ecoh:ECRM13516_2003	K07639	rstB	two-component system, OmpR family, sensor histidine kinase RstB	2.7.13.3	Environmental Information Processing--Signal transduction--Two-component system

[denovogenes]_112837	43.72	7e-58	scc:Spico_1756	K07090	K07090	uncharacterized protein	--	--

[denovogenes]_112838	61.13	9e-106	cld:CLSPO_c17520	K03436	fruR2, fruR	DeoR family transcriptional regulator, fructose operon transcriptional repressor	--	--

[denovogenes]_112839	95.22	1e-166	cle:Clole_3548	K02358	tuf, TUFM	elongation factor Tu	--	--

[denovogenes]_112841	98.80	1e-180	ecoh:ECRM13516_2102	K12507	fadK	acyl-CoA synthetase	6.2.1.-	--

[denovogenes]_112842	99.20	0.0	sbc:SbBS512_E0677	K01785	galM, GALM	aldose 1-epimerase	5.1.3.3	Metabolism--Carbohydrate metabolism--Glycolysis / Gluconeogenesis|Metabolism--Carbohydrate metabolism--Galactose metabolism|Metabolism--Global and overview maps--Metabolic pathways|Metabolism--Global and overview maps--Biosynthesis of secondary metabolites|Metabolism--Global and overview maps--Microbial metabolism in diverse environments|Metabolism--Global and overview maps--Biosynthesis of antibiotics

[denovogenes]_112843	67.83	6e-126	pft:JBW_03555	K02030	ABC.PA.S	polar amino acid transport system substrate-binding protein	--	--

[denovogenes]_112844	49.59	7e-74	cpf:CPF_2570	K02652	pilB	type IV pilus assembly protein PilB	--	--

[denovogenes]_112849	60.80	3e-98	cpf:CPF_0853	K03299	TC.GNTP	gluconate:H+ symporter, GntP family	--	--

[denovogenes]_112850	86.40	1e-163	prg:RB151_039300	K00880	lyxK	L-xylulokinase	2.7.1.53	Metabolism--Carbohydrate metabolism--Pentose and glucuronate interconversions|Metabolism--Carbohydrate metabolism--Ascorbate and aldarate metabolism

[denovogenes]_112851	100.00	4e-175	pvl:AOB99_09500	K08159	sotB	MFS transporter, DHA1 family, L-arabinose/isopropyl-beta-D-thiogalactopyranoside export protein	--	--

[denovogenes]_112852	72.47	4e-135	cpf:CPF_1251	K04070	pflX	putative pyruvate formate lyase activating enzyme	1.97.1.4	--

[denovogenes]_112853	99.20	0.0	boa:Bovatus_04007	K00566	mnmA, trmU	tRNA-uridine 2-sulfurtransferase	2.8.1.13	Genetic Information Processing--Folding, sorting and degradation--Sulfur relay system

[denovogenes]_112858	40.42	1e-43	got:AXE85_06235	K01261	pepA	glutamyl aminopeptidase	3.4.11.7	--

[denovogenes]_11286	41.27	4e-67	tte:TTE0012	K00354	E1.6.99.1	NADPH2 dehydrogenase	1.6.99.1	--

[denovogenes]_112860	86.45	1e-152	cpr:CPR_2145	K00789	metK	S-adenosylmethionine synthetase	2.5.1.6	Metabolism--Amino acid metabolism--Cysteine and methionine metabolism|Metabolism--Global and overview maps--Metabolic pathways|Metabolism--Global and overview maps--Biosynthesis of secondary metabolites|Metabolism--Global and overview maps--Biosynthesis of amino acids

[denovogenes]_112862	42.86	1e-61	vpr:Vpar_1658	K07098	K07098	uncharacterized protein	--	--

[denovogenes]_112863	100.00	0.0	sfv:SFV_3974	K10543	xylF	D-xylose transport system substrate-binding protein	--	Environmental Information Processing--Membrane transport--ABC transporters

[denovogenes]_112870	99.60	0.0	eun:UMNK88_432	K01077	E3.1.3.1, phoA, phoB	alkaline phosphatase	3.1.3.1	Metabolism--Xenobiotics biodegradation and metabolism--Aminobenzoate degradation|Metabolism--Metabolism of cofactors and vitamins--Thiamine metabolism|Metabolism--Metabolism of cofactors and vitamins--Folate biosynthesis|Metabolism--Global and overview maps--Metabolic pathways|Metabolism--Global and overview maps--Microbial metabolism in diverse environments|Environmental Information Processing--Signal transduction--Two-component system

[denovogenes]_112873	56.85	2e-90	cbv:U729_932	K01992	ABC-2.P	ABC-2 type transport system permease protein	--	--

[denovogenes]_112875	46.09	4e-25	cbt:CLH_1555	K14475	ICP	inhibitor of cysteine peptidase	--	Human Diseases--Infectious diseases: Parasitic--African trypanosomiasis

[denovogenes]_112877	49.20	2e-71	clt:CM240_1352	K03657	uvrD, pcrA	DNA helicase II / ATP-dependent DNA helicase PcrA	3.6.4.12	Genetic Information Processing--Replication and repair--Nucleotide excision repair|Genetic Information Processing--Replication and repair--Mismatch repair

[denovogenes]_112878	77.47	4e-131	csr:Cspa_c49580	K02755	PTS-Bgl-EIIA, bglF, bglP	PTS system, beta-glucoside-specific IIA component	2.7.1.-	Environmental Information Processing--Membrane transport--Phosphotransferase system (PTS)

[denovogenes]_112878	77.47	4e-131	csr:Cspa_c49580	K02756	PTS-Bgl-EIIB, bglF, bglP	PTS system, beta-glucoside-specific IIB component	2.7.1.-	Environmental Information Processing--Membrane transport--Phosphotransferase system (PTS)

[denovogenes]_112878	77.47	4e-131	csr:Cspa_c49580	K02757	PTS-Bgl-EIIC, bglF, bglP	PTS system, beta-glucoside-specific IIC component	--	Environmental Information Processing--Membrane transport--Phosphotransferase system (PTS)

[denovogenes]_112879	42.00	2e-58	eun:UMNK88_4342	K07497	K07497	putative transposase	--	--

[denovogenes]_112881	91.39	7e-172	bhl:Bache_0322	K00806	uppS	undecaprenyl diphosphate synthase	2.5.1.31	Metabolism--Metabolism of terpenoids and polyketides--Terpenoid backbone biosynthesis|Metabolism--Global and overview maps--Biosynthesis of secondary metabolites

[denovogenes]_112882	93.85	5e-173	bhl:Bache_2916	K00721	DPM1	dolichol-phosphate mannosyltransferase	2.4.1.83	Metabolism--Glycan biosynthesis and metabolism--N-Glycan biosynthesis|Metabolism--Global and overview maps--Metabolic pathways

[denovogenes]_112883	81.20	3e-154	bprl:CL2_11940	K01142	E3.1.11.2, xthA	exodeoxyribonuclease III	3.1.11.2	Genetic Information Processing--Replication and repair--Base excision repair

[denovogenes]_112884	53.01	9e-94	rho:RHOM_14155	K06889	K06889	uncharacterized protein	--	--

[denovogenes]_112887	86.99	1e-158	btho:Btheta7330_03680	K06201	cutC	copper homeostasis protein	--	--

[denovogenes]_112888	81.93	4e-154	rum:CK1_24810	K02036	pstB	phosphate transport system ATP-binding protein	3.6.3.27	Environmental Information Processing--Membrane transport--ABC transporters

[denovogenes]_11289	45.83	0.0	ova:OBV_27590	K03406	mcp	methyl-accepting chemotaxis protein	--	Environmental Information Processing--Signal transduction--Two-component system|Cellular Processes--Cell motility--Bacterial chemotaxis

[denovogenes]_112890	82.26	2e-152	rto:RTO_18110	K03218	rlmB	23S rRNA (guanosine2251-2'-O)-methyltransferase	2.1.1.185	--

[denovogenes]_112894	94.78	2e-174	bxy:BXY_35390	K06201	cutC	copper homeostasis protein	--	--

[denovogenes]_112897	45.15	4e-68	csh:Closa_3446	K02392	flgG	flagellar basal-body rod protein FlgG	--	Cellular Processes--Cell motility--Flagellar assembly

[denovogenes]_112898	99.60	0.0	btho:Btheta7330_04893	K09013	sufC	Fe-S cluster assembly ATP-binding protein	--	--

[denovogenes]_112899	50.68	2e-70	tae:TepiRe1_1785	K09812	ftsE	cell division transport system ATP-binding protein	--	Environmental Information Processing--Membrane transport--ABC transporters

[denovogenes]_112900	67.49	7e-122	cso:CLS_38530	K12410	npdA	NAD-dependent deacetylase	3.5.1.-	--

[denovogenes]_112901	40.98	4e-23	rim:ROI_08810	K07720	yesN	two-component system, response regulator YesN	--	Environmental Information Processing--Signal transduction--Two-component system

[denovogenes]_112903	41.80	2e-57	cid:P73_2157	K06989	nadX	aspartate dehydrogenase	1.4.1.21	Metabolism--Metabolism of cofactors and vitamins--Nicotinate and nicotinamide metabolism|Metabolism--Global and overview maps--Metabolic pathways

[denovogenes]_112904	100.00	0.0	bxy:BXY_03260	K00979	kdsB	3-deoxy-manno-octulosonate cytidylyltransferase (CMP-KDO synthetase)	2.7.7.38	Metabolism--Glycan biosynthesis and metabolism--Lipopolysaccharide biosynthesis|Metabolism--Global and overview maps--Metabolic pathways

[denovogenes]_112905	96.79	3e-180	bxy:BXY_03260	K00979	kdsB	3-deoxy-manno-octulosonate cytidylyltransferase (CMP-KDO synthetase)	2.7.7.38	Metabolism--Glycan biosynthesis and metabolism--Lipopolysaccharide biosynthesis|Metabolism--Global and overview maps--Metabolic pathways

[denovogenes]_112906	78.00	7e-149	fpla:A4U99_09720	K02013	ABC.FEV.A	iron complex transport system ATP-binding protein	3.6.3.34	Environmental Information Processing--Membrane transport--ABC transporters

[denovogenes]_112908	71.37	3e-130	rho:RHOM_04530	K02028	ABC.PA.A	polar amino acid transport system ATP-binding protein	3.6.3.21	--

[denovogenes]_112909	48.72	1e-70	byl:A4V09_12980	K06987	K06987	uncharacterized protein	--	--

[denovogenes]_112910	49.11	1e-71	tki:TKV_c18060	K09812	ftsE	cell division transport system ATP-binding protein	--	Environmental Information Processing--Membrane transport--ABC transporters

[denovogenes]_112911	56.28	4e-93	rim:ROI_34300	K03436	fruR2, fruR	DeoR family transcriptional regulator, fructose operon transcriptional repressor	--	--

[denovogenes]_112912	85.60	1e-160	fpla:A4U99_09720	K02013	ABC.FEV.A	iron complex transport system ATP-binding protein	3.6.3.34	Environmental Information Processing--Membrane transport--ABC transporters

[denovogenes]_112913	88.00	4e-160	rim:ROI_21270	K02967	RP-S2, MRPS2, rpsB	small subunit ribosomal protein S2	--	Genetic Information Processing--Translation--Ribosome

[denovogenes]_112914	93.52	1e-173	bhl:Bache_1909	K02036	pstB	phosphate transport system ATP-binding protein	3.6.3.27	Environmental Information Processing--Membrane transport--ABC transporters

[denovogenes]_112916	61.60	3e-113	psn:Pedsa_1191	K00059	fabG	3-oxoacyl-[acyl-carrier protein] reductase	1.1.1.100	Metabolism--Lipid metabolism--Fatty acid biosynthesis|Metabolism--Metabolism of cofactors and vitamins--Biotin metabolism|Metabolism--Lipid metabolism--Biosynthesis of unsaturated fatty acids|Metabolism--Global and overview maps--Metabolic pathways|Metabolism--Global and overview maps--Fatty acid metabolism

[denovogenes]_112918	81.60	4e-156	bprl:CL2_11940	K01142	E3.1.11.2, xthA	exodeoxyribonuclease III	3.1.11.2	Genetic Information Processing--Replication and repair--Base excision repair

[denovogenes]_112919	85.20	4e-158	fpla:A4U99_08000	K02564	nagB, GNPDA	glucosamine-6-phosphate deaminase	3.5.99.6	Metabolism--Carbohydrate metabolism--Amino sugar and nucleotide sugar metabolism|Metabolism--Global and overview maps--Metabolic pathways

[denovogenes]_112920	70.49	2e-132	bcel:BcellWH2_03585	K03800	lplA, lplJ	lipoate---protein ligase	6.3.1.20	Metabolism--Metabolism of cofactors and vitamins--Lipoic acid metabolism|Metabolism--Global and overview maps--Metabolic pathways

[denovogenes]_112921	56.68	2e-99	ere:EUBREC_2180	K02654	pilD, pppA	leader peptidase (prepilin peptidase) / N-methyltransferase	3.4.23.43 2.1.1.-	--

[denovogenes]_112922	99.60	0.0	bxy:BXY_27330	K09013	sufC	Fe-S cluster assembly ATP-binding protein	--	--

[denovogenes]_112923	99.60	0.0	bvu:BVU_4037	K00979	kdsB	3-deoxy-manno-octulosonate cytidylyltransferase (CMP-KDO synthetase)	2.7.7.38	Metabolism--Glycan biosynthesis and metabolism--Lipopolysaccharide biosynthesis|Metabolism--Global and overview maps--Metabolic pathways

[denovogenes]_112925	81.60	5e-156	pru:PRU_0445	K01627	kdsA	2-dehydro-3-deoxyphosphooctonate aldolase (KDO 8-P synthase)	2.5.1.55	Metabolism--Glycan biosynthesis and metabolism--Lipopolysaccharide biosynthesis|Metabolism--Global and overview maps--Metabolic pathways

[denovogenes]_112926	61.20	3e-114	rho:RHOM_02195	K04069	pflA, pflC, pflE	pyruvate formate lyase activating enzyme	1.97.1.4	--

[denovogenes]_11293	98.36	0.0	boa:Bovatus_00854	K00700	GBE1, glgB	1,4-alpha-glucan branching enzyme	2.4.1.18	Metabolism--Carbohydrate metabolism--Starch and sucrose metabolism|Metabolism--Global and overview maps--Metabolic pathways|Metabolism--Global and overview maps--Biosynthesis of secondary metabolites

[denovogenes]_112930	60.80	2e-110	esr:ES1_20700	K00215	dapB	4-hydroxy-tetrahydrodipicolinate reductase	1.17.1.8	Metabolism--Biosynthesis of other secondary metabolites--Monobactam biosynthesis|Metabolism--Amino acid metabolism--Lysine biosynthesis|Metabolism--Global and overview maps--Metabolic pathways|Metabolism--Global and overview maps--Biosynthesis of secondary metabolites|Metabolism--Global and overview maps--Microbial metabolism in diverse environments|Metabolism--Global and overview maps--Biosynthesis of antibiotics|Metabolism--Global and overview maps--Biosynthesis of amino acids

[denovogenes]_112933	53.30	4e-66	clo:HMPREF0868_0923	K13012	wbqP	O-antigen biosynthesis protein WbqP	--	--

[denovogenes]_112938	90.40	2e-171	bfg:BF638R_0805	K16698	tuaG	teichuronic acid biosynthesis glycosyltransferase TuaG	2.4.-.-	--

[denovogenes]_112939	84.40	7e-159	fpla:A4U99_09720	K02013	ABC.FEV.A	iron complex transport system ATP-binding protein	3.6.3.34	Environmental Information Processing--Membrane transport--ABC transporters

[denovogenes]_11294	94.78	0.0	boa:Bovatus_00854	K00700	GBE1, glgB	1,4-alpha-glucan branching enzyme	2.4.1.18	Metabolism--Carbohydrate metabolism--Starch and sucrose metabolism|Metabolism--Global and overview maps--Metabolic pathways|Metabolism--Global and overview maps--Biosynthesis of secondary metabolites

[denovogenes]_112940	48.78	4e-79	bha:BH3661	K16698	tuaG	teichuronic acid biosynthesis glycosyltransferase TuaG	2.4.-.-	--

[denovogenes]_112942	100.00	0.0	bvu:BVU_3937	K02036	pstB	phosphate transport system ATP-binding protein	3.6.3.27	Environmental Information Processing--Membrane transport--ABC transporters

[denovogenes]_112943	52.17	3e-50	tsa:AciPR4_3094	K00558	DNMT1, dcm	DNA (cytosine-5)-methyltransferase 1	2.1.1.37	Metabolism--Amino acid metabolism--Cysteine and methionine metabolism|Metabolism--Global and overview maps--Metabolic pathways|Human Diseases--Cancers: Overview--MicroRNAs in cancer

[denovogenes]_112947	41.27	6e-22	bprm:CL3_14780	K07720	yesN	two-component system, response regulator YesN	--	Environmental Information Processing--Signal transduction--Two-component system

[denovogenes]_112948	62.80	7e-120	bprs:CK3_18400	K03785	aroD	3-dehydroquinate dehydratase I	4.2.1.10	Metabolism--Amino acid metabolism--Phenylalanine, tyrosine and tryptophan biosynthesis|Metabolism--Global and overview maps--Metabolic pathways|Metabolism--Global and overview maps--Biosynthesis of secondary metabolites|Metabolism--Global and overview maps--Biosynthesis of antibiotics|Metabolism--Global and overview maps--Biosynthesis of amino acids

[denovogenes]_112949	69.88	7e-123	ibu:IB211_00435c	K01297	ldcA	muramoyltetrapeptide carboxypeptidase	3.4.17.13	--

[denovogenes]_11295	99.70	0.0	bvu:BVU_2318	K00615	E2.2.1.1, tktA, tktB	transketolase	2.2.1.1	Metabolism--Carbohydrate metabolism--Pentose phosphate pathway|Metabolism--Energy metabolism--Carbon fixation in photosynthetic organisms|Metabolism--Metabolism of terpenoids and polyketides--Biosynthesis of ansamycins|Metabolism--Global and overview maps--Metabolic pathways|Metabolism--Global and overview maps--Biosynthesis of secondary metabolites|Metabolism--Global and overview maps--Microbial metabolism in diverse environments|Metabolism--Global and overview maps--Biosynthesis of antibiotics|Metabolism--Global and overview maps--Carbon metabolism|Metabolism--Global and overview maps--Biosynthesis of amino acids

[denovogenes]_112950	98.40	3e-180	lacy:A4V08_14905	K00975	glgC	glucose-1-phosphate adenylyltransferase	2.7.7.27	Metabolism--Carbohydrate metabolism--Starch and sucrose metabolism|Metabolism--Carbohydrate metabolism--Amino sugar and nucleotide sugar metabolism|Metabolism--Global and overview maps--Metabolic pathways|Metabolism--Global and overview maps--Biosynthesis of secondary metabolites|Cellular Processes--Cellular community - prokaryotes--Biofilm formation - Escherichia coli

[denovogenes]_112951	62.35	9e-119	bprs:CK3_18400	K03785	aroD	3-dehydroquinate dehydratase I	4.2.1.10	Metabolism--Amino acid metabolism--Phenylalanine, tyrosine and tryptophan biosynthesis|Metabolism--Global and overview maps--Metabolic pathways|Metabolism--Global and overview maps--Biosynthesis of secondary metabolites|Metabolism--Global and overview maps--Biosynthesis of antibiotics|Metabolism--Global and overview maps--Biosynthesis of amino acids

[denovogenes]_112952	99.60	0.0	btho:Btheta7330_02365	K00979	kdsB	3-deoxy-manno-octulosonate cytidylyltransferase (CMP-KDO synthetase)	2.7.7.38	Metabolism--Glycan biosynthesis and metabolism--Lipopolysaccharide biosynthesis|Metabolism--Global and overview maps--Metabolic pathways

[denovogenes]_112953	100.00	0.0	bvu:BVU_1358	K09013	sufC	Fe-S cluster assembly ATP-binding protein	--	--

[denovogenes]_112955	97.20	0.0	fpla:A4U99_18005	K01142	E3.1.11.2, xthA	exodeoxyribonuclease III	3.1.11.2	Genetic Information Processing--Replication and repair--Base excision repair

[denovogenes]_112958	54.88	6e-78	pary:A4V02_08670	K10716	kch, trkA, mthK, pch	voltage-gated potassium channel	--	--

[denovogenes]_112959	99.60	0.0	bxy:BXY_47740	K01921	ddl	D-alanine-D-alanine ligase	6.3.2.4	Metabolism--Metabolism of other amino acids--D-Alanine metabolism|Metabolism--Glycan biosynthesis and metabolism--Peptidoglycan biosynthesis|Metabolism--Global and overview maps--Metabolic pathways|Human Diseases--Drug resistance: Antimicrobial--Vancomycin resistance

[denovogenes]_11296	80.21	0.0	bdo:EL88_18420	K03427	hsdM	type I restriction enzyme M protein	2.1.1.72	--

[denovogenes]_112960	42.67	5e-49	tte:TTE2689	K07668	vicR	two-component system, OmpR family, response regulator VicR	--	Environmental Information Processing--Signal transduction--Two-component system

[denovogenes]_112961	71.43	2e-118	cpy:Cphy_2584	K05896	scpA	segregation and condensation protein A	--	--

[denovogenes]_112964	100.00	0.0	shq:A0259_07045	K02566	nagD	NagD protein	--	--

[denovogenes]_112966	46.40	8e-75	eyy:EGYY_23750	K00059	fabG	3-oxoacyl-[acyl-carrier protein] reductase	1.1.1.100	Metabolism--Lipid metabolism--Fatty acid biosynthesis|Metabolism--Metabolism of cofactors and vitamins--Biotin metabolism|Metabolism--Lipid metabolism--Biosynthesis of unsaturated fatty acids|Metabolism--Global and overview maps--Metabolic pathways|Metabolism--Global and overview maps--Fatty acid metabolism

[denovogenes]_112968	96.80	2e-176	bvu:BVU_1358	K09013	sufC	Fe-S cluster assembly ATP-binding protein	--	--

[denovogenes]_112969	41.56	4e-57	ccel:CCDG5_1289	K20074	prpC, phpP	PPM family protein phosphatase	3.1.3.16	--

[denovogenes]_112970	88.35	1e-154	bprm:CL3_14360	K01265	map	methionyl aminopeptidase	3.4.11.18	--

[denovogenes]_112972	100.00	0.0	ecos:EC958_0867	K01834	PGAM, gpmA	2,3-bisphosphoglycerate-dependent phosphoglycerate mutase	5.4.2.11	Metabolism--Carbohydrate metabolism--Glycolysis / Gluconeogenesis|Metabolism--Amino acid metabolism--Glycine, serine and threonine metabolism|Metabolism--Energy metabolism--Methane metabolism|Metabolism--Global and overview maps--Metabolic pathways|Metabolism--Global and overview maps--Biosynthesis of secondary metabolites|Metabolism--Global and overview maps--Microbial metabolism in diverse environments|Metabolism--Global and overview maps--Biosynthesis of antibiotics|Metabolism--Global and overview maps--Carbon metabolism|Metabolism--Global and overview maps--Biosynthesis of amino acids|Organismal Systems--Endocrine system--Glucagon signaling pathway|Human Diseases--Cancers: Overview--Central carbon metabolism in cancer

[denovogenes]_112975	57.20	9e-106	cct:CC1_18920	K02026	ABC.MS.P1	multiple sugar transport system permease protein	--	--

[denovogenes]_112976	97.60	0.0	cfd:CFNIH1_21280	K19697	pduW	propionate kinase	2.7.2.15	Metabolism--Carbohydrate metabolism--Propanoate metabolism|Metabolism--Global and overview maps--Metabolic pathways

[denovogenes]_112979	98.00	0.0	boa:Bovatus_01412	K02500	hisF	cyclase	4.1.3.-	Metabolism--Amino acid metabolism--Histidine metabolism|Metabolism--Global and overview maps--Metabolic pathways|Metabolism--Global and overview maps--Biosynthesis of secondary metabolites|Metabolism--Global and overview maps--Biosynthesis of amino acids

[denovogenes]_11298	44.17	7e-175	elm:ELI_3481	K02004	ABC.CD.P	putative ABC transport system permease protein	--	--

[denovogenes]_112981	96.40	4e-179	boa:Bovatus_01412	K02500	hisF	cyclase	4.1.3.-	Metabolism--Amino acid metabolism--Histidine metabolism|Metabolism--Global and overview maps--Metabolic pathways|Metabolism--Global and overview maps--Biosynthesis of secondary metabolites|Metabolism--Global and overview maps--Biosynthesis of amino acids

[denovogenes]_112983	96.40	0.0	bdh:GV66_20970	K02036	pstB	phosphate transport system ATP-binding protein	3.6.3.27	Environmental Information Processing--Membrane transport--ABC transporters

[denovogenes]_112984	99.60	0.0	cif:AL515_08385	K01834	PGAM, gpmA	2,3-bisphosphoglycerate-dependent phosphoglycerate mutase	5.4.2.11	Metabolism--Carbohydrate metabolism--Glycolysis / Gluconeogenesis|Metabolism--Amino acid metabolism--Glycine, serine and threonine metabolism|Metabolism--Energy metabolism--Methane metabolism|Metabolism--Global and overview maps--Metabolic pathways|Metabolism--Global and overview maps--Biosynthesis of secondary metabolites|Metabolism--Global and overview maps--Microbial metabolism in diverse environments|Metabolism--Global and overview maps--Biosynthesis of antibiotics|Metabolism--Global and overview maps--Carbon metabolism|Metabolism--Global and overview maps--Biosynthesis of amino acids|Organismal Systems--Endocrine system--Glucagon signaling pathway|Human Diseases--Cancers: Overview--Central carbon metabolism in cancer

[denovogenes]_112985	55.98	9e-93	fpp:FPB0191_01813	K02029	ABC.PA.P	polar amino acid transport system permease protein	--	--

[denovogenes]_112986	86.85	2e-156	lacy:A4V08_25575	K06147	ABCB-BAC	ATP-binding cassette, subfamily B, bacterial	--	--

[denovogenes]_112987	99.60	4e-180	ecm:EcSMS35_1265	K10010	ABC.CYST.A	cystine transport system ATP-binding protein	3.6.3.-	Environmental Information Processing--Membrane transport--ABC transporters

[denovogenes]_112988	65.85	9e-110	fpla:A4U99_00115	K03497	parB, spo0J	chromosome partitioning protein, ParB family	--	--

[denovogenes]_112989	92.00	5e-172	lacy:A4V08_00195	K01092	E3.1.3.25, IMPA, suhB	myo-inositol-1(or 4)-monophosphatase	3.1.3.25	Metabolism--Biosynthesis of other secondary metabolites--Streptomycin biosynthesis|Metabolism--Carbohydrate metabolism--Inositol phosphate metabolism|Metabolism--Global and overview maps--Metabolic pathways|Environmental Information Processing--Signal transduction--Phosphatidylinositol signaling system

[denovogenes]_11299	99.70	0.0	eoi:ECO111_3215	K12146	hyfR	hydrogenase-4 transcriptional activator	--	--

[denovogenes]_112992	62.40	1e-109	fpa:FPR_11080	K16787	ecfA2	energy-coupling factor transport system ATP-binding protein	3.6.3.-	Environmental Information Processing--Membrane transport--ABC transporters

[denovogenes]_112993	54.97	7e-66	bcf:bcf_00645	K03046	rpoC	DNA-directed RNA polymerase subunit beta'	2.7.7.6	Metabolism--Nucleotide metabolism--Purine metabolism|Metabolism--Nucleotide metabolism--Pyrimidine metabolism|Metabolism--Global and overview maps--Metabolic pathways|Genetic Information Processing--Transcription--RNA polymerase

[denovogenes]_112996	98.11	2e-112	lacy:A4V08_30995	K05303	K05303	O-methyltransferase	2.1.1.-	--

[denovogenes]_112998	91.80	6e-168	bhl:Bache_2916	K00721	DPM1	dolichol-phosphate mannosyltransferase	2.4.1.83	Metabolism--Glycan biosynthesis and metabolism--N-Glycan biosynthesis|Metabolism--Global and overview maps--Metabolic pathways

[denovogenes]_113	99.24	0.0	mmk:MU9_3298	K06894	K06894	uncharacterized protein	--	--

[denovogenes]_113000	95.98	1e-171	lacy:A4V08_27145	K00266	gltD	glutamate synthase (NADPH/NADH) small chain	1.4.1.13 1.4.1.14	Metabolism--Amino acid metabolism--Alanine, aspartate and glutamate metabolism|Metabolism--Energy metabolism--Nitrogen metabolism|Metabolism--Global and overview maps--Metabolic pathways|Metabolism--Global and overview maps--Biosynthesis of secondary metabolites|Metabolism--Global and overview maps--Microbial metabolism in diverse environments|Metabolism--Global and overview maps--Biosynthesis of antibiotics|Metabolism--Global and overview maps--Biosynthesis of amino acids

[denovogenes]_113001	93.20	3e-170	bcel:BcellWH2_03275	K03324	yjbB	phosphate:Na+ symporter	--	--

[denovogenes]_113002	94.80	7e-176	fpla:A4U99_03305	K02392	flgG	flagellar basal-body rod protein FlgG	--	Cellular Processes--Cell motility--Flagellar assembly

[denovogenes]_113006	97.61	0.0	cfd:CFNIH1_01145	K02529	lacI, galR	LacI family transcriptional regulator	--	--

[denovogenes]_113007	52.80	1e-83	lmi:LMXM_16_0440	K05305	FUK	fucokinase	2.7.1.52	Metabolism--Carbohydrate metabolism--Fructose and mannose metabolism|Metabolism--Carbohydrate metabolism--Amino sugar and nucleotide sugar metabolism|Metabolism--Global and overview maps--Metabolic pathways

[denovogenes]_11301	41.47	3e-129	afg:AFULGI_00004610	K00219	fadH	2,4-dienoyl-CoA reductase (NADPH2)	1.3.1.34	--

[denovogenes]_113010	93.57	9e-175	lacy:A4V08_21420	K09691	ABC-2.LPSE.A	lipopolysaccharide transport system ATP-binding protein	--	Environmental Information Processing--Membrane transport--ABC transporters

[denovogenes]_113012	56.47	2e-99	csr:Cspa_c41850	K01424	E3.5.1.1, ansA, ansB	L-asparaginase	3.5.1.1	Metabolism--Amino acid metabolism--Alanine, aspartate and glutamate metabolism|Metabolism--Metabolism of other amino acids--Cyanoamino acid metabolism|Metabolism--Global and overview maps--Metabolic pathways|Metabolism--Global and overview maps--Biosynthesis of secondary metabolites

[denovogenes]_113015	79.91	1e-126	bhl:Bache_1411	K06872	K06872	uncharacterized protein	--	--

[denovogenes]_113016	78.78	2e-143	cle:Clole_3243	K02041	phnC	phosphonate transport system ATP-binding protein	3.6.3.28	Environmental Information Processing--Membrane transport--ABC transporters

[denovogenes]_113017	79.03	3e-148	dhd:Dhaf_0457	K02032	ABC.PE.A1	peptide/nickel transport system ATP-binding protein	--	Cellular Processes--Cellular community - prokaryotes--Quorum sensing

[denovogenes]_113018	98.40	3e-174	lacy:A4V08_31165	K02421	fliR	flagellar biosynthetic protein FliR	--	Cellular Processes--Cell motility--Flagellar assembly

[denovogenes]_11302	99.70	0.0	bvu:BVU_2015	K01277	DPP3	dipeptidyl-peptidase III	3.4.14.4	--

[denovogenes]_113020	84.55	3e-152	bdh:GV66_03925	K03797	E3.4.21.102, prc, ctpA	carboxyl-terminal processing protease	3.4.21.102	--

[denovogenes]_113021	54.70	2e-81	bon:A361_24415	K02809	PTS-Scr-EIIB, scrA, sacP, sacX, ptsS	PTS system, sucrose-specific IIB component	2.7.1.211	Metabolism--Carbohydrate metabolism--Starch and sucrose metabolism|Environmental Information Processing--Membrane transport--Phosphotransferase system (PTS)

[denovogenes]_113021	54.70	2e-81	bon:A361_24415	K02810	PTS-Scr-EIIC, scrA, sacP, sacX, ptsS	PTS system, sucrose-specific IIC component	--	Metabolism--Carbohydrate metabolism--Starch and sucrose metabolism|Environmental Information Processing--Membrane transport--Phosphotransferase system (PTS)

[denovogenes]_113022	77.51	4e-140	bprs:CK3_28690	K00604	MTFMT, fmt	methionyl-tRNA formyltransferase	2.1.2.9	Metabolism--Metabolism of cofactors and vitamins--One carbon pool by folate|Genetic Information Processing--Translation--Aminoacyl-tRNA biosynthesis

[denovogenes]_113023	43.44	1e-58	dew:DGWBC_1561	K04759	feoB	ferrous iron transport protein B	--	--

[denovogenes]_113025	99.20	2e-176	cif:AL515_10340	K11071	potB	spermidine/putrescine transport system permease protein	--	Environmental Information Processing--Membrane transport--ABC transporters

[denovogenes]_113027	80.08	1e-144	sbr:SY1_04290	K10541	mglC	methyl-galactoside transport system permease protein	--	Environmental Information Processing--Membrane transport--ABC transporters

[denovogenes]_113028	57.87	2e-99	eac:EAL2_c19180	K01464	DPYS, dht, hydA	dihydropyrimidinase	3.5.2.2	Metabolism--Nucleotide metabolism--Pyrimidine metabolism|Metabolism--Metabolism of other amino acids--beta-Alanine metabolism|Metabolism--Metabolism of cofactors and vitamins--Pantothenate and CoA biosynthesis|Metabolism--Xenobiotics biodegradation and metabolism--Drug metabolism - other enzymes|Metabolism--Global and overview maps--Metabolic pathways

[denovogenes]_113029	61.35	1e-101	tjr:TherJR_0351	K00821	argD	acetylornithine/N-succinyldiaminopimelate aminotransferase	2.6.1.11 2.6.1.17	Metabolism--Amino acid metabolism--Arginine biosynthesis|Metabolism--Amino acid metabolism--Lysine biosynthesis|Metabolism--Global and overview maps--Metabolic pathways|Metabolism--Global and overview maps--Biosynthesis of secondary metabolites|Metabolism--Global and overview maps--Microbial metabolism in diverse environments|Metabolism--Global and overview maps--Biosynthesis of antibiotics|Metabolism--Global and overview maps--2-Oxocarboxylic acid metabolism|Metabolism--Global and overview maps--Biosynthesis of amino acids

[denovogenes]_11303	64.28	0.0	awo:Awo_c31510	K10797	enr	2-enoate reductase	1.3.1.31	Metabolism--Amino acid metabolism--Phenylalanine metabolism|Metabolism--Global and overview maps--Microbial metabolism in diverse environments

[denovogenes]_113030	92.43	3e-168	cif:AL515_19290	K02669	pilT	twitching motility protein PilT	--	--

[denovogenes]_113033	91.84	2e-167	lacy:A4V08_00810	K01647	CS, gltA	citrate synthase	2.3.3.1	Metabolism--Carbohydrate metabolism--Citrate cycle (TCA cycle)|Metabolism--Carbohydrate metabolism--Glyoxylate and dicarboxylate metabolism|Metabolism--Global and overview maps--Metabolic pathways|Metabolism--Global and overview maps--Biosynthesis of secondary metabolites|Metabolism--Global and overview maps--Microbial metabolism in diverse environments|Metabolism--Global and overview maps--Biosynthesis of antibiotics|Metabolism--Global and overview maps--Carbon metabolism|Metabolism--Global and overview maps--2-Oxocarboxylic acid metabolism|Metabolism--Global and overview maps--Biosynthesis of amino acids

[denovogenes]_113034	76.10	2e-137	bprs:CK3_35160	K02035	ABC.PE.S	peptide/nickel transport system substrate-binding protein	--	Cellular Processes--Cellular community - prokaryotes--Quorum sensing

[denovogenes]_113035	55.78	2e-95	cle:Clole_4239	K09759	aspC	nondiscriminating aspartyl-tRNA synthetase	6.1.1.23	Genetic Information Processing--Translation--Aminoacyl-tRNA biosynthesis

[denovogenes]_113036	82.87	9e-154	bhl:Bache_2637	K00018	hprA	glycerate dehydrogenase	1.1.1.29	Metabolism--Amino acid metabolism--Glycine, serine and threonine metabolism|Metabolism--Carbohydrate metabolism--Glyoxylate and dicarboxylate metabolism|Metabolism--Energy metabolism--Methane metabolism|Metabolism--Global and overview maps--Metabolic pathways|Metabolism--Global and overview maps--Biosynthesis of secondary metabolites|Metabolism--Global and overview maps--Microbial metabolism in diverse environments|Metabolism--Global and overview maps--Biosynthesis of antibiotics|Metabolism--Global and overview maps--Carbon metabolism

[denovogenes]_113038	75.30	2e-120	pna:Pnap_4003	K19577	ydhP	MFS transporter, DHA1 family, inner membrane transport protein	--	--

[denovogenes]_113041	85.66	3e-157	bprs:CK3_08430	K15986	ppaC	manganese-dependent inorganic pyrophosphatase	3.6.1.1	Metabolism--Energy metabolism--Oxidative phosphorylation

[denovogenes]_113042	95.18	3e-174	pfr:PFREUD_22460	K20905	lerI	L-erythrulose 4-phosphate epimerase	5.1.3.39	--

[denovogenes]_113043	40.32	4e-35	strp:F750_6871	K18568	thnR	CoA pyrophosphatase	3.6.1.-	Metabolism--Biosynthesis of other secondary metabolites--Carbapenem biosynthesis|Metabolism--Global and overview maps--Biosynthesis of antibiotics

[denovogenes]_113044	48.33	6e-65	sma:SAV_7146	K03710	K03710	GntR family transcriptional regulator	--	--

[denovogenes]_113045	92.34	5e-167	pfre:RM25_1428	K01057	PGLS, pgl, devB	6-phosphogluconolactonase	3.1.1.31	Metabolism--Carbohydrate metabolism--Pentose phosphate pathway|Metabolism--Global and overview maps--Metabolic pathways|Metabolism--Global and overview maps--Biosynthesis of secondary metabolites|Metabolism--Global and overview maps--Microbial metabolism in diverse environments|Metabolism--Global and overview maps--Biosynthesis of antibiotics|Metabolism--Global and overview maps--Carbon metabolism

[denovogenes]_113046	93.60	1e-179	cce:Ccel_2852	K07741	antB	anti-repressor protein	--	--

[denovogenes]_113048	42.54	1e-20	scw:TU94_21285	K15525	mshB	N-acetyl-1-D-myo-inositol-2-amino-2-deoxy-alpha-D-glucopyranoside deacetylase	3.5.1.103	--

[denovogenes]_11305	47.06	8e-179	eha:Ethha_2110	K12132	prkC, stkP	eukaryotic-like serine/threonine-protein kinase	2.7.11.1	--

[denovogenes]_113050	45.24	5e-62	pdi:BDI_0805	K02477	K02477	two-component system, LytT family, response regulator	--	--

[denovogenes]_113051	97.60	4e-178	pdi:BDI_3537	K09013	sufC	Fe-S cluster assembly ATP-binding protein	--	--

[denovogenes]_113052	92.00	4e-173	pdi:BDI_2420	K02036	pstB	phosphate transport system ATP-binding protein	3.6.3.27	Environmental Information Processing--Membrane transport--ABC transporters

[denovogenes]_113053	43.60	2e-73	pdf:CD630DERM_16270	K18866	vanXY	D-alanyl-D-alanine dipeptidase/carboxypeptidase	3.4.13.22 3.4.16.4	Metabolism--Glycan biosynthesis and metabolism--Peptidoglycan biosynthesis|Metabolism--Global and overview maps--Metabolic pathways|Human Diseases--Drug resistance: Antimicrobial--Vancomycin resistance|Environmental Information Processing--Signal transduction--Two-component system

[denovogenes]_113054	62.08	3e-105	sbu:SpiBuddy_0193	K02564	nagB, GNPDA	glucosamine-6-phosphate deaminase	3.5.99.6	Metabolism--Carbohydrate metabolism--Amino sugar and nucleotide sugar metabolism|Metabolism--Global and overview maps--Metabolic pathways

[denovogenes]_113055	63.20	2e-115	rto:RTO_04200	K07074	K07074	uncharacterized protein	--	--

[denovogenes]_113056	100.00	0.0	btho:Btheta7330_04893	K09013	sufC	Fe-S cluster assembly ATP-binding protein	--	--

[denovogenes]_113059	80.32	5e-145	pdi:BDI_3955	K02233	E2.7.8.26, cobS, cobV	adenosylcobinamide-GDP ribazoletransferase	2.7.8.26	Metabolism--Metabolism of cofactors and vitamins--Porphyrin and chlorophyll metabolism|Metabolism--Global and overview maps--Metabolic pathways

[denovogenes]_113060	99.20	4e-165	paec:M802_283	K07090	K07090	uncharacterized protein	--	--

[denovogenes]_113062	42.21	1e-67	bfr:BF3365	K02477	K02477	two-component system, LytT family, response regulator	--	--

[denovogenes]_113064	80.41	2e-144	bhu:bhn_I2632	K02041	phnC	phosphonate transport system ATP-binding protein	3.6.3.28	Environmental Information Processing--Membrane transport--ABC transporters

[denovogenes]_113065	94.80	2e-176	pdi:BDI_2399	K06861	lptB	lipopolysaccharide export system ATP-binding protein	3.6.3.-	Environmental Information Processing--Membrane transport--ABC transporters

[denovogenes]_113066	98.80	0.0	fpla:A4U99_03305	K02392	flgG	flagellar basal-body rod protein FlgG	--	Cellular Processes--Cell motility--Flagellar assembly

[denovogenes]_113067	50.80	2e-80	fpa:FPR_30220	K20074	prpC, phpP	PPM family protein phosphatase	3.1.3.16	--

[denovogenes]_113068	98.40	0.0	bfb:VU15_21585	K01719	hemD, UROS	uroporphyrinogen-III synthase	4.2.1.75	Metabolism--Metabolism of cofactors and vitamins--Porphyrin and chlorophyll metabolism|Metabolism--Global and overview maps--Metabolic pathways|Metabolism--Global and overview maps--Biosynthesis of secondary metabolites

[denovogenes]_113070	99.20	0.0	cfd:CFNIH1_15620	K07251	thiK	thiamine kinase	2.7.1.89	Metabolism--Metabolism of cofactors and vitamins--Thiamine metabolism|Metabolism--Global and overview maps--Metabolic pathways

[denovogenes]_113072	61.04	9e-109	fpr:FP2_07850	K00215	dapB	4-hydroxy-tetrahydrodipicolinate reductase	1.17.1.8	Metabolism--Biosynthesis of other secondary metabolites--Monobactam biosynthesis|Metabolism--Amino acid metabolism--Lysine biosynthesis|Metabolism--Global and overview maps--Metabolic pathways|Metabolism--Global and overview maps--Biosynthesis of secondary metabolites|Metabolism--Global and overview maps--Microbial metabolism in diverse environments|Metabolism--Global and overview maps--Biosynthesis of antibiotics|Metabolism--Global and overview maps--Biosynthesis of amino acids

[denovogenes]_113073	100.00	0.0	paeo:M801_5176	K02030	ABC.PA.S	polar amino acid transport system substrate-binding protein	--	--

[denovogenes]_113075	100.00	0.0	paeo:M801_5194	K02030	ABC.PA.S	polar amino acid transport system substrate-binding protein	--	--

[denovogenes]_113076	70.00	3e-119	dor:Desor_5138	K00059	fabG	3-oxoacyl-[acyl-carrier protein] reductase	1.1.1.100	Metabolism--Lipid metabolism--Fatty acid biosynthesis|Metabolism--Metabolism of cofactors and vitamins--Biotin metabolism|Metabolism--Lipid metabolism--Biosynthesis of unsaturated fatty acids|Metabolism--Global and overview maps--Metabolic pathways|Metabolism--Global and overview maps--Fatty acid metabolism

[denovogenes]_113078	62.95	3e-108	dvg:Deval_0348	K02030	ABC.PA.S	polar amino acid transport system substrate-binding protein	--	--

[denovogenes]_11308	100.00	0.0	btho:Btheta7330_02394	K00700	GBE1, glgB	1,4-alpha-glucan branching enzyme	2.4.1.18	Metabolism--Carbohydrate metabolism--Starch and sucrose metabolism|Metabolism--Global and overview maps--Metabolic pathways|Metabolism--Global and overview maps--Biosynthesis of secondary metabolites

[denovogenes]_113080	100.00	0.0	stb:SGPB_0936	K02003	ABC.CD.A	putative ABC transport system ATP-binding protein	--	--

[denovogenes]_113082	81.38	7e-147	dvg:Deval_1138	K02028	ABC.PA.A	polar amino acid transport system ATP-binding protein	3.6.3.21	--

[denovogenes]_113087	99.20	0.0	cfd:CFNIH1_17045	K02013	ABC.FEV.A	iron complex transport system ATP-binding protein	3.6.3.34	Environmental Information Processing--Membrane transport--ABC transporters

[denovogenes]_113088	42.51	4e-62	bha:BH3661	K16698	tuaG	teichuronic acid biosynthesis glycosyltransferase TuaG	2.4.-.-	--

[denovogenes]_113089	99.20	0.0	fpla:A4U99_09720	K02013	ABC.FEV.A	iron complex transport system ATP-binding protein	3.6.3.34	Environmental Information Processing--Membrane transport--ABC transporters

[denovogenes]_113090	99.60	0.0	bfb:VU15_00675	K09013	sufC	Fe-S cluster assembly ATP-binding protein	--	--

[denovogenes]_113091	59.92	2e-102	ddh:Desde_0362	K07045	K07045	uncharacterized protein	--	--

[denovogenes]_113092	46.08	1e-17	mym:A176_002834	K07506	K07506	AraC family transcriptional regulator	--	--

[denovogenes]_113093	100.00	7e-178	paem:U769_22280	K02030	ABC.PA.S	polar amino acid transport system substrate-binding protein	--	--

[denovogenes]_113094	100.00	0.0	paeo:M801_303	K07010	K07010	putative glutamine amidotransferase	--	--

[denovogenes]_113095	100.00	0.0	btho:Btheta7330_02996	K07257	spsF	spore coat polysaccharide biosynthesis protein SpsF	--	--

[denovogenes]_113096	98.80	7e-171	paeo:M801_4085	K00059	fabG	3-oxoacyl-[acyl-carrier protein] reductase	1.1.1.100	Metabolism--Lipid metabolism--Fatty acid biosynthesis|Metabolism--Metabolism of cofactors and vitamins--Biotin metabolism|Metabolism--Lipid metabolism--Biosynthesis of unsaturated fatty acids|Metabolism--Global and overview maps--Metabolic pathways|Metabolism--Global and overview maps--Fatty acid metabolism

[denovogenes]_113097	100.00	2e-179	paeo:M801_2918	K05936	cobM, cbiF	precorrin-4/cobalt-precorrin-4 C11-methyltransferase	2.1.1.133 2.1.1.271	Metabolism--Metabolism of cofactors and vitamins--Porphyrin and chlorophyll metabolism|Metabolism--Global and overview maps--Metabolic pathways

[denovogenes]_113100	67.52	9e-111	csh:Closa_0624	K14742	tsaB	tRNA threonylcarbamoyladenosine biosynthesis protein TsaB	--	--

[denovogenes]_113101	54.44	1e-99	tjr:TherJR_1677	K00175	korB, oorB, oforB	2-oxoglutarate/2-oxoacid ferredoxin oxidoreductase subunit beta	1.2.7.3 1.2.7.11	Metabolism--Carbohydrate metabolism--Glycolysis / Gluconeogenesis|Metabolism--Carbohydrate metabolism--Citrate cycle (TCA cycle)|Metabolism--Carbohydrate metabolism--Pyruvate metabolism|Metabolism--Carbohydrate metabolism--Butanoate metabolism|Metabolism--Energy metabolism--Carbon fixation pathways in prokaryotes|Metabolism--Global and overview maps--Metabolic pathways|Metabolism--Global and overview maps--Microbial metabolism in diverse environments|Metabolism--Global and overview maps--Biosynthesis of antibiotics|Metabolism--Global and overview maps--Carbon metabolism

[denovogenes]_113103	100.00	0.0	cfd:CFNIH1_20810	K10010	ABC.CYST.A	cystine transport system ATP-binding protein	3.6.3.-	Environmental Information Processing--Membrane transport--ABC transporters

[denovogenes]_113105	95.60	2e-172	psa:PST_0722	K07180	prkA	serine protein kinase	--	--

[denovogenes]_113106	99.20	4e-170	lacy:A4V08_20645	K07090	K07090	uncharacterized protein	--	--

[denovogenes]_113107	56.57	7e-94	era:ERE_24630	K02529	lacI, galR	LacI family transcriptional regulator	--	--

[denovogenes]_113108	94.80	1e-179	lacy:A4V08_19695	K04069	pflA, pflC, pflE	pyruvate formate lyase activating enzyme	1.97.1.4	--

[denovogenes]_113109	100.00	0.0	cfd:CFNIH1_13895	K01834	PGAM, gpmA	2,3-bisphosphoglycerate-dependent phosphoglycerate mutase	5.4.2.11	Metabolism--Carbohydrate metabolism--Glycolysis / Gluconeogenesis|Metabolism--Amino acid metabolism--Glycine, serine and threonine metabolism|Metabolism--Energy metabolism--Methane metabolism|Metabolism--Global and overview maps--Metabolic pathways|Metabolism--Global and overview maps--Biosynthesis of secondary metabolites|Metabolism--Global and overview maps--Microbial metabolism in diverse environments|Metabolism--Global and overview maps--Biosynthesis of antibiotics|Metabolism--Global and overview maps--Carbon metabolism|Metabolism--Global and overview maps--Biosynthesis of amino acids|Organismal Systems--Endocrine system--Glucagon signaling pathway|Human Diseases--Cancers: Overview--Central carbon metabolism in cancer

[denovogenes]_11311	55.24	0.0	awo:Awo_c31510	K10797	enr	2-enoate reductase	1.3.1.31	Metabolism--Amino acid metabolism--Phenylalanine metabolism|Metabolism--Global and overview maps--Microbial metabolism in diverse environments

[denovogenes]_113110	41.41	4e-23	byl:A4V09_22815	K07720	yesN	two-component system, response regulator YesN	--	Environmental Information Processing--Signal transduction--Two-component system

[denovogenes]_113111	46.93	1e-67	pmr:PMI0300	K12519	papD	chaperone protein PapD	--	--

[denovogenes]_113116	96.00	4e-175	lacy:A4V08_25920	K12410	npdA	NAD-dependent deacetylase	3.5.1.-	--

[denovogenes]_11312	47.68	5e-101	vbl:L21SP4_00868	K20974	K20974	two-component system, sensor histidine kinase	2.7.13.3	Environmental Information Processing--Signal transduction--Two-component system|Cellular Processes--Cellular community - prokaryotes--Biofilm formation - Pseudomonas aeruginosa

[denovogenes]_113120	84.62	3e-155	pdi:BDI_0596	K01834	PGAM, gpmA	2,3-bisphosphoglycerate-dependent phosphoglycerate mutase	5.4.2.11	Metabolism--Carbohydrate metabolism--Glycolysis / Gluconeogenesis|Metabolism--Amino acid metabolism--Glycine, serine and threonine metabolism|Metabolism--Energy metabolism--Methane metabolism|Metabolism--Global and overview maps--Metabolic pathways|Metabolism--Global and overview maps--Biosynthesis of secondary metabolites|Metabolism--Global and overview maps--Microbial metabolism in diverse environments|Metabolism--Global and overview maps--Biosynthesis of antibiotics|Metabolism--Global and overview maps--Carbon metabolism|Metabolism--Global and overview maps--Biosynthesis of amino acids|Organismal Systems--Endocrine system--Glucagon signaling pathway|Human Diseases--Cancers: Overview--Central carbon metabolism in cancer

[denovogenes]_113121	92.40	4e-174	pdi:BDI_2420	K02036	pstB	phosphate transport system ATP-binding protein	3.6.3.27	Environmental Information Processing--Membrane transport--ABC transporters

[denovogenes]_113122	55.56	1e-85	csh:Closa_3886	K09807	K09807	uncharacterized protein	--	--

[denovogenes]_113124	69.48	3e-120	lacy:A4V08_31165	K02421	fliR	flagellar biosynthetic protein FliR	--	Cellular Processes--Cell motility--Flagellar assembly

[denovogenes]_113125	98.00	0.0	pdi:BDI_1805	K04652	hypB	hydrogenase nickel incorporation protein HypB	--	--

[denovogenes]_113127	45.85	2e-64	bha:BH3444	K07497	K07497	putative transposase	--	--

[denovogenes]_113128	57.14	9e-89	cph:Cpha266_2288	K05540	dusB	tRNA-dihydrouridine synthase B	1.-.-.-	--

[denovogenes]_113129	86.29	1e-148	pfre:RM25_0448	K01992	ABC-2.P	ABC-2 type transport system permease protein	--	--

[denovogenes]_11313	40.90	0.0	ssm:Spirs_3619	K10439	rbsB	ribose transport system substrate-binding protein	--	Environmental Information Processing--Membrane transport--ABC transporters|Cellular Processes--Cell motility--Bacterial chemotaxis

[denovogenes]_113130	100.00	0.0	cfd:CFNIH1_13480	K02566	nagD	NagD protein	--	--

[denovogenes]_113133	64.78	1e-111	dvm:DvMF_1790	K17103	CHO1, pssA	CDP-diacylglycerol---serine O-phosphatidyltransferase	2.7.8.8	Metabolism--Amino acid metabolism--Glycine, serine and threonine metabolism|Metabolism--Lipid metabolism--Glycerophospholipid metabolism|Metabolism--Global and overview maps--Metabolic pathways|Metabolism--Global and overview maps--Biosynthesis of secondary metabolites

[denovogenes]_113134	100.00	0.0	paeo:M801_2049	K06137	pqqC	pyrroloquinoline-quinone synthase	1.3.3.11	--

[denovogenes]_113135	78.33	4e-140	cbz:Cbs_3220	K01744	aspA	aspartate ammonia-lyase	4.3.1.1	Metabolism--Amino acid metabolism--Alanine, aspartate and glutamate metabolism|Metabolism--Global and overview maps--Metabolic pathways

[denovogenes]_113136	67.07	2e-122	cbi:CLJ_B1379	K00571	E2.1.1.72	site-specific DNA-methyltransferase (adenine-specific)	2.1.1.72	--

[denovogenes]_113137	41.86	9e-69	sli:Slin_2091	K02477	K02477	two-component system, LytT family, response regulator	--	--

[denovogenes]_113138	51.84	4e-72	ecas:ECBG_02011	K06201	cutC	copper homeostasis protein	--	--

[denovogenes]_113139	100.00	0.0	pdf:CD630DERM_01170	K00175	korB, oorB, oforB	2-oxoglutarate/2-oxoacid ferredoxin oxidoreductase subunit beta	1.2.7.3 1.2.7.11	Metabolism--Carbohydrate metabolism--Glycolysis / Gluconeogenesis|Metabolism--Carbohydrate metabolism--Citrate cycle (TCA cycle)|Metabolism--Carbohydrate metabolism--Pyruvate metabolism|Metabolism--Carbohydrate metabolism--Butanoate metabolism|Metabolism--Energy metabolism--Carbon fixation pathways in prokaryotes|Metabolism--Global and overview maps--Metabolic pathways|Metabolism--Global and overview maps--Microbial metabolism in diverse environments|Metabolism--Global and overview maps--Biosynthesis of antibiotics|Metabolism--Global and overview maps--Carbon metabolism

[denovogenes]_11314	99.85	0.0	paeo:M801_3116	K03702	uvrB	excinuclease ABC subunit B	--	Genetic Information Processing--Replication and repair--Nucleotide excision repair

[denovogenes]_113140	64.26	1e-105	fpla:A4U99_16930	K02057	ABC.SS.P	simple sugar transport system permease protein	--	--

[denovogenes]_113142	91.87	9e-167	pdi:BDI_3054	K01719	hemD, UROS	uroporphyrinogen-III synthase	4.2.1.75	Metabolism--Metabolism of cofactors and vitamins--Porphyrin and chlorophyll metabolism|Metabolism--Global and overview maps--Metabolic pathways|Metabolism--Global and overview maps--Biosynthesis of secondary metabolites

[denovogenes]_113143	80.40	2e-144	seeh:SEEH1578_01230	K00059	fabG	3-oxoacyl-[acyl-carrier protein] reductase	1.1.1.100	Metabolism--Lipid metabolism--Fatty acid biosynthesis|Metabolism--Metabolism of cofactors and vitamins--Biotin metabolism|Metabolism--Lipid metabolism--Biosynthesis of unsaturated fatty acids|Metabolism--Global and overview maps--Metabolic pathways|Metabolism--Global and overview maps--Fatty acid metabolism

[denovogenes]_113146	98.80	2e-180	pdi:BDI_0629	K05878	dhaK	dihydroxyacetone kinase, N-terminal domain	2.7.1.-	Metabolism--Lipid metabolism--Glycerolipid metabolism|Metabolism--Global and overview maps--Metabolic pathways

[denovogenes]_113147	68.98	1e-126	eac:EAL2_808p05010	K00175	korB, oorB, oforB	2-oxoglutarate/2-oxoacid ferredoxin oxidoreductase subunit beta	1.2.7.3 1.2.7.11	Metabolism--Carbohydrate metabolism--Glycolysis / Gluconeogenesis|Metabolism--Carbohydrate metabolism--Citrate cycle (TCA cycle)|Metabolism--Carbohydrate metabolism--Pyruvate metabolism|Metabolism--Carbohydrate metabolism--Butanoate metabolism|Metabolism--Energy metabolism--Carbon fixation pathways in prokaryotes|Metabolism--Global and overview maps--Metabolic pathways|Metabolism--Global and overview maps--Microbial metabolism in diverse environments|Metabolism--Global and overview maps--Biosynthesis of antibiotics|Metabolism--Global and overview maps--Carbon metabolism

[denovogenes]_113148	85.48	1e-162	pdi:BDI_1936	K06173	truA, PUS1	tRNA pseudouridine38-40 synthase	5.4.99.12	--

[denovogenes]_113149	99.60	4e-175	paeg:AI22_13690	K12410	npdA	NAD-dependent deacetylase	3.5.1.-	--

[denovogenes]_11315	99.70	0.0	fpla:A4U99_05985	K03406	mcp	methyl-accepting chemotaxis protein	--	Environmental Information Processing--Signal transduction--Two-component system|Cellular Processes--Cell motility--Bacterial chemotaxis

[denovogenes]_113150	47.39	9e-54	ypq:DJ40_3133	K18320	--	--	--	--

[denovogenes]_113152	100.00	0.0	ecoa:APECO78_18935	K03710	K03710	GntR family transcriptional regulator	--	--

[denovogenes]_113156	44.31	6e-74	lio:JL53_03185	K03436	fruR2, fruR	DeoR family transcriptional regulator, fructose operon transcriptional repressor	--	--

[denovogenes]_113157	92.00	2e-172	pdi:BDI_2563	K03524	birA	BirA family transcriptional regulator, biotin operon repressor / biotin-[acetyl-CoA-carboxylase] ligase	6.3.4.15	Metabolism--Metabolism of cofactors and vitamins--Biotin metabolism|Metabolism--Global and overview maps--Metabolic pathways

[denovogenes]_113159	61.13	1e-113	cls:CXIVA_11480	K15460	yfiC, trmX	tRNA1Val (adenine37-N6)-methyltransferase	2.1.1.223	--

[denovogenes]_11316	64.79	0.0	dvm:DvMF_0503	K02355	fusA, GFM, EFG	elongation factor G	--	--

[denovogenes]_113160	78.80	5e-145	pdi:BDI_0380	K03585	acrA, mexA, adeI, smeD, mtrC, cmeA	membrane fusion protein, multidrug efflux system	--	Human Diseases--Drug resistance: Antimicrobial--beta-Lactam resistance|Human Diseases--Drug resistance: Antimicrobial--Cationic antimicrobial peptide (CAMP) resistance

[denovogenes]_113161	41.37	8e-56	ljf:FI9785_1605	K02755	PTS-Bgl-EIIA, bglF, bglP	PTS system, beta-glucoside-specific IIA component	2.7.1.-	Environmental Information Processing--Membrane transport--Phosphotransferase system (PTS)

[denovogenes]_113161	41.37	8e-56	ljf:FI9785_1605	K02756	PTS-Bgl-EIIB, bglF, bglP	PTS system, beta-glucoside-specific IIB component	2.7.1.-	Environmental Information Processing--Membrane transport--Phosphotransferase system (PTS)

[denovogenes]_113161	41.37	8e-56	ljf:FI9785_1605	K02757	PTS-Bgl-EIIC, bglF, bglP	PTS system, beta-glucoside-specific IIC component	--	Environmental Information Processing--Membrane transport--Phosphotransferase system (PTS)

[denovogenes]_113163	51.21	3e-75	dfi:AXF13_09695	K01779	racD	aspartate racemase	5.1.1.13	Metabolism--Amino acid metabolism--Alanine, aspartate and glutamate metabolism|Metabolism--Metabolism of terpenoids and polyketides--Nonribosomal peptide structures

[denovogenes]_113166	51.39	3e-87	bsca:BBSC_1816	K01191	E3.2.1.24	alpha-mannosidase	3.2.1.24	Metabolism--Glycan biosynthesis and metabolism--Other glycan degradation

[denovogenes]_113167	58.26	1e-85	pary:A4V02_08670	K10716	kch, trkA, mthK, pch	voltage-gated potassium channel	--	--

[denovogenes]_11317	100.00	0.0	paf:PAM18_4195	K12349	ASAH2	neutral ceramidase	3.5.1.23	Metabolism--Lipid metabolism--Sphingolipid metabolism|Metabolism--Global and overview maps--Metabolic pathways|Environmental Information Processing--Signal transduction--Sphingolipid signaling pathway

[denovogenes]_113170	97.21	5e-176	bcel:BcellWH2_00527	K02314	dnaB	replicative DNA helicase	3.6.4.12	Genetic Information Processing--Replication and repair--DNA replication|Cellular Processes--Cell growth and death--Cell cycle - Caulobacter

[denovogenes]_113175	90.00	2e-174	bcel:BcellWH2_04160	K06173	truA, PUS1	tRNA pseudouridine38-40 synthase	5.4.99.12	--

[denovogenes]_113177	86.69	5e-156	pdi:BDI_3955	K02233	E2.7.8.26, cobS, cobV	adenosylcobinamide-GDP ribazoletransferase	2.7.8.26	Metabolism--Metabolism of cofactors and vitamins--Porphyrin and chlorophyll metabolism|Metabolism--Global and overview maps--Metabolic pathways

[denovogenes]_113180	41.04	4e-21	eic:NT01EI_3776	K03755	adiY	AraC family transcriptional regulator, transcriptional activator of adiA	--	--

[denovogenes]_113182	98.80	2e-173	sdy:SDY_0490	K16053	mscM	miniconductance mechanosensitive channel	--	--

[denovogenes]_113183	48.55	4e-85	pdf:CD630DERM_04660	K03481	glvR	RpiR family transcriptional regulator, glv operon transcriptional regulator	--	--

[denovogenes]_113184	99.60	0.0	cfd:CFNIH1_01455	K04763	xerD	integrase/recombinase XerD	--	--

[denovogenes]_113186	100.00	0.0	kpne:KU54_01075	K19276	aac3-IV	aminoglycoside 3-N-acetyltransferase IV	2.3.1.81	--

[denovogenes]_113187	72.11	3e-136	bdo:EL88_07255	K01012	bioB	biotin synthase	2.8.1.6	Metabolism--Metabolism of cofactors and vitamins--Biotin metabolism|Metabolism--Global and overview maps--Metabolic pathways

[denovogenes]_113188	63.97	1e-111	ctc:CTC_01012	K01255	CARP, pepA	leucyl aminopeptidase	3.4.11.1	Metabolism--Metabolism of other amino acids--Glutathione metabolism|Metabolism--Global and overview maps--Metabolic pathways

[denovogenes]_113189	100.00	0.0	cfd:CFNIH1_14720	K01893	NARS, asnS	asparaginyl-tRNA synthetase	6.1.1.22	Genetic Information Processing--Translation--Aminoacyl-tRNA biosynthesis

[denovogenes]_113190	100.00	0.0	sft:NCTC1_03350	K03272	gmhC, hldE, waaE, rfaE	D-beta-D-heptose 7-phosphate kinase / D-beta-D-heptose 1-phosphate adenosyltransferase	2.7.1.167 2.7.7.70	Metabolism--Glycan biosynthesis and metabolism--Lipopolysaccharide biosynthesis|Metabolism--Global and overview maps--Metabolic pathways

[denovogenes]_113195	100.00	0.0	ecoh:ECRM13516_1739	K14058	ttcA	tRNA 2-thiocytidine biosynthesis protein TtcA	--	--

[denovogenes]_113196	100.00	0.0	pdf:CD630DERM_18280	K03798	ftsH, hflB	cell division protease FtsH	3.4.24.-	--

[denovogenes]_113198	96.85	8e-144	bcel:BcellWH2_04551	K07507	mgtC	putative Mg2+ transporter-C (MgtC) family protein	--	--

[denovogenes]_113199	92.43	3e-171	bcel:BcellWH2_01070	K02481	K02481	two-component system, NtrC family, response regulator	--	--

[denovogenes]_1132	99.92	0.0	paes:SCV20265_1415	K02337	DPO3A1, dnaE	DNA polymerase III subunit alpha	2.7.7.7	Metabolism--Nucleotide metabolism--Purine metabolism|Metabolism--Nucleotide metabolism--Pyrimidine metabolism|Metabolism--Global and overview maps--Metabolic pathways|Genetic Information Processing--Replication and repair--DNA replication|Genetic Information Processing--Replication and repair--Mismatch repair|Genetic Information Processing--Replication and repair--Homologous recombination

[denovogenes]_113202	99.20	0.0	pdi:BDI_2420	K02036	pstB	phosphate transport system ATP-binding protein	3.6.3.27	Environmental Information Processing--Membrane transport--ABC transporters

[denovogenes]_113207	67.51	7e-117	csh:Closa_3596	K01488	add, ADA	adenosine deaminase	3.5.4.4	Metabolism--Nucleotide metabolism--Purine metabolism|Metabolism--Global and overview maps--Metabolic pathways|Human Diseases--Immune diseases--Primary immunodeficiency

[denovogenes]_113208	100.00	0.0	cfd:CFNIH1_20810	K10010	ABC.CYST.A	cystine transport system ATP-binding protein	3.6.3.-	Environmental Information Processing--Membrane transport--ABC transporters

[denovogenes]_113209	53.62	8e-79	hsd:SD1D_0727	K07150	K07150	uncharacterized protein	--	--

[denovogenes]_11321	40.94	2e-87	fre:Franean1_2292	K06048	ybdK	carboxylate-amine ligase	6.3.-.-	--

[denovogenes]_113210	98.80	8e-178	pmr:PMI0221	K01689	ENO, eno	enolase	4.2.1.11	Metabolism--Carbohydrate metabolism--Glycolysis / Gluconeogenesis|Metabolism--Energy metabolism--Methane metabolism|Metabolism--Global and overview maps--Metabolic pathways|Metabolism--Global and overview maps--Biosynthesis of secondary metabolites|Metabolism--Global and overview maps--Microbial metabolism in diverse environments|Metabolism--Global and overview maps--Biosynthesis of antibiotics|Metabolism--Global and overview maps--Carbon metabolism|Metabolism--Global and overview maps--Biosynthesis of amino acids|Genetic Information Processing--Folding, sorting and degradation--RNA degradation|Environmental Information Processing--Signal transduction--HIF-1 signaling pathway

[denovogenes]_113211	84.74	3e-157	csh:Closa_1003	K01813	rhaA	L-rhamnose isomerase	5.3.1.14	Metabolism--Carbohydrate metabolism--Fructose and mannose metabolism|Metabolism--Global and overview maps--Microbial metabolism in diverse environments

[denovogenes]_113214	53.53	1e-90	vha:VIBHAR_05410	K01297	ldcA	muramoyltetrapeptide carboxypeptidase	3.4.17.13	--

[denovogenes]_113216	51.81	6e-84	erb:A4V01_01065	K02761	PTS-Cel-EIIC, celB, chbC	PTS system, cellobiose-specific IIC component	--	Metabolism--Carbohydrate metabolism--Starch and sucrose metabolism|Environmental Information Processing--Membrane transport--Phosphotransferase system (PTS)

[denovogenes]_113217	95.62	2e-167	pvl:AOB99_09875	K03305	TC.POT	proton-dependent oligopeptide transporter, POT family	--	--

[denovogenes]_113219	92.03	8e-150	lacy:A4V08_08015	K09807	K09807	uncharacterized protein	--	--

[denovogenes]_11322	98.51	0.0	cfd:CFNIH1_14590	K02238	comEC	competence protein ComEC	--	--

[denovogenes]_113220	98.80	0.0	bxy:BXY_03260	K00979	kdsB	3-deoxy-manno-octulosonate cytidylyltransferase (CMP-KDO synthetase)	2.7.7.38	Metabolism--Glycan biosynthesis and metabolism--Lipopolysaccharide biosynthesis|Metabolism--Global and overview maps--Metabolic pathways

[denovogenes]_113221	80.40	3e-143	bprs:CK3_09000	K01625	eda	2-dehydro-3-deoxyphosphogluconate aldolase / (4S)-4-hydroxy-2-oxoglutarate aldolase	4.1.2.14 4.1.3.42	Metabolism--Carbohydrate metabolism--Pentose phosphate pathway|Metabolism--Carbohydrate metabolism--Glyoxylate and dicarboxylate metabolism|Metabolism--Global and overview maps--Metabolic pathways|Metabolism--Global and overview maps--Microbial metabolism in diverse environments|Metabolism--Global and overview maps--Carbon metabolism

[denovogenes]_113223	47.24	7e-58	shi:Shel_08840	K03588	ftsW, spoVE	cell division protein FtsW	--	Cellular Processes--Cell growth and death--Cell cycle - Caulobacter

[denovogenes]_113224	62.60	4e-111	sus:Acid_6953	K01067	E3.1.2.1, ACH1	acetyl-CoA hydrolase	3.1.2.1	Metabolism--Carbohydrate metabolism--Pyruvate metabolism

[denovogenes]_113225	98.80	1e-179	stu:STH8232_0659	K00558	DNMT1, dcm	DNA (cytosine-5)-methyltransferase 1	2.1.1.37	Metabolism--Amino acid metabolism--Cysteine and methionine metabolism|Metabolism--Global and overview maps--Metabolic pathways|Human Diseases--Cancers: Overview--MicroRNAs in cancer

[denovogenes]_113227	40.66	6e-54	nth:Nther_0884	K00533	E1.12.7.2L	ferredoxin hydrogenase large subunit	1.12.7.2	--

[denovogenes]_113229	97.61	1e-179	pvl:AOB99_06850	K18850	ycfD	50S ribosomal protein L16 3-hydroxylase	1.14.11.47	--

[denovogenes]_11323	98.21	0.0	sot:102588246	K03283	HSPA1_8	heat shock 70kDa protein 1/8	--	Genetic Information Processing--Transcription--Spliceosome|Environmental Information Processing--Signal transduction--MAPK signaling pathway|Genetic Information Processing--Folding, sorting and degradation--Protein processing in endoplasmic reticulum|Cellular Processes--Transport and catabolism--Endocytosis|Organismal Systems--Aging--Longevity regulating pathway - multiple species|Organismal Systems--Immune system--Antigen processing and presentation|Organismal Systems--Endocrine system--Estrogen signaling pathway|Human Diseases--Infectious diseases: Bacterial--Legionellosis|Human Diseases--Infectious diseases: Parasitic--Toxoplasmosis|Human Diseases--Infectious diseases: Viral--Measles|Human Diseases--Infectious diseases: Viral--Influenza A|Human Diseases--Infectious diseases: Viral--Epstein-Barr virus infection

[denovogenes]_113233	100.00	3e-67	bbrc:B7019_1261	K07506	K07506	AraC family transcriptional regulator	--	--

[denovogenes]_113235	60.43	1e-52	clr:UPTC16701_1238	K15915	pglC	undecaprenyl phosphate N,N'-diacetylbacillosamine 1-phosphate transferase	2.7.8.36	--

[denovogenes]_113236	49.37	4e-76	gfo:GFO_0359	K01138	K01138	uncharacterized sulfatase	3.1.6.-	--

[denovogenes]_113237	54.98	7e-86	bado:BBMN23_0470	K05349	bglX	beta-glucosidase	3.2.1.21	Metabolism--Metabolism of other amino acids--Cyanoamino acid metabolism|Metabolism--Carbohydrate metabolism--Starch and sucrose metabolism|Metabolism--Biosynthesis of other secondary metabolites--Phenylpropanoid biosynthesis|Metabolism--Global and overview maps--Metabolic pathways|Metabolism--Global and overview maps--Biosynthesis of secondary metabolites

[denovogenes]_113239	98.80	0.0	bhl:Bache_2263	K01881	PARS, proS	prolyl-tRNA synthetase	6.1.1.15	Genetic Information Processing--Translation--Aminoacyl-tRNA biosynthesis

[denovogenes]_113241	79.28	2e-144	byl:A4V09_20075	K00266	gltD	glutamate synthase (NADPH/NADH) small chain	1.4.1.13 1.4.1.14	Metabolism--Amino acid metabolism--Alanine, aspartate and glutamate metabolism|Metabolism--Energy metabolism--Nitrogen metabolism|Metabolism--Global and overview maps--Metabolic pathways|Metabolism--Global and overview maps--Biosynthesis of secondary metabolites|Metabolism--Global and overview maps--Microbial metabolism in diverse environments|Metabolism--Global and overview maps--Biosynthesis of antibiotics|Metabolism--Global and overview maps--Biosynthesis of amino acids

[denovogenes]_113244	42.80	6e-54	fpy:FPG101_02695	K07316	mod	adenine-specific DNA-methyltransferase	2.1.1.72	--

[denovogenes]_113246	100.00	5e-172	paep:PA1S_15380	K13896	yejF	microcin C transport system ATP-binding protein	--	Environmental Information Processing--Membrane transport--ABC transporters

[denovogenes]_113247	96.81	0.0	pmib:BB2000_1635	K03601	xseA	exodeoxyribonuclease VII large subunit	3.1.11.6	Genetic Information Processing--Replication and repair--Mismatch repair

[denovogenes]_113249	46.24	1e-45	ctet:BN906_00769	K06042	cobH-cbiC	precorrin-8X/cobalt-precorrin-8 methylmutase	5.4.99.61 5.4.99.60	Metabolism--Metabolism of cofactors and vitamins--Porphyrin and chlorophyll metabolism|Metabolism--Global and overview maps--Metabolic pathways

[denovogenes]_11325	75.97	0.0	bcel:BcellWH2_00356	K16089	TC.FEV.OM2, cirA, cfrA, hmuR	outer membrane receptor for ferrienterochelin and colicins	--	--

[denovogenes]_113252	54.33	1e-90	pth:PTH_1060	K01872	AARS, alaS	alanyl-tRNA synthetase	6.1.1.7	Genetic Information Processing--Translation--Aminoacyl-tRNA biosynthesis

[denovogenes]_113255	100.00	0.0	pdf:CD630DERM_36640	K05825	LYSN	2-aminoadipate transaminase	2.6.1.-	Metabolism--Amino acid metabolism--Lysine biosynthesis|Metabolism--Global and overview maps--Metabolic pathways|Metabolism--Global and overview maps--Biosynthesis of antibiotics|Metabolism--Global and overview maps--2-Oxocarboxylic acid metabolism|Metabolism--Global and overview maps--Biosynthesis of amino acids

[denovogenes]_113258	49.33	3e-69	efu:HMPREF0351_10904	K20444	rfbC	O-antigen biosynthesis protein	2.4.1.-	--

[denovogenes]_11326	77.83	0.0	btho:Btheta7330_05148	K07407	E3.2.1.22B, galA, rafA	alpha-galactosidase	3.2.1.22	Metabolism--Carbohydrate metabolism--Galactose metabolism|Metabolism--Lipid metabolism--Glycerolipid metabolism|Metabolism--Lipid metabolism--Sphingolipid metabolism|Metabolism--Glycan biosynthesis and metabolism--Glycosphingolipid biosynthesis - globo and isoglobo series

[denovogenes]_113260	55.82	4e-95	toh:BCB71_05930	K09181	yfiQ	acetyltransferase	--	--

[denovogenes]_113262	99.60	9e-179	pvl:AOB99_14025	K07783	uhpC	MFS transporter, OPA family, sugar phosphate sensor protein UhpC	--	Environmental Information Processing--Signal transduction--Two-component system

[denovogenes]_113263	59.92	1e-94	gfe:Gferi_14940	K06714	rocR	arginine utilization regulatory protein	--	--

[denovogenes]_113266	60.80	6e-112	psn:Pedsa_1191	K00059	fabG	3-oxoacyl-[acyl-carrier protein] reductase	1.1.1.100	Metabolism--Lipid metabolism--Fatty acid biosynthesis|Metabolism--Metabolism of cofactors and vitamins--Biotin metabolism|Metabolism--Lipid metabolism--Biosynthesis of unsaturated fatty acids|Metabolism--Global and overview maps--Metabolic pathways|Metabolism--Global and overview maps--Fatty acid metabolism

[denovogenes]_113267	100.00	0.0	boa:Bovatus_03543	K09013	sufC	Fe-S cluster assembly ATP-binding protein	--	--

[denovogenes]_113268	99.60	0.0	boa:Bovatus_01412	K02500	hisF	cyclase	4.1.3.-	Metabolism--Amino acid metabolism--Histidine metabolism|Metabolism--Global and overview maps--Metabolic pathways|Metabolism--Global and overview maps--Biosynthesis of secondary metabolites|Metabolism--Global and overview maps--Biosynthesis of amino acids

[denovogenes]_11327	99.70	0.0	boa:Bovatus_00960	K01187	malZ	alpha-glucosidase	3.2.1.20	Metabolism--Carbohydrate metabolism--Galactose metabolism|Metabolism--Carbohydrate metabolism--Starch and sucrose metabolism|Metabolism--Global and overview maps--Metabolic pathways

[denovogenes]_113272	40.76	9e-47	lla:L33782	K00897	aphA	kanamycin kinase	2.7.1.95	--

[denovogenes]_113273	96.40	0.0	boa:Bovatus_04355	K01815	kduI	4-deoxy-L-threo-5-hexosulose-uronate ketol-isomerase	5.3.1.17	Metabolism--Carbohydrate metabolism--Pentose and glucuronate interconversions

[denovogenes]_113275	60.24	2e-107	rto:RTO_10390	K00059	fabG	3-oxoacyl-[acyl-carrier protein] reductase	1.1.1.100	Metabolism--Lipid metabolism--Fatty acid biosynthesis|Metabolism--Metabolism of cofactors and vitamins--Biotin metabolism|Metabolism--Lipid metabolism--Biosynthesis of unsaturated fatty acids|Metabolism--Global and overview maps--Metabolic pathways|Metabolism--Global and overview maps--Fatty acid metabolism

[denovogenes]_113276	47.11	3e-76	ckr:CKR_0014	K18349	vanRC, vanRE, vanRG	two-component system, OmpR family, response regulator VanR	--	Human Diseases--Drug resistance: Antimicrobial--Vancomycin resistance|Environmental Information Processing--Signal transduction--Two-component system

[denovogenes]_113277	45.45	8e-65	bco:Bcell_2513	K04096	smf	DNA processing protein	--	--

[denovogenes]_113278	92.00	7e-175	bfg:BF638R_3662	K00571	E2.1.1.72	site-specific DNA-methyltransferase (adenine-specific)	2.1.1.72	--

[denovogenes]_113281	51.63	3e-86	dhd:Dhaf_0239	K09861	K09861	uncharacterized protein	--	--

[denovogenes]_113283	99.20	0.0	kpv:KPNIH29_26945	K06889	K06889	uncharacterized protein	--	--

[denovogenes]_113283	99.20	0.0	kpv:KPNIH29_26945	K07397	yhfA	putative redox protein	--	--

[denovogenes]_113284	72.40	8e-138	erb:A4V01_09300	K01834	PGAM, gpmA	2,3-bisphosphoglycerate-dependent phosphoglycerate mutase	5.4.2.11	Metabolism--Carbohydrate metabolism--Glycolysis / Gluconeogenesis|Metabolism--Amino acid metabolism--Glycine, serine and threonine metabolism|Metabolism--Energy metabolism--Methane metabolism|Metabolism--Global and overview maps--Metabolic pathways|Metabolism--Global and overview maps--Biosynthesis of secondary metabolites|Metabolism--Global and overview maps--Microbial metabolism in diverse environments|Metabolism--Global and overview maps--Biosynthesis of antibiotics|Metabolism--Global and overview maps--Carbon metabolism|Metabolism--Global and overview maps--Biosynthesis of amino acids|Organismal Systems--Endocrine system--Glucagon signaling pathway|Human Diseases--Cancers: Overview--Central carbon metabolism in cancer

[denovogenes]_113285	58.00	2e-101	bbag:E1O_22620	K09691	ABC-2.LPSE.A	lipopolysaccharide transport system ATP-binding protein	--	Environmental Information Processing--Membrane transport--ABC transporters

[denovogenes]_113286	99.60	0.0	pdi:BDI_3439	K03100	lepB, TPP	signal peptidase I	3.4.21.89	Cellular Processes--Cellular community - prokaryotes--Quorum sensing|Genetic Information Processing--Folding, sorting and degradation--Protein export

[denovogenes]_113289	43.20	2e-66	pmr:PMI0300	K12519	papD	chaperone protein PapD	--	--

[denovogenes]_11329	59.76	0.0	cpro:CPRO_03770	K04759	feoB	ferrous iron transport protein B	--	--

[denovogenes]_113290	44.58	9e-73	bco:Bcell_3649	K16698	tuaG	teichuronic acid biosynthesis glycosyltransferase TuaG	2.4.-.-	--

[denovogenes]_113291	42.50	1e-56	smw:SMWW4_v1c06200	K12519	papD	chaperone protein PapD	--	--

[denovogenes]_113293	43.75	3e-62	llu:AKJ09_10033	K04656	hypF	hydrogenase maturation protein HypF	--	--

[denovogenes]_113296	63.49	6e-111	dpn:BCB69_04340	K03798	ftsH, hflB	cell division protease FtsH	3.4.24.-	--

[denovogenes]_113298	60.64	6e-101	efq:DR75_2481	K17686	copA, ATP7	Cu+-exporting ATPase	3.6.3.54	Human Diseases--Drug resistance: Antineoplastic--Platinum drug resistance|Environmental Information Processing--Signal transduction--MAPK signaling pathway - plant

[denovogenes]_1133	57.75	0.0	fpa:FPR_03790	K03529	smc	chromosome segregation protein	--	--

[denovogenes]_11330	99.10	0.0	boa:Bovatus_00854	K00700	GBE1, glgB	1,4-alpha-glucan branching enzyme	2.4.1.18	Metabolism--Carbohydrate metabolism--Starch and sucrose metabolism|Metabolism--Global and overview maps--Metabolic pathways|Metabolism--Global and overview maps--Biosynthesis of secondary metabolites

[denovogenes]_113300	100.00	0.0	cpe:CPE0286	K01784	galE, GALE	UDP-glucose 4-epimerase	5.1.3.2	Metabolism--Carbohydrate metabolism--Galactose metabolism|Metabolism--Carbohydrate metabolism--Amino sugar and nucleotide sugar metabolism|Metabolism--Global and overview maps--Metabolic pathways

[denovogenes]_113302	100.00	0.0	pdi:BDI_2399	K06861	lptB	lipopolysaccharide export system ATP-binding protein	3.6.3.-	Environmental Information Processing--Membrane transport--ABC transporters

[denovogenes]_113306	97.21	5e-169	pdi:BDI_1863	K00845	glk	glucokinase	2.7.1.2	Metabolism--Carbohydrate metabolism--Glycolysis / Gluconeogenesis|Metabolism--Carbohydrate metabolism--Galactose metabolism|Metabolism--Carbohydrate metabolism--Starch and sucrose metabolism|Metabolism--Carbohydrate metabolism--Amino sugar and nucleotide sugar metabolism|Metabolism--Biosynthesis of other secondary metabolites--Streptomycin biosynthesis|Metabolism--Biosynthesis of other secondary metabolites--Neomycin, kanamycin and gentamicin biosynthesis|Metabolism--Global and overview maps--Metabolic pathways|Metabolism--Global and overview maps--Biosynthesis of secondary metabolites|Metabolism--Global and overview maps--Microbial metabolism in diverse environments|Metabolism--Global and overview maps--Biosynthesis of antibiotics|Metabolism--Global and overview maps--Carbon metabolism

[denovogenes]_113308	66.26	7e-126	vpr:Vpar_1658	K07098	K07098	uncharacterized protein	--	--

[denovogenes]_113312	99.60	0.0	eoc:CE10_0875	K05844	rimK	ribosomal protein S6--L-glutamate ligase	6.3.2.-	--

[denovogenes]_113316	66.02	5e-115	cdf:CD630_10540	K00248	ACADS, bcd	butyryl-CoA dehydrogenase	1.3.8.1	Metabolism--Lipid metabolism--Fatty acid degradation|Metabolism--Amino acid metabolism--Valine, leucine and isoleucine degradation|Metabolism--Carbohydrate metabolism--Butanoate metabolism|Metabolism--Global and overview maps--Metabolic pathways|Metabolism--Global and overview maps--Biosynthesis of secondary metabolites|Metabolism--Global and overview maps--Carbon metabolism|Metabolism--Global and overview maps--Fatty acid metabolism

[denovogenes]_113317	73.31	6e-133	ipo:Ilyop_0622	K01937	pyrG, CTPS	CTP synthase	6.3.4.2	Metabolism--Nucleotide metabolism--Pyrimidine metabolism|Metabolism--Global and overview maps--Metabolic pathways

[denovogenes]_113319	100.00	0.0	shq:A0259_10560	K00208	fabI	enoyl-[acyl-carrier protein] reductase I	1.3.1.9 1.3.1.10	Metabolism--Lipid metabolism--Fatty acid biosynthesis|Metabolism--Metabolism of cofactors and vitamins--Biotin metabolism|Metabolism--Global and overview maps--Metabolic pathways|Metabolism--Global and overview maps--Fatty acid metabolism

[denovogenes]_113322	76.42	8e-135	cbv:U729_32	K03584	recO	DNA repair protein RecO (recombination protein O)	--	Genetic Information Processing--Replication and repair--Homologous recombination

[denovogenes]_113323	67.89	5e-118	cbv:U729_1649	K03436	fruR2, fruR	DeoR family transcriptional regulator, fructose operon transcriptional repressor	--	--

[denovogenes]_113324	76.13	2e-129	cbv:U729_1171	K09697	natA	sodium transport system ATP-binding protein	3.6.3.7	Environmental Information Processing--Membrane transport--ABC transporters|Environmental Information Processing--Signal transduction--Two-component system

[denovogenes]_113325	73.66	2e-133	hpr:PARA_05020	K02028	ABC.PA.A	polar amino acid transport system ATP-binding protein	3.6.3.21	--

[denovogenes]_113326	68.15	1e-109	cbk:CLL_A2366	K05896	scpA	segregation and condensation protein A	--	--

[denovogenes]_113327	89.96	3e-170	cbv:U729_2634	K02036	pstB	phosphate transport system ATP-binding protein	3.6.3.27	Environmental Information Processing--Membrane transport--ABC transporters

[denovogenes]_113328	49.57	6e-81	nth:Nther_1461	K01358	clpP, CLPP	ATP-dependent Clp protease, protease subunit	3.4.21.92	Cellular Processes--Cell growth and death--Cell cycle - Caulobacter|Organismal Systems--Aging--Longevity regulating pathway - worm

[denovogenes]_113329	100.00	0.0	mmk:MU9_1783	K02393	flgH	flagellar L-ring protein precursor FlgH	--	Cellular Processes--Cell motility--Flagellar assembly

[denovogenes]_113330	100.00	0.0	mmk:MU9_2235	K16066	ydfG	3-hydroxy acid dehydrogenase / malonic semialdehyde reductase	1.1.1.381 1.1.1.-	Metabolism--Nucleotide metabolism--Pyrimidine metabolism|Metabolism--Amino acid metabolism--Glycine, serine and threonine metabolism|Metabolism--Global and overview maps--Metabolic pathways

[denovogenes]_113331	99.60	0.0	mmk:MU9_1540	K00979	kdsB	3-deoxy-manno-octulosonate cytidylyltransferase (CMP-KDO synthetase)	2.7.7.38	Metabolism--Glycan biosynthesis and metabolism--Lipopolysaccharide biosynthesis|Metabolism--Global and overview maps--Metabolic pathways

[denovogenes]_113334	100.00	0.0	mmk:MU9_2508	K09474	phoN	acid phosphatase (class A)	3.1.3.2	Metabolism--Xenobiotics biodegradation and metabolism--Aminobenzoate degradation|Metabolism--Metabolism of cofactors and vitamins--Riboflavin metabolism|Metabolism--Global and overview maps--Metabolic pathways|Metabolism--Global and overview maps--Microbial metabolism in diverse environments|Environmental Information Processing--Signal transduction--Two-component system

[denovogenes]_113336	99.20	0.0	mmk:MU9_575	K00528	fpr	ferredoxin--NADP+ reductase	1.18.1.2	--

[denovogenes]_113337	73.60	9e-133	efm:M7W_1562	K01628	fucA	L-fuculose-phosphate aldolase	4.1.2.17	Metabolism--Carbohydrate metabolism--Fructose and mannose metabolism|Metabolism--Global and overview maps--Microbial metabolism in diverse environments

[denovogenes]_113342	91.91	2e-158	lacy:A4V08_34270	K01923	purC	phosphoribosylaminoimidazole-succinocarboxamide synthase	6.3.2.6	Metabolism--Nucleotide metabolism--Purine metabolism|Metabolism--Global and overview maps--Metabolic pathways|Metabolism--Global and overview maps--Biosynthesis of secondary metabolites|Metabolism--Global and overview maps--Biosynthesis of antibiotics

[denovogenes]_113343	79.12	3e-148	csb:CLSA_c02240	K01265	map	methionyl aminopeptidase	3.4.11.18	--

[denovogenes]_113344	88.71	1e-166	cbv:U729_1515	K00989	rph	ribonuclease PH	2.7.7.56	--

[denovogenes]_113345	65.89	4e-120	lacy:A4V08_17720	K03501	gidB, rsmG	16S rRNA (guanine527-N7)-methyltransferase	2.1.1.170	--

[denovogenes]_113346	42.06	5e-57	soz:Spy49_0620	K00786	E2.4.-.-	--	--	--

[denovogenes]_11335	100.00	0.0	bfg:BF638R_3040	K04084	dsbD	thiol:disulfide interchange protein DsbD	1.8.1.8	--

[denovogenes]_113350	46.34	7e-76	tte:TTE1023	K02564	nagB, GNPDA	glucosamine-6-phosphate deaminase	3.5.99.6	Metabolism--Carbohydrate metabolism--Amino sugar and nucleotide sugar metabolism|Metabolism--Global and overview maps--Metabolic pathways

[denovogenes]_113352	99.60	3e-175	mmk:MU9_747	K07090	K07090	uncharacterized protein	--	--

[denovogenes]_113353	73.88	8e-137	lacy:A4V08_00620	K06201	cutC	copper homeostasis protein	--	--

[denovogenes]_113354	88.43	1e-153	lacy:A4V08_09990	K02028	ABC.PA.A	polar amino acid transport system ATP-binding protein	3.6.3.21	--

[denovogenes]_113355	70.00	8e-113	cbv:U729_2681	K05896	scpA	segregation and condensation protein A	--	--

[denovogenes]_113357	63.16	1e-100	csb:CLSA_c24560	K03424	--	--	--	--

[denovogenes]_113359	86.18	2e-161	ecas:ECBG_02889	K00554	trmD	tRNA (guanine37-N1)-methyltransferase	2.1.1.228	--

[denovogenes]_11336	47.35	0.0	cpe:CPE0851	K02004	ABC.CD.P	putative ABC transport system permease protein	--	--

[denovogenes]_113360	99.20	0.0	efq:DR75_1837	K20074	prpC, phpP	PPM family protein phosphatase	3.1.3.16	--

[denovogenes]_113363	89.16	5e-168	cbv:U729_2634	K02036	pstB	phosphate transport system ATP-binding protein	3.6.3.27	Environmental Information Processing--Membrane transport--ABC transporters

[denovogenes]_113364	100.00	0.0	efq:DR75_2264	K06173	truA, PUS1	tRNA pseudouridine38-40 synthase	5.4.99.12	--

[denovogenes]_113365	46.52	3e-83	ibu:IB211_00147	K01779	racD	aspartate racemase	5.1.1.13	Metabolism--Amino acid metabolism--Alanine, aspartate and glutamate metabolism|Metabolism--Metabolism of terpenoids and polyketides--Nonribosomal peptide structures

[denovogenes]_113367	100.00	0.0	stb:SGPB_0191	K00806	uppS	undecaprenyl diphosphate synthase	2.5.1.31	Metabolism--Metabolism of terpenoids and polyketides--Terpenoid backbone biosynthesis|Metabolism--Global and overview maps--Biosynthesis of secondary metabolites

[denovogenes]_11337	88.49	0.0	efm:M7W_2283	K01874	MARS, metG	methionyl-tRNA synthetase	6.1.1.10	Metabolism--Metabolism of other amino acids--Selenocompound metabolism|Genetic Information Processing--Translation--Aminoacyl-tRNA biosynthesis

[denovogenes]_113372	66.53	2e-122	lacy:A4V08_22415	K00991	ispD	2-C-methyl-D-erythritol 4-phosphate cytidylyltransferase	2.7.7.60	Metabolism--Metabolism of terpenoids and polyketides--Terpenoid backbone biosynthesis|Metabolism--Global and overview maps--Metabolic pathways|Metabolism--Global and overview maps--Biosynthesis of secondary metabolites|Metabolism--Global and overview maps--Biosynthesis of antibiotics

[denovogenes]_113375	77.91	9e-141	cpr:CPR_2526	K02528	ksgA	16S rRNA (adenine1518-N6/adenine1519-N6)-dimethyltransferase	2.1.1.182	--

[denovogenes]_113379	76.71	7e-144	cpr:CPR_2377	K01265	map	methionyl aminopeptidase	3.4.11.18	--

[denovogenes]_113380	99.20	0.0	stb:SGPB_0239	K06173	truA, PUS1	tRNA pseudouridine38-40 synthase	5.4.99.12	--

[denovogenes]_113382	100.00	0.0	smn:SMA_1866	K02036	pstB	phosphate transport system ATP-binding protein	3.6.3.27	Environmental Information Processing--Membrane transport--ABC transporters

[denovogenes]_113383	63.31	8e-117	lin:pli0072	K07497	K07497	putative transposase	--	--

[denovogenes]_113385	100.00	0.0	efi:OG1RF_10584	K03100	lepB, TPP	signal peptidase I	3.4.21.89	Cellular Processes--Cellular community - prokaryotes--Quorum sensing|Genetic Information Processing--Folding, sorting and degradation--Protein export

[denovogenes]_113386	99.60	0.0	bfb:VU15_21015	K08641	vanX	D-alanyl-D-alanine dipeptidase	3.4.13.22	Human Diseases--Drug resistance: Antimicrobial--Vancomycin resistance|Environmental Information Processing--Signal transduction--Two-component system

[denovogenes]_113387	81.38	2e-149	cdf:CD630_35470	K15460	yfiC, trmX	tRNA1Val (adenine37-N6)-methyltransferase	2.1.1.223	--

[denovogenes]_113388	67.48	3e-123	cbv:U729_32	K03584	recO	DNA repair protein RecO (recombination protein O)	--	Genetic Information Processing--Replication and repair--Homologous recombination

[denovogenes]_113391	54.51	4e-90	pch:EY04_18730	K00065	kduD	2-deoxy-D-gluconate 3-dehydrogenase	1.1.1.125	Metabolism--Carbohydrate metabolism--Pentose and glucuronate interconversions

[denovogenes]_113392	74.80	8e-133	cbv:U729_32	K03584	recO	DNA repair protein RecO (recombination protein O)	--	Genetic Information Processing--Replication and repair--Homologous recombination

[denovogenes]_113393	40.16	1e-56	llm:llmg_0412	K00784	rnz	ribonuclease Z	3.1.26.11	Genetic Information Processing--Translation--RNA transport

[denovogenes]_113394	100.00	0.0	stb:SGPB_0214	K03218	rlmB	23S rRNA (guanosine2251-2'-O)-methyltransferase	2.1.1.185	--

[denovogenes]_113395	64.59	6e-100	cle:Clole_1395	K01448	E3.5.1.28B, amiA, amiB, amiC	N-acetylmuramoyl-L-alanine amidase	3.5.1.28	Human Diseases--Drug resistance: Antimicrobial--Cationic antimicrobial peptide (CAMP) resistance

[denovogenes]_113396	42.57	5e-66	pmr:PMI0300	K12519	papD	chaperone protein PapD	--	--

[denovogenes]_113398	100.00	0.0	pvl:AOB99_13660	K00655	plsC	1-acyl-sn-glycerol-3-phosphate acyltransferase	2.3.1.51	Metabolism--Lipid metabolism--Glycerolipid metabolism|Metabolism--Lipid metabolism--Glycerophospholipid metabolism|Metabolism--Global and overview maps--Metabolic pathways|Metabolism--Global and overview maps--Biosynthesis of secondary metabolites

[denovogenes]_1134	96.62	0.0	bhl:Bache_3115	K03737	por, nifJ	pyruvate-ferredoxin/flavodoxin oxidoreductase	1.2.7.1 1.2.7.-	Metabolism--Carbohydrate metabolism--Glycolysis / Gluconeogenesis|Metabolism--Carbohydrate metabolism--Citrate cycle (TCA cycle)|Metabolism--Carbohydrate metabolism--Pyruvate metabolism|Metabolism--Carbohydrate metabolism--Butanoate metabolism|Metabolism--Energy metabolism--Carbon fixation pathways in prokaryotes|Metabolism--Global and overview maps--Metabolic pathways|Metabolism--Global and overview maps--Microbial metabolism in diverse environments|Metabolism--Global and overview maps--Biosynthesis of antibiotics|Metabolism--Global and overview maps--Carbon metabolism

[denovogenes]_11340	57.85	0.0	cld:CLSPO_c21850	K02004	ABC.CD.P	putative ABC transport system permease protein	--	--

[denovogenes]_113401	70.20	3e-124	ctc:CTC_00405	K02049	ABC.SN.A	NitT/TauT family transport system ATP-binding protein	--	--

[denovogenes]_113404	100.00	1e-180	efd:EFD32_1728	K19973	mntA	manganese/zinc transport system ATP-binding protein	3.6.3.35	Environmental Information Processing--Membrane transport--ABC transporters

[denovogenes]_113405	51.04	7e-81	cdf:CD630_35470	K15460	yfiC, trmX	tRNA1Val (adenine37-N6)-methyltransferase	2.1.1.223	--

[denovogenes]_113406	99.20	0.0	cbut:ATN24_02530	K01265	map	methionyl aminopeptidase	3.4.11.18	--

[denovogenes]_11341	86.64	0.0	lacy:A4V08_05290	K04079	HSP90A, htpG	molecular chaperone HtpG	--	Genetic Information Processing--Folding, sorting and degradation--Protein processing in endoplasmic reticulum|Environmental Information Processing--Signal transduction--PI3K-Akt signaling pathway|Organismal Systems--Immune system--Antigen processing and presentation|Organismal Systems--Immune system--NOD-like receptor signaling pathway|Organismal Systems--Environmental adaptation--Plant-pathogen interaction|Organismal Systems--Immune system--IL-17 signaling pathway|Organismal Systems--Immune system--Th17 cell differentiation|Organismal Systems--Endocrine system--Progesterone-mediated oocyte maturation|Organismal Systems--Endocrine system--Estrogen signaling pathway|Human Diseases--Cancers: Overview--Pathways in cancer|Human Diseases--Cancers: Specific types--Prostate cancer|Human Diseases--Cardiovascular diseases--Fluid shear stress and atherosclerosis

[denovogenes]_113410	44.70	2e-58	eac:EAL2_808p03000	K07496	K07496	putative transposase	--	--

[denovogenes]_113412	100.00	0.0	pvl:AOB99_07565	K15256	cmoA	tRNA (cmo5U34)-methyltransferase	2.1.1.-	--

[denovogenes]_113414	100.00	0.0	pvl:AOB99_15150	K03148	thiF	sulfur carrier protein ThiS adenylyltransferase	2.7.7.73	Metabolism--Metabolism of cofactors and vitamins--Thiamine metabolism|Metabolism--Global and overview maps--Metabolic pathways|Genetic Information Processing--Folding, sorting and degradation--Sulfur relay system

[denovogenes]_113415	99.20	0.0	cfd:CFNIH1_23170	K00647	fabB	3-oxoacyl-[acyl-carrier-protein] synthase I	2.3.1.41	Metabolism--Lipid metabolism--Fatty acid biosynthesis|Metabolism--Metabolism of cofactors and vitamins--Biotin metabolism|Metabolism--Global and overview maps--Metabolic pathways|Metabolism--Global and overview maps--Fatty acid metabolism

[denovogenes]_113417	48.19	4e-72	ctyk:CTK_C25580	K02233	E2.7.8.26, cobS, cobV	adenosylcobinamide-GDP ribazoletransferase	2.7.8.26	Metabolism--Metabolism of cofactors and vitamins--Porphyrin and chlorophyll metabolism|Metabolism--Global and overview maps--Metabolic pathways

[denovogenes]_113418	82.33	1e-149	cbj:H04402_03523	K00175	korB, oorB, oforB	2-oxoglutarate/2-oxoacid ferredoxin oxidoreductase subunit beta	1.2.7.3 1.2.7.11	Metabolism--Carbohydrate metabolism--Glycolysis / Gluconeogenesis|Metabolism--Carbohydrate metabolism--Citrate cycle (TCA cycle)|Metabolism--Carbohydrate metabolism--Pyruvate metabolism|Metabolism--Carbohydrate metabolism--Butanoate metabolism|Metabolism--Energy metabolism--Carbon fixation pathways in prokaryotes|Metabolism--Global and overview maps--Metabolic pathways|Metabolism--Global and overview maps--Microbial metabolism in diverse environments|Metabolism--Global and overview maps--Biosynthesis of antibiotics|Metabolism--Global and overview maps--Carbon metabolism

[denovogenes]_113419	42.46	4e-41	sdz:Asd1617_02960	K18326	mdtD	MFS transporter, DHA2 family, multidrug resistance protein	--	Environmental Information Processing--Signal transduction--Two-component system

[denovogenes]_11342	47.20	0.0	cpe:CPE0851	K02004	ABC.CD.P	putative ABC transport system permease protein	--	--

[denovogenes]_113421	82.11	1e-151	cbv:U729_1515	K00989	rph	ribonuclease PH	2.7.7.56	--

[denovogenes]_113424	100.00	2e-180	pvl:AOB99_04140	K12538	hasF, prtF	outer membrane protein, protease secretion system	--	--

[denovogenes]_113425	62.35	7e-106	xbv:XBW1_mp0151	K00558	DNMT1, dcm	DNA (cytosine-5)-methyltransferase 1	2.1.1.37	Metabolism--Amino acid metabolism--Cysteine and methionine metabolism|Metabolism--Global and overview maps--Metabolic pathways|Human Diseases--Cancers: Overview--MicroRNAs in cancer

[denovogenes]_113426	100.00	1e-174	ene:ENT_27230	K17466	PTS-Dga-EIIC, dgaC	PTS system, D-glucosaminate-specific IIC component	--	Metabolism--Carbohydrate metabolism--Pentose phosphate pathway|Metabolism--Global and overview maps--Microbial metabolism in diverse environments|Environmental Information Processing--Membrane transport--Phosphotransferase system (PTS)

[denovogenes]_113427	40.33	8e-60	vvl:VV93_v1c44590	K13637	uxuR	GntR family transcriptional regulator, uxu operon transcriptional repressor	--	--

[denovogenes]_113429	96.79	3e-176	cfd:CFNIH1_16660	K06074	ABC.VB12.A, btuD	vitamin B12 transport system ATP-binding protein	3.6.3.33	Environmental Information Processing--Membrane transport--ABC transporters

[denovogenes]_113430	91.09	2e-167	cfd:CFNIH1_03960	K00757	udp, UPP	uridine phosphorylase	2.4.2.3	Metabolism--Nucleotide metabolism--Pyrimidine metabolism|Metabolism--Xenobiotics biodegradation and metabolism--Drug metabolism - other enzymes|Metabolism--Global and overview maps--Metabolic pathways

[denovogenes]_113431	45.64	5e-60	pdf:CD630DERM_10000	K02050	ABC.SN.P	NitT/TauT family transport system permease protein	--	--

[denovogenes]_113432	74.40	3e-135	stm:STM1353	K03522	fixB, etfA	electron transfer flavoprotein alpha subunit	--	--

[denovogenes]_113435	58.87	2e-97	cle:Clole_1331	K07282	pgsA, capA	poly-gamma-glutamate synthesis protein (capsule biosynthesis protein)	--	--

[denovogenes]_113436	92.14	4e-150	efe:EFER_1190	K07772	torR	two-component system, OmpR family, torCAD operon response regulator TorR	--	Environmental Information Processing--Signal transduction--Two-component system

[denovogenes]_113437	41.06	9e-36	pse:NH8B_4087	K00874	kdgK	2-dehydro-3-deoxygluconokinase	2.7.1.45	Metabolism--Carbohydrate metabolism--Pentose phosphate pathway|Metabolism--Global and overview maps--Metabolic pathways|Metabolism--Global and overview maps--Microbial metabolism in diverse environments|Metabolism--Global and overview maps--Carbon metabolism

[denovogenes]_11344	92.23	0.0	bcel:BcellWH2_00937	K00615	E2.2.1.1, tktA, tktB	transketolase	2.2.1.1	Metabolism--Carbohydrate metabolism--Pentose phosphate pathway|Metabolism--Energy metabolism--Carbon fixation in photosynthetic organisms|Metabolism--Metabolism of terpenoids and polyketides--Biosynthesis of ansamycins|Metabolism--Global and overview maps--Metabolic pathways|Metabolism--Global and overview maps--Biosynthesis of secondary metabolites|Metabolism--Global and overview maps--Microbial metabolism in diverse environments|Metabolism--Global and overview maps--Biosynthesis of antibiotics|Metabolism--Global and overview maps--Carbon metabolism|Metabolism--Global and overview maps--Biosynthesis of amino acids

[denovogenes]_113440	99.20	1e-176	cbut:ATN24_06920	K02030	ABC.PA.S	polar amino acid transport system substrate-binding protein	--	--

[denovogenes]_113441	60.08	1e-110	str:Sterm_3865	K17463	dgaF	2-dehydro-3-deoxy-phosphogluconate aldolase	4.1.2.14	Metabolism--Carbohydrate metabolism--Pentose phosphate pathway|Metabolism--Global and overview maps--Metabolic pathways|Metabolism--Global and overview maps--Microbial metabolism in diverse environments

[denovogenes]_113442	54.55	2e-40	mgo:AFA91_32570	K19062	arr	rifampin ADP-ribosylating transferase	--	--

[denovogenes]_113443	97.59	9e-173	cif:AL515_03345	K03759	adiC	arginine:agmatine antiporter	--	--

[denovogenes]_113444	80.57	4e-143	cpe:CPE0611	K02837	prfC	peptide chain release factor 3	--	--

[denovogenes]_113447	100.00	0.0	pvl:AOB99_07260	K06074	ABC.VB12.A, btuD	vitamin B12 transport system ATP-binding protein	3.6.3.33	Environmental Information Processing--Membrane transport--ABC transporters

[denovogenes]_11345	84.24	0.0	bhl:Bache_0071	K03654	recQ	ATP-dependent DNA helicase RecQ	3.6.4.12	Genetic Information Processing--Folding, sorting and degradation--RNA degradation

[denovogenes]_113450	63.11	4e-101	cbk:CLL_A3505	K03101	lspA	signal peptidase II	3.4.23.36	Genetic Information Processing--Folding, sorting and degradation--Protein export

[denovogenes]_113451	54.15	6e-83	cbk:CLL_A2893	K04486	E3.1.3.15B	histidinol-phosphatase (PHP family)	3.1.3.15	Metabolism--Amino acid metabolism--Histidine metabolism|Metabolism--Global and overview maps--Metabolic pathways|Metabolism--Global and overview maps--Biosynthesis of secondary metabolites|Metabolism--Global and overview maps--Biosynthesis of amino acids

[denovogenes]_113452	100.00	0.0	elo:EC042_2024	K09815	znuA	zinc transport system substrate-binding protein	--	Environmental Information Processing--Membrane transport--ABC transporters

[denovogenes]_113453	54.36	5e-82	lacy:A4V08_31385	K18122	cat2, abfT	4-hydroxybutyrate CoA-transferase	2.8.3.-	Metabolism--Carbohydrate metabolism--Butanoate metabolism|Metabolism--Global and overview maps--Metabolic pathways|Metabolism--Global and overview maps--Carbon metabolism

[denovogenes]_113455	47.58	2e-29	mdh:AYM39_07060	K00992	murU	MurNAc alpha-1-phosphate uridylyltransferase	2.7.7.-	Metabolism--Carbohydrate metabolism--Amino sugar and nucleotide sugar metabolism|Metabolism--Global and overview maps--Metabolic pathways

[denovogenes]_113456	63.93	8e-98	bcef:BcrFT9_04138	K03702	uvrB	excinuclease ABC subunit B	--	Genetic Information Processing--Replication and repair--Nucleotide excision repair

[denovogenes]_113458	46.91	4e-66	btl:BALH_3930	K07497	K07497	putative transposase	--	--

[denovogenes]_113461	99.20	5e-177	cbut:ATN24_07735	K14441	rimO	ribosomal protein S12 methylthiotransferase	2.8.4.4	--

[denovogenes]_113462	100.00	0.0	pvl:AOB99_12830	K00892	gsk	inosine kinase	2.7.1.73	Metabolism--Nucleotide metabolism--Purine metabolism

[denovogenes]_113463	52.24	2e-85	cdf:CD630_35470	K15460	yfiC, trmX	tRNA1Val (adenine37-N6)-methyltransferase	2.1.1.223	--

[denovogenes]_113464	94.38	1e-168	cif:AL515_19290	K02669	pilT	twitching motility protein PilT	--	--

[denovogenes]_113465	42.15	6e-47	scap:AYP1020_1025	K01261	pepA	glutamyl aminopeptidase	3.4.11.7	--

[denovogenes]_113468	42.68	8e-57	lhk:LHK_00428	K03294	TC.APA	basic amino acid/polyamine antiporter, APA family	--	--

[denovogenes]_113470	100.00	0.0	ecw:EcE24377A_2658	K18702	uctC	CoA:oxalate CoA-transferase	2.8.3.19	--

[denovogenes]_113471	96.39	5e-178	cbut:ATN24_18830	K07053	E3.1.3.97	3',5'-nucleoside bisphosphate phosphatase	3.1.3.97	--

[denovogenes]_113472	100.00	0.0	bvs:BARVI_12255	K00561	ermC, ermA	23S rRNA (adenine-N6)-dimethyltransferase	2.1.1.184	--

[denovogenes]_113474	40.76	1e-57	sph:MGAS10270_Spy1168	K01091	gph	phosphoglycolate phosphatase	3.1.3.18	Metabolism--Carbohydrate metabolism--Glyoxylate and dicarboxylate metabolism|Metabolism--Global and overview maps--Metabolic pathways|Metabolism--Global and overview maps--Biosynthesis of secondary metabolites|Metabolism--Global and overview maps--Biosynthesis of antibiotics

[denovogenes]_113475	97.59	9e-173	cif:AL515_03345	K03759	adiC	arginine:agmatine antiporter	--	--

[denovogenes]_113477	77.91	2e-140	cle:Clole_2394	K18332	hndD	NADP-reducing hydrogenase subunit HndD	1.12.1.3	--

[denovogenes]_113478	68.78	2e-106	mhg:MHY_06720	K01214	ISA, treX	isoamylase	3.2.1.68	Metabolism--Carbohydrate metabolism--Starch and sucrose metabolism|Metabolism--Global and overview maps--Metabolic pathways|Metabolism--Global and overview maps--Biosynthesis of secondary metabolites

[denovogenes]_113479	44.00	4e-61	vhl:BME96_14375	K03312	gltS	glutamate:Na+ symporter, ESS family	--	--

[denovogenes]_11348	70.75	0.0	lacy:A4V08_15835	K02755	PTS-Bgl-EIIA, bglF, bglP	PTS system, beta-glucoside-specific IIA component	2.7.1.-	Environmental Information Processing--Membrane transport--Phosphotransferase system (PTS)

[denovogenes]_11348	70.75	0.0	lacy:A4V08_15835	K02756	PTS-Bgl-EIIB, bglF, bglP	PTS system, beta-glucoside-specific IIB component	2.7.1.-	Environmental Information Processing--Membrane transport--Phosphotransferase system (PTS)

[denovogenes]_11348	70.75	0.0	lacy:A4V08_15835	K02757	PTS-Bgl-EIIC, bglF, bglP	PTS system, beta-glucoside-specific IIC component	--	Environmental Information Processing--Membrane transport--Phosphotransferase system (PTS)

[denovogenes]_113480	65.41	4e-72	cle:Clole_2519	K07452	mcrB	5-methylcytosine-specific restriction enzyme B	3.1.21.-	--

[denovogenes]_113481	74.80	2e-139	cbt:CLH_0458	K06436	yabG	spore coat assemly protein	--	--

[denovogenes]_113482	44.44	1e-56	fhw:RN87_06240	K15777	DOPA	4,5-DOPA dioxygenase extradiol	1.13.11.-	Metabolism--Biosynthesis of other secondary metabolites--Betalain biosynthesis

[denovogenes]_113489	70.04	8e-127	cbt:CLH_2091	K07258	dacC, dacA, dacD	D-alanyl-D-alanine carboxypeptidase (penicillin-binding protein 5/6)	3.4.16.4	Metabolism--Glycan biosynthesis and metabolism--Peptidoglycan biosynthesis|Metabolism--Global and overview maps--Metabolic pathways

[denovogenes]_11349	89.37	0.0	bth:BT_0683	K01187	malZ	alpha-glucosidase	3.2.1.20	Metabolism--Carbohydrate metabolism--Galactose metabolism|Metabolism--Carbohydrate metabolism--Starch and sucrose metabolism|Metabolism--Global and overview maps--Metabolic pathways

[denovogenes]_113492	88.40	2e-161	csb:CLSA_c28640	K01876	DARS, aspS	aspartyl-tRNA synthetase	6.1.1.12	Genetic Information Processing--Translation--Aminoacyl-tRNA biosynthesis

[denovogenes]_113494	54.98	1e-66	cad:Curi_c10470	K07192	FLOT	flotillin	--	Organismal Systems--Endocrine system--Insulin signaling pathway

[denovogenes]_113495	67.60	5e-125	cbz:Cbs_4909	K00700	GBE1, glgB	1,4-alpha-glucan branching enzyme	2.4.1.18	Metabolism--Carbohydrate metabolism--Starch and sucrose metabolism|Metabolism--Global and overview maps--Metabolic pathways|Metabolism--Global and overview maps--Biosynthesis of secondary metabolites

[denovogenes]_113498	98.34	6e-177	ecoo:ECRM13514_2902	K05541	dusC	tRNA-dihydrouridine synthase C	1.-.-.-	--

[denovogenes]_113499	94.80	3e-174	efa:EF1456	K09961	K09961	uncharacterized protein	--	--

[denovogenes]_1135	99.92	0.0	mmk:MU9_1616	K03737	por, nifJ	pyruvate-ferredoxin/flavodoxin oxidoreductase	1.2.7.1 1.2.7.-	Metabolism--Carbohydrate metabolism--Glycolysis / Gluconeogenesis|Metabolism--Carbohydrate metabolism--Citrate cycle (TCA cycle)|Metabolism--Carbohydrate metabolism--Pyruvate metabolism|Metabolism--Carbohydrate metabolism--Butanoate metabolism|Metabolism--Energy metabolism--Carbon fixation pathways in prokaryotes|Metabolism--Global and overview maps--Metabolic pathways|Metabolism--Global and overview maps--Microbial metabolism in diverse environments|Metabolism--Global and overview maps--Biosynthesis of antibiotics|Metabolism--Global and overview maps--Carbon metabolism

[denovogenes]_113501	69.60	4e-126	ssu:SSU05_1024	K03702	uvrB	excinuclease ABC subunit B	--	Genetic Information Processing--Replication and repair--Nucleotide excision repair

[denovogenes]_113502	86.00	6e-149	prg:RB151_036630	K04655	hypE	hydrogenase expression/formation protein HypE	--	--

[denovogenes]_113505	62.50	4e-120	cpe:CPE0757	K01470	E3.5.2.10	creatinine amidohydrolase	3.5.2.10	Metabolism--Amino acid metabolism--Arginine and proline metabolism

[denovogenes]_113507	59.02	1e-100	rto:RTO_29420	K06173	truA, PUS1	tRNA pseudouridine38-40 synthase	5.4.99.12	--

[denovogenes]_113508	86.64	4e-163	byl:A4V09_21245	K02036	pstB	phosphate transport system ATP-binding protein	3.6.3.27	Environmental Information Processing--Membrane transport--ABC transporters

[denovogenes]_113511	62.70	1e-109	ctx:Clo1313_0670	K07238	TC.ZIP, zupT, ZRT3, ZIP2	zinc transporter, ZIP family	--	--

[denovogenes]_113512	81.82	4e-141	bcel:BcellWH2_01444	K02564	nagB, GNPDA	glucosamine-6-phosphate deaminase	3.5.99.6	Metabolism--Carbohydrate metabolism--Amino sugar and nucleotide sugar metabolism|Metabolism--Global and overview maps--Metabolic pathways

[denovogenes]_113513	64.92	3e-116	pft:JBW_01890	K03595	era	GTPase	--	--

[denovogenes]_113514	62.86	2e-115	rob:CK5_19300	K06173	truA, PUS1	tRNA pseudouridine38-40 synthase	5.4.99.12	--

[denovogenes]_113515	99.57	1e-171	boa:Bovatus_03029	K12410	npdA	NAD-dependent deacetylase	3.5.1.-	--

[denovogenes]_113523	95.53	1e-175	bhl:Bache_1892	K03218	rlmB	23S rRNA (guanosine2251-2'-O)-methyltransferase	2.1.1.185	--

[denovogenes]_113526	45.27	1e-52	csh:Closa_3467	K02411	fliH	flagellar assembly protein FliH	--	Cellular Processes--Cell motility--Flagellar assembly

[denovogenes]_113527	43.63	3e-51	ruk:A4V00_15035	K02392	flgG	flagellar basal-body rod protein FlgG	--	Cellular Processes--Cell motility--Flagellar assembly

[denovogenes]_113529	46.77	1e-28	mdh:AYM39_07060	K00992	murU	MurNAc alpha-1-phosphate uridylyltransferase	2.7.7.-	Metabolism--Carbohydrate metabolism--Amino sugar and nucleotide sugar metabolism|Metabolism--Global and overview maps--Metabolic pathways

[denovogenes]_113530	89.56	2e-164	lacy:A4V08_29120	K01803	TPI, tpiA	triosephosphate isomerase (TIM)	5.3.1.1	Metabolism--Carbohydrate metabolism--Glycolysis / Gluconeogenesis|Metabolism--Carbohydrate metabolism--Fructose and mannose metabolism|Metabolism--Carbohydrate metabolism--Inositol phosphate metabolism|Metabolism--Energy metabolism--Carbon fixation in photosynthetic organisms|Metabolism--Global and overview maps--Metabolic pathways|Metabolism--Global and overview maps--Biosynthesis of secondary metabolites|Metabolism--Global and overview maps--Microbial metabolism in diverse environments|Metabolism--Global and overview maps--Biosynthesis of antibiotics|Metabolism--Global and overview maps--Carbon metabolism|Metabolism--Global and overview maps--Biosynthesis of amino acids

[denovogenes]_113532	65.47	4e-102	fpla:A4U99_04320	K02003	ABC.CD.A	putative ABC transport system ATP-binding protein	--	--

[denovogenes]_113534	61.13	1e-98	bprm:CL3_25720	K06183	rsuA	16S rRNA pseudouridine516 synthase	5.4.99.19	--

[denovogenes]_113536	54.13	3e-85	rto:RTO_29420	K06173	truA, PUS1	tRNA pseudouridine38-40 synthase	5.4.99.12	--

[denovogenes]_113537	93.17	5e-177	boa:Bovatus_04509	K06173	truA, PUS1	tRNA pseudouridine38-40 synthase	5.4.99.12	--

[denovogenes]_113538	100.00	0.0	bvu:BVU_3149	K02233	E2.7.8.26, cobS, cobV	adenosylcobinamide-GDP ribazoletransferase	2.7.8.26	Metabolism--Metabolism of cofactors and vitamins--Porphyrin and chlorophyll metabolism|Metabolism--Global and overview maps--Metabolic pathways

[denovogenes]_113539	62.80	5e-102	fpla:A4U99_10160	K02823	pyrDII	dihydroorotate dehydrogenase electron transfer subunit	--	Metabolism--Nucleotide metabolism--Pyrimidine metabolism|Metabolism--Global and overview maps--Metabolic pathways

[denovogenes]_11354	99.85	0.0	bvu:BVU_2933	K03628	rho	transcription termination factor Rho	--	Genetic Information Processing--Folding, sorting and degradation--RNA degradation

[denovogenes]_113540	89.16	6e-165	bprs:CK3_33080	K01803	TPI, tpiA	triosephosphate isomerase (TIM)	5.3.1.1	Metabolism--Carbohydrate metabolism--Glycolysis / Gluconeogenesis|Metabolism--Carbohydrate metabolism--Fructose and mannose metabolism|Metabolism--Carbohydrate metabolism--Inositol phosphate metabolism|Metabolism--Energy metabolism--Carbon fixation in photosynthetic organisms|Metabolism--Global and overview maps--Metabolic pathways|Metabolism--Global and overview maps--Biosynthesis of secondary metabolites|Metabolism--Global and overview maps--Microbial metabolism in diverse environments|Metabolism--Global and overview maps--Biosynthesis of antibiotics|Metabolism--Global and overview maps--Carbon metabolism|Metabolism--Global and overview maps--Biosynthesis of amino acids

[denovogenes]_113541	100.00	0.0	bxy:BXY_15830	K01991	ABC-2.OM, wza	polysaccharide biosynthesis/export protein	--	Cellular Processes--Cellular community - prokaryotes--Biofilm formation - Escherichia coli

[denovogenes]_113545	47.17	2e-23	cdf:CD630_09370	K07741	antB	anti-repressor protein	--	--

[denovogenes]_113547	71.05	4e-108	fpla:A4U99_09570	K05845	opuC	osmoprotectant transport system substrate-binding protein	--	Environmental Information Processing--Membrane transport--ABC transporters

[denovogenes]_113547	71.05	4e-108	fpla:A4U99_09570	K05846	opuBD	osmoprotectant transport system permease protein	--	Environmental Information Processing--Membrane transport--ABC transporters

[denovogenes]_113548	47.39	2e-83	ccel:CCDG5_1265	K07284	srtA	sortase A	3.4.22.70	--

[denovogenes]_113549	100.00	0.0	bvu:BVU_2259	K00721	DPM1	dolichol-phosphate mannosyltransferase	2.4.1.83	Metabolism--Glycan biosynthesis and metabolism--N-Glycan biosynthesis|Metabolism--Global and overview maps--Metabolic pathways

[denovogenes]_11355	99.85	0.0	fpla:A4U99_01295	K02123	ATPVI, ntpI, atpI	V/A-type H+/Na+-transporting ATPase subunit I	--	Metabolism--Energy metabolism--Oxidative phosphorylation|Metabolism--Global and overview maps--Metabolic pathways

[denovogenes]_113551	89.29	7e-164	lacy:A4V08_09990	K02028	ABC.PA.A	polar amino acid transport system ATP-binding protein	3.6.3.21	--

[denovogenes]_113553	71.60	6e-130	lacy:A4V08_23695	K00784	rnz	ribonuclease Z	3.1.26.11	Genetic Information Processing--Translation--RNA transport

[denovogenes]_113554	59.44	1e-108	bfg:BF638R_2998	K03100	lepB, TPP	signal peptidase I	3.4.21.89	Cellular Processes--Cellular community - prokaryotes--Quorum sensing|Genetic Information Processing--Folding, sorting and degradation--Protein export

[denovogenes]_113555	63.01	7e-98	esu:EUS_02890	K03205	virD4, lvhD4	type IV secretion system protein VirD4	--	Environmental Information Processing--Membrane transport--Bacterial secretion system

[denovogenes]_113556	79.92	1e-146	csh:Closa_3089	K02028	ABC.PA.A	polar amino acid transport system ATP-binding protein	3.6.3.21	--

[denovogenes]_113557	100.00	0.0	btho:Btheta7330_01301	K07257	spsF	spore coat polysaccharide biosynthesis protein SpsF	--	--

[denovogenes]_11356	47.14	0.0	dvl:Dvul_0060	K01533	copB	Cu2+-exporting ATPase	3.6.3.4	--

[denovogenes]_113561	45.49	2e-62	eha:Ethha_1193	K03584	recO	DNA repair protein RecO (recombination protein O)	--	Genetic Information Processing--Replication and repair--Homologous recombination

[denovogenes]_113563	46.44	3e-75	ert:EUR_15210	K03496	parA, soj	chromosome partitioning protein	--	--

[denovogenes]_113564	41.74	2e-09	scw:TU94_13735	K00598	tam	trans-aconitate 2-methyltransferase	2.1.1.144	--

[denovogenes]_113566	71.67	1e-125	cad:Curi_c12480	K02028	ABC.PA.A	polar amino acid transport system ATP-binding protein	3.6.3.21	--

[denovogenes]_113569	70.71	4e-106	fpla:A4U99_12140	K03439	trmB, METTL1	tRNA (guanine-N7-)-methyltransferase	2.1.1.33	--

[denovogenes]_11357	85.76	0.0	lacy:A4V08_01740	K04041	fbp3	fructose-1,6-bisphosphatase III	3.1.3.11	Metabolism--Carbohydrate metabolism--Glycolysis / Gluconeogenesis|Metabolism--Carbohydrate metabolism--Pentose phosphate pathway|Metabolism--Carbohydrate metabolism--Fructose and mannose metabolism|Metabolism--Energy metabolism--Methane metabolism|Metabolism--Energy metabolism--Carbon fixation in photosynthetic organisms|Metabolism--Global and overview maps--Metabolic pathways|Metabolism--Global and overview maps--Biosynthesis of secondary metabolites|Metabolism--Global and overview maps--Microbial metabolism in diverse environments|Metabolism--Global and overview maps--Biosynthesis of antibiotics|Metabolism--Global and overview maps--Carbon metabolism

[denovogenes]_113571	61.22	5e-95	ibu:IB211_00027c	K03218	rlmB	23S rRNA (guanosine2251-2'-O)-methyltransferase	2.1.1.185	--

[denovogenes]_113573	70.97	2e-133	bprs:CK3_00480	K01142	E3.1.11.2, xthA	exodeoxyribonuclease III	3.1.11.2	Genetic Information Processing--Replication and repair--Base excision repair

[denovogenes]_113574	100.00	0.0	sdz:Asd1617_02425	K06074	ABC.VB12.A, btuD	vitamin B12 transport system ATP-binding protein	3.6.3.33	Environmental Information Processing--Membrane transport--ABC transporters

[denovogenes]_113577	40.41	2e-49	xdo:XDD1_2503	K00059	fabG	3-oxoacyl-[acyl-carrier protein] reductase	1.1.1.100	Metabolism--Lipid metabolism--Fatty acid biosynthesis|Metabolism--Metabolism of cofactors and vitamins--Biotin metabolism|Metabolism--Lipid metabolism--Biosynthesis of unsaturated fatty acids|Metabolism--Global and overview maps--Metabolic pathways|Metabolism--Global and overview maps--Fatty acid metabolism

[denovogenes]_113578	81.67	5e-146	lacy:A4V08_19730	K16511	mecA1_2	adapter protein MecA 1/2	--	--

[denovogenes]_113579	89.92	2e-143	lacy:A4V08_20645	K07090	K07090	uncharacterized protein	--	--

[denovogenes]_11358	88.51	0.0	lacy:A4V08_04755	K01681	ACO, acnA	aconitate hydratase	4.2.1.3	Metabolism--Carbohydrate metabolism--Citrate cycle (TCA cycle)|Metabolism--Carbohydrate metabolism--Glyoxylate and dicarboxylate metabolism|Metabolism--Energy metabolism--Carbon fixation pathways in prokaryotes|Metabolism--Global and overview maps--Metabolic pathways|Metabolism--Global and overview maps--Biosynthesis of secondary metabolites|Metabolism--Global and overview maps--Microbial metabolism in diverse environments|Metabolism--Global and overview maps--Biosynthesis of antibiotics|Metabolism--Global and overview maps--Carbon metabolism|Metabolism--Global and overview maps--2-Oxocarboxylic acid metabolism|Metabolism--Global and overview maps--Biosynthesis of amino acids

[denovogenes]_113580	97.99	2e-176	cif:AL515_00115	K15984	rsmJ	16S rRNA (guanine1516-N2)-methyltransferase	2.1.1.242	--

[denovogenes]_113582	44.31	8e-68	vhr:AL538_17565	K01243	mtnN, mtn, pfs	adenosylhomocysteine nucleosidase	3.2.2.9	Metabolism--Amino acid metabolism--Cysteine and methionine metabolism|Metabolism--Global and overview maps--Metabolic pathways|Metabolism--Global and overview maps--Biosynthesis of amino acids

[denovogenes]_113583	56.54	1e-90	cpr:CPR_0422	K03439	trmB, METTL1	tRNA (guanine-N7-)-methyltransferase	2.1.1.33	--

[denovogenes]_113584	41.40	1e-26	aex:Astex_0081	K00992	murU	MurNAc alpha-1-phosphate uridylyltransferase	2.7.7.-	Metabolism--Carbohydrate metabolism--Amino sugar and nucleotide sugar metabolism|Metabolism--Global and overview maps--Metabolic pathways

[denovogenes]_113585	96.15	2e-167	lacy:A4V08_30880	K01628	fucA	L-fuculose-phosphate aldolase	4.1.2.17	Metabolism--Carbohydrate metabolism--Fructose and mannose metabolism|Metabolism--Global and overview maps--Microbial metabolism in diverse environments

[denovogenes]_113586	88.31	2e-162	cso:CLS_28920	K01803	TPI, tpiA	triosephosphate isomerase (TIM)	5.3.1.1	Metabolism--Carbohydrate metabolism--Glycolysis / Gluconeogenesis|Metabolism--Carbohydrate metabolism--Fructose and mannose metabolism|Metabolism--Carbohydrate metabolism--Inositol phosphate metabolism|Metabolism--Energy metabolism--Carbon fixation in photosynthetic organisms|Metabolism--Global and overview maps--Metabolic pathways|Metabolism--Global and overview maps--Biosynthesis of secondary metabolites|Metabolism--Global and overview maps--Microbial metabolism in diverse environments|Metabolism--Global and overview maps--Biosynthesis of antibiotics|Metabolism--Global and overview maps--Carbon metabolism|Metabolism--Global and overview maps--Biosynthesis of amino acids

[denovogenes]_113588	85.94	5e-164	bvu:BVU_2546	K06173	truA, PUS1	tRNA pseudouridine38-40 synthase	5.4.99.12	--

[denovogenes]_113589	57.94	2e-96	rus:RBI_II00118	K14441	rimO	ribosomal protein S12 methylthiotransferase	2.8.4.4	--

[denovogenes]_11359	67.81	0.0	ral:Rumal_1340	K01841	pepM	phosphoenolpyruvate phosphomutase	5.4.2.9	Metabolism--Metabolism of other amino acids--Phosphonate and phosphinate metabolism|Metabolism--Global and overview maps--Metabolic pathways|Metabolism--Global and overview maps--Microbial metabolism in diverse environments|Metabolism--Global and overview maps--Biosynthesis of antibiotics

[denovogenes]_113591	47.30	1e-68	gur:Gura_1986	K07665	cusR, copR, silR	two-component system, OmpR family, copper resistance phosphate regulon response regulator CusR	--	Environmental Information Processing--Signal transduction--Two-component system

[denovogenes]_113593	64.40	3e-114	hsd:SD1D_0863	K04562	flhG, fleN	flagellar biosynthesis protein FlhG	--	--

[denovogenes]_113596	53.41	4e-98	tjr:TherJR_1677	K00175	korB, oorB, oforB	2-oxoglutarate/2-oxoacid ferredoxin oxidoreductase subunit beta	1.2.7.3 1.2.7.11	Metabolism--Carbohydrate metabolism--Glycolysis / Gluconeogenesis|Metabolism--Carbohydrate metabolism--Citrate cycle (TCA cycle)|Metabolism--Carbohydrate metabolism--Pyruvate metabolism|Metabolism--Carbohydrate metabolism--Butanoate metabolism|Metabolism--Energy metabolism--Carbon fixation pathways in prokaryotes|Metabolism--Global and overview maps--Metabolic pathways|Metabolism--Global and overview maps--Microbial metabolism in diverse environments|Metabolism--Global and overview maps--Biosynthesis of antibiotics|Metabolism--Global and overview maps--Carbon metabolism

[denovogenes]_113597	79.52	3e-144	lacy:A4V08_09235	K09797	K09797	uncharacterized protein	--	--

[denovogenes]_113598	40.31	3e-39	jeu:BJP62_07415	K02746	PTS-Aga-EIIC, agaW	PTS system, N-acetylgalactosamine-specific IIC component	--	Metabolism--Carbohydrate metabolism--Galactose metabolism|Environmental Information Processing--Membrane transport--Phosphotransferase system (PTS)

[denovogenes]_1136	100.00	0.0	bfb:VU15_14880	K03737	por, nifJ	pyruvate-ferredoxin/flavodoxin oxidoreductase	1.2.7.1 1.2.7.-	Metabolism--Carbohydrate metabolism--Glycolysis / Gluconeogenesis|Metabolism--Carbohydrate metabolism--Citrate cycle (TCA cycle)|Metabolism--Carbohydrate metabolism--Pyruvate metabolism|Metabolism--Carbohydrate metabolism--Butanoate metabolism|Metabolism--Energy metabolism--Carbon fixation pathways in prokaryotes|Metabolism--Global and overview maps--Metabolic pathways|Metabolism--Global and overview maps--Microbial metabolism in diverse environments|Metabolism--Global and overview maps--Biosynthesis of antibiotics|Metabolism--Global and overview maps--Carbon metabolism

[denovogenes]_11360	40.69	1e-100	csh:Closa_2441	K03406	mcp	methyl-accepting chemotaxis protein	--	Environmental Information Processing--Signal transduction--Two-component system|Cellular Processes--Cell motility--Bacterial chemotaxis

[denovogenes]_113600	54.69	1e-84	mvd:AWU67_04915	K06971	K06971	uncharacterized protein	--	--

[denovogenes]_113601	53.25	6e-85	gfe:Gferi_22335	K00065	kduD	2-deoxy-D-gluconate 3-dehydrogenase	1.1.1.125	Metabolism--Carbohydrate metabolism--Pentose and glucuronate interconversions

[denovogenes]_113602	99.60	0.0	bdh:GV66_05680	K01719	hemD, UROS	uroporphyrinogen-III synthase	4.2.1.75	Metabolism--Metabolism of cofactors and vitamins--Porphyrin and chlorophyll metabolism|Metabolism--Global and overview maps--Metabolic pathways|Metabolism--Global and overview maps--Biosynthesis of secondary metabolites

[denovogenes]_113604	46.11	2e-42	det:DET1328	K09153	K09153	uncharacterized protein	--	--

[denovogenes]_113605	79.52	3e-147	bvu:BVU_3149	K02233	E2.7.8.26, cobS, cobV	adenosylcobinamide-GDP ribazoletransferase	2.7.8.26	Metabolism--Metabolism of cofactors and vitamins--Porphyrin and chlorophyll metabolism|Metabolism--Global and overview maps--Metabolic pathways

[denovogenes]_113606	59.35	8e-103	ibu:IB211_01472c	K01448	E3.5.1.28B, amiA, amiB, amiC	N-acetylmuramoyl-L-alanine amidase	3.5.1.28	Human Diseases--Drug resistance: Antimicrobial--Cationic antimicrobial peptide (CAMP) resistance

[denovogenes]_113609	94.78	3e-176	cfd:CFNIH1_08580	K06889	K06889	uncharacterized protein	--	--

[denovogenes]_113610	65.20	4e-125	sbr:SY1_03880	K15581	oppB	oligopeptide transport system permease protein	--	Human Diseases--Drug resistance: Antimicrobial--beta-Lactam resistance|Environmental Information Processing--Membrane transport--ABC transporters|Cellular Processes--Cellular community - prokaryotes--Quorum sensing

[denovogenes]_113613	76.61	2e-145	rho:RHOM_00390	K02036	pstB	phosphate transport system ATP-binding protein	3.6.3.27	Environmental Information Processing--Membrane transport--ABC transporters

[denovogenes]_113615	100.00	0.0	pdi:BDI_2598	K07322	ytfE, scdA	regulator of cell morphogenesis and NO signaling	--	--

[denovogenes]_113616	79.20	1e-141	ibu:IB211_02360c	K03736	eutC	ethanolamine ammonia-lyase small subunit	4.3.1.7	Metabolism--Lipid metabolism--Glycerophospholipid metabolism|Metabolism--Global and overview maps--Metabolic pathways

[denovogenes]_113617	54.92	4e-100	ccel:CCDG5_1648	K00806	uppS	undecaprenyl diphosphate synthase	2.5.1.31	Metabolism--Metabolism of terpenoids and polyketides--Terpenoid backbone biosynthesis|Metabolism--Global and overview maps--Biosynthesis of secondary metabolites

[denovogenes]_113619	98.39	3e-172	fpla:A4U99_15210	K20460	nukE, mcdE, sboE	lantibiotic transport system permease protein	--	Environmental Information Processing--Membrane transport--ABC transporters

[denovogenes]_113620	91.97	2e-170	bvu:BVU_2259	K00721	DPM1	dolichol-phosphate mannosyltransferase	2.4.1.83	Metabolism--Glycan biosynthesis and metabolism--N-Glycan biosynthesis|Metabolism--Global and overview maps--Metabolic pathways

[denovogenes]_113622	61.29	3e-113	pace:A6070_09575	K02049	ABC.SN.A	NitT/TauT family transport system ATP-binding protein	--	--

[denovogenes]_113624	44.39	7e-45	pmh:P9215_07611	K03526	gcpE, ispG	(E)-4-hydroxy-3-methylbut-2-enyl-diphosphate synthase	1.17.7.1 1.17.7.3	Metabolism--Metabolism of terpenoids and polyketides--Terpenoid backbone biosynthesis|Metabolism--Global and overview maps--Metabolic pathways|Metabolism--Global and overview maps--Biosynthesis of secondary metabolites|Metabolism--Global and overview maps--Biosynthesis of antibiotics

[denovogenes]_113628	83.13	9e-155	rto:RTO_22230	K07444	ypsC	putative N6-adenine-specific DNA methylase	2.1.1.-	--

[denovogenes]_11363	42.63	2e-175	ccel:CCDG5_1596	K03572	mutL	DNA mismatch repair protein MutL	--	Genetic Information Processing--Replication and repair--Mismatch repair

[denovogenes]_113631	99.20	0.0	ssj:SSON53_15980	K00278	nadB	L-aspartate oxidase	1.4.3.16	Metabolism--Amino acid metabolism--Alanine, aspartate and glutamate metabolism|Metabolism--Metabolism of cofactors and vitamins--Nicotinate and nicotinamide metabolism|Metabolism--Global and overview maps--Metabolic pathways

[denovogenes]_113636	63.71	7e-104	shi:Shel_28190	K01451	hipO	hippurate hydrolase	3.5.1.32	Metabolism--Amino acid metabolism--Phenylalanine metabolism

[denovogenes]_113638	43.15	4e-64	ibu:IB211_02929c	K00975	glgC	glucose-1-phosphate adenylyltransferase	2.7.7.27	Metabolism--Carbohydrate metabolism--Starch and sucrose metabolism|Metabolism--Carbohydrate metabolism--Amino sugar and nucleotide sugar metabolism|Metabolism--Global and overview maps--Metabolic pathways|Metabolism--Global and overview maps--Biosynthesis of secondary metabolites|Cellular Processes--Cellular community - prokaryotes--Biofilm formation - Escherichia coli

[denovogenes]_11364	87.20	0.0	lacy:A4V08_26330	K02768	PTS-Fru-EIIA, fruB	PTS system, fructose-specific IIA component	2.7.1.202	Metabolism--Carbohydrate metabolism--Fructose and mannose metabolism|Metabolism--Global and overview maps--Metabolic pathways|Metabolism--Global and overview maps--Microbial metabolism in diverse environments|Environmental Information Processing--Membrane transport--Phosphotransferase system (PTS)

[denovogenes]_11364	87.20	0.0	lacy:A4V08_26330	K02769	PTS-Fru-EIIB, fruA	PTS system, fructose-specific IIB component	2.7.1.202	Metabolism--Carbohydrate metabolism--Fructose and mannose metabolism|Metabolism--Global and overview maps--Metabolic pathways|Metabolism--Global and overview maps--Microbial metabolism in diverse environments|Environmental Information Processing--Membrane transport--Phosphotransferase system (PTS)

[denovogenes]_11364	87.20	0.0	lacy:A4V08_26330	K02770	PTS-Fru-EIIC, fruA	PTS system, fructose-specific IIC component	--	Metabolism--Carbohydrate metabolism--Fructose and mannose metabolism|Metabolism--Global and overview maps--Metabolic pathways|Metabolism--Global and overview maps--Microbial metabolism in diverse environments|Environmental Information Processing--Membrane transport--Phosphotransferase system (PTS)

[denovogenes]_113642	59.29	3e-95	eel:EUBELI_20515	K08483	PTS-EI.PTSI, ptsI	phosphotransferase system, enzyme I, PtsI	2.7.3.9	Environmental Information Processing--Membrane transport--Phosphotransferase system (PTS)

[denovogenes]_113643	53.91	4e-90	cle:Clole_2032	K10206	E2.6.1.83	LL-diaminopimelate aminotransferase	2.6.1.83	Metabolism--Amino acid metabolism--Lysine biosynthesis|Metabolism--Global and overview maps--Metabolic pathways|Metabolism--Global and overview maps--Biosynthesis of secondary metabolites|Metabolism--Global and overview maps--Biosynthesis of antibiotics|Metabolism--Global and overview maps--Biosynthesis of amino acids

[denovogenes]_113644	85.20	6e-156	yel:LC20_02973	K01890	FARSB, pheT	phenylalanyl-tRNA synthetase beta chain	6.1.1.20	Genetic Information Processing--Translation--Aminoacyl-tRNA biosynthesis

[denovogenes]_113645	52.81	3e-76	rrs:RoseRS_2318	K02031	ABC.PE.A	peptide/nickel transport system ATP-binding protein	--	Cellular Processes--Cellular community - prokaryotes--Quorum sensing

[denovogenes]_113645	52.81	3e-76	rrs:RoseRS_2318	K02032	ABC.PE.A1	peptide/nickel transport system ATP-binding protein	--	Cellular Processes--Cellular community - prokaryotes--Quorum sensing

[denovogenes]_113646	98.39	3e-170	bhl:Bache_3150	K04077	groEL, HSPD1	chaperonin GroEL	--	Genetic Information Processing--Folding, sorting and degradation--RNA degradation|Organismal Systems--Aging--Longevity regulating pathway - worm|Human Diseases--Endocrine and metabolic diseases--Type I diabetes mellitus|Human Diseases--Infectious diseases: Bacterial--Legionellosis|Human Diseases--Infectious diseases: Bacterial--Tuberculosis

[denovogenes]_113648	49.44	1e-47	bprm:CL3_20190	K02238	comEC	competence protein ComEC	--	--

[denovogenes]_113649	46.72	4e-67	cbt:CLH_0602	K02015	ABC.FEV.P	iron complex transport system permease protein	--	Environmental Information Processing--Membrane transport--ABC transporters

[denovogenes]_11365	93.27	0.0	bcel:BcellWH2_00937	K00615	E2.2.1.1, tktA, tktB	transketolase	2.2.1.1	Metabolism--Carbohydrate metabolism--Pentose phosphate pathway|Metabolism--Energy metabolism--Carbon fixation in photosynthetic organisms|Metabolism--Metabolism of terpenoids and polyketides--Biosynthesis of ansamycins|Metabolism--Global and overview maps--Metabolic pathways|Metabolism--Global and overview maps--Biosynthesis of secondary metabolites|Metabolism--Global and overview maps--Microbial metabolism in diverse environments|Metabolism--Global and overview maps--Biosynthesis of antibiotics|Metabolism--Global and overview maps--Carbon metabolism|Metabolism--Global and overview maps--Biosynthesis of amino acids

[denovogenes]_113650	50.43	3e-79	euc:EC1_12090	K01002	mdoB	phosphoglycerol transferase	2.7.8.20	Metabolism--Global and overview maps--Metabolic pathways

[denovogenes]_113653	58.62	3e-89	cso:CLS_30570	K07098	K07098	uncharacterized protein	--	--

[denovogenes]_113659	55.20	1e-93	paef:R50345_13395	K01990	ABC-2.A	ABC-2 type transport system ATP-binding protein	--	--

[denovogenes]_113663	67.48	1e-123	csr:Cspa_c51690	K01208	cd, ma, nplT	cyclomaltodextrinase / maltogenic alpha-amylase / neopullulanase	3.2.1.54 3.2.1.133 3.2.1.135	Metabolism--Carbohydrate metabolism--Starch and sucrose metabolism|Metabolism--Global and overview maps--Metabolic pathways

[denovogenes]_113666	59.70	5e-45	efc:EFAU004_00063	K18220	tetM, tetO	ribosomal protection tetracycline resistance protein	--	--

[denovogenes]_113667	40.82	6e-39	esl:O3K_24375	K18530	frvX	putative aminopeptidase FrvX	3.4.11.-	--

[denovogenes]_113668	67.79	7e-106	ccu:Ccur_07230	K14087	echB	ech hydrogenase subunit B	--	--

[denovogenes]_113677	100.00	8e-176	sbc:SbBS512_E4212	K08163	mdtL	MFS transporter, DHA1 family, multidrug resistance protein	--	--

[denovogenes]_113679	100.00	1e-180	bfb:VU15_02875	K02005	ABC.CD.TX	HlyD family secretion protein	--	--

[denovogenes]_113680	81.22	1e-119	pfr:PFREUD_24170	K03501	gidB, rsmG	16S rRNA (guanine527-N7)-methyltransferase	2.1.1.170	--

[denovogenes]_113681	86.50	3e-160	pfre:RM25_2052	K00373	narJ, narW	nitrate reductase molybdenum cofactor assembly chaperone NarJ/NarW	--	Environmental Information Processing--Signal transduction--Two-component system

[denovogenes]_113682	94.38	2e-177	pfr:PFREUD_21630	K00390	cysH	phosphoadenosine phosphosulfate reductase	1.8.4.8 1.8.4.10	Metabolism--Energy metabolism--Sulfur metabolism|Metabolism--Global and overview maps--Metabolic pathways|Metabolism--Global and overview maps--Microbial metabolism in diverse environments

[denovogenes]_113684	92.77	5e-162	pfre:RM25_1789	K01834	PGAM, gpmA	2,3-bisphosphoglycerate-dependent phosphoglycerate mutase	5.4.2.11	Metabolism--Carbohydrate metabolism--Glycolysis / Gluconeogenesis|Metabolism--Amino acid metabolism--Glycine, serine and threonine metabolism|Metabolism--Energy metabolism--Methane metabolism|Metabolism--Global and overview maps--Metabolic pathways|Metabolism--Global and overview maps--Biosynthesis of secondary metabolites|Metabolism--Global and overview maps--Microbial metabolism in diverse environments|Metabolism--Global and overview maps--Biosynthesis of antibiotics|Metabolism--Global and overview maps--Carbon metabolism|Metabolism--Global and overview maps--Biosynthesis of amino acids|Organismal Systems--Endocrine system--Glucagon signaling pathway|Human Diseases--Cancers: Overview--Central carbon metabolism in cancer

[denovogenes]_113685	85.54	9e-157	pfr:PFREUD_08300	K07025	K07025	putative hydrolase of the HAD superfamily	--	--

[denovogenes]_113686	97.99	2e-179	pfre:RM25_1415	K09013	sufC	Fe-S cluster assembly ATP-binding protein	--	--

[denovogenes]_113687	89.02	1e-149	pfre:RM25_1377	K00215	dapB	4-hydroxy-tetrahydrodipicolinate reductase	1.17.1.8	Metabolism--Biosynthesis of other secondary metabolites--Monobactam biosynthesis|Metabolism--Amino acid metabolism--Lysine biosynthesis|Metabolism--Global and overview maps--Metabolic pathways|Metabolism--Global and overview maps--Biosynthesis of secondary metabolites|Metabolism--Global and overview maps--Microbial metabolism in diverse environments|Metabolism--Global and overview maps--Biosynthesis of antibiotics|Metabolism--Global and overview maps--Biosynthesis of amino acids

[denovogenes]_113693	96.77	5e-172	pfre:RM25_0726	K00989	rph	ribonuclease PH	2.7.7.56	--

[denovogenes]_113694	89.88	7e-165	pdi:BDI_0596	K01834	PGAM, gpmA	2,3-bisphosphoglycerate-dependent phosphoglycerate mutase	5.4.2.11	Metabolism--Carbohydrate metabolism--Glycolysis / Gluconeogenesis|Metabolism--Amino acid metabolism--Glycine, serine and threonine metabolism|Metabolism--Energy metabolism--Methane metabolism|Metabolism--Global and overview maps--Metabolic pathways|Metabolism--Global and overview maps--Biosynthesis of secondary metabolites|Metabolism--Global and overview maps--Microbial metabolism in diverse environments|Metabolism--Global and overview maps--Biosynthesis of antibiotics|Metabolism--Global and overview maps--Carbon metabolism|Metabolism--Global and overview maps--Biosynthesis of amino acids|Organismal Systems--Endocrine system--Glucagon signaling pathway|Human Diseases--Cancers: Overview--Central carbon metabolism in cancer

[denovogenes]_113695	72.95	2e-134	pdi:BDI_0047	K02517	lpxL, htrB	Kdo2-lipid IVA lauroyltransferase	2.3.1.241	Metabolism--Glycan biosynthesis and metabolism--Lipopolysaccharide biosynthesis|Metabolism--Global and overview maps--Metabolic pathways

[denovogenes]_11370	99.25	0.0	elx:CDCO157_0435	K03546	sbcC	exonuclease SbcC	--	--

[denovogenes]_113700	98.39	0.0	bth:BT_4416	K06201	cutC	copper homeostasis protein	--	--

[denovogenes]_113704	47.27	4e-74	kpx:PMK1_ndm00311	K03981	dsbC	thiol:disulfide interchange protein DsbC	5.3.4.1	--

[denovogenes]_113705	98.80	6e-179	stb:SGPB_0460	K19310	bcrB	bacitracin transport system permease protein	--	Environmental Information Processing--Membrane transport--ABC transporters

[denovogenes]_113707	56.36	9e-95	dtx:ATSB10_32090	K00799	GST, gst	glutathione S-transferase	2.5.1.18	Metabolism--Metabolism of other amino acids--Glutathione metabolism|Metabolism--Xenobiotics biodegradation and metabolism--Metabolism of xenobiotics by cytochrome P450|Metabolism--Xenobiotics biodegradation and metabolism--Drug metabolism - cytochrome P450|Human Diseases--Drug resistance: Antineoplastic--Platinum drug resistance|Human Diseases--Cancers: Overview--Chemical carcinogenesis|Human Diseases--Cardiovascular diseases--Fluid shear stress and atherosclerosis

[denovogenes]_113708	63.49	1e-108	rob:CK5_16440	K02108	ATPF0A, atpB	F-type H+-transporting ATPase subunit a	--	Metabolism--Energy metabolism--Oxidative phosphorylation|Metabolism--Energy metabolism--Photosynthesis|Metabolism--Global and overview maps--Metabolic pathways

[denovogenes]_113709	99.60	0.0	cfd:CFNIH1_08870	K11892	impK, ompA, vasF, dotU	type VI secretion system protein ImpK	--	Environmental Information Processing--Membrane transport--Bacterial secretion system

[denovogenes]_11371	91.02	0.0	bcef:BcrFT9_00340	K02334	dpo	DNA polymerase bacteriophage-type	2.7.7.7	--

[denovogenes]_113710	100.00	1e-178	cif:AL515_01050	K17466	PTS-Dga-EIIC, dgaC	PTS system, D-glucosaminate-specific IIC component	--	Metabolism--Carbohydrate metabolism--Pentose phosphate pathway|Metabolism--Global and overview maps--Microbial metabolism in diverse environments|Environmental Information Processing--Membrane transport--Phosphotransferase system (PTS)

[denovogenes]_113713	89.92	7e-167	pdi:BDI_0708	K03218	rlmB	23S rRNA (guanosine2251-2'-O)-methyltransferase	2.1.1.185	--

[denovogenes]_113716	56.28	5e-92	dvu:DVU0589	K03753	mobB	molybdopterin-guanine dinucleotide biosynthesis protein B	--	--

[denovogenes]_113717	81.30	8e-146	dpg:DESPIGER_0629	K02042	phnE	phosphonate transport system permease protein	--	Environmental Information Processing--Membrane transport--ABC transporters

[denovogenes]_113718	70.45	5e-129	pdi:BDI_2563	K03524	birA	BirA family transcriptional regulator, biotin operon repressor / biotin-[acetyl-CoA-carboxylase] ligase	6.3.4.15	Metabolism--Metabolism of cofactors and vitamins--Biotin metabolism|Metabolism--Global and overview maps--Metabolic pathways

[denovogenes]_11372	99.85	0.0	paeg:AI22_08060	K18930	dld	D-lactate dehydrogenase	--	--

[denovogenes]_113720	100.00	0.0	paeo:M801_1118	K02391	flgF	flagellar basal-body rod protein FlgF	--	Cellular Processes--Cell motility--Flagellar assembly

[denovogenes]_113723	100.00	0.0	fpla:A4U99_13990	K07699	spo0A	two-component system, response regulator, stage 0 sporulation protein A	--	Environmental Information Processing--Signal transduction--Two-component system|Cellular Processes--Cellular community - prokaryotes--Quorum sensing

[denovogenes]_113724	73.88	3e-131	brm:Bmur_0156	K15555	ssuB	sulfonate transport system ATP-binding protein	3.6.3.-	Metabolism--Energy metabolism--Sulfur metabolism|Environmental Information Processing--Membrane transport--ABC transporters

[denovogenes]_113725	92.14	3e-149	efe:EFER_1190	K07772	torR	two-component system, OmpR family, torCAD operon response regulator TorR	--	Environmental Information Processing--Signal transduction--Two-component system

[denovogenes]_113726	100.00	0.0	cfd:CFNIH1_16660	K06074	ABC.VB12.A, btuD	vitamin B12 transport system ATP-binding protein	3.6.3.33	Environmental Information Processing--Membrane transport--ABC transporters

[denovogenes]_11373	100.00	0.0	paeo:M801_5341	K03656	rep	ATP-dependent DNA helicase Rep	3.6.4.12	--

[denovogenes]_113730	100.00	7e-175	paeo:M801_2922	K03521	fixA, etfB	electron transfer flavoprotein beta subunit	--	--

[denovogenes]_113731	45.38	1e-75	bco:Bcell_3649	K16698	tuaG	teichuronic acid biosynthesis glycosyltransferase TuaG	2.4.-.-	--

[denovogenes]_113734	61.28	2e-98	rto:RTO_09530	K03575	mutY	A/G-specific adenine glycosylase	3.2.2.-	Genetic Information Processing--Replication and repair--Base excision repair

[denovogenes]_113737	97.99	3e-179	fpla:A4U99_05435	K03584	recO	DNA repair protein RecO (recombination protein O)	--	Genetic Information Processing--Replication and repair--Homologous recombination

[denovogenes]_113739	100.00	2e-169	paeu:BN889_03175	K07090	K07090	uncharacterized protein	--	--

[denovogenes]_11374	95.81	0.0	bfs:BF9343_2947	K04084	dsbD	thiol:disulfide interchange protein DsbD	1.8.1.8	--

[denovogenes]_113740	97.98	2e-178	cfd:CFNIH1_03960	K00757	udp, UPP	uridine phosphorylase	2.4.2.3	Metabolism--Nucleotide metabolism--Pyrimidine metabolism|Metabolism--Xenobiotics biodegradation and metabolism--Drug metabolism - other enzymes|Metabolism--Global and overview maps--Metabolic pathways

[denovogenes]_113742	99.20	0.0	cfd:CFNIH1_14260	K21029	moeB	molybdopterin-synthase adenylyltransferase	2.7.7.80	Genetic Information Processing--Folding, sorting and degradation--Sulfur relay system

[denovogenes]_113744	100.00	4e-180	paeo:M801_3614	K03787	surE	5'-nucleotidase	3.1.3.5	Metabolism--Nucleotide metabolism--Purine metabolism|Metabolism--Nucleotide metabolism--Pyrimidine metabolism|Metabolism--Metabolism of cofactors and vitamins--Nicotinate and nicotinamide metabolism|Metabolism--Global and overview maps--Metabolic pathways|Metabolism--Global and overview maps--Biosynthesis of secondary metabolites

[denovogenes]_113748	47.71	5e-63	fpa:FPR_09070	K06346	jag	spoIIIJ-associated protein	--	--

[denovogenes]_113750	100.00	3e-180	cfd:CFNIH1_07200	K01803	TPI, tpiA	triosephosphate isomerase (TIM)	5.3.1.1	Metabolism--Carbohydrate metabolism--Glycolysis / Gluconeogenesis|Metabolism--Carbohydrate metabolism--Fructose and mannose metabolism|Metabolism--Carbohydrate metabolism--Inositol phosphate metabolism|Metabolism--Energy metabolism--Carbon fixation in photosynthetic organisms|Metabolism--Global and overview maps--Metabolic pathways|Metabolism--Global and overview maps--Biosynthesis of secondary metabolites|Metabolism--Global and overview maps--Microbial metabolism in diverse environments|Metabolism--Global and overview maps--Biosynthesis of antibiotics|Metabolism--Global and overview maps--Carbon metabolism|Metabolism--Global and overview maps--Biosynthesis of amino acids

[denovogenes]_113751	42.62	2e-38	pse:NH8B_4087	K00874	kdgK	2-dehydro-3-deoxygluconokinase	2.7.1.45	Metabolism--Carbohydrate metabolism--Pentose phosphate pathway|Metabolism--Global and overview maps--Metabolic pathways|Metabolism--Global and overview maps--Microbial metabolism in diverse environments|Metabolism--Global and overview maps--Carbon metabolism

[denovogenes]_113752	92.74	7e-164	pdi:BDI_2401	K02066	mlaE, linK	phospholipid/cholesterol/gamma-HCH transport system permease protein	--	Environmental Information Processing--Membrane transport--ABC transporters

[denovogenes]_113754	97.60	0.0	bfg:BF638R_2259	K01897	ACSL, fadD	long-chain acyl-CoA synthetase	6.2.1.3	Metabolism--Lipid metabolism--Fatty acid biosynthesis|Metabolism--Lipid metabolism--Fatty acid degradation|Metabolism--Global and overview maps--Metabolic pathways|Metabolism--Global and overview maps--Fatty acid metabolism|Cellular Processes--Cellular community - prokaryotes--Quorum sensing|Organismal Systems--Endocrine system--PPAR signaling pathway|Cellular Processes--Transport and catabolism--Peroxisome|Organismal Systems--Endocrine system--Adipocytokine signaling pathway

[denovogenes]_113755	77.51	5e-143	eal:EAKF1_ch2801	K10938	acfC	accessory colonization factor AcfC	--	Cellular Processes--Cellular community - prokaryotes--Biofilm formation - Vibrio cholerae

[denovogenes]_113757	99.20	4e-180	pdi:BDI_2488	K03588	ftsW, spoVE	cell division protein FtsW	--	Cellular Processes--Cell growth and death--Cell cycle - Caulobacter

[denovogenes]_113760	98.39	0.0	bfb:VU15_04300	K00703	glgA	starch synthase	2.4.1.21	Metabolism--Carbohydrate metabolism--Starch and sucrose metabolism|Metabolism--Global and overview maps--Metabolic pathways|Metabolism--Global and overview maps--Biosynthesis of secondary metabolites|Cellular Processes--Cellular community - prokaryotes--Biofilm formation - Escherichia coli

[denovogenes]_113761	60.08	7e-83	dpg:DESPIGER_1559	K02233	E2.7.8.26, cobS, cobV	adenosylcobinamide-GDP ribazoletransferase	2.7.8.26	Metabolism--Metabolism of cofactors and vitamins--Porphyrin and chlorophyll metabolism|Metabolism--Global and overview maps--Metabolic pathways

[denovogenes]_113763	57.94	1e-90	shi:Shel_28500	K03501	gidB, rsmG	16S rRNA (guanine527-N7)-methyltransferase	2.1.1.170	--

[denovogenes]_113764	99.20	2e-179	pdi:BDI_3955	K02233	E2.7.8.26, cobS, cobV	adenosylcobinamide-GDP ribazoletransferase	2.7.8.26	Metabolism--Metabolism of cofactors and vitamins--Porphyrin and chlorophyll metabolism|Metabolism--Global and overview maps--Metabolic pathways

[denovogenes]_113766	99.20	0.0	cfd:CFNIH1_10390	K01918	panC	pantoate--beta-alanine ligase	6.3.2.1	Metabolism--Metabolism of other amino acids--beta-Alanine metabolism|Metabolism--Metabolism of cofactors and vitamins--Pantothenate and CoA biosynthesis|Metabolism--Global and overview maps--Metabolic pathways|Metabolism--Global and overview maps--Biosynthesis of secondary metabolites

[denovogenes]_113769	91.57	3e-166	lacy:A4V08_25595	K09761	rsmE	16S rRNA (uracil1498-N3)-methyltransferase	2.1.1.193	--

[denovogenes]_11377	41.35	2e-117	fjo:Fjoh_3113	K01209	abfA	alpha-N-arabinofuranosidase	3.2.1.55	Metabolism--Carbohydrate metabolism--Amino sugar and nucleotide sugar metabolism

[denovogenes]_113770	44.21	5e-67	dpg:DESPIGER_0679	K07793	tctA	putative tricarboxylic transport membrane protein	--	Environmental Information Processing--Signal transduction--Two-component system

[denovogenes]_113772	97.59	8e-166	lacy:A4V08_33430	K06213	mgtE	magnesium transporter	--	--

[denovogenes]_113773	85.83	5e-160	byl:A4V09_21245	K02036	pstB	phosphate transport system ATP-binding protein	3.6.3.27	Environmental Information Processing--Membrane transport--ABC transporters

[denovogenes]_113775	100.00	2e-158	paeo:M801_2116	K01673	cynT, can	carbonic anhydrase	4.2.1.1	Metabolism--Energy metabolism--Nitrogen metabolism

[denovogenes]_113776	70.64	3e-126	ibu:IB211_03093c	K19119	cas5d	CRISPR-associated protein Cas5d	--	--

[denovogenes]_113777	46.61	7e-78	bcel:BcellWH2_05181	K02169	bioC	malonyl-CoA O-methyltransferase	2.1.1.197	Metabolism--Metabolism of cofactors and vitamins--Biotin metabolism|Metabolism--Global and overview maps--Metabolic pathways

[denovogenes]_113778	100.00	9e-180	prp:M062_25250	K06219	smtA	S-adenosylmethionine-dependent methyltransferase	--	--

[denovogenes]_113779	90.76	8e-161	lacy:A4V08_22610	K15553	ssuA	sulfonate transport system substrate-binding protein	--	Metabolism--Energy metabolism--Sulfur metabolism|Environmental Information Processing--Membrane transport--ABC transporters

[denovogenes]_113780	47.98	2e-67	sdz:Asd1617_03512	K01972	E6.5.1.2, ligA, ligB	DNA ligase (NAD+)	6.5.1.2	Genetic Information Processing--Replication and repair--DNA replication|Genetic Information Processing--Replication and repair--Base excision repair|Genetic Information Processing--Replication and repair--Nucleotide excision repair|Genetic Information Processing--Replication and repair--Mismatch repair

[denovogenes]_113782	100.00	0.0	pdi:BDI_1302	K00721	DPM1	dolichol-phosphate mannosyltransferase	2.4.1.83	Metabolism--Glycan biosynthesis and metabolism--N-Glycan biosynthesis|Metabolism--Global and overview maps--Metabolic pathways

[denovogenes]_113784	54.55	1e-90	banr:A16R_40390	K03529	smc	chromosome segregation protein	--	--

[denovogenes]_113792	100.00	0.0	eal:EAKF1_ch0901c	K07780	appY	AraC family transcriptional regulator, transcriptional regulator required for anaerobic and stationary phase induction of genes	--	Environmental Information Processing--Signal transduction--Two-component system

[denovogenes]_113793	45.97	5e-70	cgo:Corgl_0713	K03424	--	--	--	--

[denovogenes]_113794	94.74	1e-172	cfd:CFNIH1_03960	K00757	udp, UPP	uridine phosphorylase	2.4.2.3	Metabolism--Nucleotide metabolism--Pyrimidine metabolism|Metabolism--Xenobiotics biodegradation and metabolism--Drug metabolism - other enzymes|Metabolism--Global and overview maps--Metabolic pathways

[denovogenes]_113796	95.18	1e-179	lacy:A4V08_05195	K09817	znuC	zinc transport system ATP-binding protein	3.6.3.-	Environmental Information Processing--Membrane transport--ABC transporters

[denovogenes]_113797	95.98	8e-174	pvl:AOB99_15260	K03772	fkpA	FKBP-type peptidyl-prolyl cis-trans isomerase FkpA	5.2.1.8	--

[denovogenes]_11380	98.21	0.0	pdi:BDI_3420	K18930	dld	D-lactate dehydrogenase	--	--

[denovogenes]_113800	93.57	3e-164	cif:AL515_09170	K15555	ssuB	sulfonate transport system ATP-binding protein	3.6.3.-	Metabolism--Energy metabolism--Sulfur metabolism|Environmental Information Processing--Membrane transport--ABC transporters

[denovogenes]_113802	97.60	9e-164	bhl:Bache_1682	K01572	oadB	oxaloacetate decarboxylase, beta subunit	4.1.1.3	Metabolism--Carbohydrate metabolism--Pyruvate metabolism|Metabolism--Global and overview maps--Metabolic pathways

[denovogenes]_113803	100.00	0.0	fpla:A4U99_09780	K00554	trmD	tRNA (guanine37-N1)-methyltransferase	2.1.1.228	--

[denovogenes]_113806	99.20	0.0	sdz:Asd1617_05767	K01467	ampC	beta-lactamase class C	3.5.2.6	Human Diseases--Drug resistance: Antimicrobial--beta-Lactam resistance|Environmental Information Processing--Signal transduction--Two-component system

[denovogenes]_113809	82.11	2e-147	rto:RTO_10660	K20074	prpC, phpP	PPM family protein phosphatase	3.1.3.16	--

[denovogenes]_113810	100.00	3e-177	paeo:M801_3435	K15555	ssuB	sulfonate transport system ATP-binding protein	3.6.3.-	Metabolism--Energy metabolism--Sulfur metabolism|Environmental Information Processing--Membrane transport--ABC transporters

[denovogenes]_113812	99.20	9e-178	paeo:M801_2872	K03394	cobI-cbiL	precorrin-2/cobalt-factor-2 C20-methyltransferase	2.1.1.130 2.1.1.151	Metabolism--Metabolism of cofactors and vitamins--Porphyrin and chlorophyll metabolism|Metabolism--Global and overview maps--Metabolic pathways

[denovogenes]_113819	51.79	2e-77	agl:PYTT_1290	K06907	K06907	uncharacterized protein	--	--

[denovogenes]_11382	59.70	0.0	bcel:BcellWH2_00508	K03546	sbcC	exonuclease SbcC	--	--

[denovogenes]_113820	89.60	7e-175	bvs:BARVI_04290	K18785	mp2	beta-1,4-mannooligosaccharide/beta-1,4-mannosyl-N-acetylglucosamine phosphorylase	2.4.1.319 2.4.1.320	--

[denovogenes]_113822	40.74	1e-21	adf:107331951	K19719	COL2A	collagen, type II, alpha	--	Environmental Information Processing--Signal transduction--PI3K-Akt signaling pathway|Cellular Processes--Cellular community - eukaryotes--Focal adhesion|Environmental Information Processing--Signaling molecules and interaction--ECM-receptor interaction|Organismal Systems--Digestive system--Protein digestion and absorption

[denovogenes]_113823	48.71	2e-76	rto:RTO_24890	K07025	K07025	putative hydrolase of the HAD superfamily	--	--

[denovogenes]_113827	63.90	1e-115	brm:Bmur_0155	K15555	ssuB	sulfonate transport system ATP-binding protein	3.6.3.-	Metabolism--Energy metabolism--Sulfur metabolism|Environmental Information Processing--Membrane transport--ABC transporters

[denovogenes]_113832	100.00	1e-180	bbrd:BBBR_1379	K01193	INV, sacA	beta-fructofuranosidase	3.2.1.26	Metabolism--Carbohydrate metabolism--Galactose metabolism|Metabolism--Carbohydrate metabolism--Starch and sucrose metabolism|Metabolism--Global and overview maps--Metabolic pathways

[denovogenes]_113835	52.78	1e-89	mac:MA_2835	K07492	K07492	putative transposase	--	--

[denovogenes]_113837	59.38	3e-97	cgo:Corgl_1764	K00965	galT, GALT	UDPglucose--hexose-1-phosphate uridylyltransferase	2.7.7.12	Metabolism--Carbohydrate metabolism--Galactose metabolism|Metabolism--Carbohydrate metabolism--Amino sugar and nucleotide sugar metabolism|Metabolism--Global and overview maps--Metabolic pathways|Organismal Systems--Endocrine system--Prolactin signaling pathway

[denovogenes]_113841	40.83	6e-36	cmk:103186045	K11147	DHRS4	dehydrogenase/reductase SDR family member 4	1.1.-.-	Metabolism--Metabolism of cofactors and vitamins--Retinol metabolism|Metabolism--Global and overview maps--Metabolic pathways|Cellular Processes--Transport and catabolism--Peroxisome

[denovogenes]_113842	99.20	2e-178	bdh:GV66_14910	K03086	SIG1, rpoD	RNA polymerase primary sigma factor	--	--

[denovogenes]_113844	93.60	1e-172	eum:ECUMN_3198	K03225	yscQ, sctQ, hrcQ	type III secretion protein Q	--	Environmental Information Processing--Membrane transport--Bacterial secretion system

[denovogenes]_113849	91.57	1e-163	cfd:CFNIH1_00975	K00879	fucK	L-fuculokinase	2.7.1.51	Metabolism--Carbohydrate metabolism--Fructose and mannose metabolism|Metabolism--Global and overview maps--Microbial metabolism in diverse environments
[truncated: 23,706,460 more chars]
